# Multi-label *ℓ*_2_-regularized logistic regression for predicting activation/inhibition relationships in human protein-protein interaction networks

**DOI:** 10.1038/srep36453

**Published:** 2016-11-07

**Authors:** Suyu Mei, Kun Zhang

**Affiliations:** 1 Software College, Shenyang Normal University, Shenyang, 110034, China; 2 Bioinformatics Facility of RCMI Cancer Research Center, Department of Computer Science, Xavier University of Louisiana, New Orleans, LA 70125, USA

## Abstract

Protein-protein interaction (PPI) networks are naturally viewed as infrastructure to infer signalling pathways. The descriptors of signal events between two interacting proteins such as upstream/downstream signal flow, activation/inhibition relationship and protein modification are indispensable for inferring signalling pathways from PPI networks. However, such descriptors are not available in most cases as most PPI networks are seldom semantically annotated. In this work, we extend *ℓ*_2_-regularized logistic regression to the scenario of multi-label learning for predicting the activation/inhibition relationships in human PPI networks. The phenomenon that both activation and inhibition relationships exist between two interacting proteins is computationally modelled by multi-label learning framework. The problem of GO (gene ontology) sparsity is tackled by introducing the homolog knowledge as independent homolog instances. *ℓ*_2_-regularized logistic regression is accordingly adopted here to penalize the homolog noise and to reduce the computational complexity of the double-sized training data. Computational results show that the proposed method achieves satisfactory multi-label learning performance and outperforms the existing phenotype correlation method on the experimental data of *Drosophila melanogaster*. Several predictions have been validated against recent literature. The predicted activation/inhibition relationships in human PPI networks are provided in the supplementary file for further biomedical research.

Protein-protein interactions (PPIs) play important roles in mediating gene expression & regulation, cell signalling and organismal development. Aberrant protein-protein interactions could lead to diseases^1^. From a computational point of view, PPI networks are naturally regarded as essential infrastructure to infer signalling pathways in a manner of unsupervised learning^2^,^3^,^4^. To understand the signal flows in human PPI networks, we need the descriptors of signal events between two physically interacting proteins, such as upstream/downstream signal flow, activation/inhibition relationship, chemical reaction, protein modification, etc. However, the existing human PPI networks are seldom semantically annotated.

In recent years, much effort has been made to semantically annotate protein-protein interaction networks. For instance, statistical or machine learning methods are proposed to predict the upstream/downstream directionality between two interacting proteins^3^,^4^,^5^,^6^; data mining or machine learning methods are developed to predict the PTM (post-translational protein modification) types of interaction^7^,^8^,^9^. In^10^, RNAi screens data are exploited to derive a genotype-phenotype matrix to calculate Pearson correlation coefficients of phenotypes between two genes, based on which to predict the activation/inhibition relationships in *Drosophila melanogaster* PPI networks. Activation/inhibition relationships play important roles in relaying signals between physically interacting proteins and in mediating cross-talks between signalling pathways. Activation of oncogenes and/or inhibition of tumor suppressor genes to some extent could lead to serious diseases. To the best of our knowledge, there is to date no computational method developed for predicting the activation/inhibition relationships in human PPI networks. The only existing computational method that predicts activation/inhibition relationships focuses on relatively small-scale *Drosophila melanogaster* PPI networks^10^. The assumption behind the method is that activation relationship exists between two interacting genes if they show similar phenotypic patterns; otherwise inhibition relationship exists if the phenotypes of these two genes do not occur at the same time. Based on the assumption, a phenotype correlation method was developed to predict the activation/inhibition relationships in *Drosophila melanogaster* PPI networks, wherein positive Pearson correlation coefficient between two genotypes’ phenotypes indicates activation relationship, while negative Pearson correlation coefficient indicates inhibition relationship. The idea behind the method is simple and easy to implement. Nevertheless, there are several concerns to be addressed. Firstly, the method needs phenotype data to derive genotype-phenotype matrix. The requirement may be practical for small-scale *Drosophila melanogaster* PPI networks. For large-scale human PPI networks, phenotype data may not be available and the requirement imposes demanding data constraint on computational modelling. Secondly, the method used indirect phenotype data to predict activation/inhibition relationships. Actually the experimental activation/inhibition data that contain more reliable and direct information are not exploited at all. Finally, dissimilar phenotypic patterns between two interacting genes (e.g. *a*, *b*) do not necessarily indicate an inhibition relationship between the two gene*s*. Maybe a third gene *c* inhibits the signalling interaction that gene *a* activates gene *b*.

In this work, we extend *ℓ*_2_-regularized logistic regression method to multi-label learning scenario for predicting the activation/inhibition relationships in human PPI networks. In this method, the available experimental activation/inhibition data are directly exploited as training data. The phenomenon that both activation and inhibition exist between two interacting proteins is computationally modelled by multi-label learning framework. In addition, a third class named *others* is introduced to classify those interacting protein pairs that possess neither activation relationship nor inhibition relationship. Here GO (gene ontology) terms are used as features to represent protein-protein interactions. To address the problems of GO sparsity and null-feature vectors, homolog knowledge transfer is conducted by treating the homolog knowledge as independent homolog instances. 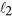-regularized logistic regression is accordingly adopted here to counteract the homolog noise and to reduce the computational complexity caused by the homolog-augmented training data. To demonstrate the efficacies of the proposed method, we conduct ten-fold cross validation &independent test on human activation/inhibition data and performance comparison with the existing phenotype correlation method on *Drosophila melanogaster* activation/inhibition data. Lastly, we apply the trained model to annotate human PPI networks with activation/inhibition relationships for further biomedical research.

## Data and Methods

### Data and materials

To our knowledge, several major databases including STRING^11^, Reactome^12^ and KEGG^13^ have collected a certain amount of activation/inhibition data. In^14^, functional PPIs are also annotated with activation/inhibition relationships. In this study, those activation/inhibition relationships annotated to functional PPIs are removed, as we primarily focus on signal transduction via physical protein-protein interactions. To date, there are several databases that collect human physical protein-protein interactions such as HPRD^15^ and HitPredict^16^. We use these two databases to choose from STRING, Reactome and KEGG those physical protein-protein interactions that have been annotated with activation/inhibition relationships (see Table 1).

As shown in Table 1, the training set is collected from the STRING database^11^. After filtering those duplicate PPIs and those functional PPIs, we obtain 4,504 activation relationships and 1,015 inhibition relationships. To construct the third class *others*, we randomly sample in the physical PPI space that is generated by combining the PPIs in HPRD and HitPredict and then excluding those activation/inhibition relationships that already occur in the training set. The size of class *others* is the same as that of the class activation to reduce the risk of predictive bias toward the large class activation. The physical PPI space minus the training set yields the prediction set that contains 151,201 PPIs.

As shown in Table 1, two independent test sets are constructed from the Reactome database and the KEGG database, respectively. For each database, those functional PPIs are filtered out and those PPIs that already occur in the training set are removed. The remaining PPIs are used as the independent test sets. The independent test set from the Reactome database contains 1,727 activation relationships and 457 inhibition relationships, while the independent test set from the KEGG database contains 339 activation relationships and 126 inhibition relationships.

### Feature construction

Gene ontology (GO) is a hierarchically organized and controlled vocabulary to characterize gene products^17^. It is composed of three aspects, i.e. biological processes (BP), cellular components (CC) and molecular functions (MF). The annotations of these three aspects of genes or gene products are provided in terms of GO terms in the GOA database^18^. Recently GO terms have been successfully used as features to predict protein-protein interactions^19^,^20^,^21^,^22^,^23^. There are two effective approaches to exploit GO terms for representing protein pairs. One approach is to exploit the shared GO terms between two proteins and construct explicit binary feature vectors as the inputs of machine learning methods^20^,^21^,^22^,^23^, and the other approach is to measure the similarity between GO terms in GO DAG (directed acyclic graph) and construct an implicit kernel matrix as the input of kernel methods^19^.

As regards explicit binary feature representation, there are also two methods to exploit GO terms. One method is to directly use the GO terms extracted from the GOA database alone^21^,^22^,^23^, and the other method is to incorporate the ancestor GO terms of each GO term concerned^20^. Incorporation of ancestor GO terms surely adds useful information to the training data and thus improves the model performance. Nevertheless, considering the relationships between GO terms into feature construction also has its adverse effects. On one hand, the ancestor GO terms are correlated with the GO term concerned, if treated as feature components, the artificially introduced correlation could make it more difficult to satisfy the rule of independence and identical distributions between feature components, so as to decrease the generalization ability of machine learning method. On the other hand, since there is generally more than one path from the GO term concerned to the root GO term, improper choice of the traversal path could introduce noise. If the relationships between the ancestors and the GO term concerned need to be considered, kernel method might as well be a better choice, because kernel method is convenient to incorporate the information of semantic similarity between the GO terms and their ancestors.

In this work, we use GO term as feature and represent protein pair in the form of flat binary feature vector, such that the shared GO terms and the distinct GO terms between two interacting proteins are easily represented. The GO terms are simply retrieved from the GOA database^18^. Here we do not exploit the relationships between the GO term concerned and its ancestors to avoid introducing correlations between feature components. To address the problems of GO-sparsity and null-feature vectors, each protein pair is represented with two instances, namely target instance and homolog instance. The target instance is constructed using the GO terms of the protein itself, while the homolog instance is constructed using the GO terms of the homologs. The homologs are extracted from the SwissProt database^24^ using PSI-BLast^25^ against all species (E-value = 10). To formally define the two instances, we introduce the following notations. Let 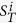 denote the GO term set of protein *i* and 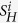 denote the GO term set of the homologs. The GO term set of the training set *U* is defined as follows.


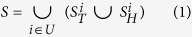


Based on these notations, the target instance and the homolog instance for each protein pair 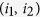 are formally defined as follows.


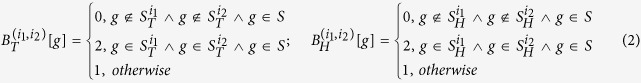


where 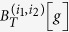 denotes the value of component *g* of the target instance 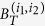 and 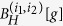 denotes the value of component *g* of the homolog instance 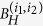. Formula (2) indicates that if protein *i*_1_ and protein *i*_2_ share the same GO term *g*, then the corresponding component in the feature vector 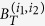 or 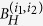 is set 2; if neither protein in the protein pair is annotated with the GO term *g*, then the component is set 0; otherwise, the component is set 1. If either 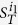 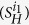 or 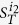

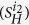
 is empty, the feature vector of the target instance (homolog instance) is defined as null and should be removed.

### Multi-label *ℓ*_2_-regularized logistic regression

Activation/inhibition relationships between two interacting proteins actually need to embrace upstream/downstream directionality. Since prediction of signal directionality is often treated as an independent computational problem^5^,^6^, we nether consider the directionality of activation/inhibition relationships for simplicity as^10^. In reality, both activation relationship and inhibition relationship probably exist between two interacting proteins. For instance, protein A activates protein B (A->B) and protein B inhibits protein A (B-|A). Without considering the signal directionality, the protein pair (A, B) belongs to two classes, i.e. class activation and class inhibition. In the field of machine learning, the phenomenon that protein pair (A, B) possesses two class labels (activation and inhibition) is fit to be computationally modelled by multi-label learning framework.

Multi-label learning is easily converted to traditional supervised learning by two approaches, namely label combination method and binary method^26^. Label combination method converts to new label encodings all possible label combinations that occur in the training data. For example, the label combination {1, 2} is encoded as {1}, the label combination {1, 2, 4} is encoded as {2}, etc. Binary method trains one binary classifier for each class label by treating the data associated with the class label as positive and treating the data associated with all the other class labels as negative. For the sake of lower computational complexity, we choose label combination method so that only one classifier is needed to be trained for multi-class classification problems.

In the scenario of multi-label learning, three metrics, i.e. exact match ratio, macro-average F-measure and micro-average F-measure, are commonly used to measure model performance. Exact match ratio is used to measure the model performance of correctly recognizing all the associated class labels. The demerit of exact match ratio is that it does not count partial label matches that also provide useful information. To take partial label matches into account, macro-average F-measure and micro-average F-measure are especially proposed for performance estimation in the scenario of multi-label learning^26^. Assume that there are *l* test instances, *y*^*i*^ denotes the true label vector of the *i*^th^ instance and 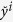 denotes the predicted label vector, exact match ratio is formally defined as follows.


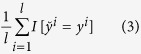


where *I* denotes an indicator function as defined below.


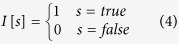


Given the set of class labels *L*={1, 2, 3, … *d*}, for the *i*^th^ instance, the true label set is denoted as *L*_*i*_ and the predicted label set is denoted as 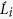, then the true class label and the predicted class label for the *i*^th^ instance are formally defined by *d*-dimensional binary vectors as follows.


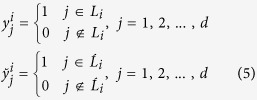


For label *j*, the performance metrics precision (P) and recall (R) are defined as follows.


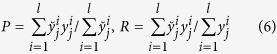


Similar to the definition of 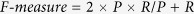, the F-measure for label *j* is formally defined as follows.


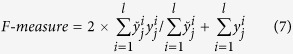


Macro-average F-measure is defined as the unweighted mean of the F-measures of all class labels.


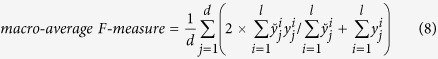


Micro-average F-measure considers the predictions from all instances and calculates the F-measure across all class labels.


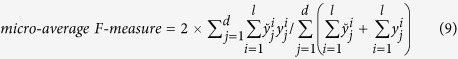


Homolog knowledge transfer via homolog instances is an effective way to tackle the problems of GO term sparsity and null-feature vectors. However, homolog instances also have two adverse effects. First, the homolog instances double the size of training data and according increase the computational complexity. For large training data, things will become much worse. Second, the homolog instances could introduce a certain level of noise. As such, a noise-tolerant machine learning framework that can handle large-scale training data is needed for the homolog knowledge transfer. To the best of our knowledge, 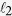-regularized logistic regression^27^ is a robust method that handle large-scale data via fast data fitting and penalize noise via regularization technique. Given a set of instance-label pairs 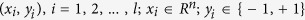, 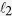-regularized logistic regression solves the following unconstrained optimization problem.


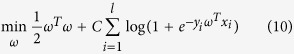


where *ω* denotes the weight vector and *C* denotes the penalty parameter or regularizer. The second term could penalize noise/outlier fitting. The optimization of the objective function (10) can be solved via its dual form:


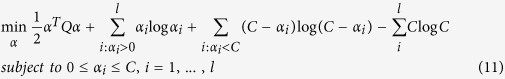


where *α*_*i*_ denotes Lagrangian operator and 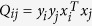.

## Results

### Cross validation performance estimation

Ten-fold cross validation is first conducted on the training data collected from the STRING database^11^ (see Table 1). The multi-label performance is provided in Table 2. In the scenario of traditional supervised learning, both the target instance and the homolog instance are predicted to one class label. The predicted class labels of the two instances can be easily combined into a final label by comparing their decision values^23^,^28^. For instance, the target instance is predicted to the label 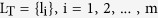, while the homolog instance is predicted to the label 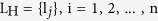. If the decision value of l_i_ is larger than that of l_*j*_, then the combined label is L_T_; otherwise, the combined label is L_*H*_. However, it is more complicated to yield the final class labels in the scenario of multi-label learning, because both the target instance and the homolog instance are predicted to multiple class labels. For example, the target instance is predicted to the labels 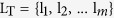, while the homolog instance is predicted to the labels 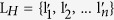. The problem is which label pair 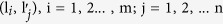 should be chosen to compare their decision values. What’s more, it is a hard problem to determine how many class labels the final combined label set should contain. A proper solution is to report the label set 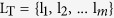 for the target instance and the label set 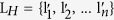 for the homolog instance, respectively. Whether to choose 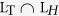 or to choose 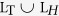 depends on the desire to obtain more reliable predictions or to obtain more informative predictions. As shown in Table 2, the proposed multi-label 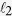-regularized logistic regression method achieves satisfactory target-instance exact match ratio (0.7684) and homolog-instance exact match ratio (0.7677), implying that this method can recognize the complete set of class labels with high accuracy. The label-level macro-average F-measure (target-instance: 0.7587; homolog-instance: 0.7600) and the instance-level micro-average F-measure (target-instance: 0.7930; homolog-instance: 0.7913) show that this method achieves acceptable rates of partial label match.

Next we further check whether the proposed method yields predictive bias. As shown in Table 1, the training data are unevenly distributed among the three classes, wherein the class inhibition (1,015) is much smaller than the other two classes (4,504). The performance on each class is provided in Table 3. The three performance metrics, i.e. F-measure, precision and recall, show that the proposed method performs well on the two larger classes (activation and others), but demonstrates relatively poor performance on the smallest class (inhibition). Take the target-instance performance as an example, the class inhibition achieves 61.04% recall rate, much lower than the 84.33% recall rate of the class activation. The poor performance on the class inhibition largely results from less experimental training data. Similarly, the phenotype correlation method also demonstrates poor performance on the class inhibition, achieving 41% recall rate^10^.

### Validation against Reactome, KEGG and NetPath

Independent test is further conducted here to study how well the proposed method generalizes to unseen data. The independent test sets are provided in Table 1. The performance metrics (i.e. F-measure, precision and recall) for the class activation and the class inhibition are provided in Table 4. We can see that the proposed method achieves satisfactory predictive performance on the class activation of both Reactome and KEGG data. Take the target-instance performance as an example, the proposed method correctly recognizes 79.73% (recall rate) activation relationships from the Reactome database and 80.24% (recall rate) activation relationships from the KEGG database. Comparatively, the homolog-instance performance is generally better than the target-instance performance, partly because the homolog instance contains more abundant GO information. However, the predictive performance on the small class inhibition is much lower than that on the class activation, which is similar to the cross validation performance of this method and the performance of the phenotype correlation method^10^. Take the target-instance performance as an example, the recall rates are 0.3260 and 0.3095 on Reactome and KEGG, respectively. Similarly, the homolog-instance performance is much better than the target-instance performance. With the accumulation of experimental inhibition relationships, the proposed method promises to achieve a certain performance improvement. The correctly recognized activation/inhibition relationships in the Reactome database and the KEGG database are provided in the supplementary file.

NetPath^29^ manually curates 35 human immune/cancer signalling pathways and about 430 activation/inhibition relationships between physically interacting proteins. Interestingly, the activation/inhibition annotations seem to have little connection with the PPIs of the 35 signalling pathways. After removing those PPIs that are already used as training data, we obtain 29 activation/inhibition relationships as independent test set. The proposed method correctly recognizes 93.33% activation relationships and 15.38% inhibition relationships. The independent test performance is similar to that on the Reactome database and the KEGG database. We need more experimental inhibition relationships in the training data to reduce predictive bias.

### Performance comparison with the existing phenotype correlation method

To further demonstrate the efficacies of the proposed method, we need to further compare it with the existing methods. To our knowledge, there are to date no other methods developed to predict the activation/inhibition relationships in human PPI networks. The only comparable method is the phenotype correlation method that is developed to predict the activation/inhibition relationships in *Drosophila melanogaster* PPI networks^10^. However, the phenotype correlation method did not predict the activation/inhibition relationships in human PPI networks, and it is infeasible to find the phenotype data for large human PPI networks. For the reason, we attempt to conduct performance comparison on the *Drosophila melanogaster* PPI networks instead of the human PPI networks. The phenotype correlation method exploits 49 phenotype data of *Drosophila melanogaster* to construct a genotype-phenotype matrix, and then calculates the Pearson correlation coefficient of the phenotypes between two genes to predict activation/inhibition relationships. In the method, activation is treated as positive class and inhibition is treated as negative class. On the independent test data that contain 69 activation relationships and 37 inhibition relationships, the method achieves 97.2% true positive rate (recall rate on the class activation) and 41% true negative rate (recall rate on the class inhibition). This independent test performance is used as the baseline for model comparison.

Before performance estimation on the independent test data of *Drosophila melanogaster* (69 activation relationships and 37 inhibition relationships), we need to first collect experimental data to train a predictive model. Since the training data of 49 phenotypes used as training data^10^ are not publicly available and the proposed method actually does not need the phenotype data, we need to resort to other data sources for model training. Fortunately, from the supplementary file 6 in^10^, we extract 270 experimental activation relationships and 111 experimental inhibition relationships of *Drosophila melanogaster* PPI networks, which are disjoint with the independent test set. This data source is used as training data. The 10-fold cross validation performance on this data set is provided in Table 5. The results show that the proposed method achieves satisfactory overall multi-label performance and per class performance on the class activation and the class *others*. Similarly, the performance on the small class inhibition is still not satisfactory.

Next we use the trained model to evaluate the performance on the independent test data used in^10^. As shown in Table 6, the proposed method achieves fairly promising performance, especially on the small class inhibition. For instance, the homolog-instance recall rate on the class inhibition is 0.8621, significantly outperforming the phenotype correlation method, whose recall rate on the class inhibition is 0.41.

### Interactome-wide predictions of activation/inhibition relationships and validation

Before interactome-wide predictions, we have attempted to train a more robust model on a larger training set by merging the training set from the STRING database with the independent test sets from the Reactome database and the KEGG database (see Table 1). However, no substantial performance gain is obtained. As such, we still use the model trained on the original training data for the interactome-wide activation/inhibition predictions. As shown in Table 1, the prediction set contains 151,201 physical PPIs from the HPRD database and the HitPredict database. The computational results show that 34,453 PPIs among the 151,201 physical PPIs are predicted to the two classes (activation and inhibition), and the remaining PPIs are predicted to the class *others*. The physical PPIs that are predicted to the class activation and/or the class inhibition are provided in the supplementary file. Here we take the human cancer/immune signalling pathways in the NetPath database^29^ as examples and illustrate the predicted activation/inhibition relationships as follows, wherein seven predictions have been validated against the latest database and recent literature.

**Figure 1:**
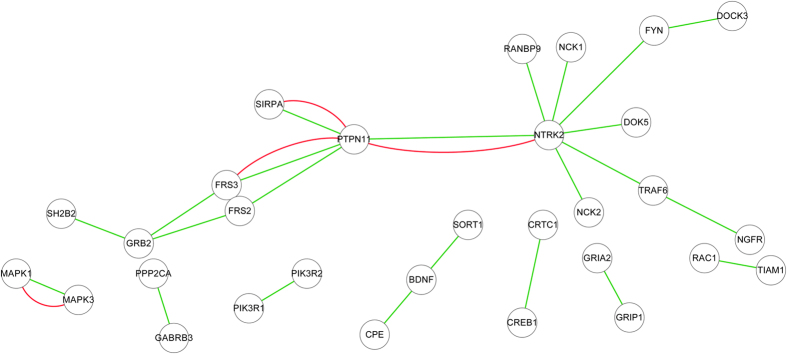
BDNF (Brain-derived neurotrophic factor) signalling pathway. Only the PPIs that are predicted with novel activation/inhibition relationships are illustrated, and the other PPIs in BDNF signalling pathway are omitted. The green line stands for activation and the red line stands for inhibition.

*BDNF (Brain-derived neurotrophic factor) signalling pathway*. Brain-derived neurotrophic factor (BDNF) is a member of family of neurotrophins that plays a major role in the growth, differentiation, plasticity and survival of neurons. BDNF is also involved in the biological processes such as energy metabolism, mental health, behavior, learning, memory, stress, pain and apoptosis^29^,^30^. The predicted activation/inhibition relationships in the BDNF signalling pathway are illustrated in Fig. 1 (those PPIs that are not predicted to the class activation and/or the class inhibition are omitted). As shown in Fig. 1, both activation and inhibition relationships are predicted to exist between *PTPN11* and {*NTRK2*, *FRS3*, *FRS2*, *SIRPA*}. According to the Uniprot database (http://www.uniprot.org/uniprot/Q06124), *PTPN11* mediates cross-talk in multiple signalling pathways, e.g. fibroblast growth factor receptor signalling pathway, epidermal growth factor receptor signalling pathway, FRS2-mediated cascade, brain development, etc. These results may suggest that *PTPN11* plays important roles in coordinating the activation/inhibition of multiple cross-talk pathways.

**Figure 2:**
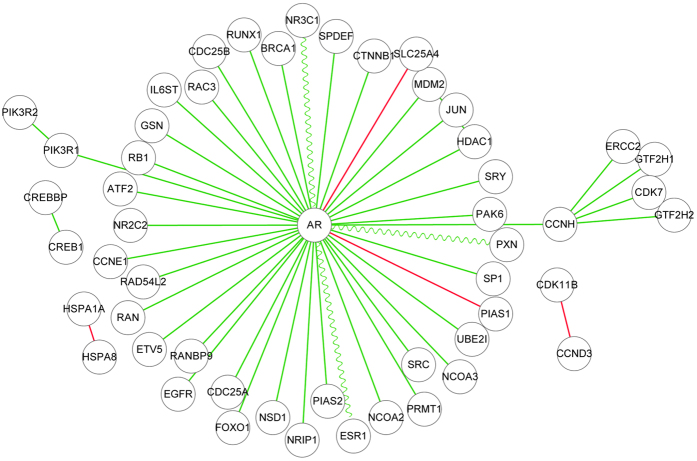
AR (Androgen receptor) signalling pathway. Only the PPIs that are predicted with novel activation/inhibition relationships are illustrated, and the other PPIs in AR signalling pathway are omitted. The green line stands for activation and the red line stands for inhibition. The sinewave green line stands for the verified activations.

*AR (Androgen receptor) signalling pathway*. The androgen receptor is a member of nuclear receptor family of ligand activated transcription factors, stimulation of which activates the SMAD signalling module^29^. The predicted activation/inhibition relationships in AR signalling pathway are illustrated in Fig. 2, where the hub gene *AR* is predicted to activate or to be activated by most of the other genes. The activation relationships between *AR* and {*NR3C1*, *PXN*, *ESR1*} have been experimentally verified^29^. As shown in Fig. 2, inhibition relationships are predicted to exist between gene *AR* and the genes {*CTNNB1*, *PIAS1*}. In^31^, it has been experimentally verified that there is a significant positive correlation between *PIAS1* and *AR* expression in the malignant tissues of prostate cancer, while the Pearson’s correlation between the expressions of these two genes is low in the benign tissues, indicating that the predicted inhibition relationship between *AR* and *PIAS1* is consistent with the experimental evidence.

**Figure 3:**
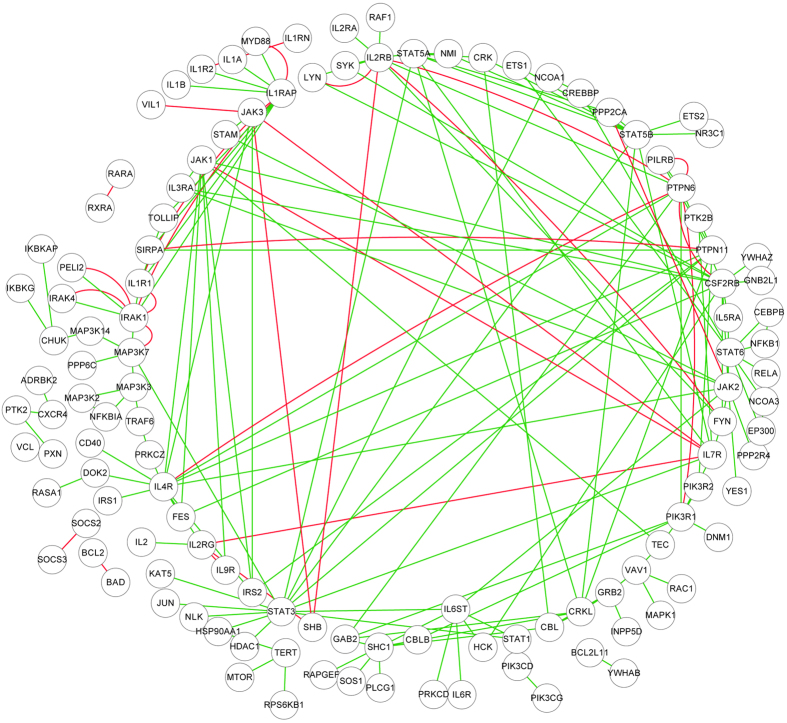
IL (Interleukin) signalling pathway. Only the PPIs that are predicted with novel activation/inhibition relationships are illustrated, and the other PPIs in IL signalling pathway are omitted. The green line stands for activation and the red line stands for inhibition.

*IL (Interleukin) signalling pathways*. Interleukin are a group of cytokines (secreted proteins and signal molecules) that were first seen to be expressed by white blood cells (leukocytes), and the function of immune system depends in a large part on interleukins^32^. The predicted activation/inhibition relationships in IL signalling pathways are illustrated in Fig. 3. As compared to other signalling pathways, much more inhibition relationships are predicted in these pathways. Among the predicted inhibition relationships, the inhibition relationships between gene *IL7R* and the genes {*IL2RG*, *JAK1*, *JAK3*} have been experimentally validated in^33^. IL7 receptor is a receptor complex that consists of the *IL7* receptor alpha chain (*IL7R*) and the common gamma chain (*IL2RG*). In^33^, it has been claimed that the binding of *IL7* to *IL7R* could activate the receptor complex and further activate Janus kinase 1 (*JAK1*) and *JAK3*. In addition, the mutation in *IL7R*, *IL2RG*, *JAK1*, or *JAK3* could also activate the receptor complex, which would lead to impaired B & T cell development, phosphorylation of STAT proteins, and thus cause the activation of survival and proliferation pathways. This statement suggests that the activation of receptor complex {*IL7R*, *IL2RG*} may activate {*JAK1*, *JAK3*} to cause immunodeficiency disease. According to this evidence, the receptor complex {*IL7R*, *IL2RG*} can be inferred to keep inactivated with {*JAK1*, *JAK3*} in normal cells, which validates our predictions.

## Discussion

Computationally annotating protein-protein interaction (PPI) networks has drawn much attention in recent years. Assignment of semantic annotations to the interactions of PPI networks facilitates the derivation of signalling pathways. At present, most existing computational methods focus on predicting the descriptors of signal events between two interacting proteins, such as upstream/downstream directionality, activation/inhibition relationship, chemical reaction, protein modification, etc. Among these signal events, activation/inhibition relationships are significant to reveal the spatiotemporal relay of signalling events in biological processes and to understand the cross-talk mechanism between signalling pathways. Activation of oncogenes and/or inhibition of tumor suppressor genes to some extent cause diseases. To our knowledge, there is only one computational method developed to predict the activation/inhibition relationships in the PPI networks of *Drosophila melanogaster*. There is to date no computational method that focuses on predicting activation/inhibition relationships in human PPI networks.

In this work, we extend 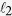-regularized logistic regression to multi-label learning scenario for predicting the activation/inhibition relationships in human PPI networks. This method exploits the available experimental activation/inhibition relationships as training data and thus is comparatively more reliable than the indirect phenotype data based method^10^. In our solution, three major concerns are explicitly addressed. First, activation/inhibition relationships are usually accompanied with the information of directionality. Since prediction of signal directionality is often treated as an independent research topic, we neither consider the directionality of activation/inhibition relationships to make things simple as^10^. If the information of directionality is ignored, both activation relationship and inhibition relationship would co-exist between two interacting proteins. In the field of machine learning, the phenomenon that an instance belongs to more than one class label is fit to be modelled by multi-label learning framework. Second, gene ontology, especially the shared GO terms, has been proven effective to represent protein-protein interactions. Nevertheless, the sparsity of GO terms is the major constraint of GO feature construction method, and in an extreme case it would yield null feature vectors. Here we tackle this problem via homolog knowledge transfer. The homolog knowledge is treated as independent homolog instances to enrich the feature information of the target instances. When the target instance is degenerated into a null feature vector, the homolog instance serves as a substitute for the target instance. Last, the homolog instances double the size of the training data. For large training data, the computational complexity will become a major concern of computational modelling. Furthermore, homolog instances will also introduce a certain level of noise that results from evolutionary divergence. To our knowledge, logistic regression is a classic method to fast fit large data and its latest 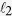-regularization version^27^ could make the model more robust against noise/outlier. For the reasons, here we choose 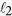-regularized logistic regression to reduce the computational complexity and meanwhile to counteract the impact of noise. As a whole, the combination of several existing techniques rationally addresses the three major concerns, so as to provide a novel solution to the problem of predicting activation/inhibition relationships in human PPI networks. From the aspect of computational contribution, there are some points that need to be pointed out. This work does not attempt to develop a completely novel computational method, and the logistic regression method is a classic method that seemingly introduces little novelty. Actually, the latest version of logistic regression 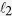-regularized logistic regression is well built on the statistic learning theory, where regularization technique is introduced to make the model more robust against noise. In fact, the 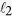-regularized logistic regression method^27^ is rarely used to solve biological problems. Nevertheless, this work to some extent computationally contributes to the methodology of bioinformatics from these major aspects: (1) we extend 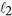-regularized logistic regression to multi-label learning scenario; (2) homolog knowledge transfer is conducted via homolog instances to enrich the feature information and address the problem of GO sparsity; (3) fast data fitting of logistic regression reduces the computational complexity that is increased by homolog instances; (4) homolog noise is counteracted by the regularization technique of 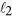-regularized logistic regression; (5) this work first computationally solves the problem of predicting activation/inhibition relationships in human PPI networks.

From the aspect of state-of-art computational modelling, we should choose the best one that could achieve the highest performance from a variety of machine learning methods, such as support vector machine (SVM), neural networks and random forest, etc. SVM^26^ is a theoretically established method that is robust against noise/outlier via regularization technique. Unfortunately, SVM is not an effective solution to large-scale training data with time complexity 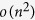. In this work, the training data contain 2x(4,504 + 1,015 + 4,504) instances. Faced up with so large a data, SVM is obviously not a rational choice. Comparatively, 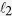-regularized logistic regression could fast fit so large a data effectively in a linear time. Besides the concern of time complexity, noise tolerance is the other major concern in choosing a proper machine learning method. To our knowledge, neural networks and random forest focus on data fitting without introducing noise-penalty mechanism, e.g. regularization technique, such that the two methods are prone to yield overfitting to noise and could not generalize well to unseen data. For the two concerns, SVM, neural networks and random forest are not applicable to this task and so we choose the 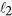-regularized version of classic logistic regression as the base classifier. Furthermore, because the methods SVM, neural networks and random forest have not been used to predict the activation/inhibition relationships in PPI networks, we do not choose these methods as baseline to compare. Instead, we choose the phenotype correlation method^10^ as comparison baseline, though it predicts the activation/inhibition relationships in *Drosophila melanogaster* PPI networks.

As regards the feature construction method using GO terms, there is still a problem that is worth further discussing. As mentioned in the section of feature construction, we adopt the simple method of using the GO terms from the GOA database directly or taking the most specific annotated GO terms. This method has its demerit. As the GO knowledge is unevenly distributed between well-studied proteins and less-studied proteins, the less-studied proteins would lack lower-level or more specific GO terms, so that no shared GO terms are found between these two kinds of proteins even though they are functionally correlated. The method of lowest-common-ancestor^34^ could enrich the information of shared GO terms between two proteins, so that the functional relationships between two GO terms at different levels of GO DAG could to some extent be recovered. Accordingly there are two major concerns for the method of lowest-common-ancestor to be addressed: (1) we need to search the lowest common ancestors in GO DAG for every pair of GO terms, so that the time complexity is increased. Although a portion of informative GO terms have been pre-calculated^35^, the coverage of GO terms is still limited; (2) the lowest common ancestors introduce correlations between feature components into feature construction, so that the independence requirement between feature components is more difficult to satisfy.

Homolog knowledge transfer is another effective way to recover the functional relationships between two GO terms at different levels of GO DAG^23^,^28^,^36^. For instance, a well-studied protein *a* is annotated with a GO term *Term*_*i*_ at the i^th^ level, and a less-studied protein *b* is only annotated to the j^th^ level with a GO term *Term*_*j*_ that is ancestor to *Term*_*i*_. As *Term*_*i*_ is distinct from *Term*_*j*_, it would be taken for granted that the set of shared GO terms is empty and protein *a* is little functionally correlated with protein *b*. Fortunately, the homologs of protein *b* could increase the coverage of well-studied proteins. If protein *a* is functionally related with protein *b*, there is a large chance that a homolog *c* of protein *b* is annotated with *Term*_*i*_. As shown in Tables 4 and 6, the homolog instance achieves better independent test performance than the target instance, especially on the *Drosophila melanogaster* PPI networks the homolog instance correctly recognizes 86.21% inhibition relationships, while the homolog instance correctly recognizes only 65.52% inhibition relationships. The results show that the homolog knowledge transfer enriches the feature information and to some extent recovers the functional relationships between two GO terms at different levels of GO DAG. Similarly, homolog knowledge transfer also has its demerit, that’s, a certain level of noise could be introduced via homolog instances. This is the reason why we introduce 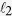-regularized version of logistic regression to counteract noise.

Imbalanced distribution of training data among multiple classes is a hard computational problem in the fields of bioinformatics and machine learning. In this work, the larger classes {activation, others} possess much more training instances than the small class inhibition. As a result, the performance on the class inhibition is much lower than that on the class activation and the class *others*. We have attempted to sample the training data to create approximately even class distributions, for instances, oversampling the small class inhibition, undersampling the larger classes {activation, others}, or developing ensemble of classifiers on the larger classes {activation, others}. Unfortunately, no substantial performance gain is obtained. Maybe accumulation of more training data for the class inhibition is the ultimate way to achieve balanced performance among the three classes.

Computational results show that the proposed method achieves excellent performance on the two large classes {activation, *others*} and relatively poor performance on the small class inhibition. Nevertheless, the performance on the class inhibition is still quite promising as compared to that of the phenotype correlation method^10^. With the accumulation of experimental data, especially the inhibition relationships, the proposed method promises to achieve less biased predictions. We use the proposed method to conduct interactome-wide predictions and the predictions are provided in the supplementary file to provide insights into signal transduction and tumorigenesis. Especially, we map the predicted activation/inhibition relationships onto human immune/cancer signalling pathways from the NetPath database, and seven predictions are found to be consistent with recent literature.

## Additional Information

**How to cite this article**: Mei, S. and Zhang, K. Multi-label *ℓ*_2_-regularized logistic regression for predicting activation/inhibition relationships in human protein-protein interaction networks. *Sci. Rep*. **6**, 36453; doi: 10.1038/srep36453 (2016).

**Publisher’s note**: Springer Nature remains neutral with regard to jurisdictional claims in published maps and institutional affiliations.

## Supplementary Material

Supplementary Information

## Figures and Tables

**Table 1:** Data distributions in the STRING, Reactome and KEGG databases.

	Training set	Independent test set		Prediction set
	STRING	Reactome	KEGG	
Activation	4,504	1,727	339	151,201
Inhibition	1,015	457	126	
Others	4,504	—	—	

**Table 2:** Multi-label learning performance estimation by 10-fold cross validation.

	Exact match ratio	Macro-average F-measure	Micro-average F-measure
Target instance	0.7684	0.7587	0.7930
Homolog instance	0.7677	0.7600	0.7913

**Table 3:** Per class performance estimation by 10-fold cross validation.

	Target instance			Homolog instance		
	F-measure	Precision	Recall	F-measure	Precision	Recall
Activation	0.8129	0.7846	0.8433	0.8083	0.7859	0.8320
Inhibition	0.6544	0.7053	0.6104	0.6624	0.6792	0.6465
Others	0.8089	0.8265	0.7920	0.8093	0.8272	0.7921

**Table 4:** Independent test performance on the Reactome and KEGG databases.

	Target instance			Homolog instance		
	F-measure	Precision	Recall	F-measure	Precision	Recall
Reactome						
Activation	0.8338	0.8737	0.7973	0.8560	0.8693	0.8431
Inhibition	0.4180	0.5820	0.3260	0.4627	0.5479	0.4004
KEGG						
Activation	0.7988	0.7953	0.8024	0.8350	0.7880	0.8879
Inhibition	0.4063	0.5909	0.3095	0.5167	0.5439	0.4921

**Table 5:** 10-fold cross validation performance on the experimental data of *Drosophila melanogaster*.

Overall performance	Exact match ratio		Macro-average F-measure		Micro-average F-measure	
Target instance	0.7940		0.7230		0.7980	
Homolog instance	0.7611		0.7198		0.7641	
**Per class performance**	**Target instance**			**Homolog instance**		
	**F-measure**	**Precision**	**Recall**	**F-measure**	**Precision**	**Recall**
Activation	0.8164	0.7842	0.8514	0.7598	0.7452	0.7751
Inhibition	0.4954	0.5094	0.4821	0.5327	0.5534	0.5135
Others	0.8780	0.9057	0.8521	0.8668	0.8651	0.8685

**Table 6:** Comparison with the existing phenotype correlation method on the independent test set of *Drosophila melanogaster*.

Multi-label l_2_-regularized regression method	Target instance			Homolog instance		
	F-measure	Precision	Recall	F-measure	Precision	Recall
Activation	0.7778	0.7778	0.7778	0.8167	0.7424	0.9074
Inhibition	0.6333	0.6129	0.6552	0.6944	0.5814	0.8621
**Genotype-phenotype correlation method^10^**	**Precision**			**Recall**		
Activation	—			0.972		
Inhibition	—			0.41		
