# Supplementary material for: Multi-label ℓ2-regularized logistic regression for predicting activation/inhibition relationships in human protein-protein interaction networks
Source: Sci Rep. 2016 Nov 7;6:36453. doi: 10.1038/srep36453 (PMC5098220; doi:10.1038/srep36453)
Supplement: Supplementary Information [file srep36453-s1.pdf]

Multi-label ℓ_2-regularized logistic regression for predicting activation/inhibition relationships in human protein-protein interaction networks

Suyu Mei[①*], Kun Zhang[②#]

① Software College, Shenyang Normal University, Shenyang, 110034, China

② Department of Computer Science, Xavier University of Louisianan, New Orleans, LA 70125, USA

Correspondence: *meisygle@gmail.com, #kzhang@xula.edu

PTK2	ROCK1	[activation]
MAPK1	SOS1	[activation]
MYC	SMAD2	[activation]
CXCR4	STAT3	[activation]
NFATC2	PPP3CB	[activation]
MAPK14	SLC9A1	[activation]
EPOR	PTPN11	[activation;inhibition]
PRKCD	RAF1	[activation]
FGF9	FGFR1	[activation]
GNA12	RASA2	[activation]
MMP9	PLG	[activation;inhibition]
MFNG	NOTCH1	[activation]
RAC1	RASGRF1	[activation]
RAC1	WASF2	[activation]
ITGA5	TNC	[activation]
LCP2	PTPN6	[activation;inhibition]
LIMK1	PAK2	[activation]
FGF4	FGFR4	[activation]
RIPK1	TRAF2	[activation]
RAPGEF4	RIMS2	[activation]
FYN	JUP	[activation]
ANAPC11	CDC23	[activation]
EFNA5	EPHA5	[activation]
APP	MAPK11	[activation]
DVL2	RHOA	[activation]
EIF2B2	EIF2S1	[activation]
GSK3B	ILK	[activation]
ITGAV	SPP1	[activation]
CCNE1	FOXM1	[activation]
IKBKB	TNF	[activation]
LAT	ZAP70	[activation]
ACVR1	BMP2	[activation]
CTNNB1	GNA13	[activation]
MAP2K6	TLR3	[activation]
LTB	LTBR	[activation]
EIF4E	RPS6KB1	[activation]
DOK2	RASA1	[activation]
KLRK1	RAET1E	[activation]
KLRK1	MICA	[activation]
NFATC1	PPP3R1	[activation]
ACVR1	SMAD9	[activation;inhibition]
F11R	MLLT4	[activation]
CCR5	GNAI3	[activation]
MAP3K7	RELA	[activation]
CREB1	CREBBP	[activation]
PARD6G	RAC1	[activation]
AURKB	DES	[activation]
GAB2	SHC1	[activation]
ITGA2B	VWF	[activation]
HSPG2	ITGB1	[activation]
CCL3L1	CCR5	[activation]
LYN	PRKCD	[activation]
FKBP1A	PPP3CA	[inhibition]
BIRC3	CASP1	[activation]
MAPKAPK2	TSC2	[activation]
ROBO2	SLIT2	[activation]
ILK	ITGB3	[activation]
CISH	PRLR	[inhibition]
IKBKG	PRKCB	[activation]
PRKCE	TRPV1	[activation]
FLNA	IKBKB	[activation]
NRAS	RASSF5	[activation]
FGG	SERPINA5	[inhibition]
IHH	PTCH1	[activation]
PIK3R1	RRAS2	[activation]
FADD	STAT1	[activation]
NCF1	PAK1	[activation]
FGF18	FGFR2	[activation]
CCL24	CCR3	[activation]
GNAS	LHCGR	[activation]
FGF7	FGFR4	[activation]
RASGRF1	RHOA	[activation]
AMH	AMHR2	[activation]
MYB	NCOR1	[activation]
ERBB3	NRG1	[activation]
FGF18	FGFR3	[activation]

ESR1	POU4F1	[activation]
FRS2	NTRK1	[activation]
MAPK1	RARA	[activation]
ACTB	WASF3	[activation]
BMP6	SMAD5	[activation]
NR3C1	SMAD3	[activation]
F2R	SNX1	[activation]
CCND1	RBL1	[inhibition]
CIT	RHOA	[activation]
LIMK1	ROCK1	[activation]
COL1A1	ITGA5	[activation]
SNAP23	VAMP2	[activation]
FRS2	PTPN11	[activation]
NOTCH4	RBPJ	[activation]
RAC1	VAV3	[activation]
PORCN	WNT6	[activation]
HDAC1	LEF1	[inhibition]
FBXW11	GLI2	[inhibition]
IL6ST	PIK3R1	[activation]
PIK3CA	RPS6KB1	[activation]
DUSP7	MAPK1	[inhibition]
CDK2	FZR1	[inhibition]
CASP8	CHUK	[activation]
CNR1	GNAI2	[activation]
AKT3	PDPK1	[activation]
SNAP25	SYT1	[activation]
NTRK1	SHC3	[activation]
INSR	PTPRF	[inhibition]
LHB	LHCGR	[activation]
BCL2L1	MAPK8	[activation]
ETS1	MAPK1	[activation]
ILK	ITGB1	[activation]
MAP2K4	MAPK1	[activation]
CSNK2B	NFKBIA	[activation]
ATG10	ATG5	[activation]
FLT4	SHC3	[activation]
CASP6	TFAP2A	[activation]
JAK3	SOCS1	[inhibition]
GRB2	SHC2	[activation]
ITGA4	PRKACA	[activation]
PAK2	RAC1	[activation]
BID	CASP8	[activation]
MAPK1	SMAD4	[activation;inhibition]
CD14	TLR4	[activation]
CSNK2A1	NFKBIA	[activation]
MARCKS	PRKCA	[activation]
IRS2	PIK3CD	[activation]
FGF6	FGFR1	[activation]
TGFB2	TGFBR1	[activation]
PTK2	PTK2B	[activation]
CASP10	CASP7	[activation]
CALM1	CAMKK1	[activation]
EFNA1	EPHA5	[activation]
ARHGAP8	RHOA	[activation]
MAPKAPK2	SRF	[activation]
MAPK1	RB1	[activation;inhibition]
BID	MAPK8	[activation]
FGF3	FGFR4	[activation]
ROBO1	SRGAP1	[activation]
EFNB2	EPHB4	[activation]
CTF1	LIFR	[activation]
PTPN2	STAT3	[activation]
FRS2	GRB2	[activation]
MAPK14	STAT4	[activation]
IGF1R	IGF2	[activation]
EPHB2	SDC2	[activation]
ARFGAP1	COPA	[activation]
RASSF5	STK4	[activation]
MAPKAPK2	YWHAZ	[activation]
JAK2	NFKBIA	[activation]
EP300	TP73	[activation]
ANAPC2	FZR1	[activation]
CHUK	PEBP1	[inhibition]
CXCL6	CXCR1	[activation]
NTRK2	SHC2	[activation]
HDAC1	SP1	[activation]

153	RRAS	SOS1	[activation]
154	FRS3	GRB2	[activation]
155	SP1	TP53	[activation]
156	ACTB	RAC2	[activation]
157	HRAS	RASGRP1	[activation]
158	ERBB3	PIK3R1	[activation]
159	CCR5	GNAO1	[activation]
160	PLD1	RALA	[activation]
161	CAV1	TNF	[activation]
162	HLA-G	KIR2DL4	[activation]
163	AKT1	PRKDC	[activation]
164	TBK1	TICAM1	[activation]
165	DUSP8	MAPK9	[inhibition]
166	GNAI1	OPRD1	[activation]
167	CHEK2	NBN	[activation]
168	PRKACA	RAP1B	[activation]
169	CHEK2	TP53BP1	[activation]
170	CSF3R	SOCS3	[inhibition]
171	BCL2L11	MAPK10	[activation]
172	JAK1	PDGFRA	[activation]
173	RAC1	TRAF6	[activation]
174	MAPK8	MAPK9	[activation]
175	BCL2	SOD1	[inhibition]
176	NTRK1	SH2B2	[activation]
177	NCF1	PRKCA	[activation]
178	MAF	MYB	[activation]
179	HHIP	SHH	[inhibition]
180	ITGA9	SPP1	[activation]
181	ACVR1B	BAMBI	[inhibition]
182	CASP6	VIM	[activation]
183	CSF2RB	STAT3	[activation]
184	RAC1	VAV2	[activation]
185	BTK	PLCG2	[activation]
186	BCL6	IRF4	[activation]
187	SOCS1	TIRAP	[inhibition]
188	CRK	IRS1	[activation]
189	PLK1	TPT1	[activation]
190	DDX54	ESR1	[activation]
191	NLGN2	NRXN1	[activation]
192	MAPK3	NTRK1	[activation]
193	CBLB	FYN	[inhibition]
194	LATS2	STK3	[activation]
195	CD55	FYN	[activation]
196	KLRC1	PTPN6	[activation;inhibition]
197	CXCR4	PTK2	[activation]
198	PAK7	RAC1	[activation]
199	CAMK4	CAMKK2	[activation]
200	MAPK14	PLA2G4A	[activation]
201	DCC	PITPNA	[activation]
202	KSR1	YWHAZ	[activation]
203	CSNK1A1	CTNNB1	[activation]
204	FGF23	FGFR3	[activation]
205	CTTN	SRC	[activation]
206	CD4	LAT	[activation]
207	F5	PROC	[inhibition]
208	DNAJB1	HSPA1A	[inhibition]
209	LIFR	PTPN6	[activation;inhibition]
210	CDH2	PIK3R1	[activation]
211	AR	GSK3B	[activation]
212	EFNA2	EPHA5	[activation]
213	OXT	OXTR	[activation]
214	ARHGAP1	RAC1	[activation]
215	ERBB3	PIK3R2	[activation]
216	FZD1	WNT3	[activation]
217	F2R	GNA15	[activation]
218	BAD	BCL2	[inhibition]
219	GAB1	MAPK3	[activation]
220	HLA-E	KLRC2	[activation]
221	PIK3R1	ZAP70	[activation]
222	FSHB	FSHR	[activation]
223	CCL8	CCR3	[activation]
224	CCL1	CCR8	[activation]
225	ABL1	WASF2	[activation]
226	COL1A2	ITGB3	[activation]
227	CRHR1	GNAS	[activation]
228	GNB2L1	PRKCD	[activation]

229	FGF3	FGFR1	[activation]
230	MYC	SMAD3	[activation]
231	CREB1	RPS6KA3	[activation]
232	DAPP1	SRC	[activation]
233	CDC42	WASF1	[activation]
234	CDKN2A	MAPK8	[activation]
235	CD247	LAT	[activation]
236	EFNA4	EPHA6	[activation]
237	PIK3R1	PLCG2	[activation]
238	KLRC1	PTPN11	[activation]
239	RHOC	ROCK1	[activation]
240	MAP3K14	MAP3K7	[activation]
241	MAPK14	NFATC1	[activation]
242	IRS1	NTRK1	[activation]
243	AR	FOXO1	[activation]
244	F8	PROS1	[inhibition]
245	ANAPC11	CDC16	[activation]
246	CXXC4	DVL1	[inhibition]
247	BIRC3	MAP3K14	[activation;inhibition]
248	CXCR2	GNAI2	[activation]
249	JAK1	STAT2	[activation]
250	MAPK1	PTPN5	[inhibition]
251	CDK5	PPP1R1B	[activation]
252	AKT2	PDPK1	[activation]
253	GNAI2	MTNR1A	[activation]
254	EIF2B3	EIF2S2	[activation]
255	DUSP2	MAPK1	[inhibition]
256	AKT1	PTPN1	[activation]
257	RAB5A	RAB7A	[activation]
258	ANAPC1	ANAPC4	[activation]
259	NLGN1	NRXN3	[activation]
260	CTNND1	FER	[activation;inhibition]
261	DAG1	LAMA1	[activation]
262	CSK	DOK1	[activation]
263	JUND	MAPK9	[activation]
264	CTSG	F2RL2	[activation]
265	PDPK1	PKN1	[activation]
266	EFNB1	EPHB2	[activation]
267	CAMK2G	STAT1	[activation]
268	F2RL2	F2RL3	[activation]
269	CDK2	SKP1	[inhibition]
270	MDM2	PPP2R4	[activation]
271	PXN	SDC4	[activation]
272	MAPK12	MAPT	[activation]
273	LEF1	NLK	[inhibition]
274	MTOR	TRAF2	[activation]
275	EPHA2	PTK2	[activation]
276	AURKA	PRKACA	[activation]
277	EP300	TWIST1	[activation]
278	CASP8	IKBKB	[activation]
279	ACTG1	VASP	[activation]
280	ARHGEF12	GNA13	[activation]
281	CHEK2	RNF8	[activation]
282	BDKRB1	GNAQ	[activation]
283	IL2RG	STAT1	[activation]
284	NCF1	PRKCB	[activation]
285	EIF4E	EIF4EBP1	[inhibition]
286	BRAF	MAPK1	[activation]
287	GRB2	MAPK1	[activation]
288	FGF4	FGFR1	[activation]
289	AKT1	TCL1A	[activation]
290	ATM	MDM4	[activation]
291	GNAI2	OPRD1	[activation]
292	CEBPA	EP300	[activation]
293	PRKCE	SRC	[activation]
294	CSF2	CSF2RA	[activation]
295	NOS2	SRC	[activation]
296	CAPN1	CDK5R1	[activation]
297	MPL	THPO	[activation]
298	EP300	SIRT1	[activation]
299	RIPK2	TRAF6	[activation]
300	ARHGEF2	RHOA	[activation]
301	KRAS	RALGDS	[activation]
302	NLGN1	NRXN2	[activation]
303	LCK	PTK2	[activation]
304	PRIM2	RPA2	[activation]

305	CCND3	RBL2	[inhibition]
306	C1S	SERPING1	[inhibition]
307	CASP7	DFFA	[activation]
308	RASGRF1	RRAS2	[activation]
309	CSF2	IL3RA	[activation]
310	IRS2	JAK2	[activation]
311	PLCB1	PRKCA	[activation]
312	ACACB	PRKAA2	[inhibition]
313	CD3E	PIK3R1	[activation]
314	CD36	THBS1	[activation]
315	PRKCA	RARA	[activation]
316	ATM	FANCD2	[activation]
317	ARF1	ARFGAP1	[activation]
318	F2	F2RL3	[activation]
319	HDAC1	RBPJ	[inhibition]
320	ITGB3	SRC	[activation]
321	NGFR	NTRK2	[activation;inhibition]
322	ACVR1B	INHBA	[activation]
323	PARD6A	RAC1	[activation]
324	RBL1	SP1	[activation]
325	ACTB	ARPC1B	[activation]
326	EPOR	GAB1	[activation]
327	IRAK1	TAB1	[activation;inhibition]
328	BAIAP2	RAC1	[activation]
329	GJA1	MAPK7	[inhibition]
330	BRAF	MRAS	[activation]
331	CCR5	GNAI2	[activation]
332	FGF4	FGFR2	[activation]
333	NRAS	RASGRP2	[activation]
334	MAP3K7	MAP4K1	[activation]
335	SETDB1	SUV39H1	[activation]
336	DUSP10	MAPK3	[inhibition]
337	AKT1	NR4A1	[inhibition]
338	C3	CR1	[activation]
339	MAPK3	PRKCZ	[activation]
340	HSPB1	MAPKAPK2	[activation]
341	GZMA	SET	[activation]
342	CD4	HLA-DQA2	[activation]
343	MAP3K1	MAPK9	[activation]
344	CDK4	PRMT5	[activation]
345	MAPK14	NCF1	[activation]
346	AR	KAT2B	[activation]
347	CASP7	VIM	[activation]
348	KDR	PTPN11	[activation]
349	GNAI2	IGF1R	[activation]
350	IL2	IL2RG	[activation]
351	HDAC1	JUN	[activation]
352	IL2RG	STAT5A	[activation]
353	ARF6	PLD1	[activation]
354	F2	PROS1	[activation;inhibition]
355	CDK6	PCNA	[inhibition]
356	FGF3	FGFR2	[activation]
357	ERBB4	ITCH	[activation]
358	CDK1	GORASP1	[activation]
359	ANAPC10	ANAPC11	[activation]
360	CD14	IRAK3	[inhibition]
361	C2	C5	[activation]
362	CDC42	PRKCZ	[activation]
363	FER	JUP	[activation;inhibition]
364	F11	SERPINA5	[inhibition]
365	NFKBIA	SRC	[activation]
366	GNAI2	P2RY12	[activation]
367	DAPP1	LCK	[activation]
368	MLLT4	RAP1A	[activation]
369	GRB2	NGFR	[activation]
370	IKBKG	TRAF6	[activation]
371	EZR	ROCK1	[activation]
372	RBL1	TFDP1	[inhibition]
373	MAPK8	RAF1	[activation]
374	AKT1	MTCP1	[activation]
375	CSK	PTPRC	[activation]
376	PARD6B	RAC1	[activation]
377	IL7R	TSLP	[activation]
378	CD44	COL1A2	[activation]
379	RCHY1	TP73	[activation;inhibition]
380	DOK1	FYN	[activation]

IL1RAP	IRAK1	[activation;inhibition]
ROBO1	SRGAP2	[activation]
CDK1	TP73	[activation;inhibition]
PDGFRB	PIK3CA	[activation]
FANCD2	USP1	[activation]
TNF	TNFRSF1A	[activation]
PLAU	PLG	[activation]
NGFR	NTRK1	[activation]
CEBPB	NFKB1	[activation]
MAPK1	RPS6KA4	[activation]
INSR	PTPN2	[activation]
EPHA2	GRB2	[activation]
KAT2A	RBPJ	[activation]
MAPK8	RAC1	[activation]
RAB5A	USP6NL	[activation]
ERBB3	PIK3R3	[activation]
ATM	MDC1	[activation]
IL12B	IL12RB2	[activation]
NTRK2	SH2B2	[activation]
PAK4	RAC1	[activation]
IKBKB	PEBP1	[inhibition]
CAMK2G	GRIA1	[activation]
CD8A	HLA-G	[activation]
GNAI3	RGS20	[activation;inhibition]
IQGAP3	RAC1	[activation]
LYN	PLCG1	[activation]
PARD3	PVRL1	[activation]
CSK	FGR	[activation]
PTK2	SYK	[activation]
ABL1	EPHB2	[activation]
PORCN	WNT4	[activation]
CASP8	IKBKG	[activation]
ELMO1	RHOG	[activation]
PLXNB1	SEMA4B	[activation]
SFRP2	WNT1	[activation;inhibition]
ITK	LAT	[activation]
APC	ARHGEF4	[activation]
TNFRSF11A	TNFSF11	[activation]
ARRB2	CSNK1A1L	[inhibition]
MAP3K7	SMAD7	[activation]
MAPK13	MAPT	[activation]
GNAQ	HTR2A	[activation]
NGF	PLG	[activation]
PTPN6	VAV1	[activation;inhibition]
CDC42	RAC1	[activation]
PDPK1	SGK2	[activation]
HRAS	RASSF5	[activation]
ADCYAP1	ADCYAP1R1	[activation]
GJA1	MAPK1	[inhibition]
BRAF	RAP1A	[activation]
CTTN	FER	[activation;inhibition]
MAPK14	SHC1	[activation]
PLCB3	PRKG1	[inhibition]
CASP6	TOP1	[activation]
PDGFRB	PTK2	[activation]
BCL2L11	MAPK9	[activation]
JAK3	STAM	[activation]
DAPP1	LYN	[activation]
CDK7	RARA	[activation]
FGF6	FGFR3	[activation]
TGFB3	TGFBR1	[activation]
EPOR	STAT5B	[activation]
DCLRE1C	TP53BP1	[activation]
TICAM1	TICAM2	[activation]
MS4A2	PTPN11	[activation]
GNAI1	IGF1R	[activation]
ACTN1	PTK2	[activation]
LRP6	WNT1	[activation;inhibition]
CDC42	WAS	[activation]
CDH1	GNA12	[activation]
PRKG1	RGS2	[activation;inhibition]
LYN	RASA1	[activation]
EGF	GRB2	[activation]
IL2RB	JAK3	[inhibition]
LCK	STAT5A	[activation]
HRAS	TIAM1	[activation]

457	RAP1A	RAPGEF6	[activation]
458	RAC1	TLR2	[activation]
459	MAP2K1	PAK1	[activation]
460	HCST	PIK3R1	[activation]
461	PIK3AP1	PIK3R1	[activation]
462	IL12B	IL12RB1	[activation]
463	JAK2	STAM	[activation]
464	LYN	PTPRC	[activation]
465	IRF3	TICAM1	[activation]
466	CHRM1	GNAI2	[activation]
467	IL23R	STAT3	[activation]
468	EFNB2	EPHB6	[activation]
469	CAMK2D	STAT1	[activation]
470	LYN	SPHK1	[activation]
471	RAC1	WAS	[activation]
472	FGFR1	GRB2	[activation]
473	RB1	TFDP2	[inhibition]
474	MTOR	RPS6KB2	[activation]
475	MTOR	RPTOR	[activation;inhibition]
476	CD3E	PIK3R2	[activation]
477	CDC42	DOCK7	[activation]
478	GABBR1	GNAO1	[activation;inhibition]
479	RBL1	TFDP2	[inhibition]
480	PTK2	RET	[activation;inhibition]
481	GAB1	MAPK1	[activation]
482	AKT1	PTEN	[activation;inhibition]
483	IKBKG	RIPK1	[activation]
484	PML	TGFBR1	[inhibition]
485	CD28	CD4	[activation]
486	ACVR1C	INHBB	[activation]
487	PRKCA	RHOA	[activation]
488	CD8A	LAT	[activation]
489	NFKB1	RIPK1	[activation]
490	ERO1L	P4HB	[activation]
491	PORCN	WNT1	[activation;inhibition]
492	CREBBP	SMAD4	[activation]
493	LIPE	PRKACA	[activation]
494	EFNB1	EPHB1	[activation]
495	NOTCH1	PSEN2	[activation]
496	MLST8	RPTOR	[activation;inhibition]
497	NRAS	PIK3CA	[activation]
498	HIST1H4A	RB1	[activation;inhibition]
499	ITGB4	PTK2	[activation]
500	HDAC1	NOTCH1	[activation]
501	ACACA	PRKAA2	[activation;inhibition]
502	CXCR5	GNAI2	[activation]
503	IRAK1	IRAK4	[activation;inhibition]
504	MAPK1	PRKCZ	[activation]
505	BUB1B	CDC20	[inhibition]
506	GH2	GHR	[activation]
507	CASP3	LMNB1	[activation]
508	CASP8	RIPK2	[activation]
509	EZR	SDC2	[activation]
510	PF4	THBD	[activation]
511	CCL28	CCR10	[activation]
512	EIF2B5	EIF2S2	[activation]
513	SMURF1	TGFBR1	[inhibition]
514	CHN2	RAC1	[activation]
515	CSF1R	PIK3R2	[activation]
516	PPP2CB	PRKCB	[inhibition]
517	PLCB1	PRKCD	[activation]
518	CTTN	DNM2	[activation]
519	CCND1	PRMT5	[activation]
520	LIMK1	PAK4	[activation;inhibition]
521	PTPN6	SYK	[activation;inhibition]
522	CALM1	EGFR	[activation]
523	ATR	CHEK2	[activation]
524	ILK	PARVA	[activation]
525	CD8A	HLA-B	[activation]
526	CXCR2	GNAI3	[activation]
527	AKT1	PFKFB2	[activation]
528	HLA-C	KIR2DL2	[activation]
529	AR	SRY	[activation]
530	ELK1	FOS	[activation]
531	IGF1R	PRKD1	[activation]
532	ERBB3	PIK3CA	[activation]

533	EFNA3	EPHA2	[activation]
534	EGFR	GSN	[activation]
535	CRK	EGFR	[activation]
536	E2F1	PRMT5	[activation]
537	PIK3R1	TYK2	[activation]
538	CRK	PXN	[activation]
539	ILK	PPP1R12A	[activation]
540	CIITA	NFYB	[activation]
541	SNCA	TOR1A	[inhibition]
542	RIPK1	TRADD	[activation]
543	FGFR1	PIK3R1	[activation]
544	CHRM2	GNG2	[activation]
545	JAK2	PTK2	[activation]
546	RDX	ROCK1	[activation]
547	DUSP10	MAPK9	[inhibition]
548	GNAI1	MTNR1A	[activation]
549	F10	F3	[activation]
550	CXCL16	CXCR6	[activation]
551	CHUK	MAVS	[activation]
552	CHEK2	RAD50	[activation]
553	FOXP3	NFATC2	[activation]
554	PRKCD	SDC4	[activation;inhibition]
555	GNAI1	RGS7	[activation;inhibition]
556	EDA	EDA2R	[activation]
557	DUSP19	MAPK9	[inhibition]
558	MAPK3	STAT3	[activation]
559	HLA-A	KIR3DL2	[activation]
560	BDKRB2	GNA11	[activation]
561	MAPK8	RET	[activation]
562	IL2RB	PIK3R1	[activation]
563	CALM1	JAK2	[activation]
564	GSK3B	TSC2	[activation;inhibition]
565	IFNGR1	SOCS1	[inhibition]
566	TNFRSF12A	TNFSF12	[activation]
567	CDC42	PLD1	[activation]
568	ADCY6	GNAS	[activation]
569	ATF1	RPS6KA5	[activation]
570	ITK	LCK	[activation]
571	CALM1	MYLK	[activation]
572	CXCL3	CXCR1	[activation]
573	IL1A	IL1RAP	[activation]
574	ACTA1	RAC1	[activation]
575	KALRN	RAC1	[activation]
576	PRKACA	RASGRF1	[activation]
577	ADRB2	GNAS	[activation]
578	CCL2	CCR10	[activation]
579	GHR	PTPN6	[activation;inhibition]
580	CBL	EPHA2	[activation]
581	ITGB3	PXN	[activation]
582	NFKB1	PRKACA	[activation]
583	FES	PLXNA1	[activation]
584	EFNB2	EPHB1	[activation]
585	FGF17	FGFR1	[activation]
586	AKT1	ILK	[activation]
587	FYN	SNCA	[activation]
588	FLT1	GRB2	[activation]
589	CHUK	MAP3K14	[activation]
590	FZD6	WNT4	[activation]
591	EZR	FAS	[activation]
592	ACTR2	WASF1	[activation]
593	PDPK1	RALGDS	[activation]
594	ASIP	MC1R	[inhibition]
595	LIF	LIFR	[activation]
596	DUSP10	MAPK11	[inhibition]
597	GNRH1	GNRHR	[activation]
598	IL2RB	PTPN6	[activation;inhibition]
599	CDC42	FGD1	[activation]
600	ADAM17	ERBB4	[activation]
601	CSNK1D	CTNNB1	[activation]
602	ACVR2B	SMAD2	[activation]
603	EEF2K	PRKAA2	[activation]
604	CASP6	LMNB1	[activation]
605	PIK3R1	RRAS	[activation]
606	PDGFRB	STAT5B	[activation]
607	KRAS	PIK3CG	[activation]
608	HRAS	RGL1	[activation]

609	CASP8	TRAF2	[activation]
610	CXCL2	CXCR1	[activation]
611	KLRK1	MICB	[activation]
612	PPP2R1B	RELA	[activation]
613	NOTCH3	PSEN1	[activation]
614	FADD	MAPK8	[activation]
615	KSR1	RAF1	[activation]
616	CASP10	CASP3	[activation]
617	CALM1	PDE1A	[activation]
618	RYR2	SRI	[activation]
619	CREB1	CREM	[inhibition]
620	BTC	EGFR	[activation]
621	CCL4	CCR8	[activation]
622	AKT1	STAT1	[activation]
623	ADAM17	NOTCH1	[activation]
624	DUSP16	MAPK11	[inhibition]
625	CTSG	SERPINB13	[inhibition]
626	INHA	INHBB	[activation]
627	FGB	SERPINA5	[inhibition]
628	CSF2RB	PTPN6	[activation;inhibition]
629	JAK3	PIK3R1	[activation]
630	CCL14	CCR1	[activation]
631	DVL2	FZD4	[activation]
632	ARF1	PIP5K1A	[activation]
633	LAT	SYK	[activation]
634	DHH	PTCH1	[activation]
635	CASP8	CRADD	[activation]
636	SMAD1	SMAD3	[activation]
637	NUP50	RAN	[activation]
638	FGF18	FGFR1	[activation]
639	PDGFD	PDGFRB	[activation]
640	FLT3	PIK3R1	[activation]
641	GNAQ	TSHR	[activation]
642	CXCR4	STAT2	[activation]
643	ANAPC11	CDC27	[activation]
644	MAP2K1	MAPK14	[activation]
645	PRKACA	RAP1A	[activation]
646	APP	CASP8	[activation]
647	CD47	THBS1	[activation]
648	CSNK1E	DVL3	[activation]
649	CD44	VAV2	[activation]
650	STX1A	VAMP2	[activation]
651	MAP3K14	TRAF6	[activation]
652	FGF7	FGFR1	[activation]
653	ARHGDIB	RHOA	[activation]
654	IRS4	PIK3CA	[activation]
655	NCF1	RAC1	[activation]
656	HLA-B	KLRD1	[activation]
657	KPNA1	NUP62	[activation]
658	EFNA4	EPHA8	[activation]
659	EP300	PML	[activation]
660	NCK2	WAS	[activation]
661	GAB1	PIK3CG	[activation]
662	CTNNA1	TJP1	[activation]
663	NTRK1	RASGRF1	[activation]
664	TNF	TNFRSF1B	[activation]
665	GNAI1	OPRM1	[activation]
666	FGFR1	PIK3R2	[activation]
667	PIK3AP1	SYK	[activation]
668	FURIN	NOTCH1	[activation]
669	RIMS1	SNAP25	[activation]
670	EIF2B3	EIF2S1	[activation]
671	C3	ITGAM	[activation]
672	EIF4EBP1	RPS6KA5	[activation]
673	IL13RA2	IL4	[activation]
674	OSBP	VAPB	[activation]
675	CD247	FYN	[activation]
676	CTNNB1	PTPN6	[activation;inhibition]
677	CHRM3	GNAQ	[activation]
678	BID	CASP10	[activation]
679	CREB3L1	CREM	[inhibition]
680	AKT1	NR3C1	[activation]
681	EPHB2	RASA1	[activation]
682	IL1B	IL1R2	[activation]
683	RASSF5	RRAS	[activation]
684	GPC3	IGF2	[activation]

AKT1	BRCA1	[activation]
DOK1	SHC1	[activation]
AR	CASP8	[activation]
ATR	MCM2	[activation]
APBB1IP	RAP1A	[activation]
CSF1R	PIK3R1	[activation]
PRKCA	SRC	[activation]
EPHB2	SRC	[activation]
ARF1	ASAP2	[activation]
HRAS	INSR	[activation]
MMP9	TIMP3	[inhibition]
EFNA3	EPHA5	[activation]
MAP3K11	RAC1	[activation]
ITGB4	PRKCA	[activation]
ATM	MRE11A	[activation]
MYC	TP73	[activation;inhibition]
ARF6	PIP5K1A	[activation]
AVPR2	GNAS	[activation]
GRB2	INPP5D	[activation]
MAGED1	NGFR	[activation]
DOK1	RASA1	[activation]
PRKCI	RHOQ	[activation]
CCL23	CCR1	[activation]
HDAC3	RXRA	[inhibition]
AURKA	GSK3B	[activation]
CDC16	FZR1	[activation]
HIF1A	NAA10	[activation]
SRC	TIAM1	[activation]
GHR	PTPN11	[activation]
LCK	NFKBIA	[activation;inhibition]
IFNAR1	PTPN6	[activation;inhibition]
KCNJ1	SGK1	[activation]
JUN	STAT4	[activation]
FGF9	FGFR4	[activation]
CFLAR	TNFRSF10A	[inhibition]
ATP2B1	PRKACA	[activation]
ARF1	CYTH1	[activation]
RAF1	RRAS2	[activation]
MAPKAPK2	TH	[activation]
LAT	LCP2	[activation]
LAT	PLCG2	[activation]
IL1R1	IRAK1	[activation;inhibition]
GNAQ	HTR2C	[activation]
ERBB4	NRG3	[activation]
GAB2	SYK	[activation]
RIN1	RRAS2	[activation]
CREB1	GSK3B	[inhibition]
PTPN6	SH3BP2	[activation;inhibition]
GAB2	PIK3R1	[activation]
CXCR1	GNAI2	[activation]
DDX54	ESR2	[activation]
ACTG1	WASF1	[activation]
ADRA1B	GNAQ	[activation]
TGFBR1	ZFYVE9	[activation]
SHC1	SYK	[activation]
EFNA1	EPHA1	[activation]
GNAS	TSHR	[activation]
ANAPC11	FZR1	[activation]
GNAO1	GRM6	[activation]
FYB	FYN	[activation]
ADRA1B	GNA11	[activation]
ARRB2	DVL2	[activation]
EFNA2	EPHA2	[activation]
LCK	SHC1	[activation]
CYFIP1	EIF4E	[inhibition]
ATF1	RPS6KA4	[activation]
LFNG	NOTCH2	[activation]
BIRC2	MAP3K14	[activation;inhibition]
CACNB2	PRKACA	[activation]
EGFR	MAPK1	[activation]
MAPK11	MAPKAPK5	[activation]
ARHGEF6	RAC1	[activation]
MAPK10	RELA	[activation]
PIK3R1	TLR3	[activation]
ADAM17	TNF	[activation]
CD19	PIK3R1	[activation]

761	ANAPC1	ANAPC7	[activation]
762	HTT	RAC1	[activation]
763	CASP3	SLK	[activation]
764	XCL2	XCR1	[activation]
765	KRAS	RAF1	[activation]
766	IGF1R	PRKCD	[activation]
767	ARRB2	CXCR4	[activation]
768	PARK2	SNCA	[activation]
769	CTF1	IL6ST	[activation]
770	CBL	PTPN11	[activation;inhibition]
771	CAPN2	CDK5R1	[activation]
772	BCL2	CYCS	[inhibition]
773	NTRK1	SH2B1	[activation]
774	APP	MAPK13	[activation]
775	ESR1	SMAD3	[activation]
776	AKT1	RAC1	[activation]
777	KRAS	RASGRP2	[activation]
778	STAT5A	TEK	[activation]
779	PIK3R1	TEK	[activation]
780	AR	SRC	[activation]
781	PAK1	RAC1	[activation]
782	IGF1R	IRS4	[activation]
783	RAC1	TRIO	[activation]
784	CCL26	CCR1	[activation]
785	TNFRSF11B	TNFSF10	[activation]
786	ARHGAP17	RAC1	[activation]
787	ELK1	SRF	[activation]
788	CASP2	CASP7	[activation]
789	KRIT1	RAP1A	[activation]
790	CDC42	WASF2	[activation]
791	C2	C3	[activation;inhibition]
792	CRK	PDGFRB	[activation]
793	CHEK2	FOXM1	[activation]
794	MAPK14	MAPK8	[activation]
795	PTK2	RAC1	[activation]
796	AR	NR2C2	[activation]
797	F2	F9	[activation]
798	LAT	PTPN1	[activation]
799	CASP1	IL33	[activation]
800	RAPGEF4	RRAS2	[activation]
801	EPHA2	SHC1	[activation]
802	INHA	INHBA	[activation]
803	KIR2DL3	PTPN6	[activation;inhibition]
804	EP300	SKP2	[activation]
805	ELK1	MAPK9	[activation]
806	AMOT	WWTR1	[activation]
807	MAP3K14	RIPK1	[activation]
808	EXOC2	RALA	[activation]
809	CXCR4	STAT5B	[activation]
810	HRAS	RASGRP4	[activation]
811	CDC25A	CDK1	[activation]
812	NTRK1	PTPRC	[activation]
813	IL2RA	STAT5B	[activation]
814	GNAI2	TBXA2R	[activation;inhibition]
815	IKBKB	PRKCZ	[activation]
816	FFAR1	GNAQ	[activation]
817	AKT1	GRB10	[activation]
818	CCL7	CCR10	[activation]
819	LAT	PLCG1	[activation]
820	NCK1	PTK2	[activation]
821	BMP6	BMPR1B	[activation]
822	CCL28	CCR3	[activation]
823	TGFB1	TGFBR1	[activation]
824	HLA-E	KLRC1	[activation]
825	CASP9	DIABLO	[activation]
826	EIF2B1	EIF2S2	[activation]
827	ADCY3	CAMK2G	[inhibition]
828	PRKCH	SRC	[activation]
829	CCL16	CCR5	[activation]
830	AURKB	CENPA	[activation]
831	HIST1H3A	TAF1B	[activation]
832	CHUK	TNFRSF1A	[activation]
833	IKBKB	MAVS	[activation]
834	NOTCH4	PSEN1	[activation]
835	RIPK1	TNF	[activation]
836	CDC42	IQGAP2	[activation;inhibition]

837	NRP1	SEMA3C	[activation]
838	GNAI1	GNB1	[activation]
839	EFNA4	EPHA5	[activation]
840	DOCK1	RAC1	[activation]
841	CAB39	PRKAA1	[activation]
842	DOCK2	RAC1	[activation]
843	GRB2	LAT	[activation]
844	FGF8	FGFR4	[activation]
845	LRP8	RELN	[inhibition]
846	CAV1	IRS1	[activation]
847	BTK	PLCG1	[activation]
848	CD79A	PTPRC	[activation]
849	RAP1A	RAPGEF5	[activation]
850	AKT2	TSC2	[activation;inhibition]
851	ARNTL	PER3	[activation;inhibition]
852	KDR	SH2D2A	[activation]
853	ESR1	MAPK14	[activation]
854	GJA1	MAPK3	[inhibition]
855	BIRC5	CASP9	[activation]
856	MAP3K7	MAPK14	[activation]
857	FCGR2A	LAT	[activation]
858	BCAR1	PTPN1	[activation]
859	HSPG2	ITGA2	[activation]
860	ACTB	PFN2	[activation]
861	JAK3	PTPN6	[activation;inhibition]
862	PEBP1	PRKCZ	[activation]
863	IL12RB1	STAT4	[activation]
864	LEPR	STAT3	[activation]
865	PRIM2	RPA1	[activation]
866	LYN	MS4A2	[activation]
867	CXCR4	STAT1	[activation]
868	JUP	SRC	[activation]
869	MAPK14	MEF2C	[activation]
870	CD244	SH2D1B	[activation]
871	NOTCH3	PSEN2	[activation]
872	ARHGEF12	CD44	[activation]
873	CRTC2	YWHAZ	[activation]
874	CTNND1	CTTN	[activation]
875	STX1A	UNC13B	[activation]
876	EFNA3	EPHA4	[activation]
877	HLA-G	KLRD1	[activation]
878	HLA-E	KLRD1	[activation]
879	APOA5	LPL	[activation]
880	NCK1	PAK3	[activation]
881	ACVR1	INHBC	[activation]
882	AKT1	AKT2	[activation]
883	IRF7	MAVS	[activation]
884	RAN	RCC1	[activation]
885	CCNA2	TP73	[activation;inhibition]
886	ANAPC11	PTTG1	[inhibition]
887	PLD1	RHOA	[activation]
888	CEBPA	CREBBP	[activation]
889	MAP2K3	TAOK1	[activation]
890	CD28	LCK	[activation]
891	IRS1	PIK3CB	[activation]
892	GNA11	HTR2A	[activation]
893	ERBB4	HBEGF	[activation]
894	ADCY7	PRKCD	[activation]
895	PLCB1	RGS4	[activation;inhibition]
896	PDE3B	PRKACA	[activation]
897	IL1B	IL1RAP	[activation]
898	IL2RA	STAT3	[activation]
899	IL1A	IL1R1	[activation]
900	CDC42BPA	MYL2	[activation]
901	ACVR2B	INHBC	[activation]
902	GNAI2	OPRM1	[activation]
903	LFNG	NOTCH1	[activation]
904	PRKCI	SRC	[activation]
905	ARHGEF11	GNA13	[activation]
906	MFNG	NOTCH2	[activation]
907	TIRAP	TRAF6	[activation]
908	FYN	PLCG1	[activation]
909	ATR	BRCA1	[activation]
910	RHOA	VAV2	[activation]
911	E2F1	E2F6	[inhibition]
912	CHUK	PRKCQ	[activation]

913	CCL11	CCR2	[activation]
914	AKT1	PIK3R1	[activation]
915	GNAI3	HTR1A	[activation]
916	IL6ST	PTPN6	[activation;inhibition]
917	CDC20	MAD2L1	[inhibition]
918	DVL2	RAC1	[activation]
919	NFATC1	PIM1	[activation]
920	CREBBP	MYB	[activation]
921	LYN	PLCG2	[activation]
922	PLCB2	RAC1	[activation]
923	IKBKG	RELA	[activation]
924	L1CAM	NRP1	[activation]
925	NCOR2	RXRA	[inhibition]
926	ACTR2	WASF2	[activation]
927	STAT5B	TEK	[activation]
928	CCR5	GNAI1	[activation]
929	IL4R	PTPN6	[activation;inhibition]
930	CHN1	RAC1	[activation]
931	TNF	TNFAIP3	[activation]
932	IL12RB1	IL23A	[activation]
933	PLXNB3	SEMA4A	[activation]
934	BRCA1	NBN	[activation]
935	ACVR1B	SMAD2	[activation]
936	MAPK1	MKNK2	[activation]
937	EIF2B4	EIF2S2	[activation]
938	CBL	EGFR	[activation]
939	CYBB	RAC1	[activation]
940	TNF	TRAF2	[activation]
941	MAPK1	MAPK8	[activation]
942	INSR	SOCS2	[inhibition]
943	BCL10	CHUK	[activation]
944	IKBKB	MAP3K14	[activation]
945	MAPK14	PPARGC1A	[activation]
946	ETV1	MAPKAPK2	[activation]
947	LIMK1	PAK1	[activation]
948	IL6ST	PIK3CG	[activation]
949	CTSG	F2R	[activation]
950	CSNK1G1	FOXO1	[activation]
951	PRNP	STIP1	[activation]
952	AMOT	YAP1	[activation]
953	IFNA1	IFNAR1	[activation]
954	CCL3L1	CCR3	[activation]
955	MCM7	RAD17	[activation]
956	GNAI1	MTNR1B	[activation]
957	SELE	SELPLG	[activation]
958	F2R	GNAI2	[activation]
959	AKT1	S1PR1	[activation]
960	CDK5R1	PAK1	[activation]
961	BRK1	RAC1	[activation]
962	CSK	LCK	[activation;inhibition]
963	ACTB	WASF2	[activation]
964	NOTCH4	PSEN2	[activation]
965	MAP2K4	MAP3K7	[activation]
966	ITGA2B	TLN1	[activation]
967	CD79A	PTPN6	[activation;inhibition]
968	RAC1	WASF1	[activation]
969	RAP1B	RASSF5	[activation]
970	DTX1	NOTCH2	[activation]
971	CRK	MAPK8	[activation]
972	ANAPC1	CDC27	[activation]
973	MAPK8	NR3C1	[activation]
974	RANBP1	XPO1	[activation]
975	ESR2	MAPK11	[activation]
976	STAT2	TYK2	[activation]
977	AR	MDM2	[activation]
978	RALGDS	RRAS2	[activation]
979	CD3E	LAT	[activation]
980	PDC	PRKACA	[activation]
981	CCL14	CCR3	[activation]
982	CEBPB	MAPK3	[activation]
983	BMP5	NOG	[inhibition]
984	AKAP13	GNA12	[activation]
985	MAGI2	PTEN	[activation]
986	ARF6	ASAP2	[activation]
987	CREB1	MAPK14	[activation]
988	MAPK11	RPS6KA5	[activation]

989	CSNK1D	PER3	[activation;inhibition]
990	CTLA4	PTPN11	[activation]
991	SP1	SP3	[activation]
992	CD244	LAT	[activation]
993	SST	SSTR2	[activation]
994	ELK4	MAPK11	[activation]
995	F12	KLKB1	[activation]
996	CASP3	RFC1	[activation]
997	NUP50	XPO1	[activation]
998	CTNND1	PTPN6	[activation;inhibition]
999	PLCG2	TEC	[activation]
1000	GNAI2	MTNR1B	[activation]
1001	FYN	PTPRC	[activation]
1002	SRC	STAT5A	[activation]
1003	FGF18	FGFR4	[activation]
1004	PRKACA	STK11	[activation]
1005	ROBO1	SLIT1	[activation]
1006	GNAI2	RASD1	[activation]
1007	CASP3	TOP1	[activation]
1008	FGF6	FGFR4	[activation]
1009	SNAP23	VAMP1	[activation]
1010	JAK2	TEC	[activation]
1011	CRHR1	GNAI1	[activation]
1012	CDH1	GNA13	[activation]
1013	NOTCH2	PSEN2	[activation]
1014	PDPK1	RPS6KB2	[activation]
1015	GNA11	HTR2B	[activation]
1016	ARAF	RRAS	[activation]
1017	C1R	SERPING1	[inhibition]
1018	PRKCE	SLC25A4	[inhibition]
1019	NRP1	SEMA3F	[activation]
1020	PIK3R1	PTPN6	[activation;inhibition]
1021	FGR	SNCA	[activation]
1022	IGF1R	INS	[activation]
1023	FGF17	FGFR4	[activation]
1024	GNAI1	RGS20	[activation;inhibition]
1025	FZD5	WNT7A	[activation]
1026	AKT1	CREB1	[activation]
1027	NCOR1	PPARA	[inhibition]
1028	ADIPOQ	ADIPOR2	[activation]
1029	GNAQ	HTR2B	[activation]
1030	APBB1IP	PFN1	[activation]
1031	RAP1A	RASGRP2	[activation]
1032	LRP5	WNT1	[activation;inhibition]
1033	IFNAR1	IFNW1	[activation]
1034	AKT1	PRKCB	[activation]
1035	ANAPC4	FZR1	[activation]
1036	ARPC3	WASF2	[activation]
1037	PTPN11	SOCS1	[inhibition]
1038	CASP6	SATB1	[activation]
1039	MAPK1	MITF	[activation]
1040	CBLB	SPRY2	[inhibition]
1041	E4F1	TP53	[activation]
1042	NUP153	SMAD2	[activation]
1043	PML	TGFBR2	[inhibition]
1044	CACNG2	PRKACA	[activation]
1045	E2F4	SMAD2	[activation;inhibition]
1046	ITGB1	SPP1	[activation]
1047	APC	PPP2R5A	[activation]
1048	GRB2	SHC3	[activation]
1049	SHC1	SRC	[activation]
1050	LCP2	PIK3R1	[activation]
1051	IRS2	PTPRF	[inhibition]
1052	ATF1	PRKACA	[activation]
1053	EEF2K	RPS6KB1	[activation]
1054	CD19	CD79B	[activation]
1055	ADRBK1	CCR5	[activation]
1056	FCGR2A	HCK	[activation]
1057	GNAS	PTGIR	[activation]
1058	HES1	STAT3	[activation]
1059	CD44	COL1A1	[activation]
1060	ACTB	EZR	[activation]
1061	EFNA5	EPHA8	[activation]
1062	EFNA1	EPHA6	[activation]
1063	IKBKG	TNF	[activation]
1064	ARHGEF6	PARVB	[activation]

1065	HHIP	IHH	[inhibition]
1066	JAM3	PARD3	[activation]
1067	BTC	ERBB4	[activation]
1068	CHEK1	RAD51	[activation]
1069	CASP6	LMNA	[activation]
1070	NCK1	RAC1	[activation]
1071	TSHB	TSHR	[activation]
1072	CTNNB1	HNF1A	[activation]
1073	ESR1	NCOA1	[activation]
1074	ABL1	CDK5	[activation]
1075	CDC42	RAC2	[activation]
1076	ADCY5	PRKCA	[activation]
1077	SNCA	SYK	[activation]
1078	FRS2	SOS1	[activation]
1079	CTBP1	EP300	[activation]
1080	MRAS	RAF1	[activation]
1081	F2R	GNA12	[activation]
1082	MKNK1	PLA2G4A	[activation]
1083	HGS	MET	[activation]
1084	C4B	C4BPA	[inhibition]
1085	ADRB2	GNAI2	[activation]
1086	DAB1	LRP8	[activation]
1087	CD44	TIAM1	[activation]
1088	BRCA1	CHEK2	[activation]
1089	PRKCI	RAC1	[activation]
1090	CXCL6	CXCR2	[activation]
1091	LCK	PTPN11	[activation;inhibition]
1092	GRB2	TEK	[activation]
1093	CCL26	CCR3	[activation]
1094	MAPK1	NOTCH1	[activation]
1095	MAPK9	SMAD3	[activation]
1096	MAPT	MARK2	[activation]
1097	F13A1	FGG	[activation]
1098	CDK2	FOXM1	[activation]
1099	RAC1	STAT3	[activation]
1100	SRPK1	SRSF8	[activation]
1101	ATM	RAD9A	[activation]
1102	CAV1	PRKACA	[activation]
1103	TNFRSF17	TNFSF13	[activation]
1104	AGAP2	PIK3R1	[activation]
1105	GNB1	KCNJ3	[activation]
1106	EP300	SMAD3	[activation]
1107	CBL	INSR	[activation]
1108	CD19	LYN	[activation]
1109	PTN	SDC3	[activation]
1110	MDM2	RANBP2	[activation]
1111	MAPK14	STAT1	[activation]
1112	PTPN2	SHC1	[activation]
1113	AKT1	PDE3B	[activation]
1114	FGA	SERPINA5	[inhibition]
1115	CHUK	PRKCI	[activation]
1116	NLGN3	NRXN3	[activation]
1117	EPOR	PTPN6	[activation;inhibition]
1118	BCR	RAC1	[activation]
1119	GRK5	SNCA	[activation]
1120	HRAS	PLCE1	[activation]
1121	MAPK3	MYC	[activation]
1122	HIST1H4A	TAF1B	[activation]
1123	FGF17	FGFR3	[activation]
1124	DNM2	KDR	[activation]
1125	EGFR	PTPN6	[activation;inhibition]
1126	ATF2	JDP2	[activation]
1127	PORCN	WNT3A	[activation]
1128	CEBPB	RELA	[activation]
1129	EP400	TP53	[activation]
1130	ESR1	SMAD4	[activation]
1131	CHEK2	PLK3	[activation]
1132	F2	PLAU	[activation]
1133	GSK3B	JUN	[activation]
1134	ATM	BRCA1	[activation]
1135	KLC1	SPAG9	[inhibition]
1136	FCGR1A	HCK	[activation]
1137	MAP2K6	MAP3K7	[activation]
1138	MYB	SKI	[activation]
1139	DUSP7	MAPK8	[inhibition]
1140	HSP90AA1	PTK2	[activation]

1141	ATM	SMC1A	[activation]
1142	CDC25B	MAPKAPK2	[activation]
1143	CAMK4	CAMKK1	[activation]
1144	HRAS	VAV1	[activation]
1145	CREM	PRKACA	[activation]
1146	ACVR1B	SMAD3	[activation]
1147	DUSP16	MAPK9	[inhibition]
1148	CEBPD	RELA	[activation]
1149	DVL1	RAC1	[activation]
1150	CSNK1A1	TP53	[activation]
1151	ADAM10	DLL1	[activation]
1152	MAP3K14	MAPK3	[activation;inhibition]
1153	CALM1	ITPKA	[activation]
1154	ADRBK2	OPRM1	[activation]
1155	KDR	PLCG2	[activation]
1156	MYC	NFYC	[activation]
1157	FZD9	WNT7A	[activation;inhibition]
1158	EFNB2	EPHB2	[activation]
1159	DPYSL2	GSK3B	[activation]
1160	CSK	HCK	[activation]
1161	F2R	PLG	[activation]
1162	RAPGEF1	RHOQ	[activation]
1163	FGFR2	PIK3R1	[activation]
1164	SMAD4	TGFBR2	[activation]
1165	DLL4	NOTCH1	[activation]
1166	E2F1	TP53	[activation]
1167	PRKCA	RAF1	[activation]
1168	EFNA1	EPHA7	[activation]
1169	ARF6	ASAP3	[activation]
1170	HCK	PIK3R1	[activation]
1171	KIT	STAT5A	[activation]
1172	PLD1	PRKCA	[activation]
1173	ACTB	WASF1	[activation]
1174	CREB1	RPS6KA4	[activation]
1175	FRS2	SRC	[activation]
1176	IRAK1	PPP2CA	[inhibition]
1177	FLT3	FLT3LG	[activation]
1178	PIK3CA	PRKCD	[activation]
1179	ANAPC1	CDC20	[activation]
1180	ACVR1	INHBB	[activation]
1181	AVP	AVPR2	[activation]
1182	MAP3K14	TRAF3	[activation]
1183	PLXNA1	RND1	[activation]
1184	ATF2	MAPK1	[activation]
1185	ARHGEF4	RAC1	[activation]
1186	ARAF	RRAS2	[activation]
1187	RAD51	RAD51C	[activation]
1188	PFKFB2	PRKAA2	[activation]
1189	CTNND1	PTPRM	[activation]
1190	IKBKG	MYD88	[activation]
1191	ARHGEF3	RHOA	[activation]
1192	CD226	PVR	[activation]
1193	ERBB4	YAP1	[activation]
1194	EPHB2	RRAS	[activation]
1195	IRF7	TICAM1	[activation]
1196	EP300	MYB	[activation]
1197	DRD2	GNAI2	[activation]
1198	FANCC	FANCD2	[activation]
1199	ETS1	MAPK3	[activation]
1200	LSP1	MAPKAPK2	[activation]
1201	CAV1	FYN	[activation]
1202	ATF4	EP300	[activation]
1203	NOTCH3	RBPJ	[activation]
1204	MAPK3	MAPK8	[activation]
1205	ANAPC11	ANAPC2	[activation]
1206	KRAS	PIK3R2	[activation]
1207	MLLT4	RRAS	[activation]
1208	PIK3R1	PLCG1	[activation]
1209	ESR1	MAPK3	[activation]
1210	EEF2K	PRKAA1	[activation]
1211	F2	SERPINC1	[inhibition]
1212	JAK1	PIK3R1	[activation]
1213	KLK6	SNCA	[activation]
1214	JUND	MAPK8	[activation]
1215	AKT2	MTCP1	[activation]
1216	CAMK2A	NOS1	[activation]

1217	IKBKB	IRAK1	[activation;inhibition]
1218	IL1RAP	TRAF6	[activation]
1219	CNR1	GNAI1	[activation]
1220	IL6ST	LIF	[activation]
1221	SMPD3	TNF	[activation]
1222	PAK3	RAC1	[activation]
1223	CCR2	JAK2	[activation]
1224	ADCY5	PRKACA	[activation]
1225	ANGPT1	ANGPT2	[inhibition]
1226	CLOCK	PER3	[activation;inhibition]
1227	MAP2K6	MAPK13	[activation]
1228	F2	F2RL2	[activation]
1229	FRS2	MAPK1	[activation]
1230	ARHGEF7	RAC1	[activation]
1231	NOTCH2	PSEN1	[activation]
1232	NTRK1	SHC2	[activation]
1233	F2	PROC	[activation;inhibition]
1234	CSK	PAG1	[activation]
1235	HRAS	MLLT4	[activation]
1236	NOS1	PRKCA	[activation]
1237	JAK1	PRKCZ	[activation]
1238	XCL1	XCR1	[activation]
1239	DNAJB1	DNAJC3	[inhibition]
1240	FGF1	FGFR4	[activation]
1241	CHRM2	GNA14	[activation]
1242	PIK3R1	RET	[activation]
1243	BMP5	BMPR1A	[activation]
1244	PIK3R1	RAC1	[activation]
1245	IRS2	PIK3R2	[activation]
1246	ADRB2	ARRB1	[activation]
1247	PAG1	PTPN11	[activation]
1248	GNAI3	LPAR1	[activation]
1249	HIST2H3A	TAF1B	[activation]
1250	SFRP1	WNT1	[activation;inhibition]
1251	IRS1	MAPK1	[activation]
1252	KIT	SH2B2	[activation]
1253	ITGB2	PTK2	[activation]
1254	CCND1	RBL2	[inhibition]
1255	CACNA1C	PRKACA	[activation]
1256	NLGN3	NRXN2	[activation]
1257	ADCY5	GNAS	[activation]
1258	CDK1	CSNK2B	[activation]
1259	ANAPC1	CDC23	[activation]
1260	ERBB4	NRG4	[activation]
1261	PROC	THBD	[activation]
1262	GNB1	MTNR1A	[activation]
1263	DDX58	TBK1	[activation]
1264	CASP6	KRT18	[activation]
1265	ATM	KAT5	[activation]
1266	MAP2K6	TRAF6	[activation]
1267	PDGFRB	PTPN2	[activation]
1268	CREB1	MAPKAPK2	[activation]
1269	AKT2	CHUK	[activation]
1270	IKBKG	IRAK4	[activation]
1271	TNFRSF13B	TNFSF13	[activation]
1272	ATM	CHEK2	[activation]
1273	NOD1	SUGT1	[activation]
1274	ACTB	PRKCD	[activation]
1275	RAC1	VAV1	[activation]
1276	ACVR2A	INHBC	[activation]
1277	OPHN1	RHOA	[activation]
1278	TGFBR2	ZFYVE9	[activation]
1279	GRB2	MS4A2	[activation]
1280	EPHB2	SYNJ1	[activation]
1281	LIMK2	ROCK1	[activation]
1282	ITGB1	PTK2	[activation]
1283	FGF3	FGFR3	[activation]
1284	PPP2CA	RPS6KB1	[activation]
1285	DNAJC3	EIF2AK2	[inhibition]
1286	IL13	IL13RA2	[activation]
1287	KSR1	MAPK1	[activation]
1288	EGFR	MAP2K2	[activation]
1289	IKBKG	PRKCQ	[activation]
1290	MAPK1	NR3C1	[activation]
1291	FZD9	WNT1	[activation;inhibition]
1292	GRIA3	LYN	[activation]

1293	OSM	OSMR	[activation]
1294	MAP4K5	TRAF2	[activation]
1295	ARHGEF12	GNA12	[activation]
1296	GAS1	SHH	[inhibition]
1297	CHEK2	MDC1	[activation]
1298	CTNNB1	PTPN1	[activation]
1299	ACVRL1	BAMBI	[inhibition]
1300	CTSG	F2RL1	[activation]
1301	MMP7	NGF	[activation]
1302	HLA-C	KIR2DS2	[activation]
1303	FZD1	WNT2	[activation]
1304	PORCN	WNT7B	[activation]
1305	LAT	LCK	[activation]
1306	LCP2	VAV3	[activation]
1307	CDK2	TP73	[activation;inhibition]
1308	CSNK1D	DVL3	[activation]
1309	PRKG1	RAF1	[activation]
1310	FGF17	FGFR2	[activation]
1311	EDNRB	GNA13	[activation]
1312	CXCR2	PPP2CA	[activation]
1313	EP300	JDP2	[activation]
1314	NCOR1	RXRA	[inhibition]
1315	ARHGEF25	RAC1	[activation]
1316	CSF2RB	STAT1	[activation]
1317	PPARA	RXRA	[inhibition]
1318	C3	C3AR1	[activation]
1319	APOC2	LPL	[activation]
1320	EP300	HDAC1	[activation]
1321	CFLAR	FAS	[inhibition]
1322	CREB1	EP300	[activation]
1323	ECSIT	TRAF6	[activation]
1324	NFATC2	PPP3R1	[activation]
1325	AKT1	CSF2RB	[activation]
1326	MAPK1	SNAI1	[activation;inhibition]
1327	ATF4	CREBBP	[activation]
1328	CALM1	ITPKB	[activation]
1329	CTNNB1	PTPRM	[activation]
1330	EFNA4	EPHA2	[activation]
1331	GHR	IRS1	[activation]
1332	IGF1R	SRC	[activation]
1333	E2F1	TFDP1	[inhibition]
1334	LCK	STAT1	[activation]
1335	ANAPC10	FZR1	[activation]
1336	FARP2	SRC	[activation]
1337	MAPK8	RB1	[activation]
1338	CHRM2	GNA11	[activation]
1339	IRAK1	TAB2	[activation;inhibition]
1340	BIRC3	TNF	[activation]
1341	CDK2	CDKN1C	[inhibition]
1342	CASP3	VIM	[activation]
1343	EXOC7	RHOQ	[activation]
1344	ACVR1B	INHBB	[activation]
1345	MAPK8	TRAF6	[activation]
1346	BRCA1	CDK2	[activation]
1347	MAPK3	PXN	[activation]
1348	GRB2	SHC4	[activation]
1349	RAPGEF3	RRAS2	[activation]
1350	ELK1	MAPK1	[activation]
1351	ACVR1B	INHBC	[activation]
1352	LCK	PRKCD	[activation]
1353	CALM1	KRAS	[activation]
1354	ACTB	RAC1	[activation]
1355	ELMO1	RAC1	[activation]
1356	HLA-C	KIR2DS1	[activation]
1357	GSN	PLCG1	[activation]
1358	PRKG1	VASP	[activation]
1359	CASP6	CASP8	[activation]
1360	GPC3	WNT3A	[activation]
1361	MAP2K3	MAPK12	[activation]
1362	CD28	PIK3CG	[activation]
1363	FYN	PLD2	[activation]
1364	IRAK1	MAP3K7	[activation;inhibition]
1365	AKT1	GAB2	[activation]
1366	ANAPC10	ANAPC7	[activation]
1367	ASF1A	TP53	[activation]
1368	EIF2AK2	NFKBIA	[activation]

F2	F2RL1	[activation]
PRKACA	VASP	[activation]
ANK1	ITPR3	[activation]
IFNGR1	PTPN11	[activation;inhibition]
AKT1	MST1R	[activation]
KPNA2	NUP153	[activation]
DUSP16	MAPK1	[inhibition]
CD247	CD28	[activation]
DRD2	GNAI3	[activation]
SIRT1	TP73	[activation;inhibition]
MAPK8	SHC1	[activation]
FZD8	WNT1	[activation]
INSR	PIK3R3	[activation]
BACE1	PSEN1	[activation]
E2F1	TFDP2	[inhibition]
MYO5A	RAB11A	[activation]
JAK2	PDGFRB	[activation]
GPC3	WNT7B	[activation]
NCK1	WAS	[activation]
DNAJC3	EIF2AK3	[inhibition]
ERBB4	MDM2	[activation]
FOXO3	YWHAG	[inhibition]
BIRC2	TRADD	[activation]
HLA-B	KIR3DL1	[activation]
EGFR	EPN1	[activation]
EFNA1	EPHA8	[activation]
CTNNA1	MLLT4	[activation]
NTRK3	SHC2	[activation]
IRF5	TRAF6	[activation]
EPHA2	RASA1	[activation]
CFLAR	FASLG	[inhibition]
CXCR4	GNAI1	[activation]
CHUK	IKBKG	[activation]
F2RL1	PLG	[activation]
NCF1	PRKCD	[activation]
INSR	SH2B2	[activation]
HDAC2	TP53	[activation]
EIF4G1	EIF5	[activation]
CD27	CD70	[activation]
GNA12	TBXA2R	[inhibition]
FXYD1	PRKACA	[activation]
EIF2AK1	EIF2S1	[activation]
ICOS	ICOSLG	[activation]
MMP3	PLG	[activation]
PDPK1	SGK3	[activation]
PTPN11	STAT3	[activation]
CTNND1	SRC	[activation]
AKT1	HSP90AB1	[activation]
GRB10	INSR	[activation]
TNFRSF11B	TNFSF11	[activation]
EIF3D	EIF5	[activation]
JUNB	MAPK14	[activation]
EFNA2	EPHA3	[activation]
APBB1IP	VASP	[activation]
LCP2	PLCG2	[activation]
IQGAP2	RAC1	[activation]
CTNNB1	TGFBR1	[activation]
EIF4B	RPS6KB1	[activation]
JUN	MAPK11	[activation]
ICAM3	ITGB2	[activation]
FLT4	PTK2	[activation]
CRHR1	GNAO1	[activation]
IKBKG	PRKCI	[activation]
CD8A	HLA-E	[activation]
PLCG1	VAV3	[activation]
IL3	PIK3CA	[activation]
HRAS	MAP2K1	[activation]
TGFB2	TGFBR2	[activation]
DCLRE1C	MRE11A	[activation]
ERBB2	PIK3R1	[activation]
ANAPC1	FZR1	[activation]
BMX	PTK2	[activation]
CREB1	RPS6KA5	[activation]
COL1A2	ITGA2B	[activation]
CHRM2	GNA15	[activation]
JUN	PPARG	[activation]

PFKFB2	PRKAA1	[activation]
LTBR	TNFSF14	[activation]
PRKCA	RAC1	[activation]
ARHGEF11	GNA12	[activation]
IRAK1	IRAK3	[activation;inhibition]
ANAPC11	ANAPC4	[activation]
PPP1R1B	PRKACA	[activation]
CDC23	FZR1	[activation]
HMGN1	RPS6KA5	[activation]
FCER1G	LYN	[activation]
CAMK4	CREBBP	[activation]
CDK5	FYN	[activation]
RB1	TFDP1	[inhibition]
PIK3R1	THRB	[activation]
MAP3K7	TGFBR1	[activation]
PRKACA	RELA	[activation]
ADORA1	GNAI2	[activation;inhibition]
CCL3L1	CCR1	[activation]
KIR2DL1	PTPN6	[activation;inhibition]
IRS1	PTPRF	[inhibition]
PAK6	RAC1	[activation]
AKT1	HSP90AA1	[activation]
AKT1	ESR1	[activation]
CREBBP	SMAD3	[activation]
CAMKK1	PRKAA1	[activation]
GNAO1	GPSM2	[inhibition]
CDK2	POLA1	[activation]
AR	HDAC1	[activation]
ARHGEF2	RAC1	[activation]
LCP2	WAS	[activation]
RANBP2	XPO1	[activation]
AKT1	YWHAZ	[activation]
LYN	SNCA	[activation]
CD28	ITK	[activation]
FZD9	WNT2	[activation;inhibition]
HRAS	PDGFB	[activation]
MAP3K7	MAPK8	[activation]
RAP1B	RAPGEF1	[activation]
DLL4	NOTCH4	[activation]
MDM2	PIAS1	[activation]
ADCY2	GNAS	[activation]
CDC42	PARD3	[activation]
DOK1	NCK1	[activation]
ERBB3	PIK3CB	[activation]
KPNA1	RANBP2	[activation]
DOK1	LCK	[activation]
JAK2	STAT2	[activation]
MAPK14	MITF	[activation]
MRAS	PIK3CA	[activation]
ARHGEF1	CD44	[activation]
EIF2B4	EIF2S1	[activation]
HIF1A	VHL	[inhibition]
CHEK2	TP53	[activation]
ADAM12	EGF	[activation]
EDNRB	GNA11	[activation]
AURKA	CDC25B	[activation]
RAC1	RAP1GDS1	[activation]
CCL14	CCR5	[activation]
CCNE1	RRN3	[activation]
F10	TFPI	[inhibition]
IL2RB	STAT5A	[activation]
MAPK14	TRAF6	[activation]
EGFR	MAP2K1	[activation]
MAPK3	PTPN5	[inhibition]
MYB	TRIM28	[activation]
DRD3	GNAI1	[activation]
ANXA2	PLG	[activation]
GRB10	GRB2	[activation]
GNB1	MTNR1B	[activation]
MYC	NFYB	[activation]
ECT2	PLK1	[activation]
DLL1	NOTCH3	[activation]
FGFR1	SHC1	[activation]
CYBA	RAC1	[activation]
NFKBIA	TNF	[activation]

GNAI1	PIK3R5	[activation]
GNA12	RASA2	[activation]
OPRD1	GNAI1	[activation]
CSF1R	IRS1	[activation]
TLR2	RAC1	[activation]
MTOR	PPARG	[activation]
RRAS2	TIAM1	[activation]
AKT3	CDKN1B	[inhibition]
TNFSF14	LTBR	[activation]
TLR4	NFKB1	[activation]
PRKD3	RAP1A	[activation]
JAK1	PIK3CA	[activation]
HRAS	BRAP	[activation]
IL10	STAT3	[activation]
TNFSF11	TNFRSF11A	[activation]
TNF	IKBKB	[activation]
CNTF	IL22RA2	[activation]
IHH	PTCH1	[activation]
STK11	FOXO6	[activation]
PRKD1	ITGAL	[activation]
ZAP70	LAT	[activation]
HTR7	GNB5	[activation]
CALML6	MYLK4	[activation]
RALBP1	ARF6	[activation]
RAP1A	MAP2K3	[activation]
CSF2	IL22RA2	[activation]
KDR	PIK3R5	[activation]
PRKACA	SLC9A1	[activation]
RAC1	MAPK8	[activation]
SOS1	RRAS2	[activation]
PLCG1	NOS3	[activation]
WIF1	WNT16	[inhibition]
MAPK1	SMAD4	[activation;inhibition]
CD14	TLR4	[activation]
CSNK2A1	NFKBIA	[activation]
CASP10	CASP7	[activation]
RAC1	PAK1	[activation]
CHRM1	GNAL	[activation]
PDPK1	PKN3	[activation]
CAMK4	PDE3A	[inhibition]
FBXW11	GLI1	[inhibition]
RASSF5	STK4	[activation]
ERBB4	SHC2	[activation]
SIPA1	RAP1A	[activation]
CDC42	ACTB	[activation]
STK11	PRKAG2	[activation]
PRKACA	FXYD1	[activation]
ADORA2A	GNAQ	[activation]
BTK	PLCG2	[activation]
TLN1	ITGA2B	[activation]
VASP	ACTB	[activation]
EIF4EBP1	EIF4E	[inhibition]
RRAS	PIK3R5	[activation]
LCP2	RAP1A	[activation]
BAD	BCL2	[inhibition]
SIPA1L1	RAP1A	[activation]
RAC1	PRKCI	[activation]
RRAS2	RGL2	[activation]
WNT16	LRP6	[activation]
ERBB4	PIK3R5	[activation]
WNT11	FZD10	[activation]
MAP3K14	MAP3K7	[activation]
CXXC4	DVL1	[inhibition]
ADIPOQ	ADIPOR1	[activation]
RRAS2	PLCE1	[activation]
RASGRP1	HRAS	[activation]
VAV3	RAC1	[activation]
CHRM1	GNAI1	[activation]
SIPA1L2	RAP1A	[activation]
PIK3AP1	PIK3CA	[activation]
RRAS2	RALBP1	[activation]
CDK2	FOXO6	[activation;inhibition]
PRKAG2	ACACA	[activation;inhibition]
PIK3R5	AKT3	[activation]
RASGRF1	MRAS	[activation]
RASGRF1	RRAS2	[activation]

TIAM1	RAC1	[activation]
RRAS	RALBP1	[activation]
MLLT4	CTNND1	[activation]
NTF3	NTRK1	[activation]
RRAS2	RIN1	[activation]
RIPK1	IKBKG	[activation]
PRKCA	PRKD3	[activation]
SFRP1	WNT16	[inhibition]
RRAS2	MOS	[activation]
CSNK1E	FOXO6	[activation;inhibition]
WNT16	FZD10	[activation]
TNF	TNFRSF1A	[activation]
SOS1	MRAS	[activation]
STAT3	FOXO6	[activation]
RAC1	PIK3CA	[activation]
RAP1A	APBB1IP	[activation]
CASP3	MAP3K1	[activation]
HRAS	RASSF5	[activation]
SOX17	LEF1	[inhibition]
PDPK1	CHUK	[activation]
PRKG1	RGS2	[activation;inhibition]
PRKACA	PPP1R1B	[activation]
HRAS	TIAM1	[activation]
AKT3	FOXO3	[inhibition]
DUSP3	MAPK8	[inhibition]
MAPK1	PLA2G4B	[activation]
NF1	RRAS2	[activation]
RAP1A	BRAF	[activation]
MRAS	PIK3R5	[activation]
ADCYAP1R1	GNAS	[activation]
NPY	CHRM1	[activation]
NKD1	DVL1	[inhibition]
PRKACA	CREB3	[activation]
RALA	EXOC2	[activation]
RASGRP3	RAP1A	[activation]
PIK3R5	RAC1	[activation]
PRKCE	KCNJ8	[activation]
ADRA1D	GNA13	[activation]
GDF7	BMPR1A	[activation]
RAP1A	PRKD1	[activation]
HRAS	PIK3R5	[activation]
RRAS2	BRAP	[inhibition]
AKT3	NR4A1	[inhibition]
PRKACA	RASGRF1	[activation]
MAPK1	CREB3	[activation]
RAPGEF5	RAP1A	[activation]
RRAS2	RAF1	[activation]
CASP10	CASP3	[activation]
BTC	EGFR	[activation]
ADAM17	NOTCH1	[activation]
CASP3	PAK1	[activation]
RALGDS	RAC1	[activation]
CALML6	RASGRF1	[activation]
CUL1	SMAD2	[inhibition]
GNAL	ADCY1	[activation]
RAP1A	VAV3	[activation]
PDPK1	AKT3	[activation]
TRAF2	BTK	[activation]
RALBP1	RAC1	[activation]
APBB1IP	TLN1	[activation]
ITPKA	CALML6	[activation]
MAPK1	FOXO6	[activation;inhibition]
RHOA	ACTB	[activation]
NLK	FOXO6	[inhibition]
TNFRSF1A	CASP3	[activation]
PRKACA	ATP2B1	[activation]
RALGDS	RALA	[activation]
RAP1A	ARAP3	[activation]
PRKACA	PPARA	[activation]
RASGRP1	RRAS2	[activation]
LAT	LCP2	[activation]
IL1R1	IRAK1	[activation;inhibition]
NOG	GDF7	[activation;inhibition]
GNAS	ADCY1	[activation]
KDR	PTK2	[activation]
SMAD2	BMPR1A	[activation;inhibition]

RAP1A	PLCE1	[activation]
TRADD	MAP3K7	[activation]
MAPK8	FOXO6	[activation]
WNT5A	FZD10	[activation]
CAMK2A	CREBBP	[activation]
CTNNB1	MAGI3	[activation]
KDR	CDC42	[activation]
MTCP1	AKT3	[activation]
PLCG1	PRKCA	[activation]
PRKACA	SOX9	[activation]
TGFBR2	RHOA	[activation]
LAT	PLCG1	[activation]
TGFB1	TGFBR1	[activation]
ERBB3	PIK3R5	[activation]
GAB1	PIK3R5	[activation]
RRAS2	BRAF	[activation]
PRKG1	BAD	[activation;inhibition]
AKT3	CHUK	[activation]
PRKG1	CACNA1C	[inhibition]
CAMKK2	PRKAG2	[activation]
SYK	BTK	[activation]
IL6R	STAT3	[activation]
RAP1A	VAV2	[activation]
PORCN	WNT16	[activation]
RAC1	MAP3K1	[activation]
KDR	SH2D2A	[activation]
DOCK4	RAP1A	[activation]
RAP1A	PIK3R5	[activation]
HBEGF	ERBB4	[activation]
ADORA1	GNAI1	[activation;inhibition]
AKT3	MDM2	[activation]
RASGRP2	RAP1A	[activation]
ADRA1D	GNA11	[activation]
RAPGEF3	RAP1A	[activation]
CSF1R	SHC2	[activation]
CDH1	RAPGEF1	[activation]
ADORA2A	GNAS	[activation]
RASGRF1	HRAS	[activation]
ARAP3	RHOA	[activation]
CSF1R	PLCB1	[activation]
IL1A	IL1R1	[activation]
LFNG	NOTCH1	[activation]
CSF1R	CRK	[activation]
DDIT4	TSC2	[activation;inhibition]
RAPGEF6	RAP1A	[activation]
BAMBI	FZD10	[activation]
HSPA1A	MAPK8	[inhibition]
DUSP3	MAPK14	[inhibition]
SFRP1	FZD10	[inhibition]
RRAS2	PIK3R5	[activation]
MAPK1	ELK1	[activation]
BDNF	NTRK1	[activation]
FSHB	ADCYAP1R1	[activation]
IL6	STAT3	[activation]
PTPN6	IL22RA2	[activation;inhibition]
STK3	LATS2	[activation]
KDR	PXN	[activation]
CASP8	CASP7	[activation]
MAPK1	TSC2	[activation;inhibition]
NCK1	PAK4	[activation]
MYC	CDKN2B	[activation;inhibition]
SRC	PIK3R5	[activation]
HHIP	IHH	[inhibition]
BTC	ERBB4	[activation]
CREBBP	RBPJL	[activation]
HDAC1	RBPJL	[inhibition]
PLCG1	PPP3CA	[activation]
CSNK1E	GSK3B	[activation;inhibition]
IL1R1	CASP3	[activation]
MAPK14	CEBPB	[activation]
IL24	IL22RA2	[activation]
NOTCH1	RBPJL	[activation]
RIPK1	MAP2K3	[activation]
RALA	MAPK8	[activation]
RASGRF1	RRAS	[activation]
PRKAG2	GYS1	[inhibition]

229	PRKG1	KCNMB2	[activation]
230	RAC1	RHOA	[activation]
231	EGFR	PIK3R5	[activation]
232	HRAS	PLCE1	[activation]
233	GNA13	RHOA	[activation]
234	RAPGEF2	RRAS2	[activation]
235	GNAI1	NOS3	[activation]
236	DVL1	GSK3B	[inhibition]
237	PRKCA	RAF1	[activation]
238	RRAS2	RGL1	[activation]
239	RAP1A	TIAM1	[activation]
240	NRG3	ERBB4	[activation]
241	RRAS2	RALGDS	[activation]
242	DLL3	NOTCH1	[activation]
243	RAB23	SMO	[inhibition]
244	AKT3	MTOR	[activation]
245	PDPK1	PRKCA	[activation]
246	CNR1	GNAI1	[activation]
247	RAC1	PAK4	[activation]
248	FOXO4	FASLG	[activation]
249	RHOA	ROCK2	[activation]
250	HRAS	MLLT4	[activation]
251	CAMK4	ADCY1	[inhibition]
252	CYSLTR1	GNA11	[activation]
253	NCSTN	NOTCH1	[activation]
254	AKT3	CREB3	[activation]
255	KRIT1	CTNNB1	[activation]
256	ARF6	RAC1	[activation]
257	TRAF2	PIK3R5	[activation]
258	RAP1A	SRC	[activation]
259	RAP1GAP	RAP1A	[activation]
260	NRG1	ERBB3	[activation]
261	PRKAG2	PFKFB1	[activation]
262	RPS6KB1	EIF4B	[activation]
263	MAPK1	ETS1	[activation]
264	VAV2	RAC1	[activation]
265	RAP1A	KRIT1	[activation]
266	PRKG1	RAF1	[activation]
267	PRKACA	NFKBIA	[activation]
268	PLCG1	PLA2G4B	[activation]
269	RAB10	SLC2A4	[activation]
270	RRAS2	PLD1	[activation]
271	GHRL	CHRM1	[activation]
272	PRKACA	ORAI1	[activation]
273	RGL1	RALA	[activation]
274	RAPGEF3	RRAS2	[activation]
275	PRKG1	VASP	[activation]
276	IRAK1	MAP3K7	[activation;inhibition]
277	RRAS	RASSF5	[activation]
278	CRTC2	AKT3	[activation]
279	TICAM2	RIPK1	[activation]
280	MLKL	PGAM5	[activation]
281	DDX58	TRAF2	[activation]
282	ACTB	STK3	[activation]
283	AKT3	BAD	[inhibition]
284	MAPK14	FOXO6	[activation]
285	TRAF6	MAP3K14	[activation]
286	RAP1A	MLLT4	[activation]
287	EGFR	CRK	[activation]
288	AKT3	NOS3	[activation]
289	APBB1IP	VASP	[activation]
290	DUSP10	MAPK1	[inhibition]
291	PRKACA	CACNA1C	[activation]
292	MAP3K8	MAP2K7	[activation]
293	CDC42	MAPK14	[activation]
294	SRC	CDC42	[activation]
295	RPS6KA4	CREB3	[activation]
296	RASA4B	RRAS2	[activation]
297	RALA	PLD1	[activation]
298	NODAL	ACVR2A	[activation]
299	GSK3B	GLI1	[inhibition]
300	RALBP1	CDC42	[activation]
301	IRS1	PIK3R5	[activation]
302	PRKCA	SPHK2	[activation]
303	NRG4	ERBB4	[activation]
304	RAPGEF3	MAPK8	[activation]

```
RRAS    PLCE1   [activation]
GNB5    RASGRF1 [activation]
CSH1    IL22RA2 [activation]
STK4    FOXO6   [activation]
NLK     LEF1    [inhibition]
EGFR    MAP2K1  [activation]
RIPK1   MAP3K7  [activation]
```

1	ABL1	WASF1	[activation]
2	ACTG1	MAP3K1	[activation]
3	ARRB1	JUN	[activation]
4	ARPC2	ARPC5	[activation]
5	LNX2	NXF1	[activation]
6	PRKCB	GSK3A	[activation]
7	CDKL3	CCDC155	[activation]
8	STMN1	TRPC5	[activation]
9	DAPK1	UNC5C	[activation]
10	TERF1	STAG1	[activation]
11	HSP90AB1	STAT2	[activation]
12	VEPH1	GSTK1	[activation]
13	CYTH1	ITGB2	[activation]
14	SNCA	GRK6	[activation]
15	IGFBP4	IGF1	[activation]
16	PTK2	ROCK1	[activation]
17	EPRS	MAP3K7	[activation]
18	CCNB1	PPP1CA	[activation;inhibition]
19	CDK11B	RANBP9	[activation]
20	CEND1	EGFR	[activation]
21	CAMK2D	CAMK2A	[activation]
22	MIF	GABARAP	[activation]
23	TRO	PRKCD	[activation]
24	LIN7C	AMOTL2	[activation]
25	QARS	GADD45A	[activation;inhibition]
26	RIPK1	PTK2	[activation]
27	TNF	BTRC	[activation]
28	SH3BP2	PLCG1	[activation]
29	ARHGEF7	PPP2R1A	[activation]
30	COPG1	ILK	[activation]
31	NPHP1	PAK2	[activation]
32	SRPK3	DHX15	[activation]
33	NRXN3	NLGN3	[activation]
34	MYB	COX7B	[activation]
35	IFT172	LHX3	[inhibition]
36	PKM	EGLN3	[activation]
37	DNAJB1	OTUD4	[inhibition]
38	NUP133	NUP153	[activation]
39	RAD21	HSP90AB1	[activation]
40	EXOC4	EGFR	[activation]
41	BUB1B	APC	[inhibition]
42	RTN4RL1	OMG	[inhibition]
43	CDK11B	HSPA8	[activation]
44	ITGA4	DARS	[activation]
45	APC	CTNNA1	[activation]
46	RAN	VRK1	[activation]
47	CHEK2	MDM4	[activation;inhibition]
48	LALBA	B4GALT1	[activation]
49	HTR1A	GPR26	[activation]
50	UNG	RPA3	[activation]
51	MNAT1	TSC1	[activation;inhibition]
52	TGFB1	APC	[inhibition]
53	MAPT	CAPN2	[activation]
54	BCL2L11	YWHAB	[activation]
55	CASP3	CTNNB1	[activation]
56	HSP90B1	TLR2	[activation]
57	CBLB	PTPN11	[inhibition]
58	PPP1CA	C1QA	[activation]
59	TPST2	CCR2	[activation]
60	GRB2	CKAP5	[activation]
61	PPP3CB	SOCS3	[inhibition]
62	LHX1	FOXA2	[activation]
63	LRRK2	PRKCZ	[activation]
64	HSPG2	BMP1	[activation]
65	DDX24	POLA2	[activation]
66	CTBP2	EHMT1	[activation]
67	ESD	TRAF6	[activation]
68	PIN1	VCAM1	[activation]
69	ERCC1	USHBP1	[activation]
70	STX11	SNAP25	[activation]
71	CUL3	CASP8	[inhibition]
72	DSP	CASP7	[activation]
73	PRMT1	NCOA1	[activation]
74	MYC	PCBP1	[activation]
75	MIF	WDYHV1	[activation]
76	LCK	PTPRF	[activation;inhibition]

77	HIST3H3	ING2	[activation]
78	ENO1	ABI2	[activation]
79	RALBP1	HSF1	[activation]
80	PFKFB2	YWHAH	[activation]
81	MFN2	BAK1	[activation]
82	SERPINE1	IGFBP5	[inhibition]
83	CRELD2	RAC1	[activation]
84	GEM	KLC3	[activation]
85	C6	GRB2	[activation]
86	GABARAP	MLH1	[activation]
87	SH2D1B	CD84	[activation]
88	TIMM50	RIPK1	[activation]
89	MMP2	ITGB1	[activation]
90	MAP1LC3C	ATG5	[activation]
91	MAP4K4	RASA1	[activation]
92	PLCG1	ARHGEF5	[activation]
93	APC	ZNF510	[inhibition]
94	PTH1R	SLC9A3R2	[activation]
95	MAPKAP1	PRR5	[activation]
96	USHBP1	STX11	[activation]
97	CTTNBP2NL	STK3	[activation]
98	PTPN6	CTNND1	[activation;inhibition]
99	PTPN11	TNFRSF1A	[activation]
100	CYP20A1	APP	[activation]
101	TBC1D3C	RAB5A	[activation]
102	KIR2DS4	HLA-C	[activation]
103	CCDC36	MRFAP1L1	[activation]
104	GADD45A	GBP2	[activation]
105	TP53BP1	TFDP1	[activation]
106	ANAPC5	FZR1	[inhibition]
107	TFG	PTPN6	[activation;inhibition]
108	NR1H3	RXRA	[inhibition]
109	TEC	LYN	[activation]
110	PARP10	CASP8	[activation]
111	PAFAH1B3	DDX24	[activation]
112	YWHAB	PUM1	[activation]
113	EPOR	PTPN11	[activation;inhibition]
114	PRR14L	PPP2CA	[inhibition]
115	YWHAQ	TSC1	[inhibition]
116	ERAS	RAF1	[activation]
117	PRNP	CSNK2B	[activation]
118	RAN	SSRP1	[activation]
119	ICT1	MTIF2	[activation]
120	ABT1	PRNP	[activation]
121	STK24	CASP3	[activation]
122	RIT2	MLLT4	[activation]
123	RPA1	CCNB1	[activation]
124	RALA	ARF6	[activation]
125	SMAD2	PIK3CA	[activation]
126	SMARCA2	H2AFX	[activation]
127	SH3GL1	LRRK2	[activation]
128	DDX24	UBE2I	[activation]
129	GAB2	ETV6	[activation]
130	VCAM1	ELANE	[activation]
131	ATG5	SNCA	[activation]
132	JUP	FHL2	[activation]
133	SRI	PSEN2	[activation]
134	MSTO1	NR1H3	[activation]
135	CRK	NTRK1	[activation]
136	TP53	LRRK2	[activation]
137	MAPK1	DCC	[activation]
138	CDK3	CDK5	[activation]
139	TP53	TBC1D4	[activation]
140	JUP	INSIG2	[activation]
141	LRRK2	MTHFD2	[activation]
142	GDNF	RET	[activation;inhibition]
143	MAP3K2	MAP3K5	[activation]
144	ERBB2	EZR	[activation]
145	GNB5	MCM2	[activation]
146	FLT1	KIAA1524	[activation]
147	KRT19	GRB2	[activation]
148	UFM1	RPS6KA6	[activation]
149	PAXIP1	WDHD1	[activation]
150	EIF3F	TRAF6	[activation]
151	NOSTRIN	NOS3	[activation]
152	LZTS2	GRB2	[activation]

ANAPC10	PPP2R1A	[activation]
PRKY	HSP90AB1	[activation]
GAB2	NCK1	[activation]
HSPD1	CASP9	[activation]
KAT2A	IRF2	[activation]
TRAF2	SPHK1	[activation]
RIBC2	USHBP1	[activation]
GAB2	YWHAG	[activation]
HSPA8	JAK2	[inhibition]
NFKB1	ERCC6	[activation]
CPNE2	CDKN1A	[activation;inhibition]
RHOC	ARHGAP1	[activation]
EGFR	BECN1	[activation]
RPS6	ESR1	[activation]
PARP11	RNF114	[activation]
PPP1R12A	YWHAZ	[activation]
TSC1	FTH1	[inhibition]
CCNB1	BRCA1	[activation]
ZNF24	DNER	[activation]
STAT1	UBE2I	[activation]
FYN	FLOT2	[activation]
PAK1	SRSF5	[activation]
TBK1	EGFR	[activation]
CR1	CR2	[activation]
VCAM1	BUB3	[activation]
CAMK2B	FAM171A2	[activation]
SRC	IL6R	[activation]
AURKB	PPFIA1	[activation]
TSC2	FBXL6	[inhibition]
H2AFX	BIRC5	[activation]
PDZK1	SLC9A3	[activation]
APP	TBCD	[inhibition]
FAF1	VAPB	[activation]
EHMT2	ANKRD28	[activation]
RELA	PPARGC1A	[activation]
YY1	SMAD4	[activation]
CNKSR1	ZDHHC17	[activation]
KCNB1	KCNG2	[activation]
TOLLIP	DAB1	[activation]
SLC30A2	TNFRSF12A	[activation]
PRKAA2	USHBP1	[activation]
HIST1H2BM	ITGA4	[activation]
ID4	PLCG1	[activation]
FAM83D	GADD45A	[activation;inhibition]
CCL2	ORC4	[activation]
TP53BP1	HIST1H3A	[activation]
CAD	MAP3K3	[activation;inhibition]
MMP9	PLG	[activation;inhibition]
GHRL	MLNR	[activation]
PRKAA1	VPS37B	[inhibition]
IGHA1	SKIV2L2	[activation]
RIMS1	CAMK2A	[activation]
ATM	TERF1	[activation]
CDC20	CDKN1A	[activation;inhibition]
CAD	GRB2	[activation;inhibition]
APOE	LRP8	[activation]
MAVS	TRAF3	[activation]
PLEC	VCAM1	[activation]
SIRT1	HNF4A	[activation]
CRK	PCDHA12	[activation]
TGM2	PAK1	[activation]
TET2	SMARCA4	[activation]
AURKA	MDM2	[activation]
BCL2L2	BAK1	[activation;inhibition]
ATF3	SUV39H1	[activation]
PRKACA	RXFP1	[activation]
SOCS1	PDCD1	[inhibition]
PAXIP1	KDM6A	[activation]
RGS7	GNAQ	[activation]
THEM6	ESR1	[activation]
RARA	KAT2B	[activation]
CYFIP1	LRRK2	[activation]
WSB2	NUDC	[activation]
MTOR	NBN	[activation]
SRGAP2	RAC1	[activation]
RMND5A	MAEA	[activation]

229	KIR2DL5A	PTPN6	[activation;inhibition]
230	EGFR	ICAM1	[activation]
231	SOX9	CREB1	[activation]
232	U2AF2	FTSJ1	[activation]
233	SRPK3	NOP2	[activation]
234	GABARAP	YWHAZ	[activation]
235	MAST3	EIF2S2	[activation]
236	NUP214	RANBP2	[activation]
237	CDK11B	TH	[activation]
238	TMEM173	TRAF3	[activation]
239	TFRC	FBXO6	[activation]
240	BMP3	WFIKKN2	[activation]
241	RAD21	EIF4G1	[activation]
242	DPP4	CXCL10	[activation]
243	SLC9A3R1	YES1	[activation]
244	CCR1	STAT3	[activation]
245	PIK3R1	TGDS	[activation]
246	ARHGAP9	MAPK1	[activation]
247	OPRD1	GNAI1	[activation]
248	RAC1	RASGRF1	[activation]
249	APP	TEK	[activation]
250	CDC37	MOS	[inhibition]
251	ZNF142	MAPK14	[activation]
252	NRP1	FGF2	[activation]
253	SMURF1	FBXL15	[inhibition]
254	CD3D	PDIA2	[activation]
255	EIF2B3	FBXO6	[activation]
256	TP53	SRPK1	[activation]
257	RAC1	WASF2	[activation]
258	QRICH2	MAGEB2	[activation]
259	PDGFRL	FBP2	[activation;inhibition]
260	ICT1	ALDH1L2	[activation]
261	APH1A	GLP1R	[activation]
262	ILK	PARVG	[activation]
263	SLC25A6	TRIP6	[activation]
264	ANXA4	SMPD1	[activation]
265	MEPCE	ACTG1	[activation]
266	NUP153	AIM2	[activation]
267	MAVS	FADD	[activation]
268	SNTA1	ADRA2A	[activation]
269	EGFR	EXOC6B	[activation]
270	ITGAD	ICAM3	[activation]
271	CDC7	CDK2	[activation]
272	RAP1B	MAP3K14	[activation]
273	IRF3	RBL2	[inhibition]
274	SOCS1	TCEB2	[inhibition]
275	ERBB3	SH2B3	[activation]
276	TNFRSF10D	CYCS	[activation]
277	LIMS1	SMURF1	[inhibition]
278	TRAF6	RRAGC	[activation]
279	PLEKHJ1	TGFBR1	[activation]
280	LCP2	PTPN6	[activation;inhibition]
281	EIF4G2	PTBP3	[activation]
282	MYC	EXOC4	[activation]
283	SERPINB13	PLG	[inhibition]
284	IGSF21	PAEP	[activation]
285	AURKB	HIST1H3A	[activation]
286	SMAD3	PITX2	[activation]
287	TBK1	PROS1	[inhibition]
288	SRPK1	PRM1	[activation]
289	NXF1	CCDC25	[activation]
290	RAB3GAP2	RAB3GAP1	[activation;inhibition]
291	ABL1	BCR	[activation]
292	JAK3	PRMT5	[activation]
293	BMPR1B	BMPR1A	[activation;inhibition]
294	CCDC87	ESR2	[activation]
295	CUL1	RICTOR	[activation]
296	CSNK1A1L	ARRB2	[inhibition]
297	SPECC1	NXF1	[activation]
298	VRK3	RAN	[activation]
299	CLIP1	NR3C1	[activation]
300	YWHAZ	RASAL3	[activation]
301	NXF1	DPY19L4	[activation]
302	CRY1	PER1	[inhibition]
303	ACVR1B	SMURF1	[inhibition]
304	CD40	OGT	[activation]

305	HDAC2	BUB1B	[inhibition]
306	PPM1D	H2AFX	[activation]
307	IL7R	YWHAE	[activation]
308	FAM117B	MYC	[activation]
309	RAF1	CALM1	[activation]
310	CASP8	CDH13	[activation]
311	RASA1	PTPRJ	[activation]
312	GLI3	SMAD1	[activation]
313	TNFRSF1A	GRB2	[activation]
314	NTRK3	IRAK3	[inhibition]
315	DDX54	SRPK3	[activation]
316	HOXD12	MEIS1	[activation]
317	KIF3A	GRB2	[activation]
318	MET	ITK	[activation]
319	SGK1	CBR3	[activation]
320	RPTOR	IKBKB	[activation]
321	MLH1	IGKC	[activation]
322	NFKB1	FBXW11	[inhibition]
323	PTPRJ	FLT3	[activation]
324	TRAF1	RIPK1	[activation]
325	INADL	CNKSR2	[activation]
326	ANAPC11	CDC23	[activation]
327	HSP90AB1	STK32B	[activation]
328	TRAF6	BECN1	[activation]
329	ESR2	SUPT6H	[activation]
330	RICTOR	YWHAZ	[activation]
331	CCNI	AKT1	[activation]
332	CTLA4	STAT5B	[activation]
333	TGFB1I1	SMAD4	[activation]
334	EFNA2	ZHX1	[activation]
335	TLN1	OBSL1	[activation]
336	SIRT1	MAPT	[activation]
337	FSCN1	FTSJ1	[activation]
338	PGRMC1	ATG5	[activation]
339	PTPN1	CRK	[activation]
340	TP53	RCN2	[activation]
341	TMSB10	FOS	[activation]
342	APP	CTF1	[activation]
343	RBL2	GSTM3	[activation;inhibition]
344	YWHAB	EIF4E2	[activation]
345	VASN	TGFB2	[activation]
346	RFC2	PITX2	[activation]
347	RXRA	RAC3	[inhibition]
348	FAS	RIF1	[inhibition]
349	PLSCR1	EP300	[activation]
350	CMKLR1	TPST2	[activation]
351	MDM2	ATF4	[activation]
352	LAMTOR5	SMAD4	[activation]
353	PAK4	ARHGEF2	[activation]
354	IKBKB	CDC37	[activation]
355	ABL2	TPD52L1	[activation]
356	SCIN	GRB2	[activation]
357	PAG1	ABL1	[activation]
358	IGSF1	ACVR2B	[activation]
359	AMH	EGFR	[activation]
360	SRPK2	BRD3	[activation]
361	TLR2	RAC1	[activation]
362	TTK	APP	[activation]
363	HSPB2	TRAF2	[activation]
364	SRC	NR1H2	[activation]
365	GAB2	BCR	[activation]
366	KIDINS220	NGFR	[activation]
367	MCM7	ARHGEF6	[activation]
368	ACTR3	ARPC1B	[activation]
369	GABRG2	PRKCA	[activation]
370	CSNK2B	PLXNA3	[activation]
371	GADD45A	IGHM	[activation]
372	CFL1	HSPH1	[activation]
373	RELA	TP53BP1	[activation]
374	JDP2	EP300	[activation]
375	ZAP70	PTPN3	[activation]
376	POLD2	CCHCR1	[activation]
377	APP	MAPK11	[activation]
378	HDAC11	HHIP	[inhibition]
379	RYR1	CAMK2G	[activation]
380	TAF1B	APP	[activation]

381	FSBP	CDC23	[activation]
382	GH1	ERLEC1	[inhibition]
383	LRRFIP2	GRB2	[activation]
384	FASTKD5	MLH1	[activation]
385	ZBTB48	ILK	[activation]
386	GNAI3	GPSM3	[activation;inhibition]
387	TIAL1	RPA1	[activation]
388	GEM	PNMA1	[activation]
389	PLCL2	DSCAML1	[activation]
390	TNFSF11	LMO4	[activation]
391	NFKBIA	BARD1	[activation]
392	FAM9B	SNAP29	[activation]
393	CDC5L	CALM1	[activation]
394	HSPB1	F13A1	[activation]
395	TRAF6	RPL38	[activation]
396	KLHL21	AURKB	[activation]
397	MARS	MDM2	[activation]
398	HSPD1	CDH1	[activation]
399	RPS19	FGF2	[activation]
400	HSP90AB1	KLHL6	[activation]
401	GRN	FAM131C	[activation]
402	MAST3	EIF2A	[activation]
403	ASS1	ELN	[activation]
404	TAGLN2	H2AFX	[activation]
405	IRS2	PTPN6	[activation;inhibition]
406	CRK	OGN	[activation]
407	LATS1	OFD1	[inhibition]
408	DVL2	PRKCA	[activation]
409	RAD51B	HELQ	[activation]
410	ACTR1A	SMURF1	[inhibition]
411	CCNA2	UBTF	[activation]
412	SKAP1	SRC	[activation]
413	PTK6	AR	[activation]
414	PGR	RELA	[activation]
415	RNF2	CASP3	[activation]
416	ITGB1	FHL2	[activation]
417	RAB5C	RAB5A	[activation]
418	APP	NGFR	[activation]
419	BTRC	RAPGEF2	[activation]
420	RANBP2	LGR4	[activation]
421	TNFSF13	SDC2	[activation]
422	ATG5	CDKN2A	[activation]
423	RAB8B	GRB2	[activation]
424	RAP2B	RALA	[activation]
425	LRRK2	HIST2H2AA3	[activation]
426	HGF	F11	[activation]
427	RNF2	ACACA	[activation;inhibition]
428	GP6	TGFB1I1	[activation]
429	DNM2	CDC5L	[activation]
430	SMARCA2	CDX2	[activation]
431	CNTNAP1	NFASC	[activation]
432	ATM	HDAC6	[activation]
433	SUMO4	NR3C1	[activation]
434	CDK13	CDK4	[activation]
435	COBL	RPA2	[activation]
436	H2AFX	ANXA5	[activation]
437	DDX58	UBE2D1	[inhibition]
438	SPP1	HMGN1	[activation]
439	EGFR	EPHB4	[activation]
440	ESR2	PROSER2	[activation]
441	CDK7	SRPK2	[activation]
442	LRRK2	LRP6	[activation]
443	MME	PIK3R1	[activation]
444	PRLR	VAV1	[inhibition]
445	NUP62	C1orf216	[activation]
446	EGFR	KPNB1	[activation]
447	DVL3	C8orf33	[activation]
448	SRGAP2	SRGAP2C	[activation]
449	C2orf44	ANXA1	[activation]
450	EIF3E	SMURF1	[inhibition]
451	PPP3R2	AURKA	[activation]
452	GADD45A	GNB2	[activation]
453	LTBP4	CACNA1A	[inhibition]
454	FZD1	UBC	[activation]
455	TRPV1	TRPV2	[activation]
456	RAB5B	EEA1	[activation]

457	RBL1	BRF1	[inhibition]
458	GRB2	REPS2	[activation]
459	EPHA2	HSP90AB1	[activation]
460	SMAD3	BPTF	[activation]
461	HTR2A	DLG4	[activation]
462	VCAN	XCL1	[activation]
463	NFKB1	TBK1	[activation]
464	DDX5	DAPK1	[activation]
465	CDK11B	YWHAZ	[activation]
466	AR	SHC3	[activation]
467	CDK1	ABL1	[activation]
468	PLK1	CENPQ	[activation]
469	SIRT1	CDK1	[activation]
470	ARRB1	MYO1C	[activation]
471	TERT	CTNNB1	[activation]
472	INSIG2	PSMD13	[activation]
473	PBX1	NR3C1	[activation]
474	ATG5	CCDC12	[activation]
475	PRKCA	VCL	[activation]
476	GNA11	ELAVL1	[activation]
477	TNS3	KIT	[activation]
478	MECOM	FOSL2	[activation]
479	CHEK2	XRCC1	[activation]
480	FCRL4	TFF1	[activation]
481	CTBP1	EHMT2	[activation]
482	ICT1	MTG1	[activation]
483	RPA2	KCNAB2	[activation]
484	GRB2	CORO1B	[activation]
485	FGFR1	IL17RD	[activation]
486	NKAPL	LYN	[activation]
487	GABARAPL2	ACTB	[activation]
488	DNAJB1	BAG2	[inhibition]
489	ANXA5	ASB9	[activation]
490	IL6ST	IL31RA	[activation]
491	SPOP	DUSP7	[inhibition]
492	PIAS1	ESR1	[activation]
493	PTPN1	NTRK2	[activation]
494	DEGS1	EGFR	[activation]
495	SUMO3	HIF1A	[inhibition]
496	FYN	SUV39H2	[activation]
497	UHRF1BP1L	YWHAB	[activation]
498	BRCA2	STAT5A	[activation]
499	RAB5A	GDI2	[activation]
500	HSPBP1	HSPA8	[inhibition]
501	MEPCE	BARD1	[activation]
502	SYK	CTTN	[activation]
503	AXIN2	SMAD1	[inhibition]
504	AR	NRIP1	[activation]
505	PTPN6	PAG1	[activation;inhibition]
506	HSPA5	BCAR1	[activation]
507	CALCA	RAMP2	[activation]
508	ILK	AUP1	[activation]
509	GAPDH	ICAM1	[activation]
510	PXN	GIT2	[activation]
511	TNR	MYC	[inhibition]
512	IRS2	YWHAG	[activation]
513	STAT6	NCOA3	[activation]
514	CTNNA1	CTNNA3	[activation]
515	DNAJA1	CUL1	[inhibition]
516	LYN	CDK4	[activation]
517	EDA	FURIN	[activation]
518	TICAM2	TLR3	[activation]
519	KIAA1377	LPL	[activation]
520	FAS	UBE2I	[activation]
521	BCAM	LAMA5	[activation;inhibition]
522	EGFR	ACTA1	[activation]
523	IGHA1	GDF10	[activation]
524	KIT	BTK	[activation]
525	GRB2	NKD2	[activation]
526	AURKA	LYPD3	[activation]
527	STK11	SPDEF	[activation]
528	CRKL	FYB	[activation]
529	HIST2H3A	VCAM1	[activation]
530	GRK6	FSHR	[activation]
531	WAS	DNM2	[activation]
532	SH3KBP1	PSRC1	[activation]

533	TULP3	SIRT1	[activation]
534	TNFRSF14	DHX9	[activation]
535	KDELR2	PARP2	[activation]
536	CDC5L	TRAK1	[activation]
537	SHC3	GAB1	[activation]
538	DKC1	NXF1	[activation]
539	ARSA	BMPR2	[activation]
540	ARPC5	EGFR	[activation]
541	RPA1	ANXA7	[activation]
542	FANCD2	FSCN1	[activation]
543	NXF1	POLE2	[activation]
544	CASP3	THAP11	[activation]
545	RELA	C1QB	[activation]
546	SP1	HIF1A	[activation]
547	NUDCD3	PAFAH1B1	[activation]
548	ACTN1	HSPB1	[activation]
549	PRKCI	CRX	[activation]
550	MOS	FKBPL	[activation]
551	MAP2K1	NFE2L2	[activation]
552	RAB5C	CD247	[activation]
553	ANG	TDGF1	[activation]
554	HDAC1	SYK	[activation]
555	DOCK1	NCK2	[activation]
556	YWHAQ	CDK11B	[activation]
557	FGFR2	S100A14	[activation]
558	PRKACA	GP1BB	[activation]
559	PLAU	SERPINA5	[inhibition]
560	HDAC1	STAT3	[activation]
561	JMJD6	HMGXB4	[activation]
562	ESR1	U2AF2	[activation]
563	ERBB2	BLK	[activation]
564	RAC1	PAK6	[activation]
565	NCK2	CBL	[activation]
566	BCAR1	MMP14	[activation]
567	TIMP1	FBXO6	[activation]
568	GSK3B	ACSBG1	[activation]
569	SMCHD1	ITGA4	[activation]
570	ILK	ARRB1	[activation]
571	VDR	PRCP	[activation]
572	GAB1	NCK1	[activation]
573	MAPRE1	VCAM1	[activation]
574	LRCH1	DOCK8	[activation]
575	YWHAE	MARK3	[activation]
576	CHD7	BRD7	[activation]
577	TFDP2	E2F2	[inhibition]
578	EGFR	RCN2	[activation]
579	TNFRSF14	HPRT1	[activation]
580	CAV1	IGHA1	[activation]
581	RHOA	FADD	[activation]
582	AURKB	SUMO1	[activation]
583	FGFR2	RHOBTB2	[activation]
584	DVL3	CTNNB1	[activation]
585	GRB2	THEMIS	[activation]
586	PRKDC	APLF	[activation]
587	CDKN1A	TNIP2	[activation]
588	FAAP24	STRA13	[activation]
589	SUMO3	NFE2L2	[activation]
590	TESK1	YWHAB	[activation]
591	PIK3R1	CTLA4	[activation]
592	FASN	PTPN11	[activation]
593	TGM3	GABARAPL2	[activation]
594	ATAD3B	ICT1	[activation]
595	TGFBR2	PAK1	[activation]
596	SUV420H1	YWHAQ	[activation]
597	RAD21	WFDC5	[inhibition]
598	MARK3	PNPLA2	[activation]
599	TGFBR1	FANCL	[activation]
600	METTL17	SPERT	[activation]
601	GRB2	RPL18A	[activation]
602	IKBKB	TNF	[activation]
603	PPP2CA	ESPL1	[inhibition]
604	MCM3	PPP2R1A	[activation]
605	DOK5	NTRK2	[activation]
606	IKBKB	MAP3K13	[activation]
607	ITCH	GSN	[activation]
608	TGFB1	PSMC3IP	[activation]

609	NCOA2	PGR	[activation]
610	ROCK1	ARRB1	[activation]
611	PACSIN1	WASF2	[activation]
612	NFE2	KAT2B	[activation]
613	RICTOR	SFN	[activation]
614	TRAPPC8	TRAPPC6B	[activation]
615	HSPA8	NLRP12	[inhibition]
616	TRAP1	CDK5	[activation]
617	ANGPTL3	ITGB3	[activation]
618	LAT	ZAP70	[activation]
619	TMC4	FLT1	[activation]
620	SMARCC1	GATA1	[activation]
621	EPSTI1	AKT1	[activation]
622	GRB10	SRC	[activation]
623	YWHAE	PRPSAP2	[inhibition]
624	RNF41	APP	[activation]
625	DOCK8	DOCK6	[activation]
626	STAT1	PRKCE	[activation]
627	CCNE1	PRC1	[activation]
628	ABI2	RAC1	[activation]
629	HLA-B	AKR7A2	[activation]
630	MAP1LC3B	DSG1	[activation]
631	PIK3CB	YWHAQ	[activation]
632	UBE2D1	HIF1A	[inhibition]
633	WIPI1	PPA1	[activation]
634	BRCA1	CCND1	[activation]
635	MAPK6	MTG2	[activation;inhibition]
636	GRM2	RGS12	[activation;inhibition]
637	PTPRJ	PXN	[activation]
638	SHC1	SH2B2	[activation]
639	MACF1	SKIL	[activation]
640	RASSF1	CNKSR1	[inhibition]
641	PKN1	CASP3	[activation]
642	ADRB2	WNK1	[activation]
643	STK3	MAP1S	[activation]
644	PIK3R3	FGB	[activation]
645	NUPR1	EP300	[activation]
646	ARPC1B	ARPC5	[activation]
647	RCN2	TRAF6	[activation]
648	CD8B	CD8A	[activation]
649	NXF1	TMEM222	[activation]
650	PIAS1	PIAS2	[inhibition]
651	PPP3CA	RCAN1	[activation]
652	RAD21	KPNB1	[activation]
653	NRAS	SRI	[activation]
654	PPP1CA	RB1	[activation;inhibition]
655	METTL25	APP	[activation]
656	CASP3	PTMA	[activation]
657	MAPK12	DLG3	[activation]
658	MIB1	JAG1	[activation]
659	ZSCAN26	LRRK2	[activation]
660	GRB2	ARHGEF11	[activation]
661	PIK3R2	DYDC1	[activation]
662	MEF2A	TBP	[activation]
663	CHUK	HSP90AA1	[activation]
664	EGFR	HOXC10	[activation]
665	CASP3	BLM	[activation]
666	RADIL	SMYD2	[activation]
667	ST14	PLAU	[activation]
668	YES1	YAP1	[activation]
669	RBM41	RAC3	[activation]
670	DEK	KAT2B	[activation]
671	CCSER2	CRADD	[activation]
672	MS4A1	IGHD	[activation]
673	SMAD3	FOXO3	[inhibition]
674	RB1	CCNB1	[activation;inhibition]
675	RPA1	CAPRIN1	[activation]
676	EGFR	RPA3	[activation]
677	FYN	CD44	[activation]
678	SRPK2	CLK1	[activation]
679	LAMTOR5	EIF4B	[activation]
680	IKBKG	EGLN3	[activation]
681	CAMKK1	YWHAH	[activation]
682	ANKRD28	NEK4	[activation]
683	ABL2	BCR	[activation]
684	CKAP4	EGFR	[activation]

685	SMAD2	PSG9	[activation]
686	BCL7C	BRD7	[activation]
687	CYCS	FDX1	[activation]
688	TRAF2	PGBD1	[activation]
689	NCAPG	EGFR	[activation]
690	CCDC33	TGFB1	[activation]
691	FAN1	YWHAE	[activation]
692	SMURF1	E2F7	[inhibition]
693	LAX1	GRB2	[activation]
694	MAP2K3	ELK1	[activation]
695	EPHB2	KSR1	[activation]
696	IL2RG	ICAM1	[activation]
697	GRIP1	CREBBP	[activation]
698	HSPA5	IKBKB	[activation]
699	TRAF5	MAVS	[activation]
700	PTPRE	GRB2	[activation;inhibition]
701	TBC1D22B	CCDC67	[activation;inhibition]
702	HNRNPA1	MLLT4	[activation]
703	MDM2	DDX3Y	[activation]
704	GTLF3B	TRAF2	[activation]
705	STAC	LRRK2	[activation]
706	RRAS2	RASIP1	[activation]
707	RIPK2	HSPA1L	[activation]
708	YWHAG	ARRB2	[activation]
709	CXCR4	PTPRC	[activation]
710	UBE2D3	NFKBIA	[activation]
711	TRAF3	TNFRSF4	[activation]
712	CACNA1A	KALRN	[activation]
713	DNM1L	SH3GL1	[activation]
714	IFRD1	CTNNB1	[activation]
715	BCL2L11	PRKACA	[activation]
716	RPA3	TIPIN	[activation]
717	EIF1AX	EIF3C	[activation]
718	DMTF1	CCND1	[inhibition]
719	SNX33	FASLG	[inhibition]
720	ARHGAP21	RHOA	[activation]
721	LRRK2	MCM3	[activation]
722	ITIH5	CHEK2	[activation]
723	LYN	CSF2RB	[activation]
724	NUP62	ABI2	[activation]
725	ARHGDIA	UFM1	[activation]
726	PRKDC	USF1	[activation]
727	CDK5	MBP	[activation]
728	MAP9	EP300	[activation]
729	RASA1	PLCG1	[activation]
730	PKIG	RAB25	[inhibition]
731	HELLS	MYC	[activation]
732	CLN6	ILK	[activation]
733	DVL1	DYNLT1	[inhibition]
734	HSPA7	CFTR	[activation]
735	STK4	ARHGAP18	[activation]
736	MAP3K2	NFKBIA	[activation]
737	HSF1	UBE2I	[activation]
738	RPA3	IQGAP1	[activation]
739	CDK1	RPA2	[activation]
740	WDR26	BARD1	[activation]
741	PPARG	ZYX	[activation]
742	PIK3R1	WASF3	[activation]
743	NXF1	CDC23	[activation]
744	A2M	IL10	[activation]
745	CD7	PI4K2A	[activation]
746	MAP9	TP53	[activation]
747	DNM2	GDF9	[activation]
748	TLR2	ANP32B	[activation]
749	VCAM1	SRSF1	[activation]
750	ACTA2	EP300	[activation]
751	EP300	CCNT1	[activation]
752	NCOA1	STAT3	[activation]
753	SMC4	CASP4	[activation]
754	INSIG2	HSPA6	[activation]
755	THBS1	CFH	[activation;inhibition]
756	MAPRE1	PIK3R4	[activation]
757	GSK3B	IQCG	[activation;inhibition]
758	HNRNPC	TRAF6	[activation]
759	TAF1C	TAF1B	[activation]
760	SRPK2	LUC7L	[activation]

761	NCOA3	FOS	[activation]
762	GRK6	ITGA5	[activation]
763	TH	PRKACA	[activation]
764	BCL2L1	BLK	[activation]
765	ACTR1A	HLA-B	[activation]
766	CDKN2C	MAPK10	[activation]
767	ENO2	SNUPN	[activation]
768	SMAD3	IRF7	[activation]
769	MAP2K1	CTNNA1	[activation]
770	CFLAR	FKBPL	[inhibition]
771	LNX2	IGSF5	[activation]
772	CBL	EIF5B	[activation]
773	MET	BCAR3	[activation]
774	NUP88	NUP214	[activation]
775	PNPLA2	CYTH2	[activation]
776	TERF1	UTP14A	[activation]
777	SH3RF1	SEMA6A	[activation]
778	ARFIP2	ARF1	[activation]
779	FOXO3	ERBB2IP	[inhibition]
780	GEM	KCTD9	[activation]
781	LAMA5	CRKL	[activation]
782	SRPK1	CIAO1	[activation]
783	PTPRC	PDGFRB	[activation]
784	CDKN1A	CDK14	[activation;inhibition]
785	PRKAA1	SSX2IP	[inhibition]
786	LRRK2	ADCK3	[activation]
787	GSG1	TRAF2	[activation]
788	KCNA5	SNTA1	[activation]
789	ITPR2	BANK1	[inhibition]
790	PCBP1	TP53	[activation]
791	GPSM2	NUMA1	[inhibition]
792	MAD2L1	KLHL13	[inhibition]
793	STAB2	MAPK14	[activation]
794	CACNB1	APP	[activation]
795	RCAN1	STAT2	[activation]
796	APP	KCNAB3	[activation]
797	CASC3	AKT1	[activation]
798	TTC3	AKT3	[activation]
799	NOTCH1	PDS5A	[activation]
800	PXN	EGFR	[activation]
801	ELSPBP1	MYF5	[activation]
802	SMAD4	CCDC180	[activation]
803	RASSF10	CCDC85C	[inhibition]
804	CDH1	ACTG1	[activation]
805	CTNNB1	UBE2D1	[inhibition]
806	FAM129A	AKT1	[activation]
807	STAT1	HDAC3	[activation]
808	RAC1	CHN1	[activation]
809	GPRIN2	SFN	[activation]
810	TUB	GRB2	[activation]
811	FHOD1	GRB2	[activation]
812	GNAS	ADCY5	[activation]
813	EIF2B1	MRPL4	[activation]
814	HLA-C	NUDC	[activation]
815	SMPD1	CASP8	[activation]
816	GSKIP	GSK3B	[inhibition]
817	F2RL2	CDH5	[activation]
818	GOLGA2	BARD1	[activation]
819	ERCC3	SRPK2	[activation]
820	IGHA1	TBC1D19	[activation;inhibition]
821	PRKACB	HSP90AB1	[activation]
822	KLRK1	MICA	[activation]
823	RHOA	TRPC1	[activation]
824	MAPK8	YWHAZ	[activation]
825	MAP3K12	TGM2	[activation]
826	MTNR1A	RAB10	[activation]
827	S1PR3	GNAI1	[activation]
828	BCL2	PKMYT1	[inhibition]
829	COPS4	IKBKB	[activation]
830	CDKN1A	SMAD4	[activation;inhibition]
831	IRF3	IKBKE	[inhibition]
832	HSP90AA1	STK36	[activation]
833	GRB2	CELF2	[activation]
834	KSR1	NME1	[activation]
835	ACTN1	CACNA1A	[activation]
836	YWHAZ	IRS2	[activation]

837	SST	SSTR5	[activation]
838	CCND1	FBXW8	[inhibition]
839	BHLHE41	SIRT1	[activation]
840	PAK1	PAK2	[activation]
841	ILK	FBL	[activation]
842	ESR1	RARA	[activation]
843	AR	ADAM10	[activation]
844	MCM7	HIPK4	[activation]
845	SOS2	ESR1	[activation]
846	APP	ELK1	[activation]
847	GAB1	FRS2	[activation]
848	GABARAPL2	VDR	[activation]
849	SMAD1	ERBB2	[activation;inhibition]
850	DVL1	CTNNB1	[activation]
851	CLU	PRNP	[activation]
852	PRMT5	YWHAB	[activation]
853	ODF2L	NXF1	[activation]
854	KCTD3	HSPB1	[activation]
855	OBSCN	DNAJB5	[inhibition]
856	SORL1	FURIN	[activation]
857	STAT1	MCM5	[activation]
858	HOXB1	PAX6	[activation]
859	TP53	GTF2H1	[activation]
860	GABARAP	HNRNPA1	[activation]
861	SMG9	TRAF2	[activation]
862	CXCR4	HSPA8	[activation]
863	SUMO2	RAN	[activation]
864	CD247	NCR1	[activation]
865	FLNA	ICAM1	[activation]
866	EGLN1	MAGEA11	[activation]
867	F2RL2	FYN	[activation]
868	STX5	STX4	[activation]
869	ZSWIM8	AURKA	[activation]
870	CD44	MMP9	[activation]
871	ABL1	MAP4K5	[activation]
872	CASP4	TRAF6	[activation]
873	BUB1	PPP3R2	[activation]
874	RHOA	GNB2L1	[activation]
875	MAP1LC3A	EPHA7	[activation]
876	KIAA0232	YWHAZ	[activation]
877	F11R	MLLT4	[activation]
878	DHPS	MAPK3	[activation]
879	EFHC2	NCK2	[activation]
880	MAVS	PCBP2	[activation]
881	CASP8	PSMC3IP	[activation]
882	MAPK3	PTPRE	[activation;inhibition]
883	IGF2	IGFBP2	[activation]
884	SEMA4C	NOTCH2NL	[activation]
885	ILK	CAV1	[activation]
886	OSMR	JAK2	[activation]
887	MYC	FANCD2	[activation]
888	SLC9A3R1	CAPN6	[activation]
889	CCL19	CCL22	[activation]
890	HSPA5	SREBF2	[activation]
891	PLG	THBS1	[activation]
892	SMARCA4	ACTB	[activation]
893	KMT2A	PPP1R15A	[activation]
894	NBN	HIST1H3A	[activation]
895	CBLB	TGFB1I1	[inhibition]
896	ATG7	APP	[activation]
897	ALK	SHC1	[activation]
898	SNCA	WDFY3	[activation]
899	MDM2	ACACA	[activation;inhibition]
900	EXT1	CNTF	[activation]
901	NUMB	PRKCE	[activation]
902	CPSF3	CDC5L	[activation]
903	TULP3	WNK1	[activation]
904	CCNL1	CDK11A	[activation]
905	SHC1	CRKL	[activation]
906	TLR2	TICAM1	[activation]
907	KCNB2	KCNG4	[activation]
908	MIF	VHL	[activation]
909	GRB2	ARPC3	[activation]
910	DDX24	FADD	[activation]
911	KPNB1	NFYA	[activation]
912	BARD1	TP53	[activation]

913 LRP1 ANAPC10 [activation]
914 GNB2L1 IGF1R [activation]
915 HSP90AA1 NPAS2 [activation]
916 NMI STAT5B [activation]
917 HSP90AA1 MET [activation]
918 ESR2 SP1 [activation]
919 CDC6 CCNE1 [activation]
920 CCR1 CREB3 [activation]
921 FANCE FANCC [activation]
922 ZBED5 SMAD4 [activation]
923 LYN FCGR2B [activation]
924 SMAD7 HDAC1 [activation]
925 TNKS1BP1 CDC5L [activation]
926 NCOA4 RXRA [inhibition]
927 SRSF1 CDK6 [activation]
928 SGK1 PA2G4 [activation]
929 ABI3 MRFAP1L1 [activation]
930 MYD88 SPOP [activation]
931 PIK3R1 SOS2 [activation]
932 PPP2R5A PPP2R1A [activation]
933 NXF1 MRPL24 [activation]
934 SPI1 CEBPB [activation]
935 GADD45G ESR1 [activation]
936 EIF3D YWHAZ [activation]
937 DYRK1A SRSF5 [activation]
938 CD81 GAPDH [activation]
939 SAV1 LATS1 [inhibition]
940 FHL2 E2F4 [inhibition]
941 SERPINA1 ELANE [inhibition]
942 MAP1LC3A ENO2 [activation]
943 SPEN DLX5 [activation]
944 AIRE PIK3R1 [activation]
945 TGM1 GABARAPL1 [activation]
946 ADRBK1 EGF [activation]
947 SH2D1A AR [activation]
948 RARA HNRNPL [activation]
949 SARM1 IRAK1 [activation;inhibition]
950 TSSC4 TAB1 [inhibition]
951 HIST1H3A BRD7 [activation]
952 FLT1 SOGA1 [activation]
953 IGSF21 CRCT1 [activation]
954 NXF1 ABCF2 [activation]
955 PALB2 BRCA2 [activation]
956 YTHDC2 TP53 [activation]
957 IQGAP1 PTK2 [activation]
958 RELA PIN1 [activation]
959 SRC TRPV4 [activation]
960 ATF2 TMED10 [activation]
961 MITF UBE2I [activation]
962 ENO1 TCAP [inhibition]
963 GRB2 MYO18A [activation]
964 CEACAM6 CEACAM5 [activation]
965 STMN1 SESTD1 [activation]
966 CDK8 CEBPB [activation]
967 ATM VPRBP [activation]
968 IDE INS [activation]
969 GSN FBXO25 [activation]
970 GRB7 PTK2 [activation]
971 SOS1 EPS8 [activation]
972 ACTB CSNK2B [activation]
973 APPL1 GPC3 [activation]
974 F7 SLX4 [activation]
975 TRAK1 SKIL [activation]
976 MYO18B ACTB [activation]
977 STK4 SNX5 [activation]
978 HSP90AB1 NEK11 [activation]
979 PSIP1 OBSL1 [activation]
980 HSP90B1 ATF2 [activation]
981 ERLIN1 VCAM1 [activation]
982 CXCR4 ELANE [activation]
983 BIRC6 VCL [activation]
984 PRKCQ BCL10 [activation]
985 CASP2 CFLAR [activation]
986 PTK2B DOCK8 [activation]
987 MDM2 JUP [activation]
988 FYN CD79A [activation]

989	RAN	NUTF2	[activation]
990	STX3	STX4	[activation]
991	PRKCE	HSP90AA1	[activation]
992	ADCYAP1	FAP	[activation]
993	SP1	GTF2B	[activation]
994	CGA	FSHB	[activation]
995	RRAS2	TRAF2	[activation]
996	NFE2L3	PPARG	[activation]
997	DDIT3	TNFSF12	[activation]
998	DVL3	WWTR1	[activation]
999	CDK7	RPA3	[activation]
1000	ACTN3	TRAF2	[activation]
1001	PHGDH	MAPK6	[activation;inhibition]
1002	SERPINC1	PLCG1	[inhibition]
1003	PARD3	AMOTL2	[activation]
1004	GSK3B	CREM	[inhibition]
1005	CCDC8	GNB1	[activation]
1006	POU3F1	HMGB2	[activation]
1007	ATP1B1	BACE1	[activation]
1008	TGFB1	LAMTOR5	[activation]
1009	FYB	LCP2	[activation]
1010	PTPN1	SNAI1	[activation]
1011	UBTF	CCNE1	[activation]
1012	IL4R	DOK2	[activation]
1013	MOV10	BID	[activation]
1014	ADD2	FYN	[activation]
1015	CSNK1E	HES1	[activation]
1016	SHC1	DDR1	[activation]
1017	SHC1	SMAP	[activation]
1018	MYC	FASTKD2	[activation]
1019	BHLHE40	RXRA	[inhibition]
1020	PVR	CD96	[activation]
1021	PPP1CA	BAX	[inhibition]
1022	TRIP10	WASF1	[activation]
1023	BECN1	ATG5	[activation]
1024	ANKIB1	NR4A1	[inhibition]
1025	RRAS2	PTPN1	[activation]
1026	PCNA	RECQL5	[activation]
1027	HSPA4	CDK5RAP3	[activation]
1028	DAB1	LGALS9C	[activation]
1029	PFN1	PIK3R1	[activation]
1030	PDGFRA	FBXO25	[activation]
1031	RAPGEF2	LAP3	[activation]
1032	AURKB	PPP2CA	[activation]
1033	ACTB	SSH2	[activation]
1034	CLEC3B	FGF13	[activation]
1035	PYHIN1	LAMB1	[activation]
1036	TP53RK	NUP43	[activation]
1037	SNCA	MAPK8IP1	[inhibition]
1038	MBP	PDGFRL	[activation;inhibition]
1039	HIST3H3	ITGA4	[activation]
1040	GRB2	NEXN	[activation]
1041	PPARG	MED1	[activation]
1042	DDX3X	RAD51C	[activation]
1043	CLOCK	ARNTL2	[activation;inhibition]
1044	CHMP4B	TP53	[activation]
1045	TIAL1	OBSL1	[activation]
1046	HIST1H2BJ	CD81	[activation]
1047	PPP2R5B	MAPK1	[activation]
1048	CFLAR	NFKB1	[inhibition]
1049	RASA1	EIF1	[activation]
1050	BEX1	NGFR	[activation]
1051	IL1A	NFKBIE	[activation]
1052	ZNF71	PRKCZ	[activation]
1053	MYLK2	PCK1	[activation]
1054	TDGF1	ACVR1C	[activation]
1055	DOCK7	GRB7	[activation]
1056	RPAP3	LRRK2	[activation]
1057	UBAC2	PRKDC	[activation]
1058	JUN	CREB3	[activation]
1059	CREBBP	HLF	[activation]
1060	BAZ1B	HIST2H2AC	[activation]
1061	RIPK1	LRRK2	[activation]
1062	ACTB	ARRB2	[activation]
1063	STK11	SMARCC1	[activation]
1064	RIPK4	PRKCD	[activation]

1065	MYB	MAPK1	[activation]
1066	RALB	CALM1	[activation]
1067	IL13RA2	ESR2	[activation]
1068	C20orf24	GCN1L1	[activation]
1069	UBE2H	MARCH10	[activation]
1070	ROCK1	MYBPH	[activation]
1071	MEF2A	HDAC4	[activation]
1072	CSNK2B	FGF2	[activation]
1073	SOCS2	TCEB1	[inhibition]
1074	AKT2	PRKCZ	[activation]
1075	NXF1	OBSCN	[activation]
1076	BRD4	GRB2	[activation]
1077	SHANK3	PAX6	[activation]
1078	PIK3R1	RB1	[activation]
1079	EP300	RPS6KA5	[activation]
1080	PEAK1	CRK	[activation]
1081	NCR1	FCER1G	[activation]
1082	SIRT1	MYC	[activation]
1083	CASP3	WNK3	[activation]
1084	SUMO2	MYB	[activation]
1085	RAD51	CHEK1	[activation]
1086	LRMP	YWHAE	[activation]
1087	ESR1	ARFGAP2	[activation]
1088	SMAD2	EIF4A2	[activation]
1089	HLA-B	VDAC3	[activation]
1090	MAP1B	LRRK2	[activation]
1091	EGFR	PYCR2	[activation]
1092	TRAF2	TRIM42	[activation]
1093	SRPK2	TNFRSF10C	[activation]
1094	RIT1	MLLT4	[activation]
1095	DDX50	ESR1	[activation]
1096	STAT3	TSLP	[activation]
1097	SMAD3	EPAS1	[activation]
1098	YAP1	ERP44	[inhibition]
1099	SERPINE2	COL4A1	[inhibition]
1100	SRPK1	MAGEB1	[activation]
1101	SPTLC1	LRRK2	[activation]
1102	RAD21	AIRE	[activation]
1103	APC	GOLGA2	[inhibition]
1104	PPP2CA	CAV1	[activation]
1105	IL6ST	HCK	[activation]
1106	RAC1	CDC42SE1	[activation]
1107	FANCI	ARF6	[activation]
1108	CREBBP	IRF7	[activation]
1109	PTPN12	GRB2	[activation]
1110	CRTC1	CREB1	[activation]
1111	MSTO1	EP300	[activation]
1112	PRKRIR	DDX58	[activation]
1113	PAWR	GRB2	[activation]
1114	SCFD1	LRRK2	[activation]
1115	HOXA3	ARPC3	[activation]
1116	EPAS1	EP300	[activation]
1117	ANXA2	FLT1	[activation]
1118	RPL26	EIF4A3	[activation]
1119	HIF1A	JUN	[activation]
1120	ILK	ATP2A2	[activation]
1121	CCL3L1	CCR5	[activation]
1122	LATS1	SSX2IP	[inhibition]
1123	SOS1	ZAP70	[activation]
1124	YWHAB	MLLT4	[activation]
1125	IRAK3	LDB1	[inhibition]
1126	MAPK1	PEA15	[inhibition]
1127	RARA	SP1	[activation]
1128	ODF2L	PIPOX	[activation;inhibition]
1129	JAK3	KIT	[activation]
1130	ETFA	UBA5	[activation]
1131	SIRPB1	TYROBP	[activation]
1132	SPRY1	STAT3	[activation]
1133	MRPL43	SRPK1	[activation]
1134	SLC26A3	SLC9A3R1	[activation]
1135	FLNA	SH2B3	[activation]
1136	GNAO1	FFAR2	[activation]
1137	ABI1	ITGA4	[activation]
1138	GNAI2	HSP90AB1	[activation]
1139	STIM1	TRPC1	[activation]
1140	TAF1C	TP53	[activation]

1141 CHD8 CDC5L [activation]
1142 SLC6A1 HTT [activation]
1143 MYO18B CRK [activation]
1144 DDX39B GRB2 [activation]
1145 HIF1A EPAS1 [activation]
1146 IGF2 IDE [activation]
1147 GABARAPL2 PPP3CB [activation]
1148 BPNT1 TRAF6 [activation]
1149 FYN SYK [activation]
1150 ANXA2 SHC1 [activation]
1151 GNGT2 GNB3 [activation]
1152 FAM50A FOS [activation]
1153 PLCB1 TFCP2 [activation]
1154 SAG CHRM2 [activation]
1155 NRAS RAP1GDS1 [activation]
1156 NUP214 NXF3 [activation]
1157 ITGA4 MIF [activation]
1158 HRAS BLID [activation]
1159 SLC41A3 APP [activation]
1160 GLTSCR1 SRC [activation]
1161 IAPP RAMP2 [activation]
1162 LIMK1 CDC42BPA [activation]
1163 HSPB1 ILK [activation]
1164 ANXA7 PDHB [activation]
1165 CTGF LRP1 [activation]
1166 IRF1 SPI1 [activation]
1167 CACNA1C GNB1 [activation]
1168 RPA3 MYO1C [activation]
1169 SETD7 SOX2 [activation]
1170 BACE1 FLOT2 [activation]
1171 RPA3 MYO1F [activation]
1172 MAPK1 TH [activation]
1173 ANGPTL4 TGFB1 [activation]
1174 CCR5 DNM2 [activation]
1175 EPSTI1 PTPRJ [activation]
1176 TRIP10 CTNNB1 [activation]
1177 KCTD15 WIPI1 [activation]
1178 CDKN1B YWHAG [inhibition]
1179 RB1 FBP2 [activation;inhibition]
1180 EFNB2 GRM1 [activation]
1181 EP300 CLOCK [activation]
1182 EGFR SAMHD1 [activation]
1183 CALM1 TRAF2 [activation]
1184 CDK4 BRCA1 [inhibition]
1185 CALM1 GLP2R [activation]
1186 ITGA4 PRDX3 [activation]
1187 BMP1 LAMB3 [activation]
1188 ULK2 CSNK1D [activation]
1189 TUB SRC [activation]
1190 TULP3 ESRRG [activation]
1191 GMCL1 IL17F [activation]
1192 PAXIP1 SCCPDH [activation]
1193 HSP90AB1 RHOBTB1 [activation]
1194 DLG4 EFNB2 [activation]
1195 GRB2 TFAP2A [activation]
1196 PTH1R SLC9A3R1 [activation]
1197 NR3C1 NR4A1 [activation]
1198 CD4 HNRNPA2B1 [activation]
1199 MC1R MRAP2 [inhibition]
1200 GFRA1 SOCS3 [activation;inhibition]
1201 HSPA6 MAP3K3 [activation]
1202 PAX7 SRC [activation]
1203 SELPLG GALNT4 [activation]
1204 KPNA1 SKP2 [activation]
1205 RPTOR LAMTOR5 [activation]
1206 APP PPP1R17 [activation]
1207 YES1 THAP1 [activation]
1208 ITGB3 NUMB [activation]
1209 MARK4 CDC42 [activation;inhibition]
1210 FAM84B NOTCH2 [activation]
1211 SKP2 NLK [inhibition]
1212 WDR90 NUDC [activation]
1213 CKAP4 PRKCZ [activation]
1214 MAPK8 SNCG [activation]
1215 CFTR PPP2R1A [inhibition]
1216 ACTA1 VAV1 [activation]

1217	PIN1	SMAD2	[activation]
1218	JMJD6	GNA14	[activation]
1219	YWHAB	CDC25C	[activation]
1220	E2F3	DHX33	[activation]
1221	DVL1	SIPA1	[activation]
1222	IFNGR1	NFKB1	[inhibition]
1223	FOXJ1	GRB2	[activation]
1224	SHC1	GRAP2	[activation]
1225	TRAF6	MCM3	[activation]
1226	BHLHE40	BTRC	[activation]
1227	RAD18	CDC7	[activation]
1228	PIK3R3	ESR1	[activation]
1229	EGLN1	ZNF281	[activation]
1230	RHOC	LNX1	[activation]
1231	FGFR4	MTA3	[activation]
1232	ANXA7	QARS	[activation]
1233	ARNT	DIABLO	[activation]
1234	LRRK2	HIST1H2BB	[activation]
1235	GRB2	PPM1B	[activation]
1236	TAF1B	TBP	[activation]
1237	HRG	C1QB	[activation]
1238	CCR1	CCL14	[activation]
1239	FANCC	STAT1	[activation]
1240	GNAI1	PCK1	[activation]
1241	NEK11	MYC	[activation]
1242	DBN1	BAK1	[activation;inhibition]
1243	CTSG	F5	[inhibition]
1244	ENSA	ENO2	[activation]
1245	CDC23	ANAPC15	[activation]
1246	NUTM2F	MORN4	[activation]
1247	VASP	PFN2	[activation]
1248	PIAS1	AR	[inhibition]
1249	ARFGEF3	EGFR	[activation]
1250	GRB2	HSP90AA1	[activation]
1251	SORBS1	ACTA1	[activation]
1252	SASH1	SFN	[activation]
1253	TIMP2	PAICS	[activation]
1254	HSPA4	HSPA8	[inhibition]
1255	HMGXB3	CDK4	[inhibition]
1256	SH3KBP1	MAP3K4	[activation]
1257	EGFR	GNB2L1	[activation]
1258	PLCG1	GSN	[activation]
1259	HIST1H4A	DNMT3L	[activation]
1260	ADAM17	PDIA3	[activation]
1261	APC	MCM3AP	[inhibition]
1262	MTNR1B	GNAI2	[activation]
1263	RPS2	FGF3	[activation]
1264	SMARCD1	GRB2	[activation]
1265	ITGA4	NUP188	[activation]
1266	STAT5B	PTPN11	[activation]
1267	ERICH2	TNF	[activation]
1268	NOS2	YWHAE	[activation]
1269	OLFM2	GNB5	[activation]
1270	CBLN4	SRPK2	[activation]
1271	ANXA7	CTSB	[activation]
1272	UBE2R2	BTRC	[activation]
1273	MAPK6	XPO1	[activation]
1274	ELANE	CSF3R	[activation]
1275	IKBKG	VAMP3	[activation]
1276	DVL2	PPM1A	[activation]
1277	RGPD4	PLK1	[activation;inhibition]
1278	PRKCA	PPARG	[activation]
1279	MAPK1	CDX2	[activation]
1280	NRAP	VCL	[activation]
1281	WBP11	ARF1	[activation]
1282	UBE2I	DDX21	[activation]
1283	ATF2	CEBPG	[activation]
1284	YAP1	SOCS6	[inhibition]
1285	TRAF3	TNFRSF14	[activation]
1286	XPO1	NUP50	[activation]
1287	MEP1A	PTH	[activation]
1288	PIK3R1	GABBR1	[activation]
1289	NOS3	ELAVL3	[activation]
1290	EIF4G2	MAGEA6	[activation]
1291	SMAD3	SMAD1	[activation]
1292	ESR1	LARS	[activation]

1293	CSF2RB	CSF2RA	[activation]
1294	CCL4	CCL3	[activation]
1295	PIK3CA	GABRB1	[activation]
1296	CAV1	PLD2	[activation]
1297	CLDN4	EPHA2	[activation]
1298	SERPINE1	VTN	[inhibition]
1299	RAC1	HPS4	[activation]
1300	RARA	KAT2A	[activation]
1301	SRPK2	SNRPC	[activation]
1302	HAND1	MYOD1	[activation]
1303	CDC25C	EGFR	[activation]
1304	LRRK2	RANBP2	[activation]
1305	GRHPR	CDC7	[activation]
1306	NEDD9	CDH1	[activation]
1307	PPIF	TP53	[activation]
1308	PLCG1	IRS2	[activation]
1309	CCNB1	CDC14A	[inhibition]
1310	INCA1	EPHB6	[activation]
1311	YWHAE	H2AFX	[activation]
1312	SH2D3C	SRC	[activation]
1313	RASD2	SMAD4	[activation]
1314	SMAP1	APP	[activation]
1315	MS4A12	PLLP	[activation]
1316	CDC5L	NSUN5	[activation]
1317	RBL1	CREG1	[inhibition]
1318	FAM53C	YWHAE	[activation]
1319	C8orf33	GABARAP	[activation]
1320	ARHGEF11	CDC42	[activation]
1321	LRRK2	YWHAB	[activation]
1322	CHTOP	SRPK2	[activation]
1323	USP20	HIF1A	[activation]
1324	CBL	EPHA8	[activation]
1325	RGS7	PRKCA	[activation;inhibition]
1326	CDK8	CCND1	[activation]
1327	FBXO5	ENO1	[inhibition]
1328	BCL6	EPHB6	[activation]
1329	HSPB1	SUMO3	[inhibition]
1330	GSK3B	BZW2	[activation]
1331	HIST1H4A	MAPK3	[activation;inhibition]
1332	MAPK1	SCNN1G	[activation]
1333	IKBKG	PRKCB	[activation]
1334	TTK	CDC27	[activation]
1335	MAP3K5	FAS	[inhibition]
1336	MAPKAP1	NBN	[activation]
1337	NR5A2	NRIP1	[activation]
1338	STK11	USP11	[activation]
1339	NXT1	PRKAB1	[activation]
1340	ROBO4	ENAH	[activation]
1341	TIMP1	CD63	[activation]
1342	IQGAP1	GRB2	[activation]
1343	GNAO1	RIC8A	[activation;inhibition]
1344	CD33	PTPN11	[activation]
1345	GRB7	FLAD1	[activation]
1346	ENGASE	EGFR	[activation]
1347	LCK	DOK3	[activation]
1348	PML	ZFYVE9	[activation]
1349	PLCG2	EPOR	[activation]
1350	TIAM1	ANK3	[activation]
1351	CSK	ARRB2	[activation]
1352	FLNA	IKBKB	[activation]
1353	ADRBK1	GIT2	[activation]
1354	BTBD2	TP53	[activation]
1355	RHOQ	PFN1	[activation]
1356	WT1	MDM2	[activation]
1357	GRN	KRTAP26-1	[activation]
1358	CDK1	PPP1CC	[inhibition]
1359	YWHAZ	HIST1H2BB	[activation]
1360	RAF1	TBXA2R	[activation;inhibition]
1361	SMARCB1	YWHAZ	[activation]
1362	PTPN1	NTRK3	[activation]
1363	CASP4	PSEN1	[activation]
1364	NRAS	RASSF5	[activation]
1365	SFN	REEP4	[activation]
1366	ADRBK1	SNCA	[activation]
1367	MCM6	MCM3	[activation]
1368	CCNG1	APP	[activation]

1369	MYO18A	MYC	[activation]
1370	FBXO28	HSP90AB1	[activation]
1371	MST1R	SRC	[activation]
1372	PASK	IL2RA	[activation]
1373	YWHAZ	FASN	[activation]
1374	CDK1	EP300	[activation]
1375	WDR44	RAB11A	[activation]
1376	GHRL	AGRP	[activation]
1377	FHL3	MAPK1	[activation;inhibition]
1378	SENP3	LRRK2	[activation]
1379	FSCN1	VCAM1	[activation]
1380	MAPK1	LIFR	[activation]
1381	RXRA	STAT1	[inhibition]
1382	MRE11A	RRM2B	[activation]
1383	BTK	TEC	[activation]
1384	HIST3H3	DEK	[activation]
1385	FBXW11	RASSF1	[inhibition]
1386	ABI3	FARSA	[activation]
1387	TBP	MDM2	[activation]
1388	ASNS	STRN4	[activation]
1389	HBEGF	CD44	[activation]
1390	TIMP3	AGTR2	[activation]
1391	CS	MDM2	[activation]
1392	FYN	SKAP2	[activation]
1393	SIRPA	PTPN11	[activation;inhibition]
1394	ABL1	GPR45	[activation]
1395	IGHG1	PDPK1	[activation]
1396	FARSA	EGFR	[activation]
1397	PMS1	ATR	[activation]
1398	CSF1R	YES1	[activation]
1399	IRS4	MTNR1B	[activation]
1400	PRKCZ	HIST1H1A	[activation]
1401	VCAM1	DIAPH1	[activation]
1402	WFIKKN1	BMP4	[activation]
1403	MUC2	MLH1	[activation]
1404	TLR4	F2RL1	[activation]
1405	PTPRK	CBL	[inhibition]
1406	SRC	MPZL1	[activation]
1407	KLHL26	NUDCD3	[activation]
1408	RLIM	ISL1	[activation]
1409	ITGB7	DOK1	[activation]
1410	SOX9	HSPA1A	[activation]
1411	DDX21	ESR1	[activation]
1412	PDPK1	PTPRG	[activation]
1413	MKNK1	DDAH2	[activation]
1414	MYC	CYR61	[activation;inhibition]
1415	CSNK2A1	PRKACB	[activation]
1416	AURKB	LATS1	[activation]
1417	CDKN1B	DCLRE1C	[activation;inhibition]
1418	TRIM24	STAT6	[activation]
1419	GABARAP	NSMAF	[activation]
1420	CDK6	VGLL4	[inhibition]
1421	EIF4EBP3	EIF4E	[inhibition]
1422	EIF1AX	PINX1	[activation]
1423	ESR1	RFX4	[activation]
1424	ENAH	CDC5L	[activation]
1425	YWHAB	SLC9A1	[activation]
1426	CALCA	NRD1	[activation]
1427	PLCL2	PPP1CA	[activation]
1428	NXF1	MIEN1	[activation]
1429	SDF4	RAP1B	[activation]
1430	MRPL4	DDX24	[activation]
1431	YWHAB	MAP3K3	[activation]
1432	STK4	COPG1	[activation]
1433	LRRK1	PTPN11	[activation]
1434	C7orf31	KLHL40	[activation]
1435	CASP8	SPP1	[activation]
1436	SMAD1	CREBBP	[activation]
1437	APC	PROCR	[activation]
1438	CBLB	SPRY1	[inhibition]
1439	ICAM1	SART3	[activation]
1440	SSH1	ACTA1	[activation]
1441	GNAS	CRHR1	[activation]
1442	PECAM1	SDC4	[activation;inhibition]
1443	CRK	SHANK3	[activation]
1444	MAPK6	OSTF1	[activation;inhibition]

1445	TP53	TUBA8	[activation]
1446	WNK4	OXSR1	[activation]
1447	HDAC1	PPARG	[activation]
1448	PRKCI	IL1RAP	[activation]
1449	LIMK1	PRKACA	[activation;inhibition]
1450	NME7	CDK5RAP2	[activation]
1451	PML	PYCARD	[inhibition]
1452	ARRB2	AVPR2	[inhibition]
1453	RPA4	MUTYH	[activation]
1454	IGFBP1	MMP26	[activation]
1455	FTSJ3	DDX56	[activation]
1456	VDAC1	TERT	[activation]
1457	PROP1	CTNNB1	[activation]
1458	NEURL4	TP53	[activation]
1459	EPB41	EGFR	[activation]
1460	RAP1GAP	PFN2	[activation]
1461	PLG	HRG	[inhibition]
1462	EGFR	VPS13A	[activation]
1463	MAPK12	MAPK15	[activation;inhibition]
1464	TES	EVL	[activation]
1465	SIGLEC12	PTPN6	[activation;inhibition]
1466	MIPEP	ILK	[activation]
1467	VCL	ITGA4	[activation]
1468	EIF2S2	KIAA1377	[activation]
1469	NBN	CDK9	[activation]
1470	SMAD4	ARL5B	[activation]
1471	DKC1	ARRB2	[activation]
1472	EP300	SRY	[activation]
1473	HSPA1A	BAG2	[inhibition]
1474	CHUK	PPP2CA	[activation]
1475	PFN1	VCAM1	[activation]
1476	CD40LG	TRAF2	[activation]
1477	APP	YWHAG	[activation]
1478	YWHAG	WEE1	[inhibition]
1479	HCK	FLT1	[activation]
1480	CREBBP	SRC	[activation]
1481	RORC	PPP1CC	[inhibition]
1482	CD19	FYN	[activation]
1483	ACVR2A	BMP10	[activation]
1484	MYC	ERP44	[activation]
1485	CRADD	IL9R	[activation]
1486	SHC1	ARAP1	[activation]
1487	LGALS13	ANXA2	[activation]
1488	NADK	GRB2	[activation]
1489	CD3E	JAK3	[activation]
1490	NCK2	NTRK2	[activation]
1491	ATP1A1	MDM2	[activation]
1492	NSD1	ESR1	[activation]
1493	SRPK1	CLK1	[activation]
1494	PRKDC	NFKB1	[activation]
1495	PDCD6	ANXA7	[activation]
1496	LMO1	GATA3	[inhibition]
1497	MYC	KALRN	[activation]
1498	EIF4A3	GRB2	[activation]
1499	TRAF6	MARS	[activation]
1500	RELA	REPS2	[activation]
1501	SMARCB1	HDAC4	[activation]
1502	FTSJ1	RHPN2	[activation]
1503	ITGB3	FGA	[activation]
1504	BCL6	PANX3	[activation]
1505	SOCS2	KIT	[inhibition]
1506	EGFR	GBF1	[activation]
1507	APP	KISS1	[activation]
1508	PRMT2	E2F1	[inhibition]
1509	ATXN10	STYXL1	[activation]
1510	HSPB1	HNRNPD	[activation]
1511	TP53	TRIM39	[activation]
1512	USP53	GRB2	[activation]
1513	MMP9	CXCL6	[activation]
1514	SREBF2	ITGB4	[activation]
1515	ACKR3	PECAM1	[activation]
1516	TRAF6	NGFRAP1	[activation]
1517	STIM1	CAV1	[activation]
1518	APP	UNG	[activation]
1519	STK38	HSP90AB1	[activation]
1520	FN1	GNAI3	[activation;inhibition]

1521	DLG5	TERF1	[activation]
1522	TIAM1	SDC3	[activation]
1523	PPP1R9B	DRD2	[activation]
1524	CEBPB	CREB1	[activation]
1525	EP300	NOXA1	[activation]
1526	APC	CSNK1A1	[inhibition]
1527	ELAVL1	DDX39B	[activation]
1528	ACTG1	EPS8L2	[activation]
1529	NRXN1	MACF1	[activation]
1530	CDC25C	LCK	[activation]
1531	APBA2	RELA	[activation]
1532	ACTN3	YWHAZ	[activation]
1533	DUSP15	PIK3R1	[activation]
1534	PIGR	SGK1	[activation]
1535	SMAD2	BPTF	[activation]
1536	MDM4	CHEK1	[activation;inhibition]
1537	CALML5	TGM3	[activation]
1538	SHC1	PLSCR1	[activation]
1539	RASA4	APMAP	[activation]
1540	PGR	SMARCA1	[activation]
1541	PROS1	PROC	[activation;inhibition]
1542	ZAK	RPS6KA5	[activation]
1543	AKT1	WNK1	[activation]
1544	DDX39B	EXOSC9	[activation]
1545	CTNNB1	CCNA2	[activation]
1546	HSP90AB1	SETDB1	[activation]
1547	HDAC1	DDX17	[activation]
1548	ARHGDIA	FADD	[activation]
1549	THBS1	IGFBP5	[inhibition]
1550	HSPA1A	TSSK6	[inhibition]
1551	ESR2	SYNGAP1	[activation]
1552	CD19	IGHM	[activation]
1553	HMGB1	CEBPB	[activation]
1554	CDC42BPG	SFN	[activation]
1555	YES1	FLT1	[activation]
1556	MAPK8	SCOC	[activation]
1557	NCF1	PAK1	[activation]
1558	NUTF2	NUP54	[activation]
1559	PDZRN3	MUSK	[inhibition]
1560	SP1	CRK	[activation]
1561	ILK	RHOT2	[activation]
1562	CDK11B	CASP3	[activation]
1563	RYR1	HOMER1	[activation]
1564	ESR1	SMARCA2	[activation]
1565	SOD1	SMAD2	[inhibition]
1566	APOB	LRP6	[activation]
1567	GJB1	CAV1	[activation]
1568	AMOTL2	MYC	[activation]
1569	PRKAA2	HOMEZ	[inhibition]
1570	KIT	PDGFRA	[activation]
1571	GSK3B	RELA	[activation]
1572	GRB2	SMAD3	[activation]
1573	PIN1	FTSJ1	[activation]
1574	PPP2CA	H2AFX	[inhibition]
1575	SMAD3	C10orf118	[inhibition]
1576	FBXO25	EGFR	[activation]
1577	RAB3IP	RAB11A	[activation]
1578	ARRB2	MLLT4	[activation]
1579	CCL24	CCR3	[activation]
1580	CDC25C	HSP90AA1	[activation]
1581	MEIS1	HOXB13	[activation]
1582	CTNNB1	PTPRO	[activation]
1583	RHOB	NME2	[activation]
1584	VCAM1	HSPA8	[activation]
1585	POLR3D	MAPK11	[activation]
1586	ERBB2	PTPRC	[activation]
1587	ADRA2B	GNAI2	[activation]
1588	PRKCQ	HSF1	[activation]
1589	ACTR2	ACTR3	[activation]
1590	SRPK1	H2AFY	[activation]
1591	PAG1	SHC1	[activation]
1592	MAPKAP1	RPS6	[activation]
1593	SUV39H1	CDC23	[activation]
1594	PRKCZ	BTK	[activation]
1595	TNF	RNF31	[activation]
1596	PDS5A	EGFR	[activation]

1597	POLR1A	NBN	[activation]
1598	MMP2	SPOCK1	[inhibition]
1599	PRMT1	EIF4A3	[activation]
1600	SRGAP2	PPP2R1A	[activation]
1601	PTGS2	ELAVL1	[activation]
1602	TEC	PIP4K2A	[activation]
1603	MAPK13	HIST1H1C	[activation]
1604	ACVR1	TGFBR2	[activation;inhibition]
1605	ETS1	NCL	[activation]
1606	PPP3CA	RXRB	[inhibition]
1607	ATR	ATRIP	[activation]
1608	GNB2L1	NR3C1	[activation]
1609	DAB1	TMTC3	[activation]
1610	GNAS	LHCGR	[activation]
1611	POLR2A	CDKN1A	[activation]
1612	CDK4	SETDB1	[activation]
1613	MCTS1	HLA-B	[activation]
1614	ROCK1	HNRNPA1	[activation]
1615	CAMK1	SYN1	[activation]
1616	NXF1	PODXL	[activation]
1617	IRF2	NFKB1	[activation]
1618	CLCN5	CFL1	[activation]
1619	NGFR	MAGED1	[activation]
1620	SOX2	CTNNB1	[activation]
1621	TBX2	SUMO1	[activation]
1622	PCDH1	SMAD3	[activation]
1623	TRAF6	PLEKHO1	[activation]
1624	PACSIN2	PACSIN1	[activation]
1625	LCP2	TXK	[activation]
1626	NR0B1	NR3C1	[activation]
1627	ANAPC1	SNW1	[activation]
1628	GAB2	TNFRSF11A	[activation]
1629	SHC1	FCGR2B	[activation]
1630	MAPK14	EEA1	[activation]
1631	RAB25	TGFBR1	[activation]
1632	ISG15	RAN	[activation]
1633	CDKN2C	REL	[inhibition]
1634	UTS2B	UTS2R	[activation]
1635	E2F3	BCL6	[activation]
1636	ARFGAP1	RGS2	[activation]
1637	MAPK4	FASTKD5	[activation;inhibition]
1638	ZAP70	FYN	[activation;inhibition]
1639	KIF23	BIRC6	[activation]
1640	LTK	PIK3R1	[activation]
1641	GRB2	MAP4K5	[activation]
1642	MAPK11	MBP	[activation]
1643	MGA	E2F1	[activation]
1644	SRPK2	TRUB1	[activation]
1645	TRAF3	DDX58	[activation]
1646	PRKG1	BMPR2	[activation]
1647	CDC42	MYO6	[activation]
1648	GAPVD1	YWHAZ	[activation]
1649	HSP90AA1	SGK223	[activation]
1650	THBS1	KNG1	[activation]
1651	CASP7	RAD21	[activation]
1652	TP53	PARD3	[activation]
1653	ITGA4	MCM2	[activation]
1654	NCK2	SRPK2	[activation]
1655	FLNA	APC	[inhibition]
1656	CRK	MAP4K1	[activation]
1657	ABL1	CTAGE5	[activation]
1658	C9orf156	PTPRJ	[activation]
1659	CTNNB1	FOXO1	[activation]
1660	MAP2K1	YAP1	[activation]
1661	GADD45G	PTPRK	[inhibition]
1662	BCL2L11	BCL2L1	[activation]
1663	HCLS1	ACTR2	[activation]
1664	PPP2R1A	STK24	[activation]
1665	BUB3	APC	[inhibition]
1666	CASQ2	ANKRD1	[activation]
1667	ABL1	SFN	[activation]
1668	WHSC1	RANGAP1	[activation]
1669	ADAMTS12	CALM1	[activation]
1670	BAX	VRK2	[activation]
1671	TEK	GRB7	[activation]
1672	ANK3	CRKL	[activation]

1673	NR4A1	PGBD1	[inhibition]
1674	INPP1	LIG1	[activation]
1675	VCAM1	IST1	[activation]
1676	SRPK1	SERF1A	[activation]
1677	ILK	ACACA	[activation;inhibition]
1678	MARS	MAP3K3	[activation]
1679	CTNNB1	STK39	[activation]
1680	CAB39L	SRPK1	[activation]
1681	SREBF2	CASP8AP2	[activation]
1682	KPNA4	RAC1	[activation]
1683	ABL1	VPREB1	[activation]
1684	SRC	DOCK1	[activation]
1685	APP	AKAP1	[activation]
1686	ZC2HC1C	SFN	[activation]
1687	NFYB	TP53	[activation]
1688	FCGR2B	FYN	[activation]
1689	IL15	STAT5B	[activation]
1690	HNF1A	KAT2B	[activation]
1691	EP300	STAT1	[activation]
1692	TNFRSF1A	RHOA	[activation]
1693	SRSF5	PPARGC1A	[activation]
1694	CDC42	BCR	[activation]
1695	SMARCB1	GATA1	[activation]
1696	CTCF	PYHIN1	[activation]
1697	SUV39H1	RB1	[activation]
1698	STX16	STX6	[activation]
1699	CYTH2	PRKCB	[activation]
1700	STK3	GABARAPL1	[activation]
1701	PPP2R2B	RAC1	[activation]
1702	ZFPM2	GATA1	[activation]
1703	MAST1	SNTA1	[activation]
1704	KMT2A	MYB	[activation]
1705	HNRNPA0	GADD45A	[activation]
1706	LRRC29	KLHL40	[activation]
1707	SMAD4	AR	[activation]
1708	MAPK14	EEF2K	[activation]
1709	VCL	VASP	[activation]
1710	EGFR	CCDC50	[activation]
1711	HMHA1	YWHAE	[activation]
1712	YTHDC2	USP42	[activation]
1713	MMRN1	F5	[inhibition]
1714	CASP2	MDM2	[activation]
1715	MAST3	PPP6C	[inhibition]
1716	LTBP3	TGFB1	[activation]
1717	CD247	PTPN6	[activation;inhibition]
1718	BRD4	TWIST1	[activation]
1719	APP	GNRH2	[activation]
1720	TGM2	SRC	[activation]
1721	COX17	PPBP	[activation]
1722	NEK8	HSP90AA1	[activation]
1723	PPID	HSF1	[activation]
1724	TBL3	ITSN2	[activation]
1725	TCF7L2	CTNNBIP1	[inhibition]
1726	YWHAB	SRPK1	[activation]
1727	MET	PTPRG	[activation;inhibition]
1728	HABP2	KNG1	[inhibition]
1729	APP	NIM1	[activation]
1730	IGF2BP1	VCAM1	[activation]
1731	SRC	PRKACA	[activation]
1732	ACTA1	CDH2	[activation]
1733	EP300	PRDM2	[activation]
1734	PTPN11	STAT5A	[activation]
1735	PKM	ENO3	[activation]
1736	RIPK2	PRMT2	[activation]
1737	SH3KBP1	RAB5A	[activation]
1738	PAK3	ARHGEF7	[activation]
1739	PAXIP1	RAD50	[activation]
1740	HSPA4	GZMA	[activation]
1741	HDAC3	SREBF2	[activation]
1742	MDC1	YWHAZ	[activation]
1743	DDX5	PHLDA3	[activation]
1744	FGF5	MAPK1	[activation]
1745	PI4K2A	NOS3	[activation]
1746	NUCKS1	DOCK9	[activation]
1747	PTPN12	KIT	[activation]
1748	ACTB	NOS2	[activation]

1749	WFS1	CFTR	[activation]
1750	FAM9B	CDC23	[activation]
1751	MXD1	CD2	[activation]
1752	CCDC8	EIF2S1	[activation]
1753	EIF3I	TNFRSF14	[activation]
1754	ACVR2B	ACVR1B	[activation]
1755	NABP2	TP53	[activation]
1756	APC	ANP32B	[inhibition]
1757	PIP4K2A	EPB41L3	[activation]
1758	CBLB	TICAM1	[activation]
1759	IL2RB	IL15RA	[inhibition]
1760	HIST2H2AB	EP300	[activation]
1761	PCDHA10	CRK	[activation]
1762	TNK2	CDC42	[activation]
1763	EGFR	EXOC3	[activation]
1764	GABARAP	IGF2BP1	[activation]
1765	FGG	ITGB3	[activation]
1766	CCDC115	PRKCZ	[activation]
1767	FLI1	ELK1	[activation]
1768	CDK11A	SKIV2L	[activation]
1769	RHOH	TGFBR1	[activation]
1770	STX1A	ABCC9	[activation]
1771	STAT1	STAT5A	[activation]
1772	GNAL	USP3	[inhibition]
1773	BTK	DAAM1	[activation]
1774	NR5A2	NCOA3	[activation]
1775	YWHAE	IRS4	[activation]
1776	RPA3	PCF11	[activation]
1777	CALM1	STK11	[activation]
1778	PML	RELA	[activation]
1779	TMEM63B	MAPK14	[activation]
1780	EEA1	IGF1R	[activation]
1781	SIRT1	KAT2B	[activation]
1782	CMBL	H2AFX	[activation]
1783	AURKA	APP	[activation]
1784	CAMLG	IER3	[activation]
1785	ARFGEF1	NUP62	[activation]
1786	HSP90AB1	TP53RK	[activation]
1787	FES	ABI1	[activation]
1788	NDEL1	YWHAG	[activation]
1789	CGA	PTPN12	[activation]
1790	KPNB1	MYC	[activation]
1791	PPP2R1B	ESPL1	[inhibition]
1792	IQCB1	VAMP2	[activation]
1793	CTTN	ESR2	[activation]
1794	FBP2	BUB1	[activation]
1795	GRIA2	PRKCA	[activation]
1796	TRABD	HIPK4	[activation]
1797	IL6ST	SHC1	[activation]
1798	CCT3	ILK	[activation]
1799	CASP7	PANX1	[activation]
1800	CUL1	HSPA8	[inhibition]
1801	EIF4E	PPP2CA	[inhibition]
1802	MDC1	PRKDC	[activation]
1803	MAPK1	PRPSAP1	[inhibition]
1804	LCE1B	OTX1	[activation]
1805	SRPK1	LBR	[activation]
1806	FOS	ARFGAP1	[activation]
1807	ABR	ECT2	[activation]
1808	SRC	ADAM17	[activation]
1809	MAP2K3	TINF2	[activation]
1810	PPP1CC	PPP1R15A	[inhibition]
1811	LAMC1	SRPK1	[activation]
1812	FNBP4	FYN	[activation]
1813	UNC13B	BARD1	[activation]
1814	FBXO6	PROS1	[inhibition]
1815	AURKA	RAB10	[activation]
1816	CDK2	CCNE2	[activation]
1817	SSFA2	WNK1	[activation]
1818	GNAI1	ESR1	[activation]
1819	OS9	EGLN1	[activation]
1820	TMSB4X	GRB7	[activation]
1821	BCAR3	ACTR2	[activation]
1822	CYFIP1	RAC1	[activation]
1823	WNK1	ARAF	[activation]
1824	MAX	CLIP2	[inhibition]

1825	SRPK3	RRP1B	[activation]
1826	OPN1LW	RANBP2	[activation]
1827	NUP93	MYC	[activation]
1828	BCL3	CTNNB1	[activation]
1829	PAX6	CDX2	[activation]
1830	HLA-B	GLRX3	[activation]
1831	PIK3CA	FANCC	[activation]
1832	DFFA	YWHAB	[activation]
1833	PRAP1	GRB2	[activation]
1834	TCF7L2	PSEN1	[inhibition]
1835	FGF7	UBQLN1	[activation]
1836	ILK	UNC45A	[activation]
1837	CHMP4A	F2R	[activation]
1838	FYN	DNASE1L2	[activation]
1839	INSR	DOK5	[activation]
1840	LRP1	YWHAG	[activation]
1841	MAP3K7	DARS	[activation]
1842	CSF1R	IL34	[activation]
1843	TSC22D1	MYO1B	[activation]
1844	APP	AURKB	[activation]
1845	CTTNBP2	PPP2CA	[inhibition]
1846	MATK	ERBB2	[activation]
1847	TCAP	MYOZ2	[inhibition]
1848	DNAJA1	TAB1	[inhibition]
1849	ARPC2	ARPIN	[activation]
1850	TNF	NT5DC3	[activation]
1851	ITGA4	SCRIB	[activation]
1852	CBL	CDKL2	[activation]
1853	GRB2	SNRNP200	[activation]
1854	CDK2	POLL	[activation]
1855	DNTTIP2	RXRA	[inhibition]
1856	C1QA	COL2A1	[activation]
1857	FOS	SUPT6H	[activation]
1858	AURKA	MTA3	[activation]
1859	KPNA2	CTCF	[activation]
1860	ANKS1B	HTR2A	[activation]
1861	ESR1	RDX	[activation]
1862	RPS6KA5	PDLIM1	[activation]
1863	EP300	MRE11A	[activation]
1864	APP	ASPSCR1	[activation]
1865	TRAF3	CFLAR	[inhibition]
1866	CRLF1	CNTFR	[activation]
1867	CCND1	FANCC	[activation]
1868	PAK1	GIT1	[activation]
1869	ILK	SDF4	[activation]
1870	PTEN	ANG	[activation]
1871	ATG7	SLX4	[activation]
1872	TRAP1	CEP250	[activation]
1873	HLA-B	SUPT16H	[activation]
1874	SMARCA4	ATF3	[activation]
1875	TRAF6	PREP	[activation]
1876	GPSM1	CUL3	[inhibition]
1877	YWHAE	MLLT4	[activation]
1878	KPNB1	NOTCH1	[activation]
1879	H3F3A	RNF41	[activation]
1880	PLG	LAMA5	[activation;inhibition]
1881	ACTG1	EZH2	[activation]
1882	H2AFX	CFL1	[activation]
1883	AURKA	IK	[activation]
1884	TRAF5	RIPK2	[activation]
1885	TP53	RRP1B	[activation]
1886	PRNP	FGF13	[activation]
1887	LAT	EGFR	[activation]
1888	BCAR1	SH2D3C	[activation]
1889	RPAP3	DPCD	[activation;inhibition]
1890	ATP13A2	COL1A1	[activation]
1891	ILK	ABCE1	[activation]
1892	DDX47	MLH1	[activation]
1893	EGFR	SH2B1	[activation]
1894	CASP2	BID	[activation]
1895	POT1	AIM2	[activation]
1896	CFLAR	IKBKB	[inhibition]
1897	MAGEC1	ROCK2	[activation]
1898	GCN1L1	FBXO6	[inhibition]
1899	CDK1	CDKN1B	[activation;inhibition]
1900	ARHGEF1	SREBF2	[activation]

1901	IFNA1	CR2	[activation]
1902	IKBKB	CREBBP	[activation]
1903	FAS	PRKCA	[activation]
1904	CCDC102B	CCHCR1	[activation]
1905	PLCB1	TRPM7	[activation]
1906	BARD1	CPSF3	[activation]
1907	PCNA	BARD1	[activation]
1908	KIR2DL4	HLA-G	[activation]
1909	RPS6KA1	L1CAM	[activation]
1910	TWF1	BTK	[activation]
1911	NCKAP1	CYFIP2	[inhibition]
1912	CCHCR1	NUP62	[activation]
1913	EXOC1	TRIO	[activation]
1914	ENO2	AKT1	[activation]
1915	KLHL1	CACNA1A	[inhibition]
1916	NCK1	DRD3	[activation]
1917	RYR1	TTN	[activation]
1918	INSR	CYTH2	[activation]
1919	PPFIBP1	TP53	[activation]
1920	GP1BA	GP9	[activation]
1921	NOS3	A2M	[inhibition]
1922	MAP3K14	HSPA6	[activation]
1923	SFN	EIF2S1	[activation]
1924	AKT2	SLC2A4	[activation]
1925	FBXW7	MAP2K1	[activation]
1926	EZH2	XPO1	[activation]
1927	TRADD	DAB2IP	[activation]
1928	MAD2L1	MAD2L1BP	[inhibition]
1929	SOCS1	CUL2	[inhibition]
1930	APBB2	EGFR	[activation]
1931	GABARAPL2	PTPRA	[activation]
1932	CCDC8	CXADR	[activation]
1933	DLX2	PIK3R1	[activation]
1934	JAG1	MLLT4	[activation]
1935	LRRK2	YWHAE	[activation]
1936	YTHDF2	RPA1	[activation]
1937	CRK	PCDHA7	[activation]
1938	TP53	STAT6	[activation]
1939	NXF1	FAM83G	[activation]
1940	GOLGA2	NDEL1	[activation]
1941	CDC16	MDC1	[activation]
1942	EVL	CRKL	[activation]
1943	IBTK	CD81	[activation]
1944	CDKN2A	SERTAD1	[activation;inhibition]
1945	PLXNB2	MST1R	[activation]
1946	CFTR	DNAJB1	[inhibition]
1947	BRI3BP	TRAF6	[activation]
1948	JAK3	SH2B2	[activation]
1949	CYCS	GSTK1	[activation]
1950	RPA1	RPS6KA5	[activation]
1951	RET	PTK2	[activation;inhibition]
1952	IK	GRB2	[activation]
1953	MTOR	SUMO1	[activation]
1954	RYR1	NCK1	[activation]
1955	TSC22D4	PRKAA1	[inhibition]
1956	BID	CSNK1E	[activation]
1957	TNFRSF1B	MAD2L1	[inhibition]
1958	GRIA2	DLG4	[activation]
1959	IL5	IL8	[activation]
1960	TRAF1	TRAF3	[activation]
1961	TRAF6	ARPC2	[activation]
1962	HLA-G	HLA-A	[activation]
1963	SDCBP	EPHB2	[activation]
1964	CD44	SCYL3	[activation]
1965	MCM7	CCNH	[activation]
1966	MAST1	YWHAZ	[activation]
1967	BARD1	TRAPPC11	[activation]
1968	LRP1	NOTCH3	[activation]
1969	PPP2CA	CCNG2	[inhibition]
1970	CREM	SPI1	[inhibition]
1971	ARHGDIA	SUMO4	[activation]
1972	SH2D2A	EGFR	[activation]
1973	MDM2	NPM3	[activation]
1974	UBE2E3	DET1	[activation]
1975	SUMO4	PRDX3	[activation;inhibition]
1976	ZNHIT3	ESR2	[activation]

1977	ADRB2	RNF41	[activation]
1978	SMAD2	CDK4	[inhibition]
1979	WHSC1	RAD50	[activation]
1980	PPP2CA	TLX1	[activation]
1981	PKM	ANXA7	[activation]
1982	SNAI1	MAPK1	[activation;inhibition]
1983	SOS1	PTPN6	[activation;inhibition]
1984	AGK	ICT1	[activation]
1985	FYN	GCFC2	[activation]
1986	EPRS	EGFR	[activation]
1987	CA4	SLC4A3	[activation]
1988	MYL12B	HSPB1	[activation]
1989	PTEN	PPP2R4	[activation]
1990	PRKACA	LCP1	[activation]
1991	POMGNT2	NOTCH2NL	[activation]
1992	FAM13B	WNK1	[activation]
1993	GSK3B	PRKCB	[inhibition]
1994	SH2D1A	SLAMF7	[activation]
1995	DNER	PSEN1	[activation]
1996	PIK3CA	ITIH1	[activation]
1997	RHOJ	WAS	[activation]
1998	MDM4	BCL2	[activation;inhibition]
1999	PPP2CA	ARL2	[inhibition]
2000	NEK3	VAV2	[activation]
2001	ANAPC7	HSPB1	[activation]
2002	DDX24	GABARAPL2	[activation]
2003	HIF1A	SAT2	[activation]
2004	CIT	RHOA	[activation]
2005	SRC	ENO1	[activation]
2006	SRPK1	SETD3	[activation]
2007	RELA	MAP3K7	[activation]
2008	ACD	SBDS	[activation]
2009	PXN	BCR	[activation]
2010	CAMK2A	DLG1	[activation]
2011	GRB2	UBA1	[activation]
2012	EIF4EBP1	REL	[inhibition]
2013	BCL3	NFKB2	[activation]
2014	AR	PXN	[activation]
2015	NME3	JAM2	[activation]
2016	BAG1	HBEGF	[activation]
2017	IRF3	BAX	[inhibition]
2018	FLI1	GATA1	[activation]
2019	NCK2	ERBB3	[activation]
2020	SORCS2	NGF	[activation]
2021	FOS	HSP90B1	[activation]
2022	F8	MAP1LC3A	[activation]
2023	PTPRJ	GAB1	[activation]
2024	HDGF	RAN	[activation]
2025	EIF4G1	TRAF6	[activation]
2026	FDX1	CYP11A1	[activation]
2027	CAMP	ELANE	[activation]
2028	STK35	TGFBR1	[activation]
2029	COX4I2	CYCS	[inhibition]
2030	GTF2B	RELA	[activation]
2031	ANXA7	GDF9	[activation]
2032	EP300	SERTAD1	[activation]
2033	PTEN	CAV1	[activation]
2034	MPL	ATXN2L	[inhibition]
2035	SMYD3	CAMK2B	[activation]
2036	ERBB3	DAPP1	[activation]
2037	CRLF2	JAK2	[activation]
2038	WDR26	EGFR	[activation]
2039	STAT3	STMN1	[activation]
2040	GNB5	MVD	[activation]
2041	VAPB	ASNA1	[activation]
2042	HPS6	CREB1	[activation]
2043	PPP2R1A	DOCK5	[inhibition]
2044	PRKDC	NOTCH1	[activation]
2045	PRNP	ZNF408	[activation]
2046	FAS	CTNNB1	[inhibition]
2047	NCAM1	MDK	[activation]
2048	MAP1LC3B	STK4	[activation]
2049	STRN4	GDF9	[activation]
2050	YWHAB	BAIAP2	[activation]
2051	APBB1	LRP1	[activation]
2052	PGAM5	ESR1	[activation]

2053	FOSL1	TRIM24	[activation]
2054	PDGFRL	CTSV	[activation;inhibition]
2055	EFHC2	ARHGEF5	[activation]
2056	SNW1	ILK	[activation]
2057	DYRK1A	TROAP	[activation]
2058	RAB1A	CAPNS1	[activation]
2059	CNTFR	PLSCR1	[activation]
2060	BHLHE41	CEBPB	[activation]
2061	SHC3	ALK	[activation]
2062	KAT2A	NOTCH1	[activation]
2063	GRB2	HDGFRP3	[activation]
2064	EIF4E	EIF4G3	[inhibition]
2065	EGFR	PRCC	[activation]
2066	SIM2	HSP90AB1	[activation]
2067	PML	IER3	[activation]
2068	CDK3	E2F3	[activation;inhibition]
2069	CASP8	NR1H4	[inhibition]
2070	MAP4K1	FYN	[activation]
2071	ESR1	HSPA1L	[activation]
2072	CSNK1D	BACE1	[activation]
2073	SERPINB4	RASSF9	[inhibition]
2074	STRN4	PPP2R1A	[inhibition]
2075	MAP3K2	WNK1	[activation]
2076	ERP44	ITPR1	[inhibition]
2077	MMP8	TIMP2	[activation]
2078	EXOC7	EXOC4	[activation]
2079	APP	CDKN2D	[inhibition]
2080	IRS1	YWHAH	[activation]
2081	CTNNB1	EP400	[activation]
2082	ESR2	PRKDC	[activation]
2083	VCAM1	PRDX3	[activation]
2084	HSP90AB1	TRAF2	[activation]
2085	EP300	APEX1	[activation]
2086	RAD21	TNFRSF14	[activation]
2087	NLK	TNKS1BP1	[activation]
2088	FYN	FRK	[activation]
2089	CHRNA7	APP	[activation]
2090	WDR48	STK11	[activation]
2091	RPA3	BUB3	[activation]
2092	CALM1	ARRB2	[activation]
2093	CASK	ARHGEF7	[activation]
2094	RARA	STAT5B	[activation]
2095	ICK	CD247	[activation]
2096	PALB2	HNRNPC	[activation]
2097	CD4	PIK3R1	[activation]
2098	HMGB1	SOX18	[activation]
2099	FRS2	PTPN11	[activation]
2100	GRB2	KIRREL	[activation]
2101	PPP1R2	LMTK2	[activation]
2102	FOXP1	IL3RA	[activation]
2103	RASA1	PTK2B	[activation]
2104	SRPK2	ADAP1	[activation]
2105	ADD1	CASP3	[activation]
2106	SHC1	GEMIN7	[activation]
2107	TENC1	FGFR1	[activation]
2108	KIT	TENC1	[activation]
2109	GNAI1	WASF1	[activation]
2110	INSR	SMAD2	[activation]
2111	TP53	GTF2I	[activation]
2112	KPNB1	NRIP1	[activation]
2113	IKBKB	CSF2RA	[activation]
2114	MYC	MARS	[activation]
2115	TGIF1	MAPK1	[inhibition]
2116	DNAJB1	SOD1	[inhibition]
2117	BMPR2	CCDC89	[activation]
2118	RAC1	VAV3	[activation]
2119	SOCS3	RASA1	[activation]
2120	VAV1	FGFR1	[activation]
2121	CREBBP	RPS6KA2	[activation]
2122	TNIP2	STK11	[activation]
2123	STAT3	BCKDK	[activation]
2124	PRIM1	CIAO1	[activation]
2125	NCAM1	ST8SIA3	[activation]
2126	EIF3J	EIF4G2	[activation]
2127	SDCBP	UBE2A	[inhibition]
2128	VRK2	TP53	[activation]

2129	USP9X	CASP4	[activation]
2130	MAPK3	IER3	[activation]
2131	ITGB3	ITGAV	[activation]
2132	CCND3	MAPK4	[inhibition]
2133	CACNA2D2	USP2	[inhibition]
2134	EPHA2	NUDT9	[activation]
2135	SH3BP1	RHOA	[activation]
2136	AR	CTNNBIP1	[inhibition]
2137	AURKB	AURKA	[activation]
2138	CD4	HNRNPD	[activation]
2139	RAN	NXT1	[activation]
2140	HIPK4	AFG3L2	[activation]
2141	BMP4	WFIKKN2	[activation]
2142	MAPK6	WDR34	[activation;inhibition]
2143	CTRB1	SERPINF2	[activation]
2144	PGK1	PYHIN1	[activation]
2145	TRMT2A	RASA1	[activation]
2146	MAP1LC3A	HSPA2	[activation]
2147	DUSP3	ZAP70	[inhibition]
2148	FOXP3	KAT8	[activation]
2149	EGFR	HIST3H3	[activation]
2150	SGK2	CCT8	[activation]
2151	YY1	ATF2	[activation]
2152	FAM193B	MAPK8	[activation]
2153	NAT10	ITGA4	[activation]
2154	CEP250	CRTC2	[activation]
2155	MAP2K7	TRAF6	[activation]
2156	UBL7	CD4	[activation]
2157	RELA	AR	[activation]
2158	ANXA7	SEMA5B	[activation]
2159	RAD18	MAPK8	[activation]
2160	PRMT1	YWHAG	[activation]
2161	PTPLAD1	GRB2	[activation]
2162	LTA	TUBA1B	[activation]
2163	PRKAA1	NUAK2	[inhibition]
2164	APP	MS4A2	[activation]
2165	F13A1	F13B	[activation]
2166	PMAIP1	BAX	[inhibition]
2167	KAT2B	TRRAP	[activation]
2168	MT-RNR2	BAK1	[activation;inhibition]
2169	DVL3	TNFAIP8L1	[activation]
2170	MAPK8	C11orf57	[activation]
2171	BIRC3	RIPK4	[activation]
2172	HIST1H1A	CCNE1	[activation]
2173	JUNB	BRCA1	[activation]
2174	PTPN12	EGFR	[activation]
2175	APP	NME3	[activation]
2176	CHGB	FGFR3	[activation]
2177	HP1BP3	WHSC1	[activation]
2178	SMURF1	NUMA1	[inhibition]
2179	USP8	RASGRF1	[activation]
2180	PRKACA	VAPA	[activation]
2181	FASLG	RHOA	[activation]
2182	MAPK1	MCL1	[activation]
2183	STK4	ARHGAP1	[activation]
2184	CYP11A1	SMAD3	[activation]
2185	MYF5	MDFI	[activation]
2186	CBL	FCGR1A	[activation]
2187	RIT1	RALGDS	[activation]
2188	UBE2A	PCNA	[inhibition]
2189	CTAGE5	RASAL2	[inhibition]
2190	LATS1	CDK1	[inhibition]
2191	CHUK	CREBBP	[activation]
2192	E2F1	NPDC1	[activation]
2193	RASGRP3	PRKCD	[activation]
2194	RARA	PML	[activation]
2195	SRPK3	KRR1	[activation]
2196	HSP90AA1	MAST2	[activation]
2197	VCAM1	CHMP2A	[activation]
2198	ARHGAP10	RHOA	[activation]
2199	STK11	TUBB4A	[activation]
2200	INHBB	ACVR1C	[activation]
2201	STX17	KRAS	[activation]
2202	PIK3CA	NEDD9	[activation]
2203	PCNA	FTSJ1	[activation]
2204	CYP4Z2P	GABARAPL1	[activation]

2205	KCNA4	KCNAB2	[activation]
2206	WASL	EVL	[activation]
2207	CRYAB	CASP6	[activation]
2208	TLR4	SIGIRR	[activation]
2209	PDCD6IP	UBA5	[activation]
2210	TRAPPC5	EGFR	[activation]
2211	VAMP3	ATP4A	[activation]
2212	G3BP1	VCAM1	[activation]
2213	PLP2	CCR1	[activation]
2214	ABI3	SNAP23	[activation]
2215	FANCE	FAAP20	[activation]
2216	SPRR2A	SRC	[activation]
2217	GSK3B	GRB14	[activation]
2218	HDAC1	LEF1	[inhibition]
2219	NXF1	TNFRSF11A	[activation]
2220	ARHGDIA	TNFRSF1A	[activation]
2221	MAPK10	CDKN1B	[inhibition]
2222	PIK3R1	ERBB3	[activation]
2223	BDNF	CADPS2	[activation]
2224	RPS6KB1	TERT	[activation]
2225	BECN1	DAPK1	[activation]
2226	AURKA	SRPK1	[activation]
2227	SMAD2	RASD2	[activation]
2228	GNB2L1	CD81	[activation]
2229	PFN1	MLLT4	[activation]
2230	RANBP9	OPRM1	[activation]
2231	CREB1	NR3C1	[activation]
2232	NXF1	NTN4	[activation]
2233	UBE2I	SEMA6A	[activation]
2234	NFE2L2	YY1	[activation]
2235	TP53BP1	KPNB1	[activation]
2236	KIAA1683	CALM1	[activation]
2237	CSNK2A2	SPP1	[activation]
2238	CRTC2	NEDD1	[activation]
2239	MAPK9	XPO7	[activation]
2240	APOC1	VKORC1	[inhibition]
2241	HSP90AA1	BTK	[activation]
2242	CASP7	CASP6	[activation]
2243	MYD88	USP7	[activation]
2244	CDK6	SIRT1	[inhibition]
2245	RYR2	RYR1	[activation]
2246	ERBB2	PTK6	[activation]
2247	STAT3	DAPK3	[activation]
2248	SARG	SMAD3	[activation]
2249	HOMER1	ABI3	[activation]
2250	CWC15	PRNP	[activation]
2251	BLK	HSP90B1	[activation]
2252	STK24	CASP8	[activation]
2253	FBXO6	NUP214	[activation]
2254	NXF1	RAD51	[activation]
2255	PARP1	MRE11A	[activation]
2256	SEMA7A	NCK1	[activation]
2257	CSNK2B	RPS6KA1	[activation]
2258	PASK	MAPT	[activation]
2259	MAP2K5	PARD6A	[activation]
2260	YWHAZ	LRRK2	[activation]
2261	ESR1	RAN	[activation]
2262	RNF114	UBE2W	[activation]
2263	HEATR4	ESR2	[activation]
2264	CXorf27	CEP44	[activation;inhibition]
2265	SNAP23	PRKCA	[activation]
2266	FER	ABI1	[activation;inhibition]
2267	DAOA	DAO	[activation]
2268	MAG	APP	[activation]
2269	CHD4	NOTCH1	[activation]
2270	EDA2R	C1QB	[activation]
2271	ICT1	RPUSD3	[activation]
2272	RHEBL1	SMAD1	[activation]
2273	MDN1	NOTCH1	[activation]
2274	SUMO4	DNAJA1	[inhibition]
2275	S1PR2	S1PR1	[activation]
2276	SDCBP	MAD2L1	[inhibition]
2277	FANCA	CRK	[activation]
2278	MBP	SRPK1	[activation]
2279	CPLX1	VAMP2	[activation]
2280	YKT6	BAG3	[activation]

2281	ARF6	HYOU1	[activation]
2282	MAPK3	TRIM54	[activation]
2283	GAS2L3	AURKB	[activation]
2284	MDM2	FASN	[activation]
2285	TRPC3	STX3	[activation]
2286	BEX1	CHEK2	[activation]
2287	EXT2	CNTF	[activation]
2288	SRSF6	MDM2	[activation]
2289	RAP1GDS1	RAC1	[activation]
2290	NTRK3	TNK2	[activation]
2291	STAT6	ANXA2	[activation]
2292	GRB2	SMARCA5	[activation]
2293	RASSF10	ANXA1	[activation]
2294	DDX39B	CHTOP	[activation]
2295	CHEK1	HSP90AA1	[activation]
2296	MYC	BRD3	[activation]
2297	CDKN1A	HSPA5	[inhibition]
2298	HIST1H2BD	ICAM1	[activation]
2299	CASP3	ATN1	[activation]
2300	TNK2	PTPRJ	[activation]
2301	BCL2L11	MAGEA11	[activation]
2302	YWHAQ	CRK	[activation]
2303	SMAD2	SARG	[activation]
2304	MAP1LC3A	NUP214	[activation]
2305	ERH	IGSF21	[activation]
2306	ITGA4	PIK3R2	[activation]
2307	GRB2	GAREM	[activation]
2308	AURKA	EPSTI1	[activation]
2309	PPARA	AIP	[inhibition]
2310	GRIA2	GTF3C2	[activation]
2311	CDC25B	PGR	[activation]
2312	AMOTL1	NXF1	[activation]
2313	TP53	NR0B2	[activation]
2314	CREM	CDK1	[inhibition]
2315	MAPRE1	MAPRE2	[activation]
2316	SMC2	CASP4	[activation]
2317	CRK	PTK2	[activation]
2318	UNC13B	STX1A	[activation]
2319	NSUN4	ICT1	[activation]
2320	YWHAZ	SLC8A2	[activation]
2321	HLA-A	GNAS	[activation]
2322	CUL1	PTTG1	[inhibition]
2323	CNTNAP1	RHOA	[activation]
2324	SPIB	E2F1	[activation]
2325	RPA2	RAD50	[activation]
2326	PTPRJ	CSK	[activation]
2327	VCAM1	DHX30	[activation]
2328	LDLRAD1	PLN	[inhibition]
2329	SUMO1	E2F1	[activation]
2330	CDC42EP4	SRPK1	[activation]
2331	SRSF6	YWHAG	[activation]
2332	YWHAH	SRGAP2	[activation]
2333	ITGA4	HIST1H1B	[activation]
2334	GRB2	SLC25A6	[activation]
2335	RXRA	CNOT1	[inhibition]
2336	MYC	IK	[activation]
2337	PIP5K1A	PLD1	[activation]
2338	MCF2L	AIMP2	[activation]
2339	RAN	MAPK13	[activation]
2340	DYRK1A	FAM117A	[activation]
2341	SCRIB	APC	[activation]
2342	FZD5	RPS6KA6	[activation]
2343	CNTNAP1	KCNA2	[activation]
2344	FBXO5	SRC	[activation]
2345	EIF4EBP1	MVD	[activation]
2346	RAD51D	C1orf94	[activation]
2347	CREBBP	EP300	[activation]
2348	HCK	FASLG	[activation]
2349	TPD52L2	TPD52L1	[activation;inhibition]
2350	HSPA8	CDC20	[inhibition]
2351	ARHGAP5	PTK2B	[activation]
2352	MAPK6	ACTG1	[activation]
2353	RPLP2	GRB2	[activation]
2354	CASP7	RAD51	[activation]
2355	ITGA4	PTPN23	[activation]
2356	NF1	PML	[activation]

2357	BTK	POTEKP	[activation]
2358	PTPN6	G6B	[activation;inhibition]
2359	YAP1	CTNND1	[activation]
2360	SOCS2	TPD52	[inhibition]
2361	EIF4A1	TRAP1	[activation]
2362	SHC1	CORO1C	[activation]
2363	STAT5B	PPP2CA	[activation]
2364	PYHIN1	SDC1	[activation]
2365	HIST1H1C	PRKDC	[activation]
2366	EP300	HNF1A	[activation]
2367	JUP	FYN	[activation]
2368	EPB41L4A	SRPK2	[activation]
2369	RPA1	PRKDC	[activation]
2370	EBLN2	CAB39	[activation]
2371	APP	SIGIRR	[activation]
2372	CRK	CDH11	[activation]
2373	HIST1H2BA	ICAM1	[activation]
2374	HSP90AA1	ACTG1	[activation]
2375	ERBB2	VAV3	[activation]
2376	PRKACA	TNP2	[activation]
2377	RAP1A	HDAC1	[activation]
2378	EEF2	MAPKAPK3	[activation]
2379	MDM2	OGDHL	[activation]
2380	YWHAZ	SLC9A1	[activation]
2381	RAD23B	ETS1	[activation]
2382	E2F1	KAT2A	[activation]
2383	ATG5	P4HB	[activation]
2384	TRAF6	ERH	[activation]
2385	APP	MAPKAPK3	[activation]
2386	ZBTB17	UBE2H	[activation]
2387	DDX52	OBSL1	[activation]
2388	CYFIP1	YWHAG	[inhibition]
2389	NR1I2	MAPK7	[activation]
2390	UBC	RAD51C	[activation]
2391	WBP2	WWTR1	[activation]
2392	PTGS2	TP53	[activation]
2393	TRIP6	PDGFRB	[activation]
2394	RANBP1	FTSJ1	[activation]
2395	PARP1	IKBKB	[activation]
2396	PLCB2	TBXA2R	[inhibition]
2397	MDM2	ETFB	[activation]
2398	LRRK2	TUBB	[activation]
2399	SRC	ROR1	[activation]
2400	PPP2CA	DOCK5	[inhibition]
2401	DOK1	ITGB3	[activation]
2402	MET	SH3BP2	[activation]
2403	EPB41L3	EIF2B2	[activation]
2404	RIF1	LAMA4	[activation]
2405	OBSL1	HMGA2	[activation]
2406	IKZF1	GATA1	[activation]
2407	RIMS4	APP	[activation]
2408	SREBF2	PIAS2	[activation]
2409	CDKN2A	CDKN2AIP	[activation;inhibition]
2410	PIK3CG	CD37	[activation]
2411	ENO1	NUDC	[activation]
2412	MYC	CACNA1G	[activation]
2413	TAF1	UBTF	[activation]
2414	UTP18	VCAM1	[activation]
2415	GSK3B	QARS	[inhibition]
2416	TUBA1A	LTA	[activation]
2417	ZNF106	MYC	[activation]
2418	DIAPH3	RAC1	[activation]
2419	CNKSR1	RHOH	[activation]
2420	FAM107A	LRRK2	[activation]
2421	CAPZB	DAPK1	[activation]
2422	RPA1	FBXO18	[activation]
2423	RNF8	UBE2E3	[activation]
2424	ZIC2	GLI2	[activation]
2425	NR3C1	SFN	[activation]
2426	CASP3	TXN	[activation]
2427	PRKCA	GRM1	[activation]
2428	MAVS	TRAF6	[activation]
2429	SMAD9	KMT2D	[activation]
2430	PML	LRRK2	[activation]
2431	DNAJA2	GRB2	[activation]
2432	HNRNPA0	DDX5	[activation]

2433	PSMG1	LRRK2	[activation]
2434	PPP2R2B	PDPK1	[activation]
2435	NUDC	WRAP73	[activation]
2436	HSPA2	SCHIP1	[activation]
2437	SMAD4	RHOJ	[activation]
2438	MAPK1	EPOR	[activation;inhibition]
2439	RPA3	AHNAK	[activation]
2440	FUT9	TSSK3	[activation]
2441	CASP8	BLID	[inhibition]
2442	ANKRD52	PPP6C	[inhibition]
2443	APP	PTGER2	[activation]
2444	PVRL1	PRNP	[activation]
2445	PAXIP1	TM2D3	[activation]
2446	TP53	PPP1R13B	[activation]
2447	BCL3	TBP	[activation]
2448	VCAM1	BLVRB	[activation]
2449	PER3	PER2	[inhibition]
2450	VDAC1	ICT1	[inhibition]
2451	GNAQ	GRM2	[activation]
2452	OBSL1	BAZ1B	[activation]
2453	DAB1	KRTAP19-7	[activation]
2454	OPALIN	CALM1	[activation]
2455	APP	CCDC115	[activation]
2456	MAFG	PAX6	[activation]
2457	PIAS1	JUN	[inhibition]
2458	AIRE	RANBP2	[activation]
2459	BLK	BCR	[activation]
2460	BEX1	PIK3CA	[activation]
2461	NF1	APP	[activation]
2462	MIA	FASLG	[activation]
2463	SMURF1	SMAD2	[inhibition]
2464	IGF1R	LEF1	[activation]
2465	NCKAP1	MYO16	[inhibition]
2466	HMGA1	RXRA	[inhibition]
2467	SFN	EIF4B	[activation]
2468	BIRC2	ABHD17A	[activation;inhibition]
2469	TOLLIP	CSN1S1	[activation]
2470	NUDC	TIMP2	[activation]
2471	TLN1	CDK5	[activation]
2472	PIK3R2	SHC1	[activation]
2473	DDR1	COL5A2	[activation]
2474	MAPT	EP300	[activation]
2475	NBN	MRE11A	[activation]
2476	ICT1	C6orf203	[activation]
2477	DAZAP2	ZFYVE9	[activation]
2478	RHO	GRK6	[activation;inhibition]
2479	A2M	CPB2	[inhibition]
2480	CTBP1	EGFR	[activation]
2481	EEA1	STX6	[activation]
2482	YWHAZ	DKC1	[activation]
2483	CCNT2	BRD4	[activation]
2484	NSD1	ESRRA	[activation]
2485	TWF2	IQCB1	[activation]
2486	FOS	SIRT1	[activation]
2487	APC	PRKACA	[activation]
2488	DDX56	TBC1D4	[activation]
2489	CNTNAP4	TIAM1	[activation]
2490	F12	MMP12	[activation]
2491	IKBKB	TRIM40	[activation]
2492	MCM7	UTP18	[activation]
2493	SYNGAP1	DLG3	[activation]
2494	IL1B	IKBKG	[activation]
2495	HELLS	LRRK2	[activation]
2496	NCOA2	ESR2	[activation]
2497	CYP11B2	CYP11A1	[activation]
2498	MAPK3	MAP3K14	[activation;inhibition]
2499	RB1	HSPA8	[inhibition]
2500	BCL2L1	MAPK8	[activation]
2501	ETS1	MAPK1	[activation]
2502	BARD1	PPM1G	[activation]
2503	ATP6AP2	USP20	[activation]
2504	POLR2C	FGF3	[activation]
2505	PAM	POMC	[activation]
2506	ACTG1	GZMA	[activation]
2507	F2RL2	PLCG1	[activation]
2508	CXCL1	HRAS	[activation]

2509	HBEGF	MMP7	[activation]
2510	ULBP1	RAET1G	[activation]
2511	RAD9A	MLH1	[activation]
2512	IGKC	CAV1	[activation]
2513	HSPB1	CCDC64	[activation]
2514	RPS6KA5	SMARCA4	[activation]
2515	SMAD2	PREX2	[activation]
2516	GGCX	F7	[activation]
2517	MAPK14	SUPT20H	[activation]
2518	PLG	F2RL1	[activation]
2519	TRAF6	TNFRSF17	[activation]
2520	EIF2AK2	CCNA2	[activation]
2521	ZFYVE28	PRNP	[activation]
2522	ESR2	PREPL	[activation]
2523	MLLT4	YWHAH	[activation]
2524	SLX4	UBA5	[activation]
2525	MAP2K5	ENO1	[activation]
2526	IGHM	PTK2	[activation]
2527	GABARAP	YTHDC2	[activation]
2528	VCL	GSN	[activation]
2529	MAPK8	DUSP22	[inhibition]
2530	MAPKAPK5	CDCA8	[activation]
2531	ANAPC7	TP53BP1	[activation]
2532	ICAM1	EIF3L	[activation]
2533	CAV1	NTRK1	[activation]
2534	CTNNA1	NFKBIA	[activation]
2535	GRB2	MCC	[activation]
2536	ESR1	ACTR2	[activation]
2537	HLA-B	MTPN	[activation]
2538	UNC13B	ECT2	[activation]
2539	THRB	NRIP1	[activation]
2540	HLA-B	TRMT112	[activation]
2541	AKAP8	CASP3	[activation]
2542	MYL12A	MAP3K3	[activation]
2543	ABL2	GPX1	[activation]
2544	KIF17	C16orf70	[activation]
2545	GABRR1	MYC	[activation]
2546	RAF1	FYN	[activation]
2547	HIF1A	DAP3	[activation]
2548	APP	APITD1	[activation]
2549	ZRANB1	APC	[inhibition]
2550	GNB2L1	IFNAR1	[activation]
2551	NPB	NPBWR2	[activation]
2552	TOLLIP	TLR9	[activation]
2553	YWHAB	MTMR12	[activation]
2554	LTB4R2	AKT1	[activation]
2555	PREX2	MTOR	[activation]
2556	TRIM28	ITGA4	[activation]
2557	TAOK3	TRAF2	[activation]
2558	XKRX	MYC	[activation]
2559	CDC5L	UBE2N	[activation]
2560	EGFR	DOCK4	[activation]
2561	MAD2L1	ESR2	[activation]
2562	CDK1	HSPA4	[activation]
2563	PTPRB	PDPK1	[activation]
2564	RPS6KA5	PLA2G4A	[activation]
2565	HSP90AB1	TP63	[activation]
2566	GRB2	KRT17	[activation]
2567	RAD54B	UBE2I	[activation]
2568	RAMP1	IAPP	[activation]
2569	DAG1	CSK	[activation]
2570	TRIM28	CHEK2	[activation]
2571	OR6B3	SRPK1	[activation]
2572	PIGK	ULBP2	[activation]
2573	ATR	PTS	[activation]
2574	NUDCD3	KLHDC8A	[activation]
2575	WAS	PTPN12	[activation]
2576	HSPB1	HSP90AB1	[activation]
2577	PRKDC	FBXO6	[inhibition]
2578	GBP2	ANXA7	[activation]
2579	FYB	PIK3R1	[activation]
2580	FTSJ1	SARS	[activation]
2581	CD3E	TOP2B	[activation]
2582	JAK2	MAP3K5	[activation]
2583	APC	AGR3	[inhibition]
2584	NFATC1	JAK3	[activation]

2585	YWHAG	VCAM1	[activation]
2586	XPA	MLH1	[activation]
2587	WHSC1	HNRNPL	[activation]
2588	SEMA3A	SUMO1	[activation]
2589	TNFRSF1A	CSNK1A1L	[activation]
2590	YWHAE	GRAP2	[activation]
2591	IGF2BP1	CDKN1A	[activation]
2592	MCM7	JUP	[activation]
2593	TRAF5	TRAF2	[activation]
2594	CASP9	MAP2K2	[activation]
2595	SHH	PTCH2	[inhibition]
2596	SMARCB1	SRC	[activation]
2597	NEDD1	IRS4	[activation]
2598	BIK	CRKL	[activation]
2599	RASAL3	YWHAE	[activation]
2600	GRB2	PTPRT	[activation]
2601	PSEN2	CASP6	[activation]
2602	MAP4K3	SH3GL2	[activation]
2603	EP300	KLF1	[activation]
2604	TMCO3	EGFR	[activation]
2605	NXF1	GRN	[activation]
2606	LGR4	ACTB	[activation]
2607	EP300	PAX5	[activation]
2608	GABARAP	TIAL1	[activation]
2609	MAP1B	SPP1	[activation]
2610	MAP3K8	CD40	[activation]
2611	APP	DDX18	[activation]
2612	KPNA3	GATA6	[activation]
2613	GIT1	PLCG1	[activation]
2614	USF1	PPP1CC	[activation]
2615	SMO	BCL6	[inhibition]
2616	HSP90AB1	CAMKK1	[activation]
2617	PRKCB	DVL2	[activation]
2618	DACH1	SIX1	[activation]
2619	SWSAP1	RAD51B	[activation]
2620	ACACA	EGFR	[activation;inhibition]
2621	LIG1	MRE11A	[activation]
2622	CASP4	PLA2G4A	[activation]
2623	PCK1	FASN	[activation]
2624	FOS	CYTH4	[activation]
2625	PAK1	PAK1IP1	[activation]
2626	PTP4A1	CREB3L2	[activation]
2627	MDM2	C19orf10	[activation]
2628	CARM1	TP53	[activation]
2629	TSC1	NF2	[activation;inhibition]
2630	ARHGEF6	SMAD3	[activation]
2631	YWHAZ	OBSL1	[activation]
2632	TCP10	PARD6A	[activation]
2633	NEDD4L	CACNB1	[inhibition]
2634	MAP2K7	HSP90AB1	[activation]
2635	PDCD6	KDR	[activation]
2636	SMAD7	SKP2	[activation]
2637	UBE2D1	CBL	[inhibition]
2638	MPP2	ERBB2IP	[inhibition]
2639	SMARCB1	MYC	[activation]
2640	CD302	BCL2L11	[activation]
2641	WDR61	KMT2A	[activation]
2642	CAT	ABL2	[activation]
2643	YWHAE	EIF4E2	[inhibition]
2644	FAM117B	SFN	[activation]
2645	CDH2	PTPRR	[activation]
2646	BAG3	GNAI3	[activation;inhibition]
2647	NAMPT	IFITM3	[activation]
2648	PAX8	TMA16	[activation]
2649	CSNK2B	NFKBIA	[activation]
2650	STK4	HSPA7	[activation]
2651	EURL	GRB2	[activation]
2652	HSPA2	NOS2	[activation]
2653	NCLN	ARF6	[activation]
2654	ERBB2	C9orf156	[activation;inhibition]
2655	CASP3	HMGB1	[activation]
2656	HABP4	SRSF9	[activation]
2657	SH3RF3	PAK2	[activation]
2658	CAV1	LRP1	[activation]
2659	NPBWR1	NPW	[activation]
2660	MDM4	SMAD3	[inhibition]

2661	KLK6	FGB	[activation]
2662	LY6E	CD247	[activation]
2663	ARNT2	USHBP1	[activation]
2664	DIAPH1	XPO6	[activation]
2665	RPTOR	PIH1D1	[activation;inhibition]
2666	DLG2	FZD7	[activation]
2667	IRAK1	HIST3H3	[activation;inhibition]
2668	PIK3CB	MME	[activation]
2669	PTK2B	NEDD9	[activation]
2670	GRB2	S100A6	[activation]
2671	CASK	IL18	[activation]
2672	CAMKK1	CAMK1	[activation]
2673	MDM2	EP300	[activation]
2674	STAU1	SRPK3	[activation]
2675	HSPD1	EGFR	[activation]
2676	SHANK3	SRC	[activation]
2677	PALB2	MORF4L1	[activation]
2678	RPS6KA5	EGF	[activation]
2679	ZSCAN9	SRPK2	[activation]
2680	DARS	GRB2	[activation]
2681	ANKRA2	MPL	[activation]
2682	TP53	CEBPB	[activation]
2683	HSPB1	EIF1AX	[activation]
2684	CTBP2	EP300	[activation]
2685	TP53	TUBG1	[activation]
2686	E2F3	MRP63	[activation;inhibition]
2687	PTK2	APP	[activation]
2688	NCAPD2	SMC4	[activation]
2689	NGLY1	EGFR	[activation]
2690	MMP9	MMP26	[activation;inhibition]
2691	NCOA3	NKX2-1	[activation]
2692	FYN	MAPT	[activation]
2693	TNFRSF10B	ITCH	[activation]
2694	KIF2A	MYB	[activation]
2695	PCM1	VCAM1	[activation]
2696	NUDT14	RAF1	[activation]
2697	KCTD12	RIPK2	[activation]
2698	CD22	PIK3R1	[activation]
2699	YAP1	RAE1	[activation]
2700	HSP90AB1	CDK15	[activation]
2701	PPARG	PPM1B	[inhibition]
2702	NRP1	FGF7	[activation]
2703	PPP6R2	PLK1	[activation]
2704	PIK3R1	TEC	[activation]
2705	OPRM1	DOK4	[activation]
2706	PAK6	TPD52L1	[activation;inhibition]
2707	H2AFX	A2M	[inhibition]
2708	ITGA4	CD82	[activation]
2709	STAT3	GADD45A	[activation]
2710	CD40	ERP44	[activation]
2711	ANXA2	PAK2	[activation]
2712	CDC25C	YWHAE	[activation]
2713	PALB2	MRC2	[activation]
2714	PFKFB4	GADD45A	[activation]
2715	INSIG2	LGALS7	[activation]
2716	RAD52	HSPA4	[activation]
2717	VAV1	MET	[activation]
2718	PPARG	NR3C1	[activation]
2719	GTF2A2	GTF2A1	[activation]
2720	FOSL1	ATF4	[activation]
2721	EPHA3	ADAM10	[activation]
2722	HNRNPD	MAP2K1	[activation]
2723	RAD21	TRAIP	[activation]
2724	RANBP2	CTNNB1	[activation]
2725	CDC37	ULK1	[inhibition]
2726	MBP	IKBKE	[inhibition]
2727	CTNNB1	HERC5	[inhibition]
2728	HSPB1	CFTR	[activation]
2729	CCHCR1	CTAGE5	[activation]
2730	ATF2	RAB11A	[activation]
2731	YWHAG	MSL2	[activation]
2732	PLK1	FBXO6	[activation]
2733	ADCY6	CHRNA7	[activation]
2734	MRE11A	CCNE1	[activation]
2735	KCNJ4	GNGT2	[activation]
2736	SHC1	VAV1	[activation]

2737	EGFR	FAR1	[activation]
2738	FLNC	GRB2	[activation]
2739	GLMN	TMEM173	[activation]
2740	MEF2A	GNB2	[activation]
2741	CASQ2	CSNK2A1	[activation]
2742	PDX1	PBX1	[activation]
2743	EIF4A2	EIF4G3	[activation]
2744	RPS6KA5	SMARCB1	[activation]
2745	NUDT5	FTSJ1	[activation]
2746	RICTOR	TELO2	[activation]
2747	EGLN3	FOXJ2	[activation]
2748	NOS3	APP	[activation]
2749	EBI3	MDFI	[activation]
2750	PRNP	DYRK3	[activation]
2751	NCKIPSD	CRK	[activation]
2752	PTPMT1	TAB1	[inhibition]
2753	NCOA1	NCOA2	[activation]
2754	BCR	YWHAZ	[activation]
2755	VCL	LPXN	[activation]
2756	PHKG2	GCN1L1	[activation]
2757	STAT2	CREBBP	[activation]
2758	RAPGEF2	MOB4	[activation]
2759	TRAF2	CASP1	[activation]
2760	BCL7C	EPAS1	[activation]
2761	ATM	XPA	[activation]
2762	SHB	KIT	[activation]
2763	HSPB1	ASB10	[activation]
2764	TSPAN6	DDX58	[activation]
2765	ABL2	JAK1	[activation]
2766	PPP2R5B	CHEK2	[activation]
2767	PTPN1	AGR3	[activation]
2768	YWHAB	HSPB1	[activation]
2769	GNB5	E2F2	[activation;inhibition]
2770	VCAM1	RAC2	[activation]
2771	EIF2B1	ARHGDIA	[activation]
2772	MDM2	RAB8A	[activation]
2773	CCR1	CCL3L1	[activation]
2774	TUBB	RIPK1	[activation]
2775	ABL2	PIK3R1	[activation]
2776	TRAF6	CSNK1G1	[activation]
2777	STAT3	KPNA1	[activation]
2778	PLCG2	ZNF512B	[activation]
2779	TCOF1	CDK2	[activation]
2780	FANCG	FAAP100	[activation]
2781	SMAD3	PARD3	[activation]
2782	NOP2	EIF2AK2	[activation]
2783	RPS24	STK17B	[activation]
2784	MYC	ZZEF1	[activation]
2785	GAB1	FES	[activation]
2786	STAT3	FGFR3	[activation]
2787	PIAS1	SNIP1	[inhibition]
2788	AATK	CDK5R1	[activation]
2789	ITGB1	ITGA6	[activation]
2790	RPL11	IGF1R	[activation]
2791	DERL1	PTGS2	[activation]
2792	PPP2CA	DVL3	[activation]
2793	VAPA	ERBB2	[activation]
2794	TRAK2	KCNJ2	[activation]
2795	PRKAA2	KIFC3	[activation]
2796	AGTRAP	HARS2	[activation]
2797	DDX39B	ITGA4	[activation]
2798	ADCYAP1	VIPR2	[activation]
2799	ITGA5	EGFR	[activation]
2800	MALT1	DIABLO	[activation]
2801	CTDP1	E2F1	[activation]
2802	PRKACA	PRKACB	[activation]
2803	VDR	TRIM24	[activation]
2804	TOP2B	TP53	[activation]
2805	ASH1L	SMAD7	[activation]
2806	ABL1	NKX2-1	[activation]
2807	HSPA4	PRKAA1	[inhibition]
2808	TLX3	SNTA1	[activation]
2809	FSHB	CGB	[activation]
2810	PPP2R2A	CHEK2	[activation]
2811	FZR1	VHL	[inhibition]
2812	EXOC5	CCDC102B	[inhibition]

2813	REL	CTNNBIP1	[inhibition]
2814	LSP1	KLK6	[activation]
2815	SRSF5	SMAD2	[activation]
2816	ACTB	CPNE4	[activation]
2817	RGS17	LCE4A	[activation;inhibition]
2818	SLX4	NUP153	[activation]
2819	ERBB3	ABL2	[activation]
2820	NR5A1	CRK	[activation]
2821	KLKB1	HGF	[activation]
2822	LEF1	VDR	[activation]
2823	CRKL	PPFIBP2	[activation]
2824	LARP1	SRPK1	[activation]
2825	EGFR	ATP1B1	[activation]
2826	ITGA4	CFL1	[activation]
2827	RB1	PAX6	[activation]
2828	ERBB2	BTC	[activation]
2829	SMARCA4	CHD7	[activation]
2830	KCNQ1	GRB2	[activation]
2831	TGFBR2	IQGAP1	[inhibition]
2832	RPA2	MDC1	[activation]
2833	GNL3	PHLDA3	[activation]
2834	PTPN1	GRB2	[activation]
2835	RARA	MAPK1	[activation]
2836	BCL3	LCK	[activation]
2837	RUNX1	WWTR1	[activation]
2838	EIF2AK2	KCTD17	[activation]
2839	OR10J1	TRAF6	[activation]
2840	EPHA2	TIAM1	[activation]
2841	HNRNPA1	GABARAPL2	[activation]
2842	CUL1	DUSP1	[inhibition]
2843	CAMK2A	CHAT	[activation]
2844	TNFRSF10D	NUDC	[activation]
2845	EGFR	DDX20	[activation]
2846	ACTA1	BTK	[activation]
2847	RPA3	MCM2	[activation]
2848	TRAF2	CASP12	[activation]
2849	JUN	FBXL12	[activation]
2850	SMAP2	DAB1	[activation]
2851	BAALC	CHEK2	[activation]
2852	RAD51C	HELQ	[activation]
2853	GKAP1	SOX18	[activation]
2854	TP53	ZHX1	[activation]
2855	RC3H1	TNF	[activation]
2856	LAPTM5	DISP1	[activation]
2857	CDC5L	ATR	[activation]
2858	CREB3L2	PXN	[activation]
2859	AVPI1	APP	[inhibition]
2860	MAPK9	ARRB2	[activation]
2861	MAP4K2	DEFB1	[activation]
2862	NR1H2	NRIP1	[activation]
2863	DAAM1	DVL2	[activation]
2864	TWF2	CAPZA2	[activation]
2865	OBSL1	NUP62	[activation]
2866	ASB12	PFN1	[activation]
2867	TUBB4A	LIG1	[activation]
2868	SMURF1	RIT1	[inhibition]
2869	RASSF9	DVL1	[activation]
2870	ANKS1A	EPHA8	[activation]
2871	CALB1	CASP3	[activation]
2872	GABARAPL1	HSPB1	[activation]
2873	EPS8L3	ABI1	[activation]
2874	PACSIN1	ANKRD24	[activation;inhibition]
2875	IRX4	RARA	[activation]
2876	USHBP1	C1orf216	[activation]
2877	HSPA1A	TCERG1	[inhibition]
2878	XIAP	E2F1	[activation]
2879	ACTA1	VCL	[activation]
2880	TBC1D1	BARD1	[activation]
2881	EGLN3	MAPK7	[activation]
2882	CNKSR1	CADM4	[activation]
2883	YTHDF1	RPA1	[activation]
2884	CASP7	AR	[activation]
2885	AGAP3	YWHAB	[activation]
2886	KIF23	YWHAZ	[activation]
2887	FASLG	MYO15A	[activation]
2888	HMGA2	PCBP2	[activation]

2889	SFN	SAMD4A	[activation]
2890	BHLHE40	DAB1	[activation]
2891	XRCC4	PRKDC	[activation]
2892	EP300	MAX	[activation]
2893	MAPKAPK5	JUN	[activation]
2894	MYC	PPA2	[activation]
2895	POGLUT1	FBXO6	[inhibition]
2896	PPA1	HSPB1	[activation]
2897	SH2D3A	SFN	[activation]
2898	PTGIR	PRKCA	[activation]
2899	OPRD1	GNAI2	[activation]
2900	CHEK2	CYP17A1	[activation]
2901	HIST1H3A	RAC1	[activation]
2902	NME5	DYDC2	[activation]
2903	NFYB	AGTRAP	[activation]
2904	TRAF7	CYLD	[inhibition]
2905	E2F1	CWC27	[activation]
2906	RPA1	TAGLN2	[activation]
2907	DAB1	PLCG1	[activation]
2908	UGT1A3	UGT1A7	[activation]
2909	PDGFB	LRP1	[activation]
2910	RSL24D1	CDC23	[activation]
2911	CDC25A	FZR1	[activation]
2912	PIM1	EPHA3	[activation]
2913	VASP	VCAM1	[activation]
2914	HMGB1	HSPA8	[inhibition]
2915	TP53	TDG	[activation]
2916	IL27RA	GOSR1	[activation]
2917	JAM3	TJP1	[activation]
2918	TTBK2	CRK	[activation]
2919	SET	ETS1	[activation]
2920	SCTR	SCT	[activation]
2921	APC	PPP1R13B	[inhibition]
2922	PLEKHG5	RND3	[activation]
2923	CDK18	TRIM54	[activation]
2924	MAPK14	ARHGAP9	[activation]
2925	RECQL5	DDX42	[activation]
2926	STX1A	CACNA1D	[activation]
2927	EGFR	CEBPB	[activation]
2928	RAD21	CCDC7	[activation]
2929	MIS12	FGA	[activation]
2930	LAMTOR5	SHB	[activation]
2931	REV3L	CHEK2	[activation]
2932	PARD3	PLCB1	[activation]
2933	SMAD3	CHUK	[activation]
2934	PHIP	STMN4	[activation]
2935	YWHAQ	FOXO1	[activation]
2936	AMBRA1	TRAF6	[activation]
2937	TNF	ST13	[activation]
2938	MAGED1	BHLHE40	[activation]
2939	HSPA1L	H2AFX	[activation]
2940	PTPRJ	GRB2	[activation]
2941	DKK3	CDH1	[inhibition]
2942	PRKCI	USP20	[activation]
2943	EGFR	HIST1H4A	[activation]
2944	TSC1	ETS1	[activation]
2945	STK11	CTSB	[activation]
2946	CALM1	PPEF1	[activation]
2947	RAB7A	RPA1	[activation]
2948	LRRK2	ACLY	[activation]
2949	ITGA4	PYCR1	[activation]
2950	ATXN10	ILK	[activation]
2951	PPP2R5A	BCL2	[activation]
2952	CAMK4	HSP90AB1	[activation]
2953	DOCK8	SMAD2	[activation]
2954	HSPD1	NR3C1	[activation]
2955	MDM2	OXCT1	[activation]
2956	DOCK3	SRC	[activation]
2957	MAP4K1	MAP3K11	[activation]
2958	PCNA	TRAF6	[activation]
2959	GNAS	GNGT1	[activation]
2960	TGFBI	COL4A1	[activation]
2961	OBSL1	CCAR2	[inhibition]
2962	SMYD2	AHI1	[activation]
2963	MAX	ZBTB17	[inhibition]
2964	CASP4	SMC3	[activation]

2965	MDM2	MRPS23	[activation;inhibition]
2966	PRKCB	YWHAG	[activation]
2967	ITGA4	YWHAZ	[activation]
2968	AGK	FBXO6	[inhibition]
2969	CDC20	ANAPC15	[inhibition]
2970	STK4	STRIP1	[activation]
2971	WAS	RHOQ	[activation]
2972	WT1	PRKACA	[activation]
2973	PDCL2	YWHAB	[activation]
2974	RPS6KA4	CDC5L	[activation]
2975	EPAS1	SMARCB1	[activation]
2976	SRPK1	CLIC5	[activation]
2977	PRC1	KIF23	[activation]
2978	RHBDL2	EFNB3	[activation]
2979	MDM2	TACO1	[activation]
2980	MYOC	TIMP1	[activation]
2981	PIK3CA	THRSP	[activation]
2982	PRKCB	PTPN11	[activation]
2983	ADAP2	IKBKG	[activation]
2984	TNIK	MAP3K7	[activation]
2985	CALM1	RELA	[activation]
2986	FRS2	PRKCZ	[activation]
2987	PXN	CBL	[activation]
2988	QPCTL	LRRK2	[activation]
2989	CNTN1	NOTCH1	[activation]
2990	NEDD8	ERCC4	[activation]
2991	C5AR1	WAS	[activation]
2992	TGFBR1	PAK1	[activation]
2993	PTGDR	HSP90AA1	[activation]
2994	VTN	PASK	[inhibition]
2995	PSEN1	EFNB2	[activation]
2996	TAF1	MDM2	[activation]
2997	RXRA	NFKB1	[inhibition]
2998	ESR2	EIF2A	[activation]
2999	HSPA5	STMN1	[activation]
3000	GRB2	SPTBN1	[activation]
3001	ENOX2	MAGEA11	[activation]
3002	UNG	RPA2	[activation]
3003	LATS1	PTPN13	[inhibition]
3004	ARRB2	CAMK2D	[activation]
3005	PIK3CA	RASD2	[activation]
3006	RAB32	NXF1	[activation]
3007	JAK1	PDGFRB	[activation]
3008	GNGT1	PLEKHB1	[activation]
3009	PDE4DIP	PRNP	[activation]
3010	HSP90AB1	ANKMY2	[activation]
3011	SRC	AKAP2	[activation]
3012	FAS	TMX1	[activation]
3013	APOA4	NR1D1	[inhibition]
3014	MAP4K4	OBSL1	[activation]
3015	RPTOR	LAMTOR1	[activation]
3016	CHEK2	NR4A1	[activation]
3017	DIAPH3	SIRT1	[activation]
3018	DIP2B	MYC	[activation]
3019	HSPA8	CTNNB1	[inhibition]
3020	WIF1	ICOS	[activation]
3021	ITGB4	SMAD3	[activation]
3022	COL1A1	COCH	[activation]
3023	RELA	CHEK1	[activation]
3024	RPA1	EIF2S1	[activation]
3025	SMAD4	DDX39B	[activation]
3026	MYC	CSF1	[activation]
3027	FYN	EPB41L3	[activation]
3028	MYO9A	MYC	[activation]
3029	CRK	MAPK4	[activation]
3030	IRF3	TICAM2	[activation]
3031	FYN	PRICKLE3	[activation]
3032	H2AFX	PBK	[activation]
3033	MDM2	EIF2AK1	[activation]
3034	HDAC3	NFKBIA	[activation]
3035	PIK3R1	TNFRSF1A	[activation]
3036	HSP90AA1	CDKN1A	[activation]
3037	DARS	VCAM1	[activation]
3038	ACTB	SMAD9	[activation]
3039	IRF4	STAT6	[activation]
3040	PIN1	DDX24	[activation]

3041	CASP7	PTMA	[activation]
3042	PTPN2	INSR	[activation]
3043	ACOT7	CAMK2B	[inhibition]
3044	CDH1	TP53BP2	[activation]
3045	LRRK2	DIAPH1	[activation]
3046	SMAD3	PPP2R1A	[inhibition]
3047	EIF4B	YWHAZ	[activation]
3048	RBPJ	SUFU	[inhibition]
3049	IRS2	PIK3CD	[activation]
3050	ANXA2	CTSB	[activation]
3051	PPP2R1A	NKD1	[inhibition]
3052	ITGA4	SNRPA1	[activation]
3053	ANXA2	PPP2R1A	[activation]
3054	CDK11B	VDR	[activation]
3055	NUMB	ITGB2	[activation]
3056	NOTCH1	GATAD2B	[activation]
3057	ERBB2	BAIAP3	[activation]
3058	ARRB2	MDM2	[activation]
3059	RAF1	CNKSR1	[activation]
3060	IDH3B	MAPK6	[activation]
3061	ERBB2	PAK1	[activation]
3062	RAC1	SH3BP1	[activation]
3063	HCK	KIT	[activation]
3064	GABARAPL2	MTPN	[activation]
3065	KIFC3	CDK18	[activation]
3066	SERTAD1	RHOB	[activation]
3067	STAT5A	CENPJ	[activation]
3068	OPRK1	GABARAP	[activation]
3069	COL3A1	PDGFB	[activation]
3070	GABARAP	DDX24	[activation]
3071	SNCA	SPTBN1	[activation]
3072	BARD1	DYRK1A	[activation]
3073	EPHB2	FOS	[activation]
3074	CRK	DOK4	[activation]
3075	CDK5	DAB1	[activation]
3076	TGFB2	TGFBR1	[activation]
3077	PRKCE	PRKD1	[activation]
3078	SPRR4	APP	[activation]
3079	KLF10	CDK6	[inhibition]
3080	MLH1	WDR61	[activation]
3081	ZNF579	ILK	[activation]
3082	HIST1H2BB	ICAM1	[activation]
3083	HNRNPD	CAPN1	[activation]
3084	NFKBIA	HSPA8	[inhibition]
3085	APP	SDC1	[activation]
3086	TP53	EP300	[activation]
3087	CD19	IFITM1	[activation]
3088	USP7	DNAJA3	[inhibition]
3089	PTK2	PTK2B	[activation]
3090	BRCA1	AKT1	[activation]
3091	CEP170P1	CLHC1	[activation]
3092	PTK2	INSR	[activation]
3093	RAC1	PAK1	[activation]
3094	STAT3	PDGFRA	[activation]
3095	PLCG1	AGAP2	[activation]
3096	EIF5	NRGN	[activation]
3097	VCAM1	HIST1H1E	[activation]
3098	SMAD4	SHC1	[activation]
3099	SIRT1	MECOM	[activation]
3100	SP1	RXRA	[inhibition]
3101	CHEK2	DAPK3	[activation]
3102	MAPT	RPS6KA1	[activation]
3103	PLG	SMAD3	[inhibition]
3104	SH2B2	NTRK1	[activation]
3105	PRNP	APP	[activation]
3106	EGFR	EIF2B5	[activation]
3107	GP6	HCK	[activation]
3108	COL4A1	PDGFB	[activation]
3109	BCL3	EP300	[activation]
3110	MTOR	MAPK8	[activation]
3111	RAF1	RFXANK	[activation]
3112	EPB41L3	NR3C1	[activation]
3113	SETX	MYC	[activation]
3114	ATF3	MDM2	[activation]
3115	CXCL11	LRRK2	[activation]
3116	ETS1	DAXX	[activation]

3117	MAST2	STK36	[activation]
3118	MAPK13	IQGAP1	[activation]
3119	PHACTR2	PLCG1	[activation]
3120	HLA-B	BCAP31	[activation]
3121	PTK2	PPP1CA	[activation]
3122	UBAP2	G3BP2	[activation]
3123	SGTB	SERPINE1	[inhibition]
3124	SRPK3	GAR1	[activation]
3125	CSNK1G1	RUNDC3B	[activation]
3126	HABP4	PRKCB	[activation]
3127	BARD1	MT2A	[activation]
3128	KAT2B	TACC3	[activation]
3129	MAP2K1	BTRC	[activation]
3130	WNK1	GLIS2	[activation]
3131	FYN	FAM110A	[activation]
3132	JAK3	LEPR	[activation]
3133	MS4A1	CD40	[activation]
3134	C11orf68	KCNE3	[activation]
3135	MAP3K14	ACTG1	[activation]
3136	SCG3	DNM1L	[activation]
3137	STX1A	SNAP25	[activation]
3138	GLI1	ITCH	[inhibition]
3139	ACTA1	PTPN1	[activation]
3140	CD81	KIT	[activation]
3141	PRTN3	ITGB2	[activation]
3142	CREM	TBP	[inhibition]
3143	NCAPD2	SIRT6	[activation]
3144	HSP90AA1	IRF2	[activation]
3145	RAD21	TMSB4X	[activation]
3146	EGFR	CMTM8	[activation]
3147	NCOA3	SMAD2	[activation]
3148	CBL	IRF1	[activation]
3149	ERP44	ERO1L	[activation]
3150	ICMT	RHOA	[activation]
3151	GNAQ	PIK3R1	[activation]
3152	EIF1	MAPK6	[activation]
3153	IQGAP3	LRRK2	[activation]
3154	CBL	VCAM1	[activation]
3155	NCKAP5	VAV2	[activation]
3156	RIPK1	HSP90AB1	[activation]
3157	TGFB1	TGFB2	[activation]
3158	AK2	TRAF6	[activation]
3159	CALM1	CAMKK1	[activation]
3160	HSPB1	RIF1	[inhibition]
3161	YWHAB	RIOK1	[activation]
3162	ZBTB18	MYC	[activation]
3163	IRF1	HIST1H3A	[activation]
3164	EIF4A3	PRMT5	[activation]
3165	TNFRSF14	DRAP1	[activation]
3166	GRIA2	GRIK2	[activation]
3167	DNAJB6	GH1	[inhibition]
3168	RPL35A	RAD21	[activation]
3169	FASTKD3	AGTRAP	[activation]
3170	DOK5	NTRK3	[activation]
3171	CD4	TFRC	[activation]
3172	MAPK1	LRPAP1	[inhibition]
3173	UBC	BCL2L1	[activation]
3174	PIK3C2B	PDGFRB	[activation]
3175	NOTCH1	XIAP	[activation]
3176	PYHIN1	ZNF428	[activation]
3177	F10	F5	[activation]
3178	CLTC	WNK1	[activation]
3179	ATG5	PAK1	[activation]
3180	FOS	RPS6KA1	[activation]
3181	SEC31B	MYC	[activation]
3182	CASP3	STAT1	[activation]
3183	DMPK	RAC1	[activation]
3184	SRSF10	OBSL1	[activation]
3185	HSPA9	RIPK1	[activation]
3186	SKIL	SASH1	[activation]
3187	GATA1	MAPK6	[activation]
3188	DYRK1A	LIN52	[activation]
3189	ME2	WNK1	[activation]
3190	YWHAQ	APC	[inhibition]
3191	STX1A	RIMS1	[activation]
3192	APPL1	FSHR	[activation]

3193	PHLDA3	IDH3B	[activation]
3194	TRIB3	PRMT5	[activation]
3195	MAGEA9	EGLN1	[activation]
3196	ABL1	RAD52	[activation]
3197	NOD1	CDC37	[activation]
3198	SRPK1	ESRRG	[activation]
3199	RAB4A	ACTB	[activation]
3200	LURAP1	DOCK10	[activation]
3201	TTI1	ATM	[activation]
3202	NFKB2	ACTG1	[activation]
3203	MYC	DNM2	[activation]
3204	APP	NDEL1	[activation]
3205	TYK2	FYN	[activation]
3206	LIN54	EIF4H	[activation]
3207	NOVA1	SRPK1	[activation]
3208	KAT2B	SMAD9	[activation]
3209	CD247	RLTPR	[activation]
3210	CTBP1	MYC	[activation]
3211	MMP1	TFPI	[activation]
3212	CR2	IFITM1	[activation]
3213	BRD7	LAMA4	[activation]
3214	NUP62	SNAPC5	[activation]
3215	RLN3	RXFP1	[activation]
3216	FYN	TRPV4	[activation]
3217	CARD8	CASP9	[activation;inhibition]
3218	GADD45G	CDKN1A	[activation]
3219	EPS15	TFRC	[activation]
3220	HOPX	SRF	[activation]
3221	PDX1	ATF3	[activation]
3222	GRB2	ITGA4	[activation]
3223	PLN	TMEM79	[inhibition]
3224	EFCAB4B	CDC23	[activation]
3225	FTSJ1	HPRT1	[activation]
3226	EGFR	SNCA	[activation]
3227	EGR1	LEF1	[activation]
3228	YWHAB	RADIL	[activation]
3229	ACTL6A	TP53	[activation]
3230	APP	SYK	[activation]
3231	MAPKAPK2	NXF1	[activation]
3232	POLK	POLD1	[activation]
3233	GZMB	GRIA3	[activation]
3234	ESR1	ARHGDIA	[activation]
3235	CDH1	EZR	[activation]
3236	ATG4B	STK3	[activation]
3237	F2RL2	JAM2	[activation]
3238	TERT	MTOR	[activation]
3239	PRKG1	PPP1R12A	[inhibition]
3240	FANCA	FANCE	[activation]
3241	ZNF24	RICTOR	[activation]
3242	CEBPA	CDK2	[activation;inhibition]
3243	C11orf49	CDC16	[activation]
3244	RPL24	TP53	[activation]
3245	SRC	ESRRA	[activation]
3246	STC2	EIF2B3	[activation]
3247	PTEN	BEX1	[activation]
3248	ANAPC7	SMARCAD1	[activation]
3249	EIF2AK2	FARSA	[activation]
3250	CAB39	BCKDK	[activation]
3251	ESR1	DDX27	[activation]
3252	ESR2	SMARCA5	[activation]
3253	EXT1	TRAP1	[activation]
3254	BARX1	MAPK6	[activation]
3255	XIAP	CASP10	[activation]
3256	MYOD1	SETD7	[activation]
3257	NUP107	NUP214	[activation]
3258	CLOCK	RARA	[activation]
3259	SRPK2	NSMCE4A	[activation]
3260	STAT5A	STAT3	[activation]
3261	PYCARD	TSHZ2	[inhibition]
3262	CDKN1C	FBXL12	[inhibition]
3263	STAT3	GADD45GIP1	[activation]
3264	CTDSP1	POLR2A	[activation]
3265	MAP3K1	MAPK1	[activation]
3266	ABCA1	DLG5	[activation]
3267	TP53	ZMIZ1	[activation]
3268	SRPK2	NXT2	[activation]

TRAF6	TRAF1	[activation]
CIITA	SIRT1	[activation]
PAXIP1	PPARG	[activation]
H2AFZ	CTCF	[activation]
MAVS	ITCH	[activation]
NOTCH2NL	GIP	[activation]
MYLK	SRC	[activation]
DNAJB2	HDAC11	[inhibition]
ULK1	TBC1D14	[inhibition]
CTNNA2	YAP1	[activation]
GNA12	CDH15	[activation]
SRPK1	ZNF514	[activation]
EIF4A3	DDX17	[activation]
SH3RF3	CDC42	[activation]
STK40	LRRK2	[activation]
COPS6	STX5	[activation]
NUDC	KLHDC8A	[activation]
DDX5	AURKB	[activation]
ATP2B1	PRKCA	[activation]
NFKB1	STAT5A	[activation]
NPHP3	CEP164	[activation]
KPNA2	SGK1	[activation]
AKT1	PEBP4	[activation]
TRAF6	PELI2	[activation]
CD28	VAV1	[activation]
FCER1G	PLCG2	[activation]
METTL21C	HSPA8	[inhibition]
MRPS27	AURKB	[activation]
TP53	COPS6	[activation]
E2F1	MOV10	[activation]
ADIPOQ	PDGFB	[activation]
GNL3	CDKN2A	[activation;inhibition]
DDX21	TRAF6	[activation]
IRAK2	YTHDF1	[activation]
BCL6	LPAR4	[activation]
GNB2L1	PRKCA	[activation]
SUV39H1	NR1H2	[activation]
MAPK9	CDC25B	[activation]
FADD	RALGDS	[activation]
UFM1	UFC1	[activation]
TNFRSF10B	CBL	[activation]
YWHAZ	NOS2	[activation]
RAC1	IQGAP2	[activation]
ATF2	FOSL1	[activation]
TTC5	CAMK2B	[activation]
GBF1	KBTBD7	[inhibition]
RASA1	PDGFRB	[activation]
FYN	GPR45	[activation]
OXSR1	BMPR1B	[activation;inhibition]
GCA	LCP1	[activation]
HIF1AN	ILK	[activation]
WDR37	SFN	[activation]
PDLIM7	GEM	[activation]
RPA2	APC	[activation]
FOSL1	HDAC1	[activation]
ACTG1	EPS8L3	[activation]
RPS14	TP53	[activation]
STRA13	TRIM54	[activation]
XIAP	RIPK4	[activation]
ACTG1	FBXO6	[activation]
FANCA	MX1	[activation]
PLCG1	IBTK	[activation]
GRM5	PRKCG	[activation]
MAGI2	ACVR2A	[activation]
MLH1	BLM	[activation]
XRCC3	PALB2	[activation]
ROR1	WNT5A	[activation]
SMYD2	PTGES3	[activation]
ARRB2	GCG	[activation]
ITGB3	ITGA5	[activation]
IL7R	KIT	[activation]
SMURF1	STK35	[inhibition]
ACTG1	LGR4	[activation]
PTTG1	BUB1B	[inhibition]
WAS	APBB1	[activation]
YAP1	ABL1	[activation]

3345	PPP1CA	MYC	[activation]
3346	FGF3	FGFR4	[activation]
3347	RHOB	FBN1	[activation]
3348	AKR1B10	ACACA	[activation;inhibition]
3349	RANBP9	LTB4R2	[activation]
3350	SIKE1	PPP2CA	[activation]
3351	TIMELESS	RPA3	[activation]
3352	SKI	TP53	[activation]
3353	HSP90AB1	SHC1	[activation]
3354	SMARCA4	HSF1	[activation]
3355	ENO1	SERPING1	[inhibition]
3356	HDAC3	NFE2L2	[activation]
3357	TSEN54	NXF1	[activation]
3358	NR3C1	YWHAH	[activation]
3359	PDIA4	TRAF6	[activation]
3360	SLC9A3R1	GNA11	[activation]
3361	ADAMTSL5	NOTCH2NL	[activation]
3362	RNPC3	PRNP	[activation]
3363	SRC	EFNB2	[activation]
3364	MAPK7	ETS1	[activation]
3365	RASL12	SMAD1	[activation]
3366	CHUK	NOTCH3	[activation]
3367	PKN3	PLD1	[activation]
3368	ANAPC7	FBXO5	[activation]
3369	GSDMB	PEBP1	[inhibition]
3370	ANAPC11	TRIM65	[activation]
3371	ABL1	XPO1	[activation]
3372	TFDP1	LIN37	[inhibition]
3373	TAB1	DNAJC7	[inhibition]
3374	HNRNPA1	ESR1	[activation]
3375	CCNG2	SKP2	[inhibition]
3376	CDK6	CDC37	[inhibition]
3377	GNAQ	CYTH3	[activation]
3378	DDX5	LRRK2	[activation]
3379	YWHAQ	PRKCQ	[activation]
3380	KIR2DS2	HLA-C	[activation]
3381	SMAD4	DVL1	[activation]
3382	NCOR2	PPARA	[activation]
3383	ADRB2	SLC9A3R1	[activation]
3384	MYO5A	MYC	[activation]
3385	YWHAZ	WEE1	[activation]
3386	CDH3	IGF1R	[activation]
3387	EGFR	PRKAR1B	[activation]
3388	SMURF1	XPO1	[activation]
3389	GNAZ	EYA2	[activation]
3390	PBX1	CREBBP	[activation]
3391	UBE2I	ESR1	[activation]
3392	MAPK9	PIK3R1	[activation]
3393	HAPLN1	MMP10	[activation]
3394	HSPB1	PHYHIPL	[activation]
3395	DMWD	PHLPP1	[inhibition]
3396	RPS17	FOS	[activation]
3397	A1BG	ANXA7	[activation]
3398	FCHSD2	WAS	[activation]
3399	MAPK3	PLCB1	[activation]
3400	VCAM1	IGF2BP2	[activation]
3401	WDR5	PRNP	[activation]
3402	TNS3	BCAR1	[activation]
3403	CAPZB	FAM21C	[activation]
3404	CDK1	SOX2	[activation]
3405	AGFG1	VAMP7	[activation]
3406	IKBKB	SRC	[activation]
3407	PTK2	SOCS3	[activation;inhibition]
3408	HSP90AB1	KLHL38	[activation]
3409	HSPA4	BCL2L11	[activation]
3410	HNF4A	TRIM24	[activation]
3411	ENO2	ASB9	[activation]
3412	BCL2	LRRK2	[activation]
3413	URM1	NUP160	[activation]
3414	SENP3	CDK4	[inhibition]
3415	TP63	EP300	[activation]
3416	SNAI2	APP	[inhibition]
3417	MMP14	FGFR2	[activation]
3418	MAP2K5	HSP90AA1	[activation]
3419	FADS2	EGFR	[activation]
3420	CRKL	CBL	[activation]

3421	C4A	APC	[inhibition]
3422	SRPK1	C9orf72	[activation]
3423	RAD21	PIGR	[activation]
3424	IFRG15	LRRK2	[activation]
3425	PPP2CA	MRPS26	[inhibition]
3426	RAC1	DVL1	[activation]
3427	MAPT	RPS6KA5	[activation]
3428	BCL2L1	BIK	[activation]
3429	PRNP	P4HB	[activation]
3430	STK16	ROCK2	[activation]
3431	ACTA1	MYLK	[activation]
3432	MARS	NFKB2	[activation]
3433	ZFYVE27	RAB11A	[activation]
3434	CCNA2	AKT3	[activation]
3435	TRAF6	ABL1	[activation]
3436	AURKB	TCEAL4	[activation]
3437	WASF1	FYN	[activation]
3438	GADD45G	PPARG	[activation]
3439	GIT1	TGFB1I1	[activation]
3440	GRB2	ABLIM1	[activation]
3441	WRAP73	CDK2	[activation]
3442	C5	C8B	[activation]
3443	ESR1	PRDX2	[activation]
3444	EGFR	PTPN18	[activation]
3445	NRAS	AKT1	[activation]
3446	TRAF1	TRAF2	[activation]
3447	E2F3	CCDC9	[activation;inhibition]
3448	HSP90AA1	CDH1	[activation]
3449	KPNA2	VCAM1	[activation]
3450	APITD1	TOP3A	[activation]
3451	TTBK1	TTBK2	[activation]
3452	ATM	SOCS1	[inhibition]
3453	NUP62	TXLNA	[activation]
3454	SMAD2	MCF2L	[activation]
3455	PRKCD	IL6ST	[activation]
3456	ACTB	CSNK1A1	[activation]
3457	PIK3R6	RAPGEF3	[activation]
3458	EGFR	MCM4	[activation]
3459	GTF2A1	GCN1L1	[activation]
3460	CDK1	GADD45G	[activation]
3461	DVL1	SMAD7	[activation]
3462	PSEN2	LIPF	[activation]
3463	GAB2	CRKL	[activation]
3464	HES1	FANCF	[activation]
3465	TFRC	TP53	[activation]
3466	MAD2L2	REV3L	[activation]
3467	YWHAZ	RAD52	[activation]
3468	PTPN2	STAT3	[activation]
3469	HSPG2	LAMA1	[activation]
3470	GNB2L1	EPOR	[activation]
3471	TNFSF13	XPO1	[activation]
3472	NCOA3	DDX5	[activation]
3473	INS	HLA-DQA2	[activation]
3474	DMPK	FXYD1	[activation]
3475	BARD1	PTN	[activation]
3476	TRAF1	CASP8	[activation]
3477	CDC42	CNTNAP1	[activation]
3478	LAMA1	SAA1	[activation]
3479	C4BPA	BMPR2	[activation]
3480	TBK1	TRAF2	[activation]
3481	CREBBP	NKX2-1	[activation]
3482	EZR	EGFR	[activation]
3483	CDC25B	BRSK2	[activation]
3484	PAK3	PLK1	[activation]
3485	SNURF	TP53	[activation]
3486	IKZF3	RHOA	[activation]
3487	LCK	CSF3R	[activation]
3488	ARF1	MDM4	[inhibition]
3489	DDX39B	SREK1	[activation]
3490	RB1	PML	[inhibition]
3491	KLKB1	MST1	[activation]
3492	NPHP3	NPHP1	[inhibition]
3493	NCK1	LCP2	[activation]
3494	MORN4	HMMR	[activation]
3495	AMOTL2	RAD51D	[activation]
3496	RGS2	DDR1	[activation]

3497 RBX1 MAP4K1 [inhibition]
3498 SMAD2 MTMR4 [activation]
3499 HDAC2 CCND1 [inhibition]
3500 FRS2 GRB2 [activation]
3501 ARHGDIB EZR [activation]
3502 TRAF6 LGMN [activation]
3503 AXIN2 AMMECR1 [inhibition]
3504 FRAT1 GRN [activation]
3505 BEND5 GRAP2 [activation]
3506 LCE4A OTX1 [activation]
3507 BCL2L11 MCL1 [activation]
3508 GNAI2 PLSCR1 [activation]
3509 PLCG1 DNM1 [activation]
3510 AIM2 NBN [activation]
3511 SPRR2A CRK [activation]
3512 ELK1 CEBPB [activation]
3513 ICAM5 ITGAL [activation]
3514 GNB2 TRAF6 [activation]
3515 HIST1H2BL MYC [activation]
3516 SRC PDIA2 [activation]
3517 NFKBIA TIMM50 [activation]
3518 ILK IRS4 [activation]
3519 HSP90AA1 EIF2AK2 [activation]
3520 SERPIND1 CTSG [inhibition]
3521 PTPN6 PRKCD [activation;inhibition]
3522 TIPRL PPP6C [inhibition]
3523 RAD21 SMARCA5 [activation]
3524 LARS RPTOR [activation]
3525 PTK2 LCK [activation]
3526 C16orf70 APP [activation]
3527 SAV1 GLMN [activation]
3528 ZFPM2 EP300 [activation]
3529 RAC1 NCF1 [activation]
3530 SSTR1 CORT [activation]
3531 PRPF6 NOTCH1 [activation]
3532 RELB ACTG1 [activation]
3533 INSR IGF1R [activation]
3534 PTGES3 ERBB3 [activation]
3535 SERPINB8 F10 [inhibition]
3536 COL4A1 MMP9 [activation]
3537 SCARB1 CAND1 [activation]
3538 FGF2 HSPG2 [activation]
3539 UBE2C ENO2 [activation]
3540 SNRNP70 IL32 [activation]
3541 GRB2 IGF2BP1 [activation]
3542 CDKL5 PRKG2 [activation]
3543 CCNG1 PPP2R1A [activation]
3544 CNKSR1 STK3 [activation]
3545 BCL7C SMARCA4 [activation]
3546 ACTB CDC5L [activation]
3547 NFE2L2 PRKCH [activation]
3548 CLIP1 RAC1 [activation]
3549 ERC2 MYB [activation]
3550 CCDC93 EXOC1 [activation]
3551 NFE2L2 PPARG [activation]
3552 HMGN2 GRB2 [activation]
3553 CPE GCG [activation]
3554 UBE2H RNF166 [activation]
3555 SRSF10 CSNK2A1 [activation]
3556 EIF4EBP3 RPA2 [activation]
3557 PALB2 CASZ1 [activation]
3558 CCNG1 PPP2CA [activation]
3559 ARRB2 KPNB1 [activation]
3560 PPP6C MOB1A [activation]
3561 FYN YTHDC1 [activation]
3562 MAPKAP1 SFN [activation]
3563 NR3C1 MDM2 [activation]
3564 OPRM1 WLS [activation]
3565 RECQL5 TOP3B [activation]
3566 PACSIN1 APBB1IP [activation]
3567 MYO1B MAP1LC3A [activation]
3568 TJP1 MLLT4 [activation]
3569 DOCK1 FYN [activation]
3570 GNG3 GNAI3 [activation;inhibition]
3571 SGK1 HYOU1 [activation]
3572 ADAM17 FHL2 [activation]

3573	IER3	TNFSF10	[activation]
3574	SSH1	YWHAB	[activation]
3575	MOB3B	MAPK14	[activation]
3576	SUCLG1	MYC	[activation]
3577	CAPN1	CTSC	[activation]
3578	BMPR2	LIMK1	[activation]
3579	CORT	MRGPRX2	[activation]
3580	PRKDC	GZMB	[activation]
3581	PLK1	ITSN1	[activation]
3582	INSR	AHSG	[inhibition]
3583	PDCD6	GRB2	[activation]
3584	MIF	HLA-B	[activation]
3585	POLD4	POLD3	[activation]
3586	CASP3	USO1	[activation]
3587	CNTN2	CNTNAP2	[activation]
3588	HIF1A	BCR	[activation]
3589	PITPNM3	CCL18	[activation]
3590	MAP2K2	IQGAP1	[activation]
3591	KRT15	GRB2	[activation]
3592	ITGB1	ICAM4	[activation]
3593	CADPS	DRD2	[activation]
3594	RASSF5	STK4	[activation]
3595	ARF6	YARS	[activation]
3596	NR3C1	EGFR	[activation]
3597	S1PR2	ITGB4	[activation]
3598	DIXDC1	DVL2	[activation]
3599	RAC2	TNFAIP8L2	[activation]
3600	IGSF8	HIST2H3A	[activation]
3601	MYB	STAT5A	[activation]
3602	APC	JUP	[inhibition]
3603	AR	CDC25A	[activation]
3604	BUB1	EIF4EBP1	[activation]
3605	BMP2	ACTR2	[activation]
3606	SLAIN2	GRB2	[activation]
3607	NFKBIE	PRKDC	[activation]
3608	SNCA	COL25A1	[activation]
3609	EED	ANXA5	[activation]
3610	RIMS1	SMAD2	[activation]
3611	DAAM1	ARHGEF11	[activation]
3612	APBB1	EGFR	[activation]
3613	SMAD4	DNAJB2	[inhibition]
3614	ANAPC7	CCDC8	[activation]
3615	SMARCB1	BAZ1B	[activation]
3616	CCDC102B	MAB21L2	[activation]
3617	PPP2R1A	PRR14	[inhibition]
3618	MZT2A	CDK5RAP2	[activation]
3619	PRPF3	AURKB	[activation]
3620	PPP1R2P3	PPP1CA	[activation;inhibition]
3621	TGFBR1	CHUK	[activation]
3622	TXNDC5	H2AFX	[activation]
3623	TJP2	SRPK1	[activation]
3624	SRC	P2RY2	[activation]
3625	SKIL	CPNE1	[activation]
3626	C1QA	RAC1	[activation]
3627	OBSL1	MGA	[inhibition]
3628	INSR	JAK2	[activation]
3629	IGF1R	LYPD3	[activation]
3630	GRB2	LIME1	[activation]
3631	PDPK1	IRS1	[activation]
3632	BTRC	CDC25A	[activation]
3633	GRB2	PKM	[activation]
3634	RALA	ARF1	[activation]
3635	NXF1	PRUNE	[activation]
3636	FOS	TSPYL4	[activation]
3637	TRMT1L	EGFR	[activation]
3638	NCK2	LCP2	[activation]
3639	CASS4	CRK	[activation]
3640	AGFG1	VCAM1	[activation]
3641	TTC3	ETS1	[activation]
3642	VAV1	ERBB3	[activation]
3643	ABL1	ERBB3	[activation]
3644	GNA14	DNAL4	[activation]
3645	BTG2	SMAD9	[activation]
3646	SMAD2	RXRA	[inhibition]
3647	OBSL1	UTP6	[activation;inhibition]
3648	BRCA1	CLSPN	[activation]

3649	ERBB3	CDK5	[activation]
3650	PTPRG	INSR	[activation]
3651	MAPKAPK2	YWHAZ	[activation]
3652	GCSH	NMI	[activation]
3653	MAPKAPK3	LNX1	[activation]
3654	CACNA1E	GNB2	[inhibition]
3655	NR5A1	DDX39B	[activation]
3656	TAF1B	EP300	[activation]
3657	SMARCA1	GRK5	[activation]
3658	SRPK3	SF3B4	[activation]
3659	LRRK2	HNRNPH3	[activation]
3660	PPARG	EP300	[activation]
3661	SH2D1A	ERBB3	[activation]
3662	PTN	ARL15	[activation]
3663	MAP2K6	LRRK2	[activation]
3664	TRIM29	SHOX2	[activation]
3665	TP53	BCL2L1	[activation]
3666	WFIKKN2	BMP8B	[activation]
3667	SOX2	IQGAP3	[inhibition]
3668	HSPA6	E2F3	[inhibition]
3669	MOS	TRAF1	[activation]
3670	EPHA4	FZR1	[activation]
3671	CASP3	BID	[activation]
3672	PTPRC	PXN	[activation]
3673	PCYT1A	CASP6	[activation]
3674	PDPK1	LUC7L2	[activation]
3675	BOLA1	RGS20	[activation;inhibition]
3676	CXCR2	GNA15	[activation]
3677	SHKBP1	CCNG1	[activation]
3678	PPP2R1B	ANXA5	[activation]
3679	HTRA2	MAPK11	[activation]
3680	PSMB3	INSIG2	[activation]
3681	NR3C1	PTMS	[activation]
3682	ELK1	CDK6	[activation]
3683	EGFR	ANKRD13D	[activation]
3684	INSC	PARD3	[activation]
3685	CCDC33	MAPK9	[activation]
3686	ACTN1	ADORA2A	[activation]
3687	HSPB1	RALA	[activation]
3688	FBXO15	SKP1	[inhibition]
3689	HLA-B	SRC	[activation]
3690	A2M	TYRO3	[inhibition]
3691	STAT4	CREBBP	[activation]
3692	TOLLIP	ANKRD13A	[activation]
3693	PRPF6	RAF1	[activation]
3694	NAT1	LRRK2	[activation]
3695	NUP62	PIN1	[activation]
3696	TRAF6	TMBIM6	[activation]
3697	CUL1	DBNL	[inhibition]
3698	MLNR	MLN	[activation]
3699	PDE6G	MAPK3	[activation;inhibition]
3700	WHSC1L1	AKT1	[activation]
3701	CTTN	PAK1	[activation]
3702	APBB1	H2AFX	[activation]
3703	TNFRSF1A	CLIP3	[activation]
3704	E2F1	NDNL2	[activation]
3705	FANCG	CDK1	[activation]
3706	PTK2B	CRK	[activation]
3707	CBLB	GLRX3	[inhibition]
3708	GADD45A	PTN	[activation]
3709	CDK5R1	HSP90AA1	[activation]
3710	PDGFB	PDGFA	[activation]
3711	COL1A1	PAK1	[activation]
3712	MYC	SLC25A11	[activation]
3713	HTR2A	MRPL28	[activation]
3714	BHMT2	APP	[activation]
3715	CRTC2	HECW2	[activation]
3716	SPP1	MMP3	[activation]
3717	ITGA4	VASP	[activation]
3718	PARD3	STK11	[activation]
3719	RFC2	PAXIP1	[activation]
3720	GIMAP5	BCL2	[activation]
3721	CDH1	AJAP1	[activation]
3722	LRRK2	RIF1	[activation]
3723	YWHAB	CHEK1	[activation]
3724	STRBP	LRRK2	[activation]

3725	NCAM1	FYN	[activation]
3726	ESRRA	MACF1	[activation]
3727	STAT3	STAT6	[activation]
3728	SHC1	HSP90AA1	[activation]
3729	ARAF	PDGFRB	[activation]
3730	RPS6	ITGA4	[activation]
3731	FASN	CDC5L	[activation]
3732	DOK4	TEK	[activation]
3733	SMAD1	ICK	[activation]
3734	SDC4	TGFB3	[activation]
3735	CDKN1B	YWHAH	[activation;inhibition]
3736	BIRC2	UBE2W	[activation]
3737	EP300	TP73	[activation]
3738	RIPK1	NOD2	[activation]
3739	SAE1	BCL6	[activation]
3740	ANAPC2	FZR1	[activation]
3741	NKX2-1	PAX8	[activation]
3742	CDC37	BRAF	[activation]
3743	ST7	ZFYVE9	[activation]
3744	U2AF2	SRPK2	[activation]
3745	HSPB1	FAM71D	[activation]
3746	SP3	TP53	[activation]
3747	VCAM1	ZYX	[activation]
3748	NLRC4	CASP8	[activation]
3749	RNH1	ANG	[activation]
3750	LUC7L2	APP	[activation]
3751	CISH	SMAD2	[inhibition]
3752	ODF1	CDK5	[activation]
3753	LRRK2	NUP153	[activation]
3754	PIK3R1	CDC27	[activation]
3755	MBP	PRKCZ	[activation]
3756	SBDS	MAGEA6	[activation]
3757	ARMC2	IGHG1	[activation]
3758	KIAA1377	PIK3R3	[activation]
3759	RNF2	EIF2S2	[activation]
3760	TNFRSF1A	SRC	[activation]
3761	ENAH	ABI2	[activation]
3762	LATS1	MOB3B	[inhibition]
3763	CTSV	APC	[inhibition]
3764	HRAS	IL24	[activation]
3765	LIMS1	CTGF	[activation]
3766	SLAMF1	FGR	[activation]
3767	NR1H3	LZTR1	[activation]
3768	PHF23	STK11	[activation]
3769	IGLL1	MME	[activation]
3770	YWHAB	SRC	[activation]
3771	KIR2DL1	PIK3R1	[activation]
3772	RPA1	ERCC6	[activation]
3773	XPO7	ILK	[activation]
3774	NOS1	CAV3	[activation]
3775	ESR1	FOXO3	[activation]
3776	RARS	HLA-B	[activation]
3777	LRRK2	WDR5	[activation]
3778	EGFR	TRAPPC3	[activation]
3779	HNRNPM	RELA	[activation]
3780	RHOG	VAV1	[activation]
3781	CDK11B	YWHAE	[activation]
3782	STK11	RPAP3	[activation]
3783	PIK3C3	PRMT5	[activation]
3784	TSPAN7	RBL1	[inhibition]
3785	NXF1	PLCG1	[activation]
3786	WASL	PACSIN1	[activation]
3787	TP53TG3	LRRK2	[activation]
3788	AANAT	F2	[activation;inhibition]
3789	IGF2BP1	ARHGDIA	[activation]
3790	SMAD1	PAX6	[activation]
3791	PAK1	PXN	[activation]
3792	CDC27	PAXIP1	[activation]
3793	LYN	RPS6KB1	[activation]
3794	EIF4G1	TNRC6C	[activation]
3795	EP300	MAML1	[activation]
3796	MOS	CCDC33	[activation]
3797	NSD1	HIST1H1C	[activation]
3798	SMARCA4	SMAD2	[activation]
3799	BCL6	ZNF443	[activation]
3800	CIAO1	FMNL2	[activation]

3801 CSNK1A1 PSEN2 [activation]
3802 NXF1 ANAPC13 [activation]
3803 ELAVL1 CHEK2 [activation]
3804 ENO2 NAT9 [activation]
3805 OLA1 SGK1 [activation]
3806 LCE3C NOTCH2NL [activation]
3807 CDK6 ISL1 [activation]
3808 MYO1D CHUK [activation]
3809 RAPGEF6 NXF1 [activation]
3810 NXF1 SH3KBP1 [activation]
3811 CHUK STAP2 [activation]
3812 ERBB3 MRPS34 [activation]
3813 INO80 H2AFX [activation]
3814 AR BLNK [activation]
3815 TRAF6 ULK1 [inhibition]
3816 RELA COMMD2 [activation]
3817 RHPN2 TGFBR1 [inhibition]
3818 JUP CTNNB1 [activation]
3819 PHF8 RBPJ [inhibition]
3820 SMC1A ATM [activation]
3821 CLTCL1 PICALM [activation]
3822 MTMR12 MTM1 [activation]
3823 MCM3 STAT1 [activation]
3824 YAP1 KLF5 [activation]
3825 RPA3 SRBD1 [activation]
3826 HELB POLA2 [activation]
3827 C5 CPN1 [activation]
3828 NOV CDKN1A [activation]
3829 F2R ELANE [activation]
3830 SYT9 SNAP25 [activation]
3831 CDC42BPB PYGM [activation]
3832 HSPB1 TARBP2 [activation]
3833 HLA-B TXNDC17 [activation]
3834 NPDC1 CCND1 [activation]
3835 ATP6V1A MYO1B [activation]
3836 PTGES3 EIF2AK2 [activation]
3837 CCND1 NRF1 [activation]
3838 DNTTIP1 RPA3 [activation]
3839 PPP2R1B CAV1 [activation]
3840 ATXN7L2 LAMB1 [activation]
3841 NUDT18 RAD54B [activation]
3842 TNIK IKBKB [activation]
3843 YARS EGFR [activation]
3844 CDC25A APP [activation]
3845 CDK5 GAK [activation]
3846 EGFR ABL2 [activation]
3847 TP53 SMARCD2 [activation]
3848 IL3RA JAK2 [activation]
3849 CDC5L DIAPH1 [activation]
3850 FBXL13 HSP90AB1 [activation]
3851 ARHGDIA MAPK6 [activation]
3852 DLGAP4 NXF1 [activation]
3853 ARNT2 NPAS2 [activation]
3854 SVIL GRB2 [activation]
3855 MDM2 ARHGEF6 [activation]
3856 TRAF6 CARHSP1 [activation]
3857 PLCG1 RHOU [activation]
3858 EIF4EBP1 MAPKAP1 [inhibition]
3859 ADRB2 IDH2 [activation]
3860 YWHAH TSC1 [activation;inhibition]
3861 RB1 HIF1A [activation]
3862 IRS4 MTNR1A [activation]
3863 XPOT NUP214 [activation]
3864 UBE2N RNF165 [activation]
3865 SMURF1 BMPR1B [inhibition]
3866 NRIP1 TULP3 [activation]
3867 CD4 EPS15 [activation]
3868 SLX1A ERCC4 [activation]
3869 CAMK2B PLCB3 [activation]
3870 HDAC1 SP1 [activation]
3871 CDK10 HSP90AB1 [activation]
3872 FBXO6 LTBP3 [inhibition]
3873 SH3KBP1 ANAPC5 [activation]
3874 ZIC3 SRF [activation]
3875 RHOH RHOA [activation]
3876 PPP2CB CDK18 [inhibition]

3877	RNF219	CNOT10	[activation]
3878	RELA	CDKN2A	[activation]
3879	EGFR	ERBB2	[activation]
3880	DVL1	CSNK1D	[activation]
3881	CACNB1	GEM	[activation]
3882	PLAC9	CCDC14	[activation]
3883	DDAH2	ANXA7	[activation]
3884	HIST1H2BC	CD81	[activation]
3885	FYN	SEPN1	[activation]
3886	DDX24	NRXN2	[activation]
3887	LRRK2	FARSA	[activation]
3888	OGT	SETD1A	[activation]
3889	SPRY4	GRB2	[activation]
3890	STAT3	FER	[activation]
3891	EZR	TSC1	[inhibition]
3892	PGAM5	STRN3	[activation]
3893	MRFAP1L1	TXN2	[activation;inhibition]
3894	MED1	GATA4	[activation]
3895	NXF1	CCNG1	[activation]
3896	SH3GL3	SHC3	[activation]
3897	NCAPD3	SLX4	[activation]
3898	PPP1CC	PPP1R3D	[inhibition]
3899	NCOA1	ESRRG	[activation]
3900	GSK3B	CTNND1	[activation]
3901	NDUFA4L2	KCNA4	[activation]
3902	AR	RAF1	[activation]
3903	TXK	ERBB3	[activation]
3904	MAPK14	GORASP2	[activation]
3905	IPO5	VCAM1	[activation]
3906	IRS2	NISCH	[activation]
3907	PPP2R1A	FTSJ1	[activation]
3908	FUS	SRSF10	[activation]
3909	FOS	HNF1A	[activation]
3910	INPP5K	HSPB1	[activation]
3911	HIST3H3	VCAM1	[activation]
3912	CFL1	RAD21	[activation]
3913	FKBPL	ESR1	[activation]
3914	F10	APOH	[activation]
3915	SUMO1	HLA-C	[activation]
3916	GAB1	PRKCI	[activation]
3917	CASP1	NFE2L2	[activation]
3918	TLE2	HMGB1	[inhibition]
3919	MSL1	CRKL	[activation]
3920	SYMPK	KMT2A	[activation]
3921	CDC25A	XPO1	[activation]
3922	TANK	MAP4K5	[activation]
3923	SMARCC1	MYC	[activation]
3924	NOXA1	PRKACA	[activation]
3925	SKIL	SMAD1	[activation]
3926	SDCBP	DAK	[inhibition]
3927	DNM2	ABL1	[activation]
3928	TGFBR1	PREX2	[activation]
3929	C8orf33	NXF1	[activation]
3930	EEF1A2	CHUK	[activation]
3931	MAPT	CALM1	[activation]
3932	ARHGEF19	DVL1	[activation]
3933	EGFR	ITCH	[activation]
3934	MAPK10	CDKN2A	[activation]
3935	CDK4	TGFBR1	[inhibition]
3936	CD300LD	TFF1	[activation]
3937	MUTYH	RPA1	[activation]
3938	HSPB1	ITGA4	[activation]
3939	ARF6	SMC2	[activation]
3940	ARF1	ATF2	[activation]
3941	CAMLG	KCNA2	[activation]
3942	CASP3	MAPK8IP3	[activation]
3943	CDC5L	ANXA7	[activation]
3944	TBK1	RELA	[activation]
3945	MAPKAPK3	CDKN2A	[activation]
3946	FAM203A	POLA2	[activation]
3947	SPTAN1	ERCC4	[activation]
3948	BAG2	DNAJC6	[inhibition]
3949	PRKACA	FXYD1	[activation]
3950	ENKD1	DVL3	[activation]
3951	PPP2CA	BMPR1B	[inhibition]
3952	PIK3R1	FLNB	[activation]

3953	PAK7	RPA3	[activation]
3954	NR1H3	FOXO3	[inhibition]
3955	ICT1	MRPL12	[activation]
3956	YES1	TRAF2	[activation]
3957	SAV1	RASSF4	[activation]
3958	SNW1	MLH1	[activation]
3959	RPA3	RAB13	[activation]
3960	NOTCH2NL	HIST1H2AG	[activation]
3961	ZAK	IGHM	[activation]
3962	BCL6	FBXO11	[activation]
3963	SMAD7	TOLLIP	[activation]
3964	CACNA1A	CACNB4	[activation]
3965	SPP1	EEF1A1	[activation]
3966	RAP1A	RAP1GDS1	[activation]
3967	RAB5A	KCNN3	[activation]
3968	PICK1	GRIA2	[activation]
3969	TUBG1	NBN	[activation]
3970	UBE2K	HLA-B	[activation]
3971	SRPK2	DHX8	[activation]
3972	DUSP15	FYN	[activation]
3973	STK4	IPO7	[activation]
3974	ROBO1	ROBO4	[activation]
3975	BLNK	ERBB2	[activation]
3976	MAP3K7	HIST1H4A	[activation;inhibition]
3977	PLEKHO1	SMURF1	[inhibition]
3978	PIK3R1	HGS	[activation]
3979	MYC	CALD1	[activation]
3980	RELA	POTEJ	[activation]
3981	CREG1	CAPN1	[activation]
3982	KIAA0232	WNK1	[activation]
3983	TXLNA	STX3	[activation]
3984	ZAP70	SLA2	[activation]
3985	EIF4G1	GK	[activation]
3986	RAB3IL1	RAB11A	[activation]
3987	HSF1	TTC5	[activation]
3988	ZBED4	NOTCH1	[activation]
3989	RRAS	ZHX2	[activation]
3990	SOCS1	FYN	[inhibition]
3991	VWF	HSP90B1	[activation]
3992	MSR1	HSPA1A	[inhibition]
3993	CALM1	ESR1	[activation]
3994	PPP2R5B	AXIN1	[activation]
3995	RSU1	LRCH3	[activation]
3996	GMPPA	BTC	[activation]
3997	ERC1	RIMS1	[activation]
3998	PLCG1	TUB	[activation]
3999	PRR20E	TOLLIP	[activation]
4000	WWOX	ATM	[activation]
4001	F2R	CAPN1	[activation]
4002	RPA1	RAD51	[activation]
4003	IKBKB	NR2C2	[activation]
4004	C7orf25	RASSF1	[inhibition]
4005	SP1	CHD3	[activation]
4006	SRPK2	MPP3	[activation]
4007	NXF1	CMPK1	[activation]
4008	MCM7	DDX21	[activation]
4009	MAPK10	HDAC1	[activation]
4010	PRPF40A	VCL	[activation]
4011	LYN	PRKDC	[activation]
4012	SETDB1	HSPB3	[activation]
4013	ANAPC15	ANAPC10	[activation]
4014	FRS2	RND1	[activation]
4015	MAPK3	RCAN1	[activation]
4016	MYC	LIMS1	[activation]
4017	ZXDA	CIITA	[activation]
4018	CRMP1	CCL18	[activation]
4019	SERPINA1	DERL2	[inhibition]
4020	TGFB1	AURKA	[activation]
4021	MDM2	PC	[activation]
4022	PIK3R2	GOLGA2	[activation]
4023	SIK2	HSPA2	[activation]
4024	NOTCH1	WWTR1	[activation]
4025	CDK7	PRKCI	[activation]
4026	SRPK2	PRPF40A	[activation]
4027	RICTOR	DHRS4	[activation]
4028	JAK3	APOA1	[inhibition]

4029	EDA2R	TRAF6	[activation]
4030	FKBP5	STK11	[activation]
4031	VEPH1	BMPR1B	[activation]
4032	SCN8A	GNAO1	[activation;inhibition]
4033	RAD21	PPP1R15A	[inhibition]
4034	GNAI1	RAD52	[activation]
4035	SERBP1	KAT2B	[activation]
4036	ATRX	EIF4A2	[activation]
4037	SENP2	PML	[activation]
4038	PTPN11	FLT1	[activation]
4039	AHNAK	MDM2	[activation]
4040	APC	PTPN13	[inhibition]
4041	PCBP1	HLA-B	[activation]
4042	MPPED1	SPP1	[activation]
4043	CYSLTR1	CYSLTR2	[activation]
4044	FBXL19	STRADA	[activation]
4045	GRIA2	SPTBN1	[activation]
4046	NCK1	FRS2	[activation]
4047	RASGRP4	RAP1A	[activation]
4048	EIF2AK3	BCL6	[activation]
4049	AKAP2	CRK	[activation]
4050	PRKDC	IKBKB	[activation]
4051	NEDD9	CRKL	[activation]
4052	CDK18	HSP90AB1	[activation]
4053	LMNA	GRB2	[activation]
4054	HIST1H2BL	CD81	[activation]
4055	SGOL1	PPM1A	[activation]
4056	MTERFD2	FOS	[activation]
4057	OLFM2	RPS6	[activation]
4058	PPIG	SP1	[activation]
4059	F13A1	CDKN1A	[activation]
4060	SYK	STAT3	[activation]
4061	ADRB2	INSR	[activation]
4062	PIK3R4	YWHAG	[activation]
4063	PPP1R12B	PRKG1	[inhibition]
4064	EHD4	CCDC8	[activation]
4065	ICT1	MRPL37	[activation]
4066	ACTB	LGALS13	[activation]
4067	HSPB1	RPAP3	[activation]
4068	PTPRG	CSK	[activation]
4069	MC4R	PRKACA	[activation]
4070	MAPKAPK2	MAPK1	[activation]
4071	MCM2	ATM	[activation]
4072	TXLNA	CCHCR1	[activation]
4073	EP300	PIN1	[activation]
4074	C11orf84	GRB2	[activation]
4075	COL1A2	PDGFA	[activation]
4076	LRRK2	CALM1	[activation]
4077	GABARAPL1	STK4	[activation]
4078	PAXIP1	MRPL33	[activation]
4079	S100A14	SMAD4	[inhibition]
4080	IRS4	P4HA2	[activation]
4081	FOXO4	CTNNB1	[activation]
4082	FOS	ATF3	[activation]
4083	GNAI3	CD59	[activation]
4084	ACVR2A	BMP6	[activation]
4085	MLH1	COPA	[activation]
4086	SRPK2	RSRC1	[activation]
4087	SNW1	ANXA7	[activation]
4088	BCAP31	IGHD	[activation]
4089	MCM5	RAD50	[activation]
4090	GNAI3	PRKAR2B	[activation]
4091	PARD3	LATS1	[inhibition]
4092	SORBS1	MLLT4	[activation]
4093	CNTNAP1	SRC	[activation]
4094	RIPK1	CRADD	[activation]
4095	RUNX1	SMAD2	[activation]
4096	CRY2	PER2	[inhibition]
4097	EGFR	STAT2	[activation]
4098	HSPA4	ERBB2	[activation]
4099	HEATR2	ARF6	[activation]
4100	GRB2	TPM2	[activation]
4101	NFKB1	TNIP3	[activation]
4102	TAF7	HSF1	[activation]
4103	PTK2	DNM2	[activation]
4104	MAPKAPK2	ETV1	[activation]

4105	LNX2	UBE2T	[activation]
4106	MPDZ	HTR2A	[activation]
4107	C20orf195	SSSCA1	[activation]
4108	JUN	HDAC1	[activation]
4109	STK4	RASSF6	[activation]
4110	HSPA8	NFKB2	[inhibition]
4111	SRC	SMARCE1	[activation]
4112	OXSR1	SMURF1	[inhibition]
4113	KLK3	SERPINA1	[inhibition]
4114	LYN	CSF2RA	[activation]
4115	GSK3A	PRKCA	[activation;inhibition]
4116	RPS6KA2	JUN	[activation]
4117	NR2E3	E2F4	[inhibition]
4118	GSK3B	CDH1	[activation]
4119	DDX27	OBSL1	[activation]
4120	XPO7	EGFR	[activation]
4121	SSH3	ACTA1	[activation]
4122	FAM13B	YWHAE	[activation]
4123	TP53	BACH1	[activation]
4124	PTPRJ	GHR	[activation]
4125	CLDN1	WNK4	[activation]
4126	RGS6	GNB1	[activation;inhibition]
4127	CDHR2	MAST2	[activation]
4128	FGF7	COL2A1	[activation]
4129	RGS4	PTAFR	[activation;inhibition]
4130	STAT6	STAT2	[activation]
4131	ADRB2	GNB2	[activation]
4132	FASTKD1	ICT1	[activation]
4133	PPFIBP1	NXF1	[activation]
4134	RRN3	TAF1B	[activation]
4135	TFRC	ERBB2	[activation]
4136	OTUD1	RIPK1	[activation]
4137	CDH2	PTPN22	[activation]
4138	CALM1	USP20	[activation]
4139	GHR	SHC1	[activation]
4140	SHC1	APP	[activation]
4141	CADM1	MPP3	[activation]
4142	TP53BP1	MYC	[activation]
4143	MAPK3	CRP	[activation]
4144	GSN	UBE2Q2	[activation]
4145	MPZL2	ATF4	[activation]
4146	PRF1	TRIM54	[activation]
4147	CSK	CBL	[activation]
4148	YWHAB	RASAL2	[activation]
4149	RPS6KB2	CDC5L	[activation]
4150	GATA1	MED21	[activation]
4151	PTPN11	PDGFRA	[activation]
4152	PPM1B	CDK2	[activation]
4153	RPA1	ATRX	[activation]
4154	ANXA2	TRAF6	[activation]
4155	PRKAA1	YWHAZ	[inhibition]
4156	LEPR	GRB2	[activation]
4157	MYC	HSD17B4	[activation]
4158	KIF23	ARF3	[activation]
4159	CRY1	GRN	[activation]
4160	HSPA8	RPA1	[activation]
4161	BMP2K	MBP	[inhibition]
4162	NFKBIA	POM121	[activation]
4163	CARD11	CBLB	[activation]
4164	ICAM1	RPLP2	[activation]
4165	ATF4	GTF2F2	[activation]
4166	JUP	FER	[activation;inhibition]
4167	RAB21	MACF1	[activation]
4168	CITED2	TFAP2A	[activation]
4169	CTTNBP2NL	PPP2R1A	[inhibition]
4170	IL6	SH3GL2	[activation;inhibition]
4171	MTFR2	GOLGA2	[activation]
4172	SAT1	GDF9	[activation]
4173	PIN1	PLK1	[activation]
4174	MAP4K4	HSP90AB1	[activation]
4175	HSF1	CDC20	[activation]
4176	TRIP10	WASL	[activation]
4177	TGFB2	FMOD	[activation]
4178	INPP5K	ANXA7	[activation]
4179	GRIK2	CDH2	[activation]
4180	ZC3H11A	RAPGEF4	[activation;inhibition]

4181	TRIM28	MYB	[activation]
4182	STX4	SNAP29	[activation]
4183	CHRND	GRB2	[activation]
4184	PLD1	ACTA1	[activation]
4185	SPATA31E1	MYC	[activation]
4186	HSPG2	CTGF	[activation]
4187	RPL13	MAPKAPK5	[activation]
4188	BAG3	TIMP2	[activation]
4189	USHBP1	TXLNA	[activation]
4190	SGOL1	RAD21	[activation]
4191	EIF4A1	CENPA	[activation]
4192	PPP2R1A	CTNNBIP1	[inhibition]
4193	CCDC8	JUP	[inhibition]
4194	VTN	LNX2	[inhibition]
4195	SAA2	COL4A1	[activation]
4196	CBLB	WAS	[activation]
4197	ACTB	CD81	[activation]
4198	SPP1	CISD2	[activation]
4199	RHOA	FAM65B	[activation]
4200	FBXW5	MYB	[activation]
4201	GRB2	SPRY2	[activation]
4202	RHEB	TPT1	[activation]
4203	IPO7	TRAF6	[activation]
4204	MAP4K3	LCP2	[activation]
4205	NBN	MDM2	[activation]
4206	PTP4A3	STRAP	[inhibition]
4207	HSPB1	SPACA7	[activation]
4208	HSPA2	SRC	[activation]
4209	ACVR1B	TDGF1	[activation]
4210	APP	AREG	[activation]
4211	PPP2R5B	SGOL1	[activation]
4212	APC	MYO6	[activation]
4213	AR	FGR	[activation]
4214	RANBP9	CD4	[activation]
4215	CREBBP	SMARCA4	[activation]
4216	CAV1	TNF	[activation]
4217	TRAPPC10	NOTCH1	[activation]
4218	RAB10	RPA2	[activation]
4219	UNK	PAX8	[activation]
4220	CDKN1A	SDF4	[activation]
4221	HDAC4	CAMK1	[activation]
4222	TGFBR3	TGFB3	[activation]
4223	PIAS1	AXIN1	[inhibition]
4224	PDGFRA	TGFBR2	[activation]
4225	TEK	TNIP2	[activation]
4226	RASA1	BBS10	[activation]
4227	YWHAB	ARHGEF2	[activation]
4228	DOCK4	EIF4A3	[activation]
4229	HYOU1	H2AFX	[activation]
4230	TBK1	TICAM1	[activation]
4231	PLS3	GRB2	[activation]
4232	BTK	FASLG	[activation]
4233	IL12A	WASF1	[activation]
4234	ACVR2A	BMP2	[activation]
4235	CSF2RB	MAD2L1	[activation]
4236	AURKB	CIB1	[activation]
4237	GABARAP	DKC1	[activation]
4238	STX1A	MDM2	[activation]
4239	PZP	TGFB2	[activation]
4240	ANAPC10	PTBP3	[activation]
4241	HSPB1	EIF5	[activation]
4242	PLEK	THBS1	[activation]
4243	OXTR	GPRASP1	[activation]
4244	PLA2G4A	RELA	[activation]
4245	PRKCH	PTPN11	[activation]
4246	ACTB	PLD2	[activation]
4247	KPNB1	RAN	[activation]
4248	PAXIP1	MCM6	[activation]
4249	SMAD2	HOXD13	[activation]
4250	PLK1	TP53	[activation]
4251	HMGA2	SMAD1	[activation]
4252	PLD2	RPS6KB1	[activation]
4253	CRK	HSPA8	[activation]
4254	LRRK2	EIF4B	[activation]
4255	ANLN	SH3KBP1	[activation]
4256	MCM4	MDM2	[activation]

4257	RPS6KA4	FOS	[activation]
4258	FHL2	FAM154A	[inhibition]
4259	GHRL	LEP	[activation]
4260	CD40	RFC5	[activation]
4261	CAMP	IGF1R	[activation]
4262	FASN	OBSL1	[activation]
4263	APOB	ARFGAP1	[activation]
4264	HSPA5	MTNR1B	[activation]
4265	FYN	DKKL1	[activation]
4266	EPAS1	EIF4A1	[activation]
4267	ITGB2	FUT4	[activation]
4268	FEM1B	FAS	[activation]
4269	NTRK1	PTPRG	[activation]
4270	NUDC	TSSC1	[activation]
4271	ZAP70	LCK	[activation;inhibition]
4272	TSC2	SMAD3	[inhibition]
4273	RAP1A	RASIP1	[activation]
4274	ACTB	CRK	[activation]
4275	BEGAIN	RBL1	[inhibition]
4276	VAMP1	VAPA	[activation]
4277	C1QA	DCN	[activation]
4278	PSD	GLRA2	[activation]
4279	YY1	CTCF	[activation]
4280	XPO7	PDPK1	[activation]
4281	EGFR	LGALS8	[activation]
4282	CIB2	MYOD1	[activation]
4283	NCAPD2	SOX2	[activation]
4284	CEBPB	SMARCC1	[activation]
4285	PYCARD	MYO1C	[inhibition]
4286	PML	CTNNB1	[activation]
4287	S100A2	MDM2	[activation]
4288	OBSL1	HNRNPA0	[activation]
4289	FREM2	MDM2	[activation]
4290	FGFR3	FGFR2	[activation]
4291	GRIA1	CXCR2	[activation]
4292	TICAM1	IRF3	[activation]
4293	CAMKK2	SLC25A11	[activation]
4294	SRSF3	NXF1	[activation]
4295	MBD3L2	POLD3	[activation]
4296	GRAP	PLCG1	[activation]
4297	BMX	RUFY2	[activation]
4298	IL3RA	JAK1	[activation]
4299	CHEK2	TP53BP1	[activation]
4300	HSPBP1	HSPA1A	[inhibition]
4301	MAP2K1	BLVRA	[activation]
4302	APP	GPR3	[activation]
4303	PPM1G	LRPAP1	[inhibition]
4304	GNAQ	RGS16	[activation]
4305	REN	CTSB	[activation]
4306	SHH	SEL1L	[inhibition]
4307	RARA	SRC	[activation]
4308	SARS	GH1	[activation]
4309	BTN3A3	CTSL	[activation]
4310	HDAC1	BUB3	[inhibition]
4311	IL1R1	TOLLIP	[activation]
4312	SYN1	GRB2	[activation]
4313	GNA11	ADRB2	[activation]
4314	YWHAB	TNIP3	[activation]
4315	SRPK2	ADPRH	[activation]
4316	TRPV4	LCK	[activation]
4317	ADCY10	SSTR5	[activation]
4318	TRAF6	NHP2	[activation]
4319	YWHAE	CDC25A	[activation]
4320	TNF	CSF1	[activation]
4321	F12	C1QBP	[activation]
4322	FYN	SH3BP2	[activation]
4323	MYC	MAPK8	[activation]
4324	MAGED2	CDK18	[activation]
4325	TP53	PPP2R2B	[activation]
4326	AKT1	RAB3D	[activation]
4327	CCDC57	TSGA10IP	[activation]
4328	ENG	ARRB2	[inhibition]
4329	SYK	RASA1	[activation]
4330	ESR1	SCYL2	[activation]
4331	KPNA2	CREBBP	[activation]
4332	EPS8	CD3E	[activation]

4333	IDE	CCL4	[activation]
4334	VDAC3	ICT1	[inhibition]
4335	GH1	SHC1	[activation]
4336	CAV3	INSR	[inhibition]
4337	REL	TSSK3	[inhibition]
4338	CHUK	UBE2E3	[activation]
4339	SSPO	ATXN7L1	[inhibition]
4340	ULK2	PLK1	[activation]
4341	RAP1GAP	CNR1	[activation]
4342	FKBPL	SIM2	[activation]
4343	ENOX2	CIB3	[activation]
4344	UBE2D3	RELA	[activation]
4345	REPS2	CDK1	[activation]
4346	RANBP2	SENP1	[activation]
4347	JUND	BCL6	[activation]
4348	ITGB1	MMP9	[activation]
4349	BID	CSNK2B	[activation]
4350	MORF4L2	PALB2	[activation]
4351	AR	GAK	[activation]
4352	C9orf9	DDX24	[activation]
4353	HDAC1	EHMT2	[activation]
4354	FST	C8orf33	[inhibition]
4355	VDAC3	IKBKE	[inhibition]
4356	ANKRD11	RAC3	[activation]
4357	MYOG	CDH18	[activation]
4358	YY1	MDM2	[activation]
4359	RTN4R	LINGO1	[activation]
4360	INSR	GRB14	[activation]
4361	CDK4	CEBPA	[inhibition]
4362	FBXO5	ANAPC11	[activation]
4363	CSF1R	SOS1	[activation]
4364	MAP2K1	BANP	[activation]
4365	LY96	CDKN2C	[inhibition]
4366	MXI1	BUB1B	[inhibition]
4367	METTL13	MYC	[activation]
4368	PRKCA	CFTR	[activation]
4369	ACTG1	MDK	[activation]
4370	SKI	GATA1	[activation]
4371	CDH13	RAD51	[activation]
4372	RAC1	TRAF6	[activation]
4373	NOTCH2NL	EIF4E2	[activation]
4374	GH1	PTGES3	[activation]
4375	UBE2E2	HERC5	[activation]
4376	MAPK8	MAPK9	[activation]
4377	HLA-E	CD8A	[activation]
4378	POLR2A	HMGN1	[activation]
4379	PCSK9	LDLR	[activation]
4380	SETD7	RELA	[activation]
4381	MAPK6	SERPINA4	[inhibition]
4382	SDCBP	FOXP2	[inhibition]
4383	TRIB3	CDC25A	[inhibition]
4384	SEC16A	LATS1	[inhibition]
4385	MATK	LYN	[activation]
4386	NFATC2	YWHAZ	[activation]
4387	JUNB	FOSL2	[activation]
4388	PUM2	ATG5	[activation]
4389	PPARG	MAFF	[activation]
4390	CCDC8	GRK6	[activation;inhibition]
4391	CCAR2	IKBKE	[inhibition]
4392	PALB2	IL24	[activation]
4393	ESR1	CACUL1	[activation]
4394	KIDINS220	VCAM1	[activation]
4395	CHRM3	GZMB	[activation]
4396	NSD1	RXRA	[inhibition]
4397	RHOV	NCK2	[activation]
4398	MYC	PDS5A	[activation]
4399	DAAM1	B3GALT4	[activation]
4400	ESR1	ANGPTL4	[activation]
4401	EIF1B	EIF2S1	[activation]
4402	PTK2	F13A1	[activation]
4403	ITGA6	ITGB4	[activation]
4404	TSSK6	HSPA8	[inhibition]
4405	GRB2	ANK2	[activation]
4406	ICT1	MRPL43	[activation]
4407	HDAC1	TWIST1	[activation]
4408	STRN	PPP2CA	[inhibition]

4409	SRPK2	SRSF12	[activation]
4410	LRRK2	PKM	[activation]
4411	SMURF1	PRPS1	[inhibition]
4412	STAT5A	TXNDC11	[activation]
4413	CYB561	EDA	[activation]
4414	ARRB2	TAB1	[inhibition]
4415	YTHDC2	IRF4	[activation]
4416	IGSF21	BCR	[activation]
4417	WHSC1L1	SMARCA2	[activation]
4418	GJA4	OPRK1	[activation]
4419	ACTG1	ABLIM1	[activation]
4420	CUTC	PIM2	[inhibition]
4421	FDPS	FGFR1	[activation]
4422	ATF2	DDIT3	[activation]
4423	LYN	HSP90AA1	[activation]
4424	CNKSR1	MAP2K7	[activation]
4425	CTNNB1	OBSL1	[activation;inhibition]
4426	RAC1	ICMT	[activation]
4427	OGDH	NXF1	[activation]
4428	NCF1	PRKCA	[activation]
4429	MYD88	SMAD3	[activation]
4430	LATS1	MARK1	[inhibition]
4431	SH2D1B	SLAMF1	[activation]
4432	PRKRA	RB1	[activation;inhibition]
4433	SRGAP2	YWHAG	[activation]
4434	SYNGAP1	KDR	[activation]
4435	CHEK1	CDKN1A	[activation;inhibition]
4436	EGFR	PRKDC	[activation]
4437	SP1	SENP6	[activation]
4438	STK38	RICTOR	[activation]
4439	CASP3	SARS2	[activation]
4440	GRB2	MAPK14	[activation]
4441	MTNR1A	RAC1	[activation]
4442	YWHAZ	SMARCA4	[activation]
4443	ATP13A2	F2R	[activation]
4444	STAT5A	MAGEA12	[activation]
4445	NOD1	NLRP1	[activation]
4446	HSP90AB1	ARRB2	[activation]
4447	BCAP31	GPRIN2	[activation]
4448	RARA	RUNX1	[activation]
4449	VAV2	MET	[activation]
4450	AHNAK	NXF1	[activation]
4451	TRAF2	NECAP2	[activation]
4452	BCL10	ATM	[activation]
4453	FYN	ASAP2	[activation]
4454	MAP4K2	SERPINA4	[inhibition]
4455	PTPN14	CDH1	[activation]
4456	LGALS8	NDP	[activation]
4457	SMAD4	CNKSR1	[activation]
4458	CSF3R	PTPN11	[activation]
4459	CASP8AP2	TRAF2	[activation]
4460	SETD5	NXF1	[activation]
4461	LIFR	PTPN11	[activation]
4462	RIMS1	LRRK1	[activation]
4463	IKBKB	COPS7A	[activation]
4464	GABARAP	TFRC	[activation]
4465	APP	CXCL14	[activation]
4466	MIB1	DAPK1	[activation]
4467	CFL1	ACTG1	[activation]
4468	LRP8	SS18	[activation]
4469	LYN	PAK2	[activation]
4470	PAK4	YWHAZ	[activation]
4471	HSPB1	POP7	[activation]
4472	SRGAP3	TNIK	[activation]
4473	IQSEC1	IRAK2	[activation]
4474	PAK2	RPS6	[activation]
4475	NR1H3	NR0B2	[activation]
4476	SMAD3	ANAPC10	[activation]
4477	POLR2C	ATF4	[activation]
4478	SPRY2	CBLB	[inhibition]
4479	MYOG	CALM1	[activation]
4480	VCAM1	SRSF9	[activation]
4481	YY1	MECOM	[activation]
4482	UBR4	AURKB	[activation]
4483	SNAI1	TP53	[activation]
4484	JUN	MACF1	[activation]

4485	NR1H3	CEP350	[activation]
4486	LATS1	XIAP	[inhibition]
4487	NOP10	SRPK3	[activation]
4488	PPP2CA	BCL2	[inhibition]
4489	TEK	PTPRB	[activation]
4490	UFM1	UBE2E3	[activation]
4491	HSPH1	ATF2	[activation]
4492	PLG	PLAU	[activation]
4493	PAX3	GRB2	[activation]
4494	DUSP3	HLA-B	[inhibition]
4495	RXRA	HDAC3	[inhibition]
4496	CTTN	CTNND1	[activation]
4497	CDH1	CA9	[activation]
4498	P2RX4	NOTCH2NL	[activation]
4499	CASP10	TNFRSF1A	[activation]
4500	AR	JUN	[activation]
4501	MDM2	DDX5	[activation]
4502	TOLLIP	CHD6	[activation]
4503	TAF1	RBL1	[activation]
4504	SIM2	NUDC	[activation]
4505	PPARA	PPARGC1A	[activation]
4506	PTPN6	FLT3	[activation;inhibition]
4507	PPP1R14A	PRKCE	[activation]
4508	FYB	SKAP2	[activation]
4509	PRKCB	PA2G4	[activation]
4510	STX4	SCAMP5	[activation]
4511	SERPINF2	PRSS1	[activation]
4512	MAD2L1	INSR	[inhibition]
4513	ITGA4	RAP2B	[activation]
4514	HOXA7	MEIS1	[activation]
4515	NR1H3	MDFI	[activation]
4516	HPN	F7	[activation]
4517	CEP19	AK4	[inhibition]
4518	STAT3	TRIP10	[activation]
4519	ELK1	MAPK11	[activation]
4520	SCRIB	PHLPP1	[activation]
4521	NDRG1	BCL2L11	[activation]
4522	NCOA1	NCOA3	[activation]
4523	DVL1	RASSF10	[inhibition]
4524	PXN	BCAR1	[activation]
4525	TLR4	TLR1	[activation]
4526	EGFR	HSP90AB1	[activation]
4527	PDCD6	FAS	[activation]
4528	TNF	NFKBIA	[activation]
4529	SGOL1	PRKDC	[activation]
4530	GDF15	MDFI	[activation]
4531	MDM2	CSNK1E	[activation]
4532	ZFYVE9	CHD6	[activation]
4533	TRAP1	MDM2	[activation]
4534	IGF2	TF	[activation]
4535	TWF2	NELFCD	[activation]
4536	ITGA4	STMN1	[activation]
4537	PRDX5	ICT1	[activation]
4538	ANAPC1	UBE2S	[activation]
4539	MAPK1	SUPT20H	[activation]
4540	NXF1	CKAP4	[activation]
4541	ACVR1B	BAMBI	[inhibition]
4542	ANXA7	GNB2	[activation]
4543	DES	ATF2	[activation]
4544	GAK	CCNG1	[activation]
4545	DNAJA2	TP53	[activation]
4546	STMN1	PRKACA	[activation]
4547	TP53	G3BP1	[activation]
4548	ATR	DCLRE1C	[activation]
4549	FGF3	EBNA1BP2	[activation]
4550	ANKRD28	RAD21	[activation]
4551	GCC1	GLI1	[inhibition]
4552	SHB	JAK3	[inhibition]
4553	GMFB	TRAF6	[activation]
4554	DYRK1A	SH3GL2	[activation]
4555	AKAP12	EGFR	[activation]
4556	CRY2	CSNK1E	[activation]
4557	SNW1	RAD50	[activation]
4558	SRF	SMAD7	[activation]
4559	MCM7	HIST3H3	[activation]
4560	NUDCD3	KLHL34	[activation]

4561	YWHAG	YAP1	[activation]
4562	RPL19	ICAM1	[activation]
4563	RPS6KA1	YWHAB	[activation]
4564	PRKCB	TNFRSF1A	[activation]
4565	IGHM	CTR9	[activation]
4566	BRAF	MAPKAPK3	[activation]
4567	E2F3	SP1	[activation]
4568	IL17RD	MAP3K7	[activation]
4569	GRIA4	SDCBP	[activation]
4570	MOB1A	LATS1	[inhibition]
4571	EEF1D	TRAF6	[activation]
4572	TCOF1	RPA3	[activation]
4573	STAT1	DUSP3	[inhibition]
4574	MAPK1	CEP55	[activation;inhibition]
4575	DLGAP5	GOLGA2	[activation]
4576	PPARGC1A	ESRRG	[activation]
4577	NOS3	ST13	[activation]
4578	SCRIB	PRKCA	[activation]
4579	SRPK1	FLOT1	[activation]
4580	SUMO4	PGK1	[activation]
4581	PHLDA3	WWC3	[activation]
4582	CCNA2	KAT2B	[activation]
4583	STK3	PTPN14	[activation]
4584	RPA2	ANXA2	[activation]
4585	ICT1	ECH1	[activation]
4586	PIAS2	TSR2	[activation]
4587	IKBKG	EPHA4	[activation]
4588	PCNA	FOXO3	[inhibition]
4589	CDC25C	PIN1	[activation]
4590	MAPK6	PVRL2	[activation]
4591	UBA5	CBR1	[activation]
4592	BCR	YWHAH	[activation]
4593	CASP1	FGF2	[activation]
4594	GM2A	PLD2	[activation]
4595	NCOA3	PGR	[activation]
4596	MYD88	CISH	[activation;inhibition]
4597	POLD3	POLD1	[activation]
4598	A2M	MAST1	[inhibition]
4599	APP	KCNK5	[activation]
4600	GCG	FAP	[activation]
4601	RIPK3	CASP8	[inhibition]
4602	PPP2CA	PPP2R3B	[activation]
4603	TGFBR1	TRAF6	[activation]
4604	KLHL23	CDKN1A	[activation;inhibition]
4605	CREB3	PSENEN	[activation]
4606	DGKD	TNFRSF14	[activation]
4607	CD4	PTPRC	[activation]
4608	CDC42	TP53	[activation]
4609	CSK	DAB2	[activation]
4610	HSPA2	MEPCE	[activation]
4611	NFKB1	E2F1	[activation]
4612	BTK	PLCG2	[activation]
4613	MLH1	SPERT	[activation]
4614	MAPKAPK2	SMNDC1	[activation]
4615	ERBB2	CRKL	[activation]
4616	KSR1	VRK2	[activation]
4617	SRC	SORBS1	[activation]
4618	RASGRP3	PRKCE	[activation]
4619	EIF4E	MTOR	[inhibition]
4620	FYN	DLGAP1	[activation]
4621	GNL3	WHSC1	[activation]
4622	RPL6	ICAM1	[activation]
4623	AURKB	FKBP5	[activation]
4624	PPARG	TFAP2A	[activation]
4625	NOTCH1	FBXW7	[activation]
4626	IKBKB	NLRC5	[activation]
4627	RPA2	STAT3	[activation]
4628	TRAF3	MAP3K14	[activation]
4629	GIMAP5	LSMEM2	[activation]
4630	EPHB3	MLLT4	[activation]
4631	PRRT2	SNAP25	[activation]
4632	RAC1	PAK7	[activation]
4633	NRTN	GFRA2	[activation]
4634	NUDCD3	KLHDC10	[activation]
4635	VCAM1	STRAP	[activation]
4636	CDH13	PTPN1	[activation]

4637	FGF7	COL6A1	[activation]
4638	NUP133	SEH1L	[activation]
4639	TP53BP2	TP53	[activation]
4640	ARRB1	CCL14	[activation]
4641	TTK	CDC16	[activation]
4642	TCP10	APP	[activation]
4643	FECH	MME	[activation]
4644	CEBPA	UBTF	[activation]
4645	SNRK	HSPA1A	[inhibition]
4646	RHEB	BRAF	[activation]
4647	SUMO3	LRPAP1	[inhibition]
4648	GARS	TRAF6	[activation]
4649	PARP8	YWHAB	[activation]
4650	TRAF1	ACTG1	[activation]
4651	TP53	LYN	[activation]
4652	TMC5	NUMBL	[inhibition]
4653	OXT	ESR2	[activation]
4654	PLCG2	PRKD1	[activation]
4655	GADD45A	NRBP1	[activation]
4656	BTRC	BTG2	[activation]
4657	CASP9	GMEB1	[inhibition]
4658	MAPK8	SH3BP5	[activation]
4659	CSF2RB	YWHAZ	[activation]
4660	RAPGEF2	SPP1	[activation]
4661	RAP1GAP	CSNK1G2	[inhibition]
4662	CENPA	DIAPH1	[activation]
4663	NUDC	HCFC2	[activation]
4664	RASL12	APP	[activation]
4665	RXRA	RELA	[inhibition]
4666	CCNE1	POLD1	[activation]
4667	DNM2	DDX39B	[activation]
4668	LUC7L	FAM173A	[activation]
4669	RPA4	APP	[activation]
4670	ACAD8	ETS1	[activation]
4671	RPL24	ICAM1	[activation]
4672	HSP90AB1	JAK2	[activation]
4673	EXOC7	HGS	[activation]
4674	PRKAR2A	ARFGEF2	[activation]
4675	RRAD	CALM1	[activation]
4676	TXNIP	TP53	[activation]
4677	GRIA4	CAMK2A	[activation]
4678	LYN	PPP1R15A	[inhibition]
4679	HLA-B	LAGE3	[activation]
4680	SMC2	SNW1	[activation]
4681	RASSF1	SKP1	[inhibition]
4682	YWHAE	VASP	[activation]
4683	LMX1A	BMPR2	[activation]
4684	CEBPB	HOMER3	[activation]
4685	MDM2	MGEA5	[activation]
4686	SGTA	SPP1	[activation]
4687	CS	HSPB1	[activation]
4688	SH2D1B	PECAM1	[activation]
4689	AURKB	SEPT1	[activation]
4690	CFTR	GNA11	[activation]
4691	TIMM50	TNFRSF10B	[activation]
4692	APOE	PLEKHA6	[activation]
4693	GNAI2	KRT40	[activation;inhibition]
4694	CDK6	CDK2	[inhibition]
4695	RPA3	CHD4	[activation]
4696	EHD4	PHLDA3	[activation]
4697	NCOA3	ARNT2	[activation]
4698	BMP4	BGN	[activation]
4699	FBXL21	CRY1	[inhibition]
4700	CAPN10	FLOT1	[activation]
4701	LGALS9	JUP	[activation]
4702	ASS1	MT2A	[activation]
4703	TRAF3	TNFRSF11A	[activation]
4704	ADRB2	JKAMP	[activation]
4705	IGFBP5	SPP1	[activation]
4706	NCAM1	PPP1CA	[activation]
4707	HIST3H3	AIRE	[activation]
4708	CD4	HSPA8	[activation]
4709	CD81	PCBP2	[activation]
4710	TTC28	SSPO	[inhibition]
4711	PAK2	SH3PXD2A	[activation]
4712	SH2D1A	SLAMF6	[activation]

4713	SMARCA2	KAT2B	[activation]
4714	CAMKK2	SMC1A	[activation]
4715	MAPK9	MACF1	[activation]
4716	AURKA	NME1	[activation]
4717	MYOD1	CALM1	[activation]
4718	YES1	MST1R	[activation]
4719	E2F1	CUL1	[inhibition]
4720	GNA12	TEC	[activation]
4721	F2	AKR7A2	[activation]
4722	YWHAE	MYC	[activation]
4723	STK11	RPS6KA3	[activation]
4724	LRP6	FZD8	[activation]
4725	MPZL1	PTPN11	[activation]
4726	FOXA2	EN2	[activation]
4727	RAB2A	GOLGA2	[activation]
4728	GCN1L1	ILK	[activation]
4729	RELA	GCN1L1	[activation]
4730	MAPK1	ICK	[activation]
4731	NFKBIA	SUMO1	[activation]
4732	PTPN1	CSNK2A1	[activation]
4733	CDC6	MYC	[activation]
4734	CTNNBIP1	CDH5	[inhibition]
4735	WDR65	FOS	[activation]
4736	HCK	ABI1	[activation]
4737	TLR1	TLR2	[activation]
4738	ERCC6	MDM2	[activation]
4739	VASP	WASL	[activation]
4740	BCL6	PIN1	[activation]
4741	KLF5	WWTR1	[activation]
4742	NCOA3	SUFU	[activation]
4743	BTRC	CTTN	[inhibition]
4744	GTF2A2	HSF1	[activation]
4745	PECAM1	ITGA5	[activation]
4746	PTK2	UBE2H	[activation]
4747	FAM57A	MAPK4	[activation]
4748	IGFBP4	LRP6	[inhibition]
4749	PPP3R1	TP53	[activation]
4750	IL23A	HERPUD1	[activation]
4751	DLX4	ABL1	[activation]
4752	BTRC	IGF2BP1	[activation]
4753	USP7	RASSF1	[inhibition]
4754	STK3	MBP	[activation]
4755	CCDC8	YTHDC2	[activation]
4756	RAN	NUPR1	[activation]
4757	RAP1A	FTSJ1	[activation]
4758	HSPA9	MAP3K7	[activation]
4759	RPL36	GRB2	[activation]
4760	PTK2B	CD2AP	[activation]
4761	CASP3	AIFM1	[activation]
4762	CXXC1	OGT	[activation]
4763	TNFRSF8	TRAF2	[activation]
4764	DCC	EIF4E	[inhibition]
4765	ILK	PNKD	[activation]
4766	RPL14	TP53	[activation]
4767	TXNRD1	TXNDC17	[activation]
4768	SMAD4	TCTA	[activation]
4769	SRPK1	TULP2	[activation]
4770	PHLDA3	RUNX1-IT1	[activation]
4771	ATF4	NFKBIA	[activation]
4772	TRAF6	IL17RB	[activation]
4773	ATF2	S100A2	[activation]
4774	TGFBR1	ANAPC5	[activation]
4775	CELA1	A2M	[inhibition]
4776	ITGA4	HSPD1	[activation]
4777	CCDC8	GRN	[activation]
4778	SOX2	RUNX1	[activation]
4779	BEX1	GSK3B	[activation]
4780	AFF4	SPP1	[activation]
4781	ITGB3	CAPN1	[activation]
4782	DDX54	ESR1	[activation]
4783	ARF1	OPRM1	[activation]
4784	ABL2	ABL1	[activation]
4785	GSPT1	TRAF6	[activation]
4786	JAK2	PPP2CA	[inhibition]
4787	MTOR	PML	[activation]
4788	FOXO1	CCNB1	[activation]

4789	CKAP4	ATF4	[activation]
4790	BUB1B	SGOL1	[inhibition]
4791	ARRB1	ARPC5	[activation]
4792	PPM1B	CHUK	[activation]
4793	RPL27	ICAM1	[activation]
4794	MRPS34	ICT1	[activation]
4795	BMPR1A	TGFB1	[activation]
4796	RAP1A	HSPA1A	[inhibition]
4797	AKT1	MAPT	[activation]
4798	ITGA1	FABP3	[activation]
4799	MAPKAPK3	AURKA	[activation]
4800	FAM20C	PROP1	[activation]
4801	GOLGA2	MOS	[activation]
4802	PDE1A	APP	[activation]
4803	MAGEB2	RARA	[activation]
4804	MOS	SPERT	[activation]
4805	DDX39A	UBA5	[activation]
4806	ARF6	ARRB2	[activation]
4807	PHLPP1	PHLPP2	[inhibition]
4808	FXR2	PRKAA1	[inhibition]
4809	LARP1	ESR1	[activation]
4810	YWHAG	DDX17	[activation]
4811	CDC7	ORC1	[activation]
4812	ITGB2	ITGAL	[activation]
4813	CASP3	YWHAE	[activation]
4814	CD2	LYAR	[activation]
4815	GRPEL1	ATF2	[activation]
4816	CCDC57	CWF19L2	[inhibition]
4817	DAPK3	LINC00846	[activation]
4818	JAK3	GTF2B	[activation]
4819	BCAR1	EFS	[activation]
4820	KATNB1	EIF4A2	[activation]
4821	ANXA8	APP	[activation]
4822	ARHGEF6	PRKDC	[activation]
4823	GNAO1	ADORA1	[activation;inhibition]
4824	MVP	PTPN11	[activation]
4825	ACVR2B	FSTL1	[activation]
4826	LAMA5	MYOC	[activation;inhibition]
4827	MAPK1	SH2D3C	[activation]
4828	GNA11	MTNR1A	[activation]
4829	TNFRSF11B	TNFSF13	[activation]
4830	ERBB2	BAAT	[activation]
4831	PTK2	TSC2	[activation;inhibition]
4832	TP73	PAX6	[activation]
4833	ULBP2	PDGFRA	[activation]
4834	CDKN1C	CDK2	[inhibition]
4835	UBC	RHOA	[activation]
4836	TCEB2	SOCS2	[inhibition]
4837	RPS8	ICAM1	[activation]
4838	CEBPB	SMARCA2	[activation]
4839	CCND2	RUNX1	[activation]
4840	ADAMTSL4	VCAM1	[activation]
4841	HSP90AB1	IRF3	[activation]
4842	GRB2	STAMBP	[activation]
4843	AR	GRB7	[activation]
4844	IFIH1	IKBKE	[inhibition]
4845	UGT1A1	UGDH	[activation]
4846	DAZAP2	CTNNB1	[activation]
4847	NOTCH1	EPN1	[activation]
4848	YWHAZ	CYR61	[activation;inhibition]
4849	MSH6	MAP2K1	[activation]
4850	ACTB	ANXA7	[activation]
4851	SRC	DLX4	[activation]
4852	PPARGC1A	NR3C1	[activation]
4853	ANAPC11	NKD2	[inhibition]
4854	GNA15	GRM4	[activation]
4855	MBP	MAPK15	[activation;inhibition]
4856	PRNP	RPL41	[activation]
4857	MAPKAPK2	CDC25A	[activation]
4858	GEM	TRIM23	[activation]
4859	MYC	ACACA	[activation;inhibition]
4860	SRGAP1	BMPR1B	[activation;inhibition]
4861	FBXW11	CDC25B	[inhibition]
4862	EIF2S1	RPA2	[activation]
4863	RHOB	NQO2	[activation]
4864	PFN1	ESR1	[activation]

4865	RFC2	DDX11	[activation]
4866	CDH2	PTPRO	[activation]
4867	ULK1	NTRK1	[activation]
4868	ATF2	TUBB	[activation]
4869	CDC37	TRAF6	[activation]
4870	MAP2K5	ASRGL1	[activation]
4871	RAC3	CIB1	[activation]
4872	MAPKAPK5	PLA2G4A	[activation]
4873	FBP2	STK11	[activation]
4874	GNAI1	TSHR	[activation]
4875	MRPS30	ICT1	[activation]
4876	MDM2	RAN	[activation]
4877	EIF4A3	HNRNPH2	[activation]
4878	KLRC1	PTPN6	[activation;inhibition]
4879	CBL	SHC1	[activation]
4880	GRB2	WDR77	[activation]
4881	GRB2	CCM2L	[activation]
4882	PARVB	STK4	[activation]
4883	ARHGEF7	HSPB1	[activation]
4884	PIAS2	C1QA	[activation]
4885	MAML2	NOTCH2	[activation]
4886	ITGA4	RAB10	[activation]
4887	MAPT	BIN1	[activation]
4888	ETS1	SMARCA2	[activation]
4889	PPP1CA	CDH1	[activation]
4890	SRPK2	CD53	[activation]
4891	CCDC88C	APP	[activation]
4892	ACTG1	MDM2	[activation]
4893	VWF	SOCS4	[inhibition]
4894	NCK2	LRRIQ3	[activation]
4895	AHNAK	GRB2	[activation]
4896	EFHD2	LRRK2	[activation]
4897	MAP1LC3B	SNCA	[activation]
4898	SMAD4	BTRC	[inhibition]
4899	EPOR	ATXN2L	[activation]
4900	EZR	DCC	[activation]
4901	IRS4	CRK	[activation]
4902	CDK2	POLD1	[activation]
4903	DOK2	SSSCA1	[activation]
4904	SPANXN4	SRPK1	[activation]
4905	ICT1	MRPL1	[activation]
4906	PTPN2	JAK3	[inhibition]
4907	BCAR1	TNS1	[activation]
4908	SMAD3	PAX8	[activation]
4909	OGFOD1	HLA-B	[activation]
4910	CTNNB1	IKBKB	[activation]
4911	NOTCH1	ANAPC7	[activation]
4912	LATS2	MOB1A	[activation]
4913	LRPAP1	POLA2	[inhibition]
4914	TGFBR1	OXSR1	[inhibition]
4915	DDX5	SMURF1	[inhibition]
4916	APP	CAMKV	[activation]
4917	RRAGA	RRAGD	[activation]
4918	EEF2K	BTRC	[activation]
4919	SRPK1	LCE3D	[activation]
4920	TP53	MNAT1	[activation]
4921	REV3L	MAD2L1	[activation]
4922	MAPK13	SMAD4	[activation]
4923	ICT1	MRPL3	[activation]
4924	GALNT12	PIK3CA	[activation]
4925	GAS7	ABI1	[activation]
4926	CSNK1D	MDM2	[activation]
4927	KCTD20	PPP1CC	[inhibition]
4928	NFATC2	PIN1	[activation]
4929	PXN	NEDD9	[activation]
4930	STK3	VAPA	[activation]
4931	TP53	TTLL5	[activation]
4932	HCFC1	HSPA1A	[inhibition]
4933	PPP1CC	C9orf50	[inhibition]
4934	PLCG2	GRB2	[activation]
4935	SLC25A6	MAP1LC3B	[activation]
4936	UBC	F12	[activation]
4937	GNAI2	MCF2	[activation]
4938	ARNTL	SIRT1	[activation;inhibition]
4939	SNRPA1	ARF6	[activation]
4940	DNAJA1	NFKB2	[inhibition]

4941	TWIST1	HOXA5	[activation]
4942	GSK3B	PPARGC1A	[activation]
4943	ARAF	HSPB1	[activation]
4944	SETD8	LEF1	[activation]
4945	DHX34	RPS6KA6	[activation]
4946	RAD21	CTSG	[activation]
4947	HSF1	SUMO1	[activation]
4948	SKP2	CDC20	[inhibition]
4949	LCK	PRKACA	[activation]
4950	HMBOX1	CCNG1	[activation]
4951	EP300	GOLGA2	[activation]
4952	RUNX1	HIPK2	[activation]
4953	CDKN2A	CDC14B	[inhibition]
4954	FYN	HEXA	[activation]
4955	TGFB1	BMP3	[activation]
4956	TFRC	CD9	[activation]
4957	SMAD9	DNAJA3	[inhibition]
4958	LYN	GTPBP3	[activation]
4959	PRKAR1B	WNK1	[activation]
4960	NFKBIA	RPS6KA1	[activation]
4961	TAF8	MEF2A	[activation]
4962	SRPK1	PPIA	[activation]
4963	BLK	BCAS2	[activation]
4964	SFXN1	UBA5	[activation]
4965	HSP90AA1	JAK2	[activation]
4966	RGS2	LRRK2	[activation]
4967	PAXIP1	PRPSAP1	[activation]
4968	KMT2E	CDK1	[activation]
4969	PIN1	ITGA4	[activation]
4970	HIST3H3	SFMBT1	[activation]
4971	LARS	YWHAZ	[activation]
4972	GADD45A	DNAJC13	[activation;inhibition]
4973	RCHY1	F7	[activation;inhibition]
4974	TUB	JAK2	[activation]
4975	PER3	PER1	[inhibition]
4976	TFRC	EGFR	[activation]
4977	PAXIP1	LARP1	[activation]
4978	APP	MDM2	[activation]
4979	CCNH	RPA1	[activation]
4980	NANS	TLR2	[activation]
4981	GRIP1	EFNB2	[activation]
4982	CDC42BPB	RIPK1	[activation]
4983	THOP1	STK11	[activation]
4984	PTMA	HSPA1A	[inhibition]
4985	HOXB4	EP300	[activation]
4986	SUMO3	BUB3	[inhibition]
4987	GRIP1	EFNB1	[activation]
4988	PFDN1	INPP5K	[activation]
4989	CSNK1D	CCNA1	[activation]
4990	CTSS	PLCG1	[activation]
4991	PARD6B	CRB3	[activation]
4992	TYK2	DNM2	[activation]
4993	DDR2	SRC	[activation]
4994	FYN	FAS	[activation]
4995	RNF4	UBE2E3	[activation]
4996	PAK2	SH3KBP1	[activation]
4997	MLLT4	YWHAZ	[activation]
4998	DENND2C	LYN	[activation;inhibition]
4999	CDC27	HOXC10	[activation]
5000	RAN	TNPO1	[activation]
5001	PRKACB	RCAN1	[activation]
5002	EPHB1	GRB7	[activation]
5003	CSNK1A1	CTNNB1	[activation]
5004	PAXIP1	SMC2	[activation]
5005	CBL	OSTF1	[inhibition]
5006	SNTA1	KCNA4	[activation]
5007	AKAP7	PRKACA	[activation]
5008	NCOA1	PRMT2	[activation]
5009	PLCG1	SHB	[activation]
5010	ACTG1	ANXA5	[activation]
5011	BCL2L11	AURKA	[activation]
5012	DOCK8	CDC5L	[activation]
5013	ACTB	ACTR2	[activation]
5014	SRPK2	TLE4	[activation]
5015	TNFRSF1B	TRAF6	[activation]
5016	HDGF	MAPK14	[activation]

5017	WBSCR16	ICT1	[activation]
5018	CDK2	CDK3	[activation]
5019	U2AF2	MDM2	[activation]
5020	NOS1	HMOX1	[activation]
5021	INS	LRP2	[activation]
5022	LAS1L	MYC	[activation]
5023	ELAVL1	RUNX1	[activation]
5024	CUL2	BCL2L11	[activation]
5025	FYN	IL7R	[activation]
5026	ITGB1	EPS8	[activation]
5027	BTK	TLR9	[activation]
5028	CBL	SH3BP2	[activation]
5029	PPP2R2D	PPME1	[activation]
5030	ASAP1	ARAP3	[activation]
5031	CDC25B	KRT35	[activation]
5032	MDM2	HNRNPL	[activation]
5033	DCC	RPS24	[activation]
5034	OBSL1	DKC1	[activation]
5035	PRPSAP1	PRPS1	[inhibition]
5036	FGFR2	MTA3	[activation]
5037	HMGB1	PRKCA	[activation]
5038	IL3	BCR	[activation]
5039	SIRPA	JAK2	[activation]
5040	YTHDF2	EPAS1	[inhibition]
5041	ENO1	TNS3	[activation]
5042	IGF1R	PTPN1	[activation]
5043	CSK	YTHDC1	[activation]
5044	FZR1	EYA1	[activation]
5045	MDM2	DAAM2	[activation]
5046	SERPINB1	ELANE	[inhibition]
5047	PPP2R4	AURKB	[activation]
5048	CAV1	POU3F3	[activation]
5049	LIN37	E2F4	[activation;inhibition]
5050	STAM	SORT1	[activation]
5051	MAPK9	ACP5	[activation]
5052	RHEB	RPS6KB1	[activation]
5053	ITGA4	RAC2	[activation]
5054	ICAM1	SRP14	[activation]
5055	MIF	STRN4	[activation]
5056	HSP90AA1	CDK15	[activation]
5057	SPTAN1	EVL	[activation]
5058	CACNA1S	YWHAQ	[activation]
5059	EIF4G3	LGR4	[activation]
5060	CAP1	ACTG1	[activation]
5061	MATK	PXN	[activation]
5062	ABL2	MAP3K10	[activation;inhibition]
5063	PRKCD	MACF1	[activation]
5064	GORASP2	CDC23	[activation]
5065	MAPRE1	TROAP	[activation]
5066	THSD7A	SKIL	[activation]
5067	FAN1	PCNA	[activation]
5068	NFE2L2	CDKN1A	[activation]
5069	AREG	MMP9	[activation]
5070	MAP4K5	NCK1	[activation]
5071	CPNE6	MAPK11	[activation]
5072	ADAP1	GADD45A	[activation]
5073	ERCC6L	CDC5L	[activation]
5074	ATP1A1	CFL1	[activation]
5075	AURKB	CDCA8	[activation]
5076	GRB2	ARHGAP12	[activation]
5077	RPL26	MTOR	[activation]
5078	GNG3	GNB1	[activation]
5079	FBXO6	EIF4A1	[activation]
5080	FLI1	UBE2I	[activation]
5081	VCAM1	MYL12B	[activation]
5082	PRKRA	SHC1	[activation]
5083	HSPA8	PARD3	[activation]
5084	THBS1	CTSG	[activation]
5085	EIF2S1	LYAR	[activation]
5086	PAG1	PRKCB	[activation]
5087	ELMO1	TRIM54	[activation]
5088	CALM1	STIM2	[activation]
5089	PIK3R1	INPP4A	[activation]
5090	AKT3	PRKCZ	[activation]
5091	PTGIR	PDZK1	[activation]
5092	ECT2	PPP2CA	[activation]

5093	CD4	LAT	[activation]
5094	PAK1	BAIAP2	[activation]
5095	HLA-B	TTLL12	[activation]
5096	RPS6KB1	EIF4EBP1	[activation]
5097	EIF1B	RPA3	[activation]
5098	PTPRT	PTPRN	[activation]
5099	MDM2	FARSA	[activation]
5100	SRF	ELK1	[activation]
5101	RAVER1	VCL	[activation]
5102	PIK3R1	PTK2B	[activation]
5103	APPL1	DYSF	[activation]
5104	PIK3R1	ZC4H2	[activation]
5105	THRB	NSD1	[activation]
5106	ECT2	RHOG	[activation]
5107	IRS4	CCDC8	[activation]
5108	KCNA5	PRKCQ	[activation]
5109	HSPB1	MSL1	[activation]
5110	E2F5	CREBBP	[activation]
5111	DYRK2	HIST3H3	[activation]
5112	FYB	PLCG1	[activation]
5113	HSP90AA1	DAPK1	[activation]
5114	CYTH3	INSR	[activation]
5115	SNX17	GRB2	[activation]
5116	PLXNA2	UBAP2L	[activation]
5117	GRB2	HSP90AB1	[activation]
5118	TP53	NUAK1	[activation]
5119	CYCS	ITGA4	[activation]
5120	MLH1	ANXA2	[activation]
5121	SKP1	FBXL21	[inhibition]
5122	TERF1	YWHAG	[activation]
5123	IDE	GCG	[activation]
5124	HIST3H3	JAK2	[activation]
5125	TIMM50	EGFR	[activation]
5126	GRB2	RPL27	[activation]
5127	RASA1	HCK	[activation]
5128	SELL	GRB2	[activation]
5129	FBXO25	ACTG1	[activation]
5130	ACTA1	AIF1	[activation]
5131	CRK	REPS1	[activation]
5132	NFKBIA	UBE2E3	[activation]
5133	PTPN11	STAT1	[activation]
5134	ADRA1B	ADRA1A	[activation]
5135	CHUK	TAX1BP1	[activation]
5136	COCH	COL4A1	[activation]
5137	PYCR1	VCAM1	[activation]
5138	SRPK2	DGCR8	[activation]
5139	XCL2	NOTCH2NL	[activation]
5140	ACTA1	RPS6KB1	[activation]
5141	ILK	RPL13	[activation]
5142	DCTN1	MAPT	[activation]
5143	PTPN18	ABL2	[activation]
5144	PUM1	EIF5	[activation]
5145	DDAH2	RPS6KA1	[activation]
5146	MYC	IQCE	[activation]
5147	YWHAG	ZAK	[activation]
5148	DLGAP1	CRK	[activation]
5149	SGK1	IGHG1	[activation]
5150	EPHA3	RUFY2	[activation]
5151	AURKB	SGOL1	[activation]
5152	RND3	SKP2	[activation]
5153	EIF2B4	EIF2B5	[activation]
5154	BRD7	DVL1	[activation]
5155	SRC	SH3KBP1	[activation]
5156	TNFSF11	AKT1	[activation]
5157	CALM1	HTR2C	[activation]
5158	RABGEF1	MAPK1	[activation]
5159	RAP1B	DDAH2	[activation]
5160	FRAT2	EGFR	[activation]
5161	HSP90AA1	SRPK3	[activation]
5162	RAF1	RSU1	[activation]
5163	SH2B1	MET	[activation]
5164	SRC	STX17	[activation]
5165	H2AFX	ARRB1	[activation]
5166	PAK2	AK2	[activation]
5167	HIST2H2AC	AURKB	[activation]
5168	LEF1	SMAD7	[activation]

5169	IGHG1	FCGR3B	[activation]
5170	ADAMTS2	PIK3R5	[activation]
5171	MAP2K4	TRIB1	[activation]
5172	SCGB1A1	LMBR1L	[activation]
5173	PIM1	HIST3H3	[activation]
5174	HCLS1	ACTR3	[activation]
5175	HSPA1L	GRB2	[activation]
5176	DESI1	FGF7	[activation]
5177	EIF3L	CD81	[activation]
5178	CDH1	CFTR	[activation]
5179	GAPDH	PLD2	[activation]
5180	SUV39H1	DBF4B	[activation]
5181	EIF4A1	EIF4G1	[activation]
5182	EMILIN1	SRPK1	[activation]
5183	PRIM1	TP53	[activation]
5184	CCT8	SMURF1	[inhibition]
5185	SH2D2A	ERBB2	[activation]
5186	SHC1	PLCG2	[activation]
5187	MYC	LATS1	[inhibition]
5188	ETFB	ADRB2	[activation]
5189	MDM2	GNAS	[activation]
5190	CLINT1	AURKB	[activation]
5191	RYR1	HOMER2	[activation]
5192	FLOT1	FRS2	[activation]
5193	CD19	BTK	[activation]
5194	SMAD4	RHOD	[activation]
5195	STRA13	FANCL	[activation]
5196	NAT9	LUC7L2	[activation]
5197	IRAK1	IKBKB	[activation;inhibition]
5198	MET	PTK6	[activation]
5199	HIST1H2AD	VCAM1	[activation]
5200	CASP9	ILK	[activation]
5201	NCOA1	HNF4A	[activation]
5202	COL6A1	PDGFB	[activation]
5203	TOP2A	PRKDC	[activation]
5204	CCNA2	PGR	[activation]
5205	TNF	LYN	[activation]
5206	MYOG	MAPK3	[activation]
5207	KAT2A	TP53	[activation]
5208	CRTC1	EP300	[activation]
5209	YWHAH	KIF23	[activation]
5210	PPP1R14A	YWHAZ	[activation]
5211	PICALM	EHD2	[activation]
5212	ALK	MAP3K4	[activation]
5213	HLA-B	FKBP3	[activation]
5214	MEIS1	TLX3	[activation]
5215	HAUS7	ATF4	[activation]
5216	NUP62	NCAPG	[activation]
5217	MEF2A	ASCL1	[activation]
5218	CHUK	MAP3K11	[activation]
5219	ERCC1	UBL7	[activation]
5220	IKBKB	EGLN3	[activation]
5221	TIRAP	MPP3	[activation]
5222	CMTM6	DUSP10	[inhibition]
5223	WIZ	EHMT1	[activation]
5224	NXF1	PXN	[activation]
5225	AK2	MDM2	[activation]
5226	WNK1	MPHOSPH9	[activation]
5227	BMP4	TWSG1	[activation]
5228	TP53	NEIL3	[activation]
5229	TAF1	CLIP4	[activation]
5230	ESR1	MVP	[activation]
5231	FYN	TRPC6	[activation;inhibition]
5232	CDK4	HSPA8	[inhibition]
5233	GSK3B	RSU1	[inhibition]
5234	RGS4	EGFR	[activation;inhibition]
5235	ANGPTL4	SMARCA4	[activation]
5236	DNAJA1	SHC1	[inhibition]
5237	CKS1B	FZR1	[inhibition]
5238	PPBP	EFNA1	[activation]
5239	PAK2	PPP2R2B	[activation]
5240	ARRDC4	ARRB2	[inhibition]
5241	LRRK2	ATP5O	[activation]
5242	CYTH1	PRKCD	[activation]
5243	CD3D	KCNA3	[activation]
5244	PAK4	YWHAG	[activation]

5245	NR2C2	TRAF2	[activation]
5246	NEDD9	FYN	[activation]
5247	ZFYVE27	APP	[activation]
5248	GAPDH	CCNB1	[inhibition]
5249	HCK	HSP90AA1	[activation]
5250	RCHY1	MDM2	[activation;inhibition]
5251	ZBED1	PRKAA1	[inhibition]
5252	NAV2	RRAS	[activation]
5253	GABARAPL2	HSPA4	[activation]
5254	NRXN2	MACF1	[activation]
5255	IGHG1	ELN	[activation]
5256	NCK1	MET	[activation]
5257	PPP2R1A	SGOL2	[inhibition]
5258	AZGP1	STK4	[activation]
5259	EIF4A3	BOLA2	[activation]
5260	YWHAE	MAP3K2	[activation]
5261	MLH1	STAP2	[activation]
5262	CDK18	CDK5	[activation]
5263	JAK3	CDK1	[activation]
5264	EHMT2	PML	[activation]
5265	PIK3R1	EXTL3	[activation]
5266	CSF2RB	JAK1	[activation]
5267	TRAF2	IQUB	[activation]
5268	FBXO4	TERF1	[activation]
5269	IGFBP5	FHL2	[inhibition]
5270	CDK11B	YWHAB	[activation]
5271	SCYL2	CDC5L	[activation]
5272	STXBP6	STX1A	[activation]
5273	TCOF1	UBTF	[activation]
5274	PRKDC	ASB5	[activation]
5275	TFCP2	MAPK1	[activation]
5276	CTBP1	BMPR2	[activation]
5277	COL4A2	SERPINE2	[inhibition]
5278	IKBKE	DDX58	[inhibition]
5279	USP33	RALA	[activation]
5280	RSBN1	PAX8	[activation]
5281	SMAD4	RHOG	[activation]
5282	CCDC151	SUV39H1	[activation]
5283	WWTR1	DVL1	[activation]
5284	CTNNB1	SOX6	[activation]
5285	TIRAP	TICAM1	[activation]
5286	SDC2	LAMA3	[activation]
5287	NGB	GNAO1	[activation;inhibition]
5288	ETV5	AMH	[activation]
5289	SLC9A1	ARRB1	[activation]
5290	CAPN2	CAPNS1	[activation]
5291	LYN	SKAP2	[activation]
5292	EGFR	RIN2	[activation]
5293	MYC	MFHAS1	[activation]
5294	ILF2	MRE11A	[activation]
5295	PNRC2	SPAG9	[inhibition]
5296	NFAM1	SYK	[activation]
5297	FYN	DOCK3	[activation]
5298	ATM	MTA3	[activation]
5299	IGF2	NOV	[activation]
5300	DDX56	MRPL43	[activation]
5301	ETS1	AR	[activation]
5302	PLEKHM1	MAPK6	[activation]
5303	FAM105B	FAM168A	[activation]
5304	AGPAT6	NXF1	[activation]
5305	ZNF106	WNK1	[activation]
5306	UBE2I	ATF3	[activation]
5307	TP53	MYL9	[activation]
5308	CAD	YWHAZ	[activation;inhibition]
5309	ATR	RPA3	[activation]
5310	CAMK2D	FAM171B	[activation]
5311	HYOU1	ESR2	[activation]
5312	ROBO2	NR1H2	[activation]
5313	PRKACA	GPR161	[activation]
5314	KCNK15	YWHAB	[activation]
5315	TRAF1	TNFSF9	[activation]
5316	ASB2	HMGB1	[inhibition]
5317	CSH1	SMAD4	[activation]
5318	MYC	IQGAP3	[inhibition]
5319	HSP90AB1	NXF1	[activation]
5320	HES6	CREBBP	[activation]

5321	CAMLG	TRAF5	[activation]
5322	FBXO5	NXF1	[activation]
5323	TBXA2R	RAB11A	[inhibition]
5324	APP	DNAJC4	[inhibition]
5325	PRDX2	PPP2R1A	[inhibition]
5326	UGT1A7	PRKCE	[activation]
5327	LCK	LIME1	[activation]
5328	SRPK1	SPAG11B	[activation]
5329	RAP1GAP	BRAF	[activation]
5330	HSP90AA1	PRKCB	[activation]
5331	CSNK1E	FARP2	[activation]
5332	KCNA2	PTK2B	[activation]
5333	GAPDH	GADD45A	[activation]
5334	ARHGAP1	RAC1	[activation]
5335	RAC3	RARA	[activation]
5336	TNFRSF13B	TRAF6	[activation]
5337	GSTK1	RAB3GAP2	[activation;inhibition]
5338	GRB2	HTRA1	[activation]
5339	ARFGEF2	SNW1	[activation]
5340	TFRC	LAPTM4A	[activation]
5341	STAT5A	BRCA1	[activation]
5342	HRAS	TLR9	[activation]
5343	BUB3	ZNF207	[inhibition]
5344	TRIM69	ASNS	[activation]
5345	CHRDL2	BMP4	[activation]
5346	CDK2	CDK13	[activation]
5347	NR4A1	HSPH1	[inhibition]
5348	SUMO1	EPAS1	[activation]
5349	PTK2	PTAFR	[activation]
5350	OLIG2	EP300	[activation]
5351	AURKB	MAPRE2	[activation]
5352	SPRR1A	EGFR	[activation]
5353	PANX1	CASP3	[activation]
5354	BAAT	CTNNA1	[activation]
5355	OTUD4	ILK	[activation]
5356	RPA3	HNRNPL	[activation]
5357	ADRB2	RHOD	[activation]
5358	EIF2B2	C9orf72	[activation]
5359	PLG	EP300	[activation]
5360	SNAP25	APP	[activation]
5361	HES1	JAK2	[activation]
5362	GRB2	PAG1	[activation]
5363	FOXA2	GSC	[activation]
5364	EP300	HERC1	[activation]
5365	RXRA	ACVR1	[inhibition]
5366	ENO2	HSF1	[activation]
5367	ACVR2A	TGFBR3	[activation]
5368	ACTB	RPA3	[activation]
5369	PRKAA1	YWHAE	[inhibition]
5370	DOK4	RET	[activation]
5371	MAP2K3	SPAG9	[inhibition]
5372	HSPA1A	PRKCA	[activation]
5373	GNAO1	OPRD1	[activation]
5374	JUNB	C19orf68	[activation]
5375	RCHY1	BIRC3	[activation;inhibition]
5376	TRAF2	BANP	[activation]
5377	ERBB3	PIK3R2	[activation]
5378	NOTCH2NL	WDR25	[activation]
5379	PTGES3	NR3C1	[activation]
5380	IGHM	OR5H6	[activation]
5381	TRAF2	NIF3L1	[activation]
5382	HMGA2	XRCC6	[activation]
5383	GABRR1	BCR	[activation]
5384	CAPN1	FANCA	[activation]
5385	TMSB4Y	ACTG1	[activation]
5386	EP300	MAGED1	[activation]
5387	ERBB4	CFL1	[activation]
5388	MAPRE2	PAXIP1	[activation]
5389	HOXC6	FGFR2	[activation]
5390	ISL2	LHX3	[activation]
5391	PPP2R5A	TP63	[activation]
5392	HDAC3	CCND1	[activation]
5393	VCAM1	MARS	[activation]
5394	GUCY2D	FYN	[activation]
5395	HSPA8	LGALS3BP	[inhibition]
5396	GNAQ	VIPR1	[activation]

5397	PML	FOXO1	[activation]
5398	CLIP4	CMYA5	[activation]
5399	VCAM1	PRKDC	[activation]
5400	CXCL1	HIPK2	[activation]
5401	TRIM28	IRF1	[activation]
5402	NCK2	FAM53C	[activation]
5403	F2R	MMP1	[activation]
5404	MCM7	MYC	[activation]
5405	ATM	PPP2R4	[activation]
5406	CCR2	CCR5	[activation]
5407	HSPH1	VAV2	[activation]
5408	VAV1	INSR	[activation]
5409	TLE4	PPM1B	[inhibition]
5410	S100A4	TP53	[activation]
5411	INHBA	INHBB	[activation]
5412	VAC14	PPP2CA	[inhibition]
5413	PPP3CC	BCL2	[inhibition]
5414	CSF1R	RASA1	[activation]
5415	HDAC1	RELA	[activation]
5416	BHLHE40	CALML3	[activation]
5417	TOLLIP	FAM115A	[activation]
5418	CCNA2	CDH2	[activation]
5419	TNF	HNRNPD	[activation]
5420	WDR6	MAP2K7	[activation]
5421	UBTF	IRS1	[activation]
5422	CERS2	ARHGEF7	[activation]
5423	PTPN5	MAPK1	[inhibition]
5424	FOS	SPI1	[activation]
5425	CASP7	PLA2G4B	[activation]
5426	GPHN	PCK1	[activation]
5427	GRB2	SNTA1	[activation]
5428	RPS6	PCK1	[activation]
5429	BARD1	GNPAT	[activation]
5430	RAC3	NCL	[activation]
5431	TNFRSF1A	SYK	[activation]
5432	GAB2	PTPN6	[activation]
5433	TOLLIP	DIABLO	[activation]
5434	HSD17B4	GABARAP	[activation]
5435	ITPR2	CCDC8	[inhibition]
5436	OBSL1	CDK1	[activation;inhibition]
5437	S1PR5	GNAI1	[activation]
5438	KIT	FYN	[activation]
5439	NCOA2	TBK1	[activation]
5440	PDGFRA	SHF	[activation]
5441	RPA2	MYO1F	[activation]
5442	MDM2	NME1	[activation]
5443	CREB5	ATF2	[activation]
5444	CDC42	SH3D19	[activation]
5445	PPP3CB	MYOZ2	[inhibition]
5446	TP53	RPL21	[activation]
5447	PRKACB	TRAP1	[activation]
5448	COL2A1	PDGFA	[activation]
5449	GATA1	ZZZ3	[activation]
5450	SMAD3	TRIB3	[inhibition]
5451	TERT	NCL	[activation]
5452	PGM3	LRRK2	[activation]
5453	EIF2AK2	NLRP3	[inhibition]
5454	SERPINB6	CEP170P1	[inhibition]
5455	FAM71C	NOTCH2NL	[activation]
5456	GPR56	ADRB2	[activation]
5457	FGB	MIS12	[activation]
5458	LDB2	ISL1	[activation]
5459	MARK3	CAPZB	[activation]
5460	NR3C1	RAN	[activation]
5461	RANBP2	NLK	[activation]
5462	SOS1	SHC1	[activation]
5463	RAD21	PRKDC	[activation]
5464	PXK	HNRNPA0	[activation]
5465	SMC4	IKBKG	[activation]
5466	NSD1	ZNF496	[activation]
5467	PRIM2	YWHAZ	[activation]
5468	CCNG1	PLEKHA4	[activation]
5469	MYD88	PELI1	[activation;inhibition]
5470	SRC	CD33	[activation]
5471	HSP90AB1	PPARG	[activation]
5472	PIK3R1	TPSB2	[activation]

5473	CRK	HSPA1L	[activation]
5474	TBK1	TLR3	[activation]
5475	APP	CSNK1G1	[activation]
5476	HLA-E	KLRC2	[activation]
5477	SOCS3	NFKB1	[activation;inhibition]
5478	ATF2	CTTN	[activation]
5479	PRKCA	IBTK	[activation]
5480	ISYNA1	PPP2CA	[inhibition]
5481	ABI1	SOS2	[activation]
5482	TP53	E2F1	[activation]
5483	ANXA3	ATF2	[activation]
5484	AREG	CCND3	[activation]
5485	EIF2AK2	TRAF2	[activation]
5486	MAPK1	PPM1A	[activation]
5487	APPL1	HSPB1	[activation]
5488	APP	CASP4	[activation]
5489	IRS1	CDH1	[activation]
5490	FES	IRS2	[activation]
5491	LCE1B	RGS20	[activation;inhibition]
5492	ATP5F1	SPP1	[activation]
5493	TBP	TAF1	[activation]
5494	UBE2M	TP53	[activation]
5495	SP1	CD2	[activation]
5496	PIN1	NFKBIA	[activation]
5497	KMT2B	CRK	[activation]
5498	DDX18	INADL	[activation]
5499	CFHR5	C3	[activation;inhibition]
5500	IGHG1	SERPIND1	[inhibition]
5501	FASLG	PIK3R1	[activation]
5502	RAPGEF2	KIF5B	[activation]
5503	PPP1CA	RPRD2	[inhibition]
5504	PKN2	CASP3	[activation]
5505	ZFC3H1	MYB	[activation]
5506	GABARAPL2	CDC5L	[activation]
5507	ACTG1	SCIN	[activation]
5508	CYFIP1	ABI1	[inhibition]
5509	KMT2A	SKP2	[activation]
5510	EGFR	TTI1	[activation]
5511	SMAD2	DGKA	[activation]
5512	SLC8A1	PPP3CB	[inhibition]
5513	STK4	FLG	[activation]
5514	SYK	MAP4K1	[activation]
5515	CDH18	CDH6	[activation]
5516	CXorf27	MYOG	[activation]
5517	APP	CAMKK2	[activation]
5518	EIF2S1	VCAM1	[activation]
5519	DNA2	RPA3	[activation]
5520	ARRB2	SPIN3	[inhibition]
5521	NSL1	LRRK2	[activation]
5522	ADCY5	PRKCZ	[activation]
5523	PELI2	IRAK4	[activation;inhibition]
5524	E2F3	CREBBP	[activation]
5525	GNL3	SRPK3	[activation]
5526	APP	LDLRAP1	[activation]
5527	CPE	BDNF	[activation]
5528	FCHSD1	DNM2	[activation]
5529	MAP2K1	AURKA	[activation]
5530	SERPINB13	PRTN3	[inhibition]
5531	NUDT5	TRAF6	[activation]
5532	PPP1CA	AKT1	[activation;inhibition]
5533	C7orf50	PAXIP1	[activation]
5534	MAP3K12	RGS1	[activation;inhibition]
5535	PTMS	EP300	[activation]
5536	MAGEA1	HLA-B	[activation]
5537	TGFBR1	TSSK4	[activation]
5538	KRIT1	NXF1	[activation]
5539	TP53	IFRG15	[activation]
5540	RAB2A	RPA1	[activation]
5541	COMMD10	NFKB1	[activation]
5542	YWHAQ	KIF23	[activation]
5543	MELK	MBP	[activation]
5544	CRTC2	PPP3CA	[activation]
5545	PPP1CA	ENO2	[activation]
5546	GSN	MAP1S	[activation]
5547	HDAC1	PITX2	[activation]
5548	TGFB3	CTGF	[activation]

5549	APC	PCNA	[inhibition]
5550	LSM3	ICAM1	[activation]
5551	HIST1H4A	PRMT7	[activation]
5552	PHLDA3	VAMP7	[activation]
5553	YWHAB	LSR	[activation]
5554	APOC1	IGHG1	[activation]
5555	PXN	ILK	[activation]
5556	YWHAQ	SSH1	[activation]
5557	FOS	EEF1D	[activation]
5558	HSPB1	GATA1	[activation]
5559	SRF	SUMO1	[activation]
5560	APP	MPP3	[activation]
5561	CSNK1E	USP9X	[activation]
5562	PIK3R1	ZAP70	[activation]
5563	PSD	GNAQ	[activation]
5564	NCK1	KIT	[activation]
5565	LLGL1	PRKCZ	[activation]
5566	CENPJ	RELA	[activation]
5567	FBXO43	CDC27	[inhibition]
5568	BCL6	FOXM1	[activation]
5569	A2M	TNFRSF14	[inhibition]
5570	GRB2	GCDH	[activation]
5571	NR1H4	RXRA	[inhibition]
5572	SP1	GABPA	[activation]
5573	USH1C	ANKS4B	[activation]
5574	TYK2	VAV1	[activation]
5575	GDF5	ACVR1	[activation]
5576	NCK1	MAP4K1	[activation]
5577	HSPA8	MDM2	[activation]
5578	PPP1R9A	RPS6KB1	[activation]
5579	INSR	SOCS6	[inhibition]
5580	PMS1	MLH1	[activation]
5581	LCE3E	RGS20	[activation;inhibition]
5582	MYH2	PTK2B	[activation]
5583	PIAS1	GLUL	[inhibition]
5584	G3BP2	RPF2	[activation]
5585	CLTCL1	EGFR	[activation]
5586	SMAD9	CTR9	[activation]
5587	DYSF	ANXA1	[activation]
5588	IRAK3	ECM1	[inhibition]
5589	LRRK2	PRPF6	[activation]
5590	CCDC8	SMC2	[activation]
5591	YAP1	YWHAH	[activation]
5592	YAP1	CDH3	[activation]
5593	ESR1	SUV420H1	[activation]
5594	NCDN	EFHC2	[activation]
5595	RNF165	SMAD7	[activation]
5596	RAC1	PRKCI	[activation]
5597	CXCR4	B2M	[activation]
5598	SRPK1	CAMK2D	[activation]
5599	EIF2AK2	CASP7	[activation]
5600	DIAPH3	MYC	[activation]
5601	LYN	HSP90AB1	[activation]
5602	BLZF1	GEM	[activation]
5603	MT-RNR2	BCL2L1	[activation]
5604	EIF3F	SGK1	[activation]
5605	HSPB1	MME	[activation]
5606	STAT1	E2F1	[activation]
5607	FOXJ1	RPUSD1	[inhibition]
5608	MAPRE3	AURKB	[activation]
5609	GNAI1	PGR	[activation]
5610	RPA1	HNRNPA0	[activation]
5611	TWF2	CAPZA1	[activation]
5612	ASAP2	PTK2B	[activation]
5613	RAC1	PAK3	[activation]
5614	CAPZB	GABARAPL2	[activation]
5615	PTPN6	PTPRC	[activation]
5616	FYN	ABL2	[activation]
5617	TICAM1	TRAF5	[activation]
5618	CDC23	BCAS3	[activation]
5619	CCL1	CCR8	[activation]
5620	TRAF6	IL1RL1	[activation]
5621	NEK8	HSP90AB1	[activation]
5622	KCNC1	KCNC2	[activation]
5623	RAD51	ATRX	[activation]
5624	PPP1R14A	CSNK1G3	[activation]

5625	AREG	S100A5	[activation]
5626	COL1A1	FGF7	[activation]
5627	CTNNB1	CCND1	[activation]
5628	SCAP	INSIG1	[activation]
5629	MAVS	ECSIT	[activation]
5630	B4GALT1	CDK11A	[activation]
5631	DET1	MDM2	[activation]
5632	JAK2	PPP2R1B	[activation]
5633	MLH1	VAMP8	[activation]
5634	RFXANK	BSG	[activation]
5635	CDC25C	YWHAZ	[activation]
5636	PIK3CA	TICAM1	[activation]
5637	HRG	CHRD	[inhibition]
5638	VASN	PSEN1	[activation]
5639	YWHAE	HSPA8	[inhibition]
5640	RASSF9	DVL3	[activation]
5641	EP300	PDHX	[activation]
5642	HOXD10	CREBBP	[activation]
5643	FBXW11	RELA	[inhibition]
5644	FRS3	RFX6	[activation]
5645	FBXW4	PPP2R1A	[inhibition]
5646	NUP155	HLA-B	[activation]
5647	NFKBIA	TBK1	[activation]
5648	SKP2	RAG2	[activation]
5649	PMAIP1	GSK3B	[activation;inhibition]
5650	PRKCG	DDX58	[activation]
5651	CREBBP	IFNAR2	[activation]
5652	EP300	DEK	[activation]
5653	EGFR	TRIM29	[activation]
5654	NXPH3	SRC	[activation]
5655	SHC1	FCGR1A	[activation]
5656	CDK5R1	NOTCH2NL	[activation]
5657	TMEM147	CASP4	[activation]
5658	RARA	SUV39H1	[activation]
5659	YTHDF1	RPA2	[activation]
5660	PTPN7	APP	[activation]
5661	PPP2R1A	KLHL15	[inhibition]
5662	GABBR1	GRB2	[activation]
5663	PAXIP1	NCAPG	[activation]
5664	ECSIT	SMURF1	[inhibition]
5665	CAD	CASP3	[activation]
5666	PIP5K1A	CADPS	[activation]
5667	CDKN1A	CDK5R1	[activation]
5668	RAF1	INSR	[activation]
5669	SH3RF3	RAC1	[activation]
5670	IDE	IGF1	[activation]
5671	SMAD4	CD59	[inhibition]
5672	RB1	HIST1H4A	[activation;inhibition]
5673	MAVS	TMEM173	[activation]
5674	HSPA4	HIF1A	[activation]
5675	OPRD1	PRKCD	[activation]
5676	APP	PDPK1	[activation]
5677	SFXN1	HLA-B	[activation]
5678	JMJD6	RSPO2	[inhibition]
5679	ACVR1B	HSP90AA1	[activation]
5680	JUN	STAT1	[activation]
5681	YWHAG	SSFA2	[activation]
5682	STK4	IQGAP2	[activation]
5683	EIF2AK2	CDC42	[activation]
5684	PRKCB	RASGRP3	[activation]
5685	GRB2	DOCK1	[activation]
5686	APC	ANKRD17	[inhibition]
5687	USP7	RELA	[activation]
5688	XPO1	EGFR	[activation]
5689	AR	PIAS2	[activation]
5690	PTPN11	ITGB3	[activation]
5691	VCAM1	STAT5B	[activation]
5692	SMAD1	RAC2	[activation]
5693	FBXL5	SMURF1	[inhibition]
5694	MSANTD3	CDH1	[activation]
5695	HDAC4	CTNNB1	[activation]
5696	TP53	DNMT1	[activation]
5697	PTEN	USP10	[activation;inhibition]
5698	RIF1	TDGF1	[activation]
5699	SEH1L	RPA2	[activation]
5700	NAV1	APC	[inhibition]

5701	PRKDC	SP1	[activation]
5702	ANAPC1	HECW2	[activation]
5703	PPP1CC	JAK2	[inhibition]
5704	RARA	NSD1	[activation]
5705	WAS	NCK2	[activation]
5706	NXF1	COMMD9	[activation]
5707	CEBPB	SRF	[activation]
5708	GNB2	GNAI2	[activation]
5709	APOL5	FYN	[activation]
5710	NRIP1	HDAC1	[activation]
5711	LCP2	PTPRC	[activation]
5712	ICAM1	RPS28	[activation]
5713	WASF2	PRKACA	[activation]
5714	ICT1	PUSL1	[activation]
5715	CYTH1	ARF6	[activation]
5716	CLEC4A	PTPN6	[activation;inhibition]
5717	RPA2	ACTB	[activation]
5718	CBL	LTK	[activation]
5719	KAT2B	SERTAD1	[activation]
5720	CTBP2	ACTG1	[activation]
5721	PTPRD	EGFR	[activation]
5722	STAT3	GNB2L1	[activation]
5723	MAP2K3	ALDOC	[activation]
5724	MORC3	KPNA2	[activation]
5725	SRF	BARX2	[activation]
5726	RTEL1	RPA3	[activation]
5727	WNK1	ZYX	[activation]
5728	PPP2R1A	PPP2R2C	[activation]
5729	PRKCB	IBTK	[activation]
5730	KCNG1	HSP90AB1	[activation]
5731	HLA-B	SMAP	[activation]
5732	NOXO1	NOXA1	[activation]
5733	GK	RXRA	[inhibition]
5734	PRKCE	ITGB1	[activation]
5735	MYO1C	RPA1	[activation]
5736	CDC27	FBXO5	[inhibition]
5737	CDK6	TJP2	[inhibition]
5738	EGFR	OXSR1	[activation]
5739	TLR3	TAB2	[activation]
5740	PAG1	SLC9A3R1	[activation]
5741	NSMCE2	RAD21	[activation]
5742	SAV1	STK3	[activation]
5743	KIDINS220	NTRK3	[activation]
5744	PPP1R12A	TP53	[activation]
5745	GRB2	PPP6R3	[activation]
5746	TRIP6	CNTF	[activation]
5747	OXSR1	PAK1	[activation]
5748	MAP1LC3B	ACTB	[activation]
5749	PPP2CA	GABARAP	[activation]
5750	DDX21	CTNNBL1	[activation]
5751	IGHG1	FCGR2B	[activation]
5752	PRKCA	TIAM1	[activation]
5753	CDK11B	TPPP	[activation]
5754	CSNK2A2	MAPK14	[activation]
5755	IRAK2	SMAD2	[activation]
5756	TAF1	HMGB1	[activation]
5757	SNAP91	PLCG1	[activation]
5758	DAB1	SERF2	[activation]
5759	SH3KBP1	PAK1	[activation]
5760	ATG5	CNN3	[activation]
5761	ARPC3	EFTUD2	[activation]
5762	GNAQ	GNB3	[activation]
5763	TSC1	MAPK14	[activation;inhibition]
5764	RPA3	SMC2	[activation]
5765	MLST8	A2M	[inhibition]
5766	STAT5A	ESR1	[activation]
5767	PCSK1	IAPP	[activation;inhibition]
5768	FN3KRP	EGFR	[activation]
5769	CDSN	GABARAPL2	[activation]
5770	SMAD4	EIF4A3	[activation]
5771	PCNA	SETDB1	[activation]
5772	GMFB	CSNK2A1	[activation]
5773	POLR2A	KMT2A	[activation]
5774	TMEM173	IFIT1	[activation]
5775	MLLT4	PVRL1	[activation]
5776	GPC1	TDGF1	[activation]

5777	CCR5	TXK	[activation]
5778	LRP1	MBL2	[activation]
5779	PXN	VCL	[activation]
5780	RPS19	GABARAPL2	[activation]
5781	BCAR3	ERBB2	[activation]
5782	MBL2	PTPRC	[activation]
5783	FGF3	FGFR1	[activation]
5784	STK11	COPS4	[activation]
5785	TSTD2	EGFR	[activation]
5786	APP	NRIP1	[activation]
5787	USP7	RARA	[activation]
5788	SPEG	PRMT1	[activation]
5789	ACTA1	GAS7	[activation]
5790	PLCG1	TGOLN2	[activation]
5791	SGK1	HNRNPL	[activation]
5792	TMPRSS11A	MYC	[activation]
5793	RARA	PRKDC	[activation]
5794	EIF4A3	STK38L	[activation]
5795	PSEN1	ECSIT	[activation]
5796	TFRC	ATF2	[activation]
5797	PTPN1	RHOBTB2	[activation]
5798	DDX60	EGFR	[activation]
5799	GSK3B	TPPP	[activation]
5800	HLA-C	BATF2	[activation]
5801	GABARAPL1	MIF	[activation]
5802	NR1H2	COL4A5	[activation]
5803	CASP3	TGM2	[activation]
5804	SENP1	PML	[activation]
5805	EED	EHMT1	[activation]
5806	BID	CRNKL1	[activation]
5807	CMTM3	SPP1	[activation]
5808	PIK3R5	CXCL2	[activation]
5809	TSSK3	HSP90AB1	[activation]
5810	MAPK6	CYTH2	[activation]
5811	KIFC2	TAB1	[inhibition]
5812	WAS	ABI3	[activation]
5813	MOV10	CDH24	[activation]
5814	TNFRSF25	TNFRSF1A	[activation]
5815	APP	CDK3	[activation]
5816	UBTD1	FZD5	[activation]
5817	PTK2B	PITPNM3	[activation]
5818	ABL2	CASS4	[activation]
5819	MEOX2	PAX1	[activation]
5820	CAMSAP1	SKIL	[activation]
5821	APP	TWF1	[activation]
5822	PTEN	CXCL1	[activation]
5823	RAC1	PTPLAD1	[activation]
5824	EGFR	TXN	[activation]
5825	GORASP2	MAPK8	[activation]
5826	MAP1B	SNCA	[activation]
5827	RIT1	GRB2	[activation]
5828	VAMP7	AP3D1	[activation]
5829	STK4	MCM4	[activation]
5830	SRPK1	ILF3	[activation]
5831	TNFSF10	RIPK1	[activation]
5832	EXOC4	SPAG9	[inhibition]
5833	ARF6	MICALL1	[activation]
5834	RELA	SRF	[activation]
5835	LIN54	RB1	[inhibition]
5836	CACNA1D	CALB1	[activation]
5837	MYC	KPNA2	[activation]
5838	IL2RB	JAK2	[inhibition]
5839	WWP1	NOTCH1	[activation]
5840	BCR	FES	[activation]
5841	DCLRE1C	BRCA1	[activation]
5842	SRPK2	SDR42E1	[activation]
5843	AKT1S1	RPS6KA5	[activation]
5844	DYRK1A	CCDC8	[activation]
5845	THAP1	OGT	[activation]
5846	CEP250	HSPA8	[inhibition]
5847	TGFBR1	ARHGEF6	[activation]
5848	CDKN2A	MAPK8	[activation]
5849	PRNP	FAM27E3	[activation]
5850	RANBP2	TP53	[activation]
5851	MYC	DHX15	[activation]
5852	CRK	GRIP2	[activation]

5853	CHD4	ATR	[activation]
5854	UBE2I	CDCA8	[activation]
5855	DYNLRB1	BCL2L11	[activation]
5856	PPFIA3	PPP2CA	[inhibition]
5857	RPS6KB1	BTK	[activation]
5858	NUP62	DGCR6	[activation]
5859	GPR158	MYC	[activation]
5860	PARD3	IGHA1	[activation]
5861	CXCL5	DARC	[activation]
5862	GAB1	VAV1	[activation]
5863	CASP3	MEF2A	[activation]
5864	EIF1	EIF3C	[activation]
5865	CDH1	TGM2	[activation]
5866	TGFBR1	CDC20	[inhibition]
5867	SH2D2A	SMAD3	[inhibition]
5868	LEF1	E2F1	[activation]
5869	SUMO4	PAFAH1B3	[activation;inhibition]
5870	HBG2	APP	[inhibition]
5871	RB1	MAPK8	[activation]
5872	ABL1	LRRK1	[activation]
5873	FASTKD2	TRAF6	[activation]
5874	PRKCA	GFPT1	[activation]
5875	PARD3	SMAD2	[activation]
5876	EIF3F	EIF4A2	[activation]
5877	DKKL1	CRK	[activation]
5878	UBXN11	ZFYVE9	[activation]
5879	DDX46	SRPK2	[activation]
5880	PLN	EDA	[activation]
5881	TWSG1	BMP1	[activation]
5882	SCRIB	OBSL1	[activation]
5883	NUP98	NUP88	[activation]
5884	MCM2	CDK4	[activation]
5885	BMP7	MTUS2	[activation]
5886	RELA	KAT2A	[activation]
5887	MDM2	TTF1	[activation]
5888	SMPD1	CASP7	[activation]
5889	RXRB	CACNA1B	[inhibition]
5890	UBC	DLX5	[activation]
5891	PRSS1	GRB2	[activation]
5892	CCNG1	LTBP3	[activation]
5893	ECT2	PGAM5	[activation]
5894	DVL3	HOMEZ	[activation]
5895	ZNF282	MAPKAPK5	[activation]
5896	MAST2	GCN1L1	[activation]
5897	SETDB1	HMOX2	[activation]
5898	NXF1	C9orf40	[activation]
5899	YWHAB	MLK4	[activation]
5900	TRA2A	SRPK2	[activation]
5901	CFL1	SMURF1	[activation;inhibition]
5902	CD247	NSUN5P2	[activation]
5903	MCM3	OBSL1	[activation]
5904	PLK1	RNF126	[activation]
5905	ELANE	SERPING1	[inhibition]
5906	KIF23	PPP2CA	[activation]
5907	CAV1	KCNA5	[activation]
5908	CD247	LAT	[activation]
5909	HTT	EHMT1	[activation]
5910	CDC42	DEF6	[activation]
5911	MAP2K4	RBBP8	[activation]
5912	GNRHR2	GNRH2	[activation]
5913	CLIP1	MAPRE1	[activation]
5914	PPARG	CTNNB1	[activation]
5915	MAPKAPK2	HNRNPA0	[activation]
5916	GRN	ELANE	[activation]
5917	SRCIN1	BCAR1	[activation]
5918	EIF2S3	METAP2	[activation]
5919	XPO1	E2F5	[activation]
5920	PRKDC	TOP1	[activation]
5921	NXF1	GRHPR	[activation]
5922	TPP1	EGFR	[activation]
5923	EP300	KCTD5	[activation]
5924	CREBBP	UBE2I	[activation]
5925	MAPK14	TCEAL1	[activation]
5926	RPL35A	GABARAPL2	[activation]
5927	GEM	RNF7	[activation]
5928	TOP1	KMT2A	[activation]

5929	PARP2	CTNNB1	[activation]
5930	NCK2	IRS1	[activation]
5931	PLA2G4A	MYC	[activation]
5932	CASP4	NUP93	[activation]
5933	PPP3CC	BMPR1B	[activation;inhibition]
5934	ATF2	SSFA2	[activation]
5935	CFP	C3	[activation;inhibition]
5936	SP1	MAPK3	[activation]
5937	NLRC4	EIF2AK2	[activation]
5938	PTPRK	MAPK3	[inhibition]
5939	CD33	CBL	[activation]
5940	PIN1	PTPN1	[activation]
5941	EIF2A	APP	[activation]
5942	SMAD7	EP300	[activation]
5943	CES1	GUSB	[activation]
5944	RPTOR	FBXO9	[activation;inhibition]
5945	MEP1A	POMC	[activation]
5946	ZKSCAN8	PRNP	[activation]
5947	NGFR	NDNL2	[activation]
5948	EHMT2	SUV39H1	[activation]
5949	PIK3R1	PLCG2	[activation]
5950	VAPA	GPR128	[activation]
5951	FYN	TULP1	[activation]
5952	AGFG1	ITGA4	[activation]
5953	CMYA5	DYSF	[activation]
5954	GEM	TRIM54	[activation]
5955	WNT4	P2RX4	[activation]
5956	RELA	BARD1	[activation]
5957	RHOC	GH1	[activation]
5958	CDK4	BMPR1B	[activation;inhibition]
5959	PDGFA	PDAP1	[activation]
5960	MARCKS	MAPK13	[activation]
5961	SVEP1	SKIL	[activation]
5962	TOLLIP	IL1RAP	[activation]
5963	ACTG1	RIPK2	[activation]
5964	USHBP1	DYDC1	[activation]
5965	RADIL	GNB1	[activation]
5966	ATF6	NFYC	[activation]
5967	TNRC6A	PRNP	[activation]
5968	PAXIP1	NUPR1	[activation]
5969	CHEK1	SETMAR	[activation]
5970	DAPK2	MLC1	[activation]
5971	NUMB	PRKCA	[activation]
5972	CTNNB1	DDX5	[activation]
5973	JAK2	FGFR1	[activation]
5974	NSMCE2	SMC5	[activation]
5975	ARHGEF6	CAPNS1	[activation]
5976	BHLHE40	BRD7	[activation]
5977	EDARADD	ATG5	[activation]
5978	ARRB1	MAPK14	[activation]
5979	MYC	ARF1	[activation]
5980	MAP3K3	PPP2R5A	[activation]
5981	PAX6	HIPK2	[activation]
5982	CDK5	CDK5R2	[activation]
5983	ARFGEF2	GABRB2	[activation]
5984	CDH1	YAP1	[activation]
5985	PTPRJ	ERBB2	[activation]
5986	RAB8A	RAB10	[activation]
5987	SBK1	TRIP10	[activation]
5988	HSPH1	CFTR	[activation]
5989	HSD17B3	CDH1	[activation]
5990	PRKACA	SI	[activation]
5991	ATXN10	PHLDA3	[activation]
5992	MEPE	GRB2	[activation]
5993	PDIA5	NXF1	[activation]
5994	PPP1CC	HSPA2	[inhibition]
5995	RAPGEF1	HCK	[activation]
5996	CLTC	RAC1	[activation]
5997	SH3GL2	TIAM2	[activation]
5998	TFRC	TRAF6	[activation]
5999	CSF1R	ITGAV	[activation]
6000	BRAF	TERF1	[activation]
6001	MLLT4	SSX2IP	[activation]
6002	OPRM1	GNAI1	[activation]
6003	NR4A1	PPM1A	[inhibition]
6004	FASLG	ARHGAP9	[activation]

6005	RICTOR	AKT1S1	[activation;inhibition]
6006	NTRK3	HTR2A	[activation]
6007	GADD45G	LUC7L	[activation]
6008	CREBBP	LDLR	[activation]
6009	APOL1	IGHM	[activation]
6010	MTPN	RELA	[activation]
6011	HSPB1	ANXA1	[activation]
6012	MEFV	PSTPIP1	[inhibition]
6013	PPP2R3B	MYC	[activation]
6014	NFYA	HDAC1	[activation]
6015	KIF7	SAV1	[activation]
6016	MAP2K2	CNKSR1	[activation]
6017	YWHAG	TIAM1	[activation]
6018	SCAMP1	TP53	[activation]
6019	MCM3	CCL2	[activation]
6020	ATP6V1B1	VAMP7	[activation]
6021	HEATR2	EGFR	[activation]
6022	TGFBR2	TGFB2	[activation]
6023	IL1R2	IL1RN	[inhibition]
6024	GRN	POT1	[activation]
6025	TP53	KLF6	[activation]
6026	MAGEA11	NR3C1	[activation]
6027	NUMB	DPYSL2	[activation]
6028	CTSB	SRPX2	[activation]
6029	CREBBP	DDIT3	[activation]
6030	STAT5A	TCF12	[activation]
6031	SH2B3	EGFR	[activation]
6032	HSP90AB1	MAP4K2	[activation]
6033	CDK6	MCM2	[activation]
6034	RAB11FIP5	ARF6	[activation]
6035	GPR78	APP	[activation]
6036	GNAQ	S1PR2	[activation]
6037	H2AFX	TRAP1	[activation]
6038	RBPMS	FOXP3	[activation]
6039	AFF1	PCSK1	[activation;inhibition]
6040	CAPN1	BCL2L1	[activation]
6041	SRSF10	SFN	[activation]
6042	MYO1F	RPA1	[activation]
6043	FLAD1	ANXA7	[activation]
6044	GNAI2	ITCH	[activation]
6045	C5orf30	MAPK7	[activation]
6046	ARRB1	DVL1	[activation]
6047	MAP3K14	MAP3K7	[activation]
6048	PDPK1	PTPRJ	[activation]
6049	ERBB3	DAB1	[activation]
6050	TP53	MRPS18B	[activation]
6051	CEBPB	STAT5A	[activation]
6052	FHL2	SMAD3	[inhibition]
6053	CORO2B	VCL	[activation]
6054	CNTROB	LATS1	[inhibition]
6055	VTN	PVR	[activation]
6056	EGFR	HEATR3	[activation]
6057	PTPRU	GNAI1	[activation;inhibition]
6058	RAD1	RPA1	[activation]
6059	PLK1	RAP1GAP	[activation;inhibition]
6060	HSPB1	EPB41L3	[activation]
6061	TPD52	TPD52L1	[activation;inhibition]
6062	EIF2S1	PRKCA	[activation]
6063	COL4A1	SMAD1	[activation]
6064	NEDD8	CASP7	[activation]
6065	SENP1	TP53	[activation]
6066	CCNE1	RBL1	[activation]
6067	PTGIR	ADCY10	[activation]
6068	PSIP1	CASP3	[activation]
6069	SIPA1L3	FMNL1	[activation]
6070	CDKN2A	RIN2	[activation;inhibition]
6071	PRKAR1A	TAB1	[inhibition]
6072	HIF1A	MAFG	[activation]
6073	FBXO6	YTHDF2	[inhibition]
6074	BMPR2	TGFBR1	[activation]
6075	MAPK14	NFATC1	[activation]
6076	NXF1	CALM1	[activation]
6077	FANCG	FAN1	[activation]
6078	YWHAB	TTC28	[activation]
6079	PTPRM	CDH1	[activation]
6080	RELA	SAT1	[activation]

6081	KPNA1	IL1RAP	[activation]
6082	HSPB1	TTC3	[activation]
6083	CAMKK2	PRKDC	[activation]
6084	ABI1	MRFAP1L1	[activation]
6085	PPP3CA	ATP2B4	[activation]
6086	RPA3	RAD52	[activation]
6087	NGF	SORT1	[activation]
6088	BCR	TP53	[activation]
6089	RAB5C	RPA2	[activation]
6090	GSK3A	HSF1	[activation]
6091	ANXA2	PRKCA	[activation]
6092	AFG3L2	EGFR	[activation]
6093	TEAD4	WWTR1	[activation]
6094	PRPSAP1	PLEKHF2	[inhibition]
6095	OR8D2	IGHG1	[activation]
6096	RELA	OGT	[activation]
6097	CD5	PRKCB	[activation]
6098	PTF1A	RBPJ	[activation]
6099	SOCS2	MET	[inhibition]
6100	SUGT1	NOD1	[activation]
6101	SFRP4	PIK3CA	[activation]
6102	KRAS	FRAT2	[inhibition]
6103	PLEKHG5	RND1	[activation]
6104	BMPR1B	PAK1	[activation]
6105	GABARAPL2	ATG16L1	[activation]
6106	ACTB	TMSB4X	[activation]
6107	KDM6A	GATA4	[activation]
6108	TOP2A	H3F3A	[activation]
6109	STAT3	SMAD1	[activation]
6110	SNRNP70	SMAD4	[activation]
6111	DNM1	CDK5	[activation]
6112	SYK	TRAIP	[activation]
6113	IL27RA	IL27	[activation]
6114	ACOT9	EGFR	[activation]
6115	WNK1	YWHAG	[activation]
6116	TOP2A	SRPK1	[activation]
6117	BAG3	ATG7	[activation]
6118	GPN1	RPAP2	[activation]
6119	GNAI3	GNB1	[activation]
6120	ICAM1	RPS15	[activation]
6121	RPS6KA4	SNW1	[activation]
6122	STMN1	HSPA8	[activation]
6123	CDKN1A	DOCK7	[activation]
6124	DDA1	PRKAA1	[inhibition]
6125	ITGA4	LONP1	[activation]
6126	YWHAQ	RASGRP3	[activation]
6127	GABARAP	ATG10	[activation]
6128	RUNX1	PAX5	[activation]
6129	MYBL2	LIN54	[inhibition]
6130	FAM98B	SGK1	[activation]
6131	NUSAP1	APP	[activation]
6132	ATF1	GABPA	[activation]
6133	NEDD9	LYN	[activation]
6134	CDK7	CCNA2	[activation]
6135	CHRM4	GRB2	[activation]
6136	TRAF3	SRC	[activation]
6137	CTR9	IGHG1	[activation]
6138	CDKN3	NCOA3	[activation]
6139	AHCYL2	TRAF6	[activation]
6140	PIK3C3	YWHAG	[activation]
6141	TP53	CALD1	[activation]
6142	MYC	SMC2	[activation]
6143	NBN	RBBP8	[activation]
6144	HMGA2	UBC	[activation]
6145	CTBP2	EHMT2	[activation]
6146	CFH	ADM	[inhibition]
6147	MYF6	CSRP3	[activation]
6148	RAF1	KRAS	[activation]
6149	SELPLG	BACE1	[activation]
6150	PRKCE	FYN	[activation]
6151	RTCA	ARF6	[activation]
6152	YWHAZ	IQGAP1	[activation]
6153	CDKN2B	KIAA1377	[activation;inhibition]
6154	TJP1	RAPGEF2	[activation]
6155	TP53	ERCC3	[activation]
6156	APP	IL26	[activation]

6157	PTK2	JAK2	[activation]
6158	TRAP1	SMAD4	[activation]
6159	CDH24	CTNND1	[activation]
6160	ANKRD11	NCOA3	[activation]
6161	IKBKB	YWHAB	[activation]
6162	MAP3K14	BIRC3	[activation;inhibition]
6163	HIF1A	UBE2I	[activation]
6164	MAST3	PTEN	[activation;inhibition]
6165	RICTOR	DHX36	[activation]
6166	SUMO1	EIF4G1	[activation]
6167	STAT1	GNB2	[activation]
6168	MAPKAPK2	LYAR	[activation]
6169	RPA1	EGFR	[activation]
6170	EIF4E2	USHBP1	[inhibition]
6171	XIAP	RIPK1	[activation]
6172	MAPK1	IFNAR1	[activation]
6173	MAPK3	CDC23	[activation]
6174	CALM1	TRDN	[activation]
6175	PHACTR2	PIK3R1	[activation]
6176	INSIG1	RNF139	[activation]
6177	EGFR	PIN4	[activation]
6178	PIAS1	CREB1	[inhibition]
6179	PINX1	RHOC	[activation]
6180	STAT1	BCL3	[activation]
6181	EGFR	MCM5	[activation]
6182	NCOA2	HNF4A	[activation]
6183	NME1	NME3	[activation]
6184	ABI2	VASP	[activation]
6185	RCAN1	CAPN1	[activation]
6186	HSPA8	YWHAZ	[inhibition]
6187	ZFYVE9	PPP1CC	[activation]
6188	SORCS2	BDNF	[activation]
6189	FBXO6	PPP1CC	[inhibition]
6190	ARHGEF5	GRB2	[activation]
6191	XPO7	RAN	[activation]
6192	APCDD1	WNT3A	[activation]
6193	PRKCA	GLI3	[activation]
6194	KMT2E	MYB	[activation]
6195	PINX1	MIF	[activation]
6196	CTNNA2	PPP2R1A	[activation]
6197	NFYA	ATF2	[activation]
6198	EXTL3	SRC	[activation]
6199	CCNE2	RBL2	[inhibition]
6200	OGT	PSG1	[activation]
6201	CD44	TRAF6	[activation]
6202	SMURF1	ING1	[inhibition]
6203	TRAF6	KCTD12	[activation]
6204	RPA2	SMC2	[activation]
6205	KCTD17	RAPGEF2	[activation]
6206	TP53	BLM	[activation]
6207	IL1RL1	MYD88	[activation]
6208	GNAS	GCGR	[activation]
6209	CYCS	VHL	[inhibition]
6210	NXF1	ZRANB1	[activation]
6211	STAT1	ATF3	[activation]
6212	DUPD1	TRAF6	[activation]
6213	CSF2RB	FYN	[activation]
6214	SEMA6A	EVL	[activation]
6215	NFIL3	AMOTL2	[activation]
6216	PPP2R5D	CDC25C	[activation]
6217	VDR	POU1F1	[activation]
6218	CDK1	RPA1	[activation]
6219	ARHGDIA	TNFRSF10B	[activation]
6220	MAGI3	DSCAML1	[activation]
6221	RIF1	HMGB1	[inhibition]
6222	KSR1	PPP2R5B	[activation]
6223	SMARCD1	TP53	[activation]
6224	PRNP	MARK4	[activation]
6225	BCL6	WDR35	[activation]
6226	USP50	HSPA8	[inhibition]
6227	CD81	EIF3C	[activation]
6228	SMARCE1	GATA1	[activation]
6229	PCDH10	NCKAP1	[inhibition]
6230	PTK2B	UBC	[activation]
6231	TNFAIP2	ERBB2	[activation]
6232	IL24	CHEK2	[activation]

6233 HIST1H3A GRB2 [activation]
6234 DGKE NUDC [activation]
6235 CREB3 BCL2L1 [activation]
6236 CALM1 ATF2 [activation]
6237 PLK1 MYC [activation]
6238 TBP HMGB1 [inhibition]
6239 TGFBR1 RASL12 [activation]
6240 SUFU BTRC [activation]
6241 GH1 STRAP [inhibition]
6242 WAS RAC1 [activation]
6243 ADAM10 MAD2L1 [activation]
6244 GAB1 LYN [activation]
6245 HRAS BUB1 [activation]
6246 HSPE1 FTSJ1 [activation]
6247 MAP2K1 HRAS [activation]
6248 HIST1H3A SIRT1 [activation]
6249 CCL5 IL8 [activation]
6250 TAB2 TNF [activation]
6251 CDK6 MEF2D [inhibition]
6252 TPD52L1 MAP3K5 [activation]
6253 MYB MAF [activation]
6254 SATB1 CTNNB1 [activation]
6255 MAPK3 PTPRG [activation;inhibition]
6256 TNFSF11 MMP7 [activation]
6257 GRB2 PRKAR1A [activation]
6258 TP53 STRA13 [activation]
6259 PTGES3 CASP3 [activation]
6260 MACF1 GOLGA4 [activation]
6261 FASN MAPK13 [activation]
6262 TSPAN10 ADAM10 [activation]
6263 LCK ABI1 [activation]
6264 GNAI2 MTNR1A [activation]
6265 GABARAPL2 HNRNPA0 [activation]
6266 CSNK1D APP [activation]
6267 ERBB2 CHN1 [activation]
6268 TLR2 PIK3R1 [activation]
6269 TRAF2 ACTG1 [activation]
6270 CD81 HIST1H2BH [activation]
6271 PPP2CA IRAK1 [inhibition]
6272 LRRK2 PLK1 [activation]
6273 EGFR GABARAPL2 [activation]
6274 GABARAPL2 JUP [activation]
6275 TNFSF11 EZH2 [activation]
6276 STX5 SEC22A [activation]
6277 SUMF2 ZFYVE9 [activation]
6278 TBK1 APP [activation]
6279 AKT1 PTPN1 [activation]
6280 ZFPM1 TOP3B [activation]
6281 BAX BCL2L1 [activation]
6282 C6orf165 TP73 [activation;inhibition]
6283 ZBTB22 GATA1 [activation]
6284 CBLB TRAF6 [inhibition]
6285 CCND1 MAPK11 [activation]
6286 YWHAG CPSF3 [activation]
6287 CBL PDGFRA [activation]
6288 ARRB2 SAG [inhibition]
6289 RPS6KA1 GBP2 [activation]
6290 HSP90AB1 MKNK1 [activation]
6291 SIRT1 SMAD7 [activation;inhibition]
6292 LATS1 VPRBP [inhibition]
6293 NMI STAT3 [activation]
6294 TP53 TFDP1 [activation]
6295 HOMER3 GRM5 [activation]
6296 SPRR2A YES1 [activation]
6297 SCAMP5 STX6 [activation]
6298 RAB5A RAB7A [activation]
6299 F9 F8 [activation]
6300 RAC1 BCR [activation]
6301 FAS UBA7 [inhibition]
6302 CRK FASLG [activation]
6303 ANAPC1 ANAPC4 [activation]
6304 DDIT3 FOS [activation]
6305 CFP CFB [activation;inhibition]
6306 FYB VASP [activation]
6307 HSP90AB1 CRK [activation]
6308 HSPG2 FGFR3 [activation]

6309	GATA4	MEF2C	[activation]
6310	CSDE1	HLA-B	[activation]
6311	MYC	FOXP1	[activation;inhibition]
6312	SERPINA1	SSR1	[inhibition]
6313	TICAM2	TIRAP	[activation]
6314	NXF1	CAPRIN1	[activation]
6315	SP1	CABP1	[activation]
6316	NR3C1	SLC25A4	[activation]
6317	PPP2R1A	MAPK6	[inhibition]
6318	SPNS1	BCL2L1	[activation]
6319	CCDC22	FAM21C	[activation]
6320	PTEN	PDGFRB	[activation]
6321	CAMK2A	ATF1	[activation]
6322	ACVR2B	BMP10	[activation]
6323	ASNS	IKBKE	[activation]
6324	WNK1	FAM53C	[activation]
6325	TMEM239	SENP2	[activation]
6326	CD81	HNRNPA1	[activation]
6327	HMGB1	CSNK1A1	[activation]
6328	ZDHHC17	STK3	[activation]
6329	EBNA1BP2	TP53	[activation]
6330	ZAK	YWHAZ	[activation]
6331	MAX	HSP90AB1	[activation]
6332	NCKIPSD	FYN	[activation]
6333	MMP13	COL2A1	[activation]
6334	JUN	HHEX	[activation]
6335	PRKCD	HABP4	[activation]
6336	TNC	NCAN	[activation]
6337	SDCBP	EFNB2	[activation]
6338	GSN	ESR2	[activation]
6339	ACACA	UBE2N	[activation;inhibition]
6340	WDR24	RAE1	[activation]
6341	CREBBP	PPARGC1A	[activation]
6342	CD3G	NR3C1	[activation]
6343	YWHAZ	CDK18	[activation]
6344	PARP2	IL21	[activation]
6345	ICT1	RARS2	[inhibition]
6346	SETDB1	TRIB3	[activation]
6347	PTK2B	VAV1	[activation]
6348	TCEB1	WNT7B	[activation]
6349	CDH1	PSEN1	[activation]
6350	IRF1	IRF8	[activation]
6351	BNIP1	BCL2L1	[activation]
6352	CDKN1A	CHGB	[activation]
6353	WHSC1L1	RAD51	[activation]
6354	ALKBH3	SRPK2	[activation]
6355	LRP1	GTPBP1	[activation]
6356	CBL	PRPSAP2	[inhibition]
6357	HNRNPK	TP53	[activation]
6358	COPB1	IRAK3	[inhibition]
6359	ESR1	SRPK1	[activation]
6360	TRIM28	EGFR	[activation]
6361	CD81	HIST1H1D	[activation]
6362	FGA	FGB	[activation]
6363	EFNB1	CCDC8	[activation]
6364	ASB7	VCL	[activation]
6365	RARA	RXRB	[inhibition]
6366	RANBP9	ADAP2	[activation]
6367	SERPINC1	PLG	[inhibition]
6368	PIAS2	TP53	[activation]
6369	ST14	CDKN2C	[inhibition]
6370	STX1A	CDK5	[activation]
6371	GABRG1	GABARAP	[activation]
6372	EIF4E2	UBXN11	[inhibition]
6373	AATF	MAPT	[activation]
6374	MAPRE3	JUN	[activation]
6375	LCK	ADAM10	[activation]
6376	MYL12B	DAPK3	[activation]
6377	APPL1	ANKRD1	[activation]
6378	IL1RAP	SIRPA	[activation]
6379	GPN1	HSP90AB1	[activation]
6380	CTNNB1	PTPRG	[activation]
6381	NCOA1	CREBBP	[activation]
6382	RAC1	CSN2	[activation]
6383	NRGN	GLP1R	[activation]
6384	AMOTL2	CYTH4	[activation]

6385	PTPN11	IL4R	[activation]
6386	ACTB	CAV1	[activation]
6387	CDKN2A	CCNG1	[activation;inhibition]
6388	LYPD3	ERBB2	[activation]
6389	GPR1	GNAI1	[activation]
6390	RAB4A	CDK1	[activation]
6391	ZRSR2	SRPK1	[activation]
6392	CSNK2A1	PRNP	[activation]
6393	SRC	CFL1	[activation;inhibition]
6394	BCL6	KIF13B	[activation]
6395	BRAF	KSR2	[activation]
6396	RHOA	KCTD13	[activation]
6397	NOTCH2NL	ITGB2	[activation]
6398	PPP2R1A	PPP2R4	[activation]
6399	FAS	APAF1	[activation]
6400	DTX3	UBE2E2	[activation]
6401	ASAP1	FYN	[activation]
6402	FAM208B	KCTD9	[activation]
6403	RPRD1B	RPA2	[activation]
6404	BCL2L13	PLLP	[activation]
6405	PPP2CA	MST4	[inhibition]
6406	ITPRIP	ITPR1	[inhibition]
6407	GRB2	NCL	[activation]
6408	IL6R	JAK1	[activation]
6409	SKIL	UBE2I	[activation]
6410	EPS15	FYN	[activation]
6411	YES1	MET	[activation]
6412	DKC1	MAPK6	[activation]
6413	COL7A1	MMP2	[inhibition]
6414	MAPK1	IER3	[activation]
6415	LDHAL6A	LRRK2	[activation]
6416	PIK3R2	CCR5	[activation]
6417	CTSL	IGHA1	[activation]
6418	NOP2	PAXIP1	[activation]
6419	TFDP1	CDK6	[inhibition]
6420	RASA1	RAP1A	[activation]
6421	JAG1	CALR	[activation]
6422	HSP90AB1	TTI2	[activation]
6423	PTPRC	IRS1	[activation]
6424	WASF2	PRPF40A	[activation]
6425	EDA	GIMAP5	[activation]
6426	RCBTB2	UBE2E3	[activation]
6427	FTSJ1	OXSR1	[activation]
6428	TRIB3	TIAF1	[inhibition]
6429	VCAM1	LYPLA2	[activation]
6430	MET	PLXNB3	[activation]
6431	ATRIP	RPA1	[activation]
6432	MAP4K2	MAP3K11	[activation]
6433	WNT2	WNT3A	[activation]
6434	XRCC3	RAD51	[activation]
6435	GRB2	PPP1CA	[activation]
6436	UTP14A	RGS1	[activation;inhibition]
6437	IL18	CASP4	[activation]
6438	NFE2L2	WAC	[activation]
6439	MARK3	DLG5	[activation]
6440	GLI2	HDAC1	[inhibition]
6441	HIST1H3A	RAD51B	[activation]
6442	EGFR	LRRK1	[activation]
6443	HMMR	BRCA1	[activation]
6444	ASB17	HSPB1	[activation]
6445	RAC1	UNKL	[activation]
6446	SPAG9	MYC	[inhibition]
6447	UBTF	RB1	[activation]
6448	NGRN	E2F1	[activation]
6449	DDX39B	HIPK2	[activation]
6450	LRRK2	RPL17	[activation]
6451	RBCK1	TNF	[activation]
6452	TFDP2	LIN9	[inhibition]
6453	VPS35	TRAF6	[activation]
6454	SRGAP2	YWHAZ	[activation]
6455	DYSF	MYOM2	[activation]
6456	CAMK2B	CAMK2A	[activation]
6457	TNFSF11	KHDRBS1	[activation]
6458	FGF2	CXCL13	[activation]
6459	HIST1H3A	FGF14	[activation]
6460	DDX5	AURKA	[activation]

6461	FHL1	SRPK1	[activation]
6462	CASP3	MLH1	[activation]
6463	STK11IP	TLR4	[activation]
6464	HIST2H2AA3	GRB2	[activation]
6465	TNFRSF1B	RIPK1	[activation]
6466	GJA5	PRKACA	[activation]
6467	GNB5	RGS11	[activation;inhibition]
6468	GIT1	TRIB3	[inhibition]
6469	MTMR4	SMAD3	[activation]
6470	WAS	PLCG1	[activation]
6471	DNM1	PRNP	[activation]
6472	SMAD2	MYO18A	[activation]
6473	KPNB1	SMURF1	[inhibition]
6474	CCND1	EP300	[activation]
6475	ICAM1	HIST3H3	[activation]
6476	SPP1	MAP1A	[activation]
6477	CCDC180	SRC	[activation]
6478	ADRBK2	ADRBK1	[activation]
6479	ITGA4	RAB5C	[activation]
6480	FDFT1	ANXA5	[activation]
6481	BPTF	SMARCA1	[activation]
6482	UGT1A4	UGT1A1	[activation]
6483	NUP214	DDX19B	[activation]
6484	MAPK14	SMAD7	[activation]
6485	PPP1CC	PPP1R15B	[inhibition]
6486	YES1	VCAM1	[activation]
6487	GOLGA2	LINGO1	[activation]
6488	CDK2	SKP1	[inhibition]
6489	RPA1	BUB3	[activation]
6490	PIK3C2A	YWHAB	[activation]
6491	NR0B1	AR	[activation]
6492	TIMM50	IKBKB	[activation]
6493	ESR2	SPRR1B	[activation]
6494	EIF2S2	UPF1	[activation]
6495	CXXC1	EP300	[activation]
6496	A2M	ADAM19	[inhibition]
6497	PRKCB	GRM5	[activation]
6498	IRF7	TRIM28	[activation]
6499	SYK	ERBB3	[activation]
6500	PAK2	GRB2	[activation]
6501	ENAH	ABI1	[activation]
6502	ATP13A2	AAK1	[activation]
6503	FANCM	FAAP24	[activation]
6504	TRIB1	MAP2K7	[activation]
6505	SRF	NCOA1	[activation]
6506	MLLT4	SMAD2	[activation]
6507	EPHB2	NUP153	[activation]
6508	DDX21	OBSL1	[activation]
6509	PTPN11	FCRL3	[activation]
6510	CACNA1S	SRI	[activation]
6511	EP300	ASH2L	[activation]
6512	PTPN11	KIR2DL5A	[activation]
6513	PRDX1	GRB2	[activation]
6514	SMC4	SMYD2	[activation]
6515	HLA-C	ADRB2	[activation]
6516	TNFSF11	MMP3	[activation]
6517	YWHAZ	HSPH1	[activation]
6518	FBXO6	PTPRC	[activation]
6519	CXCL9	PTPN5	[activation]
6520	LAS1L	FOS	[activation]
6521	SMAD2	ROCK1	[activation]
6522	PTPRC	EZR	[activation]
6523	PML	NR4A1	[inhibition]
6524	LUC7L	CTNNBL1	[activation]
6525	PTTG1	CDK1	[inhibition]
6526	CHGB	UBTF	[activation]
6527	SMURF1	USP45	[inhibition]
6528	ELAVL1	MAP2K7	[activation]
6529	FGFR1	HSP90AA1	[activation]
6530	ACSBG1	APP	[activation]
6531	MARCKS	ATF2	[activation]
6532	PACSIN3	SBK1	[activation]
6533	NXF1	CHD8	[activation]
6534	MAPK12	MAPT	[activation]
6535	BAIAP3	BAI1	[activation]
6536	STX4	STXBP4	[activation]

6537	CD84	ABL2	[activation]
6538	EVI5L	SRPK2	[activation]
6539	YWHAH	RASSF8	[activation]
6540	EIF2A	ATF4	[activation]
6541	ITGB2	PRKCD	[activation]
6542	NRD1	TP53	[activation]
6543	STAT1	NMI	[activation]
6544	HNF4A	SMAD2	[activation]
6545	CBL	ITGA4	[activation]
6546	PIK3R1	ABL1	[activation]
6547	NME7	MAP4K4	[activation]
6548	PKNOX1	MEIS2	[activation]
6549	PKNOX2	FOS	[activation]
6550	LRP1	BACE1	[activation]
6551	CHD4	VCAM1	[activation]
6552	C2orf44	CSNK1G1	[activation]
6553	CACNA1H	PPP3CB	[activation]
6554	RPA3	C7orf50	[activation]
6555	COL1A1	DDR2	[activation]
6556	STAT3	HIC1	[activation]
6557	PAM	KALRN	[activation]
6558	CAD	MAP3K7	[activation;inhibition]
6559	MYC	ACACB	[activation;inhibition]
6560	CORO1C	TP53	[activation]
6561	ICT1	MRPL22	[activation]
6562	FZR1	BTRC	[inhibition]
6563	ECHS1	TIMP2	[activation]
6564	CCHCR1	MAD1L1	[activation]
6565	EZR	YWHAZ	[activation]
6566	ATF3	DBP	[activation]
6567	ABI2	ARMC7	[activation]
6568	TRIP10	CDC42	[activation]
6569	CDX2	CREBBP	[activation]
6570	FGFR2	BEX1	[activation]
6571	PLCB3	SLC9A3R1	[activation]
6572	STK10	CD81	[activation]
6573	DFFA	BAK1	[activation]
6574	PTK2B	CBL	[activation]
6575	MAPK9	DUSP16	[inhibition]
6576	CLNS1A	LRRK2	[activation]
6577	ARHGAP12	SRPK2	[activation]
6578	CREBRF	CD2BP2	[activation]
6579	BTN1A1	PLK1	[activation]
6580	DNAJA3	MET	[inhibition]
6581	NUP93	ILK	[activation]
6582	EIF4E	CYFIP1	[inhibition]
6583	VAMP8	USP8	[activation]
6584	CACNA2D3	RAD51C	[activation]
6585	MAPK14	MKNK2	[activation]
6586	DHX57	DDX56	[activation]
6587	ROR2	MAGED1	[activation]
6588	NF2	LATS1	[inhibition]
6589	RANGAP1	EGFR	[activation]
6590	SNRPD2	GRB2	[activation]
6591	HSPB1	MRPL40	[activation]
6592	SHC1	GAB2	[activation]
6593	CBL	HSPA4	[inhibition]
6594	BCAR1	CBL	[activation]
6595	FOXO1	SMAD3	[activation]
6596	VCL	TLN1	[activation]
6597	ATF4	GOLGA1	[activation]
6598	EGFR	DHRS7B	[activation]
6599	TYK2	IFNAR2	[activation]
6600	MEOX2	NLGN3	[activation]
6601	EBI3	SMAD3	[activation]
6602	PTPRK	MET	[inhibition]
6603	E2F1	MAGEA11	[activation]
6604	INCA1	CTNNBIP1	[inhibition]
6605	MTOR	TRAF2	[activation]
6606	ESD	GRB2	[activation]
6607	AAR2	ARF6	[activation]
6608	RPA3	RFC4	[activation]
6609	MYC	CPSF2	[activation]
6610	HIST2H3A	BRD7	[activation]
6611	NCK1	FANCA	[activation]
6612	BCCIP	HIST1H2BN	[activation]

6613	AHDC1	ITSN2	[activation]
6614	HSPA8	FBXO6	[inhibition]
6615	FLNC	ADRA1B	[activation]
6616	CD40	HSPA4	[activation]
6617	PIK3CG	ADRBK1	[activation]
6618	NDEL1	PXN	[activation]
6619	CTSV	RB1	[activation;inhibition]
6620	SRC	DAB1	[activation]
6621	SRC	ALK	[activation]
6622	EPB41L3	ABL1	[activation]
6623	RAB3GAP2	ARF6	[activation]
6624	ERG	DKC1	[activation]
6625	EGF	CPM	[activation]
6626	APBB1IP	ZDHHC17	[activation]
6627	CDK16	YWHAG	[activation]
6628	HSP90AB1	ERBB4	[activation]
6629	CCNA2	ANAPC11	[activation]
6630	SP1	MYOG	[activation]
6631	DDX6	GRB2	[activation]
6632	FGFR2	ITGA5	[activation]
6633	ASIC3	MAGI1	[activation]
6634	FYN	ERBB2	[activation]
6635	MC4R	MC1R	[activation]
6636	SRPK3	BRIX1	[activation]
6637	EP300	TWIST1	[activation]
6638	SFRP4	NRAS	[activation]
6639	EIF2AK2	CHUK	[activation]
6640	ITGB4	ENPP2	[activation]
6641	NFATC1	SPI1	[activation]
6642	HDAC1	RBL1	[inhibition]
6643	UCN3	CRHR2	[activation]
6644	RELA	HSPA1L	[activation]
6645	DFNB31	MYO15A	[activation]
6646	APP	FDX1L	[activation]
6647	PRKAA2	STIM2	[activation]
6648	PRNP	PPFIBP2	[activation]
6649	NDRG4	CMTM5	[activation]
6650	RPS6KA3	HTR2A	[activation]
6651	ECSIT	NFKB1	[activation]
6652	PCBP1	TRAF6	[activation]
6653	PSD	RAB40C	[activation]
6654	CDC16	CDC5L	[activation]
6655	SHOC2	RAF1	[activation]
6656	YWHAB	SRPK2	[activation]
6657	ADTRP	VTN	[inhibition]
6658	HUNK	CFL1	[activation;inhibition]
6659	MDK	SRPK2	[activation]
6660	PUM1	CDKN1A	[activation]
6661	MAPK14	GMFB	[activation]
6662	FANCD2	TRAF6	[activation]
6663	H2AFY	ATF2	[activation]
6664	TP53	CAPZB	[activation]
6665	MDM2	UBE2E3	[activation]
6666	BANP	RIMS3	[activation]
6667	CDC5L	RAD50	[activation]
6668	TP53BP1	CDC16	[activation]
6669	DVL2	IRS2	[activation]
6670	SOX2	PRKCI	[activation]
6671	GTF2A1	NFYA	[activation]
6672	EIF3L	HLA-C	[activation]
6673	PPL	CASP6	[activation]
6674	HSP90AB1	AMHR2	[activation]
6675	BMP6	ACVR2B	[activation]
6676	ABR	TP53	[activation]
6677	MAP2K2	MAPK8	[activation]
6678	MORC4	STAT3	[activation]
6679	ICAM3	APP	[activation]
6680	KAT2B	NR4A1	[activation]
6681	F7	CEP76	[activation;inhibition]
6682	EIF3L	GH1	[inhibition]
6683	SRGAP3	SMARCA4	[activation]
6684	ANAPC1	RPAP1	[activation]
6685	TOLLIP	CSN2	[activation]
6686	ESR1	G3BP2	[activation]
6687	TUBB4A	CHUK	[activation]
6688	MAPKAPK2	ZFP36L1	[activation]

6689	CDK1	MARCKS	[activation]
6690	STRAP	FBXO6	[inhibition]
6691	HSPA8	IL32	[activation]
6692	NRAS	MAPKAPK3	[activation]
6693	MAPKAPK3	EEF2K	[activation]
6694	HCLS1	NOTCH1	[activation]
6695	RELA	COMMD5	[activation]
6696	PTK6	FGFR1	[activation]
6697	ANKRD13B	EGFR	[activation]
6698	PPP1CC	ENKD1	[inhibition]
6699	TIAF1	JAK3	[inhibition]
6700	STX4	RAB4A	[activation]
6701	IQCK	NCOA3	[activation]
6702	MAPKAPK3	SRC	[activation]
6703	CDK1	FYN	[activation]
6704	RAB7A	HLA-B	[activation]
6705	SRSF1	MDM2	[activation]
6706	TRAF6	PRPSAP1	[activation]
6707	SNW1	CDC23	[activation]
6708	NR3C1	PRKDC	[activation]
6709	CDK11B	URM1	[activation]
6710	BMPR1B	RAN	[activation]
6711	PAG1	VAV1	[activation]
6712	PTPN1	FAM84B	[activation]
6713	TNFRSF17	TRAF3	[activation]
6714	CAPN1	TP53	[activation]
6715	RAP1B	RAB7B	[activation]
6716	CDH1	CDC27	[activation]
6717	TNFRSF14	HDAC3	[activation]
6718	STK4	H2AFX	[activation]
6719	HSPA8	ERH	[inhibition]
6720	ACTG1	VASP	[activation]
6721	GEM	BMP1	[activation]
6722	CSNK2B	MGEA5	[activation]
6723	IL24	KRAS	[activation]
6724	EIF3F	EIF4G2	[activation]
6725	PRKCQ	ICAM3	[activation]
6726	RAB9A	BCL3	[activation]
6727	HIST1H2BN	OBSL1	[activation;inhibition]
6728	CDC5L	ANAPC2	[activation]
6729	NCOA1	BCL3	[activation]
6730	ATP4A	GNAQ	[activation]
6731	EP300	DYRK1B	[activation]
6732	MYLK	SVIL	[inhibition]
6733	LOX	COL1A1	[activation]
6734	EIF2B2	DCC	[activation]
6735	TRAF1	CCHCR1	[activation]
6736	LAMB1	COL6A1	[activation]
6737	IL16	MYC	[activation]
6738	VCAM1	PPIL1	[activation]
6739	PPP2CA	FOXO3	[inhibition]
6740	CDC42	PLEKHG2	[activation]
6741	PIK3CD	IL4	[activation]
6742	PIK3R1	ID4	[activation]
6743	ARF6	CS	[activation]
6744	PVR	CD226	[activation]
6745	SRSF10	SRSF4	[activation]
6746	NXF1	SCRIB	[activation]
6747	SRPK1	DGCR8	[activation]
6748	RPA1	BRCA1	[activation]
6749	PTPRC	MET	[activation]
6750	CAV1	GRK1	[activation]
6751	VEPH1	SMAD1	[activation]
6752	RAB11B	H2AFX	[activation]
6753	SDC4	SDC2	[activation]
6754	CDKN1B	CKS1B	[inhibition]
6755	ST14	CHEK2	[activation]
6756	GPS2	DBNL	[inhibition]
6757	HNF4A	RAD50	[activation]
6758	IKBKB	CDKN2A	[activation;inhibition]
6759	PAK2	SYN1	[activation]
6760	HOXD9	BARX1	[activation]
6761	GSK3B	LUC7L2	[activation;inhibition]
6762	CSNK2B	ATF1	[activation]
6763	ELAVL1	B4GALT1	[activation]
6764	GEM	AHCYL1	[activation]

6765	CD81	WARS	[activation]
6766	PAXIP1	PPP1CC	[activation]
6767	GRN	SMAD9	[activation]
6768	EIF2S1	PRKRIR	[activation]
6769	APAF1	YWHAE	[activation]
6770	PDPK1	CDAN1	[activation]
6771	KLF10	SP1	[activation]
6772	ABL1	GAB3	[activation]
6773	RAB6B	TGFBR1	[activation]
6774	SMAD4	SASH3	[activation]
6775	DYRK1A	RBL1	[activation;inhibition]
6776	BARD1	LRRC41	[activation]
6777	ATP7A	CP	[activation]
6778	MYBL2	LIN9	[inhibition]
6779	GRN	SORT1	[activation]
6780	GNAI2	NOTCH2NL	[activation]
6781	PIK3R1	ARAF	[activation]
6782	GNAI2	F2R	[activation]
6783	GRB2	YWHAE	[activation]
6784	HSP90AA1	CDK6	[activation]
6785	UBC	MAPK14	[activation]
6786	PRKCE	BAX	[activation]
6787	GABARAPL1	F7	[activation]
6788	POLD1	ABL1	[activation]
6789	PRDX2	STK4	[activation]
6790	KITLG	CLEC11A	[activation]
6791	ATF2	HSPD1	[activation]
6792	TGM1	MDK	[activation]
6793	IRAK4	HIST1H1B	[activation]
6794	USF1	TOP2B	[activation]
6795	UBA5	HSP90B1	[activation]
6796	HNF4A	CTNNB1	[activation]
6797	NRAS	ACVR1	[activation;inhibition]
6798	MDK	STAT1	[activation]
6799	YWHAH	TSC2	[activation;inhibition]
6800	CTNNB1	DDX1	[activation]
6801	IGHA1	UNC79	[activation]
6802	BIRC5	CASP3	[activation]
6803	NFE2L2	BPTF	[activation]
6804	YWHAG	PPP1R3D	[activation]
6805	PPP3CA	GABRG2	[activation]
6806	DAB2IP	TNFRSF1A	[activation]
6807	SOCS2	SOCS1	[inhibition]
6808	PRKRA	SETDB1	[activation]
6809	PRRC2B	EHMT2	[activation]
6810	MAPKAPK5	GYS1	[activation]
6811	PAFAH1B1	KATNB1	[activation]
6812	DNAJA1	PTP4A3	[inhibition]
6813	CAV1	MALL	[activation]
6814	DVL3	CSNK1D	[activation]
6815	LUC7L2	LPL	[activation]
6816	PRR5	MLST8	[activation]
6817	YWHAZ	ANXA1	[activation]
6818	HLA-B	HSPE1	[activation]
6819	PRPSAP1	ASB18	[inhibition]
6820	PKLR	PAK1	[activation]
6821	SNRPA1	HLA-B	[activation]
6822	NME2P1	ATF2	[activation]
6823	PTEN	PPP3CA	[activation]
6824	POLR2B	RECQL5	[activation]
6825	CDC27	RB1	[inhibition]
6826	DHX57	BARD1	[activation]
6827	PDGFRB	CAV1	[activation]
6828	CDK7	CDK19	[activation]
6829	TGFB1	SCGB3A1	[activation]
6830	LGR4	RSPO2	[activation]
6831	FANCC	EIF2AK2	[activation]
6832	BCL2L11	FGL1	[activation]
6833	PDGFRB	ERRFI1	[activation]
6834	DBR1	EGFR	[activation]
6835	PDCD10	MST4	[activation]
6836	PTPN1	ASS1	[activation]
6837	ATF2	LDHB	[activation]
6838	CDC5L	GNAS	[activation]
6839	RSU1	ILK	[activation]
6840	RAI1	RPS6KA5	[activation]

6841	MAP3K1	DNAJB2	[inhibition]
6842	GRB2	CSF3R	[activation]
6843	GRK5	AKT2	[activation]
6844	ANAPC5	NOTCH1	[activation]
6845	ANXA7	CDK4	[activation]
6846	CDCA2	CDK1	[activation]
6847	PLCG1	ABI1	[activation]
6848	SUMO1	ARPC1B	[activation]
6849	HOXD13	SMAD5	[activation]
6850	CALM1	PCK1	[activation]
6851	VDAC3	OBSL1	[inhibition]
6852	KCNB1	SRC	[activation]
6853	MAP2K1	CFLAR	[activation]
6854	CSNK1E	AXIN2	[activation]
6855	CMTM3	BTK	[activation]
6856	ILVBL	ILK	[activation]
6857	PIAS1	SMAD7	[inhibition]
6858	GNAI2	LPAR3	[activation]
6859	VCAM1	NUP155	[activation]
6860	HGS	MAP3K7	[activation]
6861	AASDHPPT	PAXIP1	[activation]
6862	PTPRC	PPFIA1	[activation]
6863	UBE2Q2	ACTG1	[activation]
6864	EGFR	SLC7A5	[activation]
6865	ZFYVE9	STX3	[activation]
6866	PRNP	CNTN1	[activation]
6867	ATF2	PGAM1	[activation]
6868	EIF4E	EIF4EBP1	[inhibition]
6869	VAV1	SHB	[activation]
6870	PCDHA9	CRK	[activation]
6871	ITGAV	MMP2	[activation]
6872	PRKRA	TP53	[activation]
6873	TRIP6	TLR2	[activation]
6874	SMARCC1	STAT2	[activation]
6875	MDM2	ERBB3	[activation]
6876	HNF4A	PPARGC1A	[activation]
6877	NFYC	CSN2	[activation]
6878	PPFIA1	CDKN1A	[activation;inhibition]
6879	DVL2	WAS	[activation]
6880	GAPDH	BID	[activation]
6881	CDC5L	KPNB1	[activation]
6882	ACVR1	KLHL35	[activation;inhibition]
6883	APBB1	HDAC1	[activation]
6884	EEF2	CDKN2A	[activation;inhibition]
6885	CTNNB1	NXF1	[activation]
6886	F2	KNG1	[activation]
6887	CD2	SH3KBP1	[activation]
6888	REL	HSPA1L	[inhibition]
6889	NCF1	CTTN	[activation]
6890	PML	MAPK11	[activation]
6891	ICMT	RAC2	[activation]
6892	CRK	F2RL2	[activation]
6893	BMPR2	NOP56	[activation]
6894	SNAP25	HTT	[activation]
6895	DLGAP3	PTK2	[activation]
6896	DDX39B	SUMO3	[activation]
6897	ATG5	GXYLT1	[activation]
6898	EXOC5	EXOC3	[inhibition]
6899	YWHAZ	KCNK15	[activation]
6900	CIAO1	RTEL1	[activation]
6901	ULK2	MAPK3	[activation]
6902	MAPRE1	SPDYE2	[activation]
6903	NCOA3	RELA	[activation]
6904	AGAP2	INSR	[activation]
6905	MAFK	NFE2L2	[activation]
6906	SCYL3	TXN2	[inhibition]
6907	RHOA	DGKQ	[activation]
6908	E2F1	KAT5	[activation]
6909	SLFN5	NOTCH1	[activation]
6910	YWHAQ	TP53	[activation]
6911	CRK	AGPAT4	[activation]
6912	OPRD1	OPRM1	[activation]
6913	RAC1	IQGAP1	[activation]
6914	TGM4	IGHA1	[activation]
6915	CHEK2	LYPD3	[activation]
6916	COL1A2	VWF	[activation]

6917	DOK1	FES	[activation]
6918	CD81	CNN2	[activation]
6919	MYC	KIAA0319	[activation]
6920	ARFGEF3	PTH	[activation]
6921	APLP1	EIF2S2	[activation]
6922	CFTR	PRKG1	[inhibition]
6923	ATF4	KAT2B	[activation]
6924	PIK3R1	SOCS1	[activation]
6925	GRB2	HSPA5	[activation]
6926	GNAO1	S1PR5	[activation]
6927	RHOQ	UBC	[activation]
6928	FAM46A	PSG3	[activation]
6929	CCDC120	CCDC102B	[activation]
6930	RAB11B	APP	[activation]
6931	PPIG	RHOB	[activation]
6932	ZBTB17	ESR1	[activation]
6933	PTK2B	SKAP2	[activation]
6934	SORBS1	EFNB1	[activation]
6935	LCK	CD4	[activation]
6936	KMT2A	CXXC1	[activation]
6937	ERBB2	PLXNB1	[activation]
6938	NFASC	SDCBP2	[activation]
6939	MYC	BTAF1	[activation]
6940	PACSIN3	APBB1IP	[activation]
6941	GRAP2	ASAP1	[activation]
6942	TNFSF11	PHAX	[activation]
6943	ECT2	TP53	[activation]
6944	EGFR	SYN1	[activation]
6945	ACACA	BRCA1	[activation;inhibition]
6946	DSG1	GABARAPL1	[activation]
6947	NLK	PASK	[inhibition]
6948	NFYC	GLTSCR1L	[activation]
6949	MTOR	GNB1	[activation]
6950	GRB2	PTBP1	[activation]
6951	RPA3	NTPCR	[activation]
6952	XPO7	NXF1	[activation]
6953	TRIM24	ESR1	[activation]
6954	TNF	RAB5A	[activation]
6955	RANBP2	PML	[activation]
6956	YWHAH	SNCA	[activation]
6957	LILRB2	FCGR1A	[activation]
6958	CAPN2	F2RL1	[activation]
6959	DAB1	LRP1	[activation]
6960	MAPK1	ITGB6	[activation]
6961	HSPA4	PRKCB	[activation]
6962	WHSC1	SMARCA5	[activation]
6963	SYNGAP1	ULK1	[inhibition]
6964	WHSC1L1	CASP8	[activation]
6965	NCK2	ERBB2	[activation]
6966	TNS1	GAB1	[activation]
6967	YWHAZ	SAV1	[activation]
6968	PML	NR3C1	[activation]
6969	DAB2IP	GSK3B	[inhibition]
6970	EIF4A3	SUMO1	[activation]
6971	PICALM	CASP8	[activation]
6972	ESR1	CXCL1	[activation]
6973	STRA13	FANCA	[activation]
6974	BTRC	TIAM1	[activation]
6975	NTHL1	RBL1	[activation]
6976	SLC25A13	GRB2	[activation]
6977	RAD21	SUMO1	[activation]
6978	CFLAR	TICAM1	[inhibition]
6979	CSNK1G1	FAM219A	[activation]
6980	PTPN11	CXCR4	[activation]
6981	IRS4	YWHAB	[activation]
6982	GNAI3	VCAM1	[activation]
6983	EIF2AK3	HSP90AA1	[activation]
6984	CDK1	FANCC	[activation]
6985	HOXD9	ETV5	[activation]
6986	TCF20	MAPK14	[activation]
6987	CTNNA1	PTPN14	[activation]
6988	YWHAZ	HSPA1A	[inhibition]
6989	BRCA2	CDK1	[activation]
6990	DOK1	ABL2	[activation]
6991	NFATC2	RIPK2	[activation]
6992	KSR1	MARK3	[activation]

6993	ANXA13	KIFC3	[activation]
6994	CBL	SOS1	[activation]
6995	CDC25B	CSNK2A1	[activation]
6996	SUMO3	EEA1	[activation]
6997	SPP1	UBQLN4	[activation]
6998	CEP170P1	ABI3	[activation]
6999	VEGFA	HSPA4	[activation]
7000	PRLR	NEK3	[inhibition]
7001	CCR5	CD4	[activation]
7002	SETD7	TBP	[activation]
7003	APP	PIK3R5	[activation]
7004	MAP1LC3A	MIF	[activation]
7005	RAD17	PRKDC	[activation]
7006	DLG4	PTK2B	[activation]
7007	ITGA2B	FGA	[activation]
7008	SRPK1	MRPS11	[activation]
7009	ESR1	JUNB	[activation]
7010	CD36	COL1A1	[activation]
7011	ESR2	ACTN3	[activation]
7012	TP63	YAP1	[activation]
7013	CCND1	TSTD2	[inhibition]
7014	RAB2A	PRKCI	[activation]
7015	RHOB	FNTA	[activation]
7016	RARA	FAS	[inhibition]
7017	SRPK1	EIF4A3	[activation]
7018	TP53	CHD8	[activation]
7019	BCL6	WNK4	[activation]
7020	SIRT3	NCAPD2	[activation]
7021	IGHG3	IKBKG	[activation]
7022	ITGB2	RDX	[activation]
7023	TRAPPC8	PAXIP1	[activation]
7024	PTPRC	PTPRCAP	[activation]
7025	HIST1H2BC	ICAM1	[activation]
7026	MEA1	MDM2	[activation]
7027	APP	PLK2	[activation]
7028	RPS6KB1	CSNK2B	[activation]
7029	CD247	GAB2	[activation]
7030	PDZD11	ATP7A	[activation]
7031	HLA-B	TKT	[activation]
7032	EP300	SAV1	[activation]
7033	KRIT1	ITGB1BP1	[activation]
7034	DUSP10	HTT	[inhibition]
7035	TMEM173	STAT6	[activation]
7036	APOA1	AKT2	[inhibition]
7037	CREBBP	STAT6	[activation]
7038	NANS	EGFR	[activation]
7039	NLK	TFDP1	[inhibition]
7040	MAPK6	PSIP1	[activation]
7041	MAP4K1	LRRK1	[activation]
7042	DNA2	NXF1	[activation]
7043	PLD2	SRC	[activation]
7044	GREB1	PALB2	[activation]
7045	MDM2	GSN	[activation]
7046	TCEB3B	CDK6	[inhibition]
7047	MDM2	HNRNPAB	[activation]
7048	NCCRP1	ESR1	[activation]
7049	HSP90AA1	PRKCQ	[activation]
7050	BRD7	HIST1H2AA	[activation]
7051	SPINT1	FAM46A	[inhibition]
7052	SUMO1	GNAS	[activation]
7053	CREBBP	MKNK1	[activation]
7054	IGF1R	GNAI1	[activation]
7055	RPTOR	AMBRA1	[activation]
7056	IKBKB	PPARG	[activation]
7057	CDK6	CBY1	[inhibition]
7058	PELI1	SRPK1	[activation]
7059	GRB7	RND1	[activation]
7060	ATOX1	FAM118A	[activation]
7061	PPP2R4	NXF1	[activation]
7062	PTGER4	ARRB1	[activation]
7063	GAB1	TNS3	[activation]
7064	BTK	PIP5K1A	[activation]
7065	MAP4K1	HCLS1	[activation]
7066	SHC1	TEC	[activation]
7067	RHPN2	HLA-C	[activation]
7068	CDKN1A	NKD2	[inhibition]

7069	DAG1	VCL	[activation]
7070	PARVB	CDC5L	[activation]
7071	GRB2	ITGB4	[activation]
7072	USP7	TRAF3	[activation]
7073	TRAF2	TNFSF9	[activation]
7074	PPP6C	ESR2	[activation]
7075	DLG5	TGFBR1	[activation]
7076	HIST2H2BE	GZMA	[activation]
7077	DMC1	RAD51AP1	[activation]
7078	UBC	NSD1	[activation]
7079	CDC23	PNMA5	[activation]
7080	CASP9	NOD1	[activation]
7081	PAXIP1	NOS3	[activation]
7082	HNRNPA1	ARHGAP23	[activation]
7083	ARRB1	PFKFB3	[activation]
7084	PRKCD	MAPT	[activation]
7085	CLTA	TP53	[activation]
7086	GRB2	TSKS	[activation]
7087	TP53	GSN	[activation]
7088	TJP1	GJB3	[activation]
7089	EP300	TGFB1I1	[activation]
7090	GKN2	TFF1	[activation]
7091	PBX3	AGTRAP	[activation]
7092	NRIP1	ARPC3	[activation]
7093	SH2D3C	KIT	[activation]
7094	SRC	PDPK1	[activation]
7095	S1PR4	GNAI1	[activation]
7096	ESR2	GNB3	[activation]
7097	NR3C1	PRKACA	[activation]
7098	ARF1	GOSR2	[activation]
7099	NR3C1	SRSF5	[activation]
7100	ID1	GATA4	[activation]
7101	SHC1	SUV39H2	[activation]
7102	HSP90AB1	TNK1	[activation]
7103	DACH1	SMAD4	[inhibition]
7104	GABRG3	GABARAP	[activation]
7105	GNPAT	HSPB1	[activation]
7106	CUL3	PPP2CA	[inhibition]
7107	H2AFX	PRDX3	[activation]
7108	HSPA6	TRAF3IP1	[activation]
7109	GAPDH	CAMK4	[inhibition]
7110	RAD21	BRCA2	[activation]
7111	PRKCZ	GABARAP	[activation]
7112	TOLLIP	IRAK2	[activation]
7113	ARPC2	GRB2	[activation]
7114	PRPF38A	SRPK2	[activation]
7115	LAMC2	BMP1	[activation]
7116	CEP170B	PRKAA2	[inhibition]
7117	GDAP1	NXF1	[activation]
7118	BIRC2	TNFRSF12A	[activation]
7119	LIG1	CSNK2A1	[activation]
7120	PRKAB1	CYCS	[inhibition]
7121	MAP3K12	EGFR	[activation]
7122	RHOBTB2	CHEK2	[activation]
7123	EXOC6	APP	[activation]
7124	NUMB	BMP2K	[activation]
7125	ABL2	ABI1	[activation]
7126	BMPR1A	CTSV	[activation;inhibition]
7127	CAV1	MAPK1	[activation]
7128	CCDC50	RIPK1	[activation]
7129	CDK1	MDM4	[activation;inhibition]
7130	TTLL4	NXF1	[activation]
7131	CIB1	PAK1	[activation]
7132	SMAD2	ESR2	[activation]
7133	TREM1	TYROBP	[activation]
7134	CDC5L	PUM1	[activation]
7135	PPP2CA	SCHIP1	[inhibition]
7136	GOSR2	STX5	[activation]
7137	SPATA2	GNAL	[activation]
7138	EFNA3	MEOX2	[activation]
7139	HCK	DOK2	[activation]
7140	CD19	GRB2	[activation]
7141	ICT1	NGRN	[activation]
7142	KRAS	OSGIN1	[activation]
7143	CSF1	PIK3R2	[activation]
7144	CD4	KCNAB2	[activation]

7145	HSPH1	MAP3K1	[activation]
7146	PAFAH1B3	VCAM1	[activation]
7147	EP300	SIRT1	[activation]
7148	CSMD2	ABL1	[activation]
7149	SOX2	CASP8	[activation]
7150	SAV1	YWHAH	[activation]
7151	NFYC	KAT2B	[activation]
7152	NOD2	TRAF6	[activation]
7153	VCAN	CCL5	[activation]
7154	CDH15	CDH9	[activation]
7155	SRPK1	METTL14	[activation]
7156	TEAD1	MEF2A	[activation]
7157	RPL31	TP53	[activation]
7158	TIAL1	RPA2	[activation]
7159	RIPK2	TRAF6	[activation]
7160	ANGPTL4	CASP8	[activation]
7161	GRB2	CDC42	[activation]
7162	PRMT7	H3F3C	[activation]
7163	MAPK13	PFN1	[activation]
7164	KAT2B	HSD11B2	[activation]
7165	MUC7	LAMA5	[activation;inhibition]
7166	PTK2B	MAP3K4	[activation]
7167	GNB2L1	STAT1	[activation]
7168	CBR1	GRB2	[activation]
7169	PIK3CA	PSMC3IP	[activation]
7170	CALM1	RDX	[activation]
7171	YWHAZ	EIF4E2	[activation]
7172	MAP4K2	RAB8A	[activation]
7173	CD81	JAK3	[activation]
7174	LGALS4	ARPC3	[activation]
7175	CAMK1	APP	[activation]
7176	CAPN1	VIM	[activation]
7177	BIRC2	RAC1	[activation]
7178	PSD2	PMS1	[activation]
7179	SP1	PER3	[activation]
7180	TRAF6	TRAF2	[activation]
7181	PPP3CC	CABIN1	[inhibition]
7182	PTK2	SOCS2	[inhibition]
7183	BUB3	ITGA4	[activation]
7184	PI4KA	MTNR1B	[activation]
7185	NLGN1	NRXN2	[activation]
7186	PON2	HTR2A	[activation]
7187	FCN1	TGFB1	[activation]
7188	HSPA2	FKBPL	[activation]
7189	SYK	TICAM1	[activation]
7190	RPL35A	GRB2	[activation]
7191	POLA2	RAE1	[activation]
7192	P2RX2	GABRR1	[activation]
7193	SMAD2	FBP2	[activation]
7194	CTNNB1	DOT1L	[activation]
7195	FOXO3	CREBBP	[activation]
7196	NSD1	NR6A1	[activation]
7197	HSPB1	NVL	[activation]
7198	C9orf156	TGFBR2	[activation;inhibition]
7199	F8	F2	[activation]
7200	CAV1	RPA2	[activation]
7201	JAK2	PPP2R4	[activation]
7202	TLN1	EGFR	[activation]
7203	ABL1	F2RL2	[activation]
7204	CAV1	NOS3	[activation]
7205	POU5F1	NCL	[activation]
7206	SFN	FAM53C	[activation]
7207	ARRB2	STC2	[inhibition]
7208	RAC3	NRBP1	[activation]
7209	RPA3	EPRS	[activation]
7210	ACVRL1	INHBA	[inhibition]
7211	RADIL	RAD51D	[activation]
7212	LYN	DVL2	[activation]
7213	XPO7	NUP153	[activation]
7214	RXRA	BHLHE41	[inhibition]
7215	RAD21	RPS17	[activation]
7216	GSK3B	PRKACA	[activation]
7217	MYO9A	NXF1	[activation]
7218	TNFSF14	LTB	[activation]
7219	L1CAM	NCAM1	[activation]
7220	HNF4G	SRC	[activation]

7221 NGFR TRAF3 [activation]
7222 GLUL HLA-B [activation]
7223 ERBB4 SYK [activation]
7224 PTK2B MCAM [activation]
7225 NXF1 HSP90AA1 [activation]
7226 CDC42BPB TJP1 [activation]
7227 AR BLK [activation]
7228 MAPK14 BAZ1B [activation]
7229 GADD45G PTN [activation]
7230 NR2F1 ISL1 [activation]
7231 HSP90AB1 BRAF [activation]
7232 CTR9 RTF1 [activation]
7233 BCL2A1 BCL2L11 [activation]
7234 DKC1 EPB41L3 [activation]
7235 PRKCZ PARD6G [activation]
7236 FOXJ1 SLC37A3 [inhibition]
7237 H2AFX TP53 [activation]
7238 CNOT1 GRB2 [activation]
7239 NFATC2 HNRNPA1 [activation]
7240 DNAJA2 DNAJA4 [inhibition]
7241 DPYSL2 TNIK [activation]
7242 YWHAE TIAM1 [activation]
7243 SLIT2 ETS1 [activation]
7244 GRB2 FSCN1 [activation]
7245 HSD17B3 BRAF [activation]
7246 POLK RPA1 [activation]
7247 ABI2 SH3KBP1 [activation]
7248 SERPINE1 UBQLN4 [inhibition]
7249 NCK2 SRPK1 [activation]
7250 PRKCA SHC1 [activation]
7251 RAD51 TP53 [activation]
7252 EGFR APP [activation]
7253 EP300 IL8 [activation]
7254 SNCA CSNK1D [activation]
7255 ARRB1 BTK [activation]
7256 MAPK1 CRP [activation]
7257 LRRIQ3 LYN [activation]
7258 CASC3 GRB2 [activation]
7259 ANAPC10 CDC20 [inhibition]
7260 LARS RRAGD [activation]
7261 CR1 CD55 [inhibition]
7262 CD40 RIPK2 [activation]
7263 KCNA4 NEU1 [activation]
7264 PTP4A3 MYO1B [activation]
7265 MAP3K1 CRTC1 [activation]
7266 ADORA2A NAMPT [activation]
7267 CASP8 SRF [activation]
7268 ADCY5 RGS2 [activation]
7269 CDK3 CCNE1 [activation]
7270 SIGIRR IRAK1 [activation;inhibition]
7271 SP1 E2F2 [activation]
7272 CDC42SE1 CDC42 [activation]
7273 PPP1R15A HSPA1A [inhibition]
7274 TRAP1 RAD21 [activation]
7275 CISH KIT [inhibition]
7276 CDK19 MED1 [activation]
7277 NUP62 CCDC153 [activation]
7278 DAAM1 RHOD [activation]
7279 ITGAM ELANE [activation]
7280 MYC KIF25 [activation]
7281 FAS C14orf1 [inhibition]
7282 ABL2 SRC [activation]
7283 WEE1 ARRB2 [inhibition]
7284 BNIPL CDC42 [activation]
7285 RAB10 EPB41 [activation]
7286 NBN PAXIP1 [activation]
7287 EPAS1 PIAS2 [inhibition]
7288 GH1 CALM1 [activation]
7289 MT1A GPR50 [activation]
7290 L1CAM ITGA5 [activation]
7291 GSTP1 MAPK14 [activation]
7292 TNF HSPB1 [activation]
7293 GAB3 PTPN11 [activation]
7294 SFN KIF23 [activation]
7295 CD36 SRC [activation]
7296 DNAJA3 STAT1 [inhibition]

7297	MYC	HDAC1	[activation]
7298	ZC3H12A	EP300	[activation]
7299	MCM3	HIF1A	[activation]
7300	BTRC	CCNB1	[inhibition]
7301	PLAUR	ITGB1	[activation]
7302	CHMP2A	ITGA4	[activation]
7303	TRIM41	SRPK2	[activation]
7304	IL13RA2	IGF1R	[activation]
7305	WWTR1	HIF1A	[activation]
7306	TNFRSF14	NCLN	[activation]
7307	CHEK2	PPP2CA	[activation]
7308	PIK3R3	GRB2	[activation]
7309	MYB	LIN9	[activation]
7310	LRRK2	NOMO2	[activation]
7311	ORAI3	ORAI1	[activation]
7312	CCND3	RBL2	[inhibition]
7313	STK16	NOTCH2NL	[activation]
7314	GRIP1	NCOA1	[activation]
7315	EGFR	CDC37	[activation]
7316	RAD51B	HIST1H4A	[activation]
7317	TNF	SHARPIN	[activation]
7318	YWHAB	TANC2	[activation]
7319	NR3C1	PRPF6	[activation]
7320	ELK1	HDAC1	[activation]
7321	BMP8B	FYN	[activation]
7322	PDGFRB	CRK	[activation]
7323	CAMK2G	PLCB3	[activation]
7324	PRKCG	NFE2L2	[activation]
7325	ANXA9	APP	[activation]
7326	ACVR2B	BMP2	[activation]
7327	ESR1	CAPZB	[activation]
7328	EEF2K	RPS6KB2	[activation]
7329	CYTH2	GNAQ	[activation]
7330	MYC	HSPH1	[activation]
7331	MLLT4	GRB2	[activation]
7332	RFC1	RAD50	[activation]
7333	ILK	S100A9	[activation]
7334	BCAR1	NEDD9	[activation]
7335	DNM2	APPL1	[activation]
7336	RAB11FIP1	YWHAB	[activation]
7337	TP53	UBE2K	[activation]
7338	PAX8	WBP2	[activation]
7339	CXCL5	MMP9	[activation]
7340	HIST2H2BE	GADD45A	[activation;inhibition]
7341	AGR2	MLH1	[activation]
7342	FCGR3A	CD38	[activation]
7343	LEF1	PIAS4	[inhibition]
7344	PLCB1	TRPC3	[activation]
7345	TNFRSF1A	ACTG1	[activation]
7346	CAPN1	CLEC4G	[activation]
7347	ATM	ZNF821	[activation]
7348	GRB2	CALD1	[activation]
7349	SMAD2	SEMA6A	[activation]
7350	SH3KBP1	DAB1	[activation]
7351	NOTCH2NL	CCDC26	[activation]
7352	CD48	GNAI3	[activation]
7353	SRC	PPARA	[activation]
7354	EIF4A1	SUMO1	[activation]
7355	FN1	ACACA	[activation;inhibition]
7356	PYHIN1	MDM2	[activation]
7357	BMPR1B	IGSF1	[activation]
7358	SH2B2	INSR	[activation]
7359	RB1	NDC80	[inhibition]
7360	TNFRSF14	VIM	[activation]
7361	MADCAM1	ITGA4	[activation]
7362	SP100	NXF1	[activation]
7363	RADIL	CDK2	[activation]
7364	CDK5R1	CHN1	[activation]
7365	YWHAZ	GP5	[activation]
7366	AXIN2	AXIN1	[activation]
7367	MAP2K1	BRAP	[activation]
7368	RXRA	RNF8	[inhibition]
7369	LAT	CREB3	[activation]
7370	ERBB2	SH2D5	[activation;inhibition]
7371	KIT	SH3BP2	[activation]
7372	GRB2	INCA1	[activation]

7373	LRRK2	DAPK1	[activation]
7374	IGHG1	SWAP70	[activation]
7375	DYRK1A	SMAD3	[activation]
7376	ITGB1	NME1	[activation]
7377	SUV39H1	LENG8	[activation]
7378	AR	CCNH	[activation]
7379	CDK2	BCL2	[inhibition]
7380	IL12A	IL8	[activation]
7381	IGHG1	RTKN2	[activation]
7382	KNG1	GP1BA	[activation]
7383	KCNK5	FRAT1	[activation]
7384	TP53	BAIAP2L1	[activation]
7385	TCL1A	JUN	[activation]
7386	MAPKAPK5	EIF4EBP1	[activation]
7387	NOS1	PTPRN	[activation]
7388	RAN	IKBKB	[activation]
7389	ICT1	DHX30	[activation]
7390	PTEN	XIAP	[activation]
7391	TULP4	SRC	[activation]
7392	LCK	AJUBA	[inhibition]
7393	TEC	PIK3R3	[activation]
7394	LARS	VCAM1	[activation]
7395	MLH1	CUTA	[activation]
7396	RXRG	PPARA	[activation]
7397	ESR1	PTEN	[activation]
7398	RPL14	PCK1	[activation]
7399	SERPINE2	COL4A3	[inhibition]
7400	CXCL9	SYNE4	[activation]
7401	SRPRB	TNFRSF14	[activation]
7402	ENO1	HSPA8	[inhibition]
7403	SYT4	STX3	[activation]
7404	NCL	FOS	[activation]
7405	MYBL2	CCNE1	[inhibition]
7406	DFFA	HSPB1	[activation]
7407	ERBB2IP	CTSG	[inhibition]
7408	FOS	CABP1	[activation]
7409	ECE1	KNG1	[activation]
7410	GRB2	REPS1	[activation]
7411	CDK11A	EIF3F	[activation]
7412	PRKAR1A	PYCARD	[inhibition]
7413	GCK	PFKFB1	[activation]
7414	GRIP1	PPARG	[activation]
7415	PRKAB2	MAP3K6	[inhibition]
7416	CRK	CBL	[activation]
7417	EGFR	CAMK2G	[activation]
7418	RASGRF1	RRAS2	[activation]
7419	MRE11A	MDC1	[activation]
7420	MME	ADCYAP1	[activation]
7421	KIF23	RACGAP1	[activation]
7422	HIST1H1A	IRAK4	[activation]
7423	ENPP2	LPAR1	[activation]
7424	SMARCC1	GRB2	[activation]
7425	PRKAB2	GATA1	[activation]
7426	SUFU	GLI2	[activation]
7427	MYD88	SASH1	[activation;inhibition]
7428	ERBB3	PTK2B	[activation]
7429	PRKCZ	YWHAZ	[activation]
7430	YWHAE	NGFRAP1	[activation]
7431	STX5	ASNA1	[activation]
7432	FYN	TGOLN2	[activation]
7433	MDC1	FANCD2	[activation]
7434	SFN	ARHGEF16	[activation]
7435	CDC16	EP300	[activation]
7436	CCDC33	PTK6	[activation]
7437	PRDX3	MDM2	[activation]
7438	GRK5	ARF1	[activation]
7439	COA7	MDC1	[activation]
7440	CDKN1A	TMSB4X	[activation]
7441	RALA	PPP2R1B	[activation]
7442	MVP	GIT2	[activation]
7443	TYK2	CRKL	[activation]
7444	HSP90AB1	NPR2	[activation]
7445	CTR9	HIST1H3A	[activation]
7446	DNAJA1	OBSL1	[inhibition]
7447	WAS	BTK	[activation]
7448	RCC1	RAN	[activation]

7449	STK11	EEF1A2	[activation]
7450	ARRB2	GPR56	[activation]
7451	MYC	PKM	[activation]
7452	SRPK1	FLT1	[activation]
7453	STX3	SYT7	[activation]
7454	VSNL1	CHRNA4	[activation]
7455	TFRC	ACAP1	[activation]
7456	LPA	APOH	[activation]
7457	SFN	CCNH	[activation]
7458	DDA1	EIF5	[activation]
7459	DOCK8	FAM124A	[activation]
7460	YWHAB	STK3	[activation]
7461	CDK6	PPM1B	[inhibition]
7462	KLF5	CREBBP	[activation]
7463	SIRT1	NHLH2	[activation]
7464	MAPKBP1	MAPK8	[activation]
7465	FBXO6	PRF1	[activation]
7466	PRKCB	ANXA2	[activation]
7467	ITGA4	CAPN1	[activation]
7468	KIR2DL4	FCER1G	[activation]
7469	MET	SOCS3	[activation;inhibition]
7470	GABARAPL2	GOSR1	[activation]
7471	BRPF3	PIK3R1	[activation]
7472	CREBBP	MYBL2	[activation]
7473	EIF4G1	MDM2	[activation]
7474	RALA	RALGPS1	[activation]
7475	LRP6	MESDC2	[inhibition]
7476	DLG5	CTNNB1	[activation]
7477	ACKR2	CCL5	[activation]
7478	ARL8B	DLEU1	[activation]
7479	EZH1	GATA4	[activation]
7480	OPRK1	VAPA	[activation]
7481	SRPK2	VAV2	[activation]
7482	CDH1	STX17	[activation]
7483	ZAP70	PLCG1	[activation]
7484	ERBB2	PIK3C2A	[activation]
7485	EIF4G2	VCAM1	[activation]
7486	STAG2	SGOL1	[activation]
7487	ERCC4	SLX4IP	[activation]
7488	GRB2	PIK3C2B	[activation]
7489	CLDN4	WNK4	[activation]
7490	RPS6KA1	HSP90AB1	[activation]
7491	MCM7	LYN	[activation]
7492	KLF5	PRKCA	[activation]
7493	IQGAP1	CDC5L	[activation]
7494	CAMK2A	LRRC7	[activation]
7495	VPS35	VEGFA	[activation]
7496	PDPK1	YWHAH	[activation]
7497	DOK5	EGFR	[activation]
7498	CDH2	PTPRJ	[activation]
7499	CEACAM1	PTPN11	[activation]
7500	ABL1	ERBB4	[activation]
7501	SRPK1	LUC7L	[activation]
7502	RAN	NR4A2	[activation]
7503	KMT2D	ESR1	[activation]
7504	AKAP8	CCND1	[activation]
7505	CRK	DNM2	[activation]
7506	GUCY1A3	MAPK8	[activation]
7507	EPB41L3	CADM1	[activation]
7508	GAP43	PRKCB	[activation]
7509	TGFB1	MTA3	[activation]
7510	STX7	SNAP29	[activation]
7511	DUSP3	MAP2K2	[activation]
7512	MBP	MAP3K5	[inhibition]
7513	SMAD3	FLI1	[inhibition]
7514	FAM208B	SCAMP1	[activation]
7515	SPRY1	LCE1B	[inhibition]
7516	ATM	CHD4	[activation]
7517	SRPK2	ZMAT4	[activation]
7518	OGT	MAPT	[activation]
7519	MAPK13	PGK1	[activation]
7520	PRKCH	PRKD1	[activation]
7521	BCL6	MTA3	[activation]
7522	RGS13	GNAQ	[activation]
7523	IQGAP1	LRP6	[activation]
7524	TRAF2	GSK3B	[activation]

7525	U2AF2	GH1	[activation]
7526	DYRK1A	CLASRP	[activation]
7527	ITK	GAB1	[activation]
7528	ORMDL1	APP	[activation]
7529	ERBB2	NUDCD3	[activation;inhibition]
7530	PTAFR	JAK2	[activation]
7531	UMPS	PIK3CA	[activation]
7532	AMER1	APC	[inhibition]
7533	GSTK1	EIF2B5	[activation]
7534	YWHAB	SHKBP1	[activation]
7535	CDC20	EP300	[activation]
7536	MSL2	WNK1	[activation]
7537	VAPA	EGFR	[activation]
7538	DMPK	PLN	[inhibition]
7539	HNRNPA1	ARRB2	[activation]
7540	MYB	SPTAN1	[activation]
7541	EGFR	TPT1	[activation]
7542	HEMGN	EGFR	[activation]
7543	ILK	TRABD	[activation]
7544	TIAL1	MAPK1	[activation]
7545	CAPZB	GRB2	[activation]
7546	MRE11A	AIM2	[activation]
7547	PPM1E	ARHGEF7	[activation]
7548	UBC	ATG7	[activation]
7549	ACTB	MAPT	[activation]
7550	HSPB1	SIK2	[activation]
7551	C11orf87	SRPK1	[activation]
7552	APP	CST2	[inhibition]
7553	MYC	AIFM2	[activation]
7554	SH2D2A	SMAD2	[inhibition]
7555	FGF16	APP	[activation]
7556	VAPA	GABARAPL2	[activation]
7557	CASP1	ATG16L1	[activation]
7558	EXOC1	RALA	[activation]
7559	PAK6	AR	[activation]
7560	HSPB1	EGFR	[activation]
7561	CUTA	GRB2	[activation]
7562	BICD1	MAPK14	[activation]
7563	CTBP1	HIST3H3	[activation]
7564	TYRO3	PROS1	[inhibition]
7565	RELA	IRF1	[activation]
7566	A2M	ENO2	[inhibition]
7567	GABARAPL2	SEC23B	[activation]
7568	NOTCH1	LEF1	[activation]
7569	HSPB1	NFKBIA	[activation]
7570	RAB20	NXF1	[activation]
7571	QIL1	TNFRSF14	[activation]
7572	PLCG1	PLXNB1	[activation]
7573	HLA-A	PRKCA	[activation]
7574	SMARCA4	PPARG	[activation]
7575	TRAF6	RAB10	[activation]
7576	ADRA1B	DRD4	[activation]
7577	EIF4G2	PTP4A3	[activation]
7578	JAKMIP2	CLIP4	[activation]
7579	CENPA	FYN	[activation;inhibition]
7580	NUP43	HLA-B	[activation]
7581	UTP3	DVL3	[activation]
7582	IRAK3	TRAF6	[inhibition]
7583	ANXA5	ATF2	[activation]
7584	RAD51D	LZTS2	[activation]
7585	FYN	ARHGAP32	[activation]
7586	CORO1C	LRCH3	[activation;inhibition]
7587	GNAQ	ADRBK1	[activation]
7588	CDKN1A	CDC5L	[activation]
7589	ARRB2	H2AFX	[activation]
7590	MED1	AR	[activation]
7591	ANG	TNFSF8	[activation]
7592	NCF1	AKT1	[activation]
7593	MCM7	EPAS1	[activation]
7594	GRB2	CYFIP2	[activation]
7595	PTPRD	PTPRF	[activation;inhibition]
7596	PLK1	MAPK6	[activation;inhibition]
7597	SYK	DBNL	[activation]
7598	HSPA6	GABARAPL2	[activation]
7599	GDF9	CDKN2C	[activation]
7600	INS	SYTL4	[activation]

7601	ACTN1	GRB2	[activation]
7602	TAGLN2	OBSL1	[activation;inhibition]
7603	IVL	LOR	[activation]
7604	HNRNPA1	HSP90AB1	[activation]
7605	TP53	EIF5B	[activation]
7606	APP	PSRC1	[activation]
7607	PARP11	NUP98	[activation]
7608	YWHAE	TBK1	[activation]
7609	OSGIN1	DNAJA3	[inhibition]
7610	AURKA	MAPRE3	[activation]
7611	USP10	TP53	[activation]
7612	AURKB	SHARPIN	[activation]
7613	BMI1	TP53	[activation]
7614	VCAM1	HEATR1	[activation]
7615	GPRASP1	CHRM2	[activation]
7616	AKAP1	DYSF	[activation]
7617	SRRM1	SRPK3	[activation]
7618	LAMTOR5	MYOG	[activation]
7619	RASA1	SERPINA4	[inhibition]
7620	HSP90AB1	DDR2	[activation]
7621	PRKAA1	SUPT16H	[inhibition]
7622	HMGN3	SRPK2	[activation]
7623	ERBB2	GNB3	[activation]
7624	CCL14	ACKR2	[activation]
7625	HDAC4	PPP2CA	[inhibition]
7626	FES	JAK2	[activation]
7627	ECT2	CD19	[activation]
7628	PAK2	MYL2	[activation]
7629	CCNB1	CDC6	[activation]
7630	HMGB1	DNM2	[activation]
7631	DNAJC28	PYCARD	[inhibition]
7632	HCK	TNK2	[activation]
7633	RELA	USF2	[activation]
7634	IGSF8	HIST3H3	[activation]
7635	NR3C1	STAT5A	[activation]
7636	CD1D	PTPRC	[activation]
7637	LRRK2	DAPP1	[activation]
7638	PYCARD	IKBKB	[inhibition]
7639	HSPB1	IKBKB	[activation]
7640	CDKN2A	MYL12A	[activation;inhibition]
7641	EGFR	UFL1	[activation]
7642	GRB2	DHX9	[activation]
7643	LAMTOR5	RRAGA	[activation]
7644	CSN3	CSN2	[activation]
7645	MYL1	VCAM1	[activation]
7646	AGTR2	GNAI2	[activation]
7647	MYC	ARF4	[activation]
7648	RASA1	RB1	[activation]
7649	BARD1	EXOC5	[activation]
7650	PPARA	HSP90AA1	[activation]
7651	EGFR	EFNB1	[activation]
7652	SOS1	ABI3	[activation]
7653	SRPK1	DHX8	[activation]
7654	HSPB1	ASB4	[inhibition]
7655	CCDC37	SUV39H1	[activation]
7656	CDKN2A	HSPA4	[inhibition]
7657	BCCIP	EGFR	[activation]
7658	EPB41L3	ARRB2	[activation]
7659	PRKCD	HIST1H1A	[activation]
7660	FADD	PRKCZ	[activation]
7661	ICAM1	HIST1H1A	[activation]
7662	SKAP1	FYB	[activation]
7663	HCK	SKAP2	[activation]
7664	IGF1R	MTA3	[activation]
7665	NUDCD3	KLHL1	[activation]
7666	PAK1	MBP	[activation]
7667	AURKB	STK3	[activation]
7668	HIST1H4A	ESR2	[activation]
7669	EP300	IRF7	[activation]
7670	TSC22D1	A2M	[inhibition]
7671	TSR1	BMPR2	[activation]
7672	RAG1	CRK	[activation]
7673	ACTB	CNN2	[activation]
7674	ICK	CDK20	[activation]
7675	PBK	NXF1	[activation]
7676	SGK1	CD40	[activation]

7677	UVRAG	PTPRA	[activation]
7678	CYP11A1	SMAD2	[activation]
7679	VARS2	PIK3CA	[activation]
7680	ACVR1B	ACVR2A	[activation]
7681	EIF4A3	HIST1H4A	[activation]
7682	NUP153	EGFR	[activation]
7683	NTRK1	FRS3	[activation]
7684	HLA-B	PFN2	[activation]
7685	PLK1	MCM3	[activation]
7686	ERBB3	AGTR2	[activation]
7687	LRRK2	IGKC	[activation]
7688	HUNK	PPP2R1A	[inhibition]
7689	GRN	RPS6KA1	[activation]
7690	APP	LATS1	[inhibition]
7691	PLXNB1	MRAS	[activation]
7692	AURKB	NEDD8	[activation]
7693	NME1	TERF1	[activation]
7694	DAXX	TGFB2	[activation]
7695	KAT2B	HIPK2	[activation]
7696	TRAF6	KPNB1	[activation]
7697	CHUK	TRPC4AP	[activation]
7698	ERBB2IP	APC	[inhibition]
7699	TXNDC5	GMPPB	[activation]
7700	ESR2	CXorf22	[activation]
7701	MLH1	TPRG1L	[activation]
7702	SMAD3	CITED2	[activation]
7703	LZTS2	CDK18	[activation]
7704	CSNK1E	DVL1	[activation]
7705	SDF4	MOK	[activation]
7706	PKM	ITGA4	[activation]
7707	KDR	TIMP3	[activation]
7708	C3	GC	[activation]
7709	NUP214	XPO5	[activation]
7710	CSNK1A1L	JAM2	[activation]
7711	NOXO1	CYBA	[activation]
7712	PDGFA	COL3A1	[activation]
7713	INHA	ACVR2A	[activation]
7714	FZR1	PTTG1	[inhibition]
7715	G3BP2	NFKB1	[activation]
7716	HDX	YWHAE	[activation]
7717	KCNA2	CNTNAP2	[activation]
7718	RHOQ	DOCK8	[activation]
7719	ARHGEF7	PAK2	[activation]
7720	CFL1	CAP1	[activation;inhibition]
7721	CSF2RB	KDR	[activation]
7722	GIT2	PAK1	[activation]
7723	EFNA1	ADAM10	[activation]
7724	MYO1A	SMARCA5	[inhibition]
7725	SEMA6D	PLXNA1	[activation]
7726	CDK1	PTPN1	[activation]
7727	PIK3R1	PPM1A	[activation]
7728	SMAD1	INPP4A	[activation]
7729	PRNP	NHP2	[activation]
7730	NR3C1	KMT2A	[activation]
7731	CRKL	YY1	[activation]
7732	RAP1A	SMARCA4	[activation]
7733	STAT3	FGFR4	[activation]
7734	PRKCB	KIT	[activation]
7735	DLX5	SOX8	[activation]
7736	PTPN6	KDR	[activation]
7737	GAPDH	MAPK1	[inhibition]
7738	CREM	CD34	[activation]
7739	ATP4A	APOH	[activation]
7740	FAM102A	ENKD1	[activation;inhibition]
7741	PRKCB	HIST1H1B	[activation]
7742	NCK2	ABI3	[activation]
7743	EHMT2	ENKD1	[activation]
7744	APP	SMAD3	[activation]
7745	CXCR2	ARRB1	[activation]
7746	ARPC4	ARPC3	[activation]
7747	ANK1	HSPB1	[activation]
7748	BCL2	MAPK3	[activation;inhibition]
7749	SOCS1	TEK	[inhibition]
7750	WASF2	GRB2	[activation]
7751	LCK	WASL	[activation]
7752	SMAD7	AKT1	[activation]

7753	ALK	PXN	[activation]
7754	HSF1	HDAC2	[activation]
7755	ACVR1B	INHBA	[activation]
7756	DDX54	NR3C1	[activation]
7757	RIT1	SRPK2	[activation]
7758	PARD6A	RAC1	[activation]
7759	CDK5RAP3	HSPB1	[activation]
7760	LRRK1	SOS2	[activation]
7761	RCVRN	SMAD3	[inhibition]
7762	LRRK2	HMGN2	[activation]
7763	NXF1	CENPJ	[activation]
7764	NOTCH1	IKZF3	[activation]
7765	SRPK1	LIMK1	[activation]
7766	RBL1	MCM7	[activation]
7767	CDK18	CCDC102B	[activation]
7768	YWHAG	EPB41L3	[activation]
7769	OBSL1	HIST1H2AA	[activation;inhibition]
7770	FES	PTGES3	[activation]
7771	ATF2	PRDX5	[activation]
7772	BUB1	STX17	[activation]
7773	YWHAH	MAST2	[activation]
7774	RBL1	SP1	[activation]
7775	SMAD4	SERPINA1	[inhibition]
7776	HSPB1	C11orf48	[activation]
7777	CEP55	TP53	[activation]
7778	MDC1	RPA3	[activation]
7779	RPTOR	IQGAP1	[activation;inhibition]
7780	PSEN2	MAST1	[activation]
7781	CDC25B	KAT2B	[activation]
7782	MAPK1	SNCA	[activation]
7783	LCP2	EGFR	[activation]
7784	FYB	BCL10	[activation]
7785	PIM2	FBXO18	[inhibition]
7786	FOS	SUMO1	[activation]
7787	RELA	TGM2	[activation]
7788	NR3C1	TXN	[activation]
7789	RND1	CREB3L2	[activation]
7790	ARAF	DNAJA1	[inhibition]
7791	ARRB2	RPL7L1	[inhibition]
7792	CDH2	CDH4	[activation]
7793	DDX39B	SNW1	[activation]
7794	MAVS	OAS3	[activation]
7795	MEP1A	CCK	[activation]
7796	APP	TNFSF14	[activation]
7797	NRIP1	ESR2	[activation]
7798	SEL1L	UNC93B1	[activation]
7799	IL10RB	UCN3	[activation]
7800	CDC5L	PAXIP1	[activation]
7801	PLN	DVL3	[inhibition]
7802	FYN	GABBR1	[activation]
7803	FGF2	GPC3	[activation]
7804	HSPA2	SIRT6	[activation]
7805	PFKFB4	CSNK2B	[activation]
7806	NMB	BIRC2	[activation;inhibition]
7807	HABP4	PRKCZ	[activation]
7808	ITGB1	FABP3	[activation]
7809	ICT1	MRPL41	[activation]
7810	CABLES2	TP53	[activation]
7811	NOS1	HSP90AA1	[activation]
7812	RAC1	PAK2	[activation]
7813	TBC1D4	YWHAB	[activation]
7814	HSPA1L	SIRT3	[activation]
7815	TGFB1	DIRAS3	[activation]
7816	TGFBR2	TRAP1	[activation]
7817	PLSCR1	CRKL	[activation]
7818	FAM21C	CCDC93	[activation]
7819	IRS1	PLCG2	[activation]
7820	EDA	MAL	[activation]
7821	LRRK2	RGS1	[activation;inhibition]
7822	HNRNPUL1	TP53	[activation]
7823	CASP1	TIRAP	[activation]
7824	ARRB1	STK38	[activation]
7825	MAST1	APOE	[activation]
7826	CEBPA	SMAD3	[inhibition]
7827	NUP62	IPO5	[activation]
7828	SRSF5	ESR1	[activation]

7829	OGT	GSK3B	[activation]
7830	KCNQ1	HSPA4	[activation]
7831	SCAMP5	CREB3	[activation]
7832	HLA-B	MARS	[activation]
7833	SMAD7	ITCH	[activation]
7834	TRAF1	DIABLO	[activation]
7835	SOX1	STAT3	[activation]
7836	CDK5R1	STXBP1	[activation]
7837	STX7	NSF	[activation]
7838	PLG	C7	[inhibition]
7839	MYL1	LRRK2	[activation]
7840	EP300	BRD7	[activation]
7841	MAP3K8	BMPR2	[activation]
7842	ARHGAP27	CDC42	[activation]
7843	TXN2	MLH1	[activation]
7844	HSD17B4	GSK3B	[activation]
7845	ILK	SCO2	[activation]
7846	NGFR	CAV1	[activation]
7847	CCDC155	GABARAPL2	[activation]
7848	EIF1B	DDT	[inhibition]
7849	SMC2	NCAPD2	[activation]
7850	SOCS3	NFKBIA	[activation;inhibition]
7851	GNB2	MAP3K3	[activation]
7852	TRADD	LRRK2	[activation]
7853	GADD45GIP1	ICT1	[activation]
7854	VCAM1	PTGES3	[activation]
7855	SMAD4	RRAS2	[activation]
7856	CTTN	ACTG1	[activation]
7857	TANC1	CAMK2A	[activation]
7858	DRD2	SSTR5	[activation]
7859	IKBKB	JUN	[activation]
7860	SRPK2	UBE2E2	[activation]
7861	RAD21	CP	[activation]
7862	CD81	RTF1	[activation]
7863	BRAF	MRAS	[activation]
7864	CSK	UBASH3B	[activation]
7865	RBPJ	SMARCA4	[activation]
7866	CBLB	KIT	[inhibition]
7867	CENPB	ETS1	[activation]
7868	ERBB4	CTGF	[activation]
7869	CIITA	XPO1	[activation]
7870	ITGAV	ITGB6	[activation]
7871	RPRD1B	RPA3	[activation]
7872	RASSF2	HRAS	[activation]
7873	PRKRA	KIAA1377	[activation]
7874	PTK2	HSP90AA1	[activation]
7875	KRTAP5-9	P2RX4	[activation]
7876	RANBP2	NUP62	[activation]
7877	APP	FOS	[activation]
7878	RAF1	ARRB2	[activation]
7879	SIRT1	KCNA5	[activation]
7880	PPP2CA	MID1	[inhibition]
7881	TP53	OLA1	[activation]
7882	GNAS	RIC8A	[activation]
7883	IRS1	SHC1	[activation]
7884	MAST4	SMAD1	[activation]
7885	PTPRF	PPFIA3	[inhibition]
7886	BMP1	YLPM1	[activation]
7887	PRKCI	GABARAPL1	[activation]
7888	CACNA1D	BRK1	[activation]
7889	PTCH1	YAP1	[activation]
7890	GNAI2	GPSM3	[activation;inhibition]
7891	RALA	REPS1	[activation]
7892	ARHGEF16	YWHAG	[activation]
7893	WNK4	CLDN3	[activation]
7894	RAD21	TFG	[activation]
7895	CREBBP	ONECUT1	[activation]
7896	CTR9	NXF1	[activation]
7897	PHGDH	STK4	[activation]
7898	SRC	FYN	[activation]
7899	STAT3	MOV10	[activation]
7900	PROC	F8	[activation]
7901	DUS1L	SRPK1	[activation]
7902	KMT2A	OXT	[activation]
7903	PIK3R1	NCK1	[activation]
7904	CD81	HIST1H2BK	[activation]

7905	COL5A1	PDGFB	[activation]
7906	TES	SOCS3	[inhibition]
7907	ARFGAP3	CD14	[activation]
7908	ITGB2	ICAM5	[activation]
7909	BAAT	SMAD4	[activation]
7910	CRK	DOK1	[activation]
7911	PGK1	RAD21	[activation]
7912	CDK5R1	CDK6	[activation]
7913	BACH2	BCL6	[activation]
7914	MORN4	NYAP2	[inhibition]
7915	SRSF5	CD101	[activation]
7916	DNM1	CAV1	[activation]
7917	ZNF622	FOS	[activation]
7918	PGAM5	YWHAG	[activation]
7919	LRRK2	TMOD3	[activation]
7920	TNK2	EP300	[activation]
7921	LATS1	TJP2	[inhibition]
7922	GABRB3	ARFGEF2	[activation]
7923	PIAS2	PGR	[activation]
7924	VAV3	PDGFRB	[activation]
7925	SMAD4	JUN	[activation]
7926	ATF2	AHSG	[activation]
7927	FCAR	PPP2CA	[activation]
7928	ELANE	NOTCH2NL	[activation]
7929	JUN	PIN1	[activation]
7930	RPA3	WDR3	[activation]
7931	IL2RB	SHB	[inhibition]
7932	HSPA4	ABL1	[activation]
7933	SETDB1	SUV39H1	[activation]
7934	EIF4EBP1	LRRK2	[activation]
7935	ABL1	LTBP2	[activation]
7936	PARD6A	PRKCH	[activation]
7937	PLCG1	ALK	[activation]
7938	CCL22	VCAM1	[activation]
7939	NOTCH2NL	GEM	[activation]
7940	VAV1	SH3BP2	[activation]
7941	YES1	CDCP1	[activation]
7942	MAPK1	TP53BP1	[activation]
7943	GMFB	GRB2	[activation]
7944	CALM1	CAMKMT	[activation]
7945	SRC	THRB	[activation]
7946	MARK3	SAAL1	[activation]
7947	LRRK2	EIF3J	[activation]
7948	UBE2Q2	MYO1C	[activation]
7949	KPNA1	YWHAZ	[activation]
7950	ACACA	NXF1	[activation;inhibition]
7951	WIPF1	LRRK2	[activation]
7952	DNM1	PIK3R1	[activation]
7953	HSPA9	NFKBIA	[activation]
7954	CDH2	GRIN2D	[activation]
7955	SETDB1	AKT1	[activation]
7956	RASA1	ERBB3	[activation]
7957	LPA	ELANE	[activation]
7958	GNGT1	GNB3	[activation]
7959	SCAND1	MAPK8	[activation]
7960	DAG1	PTK2	[activation]
7961	APP	DHRSX	[activation]
7962	HMGXB4	HIST3H3	[activation]
7963	DDX5	SGK2	[activation]
7964	EIF2AK4	SMAD4	[activation]
7965	C1orf109	STX11	[activation]
7966	FURIN	LRP1	[activation]
7967	SMAD2	CTNNB1	[activation]
7968	WNK1	FHOD1	[activation]
7969	TRAF3	BRAF	[activation]
7970	BMPR1B	SOCS6	[inhibition]
7971	PRNP	CIRH1A	[activation]
7972	YWHAB	TPD52L1	[activation]
7973	ISL1	ISL2	[activation]
7974	VIM	CASP8	[inhibition]
7975	PARD3	YWHAE	[activation]
7976	POLR2A	KMT2D	[activation]
7977	STK4	PRKRIR	[activation]
7978	GABARAPL2	HIST1H2AB	[activation]
7979	KHDRBS2	GRB2	[activation]
7980	FYN	CD48	[activation]

7981	EGFR	IQGAP3	[activation]
7982	PTMS	MYB	[activation]
7983	HSPB1	LUZP1	[inhibition]
7984	PTH1R	YWHAH	[activation]
7985	OR1E2	RAD21	[activation]
7986	CRCT1	AGTRAP	[activation]
7987	DYSF	TAF1	[activation]
7988	GRK6	CHRM3	[activation]
7989	MAPK3	PRKCZ	[activation]
7990	SP1	CASP3	[activation]
7991	CCDC67	EXOC5	[inhibition]
7992	PGR	PIAS1	[activation]
7993	MYB	PAX5	[activation]
7994	YWHAE	C11orf84	[activation]
7995	RPTOR	EIF4E	[inhibition]
7996	CIAO1	ACACA	[activation;inhibition]
7997	UBE2I	CDK1	[activation]
7998	SRPK1	GRK5	[activation]
7999	SRPK2	MMTAG2	[activation]
8000	NUTF2	NXF1	[activation]
8001	FANCA	PLCG1	[activation]
8002	PML	PLCG1	[activation]
8003	PARD6A	CDH5	[activation]
8004	PTTG1	ANAPC11	[inhibition]
8005	PARD3	LIMK2	[activation]
8006	PXN	NCK1	[activation]
8007	ESR1	TAF1B	[activation]
8008	DDX5	ARF6	[activation]
8009	SMAD2	ETV4	[activation]
8010	PRKACB	AKAP7	[activation]
8011	SERPINA5	FGG	[inhibition]
8012	TRADD	TRAF3	[activation]
8013	BAD	PRKCE	[activation;inhibition]
8014	PYHIN1	AGRN	[activation]
8015	LUC7L2	GABARAPL2	[activation]
8016	RRAS2	RIN1	[activation]
8017	GIP	ADAMTSL4	[activation]
8018	CUL1	SKP2	[inhibition]
8019	FADD	ABCA1	[activation]
8020	EPAS1	SP1	[activation]
8021	STK4	HSPA8	[activation]
8022	RAB5C	ARRB2	[activation]
8023	VCAM1	DDX5	[activation]
8024	CXCR4	VAV1	[activation]
8025	CRK	PCDHA2	[activation]
8026	LRRK2	PYCR1	[activation]
8027	MRPL51	ICT1	[activation]
8028	FCGR2B	SRC	[activation]
8029	CD79A	IGHM	[activation]
8030	ARRB2	EIF4B	[activation]
8031	IQSEC1	MCM7	[activation]
8032	GNA13	CDH2	[activation]
8033	TRAF2	RIPK1	[activation]
8034	CAMK1	ATF1	[activation]
8035	SMAD2	SLC25A6	[activation]
8036	GRB2	ACTL6A	[activation]
8037	MAP2K5	STAMBP	[activation]
8038	SORT1	LRPAP1	[activation]
8039	ITSN2	CBL	[activation]
8040	RIPK1	IKBKG	[activation]
8041	TTC17	ACVR1	[activation;inhibition]
8042	PTPRC	SLAMF1	[activation]
8043	LRRC4C	DFNB31	[activation]
8044	ACTG1	TNFRSF1B	[activation]
8045	HSPA1L	CBL	[inhibition]
8046	LDLR	FLT1	[activation]
8047	SFN	PLEKHF2	[activation]
8048	PIK3R2	STAB1	[activation]
8049	RAB7A	TRAF6	[activation]
8050	CRKL	DCBLD2	[activation]
8051	KPNA3	HSF1	[activation]
8052	MLKL	PBX2	[activation]
8053	CAMK2D	CDKN1A	[activation]
8054	GAB1	PTK6	[activation]
8055	MDM2	UBTF	[activation]
8056	LRRK1	PAK4	[activation]

8057	IPO5	NUP214	[activation]
8058	PRMT5	SMARCA4	[activation]
8059	TLR4	TOLLIP	[activation]
8060	PDCD6	DAPK1	[activation]
8061	RAN	ASAP2	[activation]
8062	LYN	CSF3R	[activation]
8063	IL37	CASP1	[activation]
8064	HSPB1	MAPKAPK2	[activation]
8065	HSPB1	LRRK2	[activation]
8066	TRAPPC8	TRAPPC2L	[activation]
8067	ANAPC16	ANAPC1	[activation]
8068	ITGA2	COL1A2	[activation]
8069	TP53	RPS15	[activation]
8070	CDK2	NPDC1	[activation]
8071	SKI	MYB	[activation]
8072	FIBP	MIF	[activation]
8073	RGS8	GOLGA2	[activation;inhibition]
8074	ATF2	DDX39B	[activation]
8075	CD3E	EPS8L3	[activation]
8076	PYHIN1	NBN	[activation]
8077	HCK	ACTB	[activation]
8078	CD4	HLA-DQA2	[activation]
8079	TNFRSF13B	CAMLG	[activation]
8080	PLAU	SERPINF2	[activation]
8081	AP5B1	CAMK2B	[inhibition]
8082	GIMAP6	GABARAPL2	[activation]
8083	EIF4G1	ANXA5	[activation]
8084	VCAM1	SRI	[activation]
8085	SGK1	CREB1	[activation]
8086	RAD51C	NEDD8	[activation]
8087	KMT2A	CTR9	[activation]
8088	EIF4A2	RPAP2	[activation]
8089	NSD1	PPARG	[activation]
8090	NR4A1	VHL	[inhibition]
8091	TWIST1	TP53	[activation]
8092	SPHK1	CTSB	[activation]
8093	SREBF2	EGR1	[activation]
8094	GCG	GCGR	[activation]
8095	EP300	N4BP2	[activation]
8096	COX17	TP53	[activation]
8097	MAVS	IRF3	[activation]
8098	LIN37	HIST1H4A	[activation;inhibition]
8099	SRC	PLSCR1	[activation]
8100	EGFR	CSNK1A1	[activation]
8101	SLC8A3	PPP3CB	[activation]
8102	MAP3K5	PRKAA2	[inhibition]
8103	PRMT2	RB1	[inhibition]
8104	ESR1	EIF4G3	[activation]
8105	GABARAPL2	HSPA4L	[activation]
8106	KLK9	TGFB1	[activation]
8107	TRAF2	XIAP	[activation]
8108	RPA2	CTTN	[activation]
8109	HSPB1	SLC7A9	[activation]
8110	ABL1	USP7	[activation]
8111	CDK4	PRMT5	[activation]
8112	SP1	CCND1	[activation]
8113	SIRT2	MYOD1	[activation]
8114	PLCD1	APP	[activation]
8115	AKT1	KAT2B	[activation]
8116	GIT1	ADRBK2	[activation]
8117	ATP1A1	ATF2	[activation]
8118	NEK6	SMC2	[activation]
8119	PPP1CA	RPAP3	[activation;inhibition]
8120	SERPINC1	KLK6	[inhibition]
8121	ESR1	MED1	[activation]
8122	TRAF3	SNAP29	[activation]
8123	MYO1B	CIAO1	[activation]
8124	ICT1	SLX4	[activation]
8125	FBLIM1	HIST1H3A	[activation]
8126	SH2D1B	TRIM54	[activation]
8127	HSPA2	TRIM38	[activation]
8128	PML	NFKB1	[activation]
8129	SRPK2	VRK1	[activation]
8130	SAT1	EPHB6	[activation]
8131	HCK	ERBB3	[activation]
8132	BCL2L12	HSPA4	[inhibition]

8133	ACTR3B	RBL1	[activation]
8134	FGFR1	NCAM1	[activation]
8135	NCOA2	PPARG	[activation]
8136	ZFYVE20	MAPK14	[activation]
8137	GCN1L1	CDC5L	[activation]
8138	HDAC1	CIITA	[activation]
8139	YPEL3	SRPK2	[activation]
8140	GLRA2	DDX24	[activation]
8141	TP53	PAXIP1	[activation]
8142	SMARCB1	CCNE1	[activation]
8143	FARS2	G3BP2	[activation]
8144	ACP5	RPA2	[activation]
8145	PTPRO	WAS	[activation]
8146	CSNK1A1	PPP2R5A	[activation]
8147	GRIA4	GRIA1	[activation]
8148	HSPB1	MAP1LC3B	[activation]
8149	RAB2A	ATF2	[activation]
8150	LILRB1	HLA-B	[activation]
8151	SAV1	CSNK1E	[activation]
8152	ZAP70	CARD11	[activation]
8153	HSP90AB1	KCTD8	[activation]
8154	PPARG	STMN1	[activation]
8155	DNM1L	ESR1	[activation]
8156	PCNA	ATF2	[activation]
8157	MAP3K4	AXIN1	[activation]
8158	PIK3R1	GRB10	[activation]
8159	HSF1	MAPK3	[activation]
8160	FOS	RPS6KA2	[activation]
8161	SFN	CBL	[activation]
8162	NOTCH2	ST14	[activation]
8163	GLIS2	CTNNB1	[activation]
8164	FANCG	FANCE	[activation]
8165	EIF3CL	STK4	[activation]
8166	RHOQ	CDC42EP1	[activation]
8167	CASP3	MDM2	[activation]
8168	ELAVL1	MICB	[activation]
8169	PHLPP1	BTRC	[inhibition]
8170	ZBTB8OS	PRKCH	[activation]
8171	HDAC1	E2F4	[inhibition]
8172	PDX1	SPOP	[activation]
8173	MAP3K1	BRAF	[activation]
8174	ARRB2	CTNND1	[activation]
8175	NPW	NPBWR2	[activation]
8176	HSPB1	AATK	[activation]
8177	SMC4	RPA3	[activation]
8178	VANGL2	DVL1	[activation]
8179	ARHGEF4	APC	[activation]
8180	SMARCA5	CEBPB	[activation]
8181	TP53	TUBB	[activation]
8182	FOS	TSPAN2	[activation]
8183	YWHAZ	APC	[activation]
8184	ARHGDIA	VAV1	[activation]
8185	FOS	ATRNL1	[activation]
8186	SGK1	IKBKG	[activation]
8187	TP73	MAPK8	[activation]
8188	BAD	S100A10	[activation;inhibition]
8189	ADSS	HLA-B	[activation]
8190	CAMK2B	ETS1	[activation]
8191	CD48	LCK	[activation]
8192	ARRB1	TRAF6	[activation]
8193	LIN52	LIN54	[inhibition]
8194	MDH1	HSP90AB1	[activation]
8195	FOXC1	PITX2	[activation]
8196	EZH2	MAP3K7	[activation]
8197	EP300	GPBP1	[activation]
8198	SLFN11	RPA2	[activation]
8199	NFKBIA	UBE2I	[activation]
8200	YWHAG	LUC7L2	[activation]
8201	GHR	DUSP7	[inhibition]
8202	EGFR	NCL	[activation]
8203	NARF	SFRP4	[activation]
8204	ATP1B1	NR3C1	[activation]
8205	NLK	BACH1	[inhibition]
8206	CSNK1G3	DBNDD2	[activation]
8207	YY1	ACTB	[activation]
8208	CAPRIN1	MDM2	[activation]

8209	TNFAIP3	CHUK	[activation]
8210	ACTG1	DNASE1	[activation]
8211	GNAI2	IGF1R	[activation]
8212	SMAD2	DLX4	[activation]
8213	IL2	IL2RG	[activation]
8214	WAS	SRGAP2	[activation]
8215	ESR1	GNAI2	[activation]
8216	TGFBR1	DAPK2	[activation]
8217	TNFRSF11A	TRAF5	[activation]
8218	FZR1	GRIA1	[inhibition]
8219	ASS1	LATS1	[inhibition]
8220	CDH1	BLID	[activation]
8221	LNX2	MAPK9	[activation]
8222	ATG5	RBBP4	[activation]
8223	ABI2	VARS2	[activation]
8224	CAMK2D	BANP	[activation]
8225	PDE3B	PIK3CG	[activation]
8226	HDAC1	PPP1CC	[inhibition]
8227	RPTOR	FKBP1A	[inhibition]
8228	AGPAT1	MYC	[activation]
8229	CASP3	NMT2	[activation]
8230	YWHAH	VAMP8	[activation]
8231	MAPK14	NFATC4	[inhibition]
8232	UBAC2	GCN1L1	[activation]
8233	SNAI1	TTC25	[activation;inhibition]
8234	JAK2	G3BP1	[activation]
8235	PNO1	TRAF6	[activation]
8236	GAPVD1	YWHAB	[activation]
8237	CUL1	BCL6	[activation]
8238	DNM1	PRKCA	[activation]
8239	CAPNS1	AGTRAP	[activation]
8240	KCTD17	RWDD4	[activation]
8241	OSGEP	SHC1	[activation]
8242	KISS1	MMP2	[activation]
8243	BCL2L1	HRK	[activation]
8244	SPHK2	FHL2	[activation]
8245	YWHAQ	NFATC1	[activation]
8246	DLG3	CAMK2A	[activation]
8247	GABARAPL1	ANXA1	[activation]
8248	GAB1	SUPT6H	[activation]
8249	AKAP8	LRRK2	[activation]
8250	MTFR1	DUSP23	[activation]
8251	ACP6	RSU1	[activation]
8252	CTNNA1	FBP2	[activation]
8253	L1CAM	NUMB	[activation]
8254	ETS1	SP100	[activation]
8255	RALBP1	CCNB1	[activation]
8256	CALM1	MBP	[activation]
8257	TRIM28	ARF6	[activation]
8258	CDC23	ANAPC1	[activation]
8259	PIK3R2	TGFBR1	[activation]
8260	IARS	SGK1	[activation]
8261	CDC42	RIF1	[activation]
8262	LZTR1	HDAC4	[activation]
8263	PKIB	PRKACB	[activation]
8264	SMAD4	FOXO4	[activation]
8265	HIST1H2AG	YWHAZ	[activation]
8266	MTOR	SIRT1	[activation]
8267	DDX39B	ZHX1	[activation]
8268	SMAD7	PARD3	[activation]
8269	TP53	CDK1	[activation]
8270	SPP1	KCNIP1	[activation]
8271	CDK5RAP2	TUBGCP6	[activation]
8272	OLFM4	NOD1	[activation]
8273	TRAF6	PPP2CB	[activation]
8274	RASA1	DCC	[activation]
8275	CDCA8	XPO1	[activation]
8276	RASGRF1	SNRNP70	[activation]
8277	POFUT1	JAG1	[activation]
8278	PRSS50	STK11	[activation]
8279	H2AFX	ANXA2P2	[activation]
8280	FGFR1	GRB14	[activation]
8281	RNF34	TP53	[activation]
8282	SP1	FYN	[activation]
8283	PCYT1B	ACTB	[activation]
8284	TNFRSF10B	EGFR	[activation]

8285	RPL7	TP53	[activation]
8286	SRPK1	CDK7	[activation]
8287	AXIN1	SMURF1	[inhibition]
8288	IRF4	NFATC2	[activation]
8289	HDAC6	MYD88	[activation]
8290	NUDCD3	EIF2AK4	[activation]
8291	LAT	GRB2	[activation]
8292	TSC2	MAPKAPK2	[activation]
8293	N4BP2	BCL3	[activation]
8294	TWF2	KRT31	[activation]
8295	LATS1	MAST2	[inhibition]
8296	GRAP	BCR	[activation]
8297	PKM	CD81	[activation]
8298	PIP4K2B	EPB41L3	[activation]
8299	APP	RABL3	[activation]
8300	SRC	ERBB3	[activation]
8301	EGFR	RET	[activation]
8302	VDR	NCOA3	[activation]
8303	BARD1	CDK1	[activation]
8304	EXOC5	PYCARD	[inhibition]
8305	LRRK2	MDN1	[activation]
8306	CREBBP	SUV39H1	[activation]
8307	PKM	RELA	[activation]
8308	KLK7	PTPN1	[activation]
8309	TFPI	MMP9	[inhibition]
8310	CEBPB	DDIT3	[activation]
8311	STK4	ARF1	[activation]
8312	CEP55	CDK1	[activation]
8313	RAC3	RWDD1	[activation]
8314	POLA1	PARP1	[activation]
8315	KPNA1	FOSL1	[activation]
8316	RET	NRTN	[activation]
8317	A2M	CDC42	[inhibition]
8318	TPD52L1	HDAC1	[activation]
8319	TAGLN2	HLA-B	[activation]
8320	PML	SP1	[activation]
8321	ICAM1	FAU	[activation]
8322	IL9	SNTA1	[activation]
8323	CXCR4	PECAM1	[activation]
8324	MCM6	CCND1	[activation]
8325	PRIM1	MRPL38	[activation]
8326	GRM3	GRASP	[activation]
8327	TNFRSF21	WASH2P	[activation]
8328	ARRDC3	UBE2E2	[activation]
8329	RFX6	DGCR6	[activation]
8330	XPOT	MYC	[activation]
8331	CACNA1A	C1QTNF1	[inhibition]
8332	GABARAPL2	PFN1	[activation]
8333	TRAF5	TRAF3	[activation]
8334	RFXAP	SMARCA4	[activation]
8335	ANXA7	ANGPT2	[activation]
8336	TRIM28	NR3C1	[activation]
8337	DUSP1	SUMO3	[inhibition]
8338	PSEN2	CAPN1	[activation]
8339	PPIF	ABI2	[activation]
8340	ATF3	HDAC1	[activation]
8341	ICT1	MRRF	[inhibition]
8342	BCL2L1	TPT1	[activation]
8343	GRB2	HIST1H4A	[activation]
8344	APC	CASC3	[activation]
8345	CCL7	MMP3	[activation]
8346	GATA2	ZFPM1	[activation]
8347	EXOC4	WASH1	[activation]
8348	NFKBIA	PTPN13	[activation]
8349	DVL1	TRIM69	[activation]
8350	DVL3	WT1	[activation]
8351	BMP2	ASCL1	[activation]
8352	KANSL2	APP	[activation]
8353	NANS	PDGFRL	[activation;inhibition]
8354	FAM178A	CALM1	[activation]
8355	PTEN	SMTN	[activation;inhibition]
8356	PLK1	SNW1	[activation]
8357	APBB3	EGFR	[activation]
8358	AHNAK	ATF2	[activation]
8359	NPHP1	PTK2B	[activation]
8360	AUP1	OPRM1	[activation]

8361	HES1	FANCL	[activation]
8362	TTN	OBSCN	[activation]
8363	PDGFB	THBS1	[activation]
8364	PTBP3	RAB23	[activation]
8365	TAS1R1	TAS1R3	[activation]
8366	FOS	LMO3	[activation]
8367	FOS	GNL2	[activation]
8368	RPA2	C7orf50	[activation]
8369	CREBBP	CSNK2A1	[activation]
8370	USH1C	RAC1	[activation]
8371	RAC1	CNTNAP1	[activation]
8372	IGF1R	NAT2	[activation]
8373	MYC	NMI	[activation]
8374	ACACA	SHC1	[activation;inhibition]
8375	NDRG1	MLH1	[activation]
8376	NFKB2	SMARCA4	[activation]
8377	PIGR	CD79A	[activation]
8378	BCL2	RRAS	[activation]
8379	COL4A5	APP	[activation]
8380	ENO2	GABARAP	[activation]
8381	HLA-B	CDKN2A	[activation]
8382	AR	SPDEF	[activation]
8383	VAMP8	VAMP3	[activation]
8384	LRRK2	SPIN1	[activation]
8385	PLXNB1	RAC1	[activation]
8386	HSPG2	PDGFB	[activation]
8387	HNF1A	CTNNB1	[activation]
8388	BIRC3	IKBKB	[activation]
8389	IRS4	HSPA4	[activation]
8390	HIST1H2BL	ICAM1	[activation]
8391	RANBP10	MET	[activation]
8392	HIPK2	PTCH1	[activation]
8393	KAT2B	JUN	[activation]
8394	INADL	KCNJ2	[activation]
8395	HIST1H2BJ	ICAM1	[activation]
8396	YTHDC2	AXIN1	[activation]
8397	NOS1AP	FAM133A	[inhibition]
8398	AKAP9	CDK5RAP2	[activation]
8399	TULP3	CCNB1	[activation]
8400	FLNA	GRB2	[activation]
8401	SLC23A3	IGF1R	[activation]
8402	ADORA2A	CNR1	[activation]
8403	PRKCQ	HSP90AB1	[activation]
8404	CDC25B	SFN	[activation]
8405	GRB2	MYL12A	[activation]
8406	LAMA4	PTN	[activation]
8407	ARHGEF6	SMAD2	[activation]
8408	NCF4	PRKDC	[activation]
8409	DYRK1B	MAP2K3	[activation]
8410	MAP1LC3B	DDX24	[activation]
8411	TRIM16	NXF1	[activation]
8412	HIST3H3	JADE3	[activation]
8413	MEP1B	PRKACA	[activation]
8414	CPE	CCK	[activation]
8415	CXCR4	JAK1	[activation]
8416	GNB2	GNAI3	[activation]
8417	PRKCI	FRS2	[activation]
8418	KIAA0930	YWHAE	[activation]
8419	CAPN10	FANCC	[activation]
8420	PGK1	HSPH1	[activation]
8421	CCL5	DPP4	[activation]
8422	USF1	ESR1	[activation]
8423	LRRK2	PTPN23	[activation]
8424	PRKCD	PPM1A	[activation]
8425	NOTCH2NL	HSPA12B	[activation]
8426	ACACA	STK3	[activation;inhibition]
8427	ERBB4	ITCH	[activation]
8428	HSP90AB1	SGK1	[activation]
8429	FGF2	PF4	[activation]
8430	STIP1	ATF2	[activation]
8431	JUN	UBE2I	[activation]
8432	CASP10	RIOK3	[activation]
8433	NCKAP5	APC	[inhibition]
8434	HES1	HMGB1	[activation]
8435	EPOR	PTPRC	[activation]
8436	ILK	CDIPT	[activation]

8437	EDN3	CMA1	[activation]
8438	PRKDC	PPP6C	[activation]
8439	ZNRF1	UBE2E3	[activation]
8440	GSK3B	STYK1	[activation]
8441	STK3	MPP1	[activation]
8442	PAXIP1	SRSF5	[activation]
8443	MCM3	HLA-B	[activation]
8444	GAS7	DIAPH1	[activation]
8445	DDX21	GH1	[activation]
8446	ATF2	APP	[activation]
8447	BRAF	AKT1	[activation]
8448	SIRT1	EPAS1	[activation]
8449	WNT2	HCK	[activation]
8450	LGALS9	CTNNB1	[activation]
8451	FAM154A	STK16	[activation]
8452	RAB11A	RAB11FIP5	[activation]
8453	BMPR1A	MAP2K1	[activation]
8454	YWHAE	SRSF6	[activation]
8455	ESR1	GSN	[activation]
8456	ANAPC10	ANAPC11	[activation]
8457	POU1F1	NR1I2	[activation]
8458	C5	C7	[activation]
8459	NFKB1	MTPN	[activation]
8460	AURKB	HSPA5	[activation]
8461	EGFR	SNX9	[activation]
8462	GNAQ	GALR2	[activation]
8463	SIRT1	HSF1	[activation]
8464	KDM4D	TP53	[activation]
8465	LHX1	ISL1	[activation]
8466	ROBO4	SLIT2	[activation]
8467	ASB18	ASS1	[inhibition]
8468	AR	VAV3	[activation]
8469	PELI2	MYD88	[activation;inhibition]
8470	TTC17	SMURF1	[inhibition]
8471	CTBP1	CDC23	[activation]
8472	RGS12	GRM5	[activation;inhibition]
8473	DYRK1A	HIST1H4A	[activation;inhibition]
8474	AUTS2	EP300	[activation]
8475	HP1BP3	PAXIP1	[activation]
8476	ATF4	ATF2	[activation]
8477	A2M	ADAMTS7	[inhibition]
8478	TNFRSF14	DCD	[activation]
8479	ADRB2	MAGI3	[activation]
8480	NUMA1	WHSC1	[activation]
8481	ATF4	NXF1	[activation]
8482	GNB2	EGFR	[activation]
8483	GRB2	CD22	[activation]
8484	CCDC8	MARS	[activation]
8485	CFL1	TAB1	[activation;inhibition]
8486	FBXO6	ASS1	[inhibition]
8487	PTGFR	PTGER2	[activation]
8488	SIRT1	STAT3	[activation]
8489	EP300	MAP2K1	[activation]
8490	H2AFX	POFUT1	[activation]
8491	APC	BUB1	[activation]
8492	CDK11B	CASP1	[activation]
8493	ULK1	MAP1LC3A	[inhibition]
8494	CCDC114	CCDC151	[activation]
8495	GATA1	RADIL	[activation]
8496	WIPI1	AR	[activation]
8497	VCAM1	XPO1	[activation]
8498	ITGB1	LAMTOR5	[activation]
8499	CDC7	CLSPN	[activation]
8500	CDK4	HIST1H1D	[inhibition]
8501	BLK	ATP5O	[activation]
8502	APP	FMNL1	[activation]
8503	SGTA	MOS	[activation]
8504	CEBPA	CDKN3	[inhibition]
8505	PRKAA1	ABI2	[inhibition]
8506	EIF2B5	GSK3B	[activation]
8507	ETS1	CAMK2A	[activation]
8508	EIF2B1	WDYHV1	[activation]
8509	PHGDH	MAPK13	[activation]
8510	PIN1	TAB3	[activation]
8511	INSIG1	PGRMC1	[activation]
8512	DDX5	SMAD1	[activation]

8513	ATAD3C	ESR1	[activation]
8514	DDX19B	ARF6	[activation]
8515	PRMT1	TRAF6	[activation]
8516	MAFK	NFE2	[activation]
8517	MAGEB4	USHBP1	[activation]
8518	CDC5L	BRD4	[activation]
8519	CASP2	RXRA	[inhibition]
8520	ANXA6	GRB2	[activation]
8521	MYBPC2	CMYA5	[activation]
8522	GRAP	SOS1	[activation]
8523	NXF1	LAMTOR5	[activation]
8524	RICTOR	IKBKB	[activation]
8525	SMURF1	GRIPAP1	[inhibition]
8526	BAZ1B	PHLDA3	[activation]
8527	FLT1	PTK2B	[activation]
8528	PROS1	F8	[inhibition]
8529	NOS3	PSEN1	[activation]
8530	GNAI2	LTB4R	[activation]
8531	PARD6A	LLGL2	[activation]
8532	PRKAR1A	CDK4	[inhibition]
8533	APP	MKNK1	[activation]
8534	CREBBP	MAFK	[activation]
8535	APP	PPAP2B	[activation]
8536	ITGB4	SEL1L	[activation]
8537	KLHL29	NUDCD3	[activation]
8538	KIT	CBL	[activation]
8539	FASLG	PSTPIP1	[inhibition]
8540	GLTSCR1	GRB2	[activation]
8541	HIF1A	HSP90AB1	[activation]
8542	FANCF	OLFM2	[activation]
8543	SRC	FBXO18	[activation]
8544	PIK3CA	APLP2	[activation]
8545	PRKCA	KCNQ2	[activation]
8546	RAX	PAX6	[activation]
8547	CPB2	C5	[activation]
8548	FAAP24	APP	[activation]
8549	PPARG	APP	[activation]
8550	DRD2	GIPC1	[activation]
8551	SUMO1	DHTKD1	[activation]
8552	CYLD	PMS1	[inhibition]
8553	PPP3CB	PPP1R13B	[activation]
8554	PIAS3	HMGA2	[activation]
8555	LRRK2	DBF4B	[activation]
8556	PIAS2	MAPKAPK2	[activation]
8557	BCL2L1	PDIA4	[activation]
8558	VCAM1	HIST1H2BL	[activation]
8559	SYT1	SNAP25	[activation]
8560	SP1	POU2F1	[activation]
8561	BCL2L2	BCL2L11	[activation]
8562	NCK2	CHN1	[activation]
8563	RNF114	UBE2H	[activation]
8564	STX4	SCRIB	[activation]
8565	CSF3R	STAT3	[activation]
8566	C2	C5	[activation]
8567	IGSF21	HSPB1	[activation]
8568	GADD45G	GDF9	[activation]
8569	ITGA4	HNRNPD	[activation]
8570	CRKL	ARHGEF5	[activation]
8571	MAPK12	SRPK1	[activation]
8572	BLNK	BTK	[activation]
8573	MSTN	WFIKKN2	[activation]
8574	SIRPB1	SYK	[activation]
8575	EXOC6	PSEN2	[activation]
8576	TSC2	ESR1	[activation]
8577	MAPK8	TNFRSF1A	[activation]
8578	CTNNB1	ERBB2IP	[inhibition]
8579	NR2E3	RXRB	[inhibition]
8580	DNAJB11	DNAJC13	[inhibition]
8581	ATM	CDC6	[activation]
8582	SMAD3	DDX5	[activation]
8583	RAC1	PLCG1	[activation]
8584	NREP	ATM	[activation]
8585	SYK	ITGB2	[activation]
8586	MDM2	LATS1	[inhibition]
8587	PIK3CB	ERBB3	[activation]
8588	HBQ1	HBD	[inhibition]

8589	APITD1	HOMEZ	[activation]
8590	CNR2	GNA15	[activation]
8591	EGFR	BMX	[activation]
8592	DOK3	YWHAE	[activation]
8593	AURKB	CALM1	[activation]
8594	MDFI	GNAI2	[activation]
8595	CDK9	CDK5R1	[activation]
8596	RARA	CCND3	[inhibition]
8597	SIRT5	RELA	[activation]
8598	RASA1	BIRC5	[activation]
8599	NDUFS7	ENO2	[activation]
8600	PARD6G	PRKCI	[activation]
8601	ADCYAP1	CASP2	[activation]
8602	CREBBP	CUX1	[activation]
8603	ILK	FASN	[activation]
8604	MCF2L2	MEGF10	[activation]
8605	CEBPB	NR5A1	[activation]
8606	CTNNB1	FSCN1	[activation]
8607	CISH	PRKCB	[inhibition]
8608	IRF4	BCL6	[activation]
8609	SMAD1	HIPK2	[activation]
8610	PTK2	CD79B	[activation]
8611	CKS2	CCNB1	[inhibition]
8612	CHERP	SRPK2	[activation]
8613	EIF1B	RHEB	[activation]
8614	ICAM1	EIF3CL	[activation]
8615	DDX51	MYC	[activation]
8616	TRIM24	NCOA1	[activation]
8617	FANCD2	HELQ	[activation]
8618	DIABLO	BIRC6	[activation]
8619	ATG5	NUDC	[activation]
8620	HLA-B	CYB5B	[activation]
8621	EHMT2	XAGE2	[activation]
8622	CTR9	BLK	[activation]
8623	MAP2K4	CDC5L	[activation]
8624	TBK1	PPP6C	[inhibition]
8625	FGFR1	PPP2R1B	[activation]
8626	ATR	POLD1	[activation]
8627	DRD2	CALM1	[activation]
8628	APPL1	EGFR	[activation]
8629	EGFR	ATP2B4	[activation]
8630	HSP90AA1	ICK	[activation]
8631	DAB1	PTPN11	[activation]
8632	NOTCH1	SLFN11	[activation]
8633	EPHB2	MLLT4	[activation]
8634	NOTCH1	RELA	[activation]
8635	GRB2	AGER	[activation]
8636	ITK	EGFR	[activation]
8637	AKT1	SOAT2	[activation]
8638	SRRT	STAT3	[activation]
8639	SHC1	CALD1	[activation]
8640	AGTPBP1	CYCS	[activation]
8641	APP	ABR	[activation]
8642	SDC2	TIAM1	[activation]
8643	FBXO6	HLA-C	[activation]
8644	RET	DOK2	[activation]
8645	NUDC	MLST8	[activation]
8646	PGAM5	XIAP	[inhibition]
8647	CTSB	MLH1	[activation]
8648	SFN	FAM9B	[activation]
8649	F7	DMWD	[inhibition]
8650	RNF32	MGEA5	[activation]
8651	SAA1	FPR1	[activation]
8652	TRPT1	TRAF2	[activation]
8653	KLK6	COL4A1	[activation]
8654	A2M	TGIF1	[inhibition]
8655	SCARB2	CSK	[activation]
8656	NOTCH1	WDR6	[activation]
8657	TIPARP	ATM	[activation]
8658	SMURF1	HSPA8	[inhibition]
8659	RNF216	RIPK1	[activation]
8660	TFRC	B4GALT1	[activation]
8661	PPP1CC	TPRN	[inhibition]
8662	SPG20	SMAD2	[inhibition]
8663	C4BPA	GIT2	[inhibition]
8664	CRKL	WAC	[activation]

8665	CASP8	BCAP31	[activation]
8666	CCDC53	SNAP25	[activation]
8667	WWTR1	CTNNB1	[activation]
8668	ACACA	CHGB	[activation;inhibition]
8669	FGFR1	SH3BP2	[activation]
8670	CBLB	CBL	[inhibition]
8671	GEM	LZTS2	[activation]
8672	CCNG2	PPP2R5B	[activation]
8673	HIST2H3A	PCNA	[activation]
8674	STC2	PEBP1	[inhibition]
8675	HNRNPD	ICAM1	[activation]
8676	PTPN1	IL1B	[activation]
8677	KAT2B	NCOA4	[activation]
8678	AGTR1	RAB5A	[activation]
8679	PAK2	BTF3	[activation]
8680	WDR17	ETS1	[activation]
8681	FLT1	ATR	[activation]
8682	CCNB1	RELA	[activation]
8683	HNF1A	RAC3	[activation]
8684	NSD1	HIST1H1D	[activation]
8685	AMBRA1	MTOR	[activation]
8686	UBC	CSF1	[activation]
8687	EGFR	PAK1	[activation]
8688	HSPA8	GAK	[inhibition]
8689	PPARG	RANBP9	[activation]
8690	IFITM3	GLP1R	[activation]
8691	YWHAE	SIK3	[activation]
8692	STAT5B	TFG	[activation]
8693	PLXNB1	RHOA	[activation]
8694	RAF1	LRPAP1	[inhibition]
8695	SRC	CDC25C	[activation]
8696	GSTP1	MAPK1	[inhibition]
8697	GIT2	HGD	[activation]
8698	JUP	CDK15	[activation]
8699	CHRM2	ELAVL1	[activation]
8700	RPA1	MCM7	[activation]
8701	STMN3	GLP1R	[activation]
8702	CBLB	UBASH3B	[inhibition]
8703	VAMP2	PRKD3	[activation]
8704	HDAC1	ATR	[activation]
8705	STK11	HSP90AB1	[activation]
8706	SRPK1	PPIL1	[activation]
8707	HPS6	PIK3CB	[activation]
8708	PMAIP1	BCL2L1	[activation]
8709	DDX17	CTCF	[activation]
8710	ATG12	DDX58	[inhibition]
8711	TH	MAPK3	[activation]
8712	PPP2CA	STK24	[activation]
8713	LHB	CGA	[activation]
8714	MSL2	YWHAZ	[activation]
8715	CRK	ASAP2	[activation]
8716	TYK2	PDGFRB	[activation]
8717	MLH1	TMSB4X	[activation]
8718	SCAMP5	VAMP4	[activation]
8719	CSN2	FHL2	[activation]
8720	SMAD7	KAT2B	[activation]
8721	SRPK2	DDX50	[activation]
8722	TP53	CTBP1	[activation]
8723	GNAI2	P2RY12	[activation]
8724	SIX3	TLE3	[activation]
8725	SLC9A3R2	PDPK1	[activation]
8726	TENM1	SORBS1	[activation]
8727	SRPK2	HNRNPC	[activation]
8728	COL1A1	COL1A2	[activation]
8729	MAPT	HSP90AB1	[activation]
8730	HMGB1	RBPJ	[inhibition]
8731	ATF6	CREB1	[activation]
8732	IRS1	IL4R	[activation]
8733	SNCG	FAM90A16P	[inhibition]
8734	NCDN	GSTK1	[activation]
8735	METTL3	METTL14	[activation]
8736	PAK2	TNK2	[activation]
8737	GYS1	APP	[inhibition]
8738	PDGFRB	ITGB3	[activation]
8739	MLH1	AMOT	[activation]
8740	CALM1	ESRRG	[activation]

HIST1H4A	SUV420H1	[activation]
STAT5B	INSR	[activation]
ICAM4	ITGA4	[activation]
DIAPH1	WBP4	[activation]
PAXIP1	SKA3	[activation]
GSK3B	GNB2	[inhibition]
BAK1	GIMAP5	[activation]
SLC25A4	E2F3	[inhibition]
NUMB	PRKCG	[activation]
FANCG	FSCN1	[activation]
PRKCB	AKT1	[activation]
LATS1	MOB3C	[inhibition]
RELA	DNAJA3	[inhibition]
FTSJ1	CDKN2A	[activation;inhibition]
FN3KRP	APP	[activation]
FAAH2	APP	[activation]
TP53	CCL18	[activation]
PPP3CB	SLC8A2	[activation]
IKBKG	TRAF6	[activation]
MAGOH	RAN	[activation]
PRKCA	GABRB3	[activation]
CIT	RND3	[activation]
WDFY2	NUDC	[activation]
VEGFA	EPHB2	[activation]
EIF4A2	ECT2	[activation]
ZNF32	GRB2	[activation]
PRKD2	IFNAR1	[activation]
TBK1	RARA	[activation]
POU5F1	ETS2	[activation]
RBL1	TFDP1	[inhibition]
EIF2S1	MAST3	[activation]
NR3C1	NRIP1	[activation]
ADNP	HIST3H3	[activation]
NXF1	DDX5	[activation]
CEBPE	E2F1	[activation]
MAP2	MDM2	[activation]
SLX1A	ANXA1	[activation]
ATM	SMAD7	[activation]
LZTS2	CCNG1	[activation]
MAPK8	RAF1	[activation]
PPP1CA	H2AFX	[activation]
PAK2	SH3RF1	[activation]
LRRK2	DHX16	[activation]
YWHAG	CASP3	[activation]
AKT1	MTCP1	[activation]
BAK1	BCL2A1	[activation;inhibition]
DUSP13	ACVR1	[activation;inhibition]
H3F3A	CCDC71	[activation]
BIRC3	BIRC2	[activation;inhibition]
MCM3	NFKBIA	[activation]
CREBBP	STAT5A	[activation]
ATF2	COLGALT1	[activation]
EGLN1	KRAS	[activation]
PRKAB1	EPB41L5	[inhibition]
GMEB1	CASP8	[inhibition]
ABL1	PAK2	[activation]
EEF2K	RPS6KA1	[activation]
RPA1	CCNA2	[activation]
CSNK1A1	ACACA	[activation;inhibition]
CCND1	FBXO4	[inhibition]
DDX5	STK24	[activation]
CDH1	SKP2	[activation]
EGFR	GPM6B	[activation]
CRY1	LTBP4	[inhibition]
BCL2L11	AURKB	[activation]
GREM2	BMP4	[activation]
PPP2R4	CCNG1	[activation]
SFN	MARK3	[activation]
RHOA	ARHGEF19	[activation]
FBXO6	COL6A1	[activation]
NDEL1	YWHAE	[activation]
MYOG	PRMT5	[activation]
RAC1	RTKN	[activation]
PDCD6IP	GRB2	[activation]
JAG1	FBXW7	[activation]
DUSP15	PLCG1	[activation]

8817	PRKCZ	HIST1H1B	[activation]
8818	MAP1LC3B	RANBP2	[activation]
8819	TRIP4	RXRA	[inhibition]
8820	PPP3CB	IRF2	[activation]
8821	CMSS1	S1PR1	[activation]
8822	TRIM39	UBE2E2	[activation]
8823	AK8	CCDC102B	[inhibition]
8824	ATM	RHEB	[activation]
8825	AR	ETV5	[activation]
8826	RIMS4	SRPK1	[activation]
8827	SLFN11	EGFR	[activation]
8828	RASA1	STAU1	[activation]
8829	WNT4	PORCN	[activation]
8830	XRCC2	BLM	[activation]
8831	NOS1AP	SYN2	[inhibition]
8832	STAU1	MAPT	[activation]
8833	CRK	GRB2	[activation]
8834	APP	ADAM17	[activation]
8835	UBE2I	SOX9	[activation]
8836	NFKB1	RPS6KA5	[activation]
8837	STRAP	BARD1	[activation]
8838	CXCL12	CTSG	[activation]
8839	FOXO4	ESR1	[activation]
8840	HIST1H1A	GZMA	[activation]
8841	EP300	ASCL1	[activation]
8842	PRNP	DPP6	[activation]
8843	ENKD1	SPERT	[inhibition]
8844	ARHGEF7	WAS	[activation]
8845	VAMP8	VAMP2	[activation]
8846	FZR1	ANAPC10	[activation]
8847	PRKAA2	AMOT	[activation]
8848	YWHAH	SLC25A6	[activation]
8849	JAK1	FER	[activation;inhibition]
8850	KIT	GRB7	[activation]
8851	SGK1	CAMKK1	[activation]
8852	HCLS1	CASP3	[activation]
8853	TIE1	PTPRC	[activation]
8854	BANF1	HLA-B	[activation]
8855	BUD13	MCM7	[activation]
8856	SMAD3	CBL	[inhibition]
8857	CDC37	AURKB	[activation]
8858	PLK1	MCM7	[activation]
8859	YES1	ITGB4	[activation]
8860	PARP1	CD86	[activation]
8861	TP53	NFYA	[activation]
8862	SIRT1	WDR70	[activation;inhibition]
8863	CDKN1A	TRRAP	[activation]
8864	CSK	PTPRC	[activation]
8865	DLX1	SMAD4	[inhibition]
8866	SMAD2	SENP2	[activation]
8867	APP	APOE	[activation]
8868	ARFGEF2	FKBP2	[activation]
8869	EP300	LEF1	[activation]
8870	MAL	SRC	[activation]
8871	TP53	YWHAE	[activation]
8872	PRKCD	EGFR	[activation]
8873	PRPF6	MYC	[activation]
8874	GOLGA2	TSSK3	[activation]
8875	APEX1	HIF1A	[activation]
8876	KMT2A	MAP3K5	[activation]
8877	ACOX1	SCP2	[activation]
8878	CDCP1	PRKCD	[activation]
8879	RPS6KA5	DNAJC11	[activation]
8880	NDRG1	CDH1	[activation]
8881	ARRDC1	ADRB2	[activation]
8882	ILK	HSPE1	[activation]
8883	CARD11	MAP3K7	[activation]
8884	P2RY2	KDR	[activation]
8885	RPA1	BCAP31	[activation]
8886	GRB2	HELZ	[activation]
8887	TRAF1	CASP6	[activation]
8888	RAN	SMARCB1	[activation]
8889	NFE2L2	CREBBP	[activation]
8890	FOS	CREBBP	[activation]
8891	IRF7	SMAD4	[activation]
8892	GNAI2	TTC1	[activation]

8893	STAT6	NFKB1	[activation]
8894	TP53	TRIM24	[activation]
8895	MLXIPL	NIF3L1	[activation]
8896	RARB	RXRA	[inhibition]
8897	ATG4D	CASP3	[activation]
8898	TSR1	RPA3	[activation]
8899	RAB1A	RPA3	[activation]
8900	HSP90AB1	PIM3	[activation]
8901	GATA4	SRF	[activation]
8902	NR5A1	MAPK1	[activation]
8903	NRXN1	ATP13A2	[activation]
8904	PPARA	GADD45A	[activation]
8905	EIF4A1	GABARAP	[activation]
8906	PTEN	STK11	[activation]
8907	STK11	PTGES3	[activation]
8908	STRAP	HLA-B	[activation]
8909	GATA3	FOXA1	[activation]
8910	ITGA4	RCC1	[activation]
8911	IL33	HIST2H2AA3	[activation]
8912	TP53	CDKN2C	[activation]
8913	GAB2	GAB1	[activation]
8914	PDGFA	SPARC	[activation]
8915	POLA2	POLA1	[activation]
8916	TP73	CDK2	[activation;inhibition]
8917	TRAF3	ARRB2	[activation]
8918	NR4A1	YWHAZ	[inhibition]
8919	MAD2L1	PPP2R5D	[activation]
8920	APP	TRIB3	[inhibition]
8921	TIAM2	TNIK	[activation]
8922	RAB2A	MAPK6	[activation]
8923	WFIKKN1	BMP8B	[activation]
8924	HIST3H2A	ITGA4	[activation]
8925	PRKCD	PPARA	[activation]
8926	MDM2	FAM171A1	[activation]
8927	KLHL12	PDGFRB	[activation]
8928	NRBF2	ULK1	[inhibition]
8929	C3	CFB	[activation;inhibition]
8930	YWHAZ	SIK2	[activation]
8931	TP53	PTTG1IP	[activation]
8932	SHC1	PRMT5	[activation]
8933	RALGAPA1	MYC	[activation]
8934	CFL1	ISG15	[activation;inhibition]
8935	RAD51	SIRT2	[activation]
8936	SRGAP2	FASLG	[activation]
8937	YWHAZ	RAP1GAP	[activation]
8938	RHPN2	SFN	[activation]
8939	EGFR	IRS2	[activation]
8940	ZFYVE9	ETS2	[activation]
8941	NFKBIA	MAP3K14	[activation]
8942	ZAK	SMARCB1	[activation]
8943	TBXA2R	RHOA	[activation;inhibition]
8944	DAB1	APLP2	[inhibition]
8945	SARM1	MYD88	[activation;inhibition]
8946	AGAP3	HSPB1	[activation]
8947	MAX	MGA	[inhibition]
8948	FIS1	NOS3	[activation]
8949	JMY	MDM2	[activation]
8950	PPP2CA	CTTNBP2NL	[inhibition]
8951	EGFR	NCAPG2	[activation]
8952	IKBKE	PAK1	[activation]
8953	YWHAZ	WWC2	[activation]
8954	RANBP2	SP100	[activation]
8955	KNG1	CTSS	[activation]
8956	JAK1	IL7R	[inhibition]
8957	AZI2	TBK1	[inhibition]
8958	MIF	BCL2L11	[activation]
8959	IRF9	STAT1	[activation]
8960	RNPEP	ZFYVE9	[activation]
8961	XRCC3	SCGB3A1	[activation]
8962	RRAGA	RRAGC	[activation]
8963	ITGAV	PXN	[activation]
8964	EIF4A3	ZC3H11A	[activation]
8965	TRAF6	HARS	[activation]
8966	SGK3	SLC9A3R2	[activation]
8967	TNFRSF14	ATXN10	[activation]
8968	MAPK13	MBP	[activation]

8969	EGFR	THEM6	[activation]
8970	GNAO1	APP	[activation]
8971	APC	TFF1	[activation]
8972	SRPK1	CDKL3	[activation]
8973	MAST1	YWHAE	[activation]
8974	RAC3	DDX46	[activation]
8975	MARCKS	CCNE1	[activation]
8976	RAD50	KAT2A	[activation]
8977	DHX37	MYC	[activation]
8978	AURKA	USP9X	[activation]
8979	ITIH5	NOTCH2	[activation]
8980	APBB1	ABL1	[activation]
8981	DTX3	UBE2E3	[activation]
8982	CDKN2A	HIPK4	[activation;inhibition]
8983	RAB10	ASB17	[activation]
8984	NUDT16L1	TP53	[activation]
8985	ELMSAN1	MAPK14	[activation]
8986	ESR1	BCAR1	[activation]
8987	EGFR	ARFGEF1	[activation]
8988	DOCK9	TAF1	[activation]
8989	PPP2R1A	RAB11A	[activation]
8990	IRS2	GRB10	[activation]
8991	CDH5	P2RX6	[activation]
8992	LAMB2	PLEKHA5	[activation]
8993	HSP90AB1	YWHAB	[activation]
8994	NMES1	ABL2	[activation]
8995	ESR2	FBLL1	[activation]
8996	KIF7	CCDC8	[activation]
8997	HIF1A	MCM7	[activation]
8998	PON2	EGFR	[activation]
8999	PPP2R1A	CCDC8	[inhibition]
9000	EIF2B1	EGFR	[activation]
9001	IL1RAP	IRAK1	[activation;inhibition]
9002	LARGE	B3GNT1	[activation]
9003	TGM2	HIST3H3	[activation]
9004	AFF2	GRB2	[activation]
9005	C1orf109	NXF1	[activation]
9006	DLX5	HOXC8	[activation]
9007	GABARAPL2	ENO2	[activation]
9008	MYB	FBXL5	[activation]
9009	COL4A6	DCN	[inhibition]
9010	MS4A1	CCDC155	[activation]
9011	PRKACA	WASF1	[activation]
9012	NUPL1	APC	[inhibition]
9013	KAT2B	TWIST1	[activation]
9014	ANGPT2	CSNK2B	[activation]
9015	PI4K2A	GABARAP	[activation]
9016	YWHAE	WNK1	[activation]
9017	FCGR2C	ITGA4	[activation]
9018	PPP1R3A	PLN	[inhibition]
9019	SNCB	SNCA	[activation]
9020	TLX3	PPP1CC	[activation]
9021	MAML3	MAML1	[activation]
9022	CLCF1	ESR2	[activation]
9023	FAS	TRAF3	[activation]
9024	PYY	MEP1B	[activation]
9025	C1QB	PTX3	[activation]
9026	ROBO1	SRGAP2	[activation]
9027	CASP6	CASP10	[activation]
9028	MIEF2	DNM1L	[activation]
9029	NBEA	NOTCH1	[activation]
9030	BMPR1A	RASA1	[activation]
9031	NIF3L1	DMC1	[activation]
9032	THOP1	TRAF2	[activation]
9033	PRR5L	RICTOR	[activation]
9034	RARA	NCOA3	[activation]
9035	PLEC	ITGA4	[activation]
9036	ZNF321P	SRPK1	[activation]
9037	NCF1	NFKB1	[activation]
9038	CREBBP	YWHAH	[activation]
9039	RLN3	RXFP4	[activation]
9040	FYN	HOXC8	[activation]
9041	MAPT	CASP7	[activation]
9042	POLA2	STK40	[activation]
9043	ATF2	MAPKAPK5	[activation]
9044	KIR3DS1	TYROBP	[activation]

9045	TMSB4X	ACTG1	[activation]
9046	PIK3CD	TAGLN	[activation]
9047	TGFBR3	ENG	[inhibition]
9048	GRB2	HIST1H2BH	[activation]
9049	MAPKAPK2	ZFP36	[activation]
9050	CD44	MET	[activation]
9051	NGFR	LINGO1	[activation]
9052	PRKAA1	HMBOX1	[inhibition]
9053	PDGFRB	SH2B2	[activation]
9054	IQGAP1	MYC	[activation]
9055	FOS	GATA4	[activation]
9056	HSP90AA1	VCAM1	[activation]
9057	TIAM1	SDC1	[activation]
9058	NAPB	STX6	[activation]
9059	CSNK2A1	FGF2	[activation]
9060	RAB21	APPL1	[activation]
9061	L1CAM	FBXO6	[activation]
9062	STRAP	GSK3B	[inhibition]
9063	ROCK2	CDC5L	[activation]
9064	SKP2	YY1	[activation]
9065	NFKB1	CALM1	[activation]
9066	ADAM9	TNF	[activation]
9067	NEB	PTPN11	[activation;inhibition]
9068	GFOD1	NOTCH2NL	[activation]
9069	TELO2	ATM	[activation]
9070	PRDX2	RAD52	[activation]
9071	CD4	ANXA6	[activation]
9072	TFDP1	E2F2	[inhibition]
9073	TNF	TNFRSF1A	[activation]
9074	CHEK2	STMN1	[activation]
9075	ATP6V1H	SOX9	[activation]
9076	POFUT1	NOTCH1	[activation]
9077	RYR1	HOMER3	[activation]
9078	SLC9A3R1	PLCB1	[activation]
9079	MAD2L1	CUL3	[inhibition]
9080	LRIF1	FAS	[inhibition]
9081	PTN	ACTB	[activation]
9082	VAV1	EZH2	[activation]
9083	STXBP1	STX4	[activation]
9084	CTNND2	SPHK1	[inhibition]
9085	UBE2H	TRAIP	[activation]
9086	ESR2	SCRIB	[activation]
9087	MAP2K1	BIRC6	[activation]
9088	EIF2S2	DCC	[activation]
9089	IKBKE	RHOC	[activation]
9090	TNFAIP3	WNK1	[activation]
9091	RASA4	ERBB3	[activation]
9092	PRPF40A	DIAPH1	[activation]
9093	S100A10	ATF2	[activation]
9094	KLF1	CREBBP	[activation]
9095	KRAS	DDX50	[activation]
9096	ESRRA	NR0B1	[activation]
9097	ANXA2P2	ESR1	[activation]
9098	MET	BTK	[activation]
9099	CDH1	EPSTI1	[activation]
9100	SEMA6A	SORBS1	[activation]
9101	PECAM1	SRC	[activation]
9102	TNK2	CRK	[activation]
9103	PIK3R1	EPN1	[activation]
9104	FOXO1	HNF4A	[activation]
9105	PTPRB	MAPK3	[activation]
9106	CDH2	PTPRB	[activation]
9107	CDC25B	ESR1	[activation]
9108	BTRC	FOXO3	[inhibition]
9109	CEBPB	NFKB1	[activation]
9110	AR	CRK	[activation]
9111	ADRA1B	EZR	[activation]
9112	TGFBR2	FSTL1	[activation]
9113	CDC20B	TRAF2	[activation]
9114	KCNA2	PTPRA	[activation]
9115	HDAC1	PIK3R1	[activation]
9116	NRAS	CORO2A	[activation]
9117	PDX1	PRKDC	[activation]
9118	PA2G4	ATG5	[activation]
9119	ATG5	ATG3	[activation]
9120	ANKRD7	HSPB1	[activation]

9121	GIGYF2	CD2	[activation]
9122	EGF	UBE2I	[activation]
9123	CIAO1	MYO1C	[activation]
9124	PRMT1	MBP	[activation]
9125	C9orf156	CCND1	[activation]
9126	BHLHE40	PPP2CA	[activation]
9127	APP	MST4	[activation]
9128	DLG5	CD81	[activation]
9129	RHOBTB2	PPM1D	[activation]
9130	PTBP3	FTSJ1	[activation]
9131	RAC1	ARHGAP27	[activation]
9132	NSD1	NR2E1	[activation]
9133	CSNK1D	LRRK2	[activation]
9134	PTPRJ	KIT	[activation]
9135	IGFBP7	CCL5	[activation]
9136	CLEC4M	TLR2	[activation]
9137	RHOT1	ILK	[activation]
9138	LSM1	PTPRC	[activation]
9139	CUL3	CDK4	[inhibition]
9140	DHPS	NIF3L1	[activation]
9141	RPA3	COBL	[activation]
9142	RELA	CAMK4	[activation]
9143	ETS1	NOMO2	[activation]
9144	HSP90AB1	KLHL23	[activation]
9145	SFRP4	SMAD4	[activation]
9146	DOT1L	HIST2H3A	[activation]
9147	CRK	ABAT	[activation]
9148	PIK3CA	ADAP1	[activation]
9149	EPHB6	EPHB1	[activation]
9150	LAMP2	LRRK2	[activation]
9151	CDK3	TFDP1	[activation]
9152	PTK2	MAL	[activation]
9153	YTHDF2	RPA3	[activation]
9154	SETDB1	PGAM5	[activation]
9155	CCDC97	APBB1	[activation]
9156	SMAD3	IRS1	[inhibition]
9157	CCND1	CDC14B	[inhibition]
9158	MAST3	PRKAA1	[inhibition]
9159	NXF1	BCAP31	[activation]
9160	PIK3R1	EPOR	[activation]
9161	KAT2B	CTBP1	[activation]
9162	EGFR	FRK	[activation]
9163	KAT2A	RBPJ	[activation]
9164	PROP1	EFEMP1	[activation]
9165	SMAD3	RRAS2	[activation]
9166	TWIST1	HIST1H4A	[activation]
9167	CREBBP	RXRG	[activation]
9168	CASP3	BECN1	[activation]
9169	MAPK8	RAC1	[activation]
9170	C11orf83	PHLDA3	[activation]
9171	CTNNB1	PTBP3	[activation]
9172	PSEN1	RNF32	[activation]
9173	MYO6	PAK1	[activation]
9174	UGT1A1	UGT1A8	[activation]
9175	AURKA	IRS4	[activation]
9176	GADD45G	RXRA	[inhibition]
9177	GRB2	VAV1	[activation]
9178	MAP2K1	MAPK8IP3	[activation]
9179	PRDX3	LRRK2	[activation]
9180	EZR	HLA-B	[activation]
9181	RPA3	BCAP31	[activation]
9182	WASF2	FYN	[activation]
9183	NUDC	DCAF4L2	[activation]
9184	ITGB1	ACTN1	[activation]
9185	IST1	ITGA4	[activation]
9186	GDF9	BMPR2	[activation]
9187	PDIA2	PPARG	[activation]
9188	GRB14	PDGFRA	[activation]
9189	CDKN2AIP	RAD21	[activation]
9190	TUBG1	CDK5RAP2	[activation]
9191	ESR1	XPO1	[activation]
9192	C19orf52	APOE	[activation]
9193	FAM120A	NXF1	[activation]
9194	CCNA2	CDC25C	[activation]
9195	PIK3R1	CCL14	[activation]
9196	GNB4	ADRB2	[activation]

9197	PPP1R12B	PPP2R1A	[inhibition]
9198	PSEN1	ICAM5	[activation]
9199	SSB	SPP1	[activation]
9200	TP53	IRS4	[activation]
9201	BIRC6	CASP7	[activation]
9202	DHX34	POLA2	[activation]
9203	CDH2	BOC	[activation]
9204	GNAQ	CCR5	[activation]
9205	EP300	PRMT1	[activation]
9206	HSPA4	MDM2	[activation]
9207	UTP18	KMT2A	[activation]
9208	IRS2	BCL2L1	[activation]
9209	CD4	ENO1	[activation]
9210	STAMBP	TRAF6	[activation]
9211	CCDC102B	ENKD1	[activation;inhibition]
9212	EP300	STRAP	[activation]
9213	A2M	ATF7IP	[inhibition]
9214	BARD1	NPM3	[activation]
9215	YWHAG	CDC25B	[activation]
9216	DVL2	TRAF2	[activation]
9217	PDGFRB	PTPRR	[activation]
9218	TRIM28	SMURF1	[inhibition]
9219	SUV420H2	RBL1	[activation]
9220	PGK1	YWHAZ	[activation]
9221	CAPN1	BID	[activation]
9222	ABL1	EGFR	[activation]
9223	DYRK1A	SFN	[activation]
9224	APPL1	TRAF2	[activation]
9225	UBE2W	RNF138	[activation]
9226	BRD4	JMJD6	[activation]
9227	RANBP2	MYC	[activation]
9228	MAPK6	RAB31	[activation]
9229	VPS35	VCAM1	[activation]
9230	PPP2CA	HUNK	[inhibition]
9231	EGFR	HSPA4	[activation]
9232	FOXH1	TGIF1	[inhibition]
9233	LATS2	SNAI1	[activation]
9234	HPCA	MAP3K10	[activation;inhibition]
9235	SMO	ILK	[inhibition]
9236	ICK	TP53	[activation]
9237	CDK6	TRAK1	[activation]
9238	KIR3DL1	RPS6KB1	[activation]
9239	ITGB1	PXN	[activation]
9240	VCAM1	TOP1	[activation]
9241	PML	TBX2	[inhibition]
9242	MAPT	EGFR	[activation]
9243	CALM1	CRHR1	[activation]
9244	MYOC	CD81	[activation]
9245	ABL1	MAPT	[activation]
9246	PIK3R1	DOCK3	[activation]
9247	AXIN2	AMER3	[inhibition]
9248	WDR96	YWHAE	[activation]
9249	PABPC4	SRPK2	[activation]
9250	ITGA4	PCBP2	[activation]
9251	CDKN1A	CDKN1B	[activation;inhibition]
9252	FGA	PRRC2A	[activation]
9253	BAG2	LRRK2	[activation]
9254	TRAF2	MVP	[activation]
9255	LCP2	YWHAZ	[activation]
9256	TFRC	ASB4	[activation]
9257	SOCS3	PTK2B	[activation]
9258	RPS6KA5	STAT1	[activation]
9259	ABI1	SOS1	[activation]
9260	PIAS2	CDKN2A	[inhibition]
9261	SPP1	DCX	[activation]
9262	RAD21	MTA3	[activation]
9263	DLGAP5	CDK1	[activation]
9264	C18orf56	PRNP	[activation]
9265	RPL13	MAP2K3	[activation]
9266	SMARCA4	WHSC1L1	[activation]
9267	FOXO3	SKP2	[inhibition]
9268	CTTN	ARHGAP8	[activation]
9269	SRF	PYCARD	[activation]
9270	PRKAA1	PPM1E	[inhibition]
9271	FYN	POLD1	[activation]
9272	ESR1	SFN	[activation]

9273 BEND5 CTNNBIP1 [inhibition]
9274 FOXA3 HMGB1 [activation]
9275 HIST2H3A RPS6KA5 [activation]
9276 MYO1C ARRB2 [activation]
9277 RPS17 VCAM1 [activation]
9278 HSP90AB1 ANAPC2 [activation]
9279 NR1H2 NCOA2 [activation]
9280 PIK3R1 ABI1 [activation]
9281 GATC QRSL1 [inhibition]
9282 CHUK PIAS1 [activation]
9283 RPS6KA5 HSPB2 [activation]
9284 NAB2 JAKMIP2 [activation]
9285 INSR ARRB2 [activation]
9286 PARD3 RASSF8 [activation]
9287 HLA-B NACAP1 [activation]
9288 SNX5 PIP5K1C [activation]
9289 PTP4A3 POLD1 [activation]
9290 APP MAB21L2 [activation]
9291 HABP4 ENO2 [activation]
9292 SIRT7 ACACA [activation;inhibition]
9293 CDK11A HSP90AA1 [activation]
9294 CDX2 GSK3B [activation]
9295 MAPRE1 PRKACA [activation]
9296 VASP TES [activation]
9297 GATA4 CRIP2 [activation]
9298 TRAF6 CALCOCO2 [activation]
9299 PTPRC EGFR [activation]
9300 CHEK2 KLK9 [activation]
9301 LRRK2 NCLN [activation]
9302 NDEL1 VCL [activation]
9303 TYK2 RAN [activation]
9304 PCBP2 MYC [activation]
9305 ABL1 SH3BP2 [activation]
9306 APC ANKRD28 [inhibition]
9307 CD81 HIST1H2BM [activation]
9308 RAB11A RAB11FIP4 [activation]
9309 ACTR3 GRB2 [activation]
9310 MFAP4 GRB2 [activation]
9311 PHKG1 CALM1 [activation]
9312 BAG3 STMN1 [activation]
9313 PIK3CG USP45 [activation]
9314 TLR4 HMGB1 [activation]
9315 PRKAA2 GLI1 [inhibition]
9316 FBXO6 ERO1LB [activation]
9317 TNFRSF14 GRB2 [activation]
9318 VTCN1 BTLA [activation]
9319 ANO5 EGFR [activation]
9320 CCHCR1 NAB2 [activation]
9321 FBP2 AXIN2 [activation]
9322 VAMP3 SCAMP5 [activation]
9323 ATM MDC1 [activation]
9324 DTX1 EGR2 [activation]
9325 CRK GABBR1 [activation]
9326 KCNB1 KCNS3 [activation]
9327 PTPRC CD28 [activation]
9328 ABL1 NOTCH3 [activation]
9329 YWHAZ RAPGEF2 [activation]
9330 PPP3CA MYOZ2 [activation]
9331 MAP3K3 ACTG1 [activation]
9332 PAXIP1 KRI1 [activation]
9333 PIK3R1 GSPT1 [activation]
9334 PXN PDPK1 [activation]
9335 ESR1 SRPK2 [activation]
9336 PAK3 ITSN1 [activation]
9337 RYR2 CALM1 [activation]
9338 NR3C1 BAG1 [activation]
9339 C3AR1 C4A [activation]
9340 TERT ACD [activation]
9341 STAT6 MAVS [activation]
9342 DIAPH1 SNW1 [activation]
9343 LTB LTA [activation]
9344 NEDD9 CRK [activation]
9345 COMMD9 NFKB1 [activation]
9346 DUSP9 GSK3B [inhibition]
9347 CD81 CR2 [activation]
9348 DDX58 PRKRA [activation]

9349	ABL1	CDC27	[activation]
9350	CSNK1D	LURAP1	[activation;inhibition]
9351	MYLK	PRKG2	[inhibition]
9352	CCAR2	TP53	[inhibition]
9353	POU1F1	PPARA	[activation]
9354	KNG1	CAPN2	[activation]
9355	SCG2	COPS6	[activation]
9356	DSG1	GH1	[activation]
9357	LDHB	LRRK2	[activation]
9358	EIF2AK1	CDC37	[activation]
9359	NTRK2	SH2B2	[activation]
9360	CD2	CD4	[activation]
9361	RHO	ADRBK1	[activation]
9362	RAP1GAP2	YWHAE	[activation]
9363	TSHR	GNA13	[activation]
9364	PAK4	RAC1	[activation]
9365	BRCA1	CCNA2	[activation]
9366	RPTOR	SIRT1	[activation;inhibition]
9367	IL1RAP	PIK3R1	[activation]
9368	KDM6A	SRF	[activation]
9369	ANXA3	IGSF21	[activation]
9370	GRIA4	CACNG2	[activation]
9371	CHGB	RAP1B	[activation]
9372	SAV1	YWHAE	[activation]
9373	SRPK1	TNFRSF10C	[activation]
9374	SOS2	CRKL	[activation]
9375	PPP1R16A	FAM101B	[activation]
9376	HIST1H1D	IRAK4	[activation]
9377	GNAI1	CNR1	[activation]
9378	CD14	FSTL1	[activation]
9379	ATF2	RAB7A	[activation]
9380	TEP1	TP53	[activation]
9381	COPS7A	PTGS2	[activation]
9382	PAK2	GDI2	[activation]
9383	AURKA	FBP2	[activation]
9384	CD8A	HLA-G	[activation]
9385	BTRC	BTG1	[activation]
9386	RAC2	NOXA1	[activation]
9387	VCAM1	ARF1	[activation]
9388	HSPA1L	TRADD	[activation]
9389	GLI1	SMAD2	[inhibition]
9390	FLOT1	FYN	[activation]
9391	GABRB2	PPP3CA	[activation]
9392	NFYA	APP	[activation]
9393	SH3BP2	GRB2	[activation]
9394	CENPA	DIAPH2	[activation]
9395	NOTCH1	NCKAP1L	[activation]
9396	NFIL3	TRAF2	[activation]
9397	GZMM	EZR	[activation]
9398	IL17A	TRAF3IP2	[activation]
9399	NFKB1	IL2RA	[activation]
9400	CDC5L	WHSC1	[activation]
9401	HDLBP	STK4	[activation]
9402	OGT	TET1	[activation]
9403	ATG4C	PRKDC	[activation]
9404	SERBP1	VAV2	[activation]
9405	CMA1	EDN1	[activation]
9406	PPARGC1A	MED1	[activation]
9407	MAPK3	MAGED1	[activation]
9408	SAMD3	FANCG	[activation]
9409	EHMT2	PLOD3	[activation]
9410	ITGA2B	GRB2	[activation]
9411	CAPZA1	FAM21C	[activation]
9412	ACTB	RAD51	[activation]
9413	VAV3	ESR1	[activation]
9414	CCDC155	SENP2	[activation]
9415	GDI2	TRAF6	[activation]
9416	STX16	STX4	[activation]
9417	SLC9A7	SCAMP5	[activation]
9418	MAPK3	LRPAP1	[inhibition]
9419	IGKV1-5	SLX4	[activation]
9420	LAT	PTPRJ	[activation]
9421	PPP1CC	ACTG1	[activation]
9422	HSP90AA1	EPAS1	[activation]
9423	CSF3R	JAK2	[activation]
9424	TRIP6	S1PR1	[activation]

9425	IL2RA	SNTA1	[activation]
9426	SMG5	TERT	[activation]
9427	TUBA4A	ENO2	[activation]
9428	MDM2	H1FX	[activation]
9429	NCK1	CD3E	[activation]
9430	FZD2	STAT3	[activation]
9431	CRK	SH3KBP1	[activation]
9432	TP53	RANBP9	[activation]
9433	NUMBL	EGFR	[activation]
9434	NAA38	VCAM1	[activation]
9435	GTPBP1	CDC5L	[activation]
9436	STAT3	LAMB2	[activation]
9437	PRKCA	PRKCE	[activation]
9438	ARNT	HIF1A	[activation]
9439	PCDH9	BCL6	[activation]
9440	SAA2	LAMA1	[activation]
9441	SOX10	DLX5	[activation]
9442	ASB5	ENO2	[activation]
9443	OLFM2	SRSF5	[activation]
9444	PML	RXRA	[inhibition]
9445	PRKCD	PRKCB	[activation]
9446	BRK1	ROBO1	[activation]
9447	APP	LPL	[activation]
9448	SPDYA	CDKN1B	[inhibition]
9449	DDX27	PPP1R16A	[activation]
9450	VAPA	VCAM1	[activation]
9451	MAPK13	NUDC	[activation]
9452	GH1	TMCO1	[inhibition]
9453	STK3	GAD1	[activation]
9454	RBL1	CDKN1C	[inhibition]
9455	ABL1	CENPA	[activation;inhibition]
9456	MAPT	MARK4	[activation]
9457	FTSJ1	CYCS	[activation]
9458	PAK7	ARRB2	[inhibition]
9459	PGK1	HIF1A	[activation]
9460	MS4A1	CREB3	[activation]
9461	ADAM17	JAG1	[activation]
9462	APCS	C1QA	[activation]
9463	RNF8	RAD50	[activation]
9464	TP53	TP53RK	[activation]
9465	PMS2	RASA1	[activation]
9466	EPX	PLCG1	[activation]
9467	FYN	PECAM1	[activation]
9468	SUFU	PIAS1	[inhibition]
9469	TSSK6	HSP90AA1	[activation]
9470	FANCI	CAMKK2	[activation]
9471	WNK4	KLHL2	[activation]
9472	SUMO1	EIF2B2	[activation]
9473	CDK4	H1F0	[inhibition]
9474	PAFAH1B1	DAB1	[activation]
9475	CCR1	STAT1	[activation]
9476	IKBKB	TNFAIP3	[activation]
9477	ADAP2	NCL	[activation]
9478	PGR	KAT2B	[activation]
9479	EP300	PRKCD	[activation]
9480	ILK	PARVB	[activation]
9481	SFN	KLHDC2	[activation]
9482	AK2	GH1	[inhibition]
9483	TRIP6	PRKAA2	[activation]
9484	FMNL1	TIA1	[activation]
9485	GRB2	TESPA1	[activation]
9486	HDAC4	AR	[activation]
9487	RAP1B	TP53	[activation]
9488	DAPK1	IFI30	[activation]
9489	FAM71C	DNAJB9	[inhibition]
9490	LAMTOR4	RRAGB	[activation]
9491	LAMTOR5	RRAGD	[activation]
9492	RAB11A	TRAF6	[activation]
9493	HCK	VAV1	[activation]
9494	NOS3	HDAC3	[activation]
9495	PTPN12	JAK2	[activation]
9496	BARD1	CCDC136	[activation]
9497	ACAP1	ABL1	[activation]
9498	PIK3R3	LUC7L2	[activation]
9499	GOSR1	PKP1	[activation]
9500	SLC7A6	ESR2	[activation]

9501	IGF2BP1	AURKA	[activation]
9502	XRCC1	FYN	[activation]
9503	ERBB2IP	SMAD4	[inhibition]
9504	CTNNBIP1	NACAP1	[inhibition]
9505	PCNA	DDX11	[activation]
9506	PIAS1	MYB	[activation]
9507	GSK3A	GSKIP	[activation;inhibition]
9508	CASP8	IKBKG	[activation]
9509	PTPRF	BCAR1	[inhibition]
9510	NR3C1	NCOA4	[activation]
9511	LONRF1	NR4A1	[inhibition]
9512	SMAD4	MSANTD3	[activation]
9513	JUN	SUMO4	[activation]
9514	RNF38	UBE2H	[activation]
9515	MDM2	IRF1	[activation]
9516	MAPK8	FAS	[activation]
9517	EIF4A3	RPS24	[activation]
9518	ERBB4	TAB2	[activation]
9519	YAP1	IQGAP1	[inhibition]
9520	NUDC	MAP3K3	[activation]
9521	GHRHR	GHRL	[activation]
9522	MAPKAPK5	EIF4E	[activation]
9523	CDK1	MLKL	[activation]
9524	SUMO2	NBN	[activation]
9525	BAD	GIMAP5	[activation]
9526	ABCF3	SPP1	[activation]
9527	MDM2	HSPE1	[activation]
9528	PPP2R2B	PTK2B	[activation]
9529	PDIA4	EIF2B1	[activation]
9530	ETS2	STAT5B	[activation]
9531	FYN	ENO1	[activation]
9532	COL1A2	APP	[activation]
9533	NUP62	LRRK2	[activation]
9534	SMAD2	EIF5B	[activation]
9535	PRKCI	GAPDH	[activation]
9536	VASP	XIRP1	[activation]
9537	GSK3B	PXN	[activation]
9538	CDKN1A	NGFR	[activation;inhibition]
9539	APC	ASAP1	[inhibition]
9540	NXF1	SMARCC1	[activation]
9541	NOTCH2	ANKRD28	[activation]
9542	P2RY1	SLC9A3R1	[activation]
9543	RPS19	VCAM1	[activation]
9544	CACNB1	CACNA1C	[activation]
9545	CACNA1A	BTG3	[inhibition]
9546	UBA5	ATG101	[activation]
9547	ARHGAP32	GRB2	[activation]
9548	PIN1	TRAF2	[activation]
9549	STX5	YKT6	[activation]
9550	SMAD2	PPARG	[activation]
9551	ITGA6	ADAM9	[activation]
9552	ARF6	ARRB1	[activation]
9553	ANGPT2	LYN	[activation]
9554	ARF1	TMED10	[activation]
9555	DCLK3	CDK5	[activation]
9556	ADPRH	SRPK1	[activation]
9557	KRTAP10-3	XCL1	[activation]
9558	NOS2	CALM1	[activation]
9559	ELMO1	RHOG	[activation]
9560	TRPV4	HCK	[activation]
9561	HIST2H3A	AURKB	[activation]
9562	FKBP8	CAMK2A	[activation]
9563	PTK6	KHDRBS1	[activation]
9564	TRAF6	CD81	[activation]
9565	STK16	MIIP	[activation]
9566	MCM5	EP300	[activation]
9567	NKTR	MAPK14	[activation]
9568	GEM	CCDC102B	[activation]
9569	HNRNPA0	ESR1	[activation]
9570	HIF1A	SHC1	[activation]
9571	ACTG1	SUMO3	[activation]
9572	SPRED2	KIT	[activation]
9573	BMPR2	CES1	[activation]
9574	POLR2A	TRAK1	[activation]
9575	PRKAA1	PPP2CA	[inhibition]
9576	SOS1	ITSN2	[activation]

9577	HLA-B	ARHGEF4	[activation]
9578	YWHAZ	CDKN1B	[activation;inhibition]
9579	SUMO1	PRKDC	[activation]
9580	PDGFRL	PPP3R2	[activation;inhibition]
9581	GTF2A1	BCL3	[activation]
9582	SRC	STAP2	[activation]
9583	PTPRC	JAK2	[activation]
9584	CAPN1	FANCG	[activation]
9585	PLCB1	PARD6A	[activation]
9586	MET	LCK	[activation;inhibition]
9587	RPS24	MDM2	[activation]
9588	RPS6KA1	CARHSP1	[activation]
9589	JAG1	THBS1	[activation]
9590	LAMTOR2	RPTOR	[activation]
9591	NR1H3	PPARA	[activation]
9592	WDR92	PARD6A	[activation]
9593	ATF4	NFE2	[activation]
9594	TBP	FOS	[activation]
9595	PRDX2	MAST1	[activation]
9596	MAPK3	UBTF	[activation]
9597	EP300	USF2	[activation]
9598	WAS	EGFR	[activation]
9599	IL1R1	PDGFRB	[activation]
9600	TNFSF11	SRC	[activation]
9601	RPA1	SMC2	[activation]
9602	APC	NANS	[inhibition]
9603	PAK1	ZBTB18	[activation]
9604	PPP2R1A	GABARAP	[activation]
9605	EP300	CEBPD	[activation]
9606	PRKCZ	PRG2	[activation]
9607	MDM2	CDKN1A	[activation;inhibition]
9608	CDKN1A	QARS	[activation;inhibition]
9609	MAP3K7	SMAD3	[activation]
9610	DVL1P1	APC	[inhibition]
9611	CAB39L	MLH1	[activation]
9612	TP53	DBN1	[activation]
9613	VCAM1	RAP1B	[activation]
9614	CREBBP	HNF1A	[activation]
9615	BARD1	TMEM248	[activation]
9616	SKI	SIX1	[activation]
9617	AKT1	SIRT1	[activation]
9618	PRMT5	LUC7L	[activation]
9619	TH	RPS6KA5	[activation]
9620	PDIA3	STIM1	[activation]
9621	DCN	INSR	[activation]
9622	RECQL5	PLK1	[activation]
9623	AZI1	LATS1	[inhibition]
9624	APC	DLGAP1	[inhibition]
9625	ICAM1	ACTB	[activation]
9626	RPA1	RAB5C	[activation]
9627	JUNB	EP300	[activation]
9628	OTX2	LHX1	[activation]
9629	GSK3A	PRKDC	[activation]
9630	RTN2	FANCG	[activation]
9631	RPA2	RPA1	[activation]
9632	RELA	ABCA1	[activation]
9633	CDC25A	FGF21	[activation]
9634	BET1L	GOSR1	[activation]
9635	UBE2H	RNF4	[activation]
9636	WNK1	PFKP	[activation]
9637	STK38	HSP90AA1	[activation]
9638	RFX1	NOTCH1	[activation]
9639	COL4A4	OSM	[activation]
9640	NR3C1	AKT1	[activation]
9641	CRK	NXPH3	[activation]
9642	RBL1	LAMB2	[activation;inhibition]
9643	PRKCI	GBAS	[activation]
9644	GRB2	SHB	[activation]
9645	LRRFIP2	MYD88	[activation;inhibition]
9646	ACP5	PPP2CB	[activation]
9647	CBLB	BTK	[activation]
9648	GNAQ	RIC8A	[activation]
9649	C15orf57	HSPB1	[activation]
9650	EGR2	ACP5	[activation]
9651	GRB2	MYH11	[activation]
9652	RALA	EXOC3	[activation]

9653	IL1RAP	TICAM2	[activation]
9654	MAP3K2	MAP3K3	[activation]
9655	CCND1	FBXO31	[inhibition]
9656	CEBPB	HDAC1	[activation]
9657	KLK6	COL4A6	[activation]
9658	E2F1	CDK7	[activation]
9659	DERL2	SEL1L	[activation]
9660	GNAS	ADORA1	[activation]
9661	NCOR1	NFE2L2	[activation]
9662	SRC	KIT	[activation]
9663	GNAL	BABAM1	[inhibition]
9664	RAP1B	APP	[activation]
9665	SATB2	PIAS1	[inhibition]
9666	SLC25A6	GABARAP	[activation]
9667	PPM1B	VHL	[inhibition]
9668	IQGAP1	PIK3R2	[activation]
9669	LIFR	CNTFR	[activation]
9670	ACTR2	SNW1	[activation]
9671	GAB1	VAV2	[activation]
9672	STK4	MOB4	[activation]
9673	CDC5L	CNOT10	[activation]
9674	GABARAPL1	OPRK1	[activation]
9675	SRPK1	SEC23B	[activation]
9676	KCNE4	KCNA1	[activation]
9677	IL1B	A2M	[inhibition]
9678	PRKAA1	PRKAG2	[inhibition]
9679	CDC5L	ETS1	[activation]
9680	MMP2	CCL7	[activation]
9681	WASL	CTTN	[activation]
9682	STRAP	EGFR	[activation]
9683	PRKCQ	CASP3	[activation]
9684	TRAF2	PKN1	[activation]
9685	ITGB1BP1	ITGB1	[activation]
9686	SKP2	IFI27	[inhibition]
9687	LZTR1	HCLS1	[activation]
9688	CALM1	MAP3K8	[activation]
9689	MYB	FBXW7	[activation]
9690	MAPKAPK2	UBE2J1	[activation]
9691	RFX6	ARNT2	[activation]
9692	GRB2	SPRYD4	[activation]
9693	TDRD7	CCND1	[activation]
9694	ANKRD54	BLK	[activation]
9695	PPARG	ZNHIT3	[activation]
9696	PTPRC	PKP4	[activation]
9697	CD93	C1QA	[activation]
9698	CCT8	MAPK8	[activation]
9699	SKIL	MORC4	[activation]
9700	NAIP	MAP3K7	[inhibition]
9701	ENO2	SUMO1	[activation]
9702	TRAF6	APP	[activation]
9703	C20orf112	SRPK1	[activation]
9704	NOTCH1	ANAPC1	[activation]
9705	TP73	DAB2IP	[activation]
9706	LRRK1	VGF	[activation]
9707	CEP350	PPP2CA	[inhibition]
9708	RGS14	NFKB1	[activation;inhibition]
9709	CTSC	CST7	[inhibition]
9710	ARHGAP26	SRPK1	[activation]
9711	YWHAQ	MYL3	[inhibition]
9712	PRKAA1	MARK3	[inhibition]
9713	CDC42BPG	RHOQ	[activation]
9714	PKM	POLE2	[activation]
9715	GAB1	PTPRC	[activation]
9716	SMAD4	RAC2	[activation]
9717	EPAS1	PRKD1	[activation]
9718	YWHAZ	TAB2	[activation]
9719	SERPINB3	MAPK8	[inhibition]
9720	SNCA	MAPT	[activation]
9721	CHEK1	CLSPN	[activation]
9722	LMF1	LIPC	[activation]
9723	BTK	HCK	[activation]
9724	SRGAP3	WASF1	[activation]
9725	HMGB1	HOXB3	[activation]
9726	ARFGAP1	APOE	[activation]
9727	YWHAE	KPNB1	[activation]
9728	TLR3	CD14	[activation]

9729	RBBP8	BARD1	[activation]
9730	NOS1	RASD1	[activation]
9731	EXOC5	C9orf9	[inhibition]
9732	ADAMTSL4	RHOJ	[activation]
9733	MLH1	RAD50	[activation]
9734	GIT1	PAK2	[activation]
9735	NPAS2	KAT2B	[activation]
9736	GRB2	KHDRBS1	[activation]
9737	SHC1	MAPKAPK2	[activation]
9738	RYR2	CFTR	[activation]
9739	PDGFB	COL2A1	[activation]
9740	NTF4	BDNF	[activation]
9741	MASP2	SERPING1	[inhibition]
9742	FCRL2	NCK2	[activation]
9743	TNF	SPOCK1	[activation]
9744	CCT8	ILK	[activation]
9745	PIK3R4	PIK3C3	[activation]
9746	MED24	TNFSF11	[activation]
9747	ASCC2	PIK3CD	[activation]
9748	CASP8AP2	PIAS1	[inhibition]
9749	GNB2L1	SLC25A6	[activation]
9750	RPS6KB1	XPO1	[activation]
9751	SUMO2	NFE2L2	[activation]
9752	SRGAP2	FMNL1	[activation]
9753	SUV39H1	FYN	[activation]
9754	RALA	CTCF	[activation]
9755	PAXIP1	ECSIT	[activation]
9756	PSEN2	CASP8	[activation]
9757	PRDX2	VCAM1	[activation]
9758	APP	TRPT1	[activation]
9759	RGS20	LCE2D	[activation;inhibition]
9760	CDH2	UBC	[activation]
9761	ESR1	HYOU1	[activation]
9762	MET	HGFAC	[activation;inhibition]
9763	ILF2	RAD50	[activation]
9764	CAPN1	DES	[activation]
9765	ACTB	RPA1	[activation]
9766	EGFR	CHD5	[activation]
9767	EEF1A2	PTEN	[activation;inhibition]
9768	PMS2	SFRP4	[activation]
9769	BCR	PIK3R2	[activation]
9770	SIRT1	MCF2L2	[activation]
9771	SMAD1	KMT2D	[activation]
9772	HERPUD1	CD3D	[activation]
9773	FABP3	NUP62	[activation]
9774	SUV39H1	GPATCH2L	[activation]
9775	BMP1	TP53	[activation]
9776	SMEK1	LRRK2	[activation]
9777	ORC4	CCND1	[activation]
9778	COMT	RGS2	[activation]
9779	ITGA4	LPXN	[activation]
9780	SORBS1	MAP4K3	[activation]
9781	HDAC3	SRF	[activation]
9782	CASP6	MAPT	[activation]
9783	TBC1D5	ULK1	[inhibition]
9784	ADORA1	ADORA2A	[activation]
9785	F2RL2	GRB2	[activation]
9786	FAM118B	RASGRP2	[activation]
9787	HSP90AB1	EPHB1	[activation]
9788	TRAF3	TNFRSF9	[activation]
9789	GDF5	ACVR2B	[activation]
9790	DAAM1	SRC	[activation]
9791	GRN	SGTA	[activation]
9792	GAB2	MARK2	[activation]
9793	GRB2	SKAP1	[activation]
9794	NCOA3	BMP7	[activation]
9795	HSPA4L	HLA-B	[activation]
9796	TBK1	IKBKB	[activation]
9797	TRAF6	UBE2D4	[activation]
9798	MAP3K4	MAP2K1	[activation]
9799	HLA-B	SSR1	[activation]
9800	CEP250	NUP62	[activation]
9801	IER3	PRNP	[activation]
9802	RAN	MAD2L2	[activation]
9803	GRB2	MSI2	[activation]
9804	SCYL3	EZR	[activation]

9805	RICTOR	ILK	[activation]
9806	CD1D	B2M	[activation]
9807	ITGA5	ERBB2	[activation]
9808	EIF2AK2	HSP90AB1	[activation]
9809	CD9	CD81	[activation]
9810	TGFBR2	CDC42	[activation]
9811	CD247	SIK1	[activation]
9812	UBE2B	NBN	[activation]
9813	XIAP	RAC2	[activation]
9814	FBXO18	HSP90AB1	[activation]
9815	SNAI1	PALB2	[activation]
9816	NR3C1	NFKB1	[activation]
9817	SFN	RAE1	[activation]
9818	ANXA5	HUWE1	[activation]
9819	DLG2	NOS1	[activation]
9820	SUPT4H1	ANXA5	[activation]
9821	DBNDD2	CSNK1D	[activation]
9822	SETD8	FAM9A	[activation]
9823	PICALM	PELI2	[activation]
9824	RPS6KA5	MBP	[activation]
9825	TRAF1	GIT2	[activation]
9826	ERAP1	IL6R	[activation]
9827	MAPK9	HDAC1	[activation]
9828	TNFRSF9	LRR1	[activation]
9829	XRCC1	RPA3	[activation]
9830	PAXIP1	ATHL1	[activation]
9831	CDC20	ANAPC11	[inhibition]
9832	MAPKAP1	MLST8	[activation]
9833	RARA	CDK7	[activation]
9834	ULK1	ATG13	[inhibition]
9835	MSL2	TP53	[activation]
9836	HSP90AB1	TSSK6	[activation]
9837	MAPK1	RXRA	[inhibition]
9838	BCAP31	CASP3	[activation]
9839	PROSER2	STK24	[activation]
9840	SHB	PDGFRA	[activation]
9841	RPS6KB1	POLDIP3	[activation]
9842	MAPK3	MBP	[activation;inhibition]
9843	HSPB1	RAB43	[activation]
9844	HSPA4	RICTOR	[activation]
9845	PDCD6IP	PLXNA2	[activation]
9846	MAPRE1	TRAF6	[activation]
9847	RAD52	RPS19	[activation]
9848	SGOL2	PPP2R5A	[activation]
9849	MAP2K6	SMAD7	[activation]
9850	HSP90B1	TLR4	[activation]
9851	DIABLO	CDC40	[activation]
9852	STX2	VAPB	[activation]
9853	S1PR5	GNA12	[activation]
9854	LTK	TNK2	[activation]
9855	DRD5	GABRG2	[activation]
9856	WNK1	CSDE1	[activation]
9857	CASP9	PRKCZ	[activation]
9858	LILRB1	B2M	[activation]
9859	INADL	PARD6A	[activation]
9860	CFL1	MAPK6	[activation;inhibition]
9861	MAPK8	PRKDC	[activation]
9862	RXRB	TACC2	[inhibition]
9863	FBXO18	RPA3	[activation]
9864	JUN	SUMO1	[activation]
9865	BCAR1	PTK2B	[activation]
9866	EXT2	EXT1	[activation]
9867	ESR1	PIAS2	[activation]
9868	SKIV2L2	VCAM1	[activation]
9869	HOXB1	EP300	[activation]
9870	RASIP1	RRAS	[activation]
9871	HSPA8	IKBKE	[inhibition]
9872	CASP10	CASP8AP2	[activation]
9873	ICT1	HSPA9	[activation]
9874	UGT1A1	B3GALT1	[activation]
9875	RCHY1	CAMK2A	[activation;inhibition]
9876	NCK2	MET	[activation]
9877	HLA-B	PTGES3	[activation]
9878	ELAVL1	ATM	[activation]
9879	BAX	UVRAG	[activation]
9880	SYK	AR	[activation]

9881 MYC DOCK7 [activation]
9882 LIN37 MYLPF [activation]
9883 SMAD4 USP9X [activation]
9884 PTPLAD1 LRRK2 [activation]
9885 NXF1 SHC1 [activation]
9886 PSMC3IP FGFR4 [activation]
9887 CXorf22 ESR1 [activation]
9888 CHRDL2 BMP7 [activation]
9889 H2AFX EIF2S2 [activation]
9890 YWHAG MAP3K3 [activation]
9891 PTGER2 ARRB2 [activation]
9892 MECOM JUN [activation]
9893 LRRK2 MKI67 [activation]
9894 NPRL2 ANXA7 [activation]
9895 ATG5 ATG13 [activation]
9896 MAPK13 MAPT [activation]
9897 SMAD7 MYOD1 [activation]
9898 SIX1 FZR1 [activation]
9899 RCC1 NGFR [activation]
9900 STX7 GTF2I [activation]
9901 STMN1 VCAM1 [activation]
9902 MC4R ATRNL1 [activation]
9903 VTA1 TRAF6 [activation]
9904 STX11 SNAP23 [activation]
9905 IGF1R KLK5 [activation]
9906 NTRK3 RASGRF1 [activation]
9907 ZIC1 GLI1 [inhibition]
9908 GK AOX1 [activation]
9909 NGF PLG [activation]
9910 HOXB3 CREBBP [activation]
9911 NOTCH1 CAMK4 [activation]
9912 CCL5 SDC1 [activation]
9913 EP300 DECR2 [activation]
9914 CAMK2B ITPKA [activation]
9915 ZAP70 IFNAR1 [activation]
9916 NUDC TP53BP1 [activation]
9917 NOTCH1 WDR12 [activation]
9918 PGM1 ENO3 [activation]
9919 IL2RA IL2RB [activation]
9920 EGFR RAF1 [activation]
9921 MYOD1 SP1 [activation]
9922 KPNB1 EEA1 [activation]
9923 CYP17A1 STK11 [activation]
9924 PTK2 DEF6 [activation]
9925 NIPSNAP1 MDM2 [activation]
9926 CDC42 DOCK8 [activation]
9927 CAND1 SRPK3 [activation]
9928 CD93 MYD88 [activation]
9929 RAD50 SKP2 [activation]
9930 ARRB2 IGF1R [activation]
9931 BCL3 CTBP1 [activation]
9932 YWHAZ CTNNB1 [activation]
9933 CREM CAMK2G [inhibition]
9934 LCE3E NOTCH2NL [activation]
9935 EHMT1 BMI1 [activation]
9936 STAT3 EZH2 [activation]
9937 ESR1 IRS1 [activation]
9938 MYC AHNAK2 [activation]
9939 YAE1D1 TPD52L1 [activation;inhibition]
9940 APP APBB1 [activation]
9941 LRP1 MMP15 [activation]
9942 OBSL1 TBL3 [activation;inhibition]
9943 ZFP36 CCL3 [activation]
9944 ITGA4 EIF4A1 [activation]
9945 EGFR CAV3 [activation]
9946 LYN CD24 [activation]
9947 PIGR MIS12 [activation]
9948 MAPK1 NAV1 [activation]
9949 E2F3 FHL2 [inhibition]
9950 PPP2R1B EGFR [activation]
9951 SHC1 PTPN12 [activation]
9952 SRPK2 BARD1 [activation]
9953 CHEK2 CDC25A [activation]
9954 ANXA5 TINF2 [activation]
9955 SEMA6A SORBS2 [activation]
9956 FOXO1 AR [activation]

9957	POLR1D	CRKL	[activation]
9958	STX7	VPS16	[activation]
9959	SHC1	GRB7	[activation]
9960	CD97	SRC	[activation]
9961	RB1	PAX8	[activation]
9962	RYR1	PPP2CA	[activation]
9963	PRMT5	SNAI1	[activation]
9964	NCK1	EPHB2	[activation]
9965	BTK	PIP5K1B	[activation]
9966	HSPA1L	CDC20	[inhibition]
9967	SMAD1	LEF1	[activation]
9968	NCK1	F2RL2	[activation]
9969	STK11	CRY2	[activation]
9970	ETS1	ZFR	[activation]
9971	RNF181	ITGA2B	[activation]
9972	METTL22	HSPA8	[inhibition]
9973	PRKAA2	AGL	[inhibition]
9974	GC	GRB2	[activation]
9975	CDC42	RAC1	[activation]
9976	YAP1	SCAMP3	[inhibition]
9977	SRSF1	NFYA	[activation]
9978	GRB2	TUBB	[activation]
9979	SHC1	TIMM50	[activation]
9980	EGFR	MAPK14	[activation]
9981	UNC93B1	TLR9	[activation]
9982	EIF3K	ICAM1	[activation]
9983	ABCA1	LIN7C	[activation]
9984	FZR1	ANAPC4	[activation]
9985	MDM2	ESR1	[activation]
9986	TGFB1I1	SVIL	[activation]
9987	PLCG1	RIN3	[activation]
9988	MANBAL	CACNA1A	[inhibition]
9989	BID	CRMP1	[activation]
9990	CCDC53	ABI2	[activation]
9991	EPM2A	PRKAA1	[inhibition]
9992	CMPK1	BACE1	[activation]
9993	PYHIN1	MDC1	[activation]
9994	TBX21	GATA3	[inhibition]
9995	KMT2A	MLLT4	[activation]
9996	CDKN2A	HIF1A	[inhibition]
9997	AURKB	FTH1	[activation]
9998	COPS3	CTNNB1	[activation]
9999	MAGED1	ILK	[activation]
10000	IKBKB	MAP3K11	[activation]
10001	HERPUD1	PSEN1	[activation]
10002	NMI	STAT4	[activation]
10003	HSPH1	PAFAH1B3	[activation]
10004	STK11	RPS6KA5	[activation]
10005	HNRNPA2B1	MAP4K2	[activation]
10006	CCK	CCKAR	[activation]
10007	ACTB	HSPA8	[activation]
10008	HSP90AB1	KBTBD4	[activation]
10009	STX4	STX8	[activation]
10010	EIF1B	RHOB	[activation]
10011	TP53	NR3C1	[activation]
10012	YWHAZ	WNK4	[activation]
10013	ATG5	NEDD8	[activation]
10014	SRC	FANCA	[activation]
10015	MYC	TPP1	[activation]
10016	CDC5L	STAT3	[activation]
10017	YARS	ITGA4	[activation]
10018	FKBP3	TP53	[activation]
10019	PSMC3IP	RXRA	[inhibition]
10020	CTNND1	CDH3	[activation]
10021	CD79B	CD5	[activation]
10022	PIAS1	PIAS4	[inhibition]
10023	RAC1	LRRK2	[activation]
10024	CSNK2A1	DVL3	[activation]
10025	SLC24A1	GRB2	[activation]
10026	RELA	IL1RN	[inhibition]
10027	APP	IFNA16	[activation]
10028	RIPK1	RPL23	[activation]
10029	BCAP31	RPA2	[activation]
10030	PSMC3IP	CHEK2	[activation]
10031	NOXA1	CACNA1A	[activation]
10032	HSPA6	TAB1	[inhibition]

10033	IFNAR1	PIK3R1	[activation]
10034	LRRK2	ZRANB2	[activation]
10035	RHOBTB2	RHOBTB3	[activation]
10036	CSNK2B	MAPK14	[activation]
10037	CDKN1A	SETDB1	[activation]
10038	SMARCB1	SMARCA2	[activation]
10039	GABARAP	FASN	[activation]
10040	PRKACA	AKAP13	[activation]
10041	CLSPN	PRIMPOL	[activation]
10042	CSNK2B	PAXIP1	[activation]
10043	ESR2	MCM2	[activation]
10044	MYL12A	PAXIP1	[activation]
10045	SUMO1	OBSCN	[activation]
10046	NR4A1	STAT3	[inhibition]
10047	TNF	PTPRZ1	[activation]
10048	JAK1	IRS2	[activation]
10049	ALK	PIK3R1	[activation]
10050	PRKAA1	PNMA5	[inhibition]
10051	IL37	CASP4	[activation]
10052	SETD4	NXF1	[activation]
10053	STAM	YWHAB	[activation]
10054	E2F1	BTRC	[activation]
10055	ADRB2	GPRC5B	[activation]
10056	PLEKHA8	ARF1	[activation]
10057	SKAP2	FASLG	[inhibition]
10058	PPP1R2	GSK3A	[activation;inhibition]
10059	YWHAG	MCM5	[activation]
10060	ZAP70	APP	[activation]
10061	MPG	PRNP	[activation]
10062	SFN	BCR	[activation]
10063	MAPK1	RGS19	[activation;inhibition]
10064	HSPA14	PAXIP1	[activation]
10065	TPD52L1	SFN	[activation]
10066	RERE	EHMT2	[activation]
10067	GNB2L1	AURKB	[activation]
10068	MEIS1	CREB1	[activation]
10069	RASSF9	DVL2	[activation]
10070	RANBP2	RANGAP1	[activation]
10071	QKI	VCAM1	[activation]
10072	SMAD2	PIAS4	[inhibition]
10073	CDH2	PTPRK	[activation]
10074	PRKCH	BANP	[activation]
10075	SRPK1	KCNN2	[activation]
10076	INADL	KCNJ15	[activation]
10077	AR	IL6ST	[activation]
10078	STX4	RAB11A	[activation]
10079	MAGI1	RASA1	[activation]
10080	PFKFB2	YWHAZ	[activation]
10081	SMARCC1	TP53	[activation]
10082	PRKCA	ANXA7	[activation]
10083	INSIG2	PSMD4	[activation]
10084	DNAJB1	ATG2A	[inhibition]
10085	MTOR	PREX1	[activation]
10086	RXRG	PPARG	[activation]
10087	H2AFX	SLC25A6	[activation]
10088	PRR20C	FAM168A	[activation]
10089	HLA-B	FUS	[activation]
10090	ARRB2	FZD4	[activation]
10091	EGFR	AAMP	[activation]
10092	ACTB	MYOC	[activation]
10093	WNT6	PORCN	[activation]
10094	CDK1	ITGA4	[activation]
10095	STAT6	NCOA1	[activation]
10096	CCDC8	CACNA2D1	[inhibition]
10097	BTRC	CD4	[activation]
10098	AGTRAP	HMGB1	[activation]
10099	FMOD	TGFB3	[activation]
10100	RPA1	COBL	[activation]
10101	INSIG1	ELAVL1	[activation]
10102	GCN1L1	OBSL1	[activation]
10103	SRSF10	EZH2	[activation]
10104	NBN	BARD1	[activation]
10105	HMMR	MAPK3	[activation]
10106	IQCB1	GNAI1	[activation;inhibition]
10107	NOP16	SRPK2	[activation]
10108	HIST3H3	BRD7	[activation]

10109	CSF2	HNRNPA2B1	[activation]
10110	HSP90AB1	PRKCZ	[activation]
10111	MON1A	MAPK6	[activation;inhibition]
10112	TRIM62	SKIL	[activation]
10113	PLCG1	PICALM	[activation]
10114	GRB10	PDGFRB	[activation]
10115	MBP	ABL1	[activation]
10116	MYOD1	SMAD3	[activation]
10117	LOXL1	ELN	[activation]
10118	TRAF6	EIF3I	[activation]
10119	RICTOR	RPTOR	[activation]
10120	CSNK1E	HMMR	[activation]
10121	PVRL4	TIGIT	[activation]
10122	RPL35A	TRAF6	[activation]
10123	IL20RA	MLH1	[activation]
10124	DDAH2	STK3	[activation]
10125	UNC5CL	RELA	[activation]
10126	GOLGA2	LCP2	[activation]
10127	PROP1	CNTN1	[activation]
10128	DARS	ESR1	[activation]
10129	CSNK1E	LRRK2	[activation]
10130	GFAP	CAMK2A	[activation]
10131	MIF	TP53	[activation]
10132	TRAF2	HSPA4	[activation]
10133	SCIMP	LYN	[activation]
10134	CHEK1	USP7	[activation]
10135	GADD45A	MAPK1	[activation;inhibition]
10136	CDH1	MDM2	[activation]
10137	BLOC1S6	CDK5R1	[activation]
10138	TSC2	PPP2CA	[inhibition]
10139	HSPE1	CASP3	[activation]
10140	SMARCA4	TP53	[activation]
10141	PPP2R1A	ITGA4	[activation]
10142	NCOA2	RARA	[activation]
10143	LMTK2	CDK5	[activation]
10144	COL14A1	CD44	[activation]
10145	TP53	STX5	[activation]
10146	BCL2L1	BCL2L10	[activation]
10147	UEVLD	TRAF6	[activation]
10148	STX3	SYT1	[activation]
10149	LCK	CD55	[inhibition]
10150	IRS2	YWHAE	[activation]
10151	BRD7	UBE2I	[activation]
10152	PRKCB	PKN1	[activation]
10153	GRB7	FGB	[activation]
10154	MBP	PRMT5	[activation]
10155	ANAPC11	UBE2U	[activation]
10156	L1CAM	PRNP	[activation]
10157	TRAF2	FDXACB1	[activation]
10158	SOCS3	AR	[inhibition]
10159	ITGB1	SEL1L	[activation]
10160	CDC42	ARRB1	[activation]
10161	HTR2A	GLUL	[activation]
10162	EHMT2	ESR1	[activation]
10163	UBC	BMP6	[activation]
10164	CALM1	ARHGEF7	[activation]
10165	PASK	FGA	[activation]
10166	HSP90AB1	SKP2	[activation]
10167	PPP1R1B	ROBO2	[activation]
10168	EIF4A1	GABARAPL1	[activation]
10169	EIF4A1	PPP2R1A	[activation]
10170	ACTB	EP300	[activation]
10171	PTPRJ	LYPD3	[activation]
10172	TNIP2	SRPK2	[activation]
10173	MAP3K8	HSPA1L	[inhibition]
10174	LUC7L2	SRSF6	[activation]
10175	SP1	C3orf17	[activation]
10176	PLEKHB2	MAP3K1	[activation]
10177	UBE2I	STRA13	[activation]
10178	GPR135	TPD52L1	[activation]
10179	RPS6KA6	SPTBN4	[activation]
10180	SIRT3	DNM1L	[activation]
10181	PLCB3	PRKG1	[inhibition]
10182	ATF2	HIST2H2BE	[activation]
10183	PMS1	REV3L	[activation]
10184	COPB1	RHOQ	[activation]

10185	EIF2B1	STK11	[activation]
10186	PTK2	PXN	[activation]
10187	YTHDF1	CDKN1A	[activation;inhibition]
10188	CCDC172	FAM13C	[activation]
10189	PCBP3	ARF6	[activation]
10190	HSPA4	TSSK6	[activation]
10191	INCENP	AURKB	[activation]
10192	PPP2R5A	BEST1	[activation]
10193	LAMB1	ANTXR2	[activation]
10194	CCNF	NUSAP1	[activation]
10195	FLT1	ROBO4	[activation]
10196	GCN1L1	ASB5	[activation]
10197	SRPK1	CHTOP	[activation]
10198	PLG	KNG1	[activation]
10199	TGM3	SLX4	[activation]
10200	PVRL2	MLLT4	[activation]
10201	TBL3	AURKB	[activation]
10202	VCAM1	ARPC3	[activation]
10203	H3F3A	CD81	[activation]
10204	ABL1	EP300	[activation]
10205	LYN	CASP3	[activation]
10206	TP53BP1	SPAG9	[inhibition]
10207	PRKCQ	MAP4K3	[activation]
10208	LRP1	RANBP9	[activation]
10209	CCT8	STK4	[activation]
10210	TNKS	RAPGEF2	[activation]
10211	TLR2	CREBBP	[activation]
10212	CDK1	EIF2AK2	[activation]
10213	EPHA3	RBM18	[activation]
10214	APH1A	PSEN2	[activation]
10215	KAT2B	H2AFX	[activation]
10216	RPS6KB1	EIF3C	[activation]
10217	SRPK1	RSRC1	[activation]
10218	GFAP	SMAD2	[inhibition]
10219	IGF2BP1	ITGA4	[activation]
10220	ERLIN1	FA2H	[activation]
10221	RAD50	BRCA1	[activation]
10222	SETDB1	ECSIT	[activation]
10223	ESR2	NCOA3	[activation]
10224	ARGLU1	SRPK1	[activation]
10225	HSF1	TAF9	[activation]
10226	PTPRC	GHR	[activation]
10227	PLCG2	CBLB	[activation]
10228	HSP90AB1	MUSK	[activation]
10229	LAMB1	ERBB2IP	[inhibition]
10230	CSNK1D	PML	[activation]
10231	PCNA	FANCA	[activation]
10232	BDNF	SORT1	[activation]
10233	RAD51AP2	RAD51	[activation]
10234	HSP90AA1	CDK18	[activation]
10235	GDI1	RAC2	[activation]
10236	ENOX2	ENOX1	[activation]
10237	UBE2I	WNK2	[activation]
10238	QRICH2	SSSCA1	[activation]
10239	RAB5A	RIN3	[activation]
10240	IL6ST	STAT1	[activation]
10241	MAPK8	CDKN1B	[inhibition]
10242	P4HB	ERO1L	[activation]
10243	HSP90AB1	TTC4	[activation]
10244	TRPC3	ORAI1	[activation]
10245	PRKACA	NOS1	[activation]
10246	CSNK2A1	MYO1B	[activation]
10247	FANCG	FANCB	[activation]
10248	A2M	RNF32	[inhibition]
10249	NFKB1	CCAR2	[inhibition]
10250	TSC2	FBXW5	[inhibition]
10251	NIF3L1	RAD54B	[activation]
10252	CCDC155	VAMP1	[activation]
10253	CCDC67	TXLNA	[activation]
10254	KIAA0408	ABI1	[activation]
10255	USP4	ADORA2A	[activation]
10256	RAC1	SOD1	[activation]
10257	PPP2R1A	CCDC43	[inhibition]
10258	DNM2	CDK1	[activation]
10259	EFS	SRC	[activation]
10260	F11	SERPINF2	[activation]

10261	KIAA0319	SH2B1	[activation]
10262	ELAVL1	NRAS	[activation]
10263	PTCH1	SMURF1	[inhibition]
10264	RAB3D	MAST1	[activation]
10265	SH3BP2	DBNL	[activation]
10266	TP53	SMAD2	[activation]
10267	DAB2IP	RIPK1	[activation]
10268	NUCB2	CASP3	[activation]
10269	HIST1H2AG	MME	[activation]
10270	FYN	TCAP	[inhibition]
10271	TNFRSF1B	BMX	[activation]
10272	GTPBP1	RAD21	[activation]
10273	WASF1	PFN1	[activation]
10274	ACTN1	CAMK2A	[activation]
10275	NGFR	H2AFX	[activation]
10276	BRCA1	MYC	[activation]
10277	CEP63	CAMKK2	[activation]
10278	STK4	PAK3	[activation]
10279	CAPNS1	RASA1	[activation]
10280	LUZP1	GRB2	[activation]
10281	CTSB	IRS4	[activation]
10282	BLM	MX1	[activation]
10283	WHSC1	MDM2	[activation]
10284	RPL23	GRB2	[activation]
10285	CDK2	PCED1A	[activation]
10286	DOK2	ABL1	[activation]
10287	RPS6KA3	BARX1	[activation]
10288	MAX	FBXW11	[inhibition]
10289	MST1R	PLCG1	[activation]
10290	ABL1	PRKCD	[activation]
10291	RAN	RANBP9	[activation]
10292	EP300	CCNB1	[activation]
10293	TRH	ARRB2	[activation]
10294	BMI1	SRPK2	[activation]
10295	SH3GL2	LRRK2	[activation]
10296	PPP1R12A	SETD7	[activation]
10297	ATF2	PML	[activation]
10298	CDCA3	CTDSP1	[activation]
10299	SRPK2	ABL1	[activation]
10300	NCAPG2	MYC	[activation]
10301	MRPL24	TP53	[activation]
10302	PPP2R1A	PMS1	[inhibition]
10303	STAG2	RAD21	[activation]
10304	S100B	MAPT	[activation]
10305	SRF	ALDH3A1	[activation]
10306	CCDC8	CDC23	[activation]
10307	MDM2	BUB1B	[activation]
10308	PPP2R1A	PPP2R5E	[activation]
10309	PPP1R12A	LATS1	[inhibition]
10310	ASNS	DDA1	[activation]
10311	NPRL2	CDKN1A	[activation;inhibition]
10312	ATG12	EPB41L5	[inhibition]
10313	IDE	NR3C1	[activation]
10314	FCGR2A	BLK	[activation]
10315	PGF	NRP2	[activation]
10316	KIAA1598	NME7	[activation]
10317	SMARCA4	RUNX1	[activation]
10318	HRAS	ICMT	[activation]
10319	MYC	BCR	[activation]
10320	HSPA8	RIPK2	[inhibition]
10321	GEM	KRT40	[activation]
10322	PRKCD	SPI1	[activation]
10323	MAPKAPK3	STK11	[activation]
10324	MKNK2	LRRK2	[activation]
10325	ELN	EFEMP2	[activation]
10326	PDZD3	CACNA1S	[activation]
10327	SRPK1	ALYREF	[activation]
10328	RUNX1T1	RBPJ	[inhibition]
10329	CALM1	FAS	[activation]
10330	TMOD3	DAPK1	[activation]
10331	HOXD11	MEIS1	[activation]
10332	SORBS2	PTK2B	[activation]
10333	TAF1	HSF1	[activation]
10334	IRF1	ULK2	[activation]
10335	LILRB2	HLA-A	[activation]
10336	GDF2	ACVR2B	[activation]

10337	YWHAE	KIF23	[activation]
10338	SRC	DDX58	[activation]
10339	AIMP1	MAPK14	[activation]
10340	CRYAA	BCL2L1	[activation]
10341	DCLK2	HSP90AB1	[activation]
10342	ATM	PARP1	[activation]
10343	RRM1	ATG5	[activation]
10344	ATRX	LUC7L2	[activation]
10345	RAC3	AR	[activation]
10346	DAZAP2	TCF7L1	[activation]
10347	OAZ1	EGFR	[activation]
10348	CTNNB1	NOS3	[activation]
10349	PAK6	MDM2	[activation]
10350	CARD17	CARD18	[inhibition]
10351	RBBP9	RB1	[activation;inhibition]
10352	BTK	FCER1G	[activation]
10353	ACTG1	IKBKB	[activation]
10354	CFTR	PRKDC	[activation]
10355	TRPV4	YES1	[activation]
10356	PRKCE	GRM5	[activation]
10357	TTF2	MYB	[activation]
10358	ABI3	ACTN3	[activation]
10359	LYN	MET	[activation;inhibition]
10360	IGHA1	LRRK2	[activation]
10361	RAD21	SKIV2L2	[activation]
10362	FAM98B	BCKDK	[activation]
10363	ISL1	SMAD3	[activation]
10364	SAMHD1	LRRK2	[activation]
10365	MAPK1	MTPN	[activation]
10366	SRC	DLGAP3	[activation]
10367	CDK3	KIR3DL1	[activation]
10368	YWHAZ	VASP	[activation]
10369	JAK3	STAM	[activation]
10370	RPA3	HNRNPCL1	[activation]
10371	DNPH1	FTSJ1	[activation]
10372	JUND	AR	[activation]
10373	TTC3	NR3C1	[activation]
10374	CRK	PRX	[activation]
10375	NFYA	JUN	[activation]
10376	APP	CGB	[activation]
10377	CDC27	GRB2	[activation]
10378	HSP90AB1	CSNK1E	[activation]
10379	BIRC3	SPHK1	[activation]
10380	CCDC138	SAV1	[activation]
10381	PAK4	FGFR2	[activation]
10382	GABARAP	GNL3	[activation]
10383	CTCF	AIM2	[activation]
10384	PRPF31	JAKMIP2	[activation]
10385	PARD6B	TCP10	[activation]
10386	TGM1	ALOX12B	[activation]
10387	MCM10	CDK6	[activation]
10388	BCL6B	BCL6	[activation]
10389	PRKD2	PSG3	[activation]
10390	MAPT	PEG10	[activation]
10391	IK	NUP62	[activation]
10392	TRAF2	EGFR	[activation]
10393	RAPGEF3	NUP98	[activation]
10394	PCDHA6	CRK	[activation]
10395	IGHG2	GABARAPL1	[activation]
10396	TNFSF11	ADAM19	[activation]
10397	ACAP2	HSPB1	[activation]
10398	STX7	SYNPO2	[activation]
10399	RIT2	SRPK2	[activation]
10400	CHGB	LRRK2	[activation]
10401	RASSF1	IGFBP5	[inhibition]
10402	MEOX2	RELA	[activation]
10403	IGF1R	SOCS1	[inhibition]
10404	TOLLIP	CAV1	[activation]
10405	GRB2	VPS13A	[activation]
10406	DUSP3	BNIP3L	[inhibition]
10407	ANKRD17	ITGA4	[activation]
10408	HIST3H3	RPS6KA5	[activation]
10409	TNIK	CYCS	[activation]
10410	PPP2R1A	MCC	[inhibition]
10411	NR3C1	MAPK15	[activation;inhibition]
10412	MDM4	CASP3	[activation]

10413 SMAD4 PBK [activation]
10414 PPP1CC SFRP1 [inhibition]
10415 MARCKS TRAF6 [activation]
10416 NUDCD3 KLHL33 [activation]
10417 AR GRAP2 [activation]
10418 CALM1 GLP1R [activation]
10419 RAB21 ITGB1 [activation]
10420 PPIA VCAM1 [activation]
10421 GABARAPL2 HSPH1 [activation]
10422 SRC ANXA1 [activation]
10423 PRKCI YWHAH [activation]
10424 CTNNB1 PTN [activation]
10425 REXO2 TRAF6 [activation]
10426 PRKCA PRKG1 [activation]
10427 NUP62 CTNNB1 [activation]
10428 MAPK1 MAPK3 [activation;inhibition]
10429 TXNRD1 CAV1 [activation]
10430 CDC42EP4 SRPK2 [activation]
10431 PIK3R1 ADAMTS2 [activation]
10432 ADAM19 ABI2 [activation]
10433 YWHAG RSRC1 [activation]
10434 ISL1 PECR [activation]
10435 AXIN1 DAB2 [activation]
10436 ATF2 ZDHHC17 [activation]
10437 SRPK1 FOS [activation]
10438 CDH1 CASP8 [activation]
10439 CFTR RAB5A [activation]
10440 HABP4 PPP1R15A [inhibition]
10441 PRKAB1 GRB2 [activation]
10442 AKR7A2 ITGA4 [activation]
10443 ILK SERPINA4 [activation]
10444 A2M DNAJB1 [inhibition]
10445 BTK MYD88 [activation]
10446 CHUK CDC37 [activation]
10447 PIK3CG IL6ST [activation]
10448 KCTD5 ILK [activation]
10449 NCAPG SMC2 [activation]
10450 PFN1 ASB2 [activation]
10451 KIAA1279 DOK2 [activation]
10452 FILIP1 HSF1 [activation]
10453 CCR3 HCK [activation]
10454 F2 SERPINB8 [inhibition]
10455 JUN NFATC2 [activation]
10456 BCAS3 CCND1 [activation]
10457 PLSCR1 EGFR [activation]
10458 RPAP3 HSP90AB1 [activation]
10459 CYFIP1 GNB1 [inhibition]
10460 SUV39H1 STX11 [activation]
10461 NLK RNF219 [inhibition]
10462 HSPA6 ESR2 [activation]
10463 BMX HDAC1 [activation]
10464 WDR48 DKC1 [activation]
10465 IRF9 IFNA1 [activation]
10466 FUS GRB2 [activation]
10467 MLST8 NXF1 [activation]
10468 RBX1 CCND2 [inhibition]
10469 NOTCH1 RANBP10 [activation]
10470 HSPA1A ST13 [inhibition]
10471 MSR1 LEPROTL1 [activation]
10472 BDNF F11R [activation]
10473 BCL3 GSK3A [activation]
10474 MDM2 EZR [activation]
10475 ARMC8 NOTCH1 [activation]
10476 TMEM62 PTPN6 [activation;inhibition]
10477 PAPPA IGHG1 [activation]
10478 DPH2 IL17A [activation]
10479 TGFBR1 PPP6C [inhibition]
10480 CXCR4 CTSG [activation]
10481 GABARAPL2 STK3 [activation]
10482 PSEN1 CTNND1 [activation]
10483 PLLP MMP14 [activation]
10484 TGFB3 TGFBR1 [activation]
10485 STMN1 PINX1 [activation]
10486 NR2E3 PPARG [inhibition]
10487 TBC1D23 C12orf45 [inhibition]
10488 ESR2 GAL3ST4 [activation]

10489	MYOD1	HDAC1	[activation]
10490	GRB2	RAB1C	[activation]
10491	SMURF1	WFS1	[inhibition]
10492	STX17	SNAP29	[activation]
10493	WNK4	CLDN2	[activation]
10494	FRK	ABI1	[activation]
10495	ARF6	HNRNPM	[activation]
10496	RPA1	ARRB2	[activation]
10497	GDF9	PCDHA4	[activation]
10498	NFE2	SCAND1	[activation]
10499	TDP2	ETS1	[activation]
10500	TP53BP1	BLM	[activation]
10501	CDK2	MCM4	[activation]
10502	FCGRT	ATG16L1	[activation]
10503	LCK	PTPRK	[inhibition]
10504	TP53	ACTA2	[activation]
10505	ERBB2	GRB7	[activation]
10506	CASP9	BIRC6	[inhibition]
10507	ANXA1	DAB2	[activation]
10508	NFASC	CRK	[activation]
10509	ILK	TRAFD1	[activation]
10510	DNPH1	PILRA	[activation]
10511	TNFRSF10B	IGHG1	[activation]
10512	PRKAA1	TOMM34	[inhibition]
10513	ANGPTL4	CDKN2A	[inhibition]
10514	ITGA4	STOM	[activation]
10515	PVRL4	PVRL2	[activation]
10516	MYC	PADI2	[activation]
10517	CTTN	RPA1	[activation]
10518	LCP2	PAG1	[activation]
10519	SMAD3	AXIN2	[inhibition]
10520	SETD7	STAT3	[activation]
10521	MAPK3	STMN1	[activation]
10522	SNCA	CYCS	[activation]
10523	GRB2	GRWD1	[activation]
10524	MCM3	HIST1H4A	[activation]
10525	CDK4	NCOA2	[activation]
10526	RAN	ELAVL1	[activation]
10527	EDRF1	BCL2L1	[activation]
10528	CCL21	LRRK2	[activation]
10529	YWHAZ	RBL1	[activation;inhibition]
10530	RPTOR	RAB1A	[activation]
10531	HLA-B	CAPRIN1	[activation]
10532	EP300	RBBP5	[activation]
10533	SMARCC1	SRC	[activation]
10534	SUMO1	GNAL	[activation]
10535	WDR92	RPAP3	[activation]
10536	GDI2	ATG5	[activation]
10537	PPP2R3A	PPP2CA	[inhibition]
10538	RAE1	NUP62	[activation]
10539	LRRK2	SLC25A4	[activation]
10540	PPP1R14A	PRKCZ	[activation]
10541	RARA	ARNTL	[activation]
10542	GRB2	HIPK3	[activation]
10543	A2M	PRDX2	[inhibition]
10544	HTR2A	GRM2	[activation]
10545	TNF	NEFL	[activation]
10546	CHD4	SMARCA4	[activation]
10547	HNRNPA1	YWHAZ	[activation]
10548	PRMT5	JAK2	[activation]
10549	ANAPC15	ANAPC4	[activation]
10550	DARC	CCL2	[activation]
10551	KCNIP1	KCND2	[activation]
10552	PTPN6	IFNAR1	[activation;inhibition]
10553	SLC12A2	MDM2	[activation]
10554	DNAJC6	CLTC	[inhibition]
10555	TAB1	RBX1	[inhibition]
10556	STAT5A	ESR2	[activation]
10557	CASP8	OTUD7B	[inhibition]
10558	DKK3	APC	[inhibition]
10559	MKNK1	MBP	[activation]
10560	DUSP13	TGFBR1	[inhibition]
10561	SYT13	NRXN1	[activation]
10562	FTSJ3	OBSL1	[activation]
10563	EIF3G	ICAM1	[activation]
10564	DYSF	ANKRD1	[activation]

10565	MAP1LC3B	CTNNB1	[activation]
10566	EYA1	GNAI2	[activation]
10567	SMAD9	DSTN	[activation;inhibition]
10568	ITGB1BP2	PIP5K1A	[activation]
10569	ICAM5	PSEN2	[activation]
10570	REEP3	SFN	[activation]
10571	LAT	ITK	[activation]
10572	CAMKK2	PRKACA	[activation]
10573	AGTRAP	DDX55	[activation]
10574	LYN	LCP2	[activation]
10575	PPP2R1A	STRN3	[inhibition]
10576	HSPB1	GOLGA8A	[activation]
10577	AKT1	TLR2	[activation]
10578	TICAM1	TICAM2	[activation]
10579	PPP2CA	STRN3	[inhibition]
10580	CTSG	CASP7	[activation]
10581	TFEB	MITF	[activation]
10582	VAV2	SMAD3	[activation]
10583	HRAS	STK11	[activation]
10584	PPP1CA	HYDIN	[activation;inhibition]
10585	TP73	NFYB	[activation]
10586	PPP2R1A	RORC	[inhibition]
10587	PPP2R5B	PPP2CA	[activation]
10588	PAX7	CRK	[activation]
10589	HLA-B	HPRT1	[activation]
10590	NXF1	UBAP1	[activation]
10591	CLOCK	SOX2	[activation]
10592	TNFRSF1A	HRG	[activation]
10593	PAXIP1	STRAP	[activation]
10594	RBX1	HSPB1	[inhibition]
10595	SRC	SKAP2	[activation]
10596	ADRB2	CAPNS1	[activation]
10597	SPSB1	RASA1	[activation]
10598	WAS	RAC2	[activation]
10599	SMURF1	MDM2	[activation]
10600	GSTM4	TP53	[activation]
10601	KIAA0087	TP53	[activation]
10602	KRAS	FANCC	[activation]
10603	GLRX	CASP3	[activation]
10604	MYB	SKP2	[activation]
10605	MAPKAPK2	MAP2K6	[activation]
10606	PAXIP1	SPATA18	[activation]
10607	HTT	DOCK11	[activation]
10608	MMP2	TIMP4	[inhibition]
10609	NOS3	NMT1	[activation]
10610	IRS2	SIRT1	[activation]
10611	APC	PPP3R2	[inhibition]
10612	METTL16	APP	[activation]
10613	EHD1	EGFR	[activation]
10614	IGFBP7	INS	[activation]
10615	HSPB1	AGAP4	[activation]
10616	CREB1	SMARCA4	[activation]
10617	PCSK6	CACNA1A	[inhibition]
10618	RAN	EED	[activation]
10619	JAG2	CACNA1A	[activation]
10620	ATXN10	ABCA1	[activation]
10621	PLG	APOH	[activation]
10622	AKAP2	CCDC8	[inhibition]
10623	ANAPC15	CDC16	[activation]
10624	SIPA1L2	YWHAB	[activation]
10625	PRR20E	FAM168A	[activation]
10626	CEBPG	ATF4	[activation]
10627	FGB	CSNK2B	[activation]
10628	SLC9A3R2	SGK1	[activation]
10629	EIF2AK3	HSP90B1	[activation]
10630	INSR	KRT27	[activation;inhibition]
10631	ATXN10	EGFR	[activation]
10632	MTOR	LAMTOR1	[activation]
10633	MTA3	NOTCH2	[activation]
10634	BRCA1	JAK2	[activation]
10635	DAB2	DVL3	[activation]
10636	MET	SH2B2	[activation]
10637	RAB11A	IKBKE	[activation]
10638	NOS3	BDNF	[activation]
10639	MAPK3	VDR	[activation]
10640	TGFBR2	MAP2K1	[activation]

10641	GRB2	PRICKLE3	[activation]
10642	CRK	SUV39H2	[activation]
10643	RRAD	CAMK2G	[activation]
10644	ITSN2	STX4	[activation]
10645	USP9X	RICTOR	[activation]
10646	NUAK1	PRKAA1	[inhibition]
10647	LIMS1	PLCE1	[activation]
10648	PPP2R1A	GNA12	[activation]
10649	NR0B2	TRAF6	[activation]
10650	PPP2R5A	DAPK1	[activation]
10651	ACTB	RCC1	[activation]
10652	PCBP2	MDM2	[activation]
10653	ITGA4	CD81	[activation]
10654	HSPB8	HSPB1	[activation]
10655	EPSTI1	BCAR3	[activation]
10656	MAP3K8	PIN1	[activation]
10657	NR2E3	NR3C1	[activation]
10658	GRB2	ASAP1	[activation]
10659	TRAF2	SPECC1	[activation]
10660	HSP90AA1	KCNS3	[activation]
10661	PPP1CC	CDKN2A	[inhibition]
10662	CDK11A	C18orf25	[activation]
10663	EPHA3	CALM1	[activation]
10664	SRC	WAS	[activation]
10665	LRRK2	RPS8	[activation]
10666	GTF2E1	GTF2A2	[activation]
10667	ATF4	GABBR1	[activation]
10668	DDX5	WHSC1	[activation]
10669	STX3	STXBP2	[activation]
10670	SAV1	PIBF1	[activation]
10671	CPVL	CDKN1A	[activation;inhibition]
10672	SRPK1	KIAA1841	[activation]
10673	CYCS	TRAF6	[activation]
10674	PLCG1	SUV39H2	[activation]
10675	CAMKK2	IQGAP2	[activation]
10676	PIK3R1	GAB2	[activation]
10677	TP53	IKBKB	[activation]
10678	VDAC3	DDX24	[activation]
10679	DNM1L	GSK3B	[activation]
10680	RPA1	KIAA1033	[activation]
10681	CDH1	ITGB7	[activation]
10682	ANAPC4	RAD21	[activation]
10683	RFX6	LMO3	[activation]
10684	RPL11	STAT3	[activation]
10685	BCAR1	PPP1R15A	[inhibition]
10686	EIF4A1	RAD21	[activation]
10687	FAM90A1	GNPTAB	[activation]
10688	ZFP91	FOS	[activation]
10689	TAF1B	NAPB	[activation]
10690	CBL	CDC42	[activation]
10691	OBSL1	KCNE4	[activation]
10692	CASP9	CAPN2	[activation]
10693	KAT2A	CTNNB1	[activation]
10694	BIRC2	RIPK4	[activation]
10695	PPP1CC	PHACTR4	[inhibition]
10696	MLLT4	EPHA7	[activation]
10697	RIPK1	CDC37	[activation]
10698	PRKG1	RGS2	[activation;inhibition]
10699	IRF5	GRB2	[activation]
10700	YWHAZ	CDC25B	[activation]
10701	HDAC2	GATA4	[activation]
10702	PRLR	STAT5B	[activation]
10703	RSPO1	LGR4	[activation]
10704	MYC	CLSTN1	[activation]
10705	HOXD8	HMGB1	[inhibition]
10706	ITGA4	HNRNPAB	[activation]
10707	ESR1	GNL3	[activation]
10708	TRAF5	EIF2AK2	[activation]
10709	PRKDC	TNFRSF1B	[activation]
10710	CYP11B1	CYP11B2	[activation]
10711	PPP2R1A	RBM7	[inhibition]
10712	APP	IFIH1	[activation]
10713	RPS6	VCAM1	[activation]
10714	BUB1	RASA1	[activation]
10715	MTNR1A	GNB1	[activation]
10716	CAMK2A	CHMP5	[activation]

10717 IKBKE NFKBIA [inhibition]
10718 PPARA NCOR1 [inhibition]
10719 CAMK2D GRB2 [activation]
10720 NRAS ARHGAP4 [activation]
10721 CRTAM CADM1 [activation]
10722 HSPB1 SMARCA2 [activation]
10723 RCC2 TRAF6 [activation]
10724 MAPK3 HDAC4 [activation]
10725 SGK1 MYH1 [activation]
10726 F2RL1 CAPN1 [activation]
10727 TGFBR1 LIMS1 [activation]
10728 PTPN13 RAPGEF2 [activation]
10729 PYCARD AZI1 [inhibition]
10730 ITGA4 PDCD10 [activation]
10731 CEBPB RPS6KA1 [activation]
10732 PRKCE ITGB2 [activation]
10733 AP2S1 POLQ [activation]
10734 STK3 FBXW11 [activation]
10735 PPP1CA ULK1 [inhibition]
10736 NUMA1 CDK6 [inhibition]
10737 APP MAD2L1 [inhibition]
10738 AXIN1 JUP [activation]
10739 WWOX SRPK1 [activation]
10740 HSPH1 YAP1 [activation]
10741 EIF3A EIF4A2 [activation]
10742 ITGA4 XPO1 [activation]
10743 LNX2 PDZRN3 [inhibition]
10744 ADRBK1 PDC [activation]
10745 HIST3H3 CHUK [activation]
10746 PPP2CA DAPK1 [inhibition]
10747 MPP6 ARHGAP18 [activation]
10748 HIST1H4A JMJD4 [activation;inhibition]
10749 TGM3 PRKAB1 [activation]
10750 YWHAG IGF1R [activation]
10751 OBSL1 PCNA [inhibition]
10752 IGF1R JAK2 [activation]
10753 PRKAA1 SNRK [inhibition]
10754 CDC16 NXF1 [activation]
10755 MARVELD2 MCM7 [activation]
10756 SNRPD1 ICAM1 [activation]
10757 HIST1H2BM ICAM1 [activation]
10758 NCOA1 RORB [activation]
10759 CDK5 PCNA [activation]
10760 DVL2 SMURF1 [inhibition]
10761 SORBS1 PXN [activation]
10762 APMAP ATF2 [activation]
10763 SMAD2 RASA1 [activation]
10764 HSP90AA1 MARS [activation]
10765 CAPN1 ARRB2 [activation]
10766 UBE2E1 RELA [activation]
10767 RIPK1 TLR3 [activation]
10768 NLGN3 PRDX2 [activation]
10769 SH2D2A LCK [activation]
10770 STAT3 NR3C1 [activation]
10771 GNB4 CDK18 [activation]
10772 TRAF6 CARD11 [activation]
10773 GAPDH ANXA1 [activation]
10774 BID RPA1 [activation]
10775 GNAT2 SUMO1 [activation]
10776 COL4A5 DCN [activation]
10777 MAPKBP1 MAPK10 [activation]
10778 COG8 GEM [activation]
10779 UNC119 NPHP3 [inhibition]
10780 SP1 IFI16 [activation]
10781 LCK KIR2DL3 [activation]
10782 HSPA4 MAPT [activation]
10783 IQGAP1 WHSC1 [activation]
10784 MCM2 CCND1 [activation]
10785 DAB2 DAB2IP [activation]
10786 GFAP AURKB [activation]
10787 SRPK2 SNIP1 [activation]
10788 NCKAP1 GRB2 [activation]
10789 MTOR MLST8 [activation]
10790 GABARAP TRAP1 [activation]
10791 CAV1 MAPK3 [activation]
10792 NRF1 CEBPB [activation]

10793	CTNNB1	PRKCA	[activation]
10794	BTLA	TNFRSF14	[activation]
10795	EGFR	YWHAZ	[activation]
10796	CDKN1A	PHKG2	[activation;inhibition]
10797	HLA-C	PCBP1	[activation]
10798	CCDC88C	MAPK8	[activation]
10799	TPT1	PTEN	[activation]
10800	RAB2A	FAM114A1	[activation]
10801	HNRNPA1	ITGA4	[activation]
10802	DYSF	SAMHD1	[activation]
10803	RRP15	SRPK3	[activation]
10804	ANAPC2	EPHA4	[activation]
10805	CD93	KNG1	[activation]
10806	STK24	STK11	[activation]
10807	CTNNB1	HNRNPA1	[activation]
10808	PTEN	GPR113	[activation]
10809	MAPK14	SPAG9	[inhibition]
10810	MON1A	TAB1	[inhibition]
10811	MCC	EIF2B1	[activation]
10812	ERRFI1	FGFR2	[activation]
10813	NOS1	ATP2B4	[activation]
10814	CRX	NPAS2	[activation]
10815	ENO1	MDM2	[activation]
10816	STAP2	MYD88	[activation]
10817	YES1	TP53BP2	[activation]
10818	RRP1	OBSL1	[activation;inhibition]
10819	CDK6	PPARGC1A	[activation]
10820	NR3C1	NR2F2	[activation]
10821	EGFR	DIMT1	[activation]
10822	LIPE	MAPK1	[activation]
10823	PTBP1	HMGA2	[activation]
10824	EPB41L1	DRD2	[activation]
10825	PRTN3	F2R	[activation]
10826	SDCBP	EFNB1	[activation]
10827	MYC	NUP98	[activation]
10828	GRB2	ANXA2	[activation]
10829	TFAP2A	COL1A2	[activation]
10830	MDM2	XIAP	[activation]
10831	MYH11	MAPKAPK5	[activation]
10832	PPP1R3D	EPM2A	[inhibition]
10833	CDK11B	RARA	[activation]
10834	RRAS2	ARHGEF2	[activation]
10835	AVP	AVPR1A	[activation]
10836	TXN	TP53	[activation]
10837	HSPA4	RPTOR	[activation]
10838	MIF	NME1	[activation]
10839	PTH	PTH1R	[activation]
10840	CAV1	RAB27B	[activation]
10841	CARM1	NCOA1	[activation]
10842	SRSF12	EIF4A3	[activation]
10843	FKBPL	TSSC1	[activation]
10844	PIK3R1	WBP11	[activation]
10845	GPR37	SLC6A3	[activation]
10846	NCAM1	RHEB	[activation]
10847	BET1	GOSR1	[activation]
10848	AKAP4	AKAP3	[inhibition]
10849	SOD1	PPP3CA	[inhibition]
10850	TNIK	GSN	[activation]
10851	HSP90B1	MDM2	[activation]
10852	PPP1R16B	TGFBR2	[activation]
10853	SLX4	PLK1	[activation]
10854	CCNA2	HERC5	[inhibition]
10855	JUND	MAPK1	[activation]
10856	ESR2	RPS6	[activation]
10857	PAK1	SMAD2	[activation]
10858	HCST	PIK3R1	[activation]
10859	BUB1	FANCC	[activation]
10860	TRIM28	STAT1	[activation]
10861	GNAZ	OPRD1	[activation]
10862	TNFSF11	TRMT2A	[activation]
10863	MDM2	JUN	[activation]
10864	YPEL3	SRPK1	[activation]
10865	CASP3	RBX1	[activation]
10866	GNS	SRC	[activation]
10867	LZTR1	TBX2	[activation]
10868	EIF2S3	DCC	[activation]

10869	STAT2	IWS1	[activation]
10870	CAMK2B	RPL11	[activation]
10871	PTEN	CCDC180	[activation;inhibition]
10872	FMR1	PRKAA1	[inhibition]
10873	ACTA1	CBL	[activation]
10874	MAPK13	MAP3K4	[activation]
10875	SMURF1	ACAP2	[inhibition]
10876	DDX1	ATM	[activation]
10877	THBS1	COL1A1	[activation]
10878	CFL1	EGFR	[activation]
10879	PSENEN	GSAP	[activation]
10880	VCAM1	TLN1	[activation]
10881	TAF1	CCNA2	[activation]
10882	RRAGD	RRAGB	[activation]
10883	LUC7L2	EXOC1	[activation]
10884	PAK3	SYN1	[activation]
10885	PRKAA2	ULK2	[inhibition]
10886	IGHG1	FGA	[activation]
10887	NUP62	SSC5D	[activation]
10888	URM1	OGT	[activation]
10889	LRRK2	LIMS2	[activation]
10890	FTSJ1	SGTA	[activation]
10891	IRF7	TRMT2A	[activation]
10892	GTF2B	HSF1	[activation]
10893	PML	CHEK2	[activation]
10894	CDK3	HSP90AA1	[activation]
10895	HIST1H3A	PRKDC	[activation]
10896	NAA15	UBA5	[activation]
10897	HNRNPA1	GRB2	[activation]
10898	AP4E1	ARF1	[activation]
10899	BRIP1	PALB2	[activation]
10900	COL1A2	DCN	[activation]
10901	GNG8	GNB1	[activation]
10902	CDK5	HDAC4	[activation]
10903	CALM1	CCNE1	[activation]
10904	DDX5	TP53	[activation]
10905	TICAM1	AZI2	[activation]
10906	SRPK1	AHCYL1	[activation]
10907	SP1	EGR1	[activation]
10908	KCTD5	RAPGEF2	[activation]
10909	TEC	PTPRJ	[activation]
10910	VDAC3	CD4	[inhibition]
10911	RHEB	ECSIT	[activation]
10912	ATM	TTI2	[activation]
10913	FOXE1	PLCB3	[activation]
10914	ADAM17	PTPN3	[activation]
10915	ANXA7	CPNE6	[activation]
10916	MDM2	HNRNPA1	[activation]
10917	FANCA	DDX39B	[activation]
10918	MAD2L2	CDC20	[inhibition]
10919	PRKCA	HABP4	[activation]
10920	ITK	FGFR1	[activation]
10921	CORO2A	RB1	[activation;inhibition]
10922	IRS2	RPTOR	[activation]
10923	DNAJA3	CDC42	[inhibition]
10924	CHD4	ITGA4	[activation]
10925	KRR1	SRPK1	[activation]
10926	OBSL1	ACSL3	[activation;inhibition]
10927	CHEK2	TSSK1B	[activation]
10928	LRP1	APOA5	[activation]
10929	RASGRF1	NTRK1	[activation]
10930	PTPN1	ITGB1	[activation]
10931	ACACA	RIOK2	[activation;inhibition]
10932	YAP1	FASN	[activation]
10933	IL33	IL1RL1	[activation]
10934	SUMO3	CDK11A	[activation]
10935	PSEN2	PSENEN	[activation]
10936	SMS	MAPKAPK3	[activation]
10937	TACC1	AURKB	[activation]
10938	SOX2	CDK11A	[activation]
10939	CDK1	ECT2	[activation]
10940	RPL18A	LPL	[activation]
10941	SERTAD4	PPP2R1A	[inhibition]
10942	ERBB2	FGR	[activation]
10943	ADAMTSL3	NOTCH2NL	[activation]
10944	BMPR1B	SASH3	[activation]

10945	IQGAP1	SHC1	[activation]
10946	RAD21	NUMA1	[activation]
10947	BMP4	FSTL1	[activation]
10948	HLTF	NOTCH1	[activation]
10949	JAK2	STAM	[activation]
10950	TNF	ATP2A2	[activation]
10951	CD28	IL12A	[activation]
10952	KLK6	PALB2	[activation]
10953	LYN	PTPRC	[activation]
10954	PPP2CA	VDR	[activation]
10955	FHL1	RBPJ	[inhibition]
10956	MAPK3	PTPRC	[activation]
10957	CFTR	HSPA2	[activation]
10958	RPA3	NEURL4	[activation]
10959	MYB	CEBPE	[activation]
10960	FAM65B	MAP2K2	[activation]
10961	ABI2	HGS	[activation]
10962	CDK4	DDAH2	[activation]
10963	TAGLN2	TRAF6	[activation]
10964	ELAVL1	SRPK1	[activation]
10965	CSK	EGFR	[activation]
10966	SYK	STAT1	[activation]
10967	CAPNS1	PPARGC1A	[activation]
10968	PRKDC	PCNA	[activation]
10969	RPP14	IGSF21	[activation]
10970	SFN	ATF2	[activation]
10971	IKBKB	TUBB	[activation]
10972	CSNK2A2	ACACA	[activation;inhibition]
10973	DDX21	VCAM1	[activation]
10974	NXF1	ABR	[activation]
10975	TFAP2A	APC	[activation]
10976	PLG	IGF2	[activation]
10977	FLT1	HSPG2	[activation]
10978	GAB2	MAPK4	[activation]
10979	ARHGAP24	ROCK1	[activation]
10980	CBY1	CTNNB1	[activation]
10981	HIST1H3A	ICAM1	[activation]
10982	XDH	BTN1A1	[activation]
10983	DDX5	CREBBP	[activation]
10984	WWTR1	PAX3	[activation]
10985	BTRC	MAPK14	[activation]
10986	SMURF1	PAK1	[activation]
10987	FAM175B	TP53	[activation]
10988	SLAMF6	ZAP70	[activation]
10989	TGFBR1	DAB2	[activation]
10990	LRRK2	CYFIP2	[activation]
10991	CDK5	C2CD5	[activation]
10992	NPAS4	ARNT2	[activation]
10993	FGFR1OP2	STK24	[activation]
10994	PAXIP1	CDC20	[activation]
10995	GDI2	SGK1	[activation]
10996	OGT	CSNK2A1	[activation]
10997	LLGL2	PARD6B	[activation]
10998	RBM48	TRIB3	[inhibition]
10999	SH2D1B	DAPP1	[activation]
11000	HBA1	SPP1	[activation]
11001	BUB3	SMAD2	[inhibition]
11002	RAB13	RPA2	[activation]
11003	SMC2	EGFR	[activation]
11004	EGFR	PKIA	[activation]
11005	PIP5KL1	PIP5K1C	[activation]
11006	UBE2R2	GABARAPL2	[activation]
11007	TBK1	SYK	[activation]
11008	TNIK	CTNNB1	[activation]
11009	TBP	GTF2A2	[activation]
11010	GRB2	COX6A1	[activation]
11011	MAPK13	YWHAB	[activation]
11012	TEKT1	CCHCR1	[activation]
11013	C14orf1	BID	[activation]
11014	F2	SPP1	[activation]
11015	TNNT1	FYN	[activation]
11016	EGFR	SLA2	[activation]
11017	GPSM1	ZBED1	[inhibition]
11018	MN1	EP300	[activation]
11019	CREBBP	HOXA9	[activation]
11020	CDKN2B	PYCRL	[activation;inhibition]

11021	SETDB1	LUC7L2	[activation]
11022	TGFB1	AXIN2	[activation]
11023	RAC3	CREBBP	[activation]
11024	NOTCH1	CBL	[activation]
11025	PDPK1	ILK	[activation]
11026	SULT1A1	STK4	[activation]
11027	TRIM25	STK11	[activation]
11028	EIF2AK3	IKBKB	[activation]
11029	CDC37	LRRK2	[activation]
11030	ITGA4	PRKDC	[activation]
11031	DRD3	EPB41L1	[activation]
11032	MYC	SIRT5	[activation]
11033	LEFTY2	ACSS3	[activation]
11034	CTNNB1	FOXO3	[activation;inhibition]
11035	CDK4	RELA	[activation]
11036	ABL2	ERBB2	[activation]
11037	ATF1	GABPB1	[activation]
11038	DEFB1	CCR6	[activation]
11039	CFLAR	GSN	[activation]
11040	CASR	PRKCA	[activation]
11041	SDF2L1	EGFR	[activation]
11042	RAB5B	LRRK2	[activation]
11043	CRY1	TRAF2	[activation]
11044	PIK3CD	IL3	[activation]
11045	APBB1	MAPK8	[activation]
11046	ARHGAP24	APP	[activation]
11047	RBL2	UBTF	[activation]
11048	XRCC2	RAD51C	[activation]
11049	BAX	SLC25A4	[inhibition]
11050	CTNNBIP1	CTNNA2	[inhibition]
11051	F8	EIF1B	[activation]
11052	ITGB1	CCDC8	[activation]
11053	MAP2K4	CYLC2	[activation]
11054	MYO1C	CBL	[activation]
11055	PPP6C	PPP4C	[inhibition]
11056	TGM1	HSPB1	[activation]
11057	GLMN	EIF3A	[activation]
11058	BAD	HRK	[inhibition]
11059	CALM1	YWHAE	[activation]
11060	SRPK2	DIDO1	[activation]
11061	TERT	YWHAQ	[activation]
11062	IRAK1	CAMKK2	[activation;inhibition]
11063	PTK2	DDX39A	[activation]
11064	HSPA1A	YWHAB	[inhibition]
11065	OPRD1	ADRB2	[activation]
11066	SSSCA1	CCNH	[activation]
11067	HSP90AB1	STK38L	[activation]
11068	IL7R	STAT5B	[activation]
11069	RPS19	LRRK2	[activation]
11070	PAK1	CHORDC1	[activation]
11071	ITCH	FYN	[activation]
11072	CDC5L	HNRNPA1	[activation]
11073	KCNJ2	KCNJ4	[activation]
11074	CAMK2A	ITGB1BP1	[activation]
11075	SIMC1	YWHAG	[activation]
11076	EIF2S3	CASP4	[activation]
11077	WIPF1	GRB2	[activation]
11078	CASP8	PRSS50	[inhibition]
11079	CDC37L1	XPO1	[activation]
11080	ZFYVE9	EIF4G1	[activation]
11081	MEF2A	GNG12	[activation]
11082	MESDC1	LRP6	[activation]
11083	OSM	COL4A1	[activation]
11084	FAM46A	ZFYVE9	[activation]
11085	EIF4E2	EPAS1	[inhibition]
11086	GAB2	ZAP70	[activation]
11087	ANAPC1	TP53BP1	[activation]
11088	TP53	C10orf90	[activation]
11089	EGFR	PIM1	[activation]
11090	CD84	SH2D1A	[activation]
11091	SDC4	DVL1	[activation]
11092	PTPRU	GNAI2	[activation;inhibition]
11093	TP73	HRAS	[activation]
11094	MTOR	RPTOR	[activation;inhibition]
11095	CDK17	PPP2R2A	[activation]
11096	LCK	FAS	[inhibition]

11097	ADAP1	PRKCZ	[activation]
11098	PDIA2	CRK	[activation]
11099	SH3KBP1	BCAR1	[activation]
11100	CD3E	PIK3R2	[activation]
11101	JAK1	HSP90AB1	[activation]
11102	CTTN	ABL2	[activation]
11103	MEOX2	ARHGEF7	[activation]
11104	NUP155	TRAF6	[activation]
11105	NXF1	FLAD1	[activation]
11106	IRF3	SMAD2	[inhibition]
11107	MAP2K1	PPARG	[activation]
11108	APBB1	U2AF2	[activation]
11109	USP7	BUB3	[inhibition]
11110	YWHAB	CDK18	[activation]
11111	INS	APP	[activation]
11112	KLC4	YWHAB	[activation]
11113	PRKCB	DDX58	[activation]
11114	GTF2I	BCL2L11	[activation]
11115	CREB3	RIMS3	[activation]
11116	FKBPL	SETDB1	[activation]
11117	RPTOR	TRAF6	[activation]
11118	NFKBIB	RXRA	[inhibition]
11119	ARHGAP15	PRNP	[activation]
11120	CALM1	CCND2	[activation]
11121	NFKBIA	ACTG1	[activation]
11122	EVX1	RAD21	[activation]
11123	GNB1	PLEKHG2	[activation]
11124	GAST	MEP1B	[activation]
11125	HOXD9	HMGB1	[activation]
11126	KIAA1109	CTNNB1	[activation]
11127	MMP14	FGFR4	[activation]
11128	MAP2K7	PIK3R1	[activation]
11129	CNOT10	LYN	[activation]
11130	PPP1R1A	PPP1R15A	[inhibition]
11131	LUC7L3	SRPK1	[activation]
11132	IKBKG	ANXA1	[activation]
11133	ADRB2	IL6R	[activation]
11134	SRPK1	SRRM2	[activation]
11135	ARHGEF2	YWHAH	[activation]
11136	ATR	NCOA2	[activation]
11137	SERPINA5	F11	[inhibition]
11138	EPHA8	ANKS1B	[activation]
11139	SOX2	SMC2	[activation]
11140	SIRT3	HSPA4	[activation]
11141	DDX39B	THOC3	[activation]
11142	CTF1	MAGEA6	[activation]
11143	CORO1C	BTK	[activation]
11144	RPA1	RAB1A	[activation]
11145	MYC	POLE	[activation]
11146	SRPK2	EPB41L1	[activation]
11147	PTN	PLXNB2	[activation]
11148	EHD1	IGF1R	[activation]
11149	ADRBK1	ACACA	[activation;inhibition]
11150	EIF4EBP1	KLHL25	[inhibition]
11151	EIF2AK2	FARSB	[activation]
11152	CCND1	SKP1	[inhibition]
11153	STK11	SNRK	[activation]
11154	ERCC6	CHEK2	[activation]
11155	HSP90AA1	RPS6KA5	[activation]
11156	EGLN3	ABI2	[activation]
11157	IKBKB	CCAR2	[inhibition]
11158	MANF	MDM2	[activation]
11159	UBE2E3	RANGAP1	[activation]
11160	SERBP1	CTNNB1	[activation]
11161	PTEN	PPP1CA	[activation;inhibition]
11162	RAB23	ARFGAP1	[activation]
11163	SMAD3	CTNNB1	[activation]
11164	MOS	NUDCD3	[activation]
11165	MCM7	CDKN1C	[inhibition]
11166	PIK3CA	DDX5	[activation]
11167	GLRX3	ITGA4	[activation]
11168	TERT	DKC1	[activation]
11169	ARHGEF11	CRK	[activation]
11170	PIAS4	SERPINA5	[inhibition]
11171	TRIM65	SRPK1	[activation]
11172	RALB	ULK1	[inhibition]

11173	CD48	IL18RAP	[activation]
11174	CTCF	POLR2A	[activation]
11175	BMX	FADD	[activation]
11176	IQGAP1	CALM1	[activation]
11177	EIF2S2	CRMP1	[activation]
11178	CYLD	CAMK2D	[inhibition]
11179	RPL37A	TP53	[activation]
11180	DIRAS3	PTPN1	[activation]
11181	VCAM1	HIP1	[activation]
11182	EP300	DDX5	[activation]
11183	RAD51	RAD54L	[activation]
11184	CFL1	APP	[activation]
11185	LRRK2	SFXN1	[activation]
11186	PGAM5	EIF4A3	[activation]
11187	PFN2	FLT3LG	[activation]
11188	GPN1	RPAP3	[activation]
11189	UBE2V1	UBE2N	[activation]
11190	ILK	RAF1	[activation]
11191	NOTCH2NL	SNAI1	[activation]
11192	DOCK8	RHOJ	[activation]
11193	PIK3R1	LCP2	[activation]
11194	SP1	STAT3	[activation]
11195	MYC	POLD1	[activation]
11196	MAPT	SGK1	[activation]
11197	HECTD1	APC	[inhibition]
11198	STAMBP	RECQL5	[activation]
11199	FYN	CD2AP	[activation]
11200	HSPB1	CT55	[activation]
11201	FASLG	PIK3CA	[activation]
11202	YWHAZ	SSFA2	[activation]
11203	PHLDB2	MYC	[activation]
11204	TP53	ZCCHC10	[activation]
11205	DKK3	PPM1D	[inhibition]
11206	APP	CDKN1A	[activation]
11207	EIF4A1	CDC5L	[activation]
11208	ERBB2	KDELR2	[activation]
11209	TBP	NFYB	[activation]
11210	CDK1	HIST1H2BB	[activation]
11211	CCR5	PTK2	[activation]
11212	MOK	INSR	[activation]
11213	TXLNA	SPERT	[activation]
11214	TNIK	NUDC	[activation]
11215	ERBB2	STAT3	[activation]
11216	MLLT4	CCDC8	[activation]
11217	AAR2	GJA5	[activation]
11218	RNF114	TRAIP	[activation]
11219	SERPINA3	CTSG	[inhibition]
11220	SMAD4	AKT1	[activation]
11221	SGTA	IL8	[activation]
11222	TOP1	ATM	[activation]
11223	MYO3B	GRB2	[activation]
11224	ACTB	PCK1	[activation]
11225	LTA	BIRC2	[activation]
11226	SRPK3	PDCD11	[activation]
11227	TBXA2R	YWHAZ	[inhibition]
11228	MLH1	TRIM23	[activation]
11229	HSP90AA1	STK32B	[activation]
11230	ACTN1	MDM2	[activation]
11231	RAD21	CCDC170	[activation]
11232	CDKN2C	ATM	[activation]
11233	CASP9	HIP1	[activation]
11234	BACE1	RANBP9	[activation]
11235	EIF2B2	EGFR	[activation]
11236	CASP7	SAT1	[activation]
11237	FBN3	BID	[activation]
11238	STOM	DVL3	[activation]
11239	MSANTD3	SUV39H1	[activation]
11240	GATA1	CREBBP	[activation]
11241	HSF1	CSNK2A1	[activation]
11242	MYL3	FTH1	[inhibition]
11243	LRRK2	CDC25A	[activation]
11244	MAPK14	CCDC8	[activation]
11245	NUDC	ERBB2	[activation]
11246	MBL2	MAD2L1	[activation]
11247	AR	RB1	[activation]
11248	ITGB1	ANKS1B	[activation]

11249	RIC8A	GNAI3	[activation;inhibition]
11250	EGFR	RAPGEF1	[activation]
11251	LMO2	ISL1	[activation]
11252	RACGAP1	VAV1	[activation]
11253	PRR20C	DAB1	[activation]
11254	LXN	KIAA1009	[activation]
11255	TNFSF10	ZDHHC17	[activation]
11256	BTK	TLR8	[activation]
11257	HSPE1	ATF2	[activation]
11258	PRKCB	IFNAR2	[activation]
11259	BCL2	PPP3CA	[activation]
11260	CAMKK2	ATG4B	[activation]
11261	EGFR	MAP2K3	[activation]
11262	MAP3K3	PRKDC	[activation]
11263	PAXIP1	AAMP	[activation]
11264	F2RL2	F11R	[activation]
11265	FGFR2	GLCE	[activation]
11266	LRRK2	HMMR	[activation]
11267	SOCS1	FLT3	[inhibition]
11268	RPAP3	TGFBR1	[inhibition]
11269	PRKCA	PFKFB1	[activation]
11270	JAK2	PTK2B	[activation]
11271	RIC8B	LRP1	[activation]
11272	HIST1H3A	AIRE	[activation]
11273	PLK1	EGFR	[activation]
11274	ICT1	FASTKD2	[activation]
11275	VDAC1	BAX	[inhibition]
11276	FOXO1	CDKN1A	[activation]
11277	SIRT1	MAP2K4	[activation]
11278	PTP4A3	STAT1	[activation]
11279	ESR2	CNN1	[activation]
11280	PEBP4	RAF1	[activation]
11281	SCXA	SOX9	[activation]
11282	INHBA	INHA	[activation]
11283	SKP1	FBXL3	[inhibition]
11284	MAZ	MAPK14	[activation]
11285	TNFSF10	TNFAIP3	[activation]
11286	DYRK2	RAD54B	[activation]
11287	CDK5R2	ACTN1	[activation]
11288	HSP90AA1	PDPK1	[activation]
11289	BARD1	SRSF1	[activation]
11290	PAK1	GRB2	[activation]
11291	LRWD1	HIST3H3	[activation]
11292	JUN	SPI1	[inhibition]
11293	FBXO6	DNAJA1	[inhibition]
11294	EP300	SP3	[activation]
11295	FICD	RAC1	[activation]
11296	ANXA5	UBC	[activation]
11297	ITGAM	FGG	[activation]
11298	RPS6KA1	SYK	[activation]
11299	SMAD2	CHGB	[activation]
11300	TSC2	PIN1	[inhibition]
11301	NGB	CCDC36	[inhibition]
11302	MMP7	DCN	[activation]
11303	SMAD2	GLI3	[activation]
11304	CALM1	NRGN	[activation]
11305	GABARAPL2	PFN2	[activation]
11306	HSPA5	EGFR	[activation]
11307	FHL3	CSF1	[activation]
11308	LZTS2	DYRK1A	[activation]
11309	SOCS3	RELA	[inhibition]
11310	RPA1	POLR2A	[activation]
11311	DCN	COL4A1	[activation]
11312	TERT	CIB1	[activation]
11313	CIT	RAC1	[activation]
11314	F10	PROS1	[inhibition]
11315	BMP8B	ABL1	[activation]
11316	MAGED1	PLK1	[activation]
11317	BTK	AR	[activation]
11318	CD81	EIF4A1	[activation]
11319	DDX21	WHSC1	[activation]
11320	VKORC1	ILK	[activation]
11321	CDC5L	SMARCB1	[activation]
11322	DOK4	ERBB2	[activation]
11323	SMAD5	U2AF2	[activation]
11324	CDC23	MDC1	[activation]

11325	NUPL2	GLE1	[activation]
11326	HIF1A	MAFK	[activation]
11327	HSD11B2	CTBP1	[activation]
11328	VCAM1	SAR1B	[activation]
11329	MET	FES	[activation]
11330	PTGES3	IRS4	[activation]
11331	MYOD1	ID4	[activation]
11332	HSPG2	ESR2	[activation]
11333	HES5	JAK2	[activation]
11334	CRK	FCGR2C	[activation]
11335	HSF1	PTGES3	[activation]
11336	FBXO17	SKP1	[inhibition]
11337	RPS6	HSP90AB1	[activation]
11338	HSPB1	PALM3	[activation]
11339	ILK	SLC4A1AP	[activation]
11340	CD8A	LAT	[activation]
11341	MEF2A	UBE2I	[activation]
11342	VCL	TGFB1I1	[activation]
11343	IL3RA	PPP1CC	[activation]
11344	GADD45B	GADD45GIP1	[activation]
11345	PPP1R3D	FYN	[activation;inhibition]
11346	GPRASP1	GRM5	[activation]
11347	FZR1	TTK	[activation]
11348	TTN	MYC	[activation]
11349	HOXC8	GRB2	[activation]
11350	MAPK8	MAP1B	[activation]
11351	EIF4A3	SRPK2	[activation]
11352	SNCA	HTT	[activation]
11353	JAK2	ALK	[activation]
11354	ICAM1	RPL5	[activation]
11355	CLSPN	ATR	[activation]
11356	UBXN11	PRKAA1	[inhibition]
11357	YES1	HSP90AB1	[activation]
11358	PRKAA2	STIM1	[activation]
11359	MAPK8	EP300	[activation]
11360	HIF1A	NCOA2	[activation]
11361	PORCN	WNT1	[activation;inhibition]
11362	JUP	MAP1LC3B	[activation]
11363	PRKCB	LSP1	[activation]
11364	HMGN3	GRB2	[activation]
11365	LRRK2	RHOA	[activation]
11366	APOL1	CDC23	[activation]
11367	CAV1	LATS1	[activation]
11368	TMED10	TRAF6	[activation]
11369	NOXA1	RIMBP3	[activation]
11370	SDC3	FGF2	[activation]
11371	SDC2	SDC3	[activation]
11372	ADRA1D	NOS1	[activation]
11373	RADIL	GRB2	[activation]
11374	TAGLN	IGF1R	[activation]
11375	DAPK1	TWF1	[activation]
11376	RHOB	PGGT1B	[activation]
11377	CDK6	PML	[inhibition]
11378	ADRB2	GRB2	[activation]
11379	DOK1	PTPN11	[activation]
11380	PRKACB	PITX2	[activation]
11381	NCF1	IL4R	[activation]
11382	TP53	PEO1	[activation]
11383	ATM	RNF20	[activation]
11384	RIMS2	RAPGEF4	[activation]
11385	EIF4A3	HIST1H2AA	[activation]
11386	IRAK1	MAP3K3	[activation;inhibition]
11387	FTH1	GRB2	[activation]
11388	HDAC1	BUB1	[activation]
11389	CBL	PTPRJ	[activation]
11390	MCM3	POLR2A	[activation]
11391	GABARAPL2	DDX17	[activation]
11392	KRIT1	PIK3C3	[activation]
11393	MMP17	MMP2	[activation]
11394	GNA14	CXCR1	[activation]
11395	RHOC	RTKN	[activation]
11396	YES1	GAB1	[activation]
11397	VCAM1	RANBP1	[activation]
11398	CBL	YWHAG	[activation]
11399	PTBP3	GCN1L1	[activation]
11400	A2M	MYOC	[inhibition]

11401	ABL1	ARHGEF11	[activation]
11402	CEBPG	FOS	[activation]
11403	SUMO1	PRKAA1	[activation]
11404	CTNNB1	FOXM1	[activation;inhibition]
11405	CTSV	BRAF	[activation]
11406	HSP90AA1	PAFAH1B1	[activation]
11407	HSPB1	VCAM1	[activation]
11408	MET	FBXO6	[inhibition]
11409	EHMT2	BANF1	[activation]
11410	KALRN	ENO3	[activation]
11411	MLXIPL	MLX	[activation]
11412	ATP1A1	ERBB2	[activation]
11413	FOXP2	FAM124A	[activation]
11414	ERBB2IP	SMAD3	[inhibition]
11415	PIGR	CDK6	[activation]
11416	VCAM1	HSPA9	[activation]
11417	ESR1	PIK3CA	[activation]
11418	TNFRSF11B	THBS1	[activation]
11419	CTR9	CIAO1	[activation]
11420	NOTCH2NL	NPBWR2	[activation]
11421	EZR	WHSC1	[activation]
11422	GRB2	RALGPS1	[activation]
11423	IRF2	PRKACA	[activation]
11424	MME	IRAK2	[activation]
11425	TFAP2A	RHOA	[activation]
11426	SHB	CRK	[activation]
11427	PRNP	HSPA4	[activation]
11428	NUP107	LRRK2	[activation]
11429	FBXO6	ITPRIP	[inhibition]
11430	GATA4	ALX4	[activation]
11431	HSPA1L	NFKB1	[activation]
11432	RB1	CEBPA	[activation;inhibition]
11433	STAG1	CALM1	[activation]
11434	NOL11	MARK3	[activation]
11435	F12	APOH	[activation]
11436	XIRP2	LRRK2	[activation]
11437	SMC1A	MLH1	[activation]
11438	TSC2	GRB2	[activation]
11439	IGFALS	IGF1	[activation]
11440	SNW1	NBN	[activation]
11441	MYC	HIGD1A	[activation]
11442	DYRK1A	RNF169	[activation;inhibition]
11443	MBP	CDK7	[activation]
11444	ICAM1	HNRNPA2B1	[activation]
11445	TYSND1	HSD17B4	[activation]
11446	VCAM1	PPP2CA	[activation]
11447	SMC3	NOTCH1	[activation]
11448	MDC1	PAXIP1	[activation]
11449	PLG	CXCL2	[activation]
11450	IRF3	MAPK8	[activation]
11451	HIST1H1C	EIF2AK2	[activation]
11452	SHE	ABL1	[activation]
11453	DYRK1B	PAK1	[activation]
11454	ELANE	LRP1	[activation]
11455	TP53	BTRC	[activation]
11456	TNFRSF14	ESR1	[activation]
11457	LXN	MAGEB2	[activation]
11458	PTPRC	LEPR	[activation]
11459	SGSM3	RAB5A	[activation]
11460	CRK	BUB1	[activation]
11461	CXCL12	PF4	[activation]
11462	STAT5A	IL2RB	[activation]
11463	BCAP31	APP	[activation]
11464	MYB	SUMO1	[activation]
11465	KMT2A	TP53	[activation]
11466	TULP3	RYR1	[activation]
11467	GMNN	CASP3	[activation]
11468	SHC1	CDH5	[activation]
11469	HOMER1	RYR2	[activation]
11470	MAP1LC3A	STK4	[activation]
11471	NFKBIL1	LUC7L	[activation]
11472	HSPA8	HERC5	[inhibition]
11473	HEMGN	BUB1	[activation]
11474	EZR	TRAF6	[activation]
11475	KIF23	NXF1	[activation]
11476	GADD45GIP1	GADD45G	[activation]

11477	IL37	SMAD3	[activation]
11478	DVL1	DAAM1	[activation]
11479	TP53	MAGEA2	[activation]
11480	CD19	CD82	[activation]
11481	CCL13	MMP3	[activation]
11482	NCF1	PRKDC	[activation]
11483	STRAP	HSF1	[activation]
11484	AURKB	RASSF1	[inhibition]
11485	SRPK1	HBS1L	[activation]
11486	GNB1	GNGT1	[activation]
11487	CD9	ADAM10	[activation]
11488	CCDC151	SUV39H2	[activation]
11489	GSK3B	SNAI1	[activation;inhibition]
11490	NFKB1	SPI1	[inhibition]
11491	BHLHE41	HDAC1	[activation]
11492	APC	CFTR	[inhibition]
11493	SOX2	YPEL5	[activation]
11494	IKBKE	IKBKB	[activation]
11495	PHC2	MAPKAPK2	[activation]
11496	GRB2	GAREML	[activation]
11497	APP	ANXA2	[activation]
11498	RHPN2	SMURF1	[inhibition]
11499	CYFIP2	BIRC3	[activation;inhibition]
11500	CARM1	E2F1	[activation]
11501	URM1	VIPR2	[activation]
11502	SMAD2	TRIM62	[activation]
11503	FST	ANG	[inhibition]
11504	RHOG	VAV3	[activation]
11505	HIST1H4A	GNB2	[activation]
11506	IKBKB	HSPA1L	[activation]
11507	ITGB1	DAG1	[activation]
11508	HLA-B	STMN1	[activation]
11509	PAXIP1	COMTD1	[activation]
11510	TP53	TDRD12	[activation]
11511	SOX8	OLIG2	[activation]
11512	BTRC	OGT	[activation]
11513	RPA1	PSIP1	[activation]
11514	MLST8	RPTOR	[activation;inhibition]
11515	NXF1	NIPSNAP1	[activation]
11516	YES1	NIF3L1	[activation]
11517	TPT1	FANCA	[activation]
11518	NCOA2	FOS	[activation]
11519	FAM117B	DYRK1A	[activation]
11520	MLLT4	PVRL3	[activation]
11521	APC	NUP153	[activation]
11522	ZBTB16	CASP3	[activation]
11523	EP300	STAT5A	[activation]
11524	EIF2S1	SNW1	[activation]
11525	ILK	DPM1	[activation]
11526	NCOA3	AR	[activation]
11527	EPOR	SYK	[activation]
11528	HIST1H4A	ITGA4	[activation]
11529	PML	SUV39H1	[activation]
11530	BCAR1	PPAP2B	[activation]
11531	CHN1	CDK5	[activation]
11532	CBLB	PTK2B	[activation]
11533	NUMBL	GRIP1	[inhibition]
11534	ARL8B	TUBB	[activation]
11535	HSP90AB1	DDR1	[activation]
11536	PTPRF	EGFR	[activation]
11537	CDC25B	YWHAE	[activation]
11538	PPARG	SETDB1	[activation]
11539	DDX3Y	CD81	[activation]
11540	TNF	LTA	[activation]
11541	PPP2R1B	STK24	[activation]
11542	SIK3	SMAD3	[activation]
11543	TERT	TEP1	[activation]
11544	UBE2I	AURKB	[activation]
11545	CD1D	P4HTM	[activation]
11546	TRPM7	PLCG1	[activation]
11547	PSEN1	TGFBR1	[activation]
11548	MYO16	PIK3R1	[activation]
11549	BLID	FGFR4	[activation]
11550	MDM2	LUC7L2	[activation]
11551	GFPT1	HLA-B	[activation]
11552	NXF1	SFXN4	[activation]

11553	TNFRSF13B	TRAF2	[activation]
11554	STAT1	SMURF1	[activation]
11555	SFPQ	PTK6	[activation]
11556	RANBP9	DDX4	[activation]
11557	DIABLO	NR4A1	[activation]
11558	SNAP23	WASH2P	[activation]
11559	CCND1	LPL	[activation]
11560	APP	FHL2	[activation]
11561	INSR	GAB1	[activation]
11562	VCAM1	EIF4A1	[activation]
11563	EPHA3	CRK	[activation]
11564	SMC4	RAD21	[activation]
11565	C22orf43	C19orf66	[activation]
11566	NUDCD3	EGFR	[activation]
11567	CDC25A	CCNI	[activation]
11568	LATS1	KIAA1549	[inhibition]
11569	ATAD3A	GRB2	[activation]
11570	URM1	USP47	[activation]
11571	ARFGAP1	RAD52	[activation]
11572	SIRT6	ANAPC1	[activation]
11573	SRC	PGR	[activation]
11574	GNAT2	ADRB2	[activation]
11575	KAT2A	PPARGC1A	[activation]
11576	CDK1	MBP	[activation]
11577	H2AFX	PEF1	[activation]
11578	EPPK1	CDKN1A	[activation;inhibition]
11579	HDAC1	NOTCH1	[activation]
11580	SH3BP2	SYK	[activation]
11581	SMAD4	STX17	[activation]
11582	GRIP2	GRIA3	[activation]
11583	PTPN4	CRK	[activation]
11584	VAV2	SOCS1	[inhibition]
11585	C6orf211	ARF6	[activation]
11586	S100A10	CDK16	[activation]
11587	RAC1	PTK2	[activation]
11588	SMAD4	STK35	[activation]
11589	ATF4	MOV10	[activation]
11590	NUMA1	GNAI1	[activation]
11591	GET4	HSPB1	[activation]
11592	CPLX2	STX3	[activation]
11593	SETDB1	HDAC1	[activation]
11594	GUCY1B3	GUCY1A2	[activation]
11595	PIK3C2A	EGFR	[activation]
11596	VCAM1	NUP93	[activation]
11597	GRB2	PDE6G	[activation]
11598	GADD45GIP1	NFE2L2	[activation]
11599	RACGAP1	YWHAG	[activation]
11600	SRPK2	U2AF1	[activation]
11601	LRRK2	SSR4	[activation]
11602	CXCR5	GNAI2	[activation]
11603	CD81	HNRNPA3	[activation]
11604	RAN	STAU1	[activation]
11605	ARPC1B	APP	[activation]
11606	ZBTB17	IRF8	[activation]
11607	PARD3	PARD6G	[activation]
11608	SH3D19	SOS2	[activation]
11609	IQGAP1	CYBB	[activation]
11610	TRAF3	TNFRSF13B	[activation]
11611	FOXF2	ABL1	[activation]
11612	HLA-C	EIF3F	[activation]
11613	CDC37	PRKCI	[activation]
11614	PACSIN1	GAS7	[activation]
11615	ECSIT	MAST1	[activation]
11616	SLC6A12	SCRIB	[activation]
11617	SYK	KIT	[activation]
11618	YWHAQ	HSPA1A	[inhibition]
11619	BATF3	ATF2	[activation]
11620	HNRNPA0	ITGA4	[activation]
11621	RAPGEF4	BZRAP1	[activation;inhibition]
11622	IRAK1	IRAK4	[activation;inhibition]
11623	POLD1	POLD2	[activation]
11624	PAXIP1	PDCD11	[activation]
11625	EPHA8	G3BP1	[activation]
11626	ACVR1	UBE2E3	[activation]
11627	OSCAR	FCAR	[activation]
11628	SRC	CDH5	[activation]

11629	EIF2B3	GOLM1	[activation]
11630	MAP2K1	HSPA8	[activation]
11631	BCL2L1	BNIP3	[activation]
11632	RPS6KA5	HIST2H2BE	[activation]
11633	RAC3	ESR1	[activation]
11634	HDAC1	CDC20	[inhibition]
11635	DYRK1A	SIPA1L1	[activation]
11636	CUL1	HSPA1L	[inhibition]
11637	TERF1	KPNB1	[activation]
11638	JUNB	SMARCA4	[activation]
11639	CASP12	NOD2	[inhibition]
11640	CCDC8	HLA-C	[activation]
11641	IKBKG	KIF23	[activation]
11642	CDK4	KAT2A	[activation]
11643	SHC1	GAB1	[activation]
11644	AURKB	PPP1CA	[activation]
11645	PRKAR2B	PRKACA	[activation]
11646	PRKCA	NFATC1	[activation]
11647	STAT4	MAP2K6	[activation]
11648	PRKD3	TAF1	[activation]
11649	IRS4	PPP2R1A	[activation]
11650	EDA2R	TRAF3	[activation]
11651	YWHAZ	NCKAP1	[inhibition]
11652	STK4	MAP1LC3C	[activation]
11653	YWHAZ	GCN1L1	[activation]
11654	RPS17	GABARAP	[activation]
11655	SEMA4C	NCDN	[activation]
11656	TNFRSF14	APP	[activation]
11657	DOCK2	VAV1	[activation]
11658	HSPG2	PRELP	[activation]
11659	EDN3	EDNRA	[activation]
11660	TRAF2	TEKT3	[activation]
11661	IKBKB	PPP1R15A	[activation]
11662	TUBA4A	GRB2	[activation]
11663	SGOL1	PPP2CB	[inhibition]
11664	KIAA1598	FABP3	[activation]
11665	CD5	CD79A	[activation]
11666	JUN	HSP90AA1	[activation]
11667	PIK3C3	CLPTM1L	[activation]
11668	CTNNB1	CCNA1	[activation]
11669	MAPK1	PRKCZ	[activation]
11670	RPTOR	GNB1	[activation]
11671	LTBP1	SMURF1	[inhibition]
11672	GABARAPL1	GSN	[activation]
11673	EEA1	BMI1	[activation]
11674	HBZ	HBD	[inhibition]
11675	SP1	SKIV2L2	[activation]
11676	TRAF6	MAP2K1	[activation]
11677	SAV1	PTPN14	[activation]
11678	CASP8	FIS1	[activation]
11679	GSK3B	RPL36AL	[inhibition]
11680	YAP1	CCDC85C	[inhibition]
11681	MAP1LC3B	ATG10	[activation]
11682	BUB1B	CDC20	[inhibition]
11683	RPA3	RAB7A	[activation]
11684	OBSL1	PHIP	[activation]
11685	PAX6	IPO13	[activation]
11686	AMBRA1	PIK3C3	[activation]
11687	MYC	MRPS34	[activation]
11688	PARD3B	SMAD2	[activation]
11689	KSR2	HSP90AB1	[activation]
11690	GPR183	MTA1	[activation]
11691	CD247	SH2B3	[activation]
11692	HES1	YWHAB	[activation]
11693	RELA	NKX2-1	[activation]
11694	APC	EPAS1	[inhibition]
11695	RAN	SLX1A	[activation]
11696	FGB	ASB7	[activation]
11697	TBC1D3F	CDC42	[activation;inhibition]
11698	GNB2L1	FYN	[activation]
11699	FANCB	F3	[activation]
11700	SDC4	SGTA	[activation;inhibition]
11701	SMAD1	PAK1	[activation]
11702	CYLD	AURKB	[inhibition]
11703	SKIL	IL36RN	[activation]
11704	YWHAH	MAP3K2	[activation]

11705	ABL1	RAPGEF1	[activation]
11706	PRKAA2	NOTCH2NL	[activation]
11707	RANBP2	SNW1	[activation]
11708	CD44	SLC7A11	[activation]
11709	GRB2	ITPR3	[activation]
11710	ADRM1	INSIG2	[activation]
11711	GH2	GHR	[activation]
11712	IRF2	PPP3CA	[activation]
11713	CASP3	LMNB1	[activation]
11714	PTEN	PTK2B	[activation]
11715	CTGF	FGFR1OP	[activation]
11716	IL24	APC	[activation]
11717	MASTL	RPS6KA1	[activation]
11718	NFKBIA	G3BP2	[activation]
11719	MAPK7	JUP	[activation]
11720	TERF1	ACD	[activation]
11721	ARPC1B	PAK1	[activation]
11722	NR1H3	SIRT1	[activation]
11723	GABARAPL2	PRMT1	[activation]
11724	EIF2S2	PLEKHM1	[activation]
11725	SIRT1	RICTOR	[activation]
11726	SLC9A3R1	GNAQ	[activation]
11727	LRRK2	DVL3	[activation]
11728	IGHA1	CDH1	[activation]
11729	GRB2	AMBP	[activation]
11730	CASP2	GRB2	[activation]
11731	VIPR1	VIP	[activation]
11732	CDK5R1	CDK3	[activation]
11733	MASP1	SERPING1	[inhibition]
11734	NR0B2	ZAP70	[activation]
11735	NR3C1	RPS6KA5	[activation]
11736	HSPB1	TRIM54	[activation]
11737	RASSF1	MOAP1	[inhibition]
11738	TEC	PTPRC	[activation]
11739	CREBBP	KAT2A	[activation]
11740	DDX39B	VCAM1	[activation]
11741	CRK	HSPA6	[activation]
11742	TICAM1	TRAF1	[activation]
11743	HSPA4	CDC25C	[activation]
11744	TP63	SMAD2	[activation]
11745	MEGF10	HDAC4	[activation]
11746	EIF4G1	HSPB1	[activation]
11747	EIF2S3	IKBKE	[activation]
11748	SARNP	DDX39B	[activation]
11749	OBSL1	DDX56	[activation]
11750	RANBP2	AXIN1	[activation]
11751	JAG1	NOTCH2	[activation]
11752	TP53	RPA1	[activation]
11753	CD4	GNAI2	[activation]
11754	FAM83B	YWHAB	[activation]
11755	RPP38	SRC	[activation]
11756	HNRNPL	HNRNPA1	[activation]
11757	PRKDC	HSP90AA1	[activation]
11758	S100A14	CASP8	[inhibition]
11759	CHGB	APP	[activation]
11760	RRP36	OBSL1	[activation;inhibition]
11761	RPS20	PIK3CA	[activation]
11762	GABARAPL2	TTBK1	[activation]
11763	NR1H3	KAT2B	[activation]
11764	MAPKAPK2	ELAVL1	[activation]
11765	IGHM	RUNX1	[activation]
11766	USHBP1	CCDC116	[activation]
11767	S100A4	HBEGF	[activation]
11768	STK32C	GRB2	[activation]
11769	TGFBRAP1	ACVR2B	[activation]
11770	SNCA	TH	[activation]
11771	CCAR2	PYHIN1	[inhibition]
11772	EXOC6	EGFR	[activation]
11773	SNW1	SMC4	[activation]
11774	INADL	CLDN1	[activation]
11775	NR3C1	CEBPB	[activation]
11776	NUP153	MAPK14	[activation]
11777	CDK3	CDKN3	[activation]
11778	CTR9	MYC	[activation]
11779	GNAI2	EGFR	[activation]
11780	DDX58	UBE2D2	[activation]

11781	LINC00526	USHBP1	[activation]
11782	CRK	PLSCR1	[activation]
11783	SCO2	MYC	[activation]
11784	HOXD9	PKNOX1	[activation]
11785	CDK1	NDEL1	[activation]
11786	CDC27	CDC20	[inhibition]
11787	SRC	YWHAE	[activation]
11788	HSP90AA1	TERT	[activation]
11789	PF4	THBD	[activation]
11790	CBL	YWHAB	[activation]
11791	ANXA2	GH1	[activation]
11792	ITGA4	VAPA	[activation]
11793	CTLA4	FYN	[activation]
11794	AGTRAP	HSPA4	[activation]
11795	FBXO25	CDK5RAP2	[activation]
11796	EP300	NCOA3	[activation]
11797	CASP3	MAP4K1	[activation]
11798	PINX1	TERF1	[activation]
11799	MYO18A	VCAM1	[activation]
11800	PRL	BMP1	[activation]
11801	WDR20	YWHAB	[activation]
11802	DDX5	CALM1	[activation]
11803	RIPK2	XIAP	[activation]
11804	YWHAB	CTTN	[activation]
11805	SUMO1	ACTB	[activation]
11806	EHMT2	SOX2	[activation]
11807	ELF4	SKP2	[activation]
11808	FGF7	HSPG2	[activation]
11809	MAP2K1	PTPRJ	[activation]
11810	LILRB2	PTPN6	[activation]
11811	WDR48	CTNND1	[activation]
11812	PRKCI	MAP2K5	[activation]
11813	IL10RB	IL10RA	[activation]
11814	AURKA	HMMR	[activation]
11815	FBXO30	ACVR1	[activation;inhibition]
11816	SRPK2	DDX39B	[activation]
11817	TRA2A	BARD1	[activation]
11818	RAB5C	TRAF6	[activation]
11819	TGFBR2	ARHGEF7	[activation]
11820	SHC1	HSPA6	[activation]
11821	ATP6V1A	VAMP8	[activation]
11822	PPP2CA	TWIST1	[activation]
11823	IFI16	E2F1	[activation]
11824	GRB7	SETDB1	[activation]
11825	MYC	IDH3A	[activation]
11826	EPHB6	NOTCH2NL	[activation]
11827	RFXANK	RFXAP	[inhibition]
11828	MYC	PTPN14	[activation]
11829	ITGA4	PRRC2C	[activation]
11830	SELPLG	TPST2	[activation]
11831	CCDC8	STX7	[activation]
11832	NR3C1	HNRNPA1	[activation]
11833	VPS26A	ILK	[activation]
11834	SV2A	ETS1	[activation]
11835	ARL14EP	SETDB2	[activation]
11836	APP	TGFB2	[activation]
11837	MAML1	MAML2	[activation]
11838	POP5	CAMK2B	[activation]
11839	C17orf85	OBSL1	[activation;inhibition]
11840	GRN	CCNT1	[activation]
11841	SERBP1	MAPK13	[activation]
11842	CDKN2C	TLE1	[inhibition]
11843	CTNNB1	KAT2B	[activation]
11844	TBXA2R	PRKACA	[inhibition]
11845	CDK1	SFN	[activation]
11846	NFATC1	HDAC1	[activation]
11847	STX7	UBL4A	[activation]
11848	YWHAB	MST1R	[activation]
11849	FZR1	SETD8	[activation]
11850	PTPN7	PRKACA	[activation]
11851	MBTPS1	SPHK1	[inhibition]
11852	ITGA4	HIST2H2BF	[activation]
11853	HSPB1	EPB41L1	[activation]
11854	DLX4	SP1	[activation]
11855	NCAM1	EGFR	[activation]
11856	PRMT1	HLA-C	[activation]

11857	VTN	POMC	[activation]
11858	HNRNPA1	PRKDC	[activation]
11859	EIF2B1	ARL4D	[activation]
11860	CCNE1	ARHGEF5	[activation]
11861	C9orf156	SRC	[activation]
11862	IQGAP1	IKBKB	[activation]
11863	RAC2	CD81	[activation]
11864	MST1R	SFN	[activation]
11865	DLG3	PTK2B	[activation]
11866	PPP1R15A	HSPA8	[inhibition]
11867	TWIST1	CHD4	[activation]
11868	VDAC1	CASP4	[inhibition]
11869	GATA1	SPIB	[activation]
11870	INHBA	FSTL3	[activation]
11871	NOTCH1	MOB1A	[activation]
11872	SIRT1	PIK3R1	[activation]
11873	HSP90AB1	KLHL29	[activation]
11874	FGG	ICAM1	[activation]
11875	FBXO30	APC	[inhibition]
11876	GRB2	SMARCD3	[activation]
11877	CCDC106	TP53	[activation]
11878	BCL2	IKBKB	[activation]
11879	GOLGA2	GEM	[activation]
11880	RPA3	SEH1L	[activation]
11881	HDAC1	NR3C1	[activation]
11882	CDK4	ZSCAN1	[inhibition]
11883	HRAS	PTPRJ	[activation]
11884	MUTYH	SMAD1	[activation]
11885	ITGA4	HSP90AB1	[activation]
11886	SRSF5	MAPK6	[activation]
11887	IL15	TRAF3	[activation]
11888	ITGA4	TLN1	[activation]
11889	APC	SPTBN2	[inhibition]
11890	CDK4	RFC4	[activation]
11891	DIRAS3	KRAS	[activation]
11892	USF1	NFYA	[activation]
11893	KIT	BCAR3	[activation]
11894	VCAM1	DHX16	[activation]
11895	PUM1	SAV1	[activation]
11896	EIF2B2	MRFAP1L1	[activation]
11897	NECAP1	JUN	[activation]
11898	HSP90AA1	PRKCZ	[activation]
11899	RPA3	PKP3	[activation]
11900	TRAPPC11	TRAPPC8	[activation]
11901	PTPN6	CD37	[activation;inhibition]
11902	HP1BP3	BARD1	[activation]
11903	ACTB	ATP13A2	[activation]
11904	HSPA6	MLF2	[activation]
11905	MALSU1	ICT1	[activation]
11906	SATB1	PYHIN1	[activation]
11907	GEM	CTSL	[activation]
11908	DYRK1A	YWHAG	[activation]
11909	SEPT9	STK11	[activation]
11910	SETDB1	AKT2	[activation]
11911	DAPK3	ULK1	[inhibition]
11912	CFTR	GRN	[activation]
11913	HDAC4	CAMK2D	[activation]
11914	TNFRSF4	TRAF1	[activation]
11915	STX17	STK11	[activation]
11916	NSMCE4A	HSPB1	[activation]
11917	SLC20A1	RAPGEF1	[activation]
11918	TRIB1	CEBPB	[activation]
11919	BMP10	SERINC3	[activation]
11920	FOS	SMAD3	[activation]
11921	PLK1	DCTPP1	[activation]
11922	UBA5	UFC1	[activation]
11923	MYL12A	MDC1	[activation]
11924	STX17	AURKA	[activation]
11925	CRKL	LTBP4	[activation]
11926	ADNP	PLCG1	[activation]
11927	SPP1	LRRC4	[activation]
11928	BZW2	BZW1	[activation]
11929	MBL2	MASP2	[activation]
11930	CEP164	DVL3	[activation]
11931	MAL	LYN	[activation]
11932	DLAT	MDM2	[activation]

11933 EIF2B5 CSNK2A2 [activation]
11934 USP18 IFNAR2 [activation]
11935 ARRB1 CYTH2 [activation]
11936 CSNK2B CDC25B [activation]
11937 PRKCH FYN [activation]
11938 MAST2 MXRA7 [activation]
11939 CCNC FOXP2 [activation]
11940 CSNK1D PPP1R14A [activation]
11941 JUP PTPRF [inhibition]
11942 PIK3R1 GABRA3 [activation]
11943 EGFR STAP2 [activation]
11944 MAP2K6 FBXO31 [activation]
11945 C11orf84 EGFR [activation]
11946 KAT2B CCND1 [activation]
11947 CBL EPHB6 [activation]
11948 KAT2B RPS6KB1 [activation]
11949 RAF1 NXF1 [activation]
11950 NCK1 PFN1 [activation]
11951 SP1 MEF2D [activation]
11952 BAD CREB3L3 [inhibition]
11953 STRN PPP2R1A [inhibition]
11954 GNG12 GNB1 [activation]
11955 PTK2B FGFR2 [activation]
11956 BMPR2 NBEA [activation]
11957 SUMO1 GCN1L1 [activation]
11958 CDH18 CDH12 [activation]
11959 CBL IQGAP1 [inhibition]
11960 NXF1 MCM4 [activation]
11961 PTPN6 CD33 [activation;inhibition]
11962 CACNA1C CALM1 [activation]
11963 HIST1H2BK ICAM1 [activation]
11964 TGFBR1 GNB3 [activation]
11965 CHEK2 PPP2R5C [activation]
11966 PTP4A3 NCDN [activation]
11967 LIME1 PLCG2 [activation]
11968 SMAP A2M [inhibition]
11969 THBS1 FGA [activation]
11970 CASP7 TNFRSF1A [activation]
11971 TP53 RPL26L1 [activation]
11972 VAPB ZFYVE27 [activation]
11973 ETV5 SEZ6L2 [activation]
11974 TFG GRB2 [activation]
11975 CHD8 CASR [activation]
11976 HEMGN AURKA [activation]
11977 CTNNBIP1 CASP4 [inhibition]
11978 FBXO31 SKP1 [inhibition]
11979 SAT1 HIF1A [activation]
11980 JUNB BATF2 [activation]
11981 WWP1 RASA1 [activation]
11982 NEDD8 NUP214 [activation]
11983 RANBP2 IPO5 [activation]
11984 IKBKB NAA20 [activation]
11985 ARHGAP29 RHOD [activation]
11986 ABL1 BRCA1 [activation]
11987 CSNK2A1 NCAPD2 [activation]
11988 PRKAA2 MAP3K6 [inhibition]
11989 ARHGEF6 SH3GL3 [activation]
11990 PRKG1 GAPDH [inhibition]
11991 AFF1 NSD1 [activation]
11992 DRD4 GRB2 [activation]
11993 FASLG CACNB4 [activation]
11994 ICAM1 RPL11 [activation]
11995 EPB41L3 PSEN1 [activation]
11996 OBSL1 PRDX3 [activation;inhibition]
11997 ANKRD52 PLK1 [activation;inhibition]
11998 IQGAP1 RPA2 [activation]
11999 YWHAG STK4 [activation]
12000 VCAM1 ACTR2 [activation]
12001 PTN RIT1 [activation]
12002 EGFR RIPK1 [activation]
12003 YWHAH PARD6G [activation]
12004 TWIST1 GLI3 [activation]
12005 NFATC1 PML [activation]
12006 ATG5 IGHA1 [activation]
12007 RPA1 GTF2H3 [activation]
12008 FHL2 FAM129A [activation]

12009	UBC	SHH	[inhibition]
12010	IRS4	MYC	[activation]
12011	CSNK2A1	RELA	[activation]
12012	PLCG2	PLCG1	[activation]
12013	RHOG	SMAD1	[activation]
12014	HIF1A	ARNTL	[activation]
12015	CEBPD	CREBBP	[activation]
12016	STEAP3	SETD7	[activation]
12017	APC	PTK2	[activation]
12018	DDX52	MYC	[activation]
12019	ACTBL2	PXK	[activation]
12020	APP	BDNF	[activation]
12021	TGFBR1	ARL4D	[activation]
12022	CASP6	PIN1	[activation]
12023	CD4	MME	[activation]
12024	TP53	SETD1A	[activation]
12025	CREB3	CTSW	[activation]
12026	ANXA2	ITGA4	[activation]
12027	CCDC8	SLIT2	[activation]
12028	RHOA	KTN1	[activation]
12029	HSPA1A	TRIM24	[activation]
12030	MAGED2	MYC	[activation]
12031	APC	ANXA7	[activation]
12032	RIPK1	MAP3K8	[activation]
12033	TPD52	NXF1	[activation]
12034	CASP3	PDE5A	[activation]
12035	MAVS	BIRC2	[activation]
12036	ARAP1	TNFRSF10A	[activation]
12037	NXF1	RPTOR	[activation]
12038	PRKAA1	SND1	[inhibition]
12039	LCP2	MAP4K1	[activation]
12040	FBXO6	SKP1	[inhibition]
12041	APOE	ANKH	[activation]
12042	APP	OTX2	[activation]
12043	TBK1	MBP	[inhibition]
12044	NCK2	RHOU	[activation]
12045	HOXB7	IRAK3	[inhibition]
12046	ATP1A1	TPT1	[activation]
12047	MLH1	FCGBP	[activation]
12048	CCND1	PRMT5	[activation]
12049	BRD4	MED1	[activation]
12050	HSF1	SUB1	[activation]
12051	GREB1	CDKN2C	[inhibition]
12052	TP53	RASGRF1	[activation]
12053	NUDC	HLA-B	[activation]
12054	PXN	ARHGEF7	[activation]
12055	TRAF6	PPM1G	[activation]
12056	LYN	FASLG	[activation]
12057	FAM120A	DYSF	[activation]
12058	SLC6A1	STX1A	[activation]
12059	EPAS1	ARNTL	[activation]
12060	STK4	GGCT	[activation]
12061	PIK3R1	ANK3	[activation]
12062	LIMK1	PAK4	[activation;inhibition]
12063	CASP8	FYN	[activation]
12064	FYN	CD55	[activation]
12065	GDI1	CDC42	[activation]
12066	SMAD4	FOXO3	[inhibition]
12067	GABARAPL2	RPS6	[activation]
12068	AGTRAP	NDRG4	[activation]
12069	APP	SOCS3	[inhibition]
12070	VCL	BCAR1	[activation]
12071	A2M	IFIT5	[inhibition]
12072	EP300	GRB2	[activation]
12073	CAMK2D	CREM	[activation]
12074	ISL1	APP	[activation]
12075	TRAF6	BIRC3	[activation]
12076	CCL5	TRIP6	[activation]
12077	SKIL	HIPK1	[activation]
12078	RECQL5	PRKDC	[activation]
12079	MBP	NEK9	[activation]
12080	RNMTL1	ICT1	[activation]
12081	PFN2	FMNL1	[activation]
12082	STK4	CDK3	[activation]
12083	DLGAP4	EXOC4	[activation]
12084	DIABLO	XIAP	[activation]

12085 USP9X SMURF1 [inhibition]
12086 TP53 PRKD1 [activation]
12087 HSF1 EEF1G [activation]
12088 UBE2E3 KLHL42 [activation]
12089 PTPRC CSNK2A2 [activation]
12090 CREBBP STAT5B [activation]
12091 NOP2 PTPRJ [activation]
12092 YWHAZ CDC5L [activation]
12093 FOXO3 SMAD1 [activation;inhibition]
12094 ULK1 ATG16L1 [inhibition]
12095 DAB2IP CUL1 [inhibition]
12096 ESR2 ZBTB17 [activation]
12097 TGFBR1 NUP37 [activation]
12098 CDKN1A CDC6 [activation]
12099 BCAR3 GAB1 [activation]
12100 LIN7C HTR2C [activation]
12101 BARD1 SRSF6 [activation]
12102 DOK2 DNAJB11 [inhibition]
12103 PTPN6 SYK [activation;inhibition]
12104 FYB RASA1 [activation]
12105 CD86 MARCH8 [activation]
12106 MAP3K7 BCL10 [activation]
12107 FRS3 NTRK2 [activation]
12108 RAD17 RFC5 [activation]
12109 EP300 MCM3 [activation]
12110 RPA1 PPP2CA [activation]
12111 LRRK2 RNF219 [activation]
12112 ILK ZNF133 [activation]
12113 PRPF6 GABARAP [activation]
12114 PRKCA GABRR1 [activation]
12115 PRKCD ITGA6 [activation]
12116 PTPRJ FLT1 [activation]
12117 BARD1 UBE2W [activation]
12118 PRKACA PDE3B [activation]
12119 PPP2CA STRN4 [inhibition]
12120 RAB5A PIK3C3 [activation]
12121 RXRA PLK1 [inhibition]
12122 YAP1 DDX17 [activation]
12123 ATF2 GLUD2 [activation]
12124 EIF4A2 JAK1 [activation]
12125 CTNNB1 FANCC [activation]
12126 PLIN1 PRKACA [activation]
12127 PARP1 NCAPD2 [activation]
12128 ERBB2 CD44 [activation]
12129 FYN CSMD2 [activation]
12130 SRF RXRG [activation]
12131 PRKG1 MRVI1 [inhibition]
12132 ALK PDX1 [activation]
12133 CDC42BPB CDC42 [activation]
12134 FYN HSP90AB1 [activation]
12135 ZFYVE9 SVEP1 [activation]
12136 CDH1 RAB8B [activation]
12137 SOCS3 GAB1 [inhibition]
12138 RASA1 APP [activation]
12139 PLS3 EGFR [activation]
12140 CPE POLA2 [activation]
12141 PSMC3IP NR3C1 [activation]
12142 CRMP1 DPYSL2 [activation]
12143 EVI5 RAB11A [activation]
12144 DOK1 CBLL1 [activation]
12145 JMJD1C PPARG [activation]
12146 NOS3 DNM1 [activation]
12147 WHSC1 NOL10 [activation]
12148 ESR1 SHOC2 [activation]
12149 SORCS2 NGFR [activation;inhibition]
12150 CKAP4 MLH1 [activation]
12151 ARFGEF2 PRKAR1A [activation]
12152 PTPRC WAS [activation]
12153 RUNX1 CCND1 [activation]
12154 GJB1 SRC [activation]
12155 CFL1 MYC [activation;inhibition]
12156 DYSF SLC12A6 [activation]
12157 STOM VCAM1 [activation]
12158 XPOT RANBP2 [activation]
12159 EXOC5 CDH2 [activation]
12160 MDM2 DDX39B [activation]

12161	ARRB2	STAT1	[activation]
12162	SRPK2	CASK	[activation]
12163	ICAM3	ACTB	[activation]
12164	CDKN2B	ARPC3	[activation]
12165	NUDCD3	SORT1	[activation]
12166	SPP1	MARCKS	[activation]
12167	HIF1AN	NFKBIA	[activation]
12168	DVL3	SIRT1	[activation]
12169	CASP1	BIRC3	[activation]
12170	ADAM10	TGFA	[activation]
12171	CHMP3	VAV2	[activation]
12172	A2M	IGSF8	[inhibition]
12173	MET	SH2B3	[activation]
12174	AVPR2	PRKCA	[activation]
12175	MSH4	EIF3F	[activation]
12176	EIF4G3	VAV2	[activation]
12177	F10	PRKAB1	[activation]
12178	INCA1	CDKN2D	[inhibition]
12179	RAC1	NOSTRIN	[activation]
12180	MLH1	ATF2	[activation]
12181	PPM1B	ANXA1	[activation]
12182	PRKACA	PFKFB1	[activation]
12183	WDR59	EP300	[activation]
12184	WDR82	TP53	[activation]
12185	SHC1	ACTA1	[activation]
12186	TTI2	PRKDC	[activation]
12187	NEK9	RAN	[activation]
12188	SDC2	PRKCA	[activation]
12189	HSPA2	STK4	[activation]
12190	RAC1	IFNGR1	[activation]
12191	UBE2W	RNF166	[activation]
12192	ASB12	FGG	[activation]
12193	RYR1	PDLIM3	[inhibition]
12194	TNS3	ILK	[activation]
12195	TRAF6	FAAP24	[activation]
12196	JUP	CTNND1	[activation]
12197	NUDT21	SRPK1	[activation]
12198	ESR1	NFKB2	[activation]
12199	PAK1	PGM1	[activation]
12200	CSNK2B	IKBKB	[activation]
12201	HSPB1	TBC1D1	[activation]
12202	KRT31	SNAI1	[activation;inhibition]
12203	YWHAG	HSPA8	[inhibition]
12204	CD34	CHST4	[activation]
12205	MEGF10	RANBP10	[activation]
12206	NSMAF	CD40	[activation]
12207	RPS6KA1	RPS6KA2	[activation]
12208	GHR	BTRC	[activation]
12209	PLXNB2	HMOX2	[activation]
12210	ABL1	SP1	[activation]
12211	SMURF1	RASD2	[activation]
12212	FLNA	F3	[activation]
12213	GNB2	NFATC2	[activation]
12214	MAPK3	STAT5A	[activation]
12215	WDR88	NUDC	[activation]
12216	PPP1CA	NDP	[activation]
12217	BARD1	PGAM5	[activation]
12218	RPS6KB1	CLEC3B	[activation]
12219	TRUB2	ICT1	[activation]
12220	SMARCA4	RB1	[activation]
12221	HUS1	RPA1	[activation]
12222	ACACA	IKBKAP	[activation;inhibition]
12223	CDK4	DBNL	[inhibition]
12224	CKS1B	CDKN1A	[activation;inhibition]
12225	GPC6	GABARAPL2	[activation]
12226	SRF	CASP6	[activation]
12227	RAC3	LDHB	[activation]
12228	MAPK1	ADAM17	[activation]
12229	CCZ1B	NOTCH1	[activation]
12230	SORBS1	ABI1	[activation]
12231	USP11	MRE11A	[activation]
12232	YWHAZ	PRKD1	[activation]
12233	SHCBP1	NXF1	[activation]
12234	CSNK1A1L	CEACAM3	[activation]
12235	RIMBP3C	FASLG	[inhibition]
12236	GSDMB	SERPINA1	[inhibition]

12237	YWHAB	NCKAP1	[inhibition]
12238	ARL1	HLA-B	[activation]
12239	ATRIP	MX2	[activation]
12240	AR	SRY	[activation]
12241	ATP7A	ATOX1	[activation]
12242	KPNB1	ADRB2	[activation]
12243	ODF3L2	EGFR	[activation]
12244	RAD51	RPA2	[activation]
12245	NOTCH2NL	IL2RG	[activation]
12246	CIITA	CDK1	[activation]
12247	RIC8A	GNAI2	[activation;inhibition]
12248	NXF1	RAN	[activation]
12249	TP53	HINFP	[activation]
12250	ADAM10	FBXO6	[activation]
12251	KCNE4	KCND2	[activation]
12252	CAV1	PPP2R1A	[activation]
12253	RIPK1	EP300	[activation]
12254	CDK5	MAPK10	[activation]
12255	CCDC8	SRC	[activation]
12256	C2orf50	SPP1	[activation]
12257	SPP1	PRKG1	[activation]
12258	TIAM1	IGHM	[activation]
12259	TGFA	ERBB4	[activation]
12260	CRTC2	ITPR1	[inhibition]
12261	TUBA4A	ZAP70	[activation;inhibition]
12262	CHRM4	GPRASP1	[activation]
12263	SDCBP	ULK1	[inhibition]
12264	DBF4B	CDC7	[activation]
12265	SNCG	MAPK3	[inhibition]
12266	PAK2	SNX9	[activation]
12267	PTGS2	COPS3	[activation]
12268	TSSC4	TRAF2	[activation]
12269	HSP90AB1	ATF2	[activation]
12270	STAT3	RET	[activation]
12271	YWHAZ	ACTB	[activation]
12272	APP	CASP3	[activation]
12273	HIST1H1C	PRKCA	[activation]
12274	RND3	YWHAZ	[activation]
12275	NOTCH1	PTBP3	[activation]
12276	YWHAG	ARHGEF2	[activation]
12277	TXLNA	NACAP1	[activation]
12278	GRB2	NCKAP5	[activation]
12279	RPA1	PAXIP1	[activation]
12280	SREK1	SRPK3	[activation]
12281	PEBP1	TRAF6	[inhibition]
12282	GUCY2C	GUCA2A	[activation]
12283	ICT1	MRPL21	[activation]
12284	PRKACA	ITCH	[activation]
12285	PRKCE	OPRD1	[activation]
12286	TNF	GNB2L1	[activation]
12287	HIST1H4A	CDK1	[activation;inhibition]
12288	SSX2IP	PLK1	[activation]
12289	LAMA5	MEP1A	[activation;inhibition]
12290	PIK3AP1	GRB2	[activation]
12291	TEC	BMX	[activation]
12292	DVL1	SMAD2	[activation]
12293	CDC5L	CTNND1	[activation]
12294	MYOG	TTC25	[activation]
12295	GRB2	CTTN	[activation]
12296	MAPK14	RPL22	[activation]
12297	IL13RA1	TYK2	[activation]
12298	CD40	JAK3	[activation]
12299	SLIT3	CAPN1	[activation]
12300	NCOA3	IKBKB	[activation]
12301	SREBF2	TRRAP	[activation]
12302	MYC	ECSIT	[activation]
12303	AKT1	HSPA5	[activation]
12304	EIF4A2	EIF4G1	[activation]
12305	BMX	PTPN21	[activation]
12306	GRK5	TBXA2R	[inhibition]
12307	PRKAA1	ABI1	[inhibition]
12308	LMNB1	RAC1	[activation]
12309	STK38	FBXO6	[inhibition]
12310	WISP2	IGF2	[activation]
12311	VASP	ABI3	[activation]
12312	CDK5RAP3	CDK5R1	[activation]

12313	UBE2H	RNF113B	[activation]
12314	DDX39B	IL7R	[activation]
12315	RAC1	NOS2	[activation]
12316	CALM1	TRPC3	[activation]
12317	GABRA2	MDM2	[activation]
12318	ERBB3	PIK3CA	[activation]
12319	ST5	ABL1	[activation]
12320	CAMK2A	DAPK2	[activation]
12321	SAV1	MOB1A	[activation]
12322	C1QTNF9	LARP4B	[activation]
12323	SERPINE1	UBQLN1	[inhibition]
12324	C7orf50	PRNP	[activation]
12325	SUMO2	TIMP1	[activation]
12326	ARHGAP28	HSPB1	[activation]
12327	MAPK4	ATP1A1	[activation]
12328	ABL2	TRIP10	[activation]
12329	ATG5	MAP1LC3B	[activation]
12330	EIF3C	CD4	[activation]
12331	CASP2	CASP10	[activation]
12332	NR4A1	SERPINA4	[inhibition]
12333	PML	CDK2	[activation]
12334	NCF1	GP1BB	[activation]
12335	RAD21	RAN	[activation]
12336	ARPC3	THNSL2	[activation]
12337	ELK3	MAPK14	[activation]
12338	SDF4	MYC	[activation]
12339	STK11	MOB4	[activation]
12340	KPNA1	GRM1	[activation]
12341	GSN	PXN	[activation]
12342	SMARCA5	SUMO3	[inhibition]
12343	CYBB	CYBA	[activation]
12344	PAFAH1B1	KATNA1	[activation]
12345	CDC42EP3	SRPK1	[activation]
12346	STK4	DNAJA1	[activation]
12347	PAX6	DIAPH1	[activation]
12348	MEN1	SMAD1	[activation]
12349	SNAI1	EZH2	[activation]
12350	PAK2	FYN	[activation]
12351	STAT1	GSTK1	[activation]
12352	XPO5	ARF6	[activation]
12353	TNF	RIPK1	[activation]
12354	NFYB	MYC	[activation]
12355	CHD3	CASP6	[activation]
12356	IGHM	IGKC	[activation]
12357	NME2	TERF1	[activation]
12358	SMURF1	UBE2M	[inhibition]
12359	ASNS	ATG101	[activation]
12360	WNK1	TNS1	[activation]
12361	SH3BGRL	EGFR	[activation]
12362	ICK	MCM7	[activation]
12363	PPP1R13B	LATS1	[inhibition]
12364	FZD1	DLG2	[activation]
12365	CD44	GARS	[activation]
12366	PIK3R1	IL13	[activation]
12367	TRAF6	MRPL13	[activation]
12368	HNRNPA1	H2AFX	[activation]
12369	GAB1	BMX	[activation]
12370	NXF1	RPUSD4	[activation]
12371	SLC25A4	TNFRSF1B	[inhibition]
12372	TENC1	CSK	[activation]
12373	SUMO1	FZD8	[activation]
12374	PSG2	SMAD4	[activation]
12375	MLF1	DNAJB2	[inhibition]
12376	E2F3	DDX21	[activation]
12377	UBE2D1	NBN	[activation]
12378	LRP1	SHC1	[activation]
12379	KDR	ITGB3	[activation]
12380	KCNK15	SFN	[activation]
12381	PPP2R4	PRKCB	[activation]
12382	FYN	CDC37	[activation]
12383	PRNP	MOG	[activation]
12384	SP1	SHC1	[activation]
12385	CD247	CD48	[activation]
12386	RAC1	RCC2	[activation]
12387	HNRNPD	GRB2	[activation]
12388	HPRT1	GABARAPL2	[activation]

12389	TRAIP	TRAF6	[activation]
12390	TNK1	YWHAE	[activation]
12391	SMC2	HIST2H2BE	[activation]
12392	HOXD13	CREBBP	[activation]
12393	SRC	DAB2	[activation]
12394	NCL	GZMA	[activation]
12395	SMAD1	SUV39H1	[activation]
12396	EGF	PIK3R2	[activation]
12397	GABRA1	DRD4	[activation]
12398	ERBB3	ZAP70	[activation]
12399	SKP2	ATM	[activation]
12400	ATG5	RPS16	[activation]
12401	CRK	TPX2	[activation]
12402	TP53	RNF20	[activation]
12403	HDAC4	CIITA	[activation]
12404	STARD10	GRB2	[activation]
12405	CHD7	CTCF	[activation]
12406	NUP62	IFT20	[activation]
12407	RAB2B	FAM71C	[activation]
12408	NOTCH3	KAT2B	[activation]
12409	MLH1	ACTG2	[activation]
12410	CDC5L	TANC2	[activation]
12411	EGFR	GSN	[activation]
12412	UBE2E2	XIAP	[activation]
12413	PTPRD	PTPRE	[activation;inhibition]
12414	EXOC8	EXOC4	[activation]
12415	RRAGA	IL7R	[activation]
12416	RBM48	BID	[activation]
12417	PAWR	DRD2	[activation]
12418	CNTN2	NFYB	[activation]
12419	HMGN3	SRPK1	[activation]
12420	GFPT1	MYC	[activation]
12421	CCDC17	FGFR3	[activation]
12422	PAK1	LIMK1	[activation]
12423	FMNL3	CIAO1	[activation]
12424	CRK	EGFR	[activation]
12425	FGFR1	FLRT3	[activation]
12426	GABARAPL2	TGM1	[activation]
12427	JAK3	PTPRC	[activation]
12428	LCK	CCR5	[activation]
12429	RIPK1	CLIP3	[activation]
12430	CCNE1	MCM3	[activation]
12431	RPS6KB1	TRIB2	[activation]
12432	STK4	TGM3	[activation]
12433	MCM3	HLA-C	[activation]
12434	MBP	PRKCA	[activation]
12435	HCK	FCGR1A	[activation]
12436	GRB2	UBASH3B	[activation]
12437	CTSV	CDKN2A	[activation;inhibition]
12438	COPS5	SPP1	[activation]
12439	CCDC8	CTNNA1	[activation]
12440	NFKB1	ETS1	[activation]
12441	SMARCA2	RAN	[activation]
12442	CBL	STAT5B	[activation]
12443	INO80	ACTB	[activation]
12444	NGFR	MYCN	[activation]
12445	PARD6B	PARD6G	[activation]
12446	RANGRF	RAN	[activation]
12447	PRDM2	TP53	[activation]
12448	ZNF740	PRNP	[activation]
12449	SLC20A1	MAPK6	[activation]
12450	EIF4B	RPS6KA1	[activation]
12451	TFDP1	AXIN1	[activation]
12452	BARD1	U2AF2	[activation]
12453	GNL3	TP53	[activation]
12454	RAN	TGFBR1	[activation]
12455	SPRY1	LCE2D	[inhibition]
12456	RADIL	CFLAR	[inhibition]
12457	GRB2	APP	[activation]
12458	CRKL	PTPN11	[activation]
12459	ADCK1	NXF1	[activation]
12460	GH1	RANBP1	[activation]
12461	GAB3	LYN	[activation]
12462	ATM	EEF1E1	[activation]
12463	CRK	PXN	[activation]
12464	ARF6	INSR	[activation]

12465	CDK6	SORBS1	[activation]
12466	HCK	WAS	[activation]
12467	MYO1C	SMARCA5	[activation]
12468	STAT2	SMARCE1	[activation]
12469	STAG2	CDC5L	[activation]
12470	EIF4A3	SCYL2	[activation]
12471	TP53	SAT1	[activation]
12472	GLRX	PTPN1	[activation]
12473	MCC	MAPRE1	[activation]
12474	YWHAG	ITGA4	[activation]
12475	LRRK2	MSN	[activation]
12476	RIPK1	CSNK1A1	[activation]
12477	BMPR1A	HRAS	[activation]
12478	MAPK9	SMAD2	[activation]
12479	SPP1	STK39	[activation]
12480	YWHAB	SKP2	[activation]
12481	BAAT	MLH1	[activation]
12482	BTK	PIP4K2A	[activation]
12483	RAE1	EEF2K	[activation]
12484	CD79B	CD79A	[activation]
12485	CTR9	CNTNAP2	[activation]
12486	YWHAB	DAPK1	[activation]
12487	GNAI1	GPSM3	[activation;inhibition]
12488	JPH2	CAV3	[inhibition]
12489	CIITA	SMARCA4	[activation]
12490	ST13	TNFRSF14	[activation]
12491	NCOA1	ESR2	[activation]
12492	PPARA	RXRB	[inhibition]
12493	NFKBIA	ANXA1	[activation]
12494	NCAPG2	PHLDA3	[activation]
12495	CAV3	PDGFRB	[activation]
12496	PVRL2	DBN1	[activation]
12497	HLA-B	SERBP1	[activation]
12498	SRC	ARRB1	[activation]
12499	PFN1	OBSL1	[activation]
12500	GABRA1	PPP3CA	[activation]
12501	ARHGEF7	CD244	[activation]
12502	APC	KRT15	[inhibition]
12503	HNRNPA1	RPA1	[activation]
12504	FOSL1	TAB2	[activation]
12505	BAX	SNCA	[activation]
12506	RRAS2	TRAF3	[activation]
12507	MAP3K4	PTPN6	[activation;inhibition]
12508	HSPB1	HSPA5	[inhibition]
12509	ATR	EP300	[activation]
12510	PPP2R2A	PPP2CA	[inhibition]
12511	DDX24	EIF4A2	[activation]
12512	LGR4	EIF4G1	[activation]
12513	FASLG	SRGAP3	[activation]
12514	EIF4A1	CCDC8	[activation]
12515	DHRS12	H3F3A	[activation]
12516	APP	ACYP1	[activation]
12517	MAP4K4	SLC9A1	[activation]
12518	CALM1	TNFRSF1A	[activation]
12519	HSP90AA1	IRS4	[activation]
12520	CSNK2A1	STMN1	[activation]
12521	CSNK1G2	DBNDD2	[inhibition]
12522	CAV1	CSK	[activation]
12523	CCL26	APP	[activation]
12524	BACH1	NXF1	[activation]
12525	SMARCA4	WHSC1	[activation]
12526	RAN	IPO11	[activation]
12527	PRKCB	PPP1CA	[activation]
12528	RIPK1	TRADD	[activation]
12529	YWHAB	RAPGEF6	[activation]
12530	DLG4	FZD2	[activation]
12531	RAD52	POLR2A	[activation]
12532	RELA	PRMT5	[activation]
12533	IL18BP	IL18	[activation]
12534	IFNAR1	TYK2	[activation]
12535	LCE4A	RGS20	[activation;inhibition]
12536	TLN2	FOS	[activation]
12537	LAG3	CENPJ	[activation]
12538	CPE	INS	[activation]
12539	PTPN6	CSF2RB	[activation;inhibition]
12540	TIMELESS	CRY1	[activation]

12541	SMARCA1	PYHIN1	[activation]
12542	SIRT2	FZR1	[activation]
12543	STAT5B	CENPJ	[activation]
12544	CCR10	GRB2	[activation]
12545	ARPC4	ARPC5L	[activation]
12546	FYN	NEK8	[activation]
12547	ESD	HLA-B	[activation]
12548	VAV1	BLNK	[activation]
12549	ETS1	RPL13A	[activation]
12550	OGG1	PRKCA	[activation]
12551	SRC	ID4	[activation]
12552	YWHAG	PYHIN1	[activation]
12553	BIRC2	RHOA	[activation]
12554	STAT1	PPP2R4	[activation]
12555	UFD1L	PIK3CA	[activation]
12556	ACACA	STK4	[activation;inhibition]
12557	EPS8L3	SRPK2	[activation]
12558	SMURF1	FBXO30	[inhibition]
12559	EGFR	APOB	[activation]
12560	YWHAZ	EIF5B	[activation]
12561	NR5A2	CTNNB1	[activation]
12562	LRRK2	RPL11	[activation]
12563	RND3	SFN	[activation]
12564	STC2	PTN	[activation]
12565	ASIC3	ASIC2	[activation]
12566	GRB2	RPS28	[activation]
12567	NCK2	FASLG	[inhibition]
12568	OBSL1	WDR18	[inhibition]
12569	ABI1	NCF1	[activation]
12570	GDF2	BMPR2	[activation]
12571	FCGR2C	FYN	[activation]
12572	SMAD2	SNRNP70	[activation]
12573	SERPINB12	SERPINF2	[inhibition]
12574	APOH	CDC42	[activation]
12575	IRF7	KAT2A	[activation]
12576	ADAP1	DVL3	[activation]
12577	UBE2N	MDM2	[activation]
12578	MEIS1	HOXA2	[activation]
12579	FBXL19	RAC3	[activation]
12580	PTPN14	CTNNA2	[activation]
12581	CTSG	LRP1	[activation]
12582	PRAM1	A2M	[inhibition]
12583	YWHAZ	RPRD1A	[activation]
12584	MAP3K4	CNTRL	[activation]
12585	TTC3	AKT2	[activation]
12586	DVL2	TFDP1	[activation]
12587	CTNNA1	MDM2	[activation]
12588	ACVR1	NEK8	[activation;inhibition]
12589	H2AFX	CHD1L	[activation]
12590	SRPK2	MAPKAPK5	[activation]
12591	FANCL	FANCA	[activation]
12592	ZNF598	EIF4E2	[inhibition]
12593	DGKD	IGSF21	[inhibition]
12594	ERBB2IP	MPP3	[inhibition]
12595	PRKAA1	RAF1	[activation]
12596	OSBPL6	PLK1	[activation]
12597	RAB11FIP4	ARF6	[activation]
12598	TNS3	NXF1	[activation]
12599	DNAJA1	MAP3K7	[inhibition]
12600	DHX30	NXF1	[activation]
12601	TBX2	SOX2	[activation]
12602	LRRK2	MTA1	[activation]
12603	CDK2	MCM3	[activation]
12604	WFDC5	STAG2	[inhibition]
12605	PAXIP1	SRSF4	[activation]
12606	TP53	RBBP5	[activation]
12607	EZR	MAPK10	[activation]
12608	RASA1	TSC1	[activation]
12609	CDC26	MYC	[activation]
12610	ABI2	PRKAA2	[activation]
12611	YWHAZ	TPD52L1	[activation]
12612	BLK	HSP90AB1	[activation]
12613	GP1BB	PTK2B	[activation]
12614	RAD9A	RPA1	[activation]
12615	CNTN5	MYC	[inhibition]
12616	TRAF2	FAM107A	[activation]

12617	IRS4	PRKCZ	[activation]
12618	MCM7	NXF1	[activation]
12619	RRN3	MYO1C	[activation]
12620	BAX	PYCARD	[inhibition]
12621	SIL1	CCND3	[inhibition]
12622	ILK	SRPRB	[activation]
12623	MAPK1	HSF1	[activation]
12624	GSK3B	IGF2BP1	[activation]
12625	LRRK1	TIRAP	[activation]
12626	HBG2	VCAM1	[activation]
12627	AGR3	TGFB1	[activation]
12628	CEBPG	ATF5	[activation]
12629	VEGFA	HSP90AA1	[activation]
12630	SRPK2	DBF4B	[activation]
12631	PTGES3	ESR1	[activation]
12632	ACD	ENO2	[activation]
12633	KCTD17	STK16	[activation]
12634	EP300	GATA5	[activation]
12635	PIK3R1	FLT3	[activation]
12636	STAT5B	PDGFRA	[activation]
12637	IPO5	GABARAPL2	[activation]
12638	PTK2	CXCR4	[activation]
12639	EGFR	SH2D3C	[activation]
12640	EPHA8	LRRK1	[activation]
12641	GCN1L1	EGFR	[activation]
12642	ACTB	CPNE2	[activation]
12643	DUSP3	FGF7	[activation]
12644	TRAF3	APOBEC3C	[activation]
12645	PIK3CA	PDE4D	[activation]
12646	PRKCD	TAGLN	[activation]
12647	ZDHHC17	PIK3R1	[activation]
12648	DUSP10	MAPK9	[inhibition]
12649	SORBS1	SOS1	[activation]
12650	ABCE1	LRRK2	[activation]
12651	HSP90AB1	LCK	[activation]
12652	NTN4	CDKN1A	[activation;inhibition]
12653	TACC2	AURKC	[activation]
12654	SMAD3	WEE1	[inhibition]
12655	YWHAZ	CYFIP1	[inhibition]
12656	PPP1CC	ENO2	[activation]
12657	RPP38	GRB2	[activation]
12658	MET	SH2D2A	[activation]
12659	CSMD2	PLCG1	[activation]
12660	CSNK2A1	HIF1A	[activation]
12661	GNAI1	MTNR1A	[activation]
12662	HIF1AN	NFKB1	[activation]
12663	MAD2L1	MAD2L2	[inhibition]
12664	RPA2	CCNH	[activation]
12665	ACTA1	ITPKA	[activation]
12666	RASA1	YES1	[activation]
12667	ST14	APC	[inhibition]
12668	APP	CAMK1D	[activation]
12669	NUDC	MOS	[activation]
12670	APP	CDK5R1	[activation]
12671	CDC5L	ERCC1	[activation]
12672	DUSP1	HSPA4	[inhibition]
12673	IL4R	FES	[activation]
12674	EIF1B	MIF	[activation]
12675	EIF4A2	IBTK	[activation]
12676	VCAM1	YWHAE	[activation]
12677	GABARAPL2	HBG1	[activation]
12678	RHOA	CAV1	[activation]
12679	PRMT5	CAPN1	[activation]
12680	PPP1R12A	RPA2	[activation]
12681	EGFR	UCK2	[activation]
12682	TRIM29	MLH1	[activation]
12683	MCL1	BID	[activation]
12684	ASPH	TRDN	[activation]
12685	SMARCA4	STAT1	[activation]
12686	PSEN1	PRKCZ	[activation]
12687	TRAF6	PA2G4	[activation]
12688	COPS7A	NFE2L2	[activation]
12689	STAU1	SRPK1	[activation]
12690	NDEL1	CCHCR1	[activation]
12691	YWHAE	HSP90AB1	[activation]
12692	MAVS	STAT1	[activation]

PPM1A	DVL3	[activation]
SRPK2	AMMECR1L	[activation]
KLF5	EP300	[activation]
NOTCH1	RIF1	[activation]
PRKDC	BIRC5	[activation]
FOXO3	ATM	[activation]
CTNNB1	SMAD4	[activation]
CHUK	TP53	[activation]
MED1	EPAS1	[activation]
CYLC2	EGFR	[activation]
PKP4	GABARAP	[activation]
ARF6	CAPN1	[activation]
MAPKAPK2	BAG2	[activation]
YWHAQ	HMGCS2	[activation]
ATF2	GLS	[activation]
SREBF2	CASP3	[activation]
RARA	GADD45G	[activation]
PARD6B	PRKCZ	[activation]
NCOA3	HIST3H3	[activation]
IL36RN	SMAD3	[activation]
PLD2	CFL1	[activation]
TIPRL	PPP2CA	[inhibition]
PRKAA2	HSP90AB1	[activation]
MYC	ADNP	[activation]
CD44	CD74	[activation]
EIF4EBP1	PPP2R4	[activation]
RBL1	CCNA2	[inhibition]
EIF4G3	USP10	[activation;inhibition]
IGHA1	FCAR	[activation]
AATF	CHEK2	[activation]
BCL6	JUNB	[activation]
HLA-B	RAB2A	[activation]
PCBP1	CD81	[activation]
EFEMP1	NXF1	[activation]
SYK	GAB1	[activation]
LY6D	GRB2	[activation]
IQGAP1	DVL2	[activation]
NOTCH4	SMAD4	[activation]
RASD2	RAF1	[activation]
SPRY1	CACNA1A	[inhibition]
PIK3R2	TGFBR2	[activation]
VIM	HSPB1	[inhibition]
PSIP1	RPA2	[activation]
HP1BP3	VCAM1	[activation]
BMP1	MSTN	[activation]
CHUK	MAVS	[activation]
CHEK2	RAD50	[activation]
ATF6B	NFYC	[activation]
ARRB1	MAPK9	[activation]
APP	CHEK2	[activation]
RAF1	RANBP9	[activation]
PRKCB	NCF1	[activation]
NR3C1	EIF5B	[activation]
PLXNA3	CBFA2T3	[activation]
CDKN1A	PPFIBP1	[activation;inhibition]
PAK1	PCBP1	[activation]
ABI2	TRIP6	[activation]
TBC1D4	CDK5RAP2	[activation]
WARS2	APP	[activation]
CHTOP	NXT1	[activation]
PYCARD	PSTPIP1	[inhibition]
TNFRSF17	TRAF2	[activation]
TSHR	LHCGR	[activation]
SIM2	ARNT2	[activation]
NFATC1	HNRNPA1	[activation]
CASP8	BCL2L10	[activation]
DKK1	KREMEN1	[inhibition]
ARRB2	CSN2	[inhibition]
CDK18	YWHAE	[activation]
CNOT11	CNOT10	[activation]
ATR	CDC6	[activation]
BRCA1	AR	[activation]
KLK6	COL4A5	[activation]
BCL9	CTNNB1	[activation]
TICAM2	IRAK4	[activation]
TNF	FAM105B	[activation]

12769	TIA1	ITGA4	[activation]
12770	NPY2R	RGS12	[activation;inhibition]
12771	AVEN	NXF1	[activation]
12772	TNFRSF13C	TRAF1	[activation]
12773	PEA15	L1CAM	[activation]
12774	NCK1	OXT	[activation]
12775	KPNB1	CD4	[activation]
12776	CASP3	COPS6	[activation]
12777	IRAK3	FOLR1	[inhibition]
12778	HOMER3	IKBKB	[activation]
12779	YWHAB	LRFN1	[activation]
12780	HSPD1	CD4	[activation]
12781	PIGM	CHEK1	[activation;inhibition]
12782	OPN1MW	RANBP2	[activation]
12783	HSPB2	TGFB1I1	[activation]
12784	FYN	BCL3	[activation]
12785	DISC1	SMC2	[activation]
12786	PLG	LAMA1	[activation]
12787	TSPAN10	TP53	[activation]
12788	APP	OSM	[activation]
12789	STAT5A	PPP2CA	[activation]
12790	PRKACA	GNA13	[activation]
12791	KRAS	DDX47	[activation]
12792	DDX18	OBSL1	[activation]
12793	GAB1	MAP3K3	[activation]
12794	DVL2	GABARAP	[activation]
12795	EIF4A3	HIST1H1C	[activation]
12796	C6	F2	[activation]
12797	CCR10	PRRC2A	[activation]
12798	TOLLIP	BHLHE40	[activation]
12799	EGFR	SEL1L	[activation]
12800	BACH1	ANAPC5	[activation]
12801	AURKC	HSP90AB1	[activation]
12802	PRKACA	NXPE4	[activation]
12803	CHGB	MARK3	[activation]
12804	MYC	MCM4	[activation]
12805	PDGFRB	BAG1	[activation]
12806	SMURF1	CDKL1	[inhibition]
12807	PLCG1	SPRY1	[activation]
12808	BCL2L2	TP53	[activation]
12809	GART	PXN	[activation]
12810	CASP4	SMC1A	[activation]
12811	FRK	PTEN	[activation]
12812	TP53	HSPA5	[activation]
12813	POFUT1	DLL1	[activation]
12814	IRAK4	HIST1H1C	[activation]
12815	MLH1	PIK3C2A	[activation]
12816	OLFML3	SETDB1	[activation]
12817	KALRN	SUMO3	[activation]
12818	DAPK3	AK3	[activation]
12819	IL18	SHMT2	[activation]
12820	GNG11	GNB1	[activation]
12821	MAPKAPK2	LIMK1	[activation]
12822	BID	CASP1	[activation]
12823	HTATSF1	NOTCH1	[activation]
12824	CSF3R	WSB1	[activation]
12825	CCDC8	CDK1	[activation]
12826	ABL1	YWHAG	[activation]
12827	CHEK2	FAM84B	[activation]
12828	CXorf57	MLH1	[activation]
12829	HSPB1	PASK	[activation]
12830	C4orf17	SUV39H1	[activation]
12831	SP1	BCL6	[activation]
12832	INSR	PRKCD	[activation]
12833	E2F2	GIT2	[activation;inhibition]
12834	HGF	ST14	[inhibition]
12835	PML	BCL6	[activation]
12836	STK11	YWHAG	[activation]
12837	NCOA3	ESR1	[activation]
12838	YTHDC2	HSP90AA1	[activation]
12839	APPL1	FARS2	[activation]
12840	APP	KLK6	[activation]
12841	PDGFRB	S1PR1	[activation]
12842	APP	SLFNL1	[activation]
12843	HSP90AB1	PRKD1	[activation]
12844	RB1	PIK3R3	[activation]

12845	PHACTR2	SRC	[activation]
12846	LRRK1	GRB2	[activation]
12847	GRB2	YWHAB	[activation]
12848	SPSB1	TP53	[activation]
12849	KIF2A	HIST3H3	[activation]
12850	UGT1A1	UGT1A3	[activation]
12851	STRN	PAXIP1	[activation]
12852	EPHB6	HSP90AB1	[activation]
12853	DPPA4	GRB2	[activation]
12854	IKBKB	EIF2AK2	[activation]
12855	SUMO3	RBL1	[inhibition]
12856	YWHAG	CHEK1	[activation]
12857	TRAF6	EIF4E	[inhibition]
12858	CLEC4G	NUP214	[activation]
12859	TRABD2A	MYC	[activation]
12860	BCAR1	ABL1	[activation]
12861	DNM2	SRC	[activation]
12862	TFAP2A	TP53	[activation]
12863	MAP2K7	FBXO6	[activation]
12864	GRB2	EPS15L1	[activation]
12865	SFN	CYTH2	[activation]
12866	HCK	ASAP1	[activation]
12867	CDC42BPG	TBK1	[activation]
12868	MCF2L	RHOG	[activation]
12869	ARHGDIG	KXD1	[activation]
12870	TRAF6	HEATR3	[activation]
12871	KDR	BMX	[activation]
12872	EXOC6	MAST1	[activation]
12873	STX1A	STXBP1	[activation]
12874	ID3	MYF6	[activation]
12875	GRB2	MTA1	[activation]
12876	PRR5L	MTOR	[activation]
12877	UBE2D3	HLA-B	[activation]
12878	ISYNA1	DUSP14	[inhibition]
12879	VAPA	VAMP2	[activation]
12880	CUL1	GYS1	[inhibition]
12881	HSPB1	ADPRHL2	[activation]
12882	RAPGEF2	YAP1	[activation]
12883	HDAC1	APPL1	[activation]
12884	MYL3	CASP3	[activation]
12885	TGFB2	TGFBR3	[activation]
12886	MEIS1	CRTC1	[activation]
12887	GNB2L1	IFNAR2	[activation]
12888	SUMO1	PFN1	[activation]
12889	YY1	ATF7	[activation]
12890	NOTCH1	PBRM1	[activation]
12891	EP300	ANAPC5	[activation]
12892	CPNE3	SNW1	[activation]
12893	SLC4A3	PRKCE	[activation]
12894	BTRC	FGD3	[activation]
12895	PTPRA	GRB2	[activation]
12896	SMURF1	FLNA	[inhibition]
12897	ARF6	TRIM23	[activation]
12898	APC	CSNK2A1	[activation]
12899	TGFBR1	TSSK1B	[inhibition]
12900	PPP2R4	IER3	[activation]
12901	ATM	MAP1S	[activation]
12902	EIF2S3	GH1	[activation]
12903	NCKAP1	YWHAG	[inhibition]
12904	SPN	CTNNB1	[activation]
12905	RXRA	ARID5A	[inhibition]
12906	NR1H2	PRKCA	[activation]
12907	ILK	LONP1	[activation]
12908	DNAJA3	FAM131C	[inhibition]
12909	BLM	BRIP1	[activation]
12910	ELMO1	PTGS2	[activation]
12911	DLEU1	SETDB1	[activation]
12912	C11orf57	STAC3	[activation]
12913	CCNB1	CKS1B	[inhibition]
12914	MAPRE1	AURKB	[activation]
12915	ITGA2B	ITGB3	[activation]
12916	LTA4H	ARPC3	[activation]
12917	CDK5RAP2	PRKACA	[activation]
12918	PMF1	NFE2L2	[activation]
12919	NCKAP1	CYFIP1	[inhibition]
12920	CBL	PTPRC	[activation]

12921	RPA3	RAB14	[activation]
12922	IL7R	JAK3	[inhibition]
12923	UBA5	MCM2	[activation]
12924	CDK1	MYC	[activation]
12925	CTAGE5	TRAF6	[activation]
12926	POLD1	POLD4	[activation]
12927	PRKD1	SRC	[activation]
12928	RPS19	MAGEB2	[activation]
12929	RAB5C	APPL2	[activation]
12930	AVP	OXTR	[activation]
12931	KAT2B	PTEN	[activation]
12932	EDA	EDA2R	[activation]
12933	HSP90AB1	RAF1	[activation]
12934	MMP3	CCL8	[activation]
12935	OBSL1	JUP	[inhibition]
12936	HDAC1	STAT2	[activation]
12937	TRIB2	CARD16	[inhibition]
12938	STK4	HSP90AA1	[activation]
12939	CPNE3	QARS	[activation]
12940	SPERT	PID1	[activation]
12941	RELA	IER3	[activation]
12942	AHSA2	MOS	[activation]
12943	APP	BCL2L11	[activation]
12944	DACH1	SMAD3	[inhibition]
12945	NPHS1	YES1	[activation]
12946	LIFR	FBXO6	[inhibition]
12947	ITGB1	RPA1	[activation]
12948	PPP3R2	CCND1	[activation]
12949	APP	MARS2	[activation]
12950	CREB3L1	C5	[activation]
12951	GNB2L1	BCL2L11	[activation]
12952	NR3C1	TBP	[activation]
12953	CYTH4	CNKSR1	[activation]
12954	FASLG	ITK	[activation]
12955	SMURF1	JUNB	[activation]
12956	FGA	SERPINF2	[activation]
12957	FANCC	BMPR1A	[activation]
12958	CREBBP	NCOA3	[activation]
12959	EGFR	BIRC3	[activation]
12960	HIF1A	CREBBP	[activation]
12961	NXPE3	APP	[activation]
12962	MAPKAPK3	FBXW7	[activation]
12963	NRIP1	TRAF2	[activation]
12964	NOTCH2NL	DNAL4	[activation]
12965	PRMT1	PPARA	[activation]
12966	JUNB	JDP2	[activation]
12967	YWHAZ	MYL3	[inhibition]
12968	PRKCA	GRIA1	[activation]
12969	CASP7	RAC1	[activation]
12970	LYN	SCG3	[activation]
12971	PRKACA	CSK	[activation]
12972	TRIM74	HSP90AB1	[activation]
12973	TRAF6	UBA2	[activation]
12974	SNTA1	CD3E	[activation]
12975	CAB39L	STK11	[activation]
12976	PIK3R1	BRCA1	[activation]
12977	YWHAZ	RAP1A	[activation]
12978	TBC1D3F	RAB5A	[activation]
12979	TP53	CSNK1A1	[activation]
12980	C8orf74	SUV39H2	[activation]
12981	HEXA	ABL1	[activation]
12982	HLA-A	KIR3DL2	[activation]
12983	LYN	AR	[activation]
12984	LUC7L2	NDEL1	[activation]
12985	PTK2B	PTPN11	[activation]
12986	PSKH2	HSP90AB1	[activation]
12987	PARP2	IFNA16	[activation]
12988	SDC2	NF1	[activation]
12989	DAB1	SOCS1	[inhibition]
12990	TP53	JMY	[activation]
12991	TGFBR2	SCUBE3	[activation]
12992	FAS	CCND3	[inhibition]
12993	HNRNPA1	IGF2BP1	[activation]
12994	PVR	DYNLT1	[activation]
12995	PPIA	RB1	[inhibition]
12996	CSH1	PRLR	[inhibition]

12997	C7orf25	PTN	[activation]
12998	PAK2	RPL32	[activation]
12999	GPI	MDM2	[activation]
13000	GRB7	CPNE6	[activation]
13001	TP53	TAF5	[activation]
13002	MET	VAV3	[activation]
13003	ARPC4	ARPC5	[activation]
13004	MDM2	PRPF6	[activation]
13005	VAV2	EGFR	[activation]
13006	CUL4B	FANCM	[activation]
13007	JUP	BIRC2	[activation]
13008	EPB41L3	HSPA12A	[activation]
13009	RRP1	BARD1	[activation]
13010	ILK	ARRB2	[activation]
13011	HLA-B	CALM1	[activation]
13012	TPD52L2	MDM2	[activation]
13013	MAPK8	EGFR	[activation]
13014	PAXIP1	PPP1R12A	[activation]
13015	MOV10	MAPKAPK2	[activation]
13016	RGS4	GNAQ	[activation]
13017	RETN	A2M	[inhibition]
13018	ELANE	SERPINE1	[inhibition]
13019	YWHAE	TGFB1	[activation]
13020	ARF1	GABARAPL1	[activation]
13021	ETV7	RPTOR	[activation]
13022	GRB2	LY6G6F	[activation]
13023	DDX21	JUN	[activation]
13024	FRAT1	DVL1	[inhibition]
13025	CAV1	BST1	[activation]
13026	ITGA4	HMGB1	[activation]
13027	MAGEB18	TP53	[activation]
13028	PRKCB	ADRBK1	[activation]
13029	YTHDC1	SRPK2	[activation]
13030	DAZAP2	CXCL12	[activation]
13031	NXF1	NOL6	[activation]
13032	RELA	SOCS1	[inhibition]
13033	NUP62	NCL	[activation]
13034	MAPRE1	EPB41	[activation]
13035	IFIH1	ATG12	[inhibition]
13036	CASP3	FYN	[activation]
13037	PPM1D	WHSC1L1	[activation]
13038	ACTG1	REL	[activation]
13039	SPTBN1	PLCB1	[activation]
13040	POU1F1	ETS1	[activation]
13041	SYNPO	GRB2	[activation]
13042	TTN	IGHG1	[activation]
13043	ICAM1	H3F3A	[activation]
13044	SRC	LRP1	[activation]
13045	PPP4R1	TRAF2	[activation]
13046	HIST1H1C	MDM2	[activation]
13047	CCNA1	BRCA1	[activation]
13048	HMGB1	PSEN1	[activation]
13049	TBXA2R	ARRB2	[inhibition]
13050	RB1	BCR	[activation]
13051	MAPK3	SCRIB	[activation]
13052	TRADD	NGFR	[activation]
13053	NXF1	MYO6	[activation]
13054	CASP7	DCTN1	[activation]
13055	GNA15	APP	[activation]
13056	RAC1	PRKCA	[activation]
13057	PARP1	NOTCH1	[activation]
13058	RPAP1	ILK	[activation]
13059	NXF1	SNAI2	[activation]
13060	BRCA1	CASP3	[activation]
13061	SFN	TSC2	[activation]
13062	CALM1	JAK2	[activation]
13063	REPS1	HSF1	[activation]
13064	ANGPTL4	AURKA	[activation]
13065	NOP16	SRPK1	[activation]
13066	NRXN2	NLGN3	[activation]
13067	NOTCH1	FRYL	[activation]
13068	GNB3	CXCR5	[activation]
13069	GPR3	GNPAT	[activation]
13070	BIRC2	BOLA1	[activation;inhibition]
13071	LHX4	ISL1	[activation]
13072	BARD1	NOL9	[activation]

13073	PTPN6	BTLA	[activation;inhibition]
13074	ITGB4	LPAR1	[activation]
13075	CSMD1	BCL6	[activation]
13076	ASAP2	PIK3R1	[activation]
13077	LGR4	DSCAM	[activation]
13078	TXNDC17	OXT	[activation]
13079	RET	GFRA1	[activation;inhibition]
13080	SIRT1	TSC2	[activation;inhibition]
13081	GRB2	SP1	[activation]
13082	HSPB1	AGAP5	[activation]
13083	MPP3	MCM2	[activation]
13084	AURKA	SCGB3A1	[activation]
13085	ARHGEF12	LPAR2	[activation]
13086	NOS3	NOSIP	[activation]
13087	OXSR1	APP	[activation]
13088	PARD3	PARD6B	[activation]
13089	TAF1B	THAP7	[activation]
13090	GNAS	CCDC8	[activation]
13091	FSCN1	PRKCA	[activation]
13092	EGFR	RPS15	[activation]
13093	AURKB	SNW1	[activation]
13094	ATP13A2	GPR21	[activation]
13095	TNFRSF12A	TNFSF12	[activation]
13096	IFNA8	APP	[activation]
13097	IL13RA2	AKT1	[activation]
13098	HSPA4L	TIMP2	[activation]
13099	BAMBI	SEP15	[inhibition]
13100	ERBB2IP	ACVR2B	[inhibition]
13101	MDM4	YWHAH	[activation;inhibition]
13102	OBSL1	CKAP4	[inhibition]
13103	GH1	PPP2CB	[inhibition]
13104	ICAM1	RPL8	[activation]
13105	VRK3	DSG1	[activation]
13106	TP53	CXXC1	[activation]
13107	ETS1	PPME1	[activation]
13108	AKAP1	MDM2	[activation]
13109	MCC	CYCS	[inhibition]
13110	MYC	ARHGEF2	[activation]
13111	BCL2L11	FBXW11	[activation]
13112	POLE	LRRK2	[activation]
13113	SIRT1	RAD50	[activation]
13114	BAG3	MAPK11	[activation]
13115	H1F0	PRKCB	[activation]
13116	MAPK10	STMN2	[activation]
13117	NFKBIA	PRKCI	[activation]
13118	MEGF6	CRKL	[activation]
13119	BIRC3	DAB2IP	[activation]
13120	PXN	GRB2	[activation]
13121	ABL1	SOS2	[activation]
13122	LRRK2	EPRS	[activation]
13123	OBSL1	SMARCA4	[activation]
13124	CDH6	CDH9	[activation]
13125	GMFB	PRKACA	[activation]
13126	PPM1A	DVL1	[activation]
13127	MAP4K3	ITSN2	[activation]
13128	PXN	PTEN	[activation]
13129	HIST1H3A	PRMT1	[activation]
13130	RFWD2	PTPN1	[activation]
13131	CNBP	MAPK14	[activation]
13132	ATF2	GAPDH	[activation]
13133	GPSM2	GLIS2	[inhibition]
13134	MTOR	PRKDC	[activation]
13135	CDK1	TP53BP1	[activation]
13136	RB1	LIN9	[inhibition]
13137	CDK1	EGFR	[activation]
13138	FOXM1	SMAD3	[inhibition]
13139	PVR	SLC30A2	[activation]
13140	APPL1	CMTM4	[activation]
13141	PIK3R2	ACACA	[activation;inhibition]
13142	ARF6	DHX9	[activation]
13143	POLR2A	ERCC4	[activation]
13144	STX17	VTI1B	[activation]
13145	CREBBP	NFE2	[activation]
13146	TYK2	HSP90AB1	[activation]
13147	MSH5	LRRK2	[activation]
13148	APP	LIMS2	[activation]

13149	MAPK1	PACSIN3	[activation]
13150	NUMB	RALBP1	[activation]
13151	TP53	HIPK1	[activation]
13152	CD2	CD2AP	[activation]
13153	IRF7	TNFAIP3	[activation]
13154	MYC	ACTG1	[activation]
13155	BTRC	BCL2L11	[activation]
13156	GABPA	EP300	[activation]
13157	PTPN6	SIGLEC10	[activation;inhibition]
13158	CTBP2	CCR5	[activation]
13159	RFC4	BRD4	[activation]
13160	DOCK8	RAC1	[activation]
13161	MAP3K2	BIRC2	[activation]
13162	MARK3	HDAC4	[activation]
13163	ITGA2	ACTA1	[activation]
13164	MYC	ERC1	[activation]
13165	ENG	TGFBR1	[inhibition]
13166	ARRB1	GSN	[activation]
13167	EIF2AK2	IFRD1	[activation]
13168	GABARAPL1	PIK3CG	[activation]
13169	YWHAZ	ZNF839	[activation]
13170	LRPAP1	ETV7	[inhibition]
13171	FAM168A	UBE2V1	[activation]
13172	PRKCI	BAD	[activation]
13173	E2F3	MYO1B	[activation]
13174	CRMP1	RGL2	[activation]
13175	TRH	TRHR	[activation]
13176	PTP4A3	RAB3GAP2	[activation;inhibition]
13177	SHC1	ITGB3	[activation]
13178	NBN	CASC3	[activation]
13179	SRPK2	RWDD2B	[activation]
13180	EGFR	UBA2	[activation]
13181	PLK1	GET4	[activation]
13182	PLK1	VRK3	[activation]
13183	HIF1A	MAX	[inhibition]
13184	GRB2	CLU	[activation]
13185	USP19	CDK1	[inhibition]
13186	CBLB	TNFRSF11A	[activation]
13187	UBE2E3	RNF150	[activation]
13188	JAG2	GFI1B	[activation]
13189	G3BP1	FYN	[activation]
13190	TNFAIP3	CASP8	[activation]
13191	IKBKB	STAT3	[activation]
13192	SETDB1	GUSBP1	[activation]
13193	BLK	CD79A	[activation]
13194	ADCY6	GNAS	[activation]
13195	SFN	CHEK1	[activation]
13196	TGFA	SNTA1	[activation]
13197	CCL5	RELA	[activation]
13198	GRB2	CHRD	[inhibition]
13199	STK4	GAPDH	[activation]
13200	KPNA2	ITK	[activation]
13201	ITGB3	PDGFRA	[activation]
13202	TAB1	PPP1R7	[inhibition]
13203	H3F3A	IGSF8	[activation]
13204	GRB2	ODR4	[activation]
13205	KPNA3	HSPB1	[activation]
13206	NR3C1	CAV1	[activation]
13207	STAT5A	CD247	[activation]
13208	RPS6KA2	PRKCA	[activation]
13209	EIF4G2	EIF3A	[activation]
13210	AKAP13	RANBP2	[activation]
13211	PTK2	EPHA2	[activation]
13212	PTPN6	SSTR2	[activation;inhibition]
13213	PRMT5	ESR2	[activation]
13214	ICT1	MRPS2	[activation]
13215	MYL12A	ITGA4	[activation]
13216	CRKL	CD34	[activation]
13217	CSNK2A2	PRNP	[activation]
13218	CXCL1	XRCC3	[activation]
13219	TBXA2R	GRK6	[activation;inhibition]
13220	TIMP1	EEF1B2	[activation]
13221	PRKACA	GUSB	[activation]
13222	EP300	TBP	[activation]
13223	PIN1	ERBB2	[activation]
13224	RPA2	BLM	[activation]

13225	SERPINE1	KLK2	[inhibition]
13226	RRP1B	MAPK14	[activation]
13227	RAD50	ATRX	[activation]
13228	POU3F2	PAX3	[activation]
13229	OPTN	TNF	[activation]
13230	PTPN1	ESR1	[activation]
13231	KPNB1	NLK	[inhibition]
13232	VDAC2	CYCS	[inhibition]
13233	ZNF804A	SPP1	[activation]
13234	BCL2L11	BCL2	[activation]
13235	MAPK4	ACSL3	[activation;inhibition]
13236	ERF	EPHB2	[activation]
13237	PAK2	ABI3	[activation]
13238	BUB1B	AP4B1	[inhibition]
13239	GABARAP	HNRNPA0	[activation]
13240	TTC17	BMPR1B	[activation;inhibition]
13241	C1QC	PTX3	[activation]
13242	ACTB	GSN	[activation]
13243	PTK2B	SORBS1	[activation]
13244	NLGN1	DLG4	[activation]
13245	HSP90AB1	ALPK1	[activation]
13246	ETS1	SRSF11	[activation]
13247	APP	IGSF10	[activation]
13248	PDZRN3	EFNB2	[activation]
13249	BCL6	LIMS3L	[activation]
13250	NTM	LSAMP	[inhibition]
13251	RGS19	PRKCA	[activation;inhibition]
13252	CRCT1	PTN	[activation]
13253	GNB5	GNG13	[activation]
13254	PRKACB	HSP90AA1	[activation]
13255	GSK3A	PRKACA	[activation;inhibition]
13256	YWHAE	ZNF839	[activation]
13257	SNAP29	ITSN2	[activation]
13258	APP	SOCS5	[inhibition]
13259	FBXL5	BTG1	[activation]
13260	EFNB2	PICK1	[activation]
13261	EEF1D	GRB2	[activation]
13262	ISL1	ZNF511	[activation]
13263	PRMT1	HNRNPA1	[activation]
13264	DLG1	PAX6	[activation]
13265	NOS3	TXNDC11	[activation]
13266	NFKB1	PPARGC1A	[activation]
13267	GABBR2	ATF4	[activation]
13268	HSPB1	SPATA7	[inhibition]
13269	PHYHIP	NFE2	[activation]
13270	PCF11	WHSC1	[activation]
13271	DIXDC1	AXIN1	[activation]
13272	NOTCH1	EGFL7	[activation]
13273	IDE	AR	[activation]
13274	INPP4A	CAPN1	[activation]
13275	ARF6	GDI2	[activation]
13276	HOXC10	NXF1	[activation]
13277	PPP1CA	CDK4	[inhibition]
13278	PRNP	CIRBP	[activation]
13279	GLTPD1	PHF13	[activation]
13280	STAT4	JUN	[activation]
13281	SMAD1	ERBB2IP	[inhibition]
13282	ACTR2	TRAF6	[activation]
13283	CSNK2A1	RPS6KB1	[activation]
13284	PXN	CSK	[activation]
13285	ACVR1	PLEKHJ1	[activation;inhibition]
13286	SMAD2	HGS	[activation]
13287	KIT	LCK	[activation]
13288	SRSF10	MAP1LC3A	[activation]
13289	MAP3K1	SLC25A6	[activation]
13290	VCAM1	GNB1L	[activation]
13291	CYLD	CEP350	[inhibition]
13292	MAP2K2	NFE2L2	[activation]
13293	PDE10A	CASP3	[activation]
13294	SCARB2	PLCG1	[activation]
13295	ATR	IKBKG	[activation]
13296	CCNO	HSPB1	[activation]
13297	CDK1	HIST1H2AB	[activation]
13298	CBL	MST1R	[activation]
13299	ITGA4	YWHAE	[activation]
13300	YWHAZ	ADRA2C	[activation]

13301	BMPR1A	BMPR2	[activation]
13302	WWTR1	YWHAE	[activation]
13303	DDR2	CDH2	[activation]
13304	KEL	EDN1	[activation]
13305	TP53	ZWINT	[activation]
13306	BMP7	KRTAP10-7	[activation]
13307	RPA2	RAB2A	[activation]
13308	SH2D1A	ARHGEF6	[activation]
13309	COPB2	UBA5	[activation]
13310	NXF2	NUP214	[activation]
13311	FAM84B	XRCC3	[activation]
13312	PTK6	EGFR	[activation]
13313	LYN	HBS1L	[activation;inhibition]
13314	APP	IGF1R	[activation]
13315	HSPB1	ATXN10	[activation]
13316	TRIM15	EHMT1	[activation]
13317	DCN	IGF2	[activation]
13318	SKIL	RB1	[activation]
13319	COBL	PACSIN1	[activation]
13320	FLT3	CBL	[activation]
13321	BRD7	TP53	[activation]
13322	GNB2L1	PRKCB	[activation]
13323	RASL10B	FGFR4	[activation]
13324	BTK	PIP4K2B	[activation]
13325	INSR	PRKCA	[activation]
13326	BCL2L2	BAD	[inhibition]
13327	FSTL1	FST	[inhibition]
13328	CTNNB1	SOX17	[activation]
13329	TXN	MYD88	[activation]
13330	MYC	ATP1A1	[activation]
13331	SNRNP35	TNFSF11	[activation]
13332	ADRA1A	NOS1	[activation]
13333	KIR2DS2	TYROBP	[activation]
13334	TTC23L	EXOC5	[inhibition]
13335	VAMP8	STAMBP	[activation]
13336	MET	SOCS1	[inhibition]
13337	CXCL12	ELANE	[activation]
13338	SIGIRR	ENO2	[activation]
13339	GOLGA2	LATS1	[inhibition]
13340	LRRK2	VPS4A	[activation]
13341	CASP3	AKT1	[activation]
13342	PDLIM7	SH2B2	[activation]
13343	RAB3GAP2	SHC1	[activation]
13344	BCL6	PIAS2	[activation]
13345	RPA1	VCAM1	[activation]
13346	HPCAL1	ESR1	[activation]
13347	ATP1A1	FXYD1	[activation]
13348	CASP3	DSG3	[activation]
13349	CDH15	BOC	[activation]
13350	KCNN1	GRB2	[activation]
13351	JUNB	ATF4	[activation]
13352	TNIP1	SELPLG	[activation]
13353	MLF2	DNAJB1	[inhibition]
13354	PECAM1	PTPN6	[activation;inhibition]
13355	OBSL1	ACTN1	[activation]
13356	GADD45B	MAP3K5	[inhibition]
13357	TRAF2	ANKRD36BP1	[activation]
13358	VCAM1	SMARCA5	[activation]
13359	FBXO6	LTBP4	[inhibition]
13360	DKK3	SMAD4	[inhibition]
13361	AURKA	C9orf156	[activation]
13362	MUC12	CDC42	[activation]
13363	TGFB1	ITGB6	[activation]
13364	BMX	FBXO25	[activation]
13365	TP53	CCAR1	[activation]
13366	PTPRJ	FANCC	[activation]
13367	PPP2CA	RPA2	[activation]
13368	STX17	MLH1	[activation]
13369	CYLC2	STK11	[activation]
13370	CTNNB1	NUP98	[activation]
13371	CD44	EGFR	[activation]
13372	PKP3	DSG1	[activation]
13373	MST1R	JAK2	[activation]
13374	RAD9A	RHNO1	[activation]
13375	MAP4K1	CARD11	[activation]
13376	SRPK1	DHX9	[activation]

13377	PTPN6	LILRB4	[activation;inhibition]
13378	RHBDL3	EIF2AK3	[activation]
13379	KIT	SH3KBP1	[activation]
13380	CDK10	ETS2	[activation]
13381	CEBPB	CEBPG	[activation]
13382	TGFB1I1	SDC4	[activation]
13383	DIAPH3	NXF1	[activation]
13384	CD22	SYK	[activation]
13385	ACTB	ERBB2	[activation]
13386	CDK1	CALD1	[activation]
13387	INSR	DOK1	[activation]
13388	AKT1	PRG2	[activation]
13389	ASAP3	CRK	[activation]
13390	GBF1	FBXW11	[inhibition]
13391	EZR	PIK3R1	[activation]
13392	CISH	CSF3R	[inhibition]
13393	HMCN1	RAPGEF2	[activation]
13394	ASB7	FGA	[activation]
13395	RGS20	TBC1D16	[activation;inhibition]
13396	YWHAZ	LUC7L2	[activation]
13397	H3F3A	DNMT3L	[activation]
13398	FLOT2	SELPLG	[activation]
13399	LMTK2	PPP1CA	[activation]
13400	ARFGEF2	PNPLA2	[activation]
13401	EFNB1	PICK1	[activation]
13402	SUMO1	TP53	[activation]
13403	BECN1	WASH1	[activation]
13404	USHBP1	GIT2	[activation]
13405	FBXW7	SHC1	[activation]
13406	HSPA1L	AIRE	[activation]
13407	PRKACA	STMN2	[activation]
13408	TP53BP2	RELA	[activation]
13409	GRB2	SHKBP1	[activation]
13410	CREBBP	ACTA2	[activation]
13411	FGFR3	C6orf47	[activation]
13412	PLEKHJ1	FAM109A	[activation]
13413	PIN1	CDK11A	[activation]
13414	MLH1	ARAF	[activation]
13415	CCDC70	HSPB1	[activation]
13416	TNXB	VEGFB	[activation]
13417	ACTN1	CD81	[activation]
13418	SEMA7A	FYN	[activation]
13419	PLCG1	NCK1	[activation]
13420	CSNK2A1	EIF2S2	[activation]
13421	RGS7	FOS	[activation]
13422	PPP1CA	CAD	[activation;inhibition]
13423	BMPR1B	TSC22D1	[activation;inhibition]
13424	IL1A	IL1RAP	[activation]
13425	ARRB2	PTAFR	[inhibition]
13426	PRKD3	BCL6	[activation]
13427	NCAM1	PRNP	[activation]
13428	MDK	SDC1	[activation]
13429	GRPR	GRP	[activation]
13430	SETD7	SMARCA4	[activation]
13431	CBL	HSPA6	[inhibition]
13432	CEACAM1	CEACAM6	[activation]
13433	PIK3R1	SLC9A2	[activation]
13434	EGFR	TNFRSF1A	[activation]
13435	HEATR3	MYC	[activation]
13436	SP1	RELA	[activation]
13437	SNRK	JUN	[activation]
13438	SMARCA2	CREBBP	[activation]
13439	ESR1	NOP2	[activation]
13440	PRR20E	SELV	[activation;inhibition]
13441	WDFY3	EEF2K	[activation]
13442	CD81	SUPT16H	[activation]
13443	RIN3	RAB5C	[activation]
13444	IGLC1	STK4	[activation]
13445	YWHAE	CDKN1B	[inhibition]
13446	PDCD5	TP53	[activation]
13447	NRIP1	CTBP1	[activation]
13448	PRKCA	ITGB2	[activation]
13449	YTHDF1	NXF1	[activation]
13450	CDKN2B	MDM2	[activation;inhibition]
13451	MYBL2	EP300	[activation]
13452	EIF5B	PAXIP1	[activation]

EPB41L3	SMURF1	[inhibition]
CDK6	PPHLN1	[inhibition]
TRAF3IP1	TRAF3	[activation]
WBSCR22	FOS	[activation]
MAP3K5	HSPA4	[inhibition]
ATG16L1	AGPS	[activation]
GRIA2	CACNG2	[activation]
GRASP	GRM5	[activation]
TRAF2	LRRK2	[activation]
MYO1B	RPA2	[activation]
RPS6KA3	HMGN2	[activation]
RPA2	RAB11B	[activation]
MAP3K8	NFKB2	[activation]
CASP3	PLA2G4B	[activation]
KCNA5	SRC	[activation]
ACTA1	RAC1	[activation]
WNK1	SYT2	[activation]
CCDC8	LYN	[activation]
LY6G5B	TP53	[activation]
TSC22D4	PRNP	[activation]
PIK3R3	ABI1	[activation]
ECSIT	RELA	[activation]
NFATC2	EGR1	[activation]
MSRB3	LNX2	[activation]
PRKACA	PLN	[inhibition]
DACH1	EYA1	[activation]
MYLK	GRB2	[activation]
NCOA3	NR1H2	[activation]
TSSK3	HSP90AA1	[activation]
RAB11FIP1	RAB11B	[activation]
ITGA4	DDX5	[activation]
STX8	STX7	[activation]
PRKACA	C2orf88	[activation]
AFAP1	PRKCZ	[activation]
MYC	SMC4	[activation]
STAT3	TSHR	[activation]
RAF1	JAK2	[activation]
SMAD2	KAT2A	[activation]
STAT3	ADRB2	[activation]
ANK2	TP53	[activation]
MOS	MYOD1	[activation]
PRKDC	SPI1	[inhibition]
GNAO1	PTPRU	[activation;inhibition]
TJP1	CDH5	[activation]
YWHAG	CDK17	[activation]
HSPB2	EIF4E	[inhibition]
DNAJA2	MYC	[inhibition]
LARP1	AURKA	[activation]
EGFR	FBXO6	[activation]
CSK	ARRB1	[activation]
ARHGEF7	NCKIPSD	[activation]
RAF1	HSPA4	[activation]
CNPY2	TNIK	[activation]
KALRN	RAC1	[activation]
LIN7C	KCNJ4	[activation]
SH2D2A	KIT	[activation]
KLK1	KNG1	[activation]
RNH1	G3BP2	[activation]
MAPK14	CCDC97	[activation]
RPA2	CSNK1A1L	[activation]
NXF1	STK10	[activation]
NRIP1	HNF4A	[activation]
SUMO3	CDKN1A	[inhibition]
CDC27	CDC5L	[activation]
MRFAP1L1	PIK3R2	[activation]
PLD2	PLCG1	[activation]
ATM	NR4A1	[activation]
SOS1	LAT2	[activation]
EPM2A	PPP1R3C	[inhibition]
EIF4G1	GABARAPL2	[activation]
GRB2	SH2D4A	[activation]
YWHAZ	PARD6G	[activation]
CACNA1A	SCP2	[activation]
UBE2W	FANCL	[activation]
HIST1H3A	RPS6KA5	[activation]
ACVR2B	SMAD7	[activation]

13529 EGR1 MDM2 [activation]
13530 MAPK1 TNFRSF1A [activation]
13531 SRRM1 SRPK2 [activation]
13532 LZTR1 EHMT1 [activation]
13533 YAP1 SMAD7 [activation]
13534 CCL25 ACKR4 [activation]
13535 RAB5C OSGEP [activation]
13536 PROC PROCR [activation]
13537 YWHAB KIAA0355 [activation]
13538 TBC1D1 UBE2E2 [activation]
13539 CRKL TENC1 [activation]
13540 UBE2N DTX1 [activation]
13541 PTPRK CSK [inhibition]
13542 BIRC2 RNF181 [activation;inhibition]
13543 TNS3 ERBB2 [activation]
13544 RAD1 RAD17 [activation]
13545 FOS APLP1 [activation]
13546 TP53 TMSB4X [activation]
13547 IL2 FOXK2 [activation;inhibition]
13548 NOTCH1 PPP6R1 [activation]
13549 ARHGAP21 ARF1 [activation]
13550 TRMT10B MEIS1 [activation]
13551 AGBL2 TP53 [activation]
13552 NSMAF VASP [activation]
13553 CREBBP EBF1 [activation]
13554 MACF1 PLXND1 [activation]
13555 CNTNAP1 FYN [activation]
13556 ITGA4 HIST2H3A [activation]
13557 MCC MAP2K2 [activation]
13558 ABL1 FAM127A [activation]
13559 POLR2B ILK [activation]
13560 NOTCH2 NOTCH1 [activation]
13561 ESR1 GADD45A [activation]
13562 RAD50 DCLRE1B [activation]
13563 POLA1 POLE [activation]
13564 LONP1 VCAM1 [activation]
13565 PAXIP1 SRPK1 [activation]
13566 ACTA1 ABL1 [activation]
13567 FLNA CAMK2G [activation]
13568 GRB2 DOCK3 [activation]
13569 TNFRSF8 TRAF1 [activation]
13570 CDK1 MCM4 [activation]
13571 SCG2 ATRIP [activation]
13572 BMPR2 DYNLT1 [activation]
13573 UBE2H RNF186 [activation]
13574 VDAC1 SNCA [inhibition]
13575 FANCA CD79A [activation]
13576 HSP90AB1 MAST2 [activation]
13577 E2F1 TP53BP1 [activation]
13578 NEB APC [inhibition]
13579 HSP90AB1 VCAM1 [activation]
13580 VPS45 PTPN1 [activation]
13581 TIRAP BTK [activation]
13582 MYO1B RIPK3 [activation]
13583 PPIAL4D SGK1 [activation]
13584 SAG RHO [activation;inhibition]
13585 SIK3 YWHAZ [activation]
13586 BTK PRKCQ [activation]
13587 XPO1 CCDC8 [activation]
13588 SORBS1 PILRA [activation]
13589 KCNH2 CAV1 [activation]
13590 MLH1 ZC3H11A [activation]
13591 GRB2 B2M [activation]
13592 TNFRSF10B FAS [inhibition]
13593 GNA13 CXCR5 [activation]
13594 RHOA FAS [activation]
13595 SSH1 CBX1 [activation]
13596 CD79B LCK [activation]
13597 SHC1 HSPA1L [activation]
13598 RAB1A CDKN1A [activation]
13599 AOPEP NRAS [activation]
13600 PPP2CA CFTR [inhibition]
13601 ABL1 DOK3 [activation]
13602 VCAM1 HBG1 [activation]
13603 NR2C1 POLD3 [activation]
13604 MC4R AGRP [activation]

13605	CDC42	LCK	[activation;inhibition]
13606	MAP4K4	PPARG	[activation]
13607	RPL26	ICAM1	[activation]
13608	STAM	LCP2	[activation]
13609	DLAT	PDHA1	[activation]
13610	ANLN	CDC5L	[activation]
13611	MAP3K7	DDX5	[activation]
13612	DAPK1	PPP2R2A	[activation]
13613	CDKN1B	PIN1	[inhibition]
13614	CSNK2A1	SPP1	[activation]
13615	RAB5A	ITGB1	[activation]
13616	GRB2	MAP1LC3A	[activation]
13617	HSP90AB1	FBXL18	[activation]
13618	MYC	PRKAR1B	[activation]
13619	SFN	PARD3	[activation]
13620	CSNK2A2	HMGA2	[activation]
13621	RAD21	NCAPG	[activation]
13622	CYTH2	IRS1	[activation]
13623	TP53BP2	CRK	[activation]
13624	ADAMTSL2	NECAB2	[activation]
13625	FOS	UBE2I	[activation]
13626	BEX1	CDH1	[activation]
13627	PGAM5	PAXIP1	[activation]
13628	TPST2	CCR1	[activation]
13629	MT-CO3	SNCA	[activation]
13630	AURKB	GIGYF2	[activation]
13631	TP53	NOL3	[activation]
13632	ILK	KHK	[activation]
13633	PICK1	PVRL2	[activation]
13634	ERBB4	CRKL	[activation]
13635	ESF1	FOS	[activation]
13636	HIPK4	ATP1A1	[activation]
13637	TP53	XPO1	[activation]
13638	NFKBIB	GIT2	[activation]
13639	ARF1	RAD52	[activation]
13640	HSP90AA1	KSR2	[activation]
13641	MMP13	ADAMTS5	[activation]
13642	PRKACB	CDK5RAP2	[activation]
13643	MMP2	BACE1	[activation]
13644	RPS6KA5	HSPB1	[activation]
13645	CSNK2B	CHEK1	[activation]
13646	YY1	NOTCH1	[activation]
13647	PRKACA	ITPKA	[activation]
13648	MYLK	PRKCA	[activation]
13649	SPP1	TTC1	[activation]
13650	PRKAA2	RFX6	[activation]
13651	CDH3	CTNNA1	[activation]
13652	PAK2	LIMK1	[activation]
13653	SHC1	SOS2	[activation]
13654	HSP90AB1	NTRK3	[activation]
13655	AVPR2	MAPK3	[activation]
13656	ATG5	GABARAPL1	[activation]
13657	CAMK2D	CAMK2B	[activation]
13658	FGF1	SRPK1	[activation]
13659	VPS35	MTNR1B	[activation]
13660	HSP90AB1	ACVR1C	[activation]
13661	C8A	C8B	[activation]
13662	HSPA4	KAT2B	[activation]
13663	EGFR	SNAP25	[activation]
13664	DAB2	PRKCB	[activation]
13665	LPL	APOC2	[activation]
13666	PSEN2	CSNK1D	[activation]
13667	CENPC	PTEN	[activation]
13668	C6	SART3	[activation]
13669	HSPB1	SIM2	[activation]
13670	SLC25A5	PRKAA1	[inhibition]
13671	ITSN2	WAS	[activation]
13672	PRKAA1	BRSK2	[inhibition]
13673	YWHAG	CRTC3	[activation]
13674	HSP90AA1	RPS6KL1	[activation]
13675	AMIGO2	AMIGO1	[activation]
13676	KLK7	TGFB1	[activation]
13677	CAMK2A	HSF1	[activation]
13678	LYN	BLNK	[activation]
13679	RHNO1	LRRK2	[activation]
13680	TRAF6	CDC42	[activation]

13681	BCAR1	YWHAE	[activation]
13682	TTI1	MTOR	[activation]
13683	RPA3	CTTN	[activation]
13684	HSP90AA1	NTRK2	[activation]
13685	KIAA1377	POLD1	[activation]
13686	DNMT1	SETD7	[activation]
13687	ERCC1	ERCC4	[activation]
13688	EIF4G3	HSPB1	[inhibition]
13689	GRB14	EGFR	[activation]
13690	HIPK2	DYRK1A	[activation]
13691	FTL	GRB2	[activation]
13692	AMOTL2	INADL	[activation]
13693	BCAR3	APP	[activation]
13694	TP53	CPNE7	[activation]
13695	ZCCHC10	DAB1	[activation]
13696	AKT2	HIST1H3A	[activation]
13697	GSTK1	VCAM1	[activation]
13698	SRC	FASLG	[activation]
13699	TP53	PRMT1	[activation]
13700	OBSL1	HSPB1	[inhibition]
13701	ILK	IGF2BP1	[activation]
13702	H2AFX	VPS35	[activation]
13703	MCM3	LZTR1	[activation]
13704	RAD21	H2AFJ	[activation]
13705	RAD17	MRE11A	[activation]
13706	RHOBTB2	HSP90AA1	[activation]
13707	CDK5	PSEN1	[activation]
13708	LRRK1	CCL21	[activation]
13709	PPARA	MAPK3	[activation]
13710	FAM65B	PER1	[inhibition]
13711	HBEGF	CD82	[activation]
13712	GLCCI1	DYRK1A	[activation]
13713	IFNAR1	PRKACA	[activation]
13714	RAB14	RPA2	[activation]
13715	CDH9	CDH7	[activation]
13716	MRPL9	EGFR	[activation]
13717	SHC1	HSPA8	[activation]
13718	NFATC1	YWHAZ	[activation]
13719	AURKB	CEP170	[activation]
13720	LARS	ESR2	[activation]
13721	PDC	YWHAZ	[activation]
13722	GNB2	GRB7	[activation]
13723	MT1A	TP53	[activation]
13724	PFDN1	DEFA3	[inhibition]
13725	NXF1	AGRN	[activation]
13726	GABARAP	HSDL2	[activation]
13727	CEBPB	RB1	[activation]
13728	YAP1	HSPA4	[inhibition]
13729	KIAA1377	YWHAZ	[activation]
13730	MDC1	LSM12	[activation]
13731	EP300	ETS1	[activation]
13732	RAB11B	OLFM2	[activation]
13733	AR	EGFR	[activation]
13734	BHLHE40	HDAC1	[activation]
13735	CCNE1	GSK3B	[activation]
13736	MTIF2	CSNK2B	[activation]
13737	ITGB4	PRKCD	[activation]
13738	DNM2	PACSIN2	[activation]
13739	BRCA1	ESR1	[activation]
13740	PTK2	GSK3B	[activation]
13741	KIRREL	SAV1	[activation]
13742	EP300	TP53BP2	[activation]
13743	GRB2	RPL31	[activation]
13744	ITGA4	HBZ	[activation]
13745	HTR1A	S1PR1	[activation]
13746	SRPK2	C4orf46	[activation]
13747	ELAVL3	MAST1	[activation]
13748	CDK5RAP3	RELA	[activation]
13749	CDC23	SUMO3	[activation]
13750	SRPK2	NOP2	[activation]
13751	PSD	GMFG	[activation]
13752	PRKAA1	PRKAB1	[inhibition]
13753	SERPINA5	THBD	[inhibition]
13754	MAP2	APOE	[activation]
13755	FTSJ1	TACO1	[activation]
13756	DIABLO	MDM2	[activation]

13757	TUBB	MAPT	[activation]
13758	PRPF19	NOTCH1	[activation]
13759	PPP2CA	KLHL15	[inhibition]
13760	VCAM1	MACF1	[activation]
13761	SRPK1	EPB41L4A	[activation]
13762	CDKN1B	TSC2	[activation;inhibition]
13763	EPHA4	HSP90AA1	[activation]
13764	ADAM22	NXF1	[activation]
13765	CEP76	PPP3CA	[activation]
13766	ELN	LOX	[activation]
13767	BTG2	HMGN1	[activation]
13768	GPRASP1	OPRL1	[activation]
13769	PLCD1	RALA	[activation]
13770	FANCF	STRA13	[activation]
13771	MLH1	TXN	[activation]
13772	MARCO	SCGB3A1	[activation]
13773	MAX	CASP5	[activation]
13774	SYK	PRKCA	[activation]
13775	INSRR	INSR	[activation]
13776	G3BP2	C3orf17	[activation]
13777	EGFR	VAC14	[activation]
13778	TERT	UPF1	[activation]
13779	PDPK1	ITGB3	[activation]
13780	TUB	PIK3R1	[activation]
13781	TULP3	MYO18B	[activation]
13782	TBP	PIAS1	[inhibition]
13783	CD40	PIK3CA	[activation]
13784	FGFR1	ERBB3	[activation]
13785	SOS1	CRKL	[activation]
13786	CDC5L	MDC1	[activation]
13787	APP	SRPK1	[activation]
13788	MMP7	MBP	[activation]
13789	TRAF6	TRAF7	[activation]
13790	CASP1	CEBPB	[activation]
13791	PSG3	SKIL	[activation]
13792	RIPPLY2	CCDC155	[activation]
13793	BCL6	PPAP2B	[activation]
13794	EGFR	RBM41	[activation]
13795	PEBP1	PAK2	[inhibition]
13796	CDKN1A	SIPA1	[activation]
13797	FANCG	FANCC	[activation]
13798	KAT2A	SMAD5	[activation]
13799	OTX2	ZDHHC17	[activation]
13800	ISG15	STAT1	[activation]
13801	HSP90AA1	SGK2	[activation]
13802	CDK2AP2	MRFAP1L1	[activation]
13803	HSPB11	NUP62	[activation]
13804	TP53	TNK2	[activation]
13805	PIN1	NOTCH1	[activation]
13806	CCNB1	CDKN1B	[activation;inhibition]
13807	ARRB2	YWHAB	[activation]
13808	CD69	S1PR1	[activation]
13809	IRAK4	HIST1H1E	[activation]
13810	CXCL10	DPP8	[activation]
13811	RXRA	CTCF	[inhibition]
13812	CCDC90B	ANXA7	[activation]
13813	SRPK1	SREK1	[activation]
13814	TGFB1I1	MAPK15	[activation]
13815	MYC	ETFA	[activation]
13816	SCUBE3	MMP2	[activation]
13817	CDK11B	ESR1	[activation]
13818	ASAP1	PLCG1	[activation]
13819	NFATC1	UBE2I	[activation]
13820	VASN	TGFB1	[activation]
13821	IL36RN	SSBP4	[activation]
13822	IKBKE	TBK1	[inhibition]
13823	TP53	ING2	[activation]
13824	MAP2K7	DUSP22	[inhibition]
13825	CXCL16	NOTCH2NL	[activation]
13826	ITSN2	MEGF10	[activation]
13827	SFN	MAP3K5	[activation]
13828	FYN	C6	[activation]
13829	SETD7	PRKAR1A	[activation]
13830	SIVA1	ABL2	[activation]
13831	GRB2	PSTPIP2	[activation]
13832	NDP	TSPAN12	[activation]

13833	HSP90AB1	TBK1	[activation]
13834	EP300	JMY	[activation]
13835	NUP62	CRCT1	[activation]
13836	RANBP2	CCDC8	[activation]
13837	MAPK8	SPI1	[inhibition]
13838	BCL10	AKT1	[activation]
13839	EPB41	PRKCB	[activation]
13840	RAD50	RINT1	[activation]
13841	TAB1	IRAK1	[activation;inhibition]
13842	APC	CAPZA2	[inhibition]
13843	CCM2	SRPK2	[activation]
13844	FAM222B	NLK	[inhibition]
13845	IRAK2	IRAK1	[activation;inhibition]
13846	STAT5B	SERTAD1	[activation]
13847	GABARAPL2	HIST1H4A	[activation]
13848	ITGB3	GULP1	[activation]
13849	PGRMC1	TIMP2	[activation]
13850	AURKB	PPM1B	[activation]
13851	CD4	MAPK14	[activation]
13852	DLL1	PSEN1	[activation]
13853	TUBB	CHUK	[activation]
13854	PTK7	CTNNB1	[activation]
13855	DAB1	KRTAP8-1	[activation]
13856	TNIK	DSTN	[activation]
13857	NRAS	C9orf156	[activation]
13858	PPP2CA	MAPT	[activation]
13859	CYBRD1	STX4	[activation]
13860	CDK11B	CASP8	[activation]
13861	CDK2	UBTF	[activation]
13862	UBTF	PIK3CA	[activation]
13863	IKBKE	DNAJB1	[inhibition]
13864	RLN2	RXFP1	[activation]
13865	ARPC4	UBE2Q2	[activation]
13866	FZR1	CDC23	[activation]
13867	KPNA1	CRADD	[activation]
13868	GCN1L1	TNFRSF1A	[activation]
13869	RAE1	DDX24	[activation]
13870	BAZ2A	PYHIN1	[activation]
13871	CSK	INSR	[activation]
13872	C1orf94	DAB1	[activation]
13873	CAPN2	BID	[activation]
13874	PIK3R2	PIK3R3	[activation]
13875	SDC2	CSF2	[activation]
13876	CDK16	YWHAZ	[activation]
13877	SEMA4C	DLG2	[activation]
13878	NFYA	BRCA1	[activation]
13879	HSPB1	MAGED1	[activation]
13880	ARHGEF12	ABCA1	[activation]
13881	ESR2	TXNRD1	[activation]
13882	CDC5L	CHEK1	[activation]
13883	BAZ1B	SMARCA4	[activation]
13884	SSBP1	GRB2	[activation]
13885	CCNB1	TSC1	[activation;inhibition]
13886	SRPK2	WDR5	[activation]
13887	CHUK	MAP3K14	[activation]
13888	TNFSF13	TNFRSF1A	[activation]
13889	ILK	ZNF205	[activation]
13890	TNIK	SLC9A1	[activation]
13891	EEF2	IRAK1	[activation;inhibition]
13892	ZMAT2	AMH	[activation]
13893	ETV6	CRKL	[activation]
13894	MAPK7	ZBED6CL	[activation]
13895	RANBP2	STK4	[activation]
13896	ANAPC2	EP300	[activation]
13897	PPA1	RB1	[activation]
13898	PRKCE	ATF2	[activation]
13899	CD81	SHC1	[activation]
13900	RASA1	PDE6D	[activation]
13901	H2AFX	BARD1	[activation]
13902	EGFR	ATP2B1	[activation]
13903	RHOA	MARK2	[activation]
13904	DVL1P1	PTK2	[activation]
13905	AKT3	PLEKHO1	[activation]
13906	LRIF1	RAP1B	[activation]
13907	ARHGAP31	ITSN1	[activation]
13908	GYG2	GYS1	[inhibition]

13909	PFN1	VASP	[activation]
13910	CBLB	ABI1	[inhibition]
13911	AURKA	DAB2	[activation]
13912	PPIG	FOS	[activation]
13913	ADRA1B	STAT1	[activation]
13914	ABCF2	EGFR	[activation]
13915	STAT5A	CCR5	[activation]
13916	MYC	IL1B	[activation]
13917	BUB1	TMEFF1	[activation]
13918	FAM124A	SRPK1	[activation]
13919	ROCK1	CRMP1	[activation]
13920	MYC	AHNAK	[activation]
13921	PAXIP1	G3BP1	[activation]
13922	CDKN1A	CPNE6	[activation;inhibition]
13923	PIK3R2	AR	[activation]
13924	PRKDC	NOS2	[activation]
13925	SMAD1	KAT2B	[activation]
13926	FYN	TNK2	[activation]
13927	JUN	PPP2R2B	[activation]
13928	EGFR	PPP2CB	[activation]
13929	NXF1	HEATR2	[activation]
13930	ITGA4	CSNK2B	[activation]
13931	ANK1	KCTD6	[activation]
13932	IKBKB	CDC40	[activation]
13933	TNFSF11	NFKB1	[activation]
13934	GADD45G	PPARA	[activation]
13935	FZR1	CLSPN	[activation]
13936	EGFR	PTPRB	[activation]
13937	CHGB	TWF2	[activation]
13938	PSEN1	GSK3B	[inhibition]
13939	PTCD1	ICT1	[activation]
13940	HIF1A	MTA1	[activation]
13941	SRPK3	MAPK12	[activation]
13942	NCK1	BCAR1	[activation]
13943	EZH2	ATF2	[activation]
13944	CUL3	CCND1	[inhibition]
13945	MAP4K4	DOK1	[activation]
13946	GNAI3	RIPK3	[inhibition]
13947	MLH1	ACTG1	[activation]
13948	MCM3	CDC5L	[activation]
13949	ITGAL	PTPRC	[activation]
13950	VCAM1	GRB2	[activation]
13951	IL1RAP	CCNI	[activation]
13952	RPS6KA2	L1CAM	[activation]
13953	PRKCA	GRM5	[activation]
13954	RPA2	CLSPN	[activation]
13955	KIF5A	RAPGEF2	[activation]
13956	FYN	FOXF2	[activation;inhibition]
13957	JUN	EPAS1	[activation]
13958	ARNTL	CREBBP	[activation]
13959	FKBP1A	ACVR1B	[inhibition]
13960	POLA1	HELB	[activation]
13961	HOXA1	PRNP	[activation]
13962	NXF1	TNFAIP3	[activation]
13963	OGT	NFATC1	[activation]
13964	NXF1	AASDHPPT	[activation]
13965	CIT	RHOC	[activation]
13966	ANXA7	PAX8	[activation]
13967	MEN1	IQGAP1	[activation]
13968	FCER1A	SYK	[activation]
13969	GRB2	LAT2	[activation]
13970	LRRK2	MYL12B	[activation]
13971	ANAPC16	CDC26	[activation]
13972	YWHAZ	KIAA0930	[activation]
13973	WASF1	GRB2	[activation]
13974	PPP2R5C	MAPK1	[activation]
13975	MDM2	JAK1	[activation]
13976	ERBB2	TLN1	[activation]
13977	SRC	CBLB	[activation]
13978	NUP160	LRRK2	[activation]
13979	KCNA4	KCNA3	[activation]
13980	BMP15	GDF9	[activation]
13981	RAD17	POLE2	[activation]
13982	GPR45	GRB2	[activation]
13983	APC	EIF3A	[activation]
13984	ENPEP	CCK	[activation]

13985 MYC MAST4 [activation]
13986 HSPA1A BAG1 [inhibition]
13987 PRMT1 NCOA2 [activation]
13988 FXYD3 NR4A1 [inhibition]
13989 FLI1 PIAS2 [inhibition]
13990 CYCS GH1 [inhibition]
13991 HAX1 IL1A [activation]
13992 GABARAPL2 YARS [activation]
13993 NEK1 MRE11A [activation]
13994 AKT2 ARRB2 [inhibition]
13995 NGFR MAG [activation;inhibition]
13996 RHOA NCL [activation]
13997 DNAJC12 APP [inhibition]
13998 MTA2 EHMT2 [activation]
13999 LMNA LRRK2 [activation]
14000 CREBBP ESR2 [activation]
14001 PRPSAP1 PTP4A3 [inhibition]
14002 HSPA8 RIPK3 [inhibition]
14003 TP53 CHEK2 [activation]
14004 GTDC1 APP [activation]
14005 PTPN6 PILRB [activation;inhibition]
14006 RAB4A TP53 [activation]
14007 RPA2 SMC4 [activation]
14008 SMO ARRB1 [inhibition]
14009 RPL10 GRB2 [activation]
14010 ACTB HMMR [activation]
14011 GEM CLDN15 [activation]
14012 TRAF1 CASP3 [activation]
14013 APEX1 HMGA2 [activation]
14014 MTNR1B GNB1 [activation]
14015 BCL2L1 BNIP3L [activation]
14016 CD2BP2 EIF4A2 [activation]
14017 HIST1H2BM H2AFX [activation]
14018 VCAM1 RAB10 [activation]
14019 RPS6KA4 SH3KBP1 [activation]
14020 TGFBR1 BMPR1B [activation;inhibition]
14021 YTHDF1 ITSN2 [activation]
14022 OR5A1 SRPK2 [activation]
14023 NOTCH2 IL13RA2 [activation]
14024 STK4 CTNNB1 [activation]
14025 CAMK2D EIF4B [activation]
14026 ITGA4 GSTK1 [activation]
14027 UBE2I CAPN2 [activation]
14028 ADRB2 ITCH [activation]
14029 SRPK2 RPL39L [activation]
14030 NXF1 EFNA4 [activation]
14031 PRKRA SNX5 [activation]
14032 POMGNT1 IL8 [activation]
14033 CYB5B APP [activation]
14034 BCL6 TRAF3IP3 [activation]
14035 RABEP1 EEA1 [activation]
14036 CXCL14 TEX11 [activation]
14037 GNB5 GNGT1 [activation]
14038 ERH TP53 [activation]
14039 MYC HIST1H2BM [activation]
14040 ACACA PHKG2 [activation;inhibition]
14041 RAN AR [activation]
14042 ELANE SDC1 [activation]
14043 BRCA2 TP53 [activation]
14044 STK11 D2HGDH [activation]
14045 FYN PLAUR [activation]
14046 CD2 CD247 [activation]
14047 SKIL TFPI2 [inhibition]
14048 SRPK1 HDGF [activation]
14049 CREBBP CDH2 [activation]
14050 YAP1 CTNNA1 [activation]
14051 KLK6 CSN1S1 [activation]
14052 NCK1 FYB [activation]
14053 CUL4B ENO2 [activation]
14054 DDX5 TYK2 [activation]
14055 SMURF1 CMBL [inhibition]
14056 LIF LIFR [activation]
14057 GPRIN2 YWHAE [activation]
14058 ABL1 SHB [activation]
14059 MMP7 SERPINA1 [inhibition]
14060 SDC4 MDK [activation]

14061	ATF2	BACH1	[activation]
14062	NFATC1	KAT2B	[activation]
14063	MAP2K4	MAP3K14	[activation]
14064	NOTCH2NL	PID1	[activation]
14065	OBSL1	DDX39B	[activation]
14066	BUB1	MSANTD3	[activation]
14067	RNF11	RIPK1	[activation]
14068	IGFBP5	F2	[inhibition]
14069	MAP2K1	CDKN2A	[activation]
14070	IFNAR1	PRMT1	[activation]
14071	DLX2	GRN	[activation]
14072	CREBBP	HNF4A	[activation]
14073	GNPTG	SRPK1	[activation]
14074	ESR2	CTNNB1	[activation]
14075	MSTN	FURIN	[activation]
14076	FOSL1	ATF3	[activation]
14077	KCNE1	KCNQ1	[activation]
14078	CARM1	SRC	[activation]
14079	ESR1	SKIL	[activation]
14080	LRP1	C3	[activation]
14081	PIAS1	CREBBP	[activation]
14082	MLH1	MAP2K6	[activation]
14083	SERF1A	SRPK2	[activation]
14084	NOTCH2NL	ICAM4	[activation]
14085	DNAJB5	SMAD3	[inhibition]
14086	CDKN1B	ABL1	[activation;inhibition]
14087	NABP2	EP300	[activation]
14088	NOTCH1	CDC27	[activation]
14089	MC4R	ASIP	[inhibition]
14090	CAV1	BMX	[activation]
14091	MTOR	GNB2	[activation]
14092	RGS20	GLRX3	[activation;inhibition]
14093	CDKN1A	RNF126	[activation;inhibition]
14094	HABP4	TP53	[activation]
14095	YWHAZ	EIF4A1	[activation]
14096	EPHA8	AK4	[activation]
14097	FASLG	KALRN	[activation]
14098	CCDC8	RAB5C	[activation]
14099	CDK2AP1	CDK2	[activation]
14100	RAC1	MAP3K11	[activation]
14101	FER	CDC37	[activation;inhibition]
14102	ACTB	EIF4A3	[activation]
14103	ARPC3	OBSL1	[activation]
14104	SRC	FGFR1	[activation]
14105	IRS1	ERBB2	[activation]
14106	ETS1	GATA3	[activation]
14107	ELF2	RUNX1	[activation]
14108	BCAP31	ATF2	[activation]
14109	NME3	PHLDA3	[activation]
14110	CTBP2	PLCB1	[activation]
14111	CTGF	TGFB2	[activation]
14112	BCL2A1	BAX	[inhibition]
14113	SEMA4C	PNMA1	[activation]
14114	CDK1	TOP2A	[activation]
14115	EGFR	GRB7	[activation]
14116	RXRA	PIK3R1	[inhibition]
14117	BTK	GP6	[activation]
14118	TP53	FBXO4	[activation]
14119	GRB2	ACTG2	[activation]
14120	STAP1	TEC	[activation]
14121	PTCH1	CCNB1	[activation]
14122	IL2RB	PTPN6	[activation;inhibition]
14123	FGFR2	LRRK1	[activation]
14124	BTBD6	PAXIP1	[activation]
14125	ACTR3	ACTB	[activation]
14126	E2F1	EP300	[activation]
14127	MYC	PALD1	[activation]
14128	CREG1	RB1	[inhibition]
14129	KCNK18	TGFBR1	[activation]
14130	RGL4	RALA	[activation]
14131	SIAH1	INHA	[activation]
14132	RPA3	EIF2S2	[activation]
14133	MDM2	MED1	[activation]
14134	IL6	PTHLH	[activation;inhibition]
14135	PTPRB	NTRK1	[activation]
14136	COL4A1	MAG	[activation]

14137	GABARAP	HSP90AB1	[activation]
14138	NCKAP1	GAS7	[inhibition]
14139	RPS6KA5	YWHAZ	[activation]
14140	ASPSCR1	NOTCH2NL	[activation]
14141	CCND1	BRINP1	[inhibition]
14142	WNK1	CGNL1	[activation]
14143	CAMK2A	ACTN4	[activation]
14144	HSPA1L	TNFRSF1A	[activation]
14145	TADA2A	TP53	[activation]
14146	RIPK1	MAP3K3	[activation]
14147	CREB1	TP53	[activation]
14148	MFN1	MAVS	[activation]
14149	LINC00341	LUC7L2	[activation]
14150	STAT3	STAT5B	[activation]
14151	ADAM10	SUMO1	[activation]
14152	ITGA4	GCN1L1	[activation]
14153	DVL2	ITCH	[activation]
14154	LRRK2	TTK	[activation]
14155	IL7R	EIF2AK2	[inhibition]
14156	SFN	PPP1R3D	[activation]
14157	PAK1	SHC1	[activation]
14158	YWHAB	LRFN4	[activation]
14159	PPP2R5A	TLX1	[activation]
14160	YWHAG	MLLT4	[activation]
14161	TRAF6	KIAA1551	[activation]
14162	RACGAP1	MAP3K4	[activation]
14163	FAM83G	SMAD3	[inhibition]
14164	FKBP5	ILK	[activation]
14165	THRSP	PALB2	[activation]
14166	GIPC1	NTRK2	[activation]
14167	SMAD1	RAB6B	[activation]
14168	MAP4K3	GRB2	[activation]
14169	IL1RL1	IRAK4	[activation]
14170	CBL	RET	[activation;inhibition]
14171	ACKR4	CDK2	[activation]
14172	WAS	NCKIPSD	[activation]
14173	IGSF8	CFL1	[activation;inhibition]
14174	PLG	MMP7	[activation]
14175	PPAP2C	NOTCH2NL	[activation]
14176	SRC	SRCIN1	[activation]
14177	AGGF1	TNFSF12	[activation]
14178	RAD17	USP20	[activation]
14179	LATS1	ZYX	[inhibition]
14180	HSP90AB1	OBSL1	[activation]
14181	TGFBR1	ACVR1	[activation;inhibition]
14182	UBE2W	RNF165	[activation]
14183	MDM2	CLSTN1	[activation]
14184	E2F3	CTNNB1	[activation;inhibition]
14185	BCL3	HDAC1	[activation]
14186	PTP4A3	CCAR2	[inhibition]
14187	TP53	WDR33	[activation]
14188	RDX	TSC1	[inhibition]
14189	TRAF6	JAK2	[activation]
14190	RPS9	ICAM1	[activation]
14191	TRIP10	FASLG	[activation]
14192	TRAF3	SEC16A	[activation]
14193	PPP3CA	PHLPP2	[activation]
14194	SRSF3	SRPK3	[activation]
14195	PPFIA2	PPP2CA	[inhibition]
14196	CD247	HSP90AB1	[activation]
14197	EP300	CASK	[activation]
14198	LIN37	PDAP1	[inhibition]
14199	RPA3	RPA2	[activation]
14200	CDKN2B	TGFB1I1	[activation]
14201	SGTA	TGFA	[activation]
14202	KCNE4	PRKCA	[activation]
14203	PIK3R1	ARHGAP1	[activation]
14204	STAT3	RPS6KA5	[activation]
14205	RAC1	SUMO1	[activation]
14206	RPP14	A2M	[inhibition]
14207	TBXA2R	PRDX4	[inhibition]
14208	EGFR	SSR1	[activation]
14209	TDGF1	COPS6	[activation]
14210	ERCC6L	RPA3	[activation]
14211	RIOK3	CALM1	[activation]
14212	TFAP2B	CITED2	[activation]

14213 EHMT2 SETDB1 [activation]
14214 HSPA8 TCAP [inhibition]
14215 LRRK2 RPL14 [activation]
14216 MAP4K1 PPP4C [inhibition]
14217 CASP3 GOLGA3 [activation]
14218 INSIG2 HSPA8 [activation]
14219 TACC2 KAT2B [activation]
14220 PTPRG LCK [activation;inhibition]
14221 FURIN VWF [activation]
14222 CDC42SE2 CDC42 [activation]
14223 PYCARD INPP4B [inhibition]
14224 PRKCA PRKCZ [activation]
14225 SMG1 EIF4A2 [activation]
14226 ERCC4 RAD52 [activation]
14227 SMARCA4 MYC [activation]
14228 PTPRG EPOR [activation]
14229 NRIP1 RXRA [inhibition]
14230 MYC SORBS1 [activation]
14231 BIRC3 IRF1 [activation]
14232 CASP8 RB1 [inhibition]
14233 NBN PARP1 [activation]
14234 TNIP2 MAPK1 [activation]
14235 SYNCRIP HMGA2 [activation]
14236 FZR1 BUB1B [inhibition]
14237 BAD RPS6KA2 [inhibition]
14238 GATA1 HDAC4 [activation]
14239 SMARCA1 E2F3 [activation]
14240 TP53 ASPM [activation]
14241 ADRB2 MDM2 [activation]
14242 SMAD2 NOTCH4 [activation]
14243 DVL3 LY6H [activation]
14244 NFATC1 SUMO1 [activation]
14245 PRKCD ADCY7 [activation]
14246 ARHGEF10L ANXA9 [activation]
14247 MDM2 DAPK1 [activation]
14248 HSPA8 PROP1 [inhibition]
14249 LATS1 NUAK1 [inhibition]
14250 GNAI3 TRAF6 [activation]
14251 UBA5 ALDH2 [activation]
14252 FOS TAF1 [activation]
14253 ELAVL1 YPEL5 [activation]
14254 JUN HNRNPM [activation]
14255 RRAD PRKCA [activation]
14256 MET TEC [activation]
14257 STK4 SPATA5L1 [activation]
14258 ACVR2B SMAD2 [activation]
14259 GSK3B NFKB1 [activation;inhibition]
14260 VAV2 MARCH7 [activation]
14261 AR WHSC1 [activation]
14262 GCN1L1 SNW1 [activation]
14263 HIST1H2BN ICAM1 [activation]
14264 RRAS2 ARAF [activation]
14265 HDAC4 STAT1 [activation]
14266 ATP1B3 PAXIP1 [activation]
14267 LGALS1 CD2 [activation]
14268 FGFR2 FGF3 [activation]
14269 PLK1 APP [activation]
14270 TRAF2 SOX9 [activation]
14271 RAP2B RAPGEF5 [activation]
14272 PPM1B NOS2 [activation]
14273 TGFB1I1 CSK [activation]
14274 ARFIP2 RAC2 [activation]
14275 C3 CTSG [activation]
14276 RGS17 LCE3C [activation;inhibition]
14277 ITK SOCS1 [inhibition]
14278 ENO1 MAP3K7 [activation]
14279 SGK1 SF3B2 [activation]
14280 WNK2 WNK1 [activation]
14281 EPHB6 MLLT4 [activation]
14282 BNIPL BCL2L1 [activation]
14283 CTBP1 FOXP2 [activation]
14284 PPP3CA MYOZ3 [activation]
14285 GTF2H1 PLCG1 [activation]
14286 VTN GEM [activation]
14287 KCNJ4 PRKCD [activation]
14288 CREBBP TRIM28 [activation]

14289	MPP4	FASLG	[inhibition]
14290	PRKACA	KLF1	[activation]
14291	KCNA4	KCNA2	[activation]
14292	ACTB	SSH3	[activation]
14293	APP	CNTN4	[activation]
14294	CRB3	INADL	[activation]
14295	CTBP1	APC	[activation]
14296	NME1	RAC1	[activation]
14297	IKBKB	DUSP1	[activation]
14298	COX6A2	POT1	[activation]
14299	DAB2IP	MAP3K5	[inhibition]
14300	FOXO1	HOXA10	[activation]
14301	RPS6KA5	CEBPB	[activation]
14302	YWHAZ	VCL	[activation]
14303	CDK5R1	CTNNB1	[activation]
14304	CASP8	BEX1	[activation]
14305	GNA11	CXCR5	[activation]
14306	ABL1	KIT	[activation]
14307	CDKN2C	PPP2CA	[inhibition]
14308	RAC1	METAP2	[activation]
14309	RASGEF1C	TNK2	[activation]
14310	RAB8A	RPA1	[activation]
14311	RUSC2	EGFR	[activation]
14312	NFATC2	KPNB1	[activation]
14313	PDCD11	RELA	[activation]
14314	MSANTD3	PFN2	[activation]
14315	APP	PI4K2A	[activation]
14316	NR0B1	ESR1	[activation]
14317	NFKB1	SLC25A6	[activation]
14318	PIK3R1	RRAS	[activation]
14319	NCOR2	NR1D1	[inhibition]
14320	EIF3A	ETS1	[activation]
14321	SMURF1	SMAD3	[inhibition]
14322	CFTR	BCR	[activation]
14323	JUP	WWTR1	[activation]
14324	PTPRC	JAK1	[activation]
14325	SKP2	NXF1	[activation]
14326	GRB2	HNRNPA2B1	[activation]
14327	E2F1	GTF2H1	[activation]
14328	RASGRF1	PLK1	[activation]
14329	MCM10	CDKN1A	[activation]
14330	PLD2	PIP5K1A	[activation]
14331	HTR1A	HTR1B	[activation]
14332	RAC1	NOXA1	[activation]
14333	NBN	SKP2	[activation]
14334	IGHM	CEP72	[activation]
14335	EPOR	GRAP	[activation]
14336	RRAS2	RAF1	[activation]
14337	FOXP1	LRRK1	[activation]
14338	YWHAE	SLC25A6	[activation]
14339	CDH2	PTPN7	[activation]
14340	SRF	OGT	[activation]
14341	AKT1	FANCA	[activation]
14342	HDAC1	NFE2L2	[activation]
14343	TRAF2	RHEBL1	[activation]
14344	RNF5	INSIG2	[activation]
14345	MEGF10	VWA8	[activation]
14346	RPS8	GRB2	[activation]
14347	RAPGEF2	WWOX	[activation]
14348	BARD1	PCBP2	[activation]
14349	PTK6	KIT	[activation]
14350	NUP62	CCDC53	[activation]
14351	DVL3	NXF1	[activation]
14352	HYOU1	YAP1	[activation]
14353	PTPRC	PPFIBP2	[activation]
14354	PYCARD	RIPK1	[inhibition]
14355	PLG	ANXA2	[activation]
14356	NXF1	FAM212B	[activation]
14357	AGTR1	MAPK1	[activation]
14358	EIF2S3	SNW1	[activation]
14359	PARD3B	SMAD4	[activation]
14360	ILK	STK4	[activation]
14361	MDM2	SFN	[activation]
14362	TRAF2	NGFRAP1	[activation]
14363	TRIM24	PPARG	[activation]
14364	CDK4	CDC6	[activation]

14365 ANAPC2 GRIA1 [activation]
14366 CXCL12 CD4 [activation]
14367 ZNF311 GRB2 [activation]
14368 TRIM43 MYOG [activation]
14369 MAST3 PRKAA2 [inhibition]
14370 WASL PACSIN2 [activation]
14371 PML PIAS2 [inhibition]
14372 HRAS RGL1 [activation]
14373 MAGI3 TGFA [activation]
14374 ANAPC11 CAPN11 [activation]
14375 NFKB1 COMMD3 [activation]
14376 VIM AKT2 [inhibition]
14377 CD9 KIT [activation]
14378 ENG ACVR2A [inhibition]
14379 SFN PPFIBP1 [activation]
14380 RIT1 RGL1 [activation]
14381 GNAO1 GNAI1 [activation;inhibition]
14382 PBX1 MYOD1 [activation]
14383 NFE2L2 SUMO1 [activation]
14384 EIF4EBP1 RHEBL1 [activation]
14385 TPD52 SRPK2 [activation]
14386 CDK4 SUMO3 [inhibition]
14387 HMGB1 NR3C1 [activation]
14388 TP53 GNB2 [activation]
14389 GGCX F9 [activation]
14390 TP53 KHDRBS1 [activation]
14391 CREBBP ETS2 [activation]
14392 LIN52 LIN37 [inhibition]
14393 NCOA1 RXRA [inhibition]
14394 NEK3 VAV1 [activation]
14395 FHL1 PRNP [activation]
14396 ASPSCR1 EZH2 [activation]
14397 SAMD3 C6orf165 [activation]
14398 CCDC102B C20orf195 [activation]
14399 CASP8 TRAF2 [activation]
14400 GRB2 POLR1D [activation]
14401 FOXO3 PPP2R1A [inhibition]
14402 LCK PTPRZ1 [activation;inhibition]
14403 CIT MYL12A [activation]
14404 RHOBTB2 ERBB2 [activation]
14405 TPX2 PIK3R1 [activation]
14406 TNFSF10 TRAF2 [activation]
14407 PIK3CA STAT1 [activation]
14408 NCAM1 NCAN [activation]
14409 BCAR1 EGFR [activation]
14410 FERMT2 CTNNB1 [activation]
14411 PPP6R2 NUDC [activation]
14412 DPYSL2 ROCK1 [activation]
14413 PIN1 RPS6KB1 [activation]
14414 TRIM28 CEBPB [activation]
14415 BRD2 E2F1 [activation]
14416 EFS NPAS2 [activation]
14417 SHC1 MAP4K1 [activation]
14418 ESYT1 MDM2 [activation]
14419 ATR H2AFX [activation]
14420 SRC CTSV [activation]
14421 TBK1 BTRC [inhibition]
14422 FLNA CCNB1 [inhibition]
14423 STRA13 RPA2 [activation]
14424 NUP62 NUP214 [activation]
14425 TOP2A VCAM1 [activation]
14426 PIP5K1C CDK5 [activation]
14427 ERBB3 CRK [activation]
14428 SMAD2 MFN2 [activation]
14429 NMI STAT5A [activation]
14430 JAK1 TEC [activation]
14431 RUNX1 NOTCH1 [activation]
14432 CTCF ZMYM4 [activation]
14433 CDC14A PLK1 [activation]
14434 TNFRSF9 TRAF5 [activation]
14435 SIPA1L3 MYC [activation]
14436 RAB10 PTBP3 [activation]
14437 GSK3B PPP1CC [inhibition]
14438 DLGAP3 FYN [activation]
14439 SNAP29 STX3 [activation]
14440 ISG15 RGS3 [activation;inhibition]

14441	FLNC	PLCG1	[activation]
14442	RYR1	PPP1CA	[activation]
14443	GHR	CRK	[activation]
14444	HDAC1	BAZ2A	[activation]
14445	PIK3R1	ERBB2	[activation]
14446	CRHR2	UCN2	[activation]
14447	SRSF10	WWOX	[activation]
14448	CCDC120	USHBP1	[activation]
14449	HLA-B	TFRC	[activation]
14450	GABARAP	DDX17	[activation]
14451	TRAF6	NR2C2	[activation]
14452	CDC5L	HSP90AB1	[activation]
14453	KAT2A	KAT2B	[activation]
14454	MTPN	CAPZB	[activation]
14455	MEOX2	PKIG	[inhibition]
14456	CDK7	CDK11B	[activation]
14457	CTNNBIP1	USHBP1	[inhibition]
14458	PPARGC1A	SUMO1	[activation]
14459	HOXD10	PBX1	[activation]
14460	EIF4ENIF1	AURKB	[activation]
14461	RHEBL1	SMAD4	[activation]
14462	BMPR2	MAPK8	[activation]
14463	KPNB1	ERBB2	[activation]
14464	CCL5	PHB	[activation]
14465	TNFSF13	FAS	[activation]
14466	RAD21	STAG1	[activation]
14467	ATP9A	COPS6	[activation]
14468	PRKCQ	CBL	[activation]
14469	ACTB	STX4	[activation]
14470	PREPL	NR4A1	[inhibition]
14471	AURKB	EP300	[activation]
14472	CCDC155	FIS1	[activation]
14473	GRB2	DNM2	[activation]
14474	CDH1	CSNK2A1	[activation]
14475	WASL	RHOQ	[activation]
14476	DNAJC5	VAMP7	[activation]
14477	E2F1	PRDM2	[activation]
14478	JAK1	HIST3H3	[activation]
14479	KLRK1	MICB	[activation]
14480	SYK	CD4	[activation]
14481	ETS1	RFWD2	[activation]
14482	MIF	DSTYK	[activation]
14483	ITSN1	PIK3R1	[activation]
14484	NLRP1	PYCARD	[inhibition]
14485	NOXA1	YWHAZ	[activation]
14486	NR1I3	RXRA	[inhibition]
14487	ITPRIP	RASSF1	[inhibition]
14488	PIK3R2	SOS1	[activation]
14489	PRKCZ	RPS6KB1	[activation]
14490	RNF4	SRPK2	[activation]
14491	CENPC	UBTF	[activation]
14492	TSC1	EIF3F	[activation;inhibition]
14493	TULP4	PIK3R1	[activation]
14494	CASP8	KLK5	[inhibition]
14495	YBEY	GEM	[activation]
14496	CDC5L	GRB14	[activation]
14497	HPS6	EP300	[activation]
14498	IRS1	YWHAG	[activation]
14499	PHF20L1	CASP10	[activation]
14500	IRS1	FER	[activation;inhibition]
14501	MIEF2	AGTRAP	[activation]
14502	EPS8L2	CD3E	[activation]
14503	CCR5	CST9L	[activation]
14504	ACVR2A	TCTEX1D4	[activation]
14505	DDX56	RPL26	[activation]
14506	UBE2I	SREBF2	[activation]
14507	SHC1	DUSP23	[activation]
14508	GRN	YY1	[activation]
14509	BANP	NFKB1	[activation]
14510	MEPCE	CDK11B	[activation]
14511	APC	MACF1	[activation]
14512	SMURF1	PPM1A	[inhibition]
14513	ESR1	BARD1	[activation]
14514	LAMTOR4	RRAGA	[activation]
14515	FOS	BTBD10	[activation]
14516	ARHGDIA	BID	[activation]

14517	RND2	PLXNB1	[activation]
14518	CTNNA3	CTNNB1	[activation]
14519	KLHL32	HSP90AB1	[activation]
14520	CEACAM1	ADRB2	[activation]
14521	IRF4	TRAF2	[activation]
14522	ELSPBP1	MYF6	[activation]
14523	RBX1	DAB2IP	[inhibition]
14524	CENPE	MAPK1	[activation]
14525	PTBP1	SRPK2	[activation]
14526	CDH10	PTN	[activation]
14527	EIF4E2	AMOTL2	[activation]
14528	TERT	SMARCC2	[activation]
14529	BCLAF1	BCL2L1	[activation]
14530	RAPGEF2	PRKCI	[activation]
14531	PARP10	CASP6	[activation]
14532	FYN	GAD1	[activation]
14533	EGFR	HIST1H1E	[activation]
14534	CD4	IL16	[activation]
14535	RRN3	NMI	[activation]
14536	TSC1	NGFRAP1	[inhibition]
14537	TSSK3	GP2	[activation]
14538	CCNB1	RPTOR	[activation;inhibition]
14539	LIG3	PLCG1	[activation]
14540	CDC42	CASP7	[activation]
14541	TNFRSF14	P4HB	[activation]
14542	RPA3	EMG1	[activation]
14543	APP	ARPP19	[activation]
14544	PRNP	APOE	[activation]
14545	ATR	TTI1	[activation]
14546	GSN	PTPN12	[activation]
14547	CLIP3	FADD	[activation]
14548	ARPC3	ARPC2	[activation]
14549	CTNNB1	ZFYVE9	[activation]
14550	CDH7	FGF21	[activation]
14551	CCND2	RB1	[activation;inhibition]
14552	IL1RAP	IL1R2	[activation]
14553	CNTNAP1	NCK1	[activation]
14554	TRDMT1	DDX24	[activation]
14555	EFHC2	CYFIP1	[inhibition]
14556	ITGB2	EGFR	[activation]
14557	OLFM2	EIF1	[activation]
14558	UBE2V1	KITLG	[activation]
14559	FOXO1	YWHAG	[activation]
14560	CD79B	ZAP70	[activation]
14561	GNAS	ETS1	[activation]
14562	EGFR	NCAPH2	[activation]
14563	STAU1	SRSF10	[activation]
14564	FAM49B	MAPK6	[activation;inhibition]
14565	NOTCH2	MAML1	[activation]
14566	C1QTNF9	TTN	[activation]
14567	PLCG1	RHOA	[activation]
14568	S1PR5	TP63	[activation]
14569	YWHAZ	CFL1	[activation;inhibition]
14570	ESM1	ITGAL	[activation]
14571	KPNB1	PLCD1	[activation]
14572	TRAF3	MAP2K1	[activation]
14573	MEIS1	HOXD13	[activation]
14574	BMP8B	BMP4	[activation]
14575	FES	RASA3	[activation]
14576	DUSP6	SRPK1	[activation]
14577	FAM96B	PRIM1	[activation]
14578	CTNNA1	MCM7	[activation]
14579	RNF138	LNX2	[activation]
14580	WHSC1L1	BCL7C	[activation]
14581	CHL1	JUN	[activation]
14582	GRB2	RPL24	[activation]
14583	AKT2	TRIB3	[inhibition]
14584	UBTF	TBP	[activation]
14585	SFRP2	SMAD4	[activation;inhibition]
14586	NXF1	HP1BP3	[activation]
14587	UBE2W	USHBP1	[activation]
14588	CSNK1A1	BID	[activation]
14589	NCOA2	DLX5	[activation]
14590	MAPKAPK2	CSNK2B	[activation]
14591	PLCB3	TRPC3	[activation]
14592	CBL	ACTN1	[activation]

14593	DLGAP3	PIK3R1	[activation]
14594	SNCA	EEF1A1	[activation]
14595	EGFR	SFN	[activation]
14596	RAD51D	KIFC3	[activation]
14597	CRKL	SHANK3	[activation]
14598	PBK	KIAA1377	[activation]
14599	JUNB	DDIT3	[activation]
14600	CDC16	RB1	[activation]
14601	CDK11A	PRPF40A	[activation]
14602	ROCK1	IRS1	[activation]
14603	NBN	RAD50	[activation]
14604	LXN	CPA4	[activation]
14605	SMURF1	WEE1	[inhibition]
14606	EPB41L3	CRK	[activation]
14607	MAP3K1	HSPA6	[activation]
14608	TEX33	LRRK1	[activation]
14609	RIF1	STX5	[activation]
14610	SUV39H1	KLHDC4	[activation]
14611	DHX8	NOTCH1	[activation]
14612	CDC6	CCL2	[activation]
14613	GABARAP	STK3	[activation]
14614	MTERFD2	ICT1	[activation]
14615	NSF	PTPRC	[activation]
14616	RPS6KA3	EIF3C	[activation]
14617	PPP2CA	PPM1B	[inhibition]
14618	HMOX2	INPP5K	[activation]
14619	FBXW11	AXIN2	[inhibition]
14620	PPP1CA	PPP1R3C	[activation;inhibition]
14621	RAP1GDS1	HRAS	[activation]
14622	YWHAB	DOCK4	[activation]
14623	VDR	SRPK1	[activation]
14624	CXCL11	MTUS2	[activation]
14625	TBXA2R	KCNMB1	[inhibition]
14626	CD2AP	HCK	[activation]
14627	HSPB2	TRAF6	[activation]
14628	SIX1	EYA3	[activation]
14629	GRK6	GIT1	[activation;inhibition]
14630	DOCK11	YWHAB	[activation]
14631	TTC13	FBXO6	[inhibition]
14632	RICTOR	HNRNPUL1	[activation]
14633	SRPK3	PHF5A	[activation]
14634	GRB2	MLXIPL	[activation]
14635	TRRAP	DOT1L	[activation]
14636	GTF2H1	PIK3R1	[activation]
14637	EZH2	ANXA4	[activation]
14638	CASP4	HIGD1A	[activation]
14639	BMP3	COL4A2	[activation]
14640	FASN	HSP90AB1	[activation]
14641	TFDP1	SOCS3	[inhibition]
14642	CASP2	CSNK2A1	[activation]
14643	ACTA1	EP300	[activation]
14644	CDC20	CDC16	[inhibition]
14645	CDK5RAP2	KAT5	[activation]
14646	NUP88	CD82	[activation]
14647	ACVR1	RAB25	[activation]
14648	HIST3H3	PRKCA	[activation]
14649	ESR1	SETD7	[activation]
14650	LRFN4	PPP2R1A	[inhibition]
14651	VCAM1	SCRIB	[activation]
14652	RPA1	RPA4	[activation]
14653	CPSF3	MDC1	[activation]
14654	SYK	TRAF3	[activation]
14655	TPST1	CCR2	[activation]
14656	ADRB2	CD81	[activation]
14657	MAP1B	STK3	[activation]
14658	CDH10	CDH9	[activation]
14659	SGK1	PIP	[activation]
14660	NR3C1	CD4	[activation]
14661	GPR151	GAL	[activation]
14662	PLAGL1	TP53	[activation]
14663	ABL1	SORBS1	[activation]
14664	SETDB1	BID	[activation]
14665	XPO1	STK4	[activation]
14666	GNA15	OPRK1	[activation]
14667	CDK5RAP3	UFC1	[activation]
14668	ACTN1	BTK	[activation]

14669	SIRT3	HSP90B1	[activation]
14670	GIPR	GCG	[activation]
14671	YWHAG	DDX39B	[activation]
14672	PRKAA1	MARK1	[inhibition]
14673	TRAF6	ETF1	[activation]
14674	RAC1	CDC42SE2	[activation]
14675	PAXIP1	CYB5R4	[activation]
14676	CDC5L	OXSR1	[activation]
14677	NOTCH1	WDR11	[activation]
14678	ACTN1	GIT2	[activation]
14679	SRGN	CCL3	[activation]
14680	AFAP1	SRC	[activation]
14681	TRAF6	EDARADD	[activation]
14682	POR	INSIG1	[activation]
14683	MMTAG2	RASSF10	[inhibition]
14684	C8orf33	NGEF	[activation]
14685	MYC	RPRD2	[activation]
14686	WAS	CSNK2A1	[activation]
14687	MLH1	FLNB	[activation]
14688	SH2D2A	ITK	[activation]
14689	PSEN2	RAB11A	[activation]
14690	EGFR	OS9	[activation]
14691	EP300	MORF4L1	[activation]
14692	NBN	RPA3	[activation]
14693	NR3C1	HMOX2	[activation]
14694	HEMGN	MCM3	[activation]
14695	HIRIP3	SRPK2	[activation]
14696	NUMB	EPS15L1	[activation]
14697	CDK17	NOP10	[activation]
14698	CD44	TGFBR1	[activation]
14699	TRAF6	PTGES3	[activation]
14700	BTC	EGFR	[activation]
14701	ESR1	BRI3BP	[activation]
14702	SUMO2	ERCC4	[activation]
14703	DOCK8	E2F3	[activation;inhibition]
14704	ERG	PRKDC	[activation]
14705	CAV1	RASA1	[activation]
14706	FGF2	FGFBP1	[activation]
14707	AURKA	BTRC	[activation]
14708	CDC5L	UBA5	[activation]
14709	GLRX2	TXNRD1	[activation]
14710	SRC	EIF3D	[activation]
14711	KIDINS220	MYC	[activation]
14712	APOE	LDLR	[activation]
14713	NXF1	EIF4A3	[activation]
14714	C6orf165	USHBP1	[activation]
14715	STAT5B	PGR	[activation]
14716	CDH11	CDH2	[activation]
14717	ESYT2	MAPK15	[activation;inhibition]
14718	BATF3	ATF4	[activation]
14719	ABI1	VAV1	[activation]
14720	YWHAB	SLC8A2	[activation]
14721	HSP90AB1	PRKCA	[activation]
14722	FMNL2	CDC42	[activation]
14723	GRB2	SUV39H2	[activation]
14724	HLA-B	SBDS	[activation]
14725	CHEK2	STAT1	[activation]
14726	RHOA	BID	[activation]
14727	PPM1H	NXF1	[activation]
14728	ADAM17	MAD2L2	[activation]
14729	SLC6A3	TGFB1I1	[activation]
14730	MAP1LC3B	FAS	[activation]
14731	AKT1	STAT1	[activation]
14732	CARD11	FYB	[activation]
14733	PSMA2	INSIG2	[activation]
14734	HIST3H3	AHDC1	[activation]
14735	GRB2	SNX12	[activation]
14736	CDKN1A	COL4A5	[activation;inhibition]
14737	FSCN1	HLA-B	[activation]
14738	TNIP1	PIK3CD	[activation]
14739	GNAQ	FFAR2	[activation]
14740	TNFSF12	LYN	[activation]
14741	ITK	TGFBR1	[activation]
14742	RAPGEFL1	MLH1	[activation]
14743	CDC5L	MAP2K7	[activation]
14744	RICTOR	HDAC1	[activation]

14745	RASSF1	BTRC	[inhibition]
14746	LRP1	HSP90AA1	[activation]
14747	PPARG	PRNP	[activation]
14748	HSPA12A	CDC5L	[activation]
14749	DGKA	SIRT1	[activation]
14750	APP	RIOK2	[activation]
14751	CCL19	CXCL13	[activation]
14752	SMARCB1	CEBPB	[activation]
14753	STK4	ELAVL1	[activation]
14754	HSDL2	ADRB2	[activation]
14755	TICAM1	TRAF2	[activation]
14756	NOTCH1	MCM5	[activation]
14757	PHF14	PYHIN1	[activation]
14758	CBL	NCK1	[activation]
14759	PAK6	HSP90AB1	[activation]
14760	PIK3CA	TMOD1	[activation]
14761	JMJD1C	RXRA	[inhibition]
14762	CDC25C	PCNA	[activation]
14763	RALGDS	CNKSR1	[activation]
14764	CASP1	IL1A	[activation]
14765	HSPA9	TP53	[activation]
14766	MEIS2	TLX1	[activation]
14767	FYB	SRC	[activation]
14768	ANAPC5	ESR2	[activation]
14769	EFNA1	KAT5	[activation]
14770	NBN	POLR1B	[activation]
14771	CDKN1A	HDAC4	[activation]
14772	CWC25	MDM2	[activation]
14773	COMMD4	RELA	[activation]
14774	SMARCA5	SATB1	[activation]
14775	FGA	FGG	[activation]
14776	SYK	PAG1	[activation]
14777	GRB2	FCGR2B	[activation]
14778	CAMP	CTSG	[activation]
14779	NXF1	TRIM29	[activation]
14780	HIST1H1E	CD81	[activation]
14781	APP	CDKL3	[activation]
14782	HIF1A	NR4A1	[inhibition]
14783	PLK3	PRNP	[activation]
14784	MAPK1	PAK1	[activation]
14785	INHA	INHBB	[activation]
14786	YARS2	ICT1	[activation]
14787	FGB	SERPINA5	[inhibition]
14788	PRG4	MYC	[activation]
14789	TP53	JMJD6	[activation]
14790	ANKZF1	AURKB	[activation]
14791	CES1	CREB3	[activation]
14792	PRKRA	SRPK3	[activation]
14793	YWHAB	SRSF10	[activation]
14794	ESR1	SHC1	[activation]
14795	CDK2AP2	WARS	[activation]
14796	ERH	ACP6	[activation;inhibition]
14797	HSP90AB1	STAT3	[activation]
14798	NCDN	A2M	[inhibition]
14799	HLA-C	SARS	[activation]
14800	AR	PTPN11	[activation]
14801	ERBB2	SPTBN4	[activation]
14802	NCL	ICAM1	[activation]
14803	PFN1	FMNL1	[activation]
14804	SPP1	KIAA1009	[activation]
14805	PLEKHA5	YWHAG	[activation]
14806	PHLDA3	APP	[activation]
14807	FLOT2	CCDC8	[inhibition]
14808	HERC5	CDKN1A	[inhibition]
14809	PPP1CA	PHACTR4	[activation;inhibition]
14810	BCL2	BID	[activation]
14811	PAK4	RAN	[activation]
14812	ICAM1	RPL18	[activation]
14813	GRB2	ARHGEF7	[activation]
14814	RAPGEF1	CBL	[activation]
14815	BMX	CASP3	[activation]
14816	TGFBR1	TRAP1	[activation]
14817	GSG1L	LXN	[activation]
14818	PLK1	MDM2	[activation]
14819	ARPC5	GRB2	[activation]
14820	RNF19A	ZAK	[activation]

14821	PTGS2	CASK	[activation]
14822	PAK7	BAD	[inhibition]
14823	ARHGEF7	SHANK2	[activation]
14824	FZD5	SCYL2	[activation]
14825	DNAJA1	YWHAE	[inhibition]
14826	HNRNPAB	PAK1	[activation]
14827	GRK6	LTB4R	[activation]
14828	PLCG2	FLT1	[activation]
14829	NFKBIA	HSPA4	[activation]
14830	COPA	ITGA4	[activation]
14831	IKBKB	SMARCC1	[activation]
14832	KDR	FLT1	[activation]
14833	RB1	DYRK1A	[activation;inhibition]
14834	MAPK8	CDKN2C	[activation]
14835	PIK3R3	ORM1	[activation]
14836	SH3KBP1	CTNNB1	[activation]
14837	PDGFRB	HSP90AA1	[activation]
14838	SMAD3	ATF3	[activation]
14839	CDK11B	PIN1	[activation]
14840	RAPGEF2	VSIG8	[activation]
14841	AGK	MYC	[activation]
14842	DPY30	SMARCA1	[activation]
14843	SIRT1	MRE11A	[activation]
14844	ZFYVE9	STAM2	[activation]
14845	EPHA8	FYN	[activation]
14846	CPN1	C3	[activation]
14847	GRB2	GJA9	[activation]
14848	ABI2	NXF1	[activation]
14849	GADD45A	MDM2	[activation]
14850	NCALD	ACTB	[activation]
14851	DDX58	RAI14	[activation]
14852	ATM	SNW1	[activation]
14853	CTNNB1	ISG15	[activation;inhibition]
14854	CCHCR1	AMOTL2	[activation]
14855	CCL17	DARC	[activation]
14856	NOS1AP	NOS1	[activation]
14857	TRAF2	RBM41	[activation]
14858	HLA-B	CD8A	[activation]
14859	ACVR1B	HSP90AB1	[activation]
14860	CASP3	UBC	[activation]
14861	RAD54B	MDM2	[activation]
14862	CLK4	SRPK1	[activation]
14863	TNFRSF10B	RHOA	[activation]
14864	RARS	GRB2	[activation]
14865	MCM6	EGFR	[activation]
14866	ZBTB33	CTNND1	[activation]
14867	MBP	TLK1	[inhibition]
14868	ZP4	FURIN	[activation]
14869	UBE2I	ATF2	[activation]
14870	EIF4E2	MYOG	[activation]
14871	ELK1	MAP2K1	[activation]
14872	EZR	PLCB3	[activation]
14873	ODC1	MAP2K1	[activation]
14874	RAE1	SEH1L	[activation]
14875	GLP1R	ARFRP1	[activation]
14876	SRSF10	PAXIP1	[activation]
14877	PAK1	FOXO1	[activation]
14878	POLD3	CHD4	[activation]
14879	FTSJ3	EIF2AK2	[activation]
14880	AIP	GNAQ	[activation]
14881	INPP5D	GRB2	[activation]
14882	TOPORS	TP53	[activation]
14883	ANXA7	GAPDH	[activation]
14884	GTF2B	CREBBP	[activation]
14885	APITD1	FAAP24	[activation]
14886	IGSF6	APP	[activation]
14887	CDC6	CDK6	[activation]
14888	CDH1	SFRP2	[activation;inhibition]
14889	EP300	BCL6	[activation]
14890	STX19	SUV39H1	[activation]
14891	PSEN1	KCNIP4	[activation]
14892	CACNG1	VAPA	[activation]
14893	LTB4R	GNA15	[activation]
14894	GRB7	SFN	[activation]
14895	IQGAP1	ISG15	[activation;inhibition]
14896	CDX2	SMARCB1	[activation]

14897	DNAJB4	SMAD3	[inhibition]
14898	SPERT	CDC23	[activation]
14899	SUV420H2	RBL2	[activation]
14900	UBA5	UFM1	[activation]
14901	SRGAP2	EXOC1	[activation]
14902	PXN	IQGAP1	[activation]
14903	RAC1	DOCK2	[activation]
14904	CHRM2	SET	[activation]
14905	CCND3	RB1	[inhibition]
14906	RPA1	TFRC	[activation]
14907	PID1	NME1	[activation]
14908	SIRPA	PTPN6	[activation;inhibition]
14909	MAD2L1	APC	[inhibition]
14910	FGFR2	APP	[activation]
14911	E2F1	LAMTOR5	[activation]
14912	BCL10	CASP9	[activation]
14913	VCAM1	EIF4A3	[activation]
14914	PNP	VCAM1	[activation]
14915	PAX1	MEOX1	[activation]
14916	MYC	NOL11	[activation]
14917	SRC	NR1H3	[activation]
14918	TSC2	RAB5A	[activation]
14919	PRIM1	NXF1	[activation]
14920	RXRA	MAPK7	[inhibition]
14921	APC	CEP250	[inhibition]
14922	CCNA2	CCNB1	[activation]
14923	DTNB	NUP62	[activation]
14924	PDGFA	DUSP3	[activation]
14925	BCL2L1	DOCK7	[activation]
14926	STX1B	UNC13B	[activation]
14927	DVL2	FZD4	[activation]
14928	NCOA2	JUN	[activation]
14929	CSNK2A1	CAV1	[activation]
14930	EIF3I	CD4	[activation]
14931	PAK1	SNW1	[activation]
14932	PDE6H	GNAO1	[activation;inhibition]
14933	MAP4K1	FBXW8	[activation]
14934	MAST2	TTN	[activation]
14935	CLEC5A	TYROBP	[activation]
14936	PAK4	YWHAE	[activation]
14937	RXRA	CLOCK	[inhibition]
14938	CLCA1	ITGB4	[activation]
14939	CALM1	CCNA2	[activation]
14940	PAXIP1	RNF32	[activation]
14941	DOK3	CSK	[activation]
14942	MDM2	ECH1	[activation]
14943	APP	FCGR3A	[activation]
14944	ATAD1	GOSR1	[activation]
14945	STAT5A	CRKL	[activation]
14946	PTEN	QRFPR	[activation]
14947	IKBIP	NUP62	[activation]
14948	PRKCZ	ECT2	[activation]
14949	HDAC3	MAPK11	[activation]
14950	HDAC1	HSD11B2	[activation]
14951	CREBBP	AIRE	[activation]
14952	YWHAZ	SUPT6H	[activation]
14953	TIMP2	HSP90AB2P	[activation]
14954	MAP3K5	YWHAB	[activation]
14955	EIF2B1	ADRA2A	[activation]
14956	FYN	GAB3	[activation]
14957	HSP90AB1	TESK1	[activation]
14958	PRKCZ	GSK3A	[activation]
14959	SMCHD1	OBSL1	[inhibition]
14960	MYC	IL4R	[activation]
14961	CSH1	SMAD2	[inhibition]
14962	SMARCA5	RPA1	[activation]
14963	LMNA	EGF	[activation]
14964	BCR	MAP4K5	[activation]
14965	FANCG	FTSJ3	[activation]
14966	BATF2	RELA	[activation]
14967	EIF2S3	HLA-B	[activation]
14968	MDM2	PLEC	[activation]
14969	PDIK1L	TNF	[activation]
14970	KCNIP3	CASP3	[activation]
14971	BCAR3	EGFR	[activation]
14972	LRRK2	TPM2	[activation]

14973	RPL10L	TP53	[activation]
14974	STAT5B	DMRTA1	[activation]
14975	LMNB1	LRRK2	[activation]
14976	TP53	EFEMP2	[activation]
14977	FSD2	PIK3R3	[activation]
14978	ARL15	FUNDC2	[activation]
14979	RAC1	SET	[activation]
14980	ARRDC4	AVPR2	[activation]
14981	TP53	EIF4E2	[activation]
14982	RIN3	GRB2	[activation]
14983	EIF3F	HTR2A	[activation]
14984	GAB2	LAT	[activation]
14985	EGFR	MON2	[activation]
14986	AGT	PRCP	[activation]
14987	WHSC1	HNRNPAB	[activation]
14988	INSR	PTPRJ	[activation]
14989	FYN	ARHGEF11	[activation]
14990	POLR1B	GRB2	[activation]
14991	EPRS	MAPK13	[activation]
14992	FLOT1	BACE1	[activation]
14993	STX5	NAPA	[activation]
14994	MAPK8IP1	DUSP16	[inhibition]
14995	P2RX4	CDH5	[activation]
14996	PDIA2	FYN	[activation]
14997	GATA4	EZH2	[activation]
14998	PRKCD	TIAM1	[activation]
14999	MAPT	PRKACA	[activation]
15000	FAS	TNF	[activation]
15001	GRB2	TRAIP	[activation]
15002	DAB2IP	DAB1	[activation]
15003	PRKCE	GJA1	[inhibition]
15004	CHEK2	MUS81	[activation]
15005	FGFR3	STAT1	[activation]
15006	MAPT	CASP8	[activation]
15007	PTPN6	LIFR	[activation;inhibition]
15008	PTPRC	CD8A	[activation]
15009	AR	CTNNB1	[activation]
15010	RIPPLY2	TFG	[activation]
15011	RAP1GAP2	WNK1	[activation]
15012	VCAM1	GOLGA7	[activation]
15013	GATA1	SMARCA4	[activation]
15014	FBXO25	MYO1B	[activation]
15015	GRHPR	FOS	[activation]
15016	MAX	EPAS1	[inhibition]
15017	TP53	ASH2L	[activation]
15018	POMC	HLA-DRB3	[activation]
15019	CCDC57	TBC1D22B	[activation;inhibition]
15020	KIRREL	NPHS2	[activation]
15021	HSP90AA1	CDK11B	[activation]
15022	CD3D	CD3E	[activation]
15023	MCM7	USHBP1	[activation]
15024	HTR1D	HTR1A	[activation]
15025	GABARAPL2	SRSF5	[activation]
15026	TRAF3	TFG	[activation]
15027	KAT2B	ACTB	[activation]
15028	FBXO6	ITGA2B	[activation]
15029	TGFBR1	IKBKB	[activation]
15030	CFTR	GNAI2	[activation]
15031	SMAD2	ITGB4	[activation]
15032	ACTG1	FPR1	[activation]
15033	NUP50	RAN	[activation]
15034	BCR	PTPN6	[activation;inhibition]
15035	PCBP2	H2AFX	[activation]
15036	ENPEP	AGT	[activation]
15037	ST8SIA2	NCAM1	[activation]
15038	SRCIN1	SNAP25	[activation]
15039	SMAD1	EIF2AK4	[activation;inhibition]
15040	FLNC	WNK1	[activation]
15041	DNAJA1	DNAJA4	[inhibition]
15042	NIPSNAP1	OBSL1	[activation;inhibition]
15043	PARD6A	YWHAH	[activation]
15044	CRYBB2	HSPB1	[inhibition]
15045	MAPK8	PDPK1	[activation]
15046	STAT3	TIRAP	[activation]
15047	ADRB2	RAB18	[activation]
15048	FOCAD	RAD21	[activation]

15049	MEIS1	TLX1	[activation]
15050	MYO10	PRKCI	[activation]
15051	APITD1	STRA13	[activation]
15052	YWHAB	MAPK7	[activation]
15053	MYC	RFC5	[activation]
15054	HPDL	MDM2	[activation]
15055	GRPEL1	HLA-B	[activation]
15056	MYC	MLH1	[activation]
15057	SMAD3	FOXL2	[inhibition]
15058	SRC	NR3C1	[activation]
15059	CPNE1	MAP2K1	[activation]
15060	FANCA	LIPG	[activation]
15061	PPP2R2B	JUP	[activation]
15062	ACTG1	BMI1	[activation]
15063	TRADD	FAS	[activation]
15064	SRMS	DOK1	[activation]
15065	PLK1	ERCC4	[activation]
15066	MOCS2	ABCF3	[activation]
15067	ADIPOR1	NFKBIL1	[activation]
15068	TAB1	EGFR	[activation]
15069	ATG3	CREBBP	[activation]
15070	INS	HLA-DQB1	[activation]
15071	SMAD2	ANAPC10	[activation]
15072	STRA13	FANCG	[activation]
15073	SLC25A4	ICT1	[inhibition]
15074	ACTB	GABARAP	[activation]
15075	VAPB	TTC1	[activation]
15076	BAIAP2	CDC42	[activation]
15077	BMPR2	LSP1	[activation]
15078	EPHB1	CBL	[activation]
15079	CDK4	SHOX2	[activation]
15080	ESR1	CHUK	[activation]
15081	LTBP1	CACNA1A	[inhibition]
15082	PRKCD	TOP2A	[activation]
15083	SUMO1	CASP8	[activation]
15084	PLEKHB1	BMPR1B	[activation]
15085	SMC4	YY1	[activation]
15086	LAMA1	APP	[activation]
15087	PPP3CA	CABIN1	[activation]
15088	HSP90AA1	HIST3H3	[activation]
15089	IFNAR1	PTPRC	[activation]
15090	CDH2	PTPRC	[activation]
15091	PRKDC	YWHAG	[activation]
15092	SKIL	PIAS1	[inhibition]
15093	DVL1	SMAD3	[activation]
15094	CCNA1	RPA1	[activation]
15095	TAB1	SERPINA4	[inhibition]
15096	PRKCD	PLSCR1	[activation]
15097	TYK2	KLF10	[activation]
15098	HSPB9	CTCF	[activation]
15099	NPY	NR1H2	[activation]
15100	OBSL1	RRP9	[activation]
15101	MCM3	EPAS1	[activation]
15102	M6PR	REN	[activation]
15103	ANAPC11	CDC27	[activation]
15104	STIP1	STK11	[activation]
15105	GSN	CSK	[activation]
15106	UBE2I	IQGAP1	[activation]
15107	BRF2	MYO1B	[activation]
15108	FABP3	ITGB4	[activation]
15109	AP2S1	EGFR	[activation]
15110	KIF2A	LRRK2	[activation]
15111	SHOX2	ZDHHC17	[activation]
15112	OBSL1	NUP214	[activation]
15113	MEOX2	MMP3	[activation]
15114	CTNNB1	CAV1	[activation]
15115	YWHAZ	TP53	[activation]
15116	GJA3	CAV1	[activation]
15117	STAT1	SPTAN1	[activation]
15118	UBC	F2R	[activation]
15119	GNAZ	STIP1	[activation]
15120	MDM2	TMEM87A	[activation]
15121	SRPK1	APMAP	[activation]
15122	RAPGEF2	SSSCA1	[activation]
15123	EPRS	ITGA4	[activation]
15124	BCAR3	PPP2R1A	[inhibition]

15125	PRKDC	TTI1	[activation]
15126	GRB2	DTX1	[activation]
15127	TGFB3	LTBP3	[activation]
15128	LRRK2	PPP1CA	[activation]
15129	PPP3CA	CASP3	[activation]
15130	PAX3	SRC	[activation]
15131	YWHAE	CDK16	[activation]
15132	CTCF	HMGB1	[activation]
15133	E2F4	PML	[inhibition]
15134	WDR5	KMT2A	[activation]
15135	PICK1	GRIA4	[activation]
15136	RCVRN	GIT2	[inhibition]
15137	HDAC4	MECOM	[activation]
15138	MAPK8	SIRT1	[activation]
15139	CSNK1D	PTPN14	[activation]
15140	NTN4	DCC	[activation]
15141	RXRA	TRIP10	[inhibition]
15142	IP6K2	TP53	[activation]
15143	TP53	ERCC2	[activation]
15144	PAXIP1	PCBP2	[activation]
15145	RAD54B	TRAPPC6A	[activation]
15146	PTPN18	GRB2	[activation]
15147	VHL	MSH4	[inhibition]
15148	YWHAG	MAP3K5	[activation]
15149	NLRP2	IKBKB	[activation]
15150	CDK2	PHLDA3	[activation]
15151	FCGR1A	CRKL	[activation]
15152	SARM1	TRAF2	[activation]
15153	IFNA1	IL2	[activation]
15154	EYA2	TFAP2A	[activation]
15155	MYOD1	NUPR1	[activation]
15156	TP53	ESR1	[activation]
15157	EP300	TACC2	[activation]
15158	PLXNB1	RHOD	[activation]
15159	CDS1	CDC25C	[activation]
15160	NCOR2	GEM	[activation]
15161	ANK2	L1CAM	[activation]
15162	PPP1CA	STX1A	[activation]
15163	SLC25A5	TP53	[activation]
15164	HCFC2	FOXO3	[inhibition]
15165	SLC25A6	MAP3K3	[activation]
15166	DOCK7	SNCA	[activation]
15167	FAS	EGFR	[activation]
15168	NLRP3	CARD8	[inhibition]
15169	GRB2	PNRC1	[activation]
15170	STAT1	FGFR4	[activation]
15171	MAPK14	IKBKB	[activation]
15172	RARA	GNAQ	[activation]
15173	DLGAP2	NOS1	[activation]
15174	MIF	GORASP2	[activation]
15175	CHEK2	STRAP	[activation]
15176	CD3E	CCR7	[activation]
15177	RPA3	PRKDC	[activation]
15178	MEF2A	SMAD2	[activation]
15179	MTNR1B	COPA	[activation]
15180	PAK4	GRB2	[activation]
15181	MDM2	MIF	[activation]
15182	DDX39B	EIF4E	[activation]
15183	U2AF2	DVL2	[activation]
15184	RAC2	FBXO6	[activation]
15185	MOS	FKBP5	[inhibition]
15186	SRC	ESRRG	[activation]
15187	PRMT1	AR	[activation]
15188	IRS4	SAV1	[activation]
15189	ETS1	SRC	[activation]
15190	NXF1	RAD51D	[activation]
15191	CFLAR	EPPK1	[inhibition]
15192	SRC	ANXA2	[activation]
15193	IL8	CCL4	[activation]
15194	PRKCA	ADRBK1	[activation]
15195	EZH2	SUV39H1	[activation]
15196	CKAP4	MAST3	[inhibition]
15197	DMXL1	CALM1	[activation]
15198	BCL2L1	MTOR	[activation]
15199	KALRN	COIL	[activation]
15200	CASP9	MAP2K1	[activation]

15201	LRRK2	WASF2	[activation]
15202	SPHK1	EP300	[activation]
15203	NRGN	PRKCB	[activation]
15204	LCK	SKAP1	[activation]
15205	CASP10	NFKBIL1	[activation]
15206	GIT2	PAK3	[activation]
15207	CDCA8	PIAS2	[activation]
15208	PPP6C	ARHGEF2	[activation]
15209	SMAD4	PLG	[inhibition]
15210	SLC25A6	TRADD	[activation]
15211	CTNNB1	AKAP12	[activation]
15212	APPBP2	ERO1L	[activation]
15213	PTGDR	MAPK3	[activation]
15214	GRB2	PDLIM7	[activation]
15215	CHMP4A	EGFR	[activation]
15216	EP300	FUS	[activation]
15217	BACE1	BRI3	[activation]
15218	SERPINA1	PRTN3	[inhibition]
15219	PGR	BPTF	[activation]
15220	ARF6	QTRT1	[activation]
15221	COL1A1	BMP1	[activation]
15222	MAML1	CREBBP	[activation]
15223	UBE2E2	UBOX5	[activation]
15224	CDK2	CDKN3	[activation]
15225	ATM	ACTL6B	[activation]
15226	APC	SETDB1	[activation]
15227	PHIP	PYHIN1	[activation]
15228	TNS1	KIT	[activation]
15229	SKP1	BCL6	[activation]
15230	CTNNA1	OBSL1	[activation]
15231	TYK2	PRMT5	[activation]
15232	ALKBH1	DNAJB6	[inhibition]
15233	CPNE1	CPNE4	[activation]
15234	SMURF1	ETV6	[inhibition]
15235	FOS	FIP1L1	[activation]
15236	PPP2CA	CCT8	[inhibition]
15237	NUMB	TP53	[activation]
15238	DVL3	CYLD	[inhibition]
15239	HSP90AA1	MAP2K7	[activation]
15240	SMAD4	PAX6	[activation]
15241	P2RX2	P2RX1	[activation]
15242	STX7	VPS11	[activation]
15243	APC	MBD5	[inhibition]
15244	HSP90AB1	CDK11A	[activation]
15245	HSP90AA1	NTRK3	[activation]
15246	CTNNA1	CDH1	[activation]
15247	NDRG1	ACTG1	[activation]
15248	ASB16	SRC	[activation]
15249	EIF4G3	CAMK1	[activation]
15250	SNCA	PTK2B	[activation]
15251	KLRC4	MICB	[activation]
15252	CDKN2A	MYC	[activation;inhibition]
15253	SPP1	DNTTIP2	[activation]
15254	SRPK2	C1orf63	[activation]
15255	ADCYAP1	VIPR1	[activation]
15256	PLEKHF2	TRAF6	[activation]
15257	DAB1	SIRPB1	[activation]
15258	NUDT5	GH1	[inhibition]
15259	EPB41L3	YWHAH	[activation]
15260	CMTM5	TPD52L3	[activation]
15261	SMURF1	CDK14	[inhibition]
15262	RNF126	TRAF6	[activation]
15263	CPNE1	BUB3	[inhibition]
15264	CEBPD	E2F1	[activation]
15265	ANAPC5	MDC1	[activation]
15266	MARK1	STK11	[activation]
15267	JUNB	SAT1	[activation]
15268	PAK2	PGRMC1	[activation]
15269	SWAP70	RAPGEF4	[activation]
15270	PIK3R1	CD3E	[activation]
15271	NAF1	DAB1	[activation]
15272	DOK1	ERBB2	[activation]
15273	RAB3GAP2	PAXIP1	[activation]
15274	VAMP8	VCAM1	[activation]
15275	HLA-B	KLRD1	[activation]
15276	RAF1	BCL6	[activation]

15277	RCAN1	RAF1	[activation]
15278	TRAF5	NGFR	[activation]
15279	UGDH	SIRT1	[activation]
15280	RGS14	GNA11	[activation]
15281	SHB	MET	[activation]
15282	HIST3H3	RCOR1	[activation]
15283	RELA	PRMT1	[activation]
15284	HDAC1	SMAD1	[activation]
15285	TSHR	PIK3R1	[activation]
15286	WDR18	MDC1	[activation]
15287	TP53	CRYAB	[activation]
15288	MSR1	MALL	[activation]
15289	CDK5RAP2	CRMP1	[activation]
15290	STAT5B	STAP2	[activation]
15291	IRF5	MYD88	[activation]
15292	SFN	FOXO4	[activation]
15293	RAD51D	XRCC2	[activation]
15294	WDR20	TYK2	[activation]
15295	YWHAB	RICTOR	[activation]
15296	NR2F1	NSD1	[activation]
15297	NGEF	RHOB	[activation]
15298	TNRC6C	CNOT10	[activation]
15299	GRK6	SLC9A3R1	[activation]
15300	PRKDC	NFKB2	[activation]
15301	RGS20	ABL1	[activation;inhibition]
15302	GNA13	S1PR2	[activation]
15303	ABI1	ITK	[activation]
15304	SLC9A1	MAP3K7	[activation]
15305	EP300	PPP2CA	[activation]
15306	NR1I2	RPS6KB1	[activation]
15307	ARL13B	UBE2I	[activation]
15308	PTPRC	CD22	[activation]
15309	DOCK2	CDC25B	[activation]
15310	HLA-B	SLC1A5	[activation]
15311	NUMA1	DVL1	[activation]
15312	RHOA	TGFBR1	[activation]
15313	ASB18	SLC25A6	[activation]
15314	ILK	DHX36	[activation]
15315	ANAPC7	ANAPC15	[activation]
15316	SMURF1	LHX9	[inhibition]
15317	CCDC137	SRPK3	[activation]
15318	CTNND1	MTNR1B	[activation]
15319	MYOZ2	MYB	[activation]
15320	AHCTF1	CTNNB1	[activation]
15321	PRKACB	VAPA	[activation]
15322	RALGDS	RIT2	[activation]
15323	DUSP4	CRMP1	[activation]
15324	TICAM1	TNFAIP3	[activation]
15325	SMAD4	PIAS2	[inhibition]
15326	SRPK2	PAK4	[activation]
15327	ARF6	SRPRB	[activation]
15328	JUP	PKP3	[activation]
15329	CTNNB1	SUMO1	[activation]
15330	GNB2	ERBB2	[activation]
15331	PIH1D3	P2RX4	[activation]
15332	OSBPL9	HSPB1	[activation]
15333	ANAPC7	PTEN	[activation]
15334	HLA-B	EIF3I	[activation]
15335	NAA38	UBE2E3	[activation]
15336	NFE2L2	NFE2	[activation]
15337	PDIA4	ATF2	[activation]
15338	GNA12	ACTB	[activation]
15339	CNP	LRRK2	[activation]
15340	PGR	EP300	[activation]
15341	CAMK2A	HSP90AB1	[activation]
15342	DNM1	GRB2	[activation]
15343	TNIP3	KIF11	[activation]
15344	TJP2	TRAF6	[activation]
15345	PPIG	SRPK1	[activation]
15346	PFDN1	EOMES	[activation]
15347	PLCG1	AR	[activation]
15348	GTPBP10	ICT1	[activation]
15349	PCNA	CDK2	[activation]
15350	SRGAP2	ITSN2	[activation]
15351	RUNX1	SMAD5	[activation]
15352	CD38	PECAM1	[activation]

15353	PPM1A	FGFR2	[activation]
15354	RPA1	HELQ	[activation]
15355	CARD11	TAB1	[activation]
15356	STX7	VPS18	[activation]
15357	TNF	TNFRSF1B	[activation]
15358	GRB2	HP	[activation]
15359	EGFR	NCK2	[activation]
15360	CTNNB1	CEBPA	[activation;inhibition]
15361	STC2	ADAM11	[activation]
15362	CRK	SMAD3	[activation]
15363	APOH	LRP8	[activation]
15364	TLN1	YWHAZ	[activation]
15365	RANBP2	UBE2I	[activation]
15366	TLN1	ACTA1	[activation]
15367	FOSL1	JUNB	[activation]
15368	NUP153	ESR1	[activation]
15369	MCC	SMC4	[activation]
15370	CREBBP	HCK	[activation]
15371	HSPA6	STK4	[activation]
15372	PRKG1	ITPR1	[inhibition]
15373	STK4	HAL	[activation]
15374	SDCBP	RAB5A	[activation]
15375	TWF2	ACTR3B	[activation]
15376	MAPRE1	TERF1	[activation]
15377	TEC	TP53	[activation]
15378	NCK1	EGFR	[activation]
15379	TGFB2	ENG	[activation]
15380	NT5DC2	EGFR	[activation]
15381	AKT1S1	YWHAZ	[activation;inhibition]
15382	PTPRB	EPOR	[activation]
15383	LAMTOR4	LAMTOR1	[activation]
15384	RXFP2	INSL3	[activation;inhibition]
15385	TAB3	MAP3K7	[activation]
15386	KMT2C	TP53	[activation]
15387	ATF2	ITGA2	[activation]
15388	CNTNAP4	MAST3	[activation]
15389	CEBPB	SMAD4	[activation]
15390	FRS3	GATA1	[activation]
15391	ARHGEF7	ARHGEF6	[activation]
15392	IGHG1	TNS3	[activation]
15393	FOXM1	FZR1	[inhibition]
15394	SFN	IRS1	[activation]
15395	PTCD3	ATF4	[activation]
15396	SCAP	INSIG2	[activation]
15397	CSNK2B	NPAS2	[activation]
15398	ACVR2B	MSTN	[activation]
15399	MBP	TRAF6	[activation]
15400	MAST2	YWHAB	[activation]
15401	FXYD6	BCAR1	[activation]
15402	MRPL12	MDM2	[activation]
15403	MYOG	MLH1	[activation]
15404	RHOU	PTK2	[activation]
15405	CRK	SOS1	[activation]
15406	RRM2	DNM1L	[activation]
15407	POLR2A	MYC	[activation]
15408	PCNA	MDM2	[activation]
15409	CDC5L	ESR2	[activation]
15410	HRH4	GNAI1	[activation]
15411	RB1	SKI	[inhibition]
15412	PEX14	HSD17B4	[activation]
15413	PRKCB	PPP2CB	[inhibition]
15414	KPNA2	CDK5RAP3	[activation]
15415	MYF6	ID1	[activation]
15416	PRKCB	AR	[activation]
15417	HSP90AB1	HIPK4	[activation]
15418	CDK7	SMAD1	[activation]
15419	LGR4	SMC4	[activation]
15420	YWHAB	CDC25A	[activation]
15421	HES1	NR4A1	[inhibition]
15422	MMP1	ITGA2	[activation]
15423	IL2RB	SYK	[activation]
15424	TLR4	BTK	[activation]
15425	MRPL32	ICT1	[activation]
15426	STK11	TBC1D23	[activation]
15427	SRPK2	NGLY1	[activation]
15428	CBL	GRB2	[activation]

15429	TP53	FOXO3	[activation]
15430	NXF1	INF2	[activation]
15431	CDKN1A	VIM	[inhibition]
15432	SYN1	PLCG1	[activation]
15433	FBXO6	IGF1R	[inhibition]
15434	CASP8	MAP4K4	[activation]
15435	FBXL15	BHLHE40	[activation]
15436	EIF4G1	EPB41	[activation]
15437	MYO6	GRB2	[activation]
15438	HSP90AA1	DDR2	[activation]
15439	RIBC2	RAD54B	[activation]
15440	KDR	MET	[activation]
15441	BCR	PTGES3	[activation]
15442	NUDC	MAEL	[activation]
15443	MDC1	PGAM5	[activation]
15444	GTF2B	ATF4	[activation]
15445	FKBPL	CYFIP1	[inhibition]
15446	RCC1	NXF1	[activation]
15447	CKS1B	CDK1	[activation]
15448	HOXB13	ELK1	[activation]
15449	LYN	CREBBP	[activation]
15450	NUDT18	TRAF2	[activation]
15451	FYN	DAB2	[activation]
15452	TP53	MRPS2	[activation]
15453	MCM7	SIPA1L1	[activation]
15454	CD72	PTPN6	[activation;inhibition]
15455	RAP1GAP	FBXW11	[inhibition]
15456	PLXNA1	KDR	[activation]
15457	KBTBD4	MPP3	[inhibition]
15458	NOS1	CTBP1	[activation]
15459	BRF1	TP53	[activation]
15460	CAV1	GRB7	[activation]
15461	GRB2	KIAA1549L	[activation]
15462	HLA-A	ERBB3	[activation]
15463	LRP6	CAPRIN2	[activation]
15464	ITGA4	TIAL1	[activation]
15465	ABL1	YWHAH	[activation]
15466	TLX3	HIST1H2BJ	[activation]
15467	EIF4G1	ESR1	[activation]
15468	RASA1	AURKA	[activation]
15469	UBE2V1	PELI3	[activation]
15470	PPP2R2B	ACACA	[activation;inhibition]
15471	MMP2	PZP	[inhibition]
15472	ODF2L	ERBB3	[activation]
15473	CASP7	HSPA5	[activation]
15474	TNFRSF14	TRAF5	[activation]
15475	ICAM1	DDX3X	[activation]
15476	RB1	BRCA1	[inhibition]
15477	MTOR	STK38	[activation]
15478	OPRM1	GNA15	[activation]
15479	MDC1	CXorf57	[activation]
15480	GRB2	TIAF1	[activation]
15481	MYL12B	GRB2	[activation]
15482	LRRK1	CHGB	[activation]
15483	NANS	SRC	[activation]
15484	RAN	ABL1	[activation]
15485	LRRK1	MATK	[activation]
15486	GAREM	CRKL	[activation]
15487	CTNNB1	NR4A1	[inhibition]
15488	HSPA1A	MAP3K5	[inhibition]
15489	LONP1	APOE	[activation]
15490	ARHGDIB	RAC1	[activation]
15491	OSBP	VAPB	[activation]
15492	N4BP1	CDK6	[activation]
15493	IGHG1	IGLL1	[activation]
15494	ZBTB17	HSP90AB1	[activation]
15495	LRRK2	ALDH8A1	[activation]
15496	SRC	EVL	[activation]
15497	TCAP	MYOZ3	[inhibition]
15498	YWHAH	CDK16	[activation]
15499	BIRC3	RAF1	[activation;inhibition]
15500	AURKA	MAPK6	[activation]
15501	HIF1A	RELA	[activation]
15502	IL21R	JAK1	[activation]
15503	PPP2CA	PPP2R2D	[activation]
15504	GCN1L1	CASP4	[activation]

15505	MAX	PLEKHA5	[inhibition]
15506	MAPK1	NCOA1	[activation]
15507	CNTF	STK16	[activation]
15508	TP53	ANG	[activation]
15509	MYC	SAMD9L	[activation]
15510	GAPDH	SNCA	[activation]
15511	HOOK2	EGFR	[activation]
15512	ZZEF1	SKIL	[activation]
15513	CPSF3	NXF1	[activation]
15514	RPA1	EIF2S2	[activation]
15515	PPP1CC	CCNB1	[inhibition]
15516	BUB1B	SIRT2	[activation]
15517	THSD7A	RAD21	[activation]
15518	GIPC1	ZFPM1	[activation]
15519	MYC	MICALL2	[activation]
15520	GATA1	CEBPE	[activation]
15521	CTNNB1	PTPN6	[activation;inhibition]
15522	CSRP2	PIAS1	[inhibition]
15523	ITSN2	CCDC88C	[activation]
15524	DNAJA1	TNFRSF14	[inhibition]
15525	PVR	PVRL1	[activation]
15526	MCM6	HSPB1	[activation]
15527	SUDS3	LRRK2	[activation]
15528	BCL6	EBF1	[activation]
15529	ABI1	NCK2	[activation]
15530	YWHAZ	HDAC4	[activation]
15531	TRAF3	CBLB	[inhibition]
15532	PROS1	C4BPA	[inhibition]
15533	FTSJ1	IGF2BP1	[activation]
15534	PPP6R2	PRKDC	[activation]
15535	ERF	MAPK1	[inhibition]
15536	SORBS3	WASF2	[activation]
15537	SPI1	BCL6	[activation]
15538	CAMK2A	ITPKA	[activation]
15539	TAB1	GRB2	[activation]
15540	SOCS1	VHL	[inhibition]
15541	ROBO2	KCNRG	[activation]
15542	RFX7	MYC	[activation]
15543	RPA1	DNTTIP1	[activation]
15544	IPO5	RAN	[activation]
15545	LUZP2	ESR1	[activation]
15546	CAMK2B	SEMA4G	[activation]
15547	RAP1A	TNFRSF10C	[activation]
15548	EIF1AX	TRAF6	[activation]
15549	LCK	GAB1	[activation]
15550	VCAM1	GNB1	[activation]
15551	NOTCH1	IKZF1	[activation]
15552	SREBF2	ABLIM1	[activation]
15553	ERBB3	RGS4	[activation;inhibition]
15554	RAPGEF2	SLC25A11	[activation]
15555	AKT2	CLIP3	[activation]
15556	MSX1	SP1	[activation]
15557	PRKCD	ADRA2A	[activation]
15558	CALM1	ADRB2	[activation]
15559	CSNK1E	DBNDD2	[activation]
15560	YWHAZ	RAP1GAP2	[activation]
15561	GDI2	SMURF1	[inhibition]
15562	LYPD3	SMAD4	[activation]
15563	CTSB	CDKN1A	[activation]
15564	SNX7	GRB2	[activation]
15565	MIF	TNFRSF14	[activation]
15566	ITCH	YAP1	[activation]
15567	GRIA2	REST	[activation]
15568	HSD3B7	ICAM1	[activation]
15569	PPM1A	ARRB2	[activation]
15570	RPA2	TIMELESS	[activation]
15571	IKBKE	GMFB	[activation]
15572	PDPK1	FAM213B	[activation]
15573	MDM2	G3BP1	[activation]
15574	HERC1	ARF1	[activation]
15575	YWHAZ	PAK1	[activation]
15576	POM121	PAXIP1	[activation]
15577	MMP3	SERPINE1	[activation]
15578	AURKA	PLK3	[activation]
15579	POU4F1	ESR1	[activation]
15580	MLH1	SMAD1	[activation]

15581 POLR2B BARD1 [activation]
15582 PTGS2 SUMO1 [activation]
15583 ARHGAP26 INSR [activation]
15584 VAV1 MAPK1 [activation]
15585 RASSF6 SAV1 [activation]
15586 TRIM28 H3F3A [activation]
15587 PIGR STK4 [activation]
15588 CREB3L1 CREM [inhibition]
15589 PTEN MAST1 [activation]
15590 MTM1 KMT2A [activation]
15591 CASP6 DSP [activation]
15592 DHX15 CD81 [activation]
15593 OBSL1 STAG2 [inhibition]
15594 GABARAPL2 PRKDC [activation]
15595 SLC35G2 SEMA3F [activation]
15596 ENAH MYO10 [activation]
15597 IRS4 P4HA1 [activation]
15598 OSBPL11 VAPB [activation]
15599 TGFBR2 NRP2 [activation]
15600 APOC1 APOA1 [inhibition]
15601 PAFAH1B2 PAFAH1B3 [activation;inhibition]
15602 SLC9A3R2 PIK3CA [activation]
15603 CCND1 SMAD1 [activation]
15604 SMAD2 DNAJA2 [inhibition]
15605 MAPK6 TTK [activation]
15606 TERF1 RIC8A [activation]
15607 TGFBR2 ARHGEF6 [activation]
15608 RALBP1 RAC1 [activation]
15609 ASB2 JAK2 [activation]
15610 MAPK3 TTYH3 [activation]
15611 TP53BP1 CDC27 [activation]
15612 SMAD4 STK11IP [activation]
15613 CDK2 PPP1CA [activation]
15614 TFCP2 MAPK14 [activation]
15615 SMAD3 HDAC4 [activation]
15616 CDH1 YES1 [activation]
15617 MAFA MAPK14 [activation]
15618 MAPK3 CREBBP [activation]
15619 SREBF2 PIAS1 [activation]
15620 MAPK8 WDR62 [activation]
15621 C6orf47 ENO2 [activation]
15622 STK11IP STK11 [activation]
15623 YWHAB GEM [activation]
15624 FZR1 MDC1 [activation]
15625 PPP2R2D JUN [activation]
15626 PSEN1 GSAP [activation]
15627 GATA3 RARA [inhibition]
15628 MYC AFAP1 [activation]
15629 CDK2 GSTM2 [activation]
15630 SH3GL2 IGHM [activation]
15631 TP53 WDR5 [activation]
15632 SMARCA2 STAT1 [activation]
15633 WNK1 RANBP9 [activation]
15634 AR HCK [activation]
15635 CCDC67 GEM [activation]
15636 WASH1 EXOC1 [activation]
15637 ITK BTK [activation]
15638 MS4A1 FMNL1 [activation]
15639 LTBP3 TGFB2 [activation]
15640 SIKE1 STK24 [activation]
15641 APC TAF1 [activation]
15642 NXF1 YWHAZ [activation]
15643 IFI35 MAPK1 [activation]
15644 YWHAG FAM13B [activation]
15645 ACACB HLCS [activation;inhibition]
15646 RPS6KB1 MAPK8 [activation]
15647 KLHL2 WNK1 [activation]
15648 IRS1 DVL2 [activation]
15649 HSPA8 GRB2 [activation]
15650 DAPK3 HSP90AB1 [activation]
15651 ARAF PPP6C [inhibition]
15652 YWHAZ ASS1 [activation]
15653 LUZP4 SRPK2 [activation]
15654 DAB2 LDLR [activation]
15655 GNAQ NT5C3A [activation]
15656 VCAM1 PICALM [activation]

15657	CRKL	CRK	[activation]
15658	HSP90AB1	ACVR2B	[activation]
15659	CSNK2A2	SNCA	[activation]
15660	KCNA4	INADL	[activation]
15661	TP53	USP42	[activation]
15662	MDM2	POLE	[activation]
15663	CDC23	PTBP3	[activation]
15664	DLGAP1	CTNNB1	[activation]
15665	ARF6	EIF4G1	[activation]
15666	FST	BMPR2	[activation]
15667	TP53BP2	NXF1	[activation]
15668	HSPB1	ASB6	[activation]
15669	LIG3	JTB	[activation]
15670	RRP12	FOS	[activation]
15671	CDK6	CCNE1	[activation]
15672	SPP1	SNAPIN	[activation]
15673	GRM5	PRKCZ	[activation]
15674	PAK1	LYN	[activation]
15675	MPP3	RRAGC	[activation]
15676	PRKAA1	HSP90AA1	[inhibition]
15677	RAC1	LTBP3	[activation]
15678	ZRANB1	TRAF6	[activation]
15679	PYHIN1	LIG3	[activation]
15680	SRY	SLC9A3R2	[activation]
15681	ZC3H10	DAB1	[activation]
15682	RAE1	NUPL2	[activation]
15683	JUP	CDK5RAP3	[activation]
15684	CTSE	INS	[activation]
15685	PAXIP1	RLTPR	[activation]
15686	ICT1	RPUSD4	[activation]
15687	GRB2	SYNJ1	[activation]
15688	ITSN2	ROCK1	[activation]
15689	RASSF10	S100A14	[inhibition]
15690	TBC1D22B	PNMA5	[activation;inhibition]
15691	FAM154A	CRK	[activation]
15692	FKBPL	AAR2	[activation]
15693	PPP2R5A	MAPT	[activation]
15694	RGS6	GNB4	[activation;inhibition]
15695	TNIK	TRAF6	[activation]
15696	USHBP1	CCDC148	[activation]
15697	PRMT5	PAXIP1	[activation]
15698	PLCG1	GNB2L1	[activation]
15699	RPS6KB1	EIF3F	[activation]
15700	SMPD1	ANXA7	[activation]
15701	NFKBIA	TGM2	[activation]
15702	CAPN1	PALB2	[activation]
15703	RUNX1	CBFB	[activation]
15704	ILK	TMSB4X	[activation]
15705	MEIS1	CRTC2	[activation]
15706	CTNND1	YWHAG	[activation]
15707	YWHAB	IRS1	[activation]
15708	GAD1	PPP2CA	[inhibition]
15709	NEDD1	HNRNPA1	[activation]
15710	MAP2K4	FLNC	[activation]
15711	CENPT	DHX29	[activation]
15712	EIF2B1	ADRA2B	[activation]
15713	HLA-B	CSF1	[activation]
15714	FRS2	NTRK3	[activation]
15715	POLA2	CRELD1	[activation]
15716	CDC27	FZR1	[inhibition]
15717	STAT3	FHL2	[activation]
15718	APP	DNAJB1	[inhibition]
15719	CDK5RAP2	CDK5R1	[activation]
15720	CITED2	TBP	[activation]
15721	BMPR2	UBC	[activation]
15722	RAB8B	CDH2	[activation]
15723	MICALL1	RAB10	[activation]
15724	CD81	HIST1H2BB	[activation]
15725	CALM1	OBSCN	[activation]
15726	ARHGEF11	PLXNB2	[activation]
15727	PAX8	CLSTN1	[activation]
15728	MKNK1	EIF4G3	[activation]
15729	FZR1	FBXO5	[inhibition]
15730	GRB2	PANX2	[activation]
15731	MYL12B	DAPK1	[activation]
15732	AR	CASP8	[activation]

15733	PRKCH	HSP90AB1	[activation]
15734	NFATC2	SUV39H1	[activation]
15735	SATB1	PIAS1	[inhibition]
15736	SRPK1	SFMBT1	[activation]
15737	TNNT1	NFE2L2	[activation]
15738	TDRD1	PLCG1	[activation]
15739	APLP2	CDK1	[inhibition]
15740	CRK	ERBB4	[activation]
15741	FGFR1	BNIP2	[activation]
15742	PTPN11	MPL	[activation]
15743	SGTA	SERPINE1	[inhibition]
15744	SDC1	PTN	[activation]
15745	SUMO4	STAT1	[activation]
15746	TGIF1	SMAD3	[inhibition]
15747	FYN	BRPF3	[activation]
15748	ARVCF	CDH1	[activation]
15749	MAP4K4	PAXIP1	[activation]
15750	YWHAE	SLC8A3	[activation]
15751	SMC4	RPA1	[activation]
15752	CDC14B	SKP2	[inhibition]
15753	CHEK2	KAT2B	[activation]
15754	PAFAH1B3	GDF9	[activation]
15755	HNF4G	NSD1	[activation]
15756	LTB	TNFRSF1A	[activation]
15757	KNG1	PREP	[activation]
15758	CRKL	MAPK1	[activation]
15759	SH3GL2	PAXIP1	[activation]
15760	CFHR2	APP	[activation]
15761	PLK1	GORASP1	[activation]
15762	CDK4	MCC	[inhibition]
15763	DAPK3	ATF4	[activation]
15764	UBE2E3	ARRDC1	[activation]
15765	DCC	TBC1D2	[activation]
15766	EIF1	APP	[activation]
15767	TP53	MPHOSPH6	[activation]
15768	CEBPE	ATF4	[activation]
15769	TRIM28	HLA-B	[activation]
15770	KLRB1	PTPN6	[activation;inhibition]
15771	RORC	RORB	[inhibition]
15772	PLXNB2	MET	[activation]
15773	GRIP1	RXRA	[inhibition]
15774	NOTCH2NL	FASLG	[activation]
15775	ITGB4	ERBB2	[activation]
15776	PCSK1	CTNNB1	[activation;inhibition]
15777	SPOP	GLI2	[inhibition]
15778	SYK	ITGA4	[activation]
15779	EDA2R	CASP10	[activation]
15780	TRAF2	RHEB	[activation]
15781	SERPINE1	F2	[inhibition]
15782	PLK1	CSN1S1	[activation]
15783	STX5	APLP1	[activation]
15784	RXFP3	RLN3	[activation]
15785	NXF1	NME4	[activation]
15786	AGAP8	HSPB1	[activation]
15787	RRM2B	ATM	[activation]
15788	XIAP	TBK1	[inhibition]
15789	HDAC4	BCL6	[activation]
15790	LYN	CDK2	[activation]
15791	EHBP1	EHD1	[activation]
15792	CDKN1A	CIZ1	[activation;inhibition]
15793	RAC1	PRKCD	[activation]
15794	RPUSD2	GRB2	[activation]
15795	IRF2	HMGB1	[activation]
15796	MLH1	ABHD16A	[activation]
15797	ABI1	HSPA8	[activation]
15798	WNK1	HDAC9	[activation]
15799	ESR2	WIPI1	[activation]
15800	ACAP3	TAB1	[inhibition]
15801	LYN	FCAR	[activation]
15802	YWHAB	NF1	[activation]
15803	SIGIRR	IL1R1	[activation]
15804	DCN	COL6A1	[activation]
15805	FSCN1	PRKCD	[activation]
15806	IL32	ZFPM2	[activation]
15807	GRB2	KRT33B	[activation]
15808	CDK1	PPP2R1B	[activation]

15809	CDC42BPA	PPP1R12C	[activation]
15810	BRCA2	KAT2B	[activation]
15811	EPOR	LYN	[activation]
15812	FAN1	KPNB1	[activation]
15813	SMAD3	RIOK2	[activation]
15814	MC3R	POMC	[activation]
15815	SMARCB1	BCL7C	[activation]
15816	CRK	ASAP1	[activation]
15817	RASA1	CD5	[activation]
15818	TGFBR1	GNB2	[activation]
15819	GNAS	MSANTD3	[activation]
15820	PTK2	GIT1	[activation]
15821	HSP90AB1	PTGES3	[activation]
15822	ATR	MCM2	[activation]
15823	LILRB4	PTPN11	[activation]
15824	HDAC1	IFRD1	[activation]
15825	PRKCB	ITGB7	[activation]
15826	PAK2	MAPK1	[activation]
15827	BCL6	UTP6	[activation]
15828	GET4	AKT1	[activation]
15829	MTA2	APPL1	[activation]
15830	YWHAE	TLK1	[activation]
15831	BTK	MAL	[activation]
15832	EEPD1	KCNIP4	[activation]
15833	PRKCI	DUSP1	[activation]
15834	PTPRF	RET	[activation;inhibition]
15835	UBE2I	SOX6	[activation]
15836	PPP3CB	MYOZ3	[activation]
15837	RCN1	JAK2	[activation]
15838	NFKBIA	HDAC4	[activation]
15839	RHOA	KCNA2	[activation]
15840	YES1	C1orf94	[activation]
15841	GNB2	GNAI1	[activation]
15842	STK4	CALML5	[activation]
15843	CENPJ	PLK2	[activation]
15844	KLF6	E2F1	[activation]
15845	MAX	SPAG9	[inhibition]
15846	TNK1	YWHAG	[activation]
15847	NEDD4	CAMK4	[inhibition]
15848	PLCG2	HCK	[activation]
15849	ICAM1	HIST1H2BH	[activation]
15850	PAK1	FOXL2	[activation]
15851	BMP4	CHRDL1	[activation]
15852	GRB14	DAPK3	[activation]
15853	CAMK2G	NR1H2	[activation]
15854	ZFYVE9	PPARG	[activation]
15855	WFIKKN1	TGFB1	[inhibition]
15856	SAV1	RASSF1	[inhibition]
15857	NFKBIA	CSNK1A1	[activation]
15858	GAPDH	GSK3B	[inhibition]
15859	HSP90AA1	CAMK2A	[activation]
15860	NUP93	ITGA4	[activation]
15861	YWHAQ	GCN1L1	[activation]
15862	MYC	DNAJB12	[inhibition]
15863	RAD51	PALB2	[activation]
15864	STX4	GIMAP5	[activation]
15865	MAPK9	STAT3	[activation]
15866	CSF1R	PIK3R1	[activation]
15867	CYTIP	CYTH2	[activation]
15868	SAA4	IGHG1	[activation]
15869	PTN	ITSN2	[activation]
15870	APP	BACE2	[activation]
15871	SORT1	SORCS1	[activation]
15872	CUX1	RECQL5	[activation]
15873	TRAF6	UBE2D3	[activation]
15874	STX6	MOV10	[activation]
15875	STX5	MAGEA6	[activation]
15876	SSTR4	SST	[activation]
15877	LYN	NFYB	[activation]
15878	PRPF6	WHSC1	[activation]
15879	HNF4A	TP53	[activation]
15880	DHX37	PLXNA2	[activation]
15881	RAC1	TUBA4A	[activation]
15882	BID	BAG3	[activation]
15883	VCAM1	S100A7	[activation]
15884	RASA1	BCR	[activation]

15885	THBS1	TGM2	[activation]
15886	CHUK	MYH14	[activation]
15887	NBN	RICTOR	[activation]
15888	EEA1	RAB5C	[activation]
15889	KIT	YES1	[activation]
15890	YWHAG	ERC1	[activation]
15891	CSK	SRCIN1	[activation]
15892	MASP1	GADD45A	[activation]
15893	RNASEL	IQGAP1	[activation]
15894	CAST	CASP3	[activation]
15895	CREBBP	HIPK2	[activation]
15896	PGR	NR0B1	[activation]
15897	MECOM	EHMT2	[activation]
15898	CRKL	LYN	[activation]
15899	XPO5	NOTCH1	[activation]
15900	MAPK8	EZR	[activation]
15901	ITSN1	PICALM	[activation]
15902	DHPS	NUDT18	[activation]
15903	GABARAP	GABARAPL2	[activation]
15904	HLA-B	SSSCA1	[activation]
15905	ECE2	CASP10	[activation]
15906	TNFRSF14	GET4	[activation]
15907	ABHD16A	IFITM1	[activation]
15908	DAB2	DVL1	[activation]
15909	VPS52	STX6	[activation]
15910	CDKN2AIP	MDM2	[activation]
15911	KIAA1377	MAPK9	[activation]
15912	NOL6	OBSL1	[activation]
15913	OPRM1	CALM1	[activation]
15914	UBE2E2	RNF5	[activation]
15915	IGHG2	USF2	[activation]
15916	MAPK4	ERC1	[activation]
15917	HTR1A	GABBR2	[activation]
15918	UBE2L6	RUNX1	[activation;inhibition]
15919	GNB2L1	VCAM1	[activation]
15920	EPB41L5	ESR2	[inhibition]
15921	ARF6	NCAPG	[activation]
15922	SRC	SH3BP2	[activation]
15923	MLH1	PSD3	[activation]
15924	PLCG1	NELL1	[activation]
15925	YWHAB	PTPN13	[activation]
15926	GLI2	ZIC1	[activation]
15927	HSPB1	IARS2	[inhibition]
15928	GSK3B	PIM2	[inhibition]
15929	HLA-B	RPA3	[activation]
15930	RPTOR	HIF1A	[activation]
15931	CASP8AP2	CALM1	[activation]
15932	XIAP	IRF3	[inhibition]
15933	EIF3I	ARF6	[activation]
15934	STX11	TBK1	[activation]
15935	CDH2	SKP1	[activation]
15936	SMAD1	PUM1	[activation]
15937	FXYD2	ATP1A1	[activation]
15938	ACVR1B	SMAD7	[activation]
15939	HOMEZ	PRKAA1	[inhibition]
15940	MYO1C	ESR1	[activation]
15941	DKC1	MCM7	[activation]
15942	LAT	PIK3R1	[activation]
15943	SOCS6	MET	[inhibition]
15944	PSEN2	FHL2	[activation]
15945	MAPK4	HSP90AB1	[activation]
15946	F2R	PDCD6IP	[activation]
15947	HNRNPA0	RPA3	[activation]
15948	FBXO11	MIF	[activation]
15949	GLI1	DYRK1A	[activation]
15950	ABI3	PAK1	[activation]
15951	CSK	CD44	[activation]
15952	RHPN2	TRAF6	[activation]
15953	AZI2	TRAF3	[activation]
15954	CASP8	GRIA1	[inhibition]
15955	STK16	HNF4A	[activation]
15956	GSK3B	DNAJC13	[activation;inhibition]
15957	FLT1	TMCO4	[activation]
15958	ERBB2IP	STAT3	[activation]
15959	INSIG2	AMFR	[activation]
15960	ESR1	SMAD2	[activation]

15961	SUMO3	TRDN	[activation]
15962	TRAF6	MTOR	[activation]
15963	GRK1	CALM1	[activation]
15964	GNAS	CAV3	[inhibition]
15965	CDX2	SMARCA4	[activation]
15966	EIF3L	LRRK2	[activation]
15967	NUP214	RAE1	[activation]
15968	SOS1	SH3BP5	[activation]
15969	CD2	CD59	[activation]
15970	ANAPC15	CDC27	[activation]
15971	KDM6B	WAS	[activation]
15972	METTL23	BCL2L1	[activation]
15973	APP	CLDN20	[activation]
15974	HIST1H4A	PAK1	[activation]
15975	CREBBP	SMARCB1	[activation]
15976	TTI1	RICTOR	[activation]
15977	MAPK9	GRB2	[activation]
15978	APP	PLEKHG2	[activation]
15979	SF3B1	ARF6	[activation]
15980	CD81	EEF1D	[activation]
15981	YWHAZ	EFNB3	[activation]
15982	GTF2A2	GTF2A1L	[activation]
15983	YWHAG	KIF23	[activation]
15984	FTSJ3	MAGEB4	[activation]
15985	PTK2	NEO1	[activation]
15986	HTR2A	NME3	[activation]
15987	GRB2	FGD6	[activation]
15988	DPY30	OGT	[activation]
15989	SH2B2	SORBS1	[activation]
15990	AKT1	HSPB1	[activation]
15991	CAMK2A	YWHAQ	[activation]
15992	DHX15	ITGA4	[activation]
15993	BCAS3	KRAS	[activation]
15994	SGK1	DDX6	[activation]
15995	PTPRA	PRKCD	[activation]
15996	TGFB1	BUB1	[activation]
15997	GNG12	BLK	[activation]
15998	CRK	PPP1CA	[activation]
15999	BMPR2	ACVR1	[activation]
16000	GABRR1	PRKACA	[activation]
16001	TYRO3	FYN	[activation;inhibition]
16002	FGR	KIT	[activation]
16003	ATF2	PGK1	[activation]
16004	FADD	DAPK1	[activation]
16005	MC4R	ADRBK1	[activation]
16006	TGM1	ALOXE3	[activation]
16007	APP	LRP1	[activation]
16008	HMGN1	CHEK2	[activation]
16009	CCNB1	PCNA	[inhibition]
16010	SMAD3	CDC27	[activation]
16011	INSR	JAK1	[activation]
16012	PRKACA	ESR1	[activation]
16013	VTN	IGF2	[activation]
16014	TXNDC5	TRIM42	[activation]
16015	AMER2	APC	[inhibition]
16016	HNRNPL	VCAM1	[activation]
16017	FBXO25	HSPA2	[activation]
16018	SYK	IL17RA	[activation]
16019	DVL3	RASSF10	[activation]
16020	CALM1	CBL	[activation]
16021	ESR1	RPS6KA3	[activation]
16022	KIAA1737	NXF1	[activation]
16023	IL1B	ADRB2	[activation]
16024	PRKACB	PRKAR1B	[activation]
16025	CHGB	RAC2	[activation]
16026	ROCK1	NXF1	[activation]
16027	WNT16	BCL6	[activation]
16028	STAT1	MTOR	[activation]
16029	PRNP	TBPL1	[activation]
16030	MAPT	RPS6KB1	[activation]
16031	C21orf58	GRB2	[activation]
16032	RPA3	PSIP1	[activation]
16033	PLK1	CCDC115	[activation]
16034	FHL2	ZFYVE9	[activation]
16035	PIK3R2	ABL1	[activation]
16036	CXCR2	GPRASP1	[activation]

16037	PTPN6	FCRL3	[activation;inhibition]
16038	BCL2L2	BAX	[inhibition]
16039	ADAMTS4	SRPX2	[activation]
16040	DCC	C9orf156	[activation]
16041	EHMT2	EHMT1	[activation]
16042	CDC42	TNF	[activation]
16043	ITGA4	HIST1H2AG	[activation]
16044	CTSG	GP1BA	[inhibition]
16045	CHEK1	APP	[activation]
16046	TRRAP	E2F1	[activation]
16047	MAP4K1	LYN	[activation]
16048	ENO1	ARPC4	[activation]
16049	ABCA1	SNTA1	[activation]
16050	MPP2	PLK1	[activation]
16051	MRGPRX1	GNB1	[activation]
16052	HGS	JAKMIP2	[activation]
16053	TAOK3	HSP90AB1	[activation]
16054	PRKCD	DAB2	[activation]
16055	RAD21	WNT2B	[activation]
16056	MAPT	GSK3B	[activation]
16057	FBXW7	AURKB	[activation]
16058	NXF1	ZBED2	[activation]
16059	GNA12	IL3RA	[activation]
16060	VCAM1	PIK3R2	[activation]
16061	RFC4	MYC	[activation]
16062	NXT1	NXF1	[activation]
16063	MCM2	MDM2	[activation]
16064	SMU1	GRB2	[activation]
16065	WDR27	ASNS	[activation]
16066	VCAM1	PDHA1	[activation]
16067	P2RY1	ADORA1	[activation]
16068	RPS25	FOS	[activation]
16069	EXOC7	ARFGEF2	[activation]
16070	ERBB2	HSP90AB1	[activation]
16071	USF1	KAT2B	[activation]
16072	CSNK1G2	PER1	[inhibition]
16073	PPP2R2B	SMC4	[activation]
16074	LIPE	MAPK3	[activation]
16075	ANXA5	TUBGCP3	[activation]
16076	CSK	PTPRB	[activation]
16077	PHKG1	PHKG2	[activation;inhibition]
16078	NOTCH2	IL24	[activation]
16079	CDKN1A	FLAD1	[activation]
16080	CDC23	UBE2S	[activation]
16081	RBBP5	CTNNB1	[inhibition]
16082	ERBB3	FER	[activation;inhibition]
16083	COL9A3	EGFR	[activation]
16084	ZNF747	SUFU	[activation]
16085	STAT5B	CD247	[activation]
16086	EIF4A2	EIF3D	[activation]
16087	APP	C10orf88	[activation]
16088	CD247	TFRC	[activation]
16089	SMC2	HIST2H2AC	[activation]
16090	C1QTNF9	RDX	[activation]
16091	CASP3	UBE4B	[activation]
16092	STAT5B	STAT1	[activation]
16093	ANAPC15	ANAPC2	[activation]
16094	ESR2	PIK3R4	[activation]
16095	EGFR	PIK3C2B	[activation]
16096	ZAP70	ASB3	[activation]
16097	GATA4	KLF13	[activation]
16098	CRKL	NXF1	[activation]
16099	PRKCD	ITGB7	[activation]
16100	EHMT1	HDAC2	[activation]
16101	RAN	SMAD2	[activation]
16102	PML	FAS	[inhibition]
16103	INADL	LATS1	[inhibition]
16104	RPS6KA3	BAD	[activation;inhibition]
16105	CHEK1	RB1	[activation;inhibition]
16106	PIK3R3	DRAP1	[activation]
16107	TRPC1	BMPR2	[activation]
16108	NUP62CL	APP	[activation]
16109	MRPL13	ICT1	[activation]
16110	CDK8	TP53	[activation]
16111	RARA	NR1H2	[activation]
16112	PRMT1	TNIK	[activation]

16113	RUNX1	SMARCC1	[activation]
16114	LFNG	APP	[activation]
16115	NFKB1	NCOA1	[activation]
16116	NUDC	BTRC	[activation]
16117	ZFP36	NUP214	[activation]
16118	CCR8	TPST1	[activation]
16119	EPAS1	F12	[activation]
16120	MDM4	E2F1	[activation;inhibition]
16121	GAB2	GRAP	[activation]
16122	PRKCI	FRS3	[activation]
16123	SH3RF1	RAC1	[activation]
16124	YWHAZ	EPB41L3	[activation]
16125	CDC5L	EHMT1	[activation]
16126	HDAC1	PPA1	[activation]
16127	HSPA4	PRKCD	[activation]
16128	EGFR	GAB2	[activation]
16129	PCK1	BSG	[activation]
16130	MAP3K1	NFKBIA	[activation]
16131	ARHGAP15	RAC1	[activation]
16132	YWHAG	IL7R	[activation]
16133	MAPK1	GMFB	[activation]
16134	ZC3H4	OBSL1	[inhibition]
16135	OBSL1	RAD50	[activation]
16136	CAPZB	LRRK2	[activation]
16137	RORC	EIF4EBP1	[inhibition]
16138	STAT1	PRMT3	[activation]
16139	LAMB1	TGFB1	[activation]
16140	TP53	IRF7	[activation]
16141	PFN1	RAD52	[activation]
16142	CCL7	MMP1	[activation]
16143	YWHAB	ZAK	[activation]
16144	IGF1	IGFBP1	[activation]
16145	ZYX	BCAR1	[activation]
16146	ORM2	STK3	[activation]
16147	DAB2	NECAP1	[activation]
16148	EBLN2	STK16	[activation]
16149	BPTF	MYC	[activation]
16150	GRB2	MAP2	[activation]
16151	GOSR1	YKT6	[activation]
16152	PPP6C	EGFR	[activation]
16153	EGFR	MAP4K1	[activation]
16154	HLA-B	VIPR1	[activation]
16155	YWHAG	MAPKAP1	[activation]
16156	CYBA	MYC	[activation]
16157	CASP6	NUCB1	[activation]
16158	YAP1	SLC9A3R2	[activation]
16159	CDK5RAP3	MME	[activation]
16160	RGS14	PRKACA	[activation;inhibition]
16161	MDM2	HMOX2	[activation]
16162	FAM184A	PYCARD	[inhibition]
16163	DOK2	SHC1	[activation]
16164	FGFR4	DIRAS3	[activation]
16165	NR2F2	NSD1	[activation]
16166	F12	UBE2D2	[activation]
16167	ESR2	KRI1	[activation]
16168	EXOC4	CDC5L	[activation]
16169	PRKCZ	CASP7	[activation]
16170	MSTN	SGTA	[activation]
16171	CD81	INPP4A	[activation]
16172	SMARCA2	NR3C1	[activation]
16173	EFNA4	MAP2K4	[activation]
16174	NOTCH1	DHX15	[activation]
16175	EP300	BANP	[activation]
16176	CRK	NCK1	[activation]
16177	LTBP3	PIK3R2	[activation]
16178	FAM124A	ROCK1	[activation]
16179	NR1H3	HSP90AA1	[activation]
16180	GART	ARF6	[activation]
16181	G3BP1	NUP62	[activation]
16182	PPP2CB	AXIN2	[inhibition]
16183	MDM2	PTK2	[activation]
16184	PRKCZ	LLGL2	[activation]
16185	BRCA1	TP53	[activation]
16186	DHX33	NUDCD1	[activation]
16187	VRK2	MAP2K7	[activation]
16188	FOXO1	DYRK1A	[activation]

16189	BCL2L14	BCL2L1	[activation]
16190	STAT3	BRCA1	[activation]
16191	MAP2K6	RELA	[activation]
16192	FAS	DAXX	[inhibition]
16193	PPP1CC	CDK2	[inhibition]
16194	GRB2	SH2D5	[activation]
16195	RAB13	SMAD4	[activation]
16196	OBSL1	XPO1	[activation]
16197	ATG5	HPRT1	[activation]
16198	TRIB3	GRN	[activation]
16199	EP300	KLF2	[activation]
16200	PRNP	WIBG	[activation]
16201	UBE2D2	STK11	[activation]
16202	RICTOR	FLNA	[activation]
16203	USHBP1	EXOC7	[activation]
16204	CD86	ECE2	[activation]
16205	GOLGA2	NOS3	[activation]
16206	BET1	CCDC155	[activation]
16207	CAV1	HRAS	[activation]
16208	PLCG1	PTPRJ	[activation]
16209	TRAF3	TNFRSF13C	[activation]
16210	ERBB2IP	SMAD2	[inhibition]
16211	SMURF1	RRP9	[inhibition]
16212	SCRIB	MAPK1	[activation]
16213	PPP2R5A	SGOL1	[activation]
16214	NFE2L2	ENC1	[activation]
16215	CRB2	CTTN	[activation]
16216	SHKBP1	YWHAG	[activation]
16217	THEM6	ERBB2	[activation]
16218	DNM2	ITSN2	[activation]
16219	TGFB1	EGFR	[activation]
16220	FTSJ3	OAS3	[activation]
16221	CCNA2	BRCA2	[activation]
16222	STK4	LCN1	[activation]
16223	MDM4	YWHAZ	[activation;inhibition]
16224	MOCS3	MOCS2	[activation]
16225	HIST2H2AC	PRMT7	[activation]
16226	CD37	SYK	[activation]
16227	PGR	SP1	[activation]
16228	MYD88	POLR1C	[activation;inhibition]
16229	AP1M2	A2M	[inhibition]
16230	PML	SKIL	[activation]
16231	COL9A1	MAG	[activation]
16232	RASD2	ACVR1	[activation]
16233	STRN4	STK3	[activation]
16234	EIF4A3	ACIN1	[activation]
16235	ANXA2	CD4	[activation]
16236	VAMP2	VAPB	[activation]
16237	TFAP2A	EPAS1	[activation]
16238	CRY2	SKP1	[inhibition]
16239	EFHD2	RAD51D	[activation]
16240	AVPR2	GNAS	[activation]
16241	KIAA1033	FAM21C	[activation]
16242	GRIK2	PRKAA1	[inhibition]
16243	MDH2	ATF2	[activation]
16244	TAB1	HSPA4	[inhibition]
16245	CDK7	TP53	[activation]
16246	TRAF3	TLR4	[activation]
16247	EIF4A2	PIH1D2	[activation]
16248	BCR	HSPA8	[activation]
16249	CREM	CREB1	[inhibition]
16250	CNTF	ICA1	[activation]
16251	KLHL1	TGFBR1	[activation]
16252	HSPB1	SMARCA4	[activation]
16253	PTPN11	SLAMF6	[activation]
16254	JAK1	IFNAR2	[activation]
16255	NCKAP5	FYN	[activation;inhibition]
16256	CASP3	DBNL	[activation]
16257	TYK2	LYN	[activation]
16258	FTSJ1	YARS	[activation]
16259	IRF3	RELA	[activation]
16260	ELAVL1	MAP2K1	[activation]
16261	OBSL1	CDCA8	[activation]
16262	NXF1	KPNA2	[activation]
16263	MAPK1	VCAM1	[activation]
16264	MAPK1	MAP2K1	[activation]

16265	RNF41	RNF166	[activation]
16266	IRF1	NFKB1	[activation]
16267	CDCA8	NEK3	[activation]
16268	YWHAQ	BAX	[inhibition]
16269	RIMBP2	CACNA1A	[inhibition]
16270	LRPAP1	RTN4	[inhibition]
16271	BIRC5	JTB	[activation]
16272	MYO5A	GRIA2	[activation]
16273	PRKCZ	BCL10	[activation]
16274	DYSF	CAV3	[activation]
16275	VCAM1	ARF5	[activation]
16276	FZD2	ROR2	[activation]
16277	HSP90AB1	AIRE	[activation]
16278	CEBPB	CEBPD	[activation]
16279	RPA3	KIAA0196	[activation]
16280	APP	FMNL3	[activation]
16281	DOK1	RASA1	[activation]
16282	HSP90AB1	CHEK1	[activation]
16283	CSNK1E	ACACA	[activation;inhibition]
16284	PPP2CB	CDK17	[inhibition]
16285	GHR	SGTA	[activation]
16286	HSPA2	MEOX2	[activation]
16287	PDPK1	SRPK1	[activation]
16288	AURKB	ABR	[activation]
16289	CCNB1	TGFBR2	[inhibition]
16290	SRPK1	CACNB1	[activation]
16291	NXF1	ANAPC1	[activation]
16292	EP300	ATG7	[activation]
16293	TERF1	NBN	[activation]
16294	RASA1	NCK1	[activation]
16295	VCAM1	TAGLN2	[activation]
16296	LRRK1	GAK	[activation]
16297	STK3	HSPE1	[activation]
16298	PTK2B	STAP1	[activation]
16299	KNG1	CPN1	[activation]
16300	PTPN6	IRS4	[activation;inhibition]
16301	JUN	KMT2C	[activation]
16302	AKT1	FAM110C	[inhibition]
16303	ENO1	GRB2	[activation]
16304	NOS3	ACTB	[activation]
16305	EP300	IRF3	[activation]
16306	MAGEB2	MAD2L1	[inhibition]
16307	TRAF2	CCDC130	[activation]
16308	TP53	PRKRIR	[activation]
16309	CASP8	CNBP	[inhibition]
16310	PLK2	CASK	[activation]
16311	PLCG1	PPFIA4	[activation]
16312	CD3D	CD247	[activation]
16313	NCK2	BLNK	[activation]
16314	RABEP1	HUNK	[activation]
16315	TDG	RXRA	[inhibition]
16316	SMARCA4	EPAS1	[activation]
16317	SMAD1	NAT9	[activation;inhibition]
16318	IL2RB	FYN	[activation;inhibition]
16319	PAX8	PKM	[activation]
16320	CSN2	NEK8	[activation]
16321	NR5A1	GRB2	[activation]
16322	EPHB3	CRK	[activation]
16323	SNAP25	HGS	[activation]
16324	TAX1BP3	GATA1	[activation]
16325	APP	SSH3	[activation]
16326	JDP2	CREBBP	[activation]
16327	BAIAP2	SRPK2	[activation]
16328	OGT	SMURF1	[activation]
16329	GBF1	YWHAG	[activation]
16330	ITGA4	SRI	[activation]
16331	EGLN3	ADRB2	[activation]
16332	TP53	DGCR14	[activation]
16333	SETDB1	POLA2	[activation]
16334	RAC1	ARHGEF2	[activation]
16335	ADRA2A	GNG2	[activation]
16336	TAGLN2	ATG5	[activation]
16337	TFPI	MMP7	[inhibition]
16338	MACF1	CDC5L	[activation]
16339	JUN	NCOA1	[activation]
16340	PRKAA2	HNF4A	[activation]

16341	HSPA4	TGFBR1	[inhibition]
16342	RAC1	MAGI1	[activation]
16343	HRAS	AGTR1	[activation]
16344	SNCA	UBB	[activation]
16345	KLHL35	TGFBR1	[inhibition]
16346	CDK5	EGFR	[activation]
16347	ATR	FAM133B	[activation]
16348	JAK2	VAV1	[activation]
16349	RPA1	HNRNPL	[activation]
16350	FYN	DLGAP2	[activation]
16351	CREBBP	CREB1	[activation]
16352	LRRC47	LRRK2	[activation]
16353	PNP	HLA-B	[activation]
16354	G3BP1	RAD21	[activation]
16355	BCR	YWHAB	[activation]
16356	PRNP	TSLP	[activation]
16357	IRS4	PCK1	[activation]
16358	ATM	NABP2	[activation]
16359	HPN	HGF	[inhibition]
16360	BRAP	IKBKB	[activation]
16361	MYLPF	PPP2R4	[activation]
16362	KMT2A	HCFC2	[activation]
16363	UBE2E3	ITCH	[activation]
16364	LGALS9B	DAB1	[activation]
16365	TRPV4	LYN	[activation]
16366	ATF7	TAOK3	[activation]
16367	PHKG1	CTRC	[activation;inhibition]
16368	SHC1	PRKDC	[activation]
16369	STAT3	SUMO4	[activation]
16370	SRPK1	PELI2	[activation]
16371	MS4A2	PRKCD	[activation]
16372	CAND1	ENO2	[activation]
16373	PYCARD	CCDC135	[inhibition]
16374	MT1A	GNAI1	[activation]
16375	CDKN2A	UBE2A	[inhibition]
16376	PRKCD	CD34	[activation]
16377	FOS	CARM1	[activation]
16378	HSPA1L	YWHAZ	[activation]
16379	CBLB	ASAP2	[inhibition]
16380	DFFA	LRRK2	[activation]
16381	HNRNPA1	ICAM1	[activation]
16382	RNF41	ERBB4	[activation]
16383	RIPK2	HSPA2	[activation]
16384	METTL2B	PSEN2	[activation]
16385	SNW1	LRWD1	[activation]
16386	HSP90AA1	MDM2	[activation]
16387	SH3GL3	EGFR	[activation]
16388	PLEKHB1	TGFBR1	[activation]
16389	CD274	PTPN11	[activation]
16390	BCAR3	WHSC1L1	[activation]
16391	PIM2	SOCS1	[inhibition]
16392	SPP1	CTNNBL1	[activation]
16393	UBE2I	RPS6KA6	[activation]
16394	NXF1	SRBD1	[activation]
16395	GADD45G	GADD45A	[activation]
16396	RAD17	RAD50	[activation]
16397	BLNK	HSPH1	[activation]
16398	RPA1	RAD50	[activation]
16399	PRKRIP1	EIF2AK2	[inhibition]
16400	PKIG	PRKACA	[inhibition]
16401	HSP90AB1	EPHA4	[activation]
16402	WNK4	HSP90AB1	[activation]
16403	YAP1	EGFR	[activation]
16404	EFNA1	EEF1G	[activation]
16405	TFAP2A	EP300	[activation]
16406	BCL3	RELA	[activation]
16407	STK38	KMT2E	[activation]
16408	MDC1	VAMP5	[activation]
16409	RICTOR	MLST8	[activation]
16410	HBG1	APP	[inhibition]
16411	STK11	YWHAZ	[activation]
16412	MAPRE1	STIM1	[activation]
16413	WASF3	ACTR3	[activation]
16414	PLOD1	H2AFX	[activation]
16415	MCM2	P2RX4	[activation]
16416	MST4	STK24	[activation]

16417 GSK3B IGSF21 [inhibition]
16418 STAT5A TSLP [activation]
16419 PTTG1 DECR1 [inhibition]
16420 MLH1 PRKDC [activation]
16421 ADORA2A CYTH2 [activation]
16422 CHD8 CREB1 [inhibition]
16423 FTSJ1 ETFA [activation]
16424 MAPKBP1 RGS20 [activation;inhibition]
16425 SMARCA4 MYOD1 [activation]
16426 TP53 POLDIP2 [activation]
16427 EIF2AK4 TRAP1 [activation]
16428 PTPN12 INSR [activation]
16429 NXF1 IQGAP1 [activation]
16430 SMURF1 BMPR1A [inhibition]
16431 RPP38 FYN [activation]
16432 EGFR TPI1 [activation]
16433 PTPN11 PDCD1 [activation]
16434 NUDC POC1B [activation]
16435 EIF3B ICAM1 [activation]
16436 MC2R ASIP [inhibition]
16437 NTRK1 MAPK3 [activation]
16438 CDKN2A EIF1B [activation;inhibition]
16439 S1PR1 CAV1 [activation]
16440 DRD3 RDX [activation]
16441 NMI MYCN [activation]
16442 SRPK2 PRPF4B [activation]
16443 ACTB RAD52 [activation]
16444 EPB41L3 IGSF21 [activation]
16445 FBXO6 VDAC3 [inhibition]
16446 SLU7 TNF [activation]
16447 SORBS3 MAPK1 [activation]
16448 EFNA1 SUMO2 [activation]
16449 MYD88 SMURF1 [activation]
16450 CDC25C MAPK9 [activation]
16451 SERBP1 ARF6 [activation]
16452 RAD50 MYC [activation]
16453 CDC20 EIF2A [activation]
16454 KLHL11 NUDCD3 [activation]
16455 ZNF512B IL36RN [activation]
16456 NRG1 EGF [activation]
16457 CCDC8 TRAP1 [activation]
16458 HEMGN NRAS [activation]
16459 ACTA1 NCOA3 [activation]
16460 CDH24 PPP1R16A [activation]
16461 PAX8 CXCL9 [activation]
16462 PAK2 PIN1 [activation]
16463 DAPK3 TP53 [activation]
16464 YWHAZ ADAM22 [activation]
16465 BCL6 HNF1A [activation]
16466 DIAPH1 WWOX [activation]
16467 TAF1 MAX [activation]
16468 SMAD3 ACVR1B [activation]
16469 PTEN IL24 [activation]
16470 ANAPC1 CDC5L [activation]
16471 RAD21 PCNA [activation]
16472 PIK3R1 EEF1D [activation]
16473 SMAD2 TP73 [activation;inhibition]
16474 ABL1 FYB [activation]
16475 CDK11A GADD45A [activation]
16476 MYC NUP188 [activation]
16477 PTK2 ERBB3 [activation]
16478 ACTG1 LSP1 [activation]
16479 LATS1 SDCCAG3 [inhibition]
16480 CDC27 CREBBP [activation]
16481 AURKA MYCN [activation]
16482 ARF6 EMC1 [activation]
16483 SRRT NOTCH1 [activation]
16484 MCMBP CDC7 [activation]
16485 STOML1 E2F1 [activation]
16486 HIF1A NAA10 [activation]
16487 RAPGEF1 RRAS2 [activation]
16488 SST PDIA2 [activation]
16489 BTG2 PRKCA [activation]
16490 RAC1 HTT [activation]
16491 HERC5 CCNB1 [inhibition]
16492 IGF2BP3 TP53 [activation]

16493	ACTN1	ATF2	[activation]
16494	PRKDC	H2AFX	[activation]
16495	ANXA1	DDX17	[activation]
16496	CRK	SYN1	[activation]
16497	YWHAB	MAPT	[activation]
16498	CCL8	ACKR4	[activation]
16499	INSIG2	PSMA7	[activation]
16500	ITGA4	EIF4G1	[activation]
16501	CDH1	ANP32B	[activation]
16502	RHOJ	WASL	[activation]
16503	CALM1	RANBP2	[activation]
16504	EFNB2	HOMER1	[activation]
16505	MYC	XPO1	[activation]
16506	GHR	PTPN11	[activation]
16507	PTGES3	SIM2	[activation]
16508	TDG	DDX39B	[activation]
16509	HSPA4	BMX	[activation]
16510	RAN	OBSL1	[activation]
16511	PIAS2	TRAF2	[activation]
16512	RPA3	DHX36	[activation]
16513	TRAF3	SAR1A	[activation]
16514	TNK2	YES1	[activation]
16515	GSK3B	APP	[inhibition]
16516	H3F3A	ARRB1	[activation]
16517	CCNB1	ESPL1	[inhibition]
16518	SIRT1	RELA	[activation]
16519	NAGLU	FBXO6	[activation]
16520	PPP2CA	IER3	[activation]
16521	EPHA3	TP53	[activation]
16522	RASGRF1	TNK2	[activation]
16523	STX7	STX4	[activation]
16524	LTBP4	TGFB1	[inhibition]
16525	TKT	MAPK8	[activation]
16526	HDAC4	ATF3	[activation]
16527	MYZAP	CEBPG	[activation]
16528	DENND6B	MYC	[inhibition]
16529	HIST1H2AC	ITGA4	[activation]
16530	DOK1	GABARAPL2	[activation]
16531	MNT	MAX	[inhibition]
16532	MARS	RELA	[activation]
16533	SMAD4	FANCC	[activation]
16534	VPS35	HLA-B	[activation]
16535	UBC	CD2	[activation]
16536	RIPK2	HSP90AA1	[activation]
16537	BTBD10	LAMA5	[activation;inhibition]
16538	CRIM1	CACNA1A	[inhibition]
16539	HMCES	SRPK2	[activation]
16540	SLAIN2	SRPK2	[activation]
16541	PLCG2	DAPP1	[activation]
16542	CDKN1A	PIM1	[activation]
16543	PKP4	CDH5	[activation]
16544	DCD	EIF2B1	[activation]
16545	EIF2B1	GORASP2	[activation]
16546	MAPK9	DUSP8	[inhibition]
16547	GIMAP6	APP	[activation]
16548	ITGB1	MAP4K4	[activation]
16549	ADRA2A	ADRBK1	[activation]
16550	TNFRSF10B	TNFRSF1A	[activation]
16551	EPHA1	HSP90AB1	[activation]
16552	SRC	SRF	[activation]
16553	CHEK2	MRC1	[activation]
16554	HIF1A	ESRRA	[activation]
16555	S1PR4	GNA12	[activation]
16556	ELANE	SERPIND1	[inhibition]
16557	GRB2	WDR6	[activation]
16558	NXF1	EPS15L1	[activation]
16559	ACTB	AKT1	[activation]
16560	CDC5L	TWF1	[activation]
16561	SUMO1	EIF4A2	[activation]
16562	RAB5A	ANKFY1	[activation]
16563	APP	GUCY1B3	[activation]
16564	RBL2	TAF1	[activation]
16565	CASP6	SRP72	[activation]
16566	RAC1	IQGAP3	[activation]
16567	EP300	HIST2H2AC	[activation]
16568	PTTG1	TP53	[inhibition]

16569	LAMA5	LAMC1	[activation;inhibition]
16570	RASA1	MLH3	[activation]
16571	FAM98B	ZBTB8OS	[activation]
16572	RPA2	ATM	[activation]
16573	POLD4	POLE	[activation]
16574	FTL	KNG1	[activation]
16575	NCOA3	TP53BP1	[activation]
16576	IL1B	LYN	[activation]
16577	JUP	BTRC	[activation]
16578	OBSL1	CCDC8	[inhibition]
16579	ERBB2	SFRP4	[activation]
16580	SYK	MAL	[activation]
16581	CAMP	MAPK8	[activation]
16582	RAB11A	CHMP1B	[activation]
16583	CTNNA1	PSEN1	[activation]
16584	RASA1	G3BP1	[activation]
16585	EHMT1	TP53	[activation]
16586	CTNNB1	TP53BP2	[activation]
16587	TIFA	IRAK1	[activation;inhibition]
16588	EFNB2	RGS3	[activation]
16589	ITGA4	PCBP1	[activation]
16590	PRR20E	NCK2	[activation]
16591	KIR2DS3	TYROBP	[activation]
16592	SEC22A	MSR1	[activation]
16593	SP1	ARHGAP21	[activation]
16594	MAP3K10	DNM1L	[activation]
16595	NCOA1	CIITA	[activation]
16596	PPP3CA	RCAN2	[activation]
16597	RRN3	CCNE1	[activation]
16598	PFN1	DLG5	[activation]
16599	SIRT1	STK4	[activation]
16600	BUB3	SMAD3	[inhibition]
16601	YWHAZ	FOXO4	[activation]
16602	FOS	CCDC158	[activation]
16603	TNFRSF1B	CD14	[activation]
16604	DAB2	ITGB3	[activation]
16605	MYD88	MBIP	[activation;inhibition]
16606	HSPA5	TG	[activation]
16607	MSTN	APP	[activation]
16608	ERBB2	VAV2	[activation]
16609	CDK20	RAF1	[activation]
16610	TP53	TCEAL4	[activation]
16611	PTGER1	ADRB2	[activation]
16612	SNCA	BAD	[inhibition]
16613	PDX1	CREB1	[activation]
16614	AR	NCOA2	[activation]
16615	ANAPC5	ANAPC16	[activation]
16616	GAS7	APBB1IP	[activation]
16617	APP	AKT2	[activation]
16618	ADRA1A	ABR	[inhibition]
16619	CSF3R	FZR1	[activation]
16620	BUB1B	BAG2	[inhibition]
16621	SRC	DUSP15	[activation]
16622	PTP4A3	SMC2	[activation]
16623	EIF2AK2	RAC1	[activation]
16624	SLC2A1	PTK2B	[activation]
16625	PIK3R1	PTPN4	[activation]
16626	EPS15	CRKL	[activation]
16627	LGR4	GCN1L1	[activation]
16628	PAK1	H2AFX	[activation]
16629	TSHR	ADRB2	[activation]
16630	GRAP	KIT	[activation]
16631	DDX24	PRF1	[activation]
16632	FKBP2	BARD1	[activation]
16633	ASAP2	CTTN	[activation]
16634	MYB	PPM1K	[activation]
16635	ACTR2	OBSL1	[activation]
16636	LRRK2	EOGT	[activation]
16637	ERBB4	RASA1	[activation]
16638	NAT9	MAPK6	[activation;inhibition]
16639	MOAP1	BCL2L1	[activation]
16640	SSH1	ARPC2	[activation]
16641	AMOT	LATS1	[activation]
16642	GP1BB	YWHAZ	[activation]
16643	EGFR	NCDN	[activation]
16644	CSNK2A1	BID	[activation]

16645	TP53	SOCS1	[inhibition]
16646	CCDC155	PDGFRA	[activation]
16647	INS	CRYAB	[activation]
16648	INHBB	FNTA	[activation]
16649	LRP1B	SERPINE1	[inhibition]
16650	APP	GNAT2	[activation]
16651	NCAPH2	EGLN3	[activation]
16652	FGFR1	NCK2	[activation]
16653	MAPK8	TNFSF11	[activation]
16654	CREBBP	ANAPC2	[activation]
16655	PPP1R3D	YWHAZ	[activation]
16656	SRPK1	EIF3M	[activation]
16657	RRAS	ACY3	[activation]
16658	CSF2RB	FES	[activation]
16659	AURKA	GADD45A	[activation]
16660	NFKB1	HMGA2	[activation]
16661	BACH1	BARD1	[activation]
16662	NAA25	CDK2	[activation]
16663	LILRB2	HLA-F	[activation]
16664	GATA1	CCDC24	[activation]
16665	RRAGD	LAMTOR4	[activation]
16666	PIAS4	IRF3	[inhibition]
16667	TP53	TAF1B	[activation]
16668	ERBB3	JAK3	[activation]
16669	BRAF	LIMK1	[activation]
16670	SYN1	SNCA	[activation]
16671	GAPVD1	CBL	[activation]
16672	HSP90AB1	CCDC117	[activation]
16673	FBXO6	SCAMP3	[inhibition]
16674	CLSPN	RAD17	[activation]
16675	RPA1	TIMELESS	[activation]
16676	SKP2	CDK2	[activation]
16677	VCAM1	AK2	[activation]
16678	KLK6	FGA	[activation]
16679	PCDHA6	BRK1	[activation]
16680	AGTRAP	NKG7	[activation]
16681	YWHAH	CYFIP1	[inhibition]
16682	ERBB2	SOCS1	[inhibition]
16683	MDM2	NUDT9	[activation]
16684	CFLAR	TNFRSF10A	[inhibition]
16685	AURKB	KLHL9	[activation]
16686	YWHAZ	MAP3K2	[activation]
16687	SOCS2	SOCS3	[inhibition]
16688	TP53	EIF2S2	[activation]
16689	PPIL1	ITGA4	[activation]
16690	ARF1	CHRM3	[activation]
16691	PICALM	CDC5L	[activation]
16692	OBSL1	DDX10	[activation]
16693	RPS6KA1	FGFR1	[activation]
16694	SH2D2A	MAPK7	[activation]
16695	BRCA1	CDK1	[activation]
16696	DAPK3	MET	[activation]
16697	DAB1	CRKL	[activation]
16698	SGOL2	MAD2L1	[inhibition]
16699	CCNE1	BRCA2	[activation]
16700	UBE2K	DIABLO	[activation]
16701	PRTN3	RELA	[activation]
16702	TNFSF11	MMP1	[activation]
16703	CCDC36	CDC23	[activation]
16704	APOH	TP53	[activation]
16705	C8orf74	SUV39H1	[activation]
16706	PRKCD	AKT3	[activation]
16707	AKT1	BCL2L11	[activation]
16708	EIF4A1	PRMT1	[activation]
16709	APC	BAAT	[activation]
16710	YAP1	WBP1	[activation]
16711	UBASH3B	JAK2	[activation]
16712	REL	HSPA6	[inhibition]
16713	CACNA1A	CACNB1	[inhibition]
16714	HNRNPL	DAB2	[activation]
16715	ILK	SYNPO2	[activation]
16716	HIST1H1E	MAPK13	[activation]
16717	TCIRG1	TERF1	[activation]
16718	MDM2	RRM2B	[activation]
16719	MOV10	PALB2	[activation]
16720	MIF	FN1	[activation]

16721	MYO1C	USP20	[activation]
16722	TBK1	CYLD	[inhibition]
16723	BCL2L1	TMBIM6	[activation]
16724	BIRC3	UBE2V1	[activation;inhibition]
16725	AURKB	KLHL13	[activation]
16726	PLEKHG2	RAC1	[activation]
16727	PVRL2	TNFRSF12A	[activation]
16728	SUMO1	DVL3	[activation]
16729	CEP76	LATS1	[inhibition]
16730	PLD3	STK11	[activation]
16731	MYOG	PACRGL	[activation]
16732	F2	HGFAC	[activation]
16733	CDK5RAP2	STK36	[activation]
16734	ATF1	ESR1	[activation]
16735	CSK	TLR3	[activation]
16736	BCL6	CTNNB1	[activation]
16737	SRPK1	U2AF2	[activation]
16738	DEPTOR	MTOR	[activation]
16739	SYNGAP1	TRIP6	[activation]
16740	PRKCB	LMNB1	[activation]
16741	RAP1GDS1	EGFR	[activation]
16742	EGFR	NUP93	[activation]
16743	RNF146	APC	[inhibition]
16744	ACTR8	BMPR2	[activation]
16745	CCDC8	PRKDC	[activation]
16746	TSC1	TSC2	[activation;inhibition]
16747	TNFSF4	TRAF2	[activation]
16748	CDH1	PSMC3IP	[activation]
16749	NGFR	TRAF1	[activation;inhibition]
16750	APP	METTL2B	[activation]
16751	GRB2	RIT2	[activation]
16752	ITCH	CBL	[activation]
16753	FYN	IRS1	[activation]
16754	SETDB1	TOLLIP	[activation]
16755	DAB1	LDLR	[activation]
16756	TSC2	RABEP1	[activation;inhibition]
16757	ITGA4	PPP1CA	[activation]
16758	HSPA6	ADCK5	[activation]
16759	MAGED1	DLX5	[activation]
16760	SMAD2	FAM161B	[activation]
16761	MECR	PPARA	[activation]
16762	KIT	SLA2	[activation]
16763	KMT2A	HDAC1	[activation]
16764	BTK	VAV1	[activation]
16765	NFKBIB	PPARG	[activation]
16766	GTPBP1	APP	[activation]
16767	HSP90AB1	ACTG1	[activation]
16768	CIITA	MAPK1	[activation]
16769	ESR1	EIF4A1	[activation]
16770	SHC1	ACLY	[activation]
16771	RALA	EXOC6B	[activation]
16772	ACSL3	YES1	[inhibition]
16773	CCNH	GOLGA2	[activation]
16774	CAMK2A	CDK5R1	[activation]
16775	SAA1	COL4A1	[activation]
16776	CCDC8	CTNNB1	[activation]
16777	PRKCE	CFTR	[activation]
16778	BTRC	ATF4	[activation]
16779	PPP2R1A	PPP2R5B	[activation]
16780	HIST3H3	HSPA8	[activation]
16781	IKZF4	FOXP3	[activation]
16782	C4B	C2	[inhibition]
16783	MYOD1	HIST1H3A	[activation]
16784	HIST1H2BN	ITGA4	[activation]
16785	OBSL1	CORO1C	[activation;inhibition]
16786	KISS1	MMP9	[activation;inhibition]
16787	PRKDC	EIF2S2	[activation]
16788	HES5	STAT3	[activation]
16789	PTPN6	KIR2DL3	[activation;inhibition]
16790	RAB6B	ERC1	[activation]
16791	EPAS1	CNOT10	[activation]
16792	NARF	APP	[activation]
16793	PARD6A	PRKCZ	[activation]
16794	TNFRSF14	PER2	[activation]
16795	WAS	FYN	[activation]
16796	APP	NUAK2	[activation]

GRB2	DAG1	[activation]
MXD1	MAX	[inhibition]
RBPJ	RUNX3	[inhibition]
SMAD2	EIF3D	[activation]
CCL13	APP	[activation]
CHD1L	CTCF	[activation]
GRB7	MET	[activation]
TTC9C	ITGA4	[activation]
CEBPA	ATF2	[activation]
RASD2	SMAD3	[activation]
BTRC	WEE1	[inhibition]
STMN1	GLP1R	[activation]
GNL3	STAT3	[activation]
SAV1	AMOT	[activation]
SERPINA1	ERLEC1	[inhibition]
RAD21	DAPK3	[activation]
RIPK1	ACTG1	[activation]
POLL	UBE2H	[activation]
CTNNB1	EPAS1	[activation]
ARMC7	RUNDC3A	[activation]
RAC1	KPNA2	[activation]
PIK3R3	ABCB6	[activation]
CDK1	TGFBR2	[activation]
AOX1	ABCA1	[activation]
VAPB	PITPNM1	[activation]
DOK1	PLCG1	[activation]
PIK3R1	MAP4K1	[activation]
MAPK13	IGF2BP2	[activation]
DNM1L	MAGEA1	[activation]
RAB1A	CREB1	[activation]
PTK2	SH3KBP1	[activation]
VPS13A	PIK3R1	[activation]
HSPB1	XPO1	[activation]
ATG101	FOXO3	[inhibition]
FAM3C	CREB3	[activation]
CHEK1	CHUK	[activation;inhibition]
HSPA5	EP300	[activation]
CD247	CD3E	[activation]
ARHGAP1	RASA1	[activation]
AR	ERG	[activation]
TNFSF11	TMOD3	[activation]
MS4A1	FYN	[activation]
BMX	APP	[activation]
MAG	COL9A3	[activation]
PRPSAP1	PRPS2	[inhibition]
PRKAA2	FLCN	[activation]
IRF1	SP2	[activation]
CASP1	PSEN2	[activation]
F2	PROZ	[activation]
YES1	CD46	[inhibition]
CD5	CD4	[activation]
CCR5	IL24	[activation]
CASP7	ROCK1	[activation]
GRB2	ARPC1A	[activation]
ILK	TWF2	[activation]
MC4R	POMC	[activation]
ICAM1	SET	[activation]
PRKACB	RPS6	[activation]
NFKBIA	CALM1	[activation]
TYROBP	KLRC3	[activation]
SRC	ITCH	[activation]
UGT1A7	UGT1A8	[activation]
FAF2	MYC	[activation]
KIF2A	MRPL53	[activation]
PRKRA	GDF9	[activation]
HSPB1	ENPP2	[activation]
DDX58	UBE2D3	[activation]
LRRK1	POLR2M	[activation]
HDAC1	GATA1	[activation]
ERBB2	ANXA2	[activation]
HSPA5	BRAF	[activation]
RAP1B	HRAS	[activation]
CRK	DNAJA3	[inhibition]
IL36RN	REL	[inhibition]
RHOBTB2	PALB2	[activation]
TGM3	USP53	[activation]

16873	NUP133	LRRK2	[activation]
16874	ARPC1B	ARPC3	[activation]
16875	CDKN1A	TRMT2A	[activation;inhibition]
16876	RELA	LARS	[activation]
16877	RAB14	HLA-B	[activation]
16878	CDK3	ARRB2	[activation]
16879	VAV1	ERBB2	[activation]
16880	DUSP15	CRK	[activation]
16881	CTNNBL1	STAT1	[activation]
16882	MDC1	TP53	[activation]
16883	GRB2	SLC25A3	[activation]
16884	BARD1	TRAF1	[activation]
16885	CDK14	CAMK1	[activation]
16886	RAD21	CHD4	[activation]
16887	PPP4R1	CEP63	[inhibition]
16888	G3BP1	TRAF6	[activation]
16889	SNAI2	PPP2CB	[inhibition]
16890	MAPK1	NUP153	[activation]
16891	POU1F1	CREBBP	[activation]
16892	TSC1	YWHAE	[inhibition]
16893	IRF4	SPI1	[inhibition]
16894	UBC	INSIG2	[activation]
16895	PTPN14	JUP	[activation]
16896	LYPD3	STK11	[activation]
16897	SPP1	C1RL	[activation]
16898	TMPRSS3	RXRA	[inhibition]
16899	HES1	FANCE	[activation]
16900	MCL1	BCL2L1	[activation]
16901	CBL	CTNNB1	[activation]
16902	ESR2	ACTN1	[activation]
16903	PXN	CTTN	[activation]
16904	GFRA1	TRADD	[activation]
16905	CD247	MCM7	[activation]
16906	MYD88	TNIP1	[activation]
16907	CSDC2	PIK3R1	[activation]
16908	MDC1	STAT1	[activation]
16909	SMC4	SIRT6	[activation]
16910	SMAD3	PPARG	[activation]
16911	NRAS	GRB14	[activation]
16912	PSIP1	HMGA2	[activation]
16913	PPP1CC	TRAF3	[activation]
16914	SPERT	CCNG1	[activation]
16915	LRP1	MMP13	[activation]
16916	ADCY1	GNAI2	[activation]
16917	LRPAP1	SORL1	[inhibition]
16918	ARHGDIA	ADRB2	[activation]
16919	IGF1R	ARHGEF12	[activation]
16920	LATS1	SCRIB	[inhibition]
16921	CELSR3	GRB2	[activation]
16922	DCAF13	WARS	[activation]
16923	PRLR	CREBBP	[activation;inhibition]
16924	MEP1B	CCK	[activation]
16925	CUL1	TBK1	[inhibition]
16926	EGFR	BUB3	[activation]
16927	EIF4E2	TRIM54	[inhibition]
16928	RHPN1	RHOA	[activation]
16929	SUMO1	PTPN1	[activation]
16930	SYT6	PRKCB	[activation]
16931	ASH2L	MYB	[activation]
16932	CTNNB1	KDR	[activation]
16933	TRAPPC1	TRAPPC11	[activation]
16934	HRAS	CDC25B	[activation]
16935	CDK5RAP3	IGHA1	[activation]
16936	EGFR	SLC12A2	[activation]
16937	SOCS1	ALK	[inhibition]
16938	CASP8	RNF34	[inhibition]
16939	FYN	MAP4K5	[activation]
16940	SPOP	PIAS1	[inhibition]
16941	CYC1	ATF2	[activation]
16942	PTPRA	PTPRM	[activation]
16943	MAPK12	ERBB2IP	[inhibition]
16944	PRKCD	PRKCZ	[activation]
16945	FSHR	AKT2	[activation]
16946	CDKN1A	BRAP	[activation;inhibition]
16947	GNA13	PPP5C	[activation]
16948	DEPTOR	RICTOR	[activation]

16949	ADAM22	YWHAH	[activation]
16950	DNAJC8	APP	[inhibition]
16951	RPTOR	DEPTOR	[activation;inhibition]
16952	PRKACA	LRP1	[activation]
16953	ABL1	RIMS1	[activation]
16954	XRCC1	TP53	[activation]
16955	MTNR1A	RAP1A	[activation]
16956	GABARAP	MCM3	[activation]
16957	EPHB3	FYN	[activation]
16958	TP53	CSNK2B	[activation]
16959	RARS2	PHLDA3	[activation]
16960	RHOJ	BHLHE40	[activation]
16961	IKBKG	FYN	[activation]
16962	RBL2	CCNE1	[inhibition]
16963	ACVR1	BMPR1A	[activation;inhibition]
16964	C19orf47	NTM	[inhibition]
16965	TP53	BMX	[activation]
16966	GOLGA2	ITPKB	[activation]
16967	MLLT4	CTNNA1	[activation]
16968	TP53	GPATCH8	[activation]
16969	UBE2N	RIPK1	[activation]
16970	EPB41	CD44	[activation]
16971	BCR	PTPN1	[activation]
16972	MEN1	TP53	[activation]
16973	MDM2	BTRC	[activation]
16974	DCC	CTSV	[activation]
16975	SNAP91	OGT	[activation]
16976	HLA-B	NUTF2	[activation]
16977	DDA1	PGK1	[activation]
16978	EP300	FOSB	[activation]
16979	IRS2	SFN	[activation]
16980	MAGI1	RPS6KA3	[activation]
16981	SREBF2	RAB11B	[activation]
16982	PPP1CC	BCL2L1	[activation]
16983	PF4	SDC2	[activation]
16984	CSDE1	TRAF6	[activation]
16985	WASF1	ABI2	[activation]
16986	NR3C1	RAF1	[activation]
16987	LRRK2	AURKC	[activation]
16988	TBC1D8	SPP1	[activation]
16989	NCL	RICTOR	[activation]
16990	RNF7	CASP7	[activation]
16991	ATG5	FABP5	[activation]
16992	SRC	INSR	[activation]
16993	HRAS	GREB1	[activation]
16994	ARRB2	EGFR	[activation]
16995	CD3G	CD3E	[activation]
16996	CTNNB1	NDRG1	[activation]
16997	BAG2	JAK3	[inhibition]
16998	HTRA1	COL1A1	[inhibition]
16999	MCM3	MAPK6	[activation]
17000	CALM1	LY96	[activation]
17001	SMAD1	USP9X	[activation]
17002	PIAS1	GRM8	[inhibition]
17003	CDK16	CDK5R1	[activation]
17004	EP300	SMARCA2	[activation]
17005	TNFAIP3	TP53	[activation]
17006	HSP90AB1	RPS6KA2	[activation]
17007	BANP	HIF1A	[activation]
17008	F2R	FLOT2	[activation]
17009	TAL1	ETS1	[activation]
17010	LIMK2	LIMK1	[activation;inhibition]
17011	HLA-A	BCAP31	[activation]
17012	ITPR1	LYN	[inhibition]
17013	SMARCE1	NCOA1	[activation]
17014	LAT	LCP2	[activation]
17015	IL1R1	IRAK1	[activation;inhibition]
17016	XPO1	SMAD1	[activation]
17017	SETDB1	GIPC2	[activation]
17018	RACGAP1	PPP2CA	[activation]
17019	HSP90AB1	IFIT1	[activation]
17020	ICAM1	RPS3A	[activation]
17021	SLC6A3	SNCA	[activation]
17022	INSIG2	UBQLN1	[activation]
17023	PIK3CA	ATPIF1	[activation]
17024	DNAJB1	CDC37	[inhibition]

17025	HES6	TWIST1	[activation]
17026	IGF2BP1	TP53	[activation]
17027	RXRA	GADD45A	[inhibition]
17028	BMX	CRK	[activation]
17029	TSSK4	CREB1	[activation]
17030	BACH1	HMMR	[activation]
17031	SMAD3	MAX	[inhibition]
17032	HSPB1	MRPS23	[inhibition]
17033	H2AFX	PPP2R4	[activation]
17034	COPS3	STK11	[activation]
17035	VIP	DPP4	[activation]
17036	PPP2R5E	CHEK2	[activation]
17037	VAV2	STK24	[activation]
17038	GNAS	RGS2	[activation]
17039	BAD	BCL2L11	[activation]
17040	DUSP3	EGF	[inhibition]
17041	NCL	HCK	[activation]
17042	BRCA1	SMAD2	[activation]
17043	CAMK2D	CAMK2G	[activation]
17044	ITGA4	ADAM28	[activation]
17045	CDK10	PIN1	[activation]
17046	RALA	EXOC8	[activation]
17047	EGFR	DOK2	[activation]
17048	CD38	CBL	[activation]
17049	CCND1	CKS1B	[activation]
17050	CRLF2	IL7R	[activation]
17051	RHOU	NCK1	[activation]
17052	SMAD1	TLR2	[activation]
17053	HTT	PTK6	[activation]
17054	TP53	MAPKAPK5	[activation]
17055	ITPR1	CCNB1	[inhibition]
17056	PRKD1	EGFR	[activation]
17057	MARK3	FANCF	[activation]
17058	PSEN1	PIK3R1	[activation]
17059	STK3	PRKRIR	[activation]
17060	FANCG	ZBED1	[activation]
17061	HSP90AB1	CAMKK2	[activation]
17062	FBXW11	PRLR	[inhibition]
17063	PPARG	CHD8	[inhibition]
17064	SGK1	HNRNPM	[activation]
17065	HDAC1	CDKN1A	[inhibition]
17066	EGFR	HELLS	[activation]
17067	MRPL36	RRAS	[activation]
17068	GADD45A	HIST3H3	[activation]
17069	RAN	CFTR	[activation]
17070	ARRB2	IGF2BP1	[activation]
17071	CCNF	CCNB1	[activation]
17072	DOCK7	RHOA	[activation]
17073	IQGAP1	PODXL	[inhibition]
17074	HSPA8	MAP3K7	[inhibition]
17075	MGEA5	NUDCD3	[activation]
17076	VCAM1	VCL	[activation]
17077	HIST1H3A	CD81	[activation]
17078	SMAD3	MECOM	[inhibition]
17079	DOCK9	SMAD3	[activation]
17080	SMAD9	TBCD	[inhibition]
17081	GADD45G	KIAA1377	[activation]
17082	EGFR	HSPA2	[activation]
17083	FBXL21	CRY2	[inhibition]
17084	ILK	FANCI	[activation]
17085	BRAF	ZNF189	[activation]
17086	MAATS1	MYC	[activation]
17087	CLDN3	PRKACA	[activation]
17088	PRKAR1A	WNK1	[activation]
17089	HNRNPA1	MEF2A	[activation]
17090	FYB	GRAP2	[activation]
17091	HIPK4	CTSB	[activation]
17092	ACTG1	APOE	[activation]
17093	SMURF1	ROCK1	[activation]
17094	HSPA7	GABARAPL2	[activation]
17095	TRAF2	HSPA1L	[activation]
17096	USP9X	SMAD2	[activation]
17097	TERT	MCRS1	[activation]
17098	ETFA	CDC42	[activation]
17099	SRPK1	RPS6KB2	[activation]
17100	COL1A1	ANXA1	[activation]

17101	LRRK2	SF3B2	[activation]
17102	YWHAZ	CDC25A	[activation]
17103	RIPK1	HSPA1L	[activation]
17104	BCAP31	VAMP3	[activation]
17105	MAPK8	BID	[activation]
17106	EPAS1	SMARCC1	[activation]
17107	IGHG1	NUP214	[activation]
17108	BCL6	TRAF1	[activation]
17109	TRMT2A	PIK3R3	[activation]
17110	MYD88	IL1RAP	[activation;inhibition]
17111	HSPA8	EIF4A3	[activation]
17112	NUP155	CDK4	[activation]
17113	TRIM24	NFE2L2	[activation]
17114	ULK1	GABARAPL2	[inhibition]
17115	KIF23	PPP2R5E	[activation]
17116	MAPK1	TNIP1	[activation]
17117	PPM1A	AXIN1	[activation]
17118	RARA	FOS	[activation]
17119	ATF5	EP300	[activation]
17120	EHMT1	BCL6	[activation]
17121	SETDB1	NUDCD3	[activation]
17122	HIST1H4A	RAD21	[activation]
17123	PIK3R1	WASF1	[activation]
17124	ZFP36	TNF	[activation]
17125	RLTPR	NOTCH1	[activation]
17126	RPA1	STRA13	[activation]
17127	ATM	CSNK1D	[activation]
17128	RYR1	GRB2	[activation]
17129	TPRN	PPP1CA	[activation]
17130	MDC1	CREBBP	[activation]
17131	TLR5	TLR4	[activation]
17132	PLK1	LATS1	[inhibition]
17133	HSPA1L	GABARAP	[activation]
17134	WAS	WIPF1	[activation]
17135	MRRF	APP	[inhibition]
17136	FGF2	PTX3	[activation]
17137	PRKRIP1	TRAF2	[activation]
17138	RGS4	PLCB1	[activation;inhibition]
17139	NOS1	VAC14	[activation]
17140	TNFRSF14	TNFSF13	[activation]
17141	SYN1	CAMK2G	[activation]
17142	TLR2	IRF3	[activation]
17143	TGFBR1	STYXL1	[inhibition]
17144	PRKDC	RBBP8	[activation]
17145	RNF10	GRB2	[activation]
17146	TNFRSF8	TRAF5	[activation]
17147	GRB2	PIK3R2	[activation]
17148	COMMD8	RELA	[activation]
17149	BLNK	GAB1	[activation]
17150	GRB2	PNMA2	[activation]
17151	SMAD3	HMGA2	[activation]
17152	PDCD5	ACP5	[activation]
17153	CBL	UBASH3B	[activation]
17154	MAVS	SRC	[activation]
17155	GOLGA8F	BAAT	[activation]
17156	UBE4A	CASP6	[activation]
17157	C1QTNF2	TRIM54	[activation]
17158	NCK2	SOS1	[activation]
17159	SERINC3	KDELR2	[activation]
17160	HSP90AB1	MAPK6	[activation]
17161	RINT1	RB1	[inhibition]
17162	TNFRSF10A	CD4	[activation]
17163	NTRK2	PTPN11	[activation;inhibition]
17164	ZNHIT3	MEIS2	[activation]
17165	DLGAP4	GRB2	[activation]
17166	ARF5	RAB11A	[activation]
17167	TMX1	H2AFX	[activation]
17168	LXN	PPM1A	[activation]
17169	MTOR	TP53	[activation]
17170	SRPK1	WDR55	[activation]
17171	PRKCA	XK	[activation]
17172	RELA	STAT1	[activation]
17173	DAPK1	CCNDBP1	[activation]
17174	ATM	HLA-A	[activation]
17175	LATS1	FOXL2	[inhibition]
17176	PKM	EP300	[activation]

17177	MCM7	ITGA4	[activation]
17178	TRAF6	FYN	[activation]
17179	HBZ	DOCK8	[activation]
17180	ANAPC4	CDC20	[inhibition]
17181	RAPGEF3	RAN	[activation]
17182	STAT3	SSSCA1	[activation]
17183	TRAF2	UBE2V1	[activation]
17184	E2F1	ARID3A	[activation]
17185	RAP1B	TLE1	[activation]
17186	CEACAM3	SRC	[activation]
17187	RASGRF1	YWHAE	[activation]
17188	CHEK1	XIAP	[inhibition]
17189	EFNA5	ABL1	[activation]
17190	PSIP1	SH3KBP1	[activation]
17191	CCDC90B	RGL2	[activation]
17192	EP300	RPS6KB1	[activation]
17193	NOTCH1	CACNA1A	[activation]
17194	TP53	ARRB1	[activation]
17195	CCDC8	RAB14	[activation]
17196	PTN	GSK3B	[inhibition]
17197	APBB1	YWHAG	[activation]
17198	ITGB1	CD47	[activation]
17199	KCNA5	KCNA2	[activation]
17200	PTX3	CFH	[activation]
17201	DOK1	VAV1	[activation]
17202	GAB2	YWHAZ	[activation]
17203	ASGR2	MROH5	[activation]
17204	APP	CSNK1E	[activation]
17205	ILK	GLUD1	[activation]
17206	CD81	RPS15	[activation]
17207	ITGB2	PRKCB	[activation]
17208	CETN3	HSPB1	[activation]
17209	INHBC	INHBA	[activation]
17210	SMAD2	CAD	[activation;inhibition]
17211	LARP1	VCAM1	[activation]
17212	TRAF1	SRC	[activation]
17213	ITPR2	TRPC4	[inhibition]
17214	SRPK2	CSAG1	[activation]
17215	CSF1	SOCS1	[activation]
17216	PPP2CB	ITGA4	[activation]
17217	DIABLO	BIRC5	[activation]
17218	APC	HGS	[activation]
17219	WAS	TRIP10	[activation]
17220	PAXIP1	APP	[activation]
17221	CDK5RAP3	UFM1	[activation]
17222	GATA1	NME1	[activation]
17223	STAP2	IKBKB	[activation]
17224	FOXP3	NFAT5	[activation]
17225	PRKCB	MBP	[inhibition]
17226	PPP2CA	APC	[inhibition]
17227	RPA3	RFC2	[activation]
17228	ICT1	IGF2BP2	[activation]
17229	PSD	HUS1	[activation]
17230	SSU72	MAPK9	[activation]
17231	PDPK1	PTPRC	[activation]
17232	CCL2	VCAN	[activation]
17233	ADCYAP1	SHH	[inhibition]
17234	LYN	MS4A1	[activation]
17235	SRPK1	KPTN	[activation]
17236	PIAS3	SERPINA10	[inhibition]
17237	SRCIN1	CRK	[activation]
17238	RXRB	MBP	[inhibition]
17239	PKNOX1	HOXB6	[activation]
17240	CDK1	PTCH1	[activation]
17241	STAT3	KAT5	[activation]
17242	HLA-B	WDR48	[activation]
17243	DNM1L	FIS1	[activation]
17244	CRY1	BTRC	[inhibition]
17245	HNRNPA2B1	CTNNB1	[activation]
17246	FASLG	CFLAR	[inhibition]
17247	IGFBP4	IGF2	[activation]
17248	CLASRP	DAB1	[activation]
17249	CCDC25	SMAD2	[activation]
17250	PDGFRA	CAV1	[activation]
17251	SH3KBP1	PTK2B	[activation]
17252	EGFR	EHD4	[activation]

17253	JAK2	IL4R	[activation]
17254	SH2D2A	MAP3K2	[activation]
17255	SOCS3	CSNK1E	[activation;inhibition]
17256	MAPK14	PI4K2B	[activation]
17257	CSN2	GRB2	[activation]
17258	VCAM1	QARS	[activation]
17259	CDC5L	YWHAG	[activation]
17260	CXCR4	MYBL2	[activation]
17261	CAMK2D	WASF3	[activation]
17262	SRSF5	TNK2	[activation]
17263	CDC6	CDK1	[activation]
17264	ICT1	MRPL2	[activation]
17265	ATM	ERRFI1	[activation]
17266	SMURF1	CAPZA1	[inhibition]
17267	MTNR1B	GPR50	[activation]
17268	SRPK1	TCEAL2	[activation]
17269	AKT2	EGFR	[activation]
17270	MDM2	XPO1	[activation]
17271	MKNK2	MAPK8	[activation]
17272	IGHA1	SERPIND1	[inhibition]
17273	HMGB1	HOXD10	[activation]
17274	YWHAZ	HIST1H3A	[activation]
17275	RHOA	HSPA1A	[activation]
17276	KMT2A	CTBP1	[activation]
17277	POF1B	ESR1	[activation]
17278	CDC42	CASP3	[activation]
17279	CHERP	LRRK2	[activation]
17280	BTRC	CASP3	[activation]
17281	RPLP2	TP53	[activation]
17282	DDX24	TPI1	[activation]
17283	HSP90AA1	NR3C1	[activation]
17284	RHOA	RAP1GDS1	[activation;inhibition]
17285	CRKL	KIDINS220	[activation]
17286	SHC1	KRT17	[activation]
17287	ST14	STK11	[activation]
17288	SMYD2	ASPM	[activation]
17289	PRSS23	ACTG1	[activation]
17290	DDX54	ESR2	[activation]
17291	MAP3K5	EP300	[activation]
17292	PPP3CB	GRB2	[activation]
17293	TRAF6	MAP3K8	[activation]
17294	PLAU	MYC	[activation]
17295	HSP90AB1	EIF4EBP1	[activation]
17296	TNFSF15	TNFRSF6B	[activation]
17297	PIAS2	MYCN	[activation]
17298	DAB1	VLDLR	[inhibition]
17299	MAPK8	RPTOR	[activation]
17300	CSK	PLD2	[activation]
17301	FOS	IDS	[activation]
17302	TP53	PPM1G	[activation]
17303	TGFBR1	ZFYVE9	[activation]
17304	DHX9	HLA-B	[activation]
17305	MAPK6	RARA	[activation]
17306	GCN1L1	VCAM1	[activation]
17307	ITPR2	TRPC1	[inhibition]
17308	MEF2A	HAND1	[activation]
17309	MGEA5	MAST1	[activation]
17310	PPARGC1A	UBE2I	[activation]
17311	PTPN6	THEMIS	[activation;inhibition]
17312	BARD1	MTA2	[activation]
17313	STAT3	PTK2B	[activation]
17314	CDC5L	KALRN	[activation]
17315	SRPK1	SUMO2	[activation]
17316	RAPGEF2	FBXW11	[activation]
17317	MDFI	SIX1	[activation]
17318	KMT2A	CREBBP	[activation]
17319	MMP9	COL4A3	[activation;inhibition]
17320	SERPING1	C1S	[inhibition]
17321	CREBBP	ATF1	[activation]
17322	HIST1H1B	NSD1	[activation]
17323	SPTB	MAPT	[activation]
17324	SRPK1	MAOB	[activation]
17325	SMC2	IKBKG	[activation]
17326	EPN2	APOE	[activation]
17327	VCAM1	PDCD10	[activation]
17328	FASLG	SRGAP1	[inhibition]

17329	KPNA2	ITGA4	[activation]
17330	RAD17	RFC2	[activation]
17331	ADAM22	ABI2	[activation]
17332	DBP	TEF	[activation]
17333	GNAO1	TSHR	[activation]
17334	CMAS	LRRK2	[activation]
17335	GRB2	PTPN22	[activation]
17336	CTNNA1	SMAD1	[activation]
17337	HSPB1	GARS	[activation]
17338	SOSTDC1	BMP4	[activation]
17339	SHPK	MDM2	[activation]
17340	HSP90AB1	IRF2	[activation]
17341	HDAC1	STAT5A	[activation]
17342	SHCBP1	PRKCB	[activation]
17343	CREBBP	TACC2	[activation]
17344	PTK6	EXOC5	[activation]
17345	PAXIP1	WDR18	[activation]
17346	RAB5C	RIN2	[activation]
17347	NOTCH1	RANBP9	[activation]
17348	RAC1	ARHGDIG	[activation]
17349	MTA2	TP53	[activation]
17350	TNK2	SRC	[activation]
17351	PRKCI	MAP1LC3A	[activation]
17352	EP300	NFATC1	[activation]
17353	ITGA4	HBG1	[activation]
17354	RNF114	UBE2DNL	[activation]
17355	LRP1	MMP17	[activation]
17356	ARF1	PLD1	[activation]
17357	PPARA	SUMO1	[activation]
17358	MPP5	ARF6	[activation]
17359	TBRG4	MTMR4	[activation]
17360	DVL1	RB1	[inhibition]
17361	ATR	XPC	[activation]
17362	NOTCH1	PIK3CA	[activation]
17363	CDK5	TP53	[activation]
17364	SLMAP	PPP2CA	[inhibition]
17365	FASN	IGHG1	[activation]
17366	ACVR2B	GDF11	[activation]
17367	BID	CDC5L	[activation]
17368	TCF12	CREBBP	[activation]
17369	KCNAB1	KCNA5	[activation]
17370	MYC	FANCI	[activation]
17371	MYC	HSPB1	[activation]
17372	PPP2R1A	ERBB2	[activation]
17373	CD4	EIF4A2	[activation]
17374	MAPKAPK2	DDX5	[activation]
17375	MYCN	CDKN2A	[activation;inhibition]
17376	THRB	PRKDC	[activation]
17377	PHIP	IRS1	[activation]
17378	IAPP	COL25A1	[activation]
17379	MAPKAPK2	CDC25B	[activation]
17380	ELN	MMP7	[activation]
17381	MAP1LC3B	DOK1	[activation]
17382	ABL1	ENAH	[activation]
17383	SPECC1L	APC	[inhibition]
17384	ITGB2	GULP1	[activation]
17385	NXF1	GABARAP	[activation]
17386	CALM1	KCNN3	[activation]
17387	HNRNPM	HLA-B	[activation]
17388	ANXA5	ISG15	[activation]
17389	NTRK2	NR3C1	[activation]
17390	HCK	DNM2	[activation]
17391	PER3	CHEK2	[activation]
17392	JUP	RAB8B	[activation]
17393	C14orf142	TP53RK	[activation]
17394	SMAD2	MECOM	[activation]
17395	NXF1	FER	[activation]
17396	ERH	ILK	[activation]
17397	TTBK1	CDK5R1	[activation]
17398	SMAD7	PPP1R15A	[inhibition]
17399	NGB	GNAI2	[activation]
17400	ATF2	AR	[activation]
17401	PIK3R2	GRN	[activation]
17402	YWHAB	EPB41L3	[activation]
17403	ERLIN1	INSIG1	[activation]
17404	HIF1A	HDAC1	[activation]

17405	CAST	FYN	[inhibition]
17406	MAST1	RNLS	[activation]
17407	STYK1	HSP90AB1	[activation]
17408	EP300	SNW1	[activation]
17409	FBXW7	RICTOR	[activation]
17410	DDR1	NCK2	[activation]
17411	RYR2	MDM2	[activation]
17412	LPXN	MAP2K7	[activation]
17413	DNAJA1	RELA	[inhibition]
17414	PIP5K1B	PIP5KL1	[activation]
17415	HLA-G	B2M	[activation]
17416	RAF1	PDGFRB	[activation]
17417	SIRPG	CD47	[activation]
17418	FGFR1	NOSTRIN	[activation]
17419	APP	EIF2B2	[activation]
17420	KIF23	SH3KBP1	[activation]
17421	FBXW7	HRAS	[activation]
17422	KDELR1	SERINC3	[activation]
17423	IGHG1	FCGR3A	[activation]
17424	PRKACA	CAV1	[activation]
17425	NUP153	MYC	[activation]
17426	SREBF2	SUMO1	[activation]
17427	DNAJB1	COA3	[inhibition]
17428	TANK	CBLB	[inhibition]
17429	PPARGC1A	NR5A2	[activation]
17430	ARPC2	OBSL1	[activation]
17431	HIST1H1C	SMURF1	[inhibition]
17432	MMP2	CLDN1	[activation]
17433	OBSL1	HIST2H3A	[activation]
17434	APP	SRPK2	[activation]
17435	SMURF1	BTRC	[inhibition]
17436	NSD1	RORA	[activation]
17437	TGFBR1	RHOJ	[activation]
17438	NEDD4	CASP3	[activation]
17439	GFRA1	NCAM1	[activation]
17440	RHOA	PRKCZ	[activation]
17441	EHD2	DDX56	[activation]
17442	TAGLN2	MDM2	[activation]
17443	CALM1	ADORA2A	[activation]
17444	DDX47	PRNP	[activation]
17445	RELA	HSP90AA1	[activation]
17446	CR1	FUT4	[activation]
17447	GRB2	RPL21	[activation]
17448	TXN2	FAM9B	[activation;inhibition]
17449	SORBS1	SMAD2	[activation]
17450	ANAPC11	FZR1	[activation]
17451	PPP2CA	PPP2R5A	[activation]
17452	HDAC1	SUV39H1	[activation]
17453	XPC	ATM	[activation]
17454	PRIM1	MOV10	[activation]
17455	INSR	PTPRB	[activation]
17456	SHANK1	ARHGEF7	[activation]
17457	NUDCD3	CFLAR	[inhibition]
17458	IL36RN	SSBP3	[activation]
17459	MARCKS	CALM1	[activation]
17460	CASP8AP2	FADD	[activation]
17461	RAB11A	ME2	[activation]
17462	CST6	GSK3B	[inhibition]
17463	OSR1	EGLN1	[activation]
17464	ABL1	NTRK1	[activation]
17465	MAP2K1	PEBP1	[activation]
17466	CCDC136	MOS	[activation]
17467	MYO5B	SMAD3	[activation]
17468	PLCB3	NXF1	[activation]
17469	TRAF6	STIP1	[activation]
17470	YWHAB	SH3RF3	[activation]
17471	CD3E	ZAP70	[activation]
17472	VCAM1	MYL6	[activation]
17473	RDH13	TP53	[activation]
17474	NR3C1	KPNA2	[activation]
17475	GNAI3	ELAVL1	[activation]
17476	HSPA4	PACRG	[inhibition]
17477	GATA1	PRKAA1	[activation]
17478	SMAD4	RAB2B	[activation]
17479	DNAJB7	SRPK2	[inhibition]
17480	RBM48	ANXA7	[activation]

17481	ANP32B	STK11	[activation]
17482	GABARAP	PRKCI	[activation]
17483	GRIP2	GRB2	[activation]
17484	PLEKHB2	VSIG8	[activation]
17485	BAD	WNK1	[activation]
17486	AURKB	PHKB	[activation]
17487	LYAR	PRKRA	[activation]
17488	DOCK2	FYB	[activation]
17489	PRPF6	PHLDA3	[activation]
17490	SNAI2	PPP2R2D	[activation]
17491	FGR	CCR3	[activation]
17492	ERBB3	GRB7	[activation]
17493	RXRA	GSK3B	[inhibition]
17494	EGF	S100A4	[activation]
17495	CEP350	MAPK1	[activation]
17496	YWHAB	TSC2	[activation]
17497	CELSR3	FYN	[activation]
17498	NRG1	CDC5L	[activation]
17499	POLA2	APP	[activation]
17500	YWHAZ	HIST1H4A	[activation]
17501	FYN	NTRK2	[activation]
17502	CSF1	NOTCH2NL	[activation]
17503	MDM2	CTBP1	[activation]
17504	CHEK2	CDH13	[activation]
17505	ANXA5	LAMC3	[activation]
17506	ITPR3	SIGMAR1	[activation]
17507	CREB3L2	GULP1	[activation]
17508	PPP2CA	PDCD10	[inhibition]
17509	TIRAP	SOCS1	[inhibition]
17510	ENAH	APBB1	[activation]
17511	CCNA1	CSNK1E	[activation]
17512	BCL2L1	SNCA	[activation]
17513	ATG14	RALB	[activation]
17514	REEP4	YWHAB	[activation]
17515	MDK	JAK2	[activation]
17516	IFI16	ICAM1	[activation]
17517	PIAS2	MX2	[activation]
17518	PVRL3	NOTCH2NL	[activation]
17519	ATM	PPP1CC	[activation]
17520	MIF	GABARAPL2	[activation]
17521	HIF1A	TRIM28	[activation]
17522	ITGA4	HIST1H2BH	[activation]
17523	CXCL10	IGFBP7	[activation]
17524	BACE1	FURIN	[activation]
17525	HSP90AB1	PTK2	[activation]
17526	PRKCZ	GLRX3	[activation]
17527	DDX58	NLRC5	[activation]
17528	ESR2	FTSJ3	[activation]
17529	TFDP1	CDC27	[inhibition]
17530	GNB5	RGS9	[activation;inhibition]
17531	BIRC2	MAP3K14	[activation;inhibition]
17532	SH2B1	FGFR3	[activation]
17533	HSPA8	HSPA2	[inhibition]
17534	YAP1	PARD3	[activation]
17535	GABARAP	PFN1	[activation]
17536	TET2	YWHAH	[activation]
17537	DUSP3	MCC	[inhibition]
17538	MAP3K3	TRAF6	[activation]
17539	E2F1	BTG3	[activation]
17540	PRKDC	YWHAB	[activation]
17541	ARPC2	AURKA	[activation]
17542	GYS1	CCDC36	[activation;inhibition]
17543	FRS2	CBL	[activation]
17544	SOCS1	NCK1	[inhibition]
17545	PTPN6	PDGFRB	[activation;inhibition]
17546	RB1	JUN	[activation]
17547	GUCY1A3	IKBKG	[activation]
17548	BUB1B	MIS12	[inhibition]
17549	MAPK7	PML	[activation]
17550	PBK	RAF1	[activation]
17551	RASA3	HCK	[activation]
17552	DARC	CXCL1	[activation]
17553	TRAIP	TNFRSF8	[activation]
17554	KAT2B	SMARCB1	[activation]
17555	CAMK2B	KRTAP19-5	[inhibition]
17556	NEK1	LRRK2	[activation]

17557	LRFN4	PPP2CA	[inhibition]
17558	APP	COL4A1	[activation]
17559	CTR9	HIST1H1C	[activation]
17560	RPS6KA2	HSP90AA1	[activation]
17561	IRF7	SUMO1	[activation]
17562	MRPL40	HLA-B	[activation]
17563	SRPK1	SAFB	[activation]
17564	NMI	IRF7	[activation]
17565	ALG8	RPS6KA5	[activation]
17566	EPX	PIK3R1	[activation]
17567	SYTL3	RAB10	[activation]
17568	CD19	PLCG2	[activation]
17569	CCL18	TLE1	[activation]
17570	CD300LF	PTPN6	[activation;inhibition]
17571	MAP2K3	MAP2K6	[activation]
17572	CCR7	CD247	[activation]
17573	WARS	DDA1	[activation]
17574	ATR	CREB1	[activation]
17575	CDC25B	CREBBP	[activation]
17576	SNAI1	NBN	[activation]
17577	GHRHR	MLNR	[activation]
17578	GNAS	PCK1	[activation]
17579	RAB14	APP	[activation]
17580	ABL2	HCK	[activation]
17581	HNRNPA1	RPA3	[activation]
17582	MYC	SKIV2L	[activation]
17583	PTPRC	ANP32A	[activation]
17584	RUNDC3B	STK3	[activation]
17585	SIRT2	KAT2B	[activation]
17586	GEM	A2M	[inhibition]
17587	HSPA1A	EIF2AK2	[inhibition]
17588	PTPRM	PPFIBP1	[activation]
17589	CCR5	CREB3	[activation]
17590	FEZ2	C16orf59	[activation]
17591	HSPB1	EHD1	[activation]
17592	NUDCD3	KLHL35	[activation]
17593	YWHAG	AMOT	[activation]
17594	CCNG1	MOV10	[activation]
17595	DIRAS2	CALM1	[activation]
17596	B4GALT1	TUBB	[activation]
17597	NR4A1	RPS6KA5	[activation]
17598	ITGB2	PRKCH	[activation]
17599	PPP4C	PPP4R1	[inhibition]
17600	PTPN6	GHR	[activation;inhibition]
17601	CDCA8	HIST2H3A	[activation]
17602	EHD4	CDC5L	[activation]
17603	CREB3	PLLP	[activation]
17604	SRPK2	APOBEC3C	[activation]
17605	PER1	PAXIP1	[activation]
17606	TNFRSF1B	GRN	[activation]
17607	PTPN6	TRAF6	[activation;inhibition]
17608	GORASP1	CASP3	[activation]
17609	HOXB8	MEIS1	[activation]
17610	MMP9	EPHB2	[activation]
17611	SP1	RUNX1	[activation]
17612	MDM2	RAB7A	[activation]
17613	FBXO6	PPP6C	[inhibition]
17614	RASSF1	SFN	[inhibition]
17615	GMFG	CAMK1D	[activation]
17616	UFSP2	CAPN1	[activation]
17617	NXF1	SRSF1	[activation]
17618	ESR1	LCK	[activation]
17619	HSP90AA1	IGF1R	[activation]
17620	PTGDR2	ANKRD13C	[activation]
17621	RSPO2	PLEKHF2	[activation]
17622	CEP250	ABR	[activation]
17623	PIK3R1	DAB2IP	[activation]
17624	CAMK2G	LRRC7	[activation]
17625	POLD2	ERBB2	[activation]
17626	SRPK2	RTF1	[activation]
17627	LCP2	WNK1	[activation]
17628	CASP3	GRIPAP1	[activation]
17629	DHX15	ANXA1	[activation]
17630	PLCG1	SOS2	[activation]
17631	CCDC8	TP53	[activation]
17632	SRPK2	CHUK	[activation]

17633	TTC38	PFDN1	[inhibition]
17634	DES	MLH1	[activation]
17635	MYC	TNFRSF18	[activation]
17636	ARHGEF6	RAC1	[activation]
17637	NOTCH2NL	CTSG	[activation]
17638	CACNB2	CACNA1C	[activation]
17639	FABP1	GRB2	[activation]
17640	GRB2	RPL26	[activation]
17641	AAMP	C8orf33	[inhibition]
17642	MYC	C2orf16	[activation]
17643	DDAH2	VCAM1	[activation]
17644	CDK2AP1	POLA1	[activation]
17645	EPHB6	CRKL	[activation]
17646	SNRNP70	MYC	[activation]
17647	MADCAM1	CD44	[activation]
17648	AKT1	MTA3	[activation]
17649	USHBP1	BET1	[activation]
17650	LRPAP1	EIF4EBP1	[inhibition]
17651	SKP2	E2F1	[activation]
17652	LAMP2	RPTOR	[activation;inhibition]
17653	RPA1	SMAD3	[activation]
17654	SHC1	PTPN2	[activation]
17655	JUNB	MECOM	[activation]
17656	SIGLEC11	PTPN6	[activation;inhibition]
17657	APP	TNFSF13B	[activation]
17658	NR4A1	RPS6KA1	[activation]
17659	PIAS2	TICAM2	[activation]
17660	SUMO3	STIM1	[activation]
17661	SERPINA1	CELA1	[inhibition]
17662	ADAM17	TNF	[activation]
17663	CAMK1D	TNFSF13	[activation]
17664	C1orf109	SPERT	[inhibition]
17665	CALM1	ADD2	[activation]
17666	MYH3	DYSF	[activation]
17667	CD19	PIK3R1	[activation]
17668	VAV1	ABL1	[activation]
17669	SNCA	A1BG	[activation]
17670	PRKCE	PECAM1	[activation]
17671	BAK1	ARHGDIA	[activation]
17672	BVES	HSPB1	[activation]
17673	GORASP2	NUP62	[activation]
17674	TP53	SUMO2	[activation]
17675	S1PR3	GNA13	[activation]
17676	EEF2	WEE1	[activation;inhibition]
17677	PRKAA1	FNIP1	[inhibition]
17678	MDM2	BHLHE40	[activation]
17679	MAD2L1	DSTYK	[inhibition]
17680	LYN	SH2B2	[activation]
17681	PDHX	CDKN1A	[activation;inhibition]
17682	CSNK2B	RPS6KA5	[activation]
17683	NR2F6	HSBP1L1	[inhibition]
17684	CRMP1	RPS6KA5	[activation]
17685	APP	ART4	[activation]
17686	MYO1D	ILK	[activation]
17687	RFC4	VCAM1	[activation]
17688	ANAPC1	ANAPC7	[activation]
17689	FOSL1	EP300	[activation]
17690	TNFRSF1A	CBL	[activation]
17691	HSPA8	RAF1	[activation]
17692	PCNA	EGFR	[activation]
17693	CHD8	CTDP1	[inhibition]
17694	STAT1	POR	[activation]
17695	ETS2	SRC	[activation]
17696	DVL2	WWTR1	[activation]
17697	HIST2H2BE	TAF1B	[activation]
17698	PDIA2	GRB2	[activation]
17699	CBL	CD19	[activation]
17700	F5	SERPINA3	[inhibition]
17701	NXF1	PCF11	[activation]
17702	CDK1	RAP1GAP	[activation]
17703	MOGS	AURKB	[activation]
17704	ICAM1	ILF2	[activation]
17705	SYNPO2	WNK1	[activation]
17706	YWHAZ	WWTR1	[activation]
17707	PIH1D2	MVP	[activation]
17708	PTPRJ	JAK2	[activation]

17709	EGFR	ATAD3B	[activation]
17710	MYO10	DCC	[activation]
17711	SRPK2	BCE1	[activation]
17712	DGKA	SRC	[activation]
17713	EP300	EMB	[activation]
17714	MAPK7	MYC	[activation]
17715	MAPK8	EEF2K	[activation]
17716	SCN5A	FGF13	[activation]
17717	ACTN1	CDK5	[activation]
17718	KMT2A	KMT2B	[activation]
17719	BTG2	FBXL3	[activation]
17720	EVI5L	A2M	[inhibition]
17721	RAB5A	IKBKE	[activation]
17722	LRRK2	CAPZA2	[activation]
17723	ADRBK2	CXCR4	[activation]
17724	LCK	PTPRM	[activation]
17725	DMC1	TRIM23	[activation]
17726	AMOT	PARD3	[activation]
17727	BCL2L1	RBM5	[activation]
17728	SUV39H1	C17orf82	[activation]
17729	RPAP3	EIF4A1	[activation]
17730	MAP3K7	FOS	[activation]
17731	STAT5A	LEF1	[activation]
17732	BCL6	IDI2	[activation]
17733	RXRB	PPARG	[inhibition]
17734	AHCYL1	BID	[activation]
17735	SMAD4	BRCA1	[activation]
17736	BCR	WDR48	[activation]
17737	SMAD5	SUV39H2	[activation]
17738	TRAF6	UBE2M	[activation]
17739	CDK11B	POU2F1	[activation]
17740	YWHAZ	BCAR1	[activation]
17741	DOK3	LRRK1	[activation]
17742	TRIP6	PRKAA1	[inhibition]
17743	STK25	ACACA	[activation;inhibition]
17744	STAT1	HSF1	[activation]
17745	RANBP9	NTRK2	[activation]
17746	RAB5A	ITSN1	[activation]
17747	GSK3B	RGS22	[activation;inhibition]
17748	CYTH4	KIFC3	[activation]
17749	GRB2	RACGAP1	[activation]
17750	NCOA3	HIST1H4A	[activation]
17751	AKAP2	PIK3R1	[activation]
17752	CDC16	DAB2	[activation]
17753	SDC4	CCL5	[activation]
17754	YWHAG	PFKFB2	[activation]
17755	MOB4	EGLN2	[activation]
17756	AURKB	CDC5L	[activation]
17757	PRKCA	AKAP12	[activation]
17758	NLRX1	FASTKD5	[inhibition]
17759	UBTF	TAF1C	[activation]
17760	DAPK3	PPP1R12A	[activation]
17761	CASP3	HNRNPU	[activation]
17762	ICT1	MRPL9	[activation]
17763	RICTOR	DHRS7	[activation]
17764	IGF2BP2	MYC	[activation]
17765	HSD17B4	UBA5	[activation]
17766	GZMA	HNRNPK	[activation]
17767	PPP2CA	RHO	[activation]
17768	HNRNPC	PDGFB	[activation]
17769	PAXIP1	WDR48	[activation]
17770	PGK1	MDM2	[activation]
17771	DLG3	SEMA4C	[activation]
17772	USP9Y	MAPT	[activation]
17773	TGFB1I1	SMAD7	[activation]
17774	RAD51B	XRCC2	[activation]
17775	ROS1	EGFR	[activation]
17776	CALM1	MYC	[activation]
17777	HSPA4	LIN37	[inhibition]
17778	HTATIP2	ETS1	[activation]
17779	NFE2L3	NFE2L2	[activation]
17780	HEMGN	DCC	[activation]
17781	ANXA1	MAP1S	[activation]
17782	HMGB1	HOXB1	[activation]
17783	TRPV1	CBL	[activation]
17784	IL21	DGKA	[activation]

17785	DDX47	MYC	[activation]
17786	CTNND1	GRIK2	[activation]
17787	PRMT1	RUNX1	[activation]
17788	HLA-B	GSPT1	[activation]
17789	ERBB2	UBASH3B	[activation]
17790	TRAF4	PTK2B	[activation]
17791	LRRK2	MYL6	[activation]
17792	SLA	ERBB2	[activation]
17793	EPRS	NXF1	[activation]
17794	PRKCI	HRAS	[activation]
17795	DDX39B	TUBB4A	[activation]
17796	MAP3K2	PIBF1	[activation]
17797	UBC	PIK3CG	[activation]
17798	MARK3	CPSF4	[activation]
17799	SH2B1	SH2B2	[activation]
17800	PLSCR3	PRKCD	[activation]
17801	PPARGC1A	USF2	[activation]
17802	SHC1	RASA1	[activation]
17803	LCK	SMAD2	[activation]
17804	CSF2RB	JAK2	[activation]
17805	CREBBP	TP53	[activation]
17806	FASLG	MMP7	[activation]
17807	SKP2	BTG2	[activation]
17808	HIPK3	TP53	[activation]
17809	MAPK3	TGIF1	[inhibition]
17810	WIPF1	HCK	[activation]
17811	USF1	PLEKHF2	[activation]
17812	LRRK2	PRPSAP2	[activation]
17813	DNAJB5	SMAD2	[inhibition]
17814	SRC	MAP4K1	[activation]
17815	FLNA	DRD2	[activation]
17816	LIN37	MYL12A	[activation]
17817	SRPK2	MARCH10	[activation]
17818	APP	CRIP2	[activation]
17819	PTEN	DBN1	[activation;inhibition]
17820	ATF2	PPIB	[activation]
17821	ZBTB17	EP300	[activation]
17822	CRK	TGOLN2	[activation]
17823	SIRT2	RELA	[activation]
17824	GNB3	MTOR	[activation]
17825	GSTK1	GRB2	[activation]
17826	PPP3CA	ABL1	[activation]
17827	MCF2L	GNB1	[activation]
17828	CEBPB	EP300	[activation]
17829	SUV39H1	SRGAP3	[activation]
17830	EFHC2	EGLN3	[activation]
17831	HSPA2	TP53	[activation]
17832	SMURF1	NUDT5	[inhibition]
17833	CCDC114	C1orf216	[activation]
17834	DCDC2	APP	[activation]
17835	YWHAB	SLITRK1	[activation]
17836	LYN	TNK2	[activation]
17837	IDE	IFIH1	[activation]
17838	RASGRP3	PRKCQ	[activation]
17839	KCNH1	FASTKD5	[activation]
17840	NCAPG	SMC4	[activation]
17841	HSP90AA1	PLCE1	[activation]
17842	RAE1	KLRK1	[activation]
17843	SRPK2	ARRB2	[activation]
17844	MAP3K3	GNAI2	[activation;inhibition]
17845	YWHAB	BID	[activation]
17846	HSPA2	MAP3K3	[activation]
17847	NISCH	GRB2	[activation]
17848	PTPN5	FYN	[activation]
17849	PRKCB	GCNT1	[activation]
17850	NGFR	MAPK3	[activation;inhibition]
17851	UBE2I	HSPB1	[activation]
17852	GRB2	IPO7	[activation]
17853	MLH1	NT5C3B	[activation]
17854	RAB6B	BMPR1B	[activation]
17855	ESR1	SMAD3	[activation]
17856	CTNNA2	CTNNB1	[activation]
17857	PIK3R1	HDAC3	[activation]
17858	EHMT2	RD3	[activation]
17859	MAPK6	SHC1	[activation]
17860	SIRT6	SMC2	[activation]

17861	APOL2	AR	[activation]
17862	TAB1	C2orf44	[inhibition]
17863	C1QA	CR1	[activation]
17864	F9	LRP1	[activation]
17865	PSEN1	RHEB	[activation]
17866	WAS	FYB	[activation]
17867	EIF4G1	MCC	[activation]
17868	KNG1	PLAUR	[activation]
17869	CDKN2A	HRAS	[activation]
17870	GAPDH	EP300	[activation]
17871	MAPRE3	EIF4E2	[activation]
17872	ZAP70	SHB	[activation]
17873	SPP1	BAG6	[activation]
17874	TRAF1	TNFRSF9	[activation]
17875	RRAGA	CPSF3	[activation]
17876	PPIA	OBSL1	[inhibition]
17877	MAPK3	BAZ1B	[activation]
17878	HLA-B	EEF1D	[activation]
17879	FLT3	SOCS2	[inhibition]
17880	NCK1	C6	[activation]
17881	KRBA1	PLK1	[activation;inhibition]
17882	HSPA2	CDKN1A	[activation]
17883	SRSF1	TP53	[activation]
17884	NTRK3	APP	[activation]
17885	IRS2	PTPN11	[activation]
17886	TP53	NCOA3	[activation]
17887	GSK3B	BAX	[inhibition]
17888	TP53	DNAJA1	[inhibition]
17889	CDC42BPG	RAC1	[activation]
17890	SNAI1	CHD4	[activation]
17891	CDC42BPB	LGR4	[activation]
17892	MAGEB4	ROCK1	[activation]
17893	CRHR1	UCN3	[activation]
17894	DSG1	CCDC8	[activation]
17895	MKRN3	UBE2E2	[activation]
17896	C1QA	CSPG4	[activation]
17897	NRIP1	RXRB	[inhibition]
17898	FZR1	C7orf25	[inhibition]
17899	CCNB1	ANAPC11	[activation]
17900	FAM74A4	STX11	[activation]
17901	CSNK1A1	VCAM1	[activation]
17902	ADRB2	VEGFB	[activation]
17903	HIST2H3A	STAT1	[activation]
17904	EFS	PLCG1	[activation]
17905	TP53	TTK	[activation]
17906	GNPTAB	MAPK1	[activation]
17907	EEF2	GRB2	[activation]
17908	PTPN11	MAP3K5	[inhibition]
17909	YWHAG	MAP3K6	[activation]
17910	MAPK1	DYRK1B	[activation]
17911	TPT1	CALM1	[activation]
17912	RRAGB	APP	[activation]
17913	APEX1	HLA-B	[activation]
17914	LNX2	NUMB	[activation]
17915	YWHAH	CBL	[activation]
17916	SIX4	SUMO2	[activation]
17917	IDE	CCL3	[activation]
17918	WDR20	PHLPP1	[inhibition]
17919	GNB2	ATF2	[activation]
17920	BCAS3	PTPN1	[activation]
17921	CYTH2	APC	[activation]
17922	CGN	LATS1	[inhibition]
17923	SOCS3	YES1	[inhibition]
17924	CDC42BPB	CDC42BPA	[activation]
17925	TBL3	URM1	[activation]
17926	TP53	PATZ1	[activation]
17927	GRB2	WDR1	[activation]
17928	FANCC	FANCF	[activation]
17929	AATF	MAPKAPK2	[activation]
17930	NFYC	KIAA2018	[activation]
17931	AXIN2	CDC20	[inhibition]
17932	ERBB2	TXK	[activation]
17933	EIF4E2	SPRY2	[inhibition]
17934	SCFD1	STX5	[activation]
17935	MMP14	MMP13	[activation]
17936	SUV39H1	SLFN12	[activation]

17937	GNB4	GNAI3	[activation;inhibition]
17938	MAPK3	GAB2	[activation]
17939	FOXO1	RFWD2	[activation]
17940	FARS2	ICT1	[activation]
17941	MAGEB2	SETD5	[activation]
17942	DAP3	PPARA	[activation]
17943	SNCA	CDK4	[activation]
17944	CSNK2B	APC	[activation]
17945	UNC13B	STX2	[activation]
17946	TP53TG1	TP53	[activation]
17947	USP20	ADRB2	[activation]
17948	EDA	LEPROTL1	[activation]
17949	PSEN2	CFLAR	[inhibition]
17950	RPS16	MLH1	[activation]
17951	EPS8	GRB2	[activation]
17952	CASP3	DEDD	[activation]
17953	OBSL1	SMARCA5	[inhibition]
17954	RIPK3	DNAJA1	[inhibition]
17955	RAI14	GRB2	[activation]
17956	KIAA1377	BARD1	[activation]
17957	NOXA1	NCF1	[activation]
17958	PAK2	HCK	[activation]
17959	DNAJC3	PRKRIR	[inhibition]
17960	HSP90AB1	MAP2K5	[activation]
17961	TBL3	EGFR	[activation]
17962	NR2F1	RXRA	[inhibition]
17963	HSPB1	MAP1LC3A	[activation]
17964	PRKCZ	EGFR	[activation]
17965	DNM2	SORBS1	[activation]
17966	CNKSR1	BRAP	[activation]
17967	ROBO2	ROBO1	[activation]
17968	SRPK2	MCOLN3	[activation]
17969	EVL	RAD51	[activation]
17970	LYPD3	PALB2	[activation]
17971	MAPK3	MAP3K3	[activation]
17972	ELSPBP1	EP300	[activation]
17973	FAM122B	MYC	[activation]
17974	MDM2	CLPP	[activation]
17975	ABL1	HSP90AB1	[activation]
17976	JUP	NFKBIE	[activation]
17977	SGTA	CSF1	[activation]
17978	CASP8AP2	CASP8	[activation]
17979	LRRK2	CKAP5	[activation]
17980	CSNK1D	KDR	[activation]
17981	MAPK3	SREBF2	[activation]
17982	FBXL5	PLK1	[activation]
17983	LAD1	SFN	[activation]
17984	CAMK2D	CD5	[activation]
17985	MAPKAP1	PRKDC	[activation]
17986	SCARB2	CAV1	[activation]
17987	TP53	DAB2IP	[activation]
17988	MAPK15	PIGR	[activation;inhibition]
17989	CDCA8	TTK	[activation]
17990	F2RL1	ST14	[activation]
17991	ACTG1	ANXA1	[activation]
17992	CD4	CXCR4	[activation]
17993	TP53	ATRX	[activation]
17994	NUP214	G3BP2	[activation]
17995	CDKN2AIP	RPA3	[activation]
17996	TNFRSF14	RNMTL1	[activation]
17997	RPS6KA5	GPRASP2	[activation]
17998	PA2G4	UBA5	[activation]
17999	CD81	MME	[activation]
18000	SPERT	PLEKHN1	[activation]
18001	CSNK1D	HN1L	[activation]
18002	DDX10	G3BP2	[activation]
18003	ATF4	GTF2A1L	[activation]
18004	PAK1	CAV1	[activation]
18005	SMAD4	FBXO34	[activation;inhibition]
18006	ANAPC7	SIRT6	[activation]
18007	RAB5A	RAB37	[activation]
18008	HSPA6	NFKB2	[activation]
18009	PIK3R2	HSPA6	[activation]
18010	PTBP3	RAC3	[activation]
18011	USHBP1	RASAL2	[activation]
18012	SRSF3	SRPK2	[activation]

18013	TIA1	VCAM1	[activation]
18014	ANXA5	TERF2IP	[activation]
18015	ARF6	RCC2	[activation]
18016	GRB2	CFL1	[activation]
18017	SMAD3	EIF4B	[activation]
18018	ERN1	FBXL21	[inhibition]
18019	AGTRAP	MUTYH	[activation]
18020	NAT8	SMAD4	[activation]
18021	APP	HSPG2	[activation]
18022	PTPN14	PPP2R1A	[inhibition]
18023	TNFRSF1B	SLC25A6	[activation]
18024	POLR2A	CASP10	[activation]
18025	E2F1	RARA	[activation]
18026	EP300	PTGS2	[activation]
18027	CDK5RAP2	TUBGCP5	[activation]
18028	CCNL2	CDK11A	[activation]
18029	OTX2	CDK4	[activation]
18030	RBM48	HSPB1	[activation]
18031	KPNB1	NXF1	[activation]
18032	SLC9A3R2	WWTR1	[activation]
18033	NXF1	TRAP1	[activation]
18034	EGFR	ADRBK1	[activation]
18035	GNG12	FHL2	[inhibition]
18036	NFE2L2	PGAM5	[activation]
18037	SRC	EZH2	[activation]
18038	MOS	SSX2IP	[inhibition]
18039	NUP62	THAP1	[activation]
18040	KLRC4	TYROBP	[activation]
18041	PIP5K1C	BTK	[activation]
18042	MDM2	PRRC2C	[activation]
18043	DSG1	GABARAPL2	[activation]
18044	SHB	IL2RG	[inhibition]
18045	GABRB2	PRKCB	[activation]
18046	IL24	ATM	[activation]
18047	IL6ST	CNTFR	[inhibition]
18048	CSNK2B	STAT5A	[activation]
18049	PLCG1	TRAIP	[activation]
18050	APOE	CNTF	[activation]
18051	STRN4	WNT7A	[inhibition]
18052	HNRNPH2	MDM2	[activation]
18053	LCK	PLCG1	[activation]
18054	TRAF6	NUMBL	[inhibition]
18055	FST	INHA	[activation]
18056	HNRNPAB	HIST3H3	[activation]
18057	GNAI2	TUBA4A	[activation;inhibition]
18058	BMX	BCAR1	[activation]
18059	NFKB1	NR4A1	[inhibition]
18060	LATS1	FSD2	[inhibition]
18061	IRF3	HERC5	[inhibition]
18062	TRIB3	RPGRIP1	[inhibition]
18063	F13A1	PLEK	[activation]
18064	CCNG1	GRN	[activation]
18065	MYC	TKT	[activation]
18066	KMT2D	AR	[activation]
18067	EPHA4	FGFR4	[activation]
18068	MAPK3	CREM	[inhibition]
18069	MTOR	LARS	[activation]
18070	EPAS1	BATF2	[activation]
18071	GNAI2	CXCR2	[activation]
18072	ANXA7	PAAF1	[activation]
18073	SHC1	BCL3	[activation]
18074	SUMO1	CAMK2D	[activation]
18075	SMAD3	RELA	[activation]
18076	TCAP	MSTN	[activation]
18077	GDF9	CCDC53	[activation]
18078	FBXO9	MTOR	[activation]
18079	STAT6	IFNAR1	[activation]
18080	SCAMP1	SNAP23	[activation]
18081	CCHCR1	EFHC2	[activation]
18082	SYK	LCK	[activation]
18083	RASSF8	PPP1CC	[inhibition]
18084	LRRK2	CEP72	[activation]
18085	RIPK2	HSPA6	[activation]
18086	EIF3F	HLA-B	[activation]
18087	TSSK6	MBP	[activation]
18088	BTK	PLAU	[activation]

18089	TERT	PINX1	[activation]
18090	NXF1	LRRC42	[activation]
18091	NCOA1	NR4A1	[activation]
18092	FOSB	JUNB	[activation]
18093	GNAI2	CCR5	[activation]
18094	TRAF6	SOCS3	[inhibition]
18095	KPNA2	RGL2	[activation]
18096	APP	VAMP3	[activation]
18097	FOXO3	GBAS	[inhibition]
18098	ZNF207	MAP2K2	[activation]
18099	ADRB2	STAT4	[activation]
18100	SMAD3	MAPK14	[activation]
18101	ERO1L	PELI2	[activation]
18102	WNK1	ZNF839	[activation]
18103	CXCL13	ACKR4	[activation]
18104	APBB1	ERBB2	[activation]
18105	BUB1B	KAT2B	[activation]
18106	CFL1	ARRB2	[activation]
18107	FYN	FCGR2A	[activation]
18108	MAFK	HOXD12	[activation]
18109	BHLHE40	CDPF1	[activation]
18110	CCL26	CCR1	[activation]
18111	PPP1CC	ANKRD42	[activation]
18112	RALGDS	ARRB1	[activation]
18113	MAPKAPK2	HSF1	[activation]
18114	WDR18	ESR2	[activation]
18115	LYN	CASP9	[activation;inhibition]
18116	MAP3K2	XIAP	[activation]
18117	FASLG	TEC	[activation]
18118	MYC	TEX2	[activation]
18119	CSF2RA	FATE1	[activation]
18120	CALM1	PTH1R	[activation]
18121	BCL2L1	TP53BP2	[activation]
18122	RFX7	IGHG1	[activation]
18123	DMC1	KCTD17	[activation]
18124	EGFR	GGCT	[activation]
18125	NTPCR	VCAM1	[activation]
18126	MAPK14	CAMKK2	[activation]
18127	FASN	MAPK3	[activation]
18128	MEIS2	APP	[activation]
18129	PML	CDK1	[activation]
18130	EIF1AX	EIF5B	[activation]
18131	CSNK2A1	ACACA	[activation;inhibition]
18132	NXF1	SFXN3	[activation]
18133	GCNT1	RSU1	[activation]
18134	ITGB1	ITGA2	[activation]
18135	ARRB2	HTR2C	[activation]
18136	ATP2B4	SRC	[activation]
18137	C3	MASP1	[activation]
18138	SUMO3	KIF23	[activation]
18139	TRAP1	APP	[activation]
18140	SH2D3A	GAB1	[activation]
18141	RGS13	PRKACA	[activation;inhibition]
18142	ARL5A	KPNA2	[activation]
18143	CP	IGHG1	[activation]
18144	RALGDS	LRPAP1	[inhibition]
18145	CXCR1	CDK1	[activation]
18146	RAD21	ACTR3	[activation]
18147	BATF2	ATF4	[activation]
18148	SNCA	CALM1	[activation]
18149	HNRNPA1	APP	[activation]
18150	TRAF6	HSPA1L	[activation]
18151	H3F3A	ITGA4	[activation]
18152	PARD6A	CRB3	[activation]
18153	NDRG1	SLC25A6	[activation]
18154	SMARCD1	HSPB1	[activation]
18155	PPM1A	CTNNB1	[activation]
18156	TRAP1	TNFRSF1A	[activation]
18157	BCL6	CACNA1A	[activation]
18158	SF3B4	FYN	[activation]
18159	HDAC3	BUB1B	[inhibition]
18160	YWHAZ	MAP3K4	[activation]
18161	CASP6	UBE4B	[activation]
18162	TP53	MTHFSD	[activation]
18163	PSEN1	EFHD1	[activation]
18164	CORT	SSTR3	[activation]

18165	RNFT1	NXF1	[activation]
18166	CDK4	PPP1CC	[inhibition]
18167	MAPKAPK3	DCC	[activation]
18168	PPP2CA	TRAF3IP3	[inhibition]
18169	WDR33	TGFBR1	[inhibition]
18170	CALM1	NR3C1	[activation]
18171	GLRX5	HLA-B	[activation]
18172	ABL1	GSX2	[activation]
18173	RAD21	APOB	[activation]
18174	PAK2	SORBS3	[activation]
18175	CDC42	WASF2	[activation]
18176	PAK1	SORBS3	[activation]
18177	VAPB	STX4	[activation]
18178	PPP1R9B	GRB2	[activation]
18179	GABARAP	NUP88	[activation]
18180	MCM2	PYHIN1	[activation]
18181	SYK	LAT	[activation]
18182	TRPV4	KRIT1	[activation]
18183	PRDM7	TRAF1	[activation]
18184	ATG14	PIK3C3	[activation]
18185	SRPK1	HIRIP3	[activation]
18186	JAK3	CCT8	[inhibition]
18187	GRB7	A1BG	[activation]
18188	RHPN2	CNKSR1	[activation]
18189	EIF4A1	CHD1L	[activation]
18190	BRCA2	PLK1	[activation]
18191	C2	C3	[activation;inhibition]
18192	MAFK	SMAD3	[activation]
18193	MDM2	RPS6	[activation]
18194	ABL1	TERT	[activation]
18195	BCL2A1	NR4A1	[inhibition]
18196	PAFAH1B3	BRD7	[activation]
18197	SUPT5H	HSPB1	[activation]
18198	CYTH3	FRMD4B	[activation]
18199	TDRD1	FYN	[activation]
18200	KIT	SH2D1A	[activation]
18201	INSIG1	SREBF2	[activation]
18202	APLP2	ETS1	[activation]
18203	CCDC70	FAM9B	[activation]
18204	FGFR1	HSP90AB1	[activation]
18205	EP300	RXRA	[inhibition]
18206	PARD6G	CDH5	[activation]
18207	AMBP	ENO1	[inhibition]
18208	C9orf78	MAPK11	[activation]
18209	EGFR	ZAP70	[activation]
18210	EDAR	TRAF3	[activation]
18211	RAD51	FANCD2	[activation]
18212	CREBBP	BCL3	[activation]
18213	DTX3L	UBE2E2	[activation]
18214	ADAM22	LYN	[activation]
18215	RAC1	PIP4K2A	[activation]
18216	KLRD1	KLRC1	[activation]
18217	NUDT3	CCL11	[activation]
18218	IGF1R	CAV1	[activation]
18219	GOSR1	DSC1	[activation]
18220	FGFR2	FRS2	[activation]
18221	PRKCZ	YWHAH	[activation]
18222	CYTH2	GRB2	[activation]
18223	GNA13	GPR17	[activation]
18224	RAB35	LATS1	[inhibition]
18225	CORO1C	DAPK1	[activation]
18226	ATG5	GSTM3	[activation]
18227	CAMK2B	CCM2L	[inhibition]
18228	KIFC3	NUP62	[activation]
18229	GRB2	GRB7	[activation]
18230	STAT3	ALK	[activation]
18231	PIK3R1	DARS	[activation]
18232	VCAM1	FARSB	[activation]
18233	EP300	NR2F2	[activation]
18234	TP53	ZNF24	[activation]
18235	EPS15L1	VCAM1	[activation]
18236	SYK	TLR4	[activation]
18237	CNTFR	CRAMP1L	[inhibition]
18238	AURKA	JTB	[activation]
18239	FAM173A	TP53	[activation]
18240	GRB2	CCL5	[activation]

18241	NXF1	CAV1	[activation]
18242	TERT	SMARCA2	[activation]
18243	CS	NXF1	[activation]
18244	LIMK1	MBP	[activation;inhibition]
18245	PHF13	SUMO3	[activation]
18246	ITPA	EGFR	[activation]
18247	TBK1	HMMR	[activation]
18248	ARPC5	LRRK2	[activation]
18249	MARCH1	TFRC	[activation]
18250	RUNX1	VDR	[activation]
18251	TGFBR2	CD44	[activation]
18252	PTPN6	FCGR2B	[activation;inhibition]
18253	MAP2K3	MAPK3	[activation]
18254	APP	IER3	[activation]
18255	DIRAS3	STAT3	[activation]
18256	SIRT1	DVL1	[activation]
18257	TP53	TOP2A	[activation]
18258	NDUFAF7	LRRK2	[activation]
18259	MAPK14	SLC12A2	[activation]
18260	PPP1CA	TP53	[activation]
18261	RAC1	CASP3	[activation]
18262	TUBB4A	RIPK1	[activation]
18263	WDR89	PHYHIP	[activation]
18264	SORBS1	YWHAB	[activation]
18265	BHLHE40	PRKAA1	[activation]
18266	SORBS2	VCL	[activation]
18267	LYN	ERO1L	[activation]
18268	CD163	PRKCA	[activation]
18269	ANAPC5	TFDP1	[inhibition]
18270	VCAM1	CAPNS1	[activation]
18271	BHLHE41	BHLHE40	[activation]
18272	VDAC1	CYCS	[inhibition]
18273	ATM	TRAF6	[activation]
18274	HSF1	SUMO2	[activation]
18275	APC	FAM214A	[inhibition]
18276	RAB18	EGFR	[activation]
18277	TP53	SKP1	[activation]
18278	MAP3K3	YWHAH	[activation]
18279	SRPK1	SPATS2	[activation]
18280	ABI2	CCDC102B	[activation]
18281	KMT2A	CSNK2A2	[activation]
18282	OPRL1	PNOC	[activation]
18283	PTPRE	PTPRS	[activation;inhibition]
18284	C16orf78	SRPK2	[activation]
18285	CAV1	APP	[activation]
18286	TCF19	NGFR	[activation;inhibition]
18287	ARF6	POLD1	[activation]
18288	UBXN7	APP	[activation]
18289	EIF4A3	PPM1B	[activation]
18290	LRRK2	AFG3L2	[activation]
18291	IRS2	CRK	[activation]
18292	SKIV2L2	RPA3	[activation]
18293	FBXO44	C17orf59	[inhibition]
18294	CCDC102B	PNMA5	[inhibition]
18295	DUSP1	CKS1B	[inhibition]
18296	NCL	STK4	[activation]
18297	PPP2R5A	PTTG1	[activation]
18298	MAPK14	GADD45A	[activation]
18299	CEBPA	FOS	[activation]
18300	PLD1	RAC1	[activation]
18301	HSPB1	MYLK	[inhibition]
18302	PRKCI	NIPSNAP1	[activation]
18303	TP53	AXIN1	[activation]
18304	HLA-B	PLS3	[activation]
18305	HRH4	CCL16	[activation]
18306	SRPK1	ODF3L2	[activation]
18307	HEATR1	MYC	[activation]
18308	MAP1LC3A	EGFR	[activation]
18309	MEN1	RPA1	[activation]
18310	FOS	YWHAB	[activation]
18311	STAT6	TBK1	[activation]
18312	EP300	GATA4	[activation]
18313	SMPD2	PIK3CG	[activation]
18314	S100B	MDM2	[activation]
18315	MADD	WNK1	[activation]
18316	EGFR	PUS1	[activation]

18317	CRK	GP9	[activation]
18318	MBL2	MASP1	[activation]
18319	PIK3R2	LAMB2	[activation]
18320	BMPR1B	AMHR2	[activation;inhibition]
18321	SMURF1	TRIB2	[inhibition]
18322	PTPRB	PXN	[activation]
18323	NOS2	SERPINE1	[inhibition]
18324	ANAPC7	EP300	[activation]
18325	TAOK1	MST4	[activation]
18326	ITPR1	FKBP1A	[inhibition]
18327	SGK2	APP	[activation]
18328	LAT	GRAP	[activation]
18329	DIAPH1	BAIAP2	[activation]
18330	EGFR	CDC25A	[activation]
18331	CDK5R1	ODF1	[activation]
18332	HSP90AB1	CD4	[activation]
18333	MDC1	RECQL5	[activation]
18334	ETS1	PAX5	[activation]
18335	VCAM1	PPP1CA	[activation]
18336	MTOR	PPP2R2A	[activation]
18337	TNK1	SFN	[activation]
18338	SYMPK	HSF1	[activation]
18339	RHOA	PITPNM1	[activation]
18340	YWHAB	KIAA1671	[activation]
18341	GNAI2	SUMO2	[activation;inhibition]
18342	ANXA5	TERF2	[activation]
18343	KCTD12	ATG5	[activation]
18344	ACACA	CBL	[activation;inhibition]
18345	CEBPA	TGFB2	[activation]
18346	ERRFI1	PALB2	[activation]
18347	KIT	SOCS6	[inhibition]
18348	SERTAD1	CREBBP	[activation]
18349	ALOX15B	RXRA	[inhibition]
18350	NCOA2	NCOA3	[activation]
18351	TP53	WDR77	[activation]
18352	LAT	PTPN1	[activation]
18353	KCNJ3	GNB1	[activation]
18354	OGN	SRC	[activation]
18355	RB1	PRKCB	[inhibition]
18356	HSPA8	ATM	[activation]
18357	PRKRIR	EIF2AK2	[activation]
18358	CD53	PRKCA	[activation]
18359	VDAC3	ATF2	[activation]
18360	HMMR	CD44	[activation]
18361	FCAR	PILRA	[activation]
18362	YWHAZ	MVP	[activation]
18363	FANCA	GRB2	[activation]
18364	FAIM2	STK11	[activation]
18365	ICT1	NME4	[activation]
18366	CEBPG	DDIT3	[activation]
18367	SPP1	PEMT	[activation]
18368	TREX1	ATR	[activation]
18369	POLR2A	RECQL5	[activation]
18370	MZT2B	CDK5RAP2	[activation]
18371	SMAD2	NFYC	[activation]
18372	GRB2	SLCO2A1	[activation]
18373	PAFAH1B1	NDEL1	[activation]
18374	CASP1	IL33	[activation]
18375	MSH4	MLH1	[activation]
18376	RAPGEF4	RRAS2	[activation]
18377	APOE	PSEN1	[activation]
18378	POLR3GL	CCND3	[inhibition]
18379	ARHGDIA	TWF2	[activation]
18380	ZBTB7B	GRB2	[activation]
18381	STK4	MBP	[activation]
18382	CAMK2A	SRF	[activation]
18383	CCDC137	MPP3	[inhibition]
18384	DBNL	MAP4K1	[activation]
18385	GSK3B	CEBPA	[activation;inhibition]
18386	ATF7IP	LAMA5	[activation;inhibition]
18387	OBSL1	IGF2BP1	[activation]
18388	PRKACA	EGFR	[activation]
18389	BRCA1	MLH1	[activation]
18390	CASP4	ELF4	[activation]
18391	SGK2	IL3RA	[activation]
18392	ASB5	DNAJA1	[inhibition]

18393	TRIP10	RHOQ	[activation]
18394	GRB2	DLGAP1	[activation]
18395	EHD4	RASGRF1	[activation]
18396	ZNF76	PIAS1	[inhibition]
18397	PBK	CDK1	[activation]
18398	FGG	ASB7	[activation]
18399	PRTN3	CAMP	[inhibition]
18400	PLXNB2	PLCG1	[activation]
18401	PPP3R2	PTPRJ	[activation]
18402	HNRNPA2B1	TP53	[activation]
18403	EGLN1	HIF3A	[activation]
18404	FANCD2	MEN1	[activation]
18405	VCAM1	PUM1	[activation]
18406	PRKAA1	NOTCH1	[activation]
18407	GRB2	CFH	[activation]
18408	EP300	SKP2	[activation]
18409	FGFR1	STAT3	[activation]
18410	PVR	NOTCH2NL	[activation]
18411	TMEM45B	TINAG	[activation]
18412	FAM124A	STAC3	[activation]
18413	HSPB1	CDC123	[activation]
18414	PTPRC	TEK	[activation]
18415	APP	CTNNB1	[activation]
18416	DYSF	COL12A1	[activation]
18417	FGF21	MAPK7	[activation]
18418	VAV3	LCP2	[activation]
18419	BCL2L1	BLID	[activation]
18420	BRD7	KIAA1377	[activation]
18421	GNB2	GNGT1	[activation]
18422	ASB16	RAN	[activation]
18423	SMYD3	TRIT1	[activation]
18424	CREB1	GLI2	[activation]
18425	NR3C1	MED1	[activation]
18426	ASPM	GPRASP2	[activation;inhibition]
18427	APOE	FBXL12	[activation]
18428	PTPN11	RET	[activation;inhibition]
18429	VCL	PTK2	[activation]
18430	ABL1	YWHAE	[activation]
18431	DDX21	GABARAP	[activation]
18432	DYRK1A	PRKACB	[activation]
18433	EGLN3	HIF3A	[activation]
18434	FOS	ZMYM6	[activation]
18435	SMAD3	EPB41L5	[inhibition]
18436	HSPG2	PYHIN1	[activation]
18437	FAS	CASP8AP2	[activation]
18438	RPL31	ICAM1	[activation]
18439	LTK	PTPN1	[activation]
18440	NAPA	VAMP8	[activation]
18441	AIRE	XPO1	[activation]
18442	XPO1	PPARG	[activation]
18443	PSG9	SMAD4	[activation]
18444	MAPK1	BCL6	[activation]
18445	ITCH	CYLD	[inhibition]
18446	CHD8	CHD7	[activation]
18447	WNK1	PVR	[activation]
18448	GLS	PARD6A	[activation]
18449	GADD45A	FAS	[inhibition]
18450	ARHGAP5	RASA1	[activation]
18451	CCNC	SOCS2	[inhibition]
18452	APEH	HLA-B	[activation]
18453	PRNP	AIFM3	[activation]
18454	PRKCI	MAP1LC3B	[activation]
18455	BET1	STX4	[activation]
18456	IGFBP7	INHBA	[activation]
18457	CDX2	CDK2	[activation]
18458	RAPH1	VASP	[activation]
18459	CRK	GAB2	[activation]
18460	NOS3	GUCY1B3	[activation]
18461	AGER	RHOA	[activation]
18462	CDK5	RNF32	[activation]
18463	FCGR1A	SYK	[activation]
18464	ELK1	MAPK9	[activation]
18465	SERPINE1	PTBP3	[inhibition]
18466	CD3D	CD8A	[activation]
18467	VPS33B	ZAK	[activation]
18468	ABL1	CREB1	[activation]

18469	ATG5	GABARAPL2	[activation]
18470	STAT1	PRMT1	[activation]
18471	PFDN1	PLXNB2	[activation]
18472	CAPZA3	ACTG1	[activation]
18473	IRAK3	ADH1B	[inhibition]
18474	H2AFX	PGK1	[activation]
18475	FYN	TRAIP	[activation]
18476	FOS	JUNB	[activation]
18477	IL1RAP	IL1R1	[activation;inhibition]
18478	KALRN	GNB1	[activation]
18479	NFKBIA	SPRR3	[activation]
18480	SRPK1	OCEL1	[activation]
18481	ALS2CR11	SSSCA1	[activation]
18482	C1orf123	CDKN1A	[activation;inhibition]
18483	KITLG	PIK3CD	[activation]
18484	WEE1	FBXW11	[inhibition]
18485	RPA3	LIMCH1	[activation]
18486	ASAP1	PXN	[activation]
18487	HRAS	SMAD4	[activation]
18488	U2AF2	SMAD2	[activation]
18489	SHC1	MOV10	[activation]
18490	TULP1	GRB2	[activation]
18491	AGTRAP	ZFYVE21	[activation]
18492	PPP1CA	PPP1R3B	[activation;inhibition]
18493	TP53	RPL11	[activation]
18494	IGHD	CD19	[activation]
18495	RRN3	UBTF	[activation]
18496	HSP90AA1	PTK2B	[activation]
18497	ANAPC2	HSP90AA1	[activation]
18498	SRC	AR	[activation]
18499	ABL1	CD19	[activation]
18500	ARFGEF2	GABRB1	[activation]
18501	SPARCL1	PRNP	[activation]
18502	CR2	C3	[activation]
18503	KAT2B	IRF1	[activation]
18504	CLIP4	TULP3	[activation]
18505	PRKAA1	BRSK1	[inhibition]
18506	ANXA7	NGFR	[activation]
18507	ABCC5	SMC2	[activation]
18508	EPS8L1	ACTG1	[activation]
18509	KDR	TXNIP	[activation]
18510	ACTR2	CTTN	[activation]
18511	SMARCA4	STAT3	[activation]
18512	CD47	FAS	[inhibition]
18513	CSK	PECAM1	[activation]
18514	RBPJ	KDM5A	[inhibition]
18515	FRMPD4	MYC	[activation]
18516	VIL1	JAK3	[inhibition]
18517	HLA-B	TXN	[activation]
18518	NXF1	SMARCE1	[activation]
18519	S1PR1	HTR1D	[activation]
18520	EPHA8	PIK3CG	[activation]
18521	PPP2R1A	LRRK2	[activation]
18522	YWHAB	CDK16	[activation]
18523	VPREB3	VPREB1	[activation]
18524	KRT40	HCK	[activation]
18525	FOXH1	AR	[inhibition]
18526	UBE2D2	TRAF6	[activation]
18527	GNAI1	DRD3	[activation]
18528	SRC	EPOR	[activation]
18529	TXNRD1	DMRTC2	[activation]
18530	HSP90AA1	BCR	[activation]
18531	GNB1	GNGT2	[activation]
18532	EGR1	CTNNB1	[activation]
18533	FNBP4	PLXNA2	[activation]
18534	CDK1	DAB2	[activation]
18535	KIF20A	RAB6B	[activation]
18536	CTSB	IGF1	[activation]
18537	CALM1	NFKB2	[activation]
18538	PML	HHEX	[activation]
18539	MET	BLK	[activation]
18540	FHL2	SMAD4	[activation]
18541	BTK	GAB1	[activation]
18542	HSPA4	BCL3	[activation]
18543	CLK1	PTPN1	[activation]
18544	HMGN1	PRKCA	[activation]

18545	CTU1	URM1	[activation]
18546	CSF2	PABPC1	[activation]
18547	CNPY4	TLR4	[activation]
18548	CDK5RAP3	CREBBP	[activation]
18549	EIF4EBP1	LATS1	[inhibition]
18550	MOB3A	LATS1	[inhibition]
18551	SMAD3	PTPN6	[activation;inhibition]
18552	LNX2	PIK3R3	[activation]
18553	UBE2V1	SUMO4	[activation]
18554	MAP3K14	RIPK1	[activation]
18555	PRC1	PLK1	[activation]
18556	TUBB	ZAP70	[activation]
18557	RABGAP1	TP53	[activation]
18558	ADRB2	CD4	[activation]
18559	SNX9	WAS	[activation]
18560	PFN1	CMA1	[activation]
18561	CHRD	NOTCH2NL	[inhibition]
18562	RAP1B	ZNF135	[activation]
18563	KMT2A	AFF4	[activation]
18564	PICALM	SIRT1	[activation]
18565	NUP210	ITGA4	[activation]
18566	EGLN3	OS9	[activation]
18567	MED27	RBPJ	[inhibition]
18568	PAXIP1	CATSPERG	[activation]
18569	HLA-B	AASDHPPT	[activation]
18570	YWHAB	SNCA	[activation]
18571	CDK4	HSP90AA1	[activation]
18572	RAD50	USP53	[activation]
18573	ZBTB7A	SOX9	[activation]
18574	SRC	DOK2	[activation]
18575	KMT2C	MAPK14	[activation]
18576	OSTF1	SRC	[activation]
18577	SRPK1	LYAR	[activation]
18578	EGFR	PPP2R5E	[activation]
18579	ENG	ACVR2B	[inhibition]
18580	VCAM1	IGHG1	[activation]
18581	STAT5B	APP	[activation]
18582	NR3C1	LCK	[activation]
18583	USP11	RAD50	[activation]
18584	FGD1	CTTN	[activation]
18585	ANXA1	RIPK1	[activation]
18586	FCHO1	SMURF1	[inhibition]
18587	SMARCAL1	RPA3	[activation]
18588	ACTB	HSD17B4	[activation]
18589	MDH1	TP53	[activation]
18590	PRPF8	ICAM1	[activation]
18591	PRG2	CSF2	[activation]
18592	ANAPC10	TP53BP1	[activation]
18593	ACTR2	CD44	[activation]
18594	SMAD1	PLEKHB1	[activation]
18595	MCM3	RAD52	[activation]
18596	MYC	SHOC2	[activation]
18597	HK1	ENO2	[activation]
18598	PRKACB	ARFGAP1	[activation]
18599	CAPNS1	GNB2	[activation]
18600	FBXO31	PARD6A	[inhibition]
18601	RACGAP1	ARF6	[activation]
18602	AURKA	UBE2N	[activation]
18603	NCOA1	SUMO1	[activation]
18604	BMPR1B	TTC27	[activation;inhibition]
18605	CREBBP	NR5A1	[activation]
18606	CHEK2	CCL5	[activation]
18607	HMGN3	SMAD2	[activation]
18608	STRN3	STK4	[activation]
18609	CD5	PRKCA	[activation]
18610	YWHAQ	TNF	[activation]
18611	PLG	MAPK14	[activation]
18612	PPP2CA	PXN	[activation]
18613	RXRA	ESR2	[inhibition]
18614	STK38	YWHAB	[activation]
18615	SMARCA4	KPNA2	[activation]
18616	MLLT4	PVRL4	[activation]
18617	EIF2S1	PPP1CC	[activation]
18618	ETS1	UBE2I	[activation]
18619	ARPC3	ITGA4	[activation]
18620	PFDN1	EIF4A2	[activation]

18621	PFN1	ATF2	[activation]
18622	PAK2	MKNK1	[activation]
18623	AURKA	LEF1	[activation]
18624	NOTCH1	MDM2	[activation]
18625	SHC1	ARHGEF5	[activation]
18626	KPNB1	SRY	[activation]
18627	KDR	FRS2	[activation]
18628	LATS1	RASSF1	[inhibition]
18629	HNRNPH3	CCL13	[activation]
18630	XRCC2	MEOX2	[activation]
18631	MDM2	TRIM4	[activation]
18632	DSCAM	PAK1	[activation]
18633	CACNA1A	WBP1	[inhibition]
18634	CRKL	WAS	[activation]
18635	CDCA8	SGOL1	[activation]
18636	PPP5C	MAPT	[activation]
18637	PTGDR	APP	[activation]
18638	RAC1	RPS6KB1	[activation]
18639	PDIA2	PLCG1	[activation]
18640	AURKB	CBX3	[activation]
18641	RANBP9	L1CAM	[activation]
18642	EPHB2	SH2D3C	[activation]
18643	EIF4EBP1	UBAC1	[inhibition]
18644	NR3C1	ALDH5A1	[activation]
18645	UBE2R2	PNPLA2	[activation]
18646	BUB1B	HDAC1	[inhibition]
18647	PIK3R1	CD2AP	[activation]
18648	MCM7	GABARAPL2	[activation]
18649	MAP3K4	BUB1B	[inhibition]
18650	CADPS2	UBE2E3	[activation]
18651	FGFR2	CBL	[activation]
18652	MYOD1	PRKCA	[activation]
18653	CAV1	KCNN3	[activation]
18654	CDK5R1	HDAC1	[activation]
18655	PPP2CA	SGOL2	[inhibition]
18656	ITGAV	PAK4	[activation]
18657	HSP90AB1	CHUK	[activation]
18658	ITK	HSP90AB1	[activation]
18659	TIFA	TRAF6	[activation]
18660	STX6	NXF1	[activation]
18661	SDC3	ARRB2	[activation]
18662	CPNE1	YWHAZ	[activation]
18663	EP300	NUTM1	[activation]
18664	ATR	HELQ	[activation]
18665	CREBBP	PRKCD	[activation]
18666	BMX	FAS	[activation]
18667	NR4A1	PPBP	[inhibition]
18668	BRCA1	JUN	[activation]
18669	PTPRA	HSPB1	[activation]
18670	PTK2	CSPG4	[activation]
18671	KCNB1	SNAP25	[activation]
18672	CDKN1A	GNB5	[activation;inhibition]
18673	BCL6	PIKFYVE	[activation]
18674	SMAD2	PCID2	[inhibition]
18675	NOV	CTGF	[activation]
18676	NGFR	RTN4R	[activation]
18677	PRDM5	EHMT2	[activation]
18678	TRDN	RYR1	[activation]
18679	NFE2L2	UBE2E3	[activation]
18680	NR4A1	MAPK8	[inhibition]
18681	UBE2F	STAT1	[activation]
18682	PPP2CB	HLA-B	[activation]
18683	IKBKB	IKBKAP	[activation]
18684	NCOA1	CCND1	[activation]
18685	SLC12A6	OXSR1	[activation]
18686	ERBB3	S100A10	[activation]
18687	RPA4	RPA2	[activation]
18688	SREBF2	TP53	[activation]
18689	PPP2R1A	CCDC6	[inhibition]
18690	OBSL1	RPL7L1	[activation;inhibition]
18691	CSNK1E	NCOA3	[activation]
18692	CCL20	TGFB3	[activation]
18693	VPREB3	IGHM	[activation]
18694	TP53	ACTB	[activation]
18695	HSD17B4	PTBP3	[activation]
18696	DDX5	IL7R	[activation]

18697	MSI2	VCAM1	[activation]
18698	TRAF2	LTBR	[activation]
18699	CAPZB	MDC1	[activation]
18700	NRXN1	NLGN2	[activation]
18701	CIAO1	POLA2	[activation]
18702	SH2D1B	KIT	[activation]
18703	SCD	MYC	[activation]
18704	GABARAP	PRKDC	[activation]
18705	ITGA5	ANGPT1	[activation]
18706	FAM133A	STAC3	[activation]
18707	BMP5	CHRDL2	[activation]
18708	FYN	SH2B2	[activation]
18709	F8	F10	[activation]
18710	HSPA8	PPP2R1A	[inhibition]
18711	IQGAP1	VCAM1	[activation]
18712	ACACA	MLLT1	[activation;inhibition]
18713	DGKD	PRKCA	[activation]
18714	PIK3R1	CSDE1	[activation]
18715	ARF1	FBXW8	[activation]
18716	CSF2	KITLG	[activation]
18717	TGFB1I1	PTPN12	[activation]
18718	CASP5	PPHLN1	[activation]
18719	TRIM24	PML	[activation]
18720	CDK4	SMC3	[inhibition]
18721	ABL1	EIF2A	[activation]
18722	LRRK2	TEX33	[activation]
18723	TP53	VPRBP	[activation]
18724	FZR1	E2F1	[inhibition]
18725	NEDD4	CBLB	[inhibition]
18726	GNAI2	TBXA2R	[activation;inhibition]
18727	GABARAPL2	TTBK2	[activation]
18728	TP53	RPS6	[activation]
18729	EGFR	IDH2	[activation]
18730	THRB	PPARGC1A	[activation]
18731	STAT6	ETS1	[activation]
18732	HNRNPD	EIF4G1	[activation]
18733	HCK	APP	[activation]
18734	DDX39B	STRN4	[activation]
18735	VAV2	ERBB3	[activation]
18736	PTK2B	HSP90AB1	[activation]
18737	ABI1	NIN	[activation]
18738	TP53	LAMA4	[activation]
18739	CSF1R	FYN	[activation]
18740	ABL1	TP53	[activation]
18741	F9	F7	[activation]
18742	BARD1	CSTF2	[activation]
18743	TNK2	WAS	[activation]
18744	PRKCI	YWHAZ	[activation]
18745	TAS1R3	TAS1R2	[activation]
18746	EZH2	AR	[activation]
18747	GNAS	FSCN1	[activation]
18748	MAP2K6	MAP2K4	[activation]
18749	WIPF3	GRB2	[activation]
18750	SUV39H1	MDM2	[activation]
18751	VCL	SORBS1	[activation]
18752	NCK2	CD3E	[activation]
18753	WHSC1	HNRNPA1	[activation]
18754	MEP1A	KNG1	[activation]
18755	ECH1	STAT3	[activation]
18756	MEIS1	HOXD4	[activation]
18757	GMPS	ITGA4	[activation]
18758	YWHAZ	PDE1A	[activation]
18759	GNAI3	S1PR1	[activation]
18760	KALRN	SNCAIP	[activation]
18761	ICK	APP	[activation]
18762	PARD6B	YWHAB	[activation]
18763	MMTAG2	EPB41L3	[activation]
18764	PDGFRB	SYNGAP1	[activation]
18765	JUN	SARS	[activation]
18766	MAPK1	TNIK	[activation]
18767	CAMKV	HSP90AB1	[activation]
18768	PPP1CC	EIF2A	[activation]
18769	BAX	ERN1	[inhibition]
18770	SIM2	NUDCD3	[activation]
18771	CASC1	BARD1	[activation]
18772	TERT	TPP1	[activation]

18773	VAV3	KIT	[activation]
18774	VCAM1	EIF3D	[activation]
18775	MRPS27	ERBB3	[activation]
18776	ZFYVE9	SUPT5H	[activation]
18777	SLAMF1	PTPN11	[activation]
18778	XPO1	HSP90AB1	[activation]
18779	ATF4	BATF	[activation]
18780	MARK3	PRKCQ	[activation]
18781	AURKB	FZR1	[activation]
18782	ARID1A	TP53	[activation]
18783	FASN	RAD21	[activation]
18784	RGS1	GDE1	[activation;inhibition]
18785	GRAP2	MAP4K1	[activation]
18786	KRT33A	GRB2	[activation]
18787	ABL2	CDC37	[activation]
18788	GSK3B	GPR39	[activation]
18789	CSMD2	CRK	[activation]
18790	A2M	SHBG	[inhibition]
18791	LUC7L2	HDHD2	[activation]
18792	TSPAN4	ITGB1	[activation]
18793	LAS1L	ESR2	[activation]
18794	SUMO3	FOS	[activation]
18795	CCL22	CRYAB	[activation]
18796	PRKCD	GABRA1	[activation]
18797	MPP6	SMARCA4	[activation]
18798	HOXD12	CREBBP	[activation]
18799	LSP1	MAP2K1	[activation]
18800	FTSJ3	GABARAPL2	[activation]
18801	CSNK2A1	MAPK14	[activation]
18802	COMMD8	NFKB1	[activation]
18803	CBL	ARHGEF7	[activation]
18804	FER	ERBB2	[activation;inhibition]
18805	NUMB	PRKCB	[activation]
18806	GRB2	SIRPA	[activation]
18807	EFNB1	ERBB2	[activation]
18808	BLK	EEF1E1	[activation]
18809	CAMLG	TRAF6	[activation]
18810	SPTAN1	STK4	[activation]
18811	STAT5B	CCR5	[activation]
18812	EIF2S2	SUMO3	[activation]
18813	PPP1CC	PCNA	[inhibition]
18814	ESR2	WNK4	[activation]
18815	MLLT4	LGR4	[activation]
18816	NLK	CUL1	[inhibition]
18817	RHNO1	GOLGA2	[activation]
18818	ERLEC1	STK11	[activation]
18819	PRRC2B	IRF4	[activation]
18820	EPHB1	PXN	[activation]
18821	LILRB2	HLA-C	[activation]
18822	TP73	RCHY1	[activation;inhibition]
18823	MITF	TFE3	[activation]
18824	CALM1	MYO10	[activation]
18825	CASP1	BCAP31	[activation]
18826	TTK	RPS6KA1	[activation]
18827	RGS10	ACP6	[activation;inhibition]
18828	HSPB1	GRIPAP1	[activation]
18829	SHANK3	PLCG1	[activation]
18830	CAMK2B	MORF4L1	[activation]
18831	RARG	SPHK1	[activation]
18832	FBXO6	LAMB1	[inhibition]
18833	NSMAF	GABARAPL2	[activation]
18834	SHC1	PLCG1	[activation]
18835	SOCS1	CRK	[inhibition]
18836	MYC	NFIL3	[activation]
18837	LAT	PLCG1	[activation]
18838	EHMT2	E4F1	[activation]
18839	CD40LG	IGKC	[activation]
18840	PAFAH1B1	NUDC	[activation]
18841	NOTCH1	NOV	[activation]
18842	THOP1	EGFR	[activation]
18843	VCAM1	HNRNPM	[activation]
18844	CCKBR	GRB2	[activation]
18845	YTHDF3	RPA1	[activation]
18846	NR0B2	PPARG	[activation]
18847	WASL	CD44	[activation]
18848	ABL1	AIRE	[activation]

18849	JAK1	CCR5	[activation]
18850	LYN	PECAM1	[activation]
18851	FOXO1	SRC	[activation]
18852	CHEK2	BRCA2	[activation]
18853	MYC	MFGE8	[inhibition]
18854	PARD6G	LLGL2	[activation]
18855	GNAT1	UNC119B	[activation;inhibition]
18856	ACTG1	VIL1	[activation]
18857	PRKCQ	WIPF1	[activation]
18858	ENO1	VCAM1	[activation]
18859	SPRY1	GRB2	[activation]
18860	IKBKE	CYLD	[inhibition]
18861	MMP13	TIMP3	[activation]
18862	RAD21	EVL	[activation]
18863	GRB2	DCTN2	[activation]
18864	SMC2	SUMO2	[activation]
18865	PHLDA3	SMC4	[activation]
18866	PGR	ZBTB17	[activation]
18867	PPAP2C	MLH1	[activation]
18868	TP53	ANXA3	[activation]
18869	ACTN1	PSEN1	[activation]
18870	ATF2	ERP29	[activation]
18871	CDC23	ATRIP	[activation]
18872	CACNB2	REM1	[activation]
18873	PSIP1	PPP2R1A	[activation]
18874	FAS	FADD	[inhibition]
18875	LNX2	TRAF2	[activation]
18876	OTUD4	STAT1	[activation]
18877	ACTN4	BMP7	[activation]
18878	FOS	GATA3	[activation]
18879	TNFRSF11A	TRAF1	[activation]
18880	TMX1	ATF2	[activation]
18881	RPL38	HLA-B	[activation]
18882	PRKDC	CHEK2	[activation]
18883	TP53	PARP1	[activation]
18884	TGFBR1	CHN1	[activation]
18885	GRB2	WBP11	[activation]
18886	GHR	NCK1	[activation]
18887	GRB2	ESR1	[activation]
18888	ICT1	MRPL16	[inhibition]
18889	GOLGA2	PPP2CA	[inhibition]
18890	HMGA2	PRMT6	[activation]
18891	SMAD3	SUV39H1	[activation]
18892	ZFP36	TFRC	[activation]
18893	PPP2R1A	PACSIN1	[activation]
18894	TNFSF11	NFKBIA	[activation]
18895	ULK2	CREB1	[activation]
18896	TNS3	MET	[activation]
18897	NUP214	ITGA4	[activation]
18898	ATF2	BSG	[activation]
18899	CREBBP	ACVR1	[activation;inhibition]
18900	ARHGEF11	LPAR2	[activation]
18901	AURKB	TP53	[activation]
18902	ATM	TPT1	[activation]
18903	NDP	BAG3	[activation]
18904	MMP9	CXCL1	[activation]
18905	RNF10	UBE2E2	[activation]
18906	CCDC59	ILK	[activation]
18907	CSK	ADRB2	[activation]
18908	MYOD1	JUN	[activation]
18909	HNRNPL	EIF2B1	[activation]
18910	HSPA5	EIF2AK3	[activation]
18911	ITGB2	ITGAX	[activation]
18912	RDH12	ANXA8	[activation]
18913	YPEL2	SRPK2	[activation]
18914	MTUS2	HCK	[activation]
18915	FYN	GP6	[activation]
18916	MLH1	ATAD3B	[activation]
18917	SMAD6	MAPK6	[activation;inhibition]
18918	RPS6KA5	RELA	[activation]
18919	RAB6B	RPA3	[activation]
18920	PRDX2	CA4	[activation]
18921	ZNF598	RPA3	[activation]
18922	NUDCD3	TSSC1	[activation]
18923	SRSF10	SREK1	[activation]
18924	SGK1	IGHA1	[activation]

18925	BTK	PRKCA	[activation]
18926	HSP90AB1	STK33	[activation]
18927	C1orf216	CEP170P1	[activation]
18928	IGFBP3	RXRA	[inhibition]
18929	STK4	GABARAP	[activation]
18930	JUN	PRKD1	[activation]
18931	PLG	PRNP	[activation]
18932	CDK4	MTFP1	[inhibition]
18933	CDKN1A	STAT3	[activation]
18934	EMILIN1	CDC23	[activation]
18935	DDX21	SRPK2	[activation]
18936	RHOC	CAV1	[activation]
18937	PLCG1	EXTL3	[activation]
18938	CBL	ABI1	[activation]
18939	MECOM	SMAD1	[activation;inhibition]
18940	EDN2	KEL	[activation]
18941	YKT6	USO1	[activation]
18942	PTPRB	CDH5	[activation]
18943	EFNB2	RHBDL2	[activation]
18944	MEN1	CHEK1	[activation;inhibition]
18945	GHRH	DPP4	[activation]
18946	TANGO6	MACF1	[activation]
18947	AMBP	F2	[activation]
18948	GABARAPL2	EZR	[activation]
18949	CBLB	GRB2	[activation]
18950	RPA2	EIF4G2	[activation]
18951	HLA-E	KLRC1	[activation]
18952	PIAS1	PTK2	[activation]
18953	RPTOR	RPS6	[activation]
18954	MAPK6	CCND3	[inhibition]
18955	GRM5	PRKCQ	[activation]
18956	PRKCB	SDC2	[activation]
18957	SPTBN1	MAPK14	[activation]
18958	GNB2	CDKN1A	[activation]
18959	ARAP1	VCAM1	[activation]
18960	RECQL5	CREB1	[activation]
18961	NFKB1	ANXA4	[activation]
18962	BMP7	ACVR2B	[activation]
18963	IL27RA	MAPK8	[activation]
18964	SMYD3	HSP90AA1	[activation]
18965	TOMM70A	HSPA1A	[inhibition]
18966	MAPK1	ATF2	[activation]
18967	RIPK1	NUMBL	[inhibition]
18968	CUL1	CHEK1	[inhibition]
18969	SHARPIN	ITGA1	[activation]
18970	WASF1	BAD	[inhibition]
18971	PKN2	AKT1	[activation]
18972	DOPEY2	EGFR	[activation]
18973	ITGA4	RPS24	[activation]
18974	BARD1	BEND3	[activation]
18975	CD3E	PTPN22	[activation]
18976	AXIN1	SKIL	[activation]
18977	PVRL3	PVRL1	[activation]
18978	RELA	TRIB3	[activation]
18979	MAPK1	SHANK3	[activation]
18980	HSPB1	STAT2	[activation]
18981	MRPL39	TP53	[activation]
18982	EPHB2	PTP4A2	[activation]
18983	KPNA2	CHEK2	[activation]
18984	TP53	CSNK2A2	[activation]
18985	SMURF1	GNG11	[inhibition]
18986	ADRB2	DLAT	[activation]
18987	NXF1	EIF1AD	[activation]
18988	SGK223	HSP90AB1	[activation]
18989	AMOTL1	LATS1	[inhibition]
18990	PCK2	EP300	[activation]
18991	AURKB	CDK1	[activation]
18992	RGS11	CDC37	[activation;inhibition]
18993	MAPK1	HMMR	[activation]
18994	ITGAV	PPAP2B	[activation]
18995	RUNX1	EFNA2	[activation]
18996	TGM3	ZBTB1	[activation]
18997	MAP2K4	ITCH	[activation]
18998	PRMT1	IGSF21	[activation]
18999	ANXA6	RASA1	[activation]
19000	SRPK1	GABARAPL1	[activation]

19001	ABI1	MINK1	[activation]
19002	TYSND1	SCP2	[activation]
19003	TXLNA	TBK1	[activation]
19004	CDC25C	CHEK2	[activation]
19005	SCTR	PRKCA	[activation]
19006	CFHR3	C3	[activation]
19007	MDH1	ATG5	[activation]
19008	RASL10B	FGFR2	[activation]
19009	NEDD1	ACTB	[activation]
19010	CEP120	STAT3	[activation]
19011	ITGB1	RANBP9	[activation]
19012	PRMT1	MLST8	[activation]
19013	TP53	GRB2	[activation]
19014	HRG	SPRY1	[inhibition]
19015	EPHA2	GATAD1	[activation]
19016	STAT5A	UGT2B4	[activation]
19017	ELN	ELANE	[activation]
19018	RXFP3	APP	[activation]
19019	IRF7	IQSEC1	[activation]
19020	DIXDC1	MAP3K4	[activation]
19021	PSEN1	CASP3	[activation]
19022	SMAD3	SRC	[activation]
19023	TAB1	HSPA4L	[inhibition]
19024	PIAS4	NR4A2	[inhibition]
19025	CSNK1E	ADAM22	[activation]
19026	MYC	NPC1L1	[activation]
19027	TPX2	AURKB	[activation]
19028	VCAM1	HSPE1	[activation]
19029	C3	TGM2	[activation]
19030	MYC	TIAM1	[activation]
19031	CD3G	CD3D	[activation]
19032	HLA-B	RAB1B	[activation]
19033	HNF1B	HIST1H3A	[activation]
19034	ITGA4	TTLL12	[activation]
19035	RASGRF1	MDM2	[activation]
19036	AURKA	IKBKB	[activation]
19037	KLC2	PIK3R3	[activation]
19038	CAND1	SRPK1	[activation]
19039	ERH	SETDB1	[activation]
19040	PRKG2	HSP90AB1	[activation]
19041	GNB1	GNAI2	[activation]
19042	GRB2	CD164	[activation]
19043	BMPR1A	GDF9	[activation]
19044	ABL1	ROS1	[activation]
19045	MAST3	HNRNPA0	[activation]
19046	TSHR	GNAS	[activation]
19047	TLR1	TRAP1	[activation]
19048	JUP	CCDC130	[activation]
19049	TRAF3	NMRAL1	[activation]
19050	PLCB3	PARD6A	[activation]
19051	NCK1	NEK8	[activation]
19052	NCK1	DAG1	[activation]
19053	WDR5	MYB	[activation]
19054	MAP3K3	MAP3K7	[activation]
19055	HIF1A	FHL2	[activation]
19056	LIFR	IL31RA	[activation]
19057	DYSF	ANXA2	[activation]
19058	ANP32B	VCAM1	[activation]
19059	IKBKB	FAF1	[activation]
19060	PAXIP1	ANAPC7	[activation]
19061	CASP8	EDA2R	[activation]
19062	SHC1	MAPK1	[activation]
19063	A2M	RAP1B	[inhibition]
19064	ZMYM4	HIST3H3	[activation]
19065	VCAM1	CLUH	[activation]
19066	RNF8	MDM2	[activation]
19067	MACF1	CCDC8	[activation]
19068	YAP1	ENO1	[inhibition]
19069	AR	CCNE1	[activation]
19070	ARRB1	PTGER2	[activation]
19071	FANCB	FANCA	[activation]
19072	BRAF	HSPA8	[activation]
19073	DZIP3	UBE2E2	[activation]
19074	EXOC6	PHLDA3	[activation]
19075	SRPK2	CAND1	[activation]
19076	PIK3C3	CALM1	[activation]

19077	STAT3	HNF1A	[activation]
19078	LYN	RDX	[activation]
19079	ABI2	PRR16	[activation]
19080	CHUK	TANK	[activation]
19081	BAG3	GRB2	[activation]
19082	PPP2R5D	YWHAB	[activation]
19083	SRC	SPTAN1	[activation]
19084	STAT3	CAPN1	[activation]
19085	BAAT	RB1	[activation]
19086	MAPK1	PARP1	[activation]
19087	BCL2L11	FEM1C	[activation]
19088	HRNR	MDM2	[activation]
19089	HIST1H3A	TAF1B	[activation]
19090	NCOA2	YWHAH	[activation]
19091	SLC9A3R1	FZD4	[activation]
19092	PALB2	CST6	[activation]
19093	RASA1	KHDRBS1	[activation]
19094	CD81	CSNK2B	[activation]
19095	IGHG1	TNFSF10	[activation]
19096	PTEN	AMHR2	[activation]
19097	EIF2AK2	TYK2	[activation]
19098	FOXP4	FOXP2	[activation]
19099	PITX2	PROP1	[activation]
19100	TNK1	DDX5	[activation]
19101	SNW1	NOTCH3	[activation]
19102	F2RL2	ECT2	[activation]
19103	E2F3	WNK1	[activation]
19104	PRKCZ	CFL1	[activation]
19105	DDX5	CRK	[activation]
19106	PRKDC	CENPA	[activation]
19107	YAE1D1	GABARAP	[activation]
19108	FAAP24	EME2	[activation]
19109	SRPK1	FBXO15	[activation]
19110	ID1	CAV1	[activation]
19111	DDX43	APP	[activation]
19112	CSNK2A1	PAK1	[activation]
19113	IRAK2	TIRAP	[activation]
19114	GNB5	PSD	[activation]
19115	PTEN	C9orf156	[activation;inhibition]
19116	PIK3CB	AGTR2	[activation]
19117	IKBKB	MAVS	[activation]
19118	IL12B	IL12A	[activation]
19119	PIK3R1	ASAP1	[activation]
19120	NAMPT	DDA1	[activation]
19121	MAP3K10	CNKSR1	[activation]
19122	RHOB	PHGDH	[activation]
19123	ENG	TGFBR2	[inhibition]
19124	TRIM59	TP53	[activation]
19125	DOK1	ITGB1	[activation]
19126	PTBP3	APC	[activation]
19127	MYL6	GRB2	[activation]
19128	LATS1	TRAF1	[inhibition]
19129	RANBP10	PRKCD	[activation]
19130	PTCH1	ADRBK1	[activation]
19131	ETS1	PHF14	[activation]
19132	MSL1	RAN	[activation]
19133	STK11	ADIPOR1	[activation]
19134	NUDCD3	GNAZ	[activation;inhibition]
19135	PTPN6	PRKCA	[activation;inhibition]
19136	EVL	APBB1IP	[activation]
19137	MAP2K5	GRB2	[activation]
19138	PPM1H	YWHAB	[activation]
19139	MYC	NRXN3	[activation]
19140	SRC	EPS8	[activation]
19141	PYHIN1	RAD50	[activation]
19142	NR4A1	GADD45GIP1	[inhibition]
19143	EIF2S2	EIF4G2	[activation]
19144	PEBP4	MAP2K1	[activation]
19145	MAPK1	PPP1CC	[inhibition]
19146	MYC	CYC1	[activation]
19147	PAX8	SERINC1	[activation]
19148	GOLGA8F	ATF4	[activation]
19149	YES1	AR	[activation]
19150	TIMP1	MMP3	[activation]
19151	NR3C1	HSPA1A	[activation]
19152	PPP2R2D	DAPK1	[activation]

19153	ACTB	ASB12	[activation]
19154	TNFRSF14	C2orf47	[activation]
19155	MACF1	ACTA1	[activation]
19156	KIFC3	ATF2	[activation]
19157	ATF2	PRDX3	[activation]
19158	ARHGEF7	NCK1	[activation]
19159	PTPRJ	EPOR	[activation]
19160	CDC27	CASP3	[activation]
19161	RAP2B	CAMK2B	[activation]
19162	VCAM1	YARS	[activation]
19163	FYB	ENAH	[activation]
19164	SMARCA4	NR1H2	[activation]
19165	PAX7	FYN	[activation]
19166	CDK11B	CCND3	[inhibition]
19167	NR1H3	TMEM161A	[activation]
19168	STK3	WWTR1	[activation]
19169	TNF	LIN54	[activation]
19170	ENO1	RIPK2	[activation]
19171	BFAR	CASP8	[inhibition]
19172	RNF32	EXOC6	[activation]
19173	TP53BP1	MYL12A	[activation]
19174	PARD3B	SMAD1	[activation]
19175	PRKDC	YY1	[activation]
19176	CALD1	OBSL1	[activation;inhibition]
19177	GRN	CCDC33	[activation]
19178	GSK3B	CDK5	[activation]
19179	IGF1R	DOK5	[activation]
19180	RNASE1	RELA	[activation]
19181	POLR2C	STC2	[activation]
19182	APP	NR1D1	[inhibition]
19183	SUMO3	RAN	[activation]
19184	FGR	WAS	[activation]
19185	FTSJ1	EIF1B	[activation]
19186	ALDH9A1	HLA-B	[activation]
19187	HNRNPA1	CARF	[activation]
19188	PAK2	HTT	[activation]
19189	STX17	EGFR	[activation]
19190	LYN	CDKN1B	[activation;inhibition]
19191	KAT2B	HMGN2	[activation]
19192	PSTPIP1	PTPN1	[inhibition]
19193	DNM1	SNAP25	[activation]
19194	YWHAZ	CACNA1S	[activation]
19195	LATS1	PTPN14	[inhibition]
19196	LAT	FCGR1A	[activation]
19197	ESR1	SP1	[activation]
19198	YWHAZ	MAGEB2	[activation]
19199	VAT1	TP53	[activation]
19200	HES1	PRKCA	[activation]
19201	NXF1	AGGF1	[activation]
19202	SKP2	CCNA2	[activation]
19203	S100A2	AREG	[activation]
19204	C5orf42	PAK1	[activation]
19205	RASA1	RAB5A	[activation]
19206	CXCR5	GNAO1	[activation]
19207	EGFR	NRG1	[activation]
19208	RPA2	SMARCA5	[activation]
19209	CDK5	CCNE1	[activation]
19210	TGFBR3	TGFBR2	[inhibition]
19211	NMT2	PRKCZ	[activation]
19212	CDC23	KCTD6	[activation]
19213	EGFR	EIF2B3	[activation]
19214	APLP1	SNCA	[activation]
19215	PLD2	STXBP1	[activation]
19216	PADI4	TP53	[activation]
19217	CCDC8	SERPINB12	[inhibition]
19218	THOP1	CALM1	[activation]
19219	GNB1	AVPR2	[activation]
19220	IQGAP1	SUMO3	[inhibition]
19221	ACTG1	SUMO1	[activation]
19222	GNG12	CAPN1	[activation]
19223	H2AFX	RNF8	[activation]
19224	MARK4	PRKCI	[activation]
19225	LCP1	CNN3	[activation]
19226	MRE11A	SNW1	[activation]
19227	CDK1	VCAM1	[activation]
19228	NRP1	PLXNA2	[activation]

19229	RFC2	RPA1	[activation]
19230	IFNAR1	CRKL	[activation]
19231	PRKCA	NRGN	[activation]
19232	PKIB	E2F3	[activation;inhibition]
19233	TP53	PPP2R5C	[activation]
19234	EIF4A3	PFKFB3	[activation]
19235	BCL2L1	BMF	[activation]
19236	EGFR	NCAPH	[activation]
19237	APP	SETD3	[activation]
19238	DNAJA2	RB1	[inhibition]
19239	SMAD2	IRF7	[activation]
19240	SPN	ICAM1	[activation]
19241	CRK	FYN	[activation]
19242	FADS1	DHCR7	[activation]
19243	IGHA1	STK4	[activation]
19244	HSPA4	RELA	[activation]
19245	SYVN1	SHH	[inhibition]
19246	RASSF9	HSPB1	[activation]
19247	F2R	GNAT1	[activation]
19248	CCR10	CCR7	[activation]
19249	CAMK4	HSP90AA1	[activation]
19250	ESRRG	NR0B1	[activation]
19251	TBC1D15	VCAM1	[activation]
19252	PRKCA	GABRR2	[activation]
19253	CD247	CD244	[activation]
19254	DDIT3	F2	[activation]
19255	CRK	RPP38	[activation]
19256	MAVS	TSPAN6	[activation]
19257	LRRK2	GSK3B	[activation]
19258	BUB1B	DAXX	[inhibition]
19259	HDAC7	FOXP3	[activation]
19260	XIRP2	DYSF	[activation]
19261	ITPKA	PRKCA	[activation]
19262	ESR1	GADD45B	[activation]
19263	COL1A1	MMP9	[activation;inhibition]
19264	SPP1	EPAS1	[activation]
19265	FYN	KCNA5	[activation]
19266	HLA-B	COPS3	[activation]
19267	PRKACA	GABRB3	[activation]
19268	AKT1	IRAK1	[activation;inhibition]
19269	CSN1S1	TTBK1	[activation]
19270	USP1	ANAPC1	[activation]
19271	CRK	DAB1	[activation]
19272	MYC	MAP3K5	[activation]
19273	RMDN3	WNK1	[activation]
19274	HNRNPC	FAS	[activation]
19275	EGFR	MAP2K4	[activation]
19276	ARPC4	GRB2	[activation]
19277	STAT3	NXF1	[activation]
19278	LEF1	RB1	[inhibition]
19279	KRAS	MOCS2	[activation]
19280	GNB2	ATXN10	[activation]
19281	GAD1	GRB2	[activation]
19282	BMPR1B	RASD2	[activation]
19283	APLP1	PRNP	[activation]
19284	GSC	SMAD1	[activation]
19285	APP	GIMAP5	[activation]
19286	MAPT	PKN1	[activation]
19287	PTPN11	EPHA2	[activation]
19288	MYO6	TP53	[activation]
19289	MAFF	NFE2L2	[activation]
19290	CAMK1	CDKN1B	[inhibition]
19291	SRPK3	NCL	[activation]
19292	ARHGAP21	PTK2B	[activation]
19293	CASP3	BIRC6	[activation]
19294	NLGN1	DLG3	[activation]
19295	GADD45A	PTPRK	[inhibition]
19296	LZTR1	STAT1	[activation]
19297	MAPKAPK5	TAB1	[activation]
19298	GADD45A	FTL	[activation]
19299	WASF2	ABI2	[activation]
19300	HDAC3	HIF1A	[activation]
19301	TIMM50	RELA	[activation]
19302	CD4	DDX5	[activation]
19303	CDC42	IQGAP2	[activation;inhibition]
19304	MEIS1	HOXA11	[activation]

19305	CDC5L	NUDC	[activation]
19306	NR3C1	IFNGR2	[activation]
19307	PPP2R1A	HDAC4	[activation]
19308	GLI1	SMAD4	[inhibition]
19309	SCAMP3	EGFR	[activation]
19310	RAPGEF1	SHC1	[activation]
19311	CEBPB	FOXO1	[activation]
19312	CEP76	PLCB1	[activation]
19313	PARD3	PARD6A	[activation]
19314	FANCL	FANCF	[activation]
19315	ADORA1	ATXN1L	[activation;inhibition]
19316	PLA2G10	PLA2R1	[activation]
19317	ACTA1	MAPK14	[activation]
19318	BCCIP	CTNNB1	[activation]
19319	ATG5	DDX58	[activation]
19320	ACAP1	VAMP3	[activation]
19321	CDH2	PTPN6	[activation]
19322	SETD3	RAD21	[activation]
19323	SMAD1	KAT2A	[activation]
19324	ICT1	ACOT9	[activation]
19325	CASP7	CFLAR	[inhibition]
19326	MAP3K7	CNTN2	[activation]
19327	JAG2	MIB2	[activation]
19328	GADD45A	IGSF21	[activation]
19329	MYC	KLF10	[activation]
19330	DHX57	GABARAP	[activation]
19331	APC	MCM5	[activation]
19332	SRPK1	BARD1	[activation]
19333	GRB2	MYO1B	[activation]
19334	GNAI1	GNB1	[activation]
19335	HSP90AA1	STAT2	[activation]
19336	ARPC2	ARPC1B	[activation]
19337	PLCG1	CD28	[activation]
19338	IL6R	STAT3	[activation]
19339	RPS6KA3	HSP90AB1	[activation]
19340	ITSN2	TRIO	[activation]
19341	CD247	RGAG1	[activation]
19342	SIRT2	CDC20	[activation]
19343	HSP90AA1	CAMKK1	[activation]
19344	PRPF6	AR	[activation]
19345	CSF2	ILF2	[activation]
19346	KMT2A	CTNNB1	[activation]
19347	CDC42	RAP1GDS1	[activation;inhibition]
19348	MAVS	EIF2AK2	[activation]
19349	STX5	SNAP25	[activation]
19350	DOCK1	RAC1	[activation]
19351	IL7	APP	[activation]
19352	ANKRD52	NXF1	[activation]
19353	AR	MAPK1	[activation]
19354	NBN	SIRT1	[activation]
19355	PPP6C	AURKB	[activation]
19356	XPO1	E2F4	[activation]
19357	PLK1	NEK9	[activation]
19358	FLNB	HSPB1	[activation]
19359	ABL1	CNTFR	[activation]
19360	NUDC	WDR31	[activation]
19361	SNW1	NUP153	[activation]
19362	JKAMP	PTGDR2	[activation]
19363	BAG6	INSIG2	[activation]
19364	CDK5R1	KRT40	[activation]
19365	SGTA	NME3	[activation]
19366	APP	CDK4	[inhibition]
19367	GNB1	GNA12	[activation]
19368	NOTCH2NL	PVRL2	[activation]
19369	ETS1	MAF	[activation]
19370	MCM4	CCNA1	[activation]
19371	IRF8	CDK4	[inhibition]
19372	CDC37	GLMN	[activation]
19373	PPARG	RXRA	[inhibition]
19374	ANXA1	PRKCI	[activation]
19375	CAD	RICTOR	[activation;inhibition]
19376	SERPINI2	SGTB	[inhibition]
19377	HSP90AB1	CSF1R	[activation]
19378	RAN	ITGA4	[activation]
19379	RIPK1	KHDRBS1	[activation]
19380	TDGF1	FARSA	[activation]

19381	MAP3K8	HSPA8	[inhibition]
19382	PPP1R12A	FYN	[activation]
19383	PRNP	CSN3	[activation]
19384	SNAI1	AJUBA	[inhibition]
19385	STK16	KCTD14	[activation]
19386	UBE2E2	ZNRF1	[activation]
19387	APLP2	AKT1	[inhibition]
19388	FBXW11	TNF	[activation]
19389	PPP1CA	ATM	[activation]
19390	MFAP2	ELN	[activation]
19391	TBK1	TLR9	[activation]
19392	CRK	ARHGAP17	[activation]
19393	BAK1	BCL2L11	[activation]
19394	TWF2	FOXJ1	[activation]
19395	COL4A3BP	PARP2	[activation]
19396	ERBB3	EGR1	[activation]
19397	GIT2	YWHAG	[activation]
19398	HSPA8	TRAF6	[activation]
19399	HSP90AB1	RPS6KL1	[activation]
19400	GABARAPL2	AGTRAP	[activation]
19401	MAVS	CYLD	[inhibition]
19402	DTX3	UBE2H	[activation]
19403	UBE2N	TRAF2	[activation]
19404	GRB2	DUSP15	[activation]
19405	CD59	GRB2	[activation]
19406	PUM1	CCDC8	[inhibition]
19407	RAD51B	RAD51D	[activation]
19408	RPS19BP1	TP53	[activation]
19409	EGFR	USP9X	[activation]
19410	BTK	PLCG1	[activation]
19411	STK4	MCM3	[activation]
19412	CD79A	PTPRC	[activation]
19413	NXF1	EIF2A	[activation]
19414	EZR	TNFRSF10B	[activation]
19415	EZH2	RASA1	[activation]
19416	WAS	CDC42	[activation]
19417	MOS	BMPR2	[activation]
19418	PIK3CA	DNAJB6	[activation]
19419	ATF2	UTF1	[activation]
19420	PTN	TBRG4	[activation]
19421	PDGFRB	CBL	[activation]
19422	PROP1	NCDN	[activation]
19423	APC	STRN	[inhibition]
19424	IFIT2	DNAJA2	[inhibition]
19425	TFDP2	LIN37	[inhibition]
19426	TOPBP1	BMPR2	[activation]
19427	SLC9A1	MAPK1	[activation]
19428	ESR1	HSP90AB1	[activation]
19429	PPARA	SIRT1	[activation]
19430	PIAS2	AKT1	[activation]
19431	CHN1	HEMK1	[activation]
19432	SNX17	LRP8	[activation]
19433	PRKDC	YWHAZ	[activation]
19434	HSPA5	PRKAA1	[inhibition]
19435	HES1	FANCG	[activation]
19436	MPP1	RPS6KA1	[activation]
19437	YWHAH	EIF4E2	[inhibition]
19438	GOLGA2	CDK2	[activation]
19439	CRK	KRT12	[activation]
19440	RAB5A	PTGIR	[activation]
19441	KSR2	FKBP6	[activation]
19442	SRPK1	P2RX6	[activation]
19443	GHR	STAT3	[activation]
19444	ZHX1	TERF1	[activation]
19445	KIAA1551	EXOC1	[activation]
19446	KIAA0196	WASH1	[activation]
19447	ZNF106	NXF1	[activation]
19448	SNW1	DNM1L	[activation]
19449	TOLLIP	RAC1	[activation]
19450	STK24	PDCD10	[activation]
19451	BLZF1	FHL2	[inhibition]
19452	ARAF	IRF7	[activation]
19453	ADRA2A	YWHAZ	[activation]
19454	TRAF2	SRSF1	[activation]
19455	TNS1	AR	[activation]
19456	LIN52	RBL2	[activation;inhibition]

19457	DAB1	MYO6	[activation]
19458	ITPR1	VDAC1	[inhibition]
19459	PLEC	GRB2	[activation]
19460	SKIL	RNF165	[activation]
19461	SFN	ARHGAP11A	[activation]
19462	IL2RG	IL7R	[inhibition]
19463	MAPK7	HSP90AB1	[activation]
19464	HSPB1	PTBP3	[activation]
19465	RABEPK	PIKFYVE	[activation]
19466	VEGFA	VEGFB	[activation]
19467	BARD1	SRSF4	[activation]
19468	VAPB	STX1B	[activation]
19469	AURKA	HSPA2	[activation]
19470	RPL35A	ICAM1	[activation]
19471	SRPK2	NSG1	[activation]
19472	PAK3	PXN	[activation]
19473	CBL	VAV2	[activation]
19474	TRAF6	TRMT112	[activation]
19475	ITGA2	FHL2	[activation]
19476	IQGAP3	LGR4	[inhibition]
19477	STAT6	EP300	[activation]
19478	GABARAPL2	ANXA2	[activation]
19479	MMP2	HSP90AA1	[activation]
19480	ARNTL	KAT2B	[activation]
19481	ADI1	SRPK1	[activation]
19482	SMARCB1	SMARCA4	[activation]
19483	TGM1	RARRES3	[activation]
19484	NOD1	MAGED1	[activation]
19485	ZMIZ2	TP53	[activation]
19486	PRX	FYN	[activation]
19487	KAT2A	ESRRA	[activation]
19488	SMAD4	RAB25	[activation]
19489	PRKCZ	FEZ2	[activation]
19490	HNRNPA2B1	SHC1	[activation]
19491	GNAT3	SNTA1	[activation]
19492	LRRK2	MBP	[activation]
19493	DIABLO	BIRC8	[activation]
19494	SYT4	STX4	[activation]
19495	DLAT	ESR2	[activation]
19496	WNK2	ABL1	[activation]
19497	STK32C	HSP90AB1	[activation]
19498	GPR158	PITX2	[activation]
19499	BCL2L1	BCL2L12	[activation]
19500	TFRC	RAB5B	[activation]
19501	PDGFRB	EIF2AK2	[activation]
19502	SIRT1	FHL2	[activation]
19503	PYY	NPY2R	[activation]
19504	ERBB2	SUPT6H	[activation]
19505	PIK3R1	VAV3	[activation]
19506	KMT2B	ANXA1	[activation]
19507	RANBP2	TAF1	[activation]
19508	VAPA	APP	[activation]
19509	HSPA1A	HSPA8	[inhibition]
19510	GORASP1	CASP7	[activation]
19511	ZAP70	HSPA4	[activation]
19512	HDAC2	NFE2L2	[activation]
19513	SPTAN1	SHC1	[activation]
19514	CFLAR	CDC37	[inhibition]
19515	BCL10	CASP8	[activation]
19516	MYO1B	DAPK1	[activation]
19517	CHEK2	MSH2	[activation]
19518	HMGB1	ERF	[inhibition]
19519	TNFSF11	FAM213B	[activation]
19520	TGFB1	ERBB2	[activation]
19521	ACTG1	CFL2	[activation]
19522	PHKG2	BZW2	[activation]
19523	HSPA4	NOX5	[activation]
19524	PLOD2	ATG5	[activation]
19525	PBK	MBP	[activation]
19526	NOTCH1	EIF3F	[activation]
19527	PRKCB	SPTAN1	[activation]
19528	TRAF2	NMUR2	[activation]
19529	MPO	RAD21	[activation]
19530	ARAP1	TP53BP1	[activation]
19531	CACNA1A	LAMB1	[activation]
19532	STX4	CCDC8	[activation]

19533	PPP1CC	C11orf74	[inhibition]
19534	TNFRSF10B	RIPK1	[activation]
19535	TAB1	DBN1	[inhibition]
19536	GSK3B	PPP1CA	[activation;inhibition]
19537	PYCARD	CASP1	[inhibition]
19538	SHC1	S100A7	[activation]
19539	MDC1	BARD1	[activation]
19540	ACVR1B	SMAD6	[inhibition]
19541	ADORA1	GNAZ	[activation;inhibition]
19542	IL1RAP	STAT3	[activation]
19543	PTK2B	GSN	[activation]
19544	NUMB	GLI1	[inhibition]
19545	PLS3	FTSJ1	[activation]
19546	CDKN2A	MMRN1	[activation;inhibition]
19547	TBC1D17	RAB5A	[activation]
19548	LRRK1	GAB2	[activation]
19549	RPA3	RPA4	[activation]
19550	ATP7A	CLU	[activation]
19551	LRRC41	NXF1	[activation]
19552	SMARCA1	PARP1	[activation]
19553	SMAD4	KLF5	[activation]
19554	ADRB2	VAPA	[activation]
19555	TFDP1	CDC20	[inhibition]
19556	MVP	SKIL	[activation]
19557	RBL1	AOX1	[activation]
19558	XPO1	CCND1	[activation]
19559	METTL17	MDC1	[activation]
19560	RPA1	CDC5L	[activation]
19561	NCK1	RAPGEF1	[activation]
19562	RPS6KA2	PRKCE	[activation]
19563	SHARPIN	IL1A	[activation]
19564	PPP1R16A	TXNRD1	[activation]
19565	PIAS1	PPP1CA	[inhibition]
19566	DAB2	DVL2	[activation]
19567	H2AFZ	MYC	[activation]
19568	GRB2	BCL2A1	[activation]
19569	LARP1	BARD1	[activation]
19570	ABI3BP	FZD5	[activation]
19571	MEF2D	CASP7	[activation]
19572	ATF2	TXNDC5	[activation]
19573	PAFAH1B1	SUMO1	[activation]
19574	RASD2	SMAD1	[activation]
19575	PRKAA1	AES	[inhibition]
19576	UBE2I	TRAF6	[activation]
19577	MDM2	MS4A1	[activation]
19578	EGFR	ATP1A1	[activation]
19579	POLD1	LRRK2	[activation]
19580	TRAF6	ACTR3	[activation]
19581	APEX1	PAK2	[activation]
19582	NEDD8	SMC2	[activation]
19583	PCNA	RPA1	[activation]
19584	LRRK2	CCDC43	[activation]
19585	F2RL1	CBL	[activation]
19586	CNOT11	NLK	[inhibition]
19587	SPR	CAMK2G	[activation]
19588	ANXA1	DLG3	[activation]
19589	PRTN3	SERPINB1	[inhibition]
19590	GIGYF1	YWHAB	[activation]
19591	HRAS	MUTYH	[activation]
19592	C5AR1	ARRB1	[activation]
19593	CHN1	CDK5R2	[activation]
19594	SMAD3	CSNK1D	[activation]
19595	CBL	CORO1C	[activation;inhibition]
19596	CDK1	PITPNM1	[activation]
19597	MME	EDN1	[activation]
19598	PLK3	MAP2K1	[activation]
19599	RAP1A	TRAF6	[activation]
19600	LEF1	BAX	[inhibition]
19601	BIRC5	CASP9	[activation]
19602	CBLB	VAV1	[activation]
19603	ANGPTL3	ITGAV	[activation]
19604	BARD1	ERCC6L	[activation]
19605	ANXA1	APBB1	[activation]
19606	RAF1	ADCY6	[activation]
19607	NFYC	GLTSCR1	[activation]
19608	SHB	KDR	[activation]

19609	HSPB1	ARRDC5	[activation]
19610	PPARA	FABP1	[activation]
19611	TGFBR1	SOCS6	[inhibition]
19612	TTLL6	MAPT	[activation]
19613	HIST1H4A	RAPGEF2	[activation]
19614	CAV1	CD40	[activation]
19615	TRIB3	ATF5	[activation]
19616	ACSF2	AGTRAP	[activation]
19617	MPP1	RPS6KA6	[activation]
19618	MDM2	PIM2	[activation]
19619	RHNO1	HUS1	[activation]
19620	JUN	ETV4	[activation]
19621	HBZ	VCAM1	[activation]
19622	FCGR2A	LAT	[activation]
19623	TP73	CCNB1	[activation;inhibition]
19624	NAB1	NXF1	[activation]
19625	HSPB1	GDI2	[activation]
19626	MCM4	MCM8	[activation]
19627	TFRC	CAV1	[activation]
19628	HDAC3	RUNX1	[activation]
19629	TNFRSF10D	MARS	[activation]
19630	TRAF2	C5orf55	[activation]
19631	HIST1H3A	EGLN1	[activation]
19632	HSP90AA1	STAT3	[activation]
19633	MAPK14	UGT2B10	[activation]
19634	GRIP1	GRIA2	[activation]
19635	CDC5L	SMC4	[activation]
19636	YAP1	WBP2	[inhibition]
19637	EP300	ARNT	[activation]
19638	MAPK10	RDX	[activation]
19639	GPRASP2	RAF1	[activation]
19640	SORT1	CDK2	[activation]
19641	EEF2	MAPK14	[activation;inhibition]
19642	ZAP70	LAX1	[activation]
19643	PTPN14	YAP1	[activation]
19644	JUN	GNB2L1	[activation]
19645	HDAC4	RFXANK	[activation]
19646	RB1	SERPINB2	[inhibition]
19647	SPI1	SKI	[inhibition]
19648	PCDHA4	IGSF21	[activation]
19649	MAD2L1	OXSR1	[inhibition]
19650	HIST3H3	RBP5	[activation]
19651	PTPRJ	BAAT	[activation]
19652	APP	DNM2	[activation]
19653	SCYL2	ARRB2	[activation]
19654	POLE2	MAPRE1	[activation]
19655	NCOA1	TBP	[activation]
19656	UBC	MAP2K1	[activation]
19657	CARD11	PDPK1	[activation]
19658	FOXO1	IRF3	[activation]
19659	ITGB1	CSF2RB	[activation]
19660	RPA1	PRPF6	[activation]
19661	ARRB1	SREBF2	[activation]
19662	RAP2B	RALGDS	[activation]
19663	PIAS1	GATA4	[activation]
19664	TLR3	MAP2K6	[activation]
19665	AKT3	CASP3	[activation]
19666	DIRAS3	CHEK2	[activation]
19667	MST1	RASSF1	[inhibition]
19668	LRRK2	RBBP8	[activation]
19669	H2AFX	KAT2A	[activation]
19670	PRKACA	ADD2	[activation]
19671	RAN	XPO5	[activation]
19672	NXT1	PAXIP1	[activation]
19673	HMGN1	RPS6KA4	[activation]
19674	VCAM1	DNM2	[activation]
19675	CAPN7	IST1	[activation]
19676	EGFR	CD2AP	[activation]
19677	CUL1	IKBKB	[activation]
19678	PPP2CA	CSNK1A1	[inhibition]
19679	SKIL	CDC27	[activation]
19680	PUM1	ITGA4	[activation]
19681	EIF4A2	SPIN3	[activation]
19682	CRELD1	GDF9	[activation]
19683	VAV1	MAP2K1	[activation]
19684	NCK1	FCGR2B	[activation]

19685	IKBKG	GCN1L1	[activation]
19686	ARPC4	ACTR3	[activation]
19687	IGF1R	RASA1	[activation]
19688	CACNA2D2	IGHA1	[activation]
19689	CNR1	GNA15	[activation]
19690	HMOX2	SHC1	[activation]
19691	PPP2R5E	BHLHE40	[activation]
19692	CAMK1D	CAMKK1	[activation]
19693	BCL2L11	TCEB2	[activation]
19694	CDH1	CDH3	[activation]
19695	UGDH	SIRT3	[activation]
19696	CSNK1G1	IKZF1	[activation]
19697	YWHAG	CRK	[activation]
19698	FYN	CNTFR	[activation]
19699	DENND3	YAP1	[inhibition]
19700	PIK3R4	NXF1	[activation]
19701	ITIH4	GRB2	[activation]
19702	NOTCH1	PPP2CA	[activation]
19703	UBE3D	CCNB1	[inhibition]
19704	SETD8	EZH2	[activation]
19705	IKBKB	NLRX1	[activation]
19706	LRRK2	MAP2K3	[activation]
19707	ZDHHC17	SREBF2	[activation]
19708	PDX1	PASK	[activation]
19709	ZBTB3	GATA4	[activation]
19710	MAX	MAD1L1	[inhibition]
19711	APPL1	C1QTNF9	[activation]
19712	LRP1	GULP1	[activation]
19713	VWF	MAP3K3	[activation]
19714	EIF4A2	SSX2IP	[activation]
19715	PYCARD	CHUK	[inhibition]
19716	TSC2	YWHAZ	[activation;inhibition]
19717	LINC00312	ESR1	[activation]
19718	TRADD	ACTG1	[activation]
19719	CD40LG	IGHG1	[activation]
19720	PROCA1	CCNA1	[activation;inhibition]
19721	PTN	DFNA5	[activation]
19722	RPL23	TP53	[activation]
19723	TNFSF11	EEF1A1	[activation]
19724	THBS1	FGF2	[activation]
19725	FGA	IGHA1	[activation]
19726	HIST1H2BD	ITGA4	[activation]
19727	ICT1	PGAM5	[activation]
19728	PSEN1	DAG1	[activation]
19729	DGKI	RASGRP3	[activation]
19730	CCR10	CCL2	[activation]
19731	UBE2E2	RNF8	[activation]
19732	PARP1	PRKAA1	[inhibition]
19733	FAM53C	YWHAH	[activation]
19734	RBBP5	MYB	[activation]
19735	APP	FKBPL	[activation]
19736	KPNA1	EGFR	[activation]
19737	SUV39H1	SMAD5	[activation]
19738	PPP2R1B	STAT3	[activation]
19739	SETD1A	MYB	[activation]
19740	PDHB	MDM2	[activation]
19741	RAB10	DOK2	[activation]
19742	CDC7	CDK4	[activation]
19743	MAPK1	VDR	[activation]
19744	STIM1	ATP2B4	[activation]
19745	SSX2IP	CXCL6	[activation]
19746	EPHB6	EPHB4	[activation]
19747	BCL6	LIMS3	[activation]
19748	MLH1	PTP4A2	[activation]
19749	DRD2	GNAZ	[activation]
19750	E2F3	ENOX2	[activation]
19751	HNF1B	CREBBP	[activation]
19752	SEC23B	SRPK2	[activation]
19753	NFKB1	GABARAPL2	[activation]
19754	AMBP	PIK3R3	[activation]
19755	FKBP1A	GLMN	[activation]
19756	CDKN1B	SRC	[activation;inhibition]
19757	GLRX3	GRN	[activation]
19758	PHC2	CRKL	[activation]
19759	FZR1	DNAJA1	[inhibition]
19760	DCTN1	MAP2K3	[activation]

19761	CP	GAST	[activation]
19762	LRRK2	PTCD3	[activation]
19763	KCNQ1	PPP1CA	[activation]
19764	ILK	ZNF568	[activation]
19765	PCSK4	IGF2	[activation]
19766	STAP2	PTK2	[activation]
19767	HMGN1	ERCC6	[activation]
19768	NLRP1	CASP9	[inhibition]
19769	POLM	UBE2H	[activation]
19770	EIF2B5	GSK3A	[activation]
19771	FCGR2B	CRK	[activation]
19772	CAMKK2	SMC3	[activation]
19773	CD2AP	GRB2	[activation]
19774	MYC	BUB1	[activation]
19775	TRAF6	NLRX1	[activation]
19776	GNAI2	CXCR1	[activation]
19777	MAP4K5	CRKL	[activation]
19778	MAPKAPK3	RAB2A	[activation]
19779	CALM1	IKBKB	[activation]
19780	JUND	FOSB	[activation]
19781	APP	C14orf93	[activation]
19782	LRRK2	RAB11FIP2	[activation]
19783	PPARGC1A	ESR1	[activation]
19784	PLCG1	TEC	[activation]
19785	MDM2	TFRC	[activation]
19786	LAMTOR1	MDM2	[activation]
19787	EIF4G2	EIF4A2	[activation]
19788	IGF1R	ITGB1	[activation]
19789	PRPSAP1	NUAK1	[activation]
19790	TUBAL3	SHC1	[activation]
19791	DNAJA3	NFKB1	[inhibition]
19792	BARD1	SLFN11	[activation]
19793	SIRT2	RIPK1	[activation]
19794	PPARG	CDK5	[activation]
19795	GULP1	SMC2	[activation]
19796	CD59	SRC	[activation]
19797	RTF1	PTPN11	[activation]
19798	SMAD3	ARHGEF7	[activation]
19799	PRIM2	RPA1	[activation]
19800	NOTCH1	ROCK1	[activation]
19801	A2M	CFTR	[inhibition]
19802	PPP2R5E	DOCK9	[activation]
19803	OPCML	PRNP	[activation]
19804	GADD45B	GADD45G	[activation]
19805	FLI1	PRKCD	[activation]
19806	C11orf63	SRPK1	[activation]
19807	TCL1A	ATM	[activation]
19808	FYN	HSP90AA1	[activation]
19809	NXF1	CEP250	[activation]
19810	RMND5B	SMAD4	[activation;inhibition]
19811	SRPK2	APEX1	[activation]
19812	EGFR	SH2D3A	[activation]
19813	CASP8	ILK	[activation]
19814	SFN	ANPEP	[activation]
19815	NPHS1	SRC	[activation]
19816	ANXA8	ACD	[activation]
19817	PAXIP1	NOL7	[activation]
19818	TFPI	ELANE	[inhibition]
19819	CYTH3	GRASP	[activation]
19820	MIPEP	MYC	[activation]
19821	PTPRC	PAEP	[activation]
19822	HN1L	RASA4	[activation]
19823	AKT1	HSPA9	[activation]
19824	PTK2B	GP6	[activation]
19825	GABARAPL2	AAK1	[activation]
19826	CTNNBIP1	JUP	[inhibition]
19827	SHC1	EPS8	[activation]
19828	ATG5	ECHS1	[activation]
19829	NOS2	RAC2	[activation]
19830	TRAF6	MAT2A	[activation]
19831	PRKCZ	MAP2K1	[activation]
19832	RARA	RXRA	[inhibition]
19833	SYK	PXN	[activation]
19834	RAP1B	RPTOR	[activation]
19835	NRAS	RASA1	[activation]
19836	MCM3AP	RAC1	[activation]

19837	TLR2	ATG16L1	[activation]
19838	NGFR	TSC1	[activation;inhibition]
19839	SHC1	DNAJA2	[inhibition]
19840	MAPKAPK3	DLC1	[activation]
19841	ACTB	ANGPTL4	[activation]
19842	CTTN	ACTR3	[activation]
19843	POMC	APP	[activation]
19844	YAP1	SLC9A3R1	[activation]
19845	FAM9B	RAD51B	[activation]
19846	EP300	KPNA2	[activation]
19847	NRD1	MYC	[activation]
19848	ITGAV	ITGB8	[activation]
19849	RAB8A	RPA2	[activation]
19850	DARC	CCL5	[activation]
19851	IFNE	TUBA1A	[activation]
19852	TP53INP1	PRKCD	[activation]
19853	MAPKBP1	MAPK9	[activation]
19854	CHRNA7	FYN	[activation]
19855	KALRN	EPHB2	[activation]
19856	PIK3R1	EP300	[activation]
19857	NOP2	GABARAP	[activation]
19858	FBXO5	ANAPC4	[activation]
19859	GADD45A	LMO4	[activation;inhibition]
19860	CDKN1A	POLD2	[activation]
19861	AQP3	PLD2	[activation]
19862	LAMC1	LAMB1	[activation]
19863	VAMP8	ITGA4	[activation]
19864	PLK1	CEP55	[activation;inhibition]
19865	DFFA	PRKAA2	[activation]
19866	IQCE	CALM1	[activation]
19867	HDAC9	MEF2A	[activation]
19868	POTEKP	ARRB2	[inhibition]
19869	ATF2	CEBPB	[activation]
19870	RPL26	MDM2	[activation]
19871	MBL2	CD93	[activation]
19872	GRB2	CKS2	[activation]
19873	CETP	PRKACG	[activation]
19874	ASF1A	CHEK2	[activation]
19875	ZFC3H1	BUB1B	[inhibition]
19876	LZTR1	BMPR1B	[activation;inhibition]
19877	FBXW11	CTNNB1	[inhibition]
19878	APP	DECR2	[activation]
19879	ITGB3	DAB1	[activation]
19880	MIEF1	DNM1L	[activation]
19881	SUMO3	MEIS1	[activation]
19882	NFKBIA	DNAJA3	[inhibition]
19883	ALPK3	MYC	[activation]
19884	GNB1	ADRB2	[activation]
19885	IKBKE	KIF11	[activation]
19886	VIM	ATF2	[activation]
19887	CDK5RAP3	IGKC	[activation]
19888	TRIM15	FAM151B	[activation]
19889	SFN	SAV1	[activation]
19890	PDPN	CLEC1B	[activation]
19891	PTK2	DVL1	[activation]
19892	SETD8	TWIST1	[activation]
19893	GRB2	ARHGAP9	[activation]
19894	PPP2CA	PPME1	[activation]
19895	ITSN2	PIK3AP1	[activation]
19896	RXFP4	KNG1	[activation]
19897	ANAPC2	CDC20	[inhibition]
19898	CBL	TEK	[activation]
19899	PLK1	ANKRD44	[activation]
19900	CRKL	NOTCH2	[activation]
19901	RHEBL1	TGFBR1	[activation]
19902	SPTAN1	CASP7	[activation]
19903	TP53	SMAD3	[activation]
19904	HSP90AA1	NUDC	[activation]
19905	APBB1	PRNP	[activation]
19906	RAB5A	ELAVL1	[activation]
19907	S100A14	STK11	[activation]
19908	ITSN2	WASF2	[activation]
19909	NIN	PRKACA	[activation]
19910	SH3BP5L	YWHAG	[activation]
19911	SERPINE2	COL4A4	[inhibition]
19912	MYC	RIMS2	[activation]

19913	TGFBR2	APP	[activation]
19914	GRB2	ACTC1	[activation]
19915	HDAC1	IDH2	[activation]
19916	TLR3	SYK	[activation]
19917	CDKN2C	DRAP1	[inhibition]
19918	FYN	CDKL5	[activation]
19919	VIPR2	APP	[activation]
19920	CHRM1	ARRB2	[activation]
19921	PFKFB2	PRKACA	[activation]
19922	CTNNB1	TFAP2A	[activation]
19923	RPTOR	MARK4	[activation;inhibition]
19924	RECQL5	POLR2I	[activation]
19925	PRKCD	RANBP9	[activation]
19926	TCEANC	DAB1	[activation]
19927	FASLG	PACSIN2	[activation]
19928	NMT1	TP53	[activation]
19929	EIF4E2	MIPOL1	[inhibition]
19930	PIK3CD	CD37	[activation]
19931	ANXA2	ARF6	[activation]
19932	PIK3CG	BCR	[activation]
19933	CCR8	TPST2	[activation]
19934	PRKAA1	ULK2	[inhibition]
19935	SENP3	EP300	[activation]
19936	TENM3	SMAD4	[activation]
19937	RCN2	RIPK1	[activation]
19938	ATF2	TAGLN2	[activation]
19939	HEATR2	PHLDA3	[activation]
19940	C20orf195	TRAF2	[activation]
19941	OPRD1	OPRK1	[activation]
19942	EIF2B1	RD3	[activation]
19943	HIF1A	SUMO1	[activation]
19944	EGFR	GAB1	[activation]
19945	MAPK14	OBSL1	[activation]
19946	ICAM1	EIF3E	[activation]
19947	SP1	CSNK2A1	[activation]
19948	SUFU	HDX	[activation]
19949	CTNNB1	NR5A1	[activation]
19950	NFKBIA	HNRNPA1	[activation]
19951	LRRC7	CDH2	[activation]
19952	AKT1	ESR2	[activation]
19953	INSR	GRB10	[activation]
19954	STAT1	RNF11	[activation]
19955	BMPR1A	SFRP4	[activation]
19956	NGEF	FAM118B	[activation]
19957	CACNB4	TBL3	[activation]
19958	KSR1	PPP2R5E	[activation]
19959	TRAF6	EEF1A2	[activation]
19960	GNA12	CDH2	[activation]
19961	RELA	PLK1	[activation]
19962	CDK3	CCNE2	[activation]
19963	TP53	MSX1	[activation]
19964	VAMP4	CCDC155	[activation]
19965	BARD1	LUC7L2	[activation]
19966	ANKRD44	PPP6C	[inhibition]
19967	APOH	AKT1	[activation]
19968	SRC	MAP2	[activation]
19969	EGFR	RAB7A	[activation]
19970	CHN2	ERBB3	[activation]
19971	RUNX1	MYC	[activation]
19972	NXF1	RPS6	[activation]
19973	EIF4G1	CCDC8	[activation]
19974	FOXO4	VDR	[activation]
19975	STK4	PARP1	[activation]
19976	PRDX2	PDGFRB	[activation]
19977	NCL	PRKCZ	[activation]
19978	PPP2R3C	IKBKB	[activation]
19979	MED1	RARA	[activation]
19980	APC	SEC23A	[inhibition]
19981	HSPA8	PPP1CC	[inhibition]
19982	PPHLN1	BCL2L1	[activation]
19983	E2F1	CREBBP	[activation]
19984	WNK1	TNRC6A	[activation]
19985	UBAP2L	NLK	[inhibition]
19986	PRKAA1	SRPK2	[activation]
19987	SMARCB1	PPP1R15A	[activation]
19988	IGFBP6	IGF2	[activation]

19989 PSEN1 BACE1 [activation]
19990 RPA3 RAB2A [activation]
19991 TSSK6 APP [activation]
19992 CCL28 DDI1 [activation]
19993 E2F5 SMAD3 [inhibition]
19994 RHOB RHPN2 [activation]
19995 C7orf43 AMOTL2 [activation]
19996 CD81 CD53 [activation]
19997 SPAG9 MAP3K3 [inhibition]
19998 TNIK ACTG1 [activation]
19999 MYC GCN1L1 [activation]
20000 TSSK6 HIST1H3A [activation]
20001 MBNL3 SRPK1 [activation]
20002 MCAM FYN [activation]
20003 DVL2 PARD6A [activation]
20004 PAK1 ZNF418 [activation]
20005 NOTCH2NL CRCT1 [activation]
20006 CSNK1E RAD54B [activation]
20007 NFKB1 HDAC4 [activation]
20008 PLG SERPINB6 [inhibition]
20009 PRR20C NCK2 [activation]
20010 PDIK1L HSP90AB1 [activation]
20011 UBA5 EIF3A [activation]
20012 RAN SPAG8 [activation]
20013 TSTD2 DCC [activation]
20014 WNK1 OXSR1 [activation]
20015 SGTA C1QA [activation]
20016 COL1A1 PDGFB [activation]
20017 NGB GNAI3 [activation;inhibition]
20018 EIF2B1 UBC [activation]
20019 RIMS1 RAB10 [activation]
20020 GATA6 HHEX [activation]
20021 CDK11B CDC37 [activation]
20022 CCNH MTA1 [activation]
20023 ETV1 EP300 [activation]
20024 SPICE1 ESRRA [activation;inhibition]
20025 RPS6KA5 APP [activation]
20026 FGFR4 WIF1 [activation]
20027 CAMK2N2 CAMK2A [activation]
20028 ACTA1 RELA [activation]
20029 NOTCH1 TRAPPC9 [activation]
20030 INADL PARD3 [activation]
20031 HLA-A HLA-B [activation]
20032 EPB41L5 RAPGEF2 [activation]
20033 LXN NUDC [activation]
20034 MDK SDC3 [activation]
20035 SNAP25 STX3 [activation]
20036 TNFRSF1A RAC1 [activation]
20037 CARHSP1 STC2 [activation]
20038 MRE11A RAD50 [activation]
20039 CTR9 PTPN11 [activation]
20040 CAV1 HSP90AA1 [activation]
20041 MAP2K4 APP [activation]
20042 TLR6 TRAP1 [activation]
20043 CACNA1A MATK [activation]
20044 RAN GADD45G [activation]
20045 BCL2L1 FKBP8 [activation]
20046 MDC1 ANAPC2 [activation]
20047 MEX3C SMC2 [activation]
20048 TSTD2 APC [inhibition]
20049 RAB1A RICTOR [activation]
20050 APC LRP8 [inhibition]
20051 KIAA1598 ESRRG [activation]
20052 MCM7 CCND1 [activation]
20053 BAG1 MAPT [activation]
20054 SST SSTR1 [activation]
20055 HLA-G KLRD1 [activation]
20056 MAP1LC3B SPAG9 [inhibition]
20057 IL7 CEBPE [activation]
20058 C18orf25 SRPK1 [activation]
20059 HSP90AB1 MDM2 [activation]
20060 HLA-E KLRD1 [activation]
20061 THRB ROBO4 [activation]
20062 HSPA8 ITGA4 [activation]
20063 SKIL CHPF [activation]
20064 KIF11 CDK1 [activation]

20065	H3F3A	ING2	[activation]
20066	TRAF6	PRKCZ	[activation]
20067	MATK	AR	[activation]
20068	GRN	OTX1	[activation]
20069	SRC	ARRB2	[activation]
20070	STOML2	ATG5	[activation]
20071	ICT1	ME2	[activation]
20072	CREBBP	FOSB	[activation]
20073	KCNA5	HSP90AB1	[activation]
20074	WHSC1	PSIP1	[activation]
20075	MLLT6	CEBPG	[activation]
20076	HCK	SH3KBP1	[activation]
20077	NFE2L2	JUN	[activation]
20078	NIPAL3	EDA	[activation]
20079	INSR	VAV3	[activation]
20080	FZR1	MAPK8	[activation]
20081	RAB11A	FTSJ1	[activation]
20082	CD47	PTK2	[activation]
20083	KIAA0196	FAM21C	[activation]
20084	KAT5	TWIST1	[activation]
20085	DTX1	EP300	[activation]
20086	HABP2	COL4A1	[activation]
20087	CAV1	PRNP	[activation]
20088	PLN	SLN	[inhibition]
20089	TP53	UTP14A	[activation]
20090	MAGEC2	ATM	[activation]
20091	CREBBP	ING1	[activation]
20092	E2F1	NXF1	[activation]
20093	PAK1	ESR1	[activation]
20094	CHUK	SRC	[activation]
20095	CD79A	FCAR	[activation]
20096	GABARAPL2	PHGDH	[activation]
20097	PRKDC	PGR	[activation]
20098	MAPK14	PML	[activation]
20099	SETD6	RELA	[activation]
20100	C1QA	C1R	[activation]
20101	PRKAA1	PPARG	[inhibition]
20102	CDKN1A	CCNO	[activation;inhibition]
20103	PKMYT1	MAPK8	[activation]
20104	CRMP1	FAS	[activation]
20105	PTCH1	SHH	[inhibition]
20106	NRP1	PGF	[activation]
20107	SRPK2	DRAP1	[activation]
20108	NF1	SUMO1	[activation]
20109	TRPM1	MYC	[activation]
20110	DNM1	LRRK2	[activation]
20111	STK3	TRAF1	[activation]
20112	NOM1	BARD1	[activation]
20113	HSP90AB1	WHSC1	[activation]
20114	RAB5C	VCAM1	[activation]
20115	PKN1	CDC25C	[activation]
20116	SRC	EPB41L3	[activation]
20117	PLK1	FABP3	[activation]
20118	HDGFRP3	LYN	[activation]
20119	NBN	RRM2B	[activation]
20120	ADAM10	TSPAN33	[activation]
20121	UBE2I	RAD54L2	[activation]
20122	MAPK8	TNFRSF10B	[activation]
20123	APOA5	LPL	[activation]
20124	PRKCA	AVPR1A	[activation]
20125	HSP90AB1	FBXO34	[activation]
20126	MAPK10	DDX5	[activation]
20127	LCK	SOCS2	[inhibition]
20128	EP300	HIST1H1D	[activation]
20129	SRPK3	DHX9	[activation]
20130	PAK2	TRIM28	[activation]
20131	HMGB2	TRAF6	[activation]
20132	CD24	FGR	[activation]
20133	SIX4	KDM6A	[activation]
20134	PPARG	NR0B1	[activation]
20135	SH2B3	ERBB2	[activation]
20136	PAK2	UBE2V1	[activation]
20137	IFNAR2	STAT3	[activation]
20138	TCEB1	KMT2A	[activation]
20139	BRK1	WASF1	[activation]
20140	VCAM1	SNAP29	[activation]

20141	FANCA	ERCC4	[activation]
20142	S1PR3	GNAQ	[activation]
20143	SUV39H1	SIRT1	[activation]
20144	ARHGAP10	PAK2	[activation]
20145	COPA	PDGFRB	[activation]
20146	ADAP1	PRKCE	[activation]
20147	STIP1	EGFR	[activation]
20148	NTN4	NEO1	[activation]
20149	PPIF	ATF2	[activation]
20150	NXF1	FAM192A	[activation]
20151	IFNAR1	JAK1	[activation]
20152	RRAS	CRK	[activation]
20153	EPSTI1	NOTCH2	[activation]
20154	NUAK2	HSP90AB1	[activation]
20155	ARRB1	MAP2K3	[activation]
20156	PIN1	FOXP2	[activation]
20157	TNFRSF25	TNFSF12	[activation]
20158	ANAPC5	CREBBP	[activation]
20159	SLC9A3R1	CTNNB1	[activation]
20160	PDE5A	PRKG1	[inhibition]
20161	GSN	BCAR1	[activation]
20162	DVL3	CSNK1E	[activation]
20163	RAD54B	TCEANC	[activation]
20164	MYC	DIMT1	[activation]
20165	GNAI1	RIC8A	[activation;inhibition]
20166	GNB1	PTH1R	[activation]
20167	SLA	FGFR1	[activation]
20168	SMAD2	ARL4D	[activation]
20169	GATAD2B	FOXP2	[activation]
20170	RPS6KB1	CDK1	[activation]
20171	MCM7	VCAM1	[activation]
20172	RRAGC	LAMTOR5	[activation]
20173	GRB2	SHBG	[activation]
20174	KCMF1	RASSF10	[inhibition]
20175	CHGB	OGG1	[activation]
20176	TJP1	F11R	[activation]
20177	MAPK1	GORASP2	[activation]
20178	GRB2	BPGM	[activation]
20179	MLLT4	RALGDS	[activation]
20180	GNG2	RICTOR	[activation]
20181	NCK1	EHMT2	[activation]
20182	TBXA2R	AAMP	[inhibition]
20183	ITGB1	CD151	[activation]
20184	MDM2	FOXO3	[activation;inhibition]
20185	COL1A1	MAG	[activation]
20186	ABHD5	PLIN1	[activation]
20187	TGFBR3	INHA	[activation]
20188	GRN	ARFGAP1	[activation]
20189	KCTD9	STX11	[activation]
20190	GRAP	LCK	[activation]
20191	CDX4	PRKAA1	[inhibition]
20192	PTBP3	GRIA4	[activation]
20193	WHSC1	TIAM1	[activation]
20194	FRAT2	NRAS	[activation]
20195	SERPINB13	ELANE	[inhibition]
20196	MAPK8	NCOA3	[activation]
20197	BCCIP	RAD51	[activation]
20198	CASP3	AR	[activation]
20199	CRKL	WIPF1	[activation]
20200	TXK	MET	[activation]
20201	LRRK2	TRIB2	[activation]
20202	ESR1	DSG1	[activation]
20203	PNO1	EGFR	[activation]
20204	RIMBP3	PRKAA1	[inhibition]
20205	MPP3	EZR	[activation]
20206	PRKCD	GRM5	[activation]
20207	SCARF2	GRB2	[activation]
20208	PIK3R1	DLGAP2	[activation]
20209	FAS	SRC	[activation]
20210	PRAM1	LCP2	[activation]
20211	GRB2	RPL12	[activation]
20212	YWHAZ	MCM3	[activation]
20213	MAPK1	HDAC4	[activation]
20214	SH2B3	LCK	[activation]
20215	BUB1B	SKIV2L2	[inhibition]
20216	ARMC8	CTNNA1	[activation]

20217	ANK1	SLC4A3	[activation]
20218	EGFR	XPOT	[activation]
20219	RAF1	RBL2	[activation;inhibition]
20220	JUN	PML	[activation]
20221	HIST2H2BE	AKT1	[activation]
20222	JUNB	BDNF	[activation]
20223	FZD7	SDCBP	[activation]
20224	PLG	RELA	[activation]
20225	EPHA7	EPHA3	[activation]
20226	AKT1	ITPR1	[inhibition]
20227	ETS1	NR3C1	[activation]
20228	EXOC5	ENO2	[activation]
20229	ARRB2	C11orf84	[inhibition]
20230	CSNK1D	PTBP3	[activation]
20231	EGFR	LONP1	[activation]
20232	DRD2	EPB41	[activation]
20233	NUP155	ZFYVE9	[activation]
20234	TP53	UCHL1	[activation]
20235	CDK4	ACTR3B	[activation]
20236	RUNX1	SMARCB1	[activation]
20237	IL2RG	PTPRJ	[activation]
20238	UBTF	SMURF1	[activation]
20239	IRF7	MAVS	[activation]
20240	YWHAH	GAB2	[activation]
20241	RET	FRS2	[activation]
20242	GADD45B	CDK1	[activation]
20243	VAV2	GAPVD1	[activation]
20244	TAB2	CALM1	[activation]
20245	SKIL	LRP1	[activation]
20246	HSD17B4	GABARAPL1	[activation]
20247	PTPN12	PTK2B	[activation]
20248	MCM8	CDC6	[activation]
20249	LRRK1	SHC1	[activation]
20250	LPL	VLDLR	[activation]
20251	CBL	ACTG2	[activation]
20252	BRK1	ABI2	[activation]
20253	TNFRSF1B	CAV1	[activation]
20254	ABL1	JUN	[activation]
20255	SMURF1	DNAJC7	[inhibition]
20256	YES1	HSP90AA1	[activation]
20257	GADD45A	MTOR	[activation;inhibition]
20258	SPP1	PDLIM7	[activation]
20259	HSF1	PRKDC	[activation]
20260	PTPRA	BCAR1	[activation]
20261	CAV1	ITCH	[activation]
20262	GRB2	DAB2	[activation]
20263	POLD1	SRC	[activation]
20264	AGPS	MDM2	[activation]
20265	RPA1	UNG	[activation]
20266	WIPI1	ESR1	[activation]
20267	IDE	IAPP	[activation]
20268	EDNRB	EDN3	[activation]
20269	YWHAG	LRCH3	[activation]
20270	NPAS2	ARNTL	[activation]
20271	HSP90AB1	SGK3	[activation]
20272	SKP2	FOXO1	[activation]
20273	CEBPB	MYC	[activation]
20274	PTEN	CDH1	[activation]
20275	TTN	TCAP	[inhibition]
20276	BTG1	SKP2	[activation]
20277	LCK	PRKCA	[activation]
20278	USP9X	RPTOR	[activation]
20279	RBPJ	SND1	[inhibition]
20280	MAPK14	ADRB2	[activation]
20281	MAP4K1	GRB2	[activation]
20282	YWHAZ	ANXA2	[activation]
20283	EEA1	ZFYVE16	[activation]
20284	ANXA5	APP	[activation]
20285	FKBPL	NOD1	[activation]
20286	WAS	ENAH	[activation]
20287	CNKSR2	RALA	[activation]
20288	SH3KBP1	ANAPC7	[activation]
20289	DDX51	MCM7	[activation]
20290	GSK3B	EIF4EBP1	[activation;inhibition]
20291	ELK1	PIAS2	[activation]
20292	HSPA5	CDH1	[activation]

20293	PRKACG	CDK17	[activation]
20294	ARPC5	MAPKAPK2	[activation]
20295	CASP8	EIF2AK2	[inhibition]
20296	WDTC1	NUDC	[activation]
20297	RANBP9	SOS1	[activation]
20298	DDI1	DVL2	[activation]
20299	NGFR	CD80	[activation]
20300	ADCY1	ADCY2	[activation]
20301	PLD3	EGFR	[activation]
20302	SBF2	FOS	[activation]
20303	TTC21B	ESR2	[activation]
20304	ARPC2	HLA-B	[activation]
20305	ESRRA	PRKCD	[activation;inhibition]
20306	HBE1	VCAM1	[activation]
20307	CYTH1	GNAQ	[activation]
20308	PIK3CA	ATR	[activation]
20309	SNW1	EIF4G2	[activation]
20310	CD28	DUSP14	[activation]
20311	MYD88	PRDX1	[activation]
20312	CCND1	TBC1D2	[activation]
20313	BMPR1B	TRAF6	[activation]
20314	WAS	GRB2	[activation]
20315	GNB5	NUDC	[activation]
20316	LRRK2	ARPC2	[activation]
20317	CCNA2	TP73	[activation;inhibition]
20318	NOTCH1	JAG2	[activation]
20319	EFNB1	SMURF1	[activation]
20320	EIF2S1	SUMO3	[activation]
20321	SNAP25	PRKACA	[activation]
20322	KCNAB2	KCNA5	[activation]
20323	SFN	PI4KB	[activation]
20324	VCAM1	RPS16	[activation]
20325	EP300	DYRK1A	[activation]
20326	FYB	CBLB	[activation]
20327	CALM1	GRK4	[activation]
20328	HEXA	H2AFX	[activation]
20329	PDGFRB	SOCS1	[activation]
20330	PKM	ATF2	[activation]
20331	RPA2	SDF4	[activation]
20332	GP5	F2	[activation]
20333	CCNB1	SNW1	[activation]
20334	CAV1	PRKCDBP	[activation]
20335	GRB2	ERRFI1	[activation]
20336	HSPA1A	TRIAP1	[inhibition]
20337	RAB5A	KDR	[activation]
20338	BRCA1	RELA	[activation]
20339	CDK6	SSBP2	[inhibition]
20340	SMG6	TERT	[activation]
20341	USP6	CDC42	[activation]
20342	YWHAG	BCR	[activation]
20343	WDR18	ANXA7	[activation]
20344	DDR1	MAPK6	[activation]
20345	BAAT	GOLGA8DP	[activation]
20346	PPM1D	DIRAS3	[activation]
20347	MDM2	NUCKS1	[activation]
20348	SOD1	RNF19A	[inhibition]
20349	GRB2	EFS	[activation]
20350	MAPRE1	EIF1B	[activation]
20351	BMPR1B	RHOD	[activation]
20352	OPRD1	GNA15	[activation]
20353	DOK1	NCK2	[activation]
20354	RPL7L1	GABARAP	[activation]
20355	PLCG1	RYR1	[activation]
20356	TTC9C	VCAM1	[activation]
20357	RELA	NSD1	[activation]
20358	BMP10	BMPR2	[activation]
20359	RAB11B	TBC1D14	[activation]
20360	PPM1A	PRKCB	[activation]
20361	RAF1	BCL2L1	[activation]
20362	FKBP3	MDM2	[activation]
20363	FOXD3	POU5F1	[activation]
20364	CACNA1A	LRP1	[activation]
20365	CDC42	MAP3K10	[activation;inhibition]
20366	RHEBL1	AKT1	[activation]
20367	ARAF	RAF1	[activation]
20368	FXYD1	PPP1CA	[activation]

DOK2	CFLAR	[inhibition]
H1F0	RAD51B	[activation]
JAK2	ABL1	[activation]
IRS1	BCL2	[inhibition]
TEC	WAS	[activation]
PPP3CC	APP	[activation]
APOC3	VKORC1	[inhibition]
SNX8	GRB2	[activation]
GRB2	DBN1	[activation]
HSPA1L	TAB1	[inhibition]
PAXIP1	LYAR	[activation]
ACAP1	ITGB1	[activation]
NOXA1	RHOU	[activation]
TXNDC5	MDM2	[activation]
EIF5B	ETS1	[activation]
MOS	FYN	[activation]
RELA	USP31	[activation]
C22orf46	APP	[activation]
PKNOX1	MEIS1	[activation]
CFP	MTOR	[activation;inhibition]
NOV	ITGAV	[activation]
FAM194A	HSPB1	[activation]
TWIST1	ELSPBP1	[activation]
IFIT1B	IFIT1	[inhibition]
CTNNB1	CTNND2	[inhibition]
GABARAPL2	NEK9	[activation]
EEF1D	ATG7	[activation]
TRIM24	ESR2	[activation]
PRKCA	RALBP1	[activation]
ARPC3	SUMO3	[activation]
GFAP	STAT1	[activation]
TFDP1	LIN54	[inhibition]
EP300	GRIP1	[activation]
CA9	CTNNA1	[activation]
CDC25B	BTRC	[activation]
HBE1	HBZ	[inhibition]
HNRNPA0	ARRB2	[activation]
IRAK2	SMURF1	[inhibition]
TFF1	RAD51	[activation]
HSP90AB1	NEK9	[activation]
SMAD2	C9orf156	[activation]
VAPA	ATF2	[activation]
EGFR	ZNF259	[activation]
VPS45	IGF1R	[activation]
SH2D2A	GAB1	[activation]
TRAF6	SYK	[activation]
ASAP2	PLCG1	[activation]
MYCN	ZBTB17	[activation]
PES1	ARRB2	[activation]
CARM1	KAT2B	[activation]
DENND5A	PRKCZ	[activation]
IRS1	PIK3CB	[activation]
CAMK4	CABIN1	[inhibition]
MMP2	HAPLN1	[inhibition]
OPRM1	ZYX	[activation]
F2RL1	GPRASP1	[activation]
SH3BP2	ERBB2	[activation]
RANBP2	CDC5L	[activation]
UBE2B	TP53	[activation]
HSP90AA1	EGFR	[activation]
ARF6	RGS10	[activation;inhibition]
TERF1	TINAG	[activation]
FES	IRS1	[activation]
YWHAH	RIMS1	[activation]
CCNC	GATA1	[activation]
IRS4	ERBB2	[activation]
CECR5	ICT1	[activation]
MDM2	HIPK2	[activation]
PRKCA	CREM	[activation]
MLH1	PPP2R2B	[activation]
DLL1	EPN1	[activation]
BCL2L1	BCAP31	[activation]
BCAR1	FES	[activation]
SRPK2	ARPC1B	[activation]
ARAF	BAD	[inhibition]
RIT2	HRAS	[activation]

20445	ARMC7	ABI3	[activation]
20446	ANXA5	SUMO2	[activation]
20447	CUL7	RAN	[activation]
20448	PRKCZ	CASP8	[activation]
20449	PLG	CPB2	[activation]
20450	PPIF	MDM2	[activation]
20451	KCNE4	KCNQ1	[activation]
20452	WWTR1	AMOT	[activation]
20453	GNA15	FKBPL	[activation]
20454	SYT16	AGTRAP	[activation]
20455	ABL2	HRAS	[activation]
20456	DDX39B	HNRNPLL	[activation]
20457	ILK	CNNM3	[activation]
20458	FOS	NCOA1	[activation]
20459	KLHL38	NUDC	[activation]
20460	CTNNB1	NUMB	[activation]
20461	RASGRF1	CEBPA	[activation]
20462	ABL1	BMP2K	[activation]
20463	SHC1	STAT5B	[activation]
20464	FGFR1	FRS3	[activation]
20465	GMPS	HLA-B	[activation]
20466	MYC	RFC2	[activation]
20467	CA9	ACACA	[activation;inhibition]
20468	FUBP3	GRB2	[activation]
20469	TNFRSF1A	RIPK2	[activation]
20470	PAK2	PACSIN3	[activation]
20471	C3	FN1	[activation;inhibition]
20472	NEB	MAPK14	[activation]
20473	CDK11A	PAK1	[activation]
20474	HMGB1	AR	[activation]
20475	CDK4	IFI27	[inhibition]
20476	GJB2	CD14	[activation]
20477	VCAM1	SMCHD1	[activation]
20478	SHC1	PIK3C2B	[activation]
20479	RARA	CREBBP	[activation]
20480	ANXA2	RPA1	[activation]
20481	YES1	PECAM1	[activation]
20482	METTL17	ICT1	[activation]
20483	DDA1	UBE2E3	[activation]
20484	PRKACA	ITGA2B	[activation]
20485	MMP12	ELN	[activation]
20486	TICAM2	IRAK2	[activation]
20487	SHB	AR	[activation]
20488	HSPA6	CDC5L	[activation]
20489	MAPKAPK3	IGHM	[activation]
20490	SCFD1	GOSR1	[activation]
20491	DYRK1A	FAM53C	[activation]
20492	ESR2	MYO1C	[activation]
20493	YAP1	YWHAZ	[activation]
20494	RPA2	HELB	[activation]
20495	PHACTR2	GRB2	[activation]
20496	ARHGAP17	FMNL1	[activation]
20497	PTK2B	MAPT	[activation]
20498	RPL7L1	EGR2	[activation]
20499	DNM2	SHC1	[activation]
20500	PALB2	CCL5	[activation]
20501	BTK	HSP90AB1	[activation]
20502	RAD51	IRS1	[activation]
20503	SMC2	GSTK1	[activation]
20504	MRPS22	SMURF1	[inhibition]
20505	KPNB1	MLH1	[activation]
20506	PLCG1	ITK	[activation]
20507	TTC23L	CTAGE5	[activation;inhibition]
20508	HSP90AB1	CDK6	[activation]
20509	NOD1	CASP2	[activation]
20510	EGFR	SYK	[activation]
20511	TGFB1	APP	[activation]
20512	MCC	TFRC	[activation]
20513	AHCYL1	CDKN2C	[inhibition]
20514	PRPF40A	ARFGEF2	[activation]
20515	UNG	HSPB1	[activation]
20516	WNK1	YWHAZ	[activation]
20517	E2F3	CTNNA1	[activation]
20518	IL1B	IL1RAP	[activation]
20519	EIF2AK2	MAP3K5	[inhibition]
20520	SMARCC1	CCNE1	[activation]

20521	PSMB1	INSIG2	[activation]
20522	PPP2CA	TP53	[activation]
20523	MTIF3	PAXIP1	[activation]
20524	CAPZA1	ATG5	[activation]
20525	STXBP1	PRKCA	[activation]
20526	HSPA8	IVNS1ABP	[inhibition]
20527	MAPK1	MAP2K6	[activation]
20528	SNAI1	HIST1H3A	[activation;inhibition]
20529	BTG1	RARA	[activation]
20530	ARRB2	PPM1B	[inhibition]
20531	SPP1	CASP3	[activation]
20532	ANXA7	SRI	[activation]
20533	LINC00312	SRPK1	[activation]
20534	TRAF6	PFN2	[activation]
20535	PIK3R1	NR5A1	[activation]
20536	FYN	LAT	[activation]
20537	RAD21	AZU1	[inhibition]
20538	PIK3R1	IL6ST	[activation]
20539	PAG1	EGFR	[activation]
20540	TRAF6	RHOC	[activation]
20541	CCNA2	DYRK1A	[activation]
20542	PLCG1	RET	[activation]
20543	NEDD8	EIF2B4	[activation]
20544	EHMT1	MDM2	[activation]
20545	GOLGA2	MDM2	[activation]
20546	GAK	AP1M2	[inhibition]
20547	BCAR1	CASP3	[activation]
20548	DLGAP5	APP	[activation]
20549	TRAF6	OTUD7A	[activation]
20550	BTK	TLR6	[activation]
20551	CCNA1	MCM3	[activation]
20552	SRPK3	SNRPC	[activation]
20553	MMP1	CD44	[activation]
20554	ACACA	FBXO6	[activation;inhibition]
20555	ICT1	MRPL54	[activation]
20556	APOL5	PIK3R1	[activation]
20557	PIK3CG	PTPN11	[activation]
20558	TRAF6	MRTO4	[activation]
20559	RPA3	BLM	[activation]
20560	TUBB4A	LRRK2	[activation]
20561	HSPA6	GABARAP	[activation]
20562	TFRC	ADRB2	[activation]
20563	SUPT6H	TP53	[activation]
20564	SUMO3	AURKB	[activation]
20565	EPHA7	GNB1	[activation]
20566	ADAM10	GRB2	[activation]
20567	SKP2	SMAD4	[inhibition]
20568	TUBB4A	IKBKB	[activation]
20569	GTPBP3	IGHA1	[activation]
20570	ANKRD11	TRAF2	[activation]
20571	ZAK	MIDN	[activation]
20572	ILK	DHCR7	[activation]
20573	ADCK3	ICT1	[activation]
20574	TRAF6	MCL1	[activation]
20575	BARD1	TOP1	[activation]
20576	EFS	PTK2B	[activation]
20577	COL4A5	SERPINE2	[inhibition]
20578	POLE2	AGFG1	[activation]
20579	ITGB1	YWHAB	[activation]
20580	KDM1B	EHMT2	[activation]
20581	RARA	RXRG	[activation]
20582	TERT	XPO1	[activation]
20583	GNL3	EIF2AK2	[activation]
20584	MAPK1	CUEDC2	[activation]
20585	RUNX1	ELF4	[activation]
20586	EYA1	H2AFX	[activation]
20587	PIK3R4	KRIT1	[activation]
20588	FXYD7	APOE	[activation]
20589	ARF6	PLS1	[activation]
20590	VIPR2	VIP	[activation]
20591	EPHA2	PIK3R2	[activation]
20592	EIF4G2	EIF5	[activation]
20593	ARPC4	ITGA4	[activation]
20594	PKIA	PRKACB	[inhibition]
20595	CRY1	CBLB	[inhibition]
20596	RAD21	CTTN	[activation]

20597	RXRA	NPAS2	[inhibition]
20598	TOP2A	RAC1	[activation]
20599	GRB2	CRYBB3	[activation]
20600	ACTN1	PTPN1	[activation]
20601	RPS6KB1	HSP90AA1	[activation]
20602	GRAP	SHC1	[activation]
20603	BMP6	SOSTDC1	[activation]
20604	IL25	ESR1	[activation]
20605	HGF	SRPX2	[activation]
20606	VCAM1	MRPL43	[activation]
20607	RELA	TCAP	[inhibition]
20608	HIST2H2AC	H2AFX	[activation]
20609	VAMP7	AP3M1	[activation]
20610	TERF1	POT1	[activation]
20611	SPERT	ACTN1	[activation]
20612	THBS1	SOX2	[activation]
20613	STK3	RASSF4	[activation]
20614	PRNP	FAM64A	[activation]
20615	GADD45G	EZH2	[activation]
20616	RFC1	ATM	[activation]
20617	TRMT2B	ERCC1	[activation]
20618	FBLL1	DDX56	[activation]
20619	DDX56	DHX37	[activation]
20620	CREBBP	EGR1	[activation]
20621	ACTB	MDM2	[activation]
20622	GRK5	EIF2S3	[activation]
20623	HUWE1	CRKL	[activation]
20624	CTNNB1	PTPRK	[inhibition]
20625	NUDC	NXF1	[activation]
20626	SMARCA4	H2AFX	[activation]
20627	CSNK2A1	EIF5	[activation]
20628	TP53	MAP1B	[activation]
20629	PTK2	DCC	[activation]
20630	STIP1	LRRK2	[activation]
20631	LIPE	PLIN1	[activation]
20632	TGFBR1	RASD2	[activation]
20633	CD4	SELL	[activation]
20634	ACVR1	RHOD	[activation]
20635	E2F1	SETD7	[activation]
20636	MAPKAPK3	SUMO3	[activation]
20637	PRKCQ	MSN	[activation]
20638	RASL10B	AKT1	[activation]
20639	ECSIT	APOE	[activation]
20640	YWHAZ	LRMP	[activation]
20641	ENO2	ANXA11	[activation]
20642	TMEFF1	PTEN	[activation]
20643	STK4	CNKSR1	[activation]
20644	PTMS	CREBBP	[activation]
20645	F2R	GPRASP1	[activation]
20646	SMAD3	ETS1	[activation]
20647	HNF1A	GATA5	[activation]
20648	DPP4	GCG	[activation]
20649	ITGA5	PPAP2B	[activation]
20650	PPP2CA	ID1	[inhibition]
20651	MAP3K7	CALM1	[activation]
20652	LNX2	PKDREJ	[activation]
20653	PTPRJ	LCK	[activation]
20654	RHOQ	TRIP6	[activation]
20655	LRRK2	C5orf45	[activation]
20656	GNAI2	IL8	[activation]
20657	RARA	NPAS2	[activation]
20658	CR1	C4B	[inhibition]
20659	ARHGEF11	ARRB2	[activation]
20660	ESR2	RFX6	[activation]
20661	TP53	YTHDF1	[activation]
20662	MYD88	RAC1	[activation]
20663	YWHAH	MAST3	[activation]
20664	RYR1	MYOM2	[activation]
20665	SMARCA5	RPA3	[activation]
20666	CUL1	PPP2CA	[inhibition]
20667	GNAI2	OPRM1	[activation]
20668	PIK3R4	YWHAE	[activation]
20669	YAP1	RAPGEF6	[activation]
20670	XIAP	HSPA4	[inhibition]
20671	CDK5RAP2	MAGEH1	[activation]
20672	LAT	SH3BP2	[activation]

20673	ZAK	MPP1	[activation]
20674	FSCN1	NGFR	[activation]
20675	CDX4	LMO1	[activation]
20676	IGF1	IGSF1	[activation]
20677	MDM2	RNF126	[activation]
20678	CLUAP1	MYOG	[activation]
20679	MIF	FTSJ1	[activation]
20680	TERF1	CAPNS1	[activation]
20681	HDAC4	IFRD1	[activation]
20682	ACVR1	TTC27	[activation;inhibition]
20683	TSC1	HBA1	[inhibition]
20684	MYOD1	TWIST1	[activation]
20685	SUMO1	ANXA5	[activation]
20686	MMP8	TFPI	[inhibition]
20687	CDK6	ATF6B	[inhibition]
20688	PALB2	BRCA1	[activation]
20689	TP53	FZR1	[activation]
20690	SLX1A	PLK1	[activation]
20691	KLK3	EPOR	[activation]
20692	DLL1	NOV	[activation]
20693	PRKDC	H3F3A	[activation]
20694	PIK3C3	CD5	[activation]
20695	DVL1	TFDP1	[inhibition]
20696	MDM2	ARHGDIA	[activation]
20697	SHC1	FAM118B	[activation]
20698	MYC	CDK5RAP2	[activation]
20699	NRBP1	RIBC2	[activation]
20700	OSR1	TGFBR2	[activation]
20701	TRAF2	RIPK2	[activation]
20702	GRB2	PRR22	[activation]
20703	ARRB1	MAPK10	[activation]
20704	RAD9A	NR3C1	[activation]
20705	CLOCK	SIRT1	[activation;inhibition]
20706	CLSPN	RPA1	[activation]
20707	A2M	HMOX2	[inhibition]
20708	TUBB4A	MAP3K7	[activation]
20709	ANAPC7	CREBBP	[activation]
20710	TRIM54	PIK3R3	[activation]
20711	CRK	STAT5B	[activation]
20712	TAB3	SMAD7	[activation]
20713	SETD8	SUMO1	[activation]
20714	A2M	ELAVL3	[inhibition]
20715	BTRC	HSF1	[activation]
20716	RAC1	ITGA4	[activation]
20717	LIFR	PLCG1	[activation]
20718	SETDB1	PPA1	[activation]
20719	HSPA5	INSIG2	[activation]
20720	RAC1	LTBP4	[activation]
20721	CDK5RAP2	REPS2	[activation]
20722	CDKN1A	VDR	[activation]
20723	PIK3CB	UBC	[activation]
20724	GAB1	TEC	[activation]
20725	ADRBK1	PRKCD	[activation]
20726	RAP1A	BMX	[activation]
20727	GNAS	CALM1	[activation]
20728	CDK6	ABI1	[activation]
20729	DNAJB5	ASB4	[inhibition]
20730	ARHGEF11	PLXNB3	[activation]
20731	WDR1	CARD11	[activation]
20732	USP3	GNA13	[activation]
20733	CSN2	CDC42	[activation]
20734	PLCG1	KIT	[activation]
20735	MCM10	CCND1	[activation]
20736	SLC25A4	PPID	[inhibition]
20737	SRI	RYR2	[activation]
20738	IL27	EBI3	[activation]
20739	HSPA1A	HSBP1	[inhibition]
20740	MKLN1	MYC	[activation]
20741	HSP90AA1	MAP3K7	[activation]
20742	RIF1	BOC	[activation]
20743	CD8A	LCK	[activation]
20744	ATG5	IFIH1	[activation]
20745	CTGF	FGFR2	[activation]
20746	PHLDA3	ARMC6	[activation]
20747	SMAD2	LRPAP1	[inhibition]
20748	RASA1	AFAP1L2	[activation]

20749	XPO1	NRIP1	[activation]
20750	SRSF6	APP	[activation]
20751	AURKC	SRPK1	[activation]
20752	HSPA2	UNC45B	[activation]
20753	CDCA4	KAT2B	[activation]
20754	IKBKG	DAPK1	[activation]
20755	EIF2B1	ADRA2C	[activation]
20756	FTSJ1	XIRP2	[activation]
20757	CASK	TIAM1	[activation]
20758	CUL1	TRAF6	[activation]
20759	PIK3CA	DGKZ	[activation]
20760	TULP3	RIOK3	[activation]
20761	CASP3	TFAP2A	[activation]
20762	PPP1R12A	MAP3K3	[activation]
20763	PPM1G	MTNR1B	[activation]
20764	ARRB1	HSPA7	[activation]
20765	CHD4	EP300	[activation]
20766	PPP2R5A	CHEK2	[activation]
20767	BMP3	PRMT6	[activation]
20768	BRAF	RAF1	[activation]
20769	GARS	HLA-B	[activation]
20770	SNAP91	NECAP1	[activation]
20771	SPRY4	RAF1	[activation]
20772	L1CAM	EZR	[activation]
20773	TP53	KPNB1	[activation]
20774	CCL2	PCNA	[activation]
20775	FGFR3	SLC25A6	[activation]
20776	UBE2G2	VAMP8	[activation]
20777	SYK	HDAC4	[activation]
20778	ELMOD2	RHOG	[activation]
20779	PLCB1	MAPK1	[activation]
20780	HRAS	TTC1	[activation]
20781	ACTB	CEP250	[activation]
20782	SYN1	PRKACA	[activation]
20783	EIF4EBP1	MAPK14	[activation]
20784	CCND3	MYB	[activation]
20785	TREX2	ATF4	[activation]
20786	BRCA2	FYN	[activation]
20787	CAMK2A	RAD23A	[activation]
20788	PHC2	MAPK14	[activation]
20789	GRB2	EHMT2	[activation]
20790	TRAF2	RIBC2	[activation]
20791	ABI1	YES1	[activation]
20792	PIK3CG	PIK3CD	[activation]
20793	CDH5	CTNND1	[activation]
20794	DUSP6	MDFI	[activation]
20795	TNFRSF11B	VWF	[activation]
20796	CHUK	NR2C2	[activation]
20797	TGFBR2	KCNK18	[activation]
20798	RAB5C	SGSM3	[activation]
20799	YWHAG	TET2	[activation]
20800	CAMSAP2	FOS	[activation]
20801	FGFR1	NRP1	[activation]
20802	KPNA2	CDK11A	[activation]
20803	CALM1	DDX21	[activation]
20804	PRKDC	CTDP1	[activation]
20805	MAPK8	TFCP2	[activation]
20806	ARF6	CHRM3	[activation]
20807	NSMCE2	TXLNA	[activation]
20808	GSK3B	MGEA5	[activation]
20809	NRAS	CCDC180	[activation]
20810	WASL	ABL1	[activation]
20811	MYOD1	BHLHE41	[activation]
20812	MTOR	HSPA4	[activation]
20813	CSK	ERBB2	[activation]
20814	BMPR1B	RAB25	[activation]
20815	ACTR3	NXF1	[activation]
20816	MCM3	NOTCH1	[activation]
20817	RAB5C	SUN2	[activation]
20818	SRC	GAB2	[activation]
20819	PGM1	PAXIP1	[activation]
20820	EGFR	BZW1	[activation]
20821	LSR	MAPKAPK5	[activation]
20822	BUB1B	HSPB1	[inhibition]
20823	APP	WARS	[activation]
20824	C1orf94	CCHCR1	[activation]

20825	NR1H3	RARA	[activation]
20826	PHKB	CAMK2B	[inhibition]
20827	EED	TNF	[activation]
20828	CAPZB	BCCIP	[activation]
20829	YWHAQ	PRKCZ	[activation]
20830	KPNB1	ITGA4	[activation]
20831	FCGR1A	FCGR3A	[activation]
20832	GSK3A	CREM	[inhibition]
20833	HIST2H2BF	CD81	[activation]
20834	TDGF1	AP2S1	[activation]
20835	PSD3	ARAP1	[activation]
20836	NCF1	GNAI1	[activation]
20837	APOA5	FAS	[inhibition]
20838	QRICH1	NLK	[inhibition]
20839	RRAS2	PIK3R1	[activation]
20840	MAGEB1	SRPK2	[activation]
20841	YWHAG	NOS2	[activation]
20842	WHSC1	RRP1	[activation]
20843	CNIH4	TNFRSF14	[activation]
20844	MAGI2	TGFA	[activation]
20845	CPNE1	ACTB	[activation]
20846	CAMK2A	KRT18	[activation]
20847	IRS4	PTPN11	[activation]
20848	IL6	OSM	[activation]
20849	STAT3	GNB2	[activation]
20850	TRAF6	MCM7	[activation]
20851	PIK3R1	RAPGEF1	[activation]
20852	PALB2	SERPINB5	[activation]
20853	NLRP1	BCL2L1	[activation]
20854	IRF8	IRF2	[activation]
20855	SQSTM1	SNCA	[activation]
20856	PTGIR	GNAS	[activation]
20857	ARHGAP44	CDC42	[activation]
20858	RAP1B	FAF1	[activation]
20859	PRPF6	ESR1	[activation]
20860	E2F1	IGF1	[activation]
20861	KCNE1	ADRB2	[activation]
20862	RAB4B	EXOC5	[activation]
20863	APP	UBL3	[activation]
20864	RXRG	CAMK2B	[activation]
20865	HSPA1L	EGFR	[activation]
20866	EIF3F	CD81	[activation]
20867	TEK	GRB14	[activation]
20868	PAK1	FRS2	[activation]
20869	TRAF6	SAE1	[activation]
20870	PPP1R12A	FOS	[activation]
20871	FBXO6	GRN	[activation]
20872	HSP90AB1	ILK	[activation]
20873	AUP1	OPRK1	[activation]
20874	PTN	BCL2L1	[activation]
20875	ACAT2	ATG5	[activation]
20876	MAP2K2	CCNDBP1	[activation]
20877	IRAK1	PELI2	[activation;inhibition]
20878	ERC1	APP	[activation]
20879	PFN1	SRPK1	[activation]
20880	IL32	PRTN3	[activation]
20881	DDX5	STK4	[activation]
20882	AKT2	LRRK2	[activation]
20883	INSR	RASA1	[activation]
20884	PRKCB	RGS2	[activation]
20885	GRB2	SH2D1A	[activation]
20886	PGR	STAT5A	[activation]
20887	CACNA1A	LTBP3	[inhibition]
20888	FOS	MAFF	[activation]
20889	MMS19	EIF2S2	[activation]
20890	FGFR1	IGHG1	[activation]
20891	TBCD	PPP2CA	[inhibition]
20892	GRB2	AR	[activation]
20893	GRB2	GSTP1	[activation]
20894	BARD1	AURKB	[activation]
20895	GORASP2	CBLB	[inhibition]
20896	TRAF6	PDLIM7	[activation]
20897	HSPB1	EIF2S1	[activation]
20898	LCK	FCGR3A	[activation]
20899	HNF4A	MAPK14	[activation]
20900	ITGAE	CDH1	[activation]

20901 YWHAZ MDM2 [activation]
20902 EP300 CRTC2 [activation]
20903 MLH1 PPP2R1A [activation]
20904 GABARAPL2 GARS [activation]
20905 CD4 CD81 [activation]
20906 VAV2 EPHB2 [activation]
20907 PRKACA RANBP9 [activation]
20908 CMA1 EDN2 [activation]
20909 TNIP2 MAP3K8 [activation]
20910 HBE1 ITGA4 [activation]
20911 DAB2IP TRAF2 [activation]
20912 ITGA4 LARS [activation]
20913 FYN SPHK2 [activation]
20914 RHOB PRAF2 [activation]
20915 HNRNPA1 OBSL1 [activation]
20916 CDKN1A GADD45A [activation;inhibition]
20917 PLEKHA4 PRKAA1 [inhibition]
20918 WAS ACTR2 [activation]
20919 YWHAZ ARHGEF16 [activation]
20920 CTDSP2 POLR2A [activation]
20921 BRCA1 RAD51 [activation]
20922 ATG5 EEF1B2 [activation]
20923 CTNNB1 NFKB1 [activation]
20924 VAPA INSIG1 [activation]
20925 SH2B2 CBL [activation]
20926 SSX2IP CDC42 [activation]
20927 WHSC1 ACTB [activation]
20928 GRB2 PBXIP1 [activation]
20929 SORBS1 FRS2 [activation]
20930 TP53 RFC1 [activation]
20931 SMC2 HIST2H3A [activation]
20932 TP53 FAU [activation]
20933 CDC5L NCAPD2 [activation]
20934 SHC1 FCGR3A [activation]
20935 IQGAP1 PPARGC1A [activation]
20936 EGFR LRWD1 [activation]
20937 CASP1 PAK1 [activation]
20938 C7orf25 NGEF [activation]
20939 ACVR1B XIAP [activation]
20940 TUBA4A MAPT [activation]
20941 CCDC97 SRPK2 [activation]
20942 CHD7 SALL3 [activation]
20943 HIST1H3A LRRK2 [activation]
20944 HIST3H3 JADE2 [activation]
20945 CCDC36 FAM124A [activation]
20946 TRAF6 NTRK2 [activation]
20947 PALLD SRC [activation]
20948 CHUK ARRB2 [activation]
20949 GSK3B SMYD2 [activation]
20950 KAT2B CEP250 [activation]
20951 PPP6R3 NOTCH1 [activation]
20952 ULK1 GABARAPL1 [inhibition]
20953 OSM COL4A3 [activation]
20954 PPP3CB PPP1CC [inhibition]
20955 ADRB2 PRKCD [activation]
20956 MAP3K2 HSP90AA1 [activation]
20957 MYC CHD4 [activation]
20958 BCL2L1 SIVA1 [activation]
20959 RANGAP1 GNAI3 [activation]
20960 SH3BP2 VAV2 [activation]
20961 TGM2 ATF4 [activation]
20962 GATA1 ZNF521 [activation]
20963 CDK4 CDC37 [inhibition]
20964 FASLG TNFRSF10B [activation]
20965 BRD4 AR [activation]
20966 ARHGAP9 APP [activation]
20967 EP300 CTTN [activation]
20968 EGFR PTPRJ [activation]
20969 HMGXB3 CDKN1A [activation;inhibition]
20970 ACTG1 MAP1A [activation]
20971 BHLHE40 MYOD1 [activation]
20972 HSP90AB1 PGK1 [activation]
20973 INSIG1 HMGCR [activation]
20974 WDR1 CFL1 [activation;inhibition]
20975 LILRB1 HLA-A [activation]
20976 EIF4EBP1 PRKCA [activation]

20977	SUMO4	PPA1	[activation]
20978	CREBBP	MEIS1	[activation]
20979	FERMT1	SRPK1	[activation]
20980	MMP2	A2M	[inhibition]
20981	FZR1	RB1	[inhibition]
20982	STK3	CSNK1E	[activation]
20983	STAT1	KDM3A	[activation]
20984	ATG7	SIRT1	[activation]
20985	RPS28	CD81	[activation]
20986	TOP1MT	TP53	[activation]
20987	GABARAPL2	YWHAZ	[activation]
20988	TEC	PIP5K1A	[activation]
20989	CDK5	CDKN1A	[activation]
20990	ATG3	CFLAR	[inhibition]
20991	FAM105B	DVL2	[activation]
20992	MAML1	SMARCA4	[activation]
20993	RASA1	BCL10	[activation]
20994	EFNB2	GRM5	[activation]
20995	ARRB2	ARHGEF6	[activation]
20996	BUB3	TNIK	[activation]
20997	TGFBR3	INHBA	[activation]
20998	RHOD	ADCK5	[activation]
20999	CMA1	FYN	[activation]
21000	BUB1B	HDAC4	[inhibition]
21001	PXN	ADAM10	[activation]
21002	MCM3	MYC	[activation]
21003	MAST1	YWHAH	[activation]
21004	PXN	ITGA4	[activation]
21005	HFE	TFR2	[activation]
21006	MAPK13	APP	[activation]
21007	STAU1	NXT1	[activation]
21008	TIPIN	RPA1	[activation]
21009	CDC37	SMYD2	[activation]
21010	COL6A1	MAG	[activation]
21011	IL6ST	PTPN6	[activation;inhibition]
21012	RECQL5	RAD51	[activation]
21013	HIF1A	PER1	[activation]
21014	TGFBRAP1	TGFB2	[activation]
21015	MYC	IDH3G	[activation]
21016	PPP2R1A	HDAC1	[inhibition]
21017	MTIF3	MAPK1	[activation]
21018	SMARCA4	FANCA	[activation]
21019	GGCX	F2	[activation]
21020	EWSR1	PTK2B	[activation]
21021	ESYT2	CAMKK2	[activation]
21022	HSP90AB1	CAMK2D	[activation]
21023	SERPINC1	SDC2	[inhibition]
21024	EIF4G1	EIF3A	[activation]
21025	PLK1	HSF1	[activation]
21026	PPP1R12A	MCM7	[activation]
21027	ITGB1	FBXO6	[activation]
21028	ANXA5	COL2A1	[activation]
21029	PPP5C	HTR2A	[activation]
21030	TP53	ANKRD2	[activation]
21031	HSPE1	ITGA4	[activation]
21032	PPP1CA	CCND3	[inhibition]
21033	TP53	HSPA8	[activation]
21034	CALML3	RAD21	[activation]
21035	PIP4K2A	GSTK1	[activation]
21036	PIK3R1	CHRNA7	[activation]
21037	MEOX2	SPP1	[activation]
21038	CRK	PNMA2	[activation]
21039	NCK1	SOS2	[activation]
21040	PIK3R2	APP	[activation]
21041	NCOA3	NPAS2	[activation]
21042	EYA2	GNAI2	[activation]
21043	SH2D1A	MET	[activation]
21044	EGFR	MOB4	[activation]
21045	PITPNM2	PTK2B	[activation]
21046	EFTUD2	LRRK2	[activation]
21047	ERBB2	RANBP2	[activation]
21048	SMURF1	IVNS1ABP	[inhibition]
21049	WASH1	CTTN	[activation]
21050	RAD21	PPM1D	[activation]
21051	FHL2	HCK	[activation]
21052	PRKCZ	PRKCQ	[activation]

21053	NCK1	PIK3CD	[activation]
21054	MME	GAST	[activation]
21055	LCK	RAF1	[activation]
21056	MYO1B	CUL1	[activation]
21057	LLPH	SRPK3	[activation]
21058	MLLT4	VCAM1	[activation]
21059	SOS1	SPTAN1	[activation]
21060	CRMP1	HMGB1	[activation]
21061	RIMS1	FYN	[activation]
21062	ATRX	MRE11A	[activation]
21063	FAM192A	TRAF2	[activation]
21064	LAPTM4B	PIK3R1	[activation]
21065	PTK2	CCNA1	[activation]
21066	CXCL2	EPS8	[activation]
21067	PIK3CG	SEC14L2	[activation]
21068	ARF6	SMARCC1	[activation]
21069	ANKS4B	APP	[activation]
21070	RAC1	ARL2BP	[activation]
21071	ISCA1	ICT1	[activation]
21072	HNRNPA1	HSPA2	[activation]
21073	SKA2	HMOX2	[activation]
21074	RELA	PRKDC	[activation]
21075	MCM3	ARF6	[activation]
21076	SRY	SMAD3	[activation]
21077	ATR	PA2G4	[activation]
21078	CASP9	DCC	[activation]
21079	NR4A1	SMAD3	[inhibition]
21080	DNM2	CTTN	[activation]
21081	COMMD10	RELA	[activation]
21082	DVL2	RAC1	[activation]
21083	NOTCH1	KIF2A	[activation]
21084	CREBBP	MYB	[activation]
21085	UBE2W	MARCH3	[activation]
21086	HDAC1	AR	[activation]
21087	MAD2L1	CDC20	[inhibition]
21088	SGTA	GAL	[activation]
21089	KCNK15	YWHAH	[activation]
21090	ITSN1	ZAK	[activation]
21091	LYN	PLCG2	[activation]
21092	ATG5	EXOC4	[activation]
21093	TRAF3	NGFRAP1	[activation]
21094	MLC1	CAV1	[activation]
21095	PRKACB	MAPRE1	[activation]
21096	SRSF12	CDK6	[activation]
21097	SEC63	PTP4A3	[inhibition]
21098	STAP2	STAT3	[activation]
21099	RBL1	PPP2CA	[inhibition]
21100	MYO5B	CCDC8	[activation]
21101	GOLGA2	RBL1	[inhibition]
21102	PLCB2	RAC1	[activation]
21103	OGT	NXF1	[activation]
21104	TRIM2	UBE2E2	[activation]
21105	HSP90AB1	ROCK1	[activation]
21106	FLT4	EPN1	[activation]
21107	KIAA1377	TNFRSF14	[activation]
21108	INSR	CALM1	[activation]
21109	KDM6A	SMARCA4	[activation]
21110	APC	CTNNB1	[inhibition]
21111	SREBF1	INSIG2	[activation]
21112	PDGFRA	ITGAV	[activation]
21113	STAP2	CBL	[activation]
21114	L1CAM	NRP1	[activation]
21115	LRRK2	TCF25	[activation]
21116	RPS6KC1	SPHK1	[activation]
21117	CDH1	MAPKAPK3	[activation]
21118	AKT2	CCL14	[activation]
21119	PFN2	ROCK1	[activation]
21120	CASP12	UBE2K	[inhibition]
21121	SRF	NFYA	[activation]
21122	HSF1	CREBBP	[activation]
21123	HNRNPL	SSSCA1	[activation]
21124	SRSF5	CALM1	[activation]
21125	TCEAL3	ETS1	[activation]
21126	PCSK9	ANXA2	[activation]
21127	CDC25A	RAF1	[activation]
21128	APP	DAB2	[activation]

21129	MAPK1	EP300	[activation]
21130	SMARCD1	GATA1	[activation]
21131	FAS	PRAM1	[inhibition]
21132	CAMKK2	CAMK1	[activation]
21133	SELPLG	SNX20	[activation]
21134	SNAP29	STX6	[activation]
21135	LRRK2	ENKUR	[activation]
21136	HDHD2	PIP5K1C	[activation]
21137	ARHGAP12	SRPK1	[activation]
21138	MAST2	PTEN	[activation]
21139	BCL2L11	MEX3D	[activation]
21140	PECAM1	PIK3R1	[activation]
21141	MAGEB10	MYC	[activation]
21142	NCOA1	NR5A2	[activation]
21143	SKP1	CDK4	[inhibition]
21144	PTPRC	TNK2	[activation]
21145	PPP1CA	OBSL1	[activation;inhibition]
21146	CDKN2C	CCDC90B	[inhibition]
21147	MAPT	PIN1	[activation]
21148	DNM1L	BCL2L1	[activation]
21149	EGFR	WASF3	[activation]
21150	FER	CALM1	[activation;inhibition]
21151	GABARAPL2	PRPF6	[activation]
21152	SRC	RASA1	[activation]
21153	ICAM4	IGHG1	[activation]
21154	ETS1	HMGB1	[activation]
21155	FRS3	BMPR2	[activation]
21156	TNFRSF1B	GCN1L1	[activation]
21157	TBP	MYC	[activation]
21158	GSK3A	PRKCH	[activation;inhibition]
21159	CALM1	SMARCB1	[activation]
21160	DAB2	SEC23B	[activation]
21161	TP53	SLC2A12	[activation]
21162	HCK	PLCG1	[activation]
21163	CSF2	HNRNPA1	[activation]
21164	GRN	EGFR	[activation]
21165	SETD7	NR2F2	[activation]
21166	SOX8	HHEX	[activation]
21167	GAB2	MET	[activation]
21168	ANXA1	PABPN1	[activation]
21169	BLK	CD79B	[activation]
21170	RIPK2	SMURF1	[inhibition]
21171	RPA3	CSNK1A1	[activation]
21172	HRAS	CTNNA1	[activation]
21173	RAD54B	RAD51	[activation]
21174	CTNNBL1	SRPK2	[activation]
21175	EIF2S2	CAND1	[activation]
21176	MYBL2	CDKN1C	[inhibition]
21177	SDC1	SDC3	[activation]
21178	MPP3	MDH1	[activation]
21179	JUN	USP9X	[activation]
21180	HCK	PLAUR	[activation]
21181	CAMK2A	ITGA2B	[activation]
21182	NXF1	PSKH1	[activation]
21183	NCOA1	DDX5	[activation]
21184	UTP14A	PRKCZ	[activation]
21185	PRKCZ	F11R	[activation]
21186	TP53	DMTF1	[activation]
21187	TP73	CCND1	[activation]
21188	TP53	HPCA	[activation]
21189	XPO1	SMARCB1	[activation]
21190	KMT2E	ACTB	[activation]
21191	ACTB	PIK3R2	[activation]
21192	ARFGAP1	CYTH2	[activation]
21193	MDK	LRP1	[activation]
21194	IQCB1	DPYSL2	[activation]
21195	EPOR	CSF2RB	[activation]
21196	RDH13	GADD45G	[activation]
21197	MAST2	FBXW11	[inhibition]
21198	NFE2L2	CHD6	[activation]
21199	HSPA1L	METTL21A	[activation]
21200	TSHR	GNA11	[activation]
21201	CDC42BPG	CDC42	[activation]
21202	SRPK1	MKNK2	[activation]
21203	OSBPL9	VAPB	[activation]
21204	IGHM	VPREB1	[activation]

21205	INO80E	GEM	[activation]
21206	STK11	SERPINB5	[activation]
21207	LEF1	KPNA1	[activation]
21208	IL4R	PTPN6	[activation;inhibition]
21209	GLMN	HSPB1	[activation]
21210	VCAM1	FLOT1	[activation]
21211	NCK2	ABI2	[activation]
21212	CAPN1	GAS2	[activation]
21213	VCAM1	SMARCA4	[activation]
21214	PPP2CB	NOTCH1	[activation]
21215	IL2	CD53	[activation]
21216	FGR	HSP90AB1	[activation]
21217	MYC	SPEG	[activation]
21218	AURKB	HSP90AA1	[activation]
21219	CCL7	DARC	[activation]
21220	TNFRSF1A	DAPK1	[activation]
21221	CDKN1A	HSPA8	[inhibition]
21222	USP9X	GPSM1	[inhibition]
21223	IRS4	YWHAH	[activation]
21224	CD79A	LYN	[activation]
21225	TRAF6	CS	[activation]
21226	WASF2	PSTPIP1	[activation]
21227	CTSG	SERPINA1	[inhibition]
21228	BCAR1	BCAR3	[activation]
21229	ZNHIT6	ETS1	[activation]
21230	REPS1	NUMB	[activation]
21231	MST1R	RELA	[activation]
21232	CDC25A	NFYA	[activation]
21233	YWHAZ	PRLR	[inhibition]
21234	NOD1	NUDCD3	[activation]
21235	ATP13A2	ICAM2	[activation]
21236	TP53	BAG2	[activation]
21237	DNAJC8	SRPK1	[activation]
21238	MYC	SHE	[activation]
21239	TP53	RPS24	[activation]
21240	PPP1R9A	ACTG1	[activation]
21241	BMP7	NOTCH2NL	[activation]
21242	RPL36	ICAM1	[activation]
21243	WASL	CRK	[activation]
21244	TAF11	RXRA	[inhibition]
21245	LSP1	SNAI1	[activation]
21246	RB1	STAT3	[activation]
21247	NFKB1	PPME1	[activation]
21248	BRD7	IRF2	[activation]
21249	NOX4	TLR4	[activation]
21250	TRAF3	RIPK1	[activation]
21251	PPBP	MAPK6	[activation]
21252	CSNK1E	DVL2	[activation]
21253	CD81	NCL	[activation]
21254	PAXIP1	LUC7L2	[activation]
21255	ERN1	SRPK2	[activation]
21256	LPL	LMF1	[activation]
21257	ARFRP1	CYTH1	[activation]
21258	PLCG1	FGFR2	[activation]
21259	RUNX1	DNMT1	[activation]
21260	MAPK11	OBSL1	[activation]
21261	E2F1	NCOA3	[activation]
21262	PREP	OXT	[activation]
21263	NR3C1	CD3D	[activation]
21264	TRAF6	HNRNPL	[activation]
21265	DNAJA2	SMAD3	[inhibition]
21266	MAPK3	GMFB	[activation]
21267	PRKCI	ADAP1	[activation]
21268	PRKAR1A	PRKACB	[activation]
21269	CSNK1D	SMAD4	[activation]
21270	HTR1B	HTR1D	[activation]
21271	VIM	CASP9	[inhibition]
21272	SNTA1	RYR2	[activation]
21273	CRMP1	ZAK	[activation]
21274	NR2C2	AR	[activation]
21275	HSPD1	AURKB	[activation]
21276	SRPK1	YTHDC1	[activation]
21277	SGK494	PSTPIP1	[inhibition]
21278	RASL11B	APP	[activation]
21279	BRINP1	RAC1	[activation]
21280	SHC1	ATAD3B	[activation]

21281	DRD3	SLC9A3	[activation]
21282	SRC	TNFRSF11A	[activation]
21283	NR4A2	BAZ1B	[activation]
21284	APC	LAMA3	[activation]
21285	PAXIP1	RNMTL1	[activation]
21286	AKT1	MAPKAPK2	[activation]
21287	SUV39H2	CCDC37	[activation]
21288	NXF1	BUB1	[activation]
21289	GNAI1	CCR5	[activation]
21290	MYF5	TCF3	[activation]
21291	APC	CGNL1	[inhibition]
21292	PIK3R1	DOK1	[activation]
21293	MRPS6	SREBF2	[activation]
21294	RAN	XPO6	[activation]
21295	ITSN2	FASLG	[activation]
21296	MCM3	PTP4A3	[activation]
21297	PAK1	CSNK2A2	[activation]
21298	ATM	RAD51	[activation]
21299	PLCG1	AIRE	[activation]
21300	SETDB1	FAM118B	[activation]
21301	EGFR	ANKS1A	[activation]
21302	RELA	MYC	[activation]
21303	SRPK2	PCBP1	[activation]
21304	ARPC4	ELAVL1	[activation]
21305	NOXA1	MAPK11	[activation]
21306	WDR5B	KMT2D	[activation]
21307	EPOR	VAV1	[activation]
21308	CHMP2A	TGFB3	[activation]
21309	JUNB	USP24	[activation]
21310	FERMT3	APP	[activation]
21311	C1QA	PPP1CC	[activation]
21312	TWF1	BMPR2	[activation]
21313	CRK	PCDHA11	[activation]
21314	YAE1D1	MCF2L	[activation]
21315	TRAF3	SUMO1	[activation]
21316	ALK	CDK13	[activation]
21317	PIK3R2	CSF1R	[activation]
21318	KIF11	ULK2	[activation]
21319	DLX4	GRB2	[activation]
21320	DHX36	RPA1	[activation]
21321	SMURF1	SMURF2	[inhibition]
21322	CACNA1A	CALM1	[activation]
21323	GRB2	GSN	[activation]
21324	MYL12A	YWHAZ	[activation]
21325	IFNA4	ALDOA	[activation]
21326	CCND2	NPDC1	[inhibition]
21327	MAPKAPK2	ESR1	[activation]
21328	SERBP1	TP53	[activation]
21329	MAPK1	MAP3K3	[activation]
21330	ERBB2	VAPB	[activation]
21331	ARHGEF7	YWHAG	[activation]
21332	GRM1	ADORA1	[activation]
21333	HIST1H4A	DNAJC9	[activation;inhibition]
21334	ASS1	ARAF	[activation]
21335	PTPN6	CASP8	[activation;inhibition]
21336	HSP90AA1	PTK6	[activation]
21337	CDC5L	TLN1	[activation]
21338	GABARAPL1	SRPK2	[activation]
21339	EPHA10	CAMK2D	[activation]
21340	FHL2	CAPN1	[activation]
21341	IL4R	IRS2	[activation]
21342	TP53	HECW1	[activation]
21343	EP300	IFNAR2	[activation]
21344	TP53	PRRC2C	[activation]
21345	MAD1L1	UBTF	[activation]
21346	GABARAP	IPO5	[activation]
21347	CDC23	NOTCH1	[activation]
21348	CAV1	PPP1CA	[activation]
21349	NLRX1	TRAF3	[activation]
21350	FZR1	RAD17	[activation]
21351	OGT	ASH2L	[activation]
21352	TXN2	KRT40	[activation;inhibition]
21353	ICAM1	RPL13A	[activation]
21354	HIST1H3A	CRTC2	[activation]
21355	MYC	FERMT3	[activation]
21356	BMP15	BMPR1B	[activation]

21357	BDNF	ESR1	[activation]
21358	RANBP1	LRRK2	[activation]
21359	SHC1	MYO1C	[activation]
21360	NME5	RELA	[activation]
21361	CD81	EIF4A2	[activation]
21362	TP53	POLA1	[activation]
21363	SENP1	APP	[activation]
21364	TSC1	RASSF7	[activation;inhibition]
21365	MAP4K1	TMEM101	[activation]
21366	ACVR1	ENG	[inhibition]
21367	PACRG	DNAJA1	[inhibition]
21368	NXF1	NFYA	[activation]
21369	SP1	YY1	[activation]
21370	SUMO3	PPP2CA	[inhibition]
21371	THPO	NUMBL	[inhibition]
21372	SYK	RPS6KA2	[activation]
21373	DHX40	G3BP1	[activation]
21374	PDS5A	WHSC1	[activation]
21375	MAP4K1	ABL1	[activation]
21376	MAP2K7	CFLAR	[activation]
21377	MYB	TAL1	[activation]
21378	ACVR1B	SMAD2	[activation]
21379	CDH1	TNS3	[activation]
21380	ILF3	CSF2	[activation]
21381	BTK	PIK3AP1	[activation]
21382	PTP4A3	DNAJC11	[inhibition]
21383	LAT	CBL	[activation]
21384	PRKCB	PEBP1	[inhibition]
21385	ST6GALNAC6	MYC	[activation]
21386	PLK1	SIMC1	[activation;inhibition]
21387	FOSL1	NME7	[activation]
21388	ARRB1	ACTB	[activation]
21389	UBE2E2	SRPK1	[activation]
21390	MFN2	LRRK2	[activation]
21391	APP	GABRA4	[activation]
21392	TGFB1I1	PXN	[activation]
21393	CEBPA	SMAD4	[activation;inhibition]
21394	KIR2DS1	HLA-C	[activation]
21395	HSF1	HSF4	[activation]
21396	PPP1R13B	EP300	[activation]
21397	ACTA2	CFL1	[activation;inhibition]
21398	CDKN1B	CASP8	[inhibition]
21399	HSPG2	GRN	[activation]
21400	ATF4	NFE2L1	[activation]
21401	IRF3	SOCS1	[inhibition]
21402	TP53BP2	GRB2	[activation]
21403	PLK1	KIF23	[activation]
21404	HIST1H2BC	RAD51B	[activation]
21405	SRPK1	IFIT5	[activation]
21406	CCNA2	CDC27	[activation]
21407	PRKAB2	PRKAA1	[inhibition]
21408	EGFR	C9orf156	[activation]
21409	SMAD4	TFE3	[activation]
21410	OSTF1	ACTB	[activation]
21411	KAT2B	SATB1	[activation]
21412	EGFR	ARHGAP1	[activation]
21413	MAPK11	BCL2	[activation]
21414	MPDZ	CAMK2A	[activation]
21415	LAMTOR4	MTOR	[activation]
21416	MDM2	PBX1	[activation]
21417	HSPA5	SRC	[activation]
21418	TRIM24	BRCA1	[activation]
21419	RASA1	HSPD1	[activation]
21420	FYN	CENPV	[activation]
21421	PRMT5	GRB2	[activation]
21422	MTOR	PRR5	[activation]
21423	CASP3	IL16	[activation]
21424	ATP1B1	PAXIP1	[activation]
21425	HDAC1	RB1	[inhibition]
21426	TRPC4AP	IKBKB	[activation]
21427	UGT1A10	UGT1A8	[activation]
21428	CCHCR1	ABI2	[activation]
21429	CARD16	RIPK2	[inhibition]
21430	TLE3	NLK	[inhibition]
21431	MARCH8	TFRC	[activation]
21432	CBL	EGFR	[activation]

21433	TRAF2	CCHCR1	[activation]
21434	CYBB	RAC1	[activation]
21435	EP300	CFH	[activation]
21436	PIH1D3	CDKL3	[activation]
21437	SPERT	ARMC7	[inhibition]
21438	RB1	ANAPC2	[activation]
21439	FYN	CD36	[activation]
21440	HSPB1	GUCA1A	[inhibition]
21441	USP20	VAPB	[activation]
21442	RXRB	ATP5I	[inhibition]
21443	EPPK1	STK4	[activation]
21444	PRKCB	EP300	[activation]
21445	RFTN1	BTK	[activation]
21446	MYC	DNAJB6	[inhibition]
21447	POLR2A	YAP1	[activation]
21448	CD2BP2	LRRK2	[activation]
21449	FANCA	IKBKB	[activation]
21450	FOXA1	RARA	[activation]
21451	EP300	E2F5	[activation]
21452	CYCS	HSPA8	[inhibition]
21453	GEM	RUNDC3A	[activation]
21454	DEPDC1B	MDC1	[activation]
21455	CIB1	CDK4	[inhibition]
21456	IRF5	CREBBP	[activation]
21457	MYH3	ICAM1	[activation]
21458	PIAS2	CCHCR1	[activation]
21459	FYN	NFASC	[activation]
21460	CEBPB	ATF4	[activation]
21461	MAD1L1	CDC27	[activation]
21462	KIF23	ENO1	[activation]
21463	EGFR	APEX1	[activation]
21464	RPS6	RAD21	[activation]
21465	SKP2	ESR1	[activation]
21466	EP300	ACSM5	[activation]
21467	TGFB1I1	FKBP1A	[inhibition]
21468	GRB14	NFKB1	[activation]
21469	RXRA	RAD54L2	[inhibition]
21470	CDC42BPB	MYO18A	[activation]
21471	TPI1	MDM2	[activation]
21472	ZAP70	NFAM1	[activation]
21473	ZNF302	TP53	[activation]
21474	INSIG2	RNF139	[activation]
21475	SMAD4	ARFRP1	[activation]
21476	PDCD11	WHSC1	[activation]
21477	JUNB	BATF	[activation]
21478	ETS1	SCOC	[activation]
21479	KMT2A	BMI1	[activation]
21480	SERTAD1	EGLN3	[activation]
21481	TOP1	EGFR	[activation]
21482	PDE4DIP	PRKACB	[activation]
21483	DDR2	CDH1	[activation]
21484	ZAP70	CD4	[activation]
21485	ACTG1	TRAF6	[activation]
21486	YWHAZ	MAP3K3	[activation]
21487	ENO2	IL17B	[activation]
21488	PLXNA1	AKT1	[activation]
21489	PPP2R5E	PPP2CA	[activation]
21490	BRD4	C7orf25	[activation]
21491	ADORA2A	DRD2	[activation]
21492	IKZF3	FOXP3	[activation]
21493	GNG12	EIF4A2	[activation]
21494	TNF	TRAF2	[activation]
21495	RBL1	SUV39H1	[activation]
21496	MYC	TOP2A	[activation]
21497	MCM7	PRKCD	[activation]
21498	CTNNB1	PXN	[activation]
21499	PRKCZ	HIST3H3	[activation]
21500	APP	GNAZ	[activation]
21501	XPOT	CAMKK2	[activation]
21502	MAP1LC3B	MMP2	[activation]
21503	KTN1	RHOG	[activation]
21504	SOCS1	MAP3K5	[inhibition]
21505	CDK2	HSP90AB1	[activation]
21506	FLNC	FYN	[activation]
21507	SMAD5	RYR2	[activation]
21508	FHL2	RUNX1	[activation]

21509	SIRT1	KCNA4	[activation]
21510	ATF3	NFKB1	[activation]
21511	ERBB3	BCAR3	[activation]
21512	H2AFX	TP53BP1	[activation]
21513	FRK	HSP90AB1	[activation]
21514	FBXL17	SUFU	[activation]
21515	GRB2	IL5RA	[activation]
21516	RGS4	CHRM3	[activation;inhibition]
21517	PTPRJ	HEMGN	[activation]
21518	HIST1H2BB	CREBBP	[activation]
21519	LZTS2	CDC23	[activation]
21520	CREM	HDAC1	[inhibition]
21521	DERL1	CD3D	[activation]
21522	CCL5	BCAR3	[activation]
21523	MBP	STK39	[inhibition]
21524	CAD	TAB2	[activation;inhibition]
21525	CCNJ	CDK2	[activation]
21526	HSP90AA1	HSPB1	[activation]
21527	TGFBR1	RRH	[activation]
21528	TWIST1	TWIST2	[activation]
21529	GAS7	WAS	[activation]
21530	ATP7A	UBC	[activation]
21531	ITGB2	ICAM4	[activation]
21532	NGFR	BFAR	[activation;inhibition]
21533	ERO1L	ATF2	[activation]
21534	GRB7	LY6G6F	[activation]
21535	PID1	LRP1	[activation]
21536	OGT	TET3	[activation]
21537	MSX1	RGS7	[activation;inhibition]
21538	IRF7	INSR	[activation]
21539	GRB2	AIRE	[activation]
21540	CASP9	MAPK1	[activation;inhibition]
21541	TFF1	FGFR2	[activation]
21542	PRKACA	BRAF	[activation]
21543	BRCA2	FANCE	[activation]
21544	CRK	HEXA	[activation]
21545	PCNA	EP300	[activation]
21546	CDKN2A	NXF1	[activation]
21547	TBC1D16	RGS17	[activation;inhibition]
21548	CRK	PCDHA3	[activation]
21549	TGFA	GORASP1	[activation]
21550	TTC39C	HSPB1	[activation]
21551	SARS2	TP53	[activation]
21552	KPNA2	RELA	[activation]
21553	RASSF1	MAST2	[inhibition]
21554	YWHAH	CRTC1	[activation]
21555	TP53BP1	FASN	[activation]
21556	NDRG1	EIF2S3	[activation]
21557	MLLT4	DBN1	[activation]
21558	FGFR1	NCK1	[activation]
21559	TIRAP	DNAJC3	[inhibition]
21560	SRPK1	C11orf52	[activation]
21561	AKT2	CASP3	[activation]
21562	RNF144A	E2F1	[activation]
21563	SMURF1	CCDC88C	[inhibition]
21564	PIK3R1	EFS	[activation]
21565	DRD2	CLIC6	[activation]
21566	CDK5R1	CDK5	[activation]
21567	ITPKC	TP53	[activation]
21568	GRIA2	DRD2	[activation]
21569	LAMTOR5	RRAGB	[activation]
21570	TP53	ZIC3	[activation]
21571	MAPK4	GAB1	[activation]
21572	SRPK2	ZRANB2	[activation]
21573	MAP2K1	APC	[activation]
21574	PPP2R2A	SMURF1	[inhibition]
21575	RIPK2	TLR2	[activation]
21576	CD82	EGFR	[activation]
21577	POU1F1	CEBPD	[activation]
21578	PARP2	GNAQ	[activation]
21579	NBEA	FYN	[activation]
21580	ITGB4	S1PR3	[activation]
21581	TF	RYR1	[activation]
21582	DSG2	EGFR	[activation]
21583	PIK3R2	CORO1C	[activation]
21584	CUL7	MAPK14	[activation]

21585	TLX1	PKNOX1	[activation]
21586	MLH1	LGALS4	[activation]
21587	TRAF2	MAVS	[activation]
21588	BLM	FANCD2	[activation]
21589	IKBKB	MAP3K14	[activation]
21590	BMX	HSP90AB1	[activation]
21591	FADD	TNFSF10	[activation]
21592	GABARAP	PRKAA1	[activation]
21593	PTPRJ	MAPK1	[activation]
21594	CCDC114	TXN2	[activation;inhibition]
21595	GH1	BMP1	[activation]
21596	MYB	NCL	[activation]
21597	DDX39B	EIF4A3	[activation]
21598	SAT2	TGFBR1	[activation]
21599	PTPN1	PTK2	[activation]
21600	PTK6	KHDRBS2	[activation]
21601	DUSP6	TEX11	[activation]
21602	RPS16	LRRK2	[activation]
21603	FGF2	CD44	[activation]
21604	TP53	CLTB	[activation]
21605	MYD88	SMAD6	[activation;inhibition]
21606	TTLL12	VCAM1	[activation]
21607	SUMF2	CACNA1A	[inhibition]
21608	GMPPA	GABARAP	[activation]
21609	PTPN4	GRB2	[activation]
21610	YWHAZ	ESR1	[activation]
21611	YTHDC1	DVL3	[activation]
21612	TP53	BTK	[activation]
21613	ANXA5	IFNGR2	[activation]
21614	ETS1	MEIS1	[activation]
21615	PIM2	HSP90AB1	[activation]
21616	ESR1	MTA1	[activation]
21617	RAD21	SERPINB1	[inhibition]
21618	COL1A1	MMP2	[activation]
21619	IPO5	CAB39	[activation]
21620	PTPN1	NTRK1	[activation]
21621	SOX10	POU5F1	[activation]
21622	SMARCA4	MDM2	[activation]
21623	NOL9	MYC	[activation]
21624	CDC5L	EIF2S3	[activation]
21625	STK4	ARF4	[activation]
21626	HSPB1	NFU1	[activation]
21627	MSX2	DLX5	[activation]
21628	PLD2	LCK	[activation]
21629	LDLRAD1	GIMAP5	[activation]
21630	MRPL50	MDM2	[activation]
21631	TRAPPC11	TP53	[activation]
21632	STK11	KLK7	[activation]
21633	RXRB	KLHL11	[inhibition]
21634	MAPK10	MCL1	[activation]
21635	BCL3	HDAC3	[activation]
21636	FYN	CACNA1F	[activation]
21637	EGFR	CAP2	[activation]
21638	CD5	PTPN6	[activation;inhibition]
21639	PFKFB3	EGFR	[activation]
21640	CDC20	AURKB	[activation]
21641	PSEN1	IRAK2	[activation]
21642	STX6	GOSR2	[activation]
21643	RPS6KA3	RPS6KA1	[activation]
21644	PLCG1	CAV1	[activation]
21645	TES	ARAP1	[activation]
21646	TEX9	CCDC112	[activation]
21647	STRAP	TRAF6	[activation]
21648	BUB1B	BUB1	[inhibition]
21649	RPS14	LRRK2	[activation]
21650	MAPK14	DDX26B	[activation]
21651	RNH1	MYC	[activation]
21652	EP300	GTF2B	[activation]
21653	RHOA	GMIP	[activation]
21654	ROS1	PTPN1	[activation]
21655	AIF1	LCP1	[activation]
21656	IRAK3	ATP6V0B	[inhibition]
21657	TSC1	DAPK1	[inhibition]
21658	DVL1	ANXA7	[activation]
21659	RPS6	RICTOR	[activation]
21660	GCN1L1	TSC22D1	[activation]

21661	CHD8	RBBP5	[inhibition]
21662	ACTN1	APC	[activation]
21663	CNOT7	STAT1	[activation]
21664	ADRB2	RAN	[activation]
21665	HSPD1	PCK1	[activation]
21666	C6orf165	RNF138	[inhibition]
21667	METTL21C	HSPA1L	[activation]
21668	CSN2	CDK1	[activation]
21669	MMP25	MMP2	[activation]
21670	FST	BMPR1A	[inhibition]
21671	SKIL	ZNF106	[activation]
21672	HLA-G	LILRB1	[activation]
21673	EZR	ARF6	[activation]
21674	GAB3	SRC	[activation]
21675	REV3L	HDAC4	[activation]
21676	ENAH	RPS6KA1	[activation]
21677	SRSF10	YWHAG	[activation]
21678	SDCBP	TGFA	[activation]
21679	RXRA	NR0B2	[inhibition]
21680	FBXL12	A2M	[inhibition]
21681	IL1B	ELAVL1	[activation]
21682	MLH1	PPP3CC	[activation]
21683	ISL1	CDKN2B	[activation]
21684	HDAC1	SKIL	[activation]
21685	YWHAG	PPP1R12A	[activation]
21686	PYHIN1	ATRX	[activation]
21687	PELO	EGFR	[activation]
21688	CDKN1A	AMOT	[activation]
21689	CDK1	EZH2	[activation]
21690	VRK3	AIM2	[activation]
21691	HMOX1	CREB3	[activation]
21692	RNF19A	RPS6KA5	[activation]
21693	CTSB	PLAU	[activation]
21694	RARA	HDAC4	[activation]
21695	RAF1	NUDCD3	[activation]
21696	PGM1	UBA5	[activation]
21697	HNRNPA1	PLOD2	[activation]
21698	MAPKAPK5	MAPK1	[activation]
21699	TOP3A	FANCG	[activation]
21700	PPARA	FOXA3	[activation]
21701	MAPK1	TTN	[activation]
21702	RAD21	DEK	[activation]
21703	RHOD	PLXNA1	[activation]
21704	PHLDA3	RFC2	[activation]
21705	SMARCC1	AURKB	[activation]
21706	ITGA4	ESYT1	[activation]
21707	CD200R1	CD200	[activation]
21708	PTPN12	GHR	[activation]
21709	DYRK1A	NXF1	[activation]
21710	KCNA4	KCNA5	[activation]
21711	HUS1B	FKBPL	[activation]
21712	MSH6	NBN	[activation]
21713	NTM	APP	[inhibition]
21714	EPHA1	ALK	[activation]
21715	PTK2B	IL7R	[activation]
21716	STAT5A	CRK	[activation]
21717	GEM	IKZF3	[activation]
21718	TNFRSF14	TRAF2	[activation]
21719	CD2	LCK	[activation]
21720	TRAF6	TRIM28	[activation]
21721	ITGB2	VNN2	[activation]
21722	CFL1	PLD1	[activation]
21723	USP7	NOTCH1	[activation]
21724	C9orf163	LRRK1	[activation]
21725	MAPK3	RPTOR	[activation;inhibition]
21726	MAPT	YWHAQ	[activation]
21727	ING1	TP53	[activation]
21728	RICTOR	PRKCZ	[activation]
21729	VWDE	FBXO6	[inhibition]
21730	SPATA31A3	RELA	[activation]
21731	NBN	ATRX	[activation]
21732	CTNNB1	VHL	[inhibition]
21733	ABL1	SHD	[activation]
21734	EP300	DDIT3	[activation]
21735	EIF4E2	APP	[inhibition]
21736	TAB1	NXPH3	[inhibition]

21737	TRIM28	NR4A1	[inhibition]
21738	XYLT1	MYC	[activation]
21739	FYN	TULP4	[activation]
21740	VCAM1	EIF4G1	[activation]
21741	GABARAP	ZC3HAV1L	[activation]
21742	F2RL2	JAM3	[activation]
21743	CEBPB	CREBBP	[activation]
21744	KDR	PDGFRB	[activation]
21745	LRRK2	EPS8L2	[activation]
21746	EGFR	AHNAK	[activation]
21747	MEF2C	MAPK14	[activation]
21748	DDX56	TTN	[activation]
21749	ACTB	NCF1	[activation]
21750	OPTC	CRK	[activation]
21751	BMP6	NCOA3	[activation]
21752	HIST2H2BE	STK4	[activation]
21753	SMAD2	DCUN1D1	[activation]
21754	TRAF6	ST13	[activation]
21755	EP300	RARA	[activation]
21756	RAC1	CCDC104	[activation]
21757	CCSER2	NDEL1	[activation]
21758	AMOT	YAP1	[activation]
21759	NLGN3	DLG2	[activation]
21760	RASSF1	MAPK8	[inhibition]
21761	TGFBR1	HMMR	[activation]
21762	PDGFB	MDFI	[activation]
21763	TNFRSF1B	FANCD2	[activation]
21764	DAXX	HSF1	[activation]
21765	HIST1H2BD	YWHAZ	[activation]
21766	TNF	UBC	[activation]
21767	RAD50	AIM2	[activation]
21768	EIF4G1	TRAF2	[activation]
21769	SLC2A2	PDX1	[activation]
21770	SLC41A3	MAPK6	[activation]
21771	CCND1	IFI27	[inhibition]
21772	GLI1	VHL	[inhibition]
21773	TP63	GRB2	[activation]
21774	TGFBR2	RCVRN	[inhibition]
21775	YWHAB	SLC8A3	[activation]
21776	ERBB4	PTPN11	[activation]
21777	MYC	TENM2	[activation]
21778	FZD2	UBC	[activation]
21779	EGFR	PHGDH	[activation]
21780	CBL	CAPN1	[activation]
21781	GRB2	AKAP2	[activation]
21782	VCAM1	DDX17	[activation]
21783	RAC1	CHN2	[activation]
21784	YWHAE	PRPSAP1	[inhibition]
21785	JUN	ETS2	[activation]
21786	SP1	NCK1	[activation]
21787	TNF	TRADD	[activation]
21788	GPRASP2	BARD1	[activation]
21789	MET	ABL2	[activation]
21790	GABARAPL1	ENO2	[activation]
21791	RAD54B	GMCL1	[activation]
21792	HSP90AB1	AURKB	[activation]
21793	PLCG2	LAT	[activation]
21794	DNAJA3	ALS2CR11	[inhibition]
21795	RHOC	FAM65B	[activation]
21796	AURKB	BIRC5	[activation]
21797	GPR20	JAM2	[activation]
21798	UBXN7	HIF1A	[activation]
21799	BRAF	HSPA9	[activation]
21800	C8orf33	KCTD6	[activation]
21801	NFYC	TP73	[activation]
21802	FES	RASA1	[activation]
21803	STAT3	NLK	[activation]
21804	UBE2H	MRPL38	[activation]
21805	YWHAH	ARRB2	[inhibition]
21806	CCL3L1	CCR3	[activation]
21807	SPERT	MMAB	[activation]
21808	CYTH4	TRIM54	[activation]
21809	USP7	RPA1	[activation]
21810	ABI2	CCDC36	[activation]
21811	NXF1	AHCTF1	[activation]
21812	EGFR	RAPH1	[activation]

21813	LCP2	CBL	[activation]
21814	RAC1	ALS2	[activation]
21815	ACVR1	SMURF1	[inhibition]
21816	ARRB1	ATR	[activation]
21817	AGAP2	STAT5A	[activation]
21818	TP73	CDK1	[activation;inhibition]
21819	YWHAZ	EIF4A3	[activation]
21820	YWHAZ	ABL1	[activation]
21821	TRIM15	MYBL2	[inhibition]
21822	MCM6	MCM8	[activation]
21823	ICT1	MRPL50	[activation]
21824	RHO	DNAJB2	[activation;inhibition]
21825	HSPA8	TRADD	[activation]
21826	NXF1	MAD2L2	[activation]
21827	ATRIP	PRKDC	[activation]
21828	SERPINI1	PLG	[inhibition]
21829	ANXA7	CHGB	[activation]
21830	ATF1	SPI1	[activation]
21831	BMP4	ZFYVE9	[activation]
21832	PTPRF	TRIO	[activation]
21833	YPEL2	SRPK1	[activation]
21834	EGFR	RPA2	[activation]
21835	PRMT1	ARPC3	[activation]
21836	CCDC149	PRNP	[activation]
21837	DVL1	HDHD2	[activation]
21838	CDKN1A	CKS2	[activation;inhibition]
21839	RAB5A	APPL2	[activation]
21840	FASN	TP63	[activation]
21841	DDX27	GABARAP	[activation]
21842	PLK3	PTPRC	[activation]
21843	HIST2H3A	DNMT3L	[activation]
21844	EIF2S2	CCDC90B	[activation]
21845	BMPR1B	CDK14	[activation;inhibition]
21846	SETD5	TRAF2	[activation]
21847	HSPB1	ATF2	[activation]
21848	WIPI2	GCN1L1	[activation]
21849	HSP90AB1	DLX6	[activation]
21850	ERBB2	CRK	[activation]
21851	CYBB	NCF1	[activation]
21852	PRKCB	TIAM1	[activation]
21853	MMP14	CCL7	[activation]
21854	ITGA4	IGF2BP2	[activation]
21855	ITGA2	KDR	[activation]
21856	CSF3R	JAK1	[activation]
21857	DIRAS3	APC	[activation]
21858	PIK3R2	CBLB	[activation]
21859	CDK3	E2F2	[activation;inhibition]
21860	CREBBP	DYRK1A	[activation]
21861	APC	STRN4	[inhibition]
21862	ARHGEF1	CNKSR1	[activation]
21863	GABARAP	HLA-B	[activation]
21864	PPP1CA	MAX	[inhibition]
21865	FAM98A	GABARAPL2	[activation]
21866	ACTG1	TRAF3IP1	[activation]
21867	USP20	NUP153	[activation]
21868	EZR	CDK5	[activation]
21869	USP29	TP53	[activation]
21870	RIBC2	JUP	[activation]
21871	NUP50	NUP153	[activation]
21872	NR3C1	TP53BP1	[activation]
21873	LEF1	MYB	[activation]
21874	MAPKAPK2	PINX1	[activation]
21875	EP300	HIST1H1A	[activation]
21876	ACTG1	FHOD1	[activation]
21877	RHEBL1	TXNL4A	[activation]
21878	YWHAZ	VCAM1	[activation]
21879	VCAM1	U2AF2	[activation]
21880	SUMO1	DDX39B	[activation]
21881	RPS20	GRB2	[activation]
21882	CREBBP	RPS6KA1	[activation]
21883	UCN2	IL10RB	[activation]
21884	OPRM1	PLD2	[activation]
21885	APP	EIF2S2	[activation]
21886	NKD2	TGFA	[activation]
21887	SMAD4	CDC14B	[inhibition]
21888	SHC1	CSF3R	[activation]

21889	LRRK2	MPC2	[activation]
21890	SRPK1	ZNF444	[activation]
21891	PTPRC	RNF11	[activation]
21892	SMARCA4	STAT2	[activation]
21893	G3BP2	ARRB2	[activation]
21894	HMMR	IL24	[activation]
21895	NCK2	KIT	[activation]
21896	GNAQ	PECAM1	[activation]
21897	GNAI1	GPR50	[activation]
21898	MIF	CUL7	[activation]
21899	FST	DIP2A	[inhibition]
21900	RPA3	PKIG	[activation]
21901	EIF5B	MAPK13	[activation]
21902	HAP1	STX5	[activation]
21903	SEMA4A	PLXND1	[activation]
21904	HSPB1	TOMM70A	[activation]
21905	NCK1	ABL2	[activation]
21906	EP300	ING2	[activation]
21907	SRGAP2	MYO1G	[activation]
21908	DRD2	NCS1	[activation]
21909	SMAD1	SMAD5	[activation;inhibition]
21910	PRKRA	ESR2	[activation]
21911	DPYSL5	DPYS	[activation]
21912	VCAM1	ITSN2	[activation]
21913	FOXP2	FOXP1	[activation;inhibition]
21914	IGF1R	DOK4	[activation]
21915	PIKFYVE	APP	[activation]
21916	IRAK4	ARNT	[activation]
21917	FYN	DLGAP4	[activation]
21918	JAK1	IL27RA	[activation]
21919	RAC3	XIAP	[activation]
21920	LRG1	TGFBR2	[activation]
21921	PRDX3	MAPK3	[activation;inhibition]
21922	NBN	UBE2A	[activation]
21923	ATM	RRM1	[activation]
21924	MYC	FHL2	[activation]
21925	PRKACA	RHOA	[activation]
21926	PIK3R1	LIG3	[activation]
21927	UBC	CD3E	[activation]
21928	NXF1	YAP1	[activation]
21929	EXOC3	EXOC4	[activation]
21930	ESR1	PRKDC	[activation]
21931	YWHAH	NCOA3	[activation]
21932	MAML1	NOTCH4	[activation]
21933	CEBPB	HSF1	[activation]
21934	SMARCA1	BAZ1B	[activation]
21935	CD5	CD247	[activation]
21936	MAP9	AURKB	[activation]
21937	TRAF6	HYOU1	[activation]
21938	PPM1B	SUMO3	[inhibition]
21939	PLCB3	PARD3	[activation]
21940	LONP1	MYC	[activation]
21941	TLN1	ITGA1	[activation]
21942	RPA3	HELB	[activation]
21943	SCYL2	RAPGEF2	[activation]
21944	PRRC2C	AURKB	[activation]
21945	ESR1	MARS	[activation]
21946	C18orf8	NOTCH1	[activation]
21947	CENPT	APP	[activation]
21948	CDKN1A	A2M	[inhibition]
21949	MME	VIP	[activation]
21950	PTH1R	ARRB1	[activation]
21951	HRAS	CDC25A	[activation]
21952	CALM1	HLA-C	[activation]
21953	ANKRD28	DNAJB1	[inhibition]
21954	SH3PXD2A	GRB2	[activation]
21955	TRAF6	SDCBP	[inhibition]
21956	FANCL	FANCG	[activation]
21957	SMAD4	ISG15	[activation;inhibition]
21958	EGFR	CDH5	[activation]
21959	EP300	CTF1	[activation]
21960	AMER3	APC	[inhibition]
21961	SRPK2	MAB21L2	[activation]
21962	YWHAB	SAMD4A	[activation]
21963	MTBP	MDM2	[activation]
21964	STAT4	TRIM28	[activation]

21965	ELMO2	GRB2	[activation]
21966	RAF1	CDC25B	[activation]
21967	DPYSL2	PLD2	[activation]
21968	MDN1	CASP4	[activation]
21969	UBE2E2	RNF25	[activation]
21970	SUV420H2	HSPA4	[activation]
21971	CTCF	RAD21	[activation]
21972	HSPB1	GABARAPL2	[activation]
21973	TRAF6	CBL	[activation]
21974	PRKCD	PPP1CA	[activation]
21975	MAPK1	MBP	[activation]
21976	SUMO3	CASP3	[activation]
21977	PPP2R5A	PPP2R1B	[activation]
21978	TP53	CABLES1	[activation]
21979	JAK3	CD247	[activation]
21980	IPO5	NOTCH1	[activation]
21981	RAD50	HNRNPA1	[activation]
21982	H2AFX	TPT1	[activation]
21983	GSN	STK39	[activation]
21984	EPOR	CISH	[inhibition]
21985	YWHAG	KIAA1598	[activation]
21986	NRAS	FBP2	[activation]
21987	TRIM23	UBE2E2	[activation]
21988	A2M	NGF	[inhibition]
21989	CASP7	STAT1	[activation]
21990	NDUFA4L2	ANXA9	[activation]
21991	HCK	AGK	[activation]
21992	HSPB1	YWHAE	[activation]
21993	KMT2B	GRB2	[activation]
21994	TESK2	CFL1	[activation;inhibition]
21995	RPS17	TP53	[activation]
21996	ASS1	ASCC2	[activation;inhibition]
21997	FCGR2A	SHC1	[activation]
21998	FAM208B	CCNG2	[activation;inhibition]
21999	YIPF1	NXF1	[activation]
22000	PRTN3	ELN	[activation]
22001	HSP90AA1	CAMK2D	[activation]
22002	STX12	SNAP25	[activation]
22003	DMC1	GORASP2	[activation]
22004	ARHGEF5	SFN	[activation]
22005	AKT1	AHSG	[inhibition]
22006	EEF1A2	STK4	[activation]
22007	PAXIP1	PPIA	[activation]
22008	ITGA4	TOP1	[activation]
22009	LUC7L2	CDK4	[activation]
22010	TIAM2	ANXA7	[activation]
22011	DOCK5	GRB2	[activation]
22012	AMOT	PRKCI	[activation]
22013	GHRH	FAP	[activation]
22014	DOK1	RET	[activation]
22015	ARMC6	ILK	[activation]
22016	RCC2	ATG5	[activation]
22017	BID	RPA2	[activation]
22018	NRP2	NRP1	[activation]
22019	MYC	FASTKD1	[activation]
22020	HDAC3	GATA1	[activation]
22021	GP6	FCGR3A	[activation]
22022	SRPK1	TAF1B	[activation]
22023	GNA14	CCR1	[activation]
22024	MAP2K3	MAPK8IP3	[activation]
22025	CDK5	WASF1	[activation]
22026	FASN	EIF4A3	[activation]
22027	BTK	JAK1	[activation]
22028	SMAD2	DOCK9	[activation]
22029	ARHGEF7	ERBB2IP	[activation]
22030	IRF5	GTPBP1	[activation]
22031	TP53	COPS3	[activation]
22032	PIK3R1	FLNC	[activation]
22033	HIST1H4A	SUV420H2	[activation]
22034	CXCL9	BMF	[activation]
22035	DPYSL2	LEF1	[activation]
22036	LIMK1	PXN	[activation]
22037	PRKG1	SMAD4	[inhibition]
22038	CUEDC2	IKBKB	[activation]
22039	SRC	TLR3	[activation]
22040	EGFR	PRKAA1	[activation]

22041	ACTB	SSH1	[activation]
22042	ADRBK1	PRKACA	[activation]
22043	RAPGEF1	NXF1	[activation]
22044	MET	YWHAZ	[activation]
22045	PRKCZ	AKT1	[activation]
22046	STAU1	RAC1	[activation]
22047	DAPK1	MCM3	[activation]
22048	SOS2	ITSN2	[activation]
22049	CBLB	DDR2	[activation]
22050	HDAC4	YWHAB	[activation]
22051	XRCC1	RAN	[activation]
22052	FANCE	FANCF	[activation]
22053	PRKCD	IRAK1	[activation;inhibition]
22054	ATG5	RALB	[activation]
22055	PDHA1	ADRB2	[activation]
22056	PRKCB	BMPR2	[activation]
22057	C1R	PCK1	[activation]
22058	TP53	CDC25C	[activation]
22059	FBP2	PIK3CA	[activation]
22060	TGFB3	TGFB2	[activation]
22061	VAPB	OSBPL10	[activation]
22062	MBL2	CALCR	[activation]
22063	STK4	NCCRP1	[activation]
22064	ERO1L	FBXO6	[activation]
22065	BLK	FCGR2B	[activation]
22066	G3BP1	EIF4G1	[activation]
22067	TNS3	EGFR	[activation]
22068	NUDC	SCAP	[activation]
22069	NXF1	CCNI	[activation]
22070	ENO1	BCL6	[activation]
22071	GRB2	CBLC	[activation]
22072	ITGA4	LYN	[activation]
22073	HUNK	GRB2	[activation]
22074	SRPK2	TMEM14A	[activation]
22075	HN1L	EGR2	[activation]
22076	EP300	PRKCA	[activation]
22077	HSPB1	NOS2	[activation]
22078	APP	ZFYVE16	[activation]
22079	LRRK2	DVL2	[activation]
22080	CFLAR	RIPK2	[inhibition]
22081	TGFBR2	PIK3R1	[activation]
22082	JUN	STAT6	[activation]
22083	SMAD3	BTRC	[inhibition]
22084	PCK1	YWHAQ	[activation]
22085	HIST3H3	MDM2	[activation]
22086	KDR	DNM2	[activation]
22087	GRB2	GSTM3	[activation]
22088	TRIM28	WHSC1	[activation]
22089	IL2RB	RAF1	[activation]
22090	HTT	CASP3	[activation]
22091	CHAC1	RHOXF2	[activation]
22092	EGFR	GNAS	[activation]
22093	PLD1	RPS6KA3	[activation]
22094	GATA1	MAPK3	[activation]
22095	TP53	PAFAH1B3	[activation]
22096	VWF	COL1A1	[activation]
22097	STK38L	YWHAB	[activation]
22098	FYN	CCR10	[activation]
22099	MLH1	KPNA2	[activation]
22100	ALB	APOC3	[inhibition]
22101	CDC42EP3	RHOQ	[activation]
22102	JUN	NCOA3	[activation]
22103	FYN	CD79B	[activation]
22104	EPB41L2	WNK1	[activation]
22105	GIT2	GUSB	[activation]
22106	IGF2BP1	BIRC2	[activation]
22107	PIM1	CDC25A	[activation]
22108	PRKD1	MAPK13	[activation]
22109	PRKD1	MAPK8	[activation]
22110	GADD45B	PPARA	[activation]
22111	DAG1	SRC	[activation]
22112	PDGFB	ART1	[activation]
22113	PLCG1	INSR	[activation]
22114	RAP2A	RALGDS	[activation]
22115	DRD5	GNA13	[activation]
22116	CFTR	ACTB	[activation]

22117	GRK5	RHO	[activation]
22118	SMC4	FBXO6	[activation]
22119	DOCK2	NOTCH2NL	[activation]
22120	KIR3DS1	HLA-B	[activation]
22121	PLCG1	TRPC3	[activation]
22122	TRAF6	MALT1	[activation]
22123	CTTNBP2NL	STK4	[activation]
22124	SMAD2	PAXIP1	[activation]
22125	YAP1	WWTR1	[activation]
22126	ATG14	PIK3R4	[activation]
22127	BCL2L11	CISH	[activation]
22128	RUNX1	CEBPB	[activation]
22129	ITGA4	TOP2A	[activation]
22130	STAT3	PIM1	[activation]
22131	VKORC1	IFITM3	[activation]
22132	MYB	MAPK11	[activation]
22133	ERBB3	PTK6	[activation]
22134	ANAPC15	ANAPC1	[activation]
22135	SPI1	CEBPA	[inhibition]
22136	PPARA	NCOA3	[activation]
22137	CLNK	ERBB2	[activation]
22138	YWHAG	CRTC1	[activation]
22139	RGS4	COPB2	[activation;inhibition]
22140	BTRC	RAP1GAP	[inhibition]
22141	MAPK1	HIF1A	[activation]
22142	SCARF2	FYN	[activation]
22143	IKBKG	CREBBP	[activation]
22144	NTRK3	MAPK1	[activation]
22145	CPVL	CTNNBIP1	[inhibition]
22146	CASP5	NLRP1	[activation]
22147	ATF4	JUN	[activation]
22148	HIF1A	ARRB1	[activation]
22149	CBL	TNFRSF10A	[activation]
22150	GPSM1	INSC	[inhibition]
22151	KCNB2	KCNB1	[activation]
22152	CREBBP	RUNX1	[activation]
22153	SMAD5	HBG2	[activation]
22154	BARD1	BCL3	[activation]
22155	SUMO3	DNAJC8	[inhibition]
22156	RASA1	CDKN2A	[activation]
22157	NFKB1	CD82	[activation]
22158	CAMK2G	ACTN4	[activation]
22159	SCAND1	MAPK3	[activation]
22160	PER1	PER2	[inhibition]
22161	PPP2R1A	ESPL1	[inhibition]
22162	IL1B	APP	[activation]
22163	BRCA1	SMARCA4	[activation]
22164	BBC3	BCL2L1	[activation]
22165	MAPK9	NFE2L2	[activation]
22166	SERPINF2	PLG	[activation]
22167	PRKACA	AANAT	[activation;inhibition]
22168	ERBB2	STAT1	[activation]
22169	ANXA2	ESR1	[activation]
22170	ELAVL1	CDC23	[activation]
22171	APOE	RHEB	[activation]
22172	FOXO1	FHL2	[activation]
22173	RBPJ	GSK3B	[inhibition]
22174	SP1	AR	[activation]
22175	FBXO6	TTC4	[inhibition]
22176	PTK2B	SH2D3C	[activation]
22177	DDX18	ESR1	[activation]
22178	FCHSD1	ITSN2	[activation]
22179	KLHL42	USHBP1	[activation]
22180	MAPK3	BLVRA	[activation]
22181	STAP2	BMP4	[activation]
22182	DYNLRB1	BCL2L1	[activation]
22183	ETV5	LRRK2	[activation]
22184	DNM3	GRB2	[activation]
22185	SUMO1	IGF1R	[activation]
22186	HSF1	HSF2	[activation]
22187	CASP3	SRF	[activation]
22188	ATF4	GCC1	[activation]
22189	RAC2	ACTB	[activation]
22190	HMGA2	HDGF	[activation]
22191	EGFR	MST4	[activation]
22192	ESM1	ITGB2	[activation]

22193	SSH1	DSTN	[activation]
22194	KPNA3	RELA	[activation]
22195	VTI1B	TNK2	[activation]
22196	IKBKG	CHUK	[activation]
22197	EHMT2	CEBPB	[activation]
22198	HIST2H3A	LALBA	[activation]
22199	CNP	PRNP	[activation]
22200	AMBP	A2M	[inhibition]
22201	RAB11FIP2	RAB11A	[activation]
22202	EPHA2	CDH5	[activation]
22203	SMARCA4	MYOG	[activation]
22204	CD79A	PTPN6	[activation;inhibition]
22205	CAD	MAPK1	[activation;inhibition]
22206	CCNH	GTF2H1	[activation]
22207	ERBB4	CD44	[activation]
22208	VAV2	CBLB	[inhibition]
22209	PPP2CA	STK25	[inhibition]
22210	TNK2	CSPG4	[activation]
22211	VCAM1	LY6G5B	[activation]
22212	DYRK1A	HIST1H3A	[activation]
22213	CCDC155	STX5	[activation]
22214	PRKAG1	EGFR	[activation]
22215	CEBPA	CDX1	[activation]
22216	DLGAP4	ABL1	[activation]
22217	HSPA1L	MAP3K7	[activation]
22218	EIF2S2	SUMO2	[activation]
22219	DNAJA1	HLA-B	[inhibition]
22220	RPS6KA3	HIST3H3	[activation]
22221	PPP1CA	IKBKB	[activation]
22222	ARL8B	PTBP3	[activation]
22223	RANBP2	CEBPA	[activation]
22224	GKAP1	PRKG1	[inhibition]
22225	RPAP3	ANAPC1	[activation]
22226	TTF1	BAZ2A	[activation]
22227	APOE	SORL1	[activation]
22228	MET	STAP1	[activation]
22229	IRAK2	HSP90AB1	[activation]
22230	RAP1B	RASSF5	[activation]
22231	FBXO34	TGFBR1	[inhibition]
22232	KAT2B	KLF10	[activation]
22233	CDK4	MARCKS	[activation]
22234	PCNA	RELA	[activation]
22235	VPS52	PRKAA1	[inhibition]
22236	EP300	HOXB9	[activation]
22237	HSPA1A	PTGES3	[inhibition]
22238	NUP62	KANSL1	[activation]
22239	PAXIP1	HSPA4L	[activation]
22240	CRMP1	SRC	[activation]
22241	CRK	MAPK8	[activation]
22242	EGFR	DOK4	[activation]
22243	CCSER2	STX1A	[activation]
22244	ANAPC1	CDC27	[activation]
22245	SYK	LAX1	[activation]
22246	MAPK8	NR3C1	[activation]
22247	PTK2	IRS1	[activation]
22248	RANBP1	XPO1	[activation]
22249	STX3	SNAP23	[activation]
22250	FAN1	PMS1	[activation]
22251	STK38	ARRB2	[inhibition]
22252	H1FX	VCAM1	[activation]
22253	TP53	RRM2	[activation]
22254	IL24	AKT1	[activation]
22255	VDAC2	SNCA	[inhibition]
22256	BMP4	HTRA1	[activation]
22257	PIK3R1	MAL	[activation]
22258	EXOC6	NOS3	[activation]
22259	RASD1	NOS1AP	[activation]
22260	LRPAP1	RALA	[inhibition]
22261	MEP1A	GNRH1	[activation]
22262	MIF	COPS6	[activation]
22263	LRRK2	RAB32	[activation]
22264	SLC9A3R1	MDM2	[activation]
22265	MAPK8	MAP3K4	[activation]
22266	PARD6B	YWHAZ	[activation]
22267	SMARCA5	MYC	[activation]
22268	SOX2	GCN1L1	[activation]

22269	NCK1	MYLK	[activation]
22270	SRSF5	MAPK14	[activation]
22271	FAM102A	SKAP1	[activation]
22272	PHLDA3	FASTKD2	[activation]
22273	AR	MDM2	[activation]
22274	RAB11A	PPP2R1B	[activation]
22275	BAK1	ACTR2	[activation]
22276	F10	GGCX	[activation]
22277	TRIM24	NR3C1	[activation]
22278	GADD45A	GADD45GIP1	[activation]
22279	CD4	GAPDH	[activation]
22280	TP53	ARL3	[activation]
22281	YWHAZ	RALGPS2	[activation]
22282	PLK1	CCNB1	[activation]
22283	CUX1	KAT2B	[activation]
22284	YWHAZ	RPS6	[activation]
22285	UBE2H	HIST2H2AA3	[activation]
22286	FOS	MAP1B	[activation]
22287	CASP8	TFCP2	[inhibition]
22288	UCN	LEP	[activation]
22289	NRAS	STX17	[activation]
22290	PARVA	ILK	[activation]
22291	HNRNPL	VEGFA	[activation]
22292	CTNNB1	CDK6	[inhibition]
22293	ENO3	APP	[activation]
22294	PIK3R2	NXF1	[activation]
22295	FXR2	SRPK2	[activation]
22296	ASB16	H3F3A	[activation]
22297	APP	GADD45G	[activation]
22298	DYRK1A	PRKACA	[activation]
22299	GAPDH	LAMA4	[activation]
22300	ATP6V1A	VAMP7	[activation]
22301	CMKLR1	TPST1	[activation]
22302	BCAR3	BEX1	[activation]
22303	ATAT1	APP	[activation]
22304	HSPA4L	HSPA8	[inhibition]
22305	BANK1	YWHAZ	[activation]
22306	SRC	HDAC3	[activation]
22307	TP53	RABL6	[activation]
22308	BRD4	C8orf33	[activation]
22309	RAD21	EIF2S3	[activation]
22310	SMAD2	SIK3	[activation]
22311	SMAD3	RBL1	[inhibition]
22312	MAP3K2	HSP90AB1	[activation]
22313	ENO3	MTMR14	[activation]
22314	STAT1	RAC1	[activation]
22315	NOTCH2NL	HCK	[activation]
22316	RAD50	DDX1	[activation]
22317	TP53	KPNA4	[activation]
22318	ABL1	G3BP2	[activation]
22319	PTN	KPNB1	[activation]
22320	IGF2	IGFBP1	[activation]
22321	TGFB1I1	CBLC	[inhibition]
22322	KDR	STAT1	[activation]
22323	WARS	GBAS	[activation]
22324	IGSF1	ACVR2A	[activation]
22325	SCARB1	CUL1	[activation]
22326	BOLA2	VCAM1	[activation]
22327	ITCH	NUMB	[activation]
22328	ALK	ACTB	[activation]
22329	HTT	PIAS1	[inhibition]
22330	EP300	RUNX1	[activation]
22331	BIRC2	TNFSF14	[activation]
22332	CTGF	FGFR3	[activation]
22333	PRDX2	PSEN1	[activation]
22334	PPP1CA	PPP1R3E	[activation;inhibition]
22335	NR4A1	SRC	[activation]
22336	ITGA4	GAPDH	[activation]
22337	EP300	TCF12	[activation]
22338	TRAF6	MCM6	[activation]
22339	MITF	EP300	[activation]
22340	SRPK1	ACIN1	[activation]
22341	LAMTOR5	YTHDC1	[activation]
22342	RPA1	CAV1	[activation]
22343	CHRNA5	CHRNB4	[activation]
22344	GRB2	NIF3L1	[activation]

22345	HIST1H2BN	CD81	[activation]
22346	SRPK2	ACIN1	[activation]
22347	MTNR1B	YWHAQ	[activation]
22348	ROR1	EGFR	[activation]
22349	GRB2	NFYB	[activation]
22350	RASAL3	WNK1	[activation]
22351	GRB2	RAB6B	[activation]
22352	PPP2CA	PRKDC	[activation]
22353	WNK1	SLC9A1	[activation]
22354	LAMA3	BMP1	[activation]
22355	TAGLN2	CDK2	[activation]
22356	FYN	SHANK3	[activation]
22357	FYN	PTPN4	[activation]
22358	EGFR	SLA	[activation]
22359	LYZ	CRK	[activation]
22360	TRAF3IP1	SMC2	[activation]
22361	GRB2	DDX17	[activation]
22362	TGFBR3	ARRB2	[inhibition]
22363	FER	YWHAB	[activation;inhibition]
22364	INSR	CEACAM1	[activation]
22365	GTF2E1	SRPK2	[activation]
22366	PRKAA2	PRKAB1	[inhibition]
22367	OTUD4	DSG1	[activation]
22368	CASP4	CCDC59	[activation]
22369	MAPK3	GH1	[activation]
22370	RBL1	MAPK6	[activation;inhibition]
22371	RELA	BANP	[activation]
22372	TRAF5	TRAF6	[activation]
22373	DVL2	POLI	[activation]
22374	APP	AGER	[activation]
22375	IRS4	AURKB	[activation]
22376	PLK1	NUAK1	[activation]
22377	SRPK2	SON	[activation]
22378	GNAI1	EPOR	[activation;inhibition]
22379	CCL14	CCR3	[activation]
22380	GSN	SRC	[activation]
22381	GDI2	TNFRSF14	[activation]
22382	C11orf65	CALM1	[activation]
22383	KRTAP10-3	BMP7	[activation]
22384	RPRD1A	BCAR3	[activation]
22385	PPBP	CMTM6	[activation]
22386	YWHAB	KIF23	[activation]
22387	PPARG	FOXO1	[activation]
22388	TGFBR1	TGFB1	[activation]
22389	CDH7	CDH6	[activation]
22390	RAB28	APP	[activation]
22391	TP53RK	OSGEP	[activation]
22392	SRSF1	SRSF5	[activation]
22393	WWC3	FTSJ1	[activation]
22394	XPR1	AMOT	[activation]
22395	RAP1A	RGL4	[activation]
22396	TGFBR1	PPP3CC	[inhibition]
22397	RCC2	FTSJ1	[activation]
22398	UBE2I	SLC9A1	[activation]
22399	ERBB4	ABL2	[activation]
22400	EEA1	CCDC8	[activation]
22401	PTPRJ	TEK	[activation]
22402	EIF4A3	RAN	[activation]
22403	CCND3	DMTF1	[inhibition]
22404	TPT1	SPP1	[activation]
22405	TXNDC17	AVP	[activation]
22406	CITED2	LHX3	[activation]
22407	HSPB1	GRB2	[activation]
22408	IRS4	RAF1	[activation]
22409	NR5A1	FYN	[activation]
22410	RUNX2	SOX9	[activation]
22411	RELA	STAT6	[activation]
22412	IFNAR2	IFNAR1	[activation]
22413	AXIN1	DAB2IP	[activation]
22414	PPP1CC	RPAP3	[inhibition]
22415	IKBKG	F7	[activation]
22416	RIPK3	HSPA1L	[inhibition]
22417	UCK2	VCAM1	[activation]
22418	HIST3H2BB	RPS6KA5	[activation]
22419	FBXO6	LEPR	[activation]
22420	SULT1E1	BMPR2	[activation]

CASP8	MAP3K14	[inhibition]
SLX4	ERCC4	[activation]
RXRB	NR1H3	[inhibition]
SMARCD3	MAPK14	[activation]
GNB1	BTK	[activation]
LATS1	MOB1B	[inhibition]
CACNA1A	VWF	[inhibition]
IKBKG	ARF6	[activation]
FADD	TNF	[activation]
CDK2	MYBL2	[inhibition]
RPA1	C7orf50	[activation]
INPP5D	CRKL	[activation]
GNA12	TSHR	[activation]
NFE2L2	PRKCD	[activation]
GNB2	STAT5A	[activation]
BID	EZR	[activation]
CTSG	SELPLG	[activation]
CBLB	PIK3R1	[activation]
C1R	C1S	[activation]
RPS6KA3	GSTK1	[activation]
GMFG	VANGL1	[activation]
PRKCA	CYTH2	[activation]
BCL6	POU2F3	[activation]
CDC23	PAXIP1	[activation]
PACSIN3	SOS1	[activation]
NUDCD3	KLHL6	[activation]
CDC42	CLIP1	[activation]
HOXB1	MEIS1	[activation]
CDK15	CDK2	[activation]
INSIG2	INSIG1	[activation]
IRS1	YWHAE	[activation]
APP	ARFGAP1	[activation]
EIF3I	SGK1	[activation]
CD3G	FYN	[activation]
VCAM1	ARPC4	[activation]
EIF4A1	PAIP1	[activation]
HGS	YWHAB	[activation]
IFITM3	FYN	[activation]
MAPT	SYK	[activation]
SMAD2	DDX3Y	[activation]
LCP2	BLNK	[activation]
STRAP	SMURF1	[inhibition]
EGFR	MARS	[activation]
CYLC2	NRAS	[activation]
CDKN1A	NRBP1	[activation]
SRPK1	SRSF12	[activation]
PRKDC	CASP2	[activation]
APH1A	PSEN1	[activation]
NXF1	C10orf76	[activation]
BHLHE40	PPP2R1A	[activation]
ATM	HDAC1	[activation]
CPSF3	HSPB1	[activation]
STRA13	FANCE	[activation]
SHANK2	GRB2	[activation]
FCER2	ATF7	[activation]
CDK11A	A2M	[inhibition]
EFEMP2	HHEX	[activation]
PPP1R12A	PTPRJ	[activation]
CTBP1	PLCB1	[activation]
RAC1	NCK1	[activation]
EIF2B2	EIF2B1	[activation]
ENOX2	SNRPA	[activation]
PRDX2	BAD	[activation;inhibition]
ATF3	HSP90AB1	[activation]
SPP1	RPL15	[activation]
CMA1	IL1B	[activation]
RALBP1	CASP8	[activation]
AFF1	KMT2A	[activation]
STK11	SIRT1	[activation]
MFAP4	TP53	[activation]
NUP62	OGT	[activation]
TDRD7	STK11	[activation]
PPP3CA	GRB2	[activation]
PAX6	SMAD3	[activation]
ANAPC4	SOX2	[activation]
CDC42	VRK2	[activation]

22497	LRP5L	PLK1	[activation;inhibition]
22498	SOS1	TNFRSF1A	[activation]
22499	TP53	ACTBL2	[activation]
22500	VCAM1	SYK	[activation]
22501	ABRA	MYC	[activation]
22502	HBEGF	BCL6	[activation]
22503	MDM2	PHGDH	[activation]
22504	CDON	CDH15	[activation]
22505	GNAS	JAK3	[activation]
22506	CSNK2A1	TP53	[activation]
22507	BCL6	NACC1	[activation]
22508	PLOD2	OS9	[activation]
22509	EIF2AK4	BAG2	[inhibition]
22510	YWHAB	RALGPS2	[activation]
22511	NRP2	VEGFA	[activation]
22512	CD93	CRK	[activation]
22513	WAS	PTPRB	[activation]
22514	TNFSF14	DIABLO	[activation]
22515	NLK	C2orf44	[inhibition]
22516	ANKRD11	SRC	[activation]
22517	BTK	PRKD1	[activation]
22518	REM1	CACNB1	[activation]
22519	TTC1	HSPA8	[inhibition]
22520	PRMT5	VCAM1	[activation]
22521	HPR	IGHM	[activation]
22522	PUM1	FBXO6	[inhibition]
22523	NCOA1	NKX2-1	[activation]
22524	ESR2	CCDC25	[activation]
22525	OBSL1	TP53	[activation]
22526	TAOK2	GRB2	[activation]
22527	TSC1	TBC1D7	[inhibition]
22528	PRKAA1	FKBP5	[inhibition]
22529	TGFBR1	SMURF1	[inhibition]
22530	DGKD	DYSF	[activation]
22531	STRN3	STK24	[activation]
22532	PTPN1	GSK3B	[activation]
22533	STK4	MCM7	[activation]
22534	VPREB1	LGALS1	[inhibition]
22535	INSC	PARD3B	[inhibition]
22536	CDKN1B	GRB2	[activation]
22537	EPHA5	STAT3	[activation]
22538	NMT1	CAPN1	[activation]
22539	PNPLA6	NXF1	[activation]
22540	PDGFA	COL6A1	[activation]
22541	SMAD4	FOXO1	[activation]
22542	ATG101	ATG5	[activation]
22543	SLC9A2	SRC	[activation]
22544	TIMELESS	ATRIP	[activation]
22545	HIC1	TCF7L2	[inhibition]
22546	RASSF5	HRAS	[activation]
22547	APP	EPHB4	[activation]
22548	PIK3R1	SUV39H2	[activation]
22549	IRS1	CYTH3	[activation]
22550	EGFR	SFXN3	[activation]
22551	XPO1	ATF2	[activation]
22552	NBAS	HSPB1	[activation]
22553	KPNA2	JUN	[activation]
22554	HSPA2	TTC1	[activation]
22555	SPI1	IRF8	[inhibition]
22556	CAPN7	UBL7	[activation]
22557	CTSG	PPBP	[activation]
22558	CASP3	MET	[activation]
22559	EP300	ESR1	[activation]
22560	TNFSF10	RBM48	[activation]
22561	PDGFRB	PTPRK	[activation]
22562	NOTCH1	SMAD9	[activation]
22563	CAV1	VAV2	[activation]
22564	DOK1	ITK	[activation]
22565	PRKCE	HSP90AB1	[activation]
22566	AGFG1	APC	[activation]
22567	SHARPIN	IKBKB	[activation]
22568	UBE2W	UFM1	[activation]
22569	APOE	CTSB	[activation]
22570	SETDB1	ULK2	[activation]
22571	STAT3	NIF3L1	[activation]
22572	RBX1	CCND1	[inhibition]

22573	FAM194B	CD247	[activation]
22574	MCM3	RAD21	[activation]
22575	MAPRE1	APC2	[activation]
22576	PLSCR1	SLC25A6	[activation]
22577	ANXA7	FGB	[activation]
22578	INSR	SH2B1	[activation]
22579	USP45	IGKV4-1	[activation]
22580	JUP	PTPRJ	[activation]
22581	SMAD5	PAX6	[activation]
22582	TP53	DAPK1	[activation]
22583	ELANE	CSF3	[activation]
22584	ARF6	UBE2D3	[activation]
22585	KLRK1	HCST	[activation]
22586	BCR	TCF7	[activation]
22587	MLH1	EIF2A	[activation]
22588	HMGA2	CSNK2A1	[activation]
22589	PTK2	TNS3	[activation]
22590	CUTA	NR4A1	[inhibition]
22591	ACACA	GABARAP	[activation;inhibition]
22592	KIF23	ARF6	[activation]
22593	TWIST1	CDK9	[activation]
22594	PHYHIP	PNPLA2	[activation]
22595	MTG2	TAB1	[inhibition]
22596	RPA3	WDHD1	[activation]
22597	SMARCA2	EPAS1	[activation]
22598	KAT2B	SAT2	[activation]
22599	TNF	SEC16A	[activation]
22600	GIP	GIPR	[activation]
22601	HNF4A	MAPK8	[activation]
22602	NR2F6	NR3C1	[activation]
22603	PRKCD	PAK1	[activation]
22604	CDK14	ACVR1	[activation;inhibition]
22605	CCND1	MYBL2	[inhibition]
22606	NOTCH1	LRCH1	[activation]
22607	SMARCA4	HSP90B1	[activation]
22608	GRB2	FCGR2C	[activation]
22609	SYN1	PAK1	[activation]
22610	SRPK2	FGF12	[activation]
22611	GP1BB	LYN	[activation]
22612	CCDC53	CCHCR1	[activation]
22613	ESR1	FTSJ3	[activation]
22614	ASCC2	LPL	[activation]
22615	MAPK9	LAMA1	[activation]
22616	KCNA3	KCNA5	[activation]
22617	VCAM1	FASN	[activation]
22618	RAB5A	RABEP2	[activation]
22619	DEF6	ZAP70	[activation]
22620	EEA1	RABGEF1	[activation]
22621	PAXIP1	PAGR1	[activation]
22622	HSPB1	NCKIPSD	[activation]
22623	STYXL1	EHD4	[activation]
22624	BCL2L1	BID	[activation]
22625	ACVR1B	SMAD4	[activation]
22626	JAK2	SH2B2	[activation]
22627	CAGE1	TBC1D23	[inhibition]
22628	NR3C1	NCL	[activation]
22629	PLCB1	KRIT1	[activation]
22630	NUP98	EP300	[activation]
22631	TNFRSF10C	BMX	[activation]
22632	AKT1	MAP3K11	[activation]
22633	NGF	ROR1	[activation]
22634	BRD4	CRK	[activation]
22635	MDM2	PIM3	[activation]
22636	PAK1	HSP90AA1	[activation]
22637	VAPA	STK4	[activation]
22638	SUV420H1	YWHAG	[activation]
22639	SMURF2	RAN	[activation]
22640	UBE2I	TRAF3	[activation]
22641	NFE2L2	NCOR2	[activation]
22642	MIF	ATG5	[activation]
22643	RECQL5	POLR2D	[activation]
22644	CMBL	SMYD2	[activation]
22645	AKT2	XIAP	[inhibition]
22646	MYL6	HLA-B	[activation]
22647	CSPP1	MYC	[activation]
22648	ASB14	NOTCH2	[activation]

22649	QTRTD1	IGHA1	[activation]
22650	LIFR	IL6ST	[activation]
22651	HSPB1	TRIM24	[activation]
22652	ZBTB7A	TP53	[activation]
22653	PRKCQ	NFE2L2	[activation]
22654	JAK2	SKP2	[activation]
22655	VAMP7	UBC	[activation]
22656	CUL1	CHEK2	[activation]
22657	HIF1AN	MTPN	[activation]
22658	CTNNB1	PTPN12	[activation]
22659	YWHAH	NRIP1	[activation]
22660	TGFBR2	FANCC	[activation]
22661	ARHGDIB	ANKRD1	[activation]
22662	H2AFX	ANXA4	[activation]
22663	PDGFB	NRP1	[activation]
22664	PPARGC1A	EP300	[activation]
22665	ATF3	CREBBP	[activation]
22666	UBE2I	MAP2K1	[activation]
22667	SORBS1	PAK2	[activation]
22668	APH1B	PSEN1	[activation]
22669	MAPT	SLC1A2	[activation]
22670	UNC13B	WDR48	[activation]
22671	MTCP1	AKT2	[activation]
22672	TRAF3	IRF1	[activation]
22673	E2F4	CEBPA	[activation;inhibition]
22674	PKDREJ	ERBB2	[activation]
22675	PXN	VCAM1	[activation]
22676	TNFRSF19	TRAF6	[activation]
22677	MOB1A	TRAF6	[activation]
22678	SP1	SP3	[activation]
22679	NCOA1	STAT5A	[activation]
22680	CASP10	MAP3K14	[activation]
22681	ADAM17	PDPK1	[activation]
22682	MYC	GSG2	[activation]
22683	EP300	THPO	[activation]
22684	NR2E3	HDAC1	[inhibition]
22685	PSPN	GFRA4	[activation]
22686	PRKCD	PRKCQ	[activation]
22687	ATF4	TBP	[activation]
22688	ARHGEF7	SRGAP1	[activation]
22689	CCND3	RBX1	[inhibition]
22690	ARHGAP17	BTK	[activation]
22691	CEP55	NOS3	[activation]
22692	USP6	CALM1	[activation]
22693	RTEL1	RPA2	[activation]
22694	TEX36	PPP1CC	[inhibition]
22695	YIF1A	VAPB	[activation]
22696	TNFRSF1A	FASLG	[activation]
22697	HSPA8	HSPH1	[activation]
22698	SUV39H1	RUNX1	[activation]
22699	RASA1	PXN	[activation]
22700	TNNT1	BMPR1B	[activation;inhibition]
22701	LMO7	GRB2	[activation]
22702	FASN	AKT1	[activation]
22703	CAV1	PTCH1	[activation]
22704	EIF2S3	SUMO2	[activation]
22705	H2AFX	STK11	[activation]
22706	CD244	LAT	[activation]
22707	ACTB	PPP2R2B	[activation]
22708	OBSL1	NDC1	[activation]
22709	GNA13	S1PR4	[activation]
22710	MYC	PDLIM4	[activation]
22711	FOSL2	EP300	[activation]
22712	PBX1	FOXC1	[activation]
22713	LRRK2	CDC42EP3	[activation]
22714	CAV1	RADIL	[activation]
22715	FANCD2	CDK20	[activation]
22716	MAPT	PHKG1	[activation]
22717	GTPBP2	E2F3	[activation;inhibition]
22718	ATXN10	PPP1R12A	[activation]
22719	SIRT2	SARS	[activation]
22720	CHEK2	RNF20	[activation]
22721	DCXR	TRAF6	[activation]
22722	GPSM1	RALBP1	[inhibition]
22723	PCNA	HLA-C	[activation]
22724	MAGED2	IRS4	[activation]

22725	LSM14A	TP53	[activation]
22726	CEP250	EIF4EBP3	[inhibition]
22727	GNB1	ERBB2	[activation]
22728	MAPK1	GAB2	[activation]
22729	HSP90AB1	KLHL34	[activation]
22730	BTLA	PTPN11	[activation]
22731	KDM5A	HIST3H3	[activation]
22732	DOCK8	MOB1A	[activation]
22733	TRIP10	SRC	[activation]
22734	MSANTD3	BMPR1A	[activation;inhibition]
22735	MLH1	BACH1	[activation]
22736	HSD17B4	PLEK	[activation]
22737	NXF1	DEPTOR	[activation]
22738	RBPJ	APP	[inhibition]
22739	BRCA1	AURKA	[activation]
22740	HSPA1A	TTC1	[inhibition]
22741	WNK1	EIF3A	[activation]
22742	RASA1	GAB1	[activation]
22743	RARS	EGFR	[activation]
22744	GLI3	YWHAE	[activation]
22745	BANP	EPHA10	[activation]
22746	DDX17	NCOA3	[activation]
22747	NR3C1	KIAA0408	[activation]
22748	RASSF4	HMGB1	[inhibition]
22749	HLA-A	HYOU1	[activation]
22750	GCGR	BMPR2	[activation]
22751	TRAFD1	TRAF6	[activation]
22752	ICAM1	RPL23A	[activation]
22753	KCNJ12	PRKACA	[activation]
22754	EFCAB4B	ORAI1	[activation]
22755	SRPK1	RSRC2	[activation]
22756	TRIP6	BCAR1	[activation]
22757	DFFA	CIDEB	[activation]
22758	F12	UBE2D1	[activation]
22759	SETDB1	UBE2I	[activation]
22760	TNF	TBK1	[activation]
22761	VCAM1	EZR	[activation]
22762	SOCS1	CSF1R	[inhibition]
22763	EP300	HIST1H2AB	[activation]
22764	ITPR1	BANK1	[inhibition]
22765	IRF7	TRMT61B	[activation]
22766	ABI1	FGR	[activation]
22767	TRAF6	CD40	[activation]
22768	BAAT	CDKN2A	[activation;inhibition]
22769	CDKN1A	TP53	[activation]
22770	CHUK	SRPK1	[activation]
22771	EGLN1	POLR2A	[activation]
22772	TNFRSF14	RNF219	[activation]
22773	PBX1	HDAC1	[activation]
22774	SUMO1	ACOT8	[activation]
22775	MDM2	DSTN	[activation]
22776	LTBP4	FRAT1	[inhibition]
22777	PIK3R1	MYLK	[activation]
22778	GNAI2	CHRM1	[activation]
22779	CCDC88B	PLEKHA5	[activation]
22780	HIST1H1C	RELA	[activation]
22781	PTPRC	GRIN2B	[activation]
22782	BMPR2	FHL2	[activation]
22783	MAPT	HSPA8	[activation]
22784	SOX6	HDAC1	[activation]
22785	CSNK1A1	EIF2B5	[activation]
22786	CDC5L	SMC2	[activation]
22787	PPP1CA	PPP1R15B	[activation;inhibition]
22788	KIAA1033	WASH1	[activation]
22789	ITGA9	FIGF	[activation]
22790	RIT1	SMAD2	[activation]
22791	DUSP8	CDC25A	[inhibition]
22792	TRPM7	MBP	[activation]
22793	ABL1	SUV39H2	[activation]
22794	RPA3	EIF2S1	[activation]
22795	CDK2	OGT	[activation]
22796	GDF9	TRIB3	[activation]
22797	TRAF6	HSPE1	[activation]
22798	MRPL43	ITGA4	[activation]
22799	INSIG1	UBC	[activation]
22800	SRPK1	CHEK2	[activation]

22801	HNRNPH2	GRB2	[activation]
22802	CXCR4	DBN1	[activation]
22803	TRAF3	PPP3CA	[activation]
22804	SMAD2	CORO2A	[activation;inhibition]
22805	CALM1	MYF5	[activation]
22806	RPA3	HSPA8	[activation]
22807	SCRIB	WWTR1	[activation]
22808	NDEL1	TACC3	[activation]
22809	SSR1	ILK	[activation]
22810	EP300	EGR1	[activation]
22811	NCAM2	PRNP	[activation]
22812	NXF1	RCN1	[activation]
22813	BARD1	FBXO5	[activation]
22814	KAT2B	RB1	[activation]
22815	CKS1B	CDK3	[activation]
22816	HSP90AB1	IGF1R	[activation]
22817	STRADB	TRAF6	[activation]
22818	PRNP	MAG	[activation]
22819	PRKDC	E2F3	[activation]
22820	LRRC4	NTNG1	[activation]
22821	FDX1	NR4A2	[activation]
22822	HRAS	GPSM2	[activation]
22823	PPP1R18	TRAF2	[activation]
22824	ARRB1	MAP2K4	[activation]
22825	KAT2B	HIF1A	[activation]
22826	CD22	LCP2	[activation]
22827	BCL2	HSPA1A	[inhibition]
22828	RBL1	EPHB2	[activation]
22829	TNIK	BCAP31	[activation]
22830	CDC5L	GULP1	[activation]
22831	EIF4G1	PAK2	[activation]
22832	NFKB1	TWIST1	[activation]
22833	MAP3K8	TAB2	[activation]
22834	WASL	PTK6	[activation]
22835	MAP1LC3B	STK3	[activation]
22836	SMURF1	SENP8	[inhibition]
22837	CXCR1	ADRA1A	[activation]
22838	WASF2	BTK	[activation]
22839	STAT5A	PRMT1	[activation]
22840	VAV3	BLNK	[activation]
22841	SARS2	ICT1	[activation]
22842	ETFA	PSEN1	[activation]
22843	CREBBP	N4BP2	[activation]
22844	ARHGDIA	JUP	[activation]
22845	ARPC2	CTTN	[activation]
22846	IL32	C1QB	[activation]
22847	AKAP4	FSIP2	[inhibition]
22848	NARF	LEF1	[activation]
22849	NOS1	ZDHHC23	[activation]
22850	LRRK2	SRPK1	[activation]
22851	IRF2	HMGN1	[activation]
22852	CCT8	NOTCH1	[activation]
22853	ACTG1	MAP3K7	[activation]
22854	ADORA2A	ACTN3	[activation]
22855	RELA	IRF5	[activation]
22856	TIAM2	DNM2	[activation]
22857	MAPT	UBE2D2	[activation]
22858	HIST3H3	MCM2	[activation]
22859	SDF4	ANXA7	[activation]
22860	MAX	MYCL	[inhibition]
22861	PPP3CA	NR3C1	[activation]
22862	TTK	NXF1	[activation]
22863	BCL2L13	VAMP3	[activation]
22864	STAT3	MAP3K7	[activation]
22865	PKN1	CD44	[activation]
22866	HNRNPR	SRPK3	[activation]
22867	KLK6	SERPINF2	[activation]
22868	PRKCQ	IKBKG	[activation]
22869	ARAP1	TAB1	[inhibition]
22870	PA2G4	HMGA2	[activation]
22871	SPP1	TMEM30A	[activation]
22872	ERBB2IP	MEF2A	[activation]
22873	RAC2	HDAC10	[activation]
22874	MMP13	CCL7	[activation]
22875	ANAPC5	ANAPC15	[activation]
22876	PSENEN	FTL	[activation]

22877	SMAD5	SMAD2	[activation]
22878	NOTCH1	ARRB1	[activation]
22879	APP	PTK6	[activation]
22880	RAE1	BUB1	[activation]
22881	SSH1	CFL2	[activation]
22882	SRPK1	ECM1	[activation]
22883	PRRC2A	GRB2	[activation]
22884	MAP3K3	PRKACA	[activation]
22885	POU1F1	JUN	[activation]
22886	LCK	FASLG	[activation]
22887	CHD8	CTCF	[activation]
22888	CAND1	EIF4EBP3	[inhibition]
22889	XIAP	AKT1	[activation]
22890	FOS	EGFR	[activation]
22891	SLC25A6	RELA	[activation]
22892	GOPC	RHOQ	[activation]
22893	UBA5	TARS	[activation]
22894	TGFBR1	ARL8B	[activation]
22895	EP400	ESR1	[activation]
22896	PWP1	SMURF1	[inhibition]
22897	PTPRK	JUP	[inhibition]
22898	BPTF	DPY30	[activation]
22899	TRAF1	CD40	[activation]
22900	PRKAA1	RFX6	[activation]
22901	LATS1	CEP152	[inhibition]
22902	MRC2	AKT1	[activation]
22903	JAG1	CD46	[activation]
22904	ADAMTSL4	TSSK3	[activation]
22905	STIM1	SUMO1	[activation]
22906	VCL	SUMO2	[activation]
22907	EIF4A3	POTEJ	[activation]
22908	TSC1	CDK1	[activation;inhibition]
22909	CFTR	TIAM1	[activation]
22910	ICAM1	NOS2	[activation]
22911	RAC1	PARD6G	[activation]
22912	KMT2A	SBF1	[activation]
22913	UGT1A1	UGT1A7	[activation]
22914	FZR1	CKAP2	[inhibition]
22915	PPIA	ATF2	[activation]
22916	RAP1GDS1	KRAS	[activation;inhibition]
22917	ESR1	CAV1	[activation]
22918	RELA	TP53	[activation]
22919	PENK	OPRM1	[activation]
22920	SPP1	ACP5	[activation]
22921	EP300	BUB1B	[inhibition]
22922	GNAI2	CNR1	[activation]
22923	PIK3R1	AR	[activation]
22924	PIK3R1	ADAM12	[activation]
22925	ACACA	SIRT1	[activation;inhibition]
22926	RHOB	DAAM1	[activation]
22927	CDK2	C12orf65	[activation]
22928	MCL1	TP53	[activation]
22929	TP53	MRPS27	[activation]
22930	EGFR	EXOC8	[activation]
22931	NRXN3	NLGN1	[activation]
22932	CD22	IGHM	[activation]
22933	WDR73	TNFRSF14	[activation]
22934	GRB2	UBR4	[activation]
22935	PALB2	BACH1	[activation]
22936	SHC1	PTPN6	[activation]
22937	GADD45G	RBM48	[activation]
22938	APP	ARHGEF5	[activation]
22939	DDIT3	PICALM	[activation]
22940	MCM7	NFKBIA	[activation]
22941	ARL2	VCAM1	[activation]
22942	NOX5	UBE2I	[activation]
22943	LARS	DDA1	[activation]
22944	SMAD6	SMURF1	[inhibition]
22945	YWHAZ	MAP2K5	[activation]
22946	MLH1	PMS2	[activation]
22947	SNRPA1	CASP4	[activation]
22948	RASSF1	GABARAPL2	[inhibition]
22949	MLST8	DEPTOR	[activation;inhibition]
22950	CCDC57	LINC00526	[activation]
22951	APP	ASS1	[activation]
22952	BCL2L1	ZNF219	[activation]

22953	FLNA	RALA	[activation]
22954	CCNG1	PNMA1	[activation]
22955	MET	DCN	[activation]
22956	ZC3HAV1	AURKB	[activation]
22957	SMURF1	PRPF31	[inhibition]
22958	ANAPC1	PAXIP1	[activation]
22959	BMX	HSP90AA1	[activation]
22960	ITGA4	IQGAP1	[activation]
22961	PIKFYVE	VAC14	[activation]
22962	CAV1	PTPRF	[activation]
22963	CREBBP	PAX5	[activation]
22964	EFNB2	PTPN13	[activation]
22965	SH3GL3	LRRK2	[activation]
22966	SRPK1	C16orf78	[activation]
22967	NUMB	PRKCH	[activation]
22968	UNC93B1	FASTKD5	[activation]
22969	TWF2	ERC1	[activation]
22970	E2F1	CSNK1A1	[activation]
22971	NCAM2	MYB	[activation]
22972	ARF6	GLS	[activation]
22973	MYC	CDK6	[inhibition]
22974	AXIN1	PRMT1	[activation]
22975	FBXW11	ATF4	[activation]
22976	SRPK3	RSL1D1	[activation]
22977	ENDOV	PRPSAP2	[activation]
22978	STK3	FAM9B	[activation]
22979	NEK6	NCAPD2	[activation]
22980	PTPN1	PRKCG	[activation]
22981	SYK	CD3E	[activation]
22982	PAXIP1	INTS7	[activation]
22983	RAP1A	RASA3	[activation]
22984	HLX	HDAC1	[activation]
22985	LATS1	STK11	[activation]
22986	SRSF3	EIF4A3	[activation]
22987	ELK1	EP300	[activation]
22988	UBL5	ITGA4	[activation]
22989	CCL5	APC	[activation]
22990	EP300	PAK2	[activation]
22991	TFEB	SRPK1	[activation]
22992	RAD50	BARD1	[activation]
22993	RANBP2	OBSL1	[activation]
22994	FGFR2	BEX2	[activation]
22995	RAC1	ARHGAP31	[activation]
22996	CSF2RA	CSF2	[activation]
22997	HSD17B4	MAPK8	[activation]
22998	NFKBIA	MTOR	[activation]
22999	HIST1H3A	IGSF8	[activation]
23000	SIGLEC14	TYROBP	[activation]
23001	PTEN	GFRA2	[activation]
23002	ARPC3	OTUD5	[activation]
23003	STARD9	CDK5RAP2	[activation]
23004	GNB1	GNG13	[activation]
23005	ITGA4	MYL12B	[activation]
23006	CREBBP	MYC	[activation]
23007	RHOB	ARHGEF1	[activation]
23008	MTMR10	SMAD5	[activation;inhibition]
23009	MKI67	FYN	[activation]
23010	PIN1	DAB2	[activation]
23011	FOXO3	MAPK6	[activation;inhibition]
23012	EXPH5	APC	[inhibition]
23013	ULK1	USP10	[inhibition]
23014	WASF1	IL8	[activation]
23015	E2F1	ASH2L	[activation]
23016	CSNK1D	MAPT	[activation]
23017	PRMT3	EGFR	[activation]
23018	CD84	PTPN11	[activation]
23019	TNIP2	APP	[activation]
23020	ARPC5	NR3C1	[activation]
23021	TBX1	TERF2	[activation]
23022	GNB1	NOL11	[activation]
23023	MYC	USP9X	[activation]
23024	STX4	SNAP25	[activation]
23025	PES1	ESR2	[activation]
23026	PRR5L	MAPKAP1	[activation]
23027	CSN2	AURKA	[activation]
23028	MAP3K3	BIRC2	[activation;inhibition]

23029	ARRB1	MDM2	[activation]
23030	STRA13	MDM2	[activation]
23031	BTF3L4	TXLNA	[activation]
23032	PFN1	ITGA4	[activation]
23033	VAV1	SLA	[activation]
23034	HSPA1L	PCK1	[activation]
23035	APEH	UBA5	[activation]
23036	ADAM10	EFNA2	[activation]
23037	PTEN	CDC27	[activation]
23038	YWHAG	LTB4R	[activation]
23039	PUF60	ILK	[activation]
23040	EPOR	BTRC	[activation]
23041	DNAJC5	PRKACA	[inhibition]
23042	GLIPR2	CAV1	[activation]
23043	PRKCB	BTK	[activation]
23044	CRHR1	GNAI1	[activation]
23045	EGFR	IRS4	[activation]
23046	C3	CFHR4	[activation;inhibition]
23047	RAP1B	PDHB	[activation]
23048	GZMA	HDC	[activation]
23049	ITK	SMAD4	[activation]
23050	THBS1	APP	[activation]
23051	LTBR	TNF	[activation]
23052	CASP3	MAP3K14	[activation]
23053	VCAM1	GP1BB	[activation]
23054	BLNK	PPP2R2C	[activation]
23055	GAPDH	OSMR	[activation]
23056	DDX5	SRPK1	[activation]
23057	FRS3	PTPN11	[activation;inhibition]
23058	ERBB3	TNS3	[activation]
23059	CCND1	NXF1	[activation]
23060	NFKBIA	SKP1	[inhibition]
23061	RAPGEF2	STOM	[activation]
23062	SFRP4	AURKA	[activation]
23063	ACKR2	APP	[activation]
23064	ESRRA	MAP2K5	[activation]
23065	RUNX1	TLE2	[activation]
23066	PPP6R2	AURKA	[activation]
23067	MAPK3	STAB2	[activation]
23068	FYN	FYB	[activation]
23069	HSP90AB1	NFKB1	[activation]
23070	ADRBK1	MAPK3	[activation]
23071	ARRB2	AKT1	[activation]
23072	SRC	LCP2	[activation]
23073	ACTB	LYN	[activation]
23074	ACTN1	NR1H3	[activation]
23075	PPP2R2A	MLH1	[activation]
23076	ESR2	IL24	[activation]
23077	EDN3	KEL	[activation]
23078	TFF1	AKT1	[activation]
23079	MTOR	IKBKB	[activation]
23080	RASA1	LCK	[activation]
23081	HMGN1	PRKACG	[activation]
23082	NFKBIA	TUBB4A	[activation]
23083	CD4	CD53	[activation]
23084	TIRAP	EIF2AK2	[activation]
23085	PAK4	SFN	[activation]
23086	YWHAZ	BCAP31	[activation]
23087	APP	IL18RAP	[activation]
23088	SMAD3	DDX4	[activation]
23089	NSG2	APOC1	[inhibition]
23090	MYEF2	MAPK14	[activation]
23091	DVL2	SMAD3	[activation]
23092	CD81	GLP1R	[activation]
23093	RAC1	BAIAP2	[activation]
23094	TRAF6	MAST2	[activation]
23095	MOS	KIFC3	[activation]
23096	WNK1	FAM96B	[activation]
23097	CDKN2A	CDC7	[activation]
23098	RAB7A	ATG5	[activation]
23099	TP53	MRPS9	[activation]
23100	EIF4A1	EIF4G3	[activation]
23101	GABARAPL2	TSR2	[activation]
23102	EFCAB4B	STIM1	[activation]
23103	JAM2	JAM3	[activation]
23104	USP28	TP53	[activation]

23105	GDF2	ACVR2A	[activation]
23106	ARAF	RRAS	[activation]
23107	CDC5L	EIF4A3	[activation]
23108	SPATA5L1	NXF1	[activation]
23109	EZH2	POLA2	[activation]
23110	NSF	DRD2	[activation]
23111	PRKAA1	KRT40	[inhibition]
23112	CBL	CSF1R	[activation]
23113	GDF6	SMAD5	[activation]
23114	GSN	VASP	[activation]
23115	UBASH3B	SHC1	[activation]
23116	PPP2CA	CFL1	[activation]
23117	TFRC	RPA3	[activation]
23118	OSGIN1	ATM	[activation]
23119	GAPDH	BLK	[activation]
23120	KRTAP19-7	CAMK2B	[inhibition]
23121	OTX1	KCNK16	[activation]
23122	RPS6KA1	ACACA	[activation;inhibition]
23123	GNAQ	CRHR1	[activation]
23124	NDEL1	CDC42	[activation]
23125	IRF3	SMAD4	[inhibition]
23126	PIK3R1	MAP3K11	[activation]
23127	SH2B2	GRB2	[activation]
23128	DOCK10	RHOJ	[activation]
23129	EFEMP2	LINGO1	[activation]
23130	SMAD3	GIT2	[inhibition]
23131	GSK3B	MITF	[activation]
23132	HSP90AB1	C20orf194	[activation]
23133	MAPK6	FBXL16	[activation;inhibition]
23134	C1R	SERPING1	[inhibition]
23135	CASP3	HSPD1	[activation]
23136	SLC25A6	MAX	[activation]
23137	PAEP	A2M	[inhibition]
23138	PPP2CA	MDC1	[activation]
23139	BIRC5	CDK4	[activation]
23140	PIK3CA	UBC	[activation]
23141	CYTH3	IPCEF1	[activation]
23142	OSGEP	CRIP2	[activation]
23143	NCOA2	OPN1LW	[activation]
23144	PPP1CA	WNK1	[activation]
23145	TMEM102	YWHAG	[activation]
23146	IL15RA	IL2RG	[inhibition]
23147	PLXNA2	KIAA1199	[activation]
23148	RAD21	HNRNPH2	[activation]
23149	ESR1	SMARCB1	[activation]
23150	KMT2B	ANXA7	[activation]
23151	TTLL3	DAPK1	[inhibition]
23152	STRADB	XIAP	[activation]
23153	RPS6KA1	PPM1G	[activation]
23154	PGR	TAF1B	[activation]
23155	CREB1	SMARCA5	[activation]
23156	TIGAR	APP	[activation]
23157	XPO1	CHEK1	[activation]
23158	EIF4E	EIF4G2	[inhibition]
23159	HIF1A	EP300	[activation]
23160	SUMO1	PPARG	[activation]
23161	SH2D3A	ERBB2	[activation]
23162	TRAF2	AMOTL2	[activation]
23163	PRKCE	SNCA	[activation]
23164	FASLG	PIN1	[inhibition]
23165	PRNP	SCNM1	[activation]
23166	TP53	SMARCAL1	[activation]
23167	AURKB	HIST3H3	[activation]
23168	SYK	MYD88	[activation;inhibition]
23169	STAT1	FLT1	[activation]
23170	HSP90AB1	WASL	[activation]
23171	SCAMP5	SNAP23	[activation]
23172	SPAG9	MAP2K4	[inhibition]
23173	ZAP70	SH3BP2	[activation]
23174	DDX50	ESR2	[activation]
23175	PRKRA	RABEPK	[activation]
23176	LCP2	FCGR1A	[activation]
23177	IL18R1	IL1RAP	[activation]
23178	TP53	CEBPZ	[activation]
23179	AURKB	CDC73	[activation]
23180	LCP2	SHB	[activation]

23181	CCNK	DVL3	[activation]
23182	WDR18	ESR1	[activation]
23183	CORT	SSTR4	[activation]
23184	CCNA2	CDC6	[activation]
23185	MAP1LC3B	TGM3	[activation]
23186	ICAM1	MSN	[activation]
23187	EPHA7	ZNF267	[activation]
23188	RAG1	RUNX1	[activation]
23189	SRPK2	MRPS6	[activation]
23190	MAGED1	DAB1	[activation]
23191	TRAF3	TNFRSF1B	[activation]
23192	KDM3A	RPS6KA5	[activation]
23193	SMAD1	ECSIT	[activation]
23194	MST4	PPP2R1A	[inhibition]
23195	EIF3A	EIF4A1	[activation]
23196	SYK	FCRL3	[activation]
23197	HIST1H2BC	ITGA4	[activation]
23198	HSPB1	NEK10	[activation]
23199	EGFR	LINGO1	[activation]
23200	DDX21	HLA-B	[activation]
23201	VCAM1	KPNB1	[activation]
23202	MAFK	TP53	[activation]
23203	TNFRSF13B	IRAK4	[activation]
23204	ROBO2	OLFM2	[activation]
23205	SCGB3A1	FGFR4	[activation]
23206	APH1A	ATF4	[activation]
23207	GNB4	NUDC	[activation]
23208	C17orf62	ETS1	[activation]
23209	YWHAZ	GSK3B	[activation]
23210	RFWD2	JUNB	[activation]
23211	IL1B	CASP4	[activation]
23212	CSF2RA	PIK3R1	[activation]
23213	STK11	XPA	[activation]
23214	ZFYVE1	BCL2L1	[activation]
23215	SUMO1	EIF2S3	[activation]
23216	RPA3	MYO1B	[activation]
23217	SOX15	BHLHE40	[activation]
23218	RALGDS	RIN1	[activation]
23219	EGLN2	FAM46A	[activation]
23220	CD4	CD28	[activation]
23221	IL4	IL13RA2	[activation]
23222	GABARAPL1	IGKV1-5	[activation]
23223	CLEC7A	TLR2	[activation]
23224	PIK3R1	PTPN6	[activation;inhibition]
23225	CEBPE	RB1	[activation;inhibition]
23226	RAC1	NGEF	[activation]
23227	EEF1D	LRRK2	[activation]
23228	CEBPB	HDAC9	[activation]
23229	IRAK1	CCDC8	[activation;inhibition]
23230	FST	MEST	[inhibition]
23231	HIST1H1B	RPS6KA5	[activation]
23232	TOP2A	MAPK1	[activation]
23233	LZTR1	SREBF2	[activation]
23234	TNFRSF11A	TMPRSS15	[activation]
23235	CHRM1	ARRB1	[activation]
23236	PBX2	NUP62	[activation]
23237	PPARG	NCOA1	[activation]
23238	CDKN1B	YWHAB	[inhibition]
23239	ABI2	MRFAP1L1	[activation]
23240	ANKFY1	RHOD	[activation]
23241	ENOX1	NIF3L1	[activation]
23242	CDKN1A	WDR73	[activation;inhibition]
23243	MYC	MAP3K7	[activation]
23244	TRIP10	CREBBP	[activation]
23245	SMG1	PRKCI	[activation]
23246	ARPC2	UBE2Q2	[activation]
23247	MDM2	MYL6	[activation]
23248	SRC	PPP2R4	[activation]
23249	LRP1	MMP16	[activation]
23250	MAPK11	ARHGAP12	[activation]
23251	CASP6	RB1	[activation]
23252	PRKCQ	GLRX3	[activation]
23253	GABARAP	PPA1	[activation]
23254	MYC	PRR11	[activation]
23255	TP53	MNDA	[activation]
23256	NXF1	CSNK2B	[activation]

23257	PFAS	SGK1	[activation]
23258	GPC4	ACP5	[activation]
23259	PAK4	APP	[activation]
23260	ANTXR2	SMAD2	[activation]
23261	CREB1	SRF	[activation]
23262	STK3	EIF4EBP1	[activation]
23263	RAN	XPO4	[activation]
23264	ICK	NUP188	[activation]
23265	IGJ	STK4	[activation]
23266	EZH2	AKT1	[activation]
23267	SPP1	CYB5R3	[activation]
23268	RAN	PTBP3	[activation]
23269	PPP1R14A	CSNK1E	[activation]
23270	MTNR1B	GNAI1	[activation]
23271	MAPRE2	CDK2	[activation]
23272	KIR2DL2	HLA-C	[activation]
23273	RAB5A	PIK3CB	[activation]
23274	MAPK1	GATA1	[activation]
23275	SLC9A3R2	PODXL	[activation]
23276	TJP1	ACTB	[activation]
23277	CAMK2G	TH	[activation]
23278	RABL5	MAGEB2	[activation]
23279	APITD1	RPA1	[activation]
23280	ATXN10	GLS	[activation]
23281	BMP6	CHRDL2	[activation]
23282	CCDC170	CCNH	[activation]
23283	ECT2	MAPK1	[activation]
23284	CREB1	SREBF2	[activation]
23285	LRRK2	SLC25A11	[activation]
23286	TTR	ATF4	[activation]
23287	WDR73	ANXA7	[activation]
23288	MYL12A	CD4	[activation]
23289	ANAPC7	E2F1	[activation]
23290	MCM7	RAD21	[activation]
23291	PIK3CA	SMAD3	[activation]
23292	HIST1H1B	PRKCD	[activation]
23293	TP53	PRMT3	[activation]
23294	STAT3	SUPT20H	[activation]
23295	PPFIBP1	YWHAB	[activation]
23296	SRC	ASAP2	[activation]
23297	STK3	PLK1	[activation]
23298	GRK4	FSHR	[activation]
23299	EIF4EBP1	CHTF8	[inhibition]
23300	SHC1	CEACAM1	[activation]
23301	MED1	HNF4A	[activation]
23302	SFN	PKP3	[activation]
23303	TRAF6	CAPZA1	[activation]
23304	CCDC85A	STMN2	[inhibition]
23305	STAT3	CAPNS1	[activation]
23306	GNGT2	GNB2	[activation]
23307	TNIP3	TBK1	[inhibition]
23308	RALGDS	KRAS	[activation]
23309	PAXIP1	NME4	[activation]
23310	AURKA	NEDD9	[activation]
23311	TBRG4	MAL2	[activation]
23312	MET	SH2D3C	[activation]
23313	HSPH1	BUB1B	[inhibition]
23314	TWIST1	NEIL3	[activation]
23315	MCM3	MCM2	[activation]
23316	LAT	VAV1	[activation]
23317	ACTB	TAGLN2	[activation]
23318	PPFIA1	PPP2CA	[inhibition]
23319	ENO3	TTN	[activation]
23320	USHBP1	CCDC146	[activation]
23321	SPP1	PLD3	[activation]
23322	BCR	MLLT4	[activation]
23323	HIST1H1C	CTNNB1	[activation]
23324	IL2	SHC1	[activation]
23325	SRPK2	CDK13	[activation]
23326	PCED1A	TNFRSF14	[activation]
23327	LRRK2	YWHAH	[activation]
23328	BTN3A3	ARL8B	[activation]
23329	NUP98	APC	[activation]
23330	MAP1LC3B	ARFGAP1	[activation]
23331	FLNC	MLH1	[activation]
23332	MYC	PRKDC	[activation]

23333	ZKSCAN8	SRPK1	[activation]
23334	CNTNAP2	MACF1	[activation]
23335	MRPL4	ICT1	[activation]
23336	DAB1	DAZAP2	[activation]
23337	UBC	HSF1	[activation]
23338	PFKFB2	YWHAB	[activation]
23339	BMP7	UBC	[activation]
23340	PRKDC	ATM	[activation]
23341	CCND3	NPDC1	[inhibition]
23342	HSP90AA1	FES	[activation]
23343	FLT3	SYK	[activation]
23344	CTNNB1	NUP153	[activation]
23345	NUP50	LRRK2	[activation]
23346	NOS3	H3F3A	[activation]
23347	ACVR2A	GDF9	[activation]
23348	SIRT1	CACNA1C	[activation]
23349	ATPIF1	BLK	[activation]
23350	E2F1	ATR	[activation]
23351	DCC	APPL1	[activation]
23352	SFN	RALGPS2	[activation]
23353	MAP3K3	NFKBIA	[activation]
23354	GRB2	DNAJA3	[activation]
23355	C1orf109	CCDC36	[activation]
23356	SOCS3	BIK	[activation;inhibition]
23357	IL9R	JAK1	[activation]
23358	RANBP2	TOP2A	[activation]
23359	PYCARD	NLRP2	[inhibition]
23360	PFKFB3	ASB12	[activation]
23361	GRB2	ELK1	[activation]
23362	CSNK1G2	NCK1	[activation]
23363	SRPK2	HOMER3	[activation]
23364	HHEX	TLE1	[activation]
23365	NGB	GNAI1	[activation]
23366	NBN	RECQL5	[activation]
23367	IL1RAP	SMAD2	[inhibition]
23368	MEMO1	ERBB2	[activation]
23369	PROSER2	ACTB	[activation]
23370	VAPB	PRKACA	[activation]
23371	WASF3	PIK3R2	[activation]
23372	PML	CSNK2A3	[inhibition]
23373	PTBP3	OGT	[activation]
23374	RFC1	NOTCH1	[activation]
23375	FBXL3	BTG1	[inhibition]
23376	HCK	ADAM22	[activation]
23377	CD4	NCL	[activation]
23378	HIST1H4A	PYHIN1	[activation;inhibition]
23379	USP20	PLEKHA7	[activation]
23380	MDM2	CHRM3	[activation]
23381	NR3C1	PIAS2	[activation]
23382	TBC1D3B	HSPB1	[inhibition]
23383	TIAM1	CSNK1A1	[activation]
23384	TLE1	PROP1	[activation]
23385	PHGDH	VCAM1	[activation]
23386	DDX24	EP300	[activation]
23387	SIX2	APP	[activation]
23388	SLC25A6	LRRK2	[activation]
23389	LEPR	RNF41	[activation]
23390	HSD17B8	RAD21	[activation]
23391	ANAPC1	SMARCAD1	[activation]
23392	SAMD9L	EEA1	[activation]
23393	MLH1	PARP12	[activation]
23394	DNPEP	RBL1	[inhibition]
23395	CTDSP2	AR	[activation]
23396	SHANK3	ARHGEF7	[activation]
23397	ARGLU1	SRPK2	[activation]
23398	FANCB	FAAP100	[activation]
23399	AKT2	SNAI1	[activation;inhibition]
23400	NTRK1	NGFR	[activation]
23401	FSHR	FOXO1	[activation]
23402	SASH1	SUV39H2	[activation]
23403	OSBPL10	MMP2	[inhibition]
23404	STK11	GNPDA1	[activation]
23405	APP	GOSR2	[activation]
23406	CFL1	CD81	[activation]
23407	MDM2	IDH3A	[activation]
23408	YWHAQ	WNK4	[activation]

23409	KPNB1	BARD1	[activation]
23410	AVIL	CRK	[activation]
23411	BLNK	DOK3	[activation]
23412	C8orf33	FYN	[activation]
23413	IL32	PRKCD	[activation]
23414	EGFR	CAMLG	[activation]
23415	FASN	MAP3K3	[activation]
23416	GRB2	ID4	[activation]
23417	LIMK1	YWHAZ	[activation;inhibition]
23418	SPECC1	GRB2	[activation]
23419	PSTPIP1	WASF1	[inhibition]
23420	TIMP2	SNCG	[activation]
23421	FGB	CDKN1A	[activation]
23422	PTPRF	SKIL	[inhibition]
23423	ARRB1	PPM1B	[activation]
23424	RPA2	APITD1	[activation]
23425	PRKCD	NUMB	[activation]
23426	EPHA2	SLA	[activation]
23427	EIF1B	EIF1AX	[activation]
23428	DVL3	VANGL1	[activation]
23429	EIF2AK2	TOLLIP	[activation]
23430	ABL1	DOK1	[activation]
23431	GNAS	LHB	[activation]
23432	GRK6	SNCG	[inhibition]
23433	GABARAPL1	JUP	[activation]
23434	HMOX1	BLVRB	[activation]
23435	HSP90AA1	H2AFX	[activation]
23436	ABL1	DDX5	[activation]
23437	CSNK1E	PRMT5	[activation]
23438	TNFRSF8	ALK	[activation]
23439	ZFYVE9	PPP2R1A	[activation]
23440	MTIF2	ALK	[activation]
23441	HEMGN	KRAS	[activation]
23442	TBX5	GATA4	[activation]
23443	PPP1CC	MAX	[inhibition]
23444	PER3	ATM	[activation]
23445	UGT1A6	UGT1A1	[activation]
23446	HNRNPA0	MYC	[activation]
23447	BMP3	COL4A1	[activation]
23448	PTEN	DBF4B	[activation]
23449	GJB2	CAV1	[activation]
23450	PPP1R14B	ROCK2	[activation]
23451	INADL	PAX6	[activation]
23452	YWHAQ	MFF	[activation]
23453	DAPK2	APP	[activation]
23454	KATNB1	NDEL1	[activation]
23455	DAPK2	FAM9B	[activation]
23456	NXF1	TXLNA	[activation]
23457	PPP2R1A	EIF1B	[inhibition]
23458	PRR5	RICTOR	[activation]
23459	PIK3CG	BECN1	[activation]
23460	MAPK1	DUSP7	[inhibition]
23461	ACVR1	ACVR1B	[activation]
23462	P4HB	F3	[activation]
23463	RAB5A	ZFYVE20	[activation]
23464	RB1	CASP9	[activation;inhibition]
23465	TRAIP	CYLD	[inhibition]
23466	HSPA5	RIPK1	[activation]
23467	RAC1	PRMT6	[activation]
23468	YWHAZ	HIST1H1C	[activation]
23469	LIMK2	CDC42BPA	[activation]
23470	HSPB2	CRYAA	[activation]
23471	HSPA8	RIPK1	[activation]
23472	ACTB	ABRA	[activation]
23473	TNC	CNTN1	[activation]
23474	NXF1	ZBED8	[activation]
23475	TRADD	HSP90AB1	[activation]
23476	DUSP3	SYK	[inhibition]
23477	DLX4	FYN	[activation]
23478	OLIG2	CUL3	[activation]
23479	ABL1	PRKCZ	[activation]
23480	NCK1	EIF2B2	[activation]
23481	PRKCZ	NUMB	[activation]
23482	NUMA1	NXF1	[activation]
23483	MYC	SARS	[activation]
23484	IL6	ZBTB16	[activation;inhibition]

23485	IRS4	YWHAG	[activation]
23486	CXCR2	ADRA1A	[activation]
23487	CDC20	RASSF1	[inhibition]
23488	KIFC3	ABI2	[activation]
23489	SEMA4D	PTPRC	[activation]
23490	CDK18	CCNK	[activation]
23491	TERF1	TRIOBP	[activation]
23492	STX19	STXBP1	[activation]
23493	FMNL1	GAS7	[activation]
23494	POU2F1	STAT5B	[activation]
23495	TRAF2	FBXO28	[activation]
23496	TRIB3	LTBP4	[inhibition]
23497	RPA1	RPRD1B	[activation]
23498	REN	KCTD15	[activation]
23499	ACVR1B	IGSF1	[activation]
23500	PTPN12	CDH2	[activation]
23501	SERINC3	VDAC3	[inhibition]
23502	AR	SHE	[activation]
23503	MCM7	CCL2	[activation]
23504	CTNNB1	PARD3	[activation]
23505	ATAD3B	MYC	[activation]
23506	POTEE	RELA	[activation]
23507	EYA1	FBXW7	[activation]
23508	NFKB2	NFKBIA	[activation]
23509	RAP2B	RUNDC3A	[activation]
23510	DLX4	PLCG1	[activation]
23511	SRC	DLGAP2	[activation]
23512	LCAT	APOE	[activation]
23513	TRAF6	RANBP1	[activation]
23514	THAP8	TP53	[activation]
23515	RANBP9	APC	[activation]
23516	ZAP70	PRLR	[activation;inhibition]
23517	GH1	JUP	[inhibition]
23518	ATG101	ULK1	[inhibition]
23519	VCAM1	HIST1H4A	[activation]
23520	MAPK14	CBL	[activation]
23521	APOH	CLEC4G	[activation]
23522	EZH2	RARA	[activation]
23523	FASLG	NCF1	[activation]
23524	SMAD7	AXIN1	[activation]
23525	WNK1	YWHAQ	[activation]
23526	OBSL1	MACF1	[activation]
23527	ERBB2	IGF1R	[activation]
23528	PPP2CB	STK11	[activation]
23529	PACSIN3	LGALS13	[activation]
23530	TRAF6	SIGIRR	[activation]
23531	PROC	SERPINB6	[inhibition]
23532	MAP1LC3B	CASP8	[activation]
23533	DNM1L	UBE2H	[activation]
23534	UPF2	MYB	[activation]
23535	GARS	YWHAZ	[activation]
23536	C1orf63	SRPK1	[activation]
23537	FTSJ3	SRPK3	[activation]
23538	WASF1	PFN2	[activation]
23539	BMX	ITK	[activation]
23540	CYB5R2	CDC23	[activation]
23541	UBXN11	TRAF2	[activation]
23542	SMURF2	RASD2	[activation]
23543	SLK	GABARAP	[activation]
23544	PKN1	PLD1	[activation]
23545	CCDC120	CEP170P1	[activation]
23546	BCL2L1	BECN1	[activation]
23547	CHEK1	SMURF1	[inhibition]
23548	PLG	F2R	[activation]
23549	NFATC2	IQGAP1	[activation]
23550	TBC1D4	AURKB	[activation]
23551	MKRN1	TP53	[activation]
23552	CDC5L	NBN	[activation]
23553	C16orf45	RAPGEF4	[activation;inhibition]
23554	PRKAG1	PRKAA1	[inhibition]
23555	MYC	MATN4	[activation]
23556	ITGAV	F2R	[activation]
23557	PPP1CC	PRR16	[inhibition]
23558	SKAP1	PTPRC	[activation]
23559	ATG5	HTT	[activation]
23560	MYO18A	NXF1	[activation]

23561	CHN2	TGFBR1	[activation]
23562	SH3KBP1	SRGAP1	[activation]
23563	ITGA1	ITGB1	[activation]
23564	TFF1	XRCC3	[activation]
23565	HRG	FCGR1A	[activation]
23566	TMEM150A	NOTCH2NL	[activation]
23567	PCK1	HLA-C	[activation]
23568	YWHAE	DDX21	[activation]
23569	MAGEH1	NGFR	[activation;inhibition]
23570	DYRK1A	KIAA0232	[activation]
23571	PTPN11	SOCS1	[inhibition]
23572	MAPKAPK3	BMI1	[activation]
23573	LGALS4	RRAS2	[activation]
23574	VTI1B	STX4	[activation]
23575	CD4	CD44	[activation]
23576	LIPC	LRP1	[activation]
23577	TNFSF13B	TNFSF12	[activation]
23578	PRKCI	APP	[activation]
23579	STK4	MAP1S	[activation]
23580	BTK	CAV1	[activation]
23581	ETV4	SRF	[activation]
23582	PLCG1	SOS1	[activation]
23583	CDK4	UBTF	[activation]
23584	YAP1	CPVL	[activation]
23585	EGFR	SOCS3	[activation]
23586	ACTR2	EIF1B	[activation]
23587	HTR2A	MAP1A	[activation]
23588	POLD3	APP	[activation]
23589	UBTD1	MRFAP1L1	[activation]
23590	NCK1	ERBB4	[activation]
23591	SLC25A6	TMEM62	[activation]
23592	TNKS	APC	[activation]
23593	UBB	MAPT	[activation]
23594	E2F1	MNAT1	[activation]
23595	DDX17	DDX5	[activation]
23596	ERBB2	HLA-A	[activation]
23597	NRXN3	MLLT4	[activation]
23598	HTR1F	CAV1	[activation]
23599	APCS	GRB2	[activation]
23600	YWHAB	CAMKK1	[activation]
23601	TMBIM6	VTI1A	[activation]
23602	NFKB1	NOTCH1	[activation]
23603	OS9	SREBF2	[activation]
23604	ANXA1	KLHL23	[activation]
23605	POU1F1	MED1	[activation]
23606	MCL1	NXF1	[activation]
23607	LILRB1	HLA-F	[activation]
23608	APC	RP1	[inhibition]
23609	RAP1B	KMT2B	[activation]
23610	LY6D	PKIB	[inhibition]
23611	NXF1	ATP6AP2	[activation]
23612	EGR2	UBE2I	[activation]
23613	CDC5L	PRKDC	[activation]
23614	ARRB2	PFKFB3	[activation]
23615	STK4	TP53	[activation]
23616	IFI16	NOTCH1	[activation]
23617	WNK1	BAG3	[activation]
23618	PTPN6	PTK2B	[activation]
23619	NXF1	HNRNPAB	[activation]
23620	MYOD1	ESR2	[activation]
23621	TCAP	ENO3	[inhibition]
23622	CTNNA1	CDC5L	[activation]
23623	VAMP3	CCDC8	[activation]
23624	YWHAB	CYLD	[inhibition]
23625	RIPK2	GXYLT1	[activation]
23626	CALCA	ATP2B4	[activation]
23627	HBG2	ITGA4	[activation]
23628	STIP1	MOS	[activation]
23629	WHSC1L1	ATM	[activation]
23630	TSSC1	DEGS1	[activation]
23631	AURKA	PPP6C	[activation]
23632	SEC16A	LRRK2	[activation]
23633	FTO	FTSJ1	[activation]
23634	CDK1	CDKN3	[activation]
23635	POLE4	POLE2	[activation]
23636	HSPA1A	TP53	[activation]

23637	CUL1	CCND1	[inhibition]
23638	EIF2B4	EPB41L3	[activation]
23639	TNF	POU2F1	[activation]
23640	CLSTN1	PRNP	[activation]
23641	CUX1	HMGB1	[activation]
23642	NRIP1	PPARG	[activation]
23643	RAD50	RRM2B	[activation]
23644	SRPK1	ERBB2	[activation]
23645	FANCG	FANCA	[activation]
23646	SRPK2	CHD2	[activation]
23647	H3F3A	ASB2	[activation]
23648	MPP5	YAP1	[inhibition]
23649	PELI3	IRAK4	[activation;inhibition]
23650	PITX2	MEF2A	[activation]
23651	ESR1	C20orf197	[activation]
23652	PXN	PTPRT	[activation]
23653	MAP3K7	STRADB	[activation]
23654	HSP90AA1	IKBKB	[activation]
23655	TERT	SRC	[activation]
23656	PPP2CA	ADCY8	[inhibition]
23657	NFKBIA	FBXW11	[inhibition]
23658	GSK3B	IVNS1ABP	[inhibition]
23659	PRKACA	AKAP14	[activation]
23660	VTI1A	STX7	[activation]
23661	ADRA2A	GNB1	[activation]
23662	IGFBP5	VTN	[inhibition]
23663	CYCS	POR	[activation]
23664	SCN8A	MAPK14	[activation]
23665	PCSK1	PCSK1N	[activation;inhibition]
23666	EGR1	VDR	[activation]
23667	ACKR4	CCL19	[activation]
23668	CTBP1	CREBBP	[activation]
23669	TYK2	NXF1	[activation]
23670	FYN	WIPF1	[activation]
23671	BUB3	EXOC3	[inhibition]
23672	CDK7	ESR1	[activation]
23673	IL23A	IL12B	[activation]
23674	PIK3R1	DLX4	[activation]
23675	TGFB1I1	ITGA4	[activation]
23676	TIRAP	NF1	[activation]
23677	MAPK3	PPP1CA	[activation;inhibition]
23678	CD81	MCM5	[activation]
23679	RAB11B	RPA3	[activation]
23680	CD19	CR2	[activation]
23681	GRB2	RPS25	[activation]
23682	CHUK	SHARPIN	[activation]
23683	ADAM10	SH3D19	[activation]
23684	ZFYVE20	RAB5C	[activation]
23685	APH1A	PSENEN	[activation]
23686	LCK	IFNAR1	[activation]
23687	ESR2	MYL6	[activation]
23688	BOD1L1	VAV2	[activation]
23689	HSP90AB1	LIMK2	[activation]
23690	LGR4	PRKDC	[activation]
23691	NXF1	TP53BP1	[activation]
23692	NXF1	C19orf10	[activation]
23693	PLIN2	ARF1	[activation]
23694	MYO1C	BAZ1B	[activation]
23695	MYC	ADIPOR1	[activation]
23696	CACNB4	SYT1	[activation]
23697	KPNB1	RAC1	[activation]
23698	NUP62	CCDC121	[activation]
23699	HSPA5	TRAF6	[activation]
23700	HRG	C1QA	[activation]
23701	SEH1L	RPA1	[activation]
23702	TNFRSF14	NRIP1	[activation]
23703	DRD2	KCNJ9	[activation]
23704	GRID2	GRIA2	[activation]
23705	NSUN2	EGFR	[activation]
23706	BARD1	POLR2A	[activation]
23707	TNFRSF25	TRADD	[activation]
23708	VCAM1	MIF	[activation]
23709	ATF2	HSP90AA1	[activation]
23710	MAPK4	CALML3	[activation;inhibition]
23711	FGF5	EGF	[activation]
23712	ACAP1	SRC	[activation]

23713	DLGAP1	SRC	[activation]
23714	PIAS2	DDX39B	[activation]
23715	MOB1A	DOCK6	[activation]
23716	GRB2	FYB	[activation]
23717	PCDH18	ATG5	[activation]
23718	CAV1	EDNRB	[activation]
23719	MYO1C	EGFR	[activation]
23720	MAP2K1	MLH3	[activation]
23721	MDC1	WHSC1	[activation]
23722	CRTC2	CREBBP	[activation]
23723	CD82	LRP1	[activation]
23724	CDK4	CDK5R1	[activation]
23725	EZR	SGK1	[activation]
23726	GNAI2	TRIP6	[activation]
23727	CCNB2	TGFBR2	[activation]
23728	WFDC5	STAG1	[inhibition]
23729	YWHAQ	DAB2IP	[inhibition]
23730	YWHAG	NUMBL	[inhibition]
23731	NFKB2	PPP2R1A	[inhibition]
23732	PRKAG2	PRKAA2	[inhibition]
23733	GRB2	PTPRN2	[activation]
23734	KAT2B	GLI1	[activation]
23735	AGR3	NUP62	[activation]
23736	MCM7	MCM8	[activation]
23737	YWHAH	PAK4	[activation]
23738	GABRR2	GABRR1	[activation]
23739	RFC5	GABARAP	[activation]
23740	PBK	TP53	[activation]
23741	ITCH	TP53	[activation]
23742	KLHL38	USHBP1	[activation]
23743	LPHN1	APC	[activation]
23744	EP300	NCOA1	[activation]
23745	MOS	CCDC102B	[activation]
23746	TXNL4A	IL31RA	[activation]
23747	RAB5A	NME2	[activation]
23748	CDK5RAP3	CDKN2A	[activation;inhibition]
23749	GDF9	BMPR1B	[activation]
23750	LOX	MYC	[activation]
23751	MYO1C	PTP4A3	[activation]
23752	YWHAZ	CBLL1	[activation]
23753	CLEC4G	GEM	[activation]
23754	FAS	MET	[inhibition]
23755	NAB2	MAPK3	[activation]
23756	ATG5	UCHL1	[activation]
23757	PREX1	RICTOR	[activation]
23758	SHB	GRAP2	[activation]
23759	ABL1	RASA1	[activation]
23760	EIF4A1	MAPK6	[activation]
23761	CSK	RGS16	[activation;inhibition]
23762	GRB2	POLR2A	[activation]
23763	RNF19A	UBE2E2	[activation]
23764	OBSL1	CTTN	[inhibition]
23765	EPHB6	GRB2	[activation]
23766	CYCS	VCAM1	[activation]
23767	YWHAZ	EIF2S1	[activation]
23768	HNRNPA0	MTNR1B	[activation]
23769	FGA	HRG	[activation]
23770	MDM2	GNB2	[activation]
23771	APP	ATF4	[activation]
23772	DSTN	ISG15	[activation;inhibition]
23773	ITGB1	ADAM17	[activation]
23774	CCDC85B	SIX1	[activation]
23775	TULP3	KLF10	[activation]
23776	PXN	CEACAM1	[activation]
23777	MCF2L	RAC1	[activation]
23778	HSPB1	MTIF2	[activation]
23779	HLA-B	PDCD5	[activation]
23780	CMPK1	ATG5	[activation]
23781	CD48	CD2	[activation]
23782	HLA-B	PMPCB	[activation]
23783	SMURF1	LONRF3	[inhibition]
23784	PRPF38B	CTNNB1	[inhibition]
23785	BLK	CCDC33	[activation]
23786	BAX	SFN	[activation]
23787	ETS1	PRKDC	[activation]
23788	TP53	FBXO42	[activation]

23789	SMURF1	ACTB	[activation]
23790	FAM175B	GRB2	[activation]
23791	TRAIP	TBK1	[activation]
23792	BCAP31	VAMP1	[activation]
23793	HIST3H3	TBP	[activation]
23794	LTBP3	HBEGF	[activation]
23795	NOTCH1	VAV1	[activation]
23796	EIF2AK4	TGFBR1	[inhibition]
23797	NOTCH1	TRIM28	[activation]
23798	SUV420H2	TUB	[activation]
23799	YWHAB	VCAM1	[activation]
23800	FKBPL	NR1H3	[activation]
23801	TNFRSF1A	ARFGEF2	[activation]
23802	EIF3F	CDK11B	[activation]
23803	LIMS1	EHMT2	[activation]
23804	NGFRAP1	SMAD2	[activation]
23805	SLC25A6	CUTC	[activation]
23806	DNAL4	SCTR	[activation]
23807	CALM1	GRM3	[activation]
23808	CASP7	XIAP	[activation]
23809	APH1A	NXF1	[activation]
23810	F2	THBS1	[activation]
23811	ESR2	CTNNA2	[activation]
23812	RALGAPB	SRC	[activation]
23813	CSNK1A1	CSNK1D	[activation]
23814	EIF2S3L	CDC5L	[activation]
23815	FES	HSPA4	[activation]
23816	POM121C	APC	[inhibition]
23817	CDC25B	YWHAH	[activation]
23818	UFM1	DIABLO	[activation]
23819	BCL2L1	PLD3	[activation]
23820	NXN	PPP2CA	[activation]
23821	ARF6	EPPK1	[activation]
23822	TRAF6	DIABLO	[activation]
23823	FOXM1	RB1	[activation;inhibition]
23824	SYK	SH2D2A	[activation]
23825	TXNDC5	APP	[activation]
23826	PTPRB	MET	[activation;inhibition]
23827	CDK2	CNKSR2	[activation]
23828	STMN2	NGFR	[activation]
23829	ERCC2	EGFR	[activation]
23830	H2AFX	POLR2A	[activation]
23831	PARP2	CHRDL1	[activation]
23832	CDX2	HNF1A	[activation]
23833	CD300E	TYROBP	[activation]
23834	MAPK3	PTPRJ	[activation]
23835	PIAS1	TP53	[activation]
23836	CASP12	XIAP	[inhibition]
23837	CTNNB1	PECAM1	[activation]
23838	VCAM1	HNRNPA0	[activation]
23839	MAP4K4	PPP1CA	[activation]
23840	SYN1	PIK3R1	[activation]
23841	A1BG	CDKN1A	[activation;inhibition]
23842	CREBBP	JUN	[activation]
23843	HSPA5	APC	[inhibition]
23844	RICTOR	YWHAE	[activation]
23845	ELAVL1	CRKL	[activation]
23846	NUP214	G3BP1	[activation]
23847	MAPK10	PKMYT1	[inhibition]
23848	MITF	RPS6KA1	[activation]
23849	STK4	MAP2K4	[activation]
23850	NOD1	CARD6	[activation]
23851	ITGA2B	F2	[activation]
23852	WRAP73	OGT	[activation]
23853	ICT1	ERLIN2	[activation]
23854	SMAD9	SMAD2	[activation]
23855	CASP8	SERPINB5	[inhibition]
23856	GRB10	GHR	[activation]
23857	B2M	A2M	[inhibition]
23858	MYD88	MAL	[activation]
23859	TNFRSF14	CHD3	[activation]
23860	PTPN14	PTEN	[activation]
23861	MYC	SLIT2	[activation]
23862	CDH2	FBXO45	[activation]
23863	TWIST1	ETS2	[activation]
23864	MYO1C	SLX4	[activation]

23865 ANXA1 TLE3 [activation]
23866 MFAP1 HSPB1 [activation]
23867 SYK TLR9 [activation]
23868 TMPRSS15 PYCARD [inhibition]
23869 ZNF226 ATF4 [activation]
23870 EIF4E IRS1 [inhibition]
23871 CDK5 MAST1 [activation]
23872 PTPN11 PRKCA [activation]
23873 WHSC1L1 CHEK2 [activation]
23874 MARK2 PRKAA1 [inhibition]
23875 ERCC1 FANCG [activation]
23876 PLCG1 RRAS [activation]
23877 RPA2 ATR [activation]
23878 WRAP73 MYO1C [activation]
23879 HDHD2 EPHB6 [activation]
23880 FZD5 RGS2 [activation]
23881 DNAJB2 FBXO25 [inhibition]
23882 KIF26B CDK6 [inhibition]
23883 HSPB1 C6orf211 [activation]
23884 ESR1 MRPS27 [activation]
23885 HSPA1L METTL22 [activation]
23886 RHOA PDE6D [activation]
23887 RELA EPRS [activation]
23888 SUMO1 ATF2 [activation]
23889 MMP14 CAV1 [activation]
23890 CREBBP MSX1 [activation]
23891 MYO1C TP53 [activation]
23892 GABARAP YWHAE [activation]
23893 TLR2 BGN [activation]
23894 RECQL5 NCL [activation]
23895 TNK2 PDGFRB [activation]
23896 RIMBP3 FANCL [activation]
23897 ELAVL1 RALA [activation]
23898 PTEN CHGB [activation]
23899 NCL PAX8 [activation]
23900 HSPB1 TXNL1 [activation]
23901 FGFR2 STAT5A [activation]
23902 C1orf51 CLOCK [activation;inhibition]
23903 MAPK15 WDR81 [activation;inhibition]
23904 MAPT PIN1P1 [activation]
23905 SRC RPS6KB1 [activation]
23906 BMP2 TGFB2 [activation]
23907 TP53 S100A6 [activation]
23908 PPP2CA ITGA4 [activation]
23909 RPS6KA5 HIST3H2A [activation]
23910 YWHAZ HSPB1 [activation]
23911 PRKACB NGFR [activation]
23912 IRF2 APP [activation]
23913 RIPK2 TLR4 [activation]
23914 PAK2 MAPK3 [activation]
23915 SERBP1 FOS [activation]
23916 CGB CGA [activation]
23917 GNAI3 NXF1 [activation]
23918 MAST2 SH3GL2 [activation]
23919 SGK223 YWHAE [activation]
23920 CDC23 COIL [activation]
23921 PRKCD ACTA2 [activation]
23922 PCSK1 PTK2 [activation;inhibition]
23923 PTPN11 ANXA1 [activation]
23924 PTGES3 NOD1 [activation]
23925 NUP214 NUPL2 [activation]
23926 CRK PTPN11 [activation]
23927 MAPK14 SUMO2 [activation]
23928 CAV1 BSG [activation]
23929 CCR5 ADRBK2 [activation]
23930 MAPK8 CEBPA [activation]
23931 PHGDH ESR1 [activation]
23932 GRB2 SEMA7A [activation]
23933 MAPT TUBB3 [activation]
23934 MYO1B ESR1 [activation]
23935 DIAPH1 RAC1 [activation]
23936 PRKAA1 L3MBTL3 [inhibition]
23937 ESRRG NRIP1 [activation]
23938 WAS ROBO4 [activation]
23939 CAMK4 NOS1 [activation]
23940 ANKRD12 CDK6 [inhibition]

23941	TNFSF14	TRAF2	[activation]
23942	MAGI3	FZD4	[activation]
23943	OBFC1	POT1	[activation]
23944	ADRA2C	GNAO1	[activation]
23945	MAPK14	MBP	[activation]
23946	SMYD2	EPB41L3	[activation]
23947	GRB2	KHSRP	[activation]
23948	XXYLT1	NXF1	[activation]
23949	SMURF1	CXXC1	[inhibition]
23950	CASP3	SOCS5	[inhibition]
23951	ITPR3	ITPR1	[inhibition]
23952	E2F4	NXF1	[activation]
23953	BECN1	F7	[activation]
23954	CHEK1	SMAD4	[activation;inhibition]
23955	MAPK15	CACNA1H	[activation;inhibition]
23956	PDGFRB	GRB14	[activation]
23957	ETS1	AARS	[activation]
23958	STAT3	TRAF6	[activation]
23959	APP	JUN	[activation]
23960	CAV1	ITPR3	[activation]
23961	SOS1	SH3KBP1	[activation]
23962	SMAD3	CDK11A	[activation]
23963	ORC5	CDC7	[activation]
23964	YAP1	ATPIF1	[inhibition]
23965	ITGB2	ABL1	[activation]
23966	DDX21	ITGA4	[activation]
23967	ABL1	TERF1	[activation]
23968	NXF1	CYB5B	[activation]
23969	RABEP1	JUN	[activation]
23970	TNC	PTPRB	[activation]
23971	AKT1	PPM1A	[activation]
23972	PRTN3	TNF	[activation]
23973	DNM2	KPNB1	[activation]
23974	CCND3	CASP2	[activation]
23975	TRAF6	NGFR	[activation]
23976	YY1	RAF1	[activation]
23977	NOTCH1	LCK	[activation]
23978	NRIP1	RARA	[activation]
23979	AHCYL1	SRPK2	[activation]
23980	CCDC8	STAT1	[activation]
23981	BAX	BAK1	[activation;inhibition]
23982	CHEK1	BLM	[activation]
23983	CALM1	TNIK	[activation]
23984	RXRG	NR1H3	[activation]
23985	PGK1	FOXO3	[inhibition]
23986	MYL12A	HSPB1	[activation]
23987	PPP3R1	PPP3CA	[activation]
23988	NR4A1	ESF1	[inhibition]
23989	SMAD9	ARHGAP9	[activation]
23990	CDKN2A	BCL2L1	[activation]
23991	CDC16	ANAPC11	[activation]
23992	YWHAB	DENND4C	[activation]
23993	GIT2	GIT1	[activation]
23994	HNRNPA0	CSF2	[activation]
23995	PPP1R12A	RPA1	[activation]
23996	SIGLEC6	FHL2	[activation]
23997	CTNNB1	AJAP1	[activation]
23998	NCR3	CD59	[activation]
23999	BCL2L11	ATP6V1G1	[activation]
24000	CDC42	PTBP3	[activation]
24001	GRB2	NR1H4	[activation]
24002	IFNG	PDIA3	[inhibition]
24003	NOS3	MPRIP	[activation]
24004	RASA1	KDR	[activation]
24005	RXRA	MECR	[inhibition]
24006	SYK	ERBB2	[activation]
24007	PRKCB	MKI67	[activation]
24008	ATF3	SMAD2	[activation]
24009	GFPT1	MAPK8	[activation]
24010	GSKIP	SMYD2	[activation]
24011	PRMT5	E2F1	[activation]
24012	GJD3	TJP1	[activation]
24013	BCL2L1	PGAM5	[activation]
24014	TNF	ELAVL1	[activation]
24015	GABARAPL2	HNRNPL	[activation]
24016	ATF1	PRKACA	[activation]

24017	TRIM42	GLI1	[inhibition]
24018	SMAD2	CALM1	[activation]
24019	COL4A1	PDGFA	[activation]
24020	SUV420H2	RB1	[activation]
24021	CDC42	RHOJ	[activation]
24022	AHSG	RRAS2	[inhibition]
24023	TLR5	SIGIRR	[activation]
24024	NCK1	SOS1	[activation]
24025	EP300	ING1	[activation]
24026	ACTR2	GRB2	[activation]
24027	CAMK2A	EGFR	[activation]
24028	CDC42SE1	NXF1	[activation]
24029	CAV1	PPARG	[activation]
24030	GRB2	RPS6KA1	[activation]
24031	RAB8A	STK4	[activation]
24032	SRSF5	RPS6KA1	[activation]
24033	BAG2	TAB1	[inhibition]
24034	WNK1	SYNPO	[activation]
24035	YY2	TWIST1	[activation]
24036	CRK	WASF1	[activation]
24037	MED1	TP53	[activation]
24038	MIF	MPG	[activation]
24039	SGSM3	NXF1	[activation]
24040	FANCA	FANCF	[activation]
24041	RNF32	APOE	[activation]
24042	C2orf44	PLK1	[activation;inhibition]
24043	ACTR3	ESR2	[activation]
24044	PTPRU	IRS1	[activation;inhibition]
24045	ESR1	CTNNB1	[activation]
24046	CNN1	ADRB2	[activation]
24047	SRSF4	TICAM1	[activation]
24048	STAT3	ZFPM2	[activation]
24049	TLN1	PXN	[activation]
24050	ANGPTL5	LILRB2	[activation]
24051	HLA-B	DDT	[activation]
24052	YWHAQ	SMURF1	[inhibition]
24053	HLA-B	VMA21	[activation]
24054	PTPRC	UBC	[activation]
24055	SNW1	MAP2K3	[activation]
24056	HSP90AB1	PRKDC	[activation]
24057	CDH15	ARVCF	[activation]
24058	CTNNB1	TOP2A	[activation]
24059	WASF2	APBB1	[activation]
24060	KLF2	KAT2B	[activation]
24061	CMTM5	DDA1	[activation]
24062	ATF5	CD34	[activation]
24063	ALPP	LATS1	[inhibition]
24064	PRR20C	SELV	[activation;inhibition]
24065	STRADB	GRB2	[activation]
24066	SMARCA5	SUMO1	[activation]
24067	NUDC	PAK2	[activation]
24068	PRKCE	GNB2L1	[activation]
24069	CHN1	RPS3A	[activation]
24070	GABRG2	GABARAP	[activation]
24071	DDX21	CDC42BPB	[activation]
24072	HCLS1	GRB2	[activation]
24073	DCSTAMP	CREB3	[activation]
24074	SMURF1	RTKN	[inhibition]
24075	SOX2	CDC26	[activation]
24076	NUP93	WHSC1	[activation]
24077	FAN1	YWHAZ	[activation]
24078	PLCG1	PRKD1	[activation]
24079	EHMT1	SUV39H1	[activation]
24080	HIST1H4A	MAP3K3	[activation;inhibition]
24081	ADAM10	TSPAN15	[activation]
24082	MET	CNR1	[activation]
24083	APP	CACNG2	[activation]
24084	TRAF6	DNAJA1	[inhibition]
24085	SELPLG	TPST1	[activation]
24086	PDGFRA	SRPK1	[activation]
24087	TNNT1	OSM	[activation]
24088	MYC	NLRX1	[inhibition]
24089	RAN	ASB2	[activation]
24090	PDLIM7	PRKCZ	[activation]
24091	SRSF6	VCAM1	[activation]
24092	RAD50	RBBP8	[activation]

24093 ZMAT2 ETS1 [activation]
24094 APP RAB10 [activation]
24095 KSR1 HSP90AB1 [activation]
24096 UBE2N RHNO1 [activation]
24097 CCL8 ACKR2 [activation]
24098 ATF2 TACSTD2 [activation]
24099 RPS6KA5 ERH [activation]
24100 MLH1 FRAT2 [activation]
24101 IL3RA CDC25A [activation]
24102 MRPS27 SMURF1 [inhibition]
24103 VCAM1 ABCE1 [activation]
24104 IL6ST TYK2 [activation]
24105 NTRK3 NGFR [activation]
24106 WNK1 CENPJ [activation]
24107 VCAM1 MCM6 [activation]
24108 RELA ANXA1 [activation]
24109 ABL2 AR [activation]
24110 RAF1 MST4 [activation]
24111 ADAM10 PHB [activation]
24112 TNFRSF10C FAM101B [activation]
24113 MYC HIST1H2AB [activation]
24114 BLNK KIT [activation]
24115 GATA1 CASP3 [activation]
24116 STAT3 OGDHL [activation]
24117 VCAM1 ACTB [activation]
24118 TGFBR1 SKIL [activation]
24119 ATF1 CAMK2G [activation]
24120 ICT1 MRPS5 [activation]
24121 TRAF1 SLC25A6 [activation]
24122 CBL PTEN [activation;inhibition]
24123 CBL PRKCA [activation]
24124 MATK PTK2B [activation]
24125 MRPL38 TP53 [activation]
24126 FAM171A1 ETS1 [activation]
24127 GLP2R GCG [activation]
24128 PRKACB AKAP1 [activation]
24129 STK4 ACTBL2 [activation]
24130 SORL1 AURKA [activation]
24131 PFDN1 DEFA1 [inhibition]
24132 BPGM AKT1 [activation;inhibition]
24133 NUTF2 KPNB1 [activation]
24134 CDK5 HSP90AB1 [activation]
24135 EHMT2 NXF1 [activation]
24136 CUL1 FBXL3 [inhibition]
24137 NUDC KLHL34 [activation]
24138 FLI1 KAT2B [activation]
24139 RAD54B BAAT [activation]
24140 PRKACA SLC9A3R2 [activation]
24141 BRCA2 AURKB [activation]
24142 NXF1 ERBB2 [activation]
24143 GHR PTPN13 [activation]
24144 PSKH2 HSP90AA1 [activation]
24145 NOS2 HSPH1 [activation]
24146 BRD4 CLDN1 [activation]
24147 INADL F2RL2 [activation]
24148 RAF1 BIRC2 [activation;inhibition]
24149 SHC1 HSPA5 [activation]
24150 NUP214 SUMO3 [activation]
24151 ATF7 BCL6 [activation]
24152 PRNP HSPD1 [activation]
24153 MARK3 SOGA2 [activation]
24154 EPHB6 SMAD2 [activation]
24155 CBL SORBS1 [activation]
24156 SMAP1 PHLDA3 [activation]
24157 NCOA3 SMAD3 [activation]
24158 PLEK PF4 [activation]
24159 C19orf10 ATF2 [activation]
24160 LARP1 YWHAZ [activation]
24161 HBS1L SRPK2 [activation]
24162 ABI1 PAK2 [activation]
24163 LRRC4C NTNG2 [activation]
24164 CDK6 AR [activation]
24165 ACTA1 YWHAZ [activation]
24166 PPARG ESR2 [activation]
24167 LIMK1 CFL1 [activation;inhibition]
24168 NFKB1 YY1 [activation]

24169	ACTG1	IKBKG	[activation]
24170	NUDCD3	KLHL38	[activation]
24171	MFNG	DLL1	[activation]
24172	RPA1	CHD1L	[activation]
24173	SRF	RXRA	[inhibition]
24174	QTRTD1	IGHG1	[activation]
24175	CHEK2	TLR3	[activation]
24176	RRAGC	WNT2	[activation]
24177	ATG5	PCMT1	[activation]
24178	CDKL1	TGFBR1	[activation]
24179	ADRBK1	NFKBIA	[activation]
24180	TP53	NCL	[activation]
24181	SPATA2	PTPN6	[activation;inhibition]
24182	MERTK	BMPR2	[activation]
24183	TYK2	UBE2F	[activation]
24184	MYOM1	DNAJB6	[inhibition]
24185	MAD2L1	HSF1	[activation]
24186	ATR	SMC1A	[activation]
24187	EHMT2	ABHD17A	[activation]
24188	NFKBIB	NGFR	[activation]
24189	PDE5A	ITPR1	[inhibition]
24190	CCAR2	CEP170P1	[inhibition]
24191	HSP90AB1	AURKA	[activation]
24192	ATF2	BATF	[activation]
24193	CCNA1	PGR	[activation]
24194	SNAP25	STXBP6	[activation]
24195	ABL1	NCOA3	[activation]
24196	ROCK1	CDK15	[activation]
24197	PTBP3	TP53	[activation]
24198	GBAS	ULK1	[inhibition]
24199	PTPRF	FRS2	[activation]
24200	CAMK4	RBPJ	[inhibition]
24201	CMA1	MMP1	[activation]
24202	CAPN1	NFKBIA	[activation]
24203	FBXW7	MAP4K1	[activation]
24204	WAS	PACSIN3	[activation]
24205	ACACA	MLLT3	[activation;inhibition]
24206	TRAF6	ETFA	[activation]
24207	LY6E	FCGR2B	[activation]
24208	GNG13	GNAT3	[activation]
24209	EPOR	KITLG	[activation]
24210	TRAF6	TOMM70A	[activation]
24211	PLCG1	DAPP1	[activation]
24212	KRT35	GRB2	[activation]
24213	SHC3	GOLGA2	[activation]
24214	USP19	GCN1L1	[inhibition]
24215	IL3RA	NR0B2	[activation]
24216	EIF4A3	EIF4G1	[activation]
24217	PPP2CA	IKBKB	[activation]
24218	PASK	CREB1	[activation]
24219	MYC	CHD5	[activation]
24220	PTPRF	IKBKE	[inhibition]
24221	HSF4	MAPK14	[activation]
24222	KIF23	STK11	[activation]
24223	KDR	CAV1	[activation]
24224	NME2	HSF1	[activation]
24225	NRAS	FANCC	[activation]
24226	GRB2	HNRNPK	[activation]
24227	KCNA3	KCNA2	[activation]
24228	DDX27	ARRB2	[activation]
24229	EIF2B5	EIF2B1	[activation]
24230	LAMB1	PA2G4	[activation]
24231	PTPRK	GHR	[inhibition]
24232	EPAS1	ARNT2	[activation]
24233	FAM9B	BARD1	[activation]
24234	CCNB1	MFN1	[activation]
24235	HLA-F	B2M	[activation]
24236	STAT5A	POU2F1	[activation]
24237	PIK3R1	ARHGEF11	[activation]
24238	EGFR	YWHAE	[activation]
24239	HMGB1	TP73	[activation]
24240	RHOA	SPRED2	[activation]
24241	TSC22D1	ACVR1	[activation;inhibition]
24242	TSC1	YWHAG	[inhibition]
24243	SMAD4	RALA	[activation]
24244	CCHCR1	ZFYVE19	[activation]

24245	LAIR1	CSK	[activation]
24246	NCK1	BRD4	[activation]
24247	FARSB	DDA1	[activation]
24248	PLCG1	RAG1	[activation]
24249	PHKG2	PRKDC	[activation]
24250	SMAD1	BUB1	[activation]
24251	TRPC4AP	RIPK1	[activation]
24252	PDGFA	A2M	[inhibition]
24253	LRP1B	LRPAP1	[inhibition]
24254	PTGES3	FTSJ1	[activation]
24255	PXN	PTPRG	[activation]
24256	SNX1	F2R	[activation]
24257	LRPAP1	RANBP2	[inhibition]
24258	XPNPEP1	POLA2	[activation]
24259	NOTCH2	CNTN1	[activation]
24260	PDDC1	APP	[activation]
24261	SRI	STAT3	[activation]
24262	MAPKAPK3	RB1	[activation]
24263	DAPK3	RHOD	[activation]
24264	GLRX5	APP	[activation]
24265	MDM2	BAIAP2	[activation]
24266	WASF2	ZDHHC17	[activation]
24267	MAPK14	MARCKSL1	[activation]
24268	GNA12	NAPA	[activation]
24269	SUMO1	MITF	[activation]
24270	HES6	HES1	[activation]
24271	AGRN	CACNA1A	[activation]
24272	CTTN	SDC3	[activation]
24273	MAP2K2	MEPCE	[activation]
24274	DDX39A	DDX39B	[activation]
24275	GPX1	MAPK6	[activation;inhibition]
24276	STC2	ARHGEF40	[activation]
24277	TAX1BP1	TBK1	[inhibition]
24278	CCNE1	RHOBTB3	[activation]
24279	CDKN1A	ACTB	[activation]
24280	ATF2	CS	[activation]
24281	CD47	GNAI1	[activation]
24282	JAK2	NCK1	[activation]
24283	YWHAG	DDX27	[activation]
24284	BCL10	TRAF6	[activation]
24285	LRRK2	CSNK1G3	[activation]
24286	EGFR	VAV3	[activation]
24287	ABCA2	CDK5RAP2	[activation]
24288	RAD54B	PRKAA1	[activation]
24289	NOS2	CAV1	[activation]
24290	PRDM2	SRC	[activation]
24291	ABI1	TLN1	[activation]
24292	ELANE	SLPI	[inhibition]
24293	POU5F1	CTCF	[activation]
24294	PRKCD	ADAM9	[activation]
24295	PLCG1	ABL2	[activation]
24296	IKBKE	TFRC	[activation]
24297	WDR91	NUDC	[activation]
24298	UBE2N	BIRC3	[activation]
24299	PPFIA3	PTPRG	[activation]
24300	MAPK1	MAP2K7	[activation]
24301	DVL2	CCDC33	[activation]
24302	HSP90AA1	NLRP3	[inhibition]
24303	RSPH3	SMAD3	[inhibition]
24304	SRPK1	DDX47	[activation]
24305	CUX1	CDK1	[activation]
24306	FYN	SH3KBP1	[activation]
24307	HMGB1	CREBBP	[activation]
24308	SERPINF2	BCAP31	[activation]
24309	GHRL	TBXA2R	[inhibition]
24310	SOCS3	DAB1	[inhibition]
24311	SUV39H2	KCTD17	[activation]
24312	RBL1	SMARCA4	[activation]
24313	GRB2	PNMA5	[activation]
24314	YWHAB	PRKCZ	[activation]
24315	RHOU	GRB2	[activation]
24316	PRKCE	EGFR	[activation]
24317	VTN	FGG	[activation]
24318	NRIP1	ESRRB	[activation]
24319	CBL	DBNL	[inhibition]
24320	MAP1LC3B	ANAPC1	[activation]

24321	ADORA2B	DCC	[activation]
24322	OPRD1	PRKCA	[activation]
24323	NOTCH2NL	RTN4RL1	[activation]
24324	MSR1	NKG7	[activation]
24325	PDE4DIP	SNCA	[activation]
24326	STK4	IPO5	[activation]
24327	FYN	NPVF	[activation]
24328	ATM	SPSB1	[activation]
24329	IQGAP1	CDH2	[activation]
24330	ATAD3B	TP53	[activation]
24331	PEX1	RELA	[activation]
24332	KPNA1	HIST3H3	[activation]
24333	PTPRA	PTPRD	[activation;inhibition]
24334	TSPAN4	CD46	[inhibition]
24335	HIST2H2BE	EP300	[activation]
24336	ARFGAP1	GABARAPL2	[activation]
24337	IQGAP1	GRIA4	[activation]
24338	TLR3	MBL2	[activation]
24339	HMGXB4	HABP4	[activation]
24340	AKT1	GATA1	[activation]
24341	CDK1	NCAPH	[activation]
24342	TFAP2A	COL1A1	[activation]
24343	APP	ABL1	[activation]
24344	IQGAP1	NPHS2	[activation]
24345	GRB2	TF	[activation]
24346	SNTA1	SCTR	[activation]
24347	FOS	SMARCD1	[activation]
24348	CCDC112	FSD2	[activation]
24349	TUBB4A	RELA	[activation]
24350	CBL	CCT8	[inhibition]
24351	ATR	EGFR	[activation]
24352	PPP2R5A	PPP2R2D	[activation]
24353	AURKA	SRPK2	[activation]
24354	TTN	DYSF	[activation]
24355	SRSF6	OBSL1	[activation]
24356	SFN	CRK	[activation]
24357	TTLL7	APP	[activation]
24358	ATM	PPP2R5C	[activation]
24359	RPS6	ICAM1	[activation]
24360	IKBKB	ZC3H12A	[activation]
24361	ST7	NFKBIA	[activation]
24362	WASF2	GAS7	[activation]
24363	TRAF2	NCAPH2	[activation]
24364	SCAP	SREBF2	[activation]
24365	WIPF2	WAS	[activation]
24366	IPO5	LRRK2	[activation]
24367	FGFR3	ATF3	[activation]
24368	APP	ARAP1	[activation]
24369	HRK	BCL2L2	[activation]
24370	UHRF2	PTPRC	[activation]
24371	RAD52	HSPA8	[activation]
24372	ESR1	ACTR3	[activation]
24373	CTTNBP2NL	STK24	[activation]
24374	TP53	RFFL	[activation]
24375	APP	PCK1	[activation]
24376	PRKCA	DLX3	[activation]
24377	HSPB1	TMCO3	[activation]
24378	TRAF2	MAP3K8	[activation]
24379	MTRNR2L1	PPA1	[activation]
24380	MYC	SGOL2	[activation]
24381	YWHAB	ARRB1	[activation]
24382	BMPR2	TUBB	[activation]
24383	MCM7	RB1	[activation]
24384	ATOX1	GGPS1	[activation]
24385	USP42	SPP1	[activation]
24386	LRRK2	TUBG1	[activation]
24387	LRRK2	MRGBP	[activation]
24388	POLA1	RB1	[activation]
24389	IGHG1	IGKC	[activation]
24390	AR	RAD9A	[activation]
24391	EFHC2	CCNG2	[activation]
24392	CHMP3	SMAD1	[activation]
24393	SHC1	ACTB	[activation]
24394	CASP1	MAPT	[activation]
24395	ABL1	NFKBIA	[activation]
24396	EGFR	ERBB4	[activation]

24397	PAXIP1	BTBD16	[activation]
24398	DVL1	LRRK2	[activation]
24399	TP53BP1	TP53	[activation]
24400	SMURF1	MAP3K3	[inhibition]
24401	APOE	NOS3	[activation]
24402	RPA3	CLINT1	[activation]
24403	PTN	BCCIP	[activation]
24404	FBXO6	PPM1B	[inhibition]
24405	GDF9	RSRC1	[activation]
24406	RTCA	HLA-B	[activation]
24407	CDC5L	TRAP1	[activation]
24408	MYC	ATM	[activation]
24409	ADRBK1	EPHA2	[activation]
24410	HDAC3	GEM	[activation]
24411	ATG7	FOXO1	[activation]
24412	NOTCH3	E2F3	[activation]
24413	MET	CTTN	[activation]
24414	MPP1	GADD45A	[activation]
24415	VCAM1	PTPN11	[activation]
24416	PTPN1	MAPK1	[activation]
24417	MOAP1	TNFRSF10A	[activation]
24418	NBN	RFC1	[activation]
24419	LYPD3	LSP1	[activation]
24420	VCAM1	H2AFY	[activation]
24421	TRADD	ATP1A1	[activation]
24422	PTH	PTH2R	[activation]
24423	UBE2W	RMND5B	[activation]
24424	YWHAH	ESR2	[activation]
24425	CD5	CD2	[activation]
24426	CTBP1	MAPK9	[activation]
24427	MYL6	ITGA4	[activation]
24428	CASP8	PIAS1	[inhibition]
24429	IPO5	NUP153	[activation]
24430	PCK1	DDX21	[activation]
24431	CDH1	HRAS	[activation]
24432	TLN1	ITGB2	[activation]
24433	EIF4A3	HNRNPL	[activation]
24434	PTPN7	CCDC57	[activation]
24435	CDC5L	BRD7	[activation]
24436	JAK2	KPNB1	[activation]
24437	SMAD2	STAG1	[activation]
24438	RPA3	SCAMP3	[activation]
24439	CRKL	GRB2	[activation]
24440	SOGA2	NXF1	[activation]
24441	STRN3	APC	[inhibition]
24442	MRPS28	TP53	[activation]
24443	CD82	ITGB2	[activation]
24444	ACTG1	GIT2	[activation]
24445	MDC1	CDC27	[activation]
24446	ATXN1L	RBPJ	[inhibition]
24447	MPP5	TP53	[activation]
24448	GRB2	ACKR3	[activation]
24449	SMYD4	MYC	[activation]
24450	FYN	GNS	[activation]
24451	MIF	TRAF6	[activation]
24452	ANGPT4	TIE1	[activation]
24453	APP	MB	[inhibition]
24454	CFLAR	CASP6	[activation]
24455	HRG	PLSCR1	[activation]
24456	SF3B4	GRB2	[activation]
24457	RAC1	FRK	[activation]
24458	PAK4	SRPK1	[activation]
24459	SMARCA4	CCNE1	[activation]
24460	GAB2	YWHAQ	[activation]
24461	MAPK7	PFDN1	[activation]
24462	SDC3	CSK	[activation]
24463	OBSL1	MDC1	[activation]
24464	IRAK1	MYC	[activation;inhibition]
24465	COPZ1	UGT1A1	[activation]
24466	RAD17	HUS1	[activation]
24467	EXOC1	MYC	[activation]
24468	YWHAG	DDX5	[activation]
24469	TNFRSF4	TNFRSF9	[activation]
24470	RARG	SRF	[activation]
24471	EIF2S2	TLE1	[activation]
24472	HLA-C	PIN1	[activation]

24473	RRAGB	RRAGA	[activation]
24474	BID	BCL2A1	[activation]
24475	TERF1	BLM	[activation]
24476	RHOU	PTK2B	[activation]
24477	LRRK2	YME1L1	[activation]
24478	IL13RA2	CASP8	[inhibition]
24479	ASPM	PLK1	[activation;inhibition]
24480	TRPM7	ANXA1	[activation]
24481	EPHA1	HSP90AA1	[activation]
24482	EVL	ABI1	[activation]
24483	MLH1	PARVA	[activation]
24484	PCED1A	CAV1	[activation]
24485	JAM3	PARD3	[activation]
24486	GAB2	YWHAE	[activation]
24487	IL15RA	CDK4	[inhibition]
24488	DYRK1A	SRSF1	[activation]
24489	TIAM1	PARD3	[activation]
24490	UNC119	CD3E	[activation]
24491	EZH2	PPARG	[activation]
24492	MAP3K7	PPP2CA	[inhibition]
24493	PSEN1	CASP7	[activation]
24494	GRM1	GRASP	[activation]
24495	BTC	ERBB4	[activation]
24496	YWHAB	IGF1R	[activation]
24497	SYK	SEC23B	[activation]
24498	TRAF2	SEC16A	[activation]
24499	PARD3	HSPA6	[activation]
24500	DVL1	NCK2	[activation]
24501	NOS3	MAST1	[activation]
24502	CDC23	SH3KBP1	[activation]
24503	PRKCZ	TRIM41	[activation]
24504	YWHAE	ARRB2	[activation]
24505	STK11	WDR6	[activation]
24506	DDX17	ESR1	[activation]
24507	MAST3	PKP3	[activation]
24508	MDM2	RASSF6	[activation]
24509	CTTN	WAS	[activation]
24510	STAT3	PTK2	[activation]
24511	PTPN1	CCL5	[activation]
24512	KCNIP4	PSEN2	[activation]
24513	SKP2	CCNB1	[inhibition]
24514	G3BP2	DDX24	[activation]
24515	TFRC	LAPTM4B	[activation]
24516	YWHAE	PRKCI	[activation]
24517	SMAD2	GFM2	[inhibition]
24518	CD44	MSN	[activation]
24519	PAX6	TBP	[activation]
24520	ESR1	ZNHIT3	[activation]
24521	WIZ	NOTCH1	[activation]
24522	TRAF6	PPP4R1	[activation]
24523	PIN1	CTNNB1	[activation]
24524	LYN	DDX21	[activation]
24525	MTNR1A	MTNR1B	[activation]
24526	BCL3	ANKRD28	[activation]
24527	RUNX1	CBFA2T3	[activation]
24528	KCNC1	CAMK2A	[activation]
24529	EGFR	NLRP10	[activation]
24530	EPSTI1	CASP8	[inhibition]
24531	NUP214	SOX2	[activation]
24532	FBXO31	ATM	[activation]
24533	RASSF8	YWHAG	[activation]
24534	MLH1	ACER3	[activation]
24535	AR	POU5F1	[activation]
24536	ACTG1	RPS6KA5	[activation]
24537	BHLHE40	PLEKHB2	[activation]
24538	EP300	HMGN2	[activation]
24539	IK	PFN2	[activation]
24540	RPA1	CSNK1A1L	[activation]
24541	PSENEN	THBD	[activation]
24542	CCDC36	MVP	[activation]
24543	PRLR	YWHAG	[inhibition]
24544	PAXIP1	H1FX	[activation]
24545	RXRA	TAF1B	[inhibition]
24546	PPP2CA	SMURF1	[inhibition]
24547	PLXNA1	PLXNB1	[activation]
24548	LCE3A	HSPB1	[activation]

24549	PTTG1	BTRC	[inhibition]
24550	TGFB1	SDC2	[activation]
24551	PRKCZ	HRAS	[activation]
24552	NAT2	ATM	[activation]
24553	KLB	FGF21	[activation]
24554	CALU	ATF2	[activation]
24555	HELQ	XRCC2	[activation]
24556	GRB2	NCK1	[activation]
24557	RBP4	GRB2	[activation]
24558	PFN1	DNM2	[activation]
24559	FOXA2	OTX2	[activation]
24560	DNAJA2	EPAS1	[inhibition]
24561	QRSL1	PPBP	[activation]
24562	NEDD9	NFKBIA	[activation]
24563	PPIA	PCNA	[inhibition]
24564	ESR1	NCOA1	[activation]
24565	GNAQ	ADHFE1	[activation]
24566	ATR	CINP	[activation]
24567	CDK4	KLHL32	[inhibition]
24568	CARD16	CARD17	[inhibition]
24569	NR1D1	INPP1	[inhibition]
24570	NR1H3	HSP90AB1	[activation]
24571	PRMT1	VCAM1	[activation]
24572	ARHGDIA	CASP3	[activation]
24573	IRS2	MPL	[activation]
24574	SIPA1L2	TGM2	[activation]
24575	CDK1	CD8A	[activation]
24576	VASN	TGFB3	[activation]
24577	IRAK4	SASH1	[activation;inhibition]
24578	CTNNB1	MAPK8	[activation]
24579	MYL12A	TRAF6	[activation]
24580	STK3	CDK3	[activation]
24581	FBXO6	EIF4A3	[activation]
24582	EIF3A	CD81	[activation]
24583	GABRA3	SDCBP2	[activation]
24584	RHOBTB3	CCNB1	[activation]
24585	PRMT7	PIK3CG	[activation]
24586	TP53RK	LRRC2	[activation]
24587	LIMK2	GSN	[activation]
24588	PLK1	USP20	[activation]
24589	BCR	PTPN11	[activation]
24590	CASP3	PSEN2	[activation]
24591	YWHAG	PRKCD	[activation]
24592	MBP	CCNH	[activation]
24593	PTPN11	PIK3R2	[activation]
24594	FBXW7	DCLRE1C	[activation]
24595	SMAD6	BAMBI	[inhibition]
24596	VRK2	MAP3K7	[activation]
24597	ARHGEF7	TUBB4A	[activation]
24598	CNTFR	HN1L	[inhibition]
24599	AIRE	FYN	[activation]
24600	GNB1	NUDCD3	[activation]
24601	SOX6	POMGNT1	[activation]
24602	TP53BP1	ANAPC2	[activation]
24603	HNRNPA3	ICAM1	[activation]
24604	KRT31	GNAI2	[activation;inhibition]
24605	INS	CTSB	[activation]
24606	BRK1	PCDH18	[activation]
24607	NXF1	DEPDC7	[activation]
24608	HOXA7	JUNB	[activation]
24609	PDHA1	ITGA4	[activation]
24610	CENPO	FOS	[activation]
24611	PRAM1	TP53	[activation]
24612	NAA16	SMURF1	[inhibition]
24613	SRPK1	ZRANB2	[activation]
24614	NXF1	HSPA4	[activation]
24615	MAP1B	STK4	[activation]
24616	FYN	PLCG2	[activation]
24617	DAB1	ERBB2	[activation]
24618	NOTCH2NL	LIMS2	[activation]
24619	CAMKK1	APP	[activation]
24620	ARNTL2	NPAS2	[activation]
24621	HSPA2	HSPH1	[activation]
24622	AURKA	KIF11	[activation]
24623	BCL2L11	UBC	[activation]
24624	HNRNPU	ICAM1	[activation]

24625	RAD51	XPO1	[activation]
24626	UNC5A	DAPK1	[activation]
24627	CSNK1G2	PPP1R14A	[inhibition]
24628	C3AR1	SNTA1	[activation]
24629	FAM154A	FAM124A	[activation]
24630	DSG1	SSSCA1	[activation]
24631	PML	H2AFX	[activation]
24632	NR3C1	NFKB2	[activation]
24633	CDC42	RAC2	[activation]
24634	ACTR2	HLA-B	[activation]
24635	PPFIBP1	PPFIA3	[activation]
24636	MCM6	CCNA1	[activation]
24637	FGFR1	EPHA4	[activation]
24638	DRD2	KCNJ6	[activation]
24639	GRB2	SPECC1L	[activation]
24640	SRC	BAAT	[activation]
24641	IGSF8	CD81	[activation]
24642	YLPM1	GRB2	[activation]
24643	SSTR3	TP53	[activation]
24644	TRBC1	TRAC	[activation]
24645	ADCY5	PRKCA	[activation]
24646	FGA	CDH5	[activation]
24647	CSNK1G1	RELA	[activation]
24648	FRS2	SOS1	[activation]
24649	NHP2L1	SRPK3	[activation]
24650	RIC8A	GNA13	[activation]
24651	STK31	ESR2	[activation]
24652	ICT1	MRPL27	[activation]
24653	NCOA2	STAT6	[activation]
24654	FAP	VIP	[activation]
24655	SNRPA1	ICAM1	[activation]
24656	RFX6	NEDD9	[activation]
24657	SCUBE1	SCUBE3	[activation]
24658	RALA	RAP2A	[activation]
24659	PRKACA	NFKB1	[activation]
24660	ERC1	SMAD2	[activation]
24661	MOB2	EIF4A3	[activation]
24662	ARPC4	EIF1B	[activation]
24663	RPL31	CRKL	[activation]
24664	CHEK1	AKT1	[activation;inhibition]
24665	NXF1	RFXANK	[activation]
24666	OXSR1	CAB39	[activation]
24667	PPP1CC	WDR92	[inhibition]
24668	CTBP1	EP300	[activation]
24669	GNAI1	ADRA2A	[activation]
24670	RAPGEF3	RANBP2	[activation]
24671	CMYA5	BZW1	[activation]
24672	IL1RL2	IL18	[activation]
24673	ARRB1	NFKBIA	[activation]
24674	PKM	EGLN1	[activation]
24675	ARF6	TRMT112	[activation]
24676	PPP1CA	PRR16	[activation;inhibition]
24677	LMNB1	ATF4	[activation]
24678	BRD7	SMARCA4	[activation]
24679	EGFR	AKT1	[activation]
24680	RAD21	EIF3D	[activation]
24681	EIF2B2	CSNK2A2	[activation]
24682	SMAD1	ARL4D	[activation]
24683	CHUK	FKBP5	[activation]
24684	MRAS	RAF1	[activation]
24685	STX7	VTI1B	[activation]
24686	PRKCA	CCDC8	[activation]
24687	NOTCH1	STK38	[activation]
24688	TES	ENAH	[activation]
24689	E2F1	ANAPC5	[activation]
24690	HSPBAP1	ARHGEF5	[activation]
24691	HSPA4L	YAP1	[inhibition]
24692	HSPA4L	DOK2	[activation]
24693	LRPAP1	SUMO2	[inhibition]
24694	DDX17	TP53	[activation]
24695	LRRK2	BAG1	[activation]
24696	IRF7	ALK	[activation]
24697	NFKB2	CCAR2	[inhibition]
24698	CDX1	SOX2	[activation]
24699	ANXA2	SOS1	[activation]
24700	LATS1	DNAJC11	[inhibition]

24701	EP300	PAX6	[activation]
24702	PIK3CG	NOTCH1	[activation]
24703	EZH2	HSPB1	[activation]
24704	CD81	HIST2H3A	[activation]
24705	CDKN1B	CHUK	[activation;inhibition]
24706	OPRM1	DOK5	[activation]
24707	NRXN1	SIPA1L1	[activation]
24708	HLA-B	CAPZA1	[activation]
24709	CAPN11	UNG	[activation]
24710	CSF2RB	GNB2L1	[activation]
24711	TSSK3	EFHC2	[activation]
24712	BCAP31	ACTG1	[activation]
24713	ASAP1	SRC	[activation]
24714	KIAA1958	SOCS3	[activation;inhibition]
24715	CCDC174	EIF4A3	[activation]
24716	LATS1	SPERT	[inhibition]
24717	DHX34	GSK3B	[activation]
24718	TGFBR1	NUAK2	[activation]
24719	HSP90AB1	MAP4K1	[activation]
24720	CREBBP	HOXB1	[activation]
24721	MLH1	ATM	[activation]
24722	SELPLG	MSN	[activation]
24723	CITED2	HNF4A	[activation]
24724	SMARCA2	MYB	[activation]
24725	MAGED2	PCK1	[activation]
24726	BTK	SYK	[activation]
24727	ASAP2	THSD7A	[activation]
24728	UBE2I	PPARG	[activation]
24729	NRXN1	NXPH3	[activation]
24730	RIPK2	EZR	[activation]
24731	MDM2	NUP188	[activation]
24732	ARRB2	RALGDS	[activation]
24733	CBLL1	CDC42	[activation]
24734	FHL3	PTPN6	[activation;inhibition]
24735	MMP14	TIMP4	[activation]
24736	HNRNPK	ICAM1	[activation]
24737	INS	RB1	[activation]
24738	PIK3R1	ARHGAP17	[activation]
24739	SMAD4	FAM84B	[activation]
24740	SPP1	RANBP9	[activation]
24741	TP73	ASPM	[activation;inhibition]
24742	AKT1	AGR3	[activation]
24743	CALM1	GRB7	[activation]
24744	HIST1H1B	EHMT2	[activation]
24745	EIF3I	CD81	[activation]
24746	NBN	CCNE1	[activation]
24747	E2F1	CDK8	[activation]
24748	ACACA	UBC	[activation;inhibition]
24749	RTKN	YWHAB	[activation]
24750	PBX1	KAT2A	[activation]
24751	SLC9A3R2	PDGFRB	[activation]
24752	ULK1	ATG5	[activation]
24753	ARF1	SPAG9	[inhibition]
24754	LNX2	CXADR	[activation]
24755	FYN	FCER2	[activation]
24756	MAP3K11	RHOG	[activation]
24757	SIK1	YWHAZ	[activation]
24758	SPRR2A	AKT2	[activation]
24759	DCD	ILK	[activation]
24760	PKM	STK4	[activation]
24761	DDX5	MAST3	[activation]
24762	TTC32	MYOG	[activation]
24763	KRIT1	FAS	[activation]
24764	UBE2E3	TGFBR1	[activation]
24765	HIST3H3	BIRC5	[activation]
24766	ICAM1	RPL26L1	[activation]
24767	SP1	HSPA8	[activation]
24768	PPP6C	MAP3K7	[activation]
24769	RAP2C	ADRB2	[activation]
24770	ACVR1	CHN1	[activation]
24771	ANKS3	NXF1	[activation]
24772	CHEK2	PRMT2	[activation]
24773	LTBP4	IGHG1	[inhibition]
24774	NEDD4	CASP7	[inhibition]
24775	BARD1	BRD7	[activation]
24776	SRPK1	NSG1	[activation]

24777	PPARGC1A	BCL6	[activation]
24778	KAT2B	RELA	[activation]
24779	PTPN6	INSR	[activation;inhibition]
24780	CASP3	DCTN1	[activation]
24781	MEP1B	PRKCD	[activation]
24782	NAB2	EGR2	[activation]
24783	PRKCQ	LYN	[activation]
24784	ASB2	KMT2A	[activation]
24785	CADPS2	DRD2	[activation]
24786	CXCL9	DPP4	[activation]
24787	NFKB2	NFKB1	[activation]
24788	MLLT4	RIN1	[activation]
24789	TLR9	PRKCZ	[activation]
24790	DNAJA3	RASA1	[activation]
24791	SPARC	PDGFB	[activation]
24792	CCBE1	STX11	[activation]
24793	PBRM1	HIST3H3	[activation]
24794	ATP1A1	SRC	[activation]
24795	ESR2	RPS17	[activation]
24796	MS4A2	SYK	[activation]
24797	F11R	PRKCA	[activation]
24798	SOD1	HOXB2	[inhibition]
24799	JUN	GSK3B	[activation]
24800	IRAK4	EIF4EBP1	[activation;inhibition]
24801	CDC25B	MELK	[activation]
24802	MYOD1	DDX5	[activation]
24803	CYCS	BCL2L1	[activation]
24804	VDR	STAT1	[activation]
24805	GLI1	RPS6KB1	[activation]
24806	FOXM1	TCF7L2	[inhibition]
24807	TIRAP	RPTOR	[activation]
24808	PPME1	PPP2R1A	[activation]
24809	MAPK1	LAMTOR3	[activation]
24810	OBSCN	MYC	[activation]
24811	RANBP3	RAN	[activation]
24812	LAS1L	PYHIN1	[activation]
24813	HSP90B1	H2AFX	[activation]
24814	NCK1	ARHGEF11	[activation]
24815	SYN1	SRC	[activation]
24816	HLA-B	SMC2	[activation]
24817	KPNB1	MDC1	[activation]
24818	PCNA	CLSPN	[activation]
24819	NCOA2	DDX17	[activation]
24820	ADRB2	GNAI2	[activation]
24821	IRF1	HSP90AA1	[activation]
24822	ENO1	TOMM40L	[inhibition]
24823	DYRK1A	SIRT1	[activation]
24824	NCKIPSD	GRB2	[activation]
24825	CR2	TP53	[activation]
24826	PRNP	KIAA1191	[activation]
24827	TNFSF14	TRAF3	[activation]
24828	DAB1	LRP8	[activation]
24829	BARD1	CDK13	[activation]
24830	PNPLA2	SMAD9	[activation]
24831	APC2	AXIN2	[inhibition]
24832	EPHB4	JUN	[activation]
24833	FAM27E3	IRS4	[activation]
24834	MDM2	FOXO4	[activation]
24835	IL6R	IL6ST	[activation]
24836	PRNP	GRB2	[activation]
24837	RAC2	RTKN	[activation]
24838	BDNF	INPP5K	[activation]
24839	FLT1	NCK1	[activation]
24840	RAD54B	DMC1	[activation]
24841	IGF2	NMRK2	[activation]
24842	NFKB2	IQGAP1	[inhibition]
24843	BRCA1	CHEK2	[activation]
24844	RASSF1	RASSF2	[inhibition]
24845	WASF1	CDC42	[activation]
24846	KAT2A	HSD11B2	[activation]
24847	PIN1	JAKMIP2	[activation]
24848	GATA1	RB1	[activation]
24849	KPNA1	BCAR3	[activation]
24850	MRPL10	ICT1	[activation]
24851	STAT3	FES	[activation]
24852	PAK1	PYCARD	[inhibition]

24853	EGFR	CLPP	[activation]
24854	POT1	CTC1	[activation]
24855	ARPC2	CDH1	[activation]
24856	PTEN	CCNE2	[activation]
24857	LIN37	MYBL2	[inhibition]
24858	MLH1	ACO2	[activation]
24859	NXF1	ZNF207	[activation]
24860	PTPRK	INSR	[inhibition]
24861	TLK1	SNAP23	[activation]
24862	PLK1	BLM	[activation]
24863	VCAM1	PPP1CC	[activation]
24864	KANSL1	HSPB1	[activation]
24865	VAV1	PTPN6	[activation;inhibition]
24866	STX4	GOSR2	[activation]
24867	AXIN1	SENP2	[activation]
24868	SMAD1	RUNX1	[activation]
24869	PRKAA2	PIK3CG	[activation]
24870	OPRM1	GJA4	[activation]
24871	PRTN3	IL1B	[activation]
24872	MYL12A	CHUK	[activation]
24873	LCA5L	SUV39H2	[activation]
24874	ARF6	ACLY	[activation]
24875	RAPGEF4	TRAF2	[activation]
24876	INSR	IGF2	[activation]
24877	FAM86A	FAM86C1	[activation]
24878	CCNG1	PAK7	[activation]
24879	PIK3R1	PDIA2	[activation]
24880	E2F1	BIRC2	[activation;inhibition]
24881	FYN	GRB10	[activation]
24882	ANXA2	NCOA3	[activation]
24883	PTPN1	STAM2	[activation]
24884	SERTAD1	CDK4	[inhibition]
24885	CLINT1	JUN	[activation]
24886	FYB	CRK	[activation]
24887	APP	BMPR1B	[activation]
24888	BTRC	NFE2	[activation]
24889	FBXW11	CASP3	[activation]
24890	HIPK4	RAF1	[activation]
24891	PFN1	GPHN	[activation]
24892	STK38	MAPKAP1	[activation]
24893	TP53BP1	CDC23	[activation]
24894	RHOG	VAV2	[activation]
24895	MLLT4	RAP2A	[activation]
24896	TNFRSF14	GCN1L1	[activation]
24897	ARHGAP1	SRC	[activation]
24898	IL15	ZNRD1	[activation]
24899	FAM179B	ESR2	[activation]
24900	SKAP2	GRB2	[activation]
24901	LONRF1	HSPB1	[inhibition]
24902	PTPRC	NTRK1	[activation]
24903	IGF1	WISP2	[activation]
24904	LRRK2	SLFN5	[activation]
24905	SOX9	NR5A1	[activation]
24906	SERPINB6	F10	[inhibition]
24907	SMAD3	ESR2	[activation]
24908	ARPC4	LRRK2	[activation]
24909	SDC3	FYN	[activation]
24910	PRKACA	CFL1	[activation;inhibition]
24911	C3	RAD21	[activation]
24912	HSPH1	MAP1LC3B	[activation]
24913	INSR	GRB7	[activation]
24914	GSK3B	MYCN	[activation]
24915	CCL26	CCR3	[activation]
24916	F2R	CHRD	[activation]
24917	NTRK2	SORT1	[activation]
24918	YWHAQ	EFNB1	[activation]
24919	INPP5F	YWHAB	[activation]
24920	CDK2	NFYA	[activation]
24921	APC	DLG3	[inhibition]
24922	TERT	SMARCE1	[activation]
24923	ABL1	YTHDC1	[activation]
24924	SOS1	ITSN1	[activation]
24925	HSPB1	EIF4G2	[activation]
24926	TNIK	PRPSAP1	[activation]
24927	EHMT1	CTBP1	[activation]
24928	ICAM4	ITGAV	[activation]

24929	CAST	PPFIA3	[inhibition]
24930	HSPB1	CDC5L	[activation]
24931	MTHFD1L	ILK	[activation]
24932	GNAQ	GRM4	[activation]
24933	RHEBL1	BMPR1B	[activation]
24934	TRAF2	USP7	[activation]
24935	NXF1	CAMKMT	[activation]
24936	LPCAT1	ILK	[activation]
24937	CYP11A1	SMAD9	[activation]
24938	GABRB3	PPP2CA	[activation]
24939	ADAM22	RAC2	[activation]
24940	MAPK1	NOTCH1	[activation]
24941	HDAC4	HIF1A	[activation]
24942	VANGL1	SRPK1	[activation]
24943	CREBBP	CDC20	[activation]
24944	IL13RA2	PALB2	[activation]
24945	RAC3	RSRC1	[activation]
24946	POLD1	NCAPG	[activation]
24947	GNAI3	ESR1	[activation]
24948	CBL	FYB	[activation]
24949	POLI	TRAF6	[activation]
24950	TRAF6	KCNQ1	[activation]
24951	RPA1	DDX5	[activation]
24952	CAMK2G	PEA15	[inhibition]
24953	C18orf25	SRPK2	[activation]
24954	MAPK9	SMAD3	[activation]
24955	PYCARD	CASP8	[inhibition]
24956	UBE2I	CDH4	[activation]
24957	TERF1	SMAD2	[activation]
24958	RHOD	SMAD2	[activation]
24959	BMP1	COL5A2	[activation]
24960	CTR9	SOX2	[activation]
24961	SNW1	NR0B1	[activation]
24962	EIF3F	ICAM1	[activation]
24963	LUZP4	FOS	[activation]
24964	C14orf105	TRAF2	[activation]
24965	ARRB1	GPR50	[activation]
24966	ZDHHC3	SMAD1	[activation]
24967	ACTB	MX1	[activation]
24968	EXT2	ANXA7	[activation]
24969	ASB6	FGB	[activation]
24970	EGFR	TJP1	[activation]
24971	MAVS	MAP3K7	[activation]
24972	BAX	ANP32B	[inhibition]
24973	SLC52A2	CDC23	[activation]
24974	RARRES3	HRAS	[activation]
24975	CD28	LRRC23	[activation]
24976	C8G	C8A	[inhibition]
24977	UBTF	MAPK1	[activation]
24978	TNIK	HARS	[activation]
24979	ZC3H13	EIF4A3	[activation]
24980	SYK	CSF2RB	[activation]
24981	TRIP6	RHOA	[activation]
24982	MYC	CNOT11	[activation]
24983	STAT5A	MRPS6	[activation]
24984	LAT2	PLCG2	[activation]
24985	YWHAH	PRKD2	[activation]
24986	RPA1	MYO1B	[activation]
24987	ITGB1	RPA3	[activation]
24988	TP53	TPT1	[activation]
24989	PRKCDBP	MYC	[activation]
24990	C3	PAPPA	[activation]
24991	PIK3CD	UBC	[activation]
24992	HSPB1	HSPA4	[inhibition]
24993	RHOD	TGFBR1	[activation]
24994	RAVER1	TP53	[activation]
24995	EZR	SPN	[activation]
24996	C3	IGHM	[activation]
24997	CEP250	HSPA2	[activation]
24998	EGR1	RELA	[activation]
24999	ZSWIM7	RAD51D	[activation]
25000	CREBBP	TBP	[activation]
25001	GNAS	GNB1	[activation]
25002	BFAR	BCL2L1	[activation]
25003	NXF1	MRPS27	[activation]
25004	PTPN1	PLD2	[activation]

25005 SRP72 CASP3 [activation]
25006 HIST1H1D ITGA4 [activation]
25007 CEP55 MYC [activation]
25008 TRAPPC11 TP73 [activation;inhibition]
25009 PTPN1 MRC2 [activation]
25010 BTK FAS [activation]
25011 AURKA BAAT [activation]
25012 ACTB PLG [activation]
25013 CIAO1 POLD1 [activation]
25014 FAM118B GNB2 [activation]
25015 MAPK9 C1orf94 [activation]
25016 SH3KBP1 ARAP1 [activation]
25017 YWHAG DFFA [activation]
25018 FYN TNF [activation]
25019 CSPG4 BCAR1 [activation]
25020 HSPB1 KCMF1 [activation]
25021 MAPK14 TGFBR1 [activation]
25022 INADL MAPK12 [activation]
25023 CCDC8 CCAR2 [inhibition]
25024 NCK1 DOK2 [activation]
25025 PTGS2 CAV1 [activation]
25026 VDR SRC [activation]
25027 BUB3 PSTPIP1 [inhibition]
25028 C15orf59 PPP1CC [inhibition]
25029 EIF1AY SRPK2 [activation]
25030 YWHAG RAB11A [activation]
25031 PTPRC LGALS1 [activation]
25032 JUN MTA1 [activation]
25033 CLCA2 ITGB4 [activation]
25034 HSP90AA1 EIF2AK1 [activation]
25035 ANLN MYC [activation]
25036 NSUN2 VCAM1 [activation]
25037 HSP90AA1 GZMA [activation]
25038 PARD6B YWHAH [activation]
25039 MTNR1A VAPB [activation]
25040 RXRA SMARCA4 [inhibition]
25041 GNB2L1 HIF1A [activation]
25042 MRPL43 MDM2 [activation]
25043 NXF1 CASC3 [activation]
25044 ISL1 LMX1A [activation]
25045 TICAM2 OPN1LW [activation]
25046 GAB2 PLCG1 [activation]
25047 ETV4 APP [activation]
25048 YWHAG PRKCZ [activation]
25049 BTRC PRKACA [activation]
25050 PPM1A MAPK14 [activation]
25051 CCAR2 CIAO1 [inhibition]
25052 YWHAE MAP3K3 [activation]
25053 PRKAG1 ULK1 [inhibition]
25054 PPARG POU1F1 [activation]
25055 VAPB PRKACB [activation]
25056 PTGDR MAPK1 [activation]
25057 ATM BCAS3 [activation]
25058 CNTNAP1 ABL1 [activation]
25059 RELA CTNNB1 [activation]
25060 POMC NRD1 [activation]
25061 NCOA3 NFKB1 [activation]
25062 PTK2B PITPNM1 [activation]
25063 AMH ARL8B [activation]
25064 RAC1 STAT3 [activation]
25065 APH1A BHLHE40 [activation]
25066 NFKB1 ACTG1 [activation]
25067 MCM3 VCAM1 [activation]
25068 PPP1R1B KLHL6 [inhibition]
25069 SRPK1 SRSF8 [activation]
25070 HIST1H2BC EGFR [activation]
25071 SRPK2 DDX39A [activation]
25072 NR1D2 NR1D1 [inhibition]
25073 PIAS1 SERBP1 [inhibition]
25074 CRY2 PER3 [inhibition]
25075 EP300 PLK1 [activation]
25076 MAG MBP [activation]
25077 PTPLAD1 VAPB [activation]
25078 IFNW1 APP [activation]
25079 BARD1 DDX20 [activation]
25080 STYK1 HSP90AA1 [activation]

25081	FST	BMP5	[inhibition]
25082	CD7	SECTM1	[activation]
25083	UGDH	GRB2	[activation]
25084	EHMT2	CBX3	[activation]
25085	C3	LAMA1	[activation]
25086	TRAFD1	CDK20	[activation]
25087	AARSD1	GNA13	[activation]
25088	BID	TLE1	[activation]
25089	SRPK2	PPIA	[activation]
25090	NXF1	PIBF1	[activation]
25091	FUNDC2	RGS1	[activation;inhibition]
25092	GSK3B	PFKFB4	[activation]
25093	DNM2	TRIP10	[activation]
25094	MAVS	RIPK2	[activation]
25095	MAP2K3	APP	[activation]
25096	APC	NAV2	[inhibition]
25097	MAP3K14	EGFR	[activation]
25098	ARHGEF6	PAK3	[activation]
25099	ARF6	APP	[activation]
25100	RASA1	DOK2	[activation]
25101	ICK	CDC7	[activation]
25102	EPRS	TAB1	[inhibition]
25103	CACNA1C	CACNB3	[activation]
25104	PIK3R2	PIK3R1	[activation]
25105	CREBBP	NUP98	[activation]
25106	COPS8	TP53	[activation]
25107	AURKB	HNRNPA1	[activation]
25108	LTA4H	HLA-B	[activation]
25109	CASP3	EIF4B	[activation]
25110	CRTC2	LGALS3BP	[activation]
25111	PRMT1	NRIP1	[activation]
25112	TFRC	SGTA	[activation]
25113	KCTD12	ARAP1	[activation]
25114	SRPK1	ABCF1	[activation]
25115	MAPK13	HNRNPA1	[activation]
25116	MAU2	NOTCH1	[activation]
25117	FANCC	FANCA	[activation]
25118	EPPK1	GRB2	[activation]
25119	TGFBR1	CDK14	[activation;inhibition]
25120	TP53	VASP	[activation]
25121	SLC26A8	NXF1	[activation]
25122	MAPK12	MBP	[activation]
25123	ASIP	F11R	[inhibition]
25124	MAP2K1	LAMTOR3	[activation]
25125	GDF9	ADPGK	[activation]
25126	TRAF6	YARS	[activation]
25127	TRAF2	NT5DC2	[activation]
25128	YWHAZ	PGLYRP1	[activation]
25129	HSP90AB1	SGK2	[activation]
25130	EPHB2	ITSN1	[activation]
25131	RHOC	DAAM1	[activation]
25132	SHC1	ITGB4	[activation]
25133	CDK1	HMGA2	[activation]
25134	IL24	LSP1	[activation]
25135	GAPDH	CDKN1A	[inhibition]
25136	NR4A1	PPARG	[inhibition]
25137	NTRK2	RASGRF1	[activation]
25138	TFPI	THBS1	[inhibition]
25139	MAP3K8	ACY3	[activation]
25140	SSTR5	PDZK1	[activation]
25141	HLA-C	TRIM28	[activation]
25142	BAI1	INADL	[activation]
25143	SFN	SIPA1L1	[activation]
25144	RABGAP1	TWIST1	[activation]
25145	HSP90AB1	RELA	[activation]
25146	HIST1H1C	STK4	[activation]
25147	MAPK9	EGFR	[activation]
25148	SMAD2	LEF1	[activation]
25149	GRB2	SIT1	[activation]
25150	H1FX	EGFR	[activation]
25151	GRB2	APOL5	[activation]
25152	YTHDC2	IRAK1	[activation;inhibition]
25153	ANXA2	MAP3K4	[activation]
25154	COPS6	EDN1	[activation]
25155	MAGED1	IRS4	[activation]
25156	DVL2	RHOXF2	[activation]

25157	MAPK3	VDAC1	[inhibition]
25158	CD200	HUS1	[activation]
25159	SRCAP	EP300	[activation]
25160	CAMK2B	MAD2L2	[inhibition]
25161	ARHGEF7	SCRIB	[activation]
25162	JUND	MDM2	[activation]
25163	JUNB	YY1	[activation]
25164	CDKN2A	HSPA8	[inhibition]
25165	COL1A2	PDGFB	[activation]
25166	LATS1	CDK2	[inhibition]
25167	STAT1	ESR1	[activation]
25168	PSD4	ARL14	[activation]
25169	PRKCD	IBTK	[activation]
25170	TNFRSF17	TNFSF13	[activation]
25171	CD226	FYN	[activation]
25172	CASP6	HTT	[activation]
25173	CDC20	SOSTDC1	[inhibition]
25174	CHCHD2	EHMT2	[activation]
25175	CDK5	CDK16	[activation]
25176	ITCH	SMAD2	[activation]
25177	EP300	SMAD3	[activation]
25178	IL17RE	IL17RA	[activation]
25179	SLC39A1	PRKCZ	[activation]
25180	RRP1	GABARAP	[activation]
25181	ANKS1A	SFN	[activation]
25182	PIK3R2	HSPA8	[activation]
25183	HSP90AA1	MAP3K12	[activation]
25184	CDC42	DAAM1	[activation]
25185	IRAK3	CDCA5	[inhibition]
25186	NOTCH2	JAG2	[activation]
25187	COPS6	MAPKAPK3	[activation]
25188	LYZ	ELN	[activation]
25189	EGFR	PLD2	[activation]
25190	RAD52	UBE2I	[activation]
25191	MYC	SMC6	[activation]
25192	ANKRD17	AURKB	[activation]
25193	ITCH	DVL3	[activation]
25194	YTHDF3	RPA3	[activation]
25195	SKP2	DUSP1	[inhibition]
25196	LYN	ARHGAP19	[activation]
25197	NOTCH2	PSMC3IP	[activation]
25198	KANSL1	TP53	[activation]
25199	ARPC3	ARAP1	[activation]
25200	SETD7	SERTAD1	[activation]
25201	PPARGC1A	NCOA1	[activation]
25202	RAD52	MCM2	[activation]
25203	OGT	FOXO4	[activation]
25204	USP2	CCND1	[inhibition]
25205	PNPLA2	GBF1	[activation]
25206	BCR	SRC	[activation]
25207	GNB3	GNAI3	[activation]
25208	OPRM1	GPRASP1	[activation]
25209	TREM2	TYROBP	[activation]
25210	APP	PARD6B	[activation]
25211	HNF4A	NR3C1	[activation]
25212	LARS	CCDC8	[activation]
25213	MTNR1B	ITM2C	[activation]
25214	MYC	MIB1	[activation]
25215	RPA3	RAB5C	[activation]
25216	ATF2	FSCN1	[activation]
25217	MTOR	LAMTOR5	[activation]
25218	DCC	PTK2B	[activation]
25219	PFKP	ILK	[activation]
25220	CFTR	DNAJA1	[inhibition]
25221	PGR	SORBS1	[activation]
25222	HCK	CRKL	[activation]
25223	RXRA	PRMT2	[inhibition]
25224	NUP62	TRAF3	[activation]
25225	GTF2A1	TAF1	[activation]
25226	SRC	KCNQ5	[activation]
25227	SRPK1	IKBKG	[activation]
25228	PAK2	EIF3I	[activation]
25229	DOCK11	CALM1	[activation]
25230	MAP3K7	PEBP1	[inhibition]
25231	VASP	NXF1	[activation]
25232	SAT1	HSPB1	[activation]

25233	MVP	TEP1	[activation]
25234	C11orf68	EIF4H	[activation]
25235	UBC	PLCE1	[activation]
25236	EHMT1	SMARCAD1	[activation]
25237	FOXM1	CHEK2	[activation]
25238	RIPK1	DNAJA1	[inhibition]
25239	ATF3	ACTB	[activation]
25240	SNAP25	GOSR1	[activation]
25241	PRKCH	PPP1R14A	[activation]
25242	CRK	DLGAP4	[activation]
25243	LPAR2	BCL6	[activation]
25244	SGTA	GIP	[activation]
25245	ARF1	INSR	[activation]
25246	MAPK13	YWHAZ	[activation]
25247	PRKAA1	PPP2R1B	[inhibition]
25248	KLHL12	C1QTNF2	[activation]
25249	FGFR1	HSPG2	[activation]
25250	SH3GL2	PAK2	[activation]
25251	TRAPPC9	IKBKB	[activation]
25252	STX7	ELAVL1	[activation]
25253	PLCG2	PLXNB1	[activation]
25254	PDGFRB	HSP90AB1	[activation]
25255	XBP1	HM13	[activation]
25256	CNTF	KRT40	[activation]
25257	AR	BTG2	[activation]
25258	OBSL1	CCT8	[inhibition]
25259	LCE2A	RGS17	[activation;inhibition]
25260	BMPR1B	FANCL	[activation]
25261	BMPR2	ACVR1B	[activation]
25262	SRPK1	CRHR1	[activation]
25263	RPS6KA5	DDAH2	[activation]
25264	NGFRAP1	NTRK3	[activation]
25265	EP300	MCM4	[activation]
25266	PGR	FGFR2	[activation]
25267	CTCF	SMAD3	[activation]
25268	MAPK3	GATA4	[activation]
25269	FGF2	DCN	[activation]
25270	H2AFX	YWHAZ	[activation]
25271	CAV1	ERBB2	[activation]
25272	GDF11	WFIKKN1	[activation]
25273	DIABLO	MAML2	[activation]
25274	TBRG4	ASAP2	[activation]
25275	LRRK2	HOGA1	[activation]
25276	YWHAE	EIF4B	[activation]
25277	MDM2	HSPH1	[activation]
25278	DDX20	MYC	[activation]
25279	PC	EGFR	[activation]
25280	MYLK3	HSP90AB1	[activation]
25281	PRKCZ	RELA	[activation]
25282	STK11	ZNF189	[activation]
25283	ARRB2	MAP3K5	[inhibition]
25284	TIMP2	ITGA3	[activation]
25285	BCL2L1	PPP1CA	[activation]
25286	HSPB1	CASP3	[activation]
25287	RAB10	RPA1	[activation]
25288	NUDC	WDR16	[activation]
25289	NEO1	TTN	[activation]
25290	GTPBP2	SUV39H1	[activation]
25291	JUP	CTNNA2	[activation]
25292	PDLIM7	PRKCB	[activation]
25293	RBL1	CD2BP2	[activation;inhibition]
25294	DDIT3	CDK6	[inhibition]
25295	KCNA2	NEU1	[activation]
25296	TUBGCP6	NXF1	[activation]
25297	STK11	CDC14B	[activation]
25298	CHGB	POLD1	[activation]
25299	RPS6KA5	ETV1	[activation]
25300	SH3KBP1	PIK3CA	[activation]
25301	POLD1	CD247	[activation]
25302	NUAK2	SMAD4	[activation;inhibition]
25303	LAS1L	LRRK2	[activation]
25304	MRPS34	DDX56	[activation]
25305	CASP4	MYCBPAP	[activation]
25306	GNAO1	CRHR1	[activation]
25307	GRB2	DLD	[activation]
25308	INADL	NLGN2	[activation]

25309	SMARCB1	CHD7	[activation]
25310	MRPL14	ICT1	[activation]
25311	PTGES3	EGFR	[activation]
25312	DAB2	HSPH1	[activation]
25313	COL5A1	BMP1	[activation]
25314	PPP1R13L	PPP1CC	[inhibition]
25315	EP300	CDX2	[activation]
25316	LHX3	ISL1	[activation]
25317	KIF5B	APC	[inhibition]
25318	USP11	MAPK14	[activation]
25319	MDM2	CFL1	[activation;inhibition]
25320	FOXP3	RELA	[activation]
25321	IL6	HRH1	[activation]
25322	GZMB	ELANE	[activation]
25323	AR	CDC25B	[activation]
25324	PHLDB2	YWHAB	[activation]
25325	BLM	RPA1	[activation]
25326	SKIV2L	CDKL5	[activation]
25327	KIT	PTPN6	[activation]
25328	F11	KNG1	[activation]
25329	OPRK1	WLS	[activation]
25330	MTA1	TP53	[activation]
25331	ACACA	MCC	[activation;inhibition]
25332	ESR1	SMARCA4	[activation]
25333	CCNH	CCDC33	[activation]
25334	KLK7	CCND1	[activation]
25335	FAM9B	UBE2E2	[activation]
25336	FBXO6	TTC17	[inhibition]
25337	CREB1	KIAA2026	[activation]
25338	APP	HMGCS2	[activation]
25339	GNAQ	CXCR5	[activation]
25340	MYC	ARFGAP1	[activation]
25341	PDHB	GRB7	[activation]
25342	RPTOR	AKT1S1	[activation;inhibition]
25343	IRS1	DDR1	[activation]
25344	REV3L	REV1	[activation]
25345	G3BP1	PDPK1	[activation]
25346	MME	EDN2	[activation]
25347	PTPN11	IQCB1	[activation;inhibition]
25348	GPSM1	TRIP13	[inhibition]
25349	BTK	CBL	[activation]
25350	NR3C1	DAP3	[activation]
25351	CDC5L	SEH1L	[activation]
25352	BAD	PIM3	[activation;inhibition]
25353	IPO5	ITGA4	[activation]
25354	AKT1	PDE3B	[activation]
25355	ESR1	RPS6KA1	[activation]
25356	NGFR	RIPK2	[activation]
25357	CTNNB1	ZIC3	[activation]
25358	RPS6KA1	VASP	[activation]
25359	NXF1	TMEM164	[activation]
25360	CAMK2G	USP19	[activation]
25361	UBE4B	CASP7	[activation]
25362	FAAP24	LRRK2	[activation]
25363	GRB2	APOH	[activation]
25364	BTG2	SMAD1	[activation]
25365	CCDC8	F5	[inhibition]
25366	APPL1	KIAA1737	[activation]
25367	SMAD2	PPP2R1A	[inhibition]
25368	ATF2	RAB14	[activation]
25369	TP53	NCOA1	[activation]
25370	TAB2	NFKB1	[activation]
25371	GNA14	ADRB2	[activation]
25372	KLF13	GATA6	[activation]
25373	UBE2N	TP53	[activation]
25374	ATF2	RAB2B	[activation]
25375	RHOA	SMURF1	[activation]
25376	GNAZ	EYA1	[activation]
25377	TNF	CARS	[activation]
25378	FMNL1	FLI1	[activation]
25379	TTC5	CAMK2D	[activation]
25380	RASA1	CASP3	[activation]
25381	RB1	CASP10	[activation]
25382	NCK1	NTRK2	[activation]
25383	CSF3R	FTH1	[activation]
25384	MYOG	ELSPBP1	[activation]

25385	IFNGR2	CAMK2D	[activation]
25386	SRPK2	ATAT1	[activation]
25387	GLOD4	NR4A1	[inhibition]
25388	MYO3A	TGFBR1	[inhibition]
25389	MRPS18B	RB1	[activation;inhibition]
25390	USP6NL	GRB2	[activation]
25391	RIPK1	SUMO1	[activation]
25392	STK3	MAP1LC3A	[activation]
25393	PHLDA3	TRIO	[activation]
25394	CELA1	CSN2	[activation]
25395	CCNDBP1	MOS	[activation]
25396	TRIM69	SKIL	[activation]
25397	PRKCB	ANXA7	[activation]
25398	MYC	PPP1R15A	[inhibition]
25399	SERPINA4	YAE1D1	[inhibition]
25400	CDK19	ERC1	[activation]
25401	NBN	CD3EAP	[activation]
25402	BAD	PPP1CA	[activation;inhibition]
25403	SLC25A6	MDM2	[activation]
25404	HLA-B	CD81	[activation]
25405	SERPINF2	F13A1	[activation]
25406	FCGR2B	PLCG1	[activation]
25407	ATPIF1	GSK3B	[inhibition]
25408	PXN	APC	[activation]
25409	EP300	DAO	[activation]
25410	VARS	MAPK11	[activation]
25411	DOC2B	UNC13B	[activation]
25412	PTK2B	DLGAP3	[activation]
25413	ST14	AKT1	[activation]
25414	NCOR1	MYB	[activation]
25415	HSPA4	RHO	[activation]
25416	CDK3	HSP90AB1	[activation]
25417	DDAH2	GADD45G	[activation]
25418	RAC1	FLNA	[activation]
25419	ETS1	CPLX2	[activation]
25420	PRMT1	SSSCA1	[activation]
25421	CTNNB1	CSNK2B	[activation]
25422	MCM7	CTNNB1	[activation]
25423	PTN	PPP2CA	[inhibition]
25424	EIF3I	NR1H3	[activation]
25425	PTPN14	YWHAB	[activation]
25426	KCNF1	OPRM1	[activation]
25427	EPOR	PTPN6	[activation;inhibition]
25428	RAB5A	PIK3R1	[activation]
25429	GABBR1	RGS4	[activation;inhibition]
25430	YTHDC1	EIF4A3	[activation]
25431	VCAM1	MCM2	[activation]
25432	CDK1	ERCC2	[activation]
25433	CDH1	CREBBP	[activation]
25434	HIST1H4A	BRD7	[activation]
25435	MAX	CASP7	[activation]
25436	ERH	PAXIP1	[activation]
25437	VGF	LRRK2	[activation]
25438	ATF2	ATF7	[activation]
25439	RALGPS2	YWHAG	[activation]
25440	PTK2B	FGFR3	[activation]
25441	SMC4	CCDC8	[activation]
25442	BARD1	MAPK1	[activation]
25443	FANCG	PRDX3	[activation]
25444	ADRA1B	NOS1	[activation]
25445	PRMT1	NXF1	[activation]
25446	CCDC155	CXCL9	[activation]
25447	GRB2	SOCS1	[activation]
25448	TRAF1	GEM	[activation]
25449	DNAJB5	CCRN4L	[inhibition]
25450	EIF2B5	CHMP2A	[activation]
25451	APP	LCP2	[activation]
25452	KCND2	KCNIP4	[activation]
25453	CEP250	DDX24	[activation]
25454	RBPJ	GADD45A	[activation]
25455	GNL3	ESR2	[activation]
25456	OSR2	LIMS1	[activation]
25457	TSC22D1	TP53	[activation]
25458	PIK3CA	BEX2	[activation]
25459	CUX1	EHMT2	[activation]
25460	MCM7	ZAK	[activation]

25461	CPNE1	ARRB2	[activation]
25462	HIST1H4A	TAF1B	[activation]
25463	CCDC8	PLXNB2	[activation]
25464	ANK1	CD44	[activation]
25465	MSANTD3	VSTM2A	[activation]
25466	LRRK2	POLDIP3	[activation]
25467	TGFBR2	HSP90AA1	[activation]
25468	MDM2	ITCH	[activation]
25469	JAK3	IRS2	[activation]
25470	NRTN	GFRA1	[activation]
25471	RAB11A	RAB11FIP1	[activation]
25472	MRPS27	YWHAZ	[activation]
25473	MYOD1	ESR1	[activation]
25474	NGFR	MAPK1	[activation;inhibition]
25475	KIF23	RNASEL	[activation]
25476	CCDC8	GAPVD1	[activation]
25477	PTPN11	PRLR	[activation;inhibition]
25478	MAPT	MAPK11	[activation]
25479	CREBBP	EIF2B1	[activation]
25480	GABARAPL2	KIAA1958	[activation]
25481	RGS6	GNB2	[activation;inhibition]
25482	C1R	EP300	[activation]
25483	CNIH4	ILK	[activation]
25484	RALB	PYCARD	[inhibition]
25485	TNFRSF13B	MYD88	[activation]
25486	MAP4K4	MDM2	[activation]
25487	CDKL5	ABL1	[activation]
25488	MAP2K1	GRB10	[activation]
25489	HELB	RPA1	[activation]
25490	SNAP25	ZDHHC17	[activation]
25491	FAM74A4	NOTCH2NL	[activation]
25492	AARSD1	GNAL	[inhibition]
25493	EIF3E	CNTF	[activation]
25494	MAPK6	SOCS3	[activation;inhibition]
25495	PPARA	STAC3	[activation]
25496	DLX4	SMAD4	[activation]
25497	TIAL1	GADD45A	[activation]
25498	PPM1B	ANXA2	[activation]
25499	XIAP	NOD1	[activation]
25500	KDELR1	SEC23B	[activation]
25501	HSP90AA1	TRAF2	[activation]
25502	HIP1	CASP8	[activation]
25503	BARD1	SETDB1	[activation]
25504	AURKA	HSPA8	[activation]
25505	MAP3K7	IRAK1	[activation;inhibition]
25506	ARF6	HLA-B	[activation]
25507	YWHAE	RAD52	[activation]
25508	POTEKP	ERBB3	[activation]
25509	BMPR1B	STK35	[activation;inhibition]
25510	FLT1	CTNNB1	[activation]
25511	CDK2	MAPK15	[activation;inhibition]
25512	PRKACA	SLC2A2	[activation]
25513	CASP7	GOLGA3	[activation]
25514	FBXL2	AURKB	[activation]
25515	ISL1	RELA	[activation]
25516	GNA13	GNA12	[activation]
25517	HSPB1	ADRBK2	[activation]
25518	CCAR2	TSSC1	[inhibition]
25519	HIF1A	FBXW7	[activation]
25520	SHC1	PPAP2B	[activation]
25521	NOTCH2NL	FAM71E2	[activation]
25522	SLC2A5	CCL5	[activation]
25523	UBE2H	DHPS	[activation]
25524	ABL2	FBXO38	[activation]
25525	TPT1	PER2	[activation]
25526	GHRL	PTGIR	[activation]
25527	RPS6KB1	PPP2R2B	[activation]
25528	KAT2B	MECOM	[activation]
25529	MGLL	CHGB	[activation]
25530	FHL2	E2F1	[activation]
25531	PPP2R4	MDM2	[activation]
25532	PPM1F	CAMK2A	[activation]
25533	SLC9A3R1	PDGFRA	[activation]
25534	DDX24	NXF1	[activation]
25535	SEMA3B	SEMA3A	[activation]
25536	EGLN3	MAPK1	[activation]

25537	COL1A1	TGFBI	[activation]
25538	SH2B2	JAK1	[activation]
25539	SEMA4A	PLXNB3	[activation]
25540	PIK3CA	IL13RA2	[activation]
25541	NOTCH1	CNOT1	[activation]
25542	LUC7L2	NUTF2	[activation]
25543	MAP4K5	SETDB1	[activation]
25544	C16orf59	NOTCH2NL	[activation]
25545	TIMP1	ECH1	[activation]
25546	NFKB2	HSP90AB1	[activation]
25547	CENPF	SNAP25	[activation]
25548	WDR34	TRAF6	[activation]
25549	RARG	SRC	[activation]
25550	STX3	UBC	[activation]
25551	GRB2	PLXNB1	[activation]
25552	NUF2	AURKB	[activation]
25553	DFNA5	EGFR	[activation]
25554	LZTS2	RAC1	[activation]
25555	PTPRG	PDGFRB	[activation]
25556	MYLK	CAMK2G	[inhibition]
25557	MAPK8	CASP3	[activation]
25558	TWIST1	BRAP	[activation]
25559	KCNK12	AXIN2	[inhibition]
25560	WDR6	ILK	[activation]
25561	CTNNB1	PTPRC	[activation]
25562	KNG1	APP	[activation]
25563	KRT40	SHC3	[activation]
25564	EFEMP1	TRAF2	[activation]
25565	CDK4	ASPM	[activation]
25566	PGR	STAT3	[activation]
25567	PPIA	ITGA4	[activation]
25568	NXF1	YARS	[activation]
25569	SNW1	EIF4A1	[activation]
25570	PDIA3	ADAM10	[activation]
25571	VAPB	STX1A	[activation]
25572	OBSL1	DDX54	[activation]
25573	SMARCB1	PDPK1	[activation]
25574	ABL1	PTGES3	[activation]
25575	UBC	C5AR1	[activation]
25576	RPS13	SPP1	[activation]
25577	SMAP2	VCAM1	[activation]
25578	PDC	CAMK2A	[activation]
25579	TIAL1	VCAM1	[activation]
25580	PAK6	LRRK1	[activation]
25581	DSC1	MAPK7	[activation]
25582	RIPK2	UBE2I	[activation]
25583	TLR2	MAP2K1	[activation]
25584	FASLG	PACSIN1	[activation]
25585	CD160	AGTRAP	[activation]
25586	IGF1	IGFBP7	[activation]
25587	ATR	MTA2	[activation]
25588	CCDC180	STK11	[activation]
25589	ACP6	ESRRB	[activation]
25590	SFN	MAP3K2	[activation]
25591	SELV	PAXIP1	[activation]
25592	SMCHD1	BARD1	[activation]
25593	BTK	GNA12	[activation]
25594	ATG5	SIRT1	[activation]
25595	STX17	TLR2	[activation]
25596	GPSM1	CCDC158	[inhibition]
25597	KRAS	EPB42	[activation]
25598	HEMGN	TLR2	[activation]
25599	SMC3	HLA-B	[activation]
25600	HLA-B	SUCLG1	[activation]
25601	PLCG1	LAT2	[activation]
25602	EGFR	PTPN6	[activation;inhibition]
25603	ESR1	YWHAH	[activation]
25604	RGS2	RAP1B	[activation]
25605	UBC	DLL1	[activation]
25606	GDF9	FXYD6	[activation]
25607	HLA-B	UBA2	[activation]
25608	MYC	DNAJA3	[inhibition]
25609	EXOC5	EGFR	[activation]
25610	GRB2	HOMEZ	[activation]
25611	BARD1	LDOC1	[activation]
25612	IQGAP1	LRRK2	[activation]

25613	GNB2L1	LRRK2	[activation]
25614	SNTA1	NOS1	[activation]
25615	PRNP	NOB1	[activation]
25616	FTSJ3	GABARAP	[activation]
25617	ATF2	JDP2	[activation]
25618	RPS15	LRRK2	[activation]
25619	CSNK2B	CDK11A	[activation]
25620	ERBB3	HLA-B	[activation]
25621	IRF8	TRAF6	[activation]
25622	EIF2S1	MELK	[activation]
25623	MTUS2	CDK5R1	[activation]
25624	BCAP31	FIS1	[activation]
25625	STK11	MLF1	[activation]
25626	MAPK1	TNFRSF25	[activation]
25627	LCK	EZR	[activation]
25628	TP53	NMT2	[activation]
25629	MYB	PCDH17	[activation]
25630	KPNB1	TRADD	[activation]
25631	STXBP1	PLD1	[activation]
25632	MAPT	STXBP1	[activation]
25633	CAB39L	BCKDK	[activation]
25634	SNW1	NCAPD2	[activation]
25635	LPAR1	KRAS	[activation]
25636	GXYLT1	TRAF6	[activation]
25637	CASP3	PICALM	[activation]
25638	SMAD4	SMAD6	[activation;inhibition]
25639	ZHX1	BCL2L1	[activation]
25640	SNAP29	EHD1	[activation]
25641	CDC23	INPP5J	[activation]
25642	E2F1	PKIB	[inhibition]
25643	PIK3R1	NTRK2	[activation]
25644	EXOC3	MLH1	[activation]
25645	HOXD4	EP300	[activation]
25646	CD2	PSTPIP1	[activation]
25647	MLH1	TAGLN	[activation]
25648	PORCN	WNT3A	[activation]
25649	CSNK2A1	PTPRC	[activation]
25650	STAT1	EIF2AK2	[activation]
25651	YWHAG	PAK1	[activation]
25652	TFF1	ESR2	[activation]
25653	DNAJB11	MYC	[inhibition]
25654	BID	NXF1	[activation]
25655	MAP3K2	YWHAB	[activation]
25656	EHMT1	DNMT3A	[activation]
25657	CCL3	TGFB1	[activation]
25658	ATM	MED1	[activation]
25659	CASP8	LRRK2	[activation]
25660	ACTG1	YWHAQ	[activation]
25661	COL4A2	OSM	[activation]
25662	MAPK9	BAZ1B	[activation]
25663	HSP90AB1	FKBPL	[activation]
25664	BAI1	ELMO1	[activation]
25665	PNP	NXF1	[activation]
25666	HIST1H2AB	ITGA4	[activation]
25667	CEBPB	RELA	[activation]
25668	RYR2	CACNA1C	[activation]
25669	DDX21	MAST3	[activation]
25670	PLK1	TP53BP2	[activation]
25671	UBE2U	MDM2	[activation]
25672	PAXIP1	WEE1	[activation]
25673	RBBP9	RBL1	[activation;inhibition]
25674	EIF2S3	CAND1	[activation]
25675	OGT	PPP2R2B	[activation]
25676	SIPA1L2	LATS1	[inhibition]
25677	FOS	TRO	[activation]
25678	GRB2	EIF4A1	[activation]
25679	MAP3K1	RAF1	[activation]
25680	PON2	GRB2	[activation]
25681	DLGAP4	SRC	[activation]
25682	SCARB1	FLT1	[activation]
25683	SNTA1	PLCB3	[activation]
25684	MEN1	RPA3	[activation]
25685	DOK1	SH2D1A	[activation]
25686	RARA	NCOA1	[activation]
25687	BUB1B	PLK3	[inhibition]
25688	STOM	EGFR	[activation]

25689	ICAM1	RPS2	[activation]
25690	CDK10	NUDCD3	[activation]
25691	SMAD3	SMAD5	[activation]
25692	MOS	USHBP1	[activation]
25693	COPA	STK4	[activation]
25694	IPO7	RAN	[activation]
25695	NCF1	MAPK14	[activation]
25696	FAM131A	PHYHIP	[activation]
25697	GNAQ	TTC1	[activation]
25698	FECH	MDM2	[activation]
25699	ZNF321P	SRPK2	[activation]
25700	LRRK2	SNX9	[activation]
25701	DUSP15	ABL1	[activation]
25702	CDK1	TFDP1	[activation]
25703	PDGFRL	SFRP4	[activation]
25704	EIF6	ACTG1	[activation]
25705	ATF1	CSNK2A1	[activation]
25706	DVL2	MAP3K1	[activation]
25707	PEPD	SPP1	[activation]
25708	YWHAG	LRRK2	[activation]
25709	DKK3	BCAR3	[inhibition]
25710	DVL2	CTNNB1	[activation]
25711	CTNNB1	DNAJC8	[inhibition]
25712	SMC1A	POLA1	[activation]
25713	CREB3L2	GAS7	[activation]
25714	FLT1	STAT3	[activation]
25715	PPP2CB	SMAD4	[inhibition]
25716	SSBP2	IL36RN	[activation]
25717	PALB2	RAD51AP1	[activation]
25718	ITGA4	CD44	[activation]
25719	BCAS3	STK11	[activation]
25720	C2orf44	CSNK1E	[activation]
25721	SERPING1	NR4A1	[inhibition]
25722	KPNB1	KPNA1	[activation]
25723	MAP3K4	RAC1	[activation]
25724	ENKD1	DVL2	[activation]
25725	SHC1	SHCBP1	[activation]
25726	BTRC	EP300	[activation]
25727	KIT	CSF2RA	[activation]
25728	TRAF2	FAM90A1	[activation]
25729	HOXC8	SMAD4	[inhibition]
25730	PAXIP1	CAPZB	[activation]
25731	DAW1	SRPK1	[activation]
25732	OLA1	BCL6	[activation]
25733	ITCH	TGFBR1	[activation]
25734	FBXO31	CUL1	[inhibition]
25735	PPP1CC	TBC1D19	[inhibition]
25736	EP400	TP53	[activation]
25737	HSPA6	TP53	[activation]
25738	CPVL	EGFR	[activation]
25739	SDC1	IL8	[activation]
25740	CASP8	IL24	[activation]
25741	LAMB1	ESR2	[activation]
25742	ESR1	SMAD4	[activation]
25743	CHEK2	PLK3	[activation]
25744	FLAD1	ICT1	[activation]
25745	ACVR1	VEPH1	[activation;inhibition]
25746	IL18R1	CD48	[activation]
25747	CCL5	EDC4	[activation]
25748	PFKFB2	YWHAE	[activation]
25749	HHEX	TRMT6	[activation]
25750	FHOD1	RAC1	[activation]
25751	FBXO28	PAK1	[activation]
25752	UTP18	ERBB2	[activation]
25753	BCAR1	SH2D3A	[activation]
25754	LRRK2	ABL1	[activation]
25755	IL3RA	NCK1	[activation]
25756	RAD9A	BCL2L1	[activation]
25757	MYB	SDC2	[activation]
25758	GRB2	DIAPH1	[activation]
25759	S100A14	AKT1	[inhibition]
25760	GADD45A	RARA	[activation]
25761	CASP8	CASC3	[activation]
25762	APPL1	UBE2O	[activation]
25763	PPM1A	PRKAA1	[inhibition]
25764	GATA6	EP300	[activation]

25765	ATAD3A	ILK	[activation]
25766	KRT17	EGFR	[activation]
25767	ARRB2	OXTR	[activation]
25768	NFKBIA	CAPN2	[activation]
25769	YWHAG	CFL1	[activation]
25770	CDC27	COMT	[activation]
25771	ADAMTS20	MYC	[activation]
25772	NFKB1	SPAG9	[inhibition]
25773	IL1RAP	MC4R	[activation]
25774	PRKCI	GLI1	[activation]
25775	CSK	FHL2	[activation]
25776	RTCB	MDM2	[activation]
25777	APP	KCNMB2	[activation]
25778	NPEPPS	TIMP2	[activation]
25779	ITGA9	PXN	[activation]
25780	UBE2I	AR	[activation]
25781	CCDC97	NXF1	[activation]
25782	HSPA4	YWHAZ	[activation]
25783	EGFR	SEPP1	[activation]
25784	HSP90AB1	HCK	[activation]
25785	TNFRSF14	ITFG1	[activation]
25786	NPHP3	UNC119B	[inhibition]
25787	PAK1	SRGAP2	[activation]
25788	DAB2	LRP6	[activation]
25789	ADAM9	ITGAV	[activation]
25790	MAPK1	SYNE2	[activation]
25791	ZAP70	CRK	[activation]
25792	EIF4A3	HIST1H2AD	[activation]
25793	SLA	LAT	[activation]
25794	ZAP70	SIT1	[activation]
25795	NRXN1	SYT2	[activation]
25796	CREBBP	HIST2H2BE	[activation]
25797	CRK	CBLB	[inhibition]
25798	MYC	MAP3K1	[activation]
25799	PIK3C3	SUMO1	[activation]
25800	C9orf156	BMPR1A	[activation;inhibition]
25801	TKT	ATG5	[activation]
25802	CDKN2C	RIF1	[inhibition]
25803	EIF2S2	HDGF	[activation]
25804	BRAP	HRAS	[activation]
25805	RAC1	TIAM1	[activation]
25806	PRKAA2	FNIP1	[activation]
25807	MYC	RPS16	[activation]
25808	GLTPD1	LRRK2	[activation]
25809	MAP1LC3B	HSPA6	[activation]
25810	HOXA11	FOXO1	[activation]
25811	ENO2	ST3GAL2	[activation]
25812	GNAI2	SVIL	[activation;inhibition]
25813	GYS1	OTUD5	[inhibition]
25814	EIF4A1	MEPCE	[activation]
25815	BCL9L	CTNNB1	[activation]
25816	DAPK2	ATF4	[activation]
25817	TAB1	FADD	[inhibition]
25818	CDK18	MIPOL1	[activation]
25819	TNF	BGN	[activation]
25820	DCC	EIF2B1	[activation]
25821	LUC7L2	SRPK1	[activation]
25822	DNAJA3	ICT1	[inhibition]
25823	DVL1	SMAD1	[activation]
25824	LRRK1	SOS1	[activation]
25825	MVP	PTEN	[activation]
25826	TMED2	F2R	[activation]
25827	AGFG1	APP	[activation]
25828	PRKCB	CASR	[activation]
25829	MAPK8	HSF1	[activation]
25830	PPP2R2B	FTSJ1	[activation]
25831	BTRC	CDC20	[inhibition]
25832	WHSC1	HDAC1	[activation]
25833	TGFBI	COL1A2	[activation]
25834	TXNIP	RELA	[activation]
25835	SRC	MAPT	[activation]
25836	ZDHHC17	DIXDC1	[activation]
25837	MEF2A	PRKCQ	[activation]
25838	GNG11	FOS	[activation]
25839	CAV1	SH2D3C	[activation]
25840	YWHAB	GBF1	[activation]

25841	MYC	SIRT2	[activation]
25842	RHNO1	LZTS2	[activation]
25843	BCL6	PPARD	[activation]
25844	ITK	CBL	[activation]
25845	CCR5	CCL14	[activation]
25846	ARHGAP12	TRIP10	[activation]
25847	FHL2	ARHGAP9	[activation]
25848	PCDH10	ABI2	[activation]
25849	MECOM	FOS	[activation]
25850	PRKCSH	PTPRC	[activation]
25851	WDR43	NXF1	[activation]
25852	NME7	C1orf189	[activation]
25853	RALA	CCDC8	[activation]
25854	GALNT12	CDH1	[activation]
25855	TP53	DLEU1	[activation]
25856	EGFR	ARMC6	[activation]
25857	PRPSAP2	MAST3	[inhibition]
25858	MAP3K3	CALM1	[activation]
25859	POLD1	GSTK1	[activation]
25860	GRB2	SEPN1	[activation]
25861	CPNE4	SKIL	[activation]
25862	ESD	VCAM1	[activation]
25863	VAV1	AR	[activation]
25864	GRB2	DOCK4	[activation]
25865	AKAP4	PRKAR2B	[inhibition]
25866	CDC7	SUMO3	[activation]
25867	IRF3	SERPINB2	[inhibition]
25868	RXRA	TBP	[inhibition]
25869	EP300	HOXB3	[activation]
25870	CTDSP1	SNAI1	[activation]
25871	C16orf45	ENO2	[activation]
25872	GOLGA2	CCHCR1	[activation]
25873	MAPRE1	APC	[activation]
25874	GABRR1	PRKG1	[activation]
25875	SPP1	CACNG4	[activation]
25876	YWHAB	CDK14	[activation]
25877	TRAPPC3L	TRAPPC8	[activation]
25878	YWHAH	CDC25C	[activation]
25879	KCTD6	OBSCN	[activation]
25880	KPNB1	OBSL1	[activation]
25881	PPARA	NRIP1	[activation]
25882	CDK17	TGFBR1	[activation]
25883	SUMO4	NFKBIA	[activation]
25884	SMC3	APC	[inhibition]
25885	C1orf106	SFN	[activation]
25886	EP300	KAT2B	[activation]
25887	SNTA1	CALM1	[activation]
25888	BUB1	BAAT	[activation]
25889	SRPK2	SNRPA	[activation]
25890	FAM76B	SRPK2	[activation]
25891	RELB	HSD17B4	[activation]
25892	NCF4	MLH1	[activation]
25893	MYL6	CHUK	[activation]
25894	RB1	PPARG	[inhibition]
25895	DNAJA1	NFKB1	[inhibition]
25896	POLA2	ASCC2	[activation]
25897	ARRB1	PPM1A	[activation]
25898	PLK1	DNAJB9	[inhibition]
25899	TRIP10	PRKACA	[activation]
25900	HIST1H3A	PAXIP1	[activation]
25901	HDAC1	HSPA4	[inhibition]
25902	HECW2	TP53	[activation]
25903	HIST1H3A	H2AFX	[activation]
25904	MIF	ILK	[activation]
25905	BCL2L1	LARP1	[activation]
25906	MTTP	HSP90B1	[activation]
25907	SNAP25	SYT3	[activation]
25908	FAM46A	BCCIP	[activation]
25909	WFS1	MAPK6	[activation]
25910	EPHB6	CRK	[activation]
25911	EGFR	AGER	[activation]
25912	BMPR1A	FKBP1A	[inhibition]
25913	TMEM5	GPR3	[activation]
25914	NCOA3	CHUK	[activation]
25915	PIK3R1	IL1R1	[activation]
25916	STAG2	MYC	[activation]

25917	HIST1H2BD	CD81	[activation]
25918	ELOVL1	TWF2	[activation]
25919	CASP9	SRF	[activation]
25920	RECQL5	MCM7	[activation]
25921	SMURF1	TP53	[activation]
25922	PPARA	LAMTOR5	[activation]
25923	MYC	FOSL2	[activation]
25924	SLX4	PRKCI	[activation]
25925	SNTA1	MAPK12	[activation]
25926	DSEL	SPP1	[activation]
25927	RPL19	TP53	[activation]
25928	CAMK4	CAMKK1	[activation]
25929	ZNF706	RAC3	[activation]
25930	PIK3CA	IRS4	[activation]
25931	APP	CDK18	[activation]
25932	SARS2	RAD21	[activation]
25933	IQGAP1	ESR1	[activation]
25934	ABL1	CASP9	[activation]
25935	BAD	BCL2A1	[activation;inhibition]
25936	PLD2	RPTOR	[activation]
25937	CSNK2A2	CASQ2	[activation]
25938	CAV1	MAP1LC3B	[activation]
25939	CLSPN	RPA3	[activation]
25940	GGCX	PROC	[activation;inhibition]
25941	TRAF3	TRAIP	[activation]
25942	TP53	CRTC2	[activation]
25943	ELANE	F5	[activation]
25944	NOTCH1	HIF1A	[activation]
25945	WIPF2	GRB2	[activation]
25946	ICAM1	ACTN1	[activation]
25947	RANBP2	APC	[activation]
25948	TP53	AIMP2	[activation]
25949	RAC1	ARHGEF19	[activation]
25950	APP	PLEKHJ1	[activation]
25951	ADRB2	IDH3A	[activation]
25952	CRK	ABI1	[activation]
25953	ARHGEF6	GPRASP2	[activation]
25954	TOP1	TP53	[activation]
25955	RGS12	ARAF	[activation;inhibition]
25956	RAD50	MDC1	[activation]
25957	RAB5A	ALS2CL	[activation]
25958	SOCS3	CXCR4	[activation]
25959	SGTA	IGLC1	[activation]
25960	CTTN	PTK2	[activation]
25961	CDK5RAP3	CDH4	[activation]
25962	DIABLO	PRKCD	[activation]
25963	GREB1	SMAD4	[activation]
25964	XRCC3	ZNHIT3	[activation]
25965	STRADA	XPO1	[activation]
25966	RASA1	DLC1	[activation]
25967	SKIV2L2	MAPK11	[activation]
25968	CDC37	STK11	[activation]
25969	ARPC4	PNMA5	[activation]
25970	CBLB	MYD88	[activation]
25971	TRAPPC9	NXF1	[activation]
25972	RAD21	HSP90AA1	[activation]
25973	ITCH	RIPK1	[activation]
25974	PRKCB	STXBP1	[activation]
25975	MAPK11	ZNHIT1	[activation]
25976	CCL2	MMP3	[activation]
25977	CCNA2	PCNA	[activation]
25978	SRPK1	SON	[activation]
25979	ICAM1	IL2RA	[activation]
25980	PALD1	IRF7	[activation]
25981	FOS	ZFYVE9	[activation]
25982	NAT2	PALB2	[activation]
25983	CACNB1	ATN1	[inhibition]
25984	MAPK1	MAP2K4	[activation]
25985	HSF1	NCOA6	[activation]
25986	PPARG	KAT2A	[activation]
25987	FKBP2	C1QC	[activation]
25988	ITGB4	PLEC	[activation]
25989	TRAF6	CALM1	[activation]
25990	PRKCH	CDK2	[activation]
25991	NFATC2	MAPK9	[activation]
25992	CASP7	STK24	[activation]

25993	HABP4	PRKCQ	[activation]
25994	PPARG	CREBBP	[activation]
25995	C1QTNF1	AVPR2	[activation]
25996	GYS1	PRKACA	[activation;inhibition]
25997	CREM	PRKACA	[activation]
25998	SRPK1	FKBP3	[activation]
25999	NXF1	TPD52L2	[activation]
26000	KCNS3	HSP90AB1	[activation]
26001	BIRC5	CASP7	[activation]
26002	MET	GRB14	[activation]
26003	EIF4E	PAK2	[activation]
26004	WASF2	DOCK1	[activation]
26005	FER	EGFR	[activation;inhibition]
26006	MLH1	HSD17B3	[activation]
26007	SRGAP1	YWHAZ	[activation]
26008	TRIP10	RHOJ	[activation]
26009	MAST3	YWHAE	[activation]
26010	CALM1	TNF	[activation]
26011	GRIA4	EPB41L1	[activation]
26012	NGF	CRYAB	[activation]
26013	AKT1	DDX5	[activation]
26014	KDR	GNAQ	[activation]
26015	PAFAH1B1	MAP2	[activation]
26016	EP300	TTC5	[activation]
26017	CDK4	ANKRD12	[inhibition]
26018	CCR5	PTK2B	[activation]
26019	MAML1	PBRM1	[activation]
26020	ADRB2	SLC25A6	[activation]
26021	ESR1	FHL2	[activation]
26022	TRAF5	TNFRSF12A	[activation]
26023	RPS6KB1	STK11	[activation]
26024	CDC37	EIF2AK4	[inhibition]
26025	GATA6	CRIP2	[activation]
26026	MET	STAT3	[activation]
26027	SMAD3	PIAS2	[inhibition]
26028	KIF23	ECT2	[activation]
26029	ATF1	CREB1	[activation]
26030	STAT5B	STAT5A	[activation]
26031	KRAS	BCL2	[inhibition]
26032	SP1	MAN1A2	[activation]
26033	TNFRSF14	CD160	[activation]
26034	FAM219A	POLA2	[activation]
26035	SLC6A3	STX1A	[activation]
26036	SF3B5	SRPK3	[activation]
26037	AGPAT1	NXF1	[activation]
26038	PCP4	CALM1	[activation]
26039	PTK2	NEDD9	[activation]
26040	SPHK2	HIST1H3A	[activation]
26041	CAB39	RAD51	[activation]
26042	IGHA1	APC	[inhibition]
26043	AVPR1B	AVP	[activation]
26044	TCAP	FAS	[inhibition]
26045	PTK2	GZMB	[activation]
26046	ANXA7	F13A1	[activation]
26047	ABCF3	NXF1	[activation]
26048	NSUN4	MTERFD2	[activation]
26049	POLE	POLE2	[activation]
26050	TNS3	SRC	[activation]
26051	NCOA3	PIN1	[activation]
26052	GNB2	RAF1	[activation]
26053	MAPK6	CASP6	[activation]
26054	PRKACA	ITPKB	[activation]
26055	GRB2	VASP	[activation]
26056	PCK1	FBXO25	[activation]
26057	HMGA2	NPM1	[activation]
26058	HOXD13	SMAD1	[activation]
26059	GRM4	CALM1	[activation]
26060	SRSF8	SRPK2	[activation]
26061	PDHA2	MYC	[activation]
26062	EGFR	SCAMP1	[activation]
26063	HCK	ARRB1	[activation]
26064	CCHCR1	USHBP1	[activation]
26065	GSTK1	CPSF3	[activation]
26066	MDM2	KPNB1	[activation]
26067	IGHA1	CTR9	[activation]
26068	MAP2K4	STX17	[activation]

26069	SMAD4	UBE2I	[activation]
26070	MAPK14	GDF15	[activation]
26071	CCDC8	HSPA4	[inhibition]
26072	DAB1	ITGB2	[activation]
26073	GSPT1	ARF6	[activation]
26074	PPP2R1A	TP53	[activation]
26075	SUMO1	OGT	[activation]
26076	RAN	RNF2	[activation]
26077	TWF2	KRT36	[activation;inhibition]
26078	PACSIN1	DNM2	[activation]
26079	MAP3K2	YWHAG	[activation]
26080	PIK3R3	ERBB3	[activation]
26081	RHOJ	SMAD2	[activation]
26082	ERBB2	RASA1	[activation]
26083	PLCG1	DDR1	[activation]
26084	APP	PCDHA7	[activation]
26085	CDH13	ADIPOQ	[activation]
26086	EPAS1	CREBBP	[activation]
26087	PPP1CA	PPP1R3D	[activation;inhibition]
26088	ABR	ABL1	[activation]
26089	BIRC5	CDK1	[activation]
26090	HMGN1	NFE2	[activation]
26091	SMURF1	ARL14	[inhibition]
26092	APP	TRAIP	[activation]
26093	RAC1	TOM1	[activation]
26094	BMPR1A	FBP2	[activation]
26095	APCDD1	RXRB	[inhibition]
26096	YWHAB	ANKS1A	[activation]
26097	BUB1	C9orf156	[activation]
26098	CAPZA1	CHUK	[activation]
26099	STIM1	STIM2	[activation]
26100	PLXNA3	PPP2CB	[activation]
26101	MYC	NFYC	[activation]
26102	EIF2S2	CSNK2B	[activation]
26103	HSPA6	TNFRSF1A	[activation]
26104	PAXIP1	CPSF3	[activation]
26105	LYN	EVL	[activation]
26106	MTRF1	MYC	[activation]
26107	IKZF3	BCL2L1	[activation]
26108	SKP1	TBK1	[inhibition]
26109	MYOD1	C2orf88	[activation]
26110	TXNDC17	INS	[activation]
26111	PTPN11	FCGR2B	[activation]
26112	DAPK1	STX1A	[activation]
26113	HSPD1	GRB2	[activation]
26114	EIF4A1	OBSL1	[activation]
26115	C1QTNF9	EIF4G2	[activation]
26116	ICAM1	RPL9	[activation]
26117	FAM210A	NXF1	[activation]
26118	MDC1	ANAPC7	[activation]
26119	HARS2	ICT1	[activation]
26120	CREBBP	ESR1	[activation]
26121	LRRK2	HSPD1	[activation]
26122	PALB2	RBBP8	[activation]
26123	PDGFA	COL1A1	[activation]
26124	EIF2B2	EIF2B5	[activation]
26125	LRRK2	ZZZ3	[activation]
26126	ITGB1	TIMP2	[activation]
26127	HDAC4	GNB1	[activation]
26128	AHSG	CDC42	[activation]
26129	PIM2	HSP90AA1	[inhibition]
26130	PICALM	SNW1	[activation]
26131	UBTF	HEMGN	[activation]
26132	CDK4	TRAP1	[activation]
26133	CD4	SPG21	[activation]
26134	MYLK	CTTN	[inhibition]
26135	EPSTI1	FGFR4	[activation]
26136	MAPK1	PECAM1	[activation]
26137	MARK3	YWHAZ	[activation]
26138	PAK4	CDC5L	[activation]
26139	HSPA4	CALM1	[activation]
26140	HLA-A	EGFR	[activation]
26141	KDR	HSPG2	[activation]
26142	AGAP10	HSPB1	[activation]
26143	GIT1	SRC	[activation]
26144	ERVK6	PYCARD	[inhibition]

26145	BAZ1B	SMARCA5	[activation]
26146	PRKAB1	MIF	[activation]
26147	FAM127B	HSPB3	[activation]
26148	CHUK	HSPA9	[activation]
26149	MAP3K1	MAPK3	[activation]
26150	CD247	NCR3	[activation]
26151	RAF1	EFEMP1	[activation]
26152	PNMAL1	PARD6G	[activation]
26153	HSPB1	PRKD1	[activation]
26154	RAB5A	RIT2	[activation]
26155	LAMTOR1	RRAGD	[activation]
26156	MRPS11	SRPK2	[activation]
26157	CAMKMT	HSP90AB1	[activation]
26158	RXRB	NR1H2	[inhibition]
26159	SMARCA5	PYHIN1	[activation]
26160	RELA	ATF4	[activation]
26161	HSPA4	SMAD2	[inhibition]
26162	CDC25B	CDC14A	[activation]
26163	SH3KBP1	GRB2	[activation]
26164	KRTAP19-5	DAB1	[activation]
26165	PPP6C	NOS2	[activation]
26166	MCC	RAP1B	[activation]
26167	PARD3	TIAM2	[activation]
26168	STAT3	CTR9	[activation]
26169	HIST1H4A	NCOA2	[activation]
26170	ATR	MCM7	[activation]
26171	CCS	SOD1	[inhibition]
26172	CDH7	CDH12	[activation]
26173	CRK	CELSR2	[activation]
26174	FOXL1	BMPR2	[activation]
26175	UBE2E2	RNF114	[activation]
26176	AKT2	CHEK1	[activation;inhibition]
26177	RPAP2	DNM2	[activation]
26178	AJUBA	BUB1B	[inhibition]
26179	MEGF6	EGFR	[activation]
26180	VAMP1	VAPB	[activation]
26181	MCM5	NFKBIA	[activation]
26182	HECW2	KMT2A	[activation]
26183	SRPK2	EGLN2	[activation]
26184	ESR2	RRP1	[activation]
26185	NUAK2	SMAD2	[activation]
26186	MDC1	PRIM1	[activation]
26187	EGFR	CDCP1	[activation]
26188	YWHAH	TNK1	[activation]
26189	SUMO3	ARPC5	[activation]
26190	HSPA4L	HSF1	[activation]
26191	SATB1	CASP6	[activation]
26192	FBXO25	KDR	[activation]
26193	RB1	TRAP1	[activation]
26194	PRKACA	RGS10	[activation;inhibition]
26195	TP53	PCDHA4	[activation]
26196	FZR1	TACC3	[inhibition]
26197	DDX24	MYC	[activation]
26198	MAGED1	NUMBL	[inhibition]
26199	MAGI2	DSCAML1	[activation]
26200	ARRB1	MAPK1	[activation]
26201	CSNK1D	ARFGAP1	[activation]
26202	EPOR	KIT	[activation]
26203	TIAM1	ANK1	[activation]
26204	PLK1	ANKRD28	[activation]
26205	RPS17	NUDC	[activation]
26206	JAK2	IKBKG	[activation]
26207	RAB14	RPA1	[activation]
26208	CTNNB1	FHL2	[activation]
26209	FZD4	CRELD2	[activation]
26210	CYFIP2	ABI3	[activation;inhibition]
26211	DDX47	EGFR	[activation]
26212	RSPO2	SUV39H1	[activation]
26213	ERAS	PIK3R1	[activation]
26214	FGFR2	PIK3R1	[activation]
26215	MYC	SP1	[activation]
26216	CD81	LUC7L2	[activation]
26217	YWHAE	CDC5L	[activation]
26218	TYK2	IRS2	[activation]
26219	CASP4	CDK2	[activation]
26220	WDR5	TWIST1	[activation]

26221	C2orf44	PDPK1	[activation]
26222	EIF4A3	CCDC9	[activation]
26223	RXRB	DNAJB14	[inhibition]
26224	CCR5	CXCR4	[activation]
26225	TNFRSF1A	ADAM17	[activation]
26226	GRB2	AHSG	[activation]
26227	A2M	FARSA	[inhibition]
26228	CISH	CCNE1	[inhibition]
26229	PRMT7	HIST1H3A	[activation]
26230	RPS6	CD81	[activation]
26231	MCM7	GFPT1	[activation]
26232	PIKFYVE	SUMO1	[activation]
26233	MED1	GATA6	[activation]
26234	SMAD3	E2F4	[inhibition]
26235	TGFB1	WHSC1L1	[activation]
26236	TAGLN2	RPA3	[activation]
26237	AR	ESR1	[activation]
26238	YWHAZ	KPNB1	[activation]
26239	ROPN1	RHPN1	[activation;inhibition]
26240	NAPB	STX5	[activation]
26241	RALGAPB	RALGAPA2	[activation;inhibition]
26242	NTPCR	LRRK2	[activation]
26243	NCOA1	FOXA2	[activation]
26244	MYO1G	CD247	[activation]
26245	HSP90AB1	CDK4	[activation]
26246	BCL6	TWIST1	[activation]
26247	ZFP36L1	MAPK14	[activation]
26248	GPR3	ARRB1	[activation]
26249	JAM3	ITGAX	[activation]
26250	VCAM1	IPO7	[activation]
26251	SMAD2	HIPK2	[activation]
26252	GCN1L1	PHLDA3	[activation]
26253	MDM2	VAPB	[activation]
26254	NTRK2	KIDINS220	[activation]
26255	APH1B	PSEN2	[activation]
26256	FKBPL	IRS4	[activation]
26257	TLR7	GRAMD1A	[activation]
26258	TNRC6C	PAN3	[activation]
26259	NOC3L	SRPK3	[activation]
26260	A2M	ADAMTS1	[inhibition]
26261	MAGEA6	EIF2B5	[activation]
26262	ADM	MME	[activation]
26263	NUMBL	APP	[inhibition]
26264	TTC1	MOS	[inhibition]
26265	VCAM1	RAN	[activation]
26266	GSTK1	CMSS1	[activation]
26267	SH3BP1	HCK	[activation]
26268	STX4	SNAP23	[activation]
26269	CHRD	SMAD3	[inhibition]
26270	GABARAPL1	DOK1	[activation]
26271	JAK2	SH2B1	[activation]
26272	ESR1	PRMT1	[activation]
26273	LYN	BTK	[activation]
26274	TGFBI	A2M	[inhibition]
26275	FADD	RAP1A	[activation]
26276	COMMD1	SPP1	[activation]
26277	HCK	CSF2RB	[activation]
26278	MAPK14	PTPN7	[activation]
26279	IFNA5	APP	[activation]
26280	VAPA	VAPB	[activation]
26281	ATP9A	ESR1	[activation]
26282	EGFR	DNAJC4	[activation]
26283	OGT	NUP62CL	[activation]
26284	ERBB3	VAV3	[activation]
26285	CDKN1C	RB1	[inhibition]
26286	PIK3R1	CD7	[activation]
26287	SNCA	GSK3B	[activation]
26288	IRAK3	CD14	[inhibition]
26289	FYB	BCAR1	[activation]
26290	TEK	ANGPTL1	[activation]
26291	EIF1B	EIF4H	[activation]
26292	STK39	PRKCQ	[activation]
26293	WRAP73	MARS	[activation]
26294	OBSL1	EP400	[activation]
26295	IL12A	EBI3	[activation]
26296	GSK3B	RNF219	[inhibition]

26297	RTEL1	RPA1	[activation]
26298	SKAP1	LYN	[activation]
26299	RRAGC	RRAGD	[activation]
26300	DEK	CREBBP	[activation]
26301	RPS26P11	EGFR	[activation]
26302	NMNAT1	NTRK2	[activation]
26303	ICAM2	EZR	[activation]
26304	JUNB	SUMO1	[activation]
26305	GPR37L1	PRCP	[activation]
26306	NR1H3	APP	[activation]
26307	BOC	CTNNB1	[activation]
26308	APP	A2M	[inhibition]
26309	TOLLIP	TGFBR1	[activation]
26310	MAVS	IRF5	[activation]
26311	PDPK1	SOCS3	[activation]
26312	MYL9	PRKAA1	[inhibition]
26313	S1PR3	HTR1A	[activation]
26314	EGFR	EIF4G2	[activation]
26315	HAVCR1	SEMA4A	[activation]
26316	LEF1	NRAS	[activation]
26317	NFKB1	STAT3	[activation]
26318	CD27	TRAF3	[activation]
26319	TWF2	VRK3	[activation]
26320	TFRC	ESR1	[activation]
26321	IGF2BP1	GABARAPL2	[activation]
26322	DDX58	IKBKG	[activation]
26323	PLCG2	JAK1	[activation]
26324	SMAD4	MEF2A	[activation]
26325	CHRM2	GRK5	[activation]
26326	ATP7B	ATOX1	[activation]
26327	DVL2	ARHGEF39	[activation]
26328	MAST2	TRAF2	[activation]
26329	CCNE1	CDK4	[activation]
26330	SNAI2	HDAC2	[inhibition]
26331	STAT3	MAP3K13	[activation]
26332	PPP2CA	PPP1CA	[inhibition]
26333	SIRT6	RAD50	[activation]
26334	NSA2	IRAK3	[inhibition]
26335	HSPA1A	MOS	[inhibition]
26336	RALB	PLCD1	[activation]
26337	RAD21	RPA2	[activation]
26338	SNAP23	STX1A	[activation]
26339	MAGEH1	ELN	[activation]
26340	PRNP	SURF2	[activation]
26341	FYN	LCP2	[activation]
26342	RFC4	PAXIP1	[activation]
26343	GNAI3	SUMO2	[activation;inhibition]
26344	TNFRSF8	BCL6	[activation]
26345	GADD45G	PLEKHM1	[activation]
26346	PSEN1	CASP1	[activation]
26347	LGR4	KMT2A	[activation]
26348	ATM	POLR2A	[activation]
26349	ESR1	SUV39H1	[activation]
26350	CRK	FRS2	[activation]
26351	TRADD	DAPK1	[activation]
26352	IKBKB	MEOX2	[activation]
26353	GRB10	MAPK1	[activation]
26354	PLEKHA7	YWHAB	[activation]
26355	KRT18	CASP3	[activation]
26356	YAP1	LIN7C	[inhibition]
26357	LRRK2	ABL2	[activation]
26358	AKAP12	TP53	[activation]
26359	TIMELESS	ATR	[activation]
26360	PIK3R1	CKAP5	[activation]
26361	PPP2CA	PPP2R4	[activation]
26362	LRP6	ATP13A2	[activation]
26363	VAV2	BRDT	[activation]
26364	CELA1	PI3	[inhibition]
26365	POLD2	POLD4	[activation]
26366	HNRNPH2	SRPK2	[activation]
26367	LPA	CELA1	[activation]
26368	HSP90AA1	RHOBTB1	[activation]
26369	PTH1R	CTNNB1	[activation]
26370	MAP2K3	PPP2R4	[activation]
26371	POLD1	CDK4	[activation]
26372	CUL7	ANXA5	[activation]

26373	UBE2E3	RMND5B	[activation]
26374	SUMO3	ATF4	[activation]
26375	CDKN1C	PCNA	[inhibition]
26376	RFC4	TP53	[activation]
26377	DIAPH2	APBB1	[activation]
26378	TAB1	BIRC3	[activation;inhibition]
26379	BRK1	PCDH8	[activation]
26380	ENOX1	TRIM43	[activation]
26381	PPP2R1A	SMURF1	[inhibition]
26382	RPA1	ZNF598	[activation]
26383	SETDB1	MPHOSPH8	[activation]
26384	CNKSR1	PIN1	[activation]
26385	GATA4	JARID2	[activation]
26386	CCDC28A	MYOG	[activation]
26387	NDC80	BUB1B	[inhibition]
26388	CR1	C4A	[inhibition]
26389	ARRB2	BOLA2	[inhibition]
26390	TCEB1	EFNB3	[activation]
26391	HCK	PIK3R1	[activation]
26392	GDF11	WFIKKN2	[activation]
26393	TFRC	VCAM1	[activation]
26394	SRPRB	CASP4	[activation]
26395	PARD3	YWHAH	[activation]
26396	CRKL	ABI1	[activation]
26397	APP	RASA3	[activation]
26398	PRKAA1	USP2	[inhibition]
26399	H2AFX	ERO1L	[activation]
26400	LIFR	POU2F1	[activation]
26401	DNAH9	BCL6	[activation]
26402	PAX6	PROX1	[activation]
26403	ZMYM3	CDK6	[inhibition]
26404	PIN1	CDC27	[activation]
26405	SMARCA2	CCNE1	[activation]
26406	ANXA2	APOH	[activation]
26407	GPHA2	GPHB5	[activation]
26408	SRPK1	RBM23	[activation]
26409	RANBP1	HLA-B	[activation]
26410	DDX5	YWHAZ	[activation]
26411	ITCH	SFN	[activation]
26412	SMO	ARRB2	[inhibition]
26413	YY1	TWIST1	[activation]
26414	LRRK2	DBN1	[activation]
26415	AURKB	BUB1B	[activation]
26416	MAPK14	FLNA	[activation]
26417	CDK17	YWHAB	[activation]
26418	ANGPTL3	ITGA5	[activation]
26419	RPAP3	LRCH2	[activation]
26420	YWHAB	WEE1	[activation]
26421	NUCB2	CASP7	[activation]
26422	RAG2	IPO5	[activation]
26423	HEATR2	DNAI2	[inhibition]
26424	ARRB2	MAP3K7	[activation]
26425	FER	CTTN	[activation;inhibition]
26426	CDC20	E2F1	[inhibition]
26427	APP	MAST2	[activation]
26428	GRB2	PHC2	[activation]
26429	AKT1	PDHB	[activation]
26430	NXF1	ZNFX1	[activation]
26431	EGFR	ANKS1B	[activation]
26432	YWHAE	HIST1H4A	[activation]
26433	PRKACA	CDK16	[activation]
26434	RAC1	ARHGAP44	[activation]
26435	RCC1	MDM2	[activation]
26436	ILK	NUP205	[activation]
26437	CD151	MMP7	[activation]
26438	ATF2	YWHAB	[activation]
26439	PTPRJ	WAS	[activation]
26440	REL	BID	[activation]
26441	AURKB	TGFBR1	[activation]
26442	AFF1	HIST3H3	[activation]
26443	ROBO2	RPS6KA5	[activation]
26444	SHANK2	PIK3R1	[activation]
26445	CSNK1D	MCC	[activation]
26446	IL2RG	STK4	[activation]
26447	OSGIN1	XRCC3	[activation]
26448	RPS6KA6	RBPJ	[activation]

26449	FBXO6	SERPINB1	[inhibition]
26450	ERO1LB	UBC	[activation]
26451	NAPA	STX7	[activation]
26452	PYGM	DNM2	[activation]
26453	GRB2	WDR44	[activation]
26454	HIST2H2AC	TSSK6	[activation]
26455	PPP1CC	LATS1	[inhibition]
26456	VCAM1	CFL1	[activation]
26457	TRAF6	BUB3	[activation]
26458	BRCA1	EP300	[activation]
26459	LTK	PLCG1	[activation]
26460	TG	HSP90B1	[activation]
26461	DEFA1	GSK3B	[inhibition]
26462	CD79B	LYN	[activation]
26463	NOTCH2NL	LCE2D	[activation]
26464	PSEN2	RHEB	[activation]
26465	RAD50	FBXO6	[activation]
26466	NFATC2	EP300	[activation]
26467	PLCG2	KIT	[activation]
26468	RAG2	RAG1	[activation]
26469	ATP4A	STX3	[activation]
26470	RAC1	CAV1	[activation]
26471	C1orf123	ANXA1	[activation]
26472	PPP2R1A	DAPK1	[inhibition]
26473	HELZ	SMYD3	[activation]
26474	EPB41	RAB5C	[activation]
26475	PPP2R1A	NOTCH1	[activation]
26476	NOTCH2NL	KLHL38	[activation]
26477	NCK1	EIF2S2	[activation]
26478	DCC	EIF4E3	[inhibition]
26479	RAD21	EIF2S1	[activation]
26480	PTPRB	GAB1	[activation]
26481	SPTAN1	GRIA2	[activation]
26482	JAK3	TYK2	[activation]
26483	BMPR1B	USP45	[activation;inhibition]
26484	ACTG1	GZMK	[activation]
26485	PTPRO	INSR	[activation]
26486	TRAF2	VWA2	[activation]
26487	EXOC1	MACF1	[activation]
26488	BUB1B	SNCG	[inhibition]
26489	HSPB1	PNISR	[activation]
26490	SFN	SIPA1L3	[activation]
26491	SMAD2	FHL2	[inhibition]
26492	MEF2A	CDK5	[activation]
26493	EZH2	CIT	[activation]
26494	KLHL1	HSP90AB1	[activation]
26495	TP53	PLOD3	[activation]
26496	AAK1	APP	[activation]
26497	FGB	FGG	[activation]
26498	ILK	CCT7	[activation]
26499	TBL3	NUDC	[activation]
26500	RPTOR	PRR5L	[activation;inhibition]
26501	EIF3J	CASP3	[activation]
26502	COL4A4	KLK6	[activation]
26503	ECSIT	PI4K2A	[activation]
26504	MAP3K7	PPM1L	[activation]
26505	MMP16	KISS1	[activation]
26506	SSH1	ELAVL1	[activation]
26507	CAMK2A	ATP2A2	[activation]
26508	RAP2B	RAPGEF2	[activation]
26509	RHOG	FBXO6	[activation]
26510	GRN	NLK	[activation]
26511	TRPC3	GNA11	[activation]
26512	JAK2	LYN	[activation]
26513	TP53	TRMT10C	[activation]
26514	MAPK8	NUP98	[activation]
26515	HIPK2	MKNK1	[activation]
26516	ANXA2	HLA-B	[activation]
26517	RAC1	ANGPTL4	[activation]
26518	PYCARD	PYDC2	[inhibition]
26519	NUAK1	CASP6	[activation]
26520	FMNL1	CORO1C	[activation]
26521	TRADD	TNFRSF10B	[activation]
26522	SMAD2	FOS	[activation]
26523	KRT31	SHC3	[activation]
26524	MAGED1	PCK1	[activation]

26525	PPP2CA	SETBP1	[inhibition]
26526	BAD	BAX	[inhibition]
26527	RPS6KB1	MDM2	[activation]
26528	SPEF2	IGHA1	[activation]
26529	COPS7A	TP53	[activation]
26530	VCL	C19orf57	[activation]
26531	GABARAPL2	RANBP2	[activation]
26532	TIPIN	TIMELESS	[activation]
26533	UTS2	UTS2R	[activation;inhibition]
26534	RAB7L1	GAK	[inhibition]
26535	GADD45B	MAP2K7	[activation]
26536	HSPB1	GTSF1	[activation]
26537	ANAPC1	SOX2	[activation]
26538	SMAD3	SREBF2	[activation]
26539	MAP1LC3B	TP53	[activation]
26540	HDAC1	ESR1	[activation]
26541	GDI1	HLA-B	[activation]
26542	CLOCK	KAT2B	[activation]
26543	PACSIN1	CYFIP2	[activation;inhibition]
26544	PHB	TP53	[activation]
26545	YWHAG	CAMKK1	[activation]
26546	BMP7	KRTAP10-8	[activation]
26547	RIPK2	KDM3A	[activation]
26548	ELAVL1	GRB2	[activation]
26549	ECT2	GRB2	[activation]
26550	CRKL	RAF1	[activation]
26551	BLK	EEF2	[activation]
26552	UBE2N	VCAM1	[activation]
26553	GPR25	MAP3K7	[activation]
26554	CDC27	CCND1	[activation]
26555	BEND5	DDR1	[activation]
26556	SNCA	TUBA1A	[activation]
26557	EIF3D	GRB2	[activation]
26558	MAPK6	MDK	[activation]
26559	FOSL1	CREBBP	[activation]
26560	ATR	HSPA4	[activation]
26561	VCAM1	YTHDF2	[activation]
26562	TP53	MAGED2	[activation]
26563	NGFR	PIAS2	[activation]
26564	HSPB1	DMWD	[inhibition]
26565	KDELR1	WBP5	[activation]
26566	HSP90AA1	IRAK3	[inhibition]
26567	HIST3H3	TNFRSF1A	[activation]
26568	APC	TMEFF1	[inhibition]
26569	KANSL1	TRAF2	[activation]
26570	CCDC8	F11R	[activation]
26571	MAPT	CDK5R1	[activation]
26572	APEX1	ARF6	[activation]
26573	ILK	HNRNPL	[activation]
26574	SUPT20H	HIST3H3	[activation]
26575	GRB2	TCEAL8	[activation]
26576	JUP	ZFYVE9	[activation]
26577	TRAF6	YES1	[activation]
26578	CCNB2	MCM2	[activation]
26579	CREBBP	MDM2	[activation]
26580	RBL1	CCNA1	[activation;inhibition]
26581	HIPK2	MBP	[activation]
26582	CDK19	AZGP1	[activation]
26583	MAP2K1	CPNE4	[activation]
26584	RPS6KA5	ITSN1	[activation]
26585	HRAS	RASIP1	[activation]
26586	RGS20	INS	[activation]
26587	ESR1	VPS13D	[activation]
26588	HIST2H3A	ICAM1	[activation]
26589	CD81	TUBA8	[activation]
26590	OBSL1	CAMKK2	[activation]
26591	MOV10	ACACA	[activation;inhibition]
26592	TRAF6	IL17RA	[activation]
26593	RAP1B	RGL2	[activation]
26594	MYC	MYO5C	[activation]
26595	NUMB	EPS15	[activation]
26596	TXK	KIT	[activation]
26597	BCR	DOK1	[activation]
26598	XRCC3	FANCG	[activation]
26599	CLDN3	CTSS	[activation]
26600	IRS4	HSPB1	[activation]

26601	PRKCD	HSP90AA1	[activation]
26602	HSPD1	TP53	[activation]
26603	PRKCB	RIPK4	[activation]
26604	WDFY3	ATG5	[activation]
26605	SNAP29	ATF4	[activation]
26606	ARL8B	APP	[activation]
26607	SMARCB1	GABARAP	[activation]
26608	KHDRBS1	CRKL	[activation]
26609	MDM2	EIF4A1	[activation]
26610	RIMS2	GRB2	[activation]
26611	LAMB2	KPNA2	[activation]
26612	IQCB1	HSPA6	[inhibition]
26613	GAPDH	MAPK7	[activation]
26614	ZNF83	PAK1	[activation]
26615	FES	EZR	[activation]
26616	MAD2L1	TP73	[activation]
26617	PPP2R1A	VCAM1	[activation]
26618	RAB5C	NXF1	[activation]
26619	NCAPH	SMC4	[activation]
26620	NLRP4	NLRC4	[activation]
26621	PAX6	EN1	[activation]
26622	UBE2I	PRKDC	[activation]
26623	PRKCDBP	MRFAP1L1	[activation;inhibition]
26624	STAT5A	CTLA4	[activation]
26625	LCK	PECAM1	[activation]
26626	EXTL3	GRB2	[activation]
26627	CDKN1A	FKBPL	[activation;inhibition]
26628	MDM2	PFN1	[activation]
26629	PBX3	ZNHIT3	[activation]
26630	RAD52	RPA1	[activation]
26631	WAS	ITSN1	[activation]
26632	CYCS	IKBKE	[inhibition]
26633	GBF1	PTP4A3	[inhibition]
26634	MAPT	LIMS1	[activation]
26635	MAP3K8	RELA	[activation]
26636	STX4	STXBP6	[activation]
26637	HSPB1	YWHAQ	[activation]
26638	PAK2	CASP7	[activation]
26639	SFN	KIAA0408	[activation]
26640	PPM1B	FOS	[activation]
26641	DIMT1	ITGA4	[activation]
26642	SNRNP70	RELA	[activation]
26643	BARD1	GIT1	[activation]
26644	CDC23	CDC5L	[activation]
26645	CDK5	SRC	[activation]
26646	RAB22A	APPL1	[activation]
26647	SGTA	REC8	[activation]
26648	ARRB2	IGKC	[activation]
26649	ANXA7	GABARAPL1	[activation]
26650	FBXO6	PLOD2	[activation]
26651	VCAM1	RPS28	[activation]
26652	NLRC5	CHUK	[activation]
26653	TRAF6	TXN	[activation]
26654	MRVI1	MME	[activation]
26655	ARF6	MEPCE	[activation]
26656	CDC16	FBXO5	[activation]
26657	ANAPC1	CDC20	[activation]
26658	TLR3	TOLLIP	[activation]
26659	NFAT5	PTPN6	[activation;inhibition]
26660	HMGB1	POU5F1	[activation]
26661	CAP1	ATF2	[activation]
26662	TBC1D15	RAB5C	[activation]
26663	PCNA	LIG1	[activation]
26664	PML	MYB	[activation]
26665	PPM1G	HIST3H3	[activation]
26666	PRKCA	RGS2	[activation]
26667	AVP	AVPR2	[activation]
26668	NCOR2	CHUK	[activation]
26669	LYN	BANK1	[activation;inhibition]
26670	TLK1	CHEK1	[activation;inhibition]
26671	YWHAZ	SHC1	[activation]
26672	SRPK1	USP39	[activation]
26673	TRAF6	RPA3	[activation]
26674	FYN	PRKCQ	[activation]
26675	PARD6A	SMURF1	[inhibition]
26676	EGFR	CALM1	[activation]

26677	S100A7	EGFR	[activation]
26678	ETS1	NCOR1	[activation]
26679	BAK1	KRT31	[activation;inhibition]
26680	EIF2S3	EIF2S1	[activation]
26681	SFN	BCAR1	[activation]
26682	RNF114	TRAF6	[activation]
26683	SH2D1A	ARHGEF7	[activation]
26684	YWHAZ	HNRNPL	[activation]
26685	PTEN	IRS4	[activation]
26686	GIMAP8	LRRK2	[activation]
26687	CD4	HNRNPA1	[activation]
26688	AHCYL1	ITPR1	[inhibition]
26689	CREBBP	CRX	[activation]
26690	VEGFA	SEMA3F	[activation]
26691	TRPV6	PRKCA	[activation]
26692	MSH5	MSH4	[inhibition]
26693	CCND3	PPP1CC	[inhibition]
26694	CCDC57	CDC20B	[activation]
26695	LIN9	NXF1	[activation]
26696	NR1H3	NCOA3	[activation]
26697	DOK2	EPO	[activation]
26698	ARHGEF4	RAC1	[activation]
26699	RXRA	NCOR2	[inhibition]
26700	RAC1	TNFAIP8L2	[activation]
26701	RORC	PPP2CA	[inhibition]
26702	POLR2A	GTF2A2	[activation]
26703	ARRB2	CTTN	[inhibition]
26704	CFTR	STX1A	[activation]
26705	STAU1	ARPC4	[activation]
26706	EIF5	CSNK2B	[activation]
26707	NCKAP1	ABI1	[inhibition]
26708	MYC	DIRAS2	[activation]
26709	CLNK	EGFR	[activation]
26710	RAF1	PAK3	[activation]
26711	TF	IGKC	[activation]
26712	AMT	HSPA8	[activation]
26713	SHD	MET	[activation;inhibition]
26714	PFN2	SNTA1	[activation]
26715	MET	PASK	[activation]
26716	PIK3R1	CXCL2	[activation]
26717	PTPRC	ZAP70	[activation]
26718	EZH2	RPS6KA5	[activation]
26719	ZNHIT1	TP53	[activation]
26720	HDAC6	MAPT	[activation]
26721	YBEY	EGFR	[activation]
26722	CCDC33	TRAF2	[activation]
26723	NUP98	NUP62	[activation]
26724	MYC	IPO7	[activation]
26725	STX1A	SNAP29	[activation]
26726	HLA-B	DIABLO	[activation]
26727	YWHAH	HSPA8	[inhibition]
26728	PTPN23	EGFR	[activation]
26729	PARD6G	YWHAE	[activation]
26730	HSP90AB1	TRIM73	[activation]
26731	ITGB2	DOK1	[activation]
26732	TGIF1	HMGB1	[inhibition]
26733	TNFRSF1A	GRN	[activation]
26734	APP	AIFM3	[activation]
26735	PRIM2	SUV39H1	[activation]
26736	DIAPH1	CLIP1	[activation]
26737	CAMKK2	HSP90AA1	[activation]
26738	RCC1	CDK1	[activation]
26739	MS4A1	IGHM	[activation]
26740	ACTG1	ACTB	[activation]
26741	RPA3	SLFN11	[activation]
26742	HDAC1	HSF1	[activation]
26743	ANAPC7	SOX2	[activation]
26744	TFE3	TFEB	[activation]
26745	IRAK1	VASP	[activation;inhibition]
26746	PIK3CA	TNFRSF1A	[activation]
26747	NANS	STK11	[activation]
26748	RHOBTB2	STK11	[activation]
26749	MIF	CFLAR	[activation]
26750	LYN	PILRB	[activation]
26751	MAPT	PPP2CB	[activation]
26752	CASP1	PLA2G4A	[activation]

26753	CRMP1	IL33	[activation]
26754	RNF41	C1orf109	[activation]
26755	CAMK2A	ZMYM1	[activation]
26756	BCL6	TRIB3	[activation]
26757	MBP	CTDSP1	[activation]
26758	PTPLAD1	CASP4	[activation]
26759	PYY	NPY1R	[activation]
26760	CRK	PCDHA5	[activation]
26761	MAP4K4	PRKCE	[activation]
26762	FAS	ANK3	[inhibition]
26763	USP38	HSPB1	[inhibition]
26764	FYN	VPS13A	[activation]
26765	NAT2	CDH1	[activation]
26766	ERBB4	NCK2	[activation]
26767	RAD51	RAD51C	[activation]
26768	CASP6	TAB1	[activation]
26769	NOP2	GABARAPL2	[activation]
26770	DAPK1	KRIT1	[activation]
26771	PPARG	CIITA	[activation]
26772	HMGB1	RB1	[inhibition]
26773	RAB11A	TBC1D14	[activation]
26774	MMP24	KISS1	[activation]
26775	CISD1	NR4A2	[activation]
26776	VCAM1	ASNS	[activation]
26777	FBXW7	CSF3R	[activation]
26778	DYSF	FLNC	[activation]
26779	RARA	STAT1	[activation]
26780	PELI3	IRAK1	[activation;inhibition]
26781	RAB5C	TBC1D17	[activation]
26782	TACC2	FTSJ1	[activation]
26783	CDC37	MAPK7	[activation]
26784	SMAD3	LEF1	[inhibition]
26785	DLGAP4	PLK2	[activation]
26786	STAT5B	ETS1	[activation]
26787	ARHGDIG	CEP170P1	[activation]
26788	C1orf109	USHBP1	[activation]
26789	ZNF408	MIF	[activation]
26790	RIN1	EGFR	[activation]
26791	EVI5L	RAB10	[activation]
26792	RASA1	STK11	[activation]
26793	PAX6	SMARCA4	[activation]
26794	SERPINA5	FGA	[inhibition]
26795	NR1D1	NR2E3	[inhibition]
26796	DDX24	IDO1	[activation]
26797	MYB	CLTC	[activation]
26798	OBSL1	EIF4A3	[activation]
26799	SLC8A1	CAV3	[inhibition]
26800	HEATR2	ILK	[activation]
26801	CDKN2A	ACTG1	[activation]
26802	CAV1	ADRBK1	[activation]
26803	NCK1	NOTCH3	[activation]
26804	EPHA3	STAT3	[activation]
26805	ARRB2	PPIA	[inhibition]
26806	KATNB1	TGFBR1	[activation]
26807	TMEM184C	SGK1	[activation]
26808	PYCRL	TP53	[activation]
26809	TLR2	CXCR4	[activation]
26810	PPP1CC	HSPA4	[inhibition]
26811	TRHDE	AKT1	[activation]
26812	KRCC1	PPP1CC	[inhibition]
26813	CDKN2A	HIST3H2BB	[activation;inhibition]
26814	PML	FOS	[activation]
26815	HSPA8	ARRB2	[inhibition]
26816	H2AFX	MAPK8	[activation]
26817	GSK3B	NRBP1	[activation]
26818	MORN4	BEND3	[activation]
26819	PTK6	FCHO1	[activation]
26820	RASA1	APC	[activation]
26821	KAT2B	HIST1H2AB	[activation]
26822	TRADD	CALM1	[activation]
26823	RAB5A	TBC1D15	[activation]
26824	TAGLN	CFL1	[activation;inhibition]
26825	SERTAD1	PPP2R1A	[inhibition]
26826	VCAM1	ITGA5	[activation]
26827	RASGRP3	SULT1C2	[activation]
26828	DAPK1	PKM	[activation]

26829	SH2B2	SYK	[activation]
26830	SRMS	HSPB1	[activation]
26831	CDK3	E2F1	[activation]
26832	SERPINF2	TGM2	[activation]
26833	GTF2H2	CCNH	[activation]
26834	PTGES3	DNAJB1	[inhibition]
26835	CHN1	GTF3C1	[activation]
26836	IRF3	TRAF3	[activation]
26837	NEDD4	SMO	[inhibition]
26838	S100A10	ANXA2	[activation]
26839	TRIP13	PPP2CA	[inhibition]
26840	BCL6	HSP90AA1	[activation]
26841	GRIPAP1	SPP1	[activation]
26842	GNB2	ESR1	[activation]
26843	FOS	ARL6IP4	[activation]
26844	RPS6KB1	EIF4E	[activation]
26845	HIST1H3A	PRKCA	[activation]
26846	CALM1	SYK	[activation]
26847	ATF2	ANXA1	[activation]
26848	CCL2	MMP8	[activation]
26849	BID	GBP2	[activation]
26850	PRKACA	MAP2	[activation]
26851	COLQ	MUSK	[activation]
26852	PTEN	TTBK2	[activation]
26853	RIPK1	TNFRSF10A	[activation]
26854	H2AFX	BRD1	[activation]
26855	CAPZB	MDM2	[activation]
26856	GRIN2A	PTK2B	[activation]
26857	NTRK2	EGFR	[activation]
26858	TXNDC17	CFL1	[activation;inhibition]
26859	TP53	ECD	[activation]
26860	SMARCE1	NUP62	[activation]
26861	AOX1	CRKL	[activation]
26862	KAT2B	JDP2	[activation]
26863	KCNA3	BAX	[activation]
26864	IKBKG	MYD88	[activation]
26865	VCAM1	APOE	[activation]
26866	SMYD2	AXIN1	[activation]
26867	C17orf82	TRAF2	[activation]
26868	C5AR1	RPS19	[activation]
26869	PARD3	ARPC2	[activation]
26870	KPNB1	SREBF2	[activation]
26871	PRIMPOL	SMARCAL1	[activation]
26872	PPP1CC	RB1	[inhibition]
26873	ITGA4	ATXN2L	[activation]
26874	RASL12	SMAD3	[activation]
26875	TES	TLN1	[activation]
26876	SGK1	NASP	[activation]
26877	KMT2A	ASH2L	[activation]
26878	C6orf165	ROCK1	[activation]
26879	H2AFZ	SMC2	[activation]
26880	CCNH	ERCC2	[activation]
26881	HSPA1L	GABARAPL2	[activation]
26882	NFATC2	PPP2R1A	[activation]
26883	TP53	PPA1	[activation]
26884	SHB	LAT	[activation]
26885	MAPK10	MAPK9	[activation]
26886	BARD1	RFC2	[activation]
26887	ANAPC4	TP53BP1	[activation]
26888	ITGB1	RPA2	[activation]
26889	SEMA4C	FBXO6	[activation]
26890	WWTR1	DDX17	[activation]
26891	RAB14	RAB11FIP1	[activation]
26892	MYC	ITGAL	[activation]
26893	CAPN1	VCAM1	[activation]
26894	NAP1L5	GRB2	[activation]
26895	KLHDC7B	NUDCD3	[activation]
26896	LPL	UBE2Z	[activation]
26897	GATA4	NR5A1	[activation]
26898	GRK6	HSP90AB1	[activation]
26899	PPP1R16A	LY6H	[activation]
26900	CAP2	ACTG1	[activation]
26901	MYOC	NOTCH2	[activation]
26902	UBE2F	EIF2B4	[activation]
26903	RAF1	RHEB	[activation]
26904	NFASC	GRB2	[activation]

26905 RAB5B CDK1 [activation]
26906 PPP4R1 HDAC3 [activation]
26907 BPIFB3 STAT1 [activation]
26908 FAM84B RAD51 [activation]
26909 NOS1 CAMK1 [activation]
26910 NR3C1 HOXB1 [activation]
26911 PLG F3 [activation]
26912 F2R CAV1 [activation]
26913 SP1 MYCN [activation]
26914 GPR25 VAPB [activation]
26915 SRPK1 WDR1 [activation]
26916 MDC1 ANAPC10 [activation]
26917 ANXA5 ITGB5 [activation]
26918 RAB3D AKT2 [activation]
26919 ZNF823 PAK1 [activation]
26920 RPA1 RPA3 [activation]
26921 SRPK1 HECW2 [activation]
26922 MME METTL7A [activation]
26923 NXF1 TIFA [activation]
26924 MYL3 ATG101 [inhibition]
26925 STX1A TXLNA [activation]
26926 USP9X MTOR [activation]
26927 HLA-B PREP [activation]
26928 KCNA3 KCNA1 [activation]
26929 DDX5 SMARCA4 [activation]
26930 VARS SGK1 [activation;inhibition]
26931 YWHAZ SH3BP5L [activation]
26932 SFN ARHGEF17 [activation]
26933 CEBPA RBL1 [activation;inhibition]
26934 APPL1 DPYSL5 [activation]
26935 RAB5A GRIK4 [activation]
26936 SNIP1 MAX [inhibition]
26937 ILK COPB2 [activation]
26938 GRWD1 TP53 [activation]
26939 ACTG1 MYO1A [activation]
26940 TNFRSF14 IMPA2 [activation]
26941 SMAD3 LCK [activation]
26942 HNRNPA0 SMURF1 [inhibition]
26943 PRKAA2 PARS2 [inhibition]
26944 NOTCH2NL CXCL5 [activation]
26945 ELK1 STK16 [activation]
26946 GSTK1 EIF4G2 [activation]
26947 MTOR GSK3B [activation;inhibition]
26948 ADRB2 RANBP2 [activation]
26949 LYAR FGF18 [activation]
26950 LYZ IKBKB [activation]
26951 PRMT5 SMARCB1 [activation]
26952 SMAD5 DDX5 [activation]
26953 LYN ABI1 [activation]
26954 MDC1 MDM2 [activation]
26955 STK4 AKT1 [activation]
26956 CALM1 CNN1 [activation]
26957 RPL7 RAN [activation]
26958 ACVR2B HSP90AA1 [activation]
26959 MAP4K4 MINK1 [activation]
26960 CSF1 SLA2 [activation]
26961 CD4 DHRS2 [activation]
26962 CASP9 MSTO1 [activation]
26963 NDUFA4 ILK [activation]
26964 UPF2 WNK1 [activation]
26965 WDR16 HSPA1A [inhibition]
26966 C9orf78 TAB1 [inhibition]
26967 MAPK9 MAP3K1 [activation]
26968 NOS3 PPP2R5A [activation]
26969 SYCP3 REC8 [activation]
26970 UBA5 LTA4H [activation]
26971 DLX5 MSX1 [activation]
26972 SMURF1 DUSP13 [inhibition]
26973 ELAVL1 RICTOR [activation]
26974 ITK CD2 [activation]
26975 YWHAZ STK38 [activation]
26976 RPS6KA1 GMFB [activation]
26977 TP53 IQGAP1 [activation]
26978 CRYAA HSPB1 [activation]
26979 CDH1 ADAM9 [activation]
26980 NFATC3 CDK6 [inhibition]

26981	ANXA2	ARRB2	[activation]
26982	RAPGEF2	FLNC	[activation]
26983	PPP2R2A	GRB2	[activation]
26984	SYNGAP1	CAMK2A	[activation]
26985	PRKCA	ADAP1	[activation]
26986	YWHAB	H2AFX	[activation]
26987	CKAP4	TP53	[activation]
26988	BRCA2	FANCG	[activation]
26989	SRPK1	KCNAB1	[activation]
26990	NR3C1	POU2F1	[activation]
26991	TRAF6	PPP1CC	[activation]
26992	PAXIP1	DDX52	[activation]
26993	NOD1	HSP90AA1	[activation]
26994	ERBB4	YAP1	[activation]
26995	CHEK1	BTG3	[activation;inhibition]
26996	ESR1	TRAF6	[activation]
26997	AMOT	AMOTL1	[activation]
26998	CASP7	COPS6	[activation]
26999	MAPK9	DUSP1	[activation]
27000	CRY1	CSNK1E	[activation]
27001	XIAP	CASP4	[activation]
27002	DNAJB1	AKT1	[inhibition]
27003	CDK3	EIF2AK2	[activation]
27004	RAB4A	PRKCI	[activation]
27005	RAN	PPP2R2B	[activation]
27006	HSPH1	PAFAH1B1	[activation]
27007	SRF	HDAC4	[activation]
27008	TRAF2	TNFRSF18	[activation]
27009	YWHAG	ATF2	[activation]
27010	DEFB103A	CCR6	[activation]
27011	VCAM1	ITGB7	[activation]
27012	RICTOR	RPL23	[activation]
27013	EIF4EBP1	ATM	[activation]
27014	FZR1	MYC	[inhibition]
27015	FOS	BATF	[activation]
27016	PPP1CA	ZFYVE16	[activation]
27017	TFF2	FCRL4	[activation]
27018	SYP	VAMP2	[activation]
27019	ITGB1	LYN	[activation]
27020	SUV39H2	NCK1	[activation]
27021	IRF7	TICAM1	[activation]
27022	HSPA1A	BRSK1	[inhibition]
27023	BRCA2	CDK2	[activation]
27024	LY96	TLR2	[activation]
27025	YWHAZ	TH	[activation]
27026	LAMA5	COL7A1	[activation;inhibition]
27027	HSP90AB1	DDX24	[activation]
27028	IER3IP1	TRAF6	[activation]
27029	EP300	MYB	[activation]
27030	TP53	RNF39	[activation]
27031	CRK	MAP4K5	[activation]
27032	CHUK	RPL27	[activation]
27033	FTSJ1	ZMPSTE24	[activation]
27034	JUND	FOS	[activation]
27035	TP73	MYC	[activation;inhibition]
27036	GRB2	ARHGAP17	[activation]
27037	UBE2U	BARD1	[activation]
27038	GRIP1	GRIA4	[activation]
27039	DDX4	SRC	[activation]
27040	CAMK2D	MOAP1	[activation]
27041	LPL	COPS6	[activation]
27042	RHOA	CNKSR1	[activation]
27043	GNL3L	TP53	[activation]
27044	GIPC1	RGS1	[activation;inhibition]
27045	OBSL1	GSN	[activation]
27046	ITGA4	ACTG1	[activation]
27047	MCM7	SUV420H2	[activation]
27048	DRD2	GNAI2	[activation]
27049	ARRB1	DVL2	[activation]
27050	MRFAP1L1	CAGE1	[activation]
27051	NUP62	G3BP2	[activation]
27052	YY1	BCL6	[activation]
27053	PIK3R3	FBN3	[activation]
27054	HSP90AB1	MAP3K8	[activation]
27055	HDAC3	MEF2A	[activation]
27056	MAP2K7	TRIB3	[activation]

27057	RPA2	EIF2S2	[activation]
27058	TAGLN2	ITGA4	[activation]
27059	MTOR	LRPAP1	[inhibition]
27060	CSNK2A1	MYC	[activation]
27061	STAT5A	INSR	[activation]
27062	SMAD1	NEDD9	[activation]
27063	CAB39	STK11	[activation]
27064	CSNK2B	VCAM1	[activation]
27065	SUPT16H	ICAM1	[activation]
27066	PIK3R1	IL7R	[activation]
27067	BLNK	MAP4K1	[activation]
27068	ACTB	BCL7C	[activation]
27069	SRPK1	CHERP	[activation]
27070	TIMM50	TP53	[activation]
27071	METTL21A	HSPH1	[activation]
27072	TP53	HDAC1	[activation]
27073	JAKMIP2	KIF9	[activation]
27074	CD5	LCK	[activation]
27075	CTNNA2	EGFR	[activation]
27076	SREBF2	INSIG2	[activation]
27077	KDR	EPN1	[activation]
27078	KDM6A	KMT2D	[activation]
27079	SDC4	TIAM1	[activation]
27080	KIT	RFC1	[activation]
27081	CSRP2	STK4	[activation]
27082	SUCLG2	MYC	[activation]
27083	HLA-C	B2M	[activation]
27084	ITGA2B	CD36	[activation]
27085	WDR36	TBXA2R	[inhibition]
27086	SLC9A3R1	EGFR	[activation]
27087	PHLDA3	RFXANK	[activation]
27088	VAMP2	TRPC3	[activation]
27089	ABCE1	ITGA4	[activation]
27090	RAC1	DEF6	[activation]
27091	LSP1	MAPKAPK2	[activation]
27092	SMARCA5	PTBP3	[activation]
27093	MYB	TRAF6	[activation]
27094	SLC9A3R1	SLC9A3	[activation]
27095	LAT	CD3E	[activation]
27096	MTUS2	PRKAA1	[inhibition]
27097	SMURF1	MAVS	[inhibition]
27098	MCAT	ICT1	[activation]
27099	ANKRD36BP1	USHBP1	[activation]
27100	MAPRE1	NUPR1	[activation]
27101	S1PR1	SP1	[activation]
27102	IGF1R	FFAR2	[activation]
27103	SAMD9	EEA1	[activation]
27104	PRKCD	NCF1	[activation]
27105	JUN	MAP2K4	[activation]
27106	HSP90AB1	ERBB3	[activation]
27107	RPS14	GRB2	[activation]
27108	BID	CSNK2A2	[activation]
27109	DDX39A	MDM2	[activation]
27110	MDM2	RASSF3	[activation]
27111	CTNNB1	YAP1	[activation]
27112	NBN	TCOF1	[activation]
27113	EHMT1	RELA	[activation]
27114	AURKB	AMOT	[activation]
27115	PLCD1	PAXIP1	[activation]
27116	CAST	GRB2	[activation]
27117	SRPK2	PPIL1	[activation]
27118	STK4	IQGAP1	[activation]
27119	GRB2	SERBP1	[activation]
27120	HLA-B	KPNA3	[activation]
27121	CDK1	RACGAP1	[activation]
27122	YTHDF1	RPA3	[activation]
27123	IFNG	TNF	[activation]
27124	ANAPC5	MAPK6	[activation]
27125	CDK18	CTSB	[activation]
27126	MAPK14	BMI1	[activation]
27127	APC	KIAA1328	[inhibition]
27128	TNFRSF14	HDAC4	[activation]
27129	CRTC1	CREBBP	[activation]
27130	A2M	MAPK6	[inhibition]
27131	BAD	GRB2	[activation;inhibition]
27132	ATF4	EP300	[activation]

27133 CUTC BCL6 [activation]
27134 MAPK14 DYRK1B [activation]
27135 RAD51 ABL1 [activation]
27136 BCL2L1 RTN4 [activation]
27137 EFNB1 TIAM1 [activation]
27138 LRP6 PDGFRB [activation]
27139 CCNG1 KRT40 [activation]
27140 DAB2IP SKP1 [inhibition]
27141 PRKAB1 EIF2S3 [activation]
27142 SERTAD1 HSPB1 [activation]
27143 FAF2 HIF1A [activation]
27144 CRK EXTL3 [activation]
27145 PIK3R1 TPSAB1 [activation]
27146 CACNG3 APP [activation]
27147 MAPK1 CREBBP [activation]
27148 YWHAZ TP53BP1 [activation]
27149 BTG1 CCNA2 [activation]
27150 NOS3 MAGEA11 [activation]
27151 CA4 PIH1D1 [activation]
27152 EP300 ILF2 [activation]
27153 TUB ABL1 [activation]
27154 SERTAD1 CCND2 [inhibition]
27155 DLG2 WNT3A [activation]
27156 LTBP2 FYN [activation]
27157 GNAS SMAD2 [activation]
27158 TP53 EHMT2 [activation]
27159 TGFB1I1 GIT2 [activation]
27160 MCM4 ITGA4 [activation]
27161 CASP8 PSEN1 [activation]
27162 KIAA0408 TRAF2 [activation]
27163 MLH1 C9orf156 [activation]
27164 TP53 WT1 [activation]
27165 SRPK1 RBMS2 [activation]
27166 RPAP2 GPN3 [activation]
27167 HSP90AB1 LATS1 [activation]
27168 EIF3A RPTOR [activation]
27169 ICAM1 EIF3I [activation]
27170 PEBP1 CDK2 [inhibition]
27171 ZBTB16 EIF2S2 [activation]
27172 NR1I2 RXRB [inhibition]
27173 IKBKB HSP90AB1 [activation]
27174 SRC GUCY2C [activation]
27175 SWSAP1 RAD51C [activation]
27176 UBTF CREBBP [activation]
27177 NXF1 FUNDC2 [activation]
27178 CREBBP TP73 [activation;inhibition]
27179 ATP6AP2 REN [activation]
27180 SETDB1 EHMT1 [activation]
27181 RELA DDX21 [activation]
27182 TNFRSF14 LHPP [activation]
27183 PTK2B PDCD6IP [activation]
27184 BBS10 TNFSF11 [activation]
27185 GSTM3 GH1 [inhibition]
27186 ILK LIMS2 [activation]
27187 DNAJB1 NRAS [inhibition]
27188 TNIP2 NFKB2 [activation]
27189 L1CAM MSN [activation]
27190 CD79A IGJ [activation]
27191 LST1 PRKCB [activation]
27192 LNP1 LYN [activation]
27193 LRRK2 ARHGEF7 [activation]
27194 CTNNB1 ERMN [activation]
27195 HSP90AB1 EIF2AK1 [activation]
27196 FOXO1 ESR1 [activation]
27197 MYC NCAPD3 [activation]
27198 LINC00526 NOTCH2NL [activation]
27199 CERS2 EGFR [activation]
27200 HSPA5 A2M [inhibition]
27201 SMARCC2 GRB2 [activation]
27202 MEMO1 VCAM1 [activation]
27203 TBC1D2 BUB1 [activation]
27204 FHL2 MCM7 [activation]
27205 CDH13 MAPK6 [activation]
27206 PRKCD APP [activation]
27207 AURKA CDH13 [activation]
27208 NOV IGF1 [activation]

27209	MAPK3	MAPK8	[activation]
27210	FKBPL	GNAZ	[activation]
27211	STAT5A	PRTFDC1	[activation]
27212	RABAC1	SNCA	[activation]
27213	STK4	HSPA9	[activation]
27214	RAB5C	HLA-B	[activation]
27215	CUZD1	PTPN6	[activation;inhibition]
27216	MARK2	MAPT	[activation]
27217	TOP1	WHSC1	[activation]
27218	GNAI1	S1PR2	[activation]
27219	DDX18	PAXIP1	[activation]
27220	MLH1	ANXA6	[activation]
27221	GRIA2	SDCBP	[activation]
27222	TNFAIP6	THBS1	[activation]
27223	GNB1	ATXN10	[activation]
27224	SRPK2	LUC7L2	[activation]
27225	MST4	PPP2R4	[activation]
27226	DOCK7	ANXA7	[activation]
27227	ACTR2	TNIK	[activation]
27228	CDK17	EPHA2	[activation]
27229	HCK	DIAPH1	[activation]
27230	IL2RG	CAPNS1	[activation]
27231	TBKBP1	OBSL1	[inhibition]
27232	RAN	BIRC5	[activation]
27233	MAPK3	FPR1	[activation]
27234	IRAK4	MBP	[inhibition]
27235	FGFR4	CDH2	[activation]
27236	VAV1	RAF1	[activation]
27237	EIF4EBP1	RICTOR	[activation]
27238	CCDC8	SMARCA5	[inhibition]
27239	RSRC2	SRPK2	[activation]
27240	RELA	IKBKG	[activation]
27241	RPS6KA2	CREB1	[activation]
27242	AR	RNASEL	[activation]
27243	WHSC1	DDX54	[activation]
27244	ZBED2	POT1	[activation]
27245	CDH1	MAP2K1	[activation]
27246	TP53BP1	RPA1	[activation]
27247	SRC	PTGER4	[activation]
27248	ANAPC11	ANAPC2	[activation]
27249	GABARAPL2	SMARCA4	[activation]
27250	MAPT	APOE	[activation]
27251	BCL2L1	UHRF2	[activation]
27252	TSPYL4	TP53	[activation]
27253	PRTN3	F2RL1	[activation]
27254	ICT1	AFG3L2	[activation]
27255	HSPA1A	APAF1	[activation]
27256	STK39	CD46	[inhibition]
27257	SAAL1	CA9	[activation]
27258	RPL41	MAPK14	[activation]
27259	BBC3	BAX	[inhibition]
27260	EP300	NFKB1	[activation]
27261	CASP7	ARHGDIA	[activation]
27262	NKX2-1	MAPK1	[activation]
27263	MYOM2	TAF1	[activation]
27264	DNAJB5	TTN	[inhibition]
27265	CCL5	KRAS	[activation]
27266	MCM5	MCM3	[activation]
27267	ACTN1	TES	[activation]
27268	CD44	ACTR3	[activation]
27269	CBLB	IRS1	[inhibition]
27270	GNB1	RICTOR	[activation]
27271	TGFBR1	PARD6A	[activation]
27272	CRY2	NXF1	[activation]
27273	PLSCR1	RASD1	[activation]
27274	TDGF1	GDF9	[activation]
27275	EIF2AK2	GABARAPL2	[activation]
27276	PIK3R1	AGAP2	[activation]
27277	ASAP1	PTK2B	[activation]
27278	PPP1R2	MYC	[activation]
27279	EEPD1	EPHB2	[activation]
27280	MAGEC2	UBE2H	[activation]
27281	CTBP2	FOXP2	[activation]
27282	PLCD1	CALM1	[activation]
27283	ESR1	BAZ1B	[activation]
27284	NXF1	STX2	[activation]

27285 REPS1 ATF4 [activation]
27286 ABL1 SPAG9 [inhibition]
27287 HSP90AA1 MYLK3 [activation]
27288 RAB5A TXNIP [activation]
27289 SMARCC1 CREBBP [activation]
27290 PIK3R1 PLCG1 [activation]
27291 TRAF6 RANBP9 [activation]
27292 KIT SOCS5 [inhibition]
27293 RFC4 EGFR [activation]
27294 RAN UBC [activation]
27295 CCL5 CDH1 [activation]
27296 SMARCA5 AIM2 [activation]
27297 RPS6KA1 HSP90AA1 [activation]
27298 SMAD4 HERC5 [inhibition]
27299 PTPN2 PDGFRB [activation]
27300 PRKCE CNN1 [activation]
27301 GNA14 SNW1 [activation]
27302 FOS USF2 [activation]
27303 PRKCZ STAT6 [activation]
27304 SAA4 COL4A1 [activation]
27305 PMS2 CYLD [inhibition]
27306 EPAS1 DBP [activation]
27307 PRMT1 MECOM [activation]
27308 PRNP ADAM23 [activation]
27309 PPP2R1A PPFIA3 [inhibition]
27310 LRRK2 CSNK1G2 [activation]
27311 PIK3R1 YWHAG [activation]
27312 ATM ATR [activation]
27313 GRB2 YWHAZ [activation]
27314 SPTAN1 GRB2 [activation]
27315 HSPA4 TRAF6 [activation]
27316 ITCH NFE2 [activation]
27317 VASH2 LRRK2 [activation]
27318 ZAP70 MET [activation;inhibition]
27319 CTNNB1 FANCG [activation]
27320 RAB2A RGS2 [activation]
27321 MBP APP [inhibition]
27322 SRC LIG3 [activation]
27323 SRPK2 DHX15 [activation]
27324 USP9X MLLT4 [activation]
27325 TNF MMP17 [activation]
27326 PBDC1 CTNNB1 [activation]
27327 ERH EIF4A3 [activation]
27328 GPRASP1 PER1 [inhibition]
27329 THRA NSD1 [activation]
27330 PIK3R2 YWHAB [activation]
27331 ARRB2 CDC42 [activation]
27332 MYB RQCD1 [activation]
27333 SUPT6H SMAD2 [activation]
27334 PPARG VDR [activation]
27335 ITGB1 CD36 [activation]
27336 SMAD1 PIAS1 [inhibition]
27337 HNRNPUL1 EP300 [activation]
27338 IL37 IL18R1 [activation]
27339 RAP2B MME [activation]
27340 ERC1 ITSN2 [activation]
27341 PTPRO PDGFRB [activation]
27342 TRIM69 NEK8 [activation]
27343 FLNA PAK1 [activation]
27344 HMGB2 VCAM1 [activation]
27345 SMURF2 RHOA [activation]
27346 RPL37 SRPK1 [activation]
27347 PRKCD KLF5 [activation]
27348 CHD3 TP53 [activation]
27349 SH2B2 ASB6 [activation]
27350 GCG MEP1B [activation]
27351 NUP153 MAPK3 [activation]
27352 NXF1 SLC31A1 [activation]
27353 PRSS23 PDPK1 [activation]
27354 TRAF3 MYD88 [activation]
27355 DVL1 SCYL2 [activation]
27356 RAD51D HELQ [activation]
27357 SMARCA4 NOTCH1 [activation]
27358 ACTB ESR2 [activation]
27359 APP SKAP1 [activation]
27360 MAPK1 NDE1 [activation]

27361	KNDC1	MYC	[activation]
27362	ZNHIT3	RXRA	[inhibition]
27363	MYD88	IRF4	[activation]
27364	HLA-B	RCC2	[activation]
27365	SORT1	LPL	[activation]
27366	JAK2	HIST2H3A	[activation]
27367	HSPA8	APP	[inhibition]
27368	PAICS	SGK1	[activation]
27369	P4HB	ATF2	[activation]
27370	NUP214	APC	[activation]
27371	MLH1	BARD1	[activation]
27372	ERCC6	HIST1H3A	[activation]
27373	CASP4	CASP10	[activation]
27374	PPP6R3	GPSM1	[inhibition]
27375	ATXN7L3B	EGFR	[activation]
27376	SOCS4	NR4A1	[inhibition]
27377	MAPK8	MAPKAP1	[activation]
27378	TNFRSF12A	RAC1	[activation]
27379	DYDC1	NME5	[activation]
27380	CTNNB1	RAPGEF2	[activation]
27381	REXO2	ATG5	[activation]
27382	EEF1D	CD48	[activation]
27383	NEURL2	CTNNB1	[inhibition]
27384	CDC16	SMAD2	[activation]
27385	ARF4	ATF2	[activation]
27386	TLR9	SIGIRR	[activation]
27387	ASAP2	PXN	[activation]
27388	TLR9	SRC	[activation]
27389	DCLRE1C	PRKDC	[activation]
27390	RAD51	UGDH	[activation]
27391	DSC1	DSG1	[activation]
27392	TSC22D3	FOS	[activation]
27393	TES	NXF1	[activation]
27394	RPL29	TIMP2	[activation]
27395	SERTAD4	AKT1	[activation]
27396	YWHAE	OBSL1	[activation]
27397	DAPK3	MDM2	[activation]
27398	APP	C17orf85	[activation]
27399	PTRH2	MDM2	[activation]
27400	SNCA	EIF3G	[activation]
27401	MIF	UPF2	[activation]
27402	PPP1R3A	RPS6KA1	[activation]
27403	EGFR	GEMIN6	[activation]
27404	FPR1	GNAI2	[activation]
27405	RAC1	VCAM1	[activation]
27406	PRKACA	RSBN1	[activation]
27407	HSPB1	CCNDBP1	[activation]
27408	PTPN11	FLT3	[activation]
27409	GCN1L1	IKBKE	[inhibition]
27410	DLGAP4	PIK3R1	[activation]
27411	SLC25A6	CHEK2	[activation]
27412	MMP3	TIMP3	[activation]
27413	GRB2	ACTG1	[activation]
27414	ICAM1	EIF3C	[activation]
27415	ITGB1	FLT4	[activation]
27416	EGFR	NTPCR	[activation]
27417	PIP5K1A	CASP3	[activation]
27418	CCDC8	PRKAA1	[inhibition]
27419	STRN4	CAV1	[activation]
27420	TAB2	TNFRSF11A	[activation]
27421	QARS	CDK4	[inhibition]
27422	GSK3B	NAT9	[activation;inhibition]
27423	EGFR	IDH3A	[activation]
27424	TNFSF13	KRAS	[activation]
27425	TRAF1	TNFRSF14	[activation]
27426	MTOR	NXF1	[activation]
27427	PRIMPOL	RPA3	[activation]
27428	EGFR	C6orf120	[activation]
27429	MDC1	ANAPC4	[activation]
27430	NRP2	FLT1	[activation]
27431	PACSIN1	SOS1	[activation]
27432	DHH	PTCH2	[activation]
27433	SRSF10	BARD1	[activation]
27434	NR3C1	EIF4A2	[activation]
27435	CD81	HIST1H1C	[activation]
27436	BARD1	TRAPPC8	[activation]

27437	NME1	PRKAA1	[activation]
27438	RRAS2	RALGDS	[activation]
27439	GABARAP	SRSF5	[activation]
27440	EIF4E2	TRAF6	[activation]
27441	SCHIP1	SCG2	[activation]
27442	CDC7	MCM10	[activation]
27443	YAP1	YWHAB	[activation]
27444	RPAP3	PARD6A	[activation]
27445	TRAF3IP3	TRAF3	[activation]
27446	CFL1	ESR1	[activation]
27447	NOTCH2NL	C19orf66	[activation]
27448	LATS1	LZTS2	[inhibition]
27449	E2F1	CHEK2	[activation]
27450	ARRB1	GNAQ	[activation]
27451	HLA-G	LILRB2	[activation]
27452	CREBBP	POU2F3	[activation]
27453	CBL	ITGAV	[activation]
27454	YWHAB	INSR	[activation]
27455	LATS1	MOAP1	[inhibition]
27456	PLD2	ACTN1	[activation]
27457	RAPGEF1	GRB2	[activation]
27458	FANCD2	IPO4	[activation]
27459	CCT8	GRB2	[activation]
27460	GADD45B	CDKN1A	[inhibition]
27461	PSIP1	MEN1	[activation]
27462	SMAD2	RHEBL1	[activation]
27463	NUP62	AHCTF1	[activation]
27464	LAIR1	PTPN6	[activation;inhibition]
27465	OSTF1	EXT1	[activation]
27466	EIF2S1	ERG	[activation]
27467	JAK2	STAP2	[activation]
27468	KPNB1	FGFR1	[activation]
27469	FTSJ1	PRMT1	[activation]
27470	SIRT3	SKP2	[activation]
27471	MEIS2	OSGIN1	[activation]
27472	ULK2	OBSL1	[inhibition]
27473	GRB2	PLD2	[activation]
27474	FYN	C7orf25	[activation]
27475	RAD51	IL24	[activation]
27476	TAF1B	HIST2H2AC	[activation]
27477	KLHL23	NUDCD3	[activation]
27478	HPRT1	FANCA	[activation]
27479	TIAM1	PRKCG	[activation]
27480	MAP3K10	MLK4	[activation;inhibition]
27481	MYC	SKP1	[inhibition]
27482	LRRK2	PCNA	[activation]
27483	H2AFX	BAZ1B	[activation]
27484	SPIN1	HSPB1	[activation]
27485	PIN1	ABI2	[activation]
27486	RAC1	ARHGAP17	[activation]
27487	RBPJ	NCOR1	[inhibition]
27488	MAP3K14	IL1R1	[activation;inhibition]
27489	A2M	RAB3A	[inhibition]
27490	NR3C2	PIAS1	[inhibition]
27491	E2F1	CDC27	[activation]
27492	ACACA	IFIT1	[activation;inhibition]
27493	BCL2L11	RIOK3	[activation]
27494	PRKCD	GSK3A	[activation;inhibition]
27495	CHPF	ARHGEF5	[activation]
27496	TRMT2A	CDK4	[activation]
27497	PAX6	PBX1	[activation]
27498	NAIP	CASP9	[inhibition]
27499	TRIM24	ATF4	[activation]
27500	EGR1	TBX2	[activation]
27501	RPL7	GRB2	[activation]
27502	HDAC1	SMC4	[activation]
27503	MAPT	TUBA1A	[activation]
27504	STK4	STRN	[activation]
27505	SPP1	NACA	[activation]
27506	GOSR1	EBI3	[activation]
27507	RHOQ	ELAVL1	[activation]
27508	PAX6	VSX2	[activation]
27509	VAMP8	CDC25A	[activation]
27510	EIF4E	RPS6KA5	[activation]
27511	RYR1	PRKG1	[activation]
27512	INHBB	INHBC	[activation]

27513	CPSF2	CPSF3	[activation]
27514	DDI1	KLHL42	[inhibition]
27515	BTRC	DLGAP5	[activation]
27516	CCNT1	KMT2A	[activation]
27517	RIMS2	FYN	[activation]
27518	EGFR	AP1M2	[activation]
27519	EIF4B	PLK1	[activation]
27520	GNAI1	RANGAP1	[activation]
27521	CDKN2B	CCDC33	[activation;inhibition]
27522	ESR1	HSPA4	[activation]
27523	EGFR	CTTN	[activation]
27524	PTK2B	TLN1	[activation]
27525	FZR1	E2F3	[inhibition]
27526	NSD1	VDR	[activation]
27527	STRADB	CAB39	[activation]
27528	IL2RG	JAK2	[activation]
27529	PIM2	BAD	[activation;inhibition]
27530	MAVS	ABL1	[activation]
27531	MAP2K5	PRKCZ	[activation]
27532	ELANE	THBS1	[activation]
27533	CBX3	LRRK2	[activation]
27534	HLA-A	SRC	[activation]
27535	PAK1	SRSF2	[activation]
27536	IL1RAP	TRAF6	[activation]
27537	DNM1L	LRRK2	[activation]
27538	MYC	OPHN1	[activation]
27539	PRKCB	HSP90AB1	[activation]
27540	IRS1	SH2B1	[activation]
27541	EZH2	EHMT1	[activation]
27542	DNAJC13	RPS6KA6	[activation]
27543	DLGAP4	SYNGAP1	[activation]
27544	PTK2	NCK1	[activation]
27545	YWHAZ	MINK1	[activation]
27546	TP53	MAD2L1BP	[activation]
27547	DOK1	PTK6	[activation]
27548	NBN	IPO5	[activation]
27549	TRIM54	CDKN1A	[inhibition]
27550	ERBB2	TEC	[activation]
27551	NFE2L2	MAFG	[activation]
27552	EIF5	IKBKE	[activation]
27553	BARD1	TSR1	[activation]
27554	MITF	FOS	[activation]
27555	GRK6	EDNRB	[activation]
27556	EHMT2	CACNA1A	[activation]
27557	ILK	EWSR1	[activation]
27558	FZR1	PTEN	[activation]
27559	NUDC	ARF6	[activation]
27560	HSF1	CDK6	[activation]
27561	NEU3	CAV1	[activation]
27562	DYRK1A	MAP3K5	[activation]
27563	HSPA4	EPAS1	[inhibition]
27564	INHBA	ENG	[inhibition]
27565	IGF2	CRELD2	[activation]
27566	CD14	DIP2A	[activation]
27567	EPOR	PTPN1	[activation]
27568	MAPK1	GABRR1	[activation]
27569	ROR1	MORC3	[activation]
27570	SUMO4	HSPA8	[inhibition]
27571	MT-CO3	RAC1	[activation]
27572	MYC	IDH3B	[activation]
27573	RASSF8	PPP1CA	[activation;inhibition]
27574	MYB	HIPK2	[activation]
27575	PCDHA8	CRK	[activation]
27576	USMG5	BLK	[activation]
27577	CEBPB	SMARCA4	[activation]
27578	SMAD2	GFER	[activation]
27579	SEMA7A	ABL1	[activation]
27580	DYRK1B	CREBBP	[activation]
27581	DHRS2	MDM2	[activation]
27582	PTBP1	STK4	[activation]
27583	PDPK1	GIT1	[activation]
27584	GRB2	ARHGAP20	[activation]
27585	SMC4	TRAF3IP1	[activation]
27586	APP	EPB41L5	[inhibition]
27587	GNAI2	C5AR1	[activation]
27588	MARK3	YWHAH	[activation]

27589	PCNA	RPA3	[activation]
27590	ACKR2	CCL13	[activation]
27591	BUB1	MIS12	[activation]
27592	NFKB2	DDX21	[activation]
27593	OBSCN	ESR2	[activation]
27594	NCOA1	DDX17	[activation]
27595	CD81	ITGB1	[activation]
27596	PRPF38A	POLA2	[activation]
27597	ATF4	NEK6	[activation]
27598	NCAPD2	RAD21	[activation]
27599	PRNP	PTRH1	[activation]
27600	EIF4G2	SKIL	[activation]
27601	MARCH1	CD86	[activation]
27602	GRIP1	CSPG4	[activation]
27603	U2AF1	SRPK3	[activation]
27604	MDM2	TOMM70A	[activation]
27605	BID	UBC	[activation]
27606	PHLDA3	NTRK1	[activation]
27607	IL6ST	LIF	[activation]
27608	E2F2	KMT2A	[activation]
27609	TGFBR1	NEK8	[activation]
27610	EXOC1	CCSER2	[activation]
27611	APOE	FARSA	[activation]
27612	CDKN1A	CCNB2	[activation;inhibition]
27613	CHRNA4	YWHAH	[activation]
27614	MAP3K4	PTPN11	[activation]
27615	PTPN13	RASSF10	[inhibition]
27616	ADRB2	ARRDC4	[activation]
27617	CTNNB1	WHSC1	[activation]
27618	APP	BTK	[activation]
27619	CD81	TSPAN4	[activation]
27620	TNFSF12	TRAF2	[activation]
27621	YWHAG	WWTR1	[activation]
27622	SMPD3	TNF	[activation]
27623	PACSIN3	SOS2	[activation]
27624	KDR	NCK1	[activation]
27625	DNAJB4	RAD21	[inhibition]
27626	SENP2	NUP153	[activation]
27627	PTP4A3	MAD2L1	[inhibition]
27628	MDC1	EP300	[activation]
27629	HOXB1	PBX1	[activation]
27630	NCOA3	KAT2B	[activation]
27631	FANCA	POLN	[activation]
27632	PCNA	CHEK1	[inhibition]
27633	TP53BP2	ANXA1	[activation]
27634	PTK6	CCT8	[activation]
27635	APP	TPPP	[activation]
27636	RGS12	PLCB3	[activation;inhibition]
27637	DYRK1A	DNM1	[activation]
27638	HLX	EZH2	[activation]
27639	MYC	PLOD2	[activation]
27640	MAPK3	PFKFB1	[activation]
27641	TSSC1	CDK2	[activation]
27642	RRAD	PRKCB	[activation]
27643	PARD6A	TGFB1	[activation]
27644	MAX	AIP	[inhibition]
27645	GNAI1	SSTR3	[activation]
27646	STK4	ABL1	[activation]
27647	AJUBA	TRAF6	[inhibition]
27648	TIMM50	ADRB2	[activation]
27649	OBSL1	CIT	[activation]
27650	SRF	CREBBP	[activation]
27651	CDC16	ESR2	[activation]
27652	SIRT1	HSPA1L	[activation]
27653	SMAD3	ATF2	[activation]
27654	GADD45A	ASCC2	[activation;inhibition]
27655	FAM193B	CDC25A	[activation]
27656	OBSL1	PHGDH	[activation;inhibition]
27657	TP53	BAK1	[activation]
27658	GRB2	H1F0	[activation]
27659	GRB2	CNTNAP1	[activation]
27660	SH3KBP1	BMPR1B	[activation]
27661	PIGR	CCL5	[activation]
27662	NPM2	SRPK2	[activation]
27663	OGT	TRAK1	[activation]
27664	FIS1	LRRK2	[activation]

27665 GNAS ADRB2 [activation]
27666 BCL2L1 CASP8AP2 [activation]
27667 AIRE EIF4EBP3 [activation]
27668 PPP1R12A GRB2 [activation]
27669 ICT1 LONP1 [activation]
27670 URM1 NXF1 [activation]
27671 TNIK PAFAH1B1 [activation]
27672 LCK PAK2 [activation]
27673 DIXDC1 APP [activation]
27674 UBC EFNB2 [activation]
27675 PIK3R1 NFKBIA [activation]
27676 CDKN2A SP1 [activation]
27677 DNM1 PFN2 [activation]
27678 PARP1 FOXO1 [activation]
27679 TGFBR1 AMHR2 [inhibition]
27680 XIRP1 EVL [activation]
27681 OBSL1 MLLT4 [activation]
27682 HIF1A USP19 [inhibition]
27683 LHCGR CHST9 [activation]
27684 EPRS VCAM1 [activation]
27685 VCAM1 PPA1 [activation]
27686 LRRK2 CAMK2G [activation]
27687 RPA3 ATRIP [activation]
27688 PFN1 MAP1LC3B [activation]
27689 YWHAB ITGB4 [activation]
27690 ADCY5 PRKACA [activation]
27691 TNFSF11 PLK1 [activation]
27692 NCAM1 PRKCB [activation]
27693 AXIN1 GAK [inhibition]
27694 GADD45G SMARCB1 [activation]
27695 SH3KBP1 PDGFRB [activation]
27696 MAPK10 CREBBP [activation]
27697 GRB2 FRS3 [activation]
27698 EXOC5 RALA [activation]
27699 DHRS7 ETV5 [activation]
27700 TRAF6 HMGB3 [activation]
27701 BRCA1 STAT1 [activation]
27702 EGFR IPO5 [activation]
27703 WEE1 CRK [activation]
27704 RAD18 NBN [activation]
27705 MAPK1 MAFA [activation]
27706 FTSJ1 APP [activation]
27707 ABL1 ABI2 [activation]
27708 MRGPRX1 GNAI2 [activation]
27709 RABEP1 CASP3 [activation]
27710 MTPN FTSJ1 [activation]
27711 ADAM10 TSPAN17 [activation]
27712 JAZF1 RXRA [inhibition]
27713 GRB2 ARAP1 [activation]
27714 UBE2I RAD51 [activation]
27715 LRRK2 FAM27E3 [activation]
27716 JAK3 IL4R [activation]
27717 RELA PYCR2 [activation]
27718 SIPA1L3 YWHAZ [activation]
27719 TGFB1I1 GP1BB [activation]
27720 PTCH1 ITCH [activation]
27721 SHC1 SEC16A [activation]
27722 PPP2R1B NUP62 [activation]
27723 NXF1 OBSL1 [activation]
27724 CEBPA IL32 [activation]
27725 NUDC IFT122 [activation]
27726 BMPR2 SMAD7 [activation]
27727 APP ERVFRD-1 [activation]
27728 HTR2A LYN [activation]
27729 TLR1 TLR5 [activation]
27730 GNAO1 ADRA2A [activation]
27731 BCL9L YAP1 [inhibition]
27732 IRS4 CDKN1A [activation]
27733 RPS17 GABARAPL2 [activation]
27734 CTNND1 ERBB2IP [activation]
27735 REM1 YWHAZ [activation]
27736 FBXO5 CDC23 [activation]
27737 MMP12 TFPI [inhibition]
27738 MAP4K1 PLCG1 [activation]
27739 EVL HCK [activation]
27740 HIF3A EGLN2 [activation]

27741	RELA	ATF3	[activation]
27742	PLEKHO2	YWHAE	[activation]
27743	UBE2E2	MARCH3	[activation]
27744	TCP11	VAV2	[activation]
27745	TBK1	HSPA8	[inhibition]
27746	IGHE	FCER1A	[activation]
27747	CDKN2AIP	WHSC1	[activation]
27748	HSP90AA1	FLT4	[activation]
27749	HSPBP1	TNFRSF14	[activation]
27750	ZAK	EZH2	[activation]
27751	CASP10	RIPK1	[activation]
27752	TCF7L2	E2F3	[inhibition]
27753	NR1H3	NR3C1	[activation]
27754	MLH1	CTSV	[activation]
27755	TRAF2	MORN3	[activation]
27756	CYLD	UBE2S	[inhibition]
27757	EGFR	FLOT2	[activation]
27758	UBTD1	RANBP2	[activation]
27759	HOXB13	IRF4	[activation]
27760	PTPRJ	KLK9	[activation]
27761	RNF125	UBE2E2	[activation]
27762	TIAM1	IGHA1	[activation]
27763	CCL14	DDX39A	[activation]
27764	BCAS3	PALB2	[activation]
27765	ROCK1	GFAP	[activation]
27766	TXNRD1	ESR1	[activation]
27767	MAP2K1	WNK1	[activation]
27768	GIT2	RUSC2	[activation]
27769	RINT1	RBL1	[inhibition]
27770	RPL7A	ICAM1	[activation]
27771	DDAH2	CDKN1A	[activation;inhibition]
27772	PTPN13	PRKACA	[activation]
27773	APP	PAK1	[activation]
27774	CCNH	RPA3	[activation]
27775	SMURF1	CSNK2A2	[inhibition]
27776	PPP1CC	GRB2	[activation]
27777	SH3GL2	ACTG1	[activation]
27778	HIF1A	SMAD3	[activation]
27779	HRAS	PRSS50	[activation]
27780	FANCG	USHBP1	[activation]
27781	BMPR2	CAV1	[activation]
27782	LRP1	WNT3A	[activation]
27783	KAT2B	ACTN1	[activation]
27784	ARHGEF7	RAC1	[activation]
27785	LRRK2	ERG	[activation]
27786	FLOT1	AURKB	[activation]
27787	LIMK1	LATS1	[activation;inhibition]
27788	STK4	KPNB1	[activation]
27789	TGFB2	VTN	[activation]
27790	EGFR	RNF126	[activation]
27791	FYN	CDCP1	[activation]
27792	PAXIP1	PCYT1B	[activation]
27793	WHSC1L1	ESR1	[activation]
27794	YWHAB	MINK1	[activation]
27795	PSEN2	CASP7	[activation]
27796	MLST8	PRR5L	[activation]
27797	STK33	LRRK2	[activation]
27798	DUSP26	TP53	[activation]
27799	CCND1	PPP1CC	[inhibition]
27800	CR2	FCER2	[activation]
27801	NCK1	BAZ2A	[activation]
27802	RBBP5	KMT2A	[activation]
27803	FYN	HRAS	[activation]
27804	NCKAP5L	FBXW11	[inhibition]
27805	CRTC2	CDKN1A	[activation]
27806	ABL1	DNASE1L2	[activation]
27807	JUNB	NEU1	[activation]
27808	MYO1C	PHLDA3	[activation]
27809	CCNE1	PIN1	[activation]
27810	TENC1	MYC	[activation]
27811	TFDP2	YWHAE	[inhibition]
27812	RBL2	CREG1	[inhibition]
27813	EZR	CD44	[activation]
27814	BLNK	CMTM3	[activation]
27815	LRP8	CLU	[activation]
27816	HIF1A	ARNT2	[activation]

27817	APBA3	EGFR	[activation]
27818	MCM2	ESR1	[activation]
27819	JAK3	FES	[activation]
27820	RPA1	CALD1	[activation]
27821	PIM1	MDM2	[activation]
27822	ATG5	LRRC59	[activation]
27823	ACTA1	TP53	[activation]
27824	MRE11A	HTT	[activation]
27825	FGD1	ELMO1	[activation]
27826	NKX2-1	SRC	[activation]
27827	MYCN	HDAC1	[activation]
27828	SLX4IP	STK4	[activation]
27829	ACKR4	CCL13	[activation]
27830	JAK2	GRB10	[activation]
27831	STK16	MBP	[activation]
27832	PAK1	BAD	[inhibition]
27833	CSK	PAG1	[activation]
27834	PCDHA4	SETDB1	[activation]
27835	HSP90AB1	ICK	[activation]
27836	MLH1	FSCN1	[activation]
27837	TRAP1	GABARAPL2	[activation]
27838	ADAM17	MAD2L1	[activation]
27839	ASAH1	SETDB1	[activation]
27840	C1orf216	LINC00521	[activation]
27841	SMAD2	KPNB1	[activation]
27842	KCNA4	KCNA1	[activation]
27843	APC	KRT19	[inhibition]
27844	SRSF6	PPARGC1A	[activation]
27845	MDM2	FOXO1	[activation]
27846	RB1	FRK	[activation;inhibition]
27847	EIF3F	EGFR	[activation]
27848	RARA	FOXO1	[activation]
27849	CCR5	ORM1	[activation]
27850	SRC	EGLN1	[activation]
27851	PRKCQ	IRS1	[activation]
27852	HRAS	RGL4	[activation]
27853	CDK2	POLD2	[activation]
27854	CLCF1	CRLF1	[activation]
27855	NPRL3	TP53	[activation]
27856	SKP2	DNAJB5	[inhibition]
27857	PRKDC	ILK	[activation]
27858	EFNB3	P2RX7	[activation]
27859	MAPK8	SPAG9	[inhibition]
27860	SASH3	APP	[activation]
27861	ARFIP2	RAC1	[activation]
27862	PTBP1	NOTCH1	[activation]
27863	MTA2	TWIST1	[activation]
27864	IKBKE	MYD88	[activation;inhibition]
27865	SATB1	EP300	[activation]
27866	PPARG	STAT6	[activation]
27867	FBXL5	SKP1	[inhibition]
27868	EGFR	IKBKG	[activation]
27869	AATF	EFNB3	[activation]
27870	EGFR	PPARGC1A	[activation]
27871	SGK3	PDK1	[activation;inhibition]
27872	RXFP1	RLN1	[activation]
27873	FTH1	HSPB1	[inhibition]
27874	PRPSAP1	PRKRA	[inhibition]
27875	URB1	TAB1	[inhibition]
27876	FXYD6	TP53	[activation]
27877	SH2D1B	APP	[activation]
27878	EP300	FOS	[activation]
27879	ACTB	TSC1	[activation]
27880	NPB	NPBWR1	[activation]
27881	CTCF	SUMO1	[activation]
27882	ETS1	ARL6IP4	[activation]
27883	WEE1	CASP3	[activation]
27884	EP300	TNIP2	[activation]
27885	ANXA1	EGFR	[activation]
27886	APP	IRS1	[activation]
27887	FES	KIT	[activation]
27888	TRMT2A	ANXA7	[activation]
27889	RASSF1	STK3	[inhibition]
27890	BTK	AFG3L2	[activation]
27891	CALM1	SPP1	[activation]
27892	ADAMTS13	VWF	[activation]

27893	NCK2	GAB1	[activation]
27894	RELA	MAPK10	[activation]
27895	TRAF2	CDC42	[activation]
27896	PLG	IGHA1	[activation]
27897	L3MBTL1	SETD7	[activation]
27898	SUMO4	STRAP	[inhibition]
27899	IL3RA	CSF2RB	[activation]
27900	CD81	DDX5	[activation]
27901	HSPB1	YWHAH	[activation]
27902	MAN1A2	FOS	[activation]
27903	MARK1	MAPT	[activation]
27904	ATG5	TECPR1	[activation]
27905	MAGEC2	TP53	[activation]
27906	CRKL	IRS4	[activation]
27907	RBL2	CCNA1	[activation;inhibition]
27908	CDK1	NCAPG	[activation]
27909	CSNK1E	FAM199X	[activation]
27910	NTRK1	RASA1	[activation]
27911	TSHR	GNAI3	[activation]
27912	PPBP	RAB10	[activation]
27913	ERBB2IP	NR2E1	[inhibition]
27914	TRAF6	EIF2S1	[activation]
27915	SDF4	MME	[activation]
27916	SMARCB1	ATM	[activation]
27917	PRKCZ	DAPK3	[activation]
27918	GNB5	GNG3	[activation]
27919	AKAP13	GNAQ	[activation]
27920	ABI1	GRB2	[activation]
27921	GABARAPL2	PRKCI	[activation]
27922	PLCG1	DLGAP3	[activation]
27923	RGS7	GNB5	[activation;inhibition]
27924	PCNA	FBXO6	[inhibition]
27925	AR	ACTB	[activation]
27926	FAM110A	GRB2	[activation]
27927	CBL	CD5	[activation]
27928	MYOM1	C1QTNF9	[activation]
27929	YWHAG	PPFIBP1	[activation]
27930	PIK3R1	CMIP	[activation]
27931	HSPA5	TAB1	[inhibition]
27932	ACP1	EPHA2	[activation]
27933	JUN	ERG	[activation]
27934	MYL6	ATF2	[activation]
27935	LRIF1	TDGF1	[activation]
27936	HCLS1	FGR	[activation]
27937	NUP88	RAE1	[activation]
27938	APP	KIR2DL3	[activation]
27939	E2F3	SLC25A6	[activation]
27940	EGFR	SPARCL1	[activation]
27941	PPARG	ZNF496	[activation]
27942	PTGS2	PTGIS	[activation]
27943	EIF4B	PAK2	[activation]
27944	ADD2	PRKCD	[activation]
27945	AURKA	BARD1	[activation]
27946	CDKN1B	RCHY1	[activation;inhibition]
27947	TPM2	PIK3CG	[activation]
27948	FTH1	TP53	[activation]
27949	CSF2RB	LCK	[activation]
27950	TFE3	YWHAG	[activation]
27951	MAPK9	DUSP4	[activation]
27952	RPA3	PPP2CA	[activation]
27953	GNB2L1	PAXIP1	[activation]
27954	PTPN6	TLR10	[activation;inhibition]
27955	CCND1	MCM3	[activation]
27956	MAP4K1	SPRY1	[inhibition]
27957	MDM2	RELA	[activation]
27958	CDK3	PPARA	[activation]
27959	ADRBK2	CCR4	[activation]
27960	PSEN2	FBXL12	[activation]
27961	IRAK2	IRAK3	[inhibition]
27962	NOTCH2NL	GLP1R	[activation]
27963	ENO1	ACACA	[activation;inhibition]
27964	ZFYVE19	CAPN7	[activation]
27965	IRAK1	SASH1	[activation;inhibition]
27966	ST5	GRB2	[activation]
27967	MAPK8	ARHGDIA	[activation]
27968	PAK2	GSTM3	[activation]

27969	CDC5L	EIF4A2	[activation]
27970	RAE1	PRIM1	[activation]
27971	GAB2	LCK	[activation]
27972	CCND1	CCNDBP1	[activation]
27973	TAL2	MAPK3	[activation]
27974	HSPB1	RBPJ	[inhibition]
27975	EIF4A3	ITGA4	[activation]
27976	AXIN1	SMAD2	[activation]
27977	PLD2	FTSJ1	[activation]
27978	GADD45A	SPTBN4	[activation]
27979	TRAP1	YWHAZ	[activation]
27980	ICT1	RBFA	[activation]
27981	LTA	UMOD	[activation]
27982	ACTB	CDK5R2	[activation]
27983	MYB	CSNK2A1	[activation]
27984	NCOA2	ESRRB	[activation]
27985	CAPRIN1	VCAM1	[activation]
27986	PXN	MAPK8	[activation]
27987	PNLIP	YWHAE	[activation]
27988	CD81	HIST3H3	[activation]
27989	IL2RG	IL9R	[inhibition]
27990	ATF1	CREM	[activation]
27991	FGF1	FGFR4	[activation]
27992	TNFSF13	TNFSF13B	[activation]
27993	TRPV4	PACSIN1	[activation]
27994	SKI	AKT2	[inhibition]
27995	POLD3	POLD2	[activation]
27996	FLNA	RHOA	[activation]
27997	SELL	PRKCQ	[activation]
27998	JAK1	ABL1	[activation]
27999	TRAF3	DYRK1A	[activation]
28000	FCAMR	TP53	[activation]
28001	ARRB1	IGF1R	[activation]
28002	CLCN3	HSPB1	[activation]
28003	ATF4	MAP2K7	[activation]
28004	DIABLO	NGFRAP1	[activation]
28005	MYO1B	MYC	[activation]
28006	BCL2L1	GIMAP5	[activation]
28007	TIE1	PIK3R1	[activation]
28008	CTNNA3	JUP	[activation]
28009	ANXA1	RPA1	[activation]
28010	TIMP3	ADAM17	[activation]
28011	EIF4A3	DDX39A	[activation]
28012	WNK1	MAP3K3	[activation]
28013	CDKN1A	DAPK3	[activation]
28014	NBN	CHEK2	[activation]
28015	PAK1	CDK11B	[activation]
28016	YWHAE	CBL	[activation]
28017	GRAP2	CD28	[activation]
28018	PIK3R1	RAC1	[activation]
28019	CBL	IGF1R	[activation]
28020	EIF4G2	EIF4A1	[activation]
28021	PTGDR	GNAS	[activation]
28022	CD46	CD151	[inhibition]
28023	AR	RUNX1	[activation]
28024	AKR7A2	VCAM1	[activation]
28025	SKIL	CDC16	[activation]
28026	ANXA1	FPR1	[activation]
28027	NTS	NTSR2	[activation]
28028	SH3BP2	PTPN6	[activation;inhibition]
28029	PTK6	EFHC2	[activation]
28030	CD81	HIST1H1A	[activation]
28031	SMAD1	PDGFRL	[activation;inhibition]
28032	TNFRSF14	CCDC59	[activation]
28033	PPARG	ALOX15B	[inhibition]
28034	NEXN	HSPB1	[inhibition]
28035	RHOA	MPRIP	[activation]
28036	RAP2B	VCAM1	[activation]
28037	PRRC2A	TP53	[activation]
28038	RAD21	IL7R	[activation]
28039	LUC7L	BARD1	[activation]
28040	PER2	MDM2	[activation]
28041	GDF7	BMP7	[activation]
28042	IRF2	EP300	[activation]
28043	CSNK1D	ZDHHC17	[activation]
28044	SEMG2	TGM1	[activation]

28045	MARS	MAP3K1	[activation]
28046	SLITRK4	LYN	[activation]
28047	HIST2H2BE	AURKB	[activation]
28048	PSTPIP2	SHC1	[activation]
28049	CYBA	NOX1	[activation]
28050	NOTCH1	RB1	[activation]
28051	FAM84B	CTNNA1	[activation]
28052	ERBB2	PHF23	[activation]
28053	MCM7	EGFR	[activation]
28054	NXF1	ZMYM4	[activation]
28055	MAP7D1	MYC	[activation]
28056	PLA2G4A	CASP3	[activation]
28057	HSF1	MAPK9	[activation]
28058	EP300	MAGEA11	[activation]
28059	RAB2A	BLZF1	[activation]
28060	STAT1	KPNA1	[activation]
28061	ESR2	NOTCH2NL	[activation]
28062	MAPT	RPS6KA3	[activation]
28063	SOS2	CRK	[activation]
28064	FBXW2	MYB	[activation]
28065	GNB1	MCF2	[activation]
28066	CDKN1B	CDK2	[activation;inhibition]
28067	HSP90AB1	MATK	[activation]
28068	KIF23	USP8	[activation]
28069	SH2D1A	TNK2	[activation]
28070	DDR1	FRS2	[activation]
28071	RUNX1	RUNX1T1	[activation]
28072	BHLHE40	STK19	[activation]
28073	MYB	KAT2A	[activation]
28074	NUCB1	GNAO1	[activation;inhibition]
28075	H2AFX	ESR1	[activation]
28076	TCEB1	SOCS1	[inhibition]
28077	C9	LAMTOR5	[activation]
28078	IL1R1	TICAM2	[activation]
28079	GDF5	CHRDL2	[activation]
28080	CDH5	WARS	[activation]
28081	ICT1	MRPS6	[inhibition]
28082	LGALS3	MICA	[activation]
28083	BTK	PRKCE	[activation]
28084	AKT1	MAP3K8	[activation]
28085	SRPK2	POLB	[activation]
28086	CDKN2C	CDKN2A	[activation;inhibition]
28087	CAMKK2	FLNC	[activation]
28088	F12	HIF1A	[activation]
28089	DDA1	PRKDC	[activation]
28090	STX7	UBC	[activation]
28091	ELAVL1	SETD3	[activation]
28092	CDK5RAP2	RBM48	[activation]
28093	STX1A	SLC6A9	[activation]
28094	PPP2R5A	JAK2	[activation]
28095	SCG2	TNF	[activation]
28096	TRIM24	HDAC1	[activation]
28097	PFN1	CRMP1	[activation]
28098	SMURF1	FZR1	[inhibition]
28099	SERPINB10	SRPK1	[inhibition]
28100	PRKCG	GRIA4	[activation]
28101	AMOTL2	MYO5B	[activation]
28102	ARHGEF11	PLCG1	[activation]
28103	PLXNA2	IFT80	[activation]
28104	SYK	CSF3R	[activation]
28105	FOXP3	HDAC1	[activation]
28106	TNIP2	MKNK1	[activation]
28107	APP	HBD	[inhibition]
28108	MYC	ASPM	[activation;inhibition]
28109	TP53	USP11	[activation]
28110	ADCY9	HSPB1	[activation]
28111	PYCARD	NLRC4	[inhibition]
28112	RPA3	ESYT1	[activation]
28113	KLK2	SERPINC1	[inhibition]
28114	PPP2CA	PAXIP1	[activation]
28115	DERL2	SHH	[inhibition]
28116	HNRNPA1	TP53	[activation]
28117	HSPB1	GAPVD1	[activation]
28118	CD44	MMP7	[activation]
28119	PYCARD	ARMC2	[inhibition]
28120	PTGIR	GNAQ	[activation]

28121	SNAP23	ABI2	[activation]
28122	POLN	HELQ	[activation]
28123	ARHGEF11	PLXNB1	[activation]
28124	FGF3	TUBG1	[activation]
28125	MDM2	SETDB1	[activation]
28126	APP	GPR12	[activation]
28127	SKIL	FZR1	[activation]
28128	PPP6R3	LATS1	[inhibition]
28129	PTBP3	YKT6	[activation]
28130	CD36	COL1A2	[activation]
28131	APOH	LDLR	[activation]
28132	STMN2	CDK1	[activation]
28133	SRPK3	SON	[activation]
28134	UBASH3B	FAM168A	[activation]
28135	TRAF6	HSPA9	[activation]
28136	PLK2	MPP3	[activation]
28137	AR	PLCG2	[activation]
28138	HEMGN	SMAD4	[activation]
28139	NFATC1	RPS6KB1	[activation]
28140	SIRT1	RARA	[activation]
28141	PEAK1	GRB2	[activation]
28142	TGFBR2	DAB2	[activation]
28143	ITGA8	ITGB1	[activation]
28144	HIST2H3A	TAF1B	[activation]
28145	ATP13A2	CXCR4	[activation]
28146	STK4	AURKB	[activation]
28147	OBSL1	HIST2H2BE	[activation;inhibition]
28148	ITGA5	FLT4	[activation]
28149	HSPB1	STK4	[activation]
28150	PRICKLE3	CRK	[activation]
28151	IRAK2	TLR4	[activation]
28152	GRN	FANCL	[activation]
28153	OLFML3	HSPB3	[activation]
28154	IGF2BP2	EIF2AK2	[activation]
28155	IRS1	SIRT1	[activation]
28156	ARSF	EP300	[activation]
28157	FLOT1	SORBS1	[activation]
28158	PPIA	JAK2	[inhibition]
28159	EPHA2	KPNA3	[activation]
28160	SRC	CTTN	[activation]
28161	DOCK9	NEDD9	[activation]
28162	SRF	MINPP1	[activation]
28163	LRRK2	COPE	[activation]
28164	PDXP	STK11	[activation]
28165	SFN	HDAC4	[activation]
28166	GAB1	PARD3	[activation]
28167	TP53	COPS4	[activation]
28168	BAD	CDKN1A	[activation;inhibition]
28169	HSPG2	PDGFA	[activation]
28170	SLC9A2	RASA1	[activation]
28171	CHRNA5	SRPK2	[activation]
28172	PXN	ITGB3	[activation]
28173	CD40	IL4R	[activation]
28174	RIN3	CRK	[activation]
28175	HNRNPA1	VCAM1	[activation]
28176	CHUK	ACTG1	[activation]
28177	HSPA7	GABARAP	[activation]
28178	SMAD1	GDF6	[activation]
28179	CITED2	CREBBP	[activation]
28180	TNFRSF21	NSD1	[activation]
28181	BACE1	PLSCR1	[activation]
28182	SRC	SH3BP1	[activation]
28183	PLXNB1	MST1R	[activation]
28184	TOMM34	VCAM1	[activation]
28185	RBL2	CCND1	[inhibition]
28186	PFN1	RAD51	[activation]
28187	MPP3	TRAF2	[activation]
28188	EPB41L5	ESR1	[activation]
28189	NGFR	PTPN13	[activation]
28190	ADRA1A	BMP1	[activation]
28191	PAK6	MAPK14	[activation]
28192	BLNK	MAP2K4	[activation]
28193	LAMB1	EIF3I	[activation]
28194	KIAA1522	SFRP1	[activation;inhibition]
28195	MATK	NTRK1	[activation]
28196	TNK2	NTRK2	[activation]

28197	CDX1	LGR4	[activation]
28198	RAD21	SMC2	[activation]
28199	ERBB2	STK24	[activation]
28200	DPYSL2	MICALL1	[activation]
28201	CDH5	SMG1	[activation]
28202	YWHAZ	TSC1	[activation;inhibition]
28203	BST2	MYD88	[activation]
28204	EGFR	YTHDF1	[activation]
28205	CNKSR1	RHOD	[activation]
28206	ZAP70	FCGR3A	[activation]
28207	PRKCB	CSF2RB	[activation]
28208	TSSK3	SMAD4	[activation]
28209	SH2D1A	LCK	[activation]
28210	EFS	ABI2	[activation]
28211	NACAD	MAP4K2	[activation]
28212	PAWR	PRKCZ	[activation]
28213	C16orf80	YWHAG	[activation]
28214	HCK	SRC	[activation]
28215	EFNB1	CAP1	[activation]
28216	CMTM5	ARMC9	[activation]
28217	CSNK2B	VRK3	[activation]
28218	SRC	JUP	[activation]
28219	HSP90AB1	TP53	[activation]
28220	ARF3	ITGA4	[activation]
28221	CREBBP	CDC16	[activation]
28222	CDK20	CCNH	[activation]
28223	KIT	SH2B2	[activation]
28224	SOCS3	KIT	[inhibition]
28225	LEO1	KMT2A	[activation]
28226	ERCC4	ERCC6	[activation]
28227	IKBKB	IKBKG	[activation]
28228	EPHA4	FGFR2	[activation]
28229	PIK3CB	GRB2	[activation]
28230	BRPF3	ABL1	[activation]
28231	RAD51D	IKZF1	[activation]
28232	PSEN1	CASP6	[activation]
28233	ITGA4	NTPCR	[activation]
28234	EGFR	ALDH3A2	[activation]
28235	BCL2L1	CLU	[activation]
28236	SAFB2	SRPK1	[activation]
28237	RPS6KA3	CREBBP	[activation]
28238	PPM1A	CACNA1A	[activation]
28239	LYZ	A2M	[inhibition]
28240	HSP90AB1	FLT4	[activation]
28241	DLGAP1	PLCG1	[activation]
28242	EPHA3	CDKN2A	[activation]
28243	MAGEB4	RUNDC3A	[activation]
28244	RHOD	HRAS	[activation]
28245	ADRB2	GNA15	[activation]
28246	TNFAIP3	TNF	[activation]
28247	SLX4	IGHA1	[activation]
28248	SMYD2	IGF2BP1	[activation]
28249	CREB1	DYRK1A	[activation]
28250	LZTFL1	FZD5	[activation]
28251	ARHGEF1	PIK3CA	[activation]
28252	SPP1	AMOTL2	[activation]
28253	TRAF2	WDR65	[activation]
28254	MRPL12	RELA	[activation]
28255	PLCG1	PECAM1	[activation]
28256	MCF2L2	MYC	[activation]
28257	FAM124A	PRKG1	[inhibition]
28258	HIF1A	USP8	[activation]
28259	SNCA	APP	[activation]
28260	TAF1B	TAF1D	[activation]
28261	HSPB1	SNW1	[activation]
28262	RAC3	WAS	[activation]
28263	FTSJ1	NUTF2	[activation]
28264	RPS14	ICAM1	[activation]
28265	GLOD4	BCL2L1	[activation]
28266	PBX1	SMARCA4	[activation]
28267	ARHGDIA	RAC1	[activation]
28268	RGS6	GNB3	[activation;inhibition]
28269	SEMA3B	PAK6	[activation]
28270	HSPB1	PPM1A	[activation]
28271	LAT	MAP4K1	[activation]
28272	OBSL1	HIST2H2AA3	[activation;inhibition]

28273	GJA1	CSK	[inhibition]
28274	CASP7	PTGES3	[activation]
28275	HPRT1	TRAF6	[activation]
28276	PLEKHA6	PLEKHA5	[activation]
28277	SRF	FLI1	[activation]
28278	RNF138	LEF1	[inhibition]
28279	CPNE3	USP42	[activation]
28280	LYVE1	VEGFA	[activation]
28281	PDGFRB	SLC9A3R1	[activation]
28282	RBL2	HDAC1	[inhibition]
28283	CTCF	TOP2A	[activation]
28284	IQGAP1	PRKACA	[activation]
28285	MYC	DUSP15	[activation]
28286	SP1	TBP	[activation]
28287	PDPK1	PAK1	[activation]
28288	ATM	ATRIP	[activation]
28289	AKT1	CAMKK1	[activation]
28290	CALM1	HMMR	[activation]
28291	PRKACA	RAP1GAP	[activation]
28292	MITF	YWHAZ	[activation]
28293	GRB2	SIGLEC7	[activation]
28294	GNL3	MYC	[activation]
28295	BCL6	HDAC1	[activation]
28296	HLA-B	SNRPB2	[activation]
28297	TFDP2	RBL1	[inhibition]
28298	IGHM	GSK3B	[activation]
28299	MMP3	HAPLN1	[activation]
28300	APP	EIF2B5	[activation]
28301	APPL1	RAB5A	[activation]
28302	KCTD3	SRC	[activation]
28303	HSPA5	F8	[activation]
28304	IGF1R	EGFR	[activation]
28305	TSC1	PLK1	[activation;inhibition]
28306	CKAP5	FYN	[activation;inhibition]
28307	TGFBR1	EP300	[activation]
28308	EP300	SMAD5	[activation]
28309	CASP4	VDAC3	[inhibition]
28310	SHC1	TUBA8	[activation]
28311	USF1	FOSL1	[activation]
28312	TFDP1	LIN9	[inhibition]
28313	RAF1	SRC	[activation]
28314	HTT	RASA1	[activation]
28315	YAP1	SUMO1	[activation]
28316	IRS4	USP9X	[activation]
28317	GNAI2	LHB	[activation]
28318	RACGAP1	FZR1	[activation]
28319	SH2B1	GRB2	[activation]
28320	ACTA2	EGFR	[activation]
28321	BCL2L1	ACTB	[activation]
28322	BUB1	CDC5L	[activation]
28323	SMAD1	APC	[inhibition]
28324	DHPS	ARF6	[activation]
28325	GNB2L1	NFATC1	[activation]
28326	DPYSL2	DPYSL3	[activation]
28327	EGFR	TELO2	[activation]
28328	CDK1	CSNK2B	[activation]
28329	GLRX2	BAG3	[activation]
28330	RPA3	CSNK1A1L	[activation]
28331	SERPING1	F2	[inhibition]
28332	HSPA8	IKBKB	[activation]
28333	HMMR	CDH13	[activation]
28334	COL1A1	BARD1	[activation]
28335	MAP4K1	HSP90AA1	[activation]
28336	APP	GOSR1	[activation]
28337	SCUBE1	SCUBE2	[activation]
28338	PPP2R1A	NUP62	[activation]
28339	ADORA2B	NTN1	[activation]
28340	PREX2	MYC	[activation]
28341	CDK1	HMGB1	[activation]
28342	ARHGEF2	FYB	[activation]
28343	NXF1	MRPL15	[activation]
28344	TLX1	ETS1	[activation]
28345	FAM90A1	SRPK2	[activation]
28346	FOXP3	PRR20E	[activation]
28347	PTPRB	GHR	[activation]
28348	ANAPC2	MAGEB2	[activation]

28349	GATA1	HSPA4	[activation]
28350	GRB2	GHR	[activation]
28351	SEMA6A	ITSN2	[activation]
28352	PPP2CA	CDK6	[inhibition]
28353	UBE2E2	RNF185	[activation]
28354	HSPA5	SMURF1	[inhibition]
28355	SKIL	TSKU	[activation]
28356	SERPINA1	MMP26	[inhibition]
28357	ARF5	PIP5K1A	[activation]
28358	SMURF1	DDX21	[inhibition]
28359	POMGNT2	APP	[activation]
28360	STAT5A	STAP2	[activation]
28361	C1orf52	ETS1	[activation]
28362	TRPV4	CALM1	[activation]
28363	CALM1	RGS2	[activation]
28364	NFASC	ABL1	[activation]
28365	RAC1	CDC23	[activation]
28366	VCAM1	RCC1	[activation]
28367	NRAP	ACTB	[activation]
28368	ABI1	RASA1	[activation]
28369	CTNNB1	SIRT1	[activation]
28370	MLLT4	RAP1GAP	[activation]
28371	DYSF	OS9	[activation]
28372	CEBPB	EGR1	[activation]
28373	IRS1	TUB	[activation]
28374	CALM1	CHUK	[activation]
28375	GATA1	STAT3	[activation]
28376	EDA2R	FADD	[activation]
28377	ELAVL1	NOS3	[activation]
28378	TH	YWHAG	[activation]
28379	CDC5L	EHD1	[activation]
28380	CFL1	FN1	[activation;inhibition]
28381	TBXA2R	GNAS	[inhibition]
28382	PPM1B	VAV2	[activation]
28383	NFYC	SMAD3	[activation]
28384	CYP51A1	ETS1	[activation]
28385	ITGB3	YES1	[activation]
28386	CDC20	CDC23	[inhibition]
28387	MOAP1	BAX	[activation]
28388	BRCA1	CHEK1	[activation]
28389	GRM5	CALM1	[activation]
28390	EPB41	EIF1	[activation]
28391	HIST1H1C	F10	[activation]
28392	MAPT	GSK3A	[activation]
28393	ERCC6	KAT2B	[activation]
28394	FCHSD2	DAPP1	[activation]
28395	JUP	PSEN1	[activation]
28396	EGFR	SAAL1	[activation]
28397	ATF2	THRB	[activation]
28398	SRSF4	PPARGC1A	[activation]
28399	CDC23	INCA1	[activation]
28400	CASP8	LYPD3	[inhibition]
28401	HSP90AB1	CAMK2B	[activation]
28402	DAB2IP	FBXW7	[activation]
28403	DDX58	TBK1	[activation]
28404	STAT3	STAT4	[activation]
28405	DAB1	ITGB7	[activation]
28406	KCNA4	FYN	[activation]
28407	ESR1	SLC25A6	[activation]
28408	AMOT	CTNNB1	[activation]
28409	MEN1	RELA	[activation]
28410	SNAPC3	GRB2	[activation]
28411	PKM	RAP1B	[activation]
28412	PLK1	TRIOBP	[activation]
28413	FAS	FAIM2	[inhibition]
28414	OTUD6A	CBLC	[inhibition]
28415	SIRT1	PHLDB3	[activation]
28416	EIF4A1	H2AFX	[activation]
28417	ULK1	PRKAA1	[inhibition]
28418	NCF1	PRKCZ	[activation]
28419	YWHAE	SRGAP2	[activation]
28420	CAV1	STRN	[activation]
28421	OPRL1	GNA14	[activation]
28422	UBTF	CCND1	[activation]
28423	BIRC2	UBE2V1	[activation;inhibition]
28424	GEMIN4	ILK	[activation]

28425	UBE2E2	RNF4	[activation]
28426	NXF1	CD2BP2	[activation]
28427	CLASRP	CDK6	[inhibition]
28428	OXSR1	TGFBR2	[activation]
28429	FOS	VDR	[activation]
28430	PPP2R5A	PPP2R5D	[activation]
28431	SPP1	EIF3K	[activation]
28432	LRP1	EGFR	[activation]
28433	GNAI1	PTH1R	[activation]
28434	MIDN	GADD45A	[activation]
28435	ELANE	F2RL1	[activation]
28436	RAB5A	RABGEF1	[activation]
28437	HSPA6	NOS2	[activation]
28438	MAP3K5	CREBBP	[activation]
28439	FARSB	FARSA	[activation]
28440	APP	NUMB	[activation]
28441	PTPN12	SFRP4	[activation]
28442	GRB2	FAU	[activation]
28443	NCL	LRRK2	[activation]
28444	MAP2K3	UBC	[activation]
28445	HSP90AB1	YWHAZ	[activation]
28446	RELN	DAB1	[inhibition]
28447	SMAD2	ANAPC2	[activation]
28448	RASGRP3	APP	[activation]
28449	STAT6	CEBPB	[activation]
28450	RPRD1A	RIOK2	[activation]
28451	HIST1H4A	CCDC8	[activation;inhibition]
28452	SUCLG2	MDM2	[activation]
28453	RPL26	FTSJ1	[activation]
28454	MCM7	CTNND1	[activation]
28455	ESRRA	NCOA1	[activation]
28456	BAAT	PDGFRL	[activation;inhibition]
28457	KLK3	A2M	[inhibition]
28458	TNFRSF13B	TNFSF13	[activation]
28459	F12	UBE2D3	[activation]
28460	ARHGDIB	APP	[activation]
28461	RPA2	PRKDC	[activation]
28462	PCBP1	SRPK3	[activation]
28463	BCL2L1	ANTXR1	[activation]
28464	POLR2A	NXF1	[activation]
28465	BAZ1B	VDR	[activation]
28466	ECT2	CDH1	[activation]
28467	TRIO	RAC1	[activation]
28468	ZYX	SREBF2	[activation]
28469	KIAA0226	EGFR	[activation]
28470	HSPA1L	PIK3R2	[activation]
28471	LEPR	LEPROT	[activation]
28472	GPR50	MTNR1A	[activation]
28473	BMP7	ENG	[activation]
28474	VCAM1	TRAPPC3	[activation]
28475	HDAC3	TP53	[activation]
28476	CDKN2C	ATR	[activation]
28477	MDM2	CKAP4	[activation]
28478	KDR	GRB10	[activation]
28479	GRB2	DLGAP3	[activation]
28480	MECOM	CREBBP	[activation]
28481	ERBB2	PTK2B	[activation]
28482	KLHL38	RGS20	[activation;inhibition]
28483	IKBKB	TRAF1	[activation]
28484	STK39	MAPK14	[activation]
28485	FKBP5	HSF1	[activation]
28486	ARL15	B4GALT3	[activation]
28487	PRKAA1	HSP90AB1	[activation]
28488	YWHAZ	PRMT5	[activation]
28489	ABI1	CACNA1A	[activation]
28490	SMAD3	RIT1	[activation]
28491	ROS1	PTPN11	[activation]
28492	LRRK2	DUX3	[activation]
28493	PRKCZ	CASP6	[activation]
28494	GNAO1	GABBR1	[activation;inhibition]
28495	CTBP2	EGFR	[activation]
28496	ABLIM3	GRB2	[activation]
28497	DIAPH1	CDC7	[activation]
28498	KATNA1	KATNB1	[activation]
28499	CSPG4	CDC42	[activation]
28500	SORBS1	PTK2	[activation]

28501	MAPK6	GLRX3	[activation;inhibition]
28502	RNF32	NOS3	[activation]
28503	IGF1	IGFBP2	[activation]
28504	CYFIP2	SMAD4	[activation;inhibition]
28505	CRK	GLTSCR1	[activation]
28506	ATM	CHEK2	[activation]
28507	JAK3	MAGED2	[activation]
28508	STX4	RAB5A	[activation]
28509	OGT	SP1	[activation]
28510	ANXA7	S100A10	[activation]
28511	IL17A	TRAF6	[activation]
28512	OSGIN1	PTEN	[activation]
28513	WNT3A	WNT1	[activation;inhibition]
28514	WWTR1	WNK1	[activation]
28515	PTGES3	HIF1A	[activation]
28516	TRIP6	VCL	[activation]
28517	HSP90AB1	MAP3K6	[activation]
28518	AR	PIK3R3	[activation]
28519	MAPK1	FRS3	[activation]
28520	CREBBP	CDKN1A	[activation]
28521	ETS1	SERBP1	[activation]
28522	VIP	MEP1A	[activation]
28523	DYSF	NPHP3	[activation]
28524	GIT1	GRB2	[activation]
28525	SPP1	FTH1	[activation]
28526	PRDX3	IKBKB	[activation]
28527	SRPK1	MAP1LC3A	[activation]
28528	RHOA	SMAD2	[activation]
28529	CC2D1B	SPP1	[activation]
28530	HDAC3	SYK	[activation]
28531	PLK1	CSN2	[activation]
28532	RIPK1	CARD6	[activation]
28533	CDK5RAP2	PTN	[activation]
28534	SST	SSTR3	[activation]
28535	RUNX1	NCOR2	[activation]
28536	CDC25C	SFN	[activation]
28537	AKT1	CCNA2	[activation]
28538	CSF3R	HCK	[activation]
28539	TDRD3	HIST3H3	[activation]
28540	NCAPD2	NCAPH	[activation]
28541	INADL	YAP1	[activation]
28542	HSP90B1	EGFR	[activation]
28543	IGF1	IGFBP6	[activation]
28544	BMX	C12orf45	[activation]
28545	MKNK1	PRKDC	[activation]
28546	RAD9A	CLSPN	[activation]
28547	THBS1	COL4A1	[activation]
28548	MMP2	EPHB2	[activation]
28549	APP	CDC7	[activation]
28550	DDX21	IL7R	[activation]
28551	YWHAB	RACGAP1	[activation]
28552	CCND1	PRKACA	[activation]
28553	AKT2	STEAP4	[activation]
28554	PAFAH1B1	PAFAH1B2	[activation]
28555	RAD21	CALML5	[activation]
28556	STK3	RASSF2	[activation]
28557	HSPB1	PPP2R3C	[activation]
28558	PDAP1	PDGFRB	[activation]
28559	RAD54B	C9orf156	[activation]
28560	FZR1	CDC16	[activation]
28561	PRKCI	PARD6B	[activation]
28562	GAD2	F10	[activation]
28563	NCOA1	HIF1A	[activation]
28564	RANBP9	MET	[activation;inhibition]
28565	MYO1C	GRB2	[activation]
28566	LATS1	KIRREL	[inhibition]
28567	SYK	JAK1	[activation]
28568	FOXO1	MST1	[activation]
28569	CAMK2G	HSP90AB1	[activation]
28570	PLCB3	TRPM7	[activation]
28571	ACTB	CBL	[activation]
28572	CHTOP	SRPK3	[activation]
28573	PPM1A	ASB2	[activation]
28574	IRAK3	IRAK1	[activation;inhibition]
28575	CACNA1S	RIMS2	[activation]
28576	KMT2B	BCL6	[activation]

28577	KCNIP3	IGLC2	[activation]
28578	STK4	ERC1	[activation]
28579	C1QTNF2	HPCAL1	[activation]
28580	PRKCZ	CASP3	[activation]
28581	NAV3	APC	[inhibition]
28582	ADRA1B	PRKCA	[activation]
28583	PRR14	PPP2CA	[inhibition]
28584	WHSC1	DDX18	[activation]
28585	MOB1A	SGK1	[activation]
28586	ACTG1	PTPRO	[activation]
28587	LYN	DOCK8	[activation]
28588	PTPN11	CAV1	[activation]
28589	IL27RA	EBI3	[activation]
28590	DYRK1A	PHYHIP	[activation]
28591	RAD54L2	AR	[activation]
28592	MATK	MET	[activation]
28593	DLG4	NLGN4X	[activation]
28594	MAPK13	ENO1	[activation]
28595	NUTM1	PRKAA2	[inhibition]
28596	FLNA	MYB	[activation]
28597	CUEDC2	MAD2L1	[inhibition]
28598	HSPA1L	CEP250	[inhibition]
28599	MAP2K5	BAD	[activation]
28600	RAC1	VAV1	[activation]
28601	MDM2	RARS	[activation]
28602	EFNA3	NOTCH2NL	[activation]
28603	CCDC8	FYN	[activation]
28604	WNK1	HIVEP2	[activation]
28605	RPF2	NXF1	[activation]
28606	PIAS2	ESR2	[activation]
28607	RALY	PIK3CD	[activation]
28608	TNK2	KCNA5	[activation]
28609	PKP4	PSEN1	[activation]
28610	FAM208B	ERH	[activation;inhibition]
28611	ANAPC5	FBXO5	[inhibition]
28612	SIPA1L2	ESR2	[activation]
28613	PF4	CCL5	[activation]
28614	STK4	KIF2A	[activation]
28615	SERPINE1	ORM1	[inhibition]
28616	PTPN1	CDH2	[activation]
28617	FYB	FASLG	[activation]
28618	CCDC8	IQGAP1	[inhibition]
28619	MDM4	RB1	[activation;inhibition]
28620	PER1	TIMELESS	[activation]
28621	TGFBR2	ZFYVE9	[activation]
28622	GRB2	ELK3	[activation]
28623	PPP2R5D	CHEK2	[activation]
28624	ARHGAP15	TGFBR1	[activation]
28625	DCLK1	APP	[activation]
28626	SPARCL1	TNF	[activation]
28627	KIAA1377	RAN	[activation]
28628	HDAC1	NFKB1	[activation]
28629	FBXO5	ANAPC2	[activation]
28630	CTCF	HIST2H2AC	[activation]
28631	GPBP1L1	LRRK2	[activation]
28632	EPHB1	GRB2	[activation]
28633	CHEK1	SLX4	[activation;inhibition]
28634	KIAA1598	MEGF10	[activation]
28635	APPL1	CTNNB1	[activation]
28636	TOP2B	NOTCH1	[activation]
28637	SMAD3	CHD8	[inhibition]
28638	SELPLG	VCAN	[activation]
28639	ROR2	FZD5	[activation]
28640	RANBP2	CDCA8	[activation]
28641	GLRX	ATP7A	[activation]
28642	RAC1	USP6	[activation]
28643	KLRC4	MICA	[activation]
28644	LUC7L2	GRB2	[activation]
28645	HSPA2	HSPBP1	[inhibition]
28646	FYN	WBP11	[activation]
28647	PSMD10	RHOA	[activation]
28648	SRPK2	STON1	[activation]
28649	TTC7A	MCM7	[activation]
28650	KNG1	ITGB2	[activation]
28651	SKAP1	FYN	[activation]
28652	TWF2	CAPZB	[activation]

28653	CMYA5	IL6ST	[inhibition]
28654	HERC5	CCND1	[inhibition]
28655	PPP2R1A	GABARAPL2	[activation]
28656	ANGPTL4	PTK2	[activation]
28657	EVL	FYN	[activation]
28658	SRPK1	ALKBH3	[activation]
28659	MAPK1	PPARG	[activation]
28660	RHOB	ARHGEF3	[activation]
28661	BRAF	TIMM50	[activation]
28662	PTPN6	GRB2	[activation;inhibition]
28663	RHOD	IHH	[activation]
28664	PAK3	MYO6	[activation]
28665	PDGFRB	SLA	[activation]
28666	MAGED2	LRRK2	[activation]
28667	YWHAE	HSF1	[activation]
28668	PRKACA	GAD1	[activation]
28669	GNAQ	HTR6	[activation]
28670	DYSF	FAM65B	[activation]
28671	DAG1	FYN	[activation]
28672	ADA	GRB2	[activation]
28673	TNS1	PTK2	[activation]
28674	CACNA1A	HHATL	[inhibition]
28675	PLCG1	FGFR4	[activation]
28676	NUP214	VCAM1	[activation]
28677	HMGCS2	YWHAZ	[activation]
28678	ARPC5	ARPC3	[activation]
28679	ASB6	FGG	[activation]
28680	MYB	SP100	[activation]
28681	BMPR1B	KLHL1	[activation;inhibition]
28682	STK4	STAT1	[activation]
28683	KCNQ1	KCNE2	[activation]
28684	C6orf136	PLK1	[activation;inhibition]
28685	SUMO1	THOP1	[activation]
28686	FURIN	PDGFA	[activation]
28687	SOCS1	TEC	[inhibition]
28688	TP53BP1	ACR	[activation]
28689	CASC1	PPP1CA	[activation;inhibition]
28690	LCP2	SHC1	[activation]
28691	ZBTB17	HSPB1	[activation]
28692	FANCA	MKNK1	[activation]
28693	C1R	C1QB	[activation]
28694	PRKCB	GABRG2	[activation]
28695	PLK1	CTBP1	[activation]
28696	AURKB	MTMR1	[activation]
28697	CHD1L	RPA3	[activation]
28698	EGFR	PNPLA2	[activation]
28699	MYBL2	LIN52	[inhibition]
28700	SRSF5	ESR2	[activation]
28701	EGFR	SNRPA1	[activation]
28702	UNC5B	NEO1	[activation]
28703	PRDM6	EHMT1	[activation]
28704	TLR9	TRAF3	[activation]
28705	LMTK2	CDK5R1	[activation]
28706	VAV2	BZW1	[activation]
28707	IKBKB	PPM1B	[activation]
28708	USP9X	PSD4	[activation]
28709	PRKG1	CRIP2	[inhibition]
28710	COL3A1	FGF7	[activation]
28711	PIN1	MTFR1	[activation]
28712	ITGA4	PAFAH1B3	[activation]
28713	DDX24	TMEM9B	[activation]
28714	HSPB1	INS	[activation]
28715	PRKCD	ACTA1	[activation]
28716	YWHAE	CRTC1	[activation]
28717	FGF3	FGFR3	[activation]
28718	RAPGEF1	NEDD9	[activation]
28719	APPL1	RHEBL1	[activation]
28720	HLA-B	PEBP1	[inhibition]
28721	CASP1	NOD1	[activation]
28722	STAT1	STAT2	[activation]
28723	DEFA1	SERPINF2	[activation]
28724	FANCG	GABPB1	[activation]
28725	EGFR	MAST1	[activation]
28726	EZH2	MAPKAPK3	[activation]
28727	GPR3	ARRB2	[activation]
28728	SMAD5	SMURF1	[inhibition]

28729	AURKB	KIF5B	[activation]
28730	GSTK1	EHD4	[activation]
28731	YWHAG	KIAA0930	[activation]
28732	LYN	LIME1	[activation]
28733	MYOD1	SMAD4	[activation]
28734	EIF3F	EIF4G1	[activation]
28735	DAG1	CAV1	[activation]
28736	MYC	PML	[activation]
28737	NCOA3	MAPK1	[activation]
28738	CDKL3	CDK6	[inhibition]
28739	TRIP6	DHX37	[activation]
28740	TNK2	AR	[activation]
28741	FAM19A4	APP	[activation]
28742	PRC1	CCNB1	[inhibition]
28743	IL13	IL13RA2	[activation]
28744	SH3D21	EGFR	[activation]
28745	CEBPB	SMAD3	[activation]
28746	PRKACA	CALD1	[activation]
28747	PAK1	ARHGEF6	[activation]
28748	YWHAB	CDC25B	[activation]
28749	AARS2	ICT1	[activation]
28750	TNF	AGO2	[activation]
28751	PXK	PIK3R1	[activation]
28752	NFATC1	EGR1	[activation]
28753	MYC	MAP2K1	[activation]
28754	BAZ1B	IGHM	[activation]
28755	PRKACG	TNP2	[activation]
28756	HLA-A	STAT3	[activation]
28757	YWHAZ	PRDX2	[activation]
28758	ARRB2	MCM3	[activation]
28759	IGHG1	KLK3	[activation]
28760	PLK1	FADD	[activation]
28761	SHC1	DOK1	[activation]
28762	LGALS13	ACTG1	[activation]
28763	MYB	ADAM22	[activation]
28764	LCK	CD3E	[activation]
28765	SMAD2	EPB41L5	[inhibition]
28766	PDIA4	VWF	[activation]
28767	APP	RASGRP2	[activation]
28768	VCAM1	CORO1C	[activation]
28769	CALM1	GAP43	[activation]
28770	ATF2	FOS	[activation]
28771	IRAK3	HSP90AB1	[inhibition]
28772	PDGFA	COL5A1	[activation]
28773	EGFR	ERCC4	[activation]
28774	ADAM17	EED	[activation]
28775	CTNNBL1	HSPA4	[inhibition]
28776	MVP	NOTCH2NL	[activation]
28777	MYO1C	MCM7	[activation]
28778	HNRNPL	SMYD2	[activation]
28779	MST1R	EPOR	[activation]
28780	PPARG	PML	[activation]
28781	MAPK1	NR3C1	[activation]
28782	PITX2	MYC	[activation]
28783	MAP2K4	CFLAR	[activation]
28784	F12	MMP14	[activation]
28785	IPCEF1	CYTH4	[activation]
28786	VCAM1	NUP210	[activation]
28787	GPR1	TPST1	[activation]
28788	CD55	CD14	[inhibition]
28789	MLST8	AKT1S1	[activation;inhibition]
28790	MVP	RIMBP3	[activation]
28791	CCDC53	HSPB1	[activation]
28792	CDK13	ARRB2	[activation]
28793	AFAP1	PRKCB	[activation]
28794	CRKL	MOV10	[activation]
28795	RAB5A	RAB4A	[activation]
28796	NR3C1	FYN	[activation]
28797	EIF2B1	EIF2B3	[activation]
28798	SCRIB	MAP3K3	[activation]
28799	LATS1	PTEN	[activation;inhibition]
28800	STX6	STX4	[activation]
28801	PIK3R1	GLTSCR1	[activation]
28802	UBC	ERO1L	[activation]
28803	APC	NUPL2	[activation]
28804	WNT2	UQCR11	[activation;inhibition]

28805	NEK9	MDM2	[activation]
28806	ACTB	DSTN	[activation]
28807	CDK6	CASC3	[activation]
28808	LRCH3	LRCH1	[activation]
28809	PIM1	BMX	[activation]
28810	APP	C11orf45	[activation]
28811	RAF1	HSP90AA1	[activation]
28812	AIRE	CRK	[activation]
28813	SHANK1	STAT1	[activation]
28814	RASA1	AURKB	[activation]
28815	WDHD1	SRC	[activation]
28816	CDK5R1	ACTN1	[activation]
28817	FGFR2	LYN	[activation]
28818	ATF4	FOS	[activation]
28819	HOXB4	MEIS1	[activation]
28820	ACTB	APP	[activation]
28821	GLTSCR1	MYC	[activation]
28822	TRAF2	DLGAP5	[activation]
28823	ABL1	SPRR2A	[activation]
28824	TP53BP1	ATM	[activation]
28825	FYB	FER	[activation;inhibition]
28826	MTPN	TRAF6	[activation]
28827	FANCL	DOCK8	[activation]
28828	PGK1	UBA5	[activation]
28829	NUP88	MCM7	[activation]
28830	RAD51C	APP	[activation]
28831	IQGAP1	RPA1	[activation]
28832	ILK	PCID2	[activation]
28833	TRAF6	LAT	[activation]
28834	NDUFS3	ILK	[activation]
28835	ACTB	PPP1CA	[activation]
28836	KIT	CSF2RB	[activation]
28837	ESR1	RELA	[activation]
28838	IRS2	BCL2	[inhibition]
28839	YWHAH	REM1	[activation]
28840	C7orf25	MTOR	[activation]
28841	FBXO28	LYN	[activation]
28842	PPP1R11	LATS1	[inhibition]
28843	RCHY1	CHEK2	[activation;inhibition]
28844	PTPRJ	PKP4	[activation]
28845	SRSF6	ESR1	[activation]
28846	CAMK1	CCND1	[activation]
28847	NDUFS1	CASP3	[activation]
28848	CENPC	MAPK14	[activation]
28849	ITK	WAS	[activation]
28850	ARHGDIB	CASP7	[activation]
28851	EXOC7	CDC5L	[activation]
28852	PIK3R1	TGOLN2	[activation]
28853	STK4	SAV1	[activation]
28854	BRD4	PIK3R1	[activation]
28855	YWHAH	IRS2	[activation]
28856	YWHAZ	EIF4G1	[activation]
28857	PPP2CA	PPP2R2C	[inhibition]
28858	NTRK2	FRS2	[activation]
28859	PRPSAP1	ILK	[activation]
28860	KRT23	APC	[inhibition]
28861	ACTA1	KIF23	[activation]
28862	BHLHE40	SETDB1	[activation]
28863	KAT2B	HIST1H2BB	[activation]
28864	TPT1	SMAD2	[activation]
28865	HIST3H3	MTA2	[activation]
28866	EGFR	AURKA	[activation]
28867	DNAJA3	IFNGR2	[inhibition]
28868	BAG2	MOS	[inhibition]
28869	SRC	TRAF6	[activation]
28870	TRAF2	USF1	[activation]
28871	CDC16	SH3KBP1	[activation]
28872	PLEK	TGFBR1	[activation]
28873	BDKRB1	KNG1	[activation]
28874	NDEL1	KATNA1	[activation]
28875	UBE2I	NR3C1	[activation]
28876	MDM4	TRIM46	[activation;inhibition]
28877	CUL1	RAC2	[activation]
28878	ANK1	TGFBR1	[activation]
28879	CACNA1C	HDAC4	[activation]
28880	TSPAN7	CACNA1A	[inhibition]

28881	DAB1	HMBOX1	[activation]
28882	NEDD1	HSPA1L	[activation]
28883	BRCA1	CREBBP	[activation]
28884	YWHAE	PPM1B	[activation]
28885	TDRD7	NRAS	[activation]
28886	RPTOR	MAPK1	[activation;inhibition]
28887	ZAK	SFN	[activation]
28888	MAP3K5	SOCS3	[inhibition]
28889	BCR	VAV1	[activation]
28890	PALB2	FAM84B	[activation]
28891	CSNK1A1	ITGA4	[activation]
28892	TNXB	E2F3	[activation;inhibition]
28893	SMAD4	MAX	[inhibition]
28894	MYBL2	LZTR1	[inhibition]
28895	CDC5L	TIAL1	[activation]
28896	SFN	SGK223	[activation]
28897	BARD1	BCCIP	[activation]
28898	SPIB	CEBPB	[activation]
28899	EGFR	SOCS1	[inhibition]
28900	HIST3H3	NCOA2	[activation]
28901	CRKL	GABARAPL1	[activation]
28902	BEX1	SMAD4	[activation]
28903	STK3	STRN3	[activation]
28904	CHRM2	GPRASP2	[activation]
28905	MRPS22	TP53	[activation]
28906	CHEK2	MDC1	[activation]
28907	DCN	MMP3	[activation]
28908	GP6	LYN	[activation]
28909	SMAD2	FOXO3	[inhibition]
28910	LDOC1	LATS1	[inhibition]
28911	MDM2	NRAS	[activation]
28912	SRPK1	GABARAP	[activation]
28913	ATF1	HNF1B	[activation]
28914	CTNNB1	PTPN1	[activation]
28915	CASP4	UFL1	[activation]
28916	STX7	CREB3	[activation]
28917	RNF8	CHEK2	[activation]
28918	SKP2	FKBPL	[activation]
28919	CCT6A	ILK	[activation]
28920	PFKFB1	PASK	[activation]
28921	SKP1	MAP4K1	[inhibition]
28922	NCOA2	CTNNB1	[activation]
28923	MAPK1	PPP1CA	[activation;inhibition]
28924	NXT1	NXF2	[activation]
28925	ST3GAL3	ACTG1	[activation]
28926	BANP	MDM2	[activation]
28927	WISP1	DCN	[inhibition]
28928	YWHAE	VAMP8	[activation]
28929	TLR2	TLR6	[activation]
28930	SMAD4	PPP3R2	[activation]
28931	ZIC2	PRKDC	[activation]
28932	STX1A	KCNB1	[activation]
28933	ITGA4	RPS17	[activation]
28934	JUP	GJB1	[activation]
28935	KMT2A	INS	[activation]
28936	CDC7	ORC6	[activation]
28937	ACVRL1	BAMBI	[inhibition]
28938	IL2	NGFR	[activation;inhibition]
28939	ZAK	DDAH2	[activation]
28940	POLR2A	EGLN2	[activation]
28941	ATG16L1	CASP6	[activation]
28942	ARVCF	CDH2	[activation]
28943	PML	STAT3	[activation]
28944	KDR	GNA11	[activation]
28945	PAG1	RASA1	[activation]
28946	CRK	DSC1	[activation]
28947	KMT2E	OGT	[activation]
28948	LHCGR	SLC9A3R1	[activation]
28949	DDX21	MDM2	[activation]
28950	PSEN1	PSEN2	[activation]
28951	RAN	MBP	[activation]
28952	RAB5C	APP	[activation]
28953	TRAF2	DIABLO	[activation]
28954	ELAVL1	FGF21	[activation]
28955	CDKN1A	NR1H2	[activation]
28956	ZNFX1	MEGF10	[activation]

28957	BID	ITCH	[activation]
28958	SERPINB4	MAPK8	[inhibition]
28959	FYN	SOS2	[activation]
28960	PTK2	HES1	[activation]
28961	NKX2-1	NCOA2	[activation]
28962	MAP3K1	EPHA1	[activation]
28963	ANKRD13C	PTGDR	[activation]
28964	PRNP	ERI3	[activation]
28965	PTEN	PDGFRA	[activation]
28966	GDI1	FTSJ1	[activation]
28967	KPNB1	RAPGEF3	[activation]
28968	TP53	CCT8	[activation]
28969	CD3E	CD3EAP	[activation]
28970	VCAM1	GLRX3	[activation]
28971	KAT2B	HIST2H2AC	[activation]
28972	C8orf59	LRRK2	[activation]
28973	ENO2	TINF2	[activation]
28974	ACTB	MAP2	[activation]
28975	RARS	VCAM1	[activation]
28976	ANAPC7	ANAPC16	[activation]
28977	MAPKAPK5	SRPK1	[activation]
28978	LIN37	LIN9	[inhibition]
28979	TAF1B	MYC	[activation]
28980	RARA	TRIM24	[activation]
28981	SMAD3	HSPA4	[inhibition]
28982	ANXA5	BAG3	[activation]
28983	SMARCC2	GATA1	[activation]
28984	CFL1	ATF2	[activation]
28985	SGK1	SLC1A3	[activation;inhibition]
28986	KDR	COL18A1	[activation]
28987	CDKN2A	PRKCA	[activation;inhibition]
28988	TTC27	EGFR	[activation]
28989	SKP2	MYBL2	[inhibition]
28990	FGFR4	OSGIN1	[activation]
28991	RANBP9	NR3C1	[activation]
28992	ACTB	ITGA4	[activation]
28993	GAPDH	GRB2	[activation]
28994	TGM2	ITGB1	[activation]
28995	ATRIP	MOS	[activation]
28996	ARHGDIA	FAS	[activation]
28997	FBP2	MAP2K4	[activation]
28998	DOK4	RASA1	[activation]
28999	PCDHA7	ABL1	[activation]
29000	PPARG	ROBO4	[activation]
29001	CBL	PTPN6	[activation;inhibition]
29002	TP53	HDAC9	[activation]
29003	SFN	LRRK2	[activation]
29004	SOCS1	CYTIP	[inhibition]
29005	EP300	HOXD10	[activation]
29006	YWHAE	ARHGEF2	[activation]
29007	TNFRSF12A	TRAF3	[activation]
29008	FYN	CNN1	[activation]
29009	RBBP4	ZFPM1	[activation]
29010	FBXO9	CALM1	[activation]
29011	VAV2	RAC1	[activation]
29012	FTSJ1	ANXA2	[activation]
29013	SERPINA4	GADD45G	[inhibition]
29014	FZR1	MOAP1	[activation]
29015	MTOR	RAB1A	[activation]
29016	PLG	SERPING1	[inhibition]
29017	EGFR	NUMB	[activation]
29018	HSBP1	HSF1	[activation]
29019	SYNGAP1	ULK2	[inhibition]
29020	AKT1	BUB1	[activation]
29021	BRCA1	ATF1	[activation]
29022	DMC1	RAD51	[activation]
29023	NFKBIA	HOXA9	[activation]
29024	MTA3	CDH1	[activation]
29025	PRKACA	RASGRP3	[activation]
29026	PRPF4B	SRPK3	[activation]
29027	FBXL21	CUL1	[inhibition]
29028	DPYSL4	DPYSL2	[activation]
29029	ATR	POLN	[activation]
29030	RXRA	ACVR1B	[inhibition]
29031	CTNNA1	ARRB2	[activation]
29032	CCNA1	SKP2	[activation]

29033	EVL	ABL1	[activation]
29034	LPAR1	ARHGEF11	[activation]
29035	DOK4	FYN	[activation]
29036	CDX2	MAPK14	[activation]
29037	SHC1	RB1	[activation]
29038	HIPK2	EP300	[activation]
29039	AR	LCK	[activation]
29040	TGOLN2	GRB2	[activation]
29041	NUP214	SUMO2	[activation]
29042	RXRA	BRD8	[inhibition]
29043	GNAI2	TSHR	[activation]
29044	TP53	FLNA	[activation]
29045	DVL3	PIK3CB	[activation]
29046	NTRK3	JUN	[activation]
29047	PIK3R2	ABI1	[activation]
29048	SDC2	PYHIN1	[activation]
29049	SRPK1	SLC4A1AP	[activation]
29050	EGFR	DARS	[activation]
29051	KCNAB2	RPA3	[activation]
29052	CASP3	MDC1	[activation]
29053	GFPT1	PRKAA2	[activation]
29054	CREBBP	RPA2	[activation]
29055	ERH	PVRL4	[activation]
29056	SRSF10	SRSF1	[activation]
29057	SUMO2	AURKB	[activation]
29058	PORCN	WNT7B	[activation]
29059	MDM2	RPS17	[activation]
29060	PAX3	FYN	[activation]
29061	IFIT1	IFIT2	[inhibition]
29062	PAXIP1	SRSF1	[activation]
29063	TIMM50	RAF1	[activation]
29064	ACTN1	ESR1	[activation]
29065	VAV2	GRB2	[activation]
29066	RPS6KA3	HMGN1	[activation]
29067	KPNB1	CDK11A	[activation]
29068	SMAD4	SUMO1	[activation]
29069	TCF4	INS	[activation]
29070	PAXIP1	EP300	[activation]
29071	ART3	PRKCD	[activation]
29072	ERCC4	TADA2A	[activation]
29073	SUMO3	EHMT1	[activation]
29074	HSPA8	HIF1A	[inhibition]
29075	NDEL1	CCSER1	[activation]
29076	SDC3	EPHB4	[activation]
29077	STAT1	PDGFRA	[activation]
29078	BARD1	KPNA3	[activation]
29079	TGFBR1	SKAP2	[inhibition]
29080	FCHO1	ACVR1	[activation;inhibition]
29081	SUMO4	IDH1	[activation]
29082	PDIA6	TNFRSF14	[activation]
29083	CYC1	CYCS	[activation]
29084	LCA5	SRPK2	[activation]
29085	TTN	SRPK2	[activation]
29086	CTNNB1	PYGO1	[activation]
29087	LATS1	NPHP4	[inhibition]
29088	SLA	GAB1	[activation]
29089	GABARAPL2	YWHAE	[activation]
29090	NF1	DNAJC7	[activation]
29091	LAT	LCK	[activation]
29092	PLG	HSPB1	[inhibition]
29093	DSG1	MAPK7	[activation]
29094	RTN1	BCL2L1	[activation]
29095	ELN	MMP9	[activation]
29096	TBC1D3F	RAC1	[activation]
29097	SRC	CSNK2A1	[activation]
29098	CASP3	IL18	[activation]
29099	MAPK10	ARRB2	[activation]
29100	FYN	CNTN1	[activation]
29101	HTR2C	GNAQ	[activation]
29102	HSPA8	MYC	[inhibition]
29103	CRIP2	KLF10	[activation]
29104	YWHAB	TBC1D22B	[activation]
29105	CDC16	SMAD3	[activation]
29106	RUNX1	JUN	[activation]
29107	LIG3	ABL1	[activation]
29108	PRKDC	ABL1	[activation]

29109	CUL1	HSPA6	[inhibition]
29110	CRK	CNTFR	[activation]
29111	PTPN1	GHR	[activation]
29112	RASSF4	STK4	[activation]
29113	SPDYA	CDK2	[inhibition]
29114	STK4	RANGAP1	[activation]
29115	MAST2	YWHAE	[activation]
29116	SLC6A9	PRKCA	[activation]
29117	ADRBK1	CCR4	[activation]
29118	NR1H3	BARD1	[activation]
29119	PRKCB	VTN	[inhibition]
29120	NUDC	VCAM1	[activation]
29121	PRKDC	TRADD	[activation]
29122	CD247	DOCK2	[activation]
29123	MAGEA1	SELPLG	[activation]
29124	TP53BP1	PLK1	[activation]
29125	APP	CCND3	[inhibition]
29126	CDKN2AIP	BARD1	[activation]
29127	RAD21	MCM2	[activation]
29128	ESR2	ARPC2	[activation]
29129	HIST1H2AG	IL33	[activation]
29130	PRPF38A	SRPK1	[activation]
29131	TRAK1	MYC	[activation]
29132	TUB	LCK	[activation]
29133	SEMA3F	NXF1	[activation]
29134	TAB2	IRAK1	[activation;inhibition]
29135	TIAM2	H2AFX	[activation]
29136	CCDC36	FANCL	[activation]
29137	NOS3	CDC37	[activation]
29138	TXN2	TIFA	[activation;inhibition]
29139	SSX2IP	GEM	[activation]
29140	CTNND1	ATF2	[activation]
29141	CCAR2	CCNA1	[inhibition]
29142	ITGA4	LARP1	[activation]
29143	NOTCH2NL	TNIP3	[activation]
29144	DDX58	TMEM173	[activation]
29145	KCNB2	KCNV1	[activation]
29146	TRAF5	MDM2	[activation]
29147	LRP1	DAB2	[activation]
29148	TLK1	NBN	[activation]
29149	MASP1	RPS6KA3	[activation]
29150	FBXO6	PLXNB2	[activation]
29151	POU5F1	MNAT1	[activation]
29152	RPS6KA6	HSP90AB1	[activation]
29153	TP53	BRD8	[activation]
29154	CTNNB1	CCNE1	[activation]
29155	SIRT3	MAP2K5	[activation]
29156	DERL1	SHH	[inhibition]
29157	TIMP3	MMP14	[activation]
29158	LRRK2	LGALS8	[activation]
29159	MCM3	PAK2	[activation]
29160	MAPK14	AES	[inhibition]
29161	SMARCB1	KMT2A	[activation]
29162	PUM1	EGFR	[activation]
29163	NGFR	TRAF2	[activation]
29164	PRKCA	ATP1A1	[activation]
29165	CRK	PCDHA1	[activation]
29166	PPIF	SLC25A4	[inhibition]
29167	ARF4	ADRB2	[activation]
29168	ASB2	SKP2	[inhibition]
29169	HSPB1	TGFB1I1	[activation]
29170	CDK11B	YWHAG	[activation]
29171	KRT31	CRH	[activation]
29172	CUL1	ENO2	[activation]
29173	CD79B	CD3E	[activation]
29174	RANBP9	SMAD2	[activation]
29175	MAP3K3	HSPA1L	[activation]
29176	USP7	PTEN	[activation]
29177	YWHAE	DYRK1A	[activation]
29178	SMC2	TNFRSF10D	[activation]
29179	FANCG	ERCC4	[activation]
29180	ICAM1	CSNK2A1	[activation]
29181	ERCC4	GTF2H1	[activation]
29182	RALA	HRAS	[activation]
29183	NGFRAP1	MIF	[activation]
29184	RGS2	LIG1	[activation]

29185	H2AFX	TSSK6	[activation]
29186	CAPN1	ECT2	[activation]
29187	BMI1	ACACA	[activation;inhibition]
29188	MYL6	MLH1	[activation]
29189	CD28	DOK1	[activation]
29190	RAB25	ITGB1	[activation]
29191	EGLN3	EGLN1	[activation]
29192	TNFRSF4	TRAF2	[activation]
29193	CDC42	MAP3K4	[activation]
29194	KIAA0930	YWHAB	[activation]
29195	PTX3	C1QA	[activation]
29196	KDM6A	POU5F1	[activation]
29197	PTRH2	PTK2	[activation]
29198	TP53	SNRPN	[activation]
29199	SUMO3	NBN	[activation]
29200	MOK	WDR18	[inhibition]
29201	FHL2	SPHK1	[inhibition]
29202	EIF4A3	SMG1	[activation]
29203	MYC	ACOT8	[activation]
29204	LRRK2	CSNK1A1	[activation]
29205	NUDC	POC1A	[activation]
29206	SKP2	NUDCD3	[inhibition]
29207	PTPRG	WAS	[activation]
29208	PRKCI	IKBKG	[activation]
29209	DTX1	CRK	[activation]
29210	CTNNA1	UBE2I	[activation]
29211	DOK1	DOK2	[activation]
29212	EXTL1	GRB2	[activation]
29213	HDAC1	SMARCB1	[activation]
29214	LRRK1	VAPA	[activation]
29215	GABARAPL2	MARS	[activation]
29216	UBTF	LEF1	[activation]
29217	CAMK2A	CDK5R2	[activation]
29218	CTNNBIP1	PNMA2	[inhibition]
29219	ANXA1	FLOT2	[activation]
29220	RIMS3	ZDHHC17	[activation]
29221	ELMO2	RHOG	[activation]
29222	FAM84B	FGFR4	[activation]
29223	AJAP1	MYC	[activation]
29224	CDC5L	ACTR2	[activation]
29225	ITGA9	ITGB1	[activation]
29226	AGER	HMGB1	[activation]
29227	APP	PSEN2	[activation]
29228	WIZ	CDKN1A	[activation;inhibition]
29229	KCNQ2	KCNQ1	[activation]
29230	KIAA0408	YWHAG	[activation]
29231	PPP1R3B	IFNAR2	[activation]
29232	ROBO1	MAPK14	[activation]
29233	MT2A	GPR50	[activation]
29234	DNM2	EGFR	[activation]
29235	TRAF2	HSPA8	[activation]
29236	FGFR3	EPHA4	[activation]
29237	BACH1	NBN	[activation]
29238	ARID1A	GATA1	[activation]
29239	STX4	NAPA	[activation]
29240	APBB1	NOTCH1	[activation]
29241	SEMA4F	NRP2	[activation]
29242	PVR	TNFRSF12A	[activation]
29243	GNAI3	CCDC88C	[activation]
29244	CSNK1G1	LRRK2	[activation]
29245	CD7	NFKBIA	[activation]
29246	TP53	KMT2E	[activation]
29247	RELA	ACTG1	[activation]
29248	MAP1LC3B	HSPA7	[activation]
29249	DSTN	ACTG1	[activation]
29250	CSK	LILRB1	[activation]
29251	NRP1	TGFBR2	[activation]
29252	PLCG1	SH2D2A	[activation]
29253	RELA	CARM1	[activation]
29254	HACE1	RAC1	[activation]
29255	DSG2	PSEN1	[activation]
29256	DRD5	GNAZ	[activation]
29257	FANCC	SRC	[activation]
29258	CFL2	STK11	[activation]
29259	SRPK1	ASAH1	[activation]
29260	NIN	PIAS1	[inhibition]

29261	ERBB2IP	MYC	[inhibition]
29262	MYC	DCLK1	[activation]
29263	SMARCA5	NOTCH1	[activation]
29264	CDC7	CDK9	[activation]
29265	RDX	ICAM2	[activation]
29266	PAXIP1	MT2A	[activation]
29267	PRPF6	CTNNB1	[activation]
29268	YY1	SMAD2	[activation]
29269	LTA	LGALS2	[activation]
29270	SMAD3	GLI3	[activation]
29271	ACTB	H2AFX	[activation]
29272	BID	ZHX1	[activation]
29273	FSTL1	BMPR1A	[activation]
29274	RASA1	PTPRC	[activation]
29275	MAPK9	DUSP19	[inhibition]
29276	SH3D19	GRB2	[activation]
29277	PHACTR4	CALM1	[activation]
29278	SRPK1	PHF7	[activation]
29279	KAT2A	EP300	[activation]
29280	TBRG4	AGTRAP	[activation]
29281	MAPK12	EEF2K	[activation]
29282	PTPN11	INSR	[activation;inhibition]
29283	DLG3	NLGN3	[activation]
29284	HRAS	CDC25C	[activation]
29285	MMP14	SDC1	[activation]
29286	ITGA4	DNM2	[activation]
29287	PIK3R1	SCG2	[activation]
29288	ESR1	CBLL1	[activation]
29289	BTRC	CDK1	[activation]
29290	TNFRSF10B	ARAP1	[activation]
29291	LRRK2	PLEC	[activation]
29292	CDKN1A	CEBPA	[activation;inhibition]
29293	APC	RBM4B	[inhibition]
29294	PDGFRL	HRAS	[activation]
29295	ARHGEF6	EPHB2	[activation]
29296	PYCARD	FSTL5	[inhibition]
29297	DAZAP2	UBE2E2	[activation]
29298	CDK6	DAB1	[activation]
29299	TNF	RNF8	[activation]
29300	AKT2	APPL1	[activation]
29301	CDK2	PTP4A3	[activation]
29302	MYC	ASS1	[activation]
29303	GZMA	LMNB1	[activation]
29304	EGF	ERBB3	[activation]
29305	CSRP1	EGFR	[activation]
29306	SOSTDC1	BMP2	[activation]
29307	LAMTOR5	MYF6	[activation]
29308	ASPSCR1	TACC3	[activation]
29309	KLHL26	NUDC	[activation]
29310	BMPR2	PMPCA	[activation]
29311	PRKAR2B	ARFGEF2	[activation]
29312	SLC25A4	GRB2	[activation]
29313	NPY	MC4R	[activation]
29314	RAP1A	PPP2R1A	[activation]
29315	FBXL18	NOTCH2NL	[activation]
29316	RANGAP1	HLA-B	[activation]
29317	PRKCZ	ESR1	[activation]
29318	SMCHD1	NOTCH1	[activation]
29319	CCNA2	CKS1B	[activation]
29320	PAK2	GIT2	[activation]
29321	STK3	LRRK2	[activation]
29322	FLNB	CRK	[activation]
29323	TBRG4	FAM9B	[activation;inhibition]
29324	NXF1	CDH24	[activation]
29325	GABARAP	HIST1H4A	[activation]
29326	CLIP4	CDK5RAP2	[activation]
29327	PTPN11	CDH5	[activation]
29328	HGS	EHMT2	[activation]
29329	MLKL	ANAPC11	[activation]
29330	MCM2	PLK1	[activation]
29331	NCOR1	RXRA	[inhibition]
29332	ARHGEF25	RAC1	[activation]
29333	FANCG	SPTAN1	[activation]
29334	PPM1J	CSN1S1	[inhibition]
29335	TNF	UBE2N	[activation]
29336	GNAQ	PIK3CA	[activation]

29337	SMC5	YWHAZ	[activation]
29338	PPP2CA	PMS2	[inhibition]
29339	CYLD	DVL1	[inhibition]
29340	CALM1	SCLT1	[activation]
29341	SLC25A5	GRB2	[activation]
29342	POT1	ACD	[activation]
29343	SMAD2	KIAA1033	[activation]
29344	ITGB1	TGFB1I1	[activation]
29345	COL4A4	MMP9	[activation;inhibition]
29346	TGFB1I1	TGFBR2	[activation]
29347	BTK	BCL2L11	[activation]
29348	RCAN1	TRAF6	[activation]
29349	PARD3	RASSF9	[activation]
29350	PCK1	NUP62	[activation]
29351	PAXIP1	EPB41L3	[activation]
29352	ICT1	MRPS27	[activation]
29353	GRB7	PDGFRB	[activation]
29354	PVR	PVRL3	[activation]
29355	IRS2	UBTF	[activation]
29356	CDK18	NTPCR	[activation]
29357	MYC	MRPL53	[activation]
29358	BLNK	CD79A	[activation]
29359	GRN	TOP3B	[activation]
29360	ALK	JAK3	[activation]
29361	MRPL40	STK3	[activation]
29362	RABGGTA	BMPR2	[activation]
29363	PPARA	RXRA	[inhibition]
29364	AMMECR1L	SRPK1	[activation]
29365	PPP2R1A	RAB18	[activation]
29366	LHX2	CITED2	[activation]
29367	SNW1	EIF2S2	[activation]
29368	AR	RANBP9	[activation]
29369	TP53	PNP	[activation]
29370	USP7	RPA3	[activation]
29371	EIF1B	DVL2	[activation]
29372	GNAQ	RGS5	[activation]
29373	FYN	FLT1	[activation]
29374	BAMBI	SMAD7	[inhibition]
29375	LPL	CETP	[activation]
29376	BLM	RAD51D	[activation]
29377	ITGA5	CBL	[activation]
29378	COL4A6	MMP9	[activation;inhibition]
29379	PRKD2	HSP90AB1	[activation]
29380	LRRK2	CUEDC1	[activation]
29381	PRKDC	GRB2	[activation]
29382	PPP2R1A	OBSL1	[inhibition]
29383	ARHGEF6	SMAD1	[activation]
29384	GRB2	CDKL5	[activation]
29385	BRAP	UBE2H	[activation]
29386	SNAI1	KRT40	[activation;inhibition]
29387	TRRAP	MYC	[activation]
29388	AIRE	H2AFX	[activation]
29389	ITGB4	FYN	[activation]
29390	PTEN	RANBP1	[activation]
29391	CDC23	SOX2	[activation]
29392	IL7R	LYN	[activation]
29393	BUB1	SFRP4	[activation]
29394	NFKBIZ	STAT3	[activation]
29395	GPRASP2	CHRM1	[activation]
29396	CDK11A	CDC37	[activation]
29397	RPA1	MSH4	[activation]
29398	ZBTB16	TIMP1	[activation]
29399	CCDC8	GCN1L1	[activation]
29400	CDC5L	DIXDC1	[activation]
29401	NXF1	IGF2BP1	[activation]
29402	SRPK2	GABARAP	[activation]
29403	NOTCH1	PPP2R2A	[activation]
29404	TRIM24	TAF1B	[activation]
29405	ESR1	WHSC1	[activation]
29406	SOX2	FAM21B	[activation]
29407	TIAM1	ABCA1	[activation]
29408	EFEMP1	NOS3	[activation]
29409	SOX2	SMARCA5	[activation]
29410	ARRB2	NFKBIA	[activation]
29411	BCL2L11	DYNLL2	[activation]
29412	GAB2	YWHAB	[activation]

29413	COPA	ARFGAP1	[activation]
29414	MKNK1	PPP2CA	[activation]
29415	PPP2R1A	TRADD	[activation]
29416	HLA-B	CYCS	[activation]
29417	CAMSAP1	SMAD1	[activation]
29418	GABRB1	NOD1	[activation]
29419	MAPK6	SPRR2D	[activation]
29420	YWHAE	ADAM22	[activation]
29421	APPL1	KXD1	[activation]
29422	FURIN	TNFSF13	[activation]
29423	TGFB1I1	SH2D1B	[activation]
29424	RPA2	RAD52	[activation]
29425	ITGB1	ITGA4	[activation]
29426	SSU72	MAPK8	[activation]
29427	KAT2B	E2F1	[activation]
29428	ABI1	FYN	[activation]
29429	CD38	CD247	[activation]
29430	PDGFB	A2M	[activation]
29431	TSC1	RAB11FIP5	[inhibition]
29432	PRKACB	ACVR1B	[activation]
29433	MPP5	LATS1	[inhibition]
29434	NCK1	CCR10	[activation]
29435	RFX6	STK16	[activation]
29436	SRPK1	HSP90AA1	[activation]
29437	OBSL1	TTN	[inhibition]
29438	TYK2	HSPB1	[activation]
29439	APP	RAB2B	[activation]
29440	PFN1	YWHAZ	[activation]
29441	WAS	VASP	[activation]
29442	ETFA	ICT1	[activation]
29443	PRKDC	MBP	[activation]
29444	KITLG	FLT3LG	[activation]
29445	SNAP25	SCAMP1	[activation]
29446	PIAS1	PTPN1	[inhibition]
29447	MLLT4	YWHAQ	[activation]
29448	NINL	JUNB	[activation]
29449	ARRB2	MAP2K4	[activation]
29450	CDK6	MSL3	[inhibition]
29451	BRD4	RELA	[activation]
29452	KIAA0196	RPA1	[activation]
29453	USP8	SPP1	[activation]
29454	CALM1	RYR1	[activation]
29455	ARPC1B	AURKA	[activation]
29456	NOTCH2	MAML3	[activation]
29457	GCN1L1	CAMKK2	[activation]
29458	APP	GTPBP8	[activation]
29459	ULK1	MBP	[inhibition]
29460	NRXN1	APBA2	[activation]
29461	RER1	EGFR	[activation]
29462	GNB2L1	ITGB1	[activation]
29463	IGF1R	GRB10	[activation]
29464	CDC6	CDK2	[activation]
29465	PASK	PIAS1	[inhibition]
29466	NR4A1	ERC1	[inhibition]
29467	AXIN1	PPP2CA	[inhibition]
29468	NR2E3	RXRA	[inhibition]
29469	VCAM1	PGAM5	[activation]
29470	SAMHD1	BARD1	[activation]
29471	SPRR3	LOR	[activation]
29472	SOCS1	RASA1	[inhibition]
29473	STK11	TP53	[activation]
29474	HSP90AB1	RPS6KA5	[activation]
29475	CD4	PIP	[activation]
29476	HIPK3	FAS	[inhibition]
29477	ANAPC5	PTEN	[activation]
29478	BLK	EFS	[activation]
29479	RTCB	OBSL1	[activation;inhibition]
29480	DDX39B	WWOX	[activation]
29481	MYB	NGFRAP1	[activation]
29482	NOTCH1	SMC1A	[activation]
29483	CFL1	NRK	[activation;inhibition]
29484	PRKAG3	PRKAA1	[inhibition]
29485	RIN3	FYN	[activation]
29486	PRR12	PUM1	[activation]
29487	EP300	HDAC1	[activation]
29488	TGFA	ELANE	[activation]

29489	RPA1	RAB6B	[activation]
29490	CHMP4A	ETS1	[activation]
29491	DDX6	SRPK2	[activation]
29492	CDH1	ANAPC7	[activation]
29493	FGFR1	ATG5	[activation]
29494	TJP2	NR3C1	[activation]
29495	SH3KBP1	PIK3R1	[activation]
29496	CFLAR	FAS	[inhibition]
29497	CD2	PTPRC	[activation]
29498	CEBPB	CAMK2A	[activation]
29499	FBXW11	EEF2K	[activation]
29500	ANAPC11	E2F1	[activation]
29501	TNF	HSPB2	[activation]
29502	VCAM1	HNRNPAB	[activation]
29503	UBASH3B	ARAP1	[activation]
29504	CREB1	EP300	[activation]
29505	PDIA4	MDM2	[activation]
29506	LDB1	ISL1	[activation]
29507	BARD1	FKBP3	[activation]
29508	GRB2	RIMS1	[activation]
29509	DAPK3	GRB2	[activation]
29510	TENC1	MET	[activation]
29511	RAC1	PARD6B	[activation]
29512	STRAP	EIF1B	[inhibition]
29513	KPNA2	SRPK1	[activation]
29514	SMAD7	PARD3B	[activation]
29515	JUN	BCL6	[activation]
29516	PTPN11	TEK	[activation;inhibition]
29517	GH1	C12orf23	[activation]
29518	FMNL2	APP	[activation]
29519	YWHAB	PARD3	[activation]
29520	RELA	CEBPD	[activation]
29521	MYC	IQGAP2	[activation;inhibition]
29522	C1QTNF2	NCALD	[activation]
29523	C4BPB	PROS1	[inhibition]
29524	PDGFRL	MAPKAPK3	[activation]
29525	COPS6	PRIM1	[activation]
29526	SKIL	SETDB1	[activation]
29527	YWHAZ	TNK1	[activation]
29528	ATR	SNW1	[activation]
29529	SMG5	TP53	[activation]
29530	CDH1	BOC	[activation]
29531	IL13	IL4	[activation]
29532	RPA2	MCM3	[activation]
29533	MAPK7	PTPRR	[activation]
29534	CASP8	ERRFI1	[inhibition]
29535	IQGAP1	ARF6	[activation]
29536	E2F6	KMT2A	[inhibition]
29537	YWHAZ	IRS1	[activation]
29538	PRKACA	MIP	[activation]
29539	PDP1	PRKCD	[activation]
29540	ACACA	EEF2K	[activation;inhibition]
29541	MYC	RARS	[activation]
29542	RIC8B	GNAS	[activation]
29543	PML	MAPK3	[activation]
29544	CASP7	IKBKE	[activation]
29545	FZD10	HILPDA	[activation]
29546	RHOC	MCC	[activation]
29547	BARD1	ATP1B1	[activation]
29548	DVL1	BUB1	[activation]
29549	NR3C1	FTH1	[activation]
29550	CCHCR1	FSD2	[activation]
29551	CNTF	VHL	[activation]
29552	AKT1	CSF2RB	[activation]
29553	YWHAE	IGF1R	[activation]
29554	ITPR3	OBSL1	[inhibition]
29555	LYN	MME	[activation]
29556	SH2D1A	FYB	[activation]
29557	DAPK1	CALM1	[activation]
29558	MACF1	SOX2	[activation]
29559	JUNB	ATF2	[activation]
29560	LEPR	CSNK2B	[activation]
29561	MDC1	GATA4	[activation]
29562	ATF4	CREBBP	[activation]
29563	CDKN1C	LIMK1	[activation;inhibition]
29564	TAF1	MSX1	[activation]

29565	EIF2B3	EIF2B5	[activation]
29566	SH3GLB2	UBA5	[activation]
29567	PEBP1	MAPK1	[inhibition]
29568	CDC42EP4	RHOQ	[activation]
29569	DDX58	MBP	[activation;inhibition]
29570	EVL	ZDHHC17	[activation]
29571	BRIX1	CD48	[activation]
29572	TRAF3	TLR3	[activation]
29573	AKAP6	GRB2	[activation]
29574	METTL2B	PSEN1	[activation]
29575	MMAB	ICT1	[activation]
29576	MYC	BAHCC1	[activation]
29577	GIT1	RAN	[activation]
29578	LRRK2	MAP2K4	[activation]
29579	CASP4	SNRNP200	[activation]
29580	PLK1	CLIP1	[activation]
29581	RUNX1	TRAF6	[activation]
29582	MAP2	FYN	[activation]
29583	NOTCH2NL	LCE1B	[activation]
29584	FBXO6	POMGNT2	[inhibition]
29585	ANKRD18A	YWHAZ	[activation]
29586	CD40	TRAF2	[activation]
29587	CEACAM1	EGFR	[activation]
29588	GDF9	DLEU1	[activation]
29589	CCL11	TPI1	[activation]
29590	CALM1	LUC7L	[activation]
29591	MYC	CTNND1	[activation]
29592	AR	VAV2	[activation]
29593	PRRC2C	NR3C1	[activation]
29594	MAPT	CASP3	[activation]
29595	NXPH1	NRXN1	[activation]
29596	HDAC1	NFYC	[activation]
29597	APBB1	FASLG	[activation]
29598	YWHAE	KIAA0232	[activation]
29599	EIF3H	ICAM1	[activation]
29600	BRCA1	SP1	[activation]
29601	RPS6KC1	ETS1	[activation]
29602	AURKA	HIST3H3	[activation]
29603	ARHGEF2	YWHAZ	[activation]
29604	EIF2AK4	ARRB2	[inhibition]
29605	CD93	F12	[activation]
29606	USP47	BTRC	[activation]
29607	RRP1B	MYC	[activation]
29608	CDK6	TGFBR1	[inhibition]
29609	RGL2	RAC1	[activation]
29610	SKIV2L	EGFR	[activation]
29611	CASP3	NFE2L2	[activation]
29612	PIK3R1	TGFBR1	[activation]
29613	FMNL1	LSP1	[activation]
29614	HSP90AB1	MYLK4	[activation]
29615	RAP1GAP	MAPK6	[activation;inhibition]
29616	CRK	XPO1	[activation]
29617	TRMT10C	EGFR	[activation]
29618	TGFB2	DCN	[activation]
29619	LRRK1	BCR	[activation]
29620	MAPK14	ATG9A	[activation]
29621	PARP2	H3F3A	[activation]
29622	ASAP1	PTK2	[activation]
29623	MET	SH2D1B	[activation]
29624	KLK2	SERPINF2	[activation]
29625	YAP1	AXIN1	[activation]
29626	GDI2	RAB11A	[activation]
29627	SLX4	IGLC1	[activation]
29628	CAMSAP3	GSK3B	[inhibition]
29629	FGFR1	CREBBP	[activation]
29630	BMPR1B	ARHGEF6	[activation]
29631	EDN1	UBQLN4	[activation]
29632	PLK1	CALML5	[activation]
29633	KCNJ8	KCNJ2	[activation]
29634	DFFA	YWHAZ	[activation]
29635	CALM1	IQGAP3	[activation]
29636	DDX5	NCOA2	[activation]
29637	EP300	HMGN1	[activation]
29638	CASP3	PARG	[activation]
29639	CSF2RA	CHUK	[activation]
29640	ILK	SNRNP35	[activation]

29641	RAD21	CAPN1	[activation]
29642	MDM4	TP73	[activation;inhibition]
29643	MAPKAPK5	HSPB2	[activation]
29644	RB1	CHEK2	[activation]
29645	CMTM5	AACS	[activation]
29646	NFASC	NCK1	[activation]
29647	CACNA1A	C9orf169	[inhibition]
29648	FASLG	EPS8L3	[inhibition]
29649	YWHAB	SRGAP2	[activation]
29650	HAUS5	ILK	[activation]
29651	MCC	RPS6KA3	[activation]
29652	MAML2	CREBBP	[activation]
29653	EIF1B	G3BP2	[activation]
29654	PRKCZ	CSNK2B	[activation]
29655	TP53	SYVN1	[activation]
29656	ITGA4	PICALM	[activation]
29657	STOML3	CAV1	[activation]
29658	GRB2	ZKSCAN3	[activation]
29659	SHB	PIK3R1	[activation]
29660	B2M	HLA-B	[activation]
29661	HRAS	IL3	[activation]
29662	ADRB2	HTR4	[activation]
29663	DHX37	WHSC1	[activation]
29664	PPP2CB	MLH1	[activation]
29665	CDKN1B	RPS6KA1	[activation;inhibition]
29666	E2F1	YWHAQ	[activation]
29667	RANGAP1	CCL5	[activation]
29668	ATF3	IGSF21	[activation]
29669	CORO1B	SSH1	[activation]
29670	PRKACA	VTN	[activation;inhibition]
29671	DLGAP4	ESR2	[activation]
29672	SMURF1	MAP3K2	[inhibition]
29673	RBL2	DYRK1A	[activation;inhibition]
29674	RABAC1	RHOA	[activation]
29675	E2F1	TFDP1	[inhibition]
29676	MLH1	ASS1	[activation]
29677	MRM1	ICT1	[activation]
29678	PLD1	EGFR	[activation]
29679	SORT1	NTRK3	[activation]
29680	CDK5R1	CDKN1B	[activation]
29681	BARD1	HSPA14	[activation]
29682	MON2	TNFRSF1A	[activation]
29683	PPP2R1A	IKBKE	[inhibition]
29684	LUC7L2	NFYA	[activation]
29685	RAPGEF2	CTTNBP2	[activation]
29686	LIG1	CDK2	[activation]
29687	RPS6KA5	SMAD6	[activation]
29688	MAPK1	PPID	[activation;inhibition]
29689	RAB31	MPP3	[activation]
29690	WNK1	SIMC1	[activation]
29691	NUP43	NUP37	[activation]
29692	YWHAH	ZBTB17	[activation]
29693	CTTN	MCM7	[activation]
29694	EHMT2	WIZ	[activation]
29695	RPA3	ACTBL2	[activation]
29696	DNAJA1	CDC16	[inhibition]
29697	GRB2	GSTM1	[activation]
29698	SHC1	IRS2	[activation]
29699	ITPKA	ITSN2	[activation]
29700	TGFBR1	ITGA5	[activation]
29701	COL1A2	MMP9	[activation]
29702	RPS17	ARRB2	[activation]
29703	GAPDH	CDK17	[inhibition]
29704	UBE2E2	RNF166	[activation]
29705	HSPA2	APP	[activation]
29706	MAPK14	PTPRR	[activation]
29707	EIF4A2	MYO1C	[activation]
29708	EHMT1	MPHOSPH8	[activation]
29709	MDM2	ANXA2	[activation]
29710	PNMA1	PRKCI	[activation]
29711	SH2D1A	SLAMF1	[activation]
29712	PTEN	CTNNB1	[activation;inhibition]
29713	HSFY1	AIF1	[activation]
29714	PRKCD	PPP2CA	[activation]
29715	KAT2B	NCOA1	[activation]
29716	GSK3B	FAM83D	[inhibition]

29717	WNK2	YWHAZ	[activation]
29718	ST8SIA4	NCAM1	[activation]
29719	OSMR	IL6ST	[activation]
29720	NBN	PIK3CB	[activation]
29721	ELANE	SERPINF2	[activation]
29722	CAV1	TRAF6	[activation]
29723	CASP8	KLK9	[inhibition]
29724	HUWE1	STMN1	[activation]
29725	APC	FANCC	[activation]
29726	PPP3CB	TBC1D10C	[activation]
29727	TRPV4	PACSIN2	[activation]
29728	KRT40	SEMA4C	[activation]
29729	SMARCB1	RB1	[activation]
29730	NUDC	LRWD1	[activation]
29731	CDH5	CSK	[activation]
29732	HIF1A	CAPN1	[activation]
29733	ATF2	CYCS	[activation]
29734	EIF2S1	EIF4G2	[activation]
29735	PPP2CA	CTDP1	[inhibition]
29736	WDTC1	DDA1	[activation]
29737	EPB41	MIF	[activation]
29738	TXLNA	HYPK	[activation]
29739	SDF2	EGFR	[activation]
29740	TRAF3	TROAP	[activation]
29741	QARS	STK3	[activation]
29742	SMAD2	RASL12	[activation]
29743	YAP1	GSK3B	[inhibition]
29744	HMGN1	YWHAZ	[activation]
29745	EIF4EBP2	EIF4E	[inhibition]
29746	ABCA1	PRKACA	[activation]
29747	GAB2	PLCG2	[activation]
29748	RPS6KA3	CSNK2B	[activation]
29749	PLK1	CDC6	[activation]
29750	KPNA2	GRB2	[activation]
29751	RAD17	FOXO3	[inhibition]
29752	BCL2L11	GIMAP5	[activation]
29753	ITGA4	BOLA2	[activation]
29754	SPERT	EIF4E2	[inhibition]
29755	TRAF6	CYB5B	[activation]
29756	NUP133	MYC	[activation]
29757	GNB5	RPA1	[activation]
29758	ATR	ARHGEF1	[activation]
29759	HTR2A	JAK2	[activation]
29760	RAP1GAP	GNAZ	[activation;inhibition]
29761	RAN	HERC5	[activation]
29762	NOS3	EFEMP2	[activation]
29763	SBK1	FCHSD1	[activation]
29764	IKBKAP	CHUK	[activation]
29765	MMP1	TIMP1	[activation]
29766	CD86	CD80	[activation]
29767	CDH2	NAA25	[activation]
29768	FBXW8	TP53	[activation]
29769	LUC7L2	PTN	[activation]
29770	CDC5L	STK24	[activation]
29771	MCM3	RPA1	[activation]
29772	PPP2R1A	CDK1	[activation]
29773	BTRC	CLSPN	[activation]
29774	GPRASP1	CHRM3	[activation]
29775	HLA-B	SEC23A	[activation]
29776	COA1	STK4	[activation]
29777	SMAD3	SOX9	[activation]
29778	CHEK2	ATR	[activation]
29779	LRRK2	ARPC1B	[activation]
29780	XIAP	RAC1	[activation]
29781	PPP1R12C	CACNA1A	[inhibition]
29782	EEA1	RAB22A	[activation]
29783	IRAK4	IKBKG	[activation]
29784	MDC1	ANAPC1	[activation]
29785	CAV1	FAS	[activation]
29786	BARD1	SNRNP70	[activation]
29787	SHC1	MME	[activation]
29788	NPIPA1	FOS	[activation]
29789	MDM2	ANXA11	[activation]
29790	SDCCAG8	TSC1	[activation;inhibition]
29791	TRPV1	CALM1	[activation]
29792	VRK2	MAPK8	[activation]

29793	BLM	RAD51	[activation]
29794	LCP2	HCK	[activation]
29795	CIAO1	EIF4G2	[activation]
29796	ATG12	TGM3	[activation]
29797	CRYAB	BCL2L1	[activation]
29798	SERPINA5	EP300	[inhibition]
29799	OTUD5	TP53	[activation]
29800	SAV1	IGF2BP2	[activation]
29801	NR4A1	CD2AP	[inhibition]
29802	SRPK2	ENOX2	[activation]
29803	BAAT	MUTYH	[activation]
29804	TRIP10	LYN	[activation]
29805	RPA3	KIAA1033	[activation]
29806	MPRIP	GRB2	[activation]
29807	FES	JAK1	[activation]
29808	VCAM1	GMPS	[activation]
29809	TRAF6	ARRB2	[activation]
29810	MYC	NEK9	[activation]
29811	FBXO5	ANAPC1	[activation]
29812	MIF	TNFRSF10D	[activation]
29813	SUV39H1	EP300	[activation]
29814	SERPINE2	FAM9B	[inhibition]
29815	PAK2	ARHGEF6	[activation]
29816	EPB41L3	EIF4A3	[activation]
29817	VTN	TNFRSF11B	[activation]
29818	SHC1	FASN	[activation]
29819	APP	CEACAM6	[activation]
29820	ATM	BRAT1	[activation]
29821	CRX	EP300	[activation]
29822	SRPK1	NKAP	[activation]
29823	SEMA3F	NRP2	[activation]
29824	GATA1	TRIM25	[activation]
29825	TNRC6B	CTNNB1	[activation]
29826	PARD6A	RICTOR	[activation]
29827	APP	EIF5	[activation]
29828	MDM2	CSNK1A1	[activation]
29829	CHGB	SGK223	[activation]
29830	SRPK2	ARL6IP4	[activation]
29831	YWHAE	YAP1	[activation]
29832	REL	EIF4E2	[inhibition]
29833	ITGAV	ITGB1	[activation]
29834	PTAFR	CALM1	[activation]
29835	CRKL	GRN	[activation]
29836	KRT18	CASP7	[activation]
29837	NCOA2	TP53	[activation]
29838	EBNA1BP2	SRPK3	[activation]
29839	REL	GRB2	[activation]
29840	FOS	TCL1A	[activation]
29841	LYN	CASP7	[activation]
29842	PIAS1	DDX21	[inhibition]
29843	OGG1	ERCC6	[activation]
29844	PPARA	CEP350	[activation]
29845	NIPSNAP1	CDH1	[activation]
29846	GTF2H3	RPA3	[activation]
29847	SPRY1	SOCS4	[inhibition]
29848	PDGFRB	VAV1	[activation]
29849	DYSF	GNL3	[activation]
29850	FERMT3	VCAM1	[activation]
29851	SPP1	PRKCA	[activation]
29852	SKP2	BARD1	[activation]
29853	PRKCZ	PPP3CA	[activation]
29854	PLD1	ACTG1	[activation]
29855	CDK1	PRKCB	[activation]
29856	E2F1	ESR1	[activation]
29857	KAT2B	CIITA	[activation]
29858	TSNAXIP1	HSPB1	[activation]
29859	ICAM1	TUBE1	[activation]
29860	SRSF5	BARD1	[activation]
29861	GPATCH2L	TULP3	[activation]
29862	STAT5A	IL7R	[activation]
29863	HIST3H3	TRPM7	[activation]
29864	APP	GABARAPL2	[activation]
29865	UBE2V1	XIAP	[activation]
29866	HIST3H3	EIF4A3	[activation]
29867	PRKCD	YWHAB	[activation]
29868	LYN	CTLA4	[activation]

29869	CBLB	CD2AP	[inhibition]
29870	MAVS	TICAM1	[activation]
29871	SPP1	APOA1	[activation]
29872	GRIA1	GRIA2	[activation]
29873	MYCN	MAX	[activation]
29874	KMT2A	AR	[activation]
29875	CBL	PTK2	[activation]
29876	EIF2S1	CASP6	[activation]
29877	MORC3	SUMO3	[inhibition]
29878	THRB	CCND1	[activation]
29879	IQGAP1	OBSL1	[inhibition]
29880	PPP1R32	APP	[activation]
29881	TELO2	RPTOR	[activation]
29882	DSG1	CFTR	[activation]
29883	HIST1H4A	RPS6KA5	[activation]
29884	MYO1C	RPA2	[activation]
29885	AVPR1B	PRKCA	[activation]
29886	TP53BP1	CCNB1	[activation]
29887	BIRC3	TNF	[activation]
29888	RAN	PTMA	[activation]
29889	CACNA1S	RYR1	[activation]
29890	DRD5	GNA12	[activation]
29891	FYN	NKX2-1	[activation]
29892	ICAM1	EZR	[activation]
29893	SH3GLB2	STK4	[activation]
29894	VAV3	GAB1	[activation]
29895	TNFSF15	TNFRSF25	[activation]
29896	PIN1	RARA	[activation]
29897	BRAF	SGK1	[activation]
29898	FYN	JAK2	[activation]
29899	PGAM5	CCDC8	[inhibition]
29900	PRKD1	ABL1	[activation]
29901	EIF4G2	CDC5L	[activation]
29902	CDK2	CDKN1A	[activation;inhibition]
29903	CALM1	GP6	[activation]
29904	PRKAR1A	MAPT	[activation]
29905	PPP2R5B	IER3	[activation]
29906	TICAM2	PRKCE	[activation]
29907	SPP1	PARP1	[activation]
29908	BTRC	CRY2	[inhibition]
29909	ARL6IP4	SRPK1	[activation]
29910	SMARCA1	SETD1A	[activation]
29911	STX7	MAPK6	[activation]
29912	CAMK2G	TIAM1	[activation]
29913	MAP1LC3B	PRKDC	[activation]
29914	CDKN1A	INPP5K	[activation]
29915	ERBB3	CSK	[activation]
29916	HTRA1	NOS3	[inhibition]
29917	GRB2	TTYH2	[activation]
29918	VTN	APP	[inhibition]
29919	FANCC	RB1	[activation;inhibition]
29920	TP53	PPP2R2A	[activation]
29921	STAMBP	MAP3K2	[activation]
29922	CREB5	FOS	[activation]
29923	TP53	COLGALT1	[activation]
29924	PAXIP1	SCAMP5	[activation]
29925	ERBB2	PGR	[activation]
29926	HAND1	PRKACA	[activation]
29927	CSK	SHC1	[activation]
29928	SCGB2A2	CASP8	[inhibition]
29929	SMS	RPS6KA3	[activation]
29930	EZR	CAPN6	[activation]
29931	CDK4	DUSP9	[inhibition]
29932	PML	MDM2	[activation]
29933	IRS4	RELA	[activation]
29934	DDX11	PFN2	[activation]
29935	SH2D2A	AR	[activation]
29936	GAD1	JUN	[activation]
29937	FBXW11	MAST3	[inhibition]
29938	ITGA4	PNP	[activation]
29939	PAK1	MYNN	[activation]
29940	IL7R	HNRNPA0	[activation]
29941	ITGA4	EIF4G2	[activation]
29942	CDK4	FKBP5	[inhibition]
29943	MRE11A	CDC5L	[activation]
29944	SYK	FGR	[activation]

29945	CACNA1E	HSPA1A	[inhibition]
29946	CDK7	RPA1	[activation]
29947	SOS2	LOX	[activation]
29948	PPP1R2	GSK3B	[activation;inhibition]
29949	MAPK14	MUC12	[activation]
29950	GUSBP1	TP53	[activation]
29951	MAP3K1	DYRK1A	[activation]
29952	USP11	NOTCH1	[activation]
29953	CFL1	ACTB	[activation]
29954	CRK	CNTNAP1	[activation]
29955	CLIP3	CYLD	[inhibition]
29956	NDP	LRP5	[activation]
29957	EPS8L2	PTK2	[activation]
29958	SOS1	EPS8L2	[activation]
29959	CBL	BLNK	[activation]
29960	LRRK2	CAMK1D	[activation]
29961	MCM3	MCM7	[activation]
29962	CDC5L	EXOC1	[activation]
29963	CCHCR1	HSPB1	[activation]
29964	YWHAZ	TGFBR1	[activation]
29965	GRB7	AGAP1	[activation]
29966	CHEK2	ENO1	[activation]
29967	KPNA2	TP53	[activation]
29968	PPP2R2D	MLH1	[activation]
29969	IRF7	P4HA1	[activation]
29970	RANBP9	ITGAL	[activation]
29971	KMT2A	PSIP1	[activation]
29972	BUB3	WBP4	[inhibition]
29973	CASP3	VAV1	[activation]
29974	CRK	CORO1C	[activation]
29975	ITK	ERBB3	[activation]
29976	PRNP	PPP1R14A	[activation]
29977	OGT	SNAP29	[activation]
29978	TRAF3IP1	TRAF6	[activation]
29979	APP	RPS6KB1	[activation]
29980	HSPBP1	HSF1	[activation]
29981	FBXO6	SCARB1	[activation]
29982	KIAA0922	MYC	[activation]
29983	AP2S1	GRB2	[activation]
29984	MDM2	HDAC1	[activation]
29985	NOD1	CASP8	[activation]
29986	CTDSP2	SNAI1	[activation]
29987	PLEK	ACVR1	[activation;inhibition]
29988	SRPK1	PCBP1	[activation]
29989	SP1	CEBPB	[activation]
29990	IQGAP1	APC	[inhibition]
29991	MAP1LC3B	ULK1	[inhibition]
29992	GC	PIK3R3	[activation]
29993	SRF	CRIP2	[activation]
29994	ROPN1	PRKAA1	[inhibition]
29995	XPO7	MYC	[activation]
29996	PTTG1	CAMK2A	[activation]
29997	NR1H4	EP300	[activation]
29998	DYRK1A	SRSF4	[activation]
29999	SP3	E2F1	[activation]
30000	DNAJB1	PSEN2	[inhibition]
30001	YWHAZ	PPFIBP1	[activation]
30002	SKP2	CDK3	[activation]
30003	EGFR	SUSD1	[activation]
30004	PAX6	SOX10	[activation]
30005	AGT	AGTR2	[activation]
30006	NUP62	KLHL32	[activation]
30007	GH1	RPS19	[activation]
30008	SRC	CBLL1	[activation]
30009	IFNAR1	EP300	[activation]
30010	GOLGA2	ARPC3	[activation]
30011	XPO1	EPAS1	[activation]
30012	MAP2K1	HLA-B	[activation]
30013	PPP6C	MYC	[activation]
30014	FBXO25	IL21	[activation]
30015	CCNE1	BTRC	[activation]
30016	HSPA9	EGFR	[activation]
30017	FSTL1	BMP2	[activation]
30018	GP1BB	GP9	[activation]
30019	SEC31A	APC	[activation]
30020	TP53	MRPS23	[activation]

30021	RELA	IRF2	[activation]
30022	NAA11	HIF1A	[activation]
30023	EHMT2	CTNNA3	[activation]
30024	CSNK2B	NRBP1	[activation]
30025	AURKA	TBC1D2	[activation]
30026	XBP1	HIF1A	[activation]
30027	FYN	NMT1	[activation;inhibition]
30028	MRPL10	EGFR	[activation]
30029	MYOD1	STAT3	[activation]
30030	UBE2I	TP53	[activation]
30031	HSPB1	CAMK1	[activation]
30032	C8orf33	TAOK1	[activation;inhibition]
30033	SH3BP5L	YWHAB	[activation]
30034	RPS6KA3	SHANK1	[activation]
30035	PIK3R1	NCL	[activation]
30036	CDC14A	CHRM1	[activation]
30037	RHPN2	RHOA	[activation]
30038	AIRE	SRC	[activation]
30039	MDM2	NOTCH4	[activation]
30040	TP53	SF3B2	[activation]
30041	CKAP4	MYC	[activation]
30042	CARM1	CEBPB	[activation]
30043	JAG1	CANX	[activation]
30044	EPOR	MAPK3	[activation;inhibition]
30045	GATA1	RAI1	[activation]
30046	PIK3R1	FCGR2A	[activation]
30047	ADCY2	ADCY5	[activation]
30048	NFKBIA	PTPN1	[activation]
30049	GCN1L1	MAGED1	[activation]
30050	YAP1	TP73	[activation]
30051	MAP2K1	PARVA	[activation]
30052	SPP1	TMEM168	[activation]
30053	FGD2	TMEM239	[activation]
30054	ANK1	NFASC	[activation]
30055	VCAM1	SARS	[activation]
30056	CACNA1A	MOAP1	[inhibition]
30057	TAB1	GIT1	[inhibition]
30058	PXN	CASP3	[activation]
30059	BID	TUBB2B	[activation]
30060	TIAM1	CSNK1E	[activation]
30061	PPP1CC	ARFGEF2	[activation]
30062	ESR1	MYL6	[activation]
30063	TRAPPC13	TRAPPC8	[activation]
30064	MGAT5B	CAMK2B	[inhibition]
30065	EGFR	FHL2	[activation]
30066	CRKL	DOK1	[activation]
30067	GDF9	ORAI2	[activation]
30068	CTNNA1	PARD3	[activation]
30069	EZH2	JAK2	[activation]
30070	CRTC2	RFWD2	[activation]
30071	SGTA	NXF1	[activation]
30072	MYOZ1	PPP3CA	[activation]
30073	ABL1	FOS	[activation]
30074	ADRB2	EIF2B1	[activation]
30075	ILK	LIMS1	[activation]
30076	STAT3	STK4	[activation]
30077	PEX19	IL23A	[activation]
30078	FCGR2C	PLCG1	[activation]
30079	DEF6	RAC2	[activation]
30080	WIPF1	ZAP70	[activation]
30081	CHRM1	GPRASP1	[activation]
30082	NR1H2	FOXO3	[inhibition]
30083	CD5	ZAP70	[activation]
30084	CDC37	A2M	[inhibition]
30085	RANBP10	RAN	[activation]
30086	IGF2BP1	IFIT1	[activation]
30087	DAB1	APP	[activation]
30088	RPS6	MTOR	[activation]
30089	RGS17	DHX37	[activation;inhibition]
30090	TGM1	BMI1	[activation]
30091	ANXA2	CDC42	[activation]
30092	PTPN23	GRB2	[activation]
30093	POLR2A	CTR9	[activation]
30094	EIF3D	ETS1	[activation]
30095	GAB1	PDGFRB	[activation]
30096	NKX2-1	GRB2	[activation]

30097	ITGA4	RPL26	[activation]
30098	OS9	DCSTAMP	[activation]
30099	MPRIP	LRRK2	[activation]
30100	VCAM1	TRAP1	[activation]
30101	TSC22D1	TBRG4	[activation;inhibition]
30102	RALGDS	MAPK3	[activation]
30103	BMPER	BMP6	[activation]
30104	PDGFRA	PDGFRB	[activation]
30105	STRADA	CAB39	[activation]
30106	FAM83B	CSNK1E	[activation]
30107	OPRK1	SLC9A3R1	[activation]
30108	TBP	HSF1	[activation]
30109	CNTFR	NCK1	[activation]
30110	ANKZF1	STAT6	[activation]
30111	RAPGEF3	RRAS2	[activation]
30112	EGFR	MAPKAPK3	[activation]
30113	DAAM1	ABL1	[activation]
30114	GRB2	CFL2	[activation]
30115	RASGRF1	ADRBK1	[activation]
30116	INS	MAPK6	[activation]
30117	HOXC6	APC	[inhibition]
30118	A2M	IL4	[activation]
30119	NAA10	ARHGEF7	[activation]
30120	SOS1	GAB1	[activation]
30121	LPA	FGB	[activation]
30122	OGT	TRAK2	[activation]
30123	GLI1	CDK4	[inhibition]
30124	CDH1	ACTR3	[activation]
30125	PVRL2	MDFI	[activation]
30126	SKIL	SNRNP70	[activation]
30127	CD160	MDM4	[inhibition]
30128	MEF2A	MEF2D	[activation]
30129	IL11	MAGEA11	[activation]
30130	EGFR	KCTD9	[activation]
30131	ACTA1	CTNND1	[activation]
30132	PAXIP1	DKC1	[activation]
30133	TP53BP1	MAPK3	[activation]
30134	ATF2	EEF1D	[activation]
30135	FYN	GPR63	[activation]
30136	MYL9	TP73	[activation;inhibition]
30137	GABARAP	ATG5	[activation]
30138	AFF1	RELA	[activation]
30139	STK4	RASSF3	[activation]
30140	SMAD2	CITED2	[activation]
30141	COL4A6	SERPINE2	[inhibition]
30142	CRADD	KCTD9	[activation]
30143	MDM2	HSPB1	[activation]
30144	BRCA1	NFKB1	[activation]
30145	PDGFRA	STAT5A	[activation]
30146	RTN3	FGFR1	[activation]
30147	SELPLG	SELL	[activation]
30148	EXOC8	ULK1	[inhibition]
30149	LDOC1	ATF4	[activation]
30150	EIF4A2	MYO1B	[activation]
30151	SHISA5	TP53	[activation]
30152	MAPK6	ARPC3	[activation]
30153	ACOT8	ISG15	[activation]
30154	HSPA6	NFKB1	[activation]
30155	ZC3HC1	MAPK1	[activation]
30156	PVRL2	PVRL3	[activation]
30157	RPS16	RELA	[activation]
30158	FZR1	SKP2	[inhibition]
30159	SYK	CD72	[activation]
30160	ELK1	MAPK1	[activation]
30161	DDX24	WHSC1	[activation]
30162	SKP1	FBXO27	[inhibition]
30163	CAD	RIPK1	[activation;inhibition]
30164	HOMER1	GRM1	[activation]
30165	H2AFX	CALM1	[activation]
30166	MAP2K7	DUSP19	[inhibition]
30167	MAD2L1	DAXX	[inhibition]
30168	SNAP29	IGF1R	[activation]
30169	ANXA7	PLD3	[activation]
30170	NR2F2	PIAS1	[inhibition]
30171	EP300	RAN	[activation]
30172	FANCA	GNB2	[activation]

30173	DCTPP1	HLA-B	[activation]
30174	PSEN2	BCL2L1	[activation]
30175	SMURF1	SASH3	[inhibition]
30176	ATM	ATMIN	[activation]
30177	MEOX2	NTF3	[activation]
30178	PARD6A	PRKCI	[activation]
30179	EIF2AK2	PPP2R5A	[activation]
30180	FXYD1	ATP1B1	[activation]
30181	NR0B2	EHMT2	[activation]
30182	SRPK2	GPR114	[activation]
30183	FOXA2	MIP	[activation]
30184	ZC3H7A	STK3	[activation]
30185	FBXO6	DNAJC11	[inhibition]
30186	BMPR1B	BMPR2	[activation]
30187	ZC3H7A	PAK1	[activation]
30188	PTBP3	RPRD1A	[activation]
30189	RAN	RGPD5	[activation]
30190	CDH2	CTNND2	[activation]
30191	CHRNA2	CHRNB4	[activation]
30192	UGT1A1	UGT1A9	[activation]
30193	PI4KA	CD4	[activation]
30194	LCK	ESR2	[activation]
30195	APP	CDK5	[activation]
30196	ACVR1	RRAS2	[activation]
30197	SUDS3	SRPK1	[activation]
30198	LAMTOR4	RRAGC	[activation]
30199	PML	EIF4E	[inhibition]
30200	SHB	FGFR1	[activation]
30201	MAPK3	STMN2	[activation]
30202	EIF3D	HLA-B	[activation]
30203	BIRC2	TRAF6	[activation]
30204	POLR2B	MYC	[activation]
30205	PALB2	PDS5B	[activation]
30206	HSPA8	CD40	[activation]
30207	SRC	YWHAH	[activation]
30208	PRKCB	HIST1H3A	[activation]
30209	TRDN	HRC	[activation]
30210	HSPA5	MAP3K7	[activation]
30211	EP400	FOS	[activation]
30212	NCAPG	NCAPD2	[activation]
30213	DLG4	LRP8	[inhibition]
30214	PAFAH1B3	SETDB1	[activation]
30215	CDK4	PPP2R1B	[inhibition]
30216	DOK4	INSR	[activation]
30217	RYR1	S100A1	[activation]
30218	KIT	PTPRU	[activation]
30219	MARS	MAPK14	[activation]
30220	CD19	MAPK1	[activation]
30221	TNPO1	NUP214	[activation]
30222	ENKD1	RAD54B	[activation]
30223	MYC	MCTP2	[activation]
30224	ITSN2	GAREML	[activation]
30225	CNTFR	GRB2	[activation]
30226	CAV1	ABL2	[activation]
30227	BCL2	NRAS	[activation]
30228	TTC1	TGFBR1	[inhibition]
30229	STXBP1	STX3	[activation]
30230	ATF2	IRF2BP1	[activation]
30231	CAMK2G	CABP2	[activation]
30232	SCP2	CAV1	[activation]
30233	YWHAE	REM1	[activation]
30234	RTKN	RHOA	[activation]
30235	PIAS1	ESR2	[inhibition]
30236	PCBP2	VCAM1	[activation]
30237	IKBKB	CSF2RB	[activation]
30238	CCNG1	TP73	[activation]
30239	ITPR1	FYN	[inhibition]
30240	TNFRSF11A	CBL	[activation]
30241	NUP62	NUP93	[activation]
30242	ACVR1B	INHBC	[activation]
30243	BRCA2	BARD1	[activation]
30244	BHLHE40	TP53	[activation]
30245	SRPK1	CSAG1	[activation]
30246	SMAD3	CAMK2G	[activation]
30247	ARF6	MAT2A	[activation]
30248	F11R	ITGAL	[activation]

30249	HNRNPR	LRRK2	[activation]
30250	SUV420H1	SMARCD1	[activation]
30251	JTB	APP	[activation]
30252	GRB2	KCNH7	[activation]
30253	ABL1	BAZ2A	[activation]
30254	EIF3A	ICAM1	[activation]
30255	EFS	CRK	[activation]
30256	GPS2	MSH5	[inhibition]
30257	RNF165	UBE2E3	[activation]
30258	FGFR4	ANGPTL4	[activation]
30259	ARHGAP11A	NXF1	[activation]
30260	GNB2	NR3C1	[activation]
30261	CRK	DLGAP3	[activation]
30262	HSP90AA1	CAMKV	[activation]
30263	NUDC	NWD1	[activation]
30264	PRKDC	AIRE	[activation]
30265	CTSG	SDC1	[activation]
30266	CASP8	PRKCI	[activation]
30267	CNKSR1	CYTH3	[activation]
30268	IL24	SMAD4	[activation]
30269	EP300	SOX9	[activation]
30270	ERBB2IP	PPP1CC	[inhibition]
30271	FRYL	BAK1	[activation;inhibition]
30272	FBXW11	OGT	[activation]
30273	PTPN13	CTNNB1	[activation]
30274	TENC1	ITGB1	[activation]
30275	HSP90AA1	GNAI2	[activation]
30276	SMAD3	YTHDF2	[inhibition]
30277	RIMS4	SPP1	[activation]
30278	GRB7	RET	[activation]
30279	PAK1	ACTA1	[activation]
30280	RHOQ	CDC42BPA	[activation]
30281	CENPJ	CREBBP	[activation]
30282	SH3KBP1	ARAP3	[activation]
30283	PEA15	CASP8	[inhibition]
30284	AR	FES	[activation]
30285	VAMP8	ATP4A	[activation]
30286	GOSR1	STX5	[activation]
30287	NR2F2	SMAD4	[activation;inhibition]
30288	HLA-C	HSPA5	[activation]
30289	TOMM70A	MAVS	[activation]
30290	RASL12	BMPR1B	[activation]
30291	XPO1	BACH1	[activation]
30292	RIPK1	HSP90AA1	[activation]
30293	GAB1	STAP1	[activation]
30294	RANBP1	MDM2	[activation]
30295	PRKCA	HMGA2	[activation]
30296	CYLD	SPATA2	[inhibition]
30297	GABARAP	SRSF10	[activation]
30298	PTGES3	TERT	[activation]
30299	SRPK2	LCE3D	[activation]
30300	HIPK1	RUNX1	[activation]
30301	C21orf2	ATOX1	[activation]
30302	SHOC2	APP	[activation]
30303	YWHAH	TNIP3	[activation]
30304	SRPX2	PLAUR	[activation]
30305	TPT1	APP	[activation]
30306	ROCK1	IKBKB	[activation]
30307	RANGAP1	URM1	[activation]
30308	TP53	RPL27	[activation]
30309	CAMK2B	COL4A3	[inhibition]
30310	EP300	NEDD1	[activation]
30311	GNAI2	GNB4	[activation]
30312	EDEM1	RHO	[activation]
30313	SOS1	TNIK	[activation]
30314	FZR1	CUL1	[inhibition]
30315	EGFR	ILF2	[activation]
30316	CDK2	EP300	[activation]
30317	NOTCH1	USP15	[activation]
30318	HSP90AA1	GNA12	[activation]
30319	RNF219	CD40	[activation]
30320	ETV7	RICTOR	[activation]
30321	SYK	IL15RA	[activation]
30322	SHB	SRC	[activation]
30323	CDK6	NIPBL	[inhibition]
30324	GRB2	BAZ2A	[activation]

30325	ULK1	AMBRA1	[inhibition]
30326	NCAPH	SMC2	[activation]
30327	ARHGEF2	KSR1	[activation]
30328	CBX3	CACNB4	[activation]
30329	NUDC	WDR60	[activation]
30330	F2	ST13	[activation]
30331	MECR	HNF4A	[activation]
30332	TP53	CD40LG	[activation]
30333	NCK1	MAP4K3	[activation]
30334	CALM1	KRAS	[activation]
30335	KIT	PLCE1	[activation]
30336	SNUPN	RAN	[activation]
30337	ANXA7	KLHL23	[activation]
30338	GAPDH	SGK1	[activation]
30339	FASLG	GRAP	[activation]
30340	ABI1	WASF1	[activation]
30341	NRP2	SEMA3C	[activation]
30342	CTNNB1	CHUK	[activation]
30343	ACTB	RAC1	[activation]
30344	GUCY1B3	HSP90AA1	[activation]
30345	E2F1	SP2	[activation]
30346	APP	FAM3B	[activation]
30347	PHF20L1	HIST1H3A	[activation]
30348	YWHAE	SIK1	[activation]
30349	GRB2	POLD1	[activation]
30350	DLG2	NLGN1	[activation]
30351	PIP4K2C	BTK	[activation]
30352	LCE2A	RGS20	[activation;inhibition]
30353	GJB3	CNST	[activation]
30354	MAPK1	SNCG	[inhibition]
30355	LCP2	GRAP2	[activation]
30356	CAMK2G	GRIA4	[activation]
30357	KRAS	IGSF21	[activation]
30358	CRK	APOL5	[activation]
30359	PTPN1	BCAR1	[activation]
30360	ADCYAP1R1	ADCYAP1	[activation]
30361	CSF1	GAB3	[activation]
30362	BMPR2	CRYAB	[activation]
30363	JUN	SIRT1	[activation]
30364	PAFAH1B3	PAFAH1B1	[activation]
30365	NXF1	TLN1	[activation]
30366	USF1	HDAC9	[activation]
30367	JAK1	IL4R	[activation]
30368	ELMO1	RAC1	[activation]
30369	SERF1A	PIK3R3	[activation]
30370	DAPK1	YWHAQ	[activation]
30371	CCNA2	AKT2	[activation]
30372	CAV3	SUMO3	[inhibition]
30373	SMAD1	STARD13	[activation]
30374	GNG2	RPTOR	[activation]
30375	SMAD3	FOXO4	[activation]
30376	IGHM	IGJ	[activation]
30377	SMARCA4	UBE2O	[activation]
30378	CD4	EIF3D	[activation]
30379	PLEKHO1	AKT2	[activation]
30380	BRD7	MAGEH1	[activation]
30381	PIAS2	IFIH1	[inhibition]
30382	UGT1A10	UGT1A3	[activation;inhibition]
30383	PTPRJ	LEPR	[activation]
30384	SH2B2	ERBB2	[activation]
30385	PLCG1	AKAP2	[activation]
30386	TLE1	FOXA2	[activation]
30387	MAP2K2	SNW1	[activation]
30388	PRKCI	MAP2K1	[activation]
30389	USP19	HSPA4	[inhibition]
30390	KLRK1	TYROBP	[activation]
30391	CAPZA1	LRRK2	[activation]
30392	CAMK2A	NR2F6	[activation]
30393	PRKG1	VASP	[activation]
30394	CD9	HBEGF	[activation]
30395	PTGS2	CTNNB1	[activation]
30396	PRKCA	ITPKB	[activation]
30397	RPAP3	MAP3K3	[activation]
30398	HSPH1	RELA	[activation]
30399	PRKDC	NBN	[activation]
30400	TP53	GIGYF2	[activation]

30401	PRKCZ	PRKCI	[activation]
30402	RAP1B	APLP1	[activation]
30403	MAP2K5	NXF1	[activation]
30404	ATG5	HSPE1	[activation]
30405	PPARGC1A	PPARG	[activation]
30406	MAP2K1	LAMTOR2	[activation]
30407	TRAF5	RIPK1	[activation]
30408	PLK1	CDC5L	[activation]
30409	SAMM50	EGFR	[activation]
30410	SUMO3	TP53	[activation]
30411	VCAM1	CAPZB	[activation]
30412	EIF4G2	MKNK1	[activation]
30413	CCR5	STAT1	[activation]
30414	LHCGR	CGB	[activation]
30415	SMAD2	SOX9	[activation]
30416	PLEKHG2	ACTN3	[activation]
30417	ACTB	DAB2	[activation]
30418	CCNA2	NFYB	[activation]
30419	RGS3	GNA11	[activation]
30420	GRN	CACNA1A	[activation]
30421	CTNNB1	GRIK2	[activation]
30422	ANK1	L1CAM	[activation]
30423	PTEN	TAGLN2	[activation;inhibition]
30424	BCL2	CDK1	[inhibition]
30425	MYL6B	ESR1	[activation]
30426	TMEM14A	LXN	[activation]
30427	SRI	SRPK2	[activation]
30428	HIST2H3A	H2AFX	[activation]
30429	CSF2	SYNCRIP	[activation]
30430	ESRRA	KAT2B	[activation]
30431	SRPK1	TAAR8	[activation]
30432	INADL	CRIPT	[activation]
30433	TLR4	LY96	[activation]
30434	NR1H3	SUV39H1	[activation]
30435	KMT2A	MEN1	[activation]
30436	FES	EGFR	[activation]
30437	ITCH	TRPV1	[activation]
30438	ATM	BMI1	[activation]
30439	SNCA	ELK1	[activation]
30440	TNFRSF11A	FHL2	[activation]
30441	RHOA	PLEKHG2	[activation]
30442	DKC1	ESR1	[activation]
30443	CFL1	ROCK1	[activation]
30444	LTBR	DIABLO	[activation]
30445	ANAPC4	PTEN	[activation]
30446	GRB10	CAV1	[activation]
30447	CHUK	PTPN11	[activation]
30448	E2F3	ITGB1	[activation]
30449	CAV1	AKAP1	[activation]
30450	LRRK2	CALML5	[activation]
30451	ZAP70	ANAPC11	[activation]
30452	ANAPC4	PAXIP1	[activation]
30453	CCNA1	TGFB1I1	[activation]
30454	RELA	HMGA2	[activation]
30455	SARM1	TRAF6	[activation]
30456	MTNR1A	HNRNPA0	[activation]
30457	HSPA2	NCOA2	[activation]
30458	GRB2	PRG4	[activation]
30459	RANBP1	NUPR1	[activation]
30460	PRKACA	RRAD	[activation]
30461	SENP3	CDK6	[inhibition]
30462	APP	DDX10	[activation]
30463	ERBB2	NRG1	[activation]
30464	UBE2E1	F12	[activation]
30465	MSH4	ABL1	[activation]
30466	ACTG1	MORF4L2	[activation]
30467	TFE3	MARS	[activation]
30468	SIGLEC6	LEP	[activation]
30469	LCK	NR2F2	[activation;inhibition]
30470	PRKDC	SRF	[activation]
30471	ABL1	C3	[activation]
30472	PRKAR1A	BCL2L11	[activation]
30473	CDK1	FBXO6	[inhibition]
30474	CDC42	FICD	[activation]
30475	HMGB1	MAPK1	[activation]
30476	PTPRC	GRB2	[activation]

30477	YWHAG	FARP2	[activation]
30478	NDEL1	DIXDC1	[activation]
30479	FHL2	HAND1	[activation]
30480	FGF12	MAPK13	[activation]
30481	DLGAP1	PIK3R1	[activation]
30482	GATA4	MAPK1	[activation]
30483	VCAM1	ITGAD	[activation]
30484	SIRT1	ESRRA	[activation;inhibition]
30485	TSC22D3	RELA	[activation]
30486	ACTR3	OBSL1	[activation]
30487	LYN	DOK3	[activation]
30488	STX7	ATP4A	[activation]
30489	LMNB1	EGF	[activation]
30490	ASAP2	GRB2	[activation]
30491	IFRG15	FBXO6	[inhibition]
30492	HIST1H4A	LRWD1	[activation]
30493	WAS	PIK3R1	[activation]
30494	COL1A1	DCN	[activation]
30495	PKM	CTNNB1	[activation]
30496	PCK1	IKBKG	[activation]
30497	MACF1	CNTNAP4	[activation]
30498	VCAM1	RPL26	[activation]
30499	RIPK1	PGAM5	[activation]
30500	YWHAG	PRMT5	[activation]
30501	DSG3	FURIN	[activation]
30502	ITPR1	BCL2	[inhibition]
30503	FBXW11	NFE2L2	[activation]
30504	CTNNB1	CDC27	[activation]
30505	MAPK8	JKAMP	[activation]
30506	DDX54	SEMA4A	[activation]
30507	CDK2AP2	A2M	[inhibition]
30508	ARF1	FBXO6	[activation]
30509	L1CAM	RABGEF1	[activation]
30510	CDH1	HSPA8	[inhibition]
30511	SCAP	VCAM1	[activation]
30512	CASP3	HIP1	[activation]
30513	CD81	HNRNPD	[activation]
30514	PNISR	APC	[inhibition]
30515	PPARA	UBE2I	[activation]
30516	DDA1	AGTRAP	[activation]
30517	ANKRD2	TCAP	[inhibition]
30518	SETDB1	PML	[activation]
30519	GABARAPL2	SRSF10	[activation]
30520	CREB1	PRKG1	[inhibition]
30521	FGFR3	BORA	[activation]
30522	SMC2	HLA-A	[activation]
30523	EGFR	CNTN2	[activation]
30524	DCBLD2	FYN	[activation]
30525	ERBB3	CRKL	[activation]
30526	TRAF3	RIPK2	[activation]
30527	SRC	SOS2	[activation]
30528	TGFB1	ENG	[inhibition]
30529	MAP3K12	HSP90AB1	[activation]
30530	MET	PLXNB1	[activation]
30531	CTNND1	NANOS1	[activation]
30532	NFE2L2	MAPK8	[activation]
30533	SKP1	PPP1CC	[inhibition]
30534	PRMT1	FGF2	[activation]
30535	CA9	XPO1	[activation]
30536	CAD	NFKB1	[activation;inhibition]
30537	APC	LAMA4	[activation]
30538	GAB1	SH2D1B	[activation]
30539	CYLD	PLK1	[inhibition]
30540	MRPL39	ICT1	[activation]
30541	SIMC1	YWHAZ	[activation]
30542	EFHC2	MAPK9	[activation]
30543	SMAD3	TGFB1I1	[activation]
30544	CAMK2B	GAPDH	[inhibition]
30545	TP53	SAE1	[activation]
30546	NCOR1	CDKN1A	[inhibition]
30547	FYB	SELH	[activation]
30548	BRK1	NLGN4X	[activation]
30549	EGFR	IQGAP1	[activation]
30550	CHN2	RACGAP1	[activation]
30551	DNAJC8	SRPK2	[activation]
30552	CDK5RAP2	SENP2	[activation]

30553	PRNP	APLP2	[activation]
30554	L1CAM	RDX	[activation]
30555	DLGAP2	NLGN4X	[activation]
30556	HCK	C2orf44	[activation]
30557	CDKN2C	NIF3L1	[inhibition]
30558	BCL3	NFKB1	[activation]
30559	PLCB3	KRIT1	[activation]
30560	TMEFF1	PTPRJ	[activation]
30561	GRB2	HTT	[activation]
30562	FAM195A	PIK3CD	[activation]
30563	CD247	GRB2	[activation]
30564	PLK1	PITPNM1	[activation]
30565	CDKN1A	CDC42	[activation;inhibition]
30566	GRB2	C1orf94	[activation]
30567	PTPN14	CTNNB1	[activation]
30568	JAG2	NOTCH3	[activation]
30569	GABPB1	FHL2	[activation]
30570	ARHGDIA	FASLG	[activation]
30571	APC	POM121	[inhibition]
30572	KIT	ZAP70	[activation]
30573	SRPK2	FOXN3	[activation]
30574	TLR2	TLR10	[activation]
30575	CBL	TGFBR2	[inhibition]
30576	GRIP1	EHMT2	[activation]
30577	LAMTOR5	GIT2	[activation]
30578	PRKCD	PLD2	[activation]
30579	NIT1	ZFYVE9	[activation]
30580	GADD45A	GADD45B	[activation]
30581	SRC	ARHGAP17	[activation]
30582	RAD21	ITGAM	[activation]
30583	TGFA	GORASP2	[activation]
30584	MED1	GATA1	[activation]
30585	TET2	OGT	[activation]
30586	CREB1	GTF2A1	[activation]
30587	SUMO1	HSPB1	[activation]
30588	MYL12A	SUV39H2	[activation]
30589	NPM1	ACACA	[activation;inhibition]
30590	PSMD11	INSIG2	[activation]
30591	MDM2	HMHA1	[activation]
30592	RALGDS	CD68	[activation]
30593	ITSN2	PSEN1	[activation]
30594	ASB6	FGA	[activation]
30595	CCDC120	CCDC57	[activation]
30596	SRF	MAPKAPK2	[activation]
30597	BCL6	ARNT2	[activation]
30598	RRAS	RASSF5	[activation]
30599	SMYD2	SNRPB2	[activation]
30600	MAPK14	IQGAP1	[activation]
30601	GCN1L1	PTP4A3	[activation]
30602	BID	BCL2L11	[activation]
30603	MIF	HSP90AA1	[activation]
30604	SBF1	TNFSF11	[activation]
30605	MAST1	PAXIP1	[activation]
30606	CD3E	EPS8L1	[activation]
30607	PIK3CA	IL24	[activation]
30608	CDH18	CDH9	[activation]
30609	PRKAA1	GOLGA2	[inhibition]
30610	EIF1AX	EIF6	[activation]
30611	PRKD2	PFDN1	[activation]
30612	MSRB2	RAD21	[activation]
30613	CDK5RAP1	CDK5R1	[activation]
30614	GRB2	MYO1A	[activation]
30615	CDH12	IGHG1	[activation]
30616	BCAR1	RAN	[activation]
30617	TSHR	SCRIB	[activation]
30618	RAN	XPO1	[activation]
30619	GNAQ	RIC8B	[activation]
30620	FKBP11	RELA	[activation]
30621	WNT4	APP	[activation]
30622	NXF1	MTHFSD	[activation]
30623	TRAF3	LTBR	[activation]
30624	SERBP1	CD81	[activation]
30625	RPA3	TFE3	[activation]
30626	STAU1	DDX39B	[activation]
30627	MYC	WDFY3	[activation]
30628	FHL2	MAPK1	[activation]

30629	PRKDC	EP300	[activation]
30630	NCOA1	PAGR1	[activation]
30631	RPL11	CD81	[activation]
30632	C12orf60	BMP4	[activation]
30633	MAPK6	METTL17	[activation]
30634	DOCK8	LRCH2	[activation]
30635	MAPK8	NFE2	[activation]
30636	BIRC2	EGFR	[activation]
30637	SFN	RACGAP1	[activation]
30638	SRC	YTHDC1	[activation]
30639	COL4A5	OSM	[activation]
30640	NLRC4	NLRP3	[inhibition]
30641	NOTCH3	CSNK2B	[activation]
30642	APOE	SCARB1	[activation]
30643	HYOU1	GABARAPL2	[activation]
30644	CCND1	HSPA8	[inhibition]
30645	SOX10	HHEX	[activation]
30646	WASF1	RAC1	[activation]
30647	HIST1H3A	ITGA4	[activation]
30648	CCDC124	EIF2AK2	[activation]
30649	DUSP19	MAP3K5	[inhibition]
30650	ACTR3	EIF1B	[activation]
30651	IGF1R	VAV3	[activation]
30652	BMPR2	PPP1CC	[activation]
30653	CD164	ACKR3	[activation]
30654	IL16	CDK1	[activation]
30655	RELA	NCL	[activation]
30656	NOTCH3	ANKRD28	[activation]
30657	SOCS3	ABL1	[activation;inhibition]
30658	ARF4	SDCBP2	[activation]
30659	SP3	ESR1	[activation]
30660	GNAI1	S1PR1	[activation]
30661	SMURF1	ARHGAP5	[inhibition]
30662	CAMK1D	CDC25A	[activation]
30663	XAGE1A	PAXIP1	[activation]
30664	CDC42	ACTR3	[activation]
30665	CELSR2	SRC	[activation]
30666	P2RY1	SLC9A3R2	[activation]
30667	MYOD1	SRF	[activation]
30668	PRKACA	MBP	[activation]
30669	SOS1	CD19	[activation]
30670	ENAH	PFN1	[activation]
30671	VCAM1	PRRC2C	[activation]
30672	PIK3R1	DAB2	[activation]
30673	MST1	STK11	[activation]
30674	PIAS4	HIF1A	[inhibition]
30675	CARD11	AKT1	[activation]
30676	UBASH3B	EGFR	[activation]
30677	HSPA8	PPP1R12B	[inhibition]
30678	EMP3	CREM	[inhibition]
30679	APP	YWHAZ	[activation]
30680	TNF	RPS18	[activation]
30681	RPS6KB1	SYK	[activation]
30682	APBB1	EVL	[activation]
30683	ANXA7	SETDB1	[activation]
30684	STAT6	IFNG	[inhibition]
30685	SMAD3	NR3C1	[activation]
30686	C19orf44	SPERT	[inhibition]
30687	BCL10	MAP2K7	[activation]
30688	FFAR1	GNAI1	[activation]
30689	ABCA1	ARHGEF11	[activation]
30690	ACTG1	SUMO4	[activation]
30691	F8	LRP1	[activation]
30692	TRAF2	CCNJL	[activation]
30693	HSF1	SUMO3	[activation]
30694	IKBIP	MYOG	[activation]
30695	RAD21	MCM4	[activation]
30696	DBP	EP300	[activation]
30697	DDX58	TRIM25	[inhibition]
30698	SMURF1	RNF141	[inhibition]
30699	OTX1	LCE2A	[activation]
30700	ACVR1	RASL12	[activation;inhibition]
30701	XPO1	ERBB2	[activation]
30702	MRPL23	SPP1	[activation]
30703	LATS1	RAC1	[activation]
30704	PTK2	BMX	[activation]

30705	YY1	RB1	[activation]
30706	TRAF6	PIN1	[activation]
30707	AVPR1A	VIP	[activation]
30708	UBE2I	SETD8	[activation]
30709	MYC	H3F3A	[activation]
30710	ROR2	HSP90AB1	[activation]
30711	SMARCAL1	RPA2	[activation]
30712	DLL1	MAGI2	[activation]
30713	PML	PAXIP1	[activation]
30714	MTOR	MAPKAP1	[activation]
30715	IL2RB	ECM1	[inhibition]
30716	PRKCI	ARHGAP17	[activation]
30717	EGFR	MCM3	[activation]
30718	SHC1	AR	[activation]
30719	SFN	MPRIP	[activation]
30720	SORCS3	NGF	[activation]
30721	PPARG	RAD54L2	[activation]
30722	MAP4K4	HSP90AA1	[activation]
30723	BHLHE40	EZH2	[activation]
30724	RELA	MAFK	[activation]
30725	PAK1	SMAD4	[activation]
30726	ESR1	HDAC4	[activation]
30727	CAMK2B	MVP	[activation]
30728	AKT2	SORBS2	[inhibition]
30729	FHL2	PTCH1	[activation]
30730	EPHA3	SRPK1	[activation]
30731	CRTC2	ATF6	[activation]
30732	IRF7	TICAM2	[activation]
30733	FOS	CEBPE	[activation]
30734	RCN1	MYC	[activation]
30735	TRAF6	MAP3K5	[activation]
30736	CTSD	INS	[activation]
30737	CTNNB1	TAX1BP3	[activation]
30738	RET	CASP3	[activation]
30739	PLEKHO2	APP	[activation]
30740	RAC1	OPHN1	[activation]
30741	ADRB2	EIF4A1	[activation]
30742	ETV4	EP300	[activation]
30743	RAB3GAP2	VAPB	[activation]
30744	STAU1	ARF6	[activation]
30745	ITGA4	ENO1	[activation]
30746	DDAH2	TNFSF11	[activation]
30747	PLCG2	PTPN11	[activation]
30748	RANGAP1	NXF1	[activation]
30749	RPA3	RAD50	[activation]
30750	HSPB3	LSM14B	[activation]
30751	S100A1	PLN	[inhibition]
30752	TLR3	IRAK2	[activation]
30753	CNKSR2	RAF1	[activation]
30754	YWHAQ	TSC2	[inhibition]
30755	HMGB1	GTF2A1	[activation]
30756	AURKB	PUM1	[activation]
30757	TPT1	MDM2	[activation]
30758	YWHAB	SIPA1L3	[activation]
30759	MECR	RARA	[activation]
30760	PRKAA1	CAMKK1	[activation]
30761	TIMP1	RECQL5	[activation]
30762	ADAMTS13	F8	[activation]
30763	SDF4	APP	[activation]
30764	RHEB	PLD1	[activation]
30765	TRIM28	VCAM1	[activation]
30766	MYO1C	E2F3	[activation]
30767	HNF4G	NCOA1	[activation]
30768	RASSF1	CUL1	[inhibition]
30769	RALA	RASSF5	[activation]
30770	CEP250	F7	[activation]
30771	TP53	LYZ	[activation]
30772	ITK	PLCG2	[activation]
30773	EP300	FBXL5	[activation]
30774	CCDC102B	FAM127C	[activation]
30775	HIST2H3A	MSL2	[activation]
30776	DDX58	PRKCA	[activation]
30777	PAXIP1	NCAPD2	[activation]
30778	ITGB4	MET	[activation]
30779	BTRC	SNAI1	[activation]
30780	EGFR	NSUN5	[activation]

30781	LCK	SH3BP2	[activation]
30782	AGTR2	GNAI3	[activation]
30783	SIRT3	CMYA5	[activation]
30784	CDK11A	CDKN2A	[activation;inhibition]
30785	SHC3	RET	[activation]
30786	UBE2H	CD99	[activation]
30787	EIF4A3	CAPZB	[activation]
30788	HIST2H2BF	YWHAZ	[activation]
30789	PXN	ITGA6	[activation]
30790	PRKCA	TRPC3	[activation]
30791	CNTN2	L1CAM	[activation]
30792	PYCARD	NLRP12	[inhibition]
30793	MDM2	PDCD5	[activation]
30794	MSTN	WFIKKN1	[activation]
30795	CD2	CD53	[activation]
30796	ATAD3A	TP53	[activation]
30797	DUSP16	MAPK1	[inhibition]
30798	RELA	CCAR2	[inhibition]
30799	MAPK14	ATM	[activation]
30800	TRAF2	TMC8	[activation]
30801	ZBTB17	CREBBP	[activation]
30802	SPERT	C14orf105	[inhibition]
30803	PTPN13	BRD7	[activation]
30804	NRXN2	MLLT4	[activation]
30805	OGDH	ESR2	[activation]
30806	MAPK3	FCGR2B	[activation]
30807	MAPT	MARK3	[activation]
30808	FASTKD5	FBXO6	[inhibition]
30809	GABARAP	TSR2	[activation]
30810	JUN	SMAD2	[activation]
30811	GRB7	KCTD6	[activation]
30812	TRRAP	TP53	[activation]
30813	EIF2S1	CSNK2A1	[activation]
30814	LTA	CLSPN	[activation]
30815	XPOT	RAN	[activation]
30816	NIF3L1	EPHB6	[activation]
30817	FYN	SPHK1	[activation]
30818	MMP14	MMP2	[activation]
30819	ARMC6	STK36	[activation]
30820	STAT3	BHLHE40	[activation]
30821	RFC4	NOTCH1	[activation]
30822	CLSPN	CASP7	[activation]
30823	ESR1	BCL3	[activation]
30824	C22orf46	SMAD2	[activation]
30825	PIK3C3	EXOC8	[activation]
30826	SMARCA4	SRF	[activation]
30827	TLR2	TGFB1	[activation]
30828	LYN	RGS16	[activation;inhibition]
30829	ULK2	POLA2	[activation]
30830	CASP3	TRAF3	[activation]
30831	PF4	IL8	[activation]
30832	FOXP3	PRR20C	[activation]
30833	GRB2	DDX5	[activation]
30834	GIT1	PAK3	[activation]
30835	HSPA8	SUMO3	[inhibition]
30836	ETS1	PCNX	[activation]
30837	TOP2A	PRKCB	[activation]
30838	ULK1	GABARAP	[inhibition]
30839	SSH2	CFL1	[activation]
30840	SUV39H2	KLHDC4	[activation]
30841	COL1A1	CAPN1	[activation]
30842	PEG10	ACVR2B	[activation]
30843	MST4	SRPK1	[activation]
30844	RASSF1	C8orf33	[inhibition]
30845	SUV39H1	CTNNB1	[activation]
30846	CASP6	IKBKG	[activation]
30847	RELA	PIAS1	[inhibition]
30848	PDE3B	PIK3R6	[activation]
30849	CACNA1C	PPM1A	[activation]
30850	FYN	SPN	[activation]
30851	ARHGDIB	UFM1	[activation]
30852	EGFR	JAK2	[activation]
30853	SLC25A6	TNFRSF10C	[activation]
30854	KCNQ1	CALM1	[activation]
30855	KPNA1	NFKB1	[activation]
30856	CTTN	PTPN1	[activation]

30857	STAT3	IL7R	[activation]
30858	VAPA	HLA-B	[activation]
30859	PLCG1	PKN2	[activation]
30860	CCL3	CEBPB	[activation]
30861	FLNB	PLCG1	[activation]
30862	XPO1	FADD	[activation]
30863	PBX1	TLX1	[activation]
30864	ERC1	IKBKB	[activation]
30865	SIRT1	KCNAB2	[activation]
30866	TRAF6	UBE2E1	[activation]
30867	SFPQ	RAC1	[activation]
30868	TP53	NME4	[activation]
30869	FAM65B	WNK1	[activation]
30870	CCND1	RB1	[inhibition]
30871	TP53	RPL12	[activation]
30872	CFLAR	RAF1	[activation;inhibition]
30873	FANCM	MYC	[activation]
30874	SF3B14	ITGA4	[activation]
30875	DDX39B	SHFM1	[activation]
30876	TUBB3	GRB2	[activation]
30877	PHGDH	GRB2	[activation]
30878	PPP2CA	NKD1	[inhibition]
30879	AKT2	SH3RF1	[inhibition]
30880	CACNA1A	ALDOA	[inhibition]
30881	DNAJB5	CACNA1A	[inhibition]
30882	CSNK1A1	CHRM3	[activation]
30883	PHF5A	VCAM1	[activation]
30884	RACGAP1	PLK1	[activation]
30885	CHD4	RPA1	[activation]
30886	MRFAP1L1	C3orf62	[activation]
30887	UBE2R2	ARHGDIA	[activation]
30888	APC	MAPRE2	[inhibition]
30889	ITGA4	PRDX2	[activation]
30890	ARRB2	CDK4	[inhibition]
30891	STX3	NAPB	[activation]
30892	SH2B3	GRB2	[activation]
30893	GRB2	SLX1A	[activation]
30894	CNPY4	ARL4D	[activation]
30895	NRAS	SMAD4	[activation]
30896	IL3RA	PLCG1	[activation]
30897	GADD45G	LUC7L2	[activation]
30898	BMP3	WFIKKN1	[activation]
30899	DYNC2LI1	SOD1	[inhibition]
30900	CXCR4	SOCS1	[activation]
30901	HOXC6	CHEK2	[activation]
30902	DYRK1A	YWHAB	[activation]
30903	CHD1L	PRKDC	[activation]
30904	KAT2B	CREBBP	[activation]
30905	CCL8	VCAN	[activation]
30906	MAP3K3	GNAI3	[activation;inhibition]
30907	CREBRF	CREB3	[activation]
30908	BCL2L1	BAG1	[activation]
30909	SERPINA10	F10	[inhibition]
30910	VAV2	ABI1	[activation]
30911	TFRC	ITGA4	[activation]
30912	TRAF2	KIFC3	[activation]
30913	MDM2	POT1	[activation]
30914	TP53	PIN1	[activation]
30915	RHEBL1	ACVR1	[activation;inhibition]
30916	CDKN2D	NR4A1	[inhibition]
30917	FAM13C	DVL3	[activation]
30918	SIX1	TLE1	[activation]
30919	TPT1	HLA-B	[activation]
30920	RPA1	PRIMPOL	[activation]
30921	PLD1	ACTB	[activation]
30922	PRKDC	HMGB1	[activation]
30923	ATG16L1	CASP3	[activation]
30924	EVL	WAS	[activation]
30925	RPA3	CAV1	[activation]
30926	ANXA1	VCAM1	[activation]
30927	PIK3CA	WDR5	[activation]
30928	RNF4	NFE2L2	[activation]
30929	NR3C1	STAT5B	[activation]
30930	PRKCE	IL32	[activation]
30931	RPA1	MCM6	[activation]
30932	MAP2K1	PSMD10	[activation]

30933	NR3C1	KDM5A	[activation]
30934	HSP90AB1	CAMK1G	[activation]
30935	TRAF6	TUBB	[activation]
30936	PTPN6	PILRA	[activation;inhibition]
30937	ARHGEF7	CBLB	[activation]
30938	ARF1	PICK1	[activation]
30939	YWHAG	RAPGEF6	[activation]
30940	SIRT1	TP73	[activation;inhibition]
30941	IGHA1	APOC1	[inhibition]
30942	PFN2	EVL	[activation]
30943	TUFM	OBSL1	[activation;inhibition]
30944	STK38	MOB1A	[activation]
30945	TGFA	RHBDF1	[activation]
30946	RASA1	CSK	[activation]
30947	MAFK	MARS	[activation]
30948	ACTN1	VCL	[activation]
30949	GRB2	AP4S1	[activation]
30950	KCTD12	RELA	[activation]
30951	SGTA	CDH15	[activation]
30952	MAGED4	PHYHIP	[activation]
30953	F13A1	SH3GL2	[activation]
30954	CSNK1G2	SMAD3	[inhibition]
30955	MBP	MAPKAPK5	[activation]
30956	RET	SHC1	[activation]
30957	ITGB2	CD14	[activation]
30958	TRAF6	PAFAH1B2	[activation]
30959	ADA	ADORA2A	[activation]
30960	KIT	BLK	[activation]
30961	AKT1	PLCG1	[activation]
30962	TNIK	PPP2R1A	[activation]
30963	PELI3	MAP3K7	[activation]
30964	CHD8	SUMO3	[inhibition]
30965	FCAR	FCGR1A	[activation]
30966	PPARG	CHD7	[activation]
30967	INPPL1	EFNA1	[activation]
30968	SDF4	SNCA	[activation]
30969	RAC1	PPP2R4	[activation]
30970	ACP6	ILK	[activation]
30971	NSD1	ATRX	[activation]
30972	EP300	DNA2	[activation]
30973	SUFU	SETDB1	[activation]
30974	RGS19	TGFBR1	[activation;inhibition]
30975	SRPK2	KCNN2	[activation]
30976	MDC1	SUPT6H	[activation]
30977	MAG	COL9A2	[activation]
30978	SH2B1	IGF1R	[activation]
30979	HDAC4	NFATC1	[activation]
30980	TBL3	SFN	[activation]
30981	MPL	YWHAQ	[inhibition]
30982	SNCA	PLD1	[activation]
30983	ZRANB1	PGAM5	[activation]
30984	CD3E	NCL	[activation]
30985	BRPF3	GRB2	[activation]
30986	FGFR2	FYN	[activation]
30987	ADAMTS12	A2M	[inhibition]
30988	BCL2L11	TRIM2	[activation]
30989	DCN	INS	[activation]
30990	EYA3	SIX4	[activation]
30991	TP53	HDAC2	[activation]
30992	GADD45G	CRIP2	[activation]
30993	CASP7	MEF2A	[activation]
30994	MCM2	CCL2	[activation]
30995	DIABLO	ELAVL1	[activation]
30996	C1orf174	SRPK2	[activation]
30997	PIK3R3	DARS	[activation]
30998	GH1	CAPZB	[activation]
30999	SNRNP70	ITGA4	[activation]
31000	PIAS1	CEBPA	[inhibition]
31001	HRAS	TLR2	[activation]
31002	NR3C1	MAFF	[activation]
31003	PRSS23	NCAPD3	[activation]
31004	TNFRSF25	TRAF2	[activation]
31005	RELA	NFKBIE	[activation]
31006	MYH11	SHC1	[activation]
31007	GABARAPL2	IRGQ	[activation]
31008	LYN	TMX1	[activation]

31009 SRPK1 SRPK3 [activation]
31010 TP53 PRKCA [activation]
31011 CREM CREBBP [activation]
31012 ROCK1 NCL [activation]
31013 CUL1 PTGS2 [activation]
31014 HNRNPA1 RPA2 [activation]
31015 TRIM16 E2F1 [activation]
31016 CTNNB1 CASP8 [activation]
31017 PPP1CC CCDC85C [inhibition]
31018 SUMO4 FOS [activation]
31019 ZAP70 RASA1 [activation]
31020 MCL1 CAV1 [activation]
31021 CSNK2A2 MYF5 [activation]
31022 PIK3CD TNPO2 [activation]
31023 MDM2 RAD50 [activation]
31024 MSH4 GPS2 [inhibition]
31025 VTI1B STX5 [activation]
31026 EED RAC1 [activation]
31027 CCND1 BTRC [inhibition]
31028 ANXA7 CPNE2 [activation]
31029 YWHAG TSC2 [activation]
31030 IRF3 CUL1 [inhibition]
31031 RAD21 PHACTR4 [activation]
31032 DCN TNF [activation]
31033 CDH1 PTPRF [inhibition]
31034 HLA-B PA2G4 [activation]
31035 FGR VDR [activation]
31036 HLA-B IGF2BP1 [activation]
31037 HIST1H2BL GABARAPL2 [activation]
31038 RHOA TGM2 [activation]
31039 C3 VHL [inhibition]
31040 PML TERT [activation]
31041 GDF9 CDKN1A [activation]
31042 RHOA ARHGAP8 [activation]
31043 PTK2 TNFRSF1A [activation]
31044 PTK2B GNA13 [activation]
31045 CDC5L EIF2B4 [activation]
31046 YWHAH PYHIN1 [activation]
31047 PLCB1 RGS12 [activation;inhibition]
31048 RARA NFE2L2 [activation]
31049 ZAP70 GRB2 [activation]
31050 THBS1 HRG [activation]
31051 ITGB1 GULP1 [activation]
31052 CDK1 LYN [activation]
31053 MBP PRKCI [activation]
31054 FBXO6 SMC2 [activation]
31055 ACTG1 ADRB2 [activation]
31056 SMAD5 HMGA2 [activation]
31057 KLHL42 FAM168A [activation]
31058 APOC3 ARFGAP1 [inhibition]
31059 SHC1 CD22 [activation]
31060 ACACA ARRB1 [activation;inhibition]
31061 MAP3K10 RAC1 [activation]
31062 INSR PIK3R3 [activation]
31063 MAP3K7 MAP3K5 [activation]
31064 VDAC1 CD4 [inhibition]
31065 PIK3CA AMBP [activation]
31066 MDM2 USP42 [activation]
31067 CDH6 CDH10 [activation]
31068 P4HB MAPT [activation]
31069 NBN MDC1 [activation]
31070 CEP44 CDC23 [activation]
31071 PECAM1 HCK [activation]
31072 PPP2R1A CHEK2 [activation]
31073 TP53 SP1 [activation]
31074 HSP90AA1 KDR [activation]
31075 PAX6 RGS3 [activation]
31076 RAB11B RPA1 [activation]
31077 PTPN6 SLAMF6 [activation;inhibition]
31078 ZNF420 TP53 [activation]
31079 HEMGN AXIN2 [activation]
31080 STK11 C9orf156 [activation]
31081 CDH5 CTNNA1 [activation]
31082 ULK2 DIXDC1 [activation]
31083 KCNH5 KCNH1 [activation]
31084 NOTCH1 LRBA [activation]

31085	ARMC7	C2orf88	[activation]
31086	ANG	MDM2	[activation]
31087	PPP3R2	CD14	[activation]
31088	DNAJC28	MAPK6	[activation;inhibition]
31089	RPS6KA6	RXRA	[inhibition]
31090	IGF2BP3	MDM2	[activation]
31091	RXRA	NR1I2	[inhibition]
31092	PCDH18	DAB1	[activation]
31093	MYF6	TCF3	[activation]
31094	YY1	SMAD3	[activation]
31095	SORCS2	NTF3	[activation]
31096	IKBKE	ACTG1	[activation]
31097	SOX2	ANAPC5	[activation]
31098	USP9X	SHC1	[activation]
31099	BLVRA	MAPK1	[activation]
31100	MIF	UBC	[activation]
31101	HSPA1L	CFTR	[inhibition]
31102	RAG2	KPNA1	[activation]
31103	MARK3	MAP2	[activation]
31104	ARRB2	HSPA1L	[inhibition]
31105	DPYSL2	DPYSL5	[activation]
31106	CD4	HSP90AA1	[activation]
31107	SIX1	VTN	[activation]
31108	FBXW11	IFNAR1	[inhibition]
31109	PLAT	LAMA5	[activation;inhibition]
31110	PARD3	CSK	[activation]
31111	FGFR1	MMP2	[activation]
31112	CDK2	GSTK1	[activation]
31113	VCAM1	DIMT1	[activation]
31114	CYLD	SPATA2L	[inhibition]
31115	COL4A5	MMP9	[activation;inhibition]
31116	MAPK1	ALK	[activation]
31117	PSEN1	BCL2	[inhibition]
31118	PRNP	MAPT	[activation]
31119	CCND1	CARD11	[activation]
31120	GRB2	BRCA2	[activation]
31121	ARHGEF7	ITCH	[activation]
31122	MAPT	CDK1	[activation]
31123	SNX33	WAS	[activation]
31124	NDRG1	PRKACA	[activation]
31125	GRB2	CORO1C	[activation]
31126	RPS6KA1	NR4A2	[activation]
31127	CDC14A	TP53	[activation]
31128	TGFBR2	TCTEX1D4	[activation]
31129	UBXN7	BRWD3	[activation]
31130	PRLR	VAV2	[inhibition]
31131	APP	RPS6KA6	[activation]
31132	RASAL2	CSNK1E	[activation]
31133	MOCS2	STK11	[activation]
31134	NCOA3	ESRRA	[activation]
31135	LAT	GRAP2	[activation]
31136	TBL3	WHSC1	[activation]
31137	LCP2	SH3GL2	[activation]
31138	CRK	LIG3	[activation]
31139	RPS6KA3	NR4A1	[activation]
31140	RRAS	NCK1	[activation]
31141	RPTOR	LAMTOR4	[activation;inhibition]
31142	COPS6	CASP6	[activation]
31143	DNM2	ERBB2	[activation]
31144	HIST1H3A	SFMBT1	[activation]
31145	HSPB1	PELO	[activation]
31146	YWHAZ	CALM1	[activation]
31147	NTRK1	PTPN11	[activation]
31148	MYL12A	CDC42BPA	[activation]
31149	PRKCB	KCNA5	[activation]
31150	JUNB	PKIA	[activation]
31151	HNRNPA1	GTF2A1	[activation]
31152	TP53	PAGR1	[activation]
31153	HSPA8	CBL	[inhibition]
31154	PRIMPOL	RPA2	[activation]
31155	MDM2	ALDH16A1	[activation]
31156	LIMK1	HUNK	[activation;inhibition]
31157	MAST2	SLC9A3	[activation]
31158	EPOR	GAB2	[activation]
31159	F10	SERPINA5	[inhibition]
31160	LAMTOR4	LAMTOR5	[activation]

31161	USHBP1	GPSM1	[inhibition]
31162	STAT3	STAT1	[activation]
31163	CFTR	HSPA6	[activation]
31164	DCC	BARD1	[activation]
31165	GFAP	MOS	[inhibition]
31166	CALM1	PTPRA	[activation]
31167	RCC1	TP53	[activation]
31168	HTRA2	MAPK14	[activation]
31169	LIMS1	RSU1	[activation]
31170	JUN	VAV1	[activation]
31171	F2	C1QBP	[activation]
31172	NFYB	HDAC1	[activation]
31173	PTPRT	STAT3	[activation]
31174	ICAM1	ALYREF	[activation]
31175	TRIM50	UBE2N	[activation]
31176	CCL20	TRAF6	[activation]
31177	ARF1	ARFIP1	[activation]
31178	TGM2	GRN	[activation]
31179	RAB13	TGFBR1	[activation]
31180	GABRD	NOTCH2NL	[activation]
31181	RALA	PIH1D2	[activation]
31182	CTTN	ARPC3	[activation]
31183	PTPN22	ZAP70	[activation]
31184	FLT4	PTPN11	[activation]
31185	DUSP4	APP	[inhibition]
31186	CCNA1	SRC	[activation]
31187	MARK3	PPM1B	[activation]
31188	TRAF1	TP53	[activation]
31189	ADAP2	PRNP	[activation]
31190	MDM2	MYD88	[activation;inhibition]
31191	CDC7	DBF4	[activation]
31192	BIRC5	HIST2H3A	[activation]
31193	TAF1B	TAF1A	[activation]
31194	PPP1CA	GPR12	[activation]
31195	DBF4	CHEK2	[activation]
31196	FCRL3	ZAP70	[activation]
31197	WASF1	ACTG1	[activation]
31198	HSP90AA1	WNK4	[activation]
31199	HLA-C	HLA-B	[activation]
31200	BMPR1B	DCAF6	[activation;inhibition]
31201	HNRNPA1	AURKA	[activation]
31202	LRRK2	DNAJA1	[activation]
31203	SUMO3	JUP	[inhibition]
31204	SOX9	KPNB1	[activation]
31205	PRRC2C	AURKA	[activation]
31206	TIMELESS	PRIMPOL	[activation]
31207	SNIP1	CDK6	[inhibition]
31208	RIPK2	CALM1	[activation]
31209	PIK3CG	ZNF282	[activation]
31210	PPP1CA	CCNA1	[activation;inhibition]
31211	E2F1	MCPH1	[activation]
31212	MAPK1	HLA-B	[activation]
31213	RASA1	GRB2	[activation]
31214	WASF2	YWHAQ	[activation]
31215	STAB2	MAPK1	[activation]
31216	MAX	EP400	[activation]
31217	PIK3CA	FTL	[activation]
31218	POLR2L	CHEK2	[activation]
31219	FAM213A	APP	[activation]
31220	TAGLN2	MAPK13	[activation]
31221	SMURF1	AVEN	[inhibition]
31222	STK4	HSPA5	[activation]
31223	IKBKE	RAB21	[inhibition]
31224	TPD52L3	AGTRAP	[activation]
31225	CAMK2G	MCM7	[activation]
31226	PCBP1	MAP4K3	[activation]
31227	BCL2	BAD	[inhibition]
31228	PRKCQ	AKT1	[activation]
31229	GABARAPL1	NUP214	[activation]
31230	UBE2E2	MID1	[activation]
31231	TP53	XPC	[activation]
31232	INSC	GPSM2	[inhibition]
31233	SMAD2	RAB34	[activation]
31234	PIN1	LEPR	[activation]
31235	SMARCA4	SMAD3	[activation]
31236	GRIA4	PRKCA	[activation]

31237	TRAF6	ITCH	[activation]
31238	ATM	ATF2	[activation]
31239	CASP6	NEDD4	[inhibition]
31240	ANXA1	TGM2	[activation]
31241	PTPN6	FAS	[inhibition]
31242	ATF3	FHL2	[activation]
31243	PPP1CC	TP53	[activation]
31244	ADRA1B	IGHG1	[activation]
31245	TIAL1	RPA3	[activation]
31246	PRDX5	MDM2	[activation]
31247	STMN1	CDKN1B	[activation]
31248	EIF1B	HYPK	[inhibition]
31249	FANCI	HELQ	[activation]
31250	NF1	CDC5L	[activation]
31251	ACTB	PHACTR4	[activation]
31252	HIST3H3	HNRNPA1	[activation]
31253	NEURL4	DBNL	[inhibition]
31254	PRKAA1	CTBP1	[inhibition]
31255	ATM	CXXC5	[activation]
31256	FCGR3A	PTPRC	[activation]
31257	UBE2E3	RNF114	[activation]
31258	NUDT5	HLA-B	[activation]
31259	SMAD5	PNKP	[activation;inhibition]
31260	TNFSF13	PIK3CA	[activation]
31261	ERBB4	MDM2	[activation]
31262	YWHAZ	ACTG1	[activation]
31263	GADD45G	AFAP1L1	[activation]
31264	ENG	TCTEX1D4	[inhibition]
31265	NR4A1	ROBO2	[activation]
31266	RUNX1	KMT2A	[activation]
31267	MAD2L2	POLD3	[activation]
31268	LIPC	ADHFE1	[activation]
31269	EIF2S1	CASP3	[activation]
31270	GNAO1	HTT	[activation]
31271	NUDCD2	RTN4RL2	[inhibition]
31272	PIP5K1A	PIP5KL1	[activation]
31273	PPM1B	GSN	[activation]
31274	HMGB1	MNT	[inhibition]
31275	FRK	CSK	[activation]
31276	SLC30A2	CCL4	[activation]
31277	PPP2R2B	CDC42	[activation]
31278	BIRC2	TRADD	[activation]
31279	ATP6V0B	YWHAG	[inhibition]
31280	ITGB2	CYTH2	[activation]
31281	ACKR4	CCL21	[activation]
31282	MMP2	THBS2	[activation]
31283	GRB2	MAPK12	[activation]
31284	PRKAA1	THAP1	[inhibition]
31285	NTRK1	HSP90AB1	[activation]
31286	CSNK1G2	C2orf44	[inhibition]
31287	NTM	NEGR1	[inhibition]
31288	ENO1	ATF2	[activation]
31289	SPG20	ZFYVE9	[activation]
31290	MMP1	CCL13	[activation]
31291	FOXH1	GRB2	[inhibition]
31292	DDX21	SRPK3	[activation]
31293	EZR	CTNNB1	[activation]
31294	HLA-B	KIR3DL1	[activation]
31295	GLI3	SMAD4	[activation]
31296	GAB1	PTPRG	[activation]
31297	SCTR	CALM1	[activation]
31298	RPS6	GABARAP	[activation]
31299	ITGB1BP1	LRP1	[activation]
31300	SUMO1	MEF2A	[activation]
31301	LTK	SHC1	[activation]
31302	FANCD2	CEBPD	[activation]
31303	LIN7B	ASIC3	[activation]
31304	AMOT	MAPK3	[activation]
31305	PIK3CG	PIK3R5	[activation]
31306	EGFR	EPN1	[activation]
31307	STX7	STX6	[activation]
31308	ARRB2	GSN	[activation]
31309	GAP43	PRKCD	[activation]
31310	RAD21	STX10	[activation]
31311	PALB2	DIRAS3	[activation]
31312	EGFR	WASL	[activation]

31313	EP300	STAT2	[activation]
31314	DRP2	DAG1	[activation]
31315	SP1	DDX5	[activation]
31316	GNB5	RGS6	[activation;inhibition]
31317	CD2	CD2BP2	[activation]
31318	CMSS1	EGFR	[activation]
31319	FOXM1	CCNE1	[activation]
31320	YME1L1	MYOG	[activation]
31321	TP53	ERCC6	[activation]
31322	GRB2	ITGA6	[activation]
31323	SP1	JUN	[activation]
31324	NUDCD3	KLHL32	[activation]
31325	HIST3H3	TAF1B	[activation]
31326	PPARA	PRMT2	[inhibition]
31327	SRPK2	C17orf85	[activation]
31328	AP2M1	ACACA	[activation;inhibition]
31329	IL1B	FYN	[activation]
31330	PLEC	RAPGEF2	[activation]
31331	MRFAP1L1	PIH1D2	[activation]
31332	PPM1B	RASGRP1	[inhibition]
31333	PIK3R1	GAB3	[activation]
31334	SHCBP1	SFN	[activation]
31335	ITCH	DVL1	[activation]
31336	PIAS1	SMAD4	[inhibition]
31337	MAP4K2	MAP2K4	[activation]
31338	YWHAE	KCNK15	[activation]
31339	CDC5L	DDX39B	[activation]
31340	STK11	APP	[activation]
31341	INADL	ARHGAP17	[activation]
31342	EGFR	FANCC	[activation]
31343	MMP13	F12	[activation]
31344	ARF1	ITGA4	[activation]
31345	TP53	SMAD1	[activation]
31346	NUDC	KLHL6	[activation]
31347	BTC	MMP9	[activation]
31348	CDC42	ARHGAP29	[activation]
31349	MYC	ROBO2	[activation]
31350	RXRA	HDAC4	[inhibition]
31351	TGFB1	THBS1	[activation]
31352	GRB2	MTA3	[activation]
31353	NRAS	RACGAP1	[activation]
31354	EGFR	NELL1	[activation]
31355	EDNRA	EDN1	[activation]
31356	ADAM17	SH3D19	[activation]
31357	DLX6	HSP90AA1	[activation]
31358	DAB1	PRR20E	[activation]
31359	TSPAN5	ADAM10	[activation]
31360	AGAP1	GUCY1A3	[activation]
31361	SERPINB4	BTRC	[inhibition]
31362	CREBBP	SPI1	[activation]
31363	DVL2	TP53	[activation]
31364	UBE2J2	XIAP	[inhibition]
31365	PPP3CA	TP53	[activation]
31366	TFCP2	PIK3CB	[activation]
31367	ARRB2	MAP2K1	[activation]
31368	UBTF	ESR1	[activation]
31369	ABL2	KIT	[activation]
31370	PLK1	IKBKB	[activation]
31371	TNFRSF1A	JAK2	[activation]
31372	CDC27	AURKB	[activation]
31373	CCNE1	NFYB	[activation]
31374	YWHAZ	PARD6A	[activation]
31375	NOTCH2NL	CCDC93	[activation]
31376	CCDC8	GNAI2	[activation;inhibition]
31377	ADAM17	PXN	[activation]
31378	BCL2L2	BID	[activation]
31379	SRPK1	C15orf39	[activation]
31380	SMURF1	PAK1IP1	[inhibition]
31381	UBE2W	XIAP	[activation]
31382	MDM2	SNAI2	[activation]
31383	UHMK1	STMN1	[activation]
31384	MYC	KIAA2026	[activation]
31385	VAV1	EGFR	[activation]
31386	VEPH1	TGFBR1	[activation]
31387	MAPK1	NR4A1	[inhibition]
31388	SLX4IP	PLK1	[activation]

31389	SRSF10	YWHAQ	[activation]
31390	TRAF2	JUN	[activation]
31391	YWHAG	ARHGEF6	[activation]
31392	SRC	RGS16	[activation;inhibition]
31393	TGFBR1	NAT8	[activation]
31394	INADL	SAV1	[activation]
31395	ILK	MARS	[activation]
31396	HEATR2	CASP4	[activation]
31397	RPL22L1	RAD21	[activation]
31398	ATF5	GIT2	[activation]
31399	TGFBR1	GNA13	[activation]
31400	UBC	IL18	[activation]
31401	ELK4	FOS	[activation]
31402	SP1	SMAD3	[activation]
31403	PRKAA2	MLF2	[activation]
31404	METTL21C	MAD1L1	[activation]
31405	PLD1	PLCG1	[activation]
31406	YAP1	RUNX1	[activation]
31407	PVRL4	PVRL1	[activation]
31408	PPP1CA	TSC2	[activation;inhibition]
31409	MAP3K14	CFLAR	[inhibition]
31410	STAT2	STAT3	[activation]
31411	SMAD4	RASL12	[activation]
31412	NFKB1	C1orf52	[activation]
31413	FANCA	CTNNB1	[activation]
31414	NUDC	FBXO24	[activation]
31415	TIMELESS	CHEK1	[activation;inhibition]
31416	MAPT	PPP2R4	[activation]
31417	CTNND1	KCTD6	[activation]
31418	EIF2AK2	NOC3L	[activation]
31419	MEIS2	ARNT2	[activation]
31420	CDC14B	TP53	[activation]
31421	SGOL2	PPM1A	[activation]
31422	PLG	SERPINE1	[inhibition]
31423	DDIT3	TRIB3	[inhibition]
31424	STK11	NAT2	[activation]
31425	FBXW5	NLK	[inhibition]
31426	ARFGAP1	ASB10	[activation]
31427	APP	PFKFB1	[activation]
31428	NR5A2	SRC	[activation]
31429	COG4	APC	[inhibition]
31430	G3BP2	RPL22L1	[activation]
31431	CNKSR1	RHOC	[activation]
31432	SIRT1	SETD7	[activation]
31433	RICTOR	EHMT2	[activation]
31434	ONECUT1	KAT2B	[activation]
31435	FBXO6	SMCHD1	[inhibition]
31436	UBASH3B	CRK	[activation]
31437	AKT2	APOB	[activation]
31438	ASB7	ATF4	[activation]
31439	SHANK3	GRB2	[activation]
31440	RELN	VLDLR	[inhibition]
31441	CSNK1E	TAOK1	[activation;inhibition]
31442	KMT2A	PAX5	[activation]
31443	LDLR	PF4	[activation]
31444	RASGRP3	PIK3CA	[activation]
31445	ANAPC15	CDC26	[activation]
31446	IL17RB	DAZAP2	[activation]
31447	GNAQ	MRGPRX1	[activation]
31448	GNAI2	ADRA2A	[activation]
31449	ZFYVE21	NOTCH2NL	[activation]
31450	FGF2	RPS6KA3	[activation]
31451	TNK2	GRB2	[activation]
31452	RPA3	RAB8A	[activation]
31453	IRF3	RBL1	[inhibition]
31454	JAK1	IGF1R	[activation]
31455	RELA	TERT	[activation]
31456	SPHK2	LYN	[activation]
31457	RAB43	APP	[activation]
31458	CDC7	GPT	[activation]
31459	PSEN2	RNF32	[activation]
31460	CEBPD	CEBPG	[activation]
31461	LGR4	TFRC	[activation]
31462	FZD4	NDP	[activation]
31463	ZFYVE9	RHO	[activation]
31464	PEBP1	SMYD2	[inhibition]

31465	PTPN11	DDR1	[activation]
31466	WWC3	CDK5RAP3	[activation]
31467	EIF1B	EIF2S3	[activation]
31468	EEF2K	MAPKAPK2	[activation]
31469	HSP90AB1	MAP3K15	[activation]
31470	CDC23	CCDC24	[activation]
31471	SRF	MYOG	[activation]
31472	CHRNA4	CHRNB4	[activation]
31473	ALG2	PTK2B	[activation]
31474	MAG	RTN4R	[activation]
31475	MAPK8	CBL	[activation]
31476	USP12	MMP2	[inhibition]
31477	NCAN	L1CAM	[activation]
31478	TBXA2R	GPRASP1	[inhibition]
31479	JUP	ESR1	[activation]
31480	HLA-C	LILRB1	[activation]
31481	FOXP4	FOXP1	[activation;inhibition]
31482	SIRT2	KAT2A	[activation]
31483	LYRM4	PVRL2	[activation]
31484	TP53	NOTCH4	[activation]
31485	WDR5B	ESR1	[activation]
31486	ATF2	SFXN1	[activation]
31487	PIK3R3	QARS	[activation]
31488	RAD17	CDH1	[activation]
31489	WHSC1	BAZ1B	[activation]
31490	IRF2	ATG7	[activation]
31491	CTSK	KNG1	[activation]
31492	MMP1	MMP7	[activation]
31493	GREB1	TGFB1	[activation]
31494	SPOCK3	MMP3	[activation]
31495	G6B	ABL2	[activation]
31496	PPP1CC	BCL2	[inhibition]
31497	E2F3	SHOC2	[activation]
31498	FTL	MYOG	[activation]
31499	HSPB1	LIG1	[activation]
31500	DNM1L	VIM	[activation]
31501	MAPK9	RB1	[activation]
31502	YWHAZ	TFRC	[activation]
31503	PDCD11	MYC	[activation]
31504	USF2	NFYA	[activation]
31505	GREB1	CASP8	[activation]
31506	MAP2K7	SNW1	[activation]
31507	PPP1R21	PPP1CA	[activation;inhibition]
31508	TRAF3	TNFRSF8	[activation]
31509	FYN	MAG	[activation]
31510	GARS	MAPK8	[activation]
31511	VTN	HGF	[inhibition]
31512	TBC1D17	CDKN2D	[inhibition]
31513	FOXO3	USP7	[inhibition]
31514	MDH1	EGFR	[activation]
31515	VCAM1	PCBP1	[activation]
31516	CCAR2	MYC	[inhibition]
31517	PTPRC	TYK2	[activation]
31518	LPL	PTPN4	[activation]
31519	PRKCZ	MARCKS	[activation]
31520	RPL38	DCC	[activation]
31521	GABARAPL2	C18orf32	[activation]
31522	YWHAB	ABL1	[activation]
31523	SPERT	CCDC146	[inhibition]
31524	CLPP	HLA-B	[activation]
31525	GATA1	TRIM29	[activation]
31526	CDK1	IL3RA	[activation]
31527	PARD3B	SFN	[activation]
31528	ZRANB1	MAP4K4	[activation]
31529	COMMD2	NFKB1	[activation]
31530	PRKG1	TBXA2R	[inhibition]
31531	HMGB2	POU5F1	[activation]
31532	ARPC4	MOV10	[activation]
31533	MRPL53	APP	[activation]
31534	ARMC8	CTNNB1	[activation]
31535	CDC37	SGK1	[activation]
31536	LCK	WAS	[activation]
31537	KCNJ1	PRKCD	[activation]
31538	LRCH3	LRCH2	[activation]
31539	NBN	HSPA4	[activation]
31540	GPRASP1	CHRM5	[activation]

31541	FOXO1	GSK3B	[activation]
31542	TOP3B	CHD8	[activation]
31543	MAPK3	RXRA	[inhibition]
31544	MCM6	IL36RN	[activation]
31545	TLE1	BCL2L1	[activation]
31546	GNAI3	C5AR1	[activation]
31547	VDR	NRIP1	[activation]
31548	MDM2	SFXN3	[activation]
31549	ID4	CRK	[activation]
31550	PAK1	ARHGEF2	[activation]
31551	GINS2	CHEK2	[activation]
31552	MAP3K19	ZDHHC17	[activation]
31553	TAB1	HSPA8	[inhibition]
31554	NAA15	HLA-B	[activation]
31555	CASK	WNT7B	[activation]
31556	HMGXB4	NR1H2	[activation]
31557	SIKE1	MST4	[activation]
31558	MAPKAP1	MYC	[activation]
31559	TRIM49	HSP90AB1	[activation]
31560	STK4	CDC37	[activation]
31561	GNA13	MCF2	[activation]
31562	MOV10	ANAPC1	[activation]
31563	PDGFRB	PTPN22	[activation]
31564	SUMO1	DYRK1A	[activation]
31565	PNPLA3	AKT2	[activation]
31566	AR	SLC25A4	[inhibition]
31567	CCDC158	C1orf216	[activation]
31568	SDCBP2	NRXN1	[activation;inhibition]
31569	CAPNS1	TRAF5	[activation]
31570	CARM1	STAT5A	[activation]
31571	NTRK3	MAPK3	[activation]
31572	CDC37	ALS2	[activation]
31573	SMURF1	TRIB3	[inhibition]
31574	SH2B2	AKT1	[activation]
31575	MAP3K5	RAF1	[activation]
31576	KNG1	CAPN1	[activation]
31577	CEBPE	PIAS1	[inhibition]
31578	MAPK14	TOLLIP	[activation]
31579	ADRB2	GRK6	[activation]
31580	STAT1	PTK2	[activation]
31581	TP53	ERBB4	[activation]
31582	CDX4	CKS1B	[inhibition]
31583	SGK1	MAGEA1	[activation]
31584	PRKCD	MBP	[activation]
31585	TBC1D4	CDKN1A	[activation;inhibition]
31586	NXF1	TATDN2	[activation]
31587	MDM2	IRF2	[activation]
31588	RBL1	NR4A1	[inhibition]
31589	CEP55	CCBE1	[activation]
31590	WAS	PACSIN1	[activation]
31591	CSN1S1	STK4	[activation]
31592	EHMT2	ESCO1	[activation]
31593	NFYA	CCNA2	[activation]
31594	YWHAH	AKT1S1	[activation;inhibition]
31595	HLA-A	HLA-C	[activation]
31596	ABL1	AR	[activation]
31597	SHC1	PPP2R5A	[activation]
31598	TNFRSF11A	MAP3K7	[activation]
31599	F2RL1	TMED10	[activation]
31600	STAB2	TMSB4X	[activation]
31601	GABBR2	KCTD12	[activation]
31602	CTBP1	CTNNB1	[activation]
31603	SERPINB6	F2	[inhibition]
31604	ARRB2	HSPA6	[inhibition]
31605	EGFR	LRRC40	[activation]
31606	GNAS	AXIN1	[activation]
31607	TRAF6	EPPK1	[activation]
31608	MAPK6	MYBL2	[inhibition]
31609	KCNB2	GRB2	[activation]
31610	ARMC8	EGFR	[activation]
31611	FANCD2	CDC5L	[activation]
31612	EGFR	ACTR2	[activation]
31613	STAT5B	EP300	[activation]
31614	BMPR1B	RRAS2	[activation]
31615	ADRB2	TMX1	[activation]
31616	ACVR1	ACVR2A	[activation]

31617	SRPK1	FGF12	[activation]
31618	RPA2	TFRC	[activation]
31619	CDK18	YWHAH	[activation]
31620	BMPR1A	HSP90AB1	[activation]
31621	CAV1	RCVRN	[activation]
31622	TGM2	ACTB	[activation]
31623	SYNJ2BP	ACVR2B	[activation]
31624	CRH	CRHR2	[activation]
31625	TERF1	GIT1	[activation]
31626	VIM	LRRK2	[activation]
31627	CDKN2C	UNC119	[inhibition]
31628	HDAC1	MEF2A	[activation]
31629	CARM1	NRIP1	[activation]
31630	CRKL	VCAM1	[activation]
31631	TP53	HSPB1	[activation]
31632	ATF3	JUNB	[activation]
31633	RANBP2	NUP153	[activation]
31634	SMAD3	PRTN3	[inhibition]
31635	KMT2D	E2F5	[activation]
31636	YWHAE	GLI1	[inhibition]
31637	CFHR4	CDC42	[activation]
31638	SMAD2	AKT1	[activation]
31639	SIVA1	TNFRSF4	[activation]
31640	NRAS	GRB10	[activation]
31641	RPL17	ICAM1	[activation]
31642	CDC16	PAXIP1	[activation]
31643	RBBP6	SPP1	[activation]
31644	IL5RA	JAK2	[activation]
31645	FGR	MET	[activation]
31646	NR1H2	SMPD1	[activation]
31647	SGK1	TUFM	[activation;inhibition]
31648	KNG1	ELANE	[activation]
31649	ESR2	NFKB2	[activation]
31650	PIAS1	SETX	[inhibition]
31651	SMAD1	RAN	[activation]
31652	LAMA5	FBLN2	[activation;inhibition]
31653	SOX9	HERC1	[activation]
31654	GNA13	AKAP3	[activation]
31655	RASSF1	KCNE3	[inhibition]
31656	SMAD4	ESR2	[activation]
31657	KPNA4	HSF1	[activation]
31658	APC	CREBBP	[activation]
31659	FYN	ASB16	[activation]
31660	H2AFX	P4HA2	[activation]
31661	TSSK3	NMES1	[activation]
31662	GEM	NDUFAF3	[activation]
31663	SDC4	CXCR4	[activation]
31664	RGS19	OPRL1	[activation;inhibition]
31665	CDKN1A	CXCL13	[activation]
31666	SLA	CBL	[activation]
31667	SIRT1	CCNB1	[activation]
31668	CACNB4	REM1	[activation]
31669	PTK2B	DNM1	[activation]
31670	TRDN	CASQ2	[activation]
31671	BTK	SH2B2	[activation]
31672	SNAP29	CD7	[activation]
31673	ANXA7	DMPK	[activation]
31674	CDAN1	GADD45A	[activation]
31675	CAV1	PROCR	[activation]
31676	YWHAH	PIK3CA	[activation]
31677	TRAF6	CYB5A	[activation]
31678	CDK6	EZH2	[activation]
31679	RBPJ	SIN3A	[inhibition]
31680	RRBP1	MLH1	[activation]
31681	ARPC4	ARPC1B	[activation]
31682	SRSF10	GRB2	[activation]
31683	CCDC146	NUP62	[activation]
31684	PPARG	NCOA3	[activation]
31685	SLA	SYK	[activation]
31686	HLA-B	LILRB2	[activation]
31687	PRKACA	PRKACG	[activation]
31688	APC	NCKAP5L	[inhibition]
31689	SELPLG	PIK3R1	[activation]
31690	CAMK2D	DNAL4	[activation]
31691	NBN	EP300	[activation]
31692	SRPK2	ZRSR2	[activation]

31693	MCCC2	STK4	[activation]
31694	GRB2	ABI3	[activation]
31695	IKBKB	TNIP2	[activation]
31696	NOTCH1	APBA1	[activation]
31697	TAB3	TAB1	[inhibition]
31698	IL1A	CAPN1	[activation]
31699	RRAS2	CCDC8	[activation]
31700	RAC1	BRK1	[activation]
31701	FBXO6	ADAM17	[activation]
31702	YWHAH	NCOA1	[activation]
31703	A2M	FBXO6	[inhibition]
31704	EXOC3	MYC	[activation]
31705	SKP2	CEBPA	[inhibition]
31706	TRIM24	AR	[activation]
31707	FANCF	FANCG	[activation]
31708	BCL2L1	PSEN1	[activation]
31709	TTI1	RPTOR	[activation;inhibition]
31710	EIF4E	PRKCB	[inhibition]
31711	ESR1	ACTB	[activation]
31712	PSEN1	CFL1	[activation]
31713	TRAF6	MAP3K14	[activation]
31714	CBL	FRS3	[activation]
31715	HLA-C	GSTM3	[activation]
31716	GRB2	GFAP	[activation]
31717	CDC37	TNFRSF14	[activation]
31718	STK32C	HSP90AA1	[activation]
31719	PLCG1	PAK1	[activation]
31720	SMAD9	DKK1	[inhibition]
31721	CMTM5	SPATA8	[activation]
31722	TEAD1	INSR	[activation]
31723	LYPLA2	ITGA4	[activation]
31724	SRF	FHL5	[activation]
31725	EFS	FYN	[activation]
31726	E2F3	TFRC	[activation]
31727	CDKN2A	CCDC180	[activation;inhibition]
31728	MAP3K3	GNB1	[activation]
31729	WEE1	SFN	[activation]
31730	ACTR3	JUND	[activation]
31731	STK11	LEF1	[activation]
31732	CATSPER1	CHRD	[inhibition]
31733	RPS6KB1	MAPK9	[activation]
31734	TRAF1	TNFRSF12A	[activation]
31735	CPNE1	STK3	[activation]
31736	NCOA3	PRMT1	[activation]
31737	BTK	TNFRSF10A	[activation]
31738	XIRP2	RAD21	[activation]
31739	TBXA2R	GNB2L1	[inhibition]
31740	MYO5A	SMAD2	[activation]
31741	CDK17	APP	[activation]
31742	GRB14	IGF1R	[activation]
31743	IGF1R	CSK	[activation]
31744	MIF	BNIPL	[activation]
31745	CHUK	PAX8	[activation]
31746	BCL3	GSK3B	[activation]
31747	CDK5RAP2	CDK5	[activation]
31748	PIP5K1C	IQGAP1	[activation]
31749	H2AFX	DDX21	[activation]
31750	LRRK2	CDK2	[activation]
31751	AR	SHC4	[activation]
31752	HSPB1	PHLDA1	[activation]
31753	RAB8A	TFRC	[activation]
31754	TGFBR1	CAV1	[activation]
31755	EGLN3	ATF4	[activation]
31756	YES1	CDKN1B	[inhibition]
31757	NUP153	OBSL1	[activation]
31758	EMP3	EDA	[activation]
31759	HSPA2	METTL21A	[activation]
31760	BLNK	TLN1	[activation]
31761	HIF1A	ATM	[activation]
31762	HMOX1	ASL	[activation]
31763	TP53	HMGB2	[activation]
31764	TSHB	CGA	[activation]
31765	GRB2	OTUD5	[activation]
31766	BIRC2	TNFSF12	[activation]
31767	PRKAA1	FSBP	[inhibition]
31768	POLR2G	NTRK2	[activation]

31769	VASP	ABI1	[activation]
31770	HIST2H2BF	ICAM1	[activation]
31771	CREBBP	SH3GL1	[activation]
31772	ABL1	NEK8	[activation]
31773	DDX55	APP	[activation]
31774	STAM2	LCP2	[activation]
31775	ESRRG	ESRRA	[activation]
31776	HSPB1	DNAJC21	[inhibition]
31777	SGMS1	STK4	[activation]
31778	CRCT1	RGS20	[activation;inhibition]
31779	BOC	TULP3	[activation]
31780	MYOD1	SUV39H1	[activation]
31781	TENC1	CSNK1E	[activation]
31782	TNF	TNIP1	[activation]
31783	USP34	ATM	[activation]
31784	SRC	AFAP1L2	[activation]
31785	C5orf24	PYHIN1	[activation]
31786	ZNHIT3	PITX2	[activation]
31787	TBX21	SP1	[activation]
31788	GJA5	RIMS3	[activation]
31789	PPP2R5A	CTLA4	[activation]
31790	PTPRC	INSR	[activation]
31791	SDC2	HGF	[activation]
31792	ESR2	PPARGC1A	[activation]
31793	HIST3H3	FOXA1	[activation]
31794	TPX2	IKBKB	[activation]
31795	TFRC	RANBP9	[activation]
31796	NCOA4	AKT1	[activation]
31797	HSPB1	RSPH3	[activation]
31798	GUCY1B3	AGAP1	[activation]
31799	CALM1	CALD1	[activation]
31800	AR	SMAD1	[activation]
31801	HDAC3	TWIST1	[activation]
31802	HES1	FANCA	[activation]
31803	MMP3	PLG	[activation]
31804	CPB2	TINAG	[activation]
31805	MORN2	MPP3	[inhibition]
31806	TLK1	AURKA	[activation]
31807	HMGCR	INSIG2	[activation]
31808	BIRC2	PGAM5	[activation]
31809	ATF4	RPS6	[activation]
31810	NFATC1	GZF1	[activation]
31811	PTPN11	STAT3	[activation]
31812	YES1	GP6	[activation]
31813	SRF	NFKB1	[activation]
31814	ITGB1	CD82	[activation]
31815	F2	SERPINE2	[inhibition]
31816	PAXIP1	CD2BP2	[activation]
31817	UBA5	SNW1	[activation]
31818	APOE	PRDX2	[activation]
31819	KMT2D	CTNNB1	[activation]
31820	CLASRP	SRPK2	[activation]
31821	JAK2	PLCG2	[activation]
31822	AR	GSN	[activation]
31823	CTSK	FGFR3	[activation]
31824	DOK5	RET	[activation]
31825	ICT1	MRPL15	[activation]
31826	CSF2RB	IL5RA	[activation]
31827	BUB1	LEF1	[activation]
31828	DOK3	GRB2	[activation]
31829	CD40	TRAF5	[activation]
31830	TYK2	GHR	[activation]
31831	CDC5L	SMARCA5	[activation]
31832	SNAP25	PRKCA	[activation]
31833	YWHAG	FGD6	[activation]
31834	FYN	DOK3	[activation]
31835	CHEK2	KLK7	[activation]
31836	DAB2	TRIO	[activation]
31837	C10orf12	EHMT2	[activation]
31838	FN1	F2	[activation;inhibition]
31839	EP300	PAX8	[activation]
31840	CCND1	RABEP1	[activation]
31841	MAP2K5	LNX2	[activation]
31842	SH2B2	EPOR	[activation]
31843	IRS2	SOCS6	[inhibition]
31844	SRPK1	NXT2	[activation]

31845	PARD3	YWHAG	[activation]
31846	ACTA1	SMARCB1	[activation]
31847	DHRS4	TP53	[activation]
31848	KCNA2	FYN	[activation]
31849	DSN1	PIK3R3	[activation]
31850	TRRAP	MDM2	[activation]
31851	FAS	ATP6V0B	[inhibition]
31852	PRKAA1	MARK4	[inhibition]
31853	SLC26A3	SLC9A3R2	[activation]
31854	RECQL5	BNIPL	[activation]
31855	JAK3	IKBKG	[activation]
31856	RNF219	CTNNA1	[activation]
31857	MAPT	PSEN1	[activation]
31858	GSN	PTK2	[activation]
31859	ABL1	PTPN1	[activation]
31860	PSEN1	RAB11A	[activation]
31861	PIK3R1	PPFIA3	[activation]
31862	HSP90AB1	MCM7	[activation]
31863	KCNN3	DNM2	[activation]
31864	TTC1	SREBF2	[activation]
31865	GAPDH	OBSL1	[inhibition]
31866	STK4	ANXA2P2	[activation]
31867	CDC25C	MARK3	[activation]
31868	GNGT2	GNB4	[activation]
31869	SMARCA4	TERT	[activation]
31870	GNA13	RDX	[activation]
31871	RAC1	KPNA6	[activation]
31872	DDX5	RPA3	[activation]
31873	CYTH2	CCDC120	[activation]
31874	CDK11A	CSNK2A1	[activation]
31875	MDC1	RPA1	[activation]
31876	VTN	EGF	[activation]
31877	SMAD4	RIPK2	[activation]
31878	DAB1	HYAL3	[activation]
31879	HSP90AB2P	CDK4	[inhibition]
31880	TNFRSF11B	TNFSF11	[activation]
31881	PPP3R1	CABP2	[activation]
31882	CFLAR	SMAD3	[inhibition]
31883	TMSB4X	ANXA7	[activation]
31884	NTRK2	HSP90AB1	[activation]
31885	SEMA3B	NRP2	[activation]
31886	UBC	FOXP3	[activation]
31887	AR	NSD1	[activation]
31888	EGFR	RIOK2	[activation]
31889	MLH1	SPRTN	[activation]
31890	SETDB1	S100A10	[activation]
31891	LRRK2	NUMA1	[activation]
31892	TBPL1	IRAK4	[activation]
31893	CRK	HLA-B	[activation]
31894	PLCG2	SH3BP2	[activation]
31895	MCM3	MDM2	[activation]
31896	SRPK1	PTK2	[activation]
31897	CHEK2	MDM2	[activation]
31898	EIF2B4	DCC	[activation]
31899	PDGFRB	MFGE8	[activation]
31900	ETS1	TLX3	[activation]
31901	NRIP1	NR0B1	[activation]
31902	MEIS2	C1orf94	[activation]
31903	YWHAB	TSC1	[inhibition]
31904	ARPC4	CTTN	[activation]
31905	FLT1	PTPRB	[activation]
31906	ARHGAP1	CHEK2	[activation]
31907	STAP1	AR	[activation]
31908	IRS4	NUDCD3	[activation]
31909	MOS	DYDC1	[activation]
31910	VRK2	MAP2K1	[activation]
31911	PDGFB	LYVE1	[activation]
31912	IQCB1	IQGAP3	[inhibition]
31913	RAB34	SMAD4	[activation]
31914	PPM1A	GRM3	[activation]
31915	FANCC	FAAP20	[activation]
31916	CDC42	OPHN1	[activation]
31917	MYC	IGF2BP3	[activation]
31918	ERCC6	BAZ1B	[activation]
31919	PRKCA	LNX2	[activation]
31920	ITGB1	PRKCA	[activation]

31921	APP	DYRK1A	[activation]
31922	ADRB2	PRKDC	[activation]
31923	FES	HSP90AB1	[activation]
31924	NUP153	XPOT	[activation]
31925	RPA2	HNRNPA0	[activation]
31926	SIK2	IRS1	[activation]
31927	HIPK3	C1QA	[activation]
31928	TTF1	KAT2B	[activation]
31929	SNRNP200	STK4	[activation]
31930	CREBBP	JUNB	[activation]
31931	SMURF1	JAK1	[inhibition]
31932	ABI1	APBB1	[activation]
31933	CDON	CDH2	[activation]
31934	SRY	HDAC3	[activation]
31935	IKBKB	TP73	[activation]
31936	AURKB	SKA3	[activation]
31937	MAPK3	CASP9	[activation;inhibition]
31938	MAPK3	FKBP2	[activation]
31939	A2M	FBXW4	[inhibition]
31940	CORO2A	DCC	[activation]
31941	SRF	UBE2I	[activation]
31942	MDM2	SMARCA2	[activation]
31943	VHL	E2F1	[inhibition]
31944	PTBP3	CDK2	[activation]
31945	TNFRSF4	TRAF5	[activation]
31946	NR3C1	SUMO1	[activation]
31947	ESR1	IRS2	[activation]
31948	NUP93	ARF6	[activation]
31949	HDAC1	NKX2-2	[activation]
31950	ACTB	ERG	[activation]
31951	CASP8	AGR3	[activation]
31952	CENPF	LRRK2	[activation]
31953	CTSC	SUMO1	[activation]
31954	HIST1H3A	PAK1	[activation]
31955	XIAP	BMPR2	[activation]
31956	SLC6A9	IGHG1	[activation]
31957	PTPN6	ZAP70	[activation;inhibition]
31958	NFKB1	HSPA4	[activation]
31959	APP	ADAP2	[activation]
31960	SHC1	CBLB	[activation]
31961	SASH1	IKBKB	[activation]
31962	G3BP1	ITGA4	[activation]
31963	KIFC3	BCL6	[activation]
31964	RGL2	NOTCH2NL	[activation]
31965	NEDD8	RAN	[activation]
31966	HSP90AB1	INSRR	[activation]
31967	FLNB	TRAF6	[activation]
31968	APPL1	PLEKHF2	[activation]
31969	TPD52L1	TPD52L3	[activation;inhibition]
31970	NCOA3	PTEN	[activation]
31971	GRB2	HNRNPC	[activation]
31972	NLRP4	CHUK	[activation]
31973	MAP4	PIK3R1	[activation]
31974	MLLT4	ITGA4	[activation]
31975	TNFRSF14	HNRNPL	[activation]
31976	BCR	HCK	[activation]
31977	CSNK2A1	IFRD1	[activation]
31978	YWHAH	TIAM1	[activation]
31979	PHACTR4	GRB2	[activation]
31980	SUPT6H	CDKN1A	[activation]
31981	MAP2K6	PLCB2	[activation]
31982	HNF4A	SREBF2	[activation]
31983	PIAS1	ARID3A	[inhibition]
31984	SRC	LYN	[activation]
31985	EGFR	DOK6	[activation]
31986	TGM2	SMAD2	[activation]
31987	BTRC	NXF1	[activation]
31988	SKP1	CRY1	[inhibition]
31989	FOS	CHMP3	[activation]
31990	CAPN2	HNRNPA1	[activation]
31991	RPS6KA4	MAPK1	[activation]
31992	JUND	ETS1	[activation]
31993	DSTN	GH1	[activation]
31994	PIK3CG	GSN	[activation]
31995	DCN	AHSG	[inhibition]
31996	ANXA2	OBSL1	[activation]

31997	CAMK2G	CHAT	[activation]
31998	DNAJB2	ERBB2	[inhibition]
31999	FLNB	GRB2	[activation]
32000	RYR1	PRKACA	[activation]
32001	CYTH3	CCDC120	[activation]
32002	PPP2R1A	FECH	[inhibition]
32003	MAPK14	HDAC3	[activation]
32004	FER	CDH1	[activation;inhibition]
32005	CD247	NLGN1	[activation]
32006	FAM98B	CD81	[activation]
32007	CASP6	PRKAB2	[activation]
32008	CBL	WAS	[activation]
32009	CARF	APP	[activation]
32010	RAD50	EP300	[activation]
32011	CREBBP	CDK2	[activation]
32012	ADRB2	PCBP2	[activation]
32013	STX2	SNAP25	[activation]
32014	ULK1	SESN2	[inhibition]
32015	TNS1	MET	[activation]
32016	SMARCA5	BAZ2A	[activation]
32017	SRC	CDCP1	[activation]
32018	CBL	NTRK1	[activation]
32019	TTI2	RPAP3	[activation]
32020	TULP1	NCK1	[activation]
32021	NXF1	TAGLN2	[activation]
32022	JAM2	TJP1	[activation]
32023	UBE2E3	RNF166	[activation]
32024	CSPG4	GRIP2	[activation]
32025	H2AFX	ANXA2	[activation]
32026	MCM3	ITGA4	[activation]
32027	CDC25A	HLA-C	[activation]
32028	ITSN2	EGFR	[activation]
32029	MAP3K8	HSP90AA1	[activation]
32030	RPS28	ITGA4	[activation]
32031	MAPK1	SREBF2	[activation]
32032	HSPA4L	ARF6	[activation]
32033	IRF3	CREBBP	[activation]
32034	LATS1	KRT31	[inhibition]
32035	SUMO2	MYO1B	[activation]
32036	RIPK2	TRAF1	[activation]
32037	CCDC14	MAPK14	[activation]
32038	ITGA4	HP1BP3	[activation]
32039	RPTOR	YWHAQ	[activation]
32040	RPRD1A	GSTK1	[activation]
32041	MAP3K11	MAP2K4	[activation]
32042	ID4	HES1	[activation]
32043	DDX54	PGR	[activation]
32044	PRDM2	HIST1H3A	[activation]
32045	RALGAPB	RALGAPA1	[activation;inhibition]
32046	VASH1	CCDC23	[activation]
32047	AVEN	BCL2L1	[activation]
32048	HIF1A	DNAJB1	[inhibition]
32049	NAB1	EGR1	[activation]
32050	VDR	JUN	[activation]
32051	PGLYRP1	HSPBP1	[activation]
32052	STK3	STK4	[activation]
32053	KALRN	NDEL1	[activation]
32054	EIF4A3	RPS6	[activation]
32055	ANGPTL4	ITGB1	[activation]
32056	CDC5L	PPP2R1A	[activation]
32057	ANAPC5	TP53BP1	[activation]
32058	RICTOR	FBXO9	[activation]
32059	PARD3	CDH5	[activation]
32060	CDK11B	HSPA4	[activation]
32061	MYC	YWHAB	[activation]
32062	PLEKHG5	LNX2	[activation]
32063	HNRNPR	BMPR2	[activation]
32064	UBE2I	WNK1	[activation]
32065	FYN	CNN3	[activation;inhibition]
32066	EP300	SREBF2	[activation]
32067	PPP2R2B	CHEK2	[activation]
32068	TAOK3	LRRK2	[activation]
32069	MAST1	DNAJB1	[inhibition]
32070	CASP7	CAST	[inhibition]
32071	YWHAQ	GRB2	[activation]
32072	MARK2	AURKB	[activation]

32073	TSPAN12	TFCP2	[activation]
32074	SCHIP1	FOS	[activation]
32075	CASP7	RASA1	[activation]
32076	NUAK1	IGHA2	[activation]
32077	PSAT1	TIMP2	[activation]
32078	BLNK	CRKL	[activation]
32079	FGFR2	YWHAZ	[activation]
32080	TSC2	DAPK1	[activation]
32081	ECSIT	SMAD4	[activation]
32082	NECAB2	ADORA2A	[activation]
32083	FYB	PRKCQ	[activation]
32084	RBPJ	SNW1	[activation]
32085	HIST3H3	HDAC1	[activation]
32086	CBX1	HSPB1	[inhibition]
32087	EGFR	CD59	[activation]
32088	IQGAP2	RHOG	[activation]
32089	JUN	MTA3	[activation]
32090	MDM2	NUMB	[activation]
32091	RB1	AURKB	[activation]
32092	EGLN2	TRIP6	[activation]
32093	VCAM1	MCM5	[activation]
32094	KAT2A	IRF1	[activation]
32095	NDEL1	CWF19L2	[activation]
32096	MYOD1	SETD3	[activation]
32097	ADRB2	DSG2	[activation]
32098	DCN	ELN	[activation]
32099	LRRK1	FLT4	[activation]
32100	RPS17L	NUDC	[activation]
32101	ENOX2	TRAF5	[activation]
32102	RASA1	PIK3R1	[activation]
32103	USP7	CRKL	[activation]
32104	FLNB	RAC1	[activation]
32105	SNRNP70	VCAM1	[activation]
32106	CDK5RAP2	PCNT	[activation]
32107	SMARCA4	VDR	[activation]
32108	PLCH1	EGFR	[activation]
32109	UBE2E3	RNF181	[activation]
32110	PLK1	MYT1	[activation]
32111	JUN	PIAS2	[activation]
32112	EIF5	DUSP12	[activation]
32113	ACSF2	CMTM5	[activation]
32114	F2R	F2	[activation]
32115	EGFR	SGSM2	[activation]
32116	F2R	F2RL2	[activation]
32117	RELA	TAF1	[activation]
32118	CTU1	HSPA4	[activation]
32119	NXF1	SETDB1	[activation]
32120	THRB	RXRA	[inhibition]
32121	NR2E3	RBL1	[inhibition]
32122	CDKN1A	ABR	[activation]
32123	CYBB	ACTB	[activation]
32124	KLHL40	FAM208B	[activation]
32125	CHUK	CUL1	[activation]
32126	HSPB1	PTGES3	[activation]
32127	WEE1	PIN1	[inhibition]
32128	MDM2	C1orf173	[activation]
32129	PYCARD	IL6ST	[inhibition]
32130	EPAS1	ARNTL2	[activation]
32131	NXF1	CEP78	[activation]
32132	RELA	NCF1	[activation]
32133	PLEKHB2	C1orf94	[activation]
32134	ZDHHC17	GATA1	[activation]
32135	TH	MAPKAPK2	[activation]
32136	APP	IL15	[activation]
32137	GRB2	UGP2	[activation]
32138	EGFR	PANK4	[activation]
32139	ACVR1	NUAK2	[activation;inhibition]
32140	MAPT	TRAF6	[activation]
32141	MMP9	MMP10	[activation]
32142	AASDHPPT	TRAF2	[activation]
32143	RICTOR	TIA1	[activation]
32144	BRD7	TRIM24	[activation]
32145	CASP3	CRYAB	[activation]
32146	CXorf56	PDCL2	[activation]
32147	ARNTL	UBE2I	[activation]
32148	SRPK1	MAGOH	[activation]

32149	SASH3	SMAD7	[activation]
32150	LYN	ITGB2	[activation]
32151	IQSEC1	GNAQ	[activation]
32152	CTSG	AGT	[activation]
32153	FBXL2	PIK3R1	[activation]
32154	IGF1R	SOCS2	[inhibition]
32155	IL15RA	CDK2	[activation]
32156	DCN	EGFR	[activation]
32157	TXNDC5	NOTCH2NL	[activation]
32158	ATRX	PRKDC	[activation]
32159	TTN	RAPGEF2	[activation]
32160	ASS1	JAK2	[activation]
32161	RPS8	TP53	[activation]
32162	TNF	RALBP1	[activation]
32163	TWIST1	RELA	[activation]
32164	TP53	GTF3C3	[activation]
32165	LRRK2	KPNB1	[activation]
32166	PLK1	NEDD9	[activation]
32167	PRKCD	AFAP1	[activation]
32168	CAPZB	TRAF6	[activation]
32169	PPARGC1A	TP53	[activation]
32170	PYCARD	NAA15	[inhibition]
32171	STK4	SMR3B	[activation]
32172	SNAI1	XRCC3	[activation]
32173	HLA-B	MCM7	[activation]
32174	PPP2R5A	PYGM	[activation]
32175	TIMP2	RAB3A	[activation]
32176	GRB2	PIK3CG	[activation]
32177	AR	YWHAH	[activation]
32178	SIK2	PRKAA1	[inhibition]
32179	PTK2	EPHB2	[activation]
32180	BANP	MAX	[inhibition]
32181	CUL1	KDR	[activation]
32182	CDC26	FBXO5	[activation]
32183	DVL1	EPS8	[inhibition]
32184	HSP90AB1	PTK6	[activation]
32185	GRB2	HNRNPR	[activation]
32186	CDH1	GBAS	[activation]
32187	NXF1	NOTCH2NL	[activation]
32188	SIPA1	FMNL1	[activation]
32189	RHOB	FBXO42	[activation]
32190	TNFSF9	IRAK2	[activation]
32191	DENND3	MYC	[activation]
32192	TP53BP1	LRRK2	[activation]
32193	PRNP	LSAMP	[activation]
32194	TMED2	F2RL1	[activation]
32195	PIK3R1	FGFR1	[activation]
32196	AURKB	PLEKHA5	[activation]
32197	PIK3R4	YWHAB	[activation]
32198	IL6ST	JAK2	[activation]
32199	PDZRN3	BMPR2	[activation]
32200	IRS1	TYK2	[activation]
32201	IRS1	LRRK2	[activation]
32202	CAPN1	CAPNS1	[activation]
32203	PTK2	CSK	[activation]
32204	TLR3	PIK3R1	[activation]
32205	TNIK	MYL12A	[activation]
32206	C9orf156	RB1	[activation;inhibition]
32207	STX6	ENO1	[activation]
32208	FBN3	GADD45A	[activation]
32209	AR	CALM1	[activation]
32210	ANKRD53	LZTR1	[activation]
32211	MAPK14	EPB42	[activation]
32212	XIAP	BMPR1B	[activation]
32213	RARA	HDAC1	[activation]
32214	MYL6	NFKB1	[activation]
32215	RAD54B	CDC23	[activation]
32216	PTK2	ACTN1	[activation]
32217	KMT2A	MYC	[activation]
32218	S100A16	NXF1	[activation]
32219	MBP	MAP3K3	[activation]
32220	IKBKB	MARK3	[activation]
32221	JUND	SMAD4	[activation]
32222	IRF2	KAT2B	[activation]
32223	SLC25A6	NOTCH2NL	[activation]
32224	HCK	MAP4K5	[activation]

32225	CD247	GNAI2	[activation]
32226	DAB2	ACOT9	[activation]
32227	SPRY2	MAPK1	[inhibition]
32228	PTPRG	TEK	[activation]
32229	ACP1	PAK2	[activation]
32230	RAD21	RPA1	[activation]
32231	ABI2	ADAM29	[activation]
32232	SPP1	SLC39A6	[activation]
32233	FCN1	ELN	[activation]
32234	MAP1A	GRB2	[activation]
32235	DNM2	VAV1	[activation]
32236	NAPA	SNAP25	[activation]
32237	HSPB1	WDR66	[activation]
32238	PRKCA	ACTA1	[activation]
32239	MAP2K7	ALK	[activation]
32240	ARRB2	ERH	[inhibition]
32241	PRKCD	HSPB1	[activation]
32242	ATF2	PTPN1	[activation]
32243	MDM2	ESR2	[activation]
32244	PCNA	SETD8	[activation]
32245	VAV3	GRB2	[activation]
32246	ACTG1	TAB2	[activation]
32247	CTSG	VCAM1	[activation]
32248	MLH1	CYLC2	[activation]
32249	LAMA5	FBXO6	[inhibition]
32250	CFTR	PLD2	[activation]
32251	RELA	HSPA9	[activation]
32252	H2AFX	RAD17	[activation]
32253	SRSF6	TP53	[activation]
32254	YWHAB	IRS2	[activation]
32255	STAT5B	FGFR2	[activation]
32256	EPHB6	HSP90AA1	[activation]
32257	MS4A1	LCK	[activation]
32258	JADE2	PACSIN1	[activation]
32259	TRAF2	ZC2HC1C	[activation]
32260	ORC2	CCL2	[activation]
32261	STK38	CALM1	[activation]
32262	A2M	KLK2	[inhibition]
32263	NLRP3	MAVS	[inhibition]
32264	APEX1	TP53	[activation]
32265	ESR1	STAT3	[activation]
32266	TRAP1	OBSL1	[activation]
32267	SET	GZMA	[activation]
32268	HNRNPL	TP53	[activation]
32269	CRY1	PER2	[inhibition]
32270	CDC25C	BRSK2	[activation]
32271	HEATR2	MYC	[activation]
32272	FBN3	ANXA7	[activation]
32273	PLD1	PEA15	[activation]
32274	DAB1	VENTX	[activation]
32275	CD2BP2	RAD54B	[activation]
32276	GRK4	HSP90AB1	[activation]
32277	SRC	EP300	[activation]
32278	PRKAR1A	CDH2	[activation]
32279	FEM1B	CHEK1	[activation]
32280	PML	RUNX1	[activation]
32281	MOK	MAPK6	[activation]
32282	STK3	RAE1	[activation]
32283	TP53BP1	NFKB1	[activation]
32284	CAPZA2	DAPK1	[activation]
32285	CCR1	CCL23	[activation]
32286	SMAD4	CAMK2G	[activation]
32287	ETS1	CREBBP	[activation]
32288	BCR	IRS1	[activation]
32289	SORT1	ELAVL1	[activation]
32290	SRPK2	RBM23	[activation]
32291	FAM208B	MORN3	[activation]
32292	EIF4A1	GABARAPL2	[activation]
32293	MAPK8	PIK3R1	[activation]
32294	HIF1A	TRAF6	[activation]
32295	FYN	CDC27	[activation]
32296	GH1	DNAJC8	[inhibition]
32297	APP	WHSC1	[activation]
32298	VCAM1	ESYT1	[activation]
32299	THRB	TP53	[activation]
32300	GNAZ	NMT1	[activation;inhibition]

32301	APH1A	THPO	[activation]
32302	BMX	BTK	[activation]
32303	CDKN1A	ESR1	[activation]
32304	PDGFRB	PTPRB	[activation]
32305	GP2	ANXA4	[activation]
32306	BAZ2A	FYN	[activation]
32307	GABARAPL2	ATG10	[activation]
32308	EIF2AK2	HIST2H2AC	[activation]
32309	STAT3	PTPN1	[activation]
32310	TRAF1	CASP10	[activation]
32311	HIST1H1A	PRKCB	[activation]
32312	LIN37	RBL2	[inhibition]
32313	CXorf57	DNAJA4	[inhibition]
32314	FERMT3	ITGA4	[activation]
32315	KCNE3	POLA2	[activation]
32316	NR0B1	ESR2	[activation]
32317	BAG3	GNAO1	[activation;inhibition]
32318	UVRAG	PIK3C3	[activation]
32319	SKI	GLI2	[inhibition]
32320	IGSF1	INHBB	[activation]
32321	SRSF7	SRPK2	[activation]
32322	SNAP23	STX6	[activation]
32323	ABL1	NBEA	[activation]
32324	ICAM1	HIST1H1C	[activation]
32325	MMP9	MMP7	[activation;inhibition]
32326	BIRC3	E2F1	[activation;inhibition]
32327	USHBP1	CCDC87	[activation]
32328	IQGAP1	MAP3K3	[activation]
32329	ATM	CREB1	[activation]
32330	PLCG1	CD22	[activation]
32331	MAPK1	PML	[activation]
32332	RHOC	ATF2	[activation]
32333	TWIST2	TP53	[activation]
32334	ITGA4	EIF3A	[activation]
32335	TRAF6	C19orf10	[activation]
32336	BCAR1	GRB2	[activation]
32337	LRRK2	NFATC2	[activation]
32338	NOX1	NOXA1	[activation]
32339	CHEK1	NEK9	[activation]
32340	ITGA4	NUDC	[activation]
32341	AHNAK	AKT1	[activation]
32342	TSC22D1	PRKCI	[activation]
32343	FGFR1	HTR1A	[activation]
32344	NLK	FAM222A	[inhibition]
32345	CBX8	SPAG9	[inhibition]
32346	EFNB2	PECAM1	[activation]
32347	PLSCR2	EP300	[activation]
32348	ICAM1	CCT3	[activation]
32349	GMPS	HLA-C	[activation]
32350	PIK3R1	DAG1	[activation]
32351	FAS	SUMO1	[activation]
32352	MRAS	SHOC2	[activation]
32353	SMAD1	GSK3B	[activation;inhibition]
32354	CBLB	CFLAR	[inhibition]
32355	TLN1	DAG1	[activation]
32356	PRNP	BAZ2B	[activation]
32357	SETDB2	IGHG1	[activation]
32358	CD46	C4B	[inhibition]
32359	TFDP2	RB1	[inhibition]
32360	UBE2A	TP53	[activation]
32361	CITED2	EP300	[activation]
32362	MAPK9	EEF2K	[activation]
32363	SPATA24	LRRK2	[activation]
32364	RAB21	HLA-B	[activation]
32365	RABGGTB	RAB11A	[activation]
32366	YWHAG	GRB2	[activation]
32367	STAT3	MRPS31	[activation]
32368	MPRIP	RPA3	[activation]
32369	CREBBP	RPS6KA5	[activation]
32370	RAC1	FMNL1	[activation]
32371	ABCC8	ENSA	[activation;inhibition]
32372	FAM154A	MVP	[activation]
32373	TRAF6	EIF2S3	[activation]
32374	JUN	ITCH	[activation]
32375	SRPK2	NUP50	[activation]
32376	PBK	CCNB1	[activation]

32377	SP1	ETS1	[activation]
32378	NUP54	APC	[activation]
32379	INHBA	CHRDL2	[activation]
32380	SPAG9	EIF4A2	[inhibition]
32381	SRC	FGR	[activation]
32382	CASP8	ST14	[inhibition]
32383	PDPK1	PTK2B	[activation]
32384	ARPC4	NXF1	[activation]
32385	PMS1	HELB	[activation]
32386	RB1	CCDC180	[activation;inhibition]
32387	SRPK3	LUC7L	[activation]
32388	CRIP2	RELA	[activation]
32389	FBP1	BCL2L1	[activation]
32390	POLR2G	PRKRA	[activation]
32391	MYC	SIRT6	[activation]
32392	BLNK	ITK	[activation]
32393	INPP5K	PYGM	[activation]
32394	LIN9	LIN52	[inhibition]
32395	GRB2	HSPG2	[activation]
32396	MAPK13	PPA1	[activation]
32397	NUP210	CDK1	[activation]
32398	CHN1	BMPR1B	[activation]
32399	CDK19	PIP	[activation]
32400	TNIK	ANXA2	[activation]
32401	BAG3	CRKL	[activation]
32402	MYC	ECT2	[activation]
32403	HIF1A	AR	[activation]
32404	HDAC1	KCTD11	[activation]
32405	STAC3	WHSC1L1	[activation]
32406	MYO10	FBXW11	[inhibition]
32407	GABARAPL2	SMARCB1	[activation]
32408	CCNG1	TNIP1	[activation]
32409	VTN	TGFB1	[inhibition]
32410	GBP3	MAP4K4	[activation]
32411	SP1	TPI1	[activation]
32412	CRK	PHACTR2	[activation]
32413	ATF2	ITGB1	[activation]
32414	CFLAR	SPATS1	[inhibition]
32415	AKT1	PI4K2B	[activation]
32416	DIAPH1	SRGAP2	[activation]
32417	C12orf65	APP	[activation]
32418	GCG	MEP1A	[activation]
32419	SHOC2	ERBB2IP	[inhibition]
32420	TRAF6	SDCCAG8	[activation]
32421	RFX6	CCNK	[activation]
32422	EP300	TBX21	[activation]
32423	STK4	CKAP5	[activation]
32424	VIP	SGTA	[activation]
32425	PRKCA	HAND1	[activation]
32426	SLC52A2	ADRB2	[activation]
32427	JAG1	UBC	[activation]
32428	PRKACA	CIITA	[activation]
32429	FGFR4	CASC3	[activation]
32430	CS	HLA-B	[activation]
32431	ANKRD1	TULP3	[activation]
32432	DGCR8	MAPK1	[activation]
32433	CRAMP1L	RASA4	[activation]
32434	ZAP70	SH2B3	[activation]
32435	MIDN	GADD45G	[activation]
32436	CDC20	TP73	[activation;inhibition]
32437	SSTR5	CORT	[activation]
32438	MDC1	RAD51	[activation]
32439	ARF6	PSD4	[activation]
32440	HSPA1L	NFKB2	[inhibition]
32441	HSPA6	RPAP3	[inhibition]
32442	NOXA1	MAPK1	[activation]
32443	BARD1	YWHAZ	[activation]
32444	CD5	FYN	[activation]
32445	HSPA8	JUN	[activation]
32446	PAX6	NKIRAS2	[activation]
32447	FAM86A	FAM86B2	[activation]
32448	TF	IGF1	[activation]
32449	TBXA2R	PTGIR	[inhibition]
32450	RBBP8	MRE11A	[activation]
32451	NCKIPSD	RAC1	[activation]
32452	CYTH3	CYTIP	[activation]

32453	RUNX1	TLE1	[activation]
32454	EIF4A2	CMBL	[activation]
32455	TRAF6	KIR2DL4	[activation]
32456	HSPA8	REL	[inhibition]
32457	NCOA3	PRKCZ	[activation]
32458	STX16	VTI1A	[activation]
32459	NCKAP1	SPP1	[activation]
32460	TERF1	PFN2	[activation]
32461	BEST1	PPP2CA	[inhibition]
32462	IKBKB	EPRS	[activation]
32463	RALGAPB	DHX15	[activation]
32464	YWHAZ	IRS4	[activation]
32465	SNURF	SRPK2	[activation]
32466	ASIC3	PRKACA	[activation]
32467	GABARAPL2	U2AF2	[activation]
32468	BHLHE40	UBE2I	[activation]
32469	ESR2	DDX5	[activation]
32470	LRRK2	AKT1	[activation]
32471	CD4	LGALS1	[inhibition]
32472	TIMELESS	CRY2	[activation]
32473	BLK	HSP90AA1	[activation]
32474	C4B	CFB	[inhibition]
32475	FOXI1	APP	[activation]
32476	APBB1	DIAPH1	[activation]
32477	TRAF2	TNFRSF12A	[activation]
32478	MET	SOCS5	[inhibition]
32479	CDK2	MYOD1	[activation]
32480	STX11	APP	[activation]
32481	ARF6	RABGAP1	[activation]
32482	RB1	IRF3	[inhibition]
32483	LUC7L2	NDUFA4L2	[activation]
32484	FAS	EZR	[activation]
32485	B2M	TFRC	[activation]
32486	MAPRE3	APC2	[activation]
32487	HIVEP1	MAPK14	[activation]
32488	COPA	EGFR	[activation]
32489	FGFR1	CDC20	[activation]
32490	GAS7	CYFIP1	[inhibition]
32491	ZNF106	SMAD2	[activation]
32492	CYFIP1	PPP1CC	[inhibition]
32493	ACKR4	CCL2	[activation]
32494	GABARAPL2	HSP90AB1	[activation]
32495	SRPK3	SNRPA	[activation]
32496	WIBG	LRRK2	[activation]
32497	CD38	CD3E	[activation]
32498	GEM	GMIP	[activation]
32499	GNAI2	LHCGR	[activation]
32500	APP	SMURF1	[inhibition]
32501	GABARAP	HSPH1	[activation]
32502	NR4A1	POLA2	[activation]
32503	STAT1	NFKBIA	[activation]
32504	EIF2B4	EGFR	[activation]
32505	RASSF3	STK3	[activation]
32506	CASP7	PSIP1	[activation]
32507	HCK	SPRR2A	[activation]
32508	EGFR	RNMTL1	[activation]
32509	GH1	GLRX3	[inhibition]
32510	SMAD5	ANKRD13A	[activation;inhibition]
32511	RRP1	GABARAPL2	[activation]
32512	TRAF6	XIAP	[activation]
32513	EP300	PRDM6	[activation]
32514	UGT1A3	UGT1A8	[activation]
32515	HSP90AB1	SRPK1	[activation]
32516	RAN	NSMF	[activation]
32517	CD81	CD82	[activation]
32518	EIF4EBP3	CDKN1A	[activation;inhibition]
32519	EGLN3	MAPK6	[activation]
32520	ESR1	AKT2	[activation]
32521	DYRK1A	ISG15	[activation;inhibition]
32522	BLZF1	BCL6	[activation]
32523	WAS	NCK1	[activation]
32524	HCK	MET	[activation]
32525	TLR4	EGFR	[activation]
32526	PRKCA	NXF1	[activation]
32527	ADIPOR1	APPL1	[activation]
32528	STAG1	STAG2	[activation]

32529	GNB1	ARHGEF18	[activation]
32530	PLCG1	VAV3	[activation]
32531	HMGN1	PRKACA	[activation]
32532	MYLK	ACTC1	[inhibition]
32533	INSR	GNB2L1	[activation]
32534	CSNK1G2	APP	[activation]
32535	SKP2	RASSF1	[inhibition]
32536	TKT	FTSJ1	[activation]
32537	TGFB1	CCL5	[activation]
32538	DCN	MMP2	[inhibition]
32539	GZMA	GOLGA2	[activation]
32540	FOXC1	HMGB1	[activation]
32541	PAFAH1B1	DNAI1	[activation]
32542	IGKC	IGHG4	[activation]
32543	CAPN1	FANCC	[activation]
32544	E2F1	NRIP1	[activation]
32545	NRAS	SMAD1	[activation]
32546	DAB1	C1orf51	[activation]
32547	MYL6B	IQGAP1	[activation]
32548	MOS	LZTS2	[activation]
32549	PIK3CA	APPL1	[activation]
32550	RPS24	VCAM1	[activation]
32551	BMX	ERBB2	[activation]
32552	PRKDC	JUN	[activation]
32553	SMAD1	RAB2B	[activation]
32554	NRBP1	PPP1CC	[activation]
32555	SS18	GRB2	[activation]
32556	RANBP1	EGFR	[activation]
32557	ANGPTL7	APP	[activation]
32558	FGFR3	ARAP1	[activation]
32559	THPO	MPL	[activation]
32560	PPARG	PIN1	[activation]
32561	GRB2	RIF1	[activation]
32562	CCDC70	GOLGA2	[activation]
32563	PTEN	INHBE	[activation]
32564	KRT36	GRB2	[activation]
32565	ANXA2	ERBB3	[activation]
32566	HLA-E	PPP1R16A	[activation]
32567	PIK3CA	GSN	[activation]
32568	XRCC1	OGG1	[activation]
32569	BCCIP	ITSN2	[activation]
32570	IKBKB	HSPA9	[activation]
32571	CASP3	RAD51	[activation]
32572	CRK	HSPA5	[activation]
32573	VGLL4	MEF2A	[activation]
32574	PLK1	C9orf114	[activation;inhibition]
32575	PLK1	DIXDC1	[activation]
32576	CFTR	SNAP23	[activation]
32577	CDC42	LRRK2	[activation]
32578	RCVRN	IL15RA	[inhibition]
32579	RALA	CALM1	[activation]
32580	YAP1	AKT1	[activation]
32581	STAT3	FOXM1	[activation]
32582	ARPC3	CETP	[activation]
32583	NOL12	CDK4	[inhibition]
32584	CCDC121	USHBP1	[activation]
32585	EEF2K	MAPK11	[activation]
32586	ATG7	SUMO2	[activation]
32587	PAX6	LHX2	[activation]
32588	SGK1	CS	[activation;inhibition]
32589	RGS20	ABHD17A	[activation;inhibition]
32590	RICTOR	TUBB	[activation]
32591	ITK	ERBB2	[activation]
32592	NKX2-1	RARA	[activation]
32593	TRMT112	RGS20	[activation;inhibition]
32594	VCAM1	CTSH	[activation]
32595	PAXIP1	ANAPC5	[activation]
32596	IQSEC1	APOE	[activation]
32597	LRRK2	PHB	[activation]
32598	TNIK	MACF1	[activation]
32599	PIAS2	PAXIP1	[activation]
32600	IRF3	DDX58	[inhibition]
32601	FANCL	ACVR1	[activation]
32602	FOXO1	YWHAZ	[activation]
32603	VRK2	RAN	[activation]
32604	IRF3	TMEM173	[activation]

32605	SFN	BAD	[inhibition]
32606	RB1	SETD7	[activation]
32607	PYHIN1	BAZ1B	[activation]
32608	BORA	BTRC	[activation]
32609	CABP4	CACNA1F	[activation]
32610	AGFG1	XPO1	[activation]
32611	APP	GSN	[activation]
32612	XPO6	STK4	[activation]
32613	SMC2	TFE3	[activation]
32614	RAD51C	HRNR	[activation]
32615	TRIM25	APC	[inhibition]
32616	CAPN2	PTPN1	[activation]
32617	FAM111B	NXF1	[activation]
32618	NXF1	ATG16L1	[activation]
32619	SLX4	DDX24	[activation]
32620	CDH13	CCND1	[activation]
32621	ANKRD27	VAMP7	[activation]
32622	KCNH1	CALM1	[activation]
32623	MAPKAPK5	EEF2K	[activation]
32624	ARL8B	ELAVL1	[activation]
32625	DNM1L	CDC5L	[activation]
32626	TRAPPC5	A2M	[inhibition]
32627	HSP90AB1	KLHL26	[activation]
32628	TGFB1	ITGB8	[activation]
32629	SRPK2	SRSF2	[activation]
32630	PRR14L	PPP2R1A	[inhibition]
32631	TP53	GAPDH	[activation]
32632	PIP5K1C	TLN2	[activation]
32633	DLG2	FZD4	[activation]
32634	CDH1	FGFR1	[activation]
32635	LRRK1	CBLB	[inhibition]
32636	BARD1	DDX39B	[activation]
32637	NR3C1	STIP1	[activation]
32638	GRB2	RCC2	[activation]
32639	GAK	BAG5	[activation;inhibition]
32640	PDGFRB	VAV2	[activation]
32641	AKAP8L	ILK	[activation]
32642	NCF4	PRKCD	[activation]
32643	DDR2	SHC1	[activation]
32644	MRPS23	ICT1	[inhibition]
32645	PIAS1	MSX1	[inhibition]
32646	LRRK2	PTGS2	[activation]
32647	RIMS1	CACNA1C	[activation]
32648	NFYB	DRAP1	[activation]
32649	REL	PTK6	[activation]
32650	SENP2	AGTRAP	[activation]
32651	JAK2	YES1	[activation]
32652	SH3RF2	RAC1	[activation]
32653	VEGFA	AKT1	[activation]
32654	UBE2H	RELA	[activation]
32655	USP7	ATG10	[activation]
32656	GNAO1	HRH4	[activation]
32657	RXRA	NR1H2	[inhibition]
32658	PLCG1	GLTSCR1	[activation]
32659	MKNK1	MAPK11	[activation]
32660	EVL	FYB	[activation]
32661	RAB5A	VPS45	[activation]
32662	YWHAG	PARD3B	[activation]
32663	VCAM1	EIF4H	[activation]
32664	DOCK8	MYD88	[activation]
32665	HNRNPA1	MRPS27	[activation]
32666	BTK	DAPP1	[activation]
32667	PRKAA2	PRKAA1	[inhibition]
32668	MYLK	MAPK3	[inhibition]
32669	NR5A2	NSD1	[activation]
32670	CASP8	RFFL	[inhibition]
32671	PRELP	COL1A1	[activation]
32672	CDC37	PRKAA1	[inhibition]
32673	ANXA1	DLAT	[activation]
32674	SUMO1	RAD54B	[activation]
32675	CRK	DLGAP2	[activation]
32676	SMAD4	FBP2	[activation]
32677	RPA3	PRPF6	[activation]
32678	HOXB4	CREBBP	[activation]
32679	TTPAL	TGFBR1	[inhibition]
32680	ANAPC1	FZR1	[activation]

32681	CPN1	CDC42	[activation]
32682	TOP3A	TP53	[activation]
32683	TRIM54	ARHGEF5	[activation]
32684	NR3C1	AR	[activation]
32685	NFKB1	SIRT1	[activation]
32686	RAB10	RPA3	[activation]
32687	KSR1	PPP2R5A	[activation]
32688	CTNNB1	EZH2	[activation]
32689	ARRB1	YES1	[activation]
32690	SMG7	TP53	[activation]
32691	NXF1	BMS1	[activation]
32692	ANXA7	A2M	[inhibition]
32693	RPA1	SLFN11	[activation]
32694	SYP	GRB2	[activation]
32695	SNCA	YWHAE	[activation]
32696	DYNC1H1	NF1	[activation]
32697	PRKCA	VTN	[activation]
32698	IKBKG	RHOA	[activation]
32699	NR4A2	EIF3A	[activation]
32700	PHLDA3	SAAL1	[activation]
32701	SRPK3	HSP90AB1	[activation]
32702	CDCA8	GAS2L3	[activation]
32703	EPSTI1	ESR1	[activation]
32704	ACTN4	ICAM1	[activation]
32705	MYO1B	CCDC8	[activation]
32706	NOTCH2NL	HPCAL1	[activation]
32707	CSNK1D	CSNK1E	[activation]
32708	CBL	GAPDH	[inhibition]
32709	HIST2H2AB	SMARCA5	[activation]
32710	PRKACA	THOP1	[activation]
32711	HSPE1	TNFRSF14	[activation]
32712	PLK1	DVL2	[activation]
32713	OBSL1	HIST1H4A	[activation;inhibition]
32714	ACTB	PFDN1	[activation]
32715	ROCK1	LIMK1	[activation]
32716	UBL4A	VAPB	[activation]
32717	PECAM1	ABL2	[activation]
32718	CACNA1A	HSPG2	[activation]
32719	CBL	PTPRG	[activation]
32720	CTCF	THRB	[activation]
32721	ADRB2	RGS2	[activation]
32722	RHOG	PLD1	[activation]
32723	FOXP2	MAPK3	[activation]
32724	HTR2A	GNAI1	[activation]
32725	CFL1	SSH3	[activation]
32726	CAV1	ABL1	[activation]
32727	MCM6	ITGA4	[activation]
32728	TRAF6	NAA50	[activation]
32729	TP53BP1	EP300	[activation]
32730	PRKAA1	EIF5B	[inhibition]
32731	KMT2A	SVIL	[activation]
32732	SMEK1	MAPK14	[activation]
32733	ACTN1	KCTD6	[activation]
32734	STK3	STRN	[activation]
32735	CCDC102B	FAM127B	[activation]
32736	PTPRJ	NTRK1	[activation]
32737	IL4R	IL2RG	[activation]
32738	INS	NOV	[activation]
32739	VCAM1	CSNK1A1L	[activation]
32740	CAPN1	TIRAP	[activation]
32741	TP53	PHKG2	[activation]
32742	HIST1H3A	PRMT5	[activation]
32743	NEDD8	EIF2A	[activation]
32744	SP1	EP300	[activation]
32745	CD44	ATF2	[activation]
32746	NBAS	OLFM2	[activation]
32747	LAS1L	ETS1	[activation]
32748	FMNL1	PPP1R12A	[activation]
32749	DVL2	SIRT1	[activation]
32750	C2orf47	LYN	[activation]
32751	YWHAB	PLEKHA5	[activation]
32752	GABARAPL2	G3BP2	[activation]
32753	GRB2	KRT32	[activation]
32754	GEM	KRT31	[activation]
32755	PRKCA	TYK2	[activation]
32756	MYB	ZFHX3	[activation]

32757	SHC1	STAT5A	[activation]
32758	PHYHIP	HNRNPA1	[activation]
32759	INHBB	ACVR1B	[activation]
32760	FKBP1A	SMAD6	[inhibition]
32761	MAPK6	MAP3K12	[activation]
32762	GRAP	FYN	[activation]
32763	ACTG1	MYOC	[activation]
32764	EIF2S2	PAXIP1	[activation]
32765	BMP1	CHRD	[activation]
32766	NBN	MLH1	[activation]
32767	FAM53C	YWHAZ	[activation]
32768	TRAF2	EP300	[activation]
32769	PRKDC	WHSC1	[activation]
32770	CAMK2B	HSP90AA1	[activation]
32771	CCNA1	KLHDC9	[activation]
32772	HMGB1	PLG	[inhibition]
32773	DNM3	FYN	[activation]
32774	CDH1	ERBB2IP	[inhibition]
32775	STAT1	EIF1AD	[activation]
32776	PXDN	FBXO6	[inhibition]
32777	SH2D1A	EGFR	[activation]
32778	FANCE	PCNA	[activation]
32779	MYC	HCFC2	[activation]
32780	ATF4	CCDC106	[activation]
32781	KITLG	CMA1	[activation]
32782	FANCA	FAAP20	[activation]
32783	MTOR	GPHN	[activation]
32784	CSNK1E	ZSWIM8	[activation]
32785	YWHAE	TSC2	[activation]
32786	JUN	PPARG	[activation]
32787	FZR1	BRSK2	[inhibition]
32788	IKBKB	COPS3	[activation]
32789	ICK	RPTOR	[activation]
32790	HSP90AB1	METTL22	[activation]
32791	NXF1	ITPA	[activation]
32792	HOPX	TLX3	[activation]
32793	VRK2	BCL2L1	[activation]
32794	ATG12	ATG5	[activation]
32795	NR3C1	GNB1	[activation]
32796	UGT1A10	UGT1A1	[activation]
32797	TRAF2	CD27	[activation]
32798	ABL1	POLR2A	[activation]
32799	HMGA2	SMAD9	[activation]
32800	ATF4	NDC80	[activation]
32801	TNFRSF10A	TRADD	[activation]
32802	CTSG	KNG1	[activation]
32803	SOX2	HSPB1	[activation]
32804	PDHB	ITGB1	[activation]
32805	IL1F10	IL1R1	[activation]
32806	RAVER1	AURKB	[activation]
32807	EGLN2	EGLN3	[activation]
32808	XPO5	NUP153	[activation]
32809	PCNA	HLA-B	[activation]
32810	ACTN1	HSP90AB1	[activation]
32811	UTP15	EGFR	[activation]
32812	MAPK9	MAPKAPK5	[activation]
32813	NOX1	TNFRSF1A	[activation]
32814	TP53	WDR48	[activation]
32815	GPANK1	GRB2	[activation]
32816	PAK1	GNB1	[activation]
32817	DYSF	DHX15	[activation]
32818	YWHAZ	IL9R	[activation]
32819	HSPB1	HNRNPH2	[activation]
32820	SRPK2	LZTS2	[activation]
32821	SAMHD1	CD81	[activation]
32822	CASP8	PARP2	[activation]
32823	ECSIT	PSEN2	[activation]
32824	MRFAP1L1	RADIL	[inhibition]
32825	CCDC28A	SRPK2	[activation]
32826	SMAD1	YY1	[activation]
32827	NR3C1	CEBPA	[activation]
32828	FECH	ATM	[activation]
32829	EIF2B4	EIF2B2	[activation]
32830	ITGA4	MCM5	[activation]
32831	TENC1	CAV1	[activation]
32832	FGF2	VTN	[activation]

32833	SUMO1	TNS3	[activation]
32834	ADRBK1	GRM1	[activation]
32835	PIK3CG	UTRN	[activation]
32836	TRIM50	UBE2E3	[activation]
32837	PIN1	FOXO4	[activation]
32838	SYT5	NRXN1	[activation]
32839	PLCG1	FAS	[activation]
32840	MEOX2	TRAF6	[activation]
32841	LCN2	HGF	[inhibition]
32842	KATNA1	CD40	[activation]
32843	FANCG	POLN	[activation]
32844	FYN	HTR6	[activation]
32845	GABPA	CREBBP	[activation]
32846	CDK4	CAMK1	[activation]
32847	NOTCH2	EGFL7	[activation]
32848	TNFRSF1A	SLC25A6	[activation]
32849	CRKL	CBLB	[activation]
32850	RNF11	UBE2E2	[activation]
32851	RNF114	MAPK3	[activation]
32852	CNTNAP1	KCNA1	[activation]
32853	MTMR9	MOS	[inhibition]
32854	GPS2	CCNA1	[inhibition]
32855	NMRAL1	ASS1	[activation]
32856	PPP2R1A	TRAF6	[activation]
32857	RND3	ROCK1	[activation]
32858	HDAC1	MAPK8	[activation]
32859	CASP3	XIAP	[activation]
32860	FCN2	LRP1	[activation]
32861	RELA	TBP	[activation]
32862	PRKAA2	ULK1	[inhibition]
32863	ATP1A1	SMAD2	[activation]
32864	DOCK6	LRCH3	[activation]
32865	APP	MTERFD2	[activation]
32866	HLA-B	RAB6A	[activation]
32867	MAPK14	ZNHIT1	[activation]
32868	FEM1B	APAF1	[activation]
32869	ULK1	ATG4B	[inhibition]
32870	IL17RB	RPS6KA5	[activation]
32871	GRIA2	GRIK5	[activation]
32872	GAPVD1	RAD21	[activation]
32873	AKR7A3	AKR7A2	[activation]
32874	PRKAA1	PA2G4	[inhibition]
32875	NFKBIA	SLC25A4	[inhibition]
32876	FOS	RUNX1	[activation]
32877	MAPRE1	MAPRE3	[activation]
32878	ASB15	MIF	[activation]
32879	BMF	MAPK8	[activation]
32880	CDC37	RHOBTB2	[activation]
32881	RUNX2	TP53	[activation]
32882	NBN	NAT2	[activation]
32883	C5AR1	ARRB2	[activation]
32884	TP73	PRKACA	[activation;inhibition]
32885	IRAK1	MBP	[activation;inhibition]
32886	MLH1	CCDC180	[activation]
32887	PKIB	PIN1	[inhibition]
32888	HIST1H1D	ICAM1	[activation]
32889	PPP3CA	JUN	[activation]
32890	NR4A1	PLXND1	[activation]
32891	HLA-C	MAPRE1	[activation]
32892	FZR1	UBE2D1	[inhibition]
32893	CTU2	URM1	[activation]
32894	TSSK1B	TGFBR2	[inhibition]
32895	TP53	ANXA7	[activation]
32896	YWHAE	HDAC4	[activation]
32897	PPARA	EP300	[activation]
32898	PDIA4	GADD45G	[activation]
32899	LSM10	BMPR2	[activation]
32900	ANAPC11	ANAPC4	[activation]
32901	HNRNPA0	MAPK6	[activation]
32902	GRB2	PEX13	[activation]
32903	DDX39B	BMI1	[activation]
32904	IRS4	GBF1	[activation]
32905	PRNP	MTG1	[activation]
32906	ABL1	C6	[activation]
32907	STK4	GABARAPL2	[activation]
32908	BTRC	IKBKB	[activation]

32909	YWHAB	CRTC3	[activation]
32910	GSTM3	TRAF6	[activation]
32911	SMAD2	ST5	[activation]
32912	LARP4B	CDK18	[activation]
32913	EHMT2	HIST1H1E	[activation]
32914	CASP3	ELAVL1	[activation]
32915	SMURF1	RHOB	[inhibition]
32916	PRKCA	RHO	[activation]
32917	NLRP3	PYCARD	[inhibition]
32918	CSDE1	PAK2	[activation]
32919	BCL3	MAPK3	[activation]
32920	GEM	TFAP4	[activation]
32921	EP300	SEPP1	[activation]
32922	B2M	HLA-E	[activation]
32923	CDC5L	DDX17	[activation]
32924	PPP1R12A	RPA3	[activation]
32925	MAPT	LRRK2	[activation]
32926	GRB2	EPHB2	[activation]
32927	HAPLN1	MMP9	[activation;inhibition]
32928	H2AFX	ATF2	[activation]
32929	TOP1	H3F3A	[activation]
32930	TXNDC11	RPS6KA5	[activation]
32931	RAF1	RALGDS	[activation]
32932	CDH1	NANS	[activation]
32933	C6	MGEA5	[activation]
32934	PYHIN1	GPC4	[activation]
32935	ELANE	YARS	[activation]
32936	CTNNB1	ETS1	[activation]
32937	NCK2	FYB	[activation]
32938	TUBB	DPYSL2	[activation]
32939	C16orf80	LRRK2	[activation]
32940	PPAP2B	CTNND1	[activation]
32941	AR	NFKB1	[activation]
32942	LIN54	TFDP2	[inhibition]
32943	IQGAP3	PASK	[inhibition]
32944	AKT1	PMS2	[activation]
32945	HLA-B	LRRC40	[activation]
32946	CYGB	DDI1	[inhibition]
32947	CYLD	TRPA1	[inhibition]
32948	YWHAB	R3HDM1	[activation]
32949	SSU72	RAD21	[activation]
32950	DEFA1	DEFA3	[inhibition]
32951	SHC1	DAG1	[activation]
32952	TFE3	HMGB1	[activation]
32953	PCSK1	REN	[activation;inhibition]
32954	RAD52	RAD51	[activation]
32955	DAB1	PIN1	[activation]
32956	NRAS	XPA	[activation]
32957	LRMP	WNK1	[activation]
32958	FLOT2	OBSL1	[inhibition]
32959	LYN	C21orf59	[activation]
32960	NCK1	ASAP2	[activation]
32961	TNFRSF1A	TRAF3	[activation]
32962	CALCOCO2	SHC1	[activation]
32963	RNPS1	SRPK1	[activation]
32964	KAT5	FOXP3	[activation]
32965	PTPRB	MAPK1	[activation]
32966	RASIP1	ARFGAP1	[inhibition]
32967	GRB2	PPP5C	[activation]
32968	NOTCH1	TGFBR1	[activation]
32969	IKBKE	GRB2	[activation]
32970	TCOF1	ICAM1	[activation]
32971	PER2	TP53	[activation]
32972	STRAP	NXF1	[activation]
32973	NUAK2	LATS1	[inhibition]
32974	UBE2U	RNF41	[activation]
32975	L1CAM	ITGAV	[activation]
32976	STK24	G3BP2	[activation]
32977	AKT1	CTNNA1	[activation]
32978	U2AF2	DDX17	[activation]
32979	CDK6	ZSCAN1	[inhibition]
32980	MYO1B	MAP3K3	[activation]
32981	PTN	CACNB4	[activation]
32982	GSK3B	ZFPM1	[activation]
32983	NRIP1	MAP3K7	[activation]
32984	TSACC	FOXP2	[activation]

32985 H2AFX EGFR [activation]
32986 XCL1 KRTAP10-11 [activation]
32987 IGHM BTK [activation]
32988 HDAC3 STAT3 [activation]
32989 ITGA4 HIST1H1C [activation]
32990 PPP2CA ERBB2 [activation]
32991 CDH1 HDAC1 [activation]
32992 DUSP10 FHL2 [inhibition]
32993 EHMT2 MX2 [activation]
32994 CREBBP BCL6 [activation]
32995 PRKCA PPARA [activation]
32996 BTRC NUMB [activation]
32997 MAPK10 SH3BP5 [activation]
32998 ITCH UBE2E2 [activation]
32999 EEF2K CDK1 [activation]
33000 HSP90AB1 CTNNB1 [activation]
33001 LYZL1 XAGE3 [activation]
33002 RHPN1 CNKSR1 [activation]
33003 TRAF6 APEX1 [activation]
33004 ELK1 EMX1 [activation]
33005 ADRBK1 PDGFRB [activation]
33006 NOTCH1 SMARCC1 [activation]
33007 BCAR3 ANXA2 [activation]
33008 HIST1H1A LIMK1 [activation;inhibition]
33009 ADAM17 TGFA [activation]
33010 DPYSL3 LRRK2 [activation]
33011 SIRT6 PRKDC [activation]
33012 CDC7 CDKN1A [activation]
33013 CRK DLX4 [activation]
33014 RPA3 RAD21 [activation]
33015 ITGA4 CAPZA1 [activation]
33016 OPRK1 GPRASP1 [activation]
33017 TFRC HFE [activation]
33018 CTNNB1 ASH2L [activation]
33019 EGFR MYO1B [activation]
33020 RELA RIOK2 [activation]
33021 METTL2A MAPK6 [activation]
33022 PAK1 ACVR1 [activation]
33023 EPPK1 EGFR [activation]
33024 ICAM1 RPS23 [activation]
33025 HSPA2 PARP2 [activation]
33026 HIST2H2AC BARD1 [activation]
33027 SH3KBP1 KATNB1 [activation]
33028 ITGA4 EIF2S1 [activation]
33029 JUN BATF2 [activation]
33030 MAP3K5 PPP3R1 [inhibition]
33031 HSP90AA1 GRK4 [activation]
33032 NOP2 MCM7 [activation]
33033 APP SMAD1 [activation]
33034 IRS1 MAPK9 [activation]
33035 ANXA2 RPA3 [activation]
33036 PTK2B MYD88 [activation]
33037 FKBPL RAF1 [activation]
33038 MOB4 LATS1 [inhibition]
33039 HIST1H2AA EGFR [activation]
33040 SRPK1 APEX1 [activation]
33041 WNK1 YWHAB [activation]
33042 BCL6 SENP7 [activation]
33043 AP1M2 PVR [activation]
33044 CASP9 CASP10 [activation]
33045 S100A1 TP53 [activation]
33046 RPA2 PRIM2 [activation]
33047 VCAM1 RCC2 [activation]
33048 TAGLN2 FTSJ1 [activation]
33049 DDX21 GABARAPL2 [activation]
33050 OCEL1 SRPK2 [activation]
33051 MIF MAP1LC3B [activation]
33052 NMI STAT6 [activation]
33053 PRNP TMEM237 [activation]
33054 SPP1 STAU1 [activation]
33055 SFN GAB2 [activation]
33056 HEATR1 ITGA4 [activation]
33057 APC SFN [activation]
33058 RB1 TFDP1 [inhibition]
33059 DSG4 GABARAPL2 [activation]
33060 BUB1 SHC1 [activation]

33061	SRBD1	RPA1	[activation]
33062	YWHAB	ADAM22	[activation]
33063	PGR	NCOA1	[activation]
33064	TPT1	HSPB1	[activation]
33065	AXIN2	PLK1	[activation]
33066	PRLR	PPIA	[inhibition]
33067	TIMP2	RANBP1	[activation]
33068	GADD45A	JMJD1C	[activation]
33069	VRK2	NFATC2	[activation]
33070	TRAF6	ERLIN1	[activation]
33071	ACTG1	NFKBIB	[activation]
33072	EXOC3	APP	[activation]
33073	A2M	RHEB	[inhibition]
33074	WDYHV1	ACTG1	[activation]
33075	ARRB2	PPP1R12A	[activation]
33076	CEP78	HSPB1	[activation]
33077	RAB13	RPA1	[activation]
33078	PELI3	TRAF6	[activation]
33079	JAK2	TSHR	[activation]
33080	NOTCH1	USP12	[activation]
33081	TRAF3	PPP3R1	[activation]
33082	CAMKK1	YWHAQ	[activation]
33083	ABI3	WASF2	[activation]
33084	HSPA4	TP53	[activation]
33085	CAPN10	EFNB1	[activation]
33086	SCAMP5	STX8	[activation]
33087	PTPN11	LEPR	[activation]
33088	HSP90AA1	RPS6KA6	[activation]
33089	OSGIN1	AKT1	[activation]
33090	SSH1	YWHAZ	[activation]
33091	NUP62	NUPL1	[activation]
33092	HMGB1	PGR	[activation]
33093	NEDD1	CAPNS1	[activation]
33094	STAT3	JUN	[activation]
33095	MTNR1A	GNA13	[activation]
33096	GABARAP	CDC5L	[activation]
33097	AKT1	STK11	[activation]
33098	QRICH2	SNAI1	[activation;inhibition]
33099	TP53	CCNH	[activation]
33100	ZAP70	ABL1	[activation]
33101	GSK3B	GBP2	[inhibition]
33102	INSIG2	ENO1	[activation]
33103	TNIK	DPYSL3	[activation]
33104	ZFP36	MAP3K4	[activation]
33105	CTNNA1	CDH15	[activation]
33106	CASP3	ACIN1	[activation]
33107	WASF1	LRRK2	[activation]
33108	RPA1	KCNAB2	[activation]
33109	DOK2	LYN	[activation]
33110	AMOT	INADL	[activation]
33111	PIK3R1	THRB	[activation]
33112	CDK1	HSP90AA1	[activation]
33113	CAV1	GNAI2	[activation]
33114	MAPK8	SMAD2	[activation]
33115	HGF	SDC1	[activation]
33116	CNTN4	BARD1	[activation]
33117	CDH7	CDH15	[activation]
33118	PARP1	ETS1	[activation]
33119	L1CAM	CNTN1	[activation]
33120	BCL6	ZBTB7A	[activation]
33121	PXN	XPO1	[activation]
33122	ASGR2	F8	[activation]
33123	COMMD1	ATP7A	[activation]
33124	SFRP4	PTPRJ	[activation]
33125	MAB21L3	STAC3	[activation]
33126	RALGDS	RAPGEF2	[activation]
33127	MAPKAPK5	ZNF576	[activation]
33128	RPS28	MDM2	[activation]
33129	ANAPC7	CDC5L	[activation]
33130	FAS	TNFRSF1A	[activation]
33131	PTPRU	CTNNB1	[activation;inhibition]
33132	NXF1	RAB5B	[activation]
33133	RIOK3	CDC42	[activation]
33134	YWHAH	WDR20	[activation]
33135	ZMYND11	SRPK1	[activation]
33136	NXF1	PRKACA	[activation]

33137	AR	CASP1	[activation]
33138	STAT1	HSP90AB1	[activation]
33139	GSTK1	EGFR	[activation]
33140	GP6	CRKL	[activation]
33141	NR1H3	ING3	[activation]
33142	PTBP3	COL1A1	[activation]
33143	TWIST1	HDAC2	[activation]
33144	PLXNB3	BTBD3	[activation]
33145	VCAM1	GART	[activation]
33146	FURIN	DDX56	[activation]
33147	STAT3	RELA	[activation]
33148	CBL	HTR2A	[activation]
33149	NSMCE4A	SRPK1	[activation]
33150	CDC25A	NXF1	[activation]
33151	CHEK2	PLK1	[activation]
33152	KCNA4	SRC	[activation]
33153	KIAA1377	TNFSF11	[activation]
33154	MAPKAP1	RICTOR	[activation]
33155	USF1	PARP1	[activation]
33156	ZNF259	CDK5RAP2	[activation]
33157	ITGAX	FCER2	[activation]
33158	OPRD1	GJA4	[activation]
33159	STX5	VAPB	[activation]
33160	CREB3	RAB23	[activation]
33161	CASP12	HSPA5	[inhibition]
33162	SMARCAL1	RPA1	[activation]
33163	PAX6	SUMO1	[activation]
33164	PRKCI	HSP90AA1	[activation]
33165	NEDD1	CRTC3	[activation]
33166	TNFRSF1A	MYL12A	[activation]
33167	HDAC1	PRKACA	[activation]
33168	PHC2	MAPKAPK3	[activation]
33169	USP7	PPARG	[activation]
33170	GRM6	GNAO1	[activation]
33171	SGK1	LETM1	[activation]
33172	CDK5R1	SET	[activation]
33173	EGFR	CARS2	[activation]
33174	PRKACA	RELA	[activation]
33175	ARHGAP1	WAS	[activation]
33176	PSEN1	FGF13	[activation]
33177	TRAP1	HLA-B	[activation]
33178	ADORA1	GNAI2	[activation;inhibition]
33179	PAK2	P4HB	[activation]
33180	TCF12	MYF6	[activation]
33181	PTPN6	NOS1	[activation;inhibition]
33182	MYF5	CSNK2A1	[activation]
33183	RXRA	PPARD	[inhibition]
33184	EP300	ANXA2	[activation]
33185	CD4	CD82	[activation]
33186	PPP2R1A	CDK4	[inhibition]
33187	PRKDC	MAPK9	[activation]
33188	TTC23	BHLHE40	[activation]
33189	TTC23	PNMA5	[inhibition]
33190	ICT1	VARS2	[activation]
33191	PDIA6	MTNR1B	[activation]
33192	WASL	PFN1	[activation]
33193	RGS2	MON1A	[activation;inhibition]
33194	ST5	SFN	[activation]
33195	CAV1	AXIN1	[activation]
33196	ACTG1	WIPF1	[activation]
33197	HSPA2	CDK1	[activation]
33198	PARK2	BCL2L1	[activation]
33199	YWHAZ	MTOR	[activation]
33200	MAPKAPK5	MAPKAPK2	[activation]
33201	EIF2S1	PRKCD	[activation]
33202	SH3BGRL3	EGFR	[activation]
33203	OBSL1	RPS6	[activation]
33204	CSNK1A1	IKBKB	[activation]
33205	SOCS3	PIN1	[inhibition]
33206	ANAPC5	SMURF1	[inhibition]
33207	NFE2L2	KPNA3	[activation]
33208	MOS	KRT40	[activation]
33209	NR3C1	ZNF496	[activation]
33210	GRIP2	GRIA2	[activation]
33211	PIK3R1	PIK3AP1	[activation]
33212	PPP2CA	KPNB1	[inhibition]

33213	ACTG1	GSN	[activation]
33214	MYC	RBFA	[activation]
33215	U2AF1	SRPK1	[activation]
33216	VEGFA	TNXB	[activation]
33217	EDA	SEC22A	[activation]
33218	YWHAG	HSPA1A	[inhibition]
33219	PGR	TRIM24	[activation]
33220	TRAF6	ERN1	[activation]
33221	EIF4E	SMYD2	[inhibition]
33222	MME	CMSS1	[activation]
33223	EPAS1	STAT5A	[activation]
33224	KIR2DL1	PTPN6	[activation;inhibition]
33225	MKNK2	ESR1	[activation]
33226	CCR1	TPST1	[activation]
33227	E2F1	CDH1	[activation]
33228	TBRG4	EGFR	[activation]
33229	CAMK1D	ASAH1	[activation]
33230	RAN	SLX4	[activation]
33231	RHOF	NXF1	[activation]
33232	BRK1	NLGN1	[activation]
33233	TERF1	SMAD9	[activation]
33234	APP	SMARCB1	[activation]
33235	MDM2	RHOA	[activation]
33236	CASP2	RB1	[activation]
33237	MET	GLMN	[activation]
33238	HIPK3	TGFB1I1	[activation]
33239	NCOA2	ESR1	[activation]
33240	LRP6	ANTXR2	[activation]
33241	FCGR2A	ITGA4	[activation]
33242	FASTKD5	IRAK2	[activation]
33243	CASC3	EIF4A3	[activation]
33244	PREX1	RPTOR	[activation]
33245	UBE2I	AURKA	[activation]
33246	MDM2	ETFA	[activation]
33247	YES1	FASLG	[activation]
33248	SMARCA5	SNW1	[activation]
33249	GPR25	CCDC155	[activation]
33250	CALM1	ATRX	[activation]
33251	SUMO1	ENO3	[activation]
33252	POLR2A	CCNH	[activation]
33253	RAC2	AFG3L2	[activation]
33254	CASP9	GZMB	[activation]
33255	IKBKE	SMC4	[activation]
33256	PTPN1	TRPV6	[activation]
33257	SMAD2	LAMA5	[activation;inhibition]
33258	STK3	GBP2	[activation]
33259	PIK3R1	FOXH1	[inhibition]
33260	MRPS23	SMURF1	[inhibition]
33261	RHEB	CREB3	[activation]
33262	CBL	YWHAZ	[activation]
33263	GNA11	PTGIR	[activation]
33264	LSR	SFN	[activation]
33265	FZR1	NINL	[inhibition]
33266	MAPK3	TNFSF11	[activation]
33267	PCBP1	ATF2	[activation]
33268	YWHAH	HDAC4	[activation]
33269	SELPLG	SYK	[activation]
33270	DNAJA1	OSGEP	[inhibition]
33271	ARRB2	DRD4	[activation]
33272	CDK17	YWHAZ	[activation]
33273	KCTD15	NOTCH2NL	[activation]
33274	FRAT2	CDKN2A	[inhibition]
33275	GHR	SKP1	[inhibition]
33276	MAPK3	HIF1A	[activation]
33277	FLT4	IKBKG	[activation]
33278	STK3	RAF1	[activation]
33279	ILK	RNF19A	[activation]
33280	FAS	RIPK1	[activation]
33281	KNG1	AGT	[activation]
33282	NR3C1	POU1F1	[activation]
33283	GNA12	PPP5C	[activation]
33284	SIRT1	HES1	[activation]
33285	MARK3	FOS	[activation]
33286	IFNAR1	STAT3	[activation]
33287	NRXN1	MLLT4	[activation]
33288	RECQL5	RAD50	[activation]

33289 RFC1 MLH1 [activation]
33290 STAT3 CCR5 [activation]
33291 HNRNPA1 SUFU [activation]
33292 SH3KBP1 RPA1 [activation]
33293 IRF1 SUMO1 [activation]
33294 ADRA2B YWHAZ [activation]
33295 HSPA4 MET [inhibition]
33296 SYN1 MAPK3 [activation]
33297 NFKBIA HSPA1L [activation]
33298 CLNK LCP2 [activation]
33299 CYTH2 MYD88 [activation]
33300 ISG15 FAM120C [activation;inhibition]
33301 PPP2R1A PDCD10 [inhibition]
33302 SPG20 APPL1 [activation]
33303 PRKCA JAK1 [activation]
33304 F2 GP1BA [activation]
33305 CSNK2A1 ATF2 [activation]
33306 EIF1B NUDC [activation]
33307 AMOT ACTB [activation]
33308 HEMGN SRC [activation]
33309 CFL1 RPS6KA1 [activation;inhibition]
33310 HIST3H3 MGA [activation]
33311 PPP1R15A POU3F3 [inhibition]
33312 ENKD1 TRAF2 [activation]
33313 LYN FRS2 [activation]
33314 PRKCZ C1QBP [activation]
33315 TP53 TOE1 [activation]
33316 CYCS ESR2 [activation]
33317 LRRN4CL SRPK2 [activation]
33318 KCTD17 TP53 [activation]
33319 NFE2L2 JUND [activation]
33320 SETDB1 ERG [activation]
33321 ARAP1 SNW1 [activation]
33322 OTX1 LCE2D [activation]
33323 SIRT3 GRN [activation]
33324 GORASP2 DPYSL2 [activation]
33325 UPF3A IFITM2 [activation]
33326 FOXO1 THRB [activation]
33327 PRKDC OBSL1 [activation]
33328 ARHGEF12 ARRB2 [activation]
33329 TP53 ASF1A [activation]
33330 APP GAK [inhibition]
33331 EHMT2 CCDC33 [activation]
33332 STK4 PIH1D1 [activation]
33333 CLEC11A MYC [activation]
33334 RTKN2 PACSIN1 [activation]
33335 SPI1 GATA3 [inhibition]
33336 ANK1 OBSCN [activation]
33337 HSP90AB1 SLFN11 [activation]
33338 PTPRU GNAI3 [activation;inhibition]
33339 SMURF2 EPHA1 [activation]
33340 JUN MAP2K7 [activation]
33341 MAPK14 YY1AP1 [activation]
33342 TGFB1 WFIKKN2 [inhibition]
33343 H3F3A MAPK9 [activation]
33344 GAL GALR1 [activation]
33345 ACTB CDKN2A [activation]
33346 SLC1A5 EGFR [activation]
33347 TSC22D1 RGS1 [activation;inhibition]
33348 SRC DOK4 [activation]
33349 SUMO3 SMARCA1 [activation]
33350 CAPN5 MLH1 [activation]
33351 MYO16 CYFIP1 [inhibition]
33352 DIRAS1 APP [activation]
33353 HSPB2 HSPB3 [activation]
33354 FYN CD2 [activation]
33355 DPP4 PTPRC [activation]
33356 BAX MAP2K1 [activation]
33357 LUC7L2 CDK5RAP2 [activation]
33358 ANAPC13 ANAPC16 [activation]
33359 MYC XDH [activation]
33360 SHC1 DDX5 [activation]
33361 TNC EGFR [activation]
33362 FAM98B NXF1 [activation]
33363 SFRP2 BMPR1A [activation;inhibition]
33364 RALGDS RAP1GAP [activation]

33365	ABI1	GRB10	[activation]
33366	ENO2	SUMO4	[activation]
33367	HMGB1	TP53	[activation]
33368	TRAF2	IL15RA	[activation]
33369	PLK1	PLK2	[activation]
33370	NR4A1	TERF1	[activation]
33371	CD38	LCK	[activation]
33372	P2RX3	P2RX2	[activation]
33373	NOTCH1	ERH	[activation]
33374	TWF2	LYAR	[activation]
33375	KRT17	APC	[inhibition]
33376	IGF1R	YWHAZ	[activation]
33377	CALM1	CCND1	[activation]
33378	MAPK14	FKBP8	[activation]
33379	KCNN2	CALM1	[activation]
33380	MTIF2	NFKBIB	[activation]
33381	WHSC1	PBRM1	[activation]
33382	CCL5	WT1	[activation]
33383	SRC	SP1	[activation]
33384	TP53	HSPA1L	[activation]
33385	PECAM1	JUP	[activation]
33386	GSN	CASP4	[activation]
33387	BLK	PLCG2	[activation]
33388	ATF4	GOLGA8DP	[activation]
33389	MAP2	JUNB	[activation]
33390	RELA	SIRT6	[activation]
33391	FOXO3	H2AFX	[inhibition]
33392	CSF2	MEOX2	[activation]
33393	EIF4A1	SUMO3	[activation]
33394	MYC	P4HA1	[activation]
33395	AURKB	CCDC8	[activation]
33396	RORC	ARNTL2	[inhibition]
33397	NCL	ITGA4	[activation]
33398	YWHAZ	PARD3	[activation]
33399	IGHA1	IGLC1	[activation]
33400	NCAM1	PLCG1	[activation]
33401	STAT3	IL6ST	[activation]
33402	HIST1H1A	CCNB1	[activation]
33403	IL4	CD53	[activation]
33404	FOXRED2	PRKDC	[activation]
33405	PHLDA3	MCM5	[activation]
33406	PLCG2	GAB1	[activation]
33407	RELA	SMAD4	[activation]
33408	CASP4	NOD1	[activation]
33409	MLH1	PCNA	[activation]
33410	STAT5B	CRKL	[activation]
33411	CALM1	ESR2	[activation]
33412	PAK1	ARHGEF7	[activation]
33413	CAMK2N2	CAMK2B	[inhibition]
33414	UNG	SEZ6L2	[activation]
33415	CSNK1E	PPP1CA	[activation;inhibition]
33416	SIT1	SYK	[activation]
33417	ANK1	NBPF3	[activation]
33418	TNFRSF10A	TNFRSF10B	[activation]
33419	EPHA7	EPHA2	[activation]
33420	SP1	FHL2	[activation]
33421	YAE1D1	GABARAPL2	[activation]
33422	GRIN2B	PIK3CA	[activation]
33423	CCDC136	STK11	[activation]
33424	DAPK1	LRRK1	[activation]
33425	CASP3	CTTN	[activation]
33426	RPA1	NTPCR	[activation]
33427	PTCH2	IHH	[activation]
33428	STK4	STRN4	[activation]
33429	PLEKHM1	BID	[activation]
33430	CD2	EP300	[activation]
33431	DOCK8	DOCK7	[activation]
33432	FANCA	FYN	[activation]
33433	UNC93B1	NXF1	[activation]
33434	SRPK1	MMTAG2	[activation]
33435	FOXP3	HIST1H1B	[activation]
33436	FOS	AGTPBP1	[activation]
33437	DDX24	GIT1	[activation]
33438	CAV3	PDGFRA	[activation]
33439	EDN1	BAG6	[activation]
33440	TRIM38	RIPK1	[activation]

33441	APP	CMTM3	[activation]
33442	USP42	RAD50	[activation]
33443	RASSF3	VAPB	[activation]
33444	MYO1C	MAP3K3	[activation]
33445	TRAF6	MSR1	[activation]
33446	OLFM4	C3	[activation]
33447	CAMK2B	MRPL11	[inhibition]
33448	YWHAG	KCNK15	[activation]
33449	SH3BGR	CAPN1	[activation]
33450	TRAF6	RANGAP1	[activation]
33451	CIAO1	HSPB1	[activation]
33452	STAT1	TP53BP1	[activation]
33453	VIM	PAK2	[activation]
33454	FGFR2	STAT3	[activation]
33455	PRKCA	HIST1H1E	[activation]
33456	TEX11	CDKN1A	[activation;inhibition]
33457	WNT4	PTEN	[activation;inhibition]
33458	KATNA1	LIPE	[activation]
33459	IGHG1	TGM4	[activation]
33460	ABL1	ERBB2	[activation]
33461	ZNF106	APC	[inhibition]
33462	GTPBP2	PRMT5	[activation]
33463	BDNF	NCAM1	[activation]
33464	STX17	CTNNA1	[activation]
33465	ANXA7	GNB5	[activation]
33466	PTCD3	IFITM2	[activation]
33467	TRAF2	ARNT2	[activation]
33468	KCNAB2	KCNA2	[activation]
33469	CEBPB	ATF3	[activation]
33470	MAPKAPK3	BUB1	[activation]
33471	ATM	TFF1	[activation]
33472	CXADR	AMICA1	[activation]
33473	NR3C1	RAD54L2	[activation]
33474	PARP1	MYB	[activation]
33475	SKI	NLK	[inhibition]
33476	PAXIP1	C2orf42	[activation]
33477	RGS19	MAPK6	[activation;inhibition]
33478	GRIA4	ACTN1	[activation]
33479	TJP2	SRPK2	[activation]
33480	CCER1	NOTCH2NL	[activation]
33481	CCDC57	PER1	[inhibition]
33482	EGFR	PHLDA1	[activation]
33483	EXTL3	FYN	[activation]
33484	F8	VWF	[activation]
33485	LCP2	WAS	[activation]
33486	TNFSF10	ACP5	[activation]
33487	DLGAP2	GRB2	[activation]
33488	PCSK2	IAPP	[activation]
33489	KAT2B	IRF7	[activation]
33490	HSPG2	FGF1	[activation]
33491	UBE2N	F12	[activation]
33492	CACNB3	FASLG	[inhibition]
33493	LRCH2	RASSF10	[inhibition]
33494	INSIG2	DSP	[activation]
33495	GAK	CREBBP	[activation]
33496	ATM	STK11	[activation]
33497	SOX3	PAX6	[activation]
33498	KAT2B	CEBPB	[activation]
33499	LPL	PLK1	[activation]
33500	PPARG	GADD45B	[activation]
33501	CSMD2	MYC	[activation]
33502	HSPA8	PPP1CA	[inhibition]
33503	EIF4A1	MAPK13	[activation]
33504	MYC	LCN12	[activation]
33505	BEND3	RIOK2	[activation]
33506	CCND1	KLK9	[activation]
33507	TNFSF11	MAPK1	[activation]
33508	TNIK	RANBP1	[activation]
33509	ARHGEF5	EGFR	[activation]
33510	VCL	ABI2	[activation]
33511	RPL12	ICAM1	[activation]
33512	NCF1	CAV1	[activation]
33513	NEIL2	EP300	[activation]
33514	LHX2	MSX1	[activation]
33515	LLGL2	PRKCI	[activation]
33516	ATP13A2	VAMP2	[activation]

33517	ATP13A2	ACKR3	[activation]
33518	SPHK1	TRAF6	[activation]
33519	PLK1	SRPX	[activation]
33520	SMAD1	EP300	[activation]
33521	LTA	TUBA4A	[activation]
33522	KAT2A	ESR1	[activation]
33523	RAD9A	CHEK2	[activation]
33524	HNRNPL	ITGA4	[activation]
33525	NFE2	YAP1	[activation]
33526	PML	RBL2	[inhibition]
33527	PDGFB	PDAP1	[activation]
33528	IRS1	PRKCZ	[activation]
33529	BHLHE40	NFIL3	[activation]
33530	SRP54	STK4	[activation]
33531	BARD1	KRI1	[activation]
33532	VCAM1	CCL17	[activation]
33533	CRK	FLNC	[activation]
33534	MMP9	RECK	[inhibition]
33535	RASA1	SYN1	[activation]
33536	NDRG1	HSD17B4	[activation]
33537	TP53	MVP	[activation]
33538	NCF1	GNAI2	[activation]
33539	PLXNA1	FBXO6	[activation]
33540	RPL5	SPP1	[activation]
33541	EGFR	YWHAQ	[activation]
33542	UBQLN1	CCL7	[activation]
33543	SMAD3	RUNX1	[activation]
33544	QRICH2	TSSC1	[activation]
33545	CTNNB1	PSEN2	[activation]
33546	PADI1	TP53	[activation]
33547	EP300	ESR2	[activation]
33548	RAC1	IL1RAP	[activation]
33549	ETS1	ELP2	[activation]
33550	PRKRA	IGF2BP1	[activation]
33551	TP53	PTCD3	[activation]
33552	CDH2	PTPRG	[activation]
33553	SUMO1	TRAF6	[activation]
33554	EEA1	ERBB2	[activation]
33555	ESR1	ISL1	[activation]
33556	OSM	COL4A6	[activation]
33557	TNFSF10	SOCS3	[activation;inhibition]
33558	SMAD3	CREBBP	[activation]
33559	NXF1	DCTPP1	[activation]
33560	STAT3	BICD1	[activation]
33561	HNF4A	ESR1	[activation]
33562	HSPA4	BCR	[activation]
33563	RADIL	CCNH	[activation]
33564	AGAP7	HSPB1	[activation]
33565	CDKN3	CDC25A	[activation]
33566	H1F0	CCNE1	[activation]
33567	RB1	TP73	[activation;inhibition]
33568	DAPP1	MAP4K1	[activation]
33569	NR4A1	SLC25A4	[inhibition]
33570	GNAQ	BTK	[activation]
33571	IGHG1	IGLC1	[activation]
33572	SMURF1	ARHGAP15	[inhibition]
33573	SLX1A	CALM1	[activation]
33574	PTPRC	GANAB	[activation]
33575	EIF2AK2	EGFR	[activation]
33576	PAXIP1	KMT2D	[activation]
33577	PECAM1	ITGB3	[activation]
33578	GMCL1	STK3	[activation]
33579	CCDC88C	DVL1	[activation]
33580	MLST8	ULK1	[inhibition]
33581	TCOF1	SRPK2	[activation]
33582	OGT	THAP3	[activation]
33583	GRB2	DRD3	[activation]
33584	AXIN1	WWTR1	[activation]
33585	VAPB	INSIG1	[activation]
33586	ELAVL1	SERPINE2	[inhibition]
33587	RAF1	PRKG1	[activation]
33588	ENAH	ABI3	[activation]
33589	TYK2	PTAFR	[activation]
33590	SRPK2	DAW1	[activation]
33591	NDUFB10	HTR2A	[activation]
33592	PASK	PKIA	[inhibition]

33593	YWHAB	EGFR	[activation]
33594	RAF1	MYC	[activation]
33595	AR	RASA1	[activation]
33596	COL4A5	ANXA7	[activation]
33597	MAP2K4	MAP3K10	[activation]
33598	CGA	PTN	[activation]
33599	TTC39C	NXF1	[activation]
33600	NR3C1	OSGEP	[activation]
33601	DSG1	PPP2R2A	[activation]
33602	SMAD4	PRKAR1B	[activation;inhibition]
33603	CABIN1	PPP3CB	[inhibition]
33604	ACIN1	LRRK2	[activation]
33605	MCM6	HLA-B	[activation]
33606	SRC	ABI1	[activation]
33607	PXN	ABL1	[activation]
33608	LYPLA1	ITGA4	[activation]
33609	NUDC	PEX7	[activation]
33610	ZAK	CCDC69	[activation]
33611	FADD	LRRK2	[activation]
33612	PRKACA	CAD	[activation;inhibition]
33613	RIPK1	PIDD	[activation]
33614	SRC	KCND3	[activation]
33615	SRC	CCNA2	[activation]
33616	PDE6G	GNAO1	[activation;inhibition]
33617	CMYA5	SIRT1	[activation]
33618	KCNA5	UBE2I	[activation]
33619	IFIT5	SRPK2	[activation]
33620	KLHDC1	NUDC	[activation]
33621	TPX2	GRB2	[activation]
33622	MAGT1	NXF1	[activation]
33623	YWHAB	ALS2	[activation]
33624	MAPK6	EIF2S3	[activation]
33625	SOCS1	TRIM8	[inhibition]
33626	TGFB1	FSTL1	[activation]
33627	DLG4	FZD1	[activation]
33628	ARRB1	MAPK3	[activation]
33629	CBLB	NCK2	[inhibition]
33630	ABI1	EPS8L2	[activation]
33631	SNAP25	ITSN1	[activation]
33632	PPP2R3A	EGFR	[activation]
33633	ATR	HSP90AA1	[activation]
33634	MAPK9	CASP3	[activation]
33635	PTPN11	PLCG1	[activation]
33636	HRAS	IKZF3	[activation]
33637	ICT1	PARS2	[activation]
33638	PIK3R1	MST1R	[activation]
33639	ARHGEF7	MCM7	[activation]
33640	HSPB3	TCAP	[inhibition]
33641	LAT	SPRY1	[inhibition]
33642	RCVRN	GRK6	[inhibition]
33643	NUCB2	CASP6	[activation]
33644	CD79A	LCK	[activation]
33645	TUBB4A	TRAF6	[activation]
33646	YWHAB	PAK4	[activation]
33647	CUL1	EIF4A1	[activation]
33648	BMPR2	CES3	[activation]
33649	TP53	SMAD7	[activation]
33650	NTS	SORT1	[activation]
33651	BCL2L1	VDAC1	[activation]
33652	DNAJA3	JAK2	[inhibition]
33653	PTK2B	ZAP70	[activation]
33654	TP53	CFLAR	[activation]
33655	KTN1	RAC1	[activation]
33656	FANCA	CDK1	[activation]
33657	TRAF3	IL1RAP	[activation]
33658	CDKN2D	C1orf94	[inhibition]
33659	OGDH	MDM2	[activation]
33660	FYN	KDR	[activation]
33661	DNM2	AHI1	[activation]
33662	TLE1	ATF2	[activation]
33663	MAPK3	DCC	[activation]
33664	RELA	BTK	[activation]
33665	NSUN2	ITGA4	[activation]
33666	HNF4A	SMAD4	[activation]
33667	APP	STK17B	[activation]
33668	CSNK1G2	RUNDC3B	[inhibition]

33669	FZD7	MAGI3	[activation]
33670	MYC	PLEKHH1	[activation]
33671	ATF2	CTNNB1	[activation]
33672	YWHAG	PRKCQ	[activation]
33673	VWF	RAC1	[activation]
33674	SF3B14	VCAM1	[activation]
33675	ITPR1	CDK1	[inhibition]
33676	FAS	RAP1A	[activation]
33677	CHST10	HSF1	[activation]
33678	HIST3H3	TRIM28	[activation]
33679	WAS	HCLS1	[activation]
33680	FBP2	SRC	[activation]
33681	SETD1A	EP300	[activation]
33682	NME1	TIAM1	[activation]
33683	PLK1	SPAG9	[inhibition]
33684	DLL4	NOTCH4	[activation]
33685	PLG	TFPI2	[inhibition]
33686	HIF1AN	PPP1R12A	[activation]
33687	GRB2	ASB16	[activation]
33688	ANXA2	MYC	[activation]
33689	PTK2B	SOCS2	[activation]
33690	RSRC1	ACTR3B	[activation]
33691	CHUK	PRKDC	[activation]
33692	HIST3H3	APLF	[activation]
33693	GRIA3	GRIA2	[activation]
33694	VCAM1	MCM4	[activation]
33695	PRKCA	DDX5	[activation]
33696	MYC	DDX17	[activation]
33697	IGF1R	SOCS3	[inhibition]
33698	RIPK1	TAX1BP1	[inhibition]
33699	ARFIP2	ARF6	[activation]
33700	KIT	RASA1	[activation]
33701	BORA	AURKA	[activation]
33702	BCAR1	YWHAH	[activation]
33703	APP	TSLP	[activation]
33704	ARF3	MAPK13	[activation]
33705	SCAMP1	NXF1	[activation]
33706	RPS6KB1	HSP90AB1	[activation]
33707	NUP62	CCDC150	[activation]
33708	PTTG1	DNAJA1	[inhibition]
33709	ADCY2	GNAS	[activation]
33710	RASA1	ATF7	[activation]
33711	RNF135	DDX58	[inhibition]
33712	EGFR	ALCAM	[activation]
33713	BTRC	KDR	[activation]
33714	RB1	USP7	[inhibition]
33715	TICAM2	IRAK3	[inhibition]
33716	GRB2	SMARCD2	[activation]
33717	CCNE1	CREBBP	[activation]
33718	CAPNS1	CDK4	[activation]
33719	DOK1	NCK1	[activation]
33720	KPNA1	RANBP2	[activation]
33721	TROVE2	LRRK2	[activation]
33722	FAM47B	LRRK2	[activation]
33723	SHC3	APP	[activation]
33724	WNK1	TSC22D1	[activation]
33725	ATF4	NFE2L2	[activation]
33726	DDX54	APP	[activation]
33727	RASSF10	SERPINB3	[inhibition]
33728	MAPRE2	CDK4	[inhibition]
33729	ADAM12	GRB2	[activation]
33730	ELK1	CREBBP	[activation]
33731	RASA1	ZNF579	[activation]
33732	PARD6G	HSPA4	[activation]
33733	TGFB1I1	NR3C1	[activation]
33734	EXOC1	EXOC4	[activation]
33735	CCDC8	RPA1	[activation]
33736	ARNTL	RXRA	[inhibition]
33737	HOXC10	YWHAG	[activation]
33738	CFTR	DCLK1	[activation]
33739	EHMT2	HIST1H1A	[activation]
33740	VAPA	OPRM1	[activation]
33741	MAP3K8	CHUK	[activation]
33742	HSP90AB1	PRKCI	[activation]
33743	ATF2	TPI1	[activation]
33744	EP300	POU3F2	[activation]

33745	EZH2	RELA	[activation]
33746	ATP1A1	ADRB2	[activation]
33747	UBE2K	DDX58	[activation]
33748	TBK1	CCDC8	[inhibition]
33749	POLR2E	SRPK1	[activation]
33750	IRAK2	HRAS	[activation]
33751	CBLB	FLT3	[inhibition]
33752	EP300	FOXP3	[activation]
33753	BMPR2	PYCARD	[activation]
33754	NFATC2	CREBBP	[activation]
33755	TIMP2	APC2	[activation]
33756	ASB14	SLC25A6	[activation]
33757	RHOA	HLA-B	[activation]
33758	TPP1	POT1	[activation]
33759	HSPA4L	TRAF6	[activation]
33760	SMAD1	TTF1	[activation]
33761	PPIA	GRB2	[activation]
33762	APP	CNDP1	[activation]
33763	SRPK1	C6orf201	[activation]
33764	BCL2L1	UBR1	[activation]
33765	PAX6	DYNLL1	[activation]
33766	INSR	FRS2	[activation]
33767	MYLPF	EZH2	[activation]
33768	HNRNPL	HIST3H3	[activation]
33769	SRF	CASP7	[activation]
33770	CUL1	GLMN	[activation]
33771	MRPS25	TP53	[activation]
33772	PCSK6	RXRB	[inhibition]
33773	RXRA	CTNNB1	[inhibition]
33774	CASP3	EIF2AK2	[activation]
33775	MET	RASA1	[activation]
33776	BTRC	IFNAR1	[activation]
33777	TIMM50	ILK	[activation]
33778	PPP2R1A	PPP2R3B	[activation]
33779	IKBKB	PRKCD	[activation]
33780	HTT	PRNP	[activation]
33781	NCL	NBN	[activation]
33782	RHOJ	ACVR1	[activation]
33783	EGFR	LSR	[activation]
33784	PPP2CA	RBL2	[inhibition]
33785	TNF	IKBKG	[activation]
33786	ABL1	DLGAP3	[activation]
33787	DVL3	NKD1	[activation]
33788	MAP1LC3C	ULK1	[inhibition]
33789	BAG6	FAS	[inhibition]
33790	IGHA1	CDK2AP1	[activation]
33791	CASP7	CRYAB	[activation]
33792	EFNA3	PRSS23	[activation]
33793	PPP2R1A	MAP3K3	[activation]
33794	BIRC2	CDC42	[activation;inhibition]
33795	GNPTAB	APP	[activation]
33796	SRC	CEACAM1	[activation]
33797	CHGB	SLC25A6	[activation]
33798	MYC	EFNB1	[activation]
33799	LCN2	MMP2	[inhibition]
33800	MAP2K3	SMAD7	[activation]
33801	CCL21	CXCL13	[activation]
33802	PGAM5	STRN	[inhibition]
33803	ARFGEF2	ARFGEF1	[activation]
33804	TTC23L	EGLN3	[activation]
33805	PRIM1	EGFR	[activation]
33806	EGFR	TRPV1	[activation]
33807	HOMER3	PAX6	[activation]
33808	ROCK1	CASP3	[activation]
33809	GNL3	PPP2R5A	[activation]
33810	GRB2	ACTB	[activation]
33811	GATA6	CDK9	[activation]
33812	YWHAG	MARK3	[activation]
33813	FSCN1	TRAF6	[activation]
33814	APP	MAST1	[activation]
33815	NKIRAS1	XRCC2	[activation]
33816	SP100	LRRK2	[activation]
33817	CD3D	CD8B	[activation]
33818	ITCH	SGK3	[activation]
33819	DAB1	FAM168A	[activation]
33820	ZDHHC17	PDPK1	[activation]

33821	FGFR3	POLA2	[activation]
33822	MIIP	PPP1CA	[activation;inhibition]
33823	PIK3CA	SLC9A3R1	[activation]
33824	CD40	PIK3R1	[activation]
33825	CSRP1	GRB2	[activation]
33826	PIK3R2	SOCS1	[activation]
33827	MET	SYK	[activation;inhibition]
33828	EVL	BAIAP2	[activation]
33829	MYB	BTRC	[activation]
33830	ZFYVE9	SCYL2	[activation]
33831	TP53	RAE1	[activation]
33832	EIF2S3	ELAVL1	[activation]
33833	CDC5L	POLD1	[activation]
33834	IRF2	HCFC2	[activation]
33835	HAP1	CDK5RAP2	[activation]
33836	FGR	FASLG	[activation]
33837	STK4	ANXA1	[activation]
33838	SMAD1	NOTCH2	[activation]
33839	HTR1A	RHOA	[activation]
33840	TNFRSF1A	PRKCD	[activation]
33841	H3F3A	LRRK2	[activation]
33842	IKBKE	MAD2L1	[inhibition]
33843	BMX	PAK1	[activation]
33844	SMAD2	MAP2K1	[activation]
33845	MYOM1	DYSF	[activation]
33846	CTNNA1	APP	[activation]
33847	BID	CAV1	[activation]
33848	SMAD2	TSC2	[activation;inhibition]
33849	MAPK1	HSF4	[inhibition]
33850	IRF2	STAT1	[activation]
33851	GRK1	SNCG	[inhibition]
33852	TMEM66	STIM1	[activation]
33853	SMARCB1	BCL2L11	[activation]
33854	FOS	ATF7	[activation]
33855	PPP2R2B	DSG1	[activation]
33856	YWHAG	FAM53C	[activation]
33857	ROBO4	RXRA	[inhibition]
33858	TGFBR2	TGFBRAP1	[activation]
33859	GRB2	GNS	[activation]
33860	KRT40	RAC1	[activation]
33861	ARHGEF15	EPHA4	[activation]
33862	SORT1	NTRK1	[activation]
33863	CDC23	SSX2IP	[activation]
33864	NRD1	HBEGF	[activation]
33865	YWHAE	BCR	[activation]
33866	CACNA1D	FGFR2	[activation]
33867	RBM33	GRB2	[activation]
33868	STAM2	EGFR	[activation]
33869	SHC1	CDC5L	[activation]
33870	FGF10	POMC	[activation]
33871	MAPK8	PIAS2	[activation]
33872	SENP1	KCNK1	[activation]
33873	MAPK14	KRT18	[activation]
33874	NPY	NPY2R	[activation]
33875	NUP153	NUP62	[activation]
33876	THRB	CAMK2B	[activation]
33877	PES1	VCAM1	[activation]
33878	PRRG2	YAP1	[activation]
33879	ATR	PPP2R2A	[activation]
33880	LGI1	ADAM22	[activation]
33881	SPTBN1	CAPN1	[activation]
33882	CAPRIN1	RPA3	[activation]
33883	EFNA4	MOV10	[activation]
33884	ESR1	HIF1A	[activation]
33885	PRKAR2B	PRKACB	[activation]
33886	GCN1L1	TRAF6	[activation]
33887	ECT2	PIK3R1	[activation]
33888	APH1B	PSENEN	[activation]
33889	SEC23B	UGT1A1	[activation]
33890	ACTG1	UBL4A	[activation]
33891	RASAL2	YWHAG	[activation]
33892	PTPN11	PTPN6	[activation;inhibition]
33893	CREB1	UBE2I	[activation]
33894	C2orf73	HSPB1	[activation]
33895	NXF1	MSL1	[activation]
33896	IL2RB	LYN	[activation;inhibition]

33897	ITGB2	FCER2	[activation]
33898	KRT16	GRB2	[activation]
33899	SMC3	EGFR	[activation]
33900	ESR2	MRPS27	[activation]
33901	EIF4A2	HSPB1	[activation]
33902	VAV1	SOCS1	[inhibition]
33903	PAK2	CTTN	[activation]
33904	SRF	ATF6	[activation]
33905	ERBB4	ERBB3	[activation]
33906	TAF1B	UBTF	[activation]
33907	CSNK2A1	L1CAM	[activation]
33908	ANXA5	COL10A1	[activation]
33909	UBA5	SARS	[activation]
33910	YWHAG	SRPK1	[activation]
33911	BLM	ERCC6L	[activation]
33912	RHOJ	BMPR1B	[activation]
33913	PRPSAP2	APP	[inhibition]
33914	NFATC1	FOXP3	[activation]
33915	ANAPC4	SH3KBP1	[activation]
33916	PKIG	KPTN	[inhibition]
33917	APP	NOTCH1	[activation]
33918	SRPK2	CACNG2	[activation]
33919	TFDP2	E2F1	[inhibition]
33920	LRRK2	DFFB	[activation]
33921	APP	KCNIP4	[activation]
33922	NFKB1	ESR1	[activation]
33923	CREB3	SCP2	[activation]
33924	CD164	CXCR4	[activation]
33925	PA2G4	E2F1	[activation]
33926	CREBBP	WT1	[activation]
33927	EPB41L3	CNTNAP2	[activation]
33928	HIF1A	VHL	[inhibition]
33929	ARPC2	ARPC4	[activation]
33930	STK4	TAOK1	[activation]
33931	ITGA4	CAPNS1	[activation]
33932	BCL2L11	VDAC1	[activation]
33933	FAAP20	FANCD2	[activation]
33934	CDKN2C	SCGB2A2	[inhibition]
33935	THADA	USHBP1	[activation]
33936	ID1	MYF5	[activation]
33937	KCNE1	PRKCA	[activation]
33938	SRI	SMAD2	[inhibition]
33939	ACACA	NEDD8	[activation;inhibition]
33940	CCDC8	SLC25A6	[activation]
33941	NRIP1	KAT2B	[activation]
33942	FCGR3A	CD4	[activation]
33943	RHOJ	BLZF1	[activation]
33944	PAK6	CDK1	[activation]
33945	ACAP1	GRB2	[activation]
33946	GEM	APOH	[activation]
33947	PDPK1	PNO1	[activation]
33948	LIMS1	STK11	[activation]
33949	SMURF1	PRKAR1A	[inhibition]
33950	HSPA2	LGR4	[activation]
33951	CDC42BPA	TAF1	[activation]
33952	MARK3	YWHAB	[activation]
33953	PML	EGFR	[activation]
33954	MAPKAPK2	CREB1	[activation]
33955	GZMA	NDUFS3	[activation]
33956	THOP1	MPP6	[activation]
33957	CHEK1	MCPH1	[activation;inhibition]
33958	CCL7	ACKR4	[activation]
33959	PRKAB2	ULK1	[inhibition]
33960	THBS1	SCARB2	[activation]
33961	AR	SH2D1B	[activation]
33962	WHSC1L1	FGFR4	[activation]
33963	EEF2K	PRKACA	[activation]
33964	TSC1	ACTA2	[activation;inhibition]
33965	GAK	LRRK2	[activation]
33966	FOS	XBP1	[activation]
33967	IL13RA2	ERBB2	[activation]
33968	ESRRB	NCOA3	[activation]
33969	HMGB1	VCAM1	[activation]
33970	DNAJA2	FBXO6	[inhibition]
33971	KIAA0930	SFN	[activation]
33972	ENO2	MAP4	[activation]

33973	ANAPC16	CDC23	[activation]
33974	CCDC33	RAD54B	[activation]
33975	GRB2	TMOD3	[activation]
33976	CCL8	DARC	[activation]
33977	GRB2	TRIM39	[activation]
33978	ICT1	MRPL55	[activation]
33979	TBX21	CREBBP	[activation]
33980	KDR	ATR	[activation]
33981	GLMN	CDC37L1	[activation]
33982	PMAIP1	PPBP	[activation]
33983	RAC1	NCKAP1	[activation]
33984	GPN1	GPN3	[activation]
33985	BCL3	FOS	[activation]
33986	SUV39H1	MTO1	[activation]
33987	NKX2-3	SRF	[activation]
33988	NKX2-1	CRK	[activation]
33989	RNF8	NBN	[activation]
33990	ERBB2	OSMR	[activation]
33991	SEZ6L2	FOXA1	[activation]
33992	ACTB	CARM1	[activation]
33993	MAPK3	RAB4A	[activation]
33994	CASP3	CDKN1A	[activation]
33995	NKX2-1	WWTR1	[activation]
33996	ARAF	BRAF	[activation]
33997	F12	GP1BA	[activation]
33998	PAXIP1	PPHLN1	[activation]
33999	TERF1	SH3GL3	[activation]
34000	JUN	NRIP1	[activation]
34001	GOLGA2	CDK18	[activation]
34002	DCN	COL4A4	[activation]
34003	CAPN1	GPT	[activation]
34004	GTF3C2	RPTOR	[activation;inhibition]
34005	EP300	PRKAA1	[activation]
34006	CALD1	RPA3	[activation]
34007	NDC80	MAD2L1	[inhibition]
34008	RAD51	RPA3	[activation]
34009	GNAS	CHEK2	[activation]
34010	MLH1	LEF1	[activation]
34011	GRB2	HIBCH	[activation]
34012	PRMT1	TNFRSF14	[activation]
34013	CEP170P1	CCDC146	[activation]
34014	DUSP22	MAP2K4	[activation]
34015	SPP1	BRD3	[activation]
34016	KDM6A	AR	[activation]
34017	CDH1	HEMGN	[activation]
34018	MS4A2	AKT1	[activation]
34019	EIF2B5	CSNK2A1	[activation]
34020	GABARAPL2	TIAL1	[activation]
34021	PIK3C3	HSP90AA1	[activation]
34022	ABI3	EVL	[activation]
34023	TNFRSF1B	GBF1	[activation]
34024	HNF4A	ZNHIT3	[activation]
34025	GABARAPL2	NUP88	[activation]
34026	CDC14B	SIRT2	[activation]
34027	DNAJB1	SPP1	[activation]
34028	GBF1	IVNS1ABP	[inhibition]
34029	KCTD12	HLA-B	[activation]
34030	STK4	FBXW11	[activation]
34031	SHC1	CRK	[activation]
34032	NUP62	KPNA1	[activation]
34033	YWHAQ	LRRK2	[activation]
34034	HDAC4	YWHAG	[activation]
34035	HGS	PAK1	[activation]
34036	MYC	ARMC6	[activation]
34037	CACNG2	INADL	[activation]
34038	SPRR3	IVL	[activation]
34039	CSNK2A1	MAPT	[activation]
34040	CEACAM1	ANXA2	[activation]
34041	CCDC8	MAPK11	[activation]
34042	GAB1	BLK	[activation]
34043	NUP188	VCAM1	[activation]
34044	TCL1A	EP300	[activation]
34045	CDK1	SNW1	[activation]
34046	RPL23	LRRK2	[activation]
34047	ANXA5	KDR	[activation]
34048	SYK	UBASH3B	[activation]

34049	ESR1	PAK6	[activation]
34050	MEOX2	VIP	[activation]
34051	EP300	MCM2	[activation]
34052	POLA2	RBM23	[activation]
34053	EDN3	CTSE	[activation]
34054	DOK1	EGFR	[activation]
34055	DLL1	CNKSR3	[activation]
34056	HSP90AA1	MAP3K6	[activation]
34057	CCDC8	LUC7L	[activation]
34058	ANXA2	VCAM1	[activation]
34059	RICTOR	MTOR	[activation]
34060	RAD21	CCAR2	[inhibition]
34061	NOS1	ADRB2	[activation]
34062	PARP12	BCL6	[activation]
34063	RRAGB	RRAGC	[activation]
34064	PFDN1	SKP2	[inhibition]
34065	H2AFX	HIST3H3	[activation]
34066	MAPK13	TFG	[activation]
34067	SRC	DOCK8	[activation]
34068	TSC1	CD63	[inhibition]
34069	F11	TFPI2	[inhibition]
34070	BRCA1	E2F1	[activation]
34071	APC	AXIN2	[inhibition]
34072	KPNA2	CASP2	[activation]
34073	TP63	MYL12A	[activation]
34074	CSNK1A1	MAPT	[activation]
34075	CASC3	PTPN1	[activation]
34076	NCF1	MAPK9	[activation]
34077	SKP2	SPDYA	[inhibition]
34078	GNAI3	SOX30	[activation;inhibition]
34079	HNRNPUL1	ATM	[activation]
34080	TLR2	EGFR	[activation]
34081	STAT1	TNFRSF1B	[activation]
34082	ESR1	PPARG	[activation]
34083	ORC4	MLH1	[activation]
34084	HSP90AA1	FOXP2	[activation]
34085	FRMPD4	STK36	[activation]
34086	TP53	DNAJB1	[inhibition]
34087	HSPA6	CISH	[inhibition]
34088	ARHGAP33	RAC1	[activation]
34089	ZMYND19	ENOX1	[activation]
34090	CALCA	ECE1	[activation]
34091	ECSIT	EXOC6	[activation]
34092	LYN	FOLR1	[activation]
34093	CTSC	FBXO6	[activation]
34094	POLD1	ERCC6	[activation]
34095	CTNNA1	SFRP4	[activation]
34096	STK4	HSPA1L	[activation]
34097	PIK3R1	FANCA	[activation]
34098	SRC	FCGR2C	[activation]
34099	MAPK1	PLK3	[activation]
34100	LARP1	YWHAB	[activation]
34101	SAMD4A	YWHAG	[activation]
34102	THRB	NCOA1	[activation]
34103	HSP90AB1	ARMC5	[activation]
34104	MAPRE1	HLA-B	[activation]
34105	EIF4A3	JAK1	[activation]
34106	PRKACA	HSP90AB1	[activation]
34107	PLXNB1	RND3	[activation]
34108	SKP2	WEE1	[inhibition]
34109	TGFBR2	TRAF6	[activation]
34110	PTPN11	PILRA	[activation]
34111	ATG5	RHPN2	[activation]
34112	PAXIP1	SNRNP70	[activation]
34113	PRKCE	ACTA1	[activation]
34114	CDKN2C	ANGPTL4	[inhibition]
34115	STAT1	SPTBN1	[activation]
34116	MAP3K7	EIF2AK2	[activation]
34117	TP53	MRPS14	[activation]
34118	POP4	MYC	[activation]
34119	CASP3	CASP4	[activation]
34120	TRDMT1	STX1A	[activation]
34121	MAP2K1	BUB1	[activation]
34122	LPAR1	ARHGEF12	[activation]
34123	TGFBR1	SKI	[inhibition]
34124	STRA13	FANCC	[activation]

34125	RUNX1	CDK1	[activation]
34126	KMT2A	E2F4	[activation]
34127	PRKD2	XPO1	[activation]
34128	CHRD	SPRY2	[inhibition]
34129	YWHAG	EIF4A3	[activation]
34130	CTNNB1	MDM2	[activation]
34131	GAPVD1	NXF1	[activation]
34132	SMURF1	TNNT1	[inhibition]
34133	RPS17	CACNA1A	[activation]
34134	DAG1	AGR3	[activation]
34135	GALR2	NPY1R	[activation]
34136	CDH5	KDR	[activation]
34137	SDC1	PECAM1	[activation]
34138	L3MBTL1	TP53	[activation]
34139	MZT2B	TP53	[activation]
34140	ICAM1	HIST1H1E	[activation]
34141	MYC	HDAC3	[activation]
34142	H2AFX	SUPT5H	[activation]
34143	EPB41L3	CDC5L	[activation]
34144	BUB1B	COMMD4	[inhibition]
34145	KIF11	SNW1	[activation]
34146	ST3GAL6	APP	[activation]
34147	KDM1A	ISL1	[activation]
34148	TRAF3	NFKB2	[activation]
34149	TP53	YWHAG	[activation]
34150	APPL1	LUC7L	[activation]
34151	CDK7	CCNH	[activation]
34152	SFRP4	CDKN2A	[inhibition]
34153	PILRB	PTPN11	[activation]
34154	RET	SOS1	[activation;inhibition]
34155	SMAD2	CSNK1D	[activation]
34156	PLG	F10	[activation]
34157	AURKA	PML	[activation]
34158	NCOA1	CXADR	[activation]
34159	FYN	IL3RA	[activation]
34160	JAK1	CCR1	[activation]
34161	NCR2	TYROBP	[activation]
34162	MYH14	GRB2	[activation]
34163	SHC1	VARS	[activation]
34164	PTPRA	PTPRF	[activation;inhibition]
34165	NEU3	GRB2	[activation]
34166	ICT1	KIAA0391	[activation]
34167	PTS	ATM	[activation]
34168	NXF1	TBC1D16	[activation]
34169	EXOC8	WASH1	[activation]
34170	STX5	STX17	[activation]
34171	STAP2	PTK6	[activation]
34172	CD5	PIK3R1	[activation]
34173	NOP10	DKC1	[activation]
34174	MAVS	BIRC3	[activation]
34175	ZAP70	SLA	[activation]
34176	SSBP4	ISL1	[activation]
34177	UNC119	EIF2S2	[activation]
34178	HIST1H2BB	DYRK1A	[activation]
34179	EGR2	RIMKLB	[activation]
34180	GMFB	PRKCA	[activation]
34181	IGF1R	MDM2	[activation]
34182	NBPF14	MYB	[activation]
34183	MYC	TRAP1	[activation]
34184	ZC3HC1	TNFSF11	[activation]
34185	VAV1	ARHGDIB	[activation]
34186	VCL	ARPC2	[activation]
34187	ESR1	PSIP1	[activation]
34188	TH	YWHAB	[activation]
34189	IDH2	MDM2	[activation]
34190	IKBKE	MIF	[activation]
34191	RELA	HSPA8	[activation]
34192	BAG3	BCL2L11	[activation]
34193	PPP2R4	CAV1	[activation]
34194	KAT2A	PRKDC	[activation]
34195	POLD1	GABARAPL2	[activation]
34196	C17orf59	MOS	[activation]
34197	GRB2	RPS16	[activation]
34198	RAC1	HMHA1	[activation]
34199	POLD2	EP300	[activation]
34200	UBE2I	EP300	[activation]

34201	SUMO1	CEBPB	[activation]
34202	VCL	TEKT1	[activation]
34203	C1QC	TFAP2A	[activation]
34204	ADRB2	GRIA3	[activation]
34205	PLD1	CAV1	[activation]
34206	TCAP	ADPGK	[inhibition]
34207	CD72	GRB2	[activation]
34208	TNKS	POT1	[activation]
34209	PPP1R15A	PPP1CA	[inhibition]
34210	ICT1	MRPL24	[activation]
34211	GH1	MAPRE1	[activation]
34212	UTP15	MYC	[activation]
34213	GRB2	MATR3	[activation]
34214	ITGA4	CAPZB	[activation]
34215	DNAJA3	NFKB2	[inhibition]
34216	MAGEA3	DNM1L	[activation]
34217	YWHAZ	PRKCE	[activation]
34218	FGF4	NRP1	[activation]
34219	RNF32	MAST1	[activation]
34220	KDR	YES1	[activation]
34221	RUNX2	ETS1	[activation]
34222	ATG7	PIK3C2A	[activation]
34223	HSP90AA1	SRC	[activation]
34224	ATF2	ITGB4	[activation]
34225	IGKV1-5	ATG5	[activation]
34226	TXLNA	STX4	[activation]
34227	GXYLT1	HLA-B	[activation]
34228	FASLG	GRB2	[activation]
34229	CD81	HNRNPA2B1	[activation]
34230	PRKCZ	IL4R	[activation]
34231	APP	F12	[activation]
34232	EGFR	MAP2K1	[activation]
34233	PPP2R2A	EIF4EBP1	[inhibition]
34234	VASP	APBB1	[activation]
34235	PTEN	CRKL	[activation]
34236	CDC25C	YWHAG	[activation]
34237	B3GNTL1	MPP3	[activation]
34238	ETV1	MAPK8	[activation]
34239	RPS6KA5	BAD	[activation]
34240	PEBP1	FTSJ1	[inhibition]
34241	RIPK1	LTA	[activation]
34242	CRMP1	ANXA7	[activation]
34243	MAPK3	PTPN5	[inhibition]
34244	ATF2	YWHAZ	[activation]
34245	PAK2	SNX33	[activation]
34246	DMWD	FTSJ1	[activation]
34247	HIST1H2BB	HIST1H3A	[activation]
34248	SERPIND1	APP	[inhibition]
34249	NCF1	TGFB1I1	[activation]
34250	BARD1	MCM6	[activation]
34251	GRAP2	FASLG	[activation]
34252	SIRT1	CTTN	[activation]
34253	SLIT2	GPC1	[activation]
34254	STX6	PTPN9	[activation]
34255	CBL	FLT1	[activation]
34256	DPYSL5	DPYSL4	[activation;inhibition]
34257	TEC	VAV1	[activation]
34258	MAP2K1	SUMO1	[activation]
34259	LRCH4	NOTCH2NL	[activation]
34260	VCAM1	PYCR2	[activation]
34261	KIF11	CDC5L	[activation]
34262	HSD17B4	CDK7	[activation]
34263	PPBP	PLEKHJ1	[activation]
34264	MAPK14	SRPK1	[activation]
34265	COL4A3BP	CSNK1G2	[activation]
34266	GUSBP1	FGFR3	[activation]
34267	MECOM	SMAD4	[activation]
34268	FSCN1	SGK1	[activation]
34269	PECAM1	PTPN11	[activation]
34270	YES1	FAS	[inhibition]
34271	RFX6	PLEKHN1	[activation]
34272	YES1	CD2AP	[inhibition]
34273	KNG1	VTN	[inhibition]
34274	NUDCD3	CXorf57	[activation]
34275	GRB2	NEK8	[activation]
34276	DDX5	GABARAPL2	[activation]

LRRK2	EEF1A2	[activation]
PRKRA	TIRAP	[activation]
TP53RK	INADL	[activation]
SRPK2	SUPT16H	[activation]
PRKCB	GJA1	[inhibition]
CCDC22	ACTG1	[activation]
ISL1	COPS5	[activation]
LARP1	YWHAG	[activation]
OSTF1	FASLG	[inhibition]
GRB10	GRB2	[activation]
SRPK1	FAM76B	[activation]
APP	TBCK	[activation]
NCF1	ACTG1	[activation]
RAC1	GRN	[activation]
MCC	SFRP4	[activation]
KCNA3	CAV1	[activation]
ERBB2IP	ABR	[inhibition]
AMBRA1	NUDC	[activation]
AP2S1	NUMB	[activation]
HDAC4	ATF2	[activation]
DAB1	RHOXF2	[activation]
ARHGAP17	NXF1	[activation]
PGAM5	MAP3K5	[inhibition]
FYN	WNK2	[activation]
ARHGEF7	EGFR	[activation]
PCBD2	STAT3	[activation]
CMTM3	MAPK1	[activation]
DOK1	TEC	[activation]
MMP8	UMOD	[activation]
ESRRA	NXF1	[activation]
CTNNB1	APC2	[activation]
COL17A1	CTNND1	[activation]
ESR1	BUD13	[activation]
LRRK1	LRRK2	[activation]
RHOA	BAIAP2	[activation]
ITGAV	TGFB1	[activation]
TRAF3	TNFRSF18	[activation]
GTF2A2	TBPL1	[activation]
NXF1	DSN1	[activation]
USP7	CLSPN	[activation]
APP	MAPT	[activation]
GRB2	RAPSN	[activation]
RELA	REV1	[activation]
STX3	SYTL4	[activation]
SPERT	DUSP14	[inhibition]
PMAIP1	BCL2L11	[activation]
WT1	AREG	[activation]
SRPK1	SLAIN2	[activation]
PPP1CA	SFRP1	[activation;inhibition]
PRKG1	HDAC1	[inhibition]
BCL2L1	IRS1	[activation]
ADRBK1	MDM2	[activation]
RUNX1	CBFA2T2	[activation]
MAPK1	FCGR2B	[activation]
RAD1	RHNO1	[activation]
ITGB1	ENO1	[activation]
CREBBP	SOX9	[activation]
BLK	BCL2	[activation]
SRPK3	CCDC86	[activation]
HSP90AB1	MAP3K7	[activation]
YWHAZ	MAPT	[activation]
CHL1	E2F1	[activation]
PDZD11	SFN	[activation]
MARS	ITGA4	[activation]
IGHG1	TRAF1	[activation]
STAB2	MAPK8	[activation]
SGTA	HLA-B	[activation]
SUMO1	EGR1	[activation]
TNFRSF13C	TRAF2	[activation]
C16orf74	PPP3CA	[activation]
CDC42	ARHGAP10	[activation]
PTPRE	MAPK1	[activation;inhibition]
RHOT2	ZFYVE9	[activation]
ACTG1	CCDC8	[activation]
JUN	MYC	[activation]
TGFBR1	RHOG	[activation]

34353	CTNNB1	USP9X	[activation]
34354	MECOM	SUV39H1	[activation]
34355	FYN	PRKCZ	[activation]
34356	GAL	GALR2	[activation]
34357	VSIG4	C3	[activation]
34358	STK11	HSP90AA1	[activation]
34359	RICTOR	RPL26	[activation]
34360	LSM2	RALA	[activation]
34361	CALM1	SLC9A1	[activation]
34362	DHX9	TRAF6	[activation]
34363	PICK1	EPHB2	[activation]
34364	ITGA4	HIST1H2BL	[activation]
34365	F2R	PROCR	[activation]
34366	HDAC1	STAT1	[activation]
34367	AMIGO1	AMIGO3	[activation]
34368	FKBP1A	RYR1	[inhibition]
34369	CD74	MIF	[activation]
34370	ANGPTL2	LILRB2	[activation]
34371	PTPN12	BCAR1	[activation]
34372	YES1	OGT	[activation]
34373	LRRK2	MATK	[activation]
34374	OBSL1	UBTF	[activation]
34375	FES	DPYSL5	[activation]
34376	GAP43	PLCD1	[activation]
34377	EGFR	MAP3K3	[activation]
34378	DDX18	VCAM1	[activation]
34379	PPARGC1A	NRIP1	[activation]
34380	NAPA	GOSR1	[activation]
34381	PLK1	CTNNB1	[activation]
34382	BARD1	POMZP3	[activation]
34383	EPRS	ETS1	[activation]
34384	EPB41L3	YWHAE	[activation]
34385	WIZ	RICTOR	[activation]
34386	EIF4EBP3	AURKB	[activation]
34387	FPR1	MAPK13	[activation]
34388	VAPA	ZFYVE27	[activation]
34389	NDP	APP	[activation]
34390	RIT1	IFRD1	[activation]
34391	RGS3	MAPK12	[activation;inhibition]
34392	DBNL	EIF2AK2	[inhibition]
34393	CDK6	ABI2	[activation]
34394	SRRM1	SRPK1	[activation]
34395	CHUK	AURKA	[activation]
34396	APC	TRIM28	[inhibition]
34397	CARM1	CTNNB1	[activation]
34398	XPO1	STAT1	[activation]
34399	PPP2R1B	PPP2R1A	[inhibition]
34400	STK38	EIF4A3	[activation]
34401	SKIL	CPNE2	[activation]
34402	ATP1B3	BARD1	[activation]
34403	SLC25A4	BCL2L13	[inhibition]
34404	SUMO3	EP300	[activation]
34405	RAF1	SH3KBP1	[activation]
34406	STRN3	CTNNB1	[activation]
34407	CDKN1A	MCM7	[activation]
34408	HLA-B	PDIA4	[activation]
34409	CRY2	PER1	[inhibition]
34410	SCARB1	PRMT2	[activation]
34411	RAC1	AKT1	[activation]
34412	POU2F2	RXRA	[inhibition]
34413	TAB2	PPP6C	[activation]
34414	CALM1	MYF6	[activation]
34415	CCDC85A	PFDN1	[inhibition]
34416	HSPB1	SLX4	[activation]
34417	IGHMBP2	NAA38	[activation]
34418	FBXL5	CUL1	[inhibition]
34419	NXF1	NXF2	[activation]
34420	USHBP1	NGFRAP1	[activation]
34421	MAPK8	MAP3K2	[activation]
34422	TWIST1	ZIC3	[activation]
34423	ENTPD2	STX7	[activation]
34424	NXT1	XPO1	[activation]
34425	ANP32B	DCC	[activation]
34426	SET	PIK3CG	[activation]
34427	YWHAZ	ITGB2	[activation]
34428	CCHCR1	NME7	[activation]

RPTOR	G3BP1	[activation]
PRKDC	HMGB2	[activation]
ACTG1	CYBB	[activation]
STAT3	AMBP	[activation]
CCS	YWHAG	[activation]
TRIB3	BMPR2	[activation]
LIMS1	TRIO	[activation]
LYPLA1	VCAM1	[activation]
SIK1	VAPA	[activation]
JUND	FOSL1	[activation]
CTTN	KCNA2	[activation]
TRMT11	TP53	[activation]
CYBA	RAC1	[activation]
CDC5L	RPA3	[activation]
METAP2	MAPK1	[activation]
NR3C1	ONECUT1	[activation]
HSP90AB1	FOXP2	[activation]
APP	REG1A	[activation]
CXCR2	GNA14	[activation]
MAPT	MARCH7	[activation]
SREK1	SRPK2	[activation]
SRC	APOL5	[activation]
SH2D1B	ERBB2	[activation]
ARRB1	EGFR	[activation]
RAD50	PASK	[activation]
